# Supplementary material for: Spatial distribution and temporal trends in social fragmentation in England, 2001−2011: a national study
Source: BMJ Open. 2019 Jan 24;9(1):e025881. doi: 10.1136/bmjopen-2018-025881 (PMC6347895; doi:10.1136/bmjopen-2018-025881)
Supplement: Supplementary file 1 [file bmjopen-2018-025881supp001.pdf]

Supplementary Appendix

Spatial distribution and temporal trends in social fragmentation in England, 2001 to 2011: a national study

Contents

Description of data and formulas used for the calculation of the underlying social fragmentation indicators at both time points 3

Spatial weighted regressions to attribute 2001 data to 2011 LSOAs 4

Spatial Maps 4

Figure A: Index of Social Fragmentation (mean) for England and English regions, over time. 5

Figure A1 Spatial autocorrelation with Moran's I for single persons (top) and one person households (bottom), by region and over time 6

Figure A2 Spatial autocorrelation with Moran's I for private renting (top) and population turnover (bottom), by region and over time 7

Figure B1: Local Moran scatterplot for social fragmentation at the LSOA level, 2001 8

Figure B2: Local Moran scatterplot for social fragmentation at the LSOA level, 2011 8

Results from LISA analysis: 9

References 10

Description of data and formulas used for the calculation of the underlying social fragmentation indicators at both time points

2001

Percentage of single people in LSOA = All singles, divorced, widowed and separated in LSOA / All usual residents aged 16 and above in LSOA (dataset available from: *https://www.nomisweb.co.uk/census/2001/ks004)*

Percentage of one person households in LSOA = Total one person households in LSOA/ All household composition in LSOA (dataset available from: *https://www.nomisweb.co.uk/census/2001/uv065*)

Percentage of private renting in LSOA = Total houses rented from a private landlord or letting agent in LSOA/ All household composition in LSOA (dataset available from: *https://www.nomisweb.co.uk/census/2001/uv063)*

Percentage of population turnover in LSOA= People who are migrants (i.e. moved into the area from within the UK & moved into the area from outside the UK within the year before the census / All usual residents in LSOA (dataset available from: *https://www.nomisweb.co.uk/census/2001/ks024*)

2011

Percentage of single people in LSOA = All singles, divorced, widowed and separated in LSOA / All usual residents aged 16 and above in LSOA (dataset available from: *https://www.nomisweb.co.uk/census/2011/ks103uk*)

Percentage of one person households in LSOA = Total one person households in LSOA/ All household composition in LSOA (dataset available from: *https://www.nomisweb.co.uk/census/2011/ks105ew*)

Percentage of private renting in LSOA = Total houses rented from a private landlord or letting agent in LSOA/ All household composition in LSOA (dataset available from: *https://www.nomisweb.co.uk/census/2011/ks402ew*)

Percentage of population turnover in LSOA= People who are migrants (i.e. moved into the area from within the UK & moved into the area from outside the UK within the year before the census / All usual residents in LSOA (dataset available from: *https://www.nomisweb.co.uk/census/2011/ukmig008*)

Spatial weighted regressions to attribute 2001 data to 2011 LSOAs

The 2003 National Statistics policy to minimise the statistical impact of frequent electoral ward boundary changes was succeeded by boundary changes across LSOAs in 2011. In that year, there was a modification of OA (Output Areas) and SOA (Super Output Areas) in England & Wales mainly due to significant population changes between the decennial censuses. As social fragmentation in 2001 was measured in 2001 LSOAs, we attributed census 2001 LSOA information to 2011 LSOAs and we used population weighted regressions to estimate 2001 data at the 2011 LSOA to enable seamless comparisons across time. More specifically, 97.4% of all LSOAs remained unchanged in 2011, and for these areas we took no action and attributed the social fragmentation values from 2001 to the 2011 LSOAs. If two or more 2001 LSOAs merged in one LSOA in 2011 (0.6% of all LSOAs), we calculated the mean value of social fragmentation weighted for 2003 LSOA population (which is the oldest population estimates dataset provided by ONS). If a 2001 LSOA split into two or more 2011 LSOAs (1.8% of all LSOAs) we assigned the relevant 2001 social fragmentation scores. Finally, there were some LSOAs for which changes did not match any of the previously described patterns (i.e. an LSOA split in two and each part merged with a different LSOA). For these very few LSOAs (0.1% of all LSOAs) we developed an algorithm to calculate population weighted mean estimates. (1) The corresponding algorithm is available from the authors.

Spatial Maps

Digital vector boundaries for the 2011 LSOAs, generalised to 20 metres and clipped to the coastline to reduce size and improve visualisation, were obtained from the ONS open geography portal (2) . The vector boundaries were inputted in the Stata shp2dta command to calculate the centroid for each LSOA in the British National Grid format (3). These were then converted from British National Grid easting and northing to longitude and latitude in degrees (4).

Figure A: Index of Social Fragmentation (mean) for England and English regions, over time.

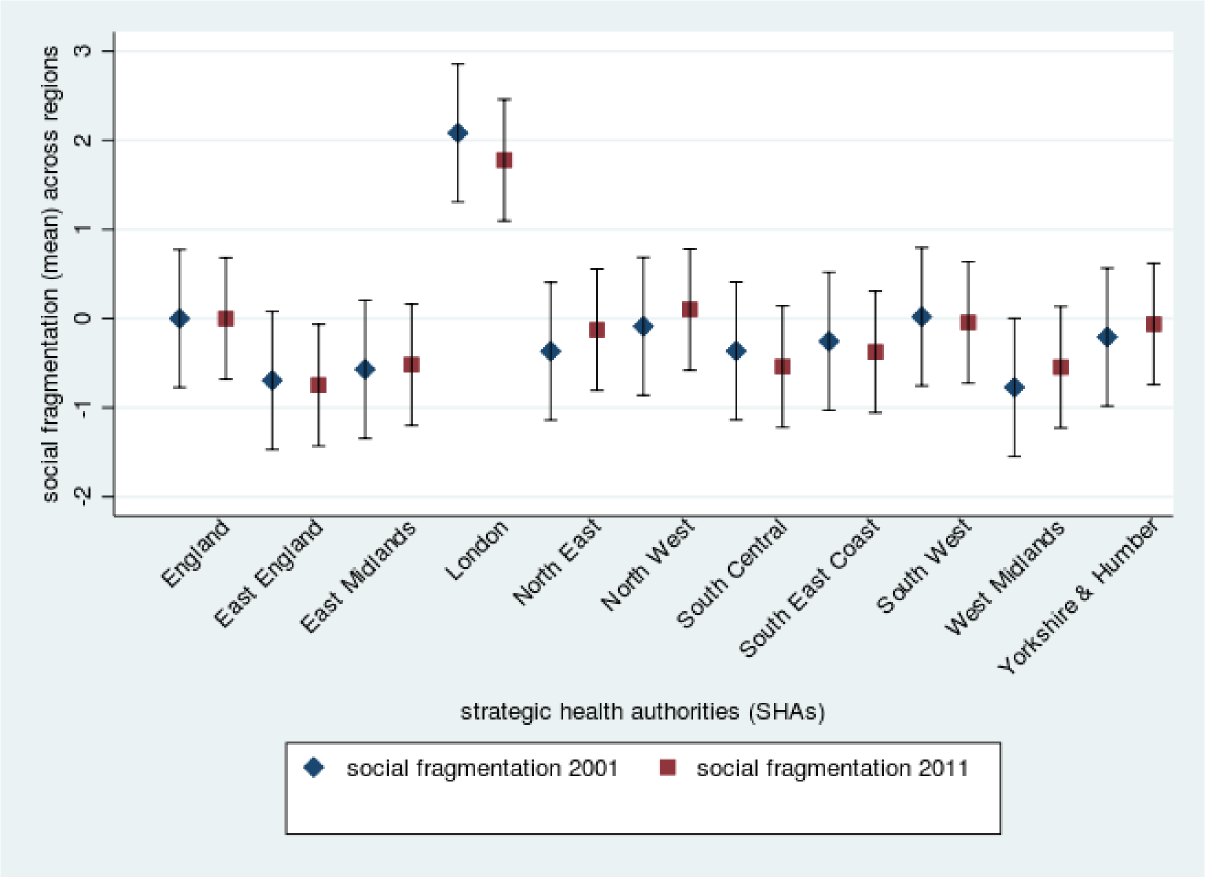

Figure A1: Spatial autocorrelation with Moran's I for single persons (top) and one person households (bottom), by region and over time

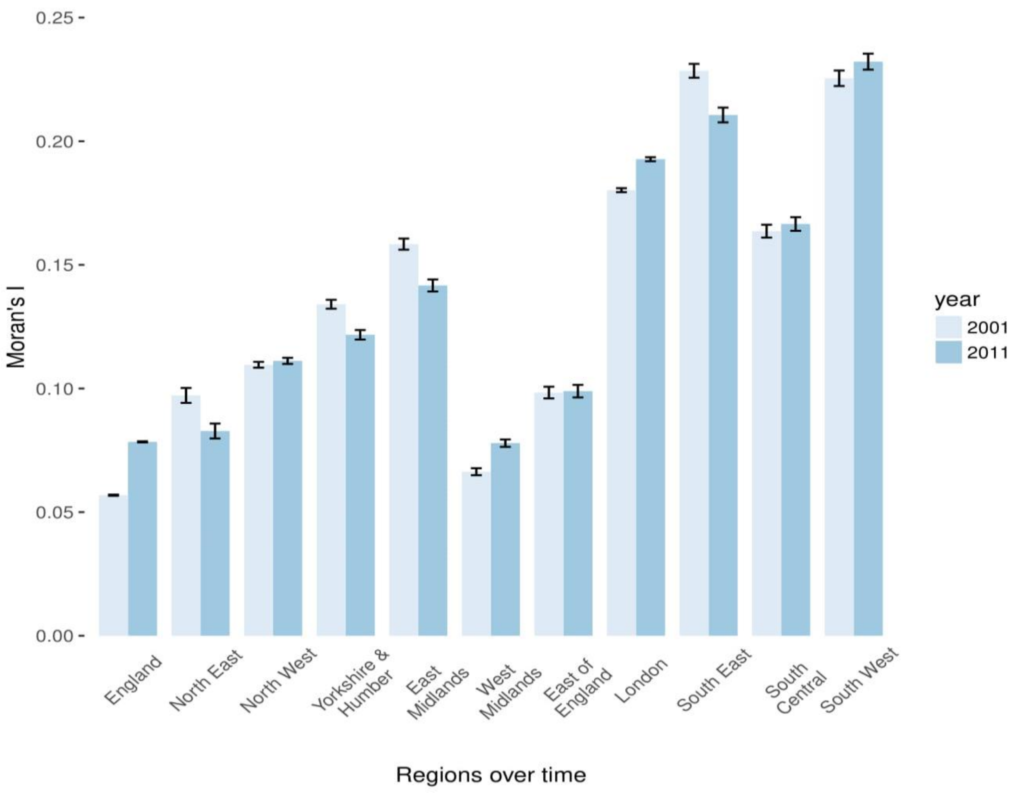

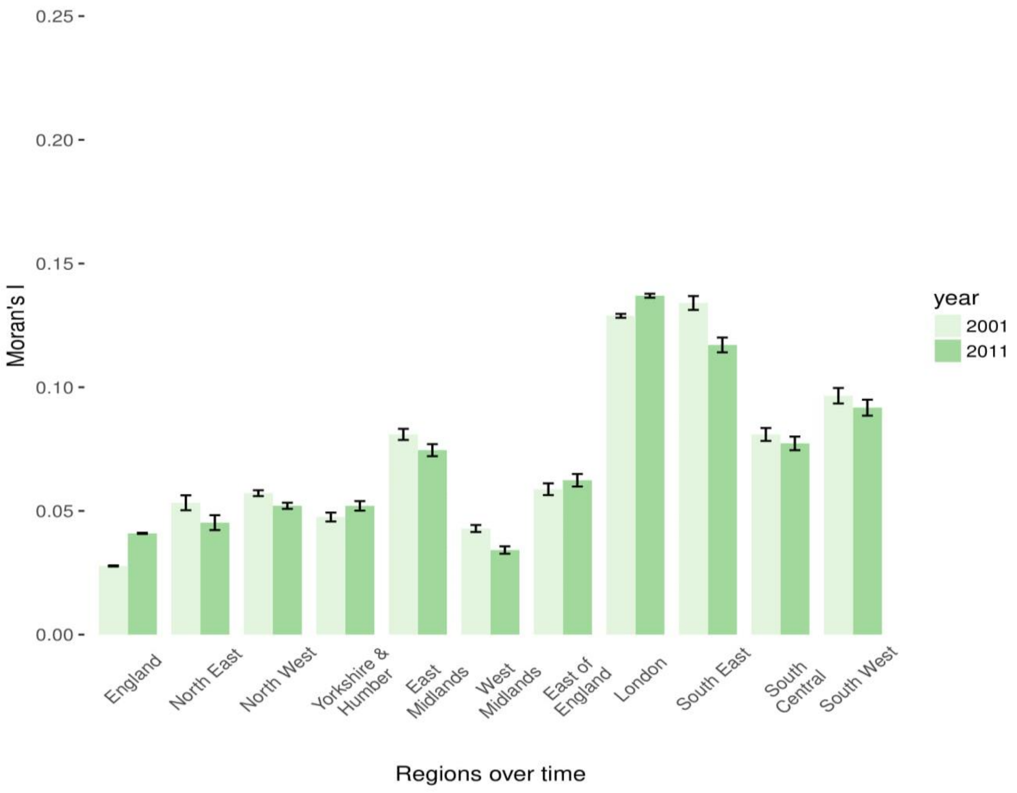

Figure A2: Spatial autocorrelation with Moran's I for private renting (top) and population turnover (bottom), by region and over time

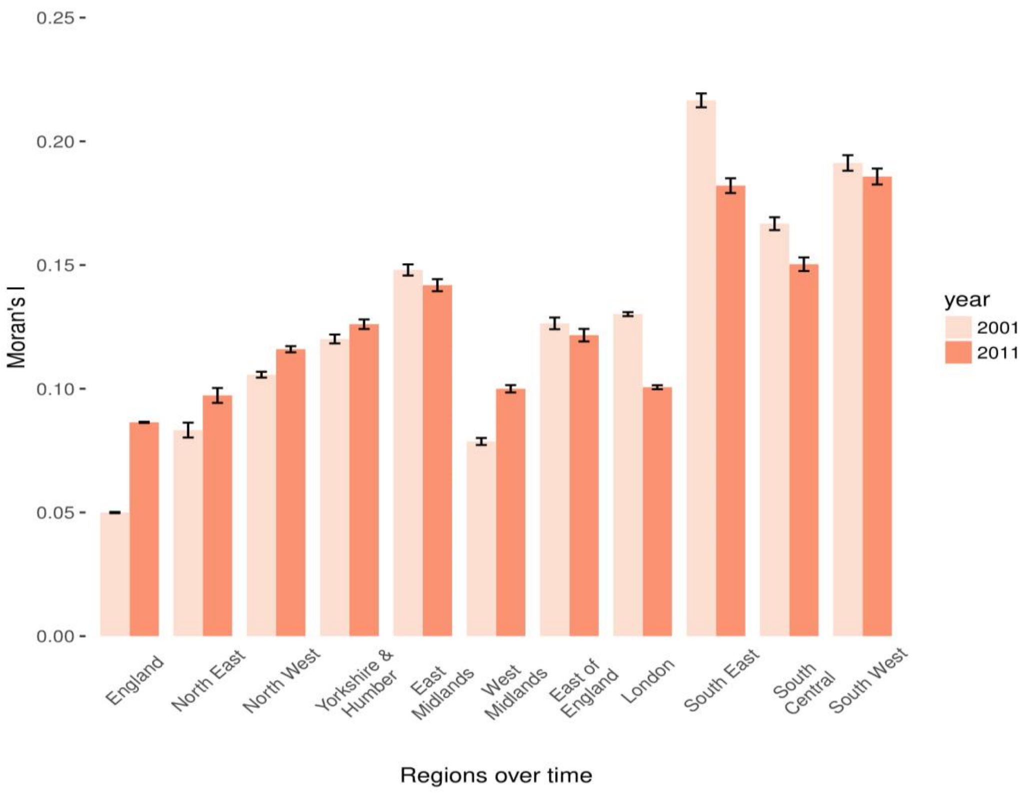

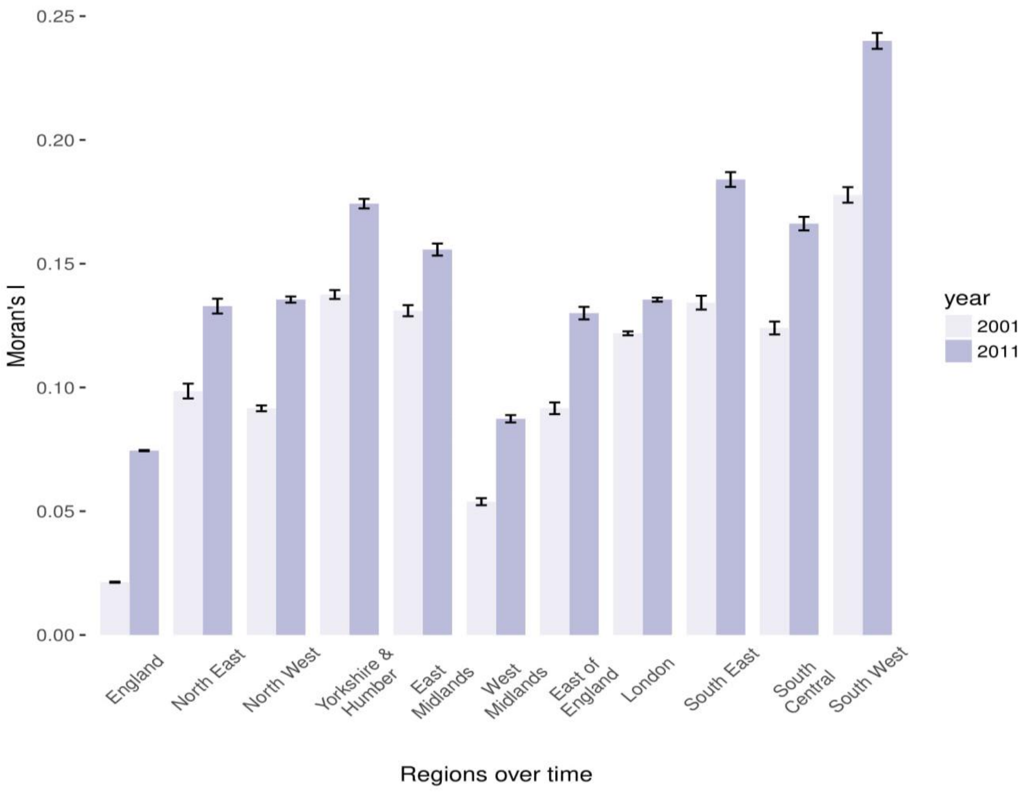

Figure B1: Local Moran scatterplot for social fragmentation at the LSOA level, 2001.

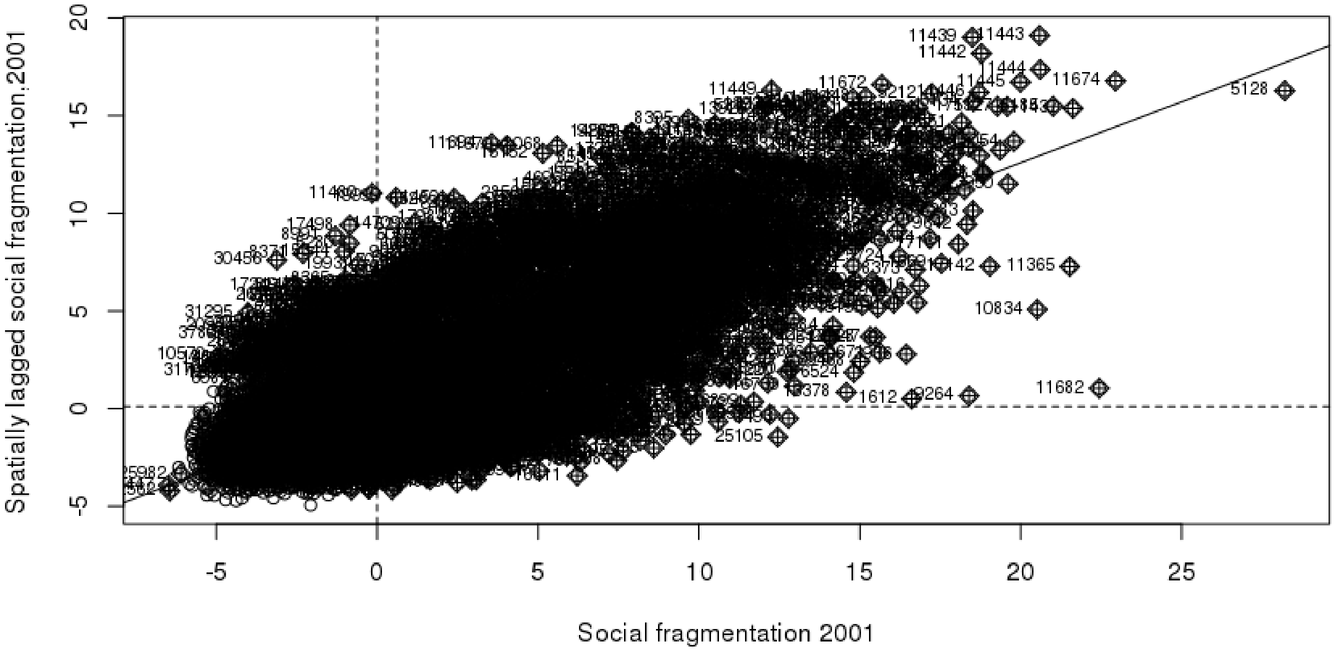

Figure B2: Local Moran scatterplot for social fragmentation at the LSOA level, 2011.

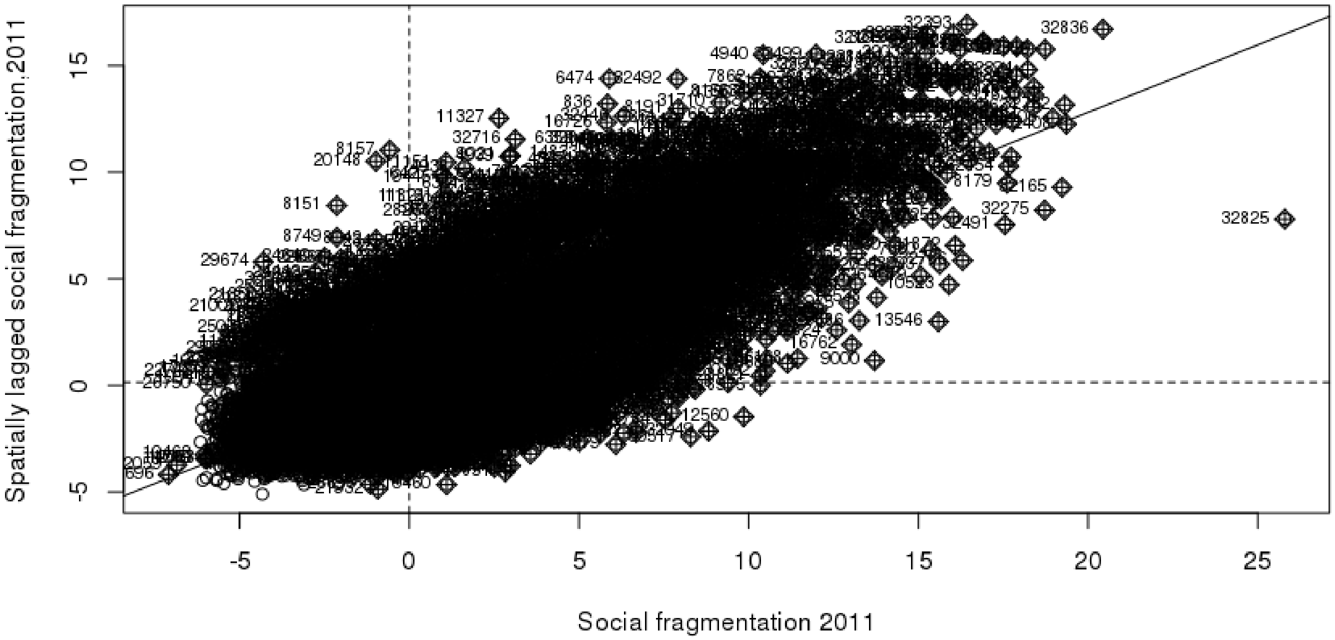

Results from Local Indicator of Spatial Association (LISA) analysis: The Moran scatterplots for social fragmentation in 2001 and 2011 are provided in Figures B1 and B2 respectively. The four quadrants in each scatterplot correspond to four types of association. The lower left and upper quadrants indicate positive spatial autocorrelation, or that areas in these quadrants have similar values (lower than the mean for those areas in the lower left quadrant and higher than the mean in the upper left quadrant). The upper left and lower right quadrant indicate spatial associations of areas with dissimilar values in social fragmentation. In both graphs the slope of the regression line indicates that there is a positive spatial association at both time points with a handful of outliers in 2001 and only a few in 2011. The pattern of increasing spatial autocorrelation across the two time points that we observed in graph 4 for global spatial autocorrelation is also confirmed by comparison of Graphs B1 and B2. Spatial autocorrelation appears to have increased in 2011 when compared to 2001. At both time points, LSOAs were mainly concentrated in the upper right quadrant (Figure B1 & B2) indicating the existence of many hot spots of social fragmentation and highlighting the fact that there are important variations among LSOAs and a finer scale spatial structure that occur below the region level.

References

1. Office for National Statistics. Changes to output areas and super output areas in England and Wales, 2001 to 2011. 2012.
2. Office for National Statistics. Open Geography Portal. 2013. https://geoportal.statistics.gov.uk/geoportal/.
3. SHP2DTA:. Stata module to converts shape boundary files to Stata datasets [program]. S456718 version: Boston College Department of Economics,. 2006.
4. Ordance Survey. A guide to coordinate systems in Great Britain. ONS, 2013:43. www.ordnancesurveycouk/docs/support/guide-coordinate-systems-great-britainpdf.
