# Supplementary material for: Spatial distribution and temporal trends in social fragmentation in England, 2001−2011: a national study
Source: BMJ Open. 2019 Jan 24;9(1):e025881. doi: 10.1136/bmjopen-2018-025881 (PMC6347895; doi:10.1136/bmjopen-2018-025881)

Social Fragmentation

England 2001, Social Fragmentation

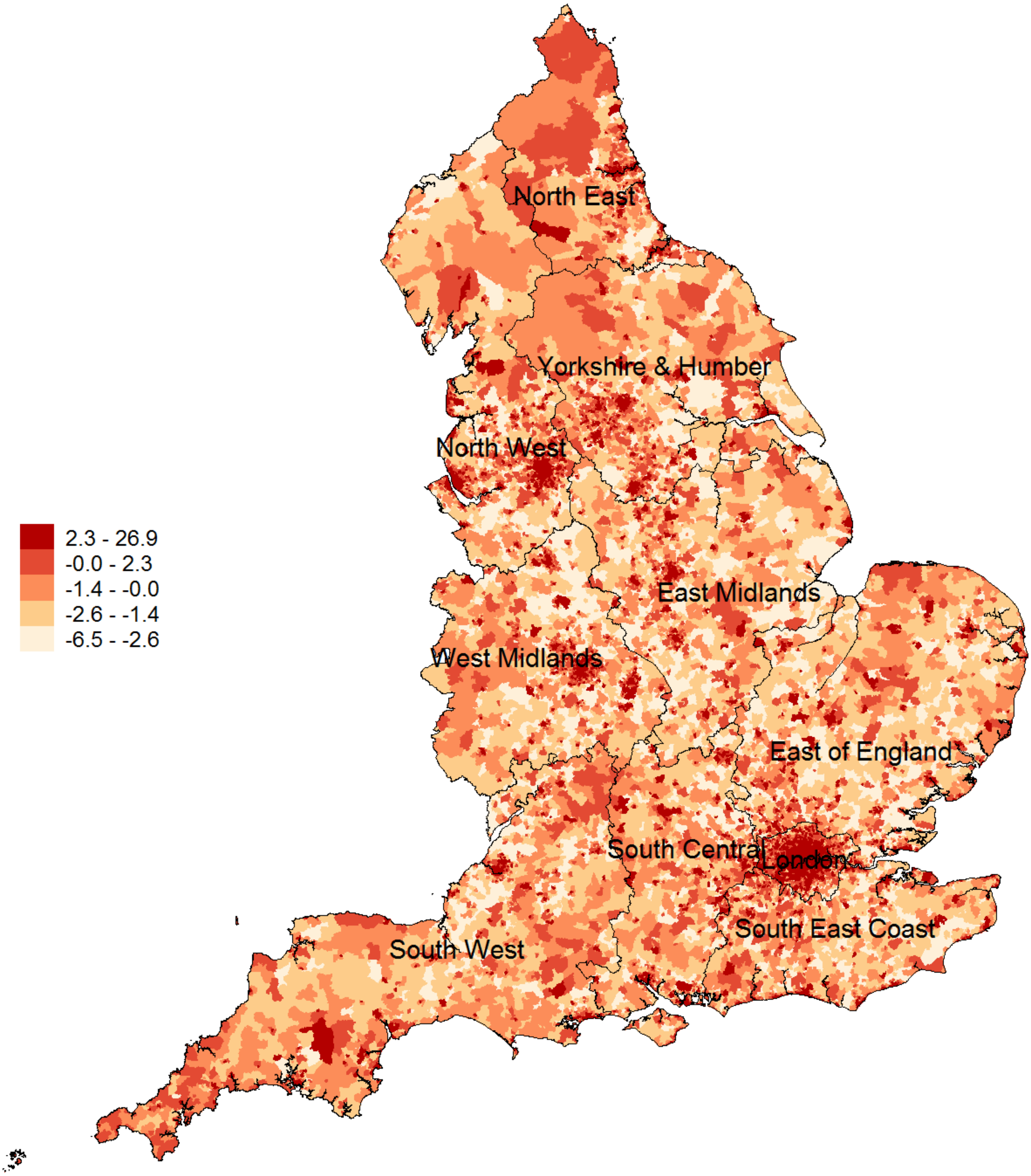

England 2011, Social Fragmentation

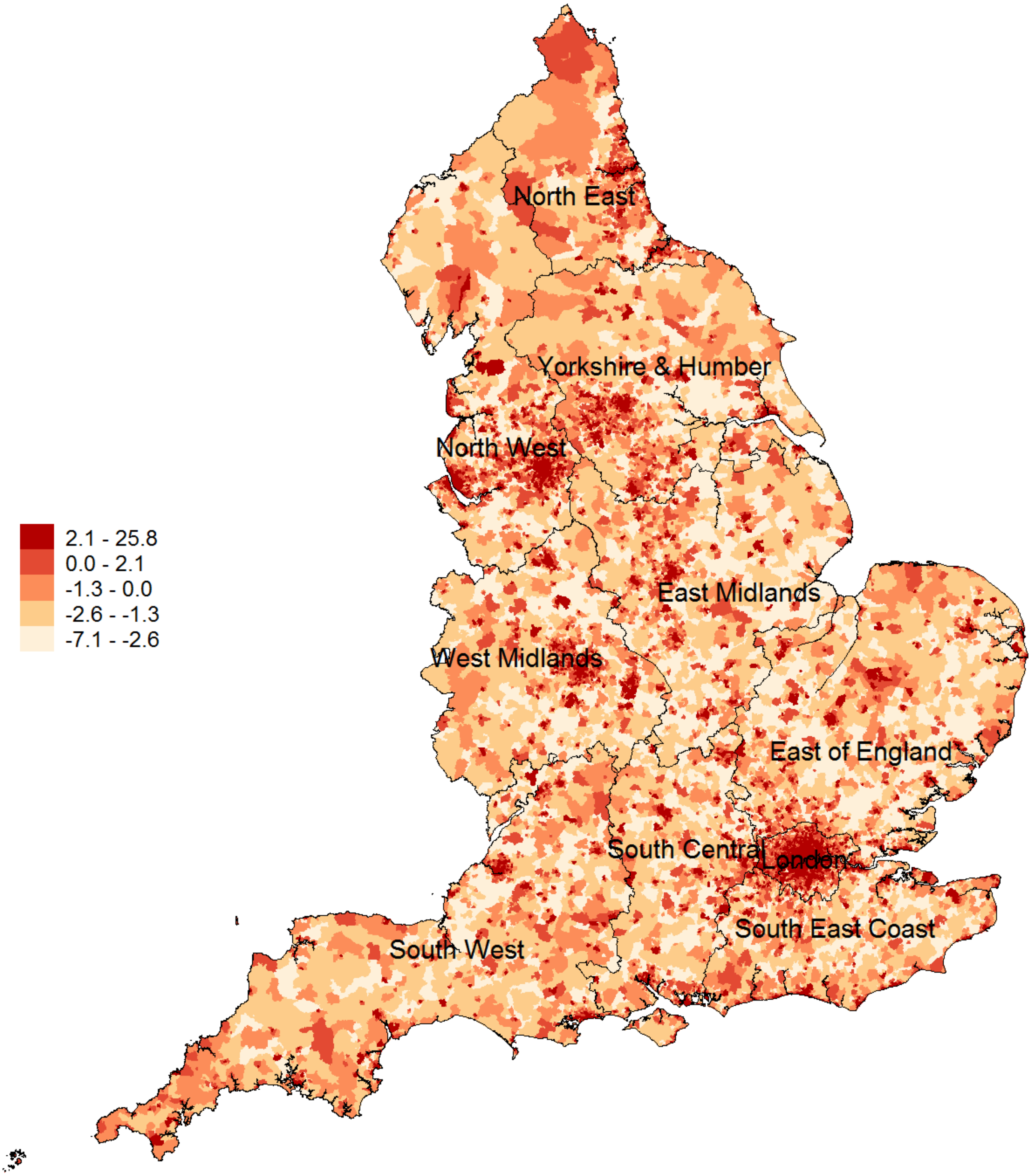

North East, Social Fragmentation

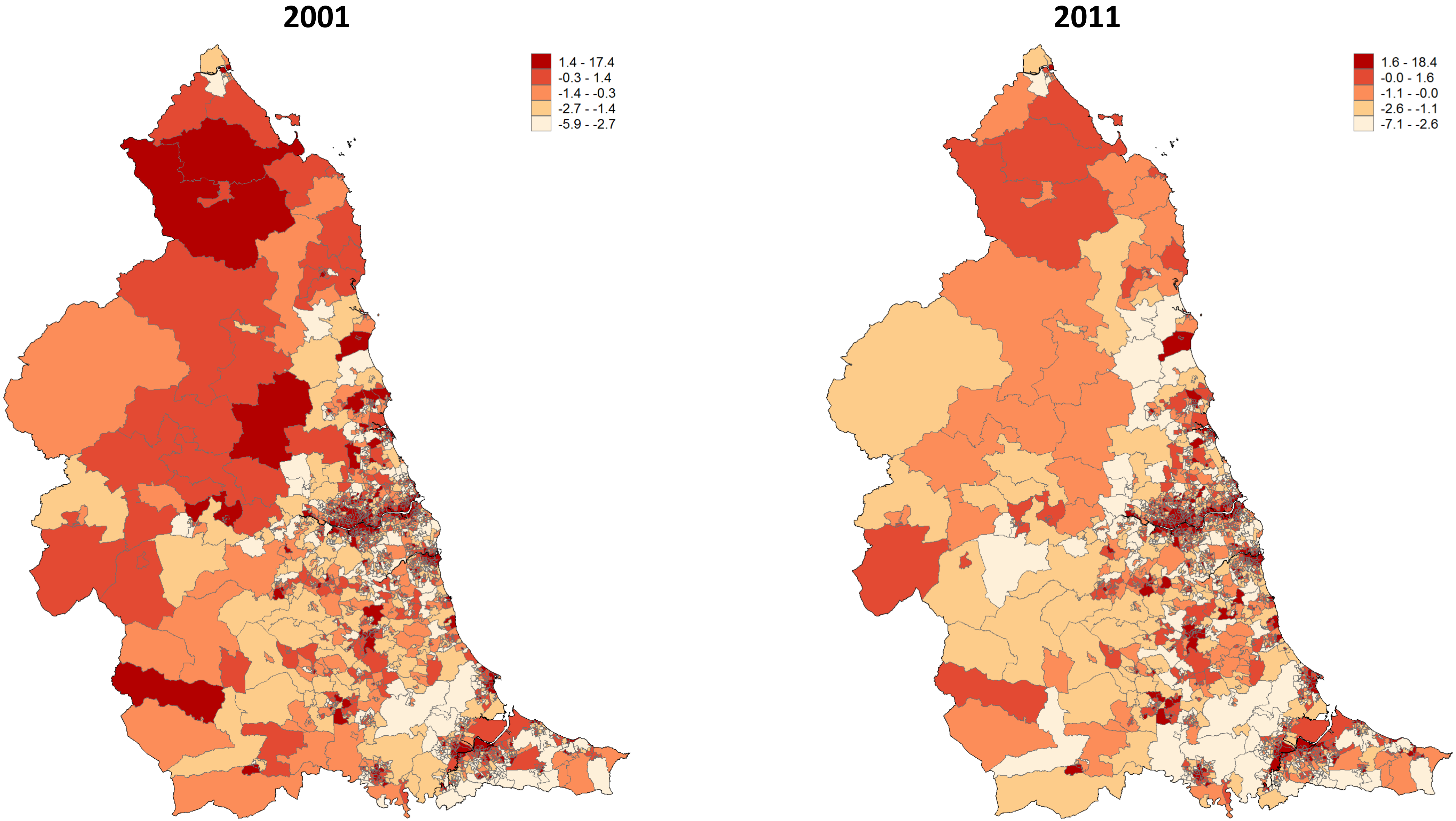

North West, Social Fragmentation

2001

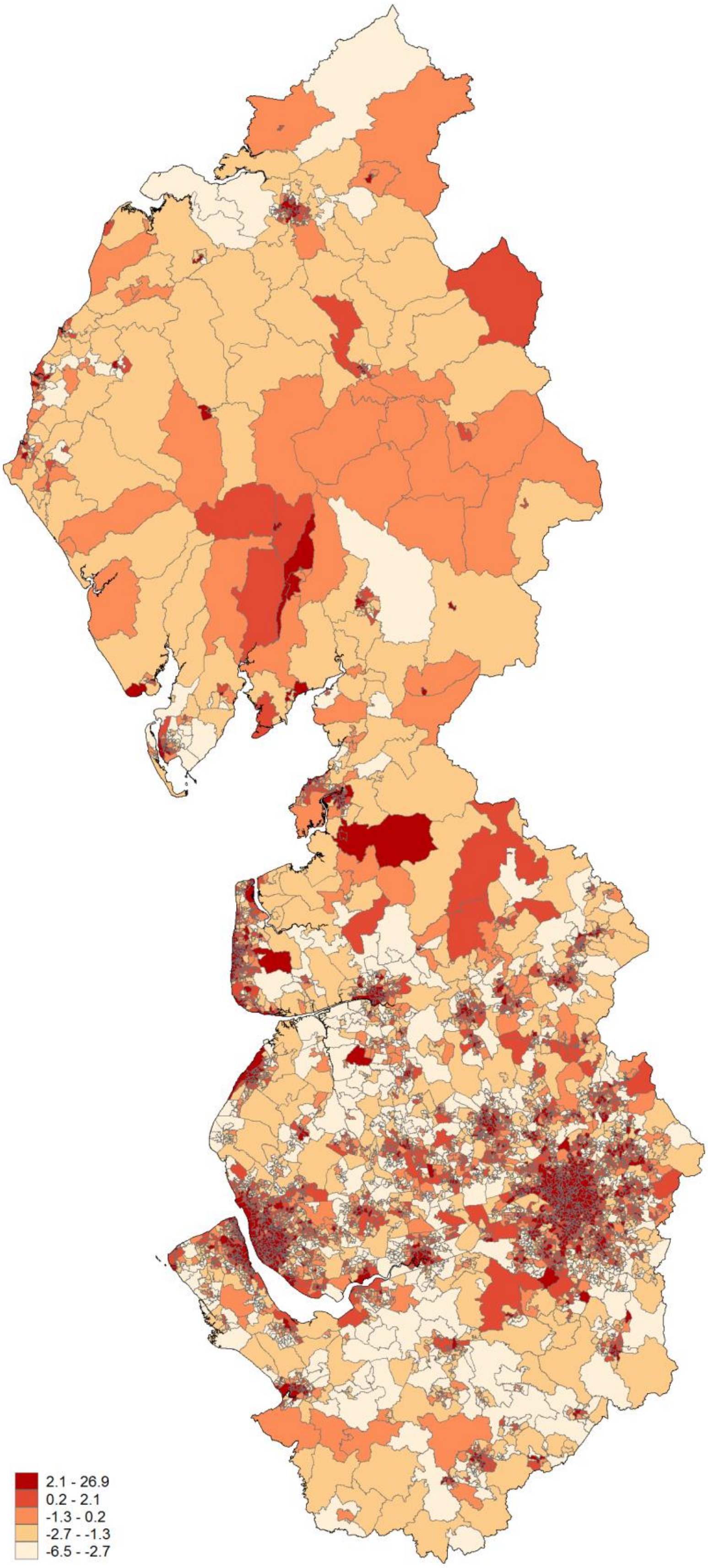

2011

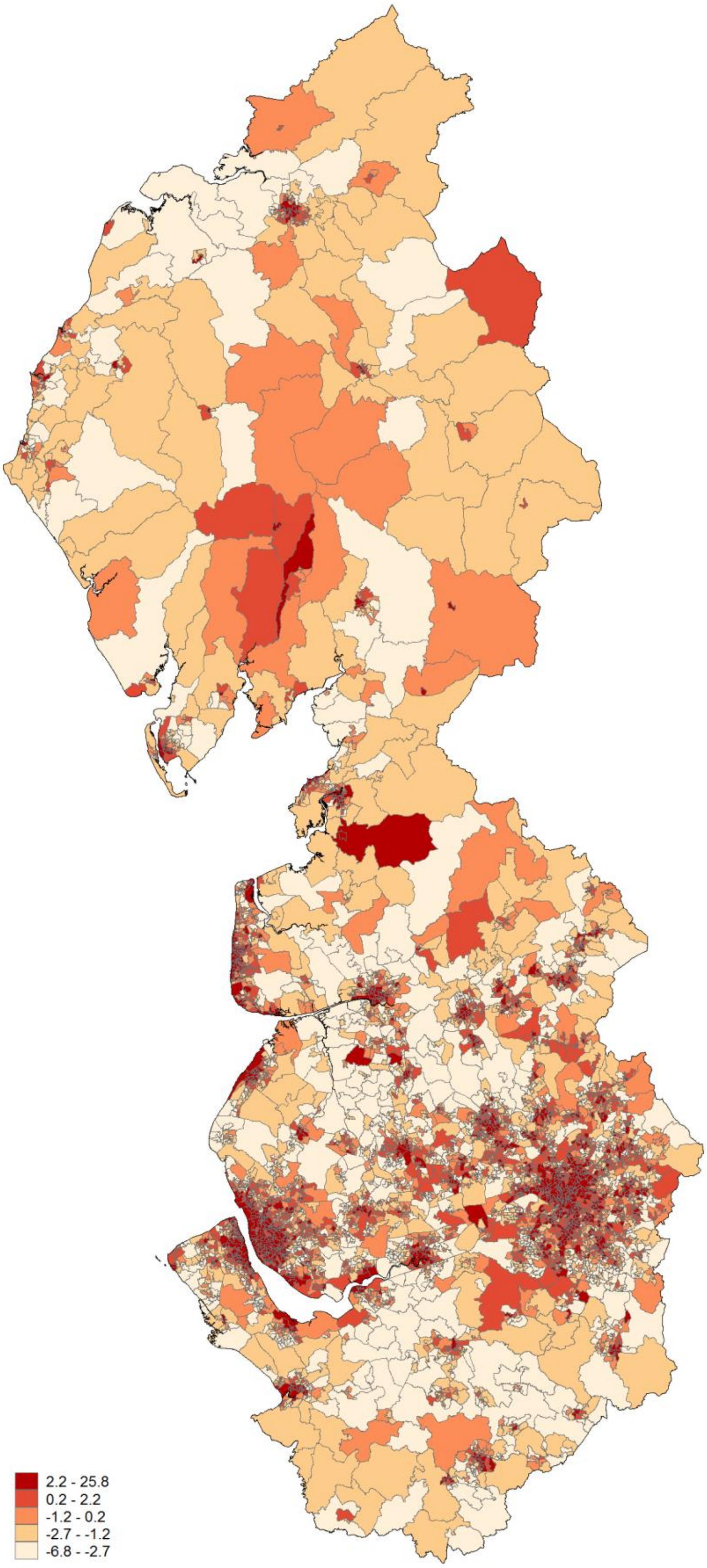

Yorkshire and the Humber, Social Fragmentation

2001

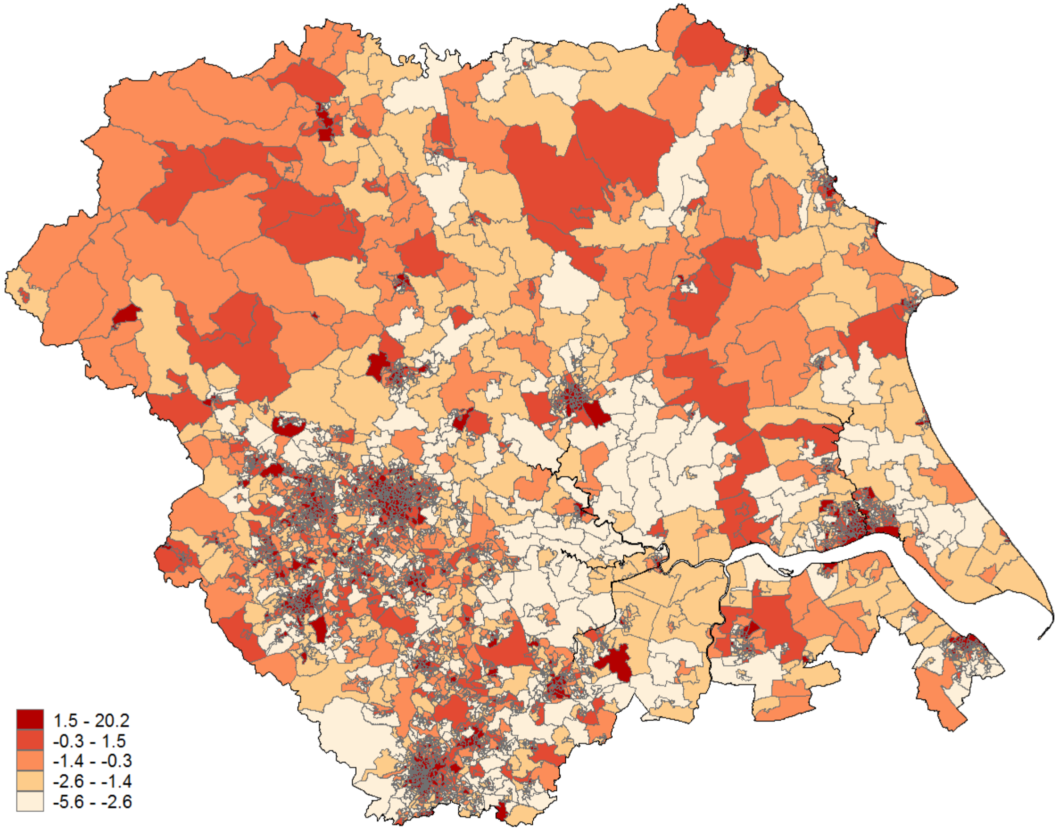

2011

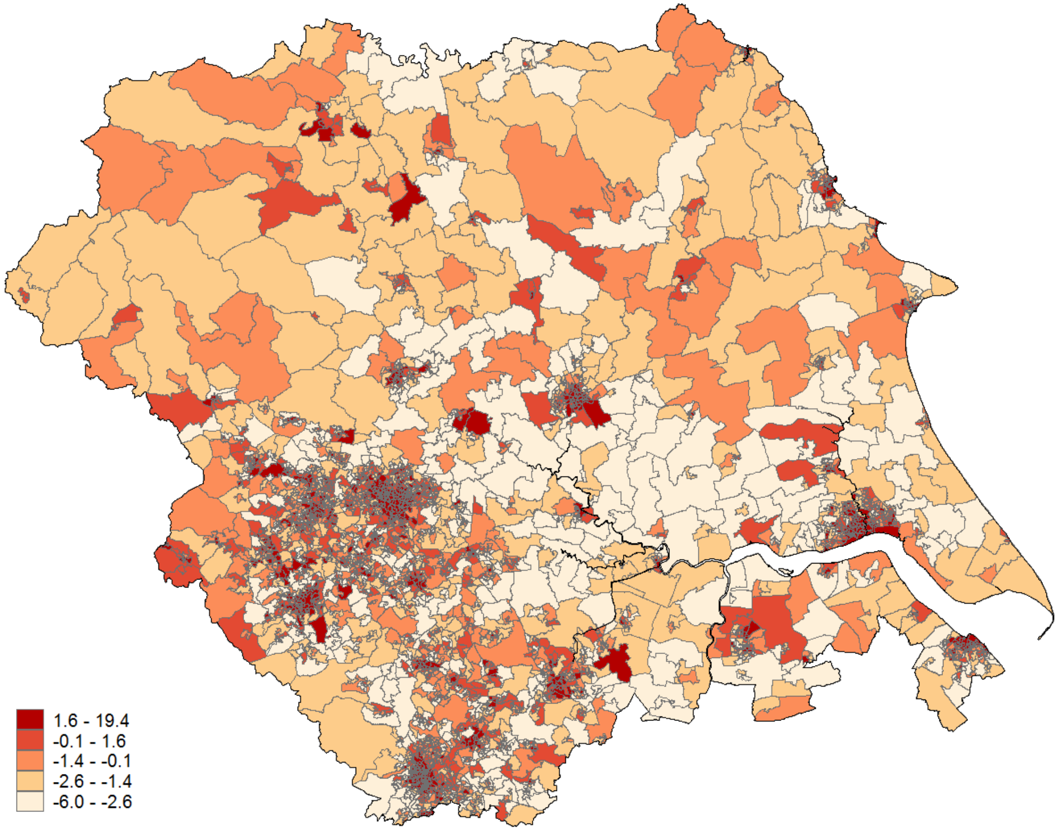

East Midlands, Social Fragmentation

2001

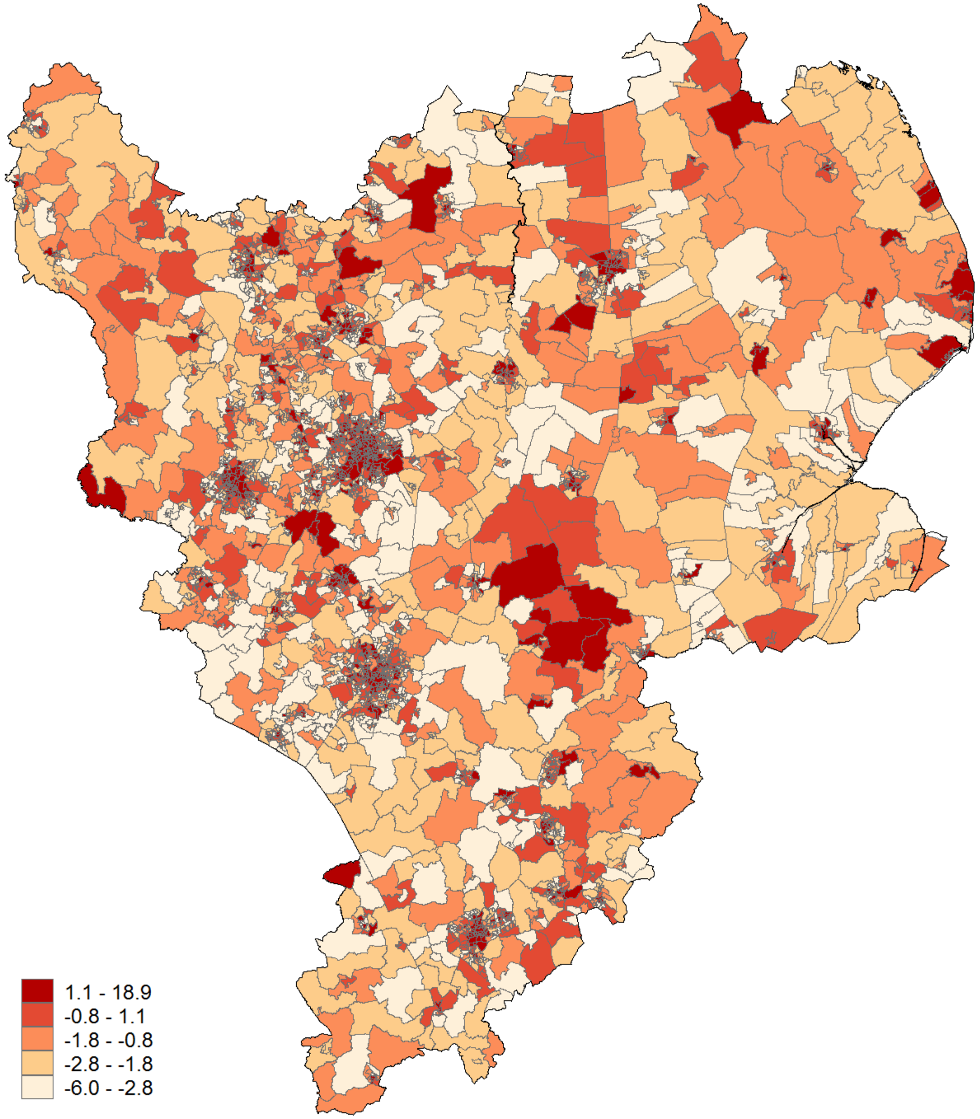

2011

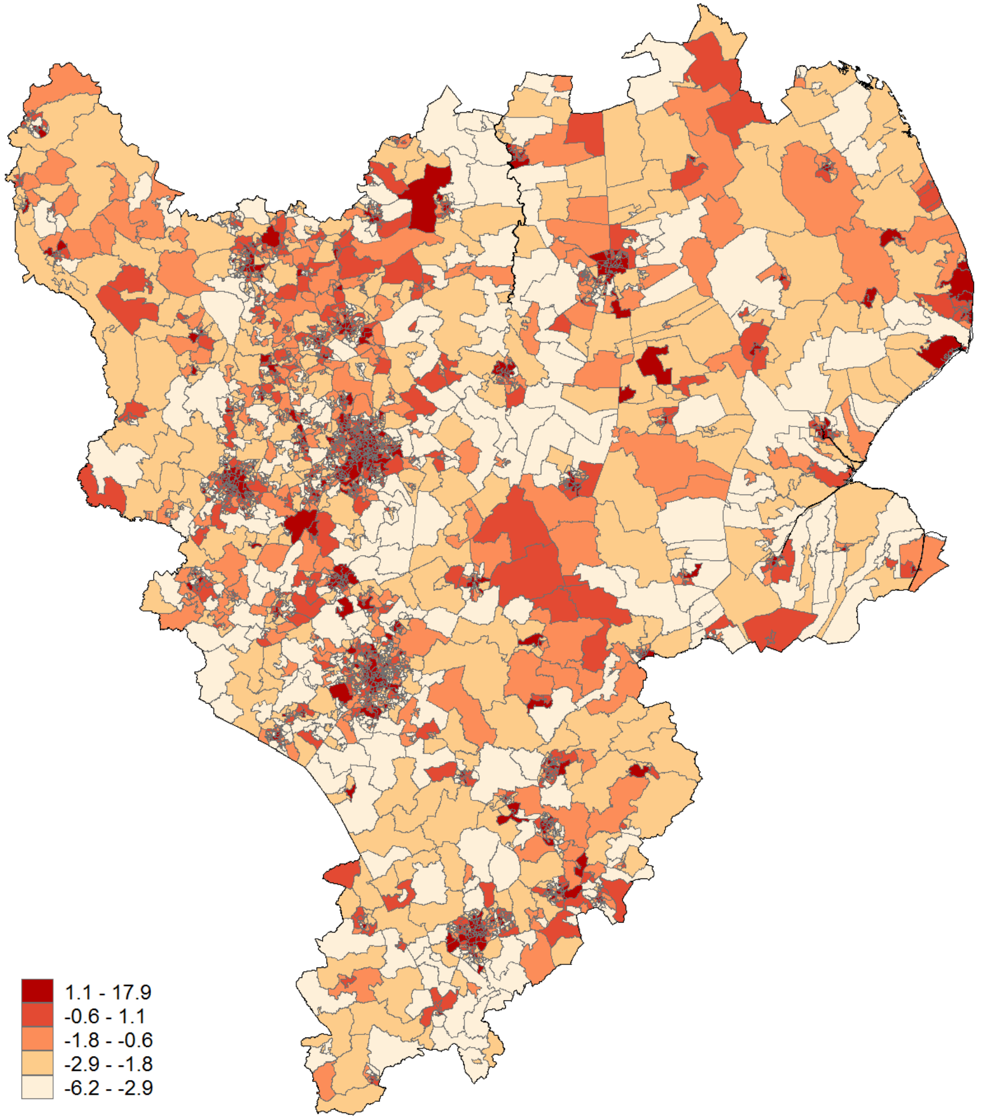

West Midlands, Social Fragmentation

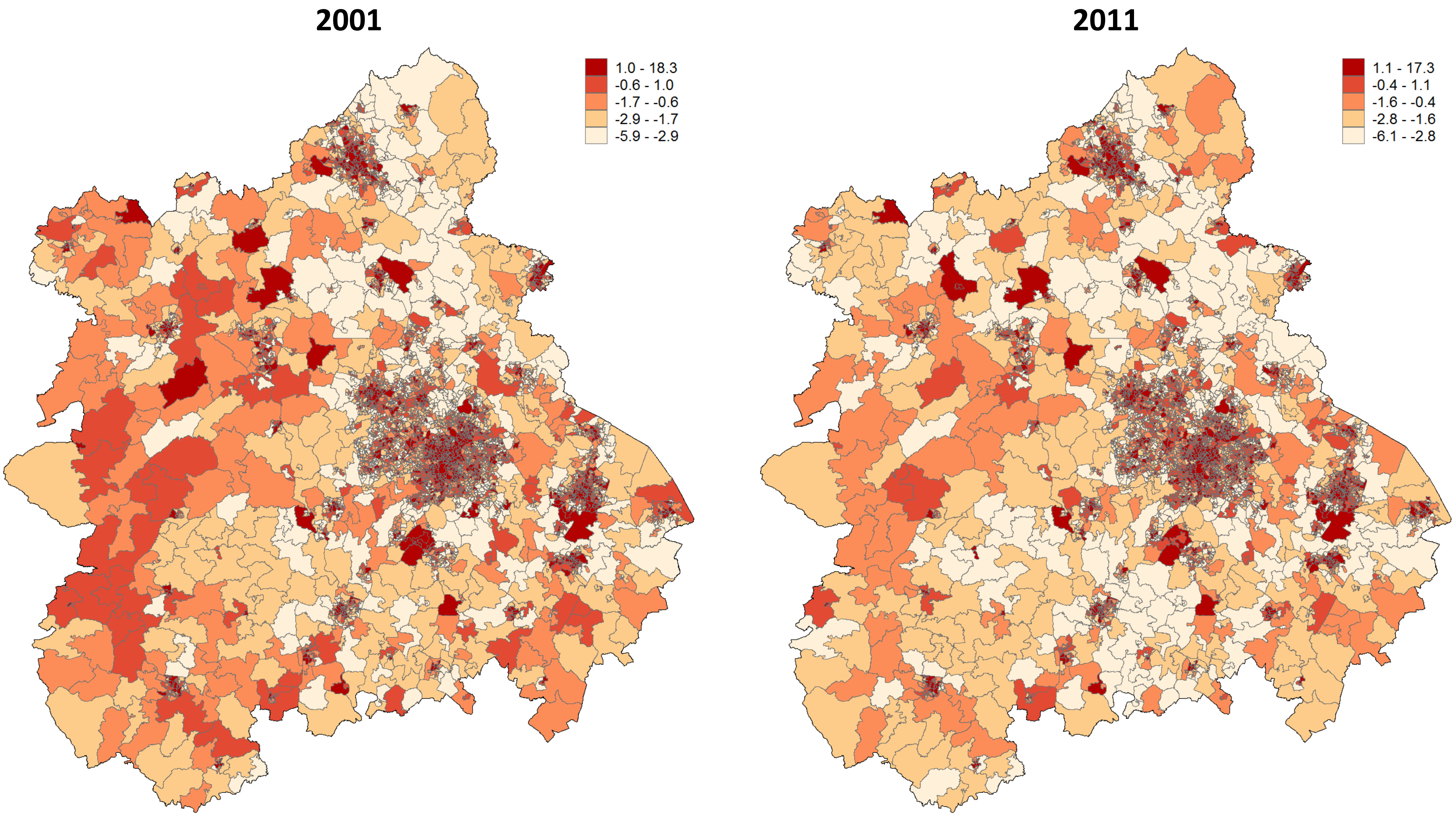

East of England, Social Fragmentation

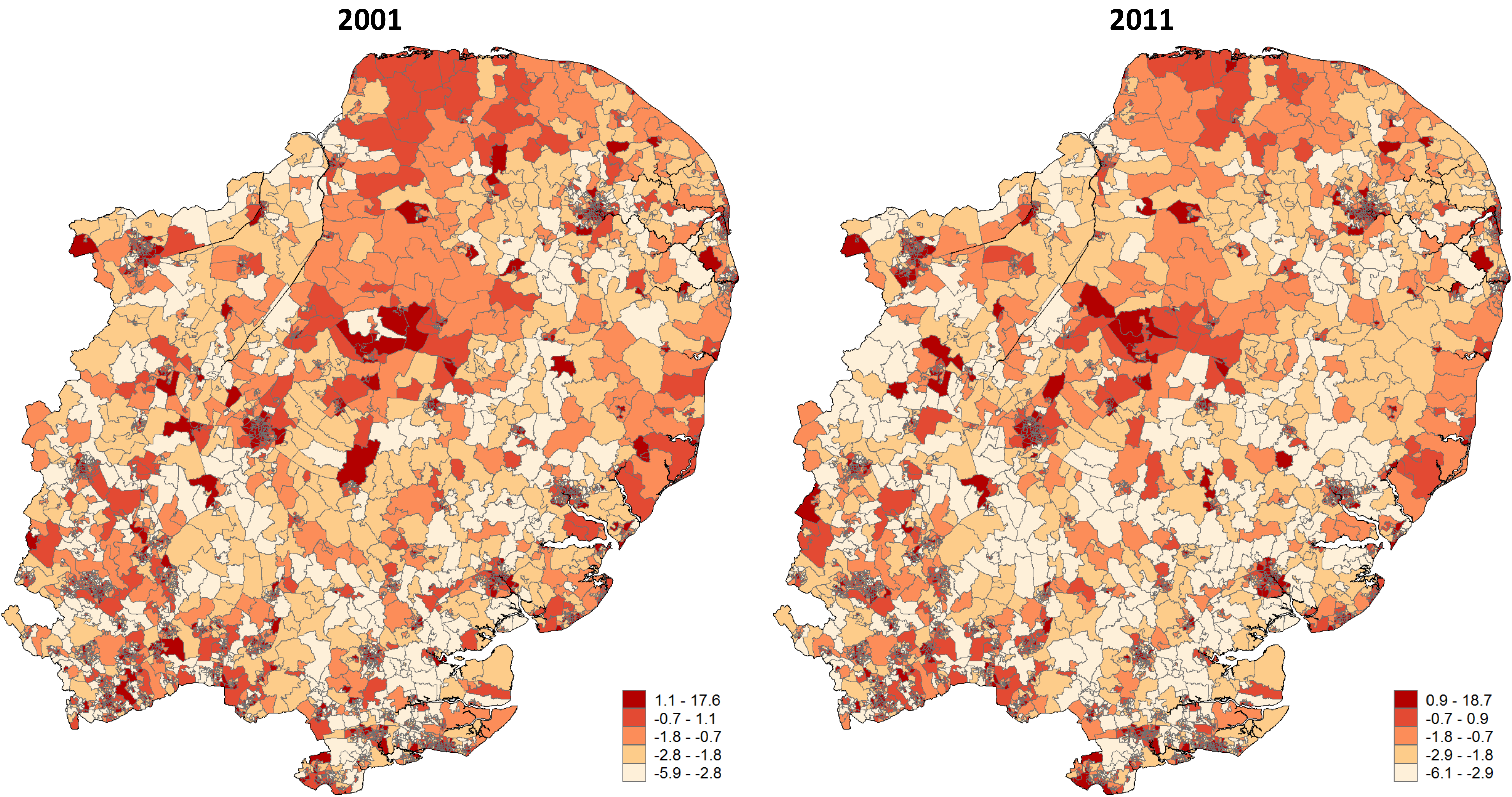

London, Social Fragmentation

2001

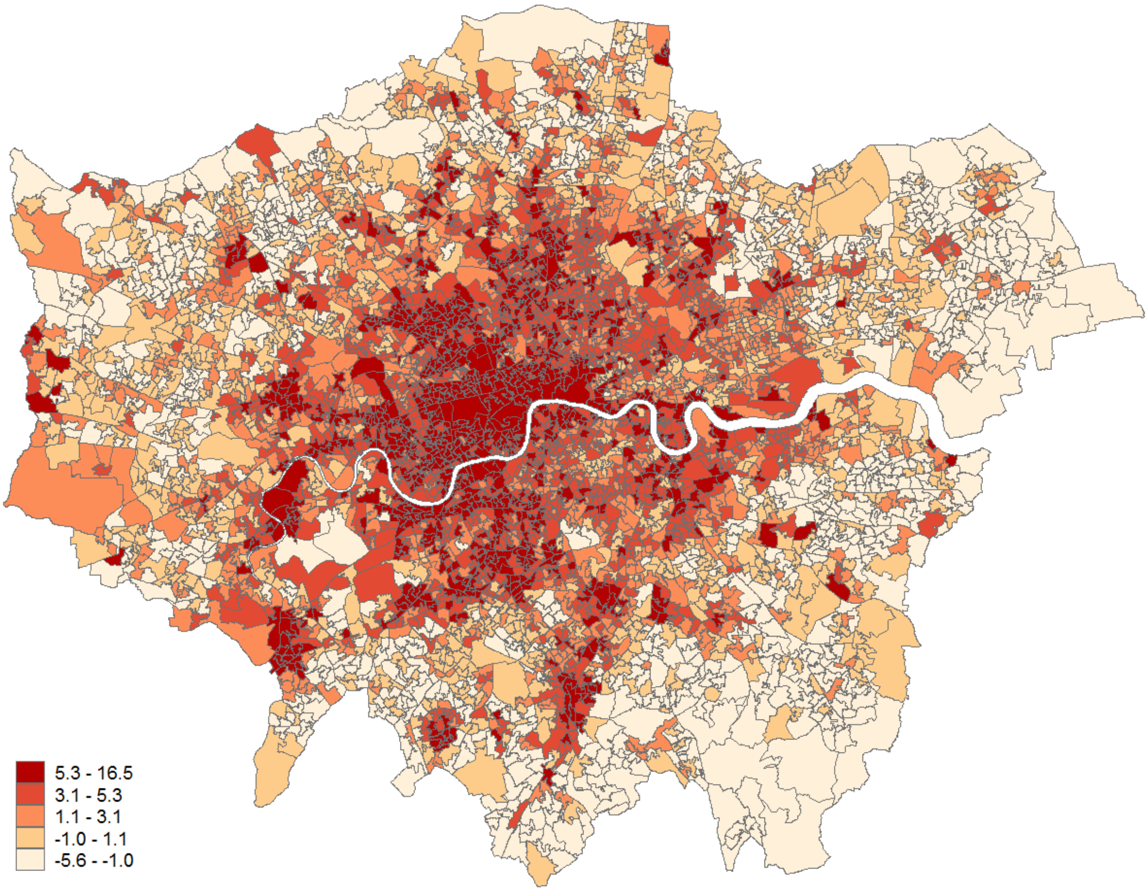

2011

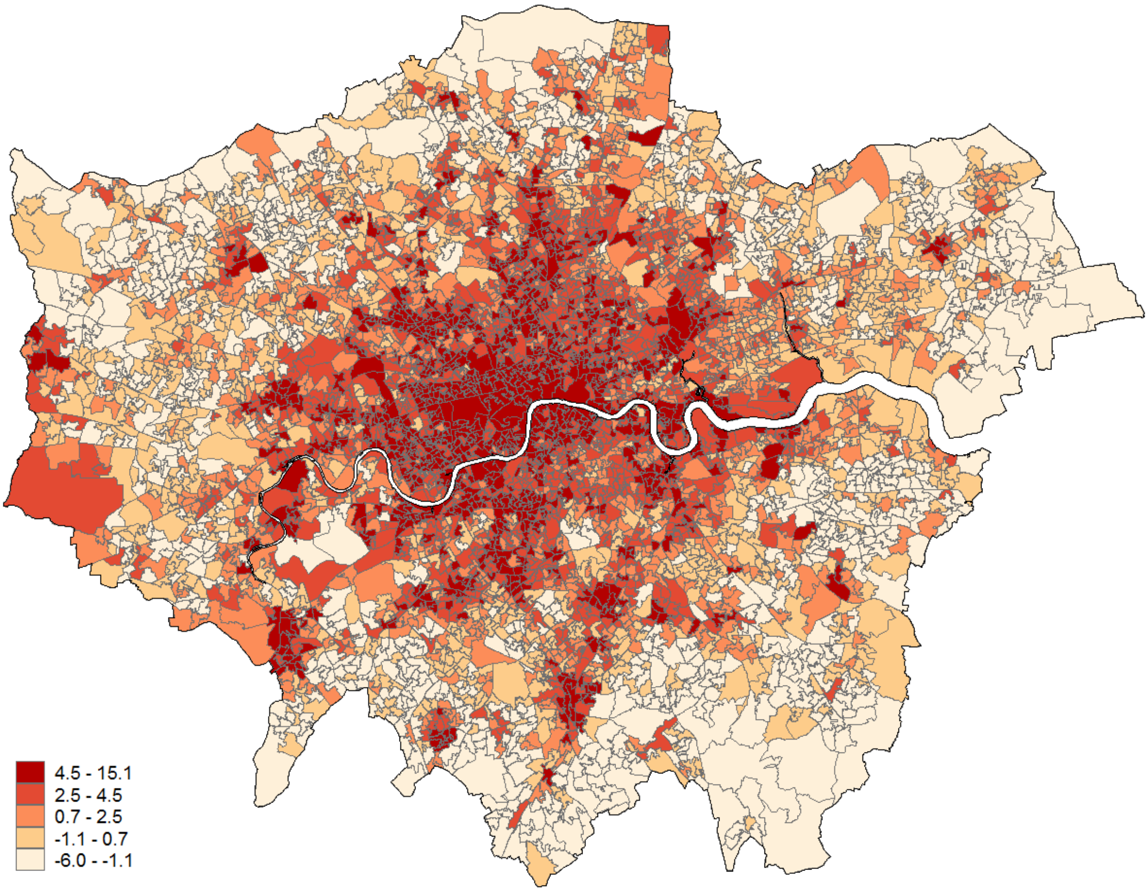

South East Coast, Social Fragmentation

2001

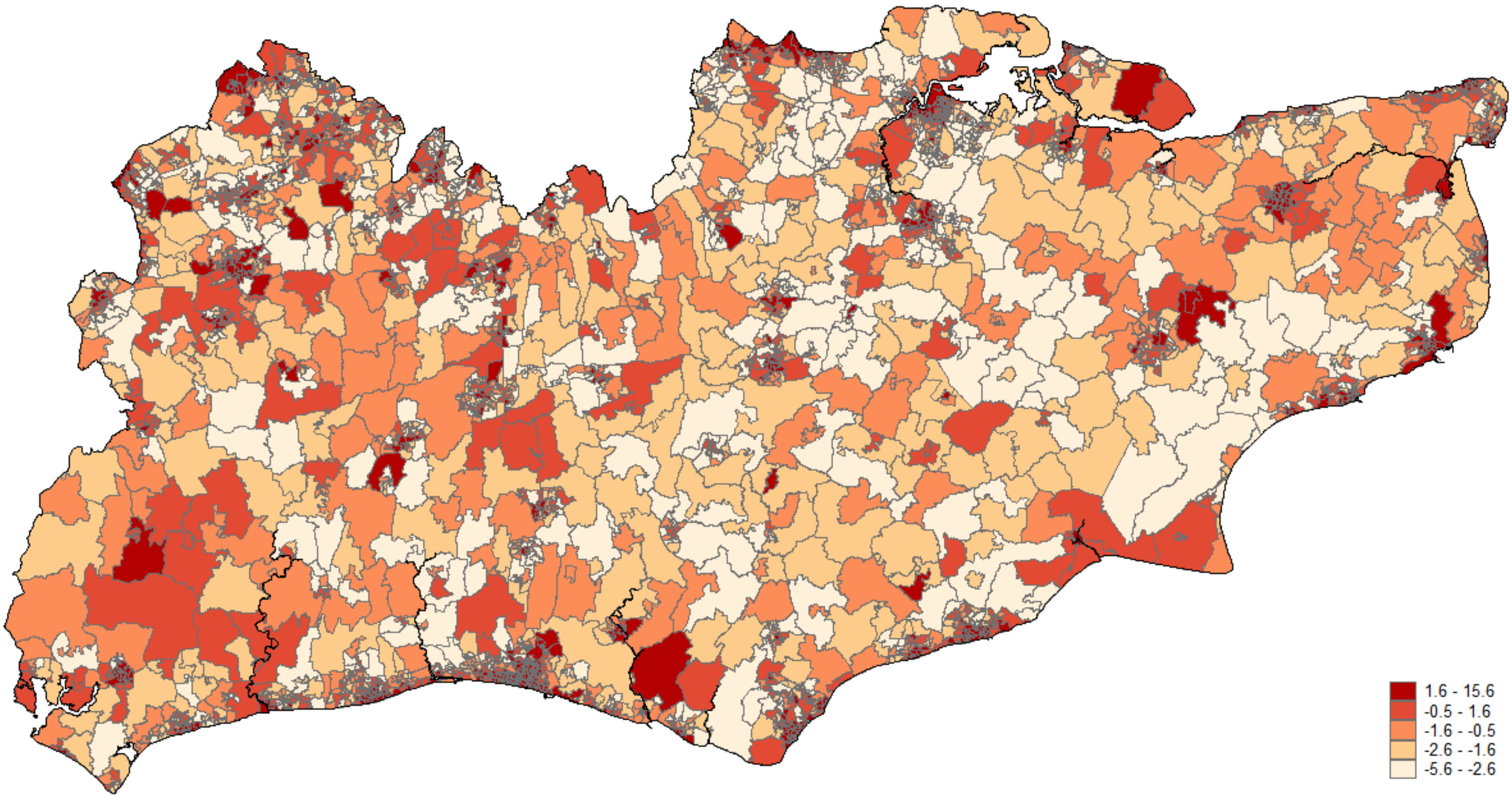

2011

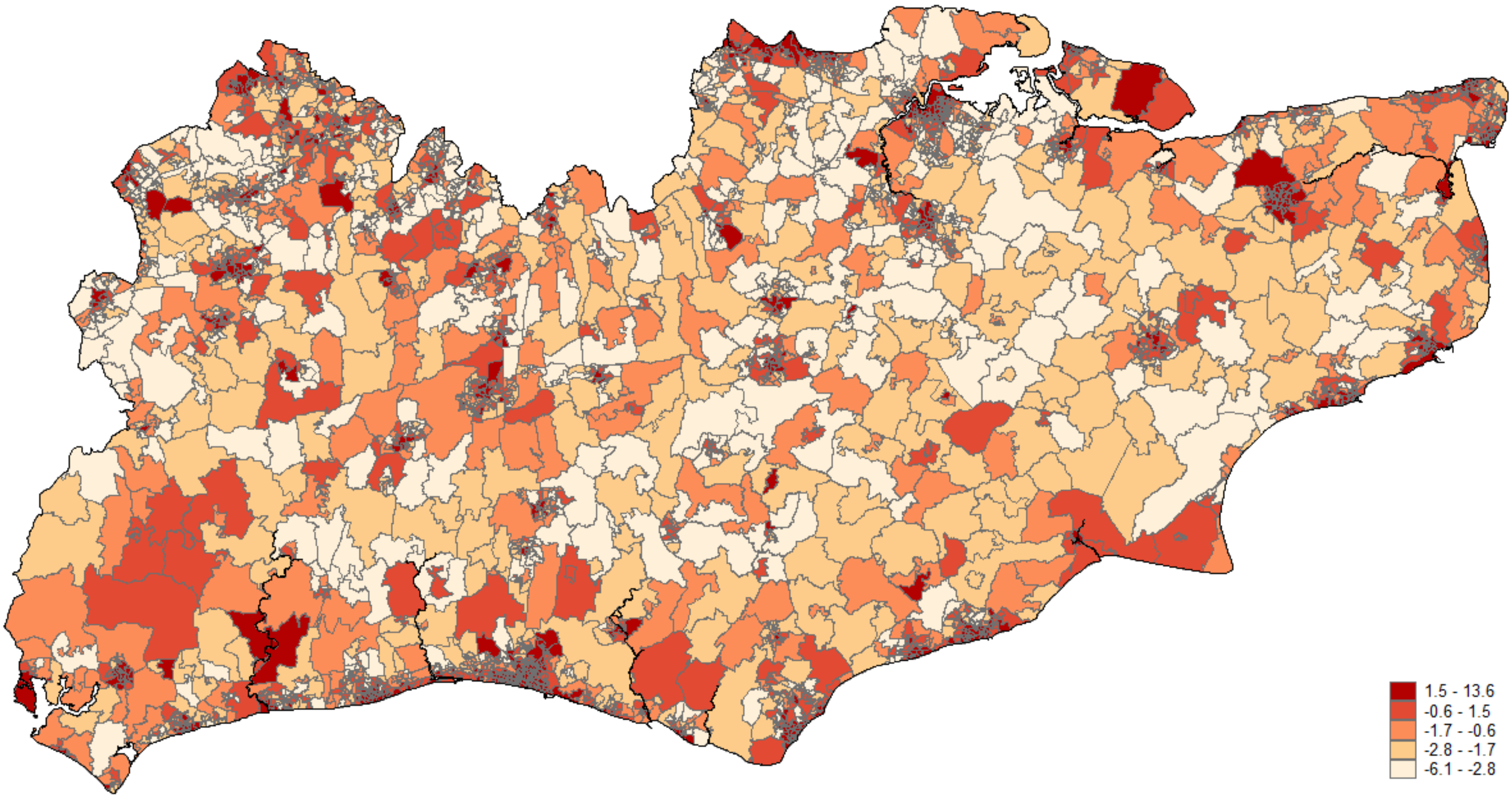

South Central, Social Fragmentation

2001

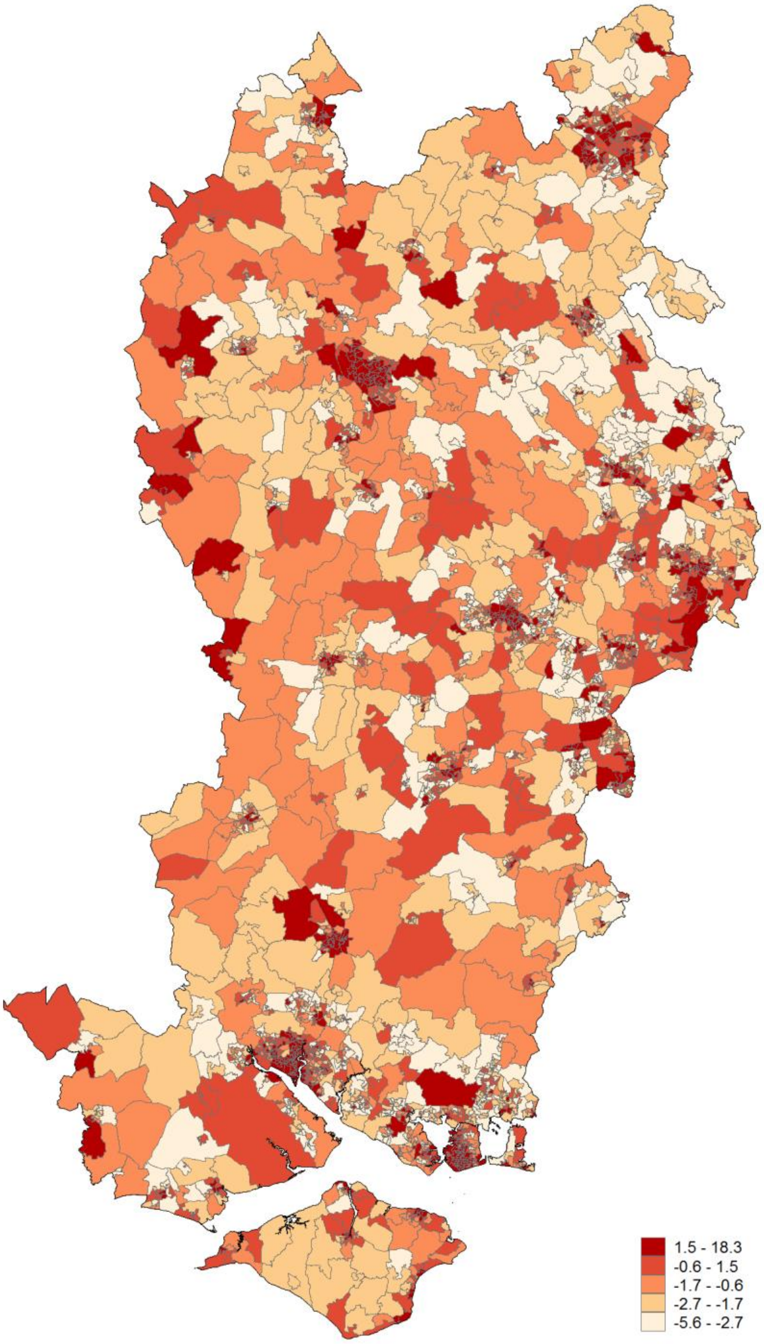

2011

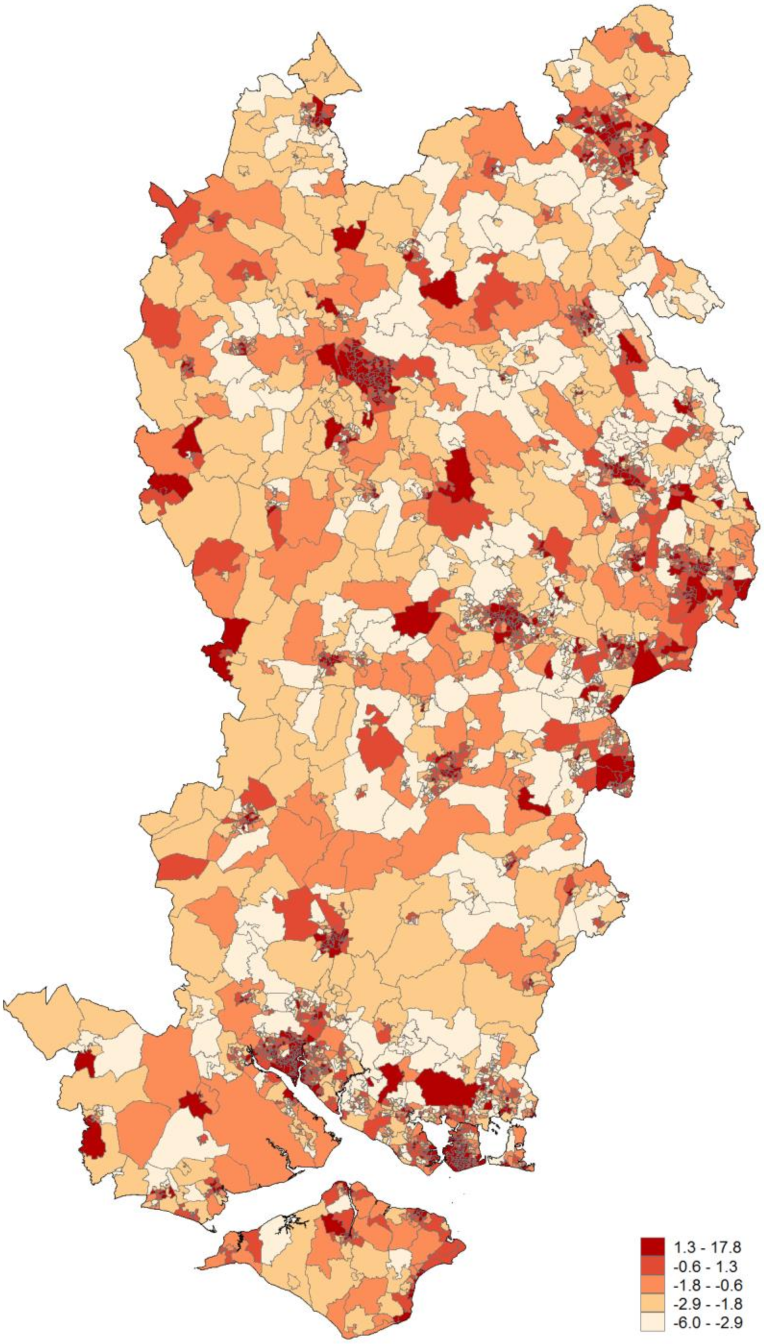

South West, Social Fragmentation

2001

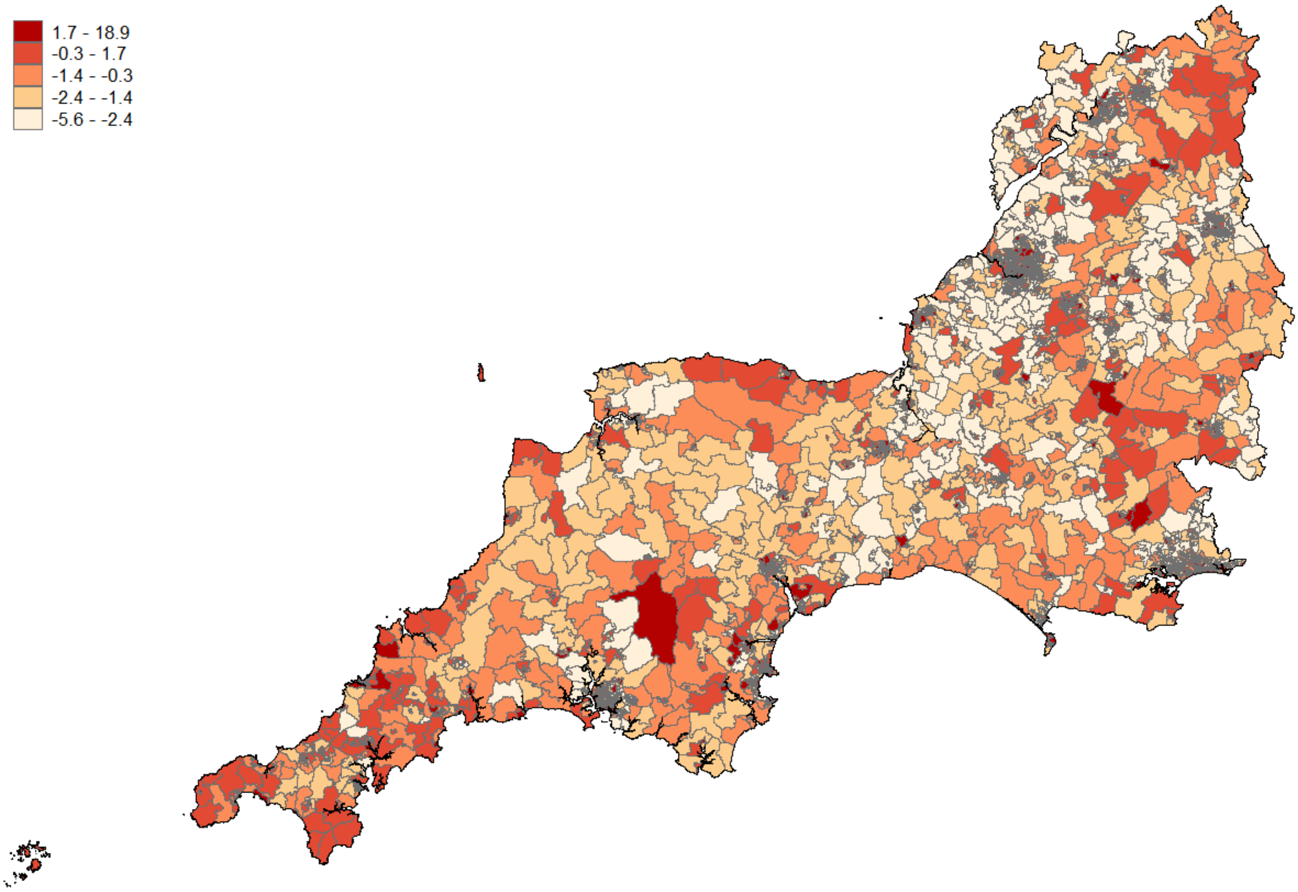

2011

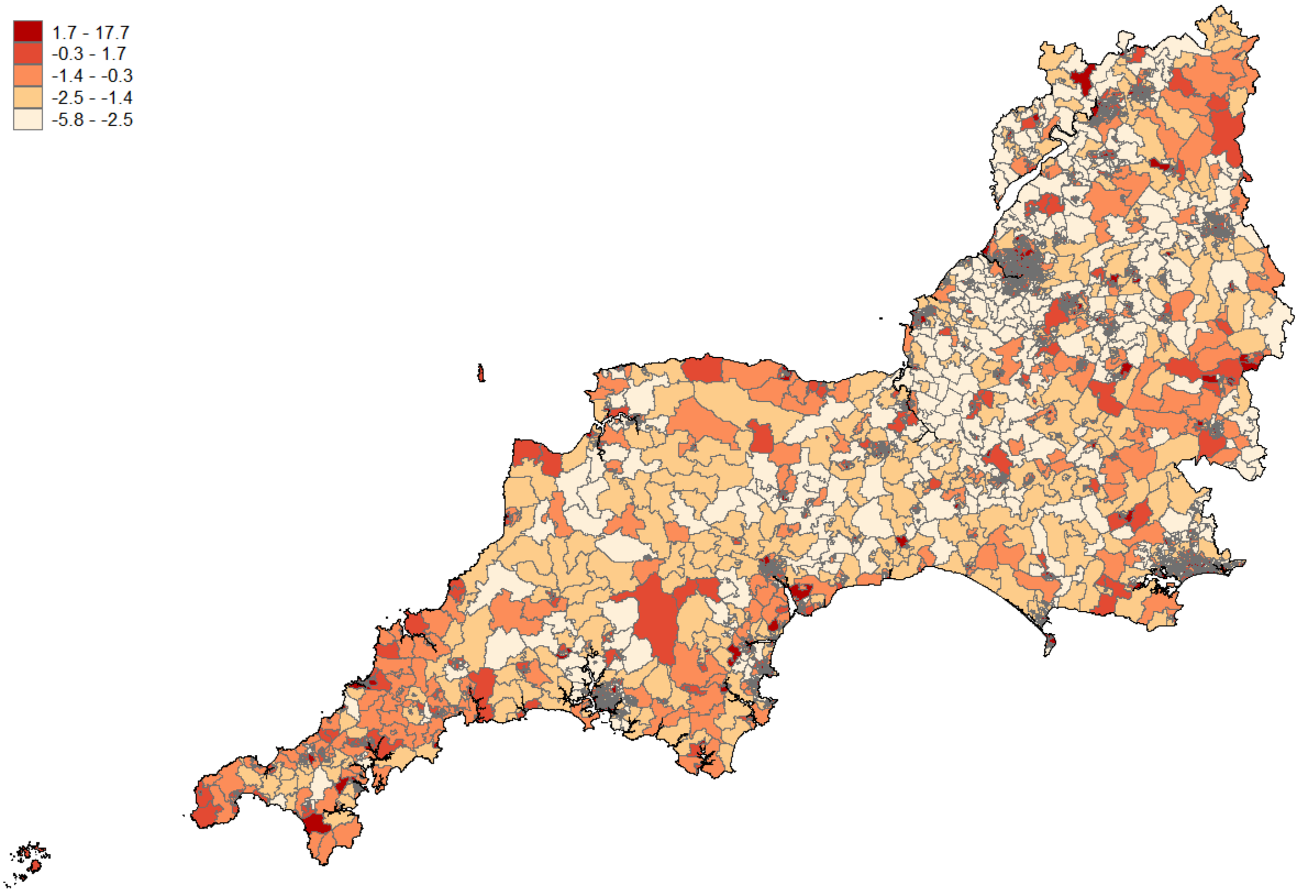

Single People

England 2001, Single People % of population

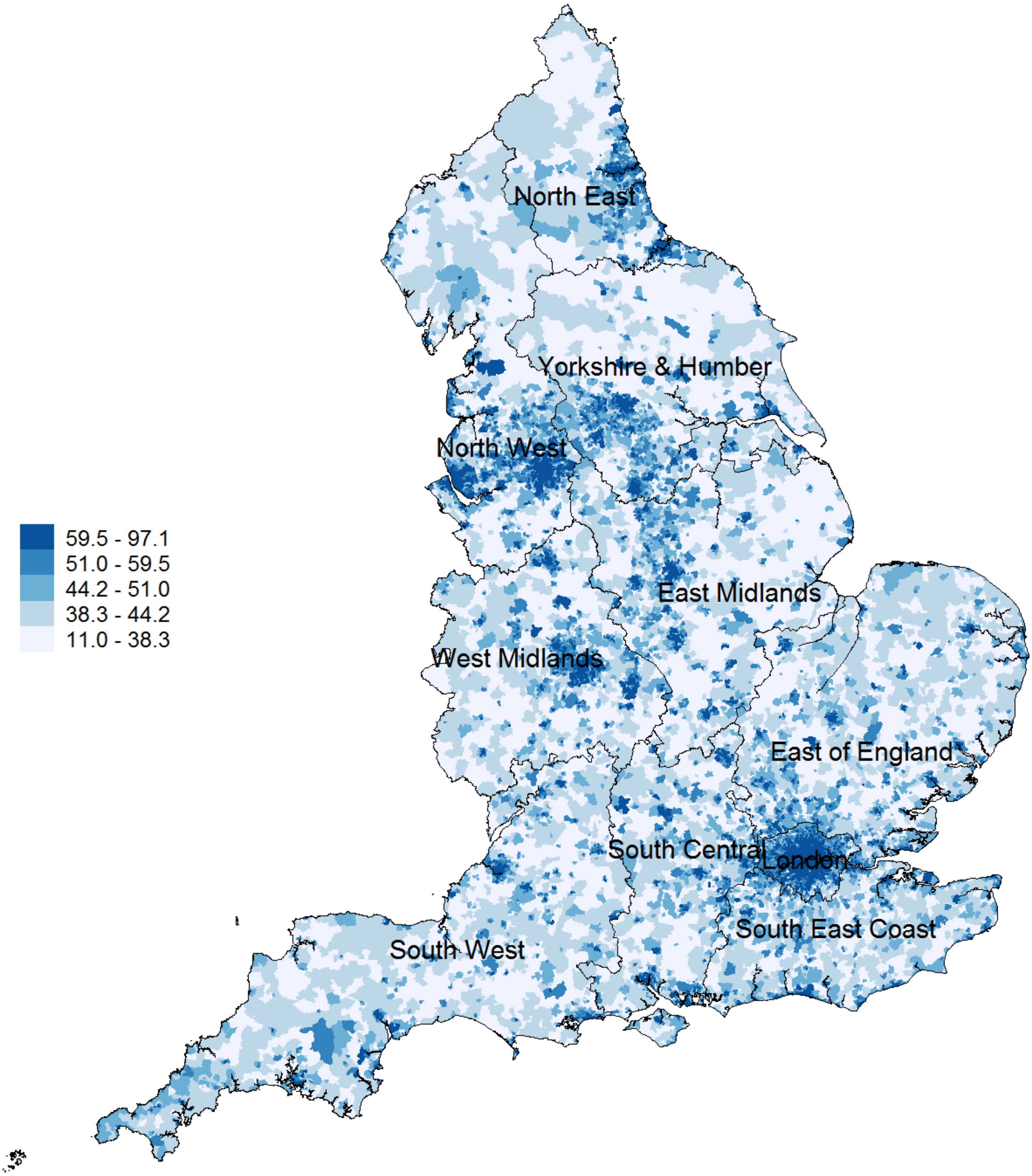

England 2011, Single People % of population

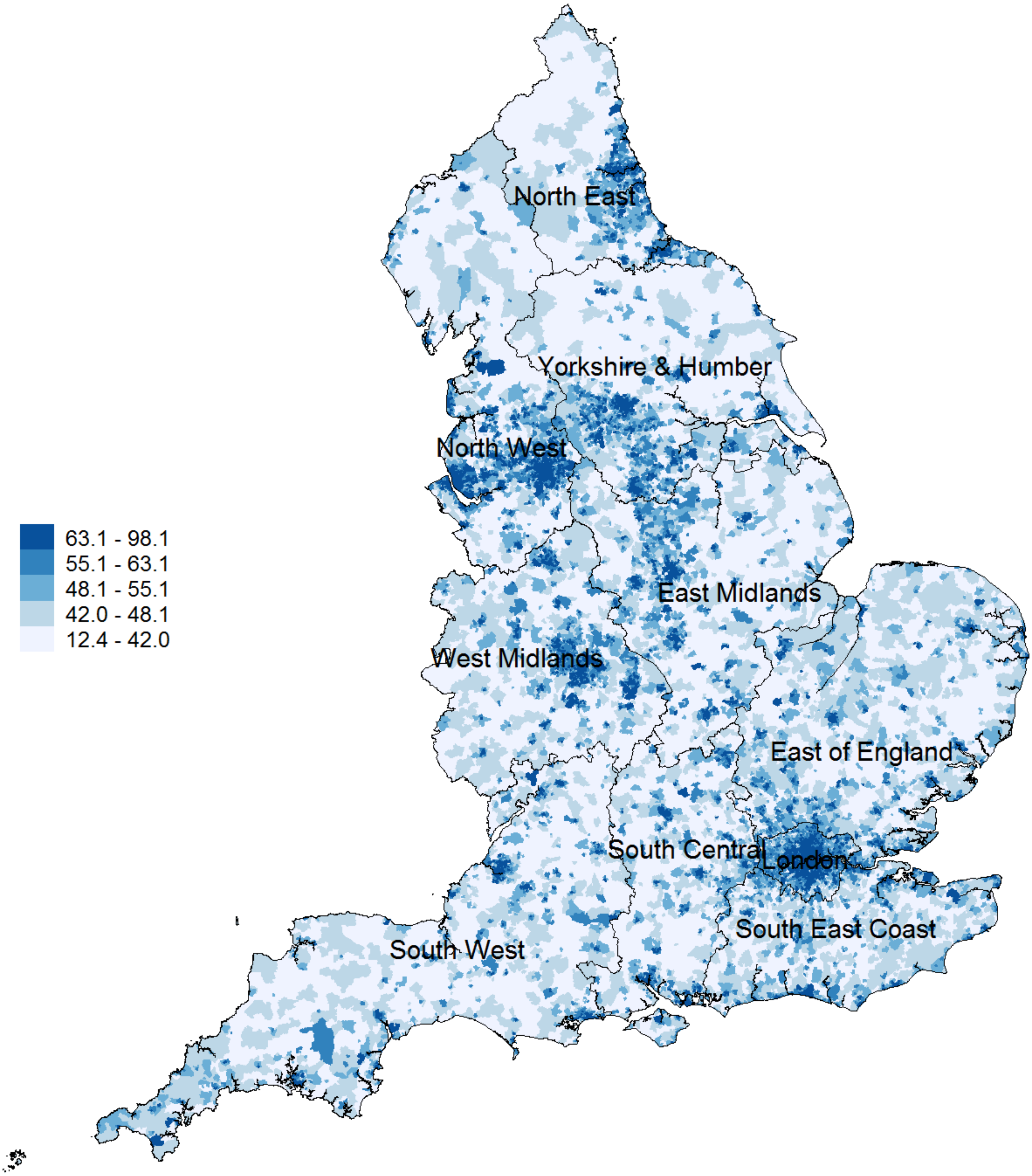

North East, Single People % of population

2001

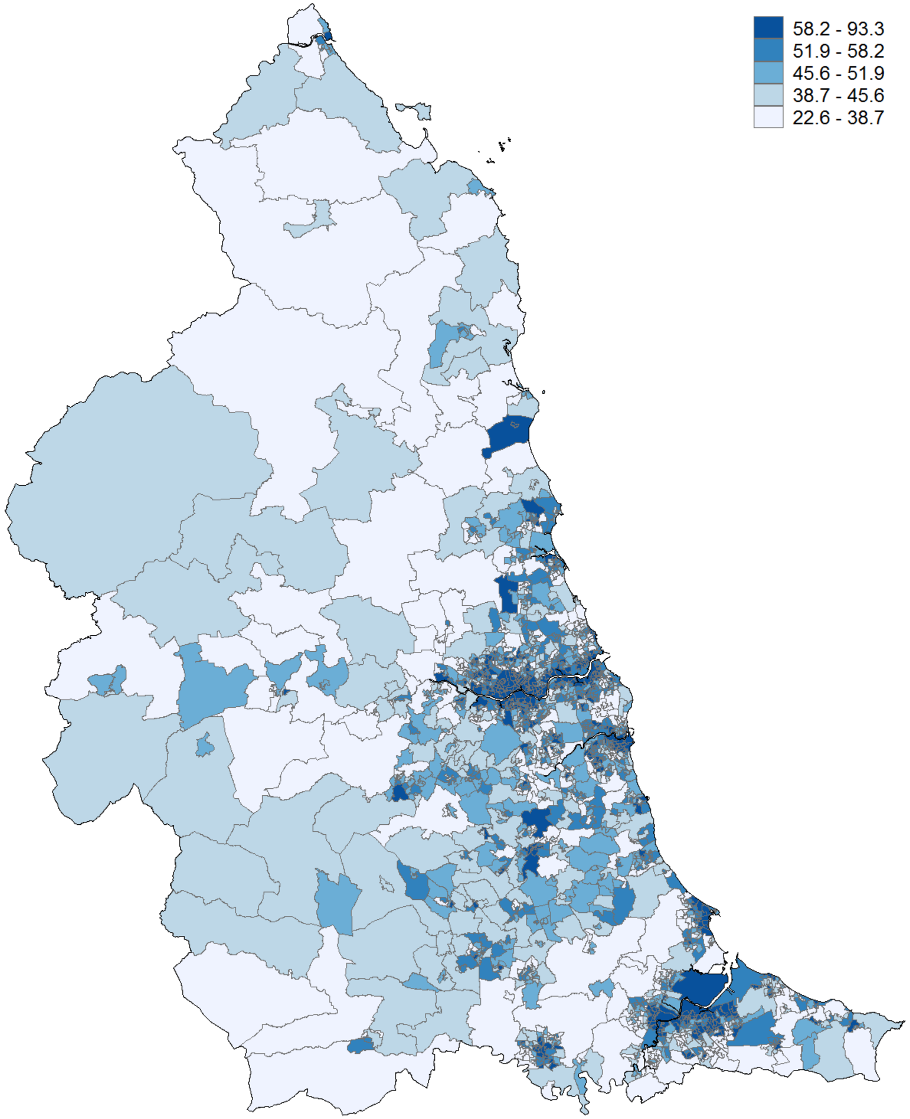

2011

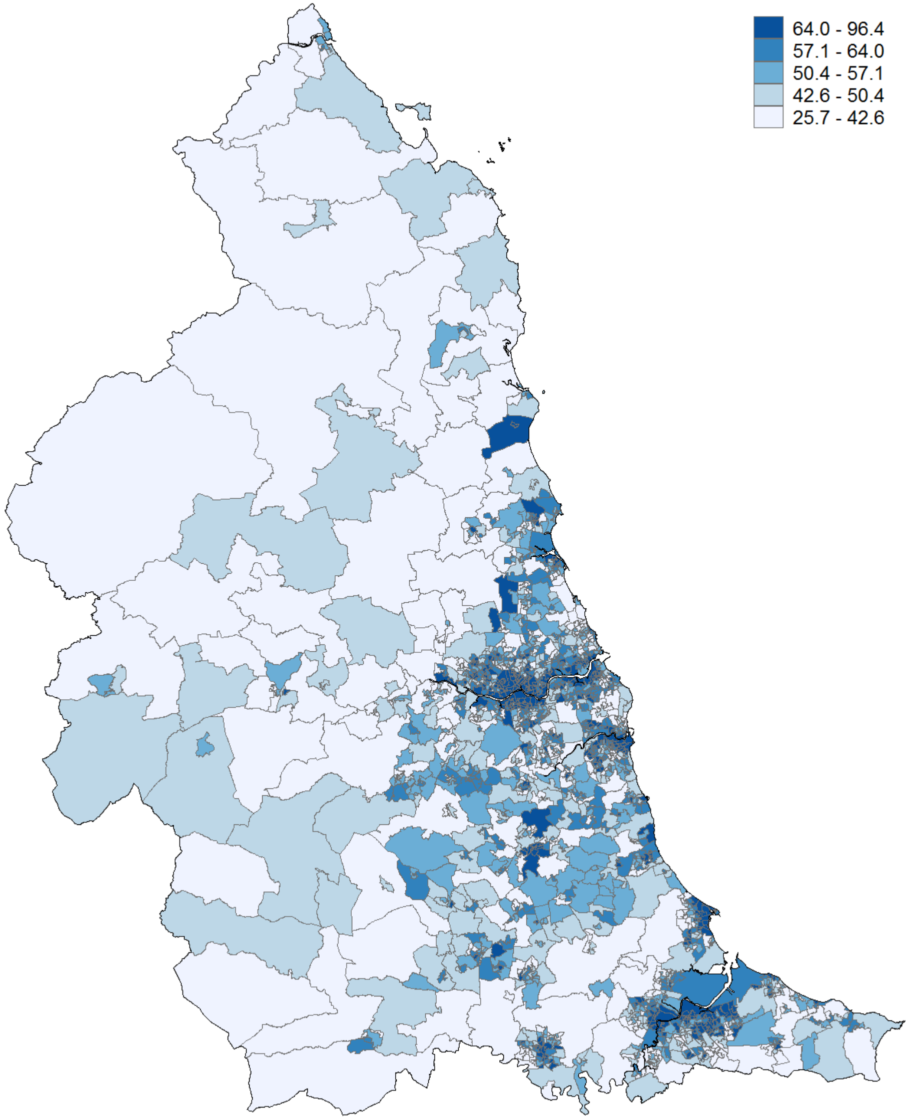

North West, Single People % of population

2001

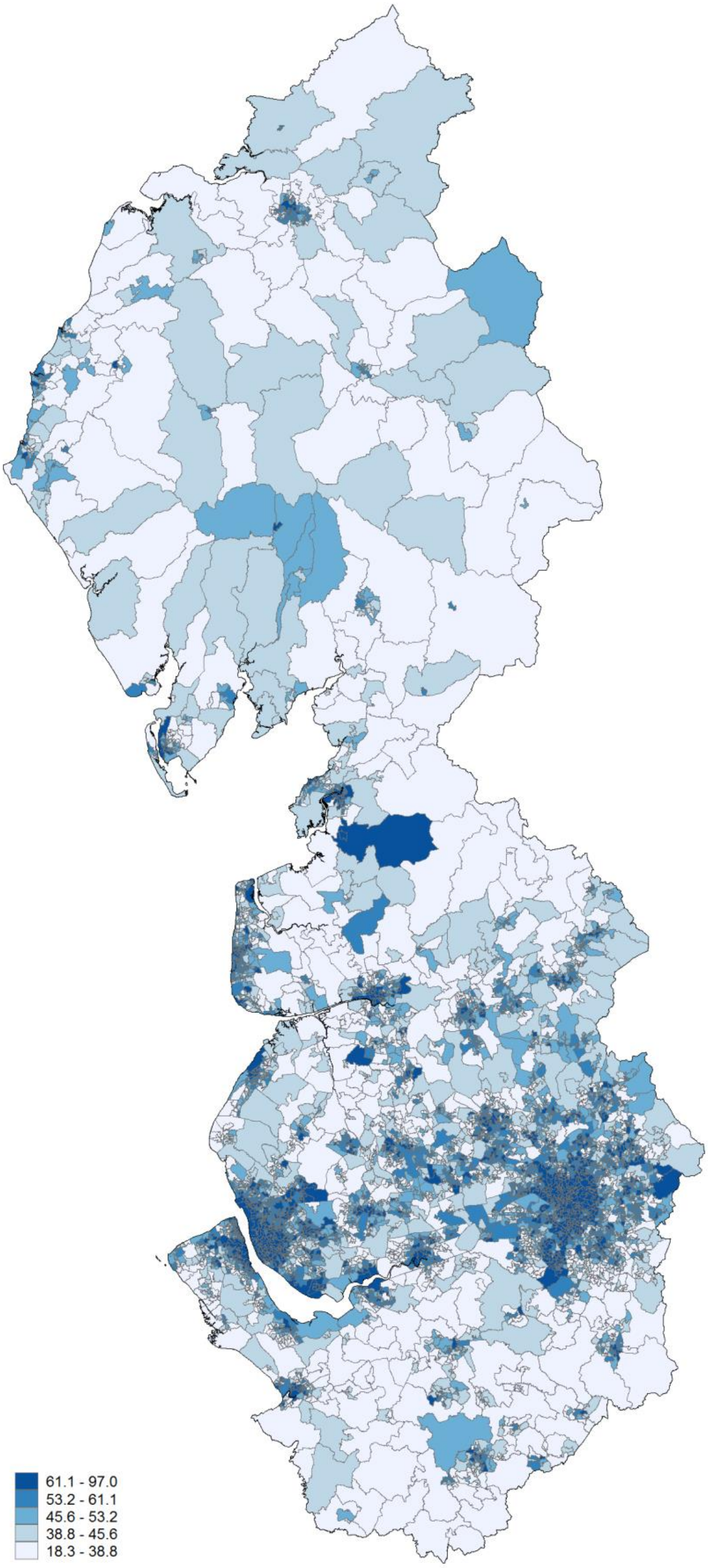

2011

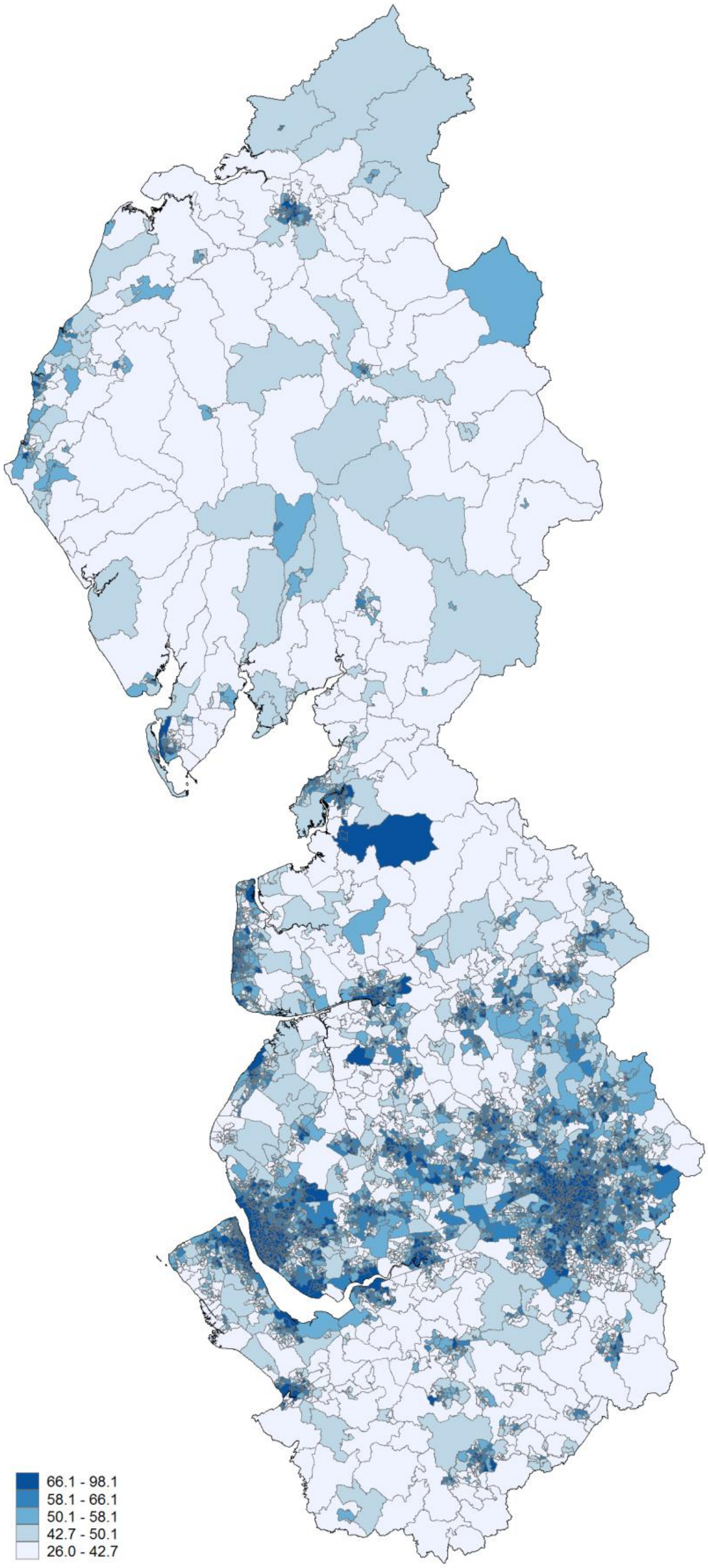

Yorkshire and the Humber, Single People % of population

2001

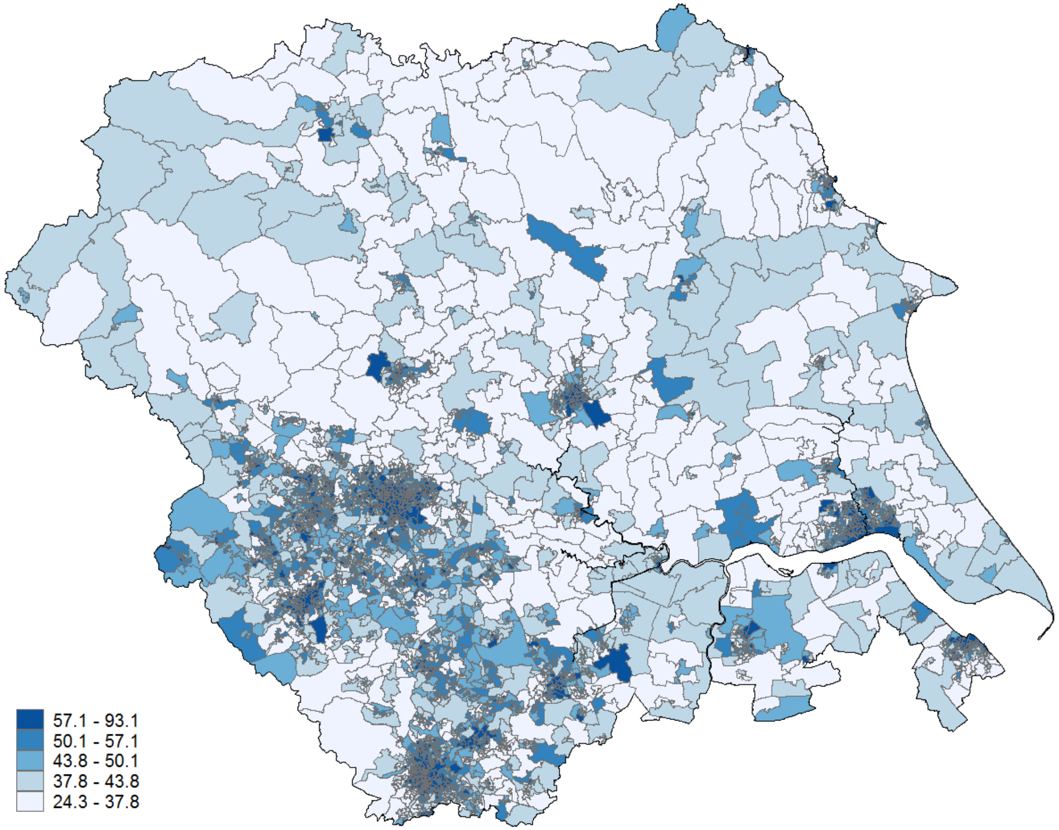

2011

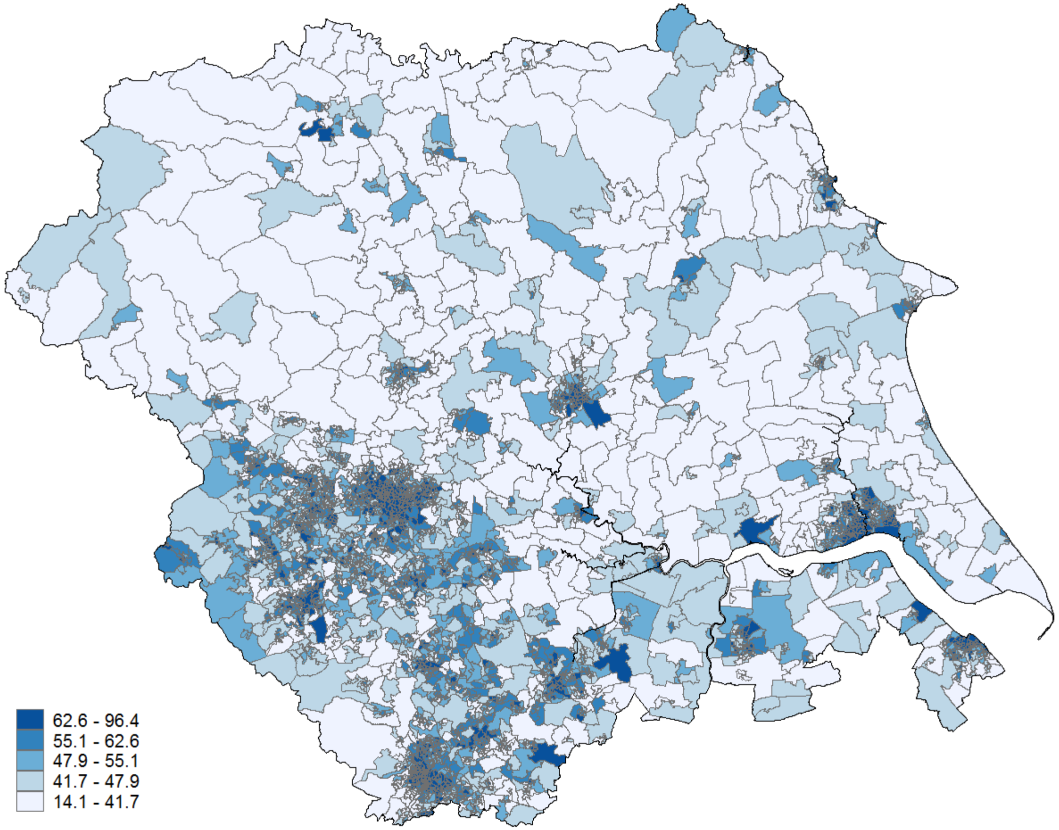

East Midlands, Single People % of population

2001

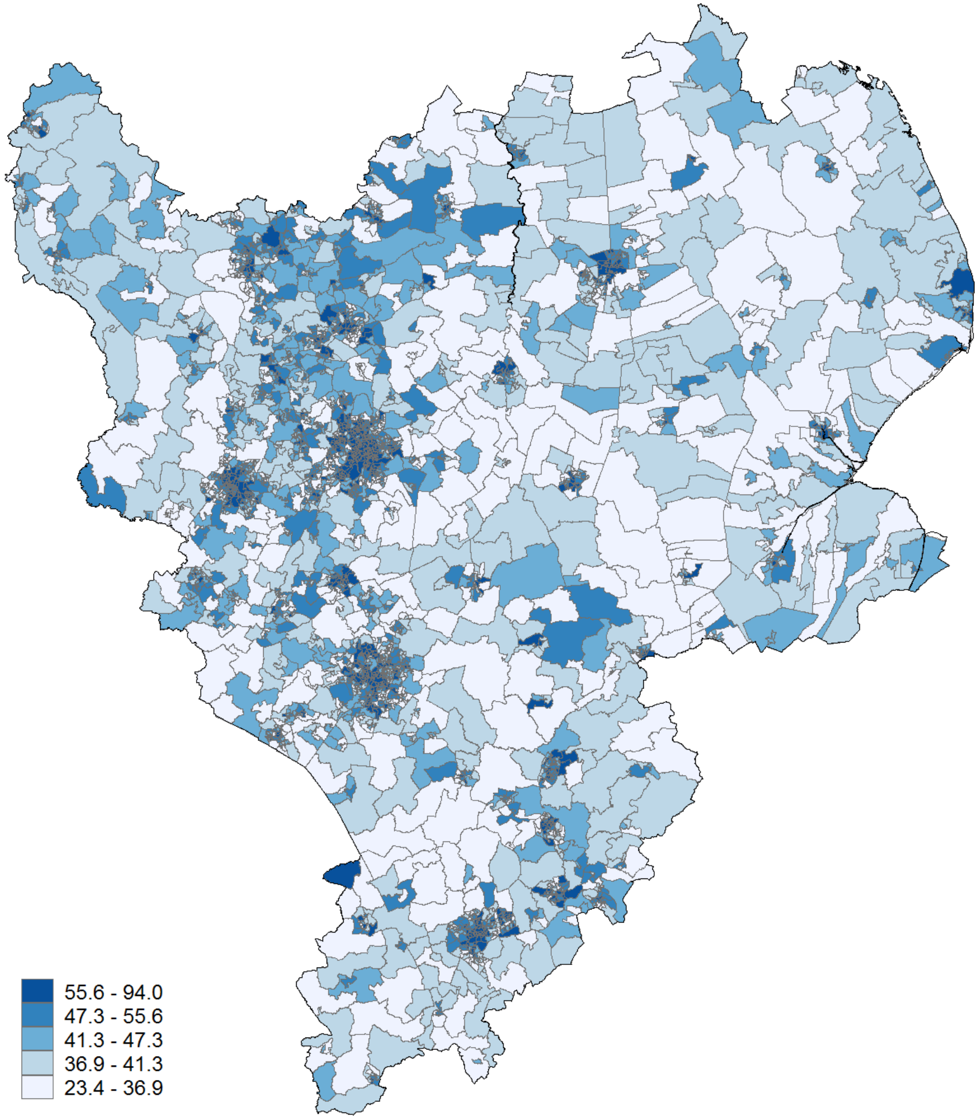

2011

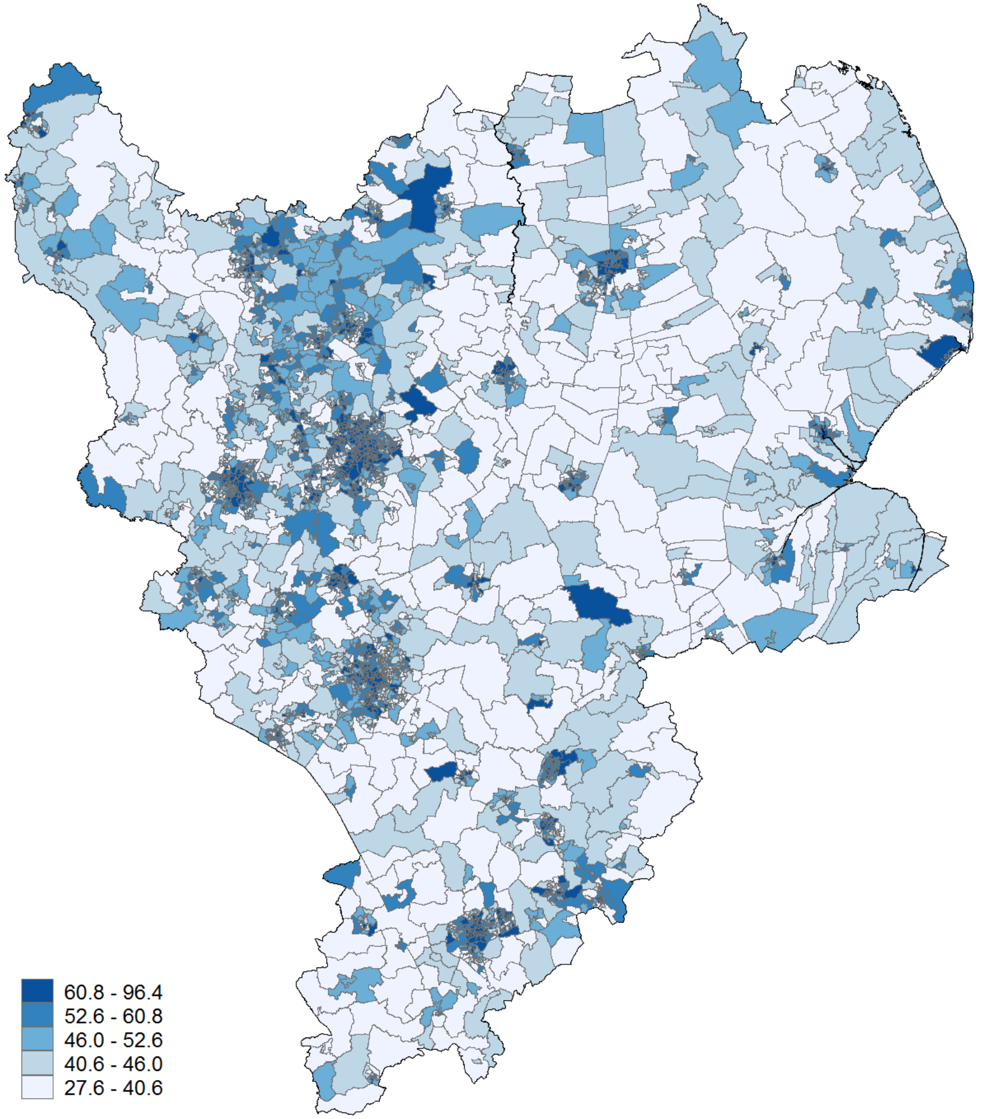

West Midlands, Single People % of population

2001

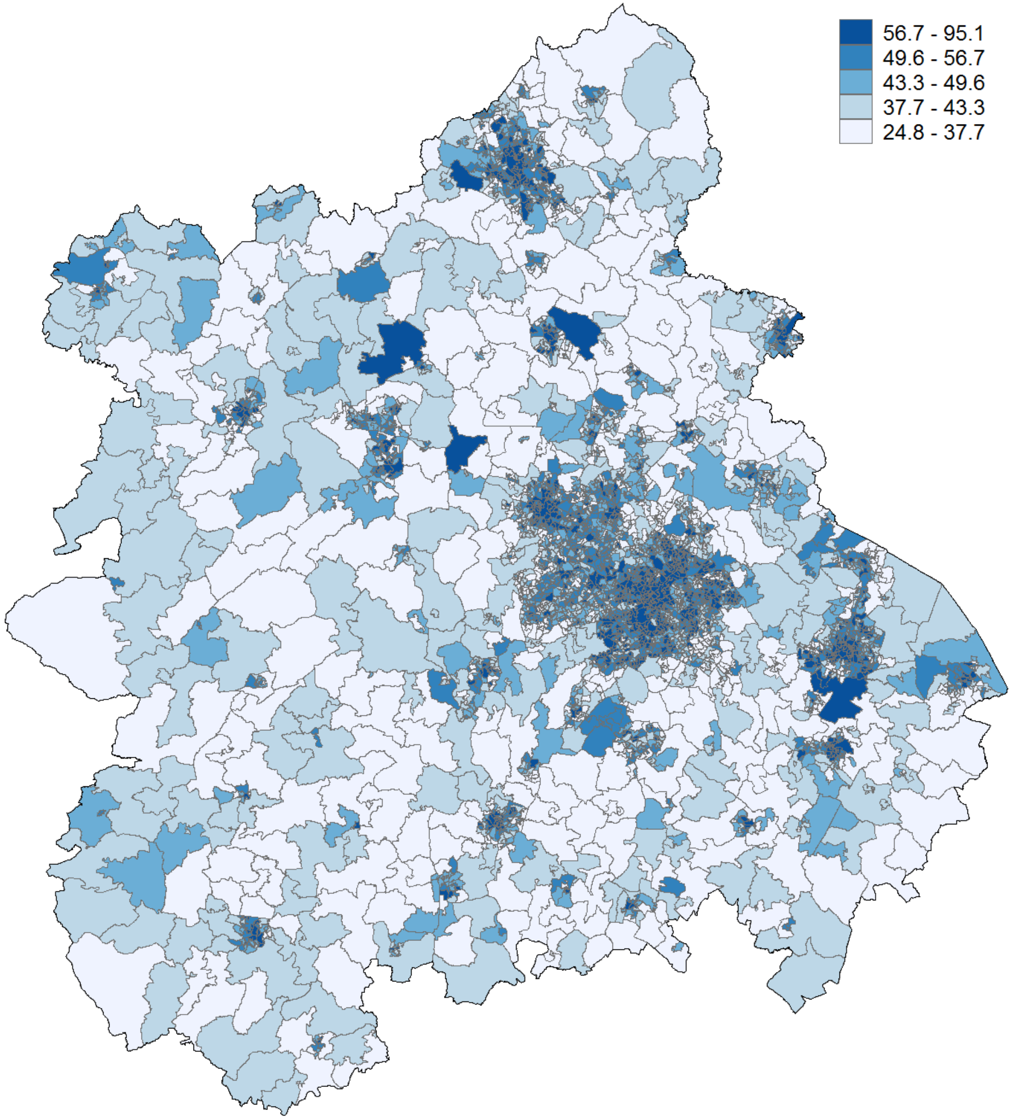

2011

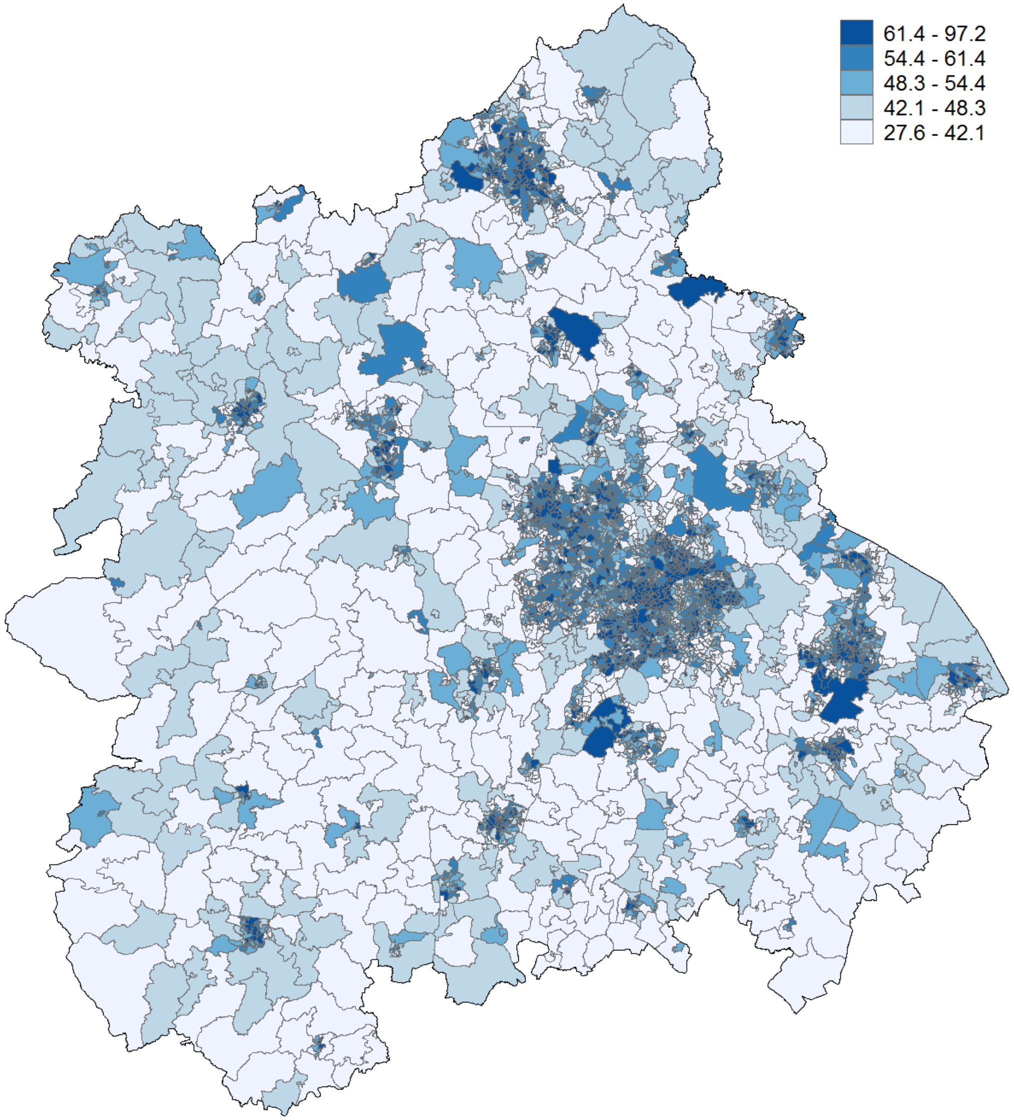

East of England, Single People % of population

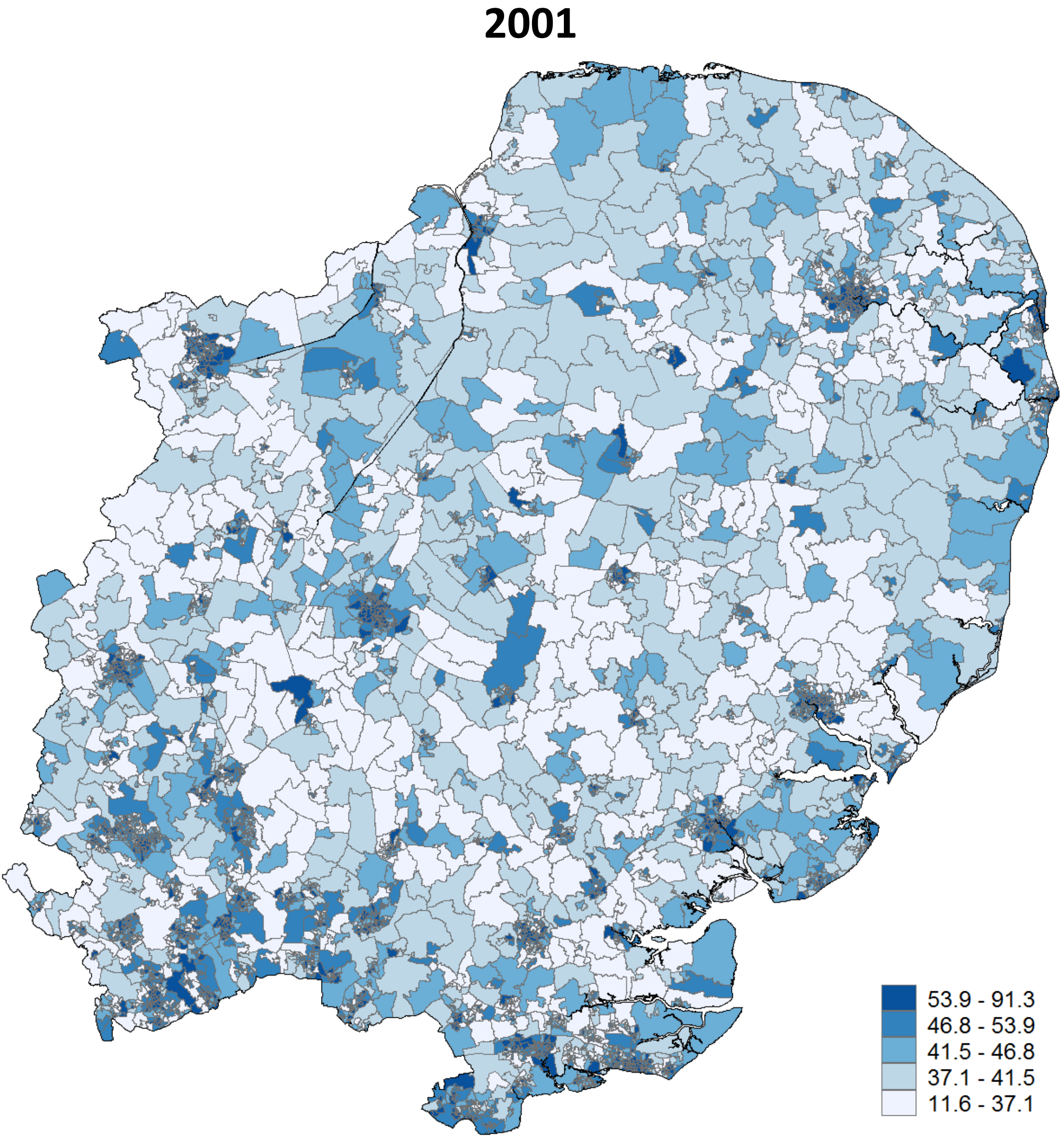

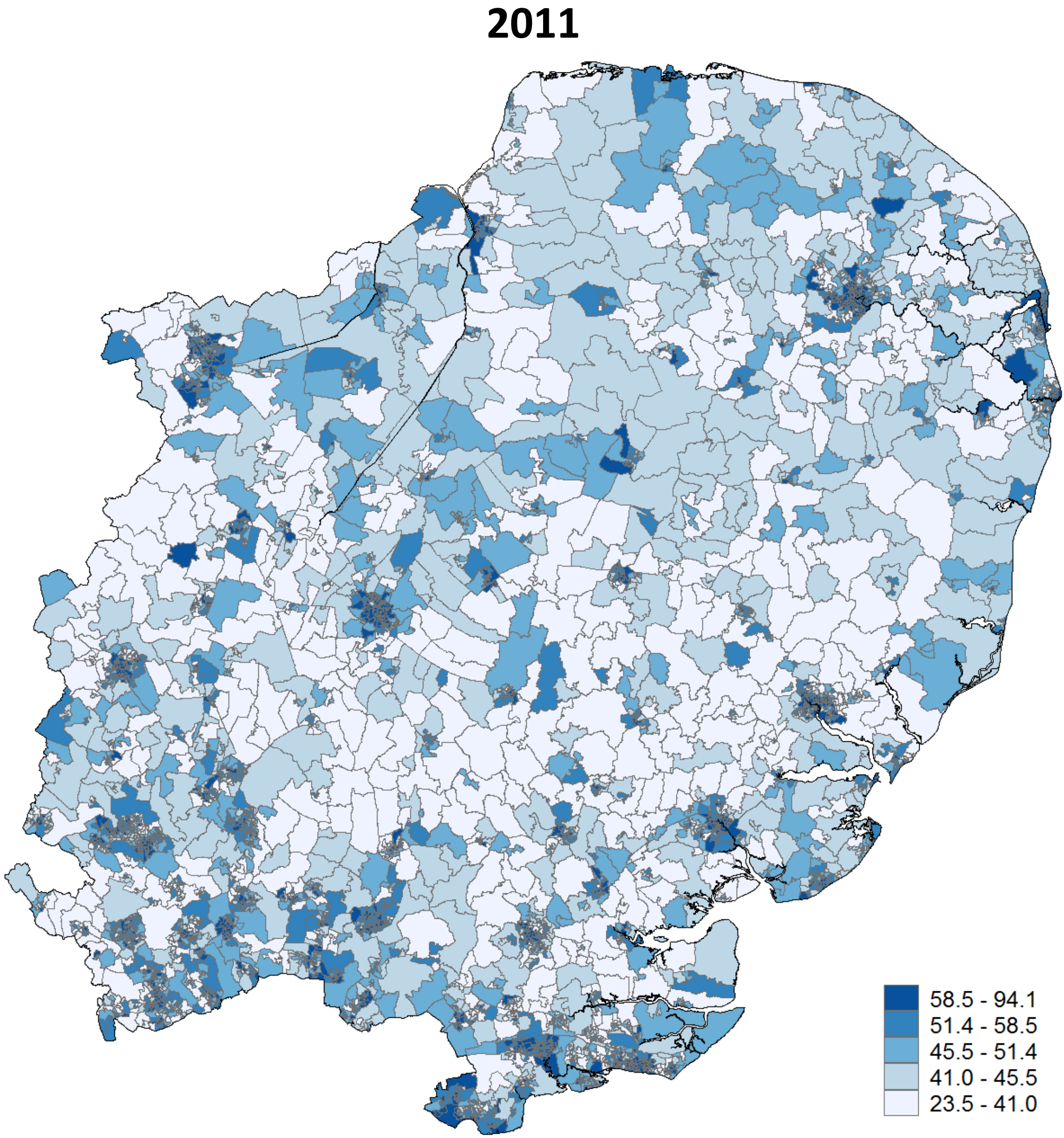

London, Single People % of population

2001

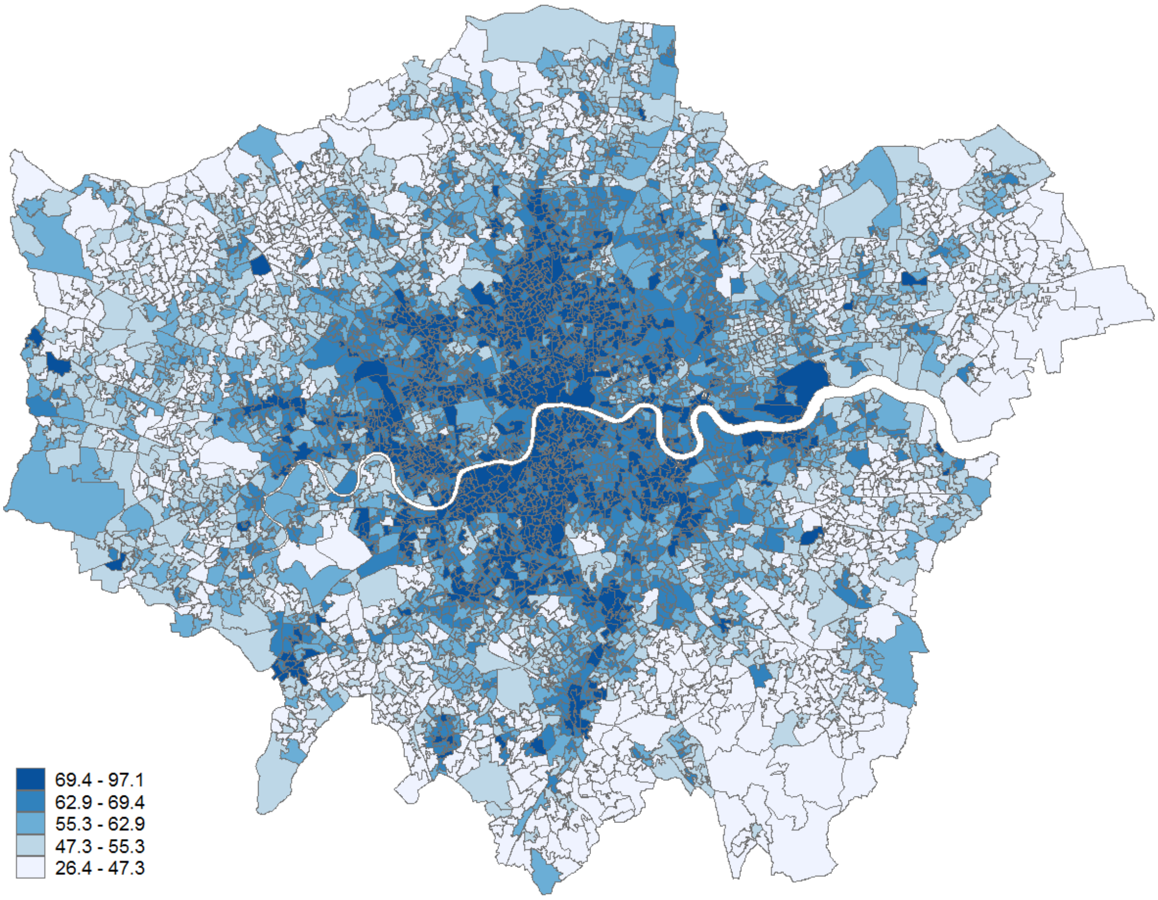

2011

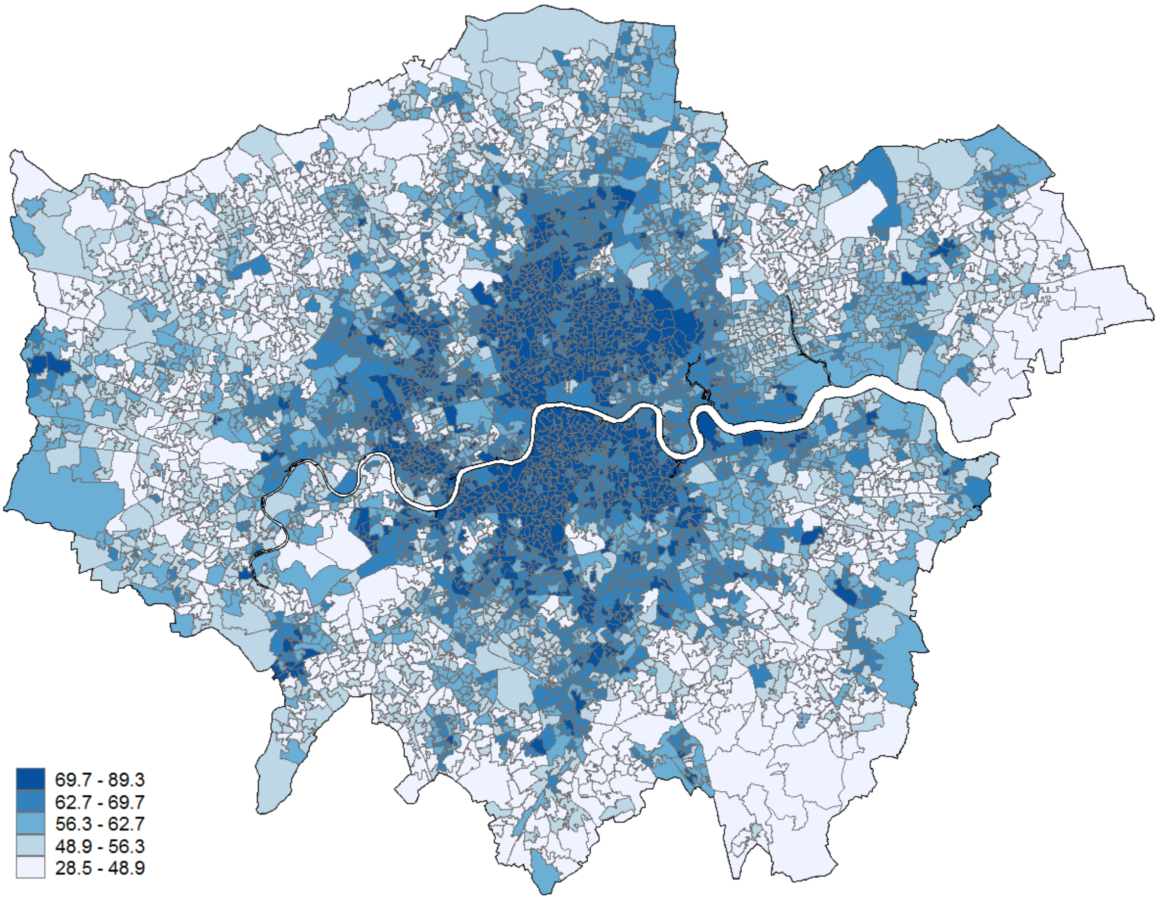

South East Coast, Single People % of population

2001

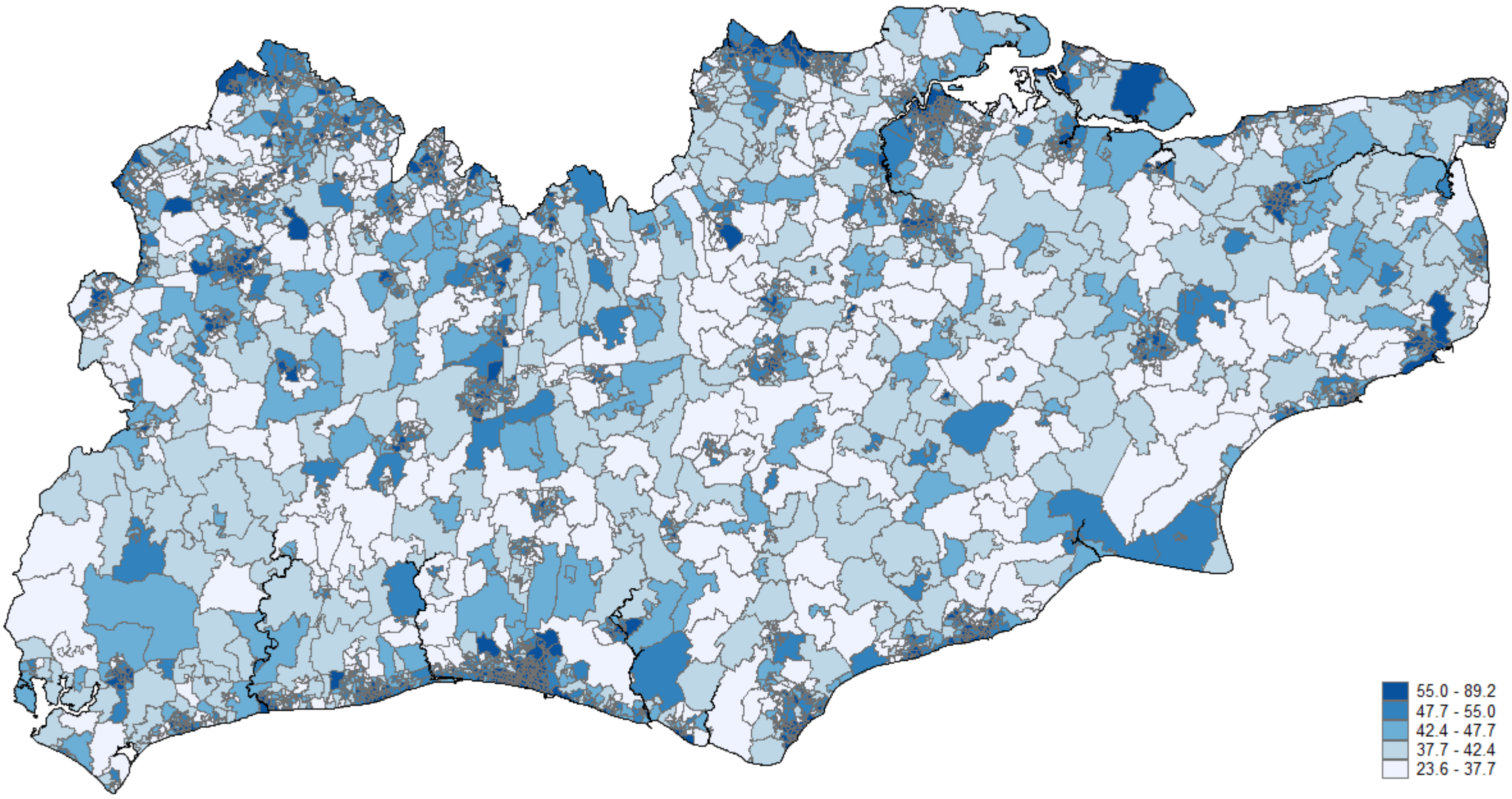

2011

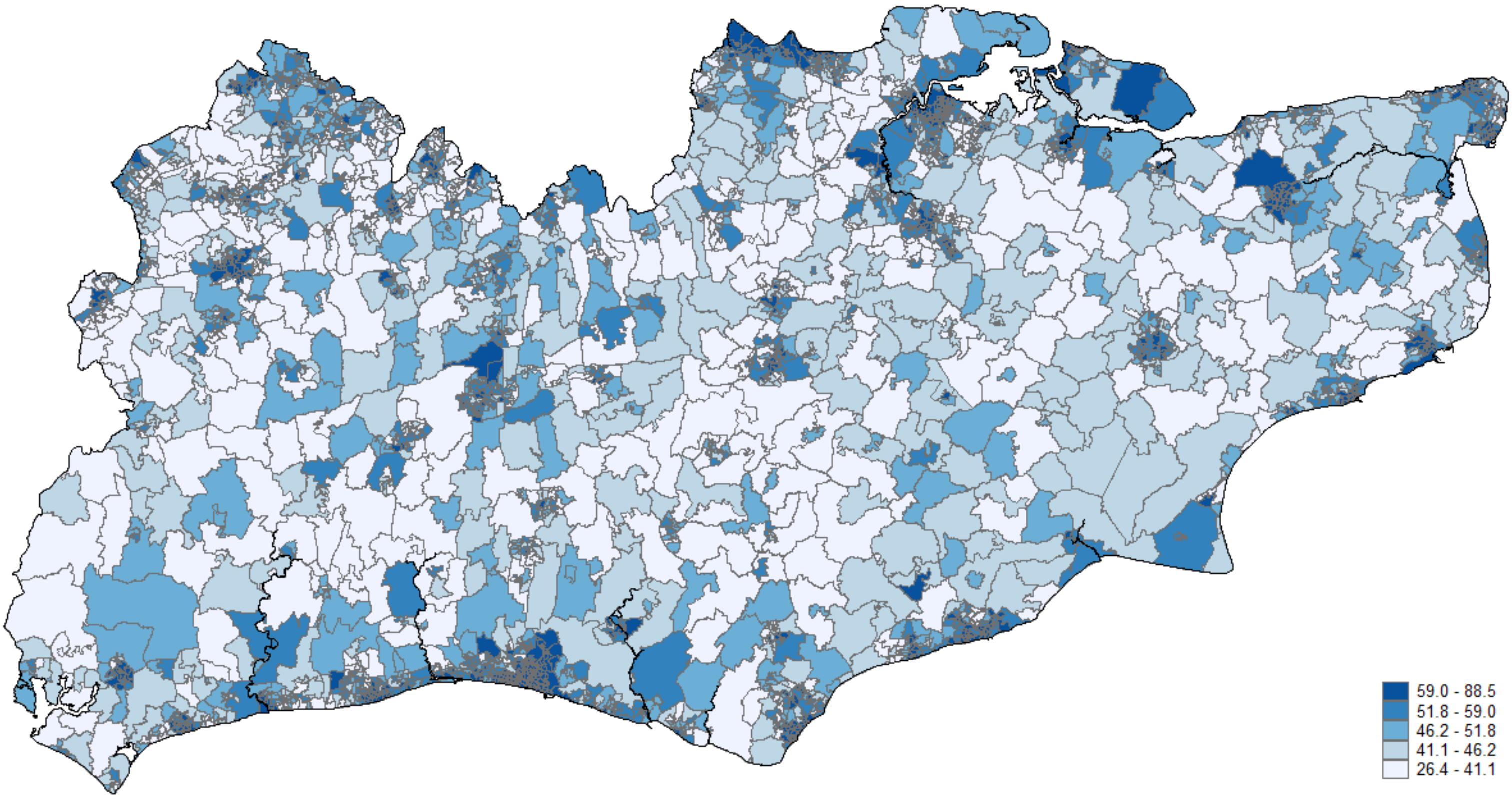

South Central, Single People % of population

2001

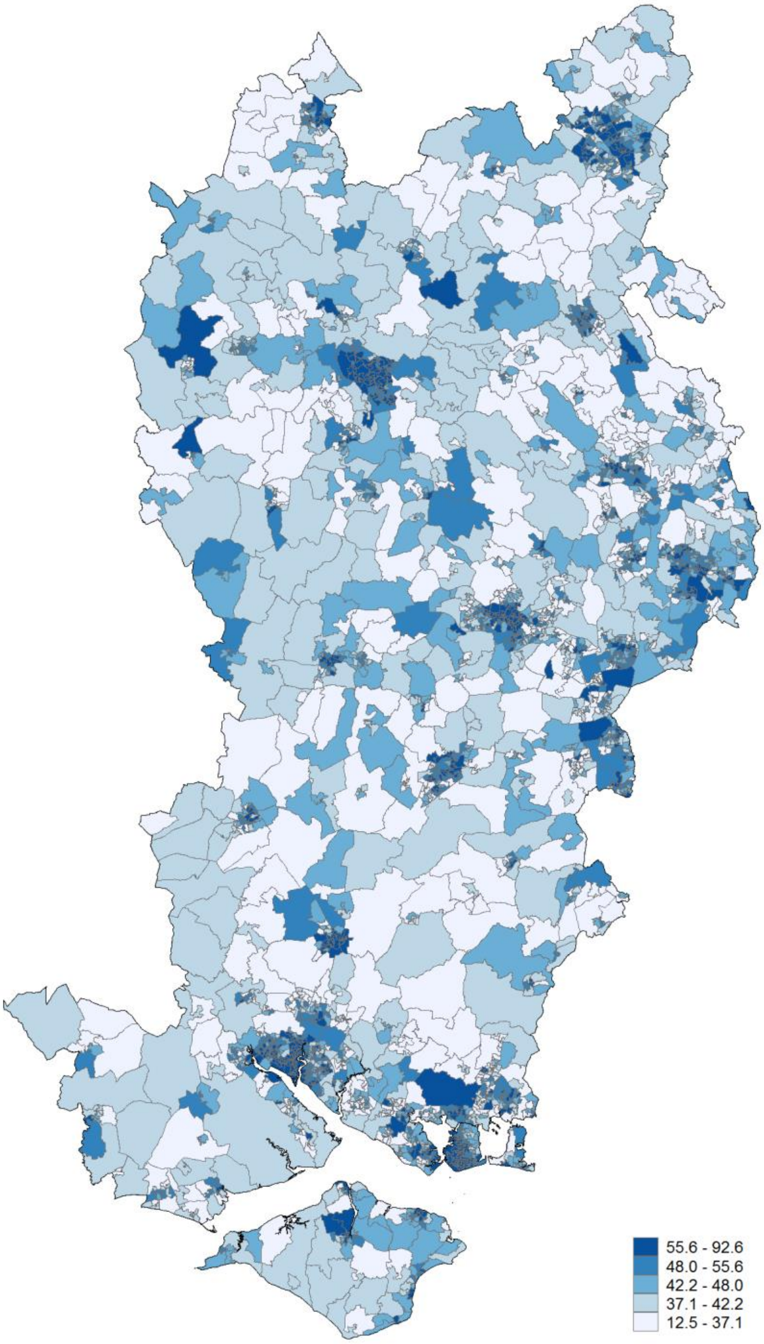

2011

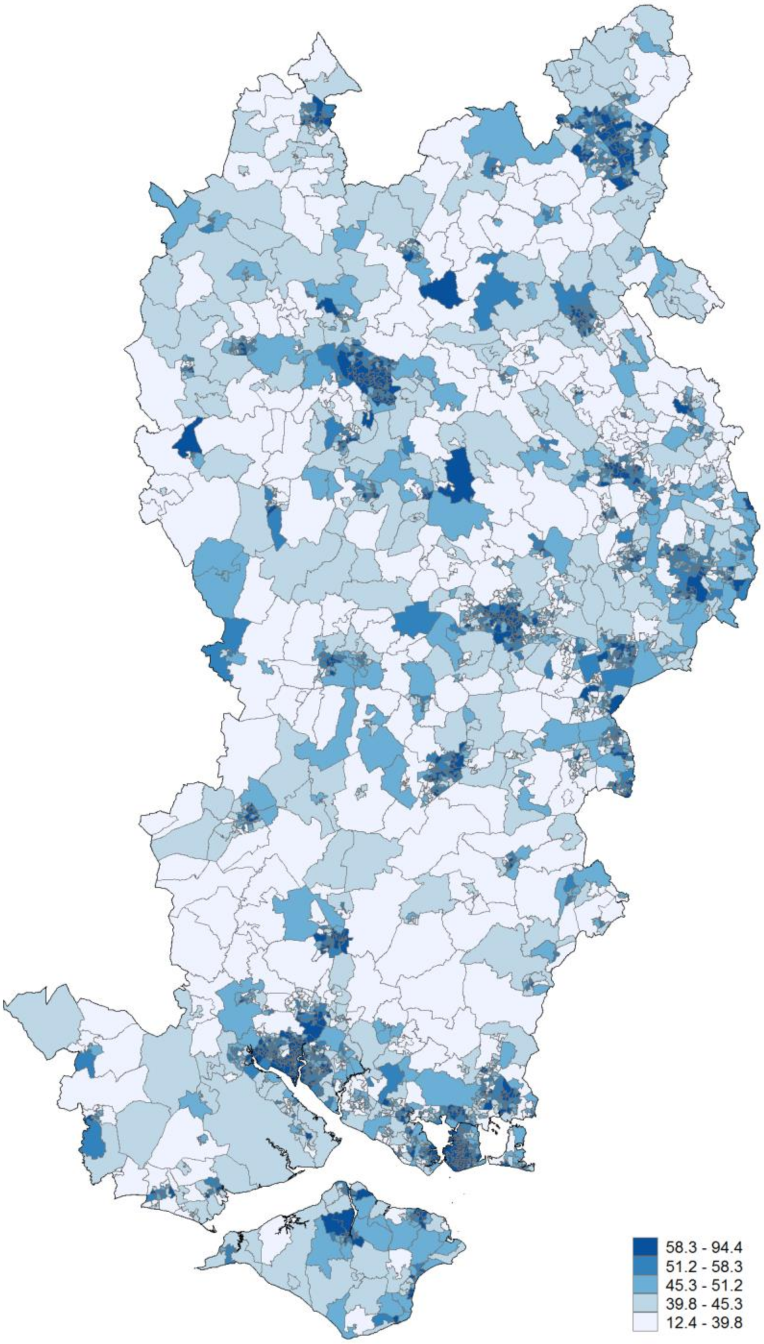

South West, Single People % of population

2001

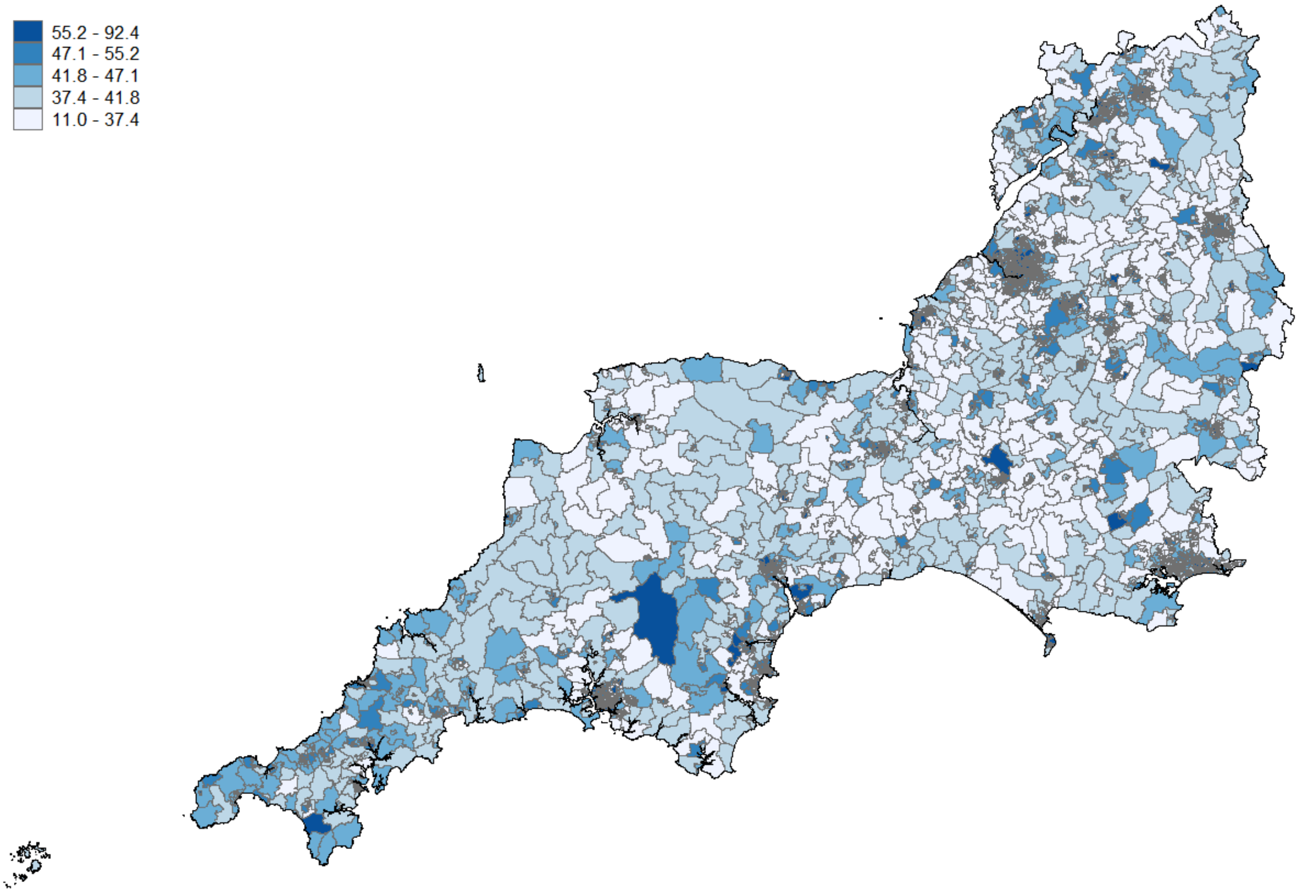

2011

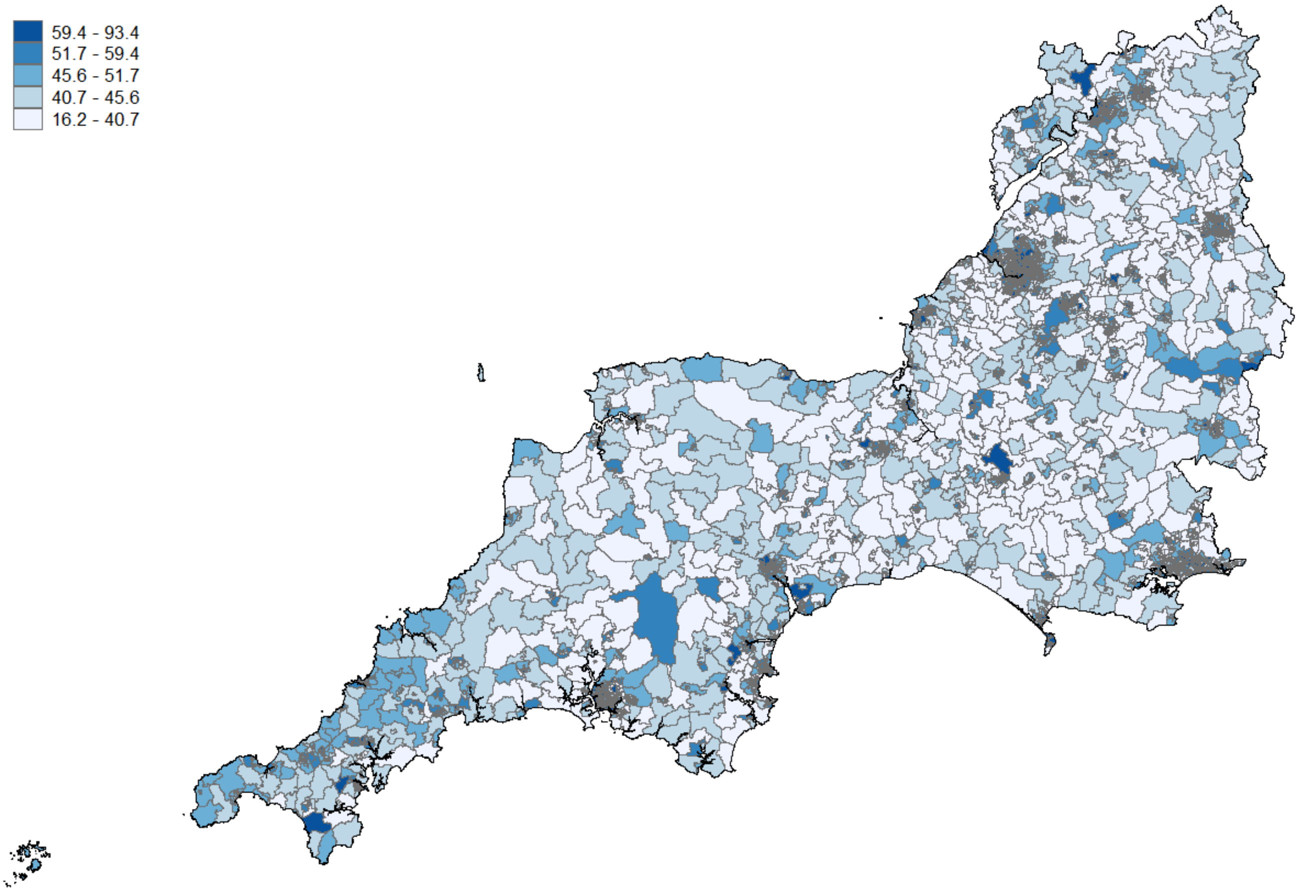

One Person Households

England 2001, One Person Households % of all households

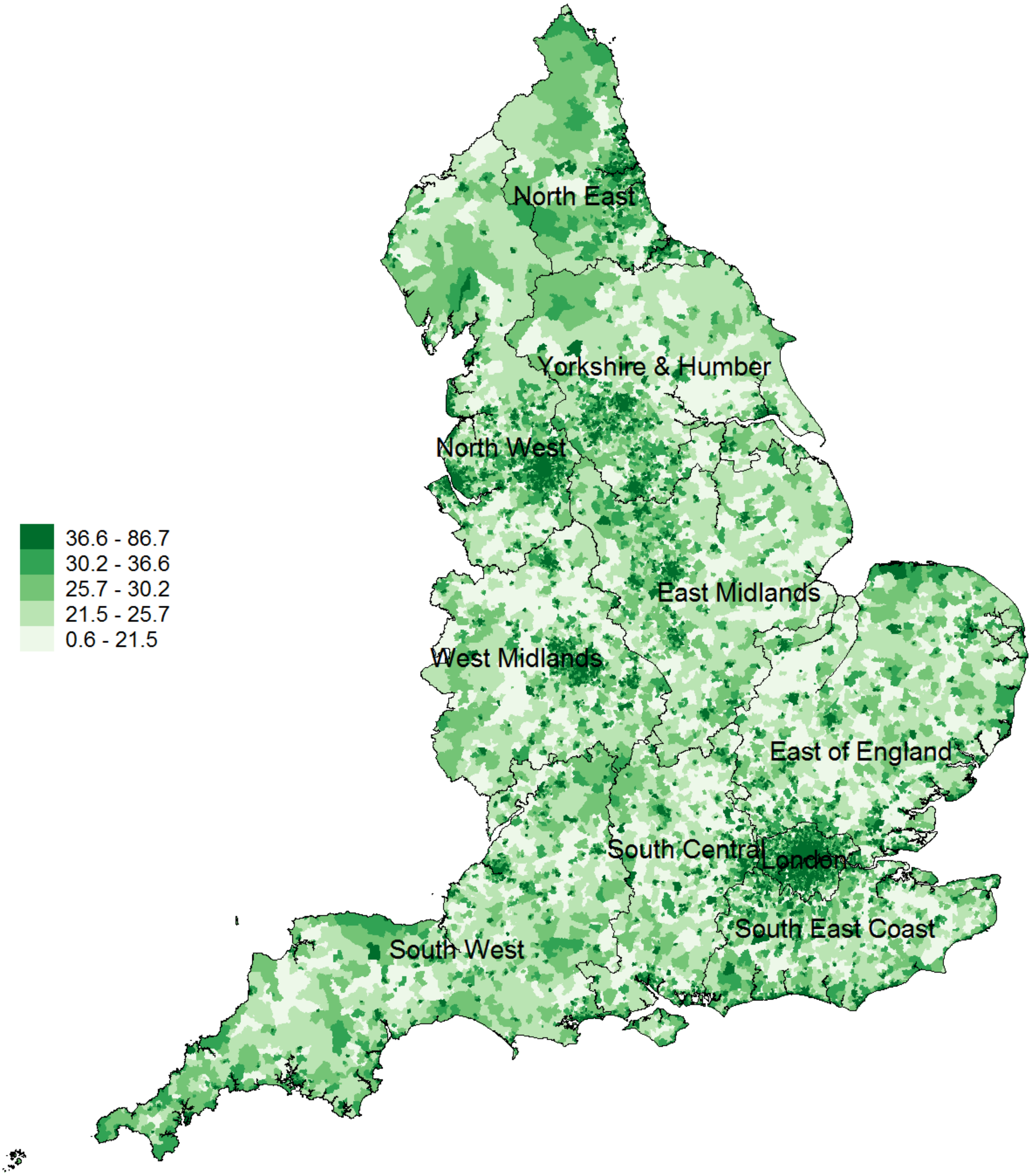

England 2011, One Person Households % of all households

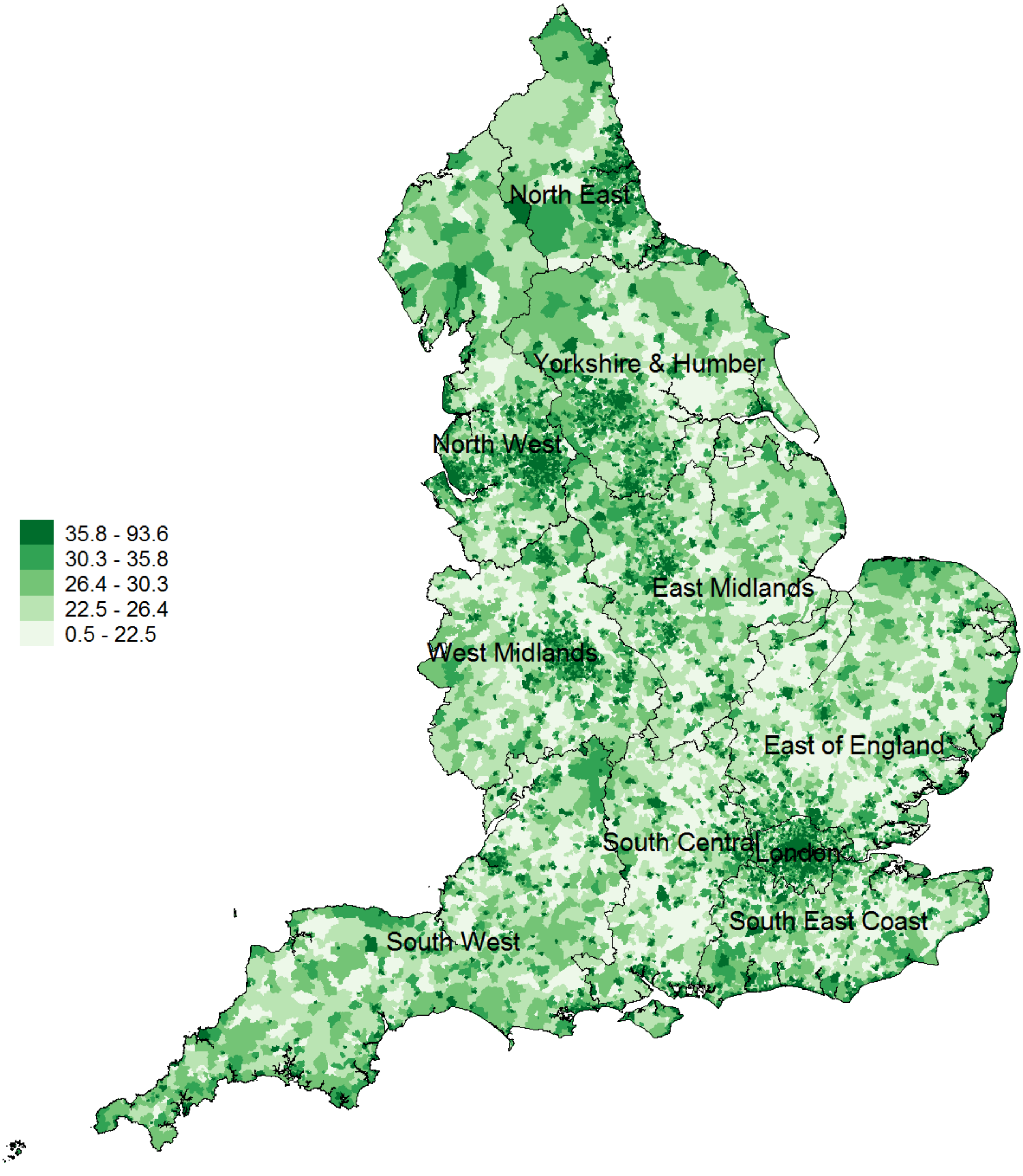

North East, One Person Households % of all households

2001

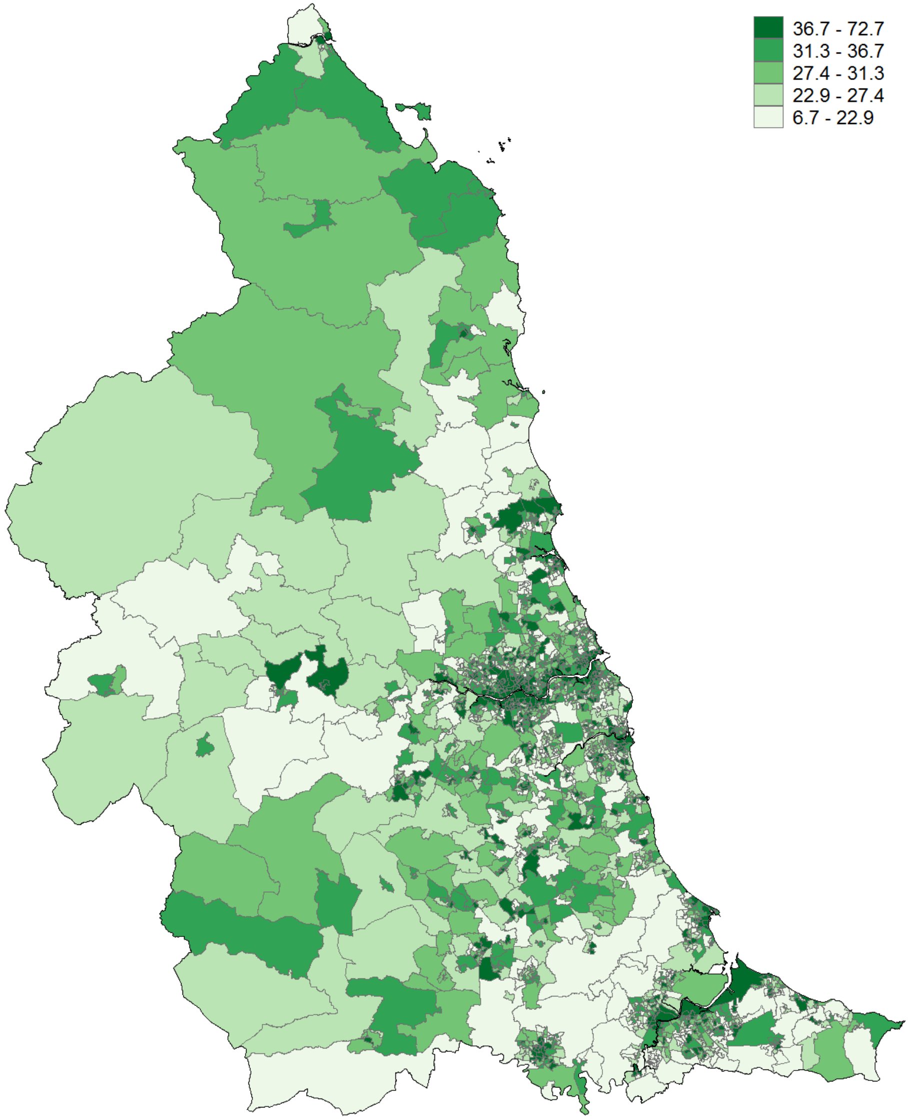

2011

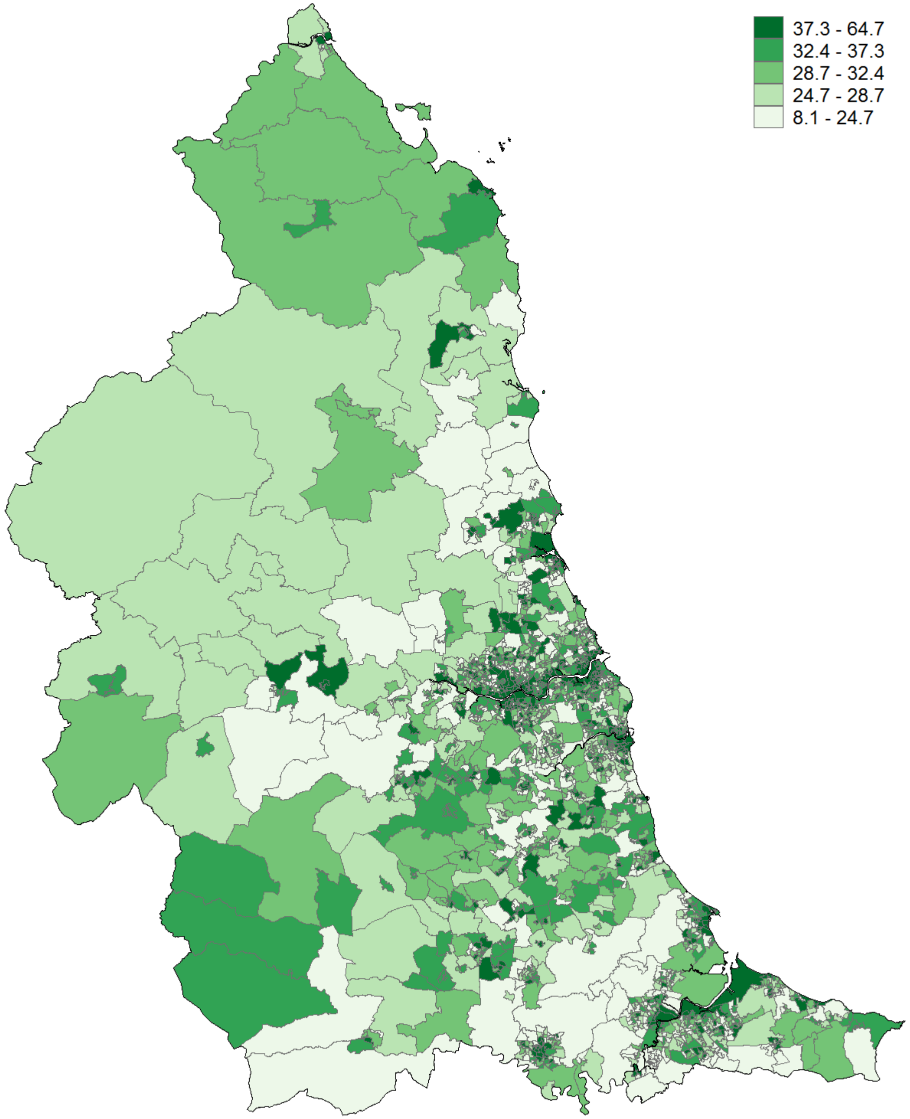

North West, One Person Households % of all households

2001

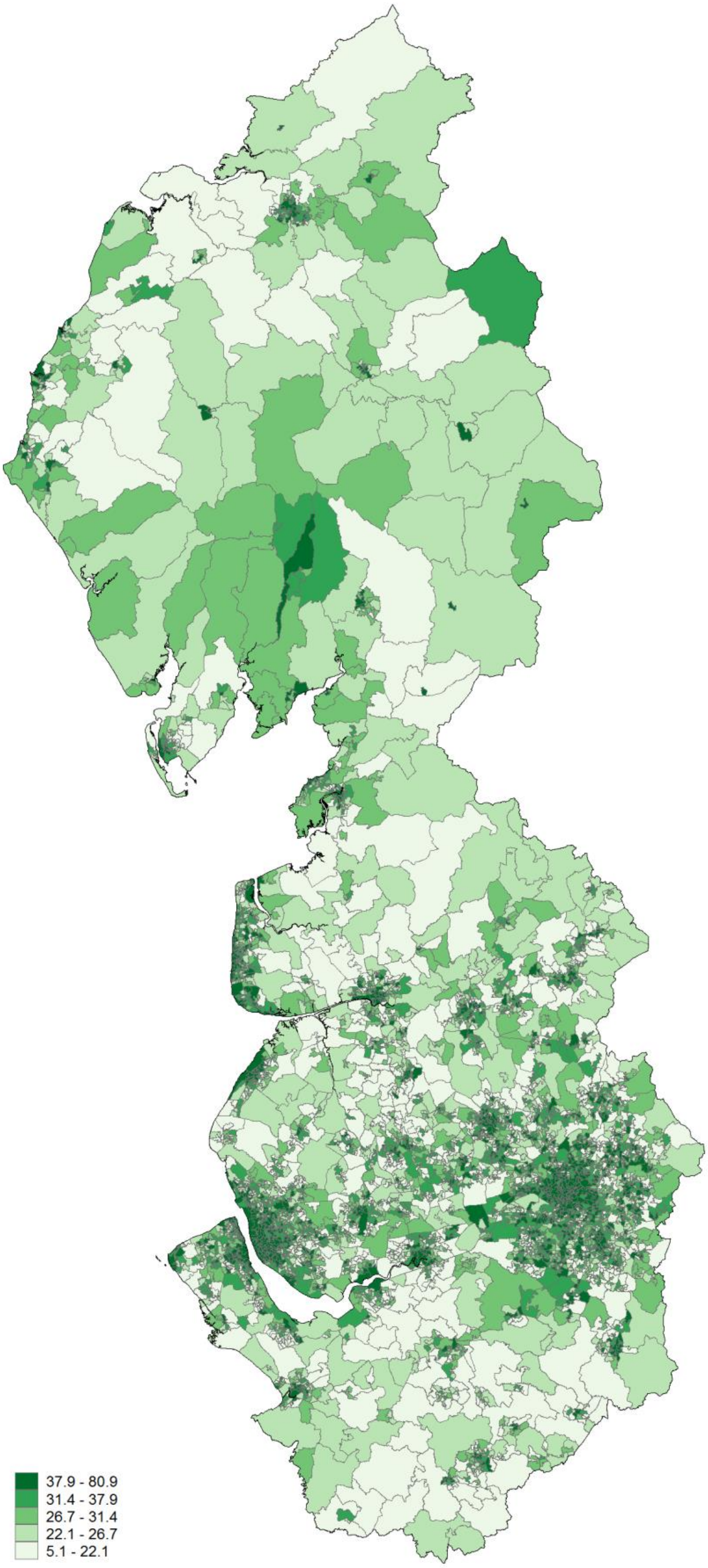

2011

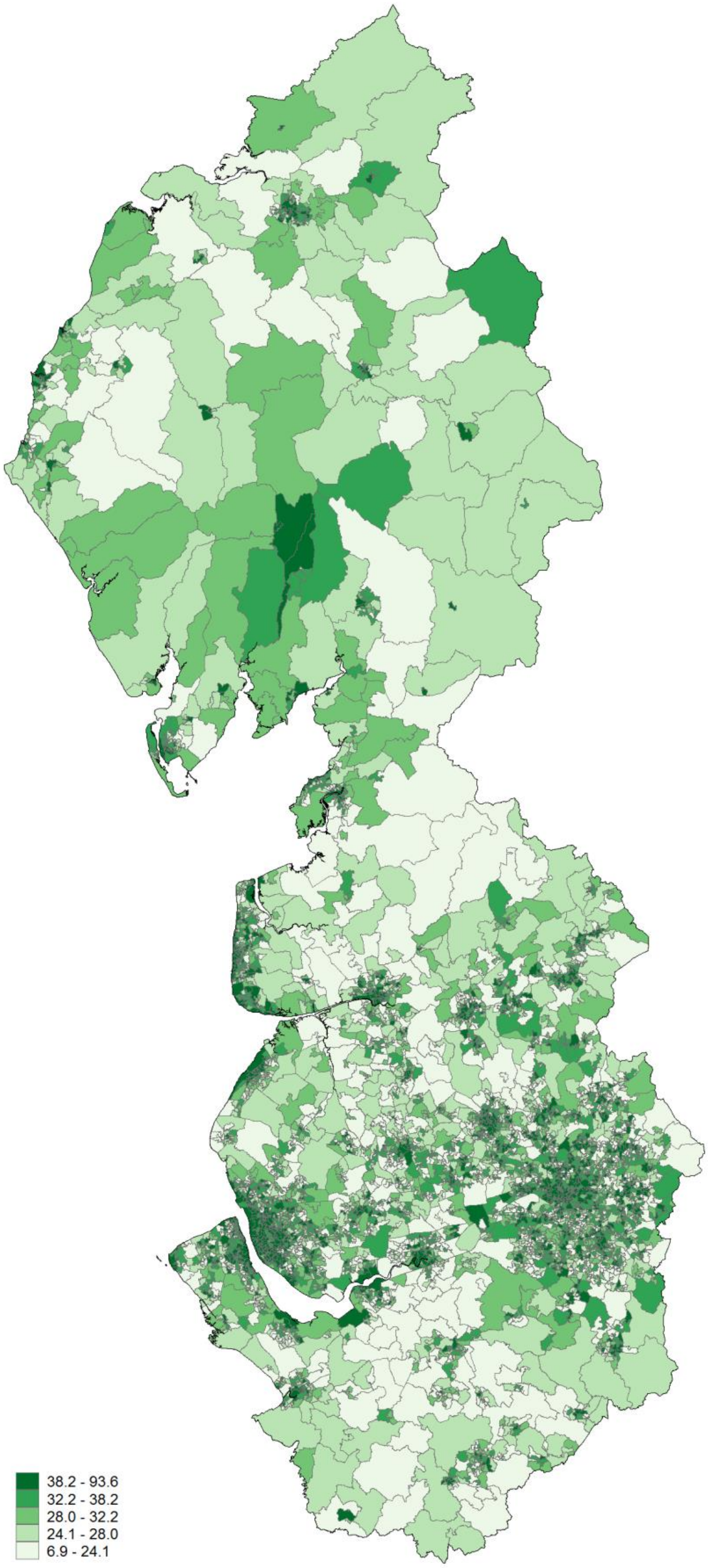

Yorkshire and the Humber, One Person Households % of all households

2001

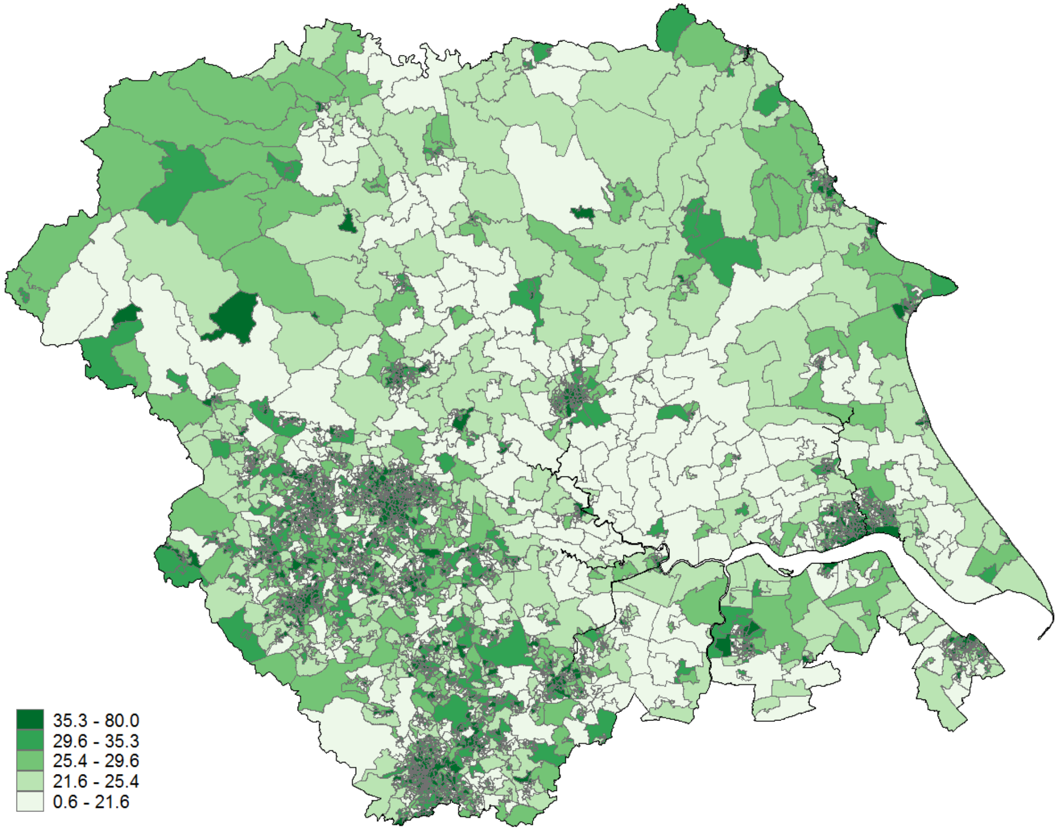

2011

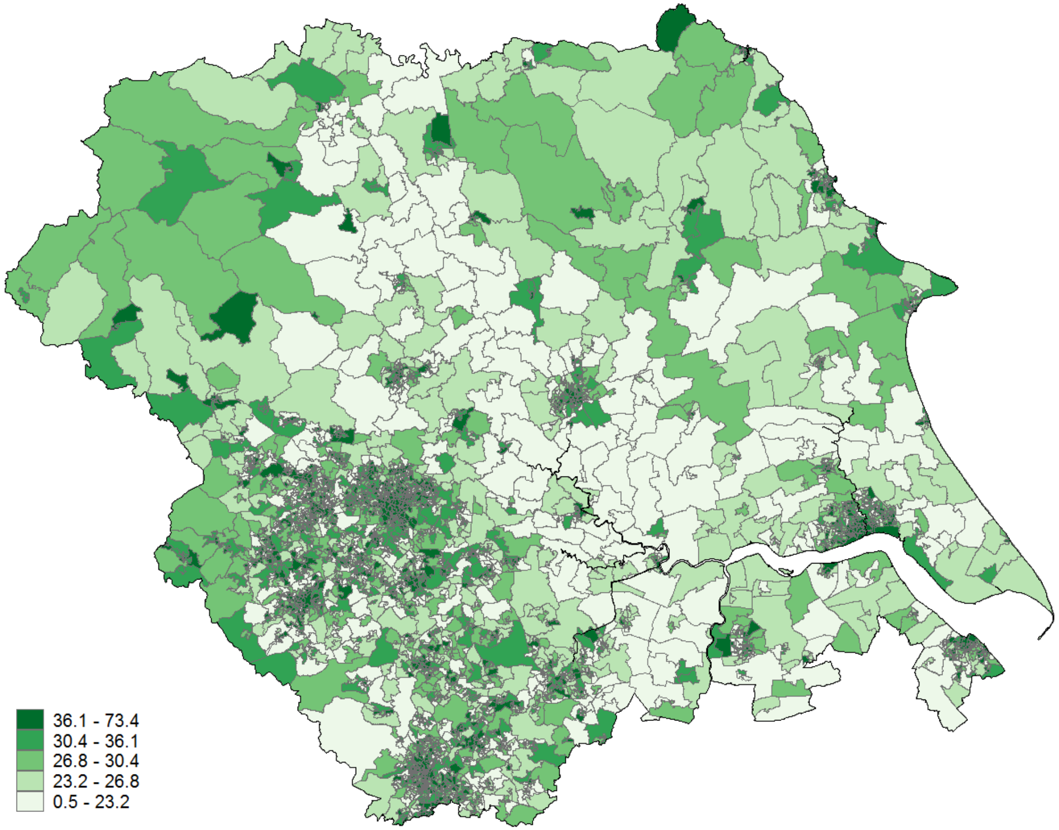

East Midlands, One Person Households % of all households

2001

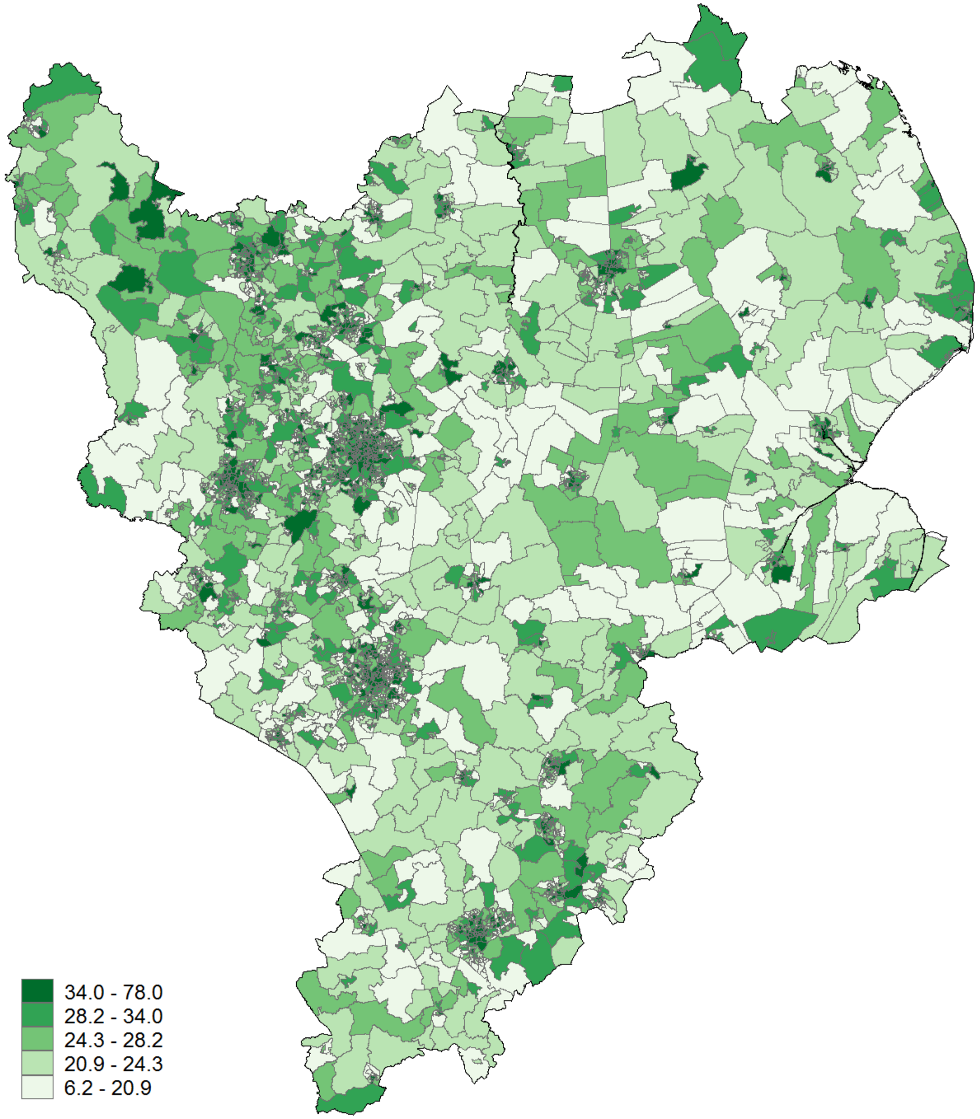

2011

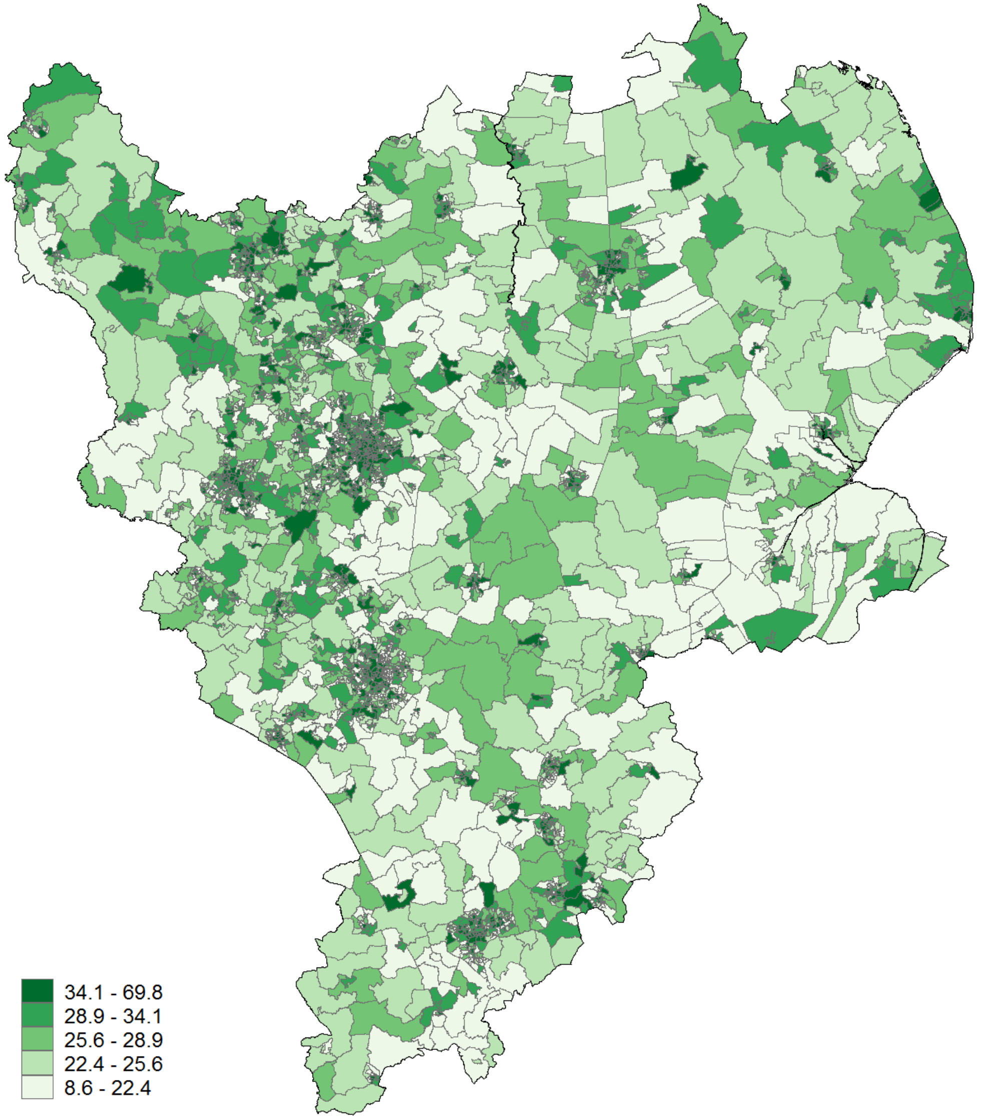

West Midlands, One Person Households % of all households

2001

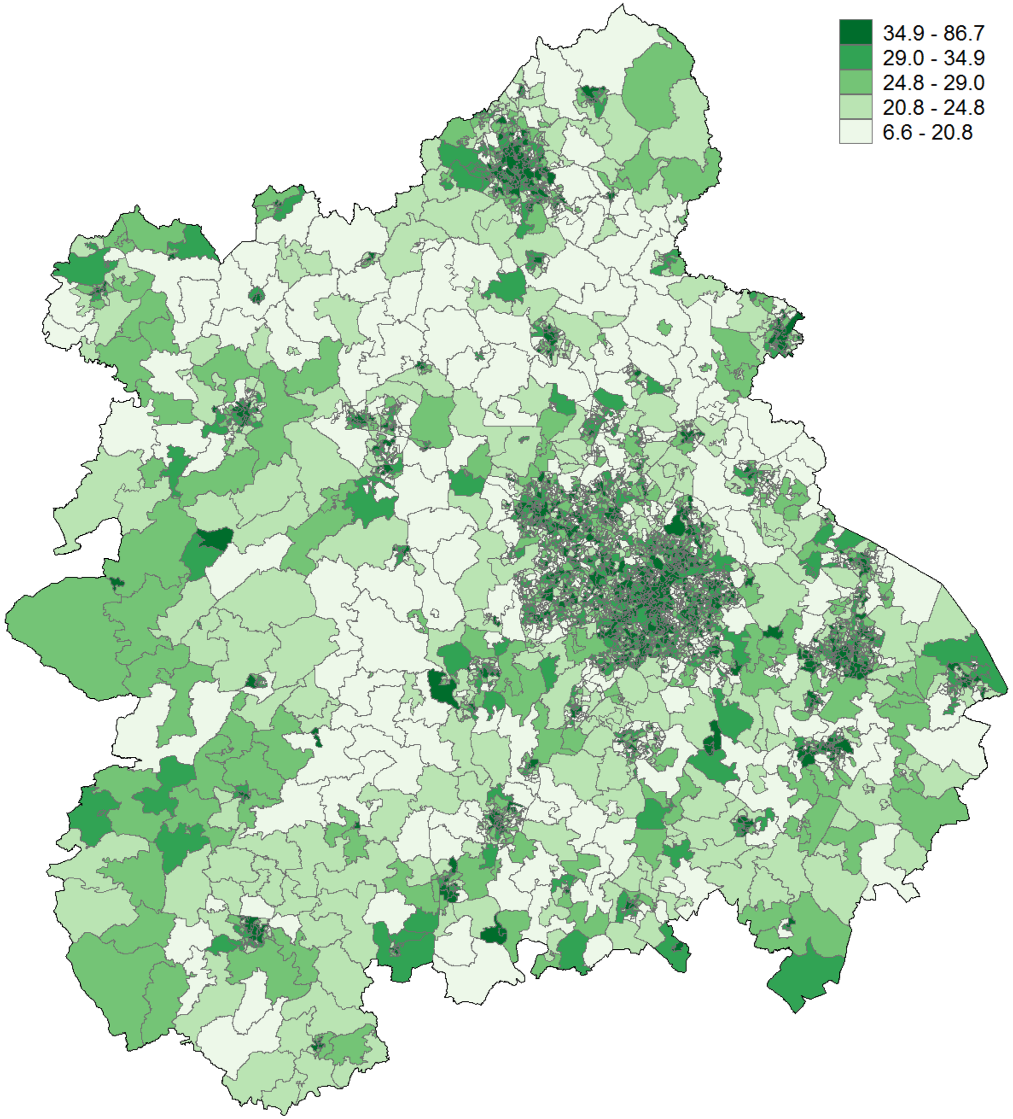

2011

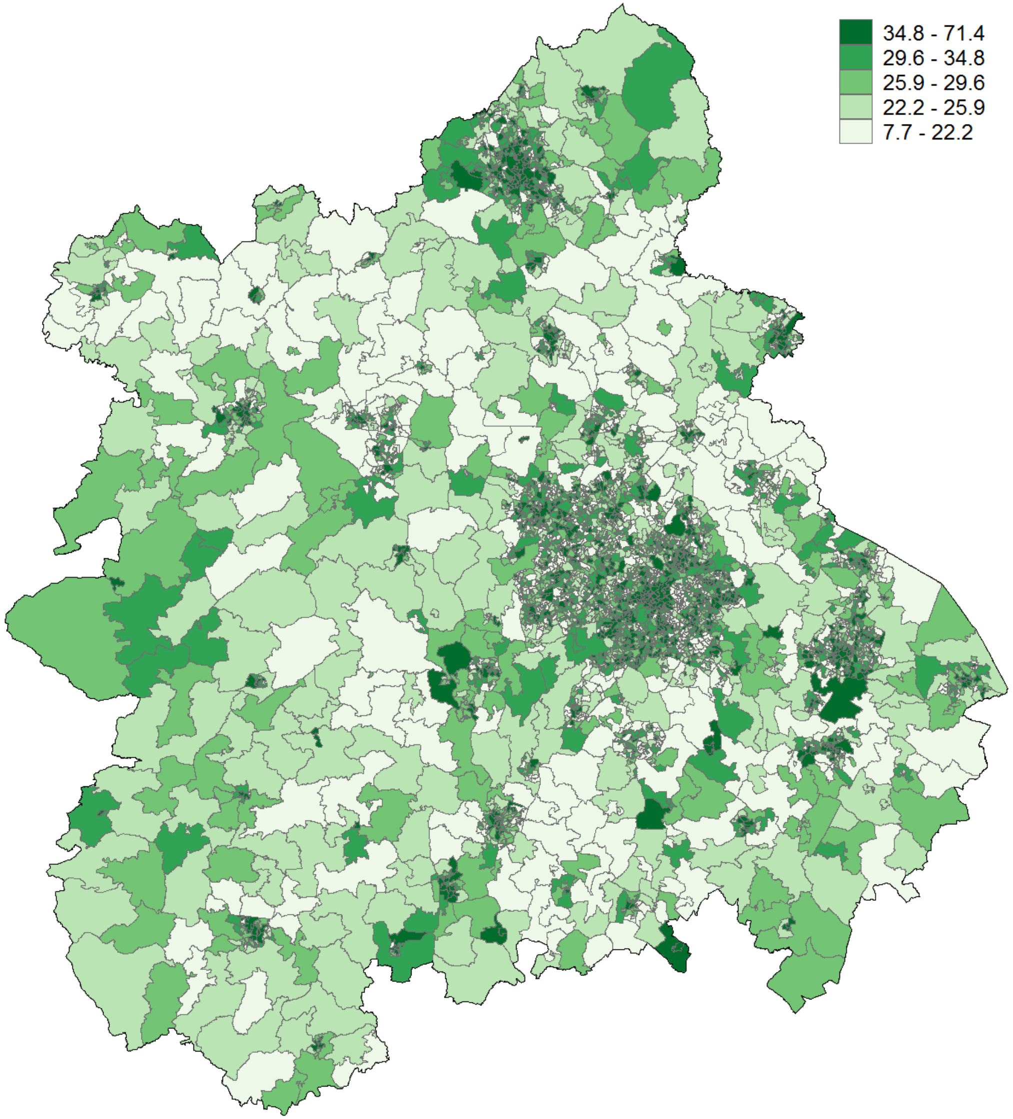

East of England, One Person Households % of all households

2001

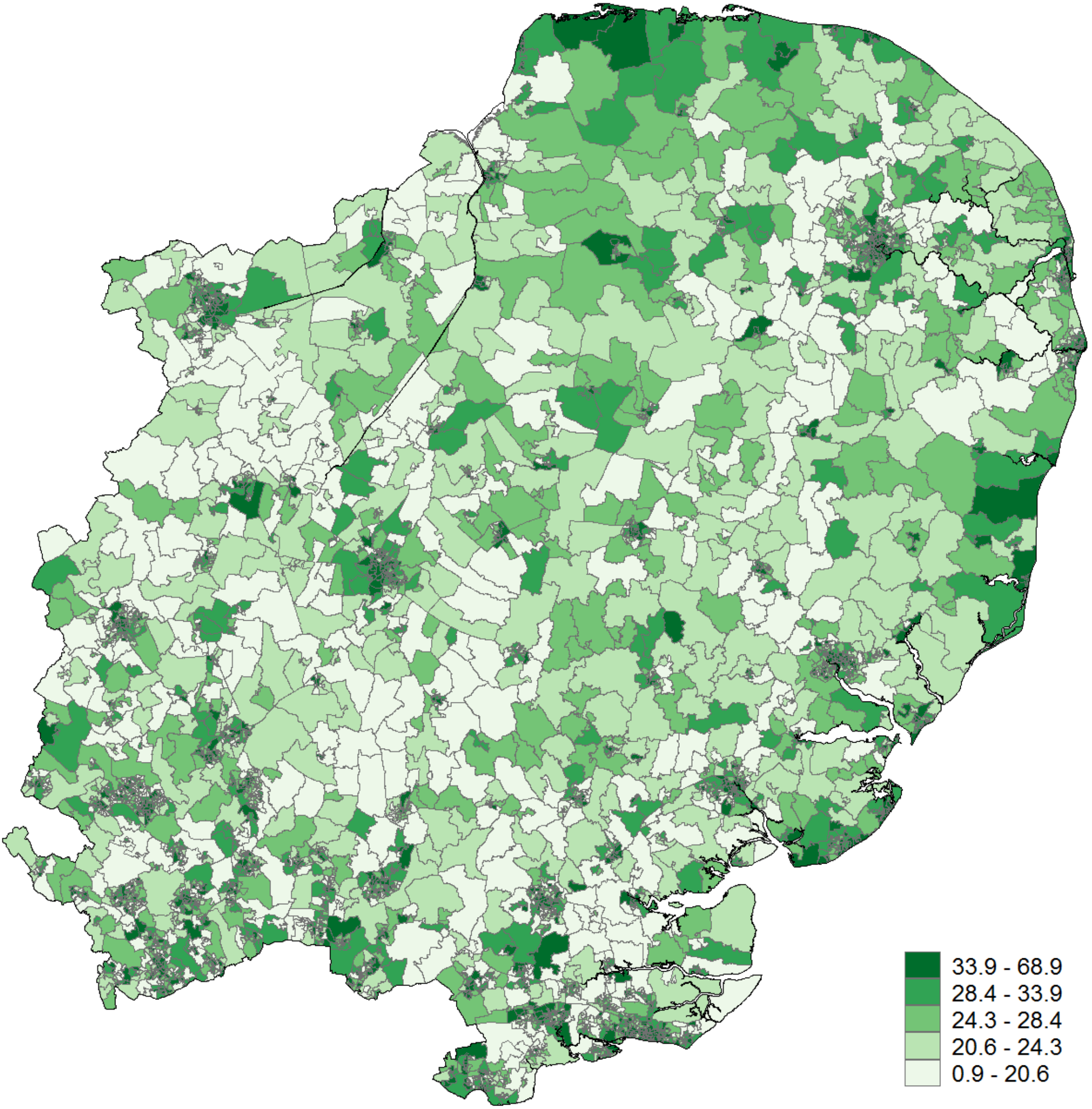

2011

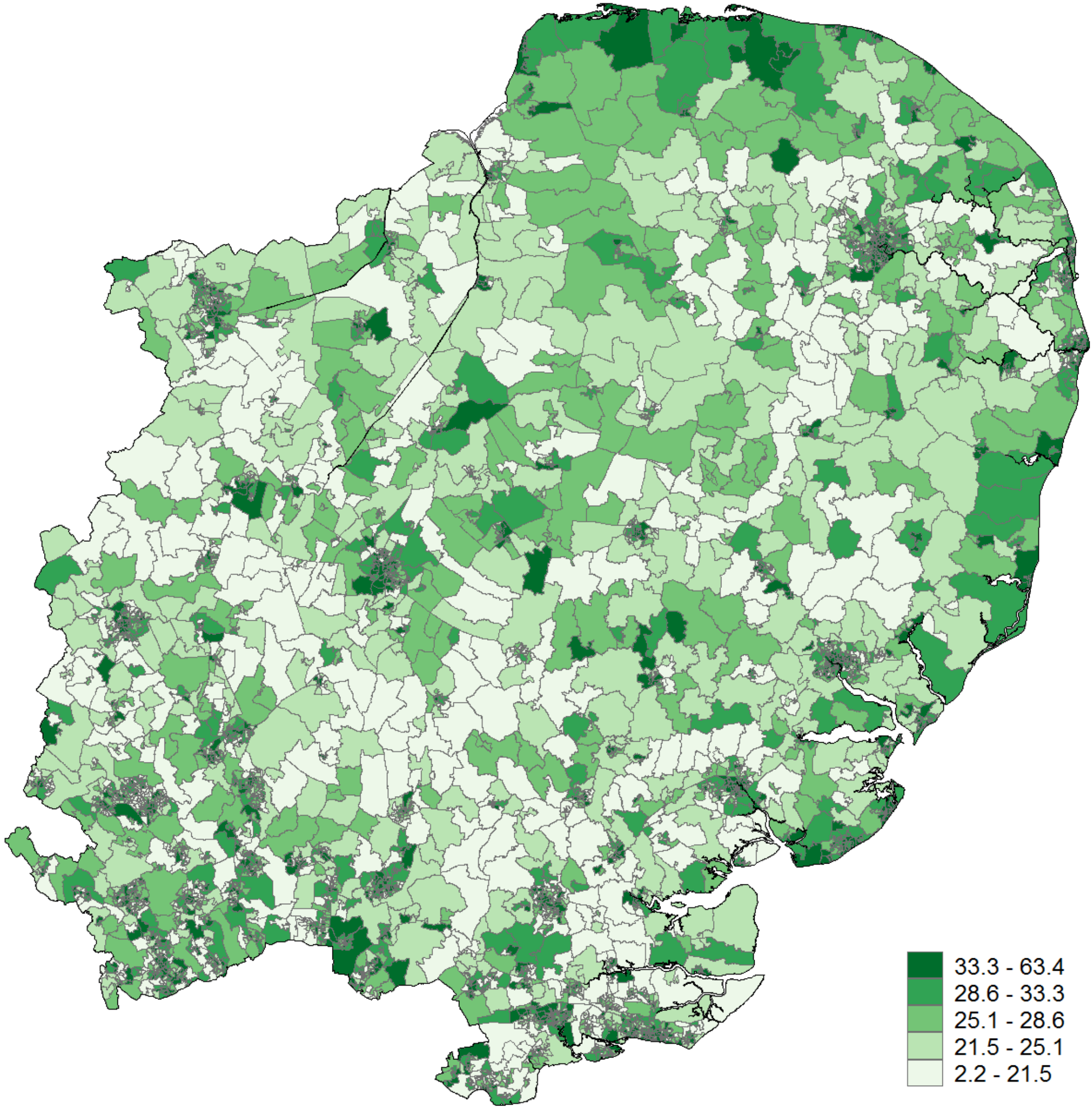

London, One Person Households % of all households

2001

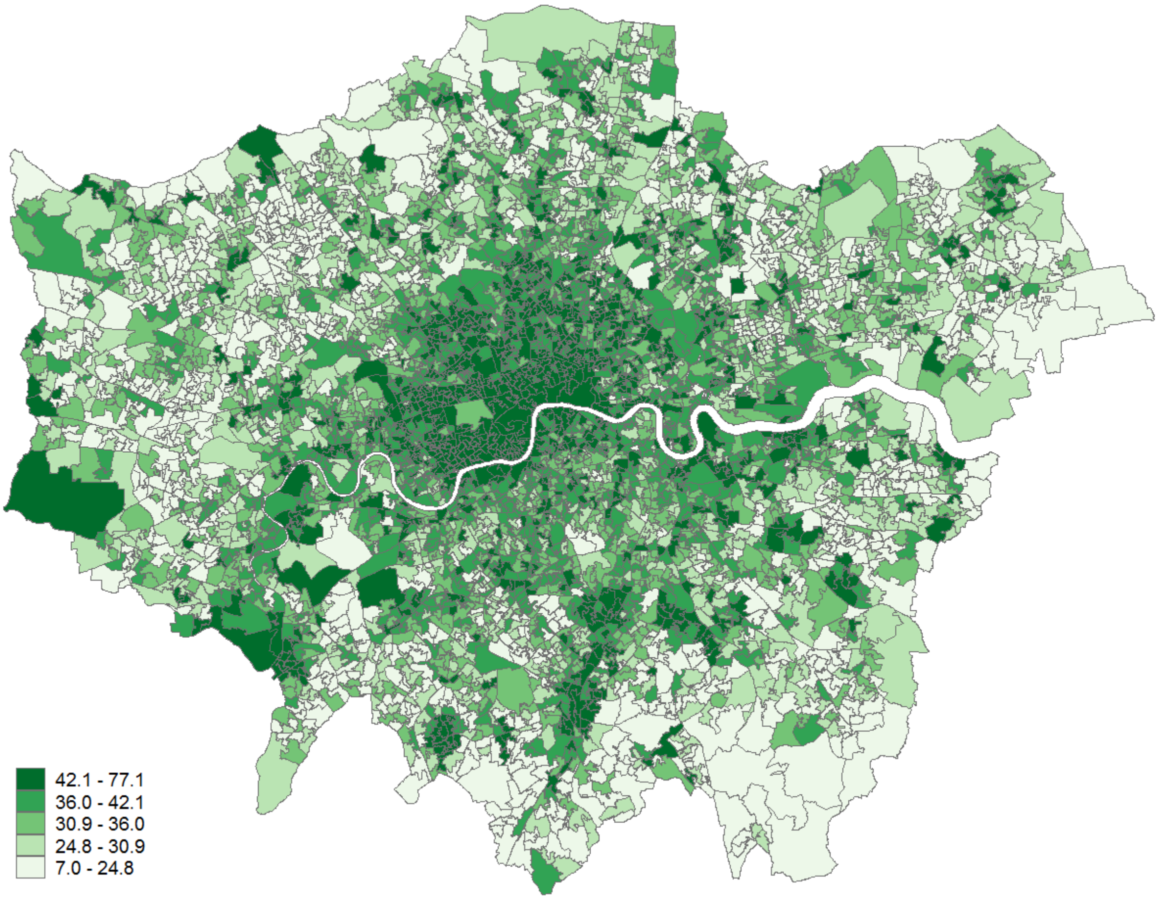

2011

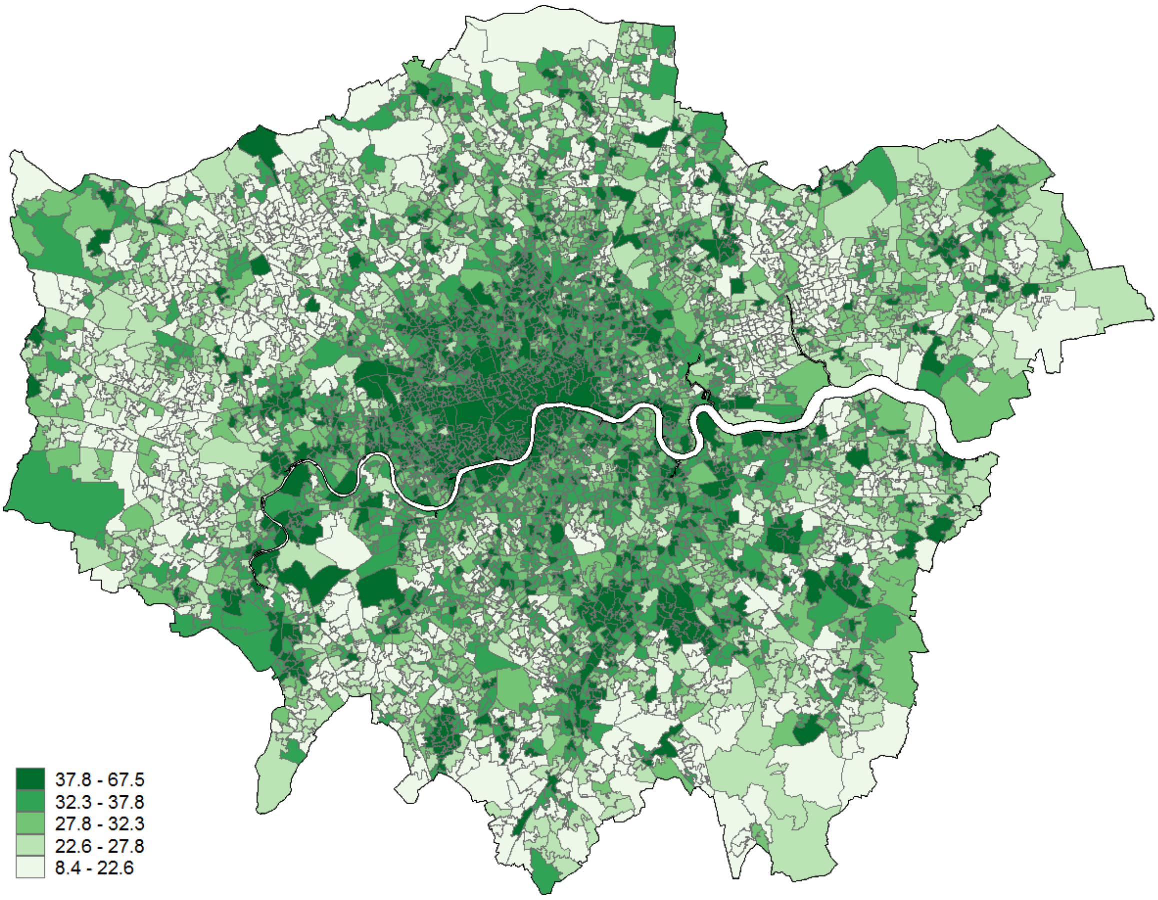

South East Coast, One Person Households % of all households

2001

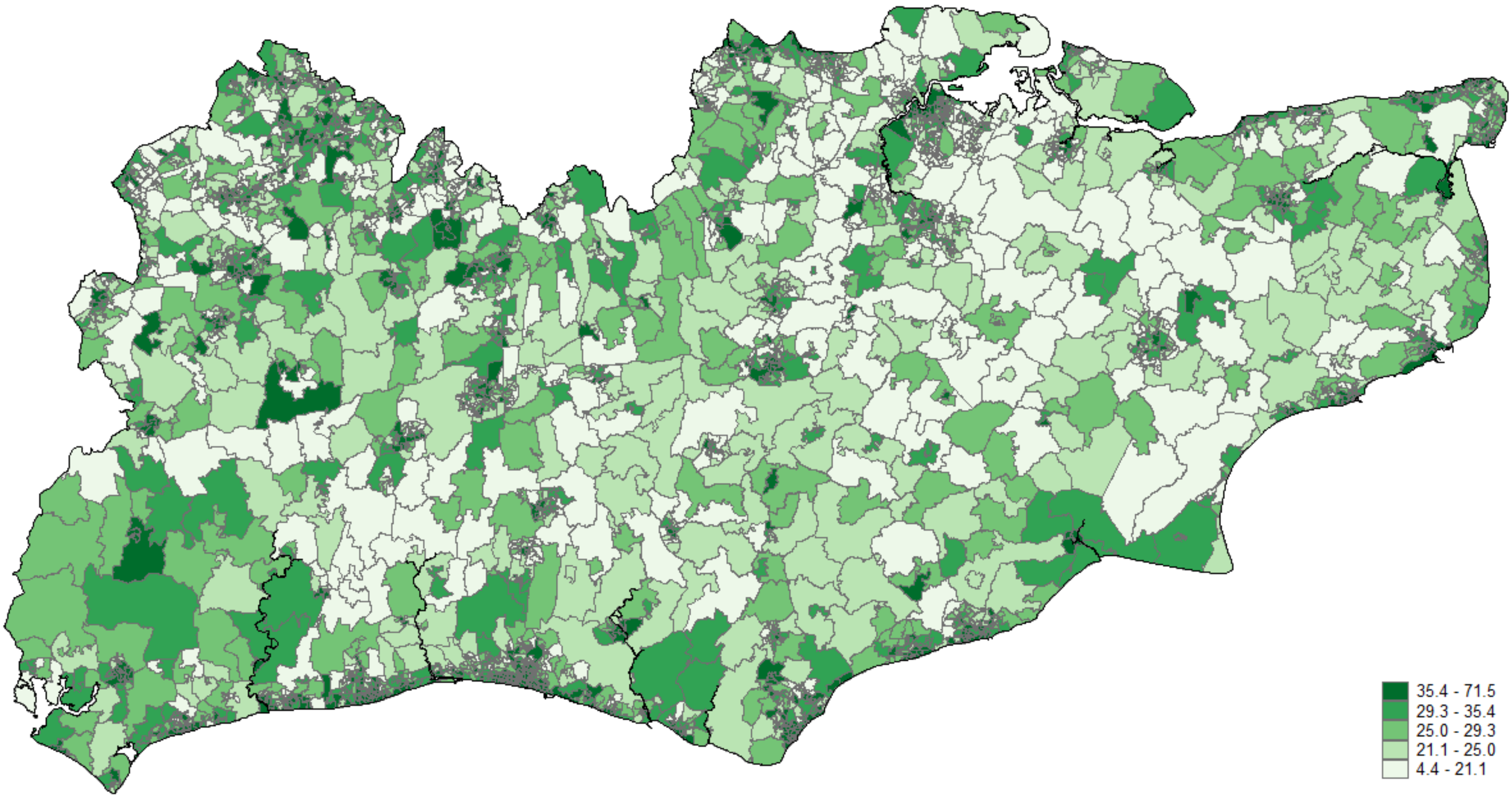

2011

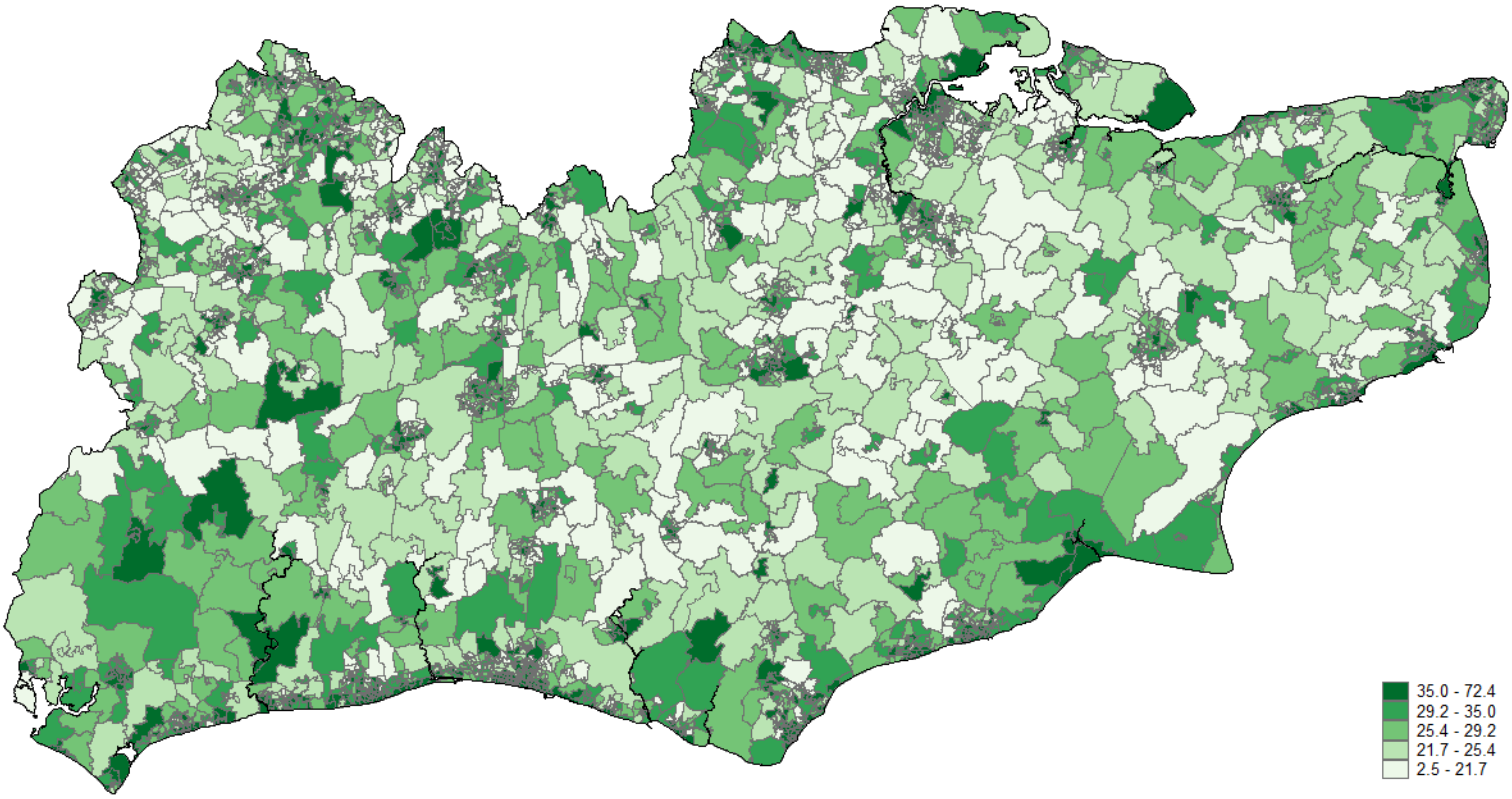

South Central, One Person Households % of all households

2001

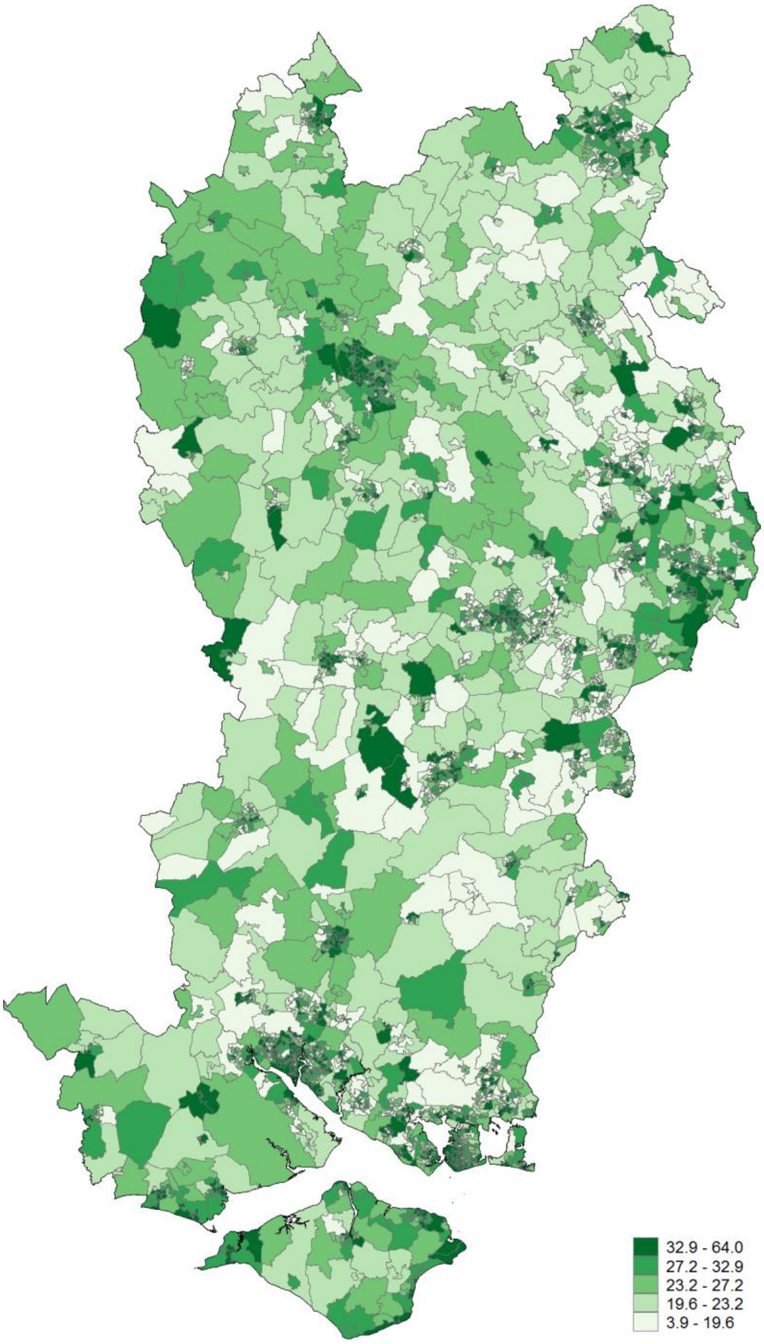

2011

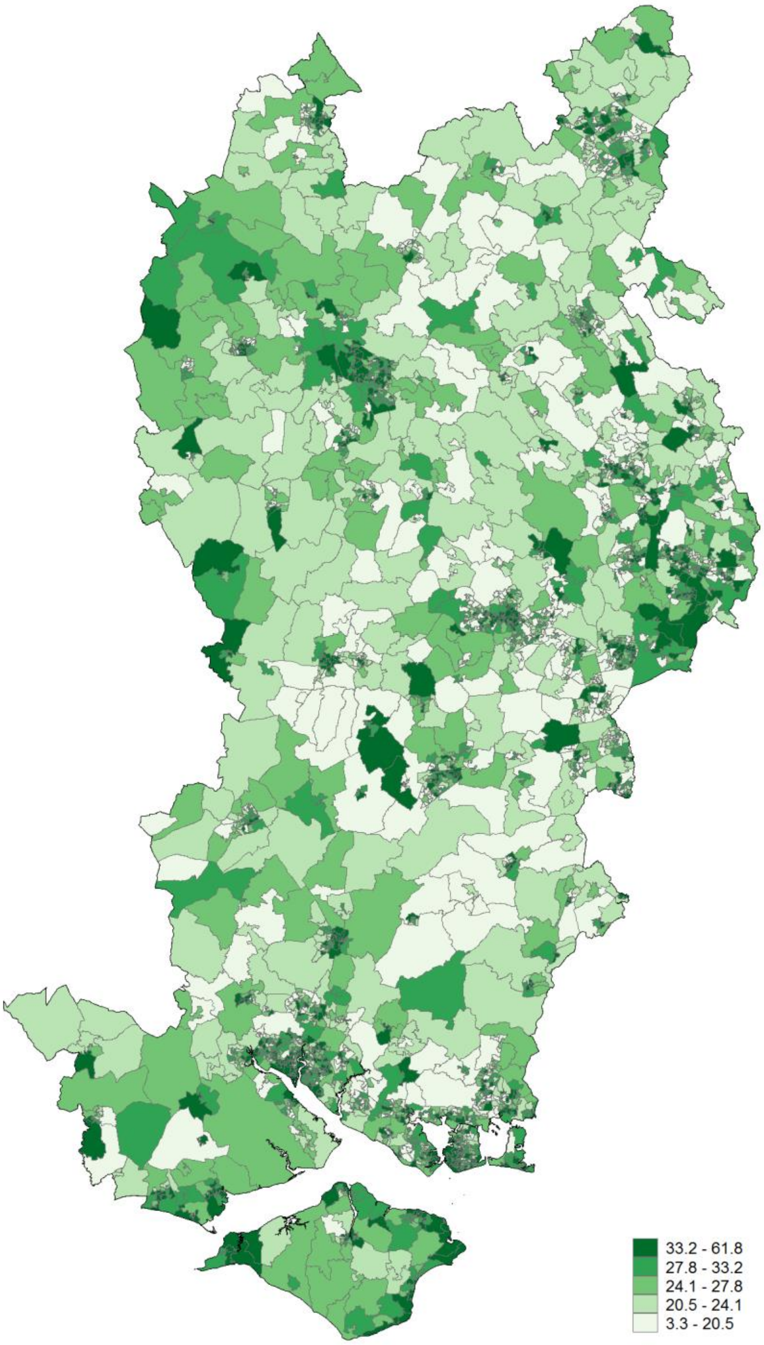

South West, One Person Households % of all households

2001

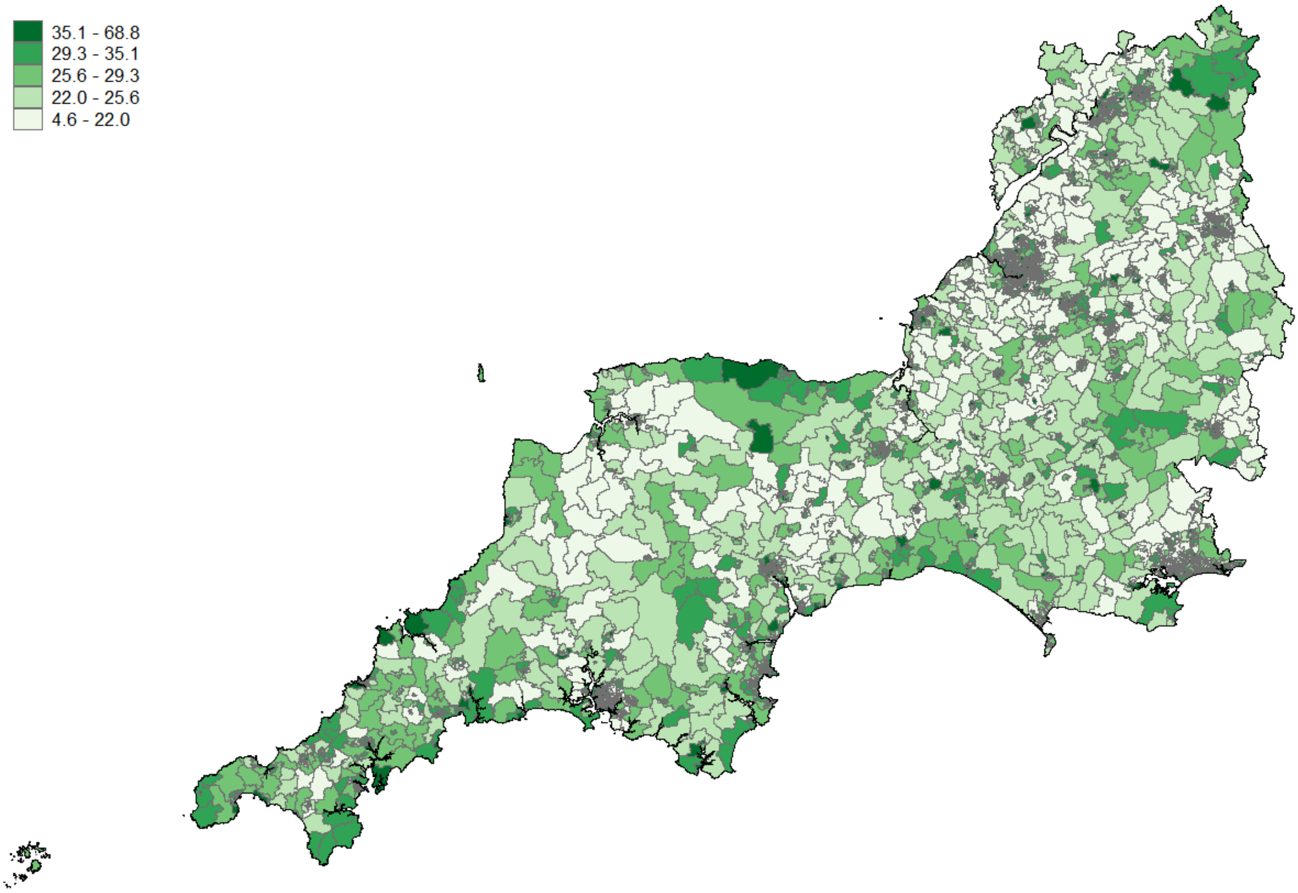

2011

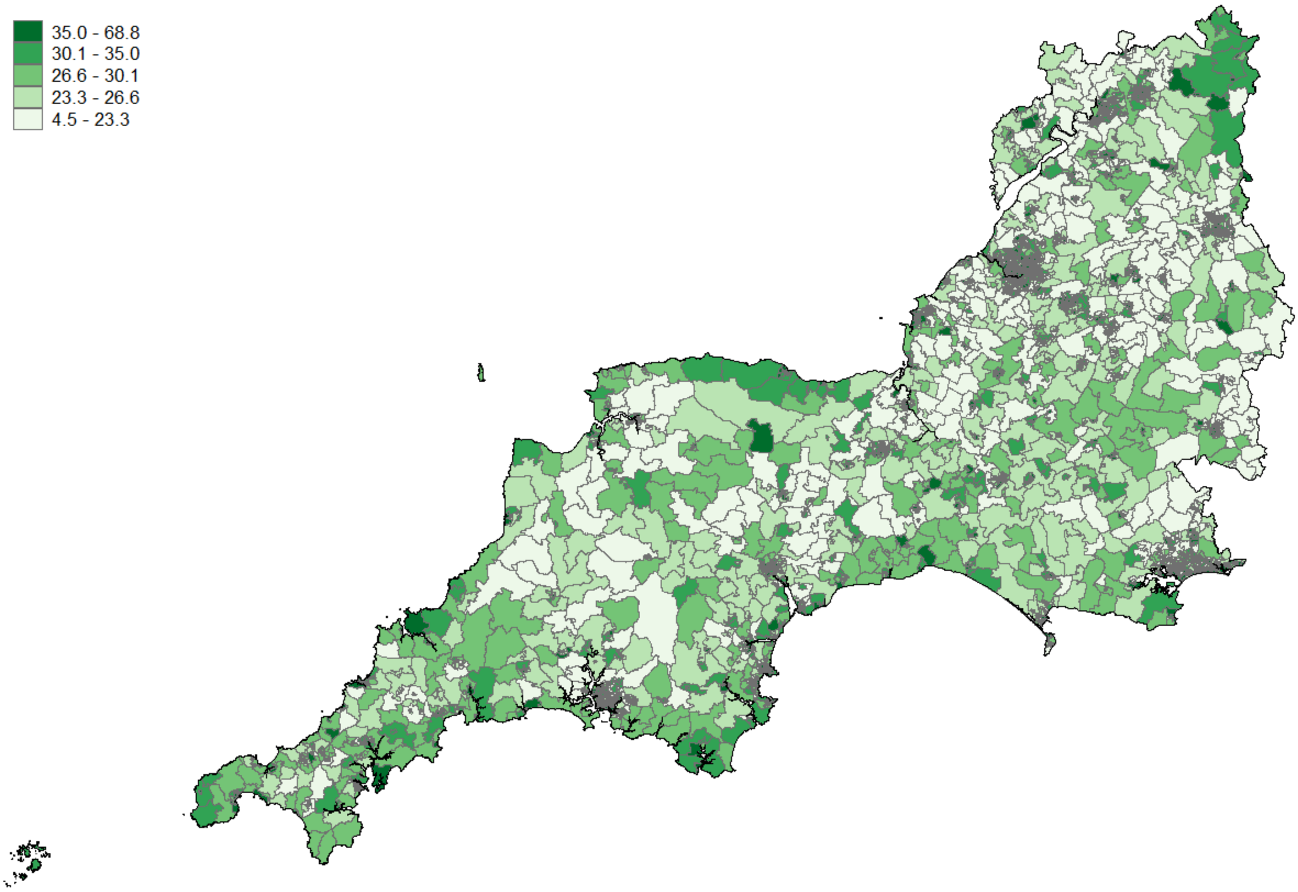

Private renting

England 2001, Private Renting % of all households

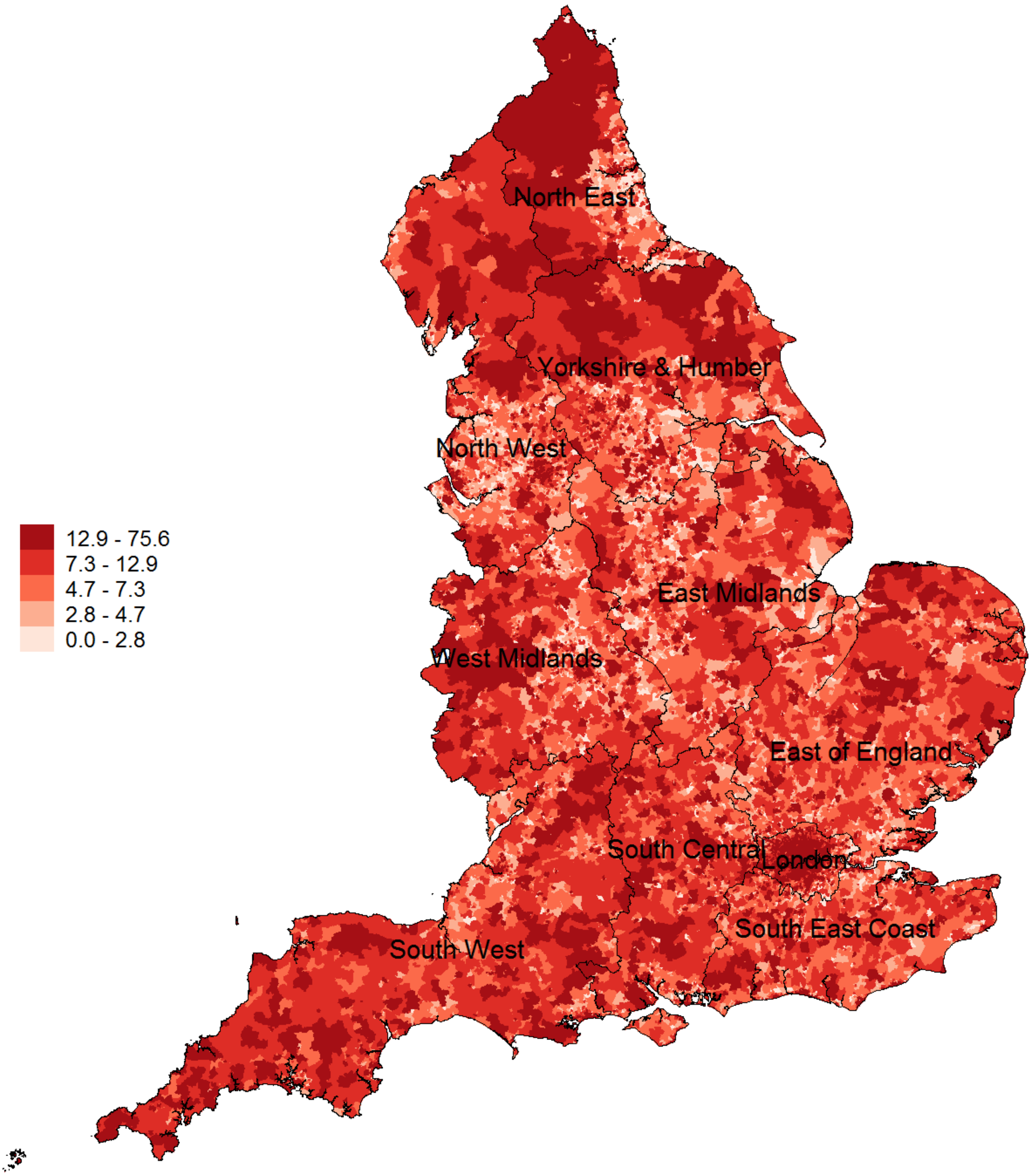

England 2011, Private Renting % of all households

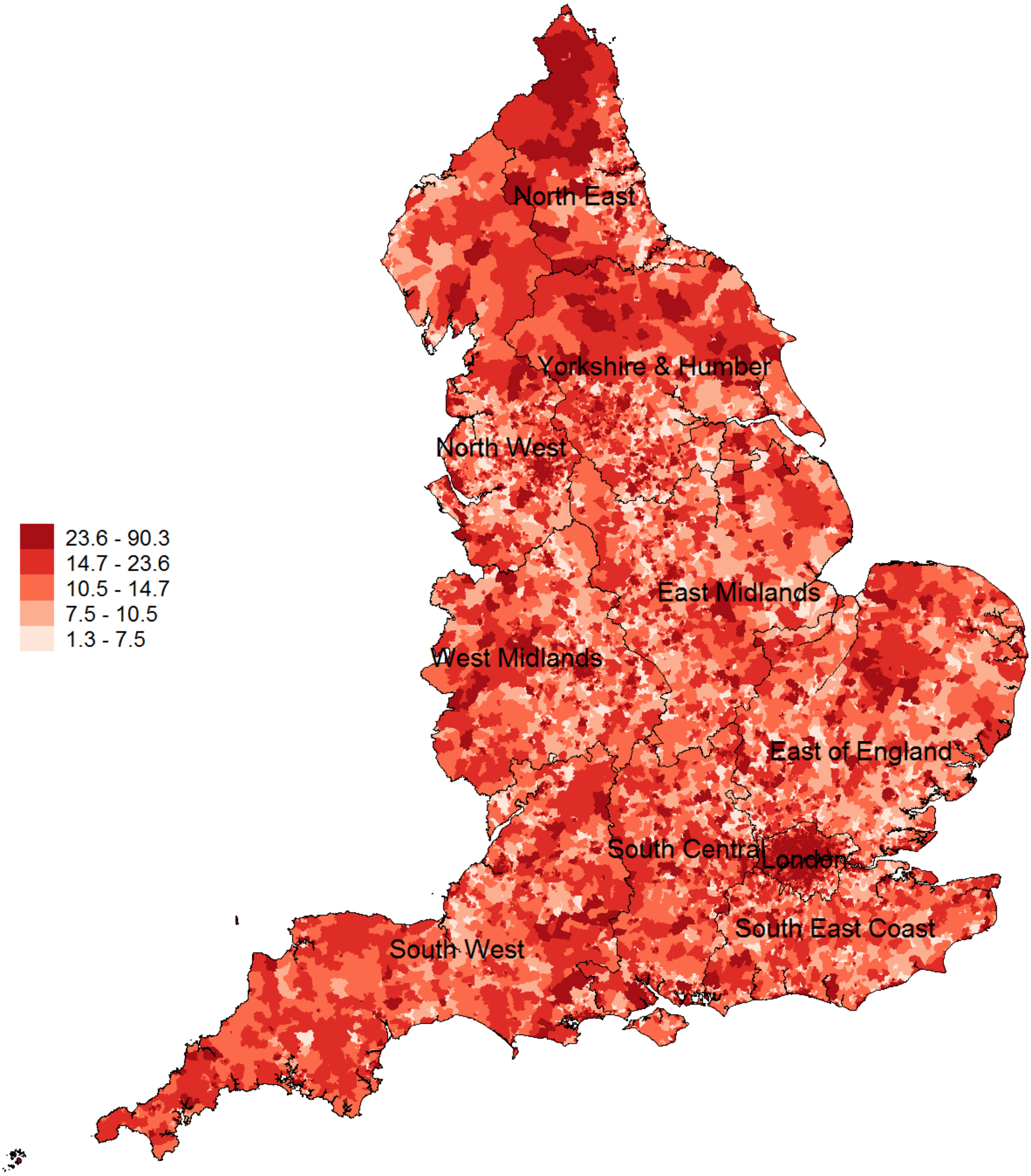

North East, Private Renting % of all households

2001

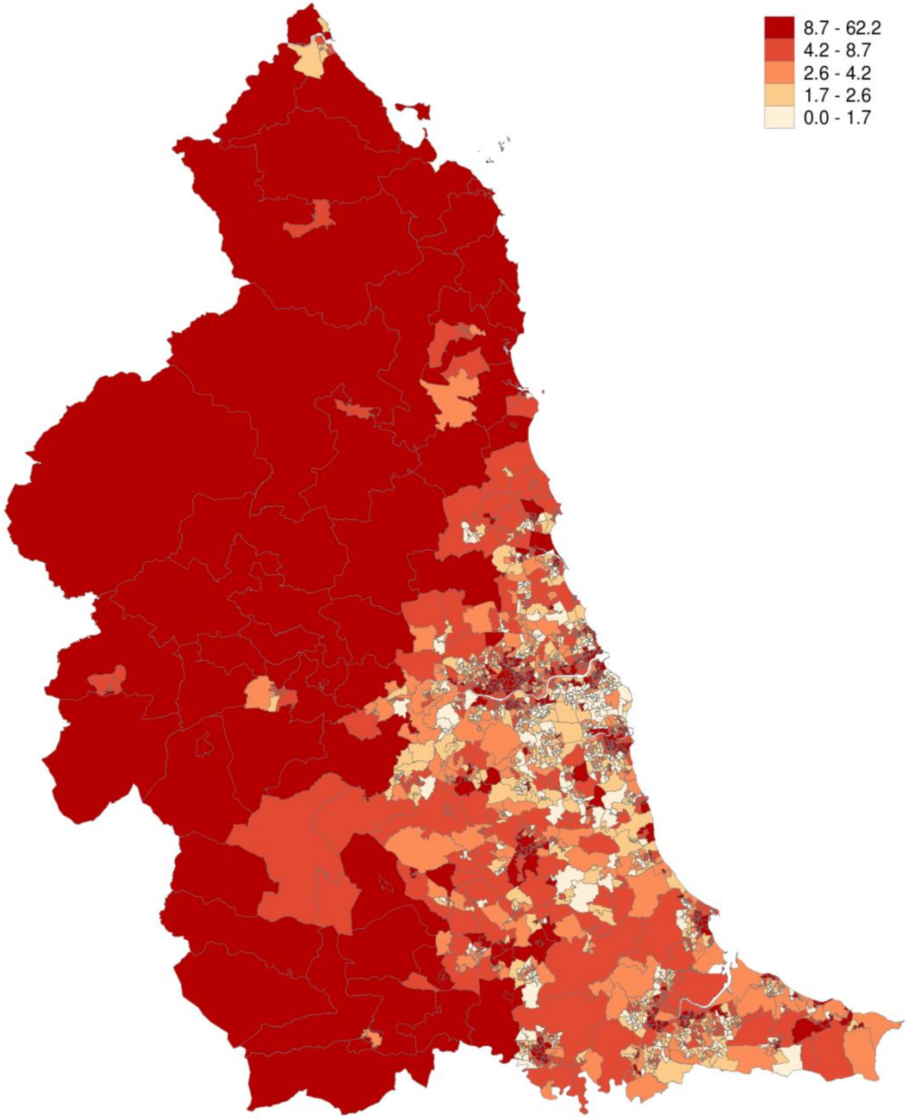

2011

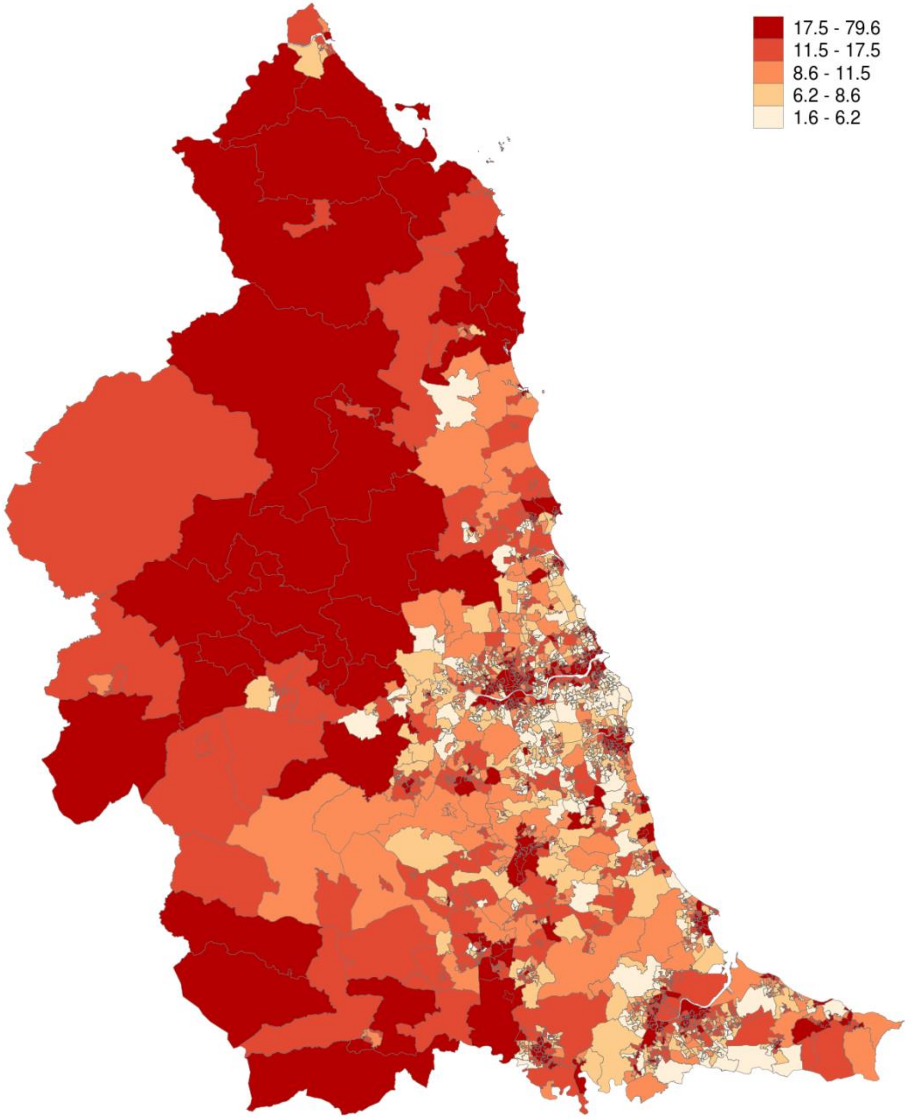

North West, Private Renting % of all households

2001

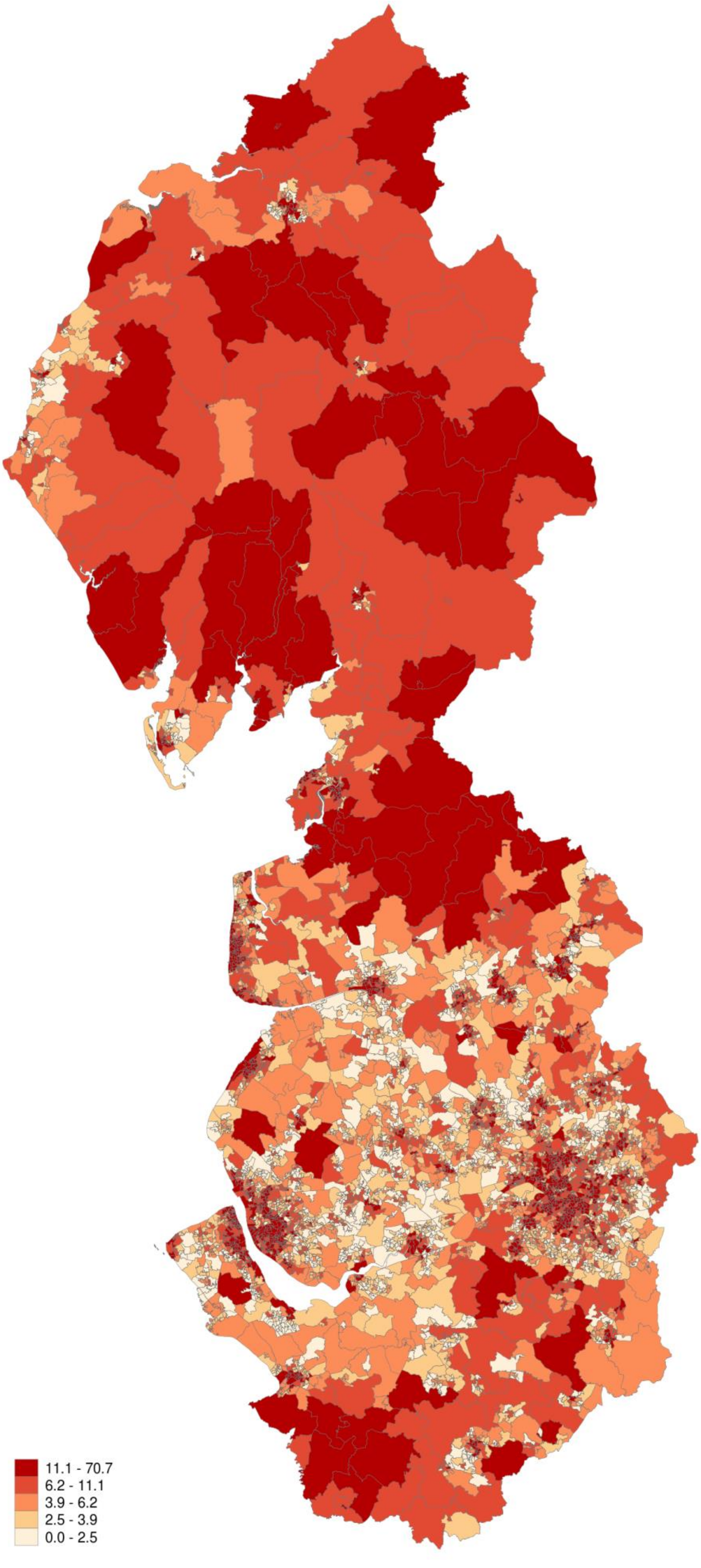

2011

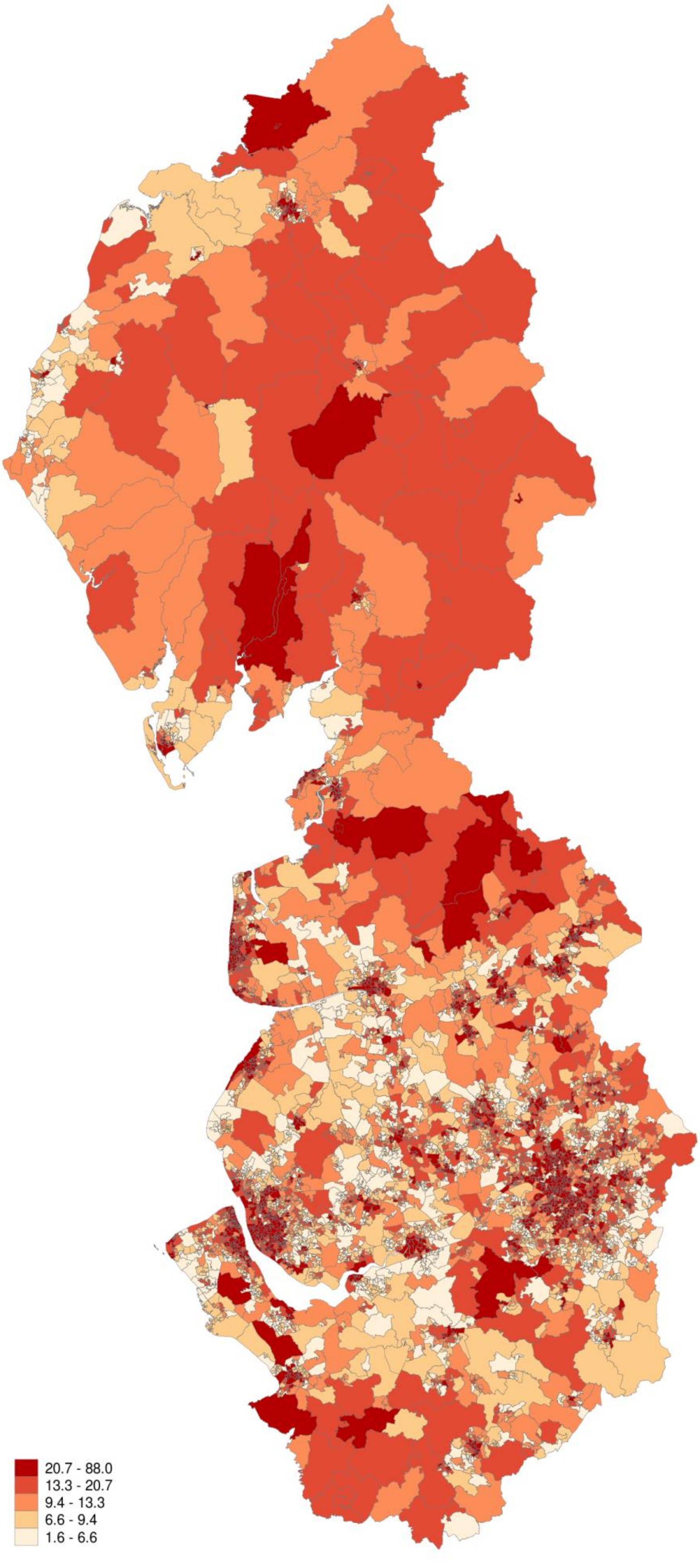

Yorkshire and the Humber, Private Renting % of all households

2001

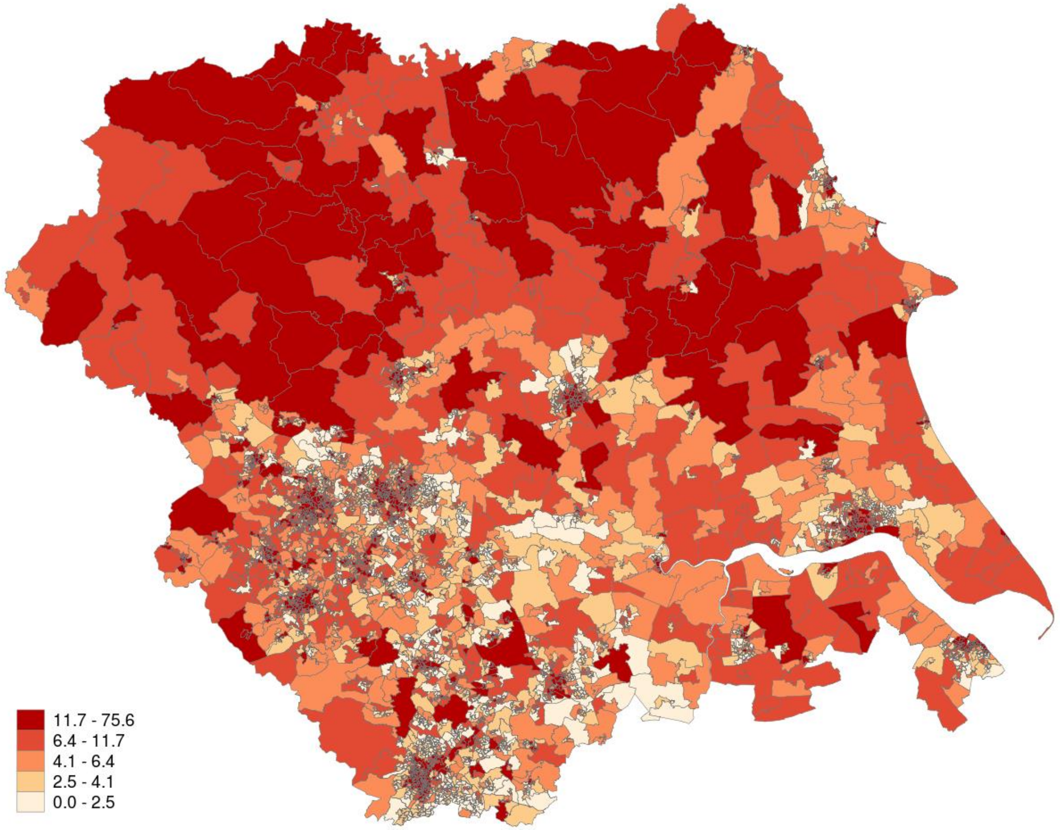

2011

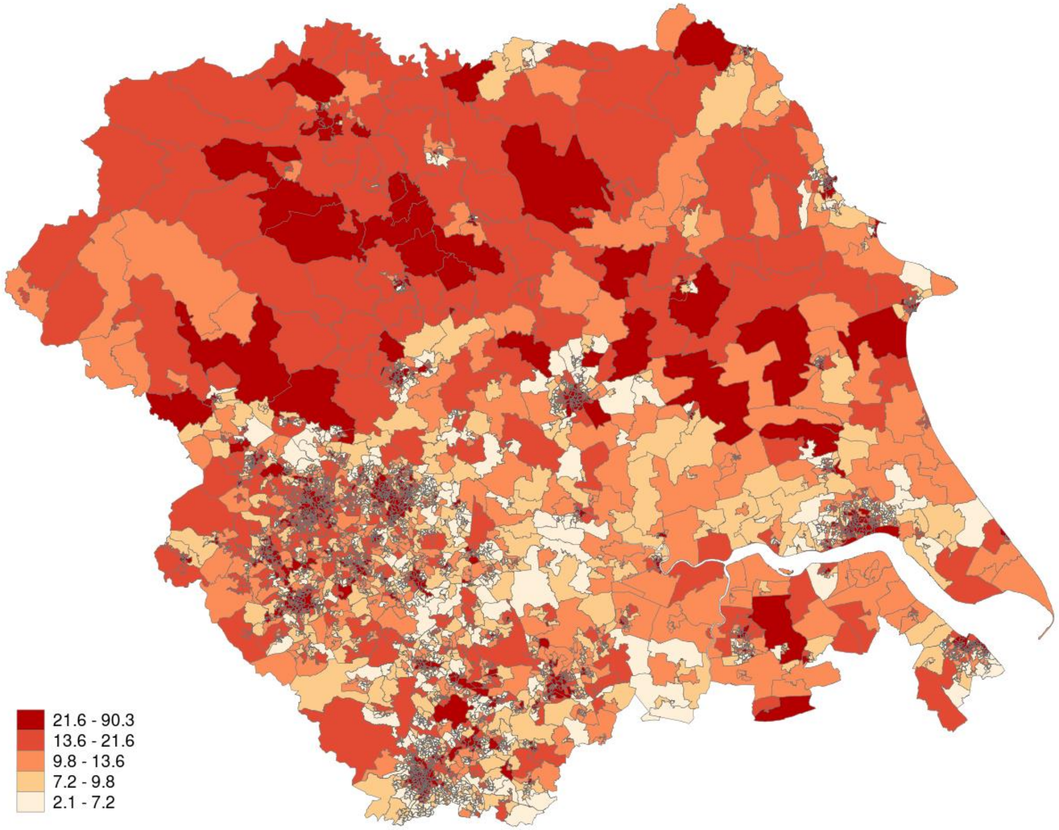

East Midlands, Private Renting % of all households

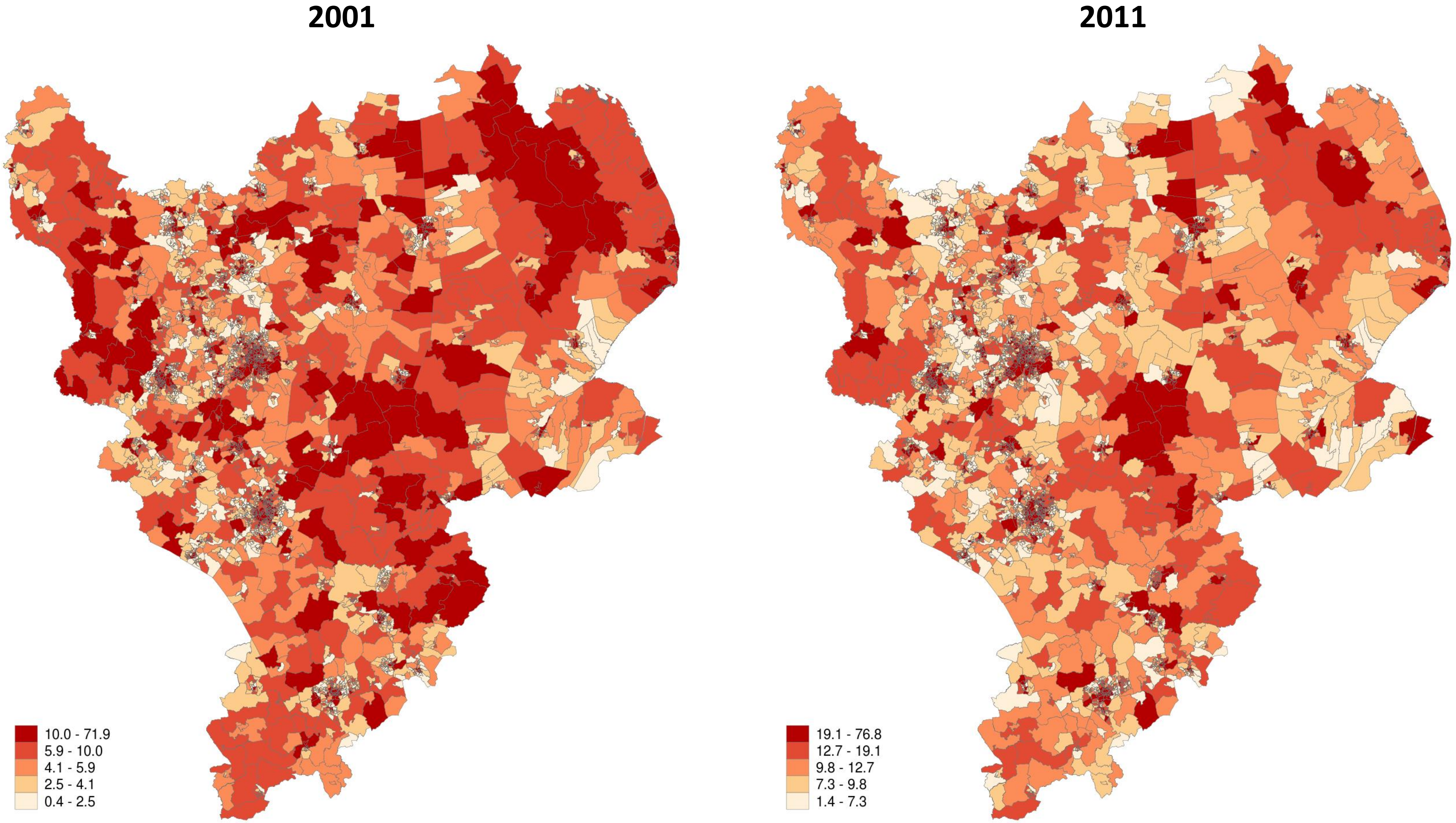

West Midlands, Private Renting % of all households

2001

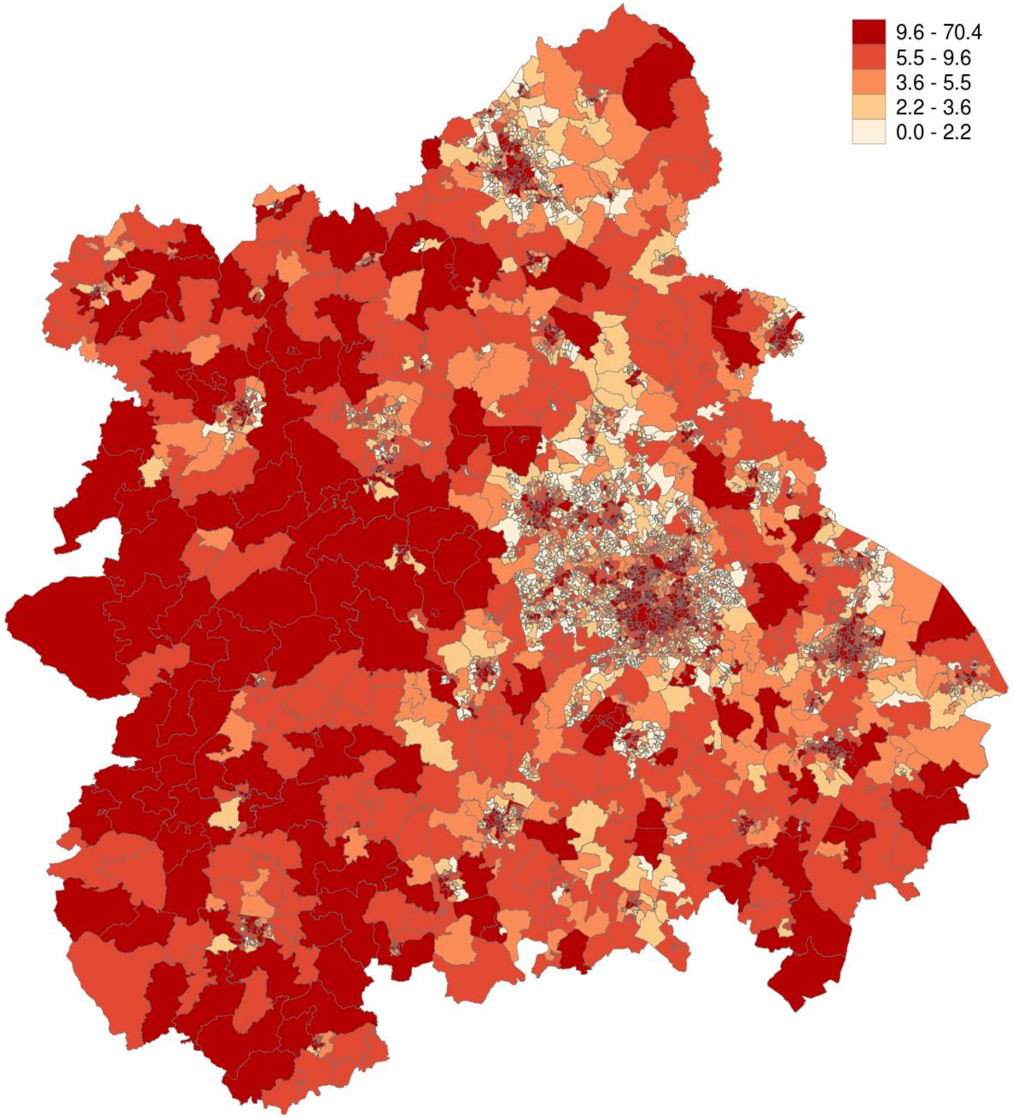

2011

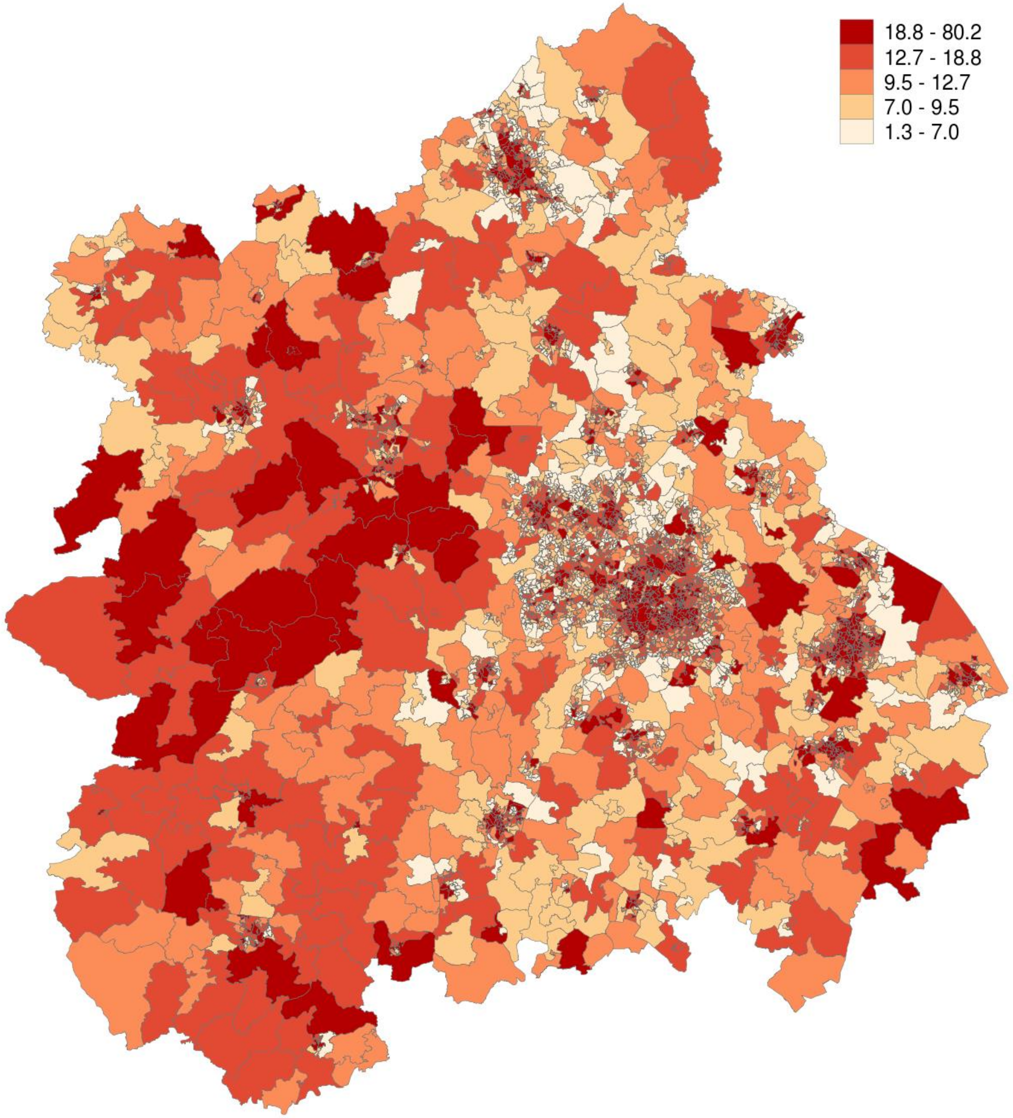

East of England, Private Renting % of all households

2001 **2011**

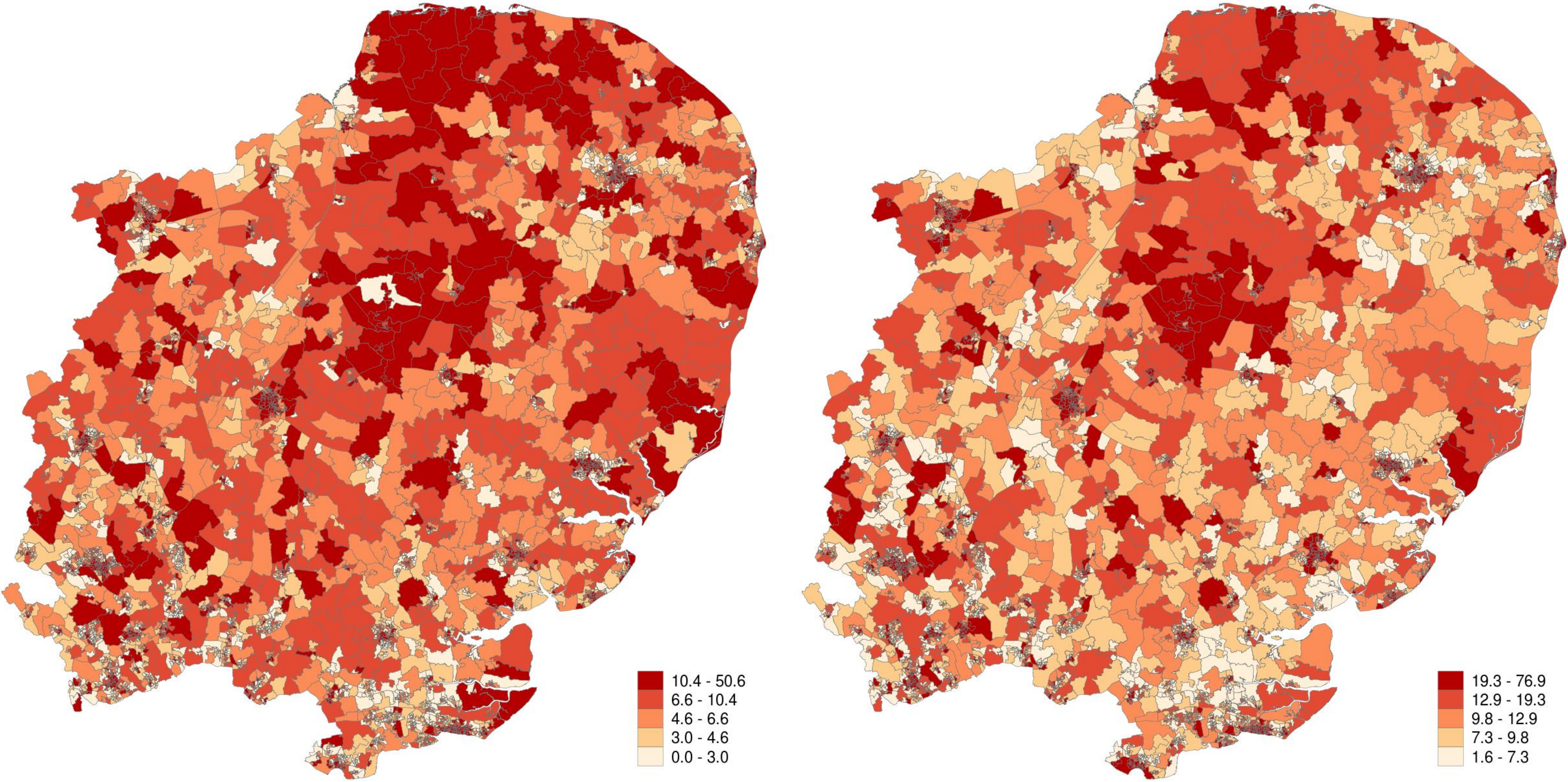

London, Private Renting % of all households

2001

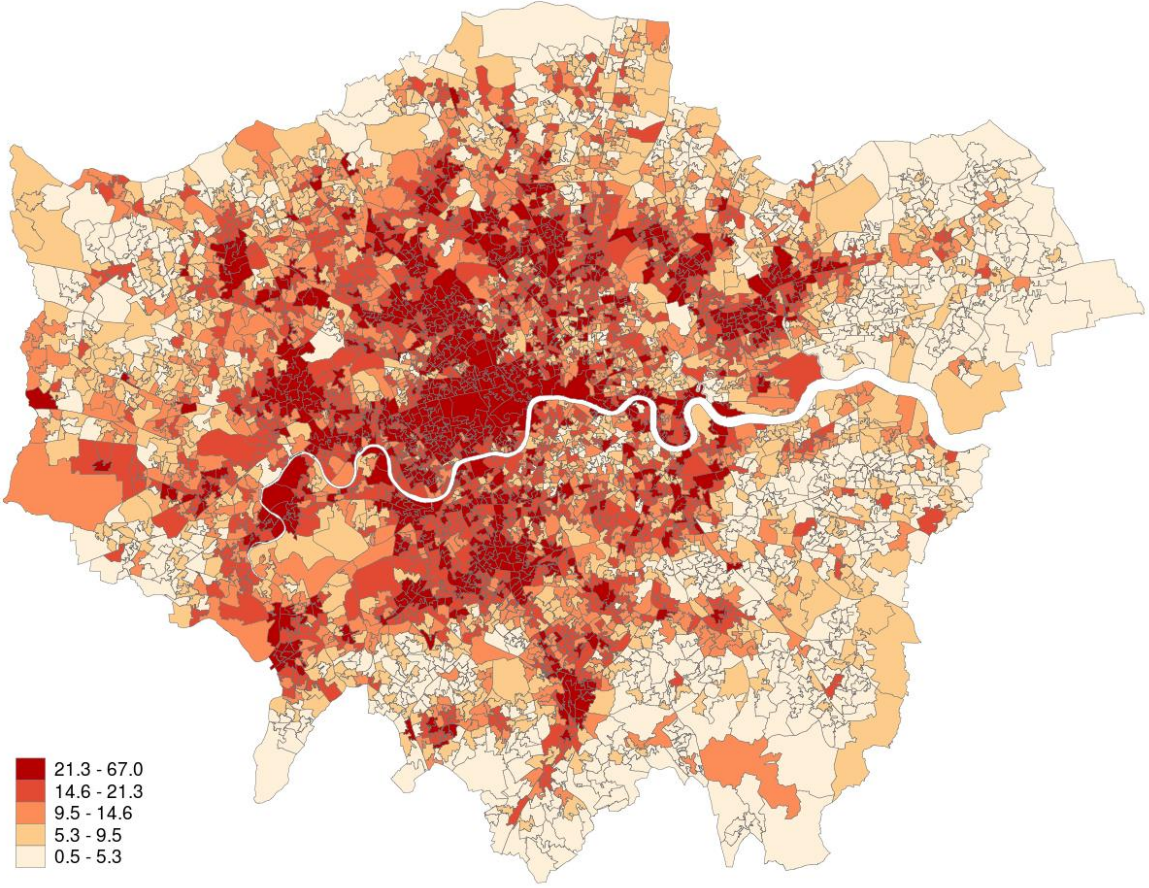

2011

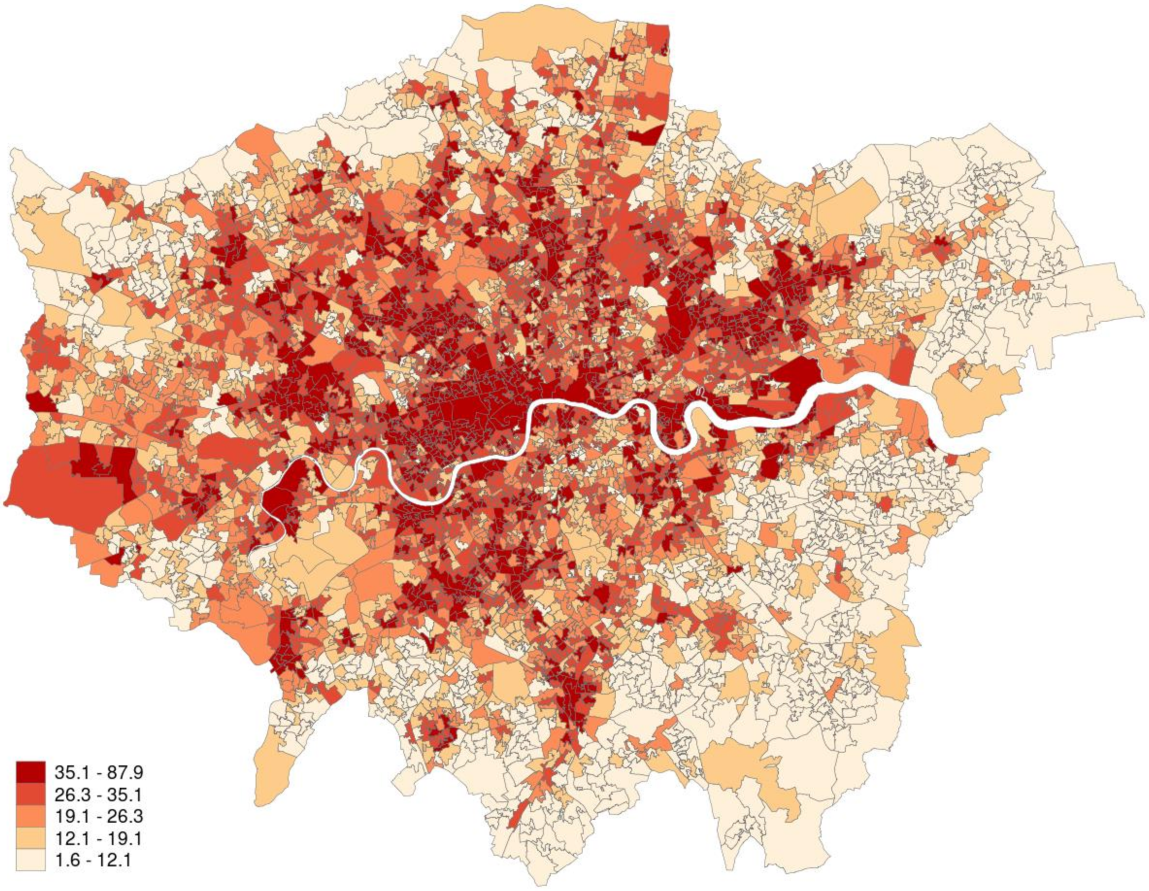

South East Coast, Private Renting % of all households

2001

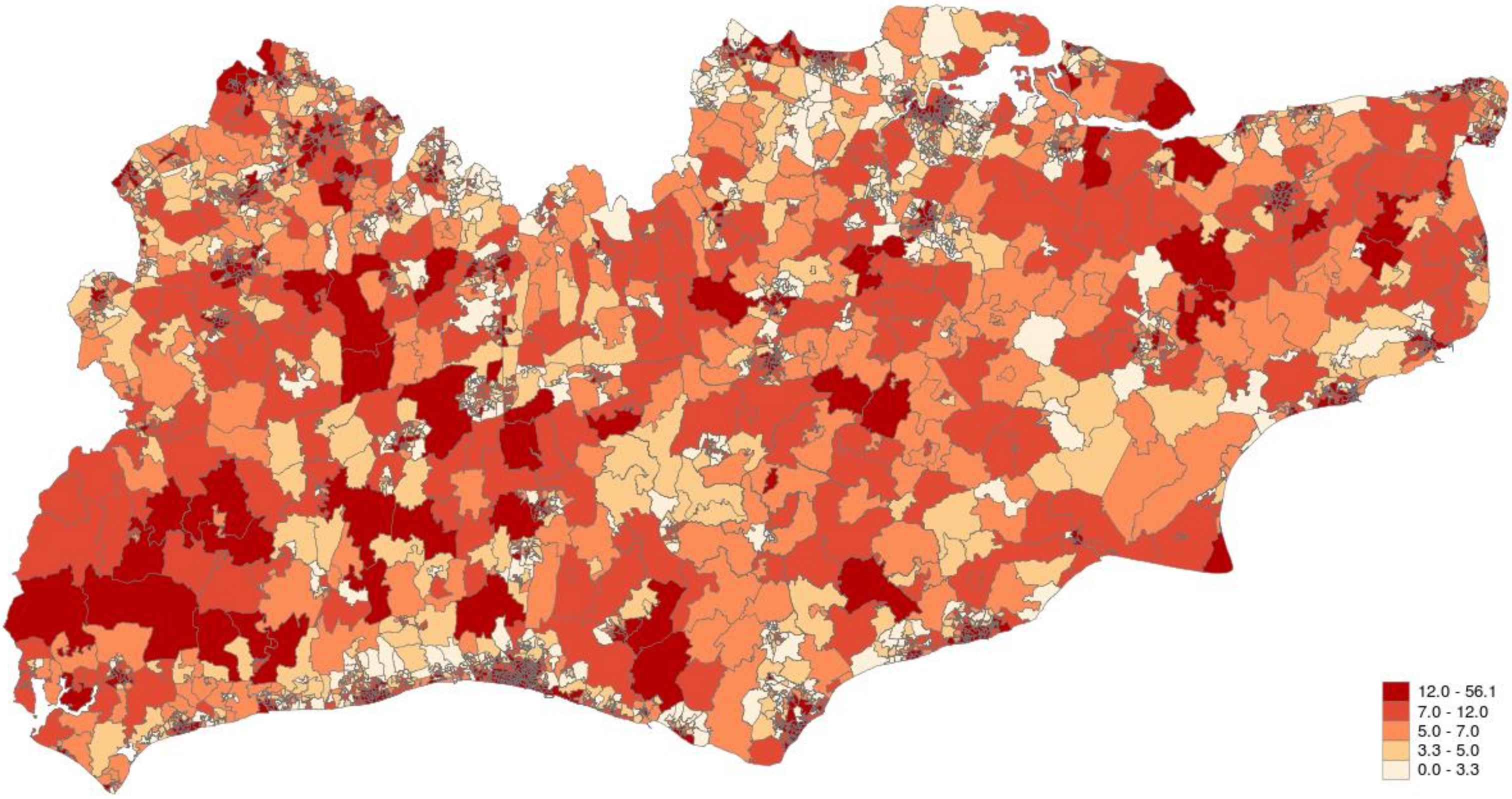

2011

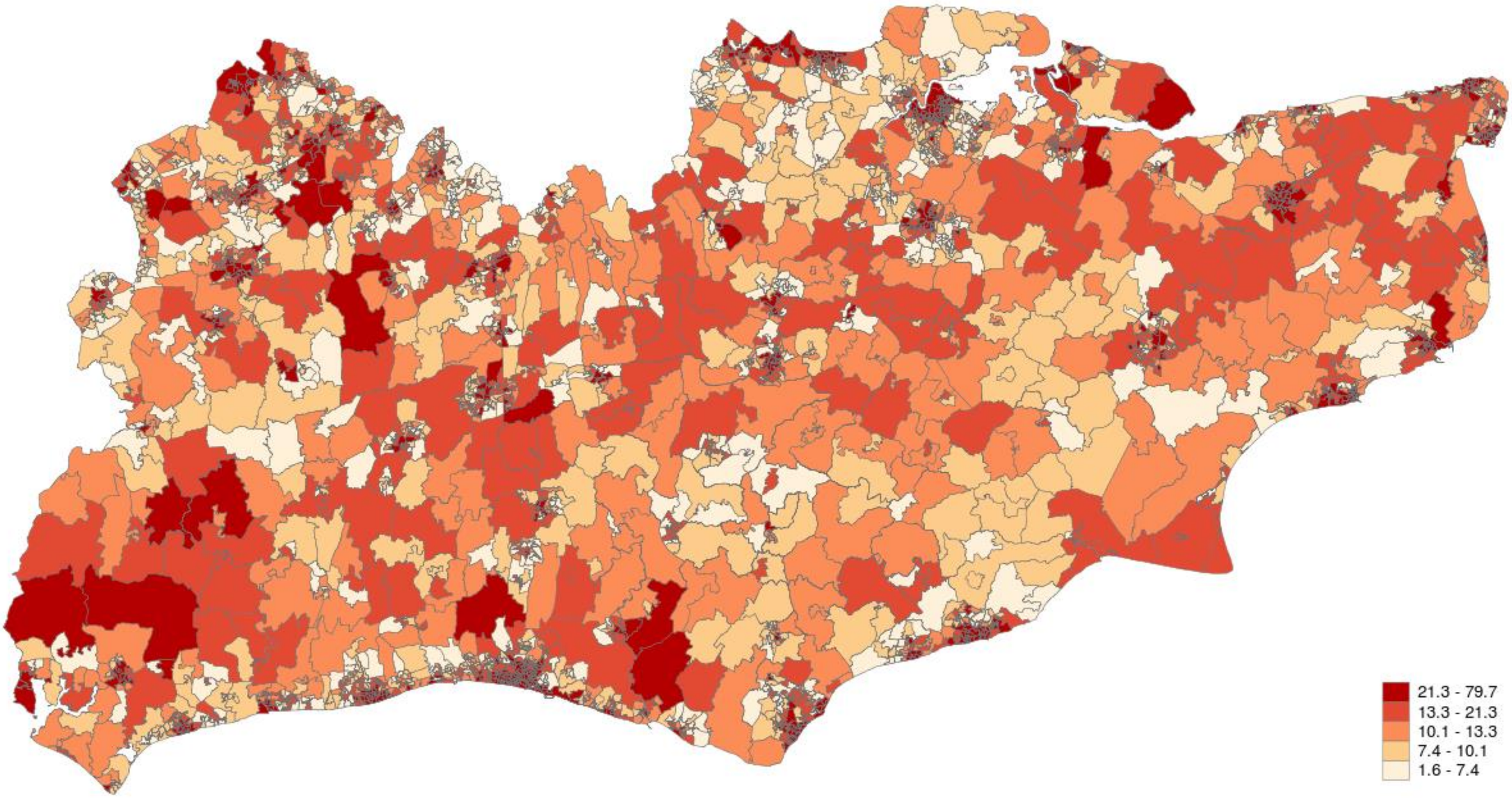

South Central, Private Renting % of all households

2001

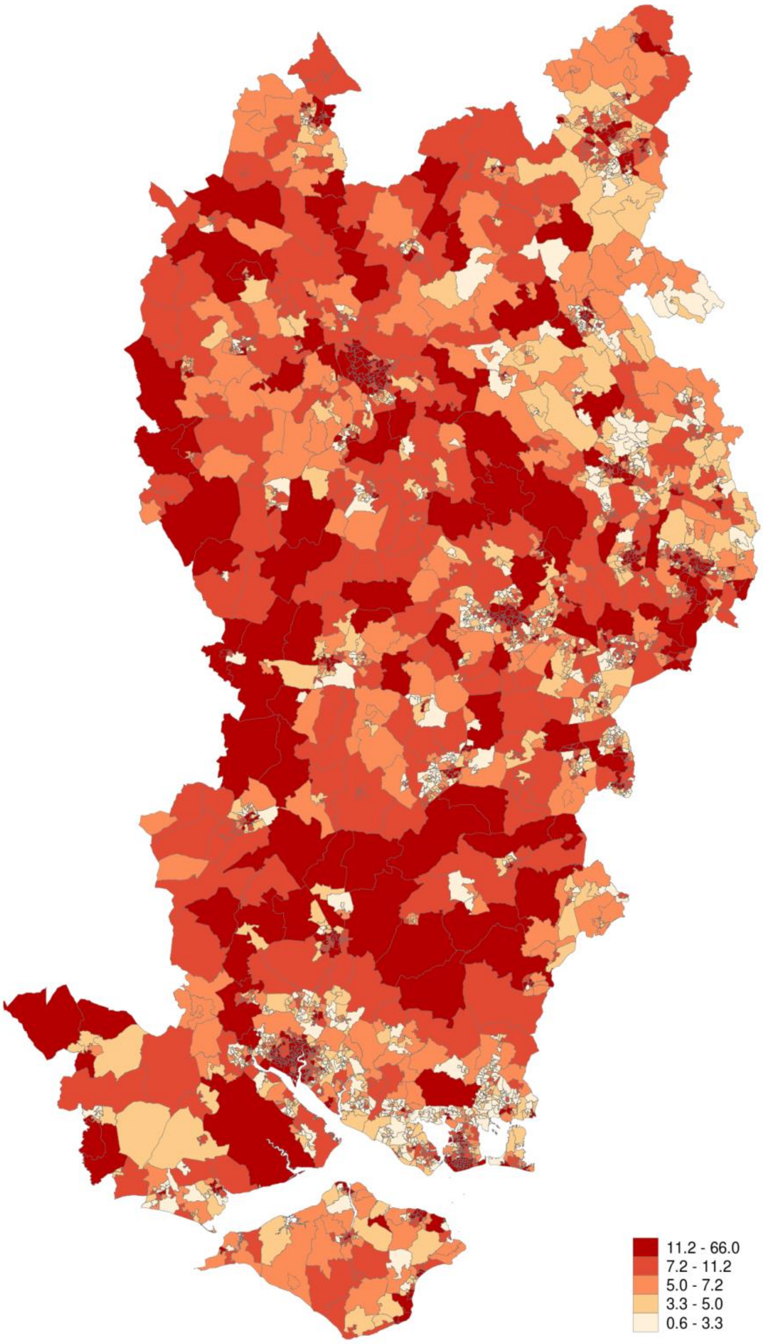

2011

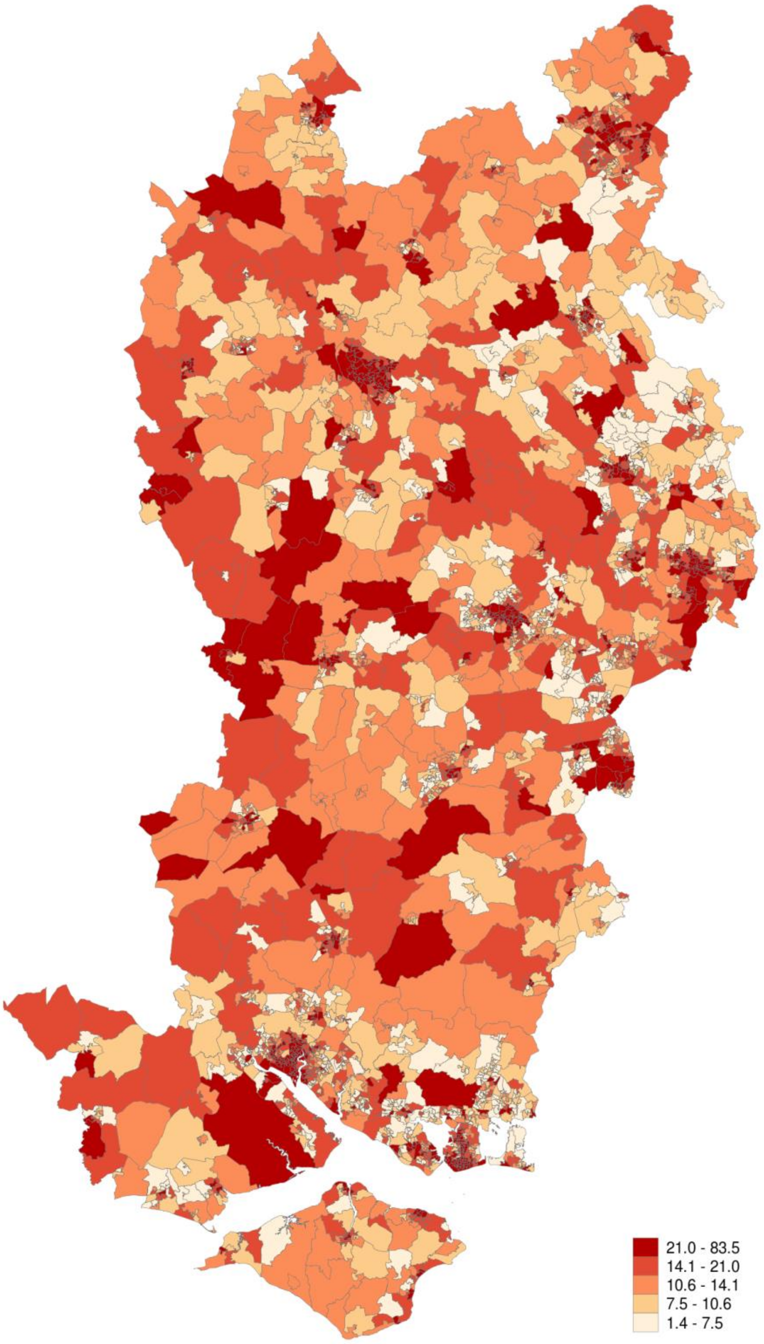

South West, Private Renting % of all households

2001

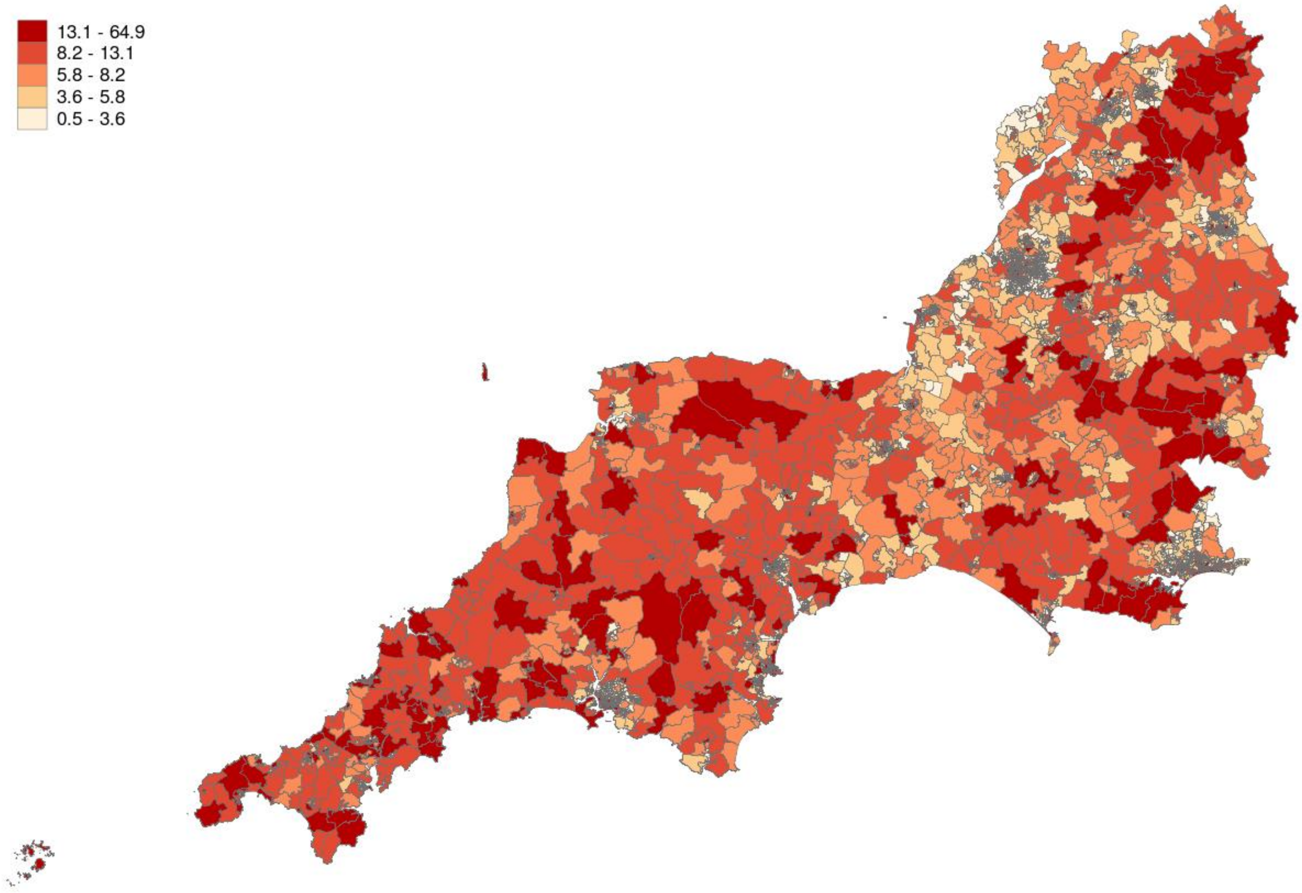

2011

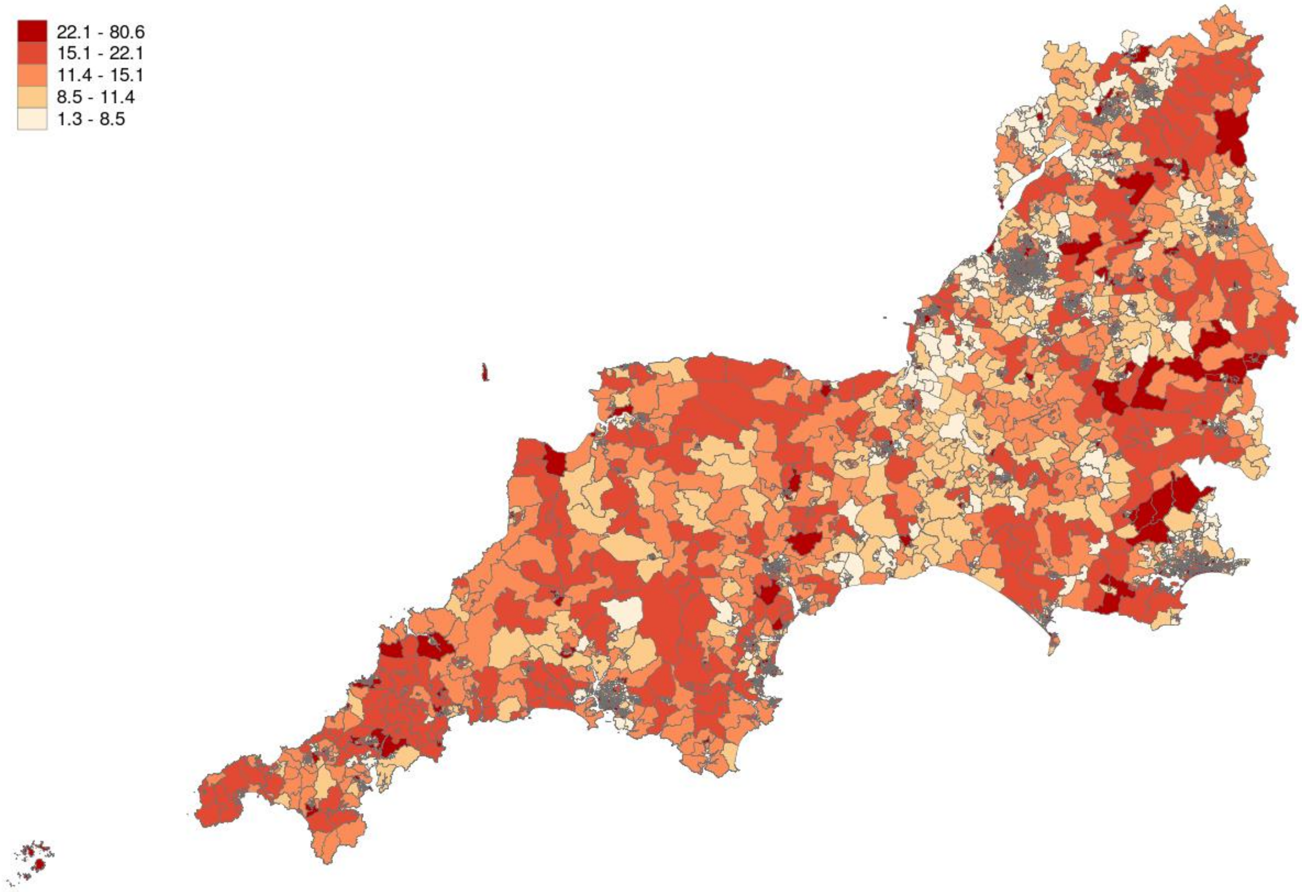

Population Turnover

England 2001, Population Turnover % of population

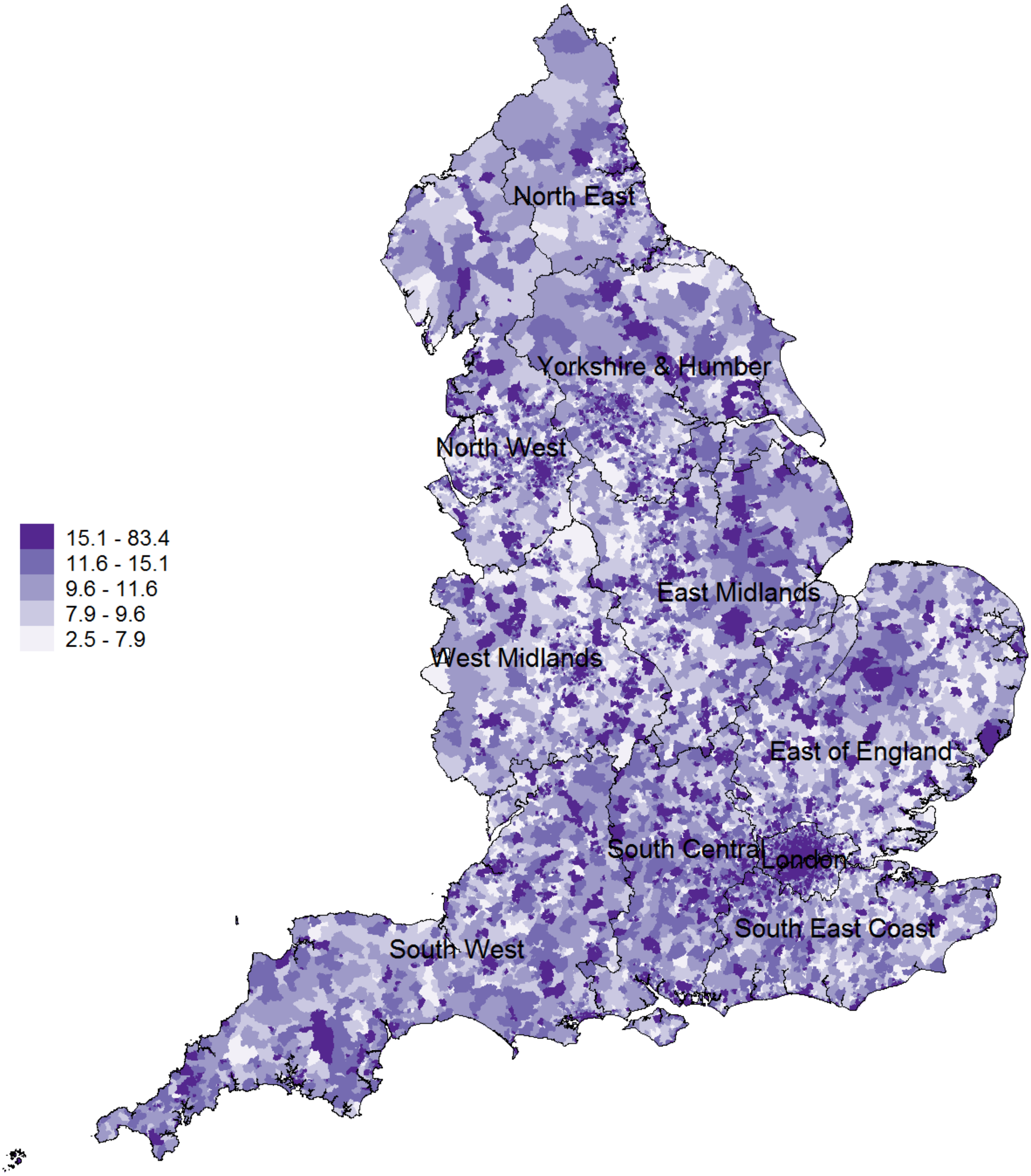

England 2011, Population Turnover % of population

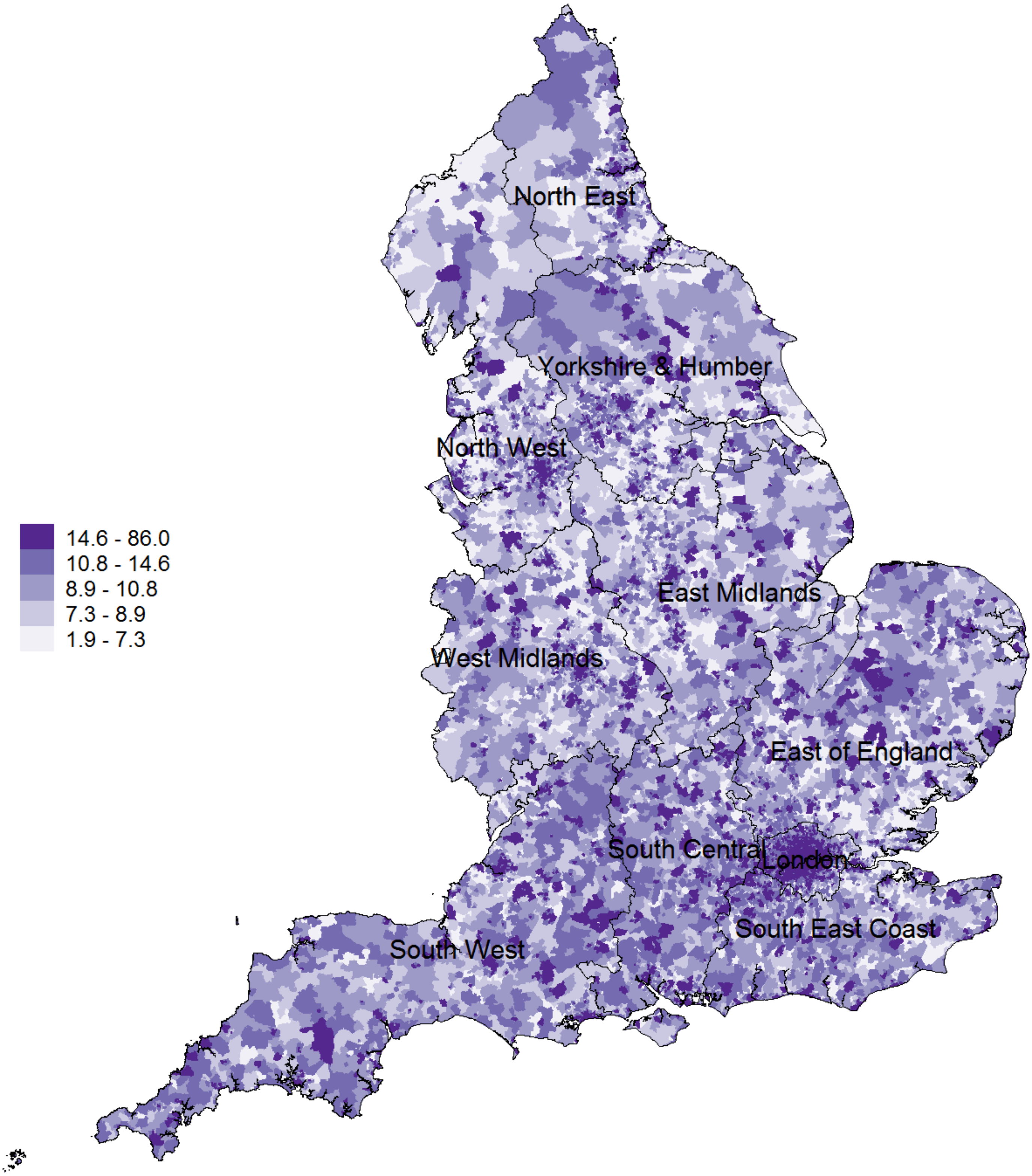

North East, Population Turnover % of population

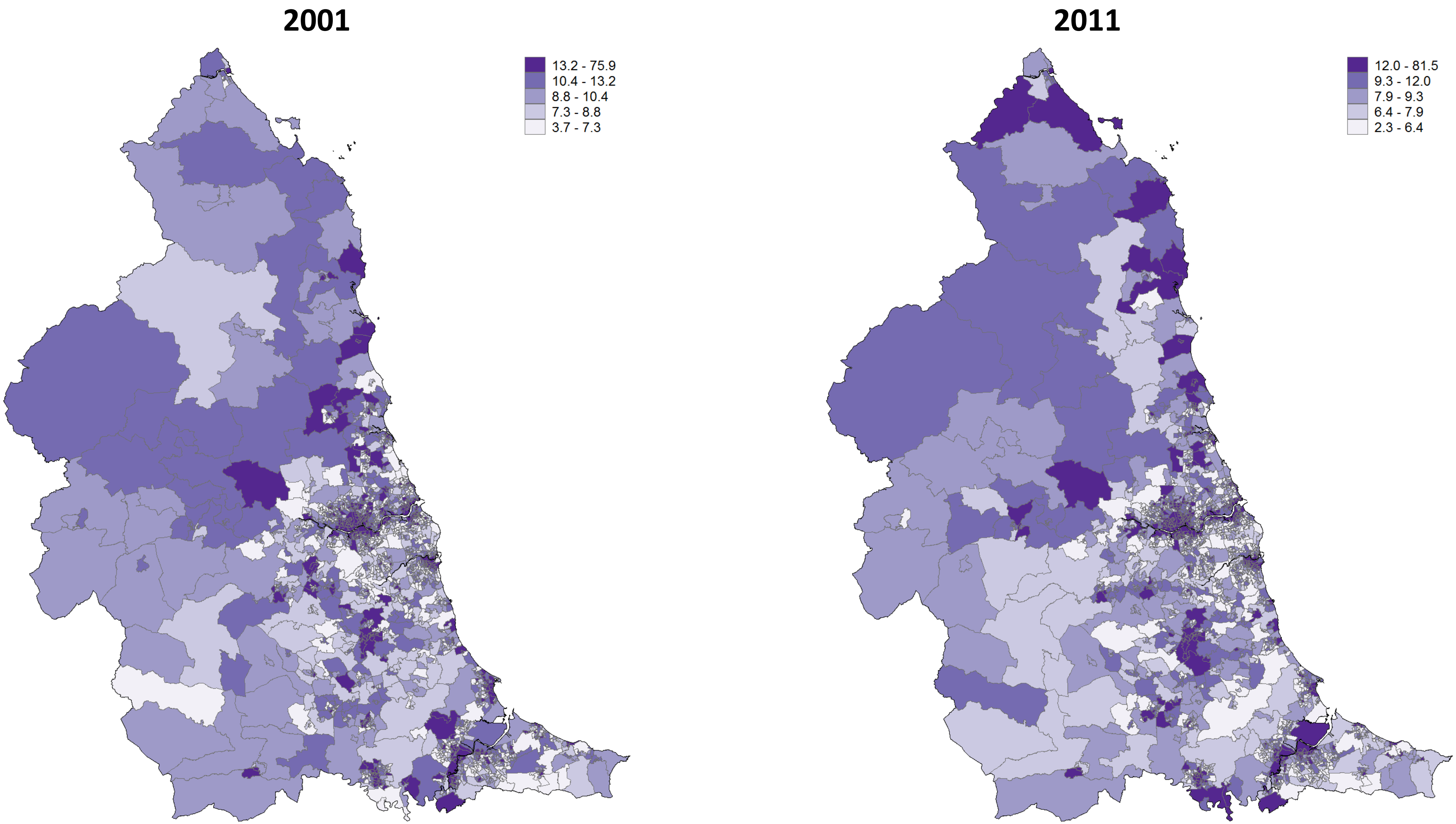

North West, Population Turnover % of population

2001

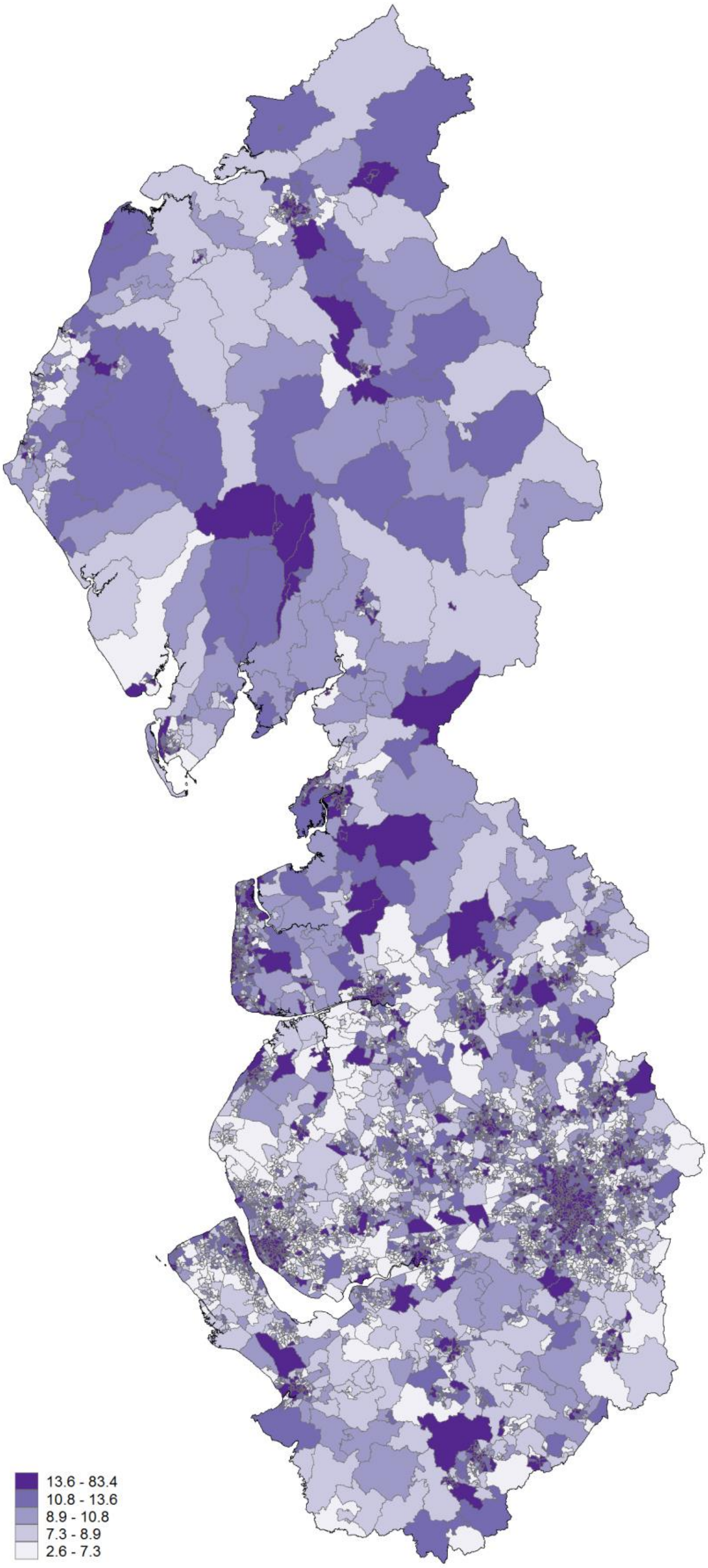

2011

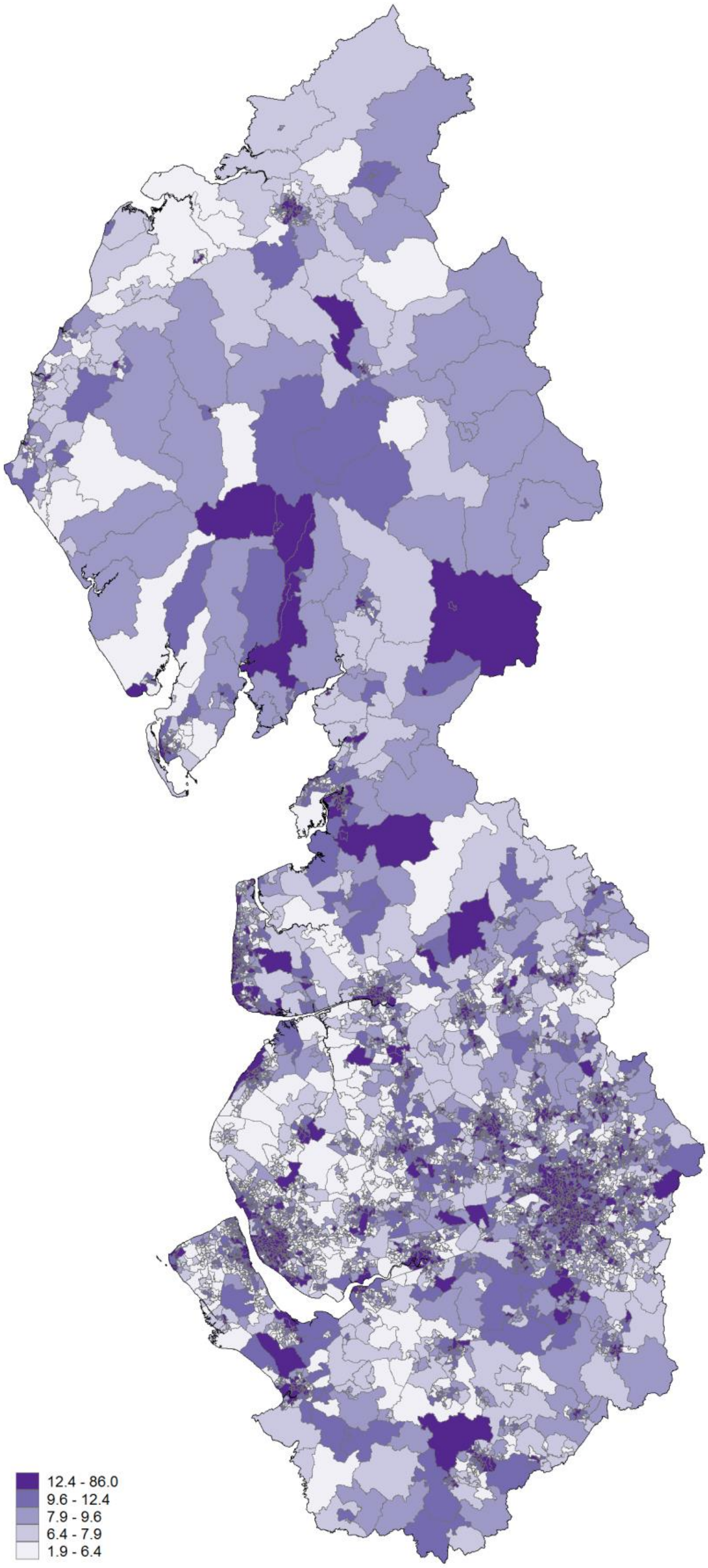

Yorkshire and the Humber, Population Turnover % of population

2001

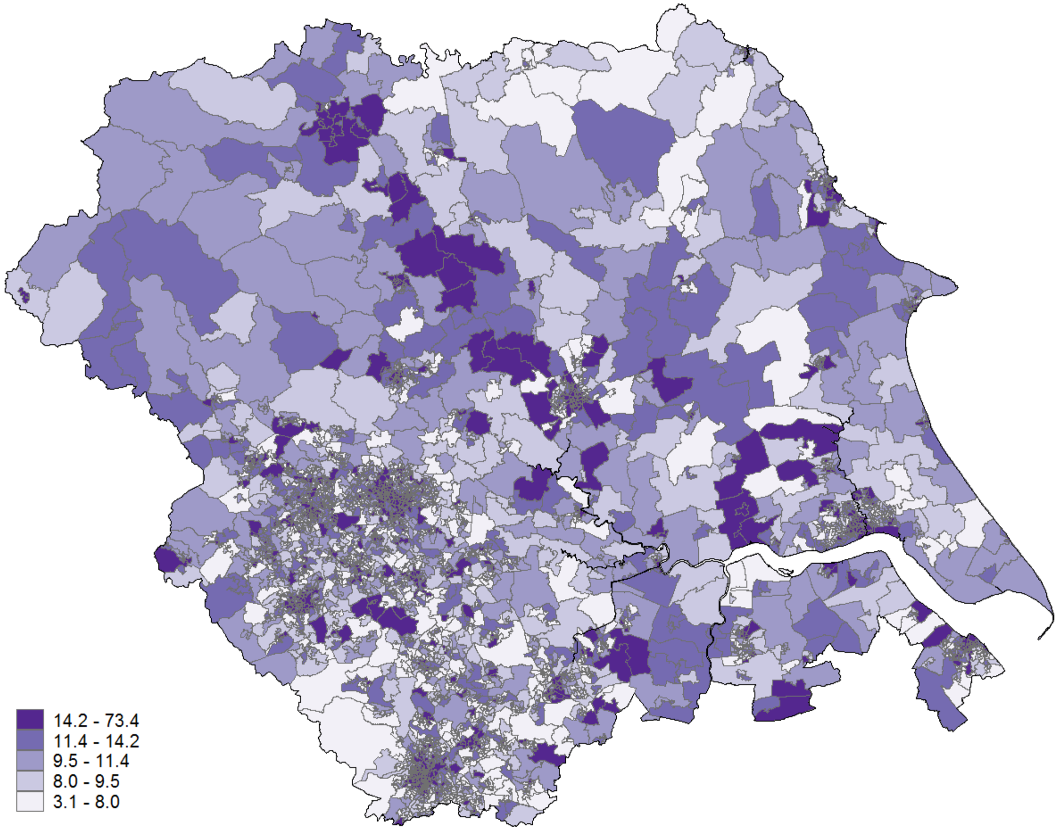

2011

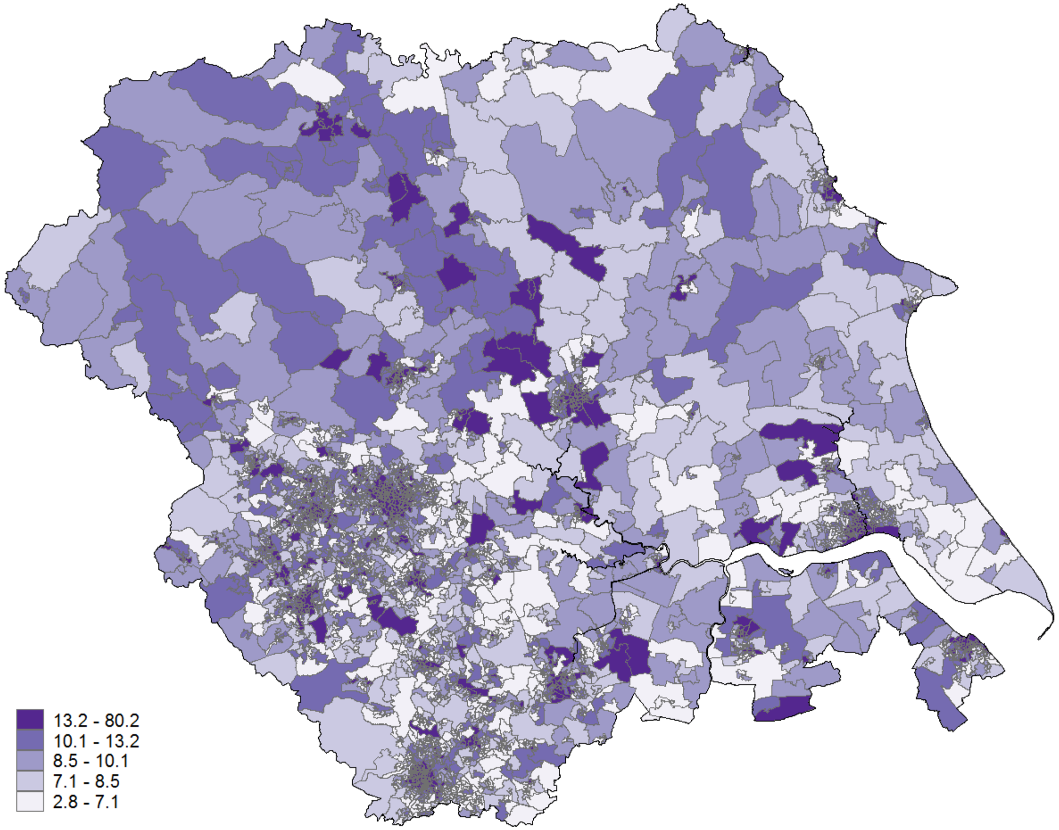

East Midlands, Population Turnover % of population

2001

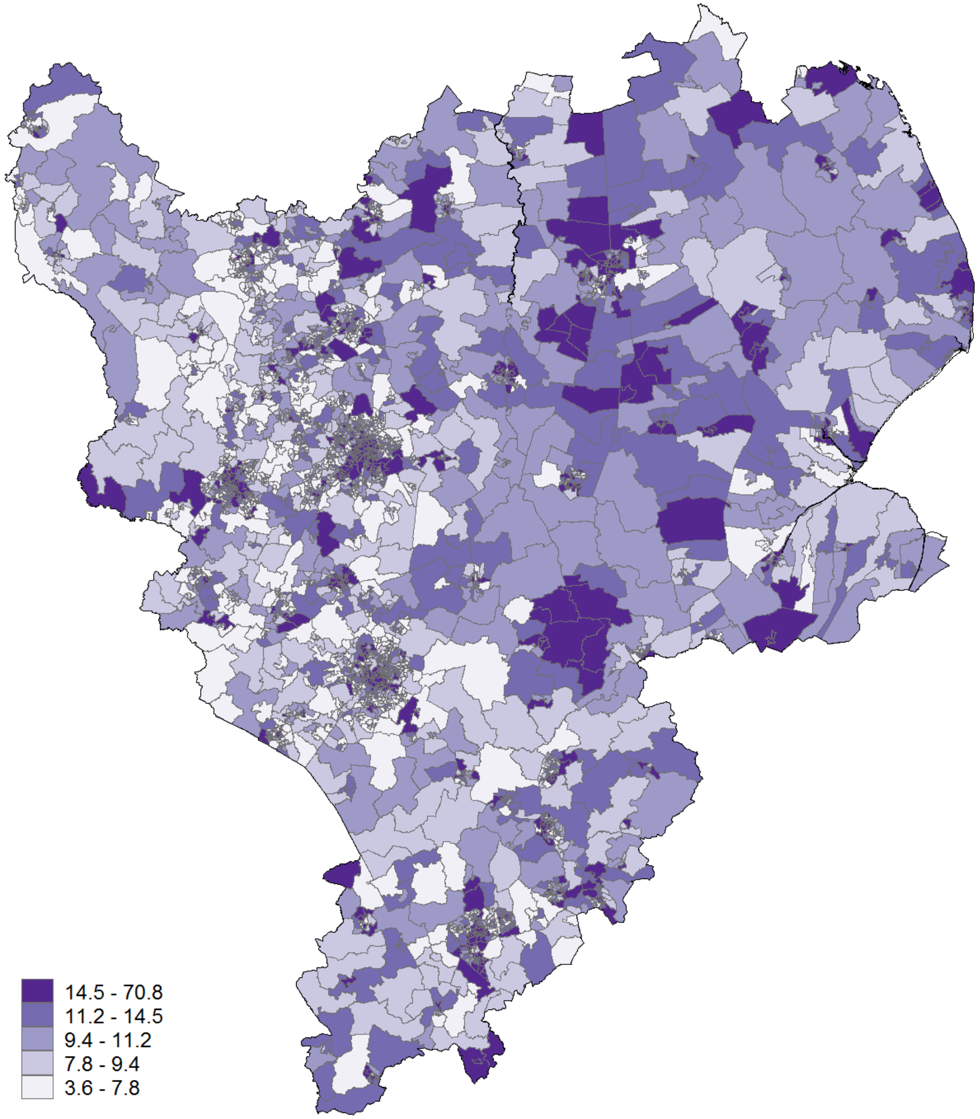

2011

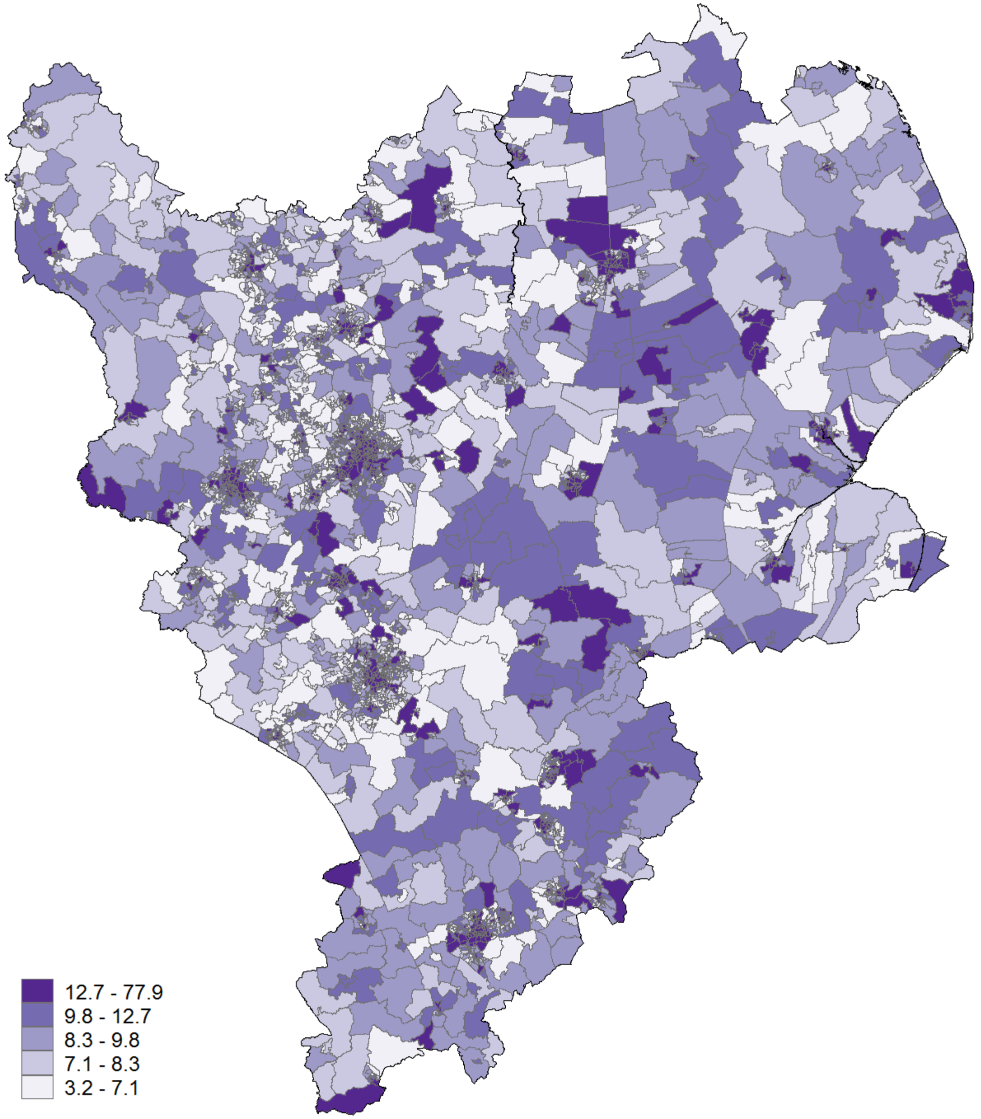

West Midlands, Population Turnover % of population

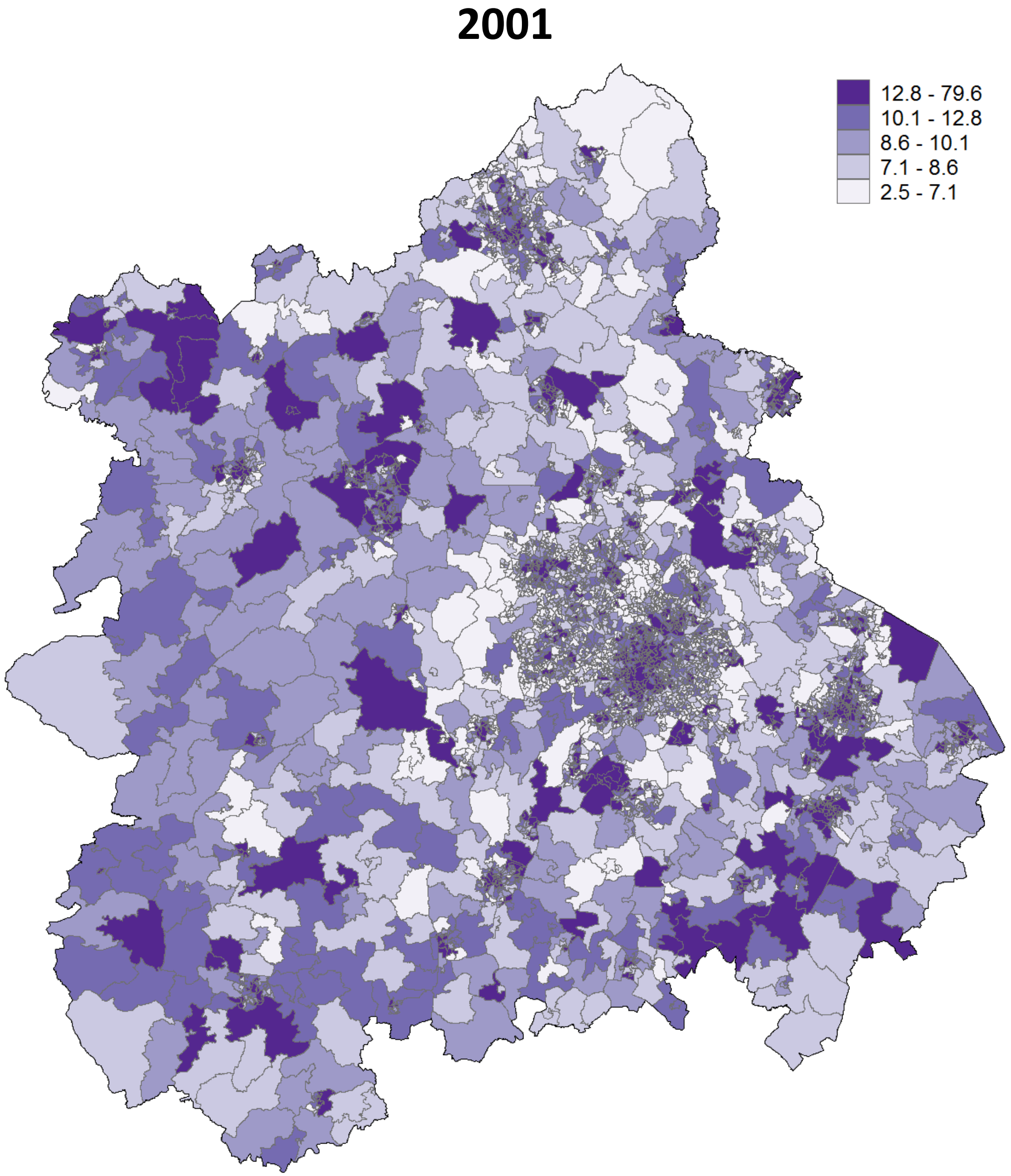

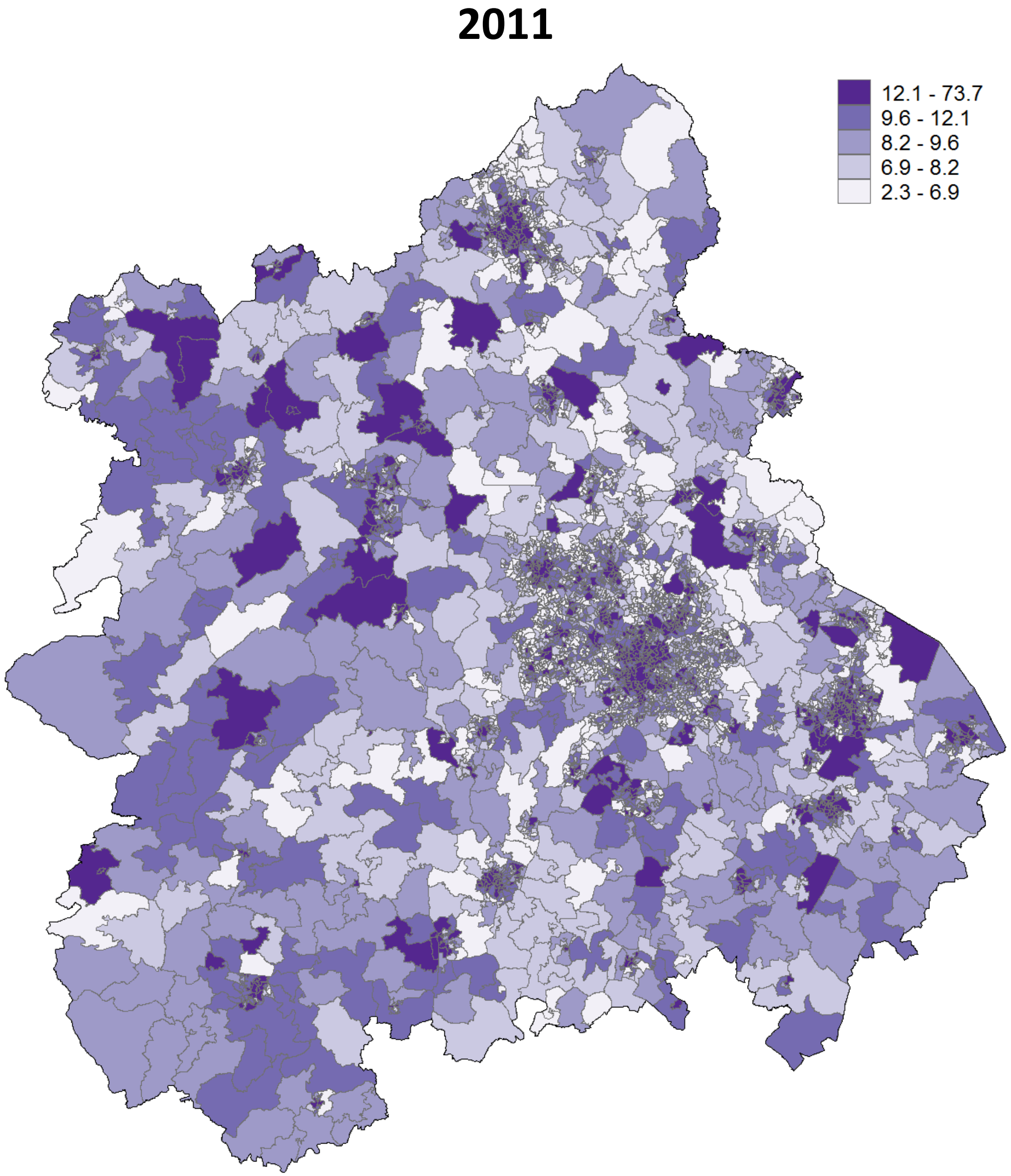

East of England, Population Turnover % of population

2001

1

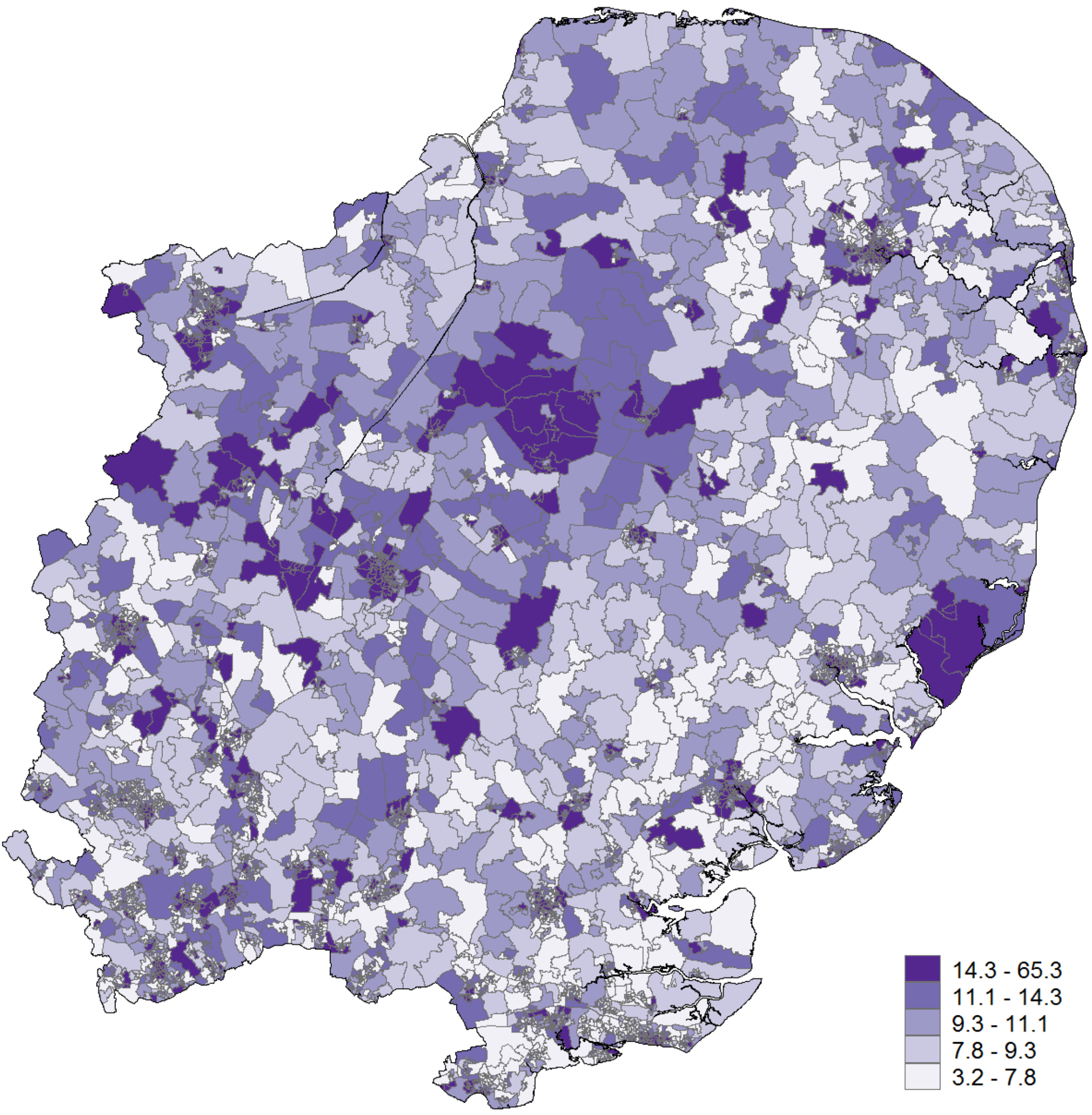

2011

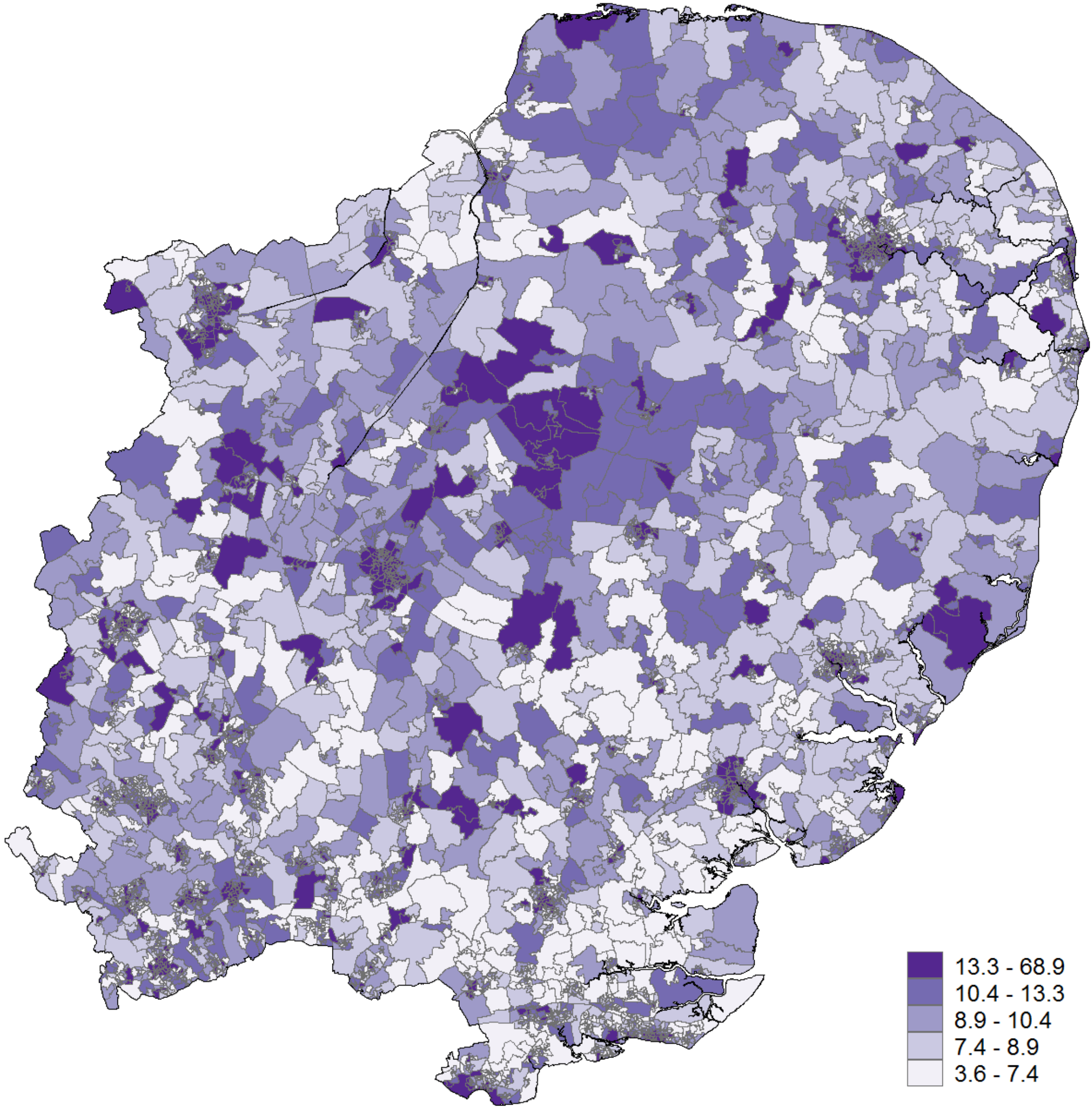

London, Population Turnover % of population

2001

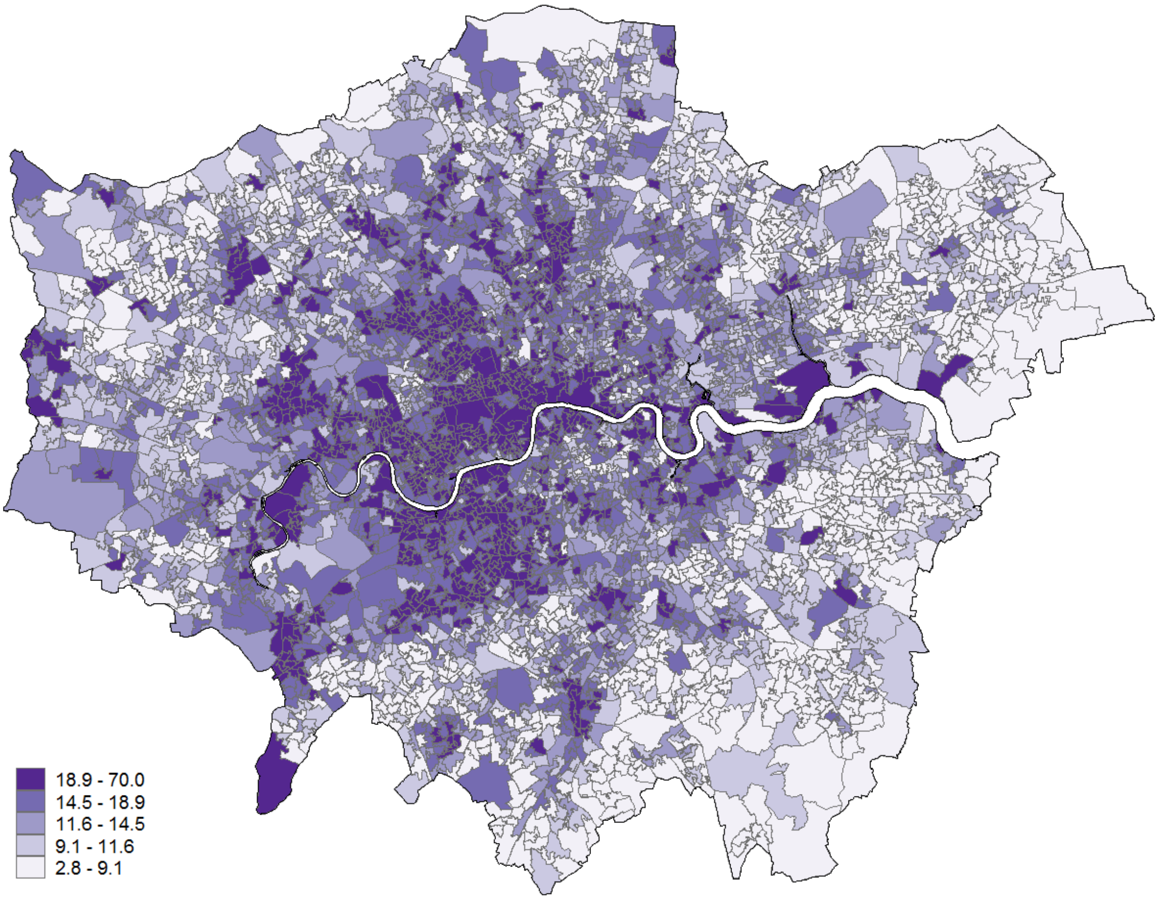

2011

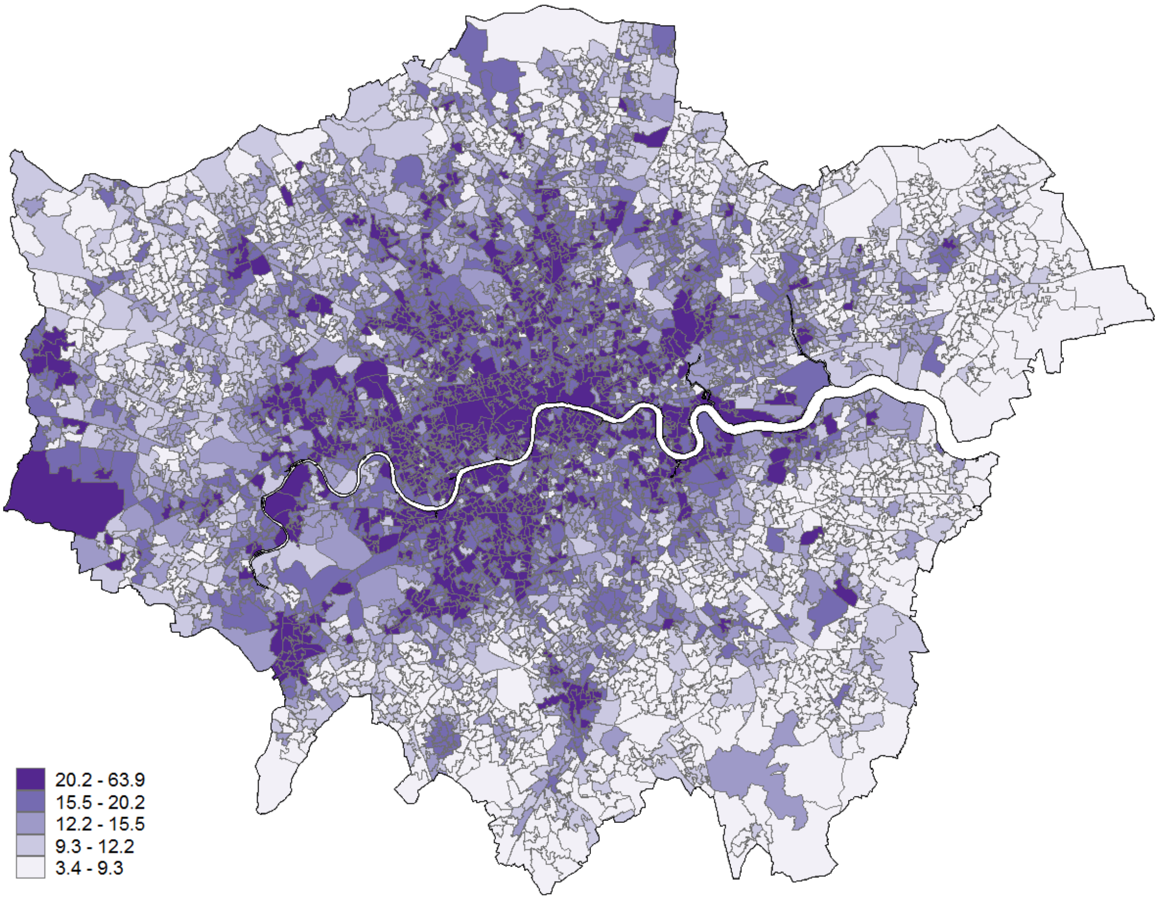

South East Coast, Population Turnover % of population

2001

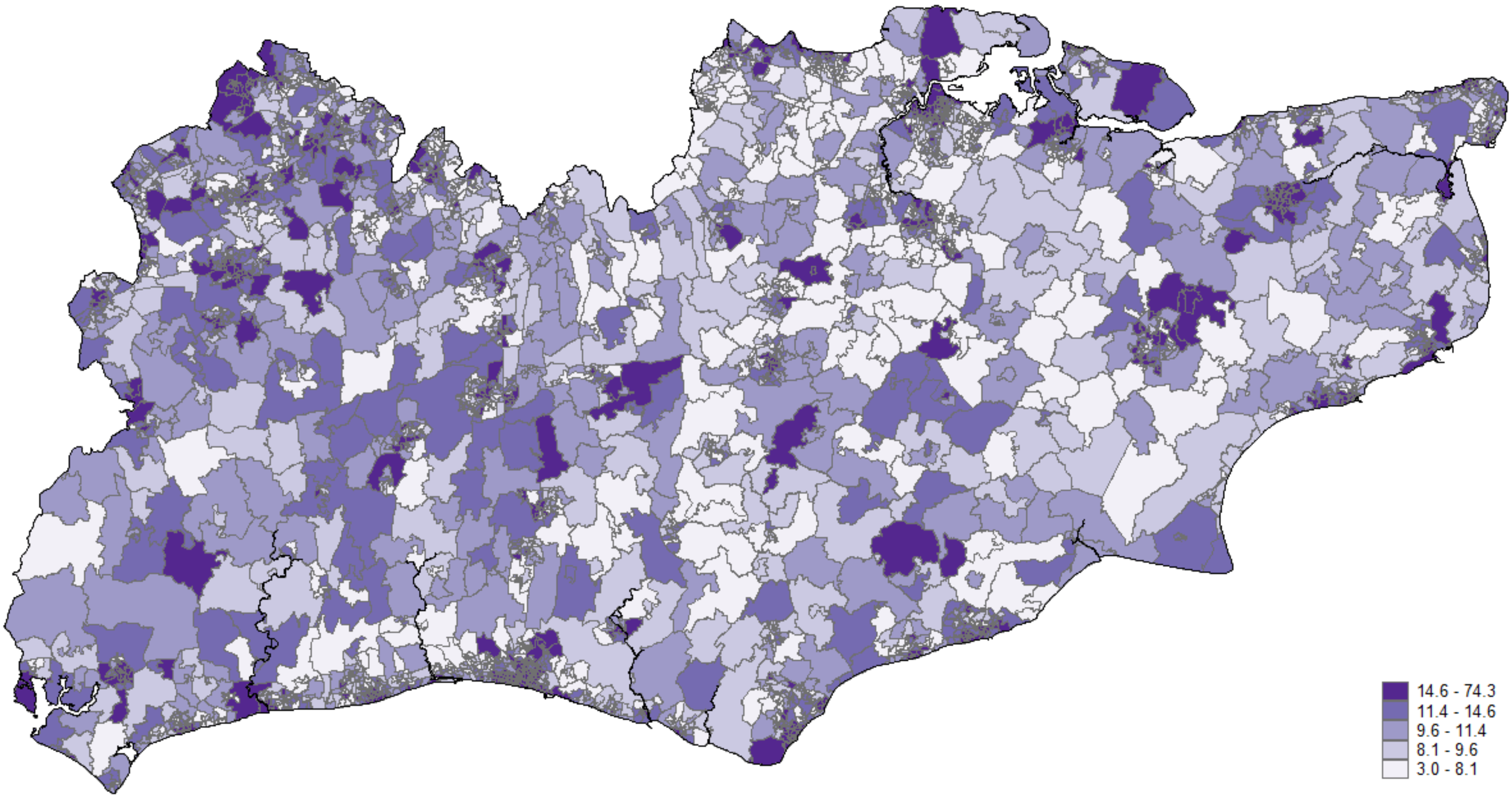

2011

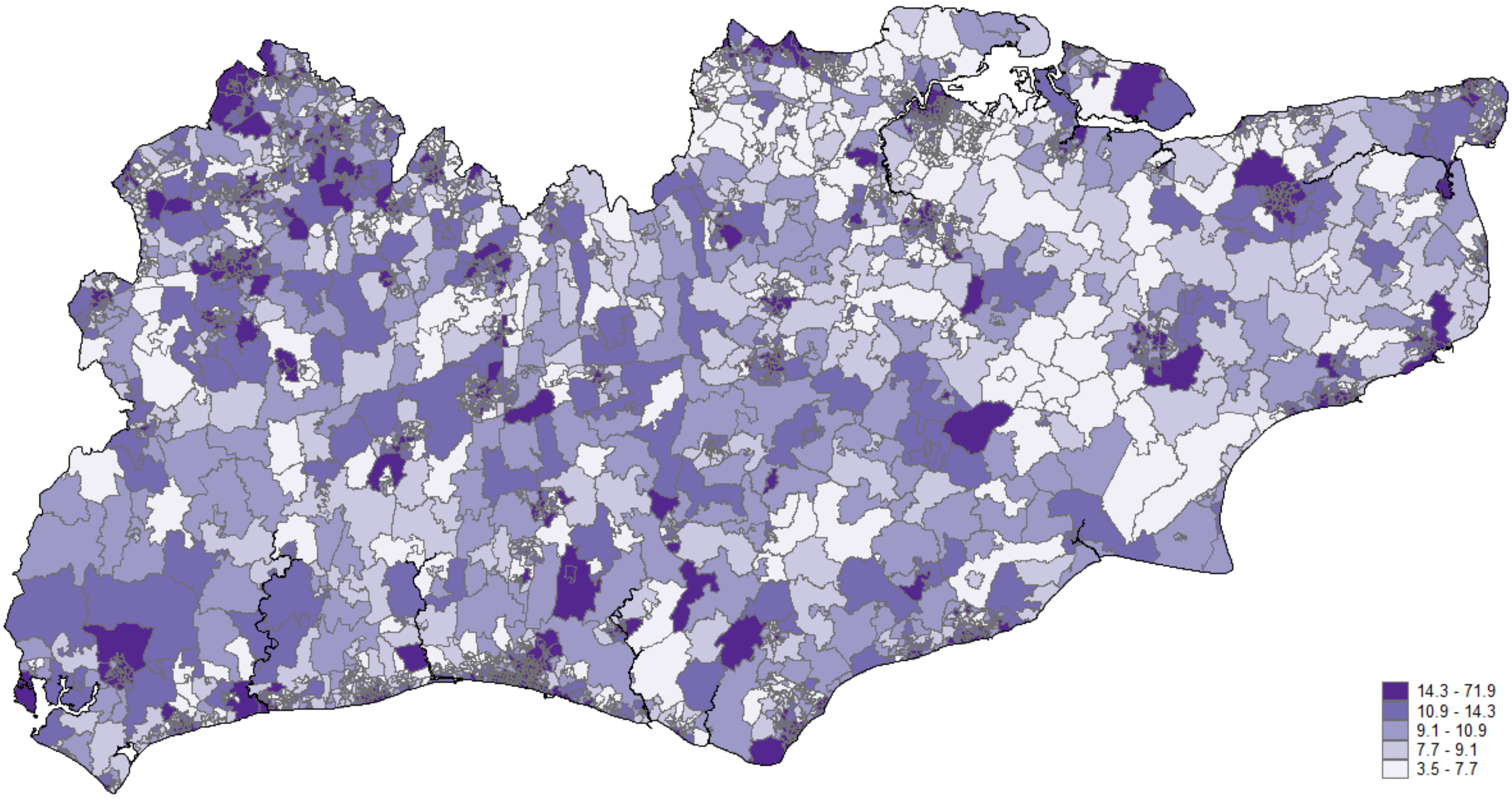

South Central, Population Turnover % of population

2001

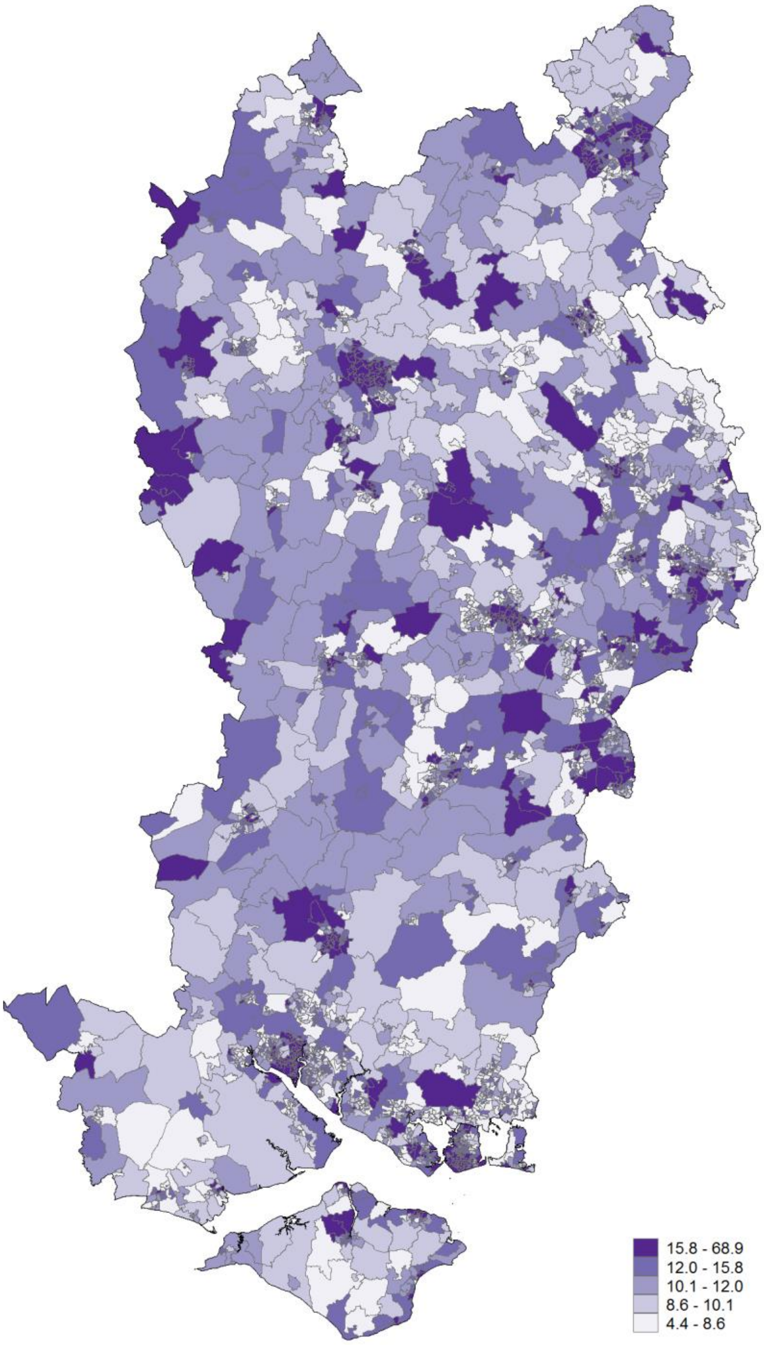

2011

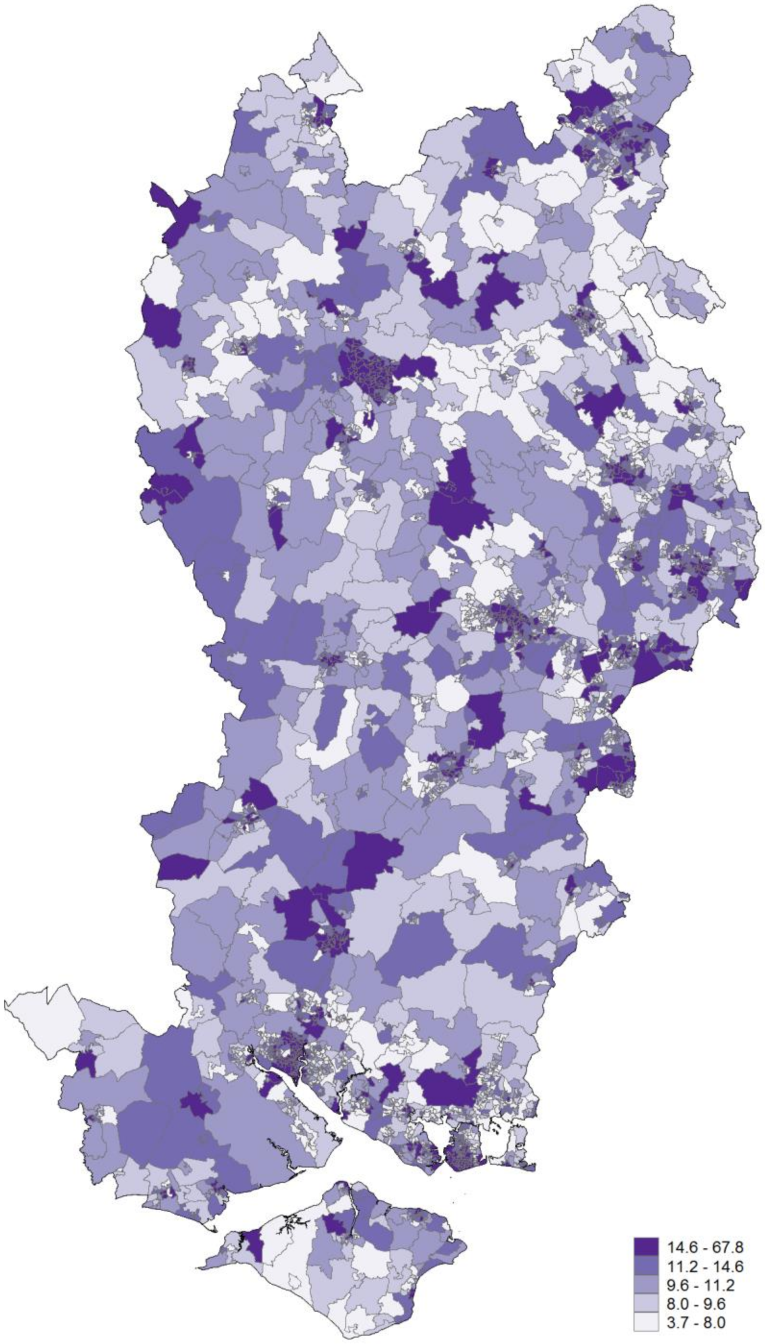

South West, Population Turnover % of population

2001

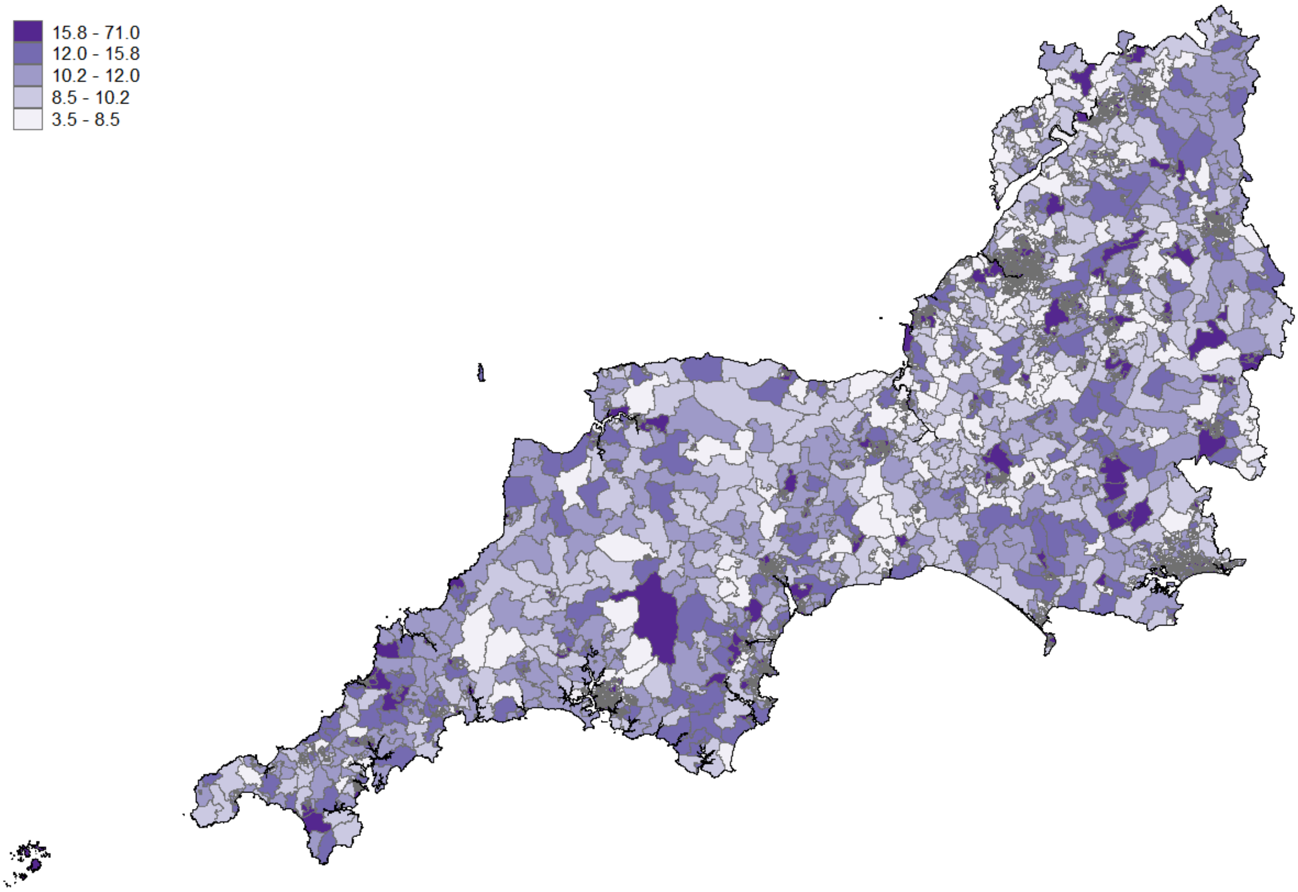

2011

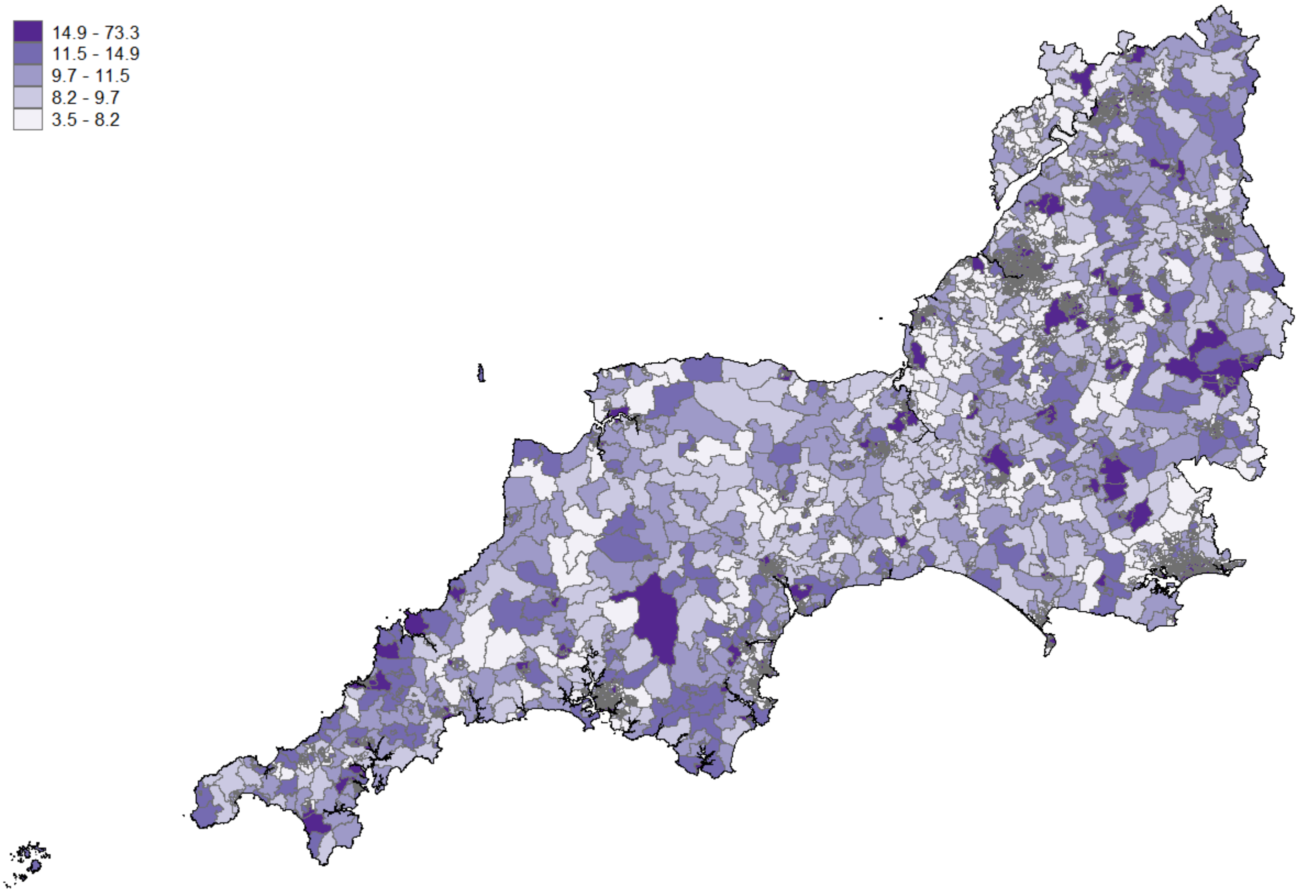

Supplement: Supplementary file 2 [file bmjopen-2018-025881supp002.pdf]
