# Supplementary material for: Spatial distribution and temporal trends in social fragmentation in England, 2001−2011: a national study
Source: BMJ Open. 2019 Jan 24;9(1):e025881. doi: 10.1136/bmjopen-2018-025881 (PMC6347895; doi:10.1136/bmjopen-2018-025881)
Supplement: Author's manuscript [file bmjopen-2018-025881.draft_revisions.pdf]

BMJ Open

BMJ Open is committed to open peer review. As part of this commitment we make the peer review history of every article we publish publicly available.

When an article is published we post the peer reviewers' comments and the authors' responses online. We also post the versions of the paper that were used during peer review. These are the versions that the peer review comments apply to.

The versions of the paper that follow are the versions that were submitted during the peer review process. They are not the versions of record or the final published versions. They should not be cited or distributed as the published version of this manuscript.

BMJ Open is an open access journal and the full, final, typeset and author-corrected version of record of the manuscript is available on our site with no access controls, subscription charges or pay-per-view fees (http://bmjopen.bmj.com).

If you have any questions on BMJ Open's open peer review process please email editorial.bmjopen@bmj.com

Spatial distribution and temporal trends in social fragmentation in England, 2001 to 2011: a national study

Journal:	*BMJ Open*
Manuscript ID	bmjopen-2018-025881
Article Type:	Research
Date Submitted by the Author:	07-Aug-2018
Complete List of Authors:	Grigoroglou, Christos; University of Manchester, Centre for Primary Care<br>Munford, Luke; University of Manchester, Manchester Centre for Health Economics<br>Webb, Roger; University of Manchester, Centre for Suicide Prevention; Centre for Mental Health and Risk<br>Kapur, Navneet; University of Manchester, Centre for Suicide Prevention, Centre for Mental Health and Risk<br>Ashcroft, Darren; University of Manchester, Centre for Pharmacoepidemiology and Drug Safety<br>Kontopantelis, Evangelos; University of Manchester, Division of Population Health, Health Services Research and Primary Care
Keywords:	MENTAL HEALTH, EPIDEMIOLOGY, Organisation of health services < HEALTH SERVICES ADMINISTRATION & MANAGEMENT, SOCIAL MEDICINE

For peer review only

Spatial distribution and temporal trends in social fragmentation in England, 2001 to 2011: a national study

Christos Grigoroglou[1,8], Luke Munford[2,8], Roger T. Webb[3,6,8], Nav Kapur[4,6,7,8], Darren M. Ashcroft [5,6,8], Evangelos Kontopantelis[1,8]

[1] NIHR School for Primary Care Research, Centre for Primary Care, Division of Population Health, Health Services Research and Primary Care, University of Manchester, Manchester, UK
[2] Centre for Health Economics, Division of Population Health, Health Services Research and Primary Care, University of Manchester, Manchester, UK
[3] Centre for Mental Health and Safety, Division of Psychology & Mental Health, University of Manchester, Manchester, UK
[4] Centre for Suicide Prevention, Division of Psychology and Mental Health, University of Manchester, Manchester, UK
[5] Centre for Pharmacoepidemiology and Drug Safety, School of Health Sciences, Faculty of Biology, Medicine and Health, , University of Manchester, Manchester Academic Health Sciences Centre (MAHSC), Manchester, UK
[6] NIHR Greater Manchester Patient Safety Translational Research Centre, Manchester Academic Health Sciences Centre (MAHSC), Manchester, UK
[7] Greater Manchester Mental Health Trust, Manchester, UK
[8] Manchester Academic Health Sciences Centre [MAHSC], Manchester, UK

Correspondence to:

Christos Grigoroglou
E-mail: christos.grigoroglou@postgrad.manchester.ac.uk
Telephone number: 0161-2750743
5th Floor, Williamson Building, University of Manchester,
Manchester, UK, M13 9PL

Word count: 2,997
5 Figures

For peer review only

For peer review only - http://bmjopen.bmj.com/site/about/guidelines.xhtml

Objective: Social fragmentation is commonly examined in epidemiological studies of mental illness as high levels of social fragmentation are often found in areas with high prevalence of mental illness. In this study we examine spatial and temporal patterns of the index of social fragmentation and its subdomains in England over time.

Setting: Data for social fragmentation and its subdomains were analysed over the decennial Censuses (2001–2011) at a small-area geographical level (mean of 1500 people). Degrees of social fragmentation and temporal changes were spatially visualised for the whole of England and its 10 administrative regions. Spatial clustering was quantified using Moran's I; changes in correlations over time were quantified using Spearman's ranking correlation.

Results: Between 2001 and 2011 we observed a strong persistence for social fragmentation nationally (Spearman's r=0.94). At the regional level, modest changes were observed over time, but marked increases were observed for two of the four social fragmentation subdomains, namely single people and those in private renting. Results supported our hypothesis of increasing spatial clustering over time. Moderate regional variability was observed in social fragmentation, its subdomains and their clustering over time.

Conclusion: Patterns of social fragmentation and its subdomains persisted in England which seem to be driven by the large increases in single people and those in private renting. Policies to improve social cohesion may have an impact the lives of persons who experience mental illness. The spatial aspect of social fragmentation can inform the targeting of health and social care interventions, particularly in areas with strong social fragmentation clustering.

Keywords: social fragmentation, mental health, clustering, inequalities, spatial autocorrelation

For peer review only - http://bmjopen.bmj.com/site/about/guidelines.xhtml

Strengths and Limitations of the study
➢ This study utilises longitudinal data for the whole of England and is the first to describe spatial and temporal patterns of social fragmentation.
➢ Spatial aspects of social fragmentation can inform organisation of mental health services and social interventions that aim to enhance social support, particularly in areas where social fragmentation is high and is spatially clustered
➢ Over the last two decennial Censuses (2001 and 2011), the index of social fragmentation in England strongly persisted at a small-area geographical level while its clustering and its subdomains increased over time.
➢ Over time social fragmentation remained relatively stable, but we found marked increases in the percentage of single people and the percentage of those living in private rented accommodation.
➢ Even though census data are considered of high quality, people's perception of social fragmentation may not be fully captured in the census while demographic factors may capture social fragmentation in some areas but not in others.

For peer review only

For peer review only - http://bmjopen.bmj.com/site/about/guidelines.xhtml

INTRODUCTION

The impact of local area characteristics, such as material deprivation, on health is well established. [1 2] However, similar to socioeconomic position, the social networks and the amount of social support in a neighbourhood may also partially determine an individual's health. [3-6] In this context, an area-level characteristic that has been the focus of much investigation in neighbourhood health research is social fragmentation. The index of social fragmentation measures lack of social cohesion, and it is used to define areas with a breakdown or absence of social capital, and was originally created to measure the non-economic deprivation aspects of areas. [7]

Social fragmentation is distinct conceptually from deprivation, since fragmentation is a feature of household composition and demographic structure and is not intrinsically linked to socioeconomic position.[8] However, the two measures tend to be positively correlated as they are both concentrated in large towns and cities. [8 9] Thus, many urban localities are both deprived and socially fragmented, although the two phenomena do not always co-exist. Several studies suggests that fragmented communities provide less stable social institutions and social bonds, although maintaining stable institutions and bonds within the communities can contribute substantially to the creation of shared identities, persistence of social relations, promotion of healthy behaviours and good physical and mental health for the population. [10-13]

Social fragmentation has received less attention from researchers than deprivation, despite the fact that it can aid the understanding of mechanisms via which the wider social environment influences mental health. [14] Research on adults has shown that social fragmentation is more closely related to mental health than physical health outcomes, independent of material deprivation and individual-level risk factors. [7 15] In the context of mental health, social fragmentation has been linked with suicide and non-fatal self-harm, [7 16-18] mental disorders, [4 11 19] psychiatric health service use, [20] first admission for psychosis, [19-21] psychological distress, [3 11] and schizophrenia. [14 22] Nonetheless, physical illness outcomes do not appear to be as clearly linked to social fragmentation. [17 23 24]

In the UK, an index of social fragmentation was developed to measure neighbourhood-level conditions which affect lack of opportunities for social integration. [7] The index was originally based on indicators of population turnover, percentage of married people, percentage of the population in private renting and percentage of one person households using data from the UK Census. The rationale for the selection of the underlying variables used for the calculation of the index was that several studies, both at ecological and individual level, have found raised mental illness prevalence in highly urbanised areas with high population turnover, many non-family households, and reduced social cohesion. [25] However, social trends towards increasing cohabitation, [10] a phenomenon that occurs most commonly in

For peer review only - http://bmjopen.bmj.com/site/about/guidelines.xhtml

urban areas [23], suggest the use of proportion of single, widowed and divorced people instead of married people in the calculation of the index.

Social fragmentation in a population is likely to have profound effects on its health and wellbeing, and yet it is incompletely examined and reported in the existing literature, and a number of important research questions remain unanswered. First, how strongly clustered is social fragmentation? Second, how strongly does social fragmentation cluster and change longitudinally at regional level? Third, does social fragmentation persist over time? Answering all three questions is important to inform the organisation of healthcare services and to target social and healthcare interventions, especially for mental health for which social fragmentation appears to be a salient risk factor. In this study, we longitudinally and spatially describe the properties of social fragmentation in England and within each of the 10 administrative English regions and make comparisons across them. Finally, we quantify the persistence of social fragmentation over the study period.

METHODS

Data sources

To derive the index of social fragmentation we accessed decennial Census data from the Nomis website [26] on: (1) One person households, % of all Census households; (2) single people (including widowed and divorced people), % all Census population; (3) Population turnover (percentage of the population that moved from other area or within the country), % Census population (4) Households in private renting, % of all Census households. Our analysis was conducted at a small-area geographical level, namely the Lower Layer Super Output Area (LSOA). LSOAs are administrative units of geography with a mean population of 1,500 people. All data were available at the LSOA level except for the population turnover data which were available at the output area level (OA). OAs provide the finest spatial scale at which the Census is enumerated. We assigned the OA level data to the LSOA level using lookup tables provided by the Office for National Statistics (ONS).

The social fragmentation index for each LSOA was calculated by adding the unweighted z scores for each of the four characteristics into a composite score. We used two time points for the index of social fragmentation, i.e. 2001 and 2011 over the two decennial Censuses. Across LSOAs the scores for the index ranged from -6.42 to 27.57 for 2001 and from -7.09 to 25.79 for 2011, with the upper boundaries indicating the most socially fragmented areas and the lower boundaries the most socially cohesive areas. Since the data used to derive the index in 2001 were reported using 2001 LSOAs, we used a weighted means algorithm to assign them to 2011 LSOAs using mid-year population estimates that were obtained from ONS. [27]

For peer review only - http://bmjopen.bmj.com/site/about/guidelines.xhtml

For peer review only

Analyses

Our outcome of interest was the index of social fragmentation and its four subdomains measured separately at the 2001 and 2011 national Censuses. We visualised temporal changes in social fragmentation and its subdomains with the use of spatial maps for all of England and each of the 10 English regions (North East, North West, Yorkshire & the Humber, East Midlands, West Midlands, East of England, London, South East Coast, South Central and South West). To assess whether levels of spatial autocorrelation for social fragmentation changed over time we compared the values of Moran's I [28] in the two time points for England and each English region to allow within-England comparisons. The measure can identify the presence or absence of spatial clusters while accounting for the multi-dimensional and multi-directional nature of spatial autocorrelation. Under a random spatial pattern a higher than expected value of Moran's I for social fragmentation would indicate that areas with high levels of social fragmentation are clustered and also that LSOAs with high social fragmentation are bordered with LSOAs with similarly high levels of social fragmentation. We also quantified the persistency of social fragmentation over the two time points by calculating Spearman's correlation between 2001 and 2011. To visualise and compare temporal changes in the mean social fragmentation levels across the 10 English regions, we plotted population-weighted box plots for the main outcome and its subdomains. Finally, we used population-weighted box plots to visualise and compare temporal changes in the social fragmentation levels between the 10 English regions. Analyses were performed with Stata v.14.1 and R v.3.3.1. Due to the size of the dataset, effectively the whole of England, statistical significance is irrelevant; observed associations of minimal strength would be statistically significant, and thus we focussed on effect sizes wherever possible.

RESULTS

We found strong correlation for social fragmentation at the two time points (rho=0.93), using Spearman's ranking correlation to account for the relative nature of the measure. We plotted spatial maps of social fragmentation for the whole of England and for each region for the two time points analysed. Maps of social fragmentation in 2001 and 2011 and their respective changes for the whole of England and London are presented in Figure 1 and Figure 2 respectively, whilst social fragmentation and its subdomains maps over time for each region are presented in supplementary Appendices 2-6. Temporal changes in social fragmentation for England and for all English regions are presented in Figure A in the online appendix. We identified a pattern of stability with modest increases over time for regions in the North, and small decreases in social fragmentation for regions in the South. However,

For peer review only - http://bmjopen.bmj.com/site/about/guidelines.xhtml

For peer review only

temporal changes of English regions for two of the four social fragmentation subdomains were much larger and they are presented in figures 3A and 3B.

Large changes in absolute levels of single person households and private renting were found in all regions. More specifically, in 2001 the average number of single persons of the total population was 38.4% while in 2011 the figure was 52.6% indicating a 31% increase. Similarly, in 2001 the average number of people in private renting was 8.5% of all English households while in 2011 this figure increased to 16.2% indicating a 62% increase. The largest increases in numbers of single people were found in the North East, North West and West Midlands while London had the highest levels of single people. Moreover, the largest increases for private renting were found in the North East, West Midlands and East Midlands while London had again the largest numbers of people living in private renting. For the other two social fragmentation subdomains, we found that the average number of one person households both nationally and regionally remained relatively stable. We also observed a modest decrease, both nationally and regionally (with the exception of London), in our measure of population turnover which reflects the levels of migration inside and outside areas. Finally, we observed great variability in levels of social fragmentation and its subdomains within regions at both time points. High levels of social fragmentation were concentrated in London, the North West and the South West. East England had the lowest levels of social fragmentation followed by West Midlands. There was a clear distinction between rural and urban areas, with the latter having far greater levels of social fragmentation.

Across the whole of England, Moran's I for social fragmentation was very low at 0.0449 in 2001 (95% CI 0.0447 to 0.0451) but by 2011 it had increased to 0.0794 (95% CI 0.0792 to 0.0797), indicating a small increase in spatial autocorrelation and clustering (Figure 4). We also observed marked regional variability in social fragmentation spatial autocorrelation levels over time (Figure 4). In both time points, the West Midlands had the lowest spatial autocorrelation in social fragmentation, followed by the East of England, while the South East and South West had the highest levels of spatial autocorrelation at both time points. In contrast to increases in spatial autocorrelation across the country between 2001 and 2011, the South East, North East and Yorkshire & the Humber had decreasing levels of spatial autocorrelation over the two time points.

Spatial clustering also appears to have increased over time for all fragmentation subdomains nationally, although its levels remained low (Figures A1 and A2 in supplementary Appendix). For one person households we found very low regional clustering and small regional variation. For single persons and private renting regional variation in clustering was moderate and their levels were also low with the exception of the South East and South West. Finally, for population turnover we observed the greatest increases of

For peer review only - http://bmjopen.bmj.com/site/about/guidelines.xhtml

spatial autocorrelation both nationally and regionally but levels of regional clustering were generally low with the exception of South West whilst regional variability was moderate.

DISCUSSION

Between 2001 and 2011, we found evidence of strong persistence of social fragmentation in England at small-area level. Regional changes over time were modest for social fragmentation, which is not surprising considering the large population denominators and the relative nature of the measure. Similarly, we observed modest increases over time for one person households and population turnover. However, changes over time for single persons and private renting were much greater, with substantial increases in both subdomains across regions, and these specific influences appear to have largely driven the heightened levels of social fragmentation overall. Furthermore, our findings provide evidence of increasing clustering for social fragmentation and its subdomains which, in conjunction with the regional variation observed between the two time points, indicates that areas in the South of England (including London) appear much more clustered than the North. Finally, we observed increased clustering for all subdomains of social fragmentation over the two time points.

Strengths and limitations of the study

The main strength of this study lies in its large longitudinal nature with data for the whole of England (53 million people in 2011), which we utilised to investigate spatial and longitudinal patterns of social fragmentation. To our knowledge, it is the first study of its kind to provide an insight into the temporal persistence of social fragmentation and its clustering.

However, some limitations exist. First, the extent to which the index captures social fragmentation may vary across the country as the demographic factors that we used may not in themselves be valid indicators of social fragmentation, or they may adequately measure social fragmentation in some areas but not in others. [21] However, the measure has been reported widely in the literature and on this basis social fragmentation has been identified as an important predictor of mental health outcomes such as suicide. Second, Census questions do not usually directly elicit the presence or quality of neighbourhood social ties or institutions, or people's relationship to their neighbourhood. Therefore, we might have failed to capture people's perception of social fragmentation through the Census data. Nevertheless, we used a measure capturing compositional aspects of neighbourhood which appear to be related to the capacity of a neighbourhood to act collectively, and is also important for the construction and maintenance of social ties and institutions. Third, whilst Census data is recognised to be of a high standard, some unobserved heterogeneity persists. [29] For example, LSOAs are defined from administrative

For peer review only - http://bmjopen.bmj.com/site/about/guidelines.xhtml

information and this may disguise local effects as contextual characteristics within administrative neighbourhoods are not homogeneous across LSOAs. [30] An alternative approach would require the use of non-administrative data to define levels, but the Census is currently the only source from which the data on the fragmentation subdomains is available. Finally, there were boundary changes following the 2011 Census that may have affected our findings, [31] but only 2.5% of LSOAs were affected and we developed an algorithm to make reasonable population weighted-based estimates for these localities, depending on whether they merged, split or some other change occurred.

Interpretation of findings

Our results suggest that urbanisation drives the high persistence of social fragmentation in England as we found that levels of social fragmentation and its subdomains were consistently higher in urban areas. This may be because urban areas are chosen by diverse social and cultural groups and living in these areas may lead to isolated spaces, lifestyles and problems of social integration. Moreover, even though the overall index of social fragmentation changed a little, the subdomains that drive the persistency of social fragmentation appear to be single people and households in private renting, a characteristic of urban environments, [32] as these were the subdomains with the largest increases (Figures 3A and 3B).

In absolute terms, private renting was considerably increased in all regions and most notably London; a finding that pertains to unaffordable housing for first-time home buyers and less availability of social housing as a result of the 2008 financial crisis.[33] Similarly, all regions had higher levels of single people in 2011, indicating that numbers of young professionals, students and divorces have increased substantially over the decade. For the other two subdomains we did not observe any marked changes across regions, although levels of population turnover appeared to have decreased between the decennial Censuses. The increases we observed in the proportion of single people and those in private renting warrant implications for the prevalence of mental illness especially in urban centres. The private house market is characterised by poor housing conditions as opposed to social houses or owner occupation and this can have detrimental effects on mental health. [34] Furthermore, it is suggested that married individuals may have better mental health outcomes than single individuals. [35] We believe that these changes are certainly of concern as they may highlight the need for more effective social interventions to target socially fragmented areas in England.

Spatial clustering of neighbourhood social fragmentation appears to have increased over time in England while we observed moderate regional variations in levels of clustering despite the small changes in levels of social fragmentation across regions. Increased levels of spatial clustering for social fragmentation across and within regions can have implications

For peer review only - http://bmjopen.bmj.com/site/about/guidelines.xhtml

for the planning and organisation of health services, as this would imply needs that are not uniformly distributed across a region with the presence of hot spots of high levels of mental illness prevalence, especially in urban areas. To address such spatial health inequalities, services may need to be redesigned locally [36] while infrastructure and spending for mental health needs to be weighted towards social fragmentation hot spots rather than uniformly distributed.

Conclusion

Social fragmentation is increasingly considered a determinant of mental illness as more epidemiological research emerges that investigates contextual effects on health. To improve mental health in the population, policymakers need to address both relational and structural issues, perhaps by investing in reorganisation of mental healthcare. Solving the problems of cohesion and integration needs to be seen as a collective responsibility across central and local government and all the local agencies. Policies needs to address social and educational inequalities by strengthening the economic well-being of areas by lowering local unemployment rates, encouraging populations to bring social support in the communities and encouraging education of the population, which are key strategies for healthy communities. Communities need to work towards tightening social networks and social bonds to help with mental illness prevalence and suicide prevention, for example by promoting participation in charity, voluntary or community groups and civic engagement. The spatial aspect of social fragmentation is often overlooked, but it can provide vital information for the effective organisation of health services and targeting of health or social interventions. The nature of fragmentation and the mechanisms underlying its association with mental illness are of increasing interest as the population gets older and the health care costs associated with mental illness escalate in industrialised countries.

For peer review only

For peer review only - http://bmjopen.bmj.com/site/about/guidelines.xhtml

Acknowledgments

We would like to thank the Office of National Statistics for the wealth of information they have collected and systematically organised, which made this study possible.

Contributors

EK, CG, DA and LM designed the study. CG extracted the data from all sources, performed the analyses and drafted the manuscript. EK, RG, DA, LM, NK critically revised the manuscript. CG is the guarantor of this work and, as such, had full access to all the data in the study and takes responsibility for the integrity of the data and the accuracy of the data analysis.

Funding

This study is funded by the National Institute for Health Research School for Primary Care Research (NIHR SPCR), through CG's PhD. This report is independent research by the National Institute for Health Research. LM acknowledges financial support from the MRC Skills Development Fellowship (MR/N015126/1). The views expressed in this publication are those of the authors and necessarily those of the NHS, the National Institute for Health Research, the Department of Health, or the MRC.

Competing interests

None declared.

Provenance and peer review

Not commissioned; externally peer reviewed

Data sharing agreement

The data used in this study are freely available and the authors are happy to share in an organised and cleaned final dataset.

Ethical approval and Patient and Public Involvement

Not applicable. The study uses freely reported data aggregates at low geography levels. No patients or members of the public were involved in the study.

Open access

This is an Open Access article distributed in accordance with the terms of the Creative Commons Attribution (CC BY 4.0) license, which permits others to distribute, remix, adapt and build upon this work, for commercial use, provided the original work is properly cited. See: http://creativecommons.org/licenses/by/4.0/ © Article author(s) (or their employer(s) unless otherwise stated in the text of the article) 2018. All rights reserved. No commercial use is permitted unless otherwise expressly granted.

For peer review only - http://bmjopen.bmj.com/site/about/guidelines.xhtml

Figure 1 Changes in overall social fragmentation for England, 2001 – 2011

Figure 2 Changes in overall social fragmentation for London, 2001 – 2011

Figure 3A: Percentage of single people (top) and one person households (bottom) for English regions, over time*

* Red line indicates mean for England in 2011

Figure 3B: Percentage of private renting (top) and population turnover (bottom) for English regions, over time

* Red line indicates mean for England in 2011

Figure 4 Spatial Autocorrelation with Moran's I for Social Fragmentation by region, over time

For peer review only

References

1. Pickett KE, Pearl M. Multilevel analyses of neighbourhood socioeconomic context and health outcomes: a critical review. *J Epidemiol Community Health* 2001;55(2):111-22. doi: 10.1136/jech.55.2.111
2. Stafford M, Marmot M. Neighbourhood deprivation and health: does it affect us all equally? *Int J Epidemiol* 2003;32(3):357-66. doi: 10.1093/ije/dyg084
3. Fagg J, Curtis S, Stansfeld SA, et al. Area social fragmentation, social support for individuals and psychosocial health in young adults: evidence from a national survey in England. *Soc Sci Med* 2008;66(2):242-54. doi: 10.1016/j.socscimed.2007.07.032
4. Stafford M, De Silva M, Stansfeld S, et al. Neighbourhood social capital and common mental disorder: testing the link in a general population sample. *Health Place* 2008;14(3):394-405. doi: 10.1016/j.healthplace.2007.08.006
5. Subramanian SV, Lochner KA, Kawachi I. Neighborhood differences in social capital: a compositional artifact or a contextual construct? *Health Place* 2003;9(1):33-44. doi: 10.1016/S1353-8292(02)00028-X
6. Veenstra G, Luginaah I, Wakefield S, et al. Who you know, where you live: social capital, neighbourhood and health. *Soc Sci Med* 2005;60(12):2799-818. doi: 10.1016/j.socscimed.2004.11.013
7. Congdon P. Suicide and parasuicide in London: A small-area study. *Urban Stud* 1996;33(1):137-58. doi: 10.1080/00420989650012194
8. Congdon P. Commentary: Contextual effects: index construction and technique. *Int J Epidemiol* 2004;33(4):741-2. doi: 10.1093/ije/dyh173
9. Understanding patterns of deprivation. *Reg Trends* 2009;41(1):93-114. doi: 10.1057/rt.2009.7
10. Whitley E, Gunnell D, Dorling D, et al. Ecological study of social fragmentation, poverty, and suicide. *BMJ* 1999;319(7216):1034-7. doi: 10.1136/bmj.319.7216.0d
11. Fagg J, Curtis S, Stansfeld S, et al. Psychological distress among adolescents, and its relationship to individual, family and area characteristics in East London. *Soc Sci Med* 2006;63(3):636-48. doi: 10.1016/j.socscimed.2006.02.012
12. Fone D, Dunstan F, Lloyd K, et al. Does social cohesion modify the association between area income deprivation and mental health? A multilevel analysis. *Int J Epidemiol* 2007;36(2):338-45. doi: 10.1093/ije/dym004
13. Evans J, Middleton N, Gunnell D. Social fragmentation, severe mental illness and suicide. *Soc Psychiatr Psychiatric Epidemiol* 2004;39(3):165-70. doi: 10.1007/s00127-004-0733-9
14. Allardyce J, Boydell J. Review: the wider social environment and schizophrenia. *Schizophr Bull* 2006;32(4):592-8. doi: 10.1093/schbul/sbl008
15. Ellen IG, Mijanovich T, Dillman K-N. Neighborhood Effects on Health: Exploring the Links and Assessing the Evidence. *J Urban Aff* 2001;23(3-4):391-408. doi: 10.1111/0735-2166.00096
16. Collings S, Ivory V, Blakely T, et al. Are neighbourhood social fragmentation and suicide associated in New Zealand? A national multilevel cohort study. *J Epidemiol Community Health* 2009;63(12):1035-42. doi: 10.1136/jech.2009.090985
17. Smith GD, Whitley E, Dorling D, et al. Area based measures of social and economic circumstances: cause specific mortality patterns depend on the choice of index. *J Epidemiol Community Health* 2001;55(2):149-50. doi: 10.1136/jech.55.2.149
18. Corcoran P, Arensman E, Perry IJ. The area-level association between hospital-treated deliberate self-harm, deprivation and social fragmentation in Ireland. *J Epidemiol Community Health* 2007;61(12):1050-55. doi: 10.1136/jech.2006.055855

For peer review only

19. Evans E, Hawton K, Rodham K. Factors associated with suicidal phenomena in adolescents: a systematic review of population-based studies. *Clin Psychol Rev* 2004;24(8):957-79. doi: 10.1016/j.cpr.2004.04.005
20. Curtis S, Copeland A, Fagg J, et al. The ecological relationship between deprivation, social isolation and rates of hospital admission for acute psychiatric care: a comparison of London and New York City. *Health Place* 2006;12(1):19-37. doi: 10.1016/j.healthplace.2004.07.002
21. Allardyce J, Gilmour H, Atkinson J, et al. Social fragmentation, deprivation and urbanicity: relation to first-admission rates for psychoses. *Br J Psychiatry* 2005;187:401-06. doi: 10.1192/bjp.187.5.401
22. van Os J, Driessen G, Gunther N, et al. Neighbourhood variation in incidence of schizophrenia. Evidence for person-environment interaction. *Br J Psychiatry* 2000;176:243-8. doi: 10.1192/bjp.176.3.243
23. Stjarne MK, Ponce de Leon A, Hallqvist J. Contextual effects of social fragmentation and material deprivation on risk of myocardial infarction--results from the Stockholm Heart Epidemiology Program (SHEEP). *Int J Epidemiol* 2004;33(4):732-41. doi: 10.1093/ije/dyh087
24. Stafford M, Gimeno D, Marmot MG. Neighbourhood characteristics and trajectories of health functioning: a multilevel prospective analysis. *Eur J Public Health* 2008;18(6):604-10. doi: 10.1093/eurpub/ckn091
25. Jarvis GK, Ferrence RG, Whitehead PC, et al. The ecology of self-injury: a multivariate approach. *Suicide Life Threat Behav* 1982;12(2):90-102. doi: 10.1111/j.1943-278X.1982.tb00916.x
26. Office for National Statistics. Nomis official labour market statistics. 2013
27. Office for National Statistics. Super Output Area mid-year population estimates for England and Wales, Mid-2011. 2016.
28. Moran PAP. Notes on Continuous Stochastic Phenomena. *Biometrika* 1950;37(1-2):17-23. doi: 10.2307/2332142
29. Fewell Z, Smith GD, Sterne JAC. The impact of residual and unmeasured confounding in epidemiologic studies: A simulation study. *Am J Epidemiol* 2007;166(6):646-55. doi: 10.1093/aje/kwm165
30. Rehkopf DH, Buka SL. The association between suicide and the socio-economic characteristics of geographical areas: a systematic review. *Psychol Med* 2006;36(2):145-57. doi: 10.1017/S003329170500588x
31. Norman P, Rees P, Boyle P. Achieving data compatibility over space and time: creating consistent geographical zones. *Popul Space Place* 2003;9(5):365-86. doi: 10.1002/ijpg.294
32. Smith CW. 2011 Census Analysis: How Have Living Arrangements and Marital Status in England and Wales Changed Since 2001. *Office for National Statistics* 2011
33. Kemp PA. Private Renting After the Global Financial Crisis. *Housing Stud* 2015;30(4):601-20. doi: 10.1080/02673037.2015.1027671
34. Barnes M, Cullinane, C., Scott, S. & Silvester, S. . People living in bad housing: Numbers and health impacts. *UK: Shelter* 2013
35. Simon RW. Revisiting the relationships among gender, marital status, and mental health. *Am J Sociol* 2002;107(4):1065-96. doi: 10.1086/339225
36. Lorant V, Thomas I, Deliege D, et al. Deprivation and mortality: the implications of spatial autocorrelation for health resources allocation. *Soc Sci Med* 2001;53(12):1711-9. doi: 10.1016/S0277-9536(00)00456-1

For peer review only

For peer review only - http://bmjopen.bmj.com/site/about/guidelines.xhtml

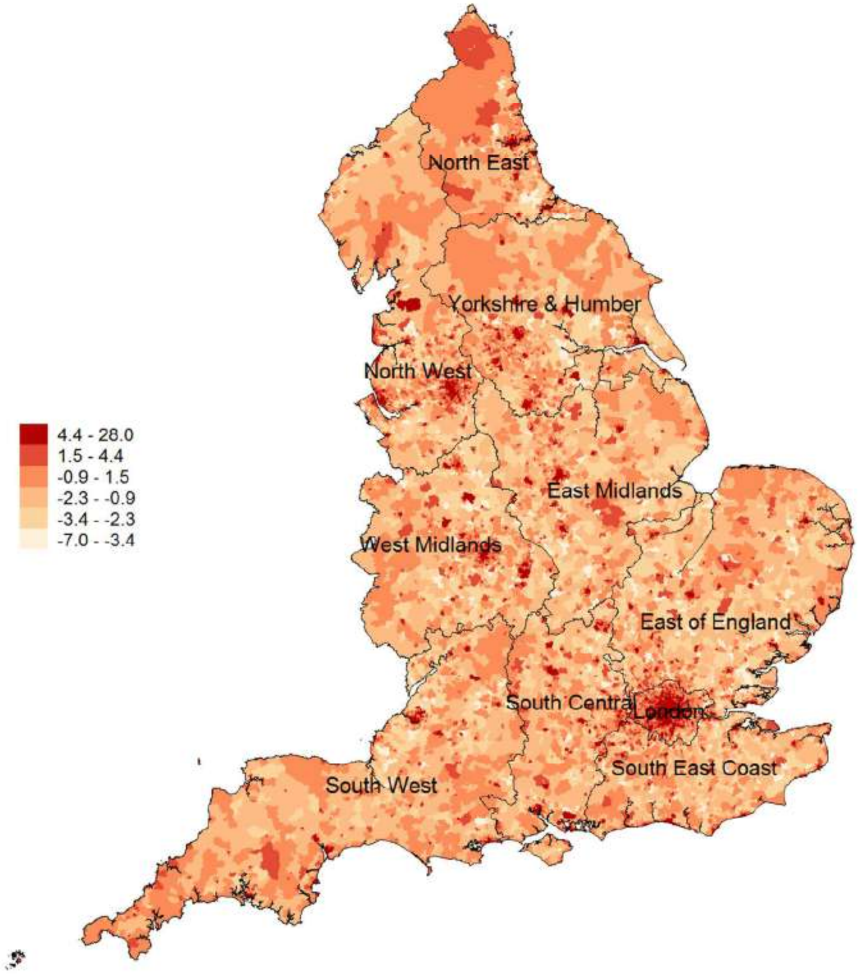
4.4 - 28.0
1.5 - 4.4
-0.9 - 1.5
-2.3 - -0.9
-3.4 - -2.3
-7.0 - -3.4
North East
Yorkshire & Humber
North West
East Midlands
West Midlands
East of England
South Central
London
South East Coast
South West

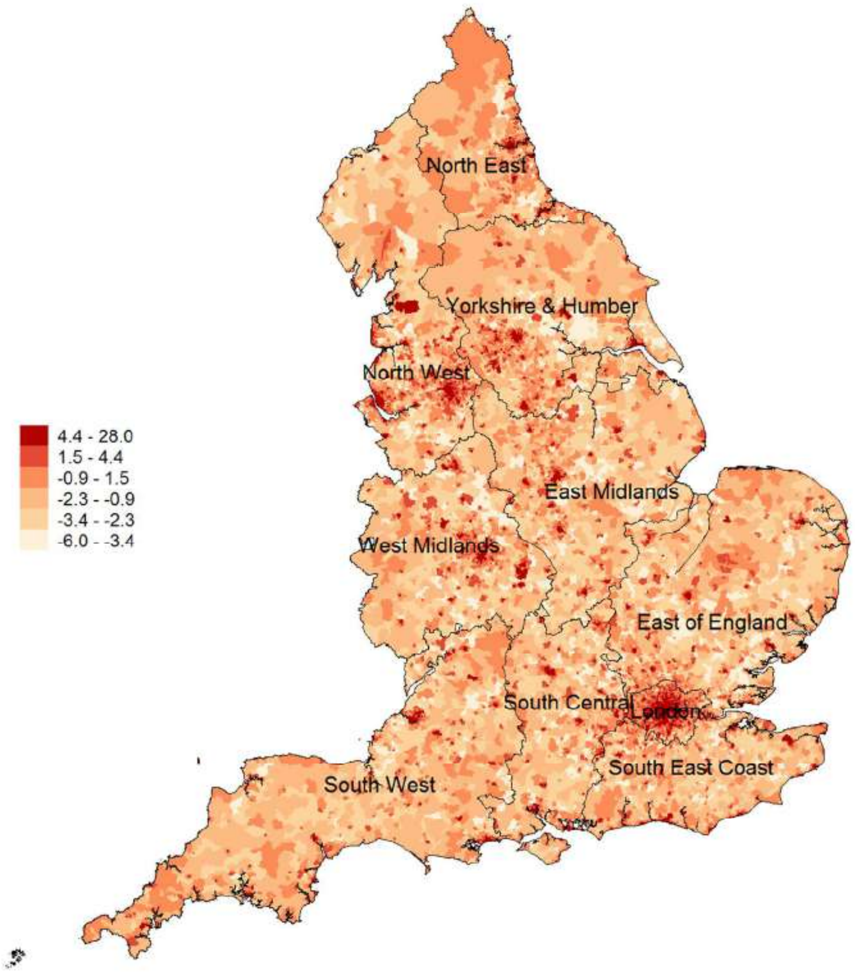
4.4 - 28.0
1.5 - 4.4
-0.9 - 1.5
-2.3 - -0.9
-3.4 - -2.3
-6.0 - -3.4
North East
Yorkshire & Humber
North West
East Midlands
West Midlands
East of England
South Central
London
South East Coast
South West

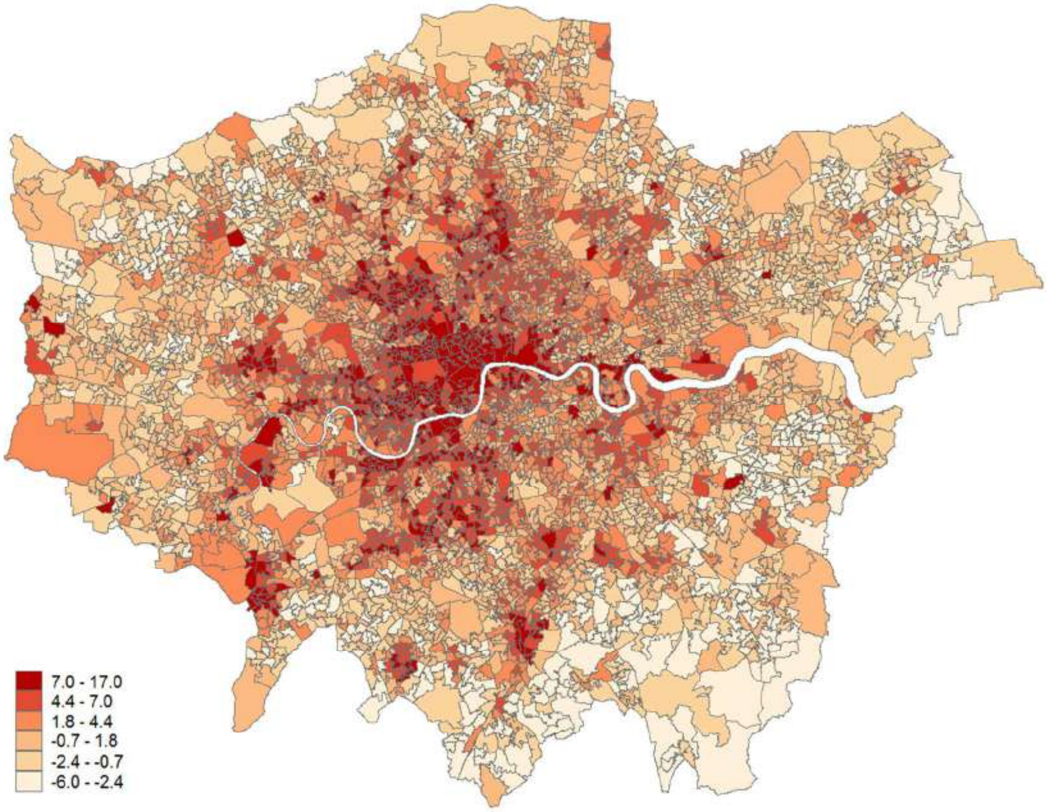
7.0 - 17.0
4.4 - 7.0
1.8 - 4.4
-0.7 - 1.8
-2.4 - -0.7
-6.0 - -2.4

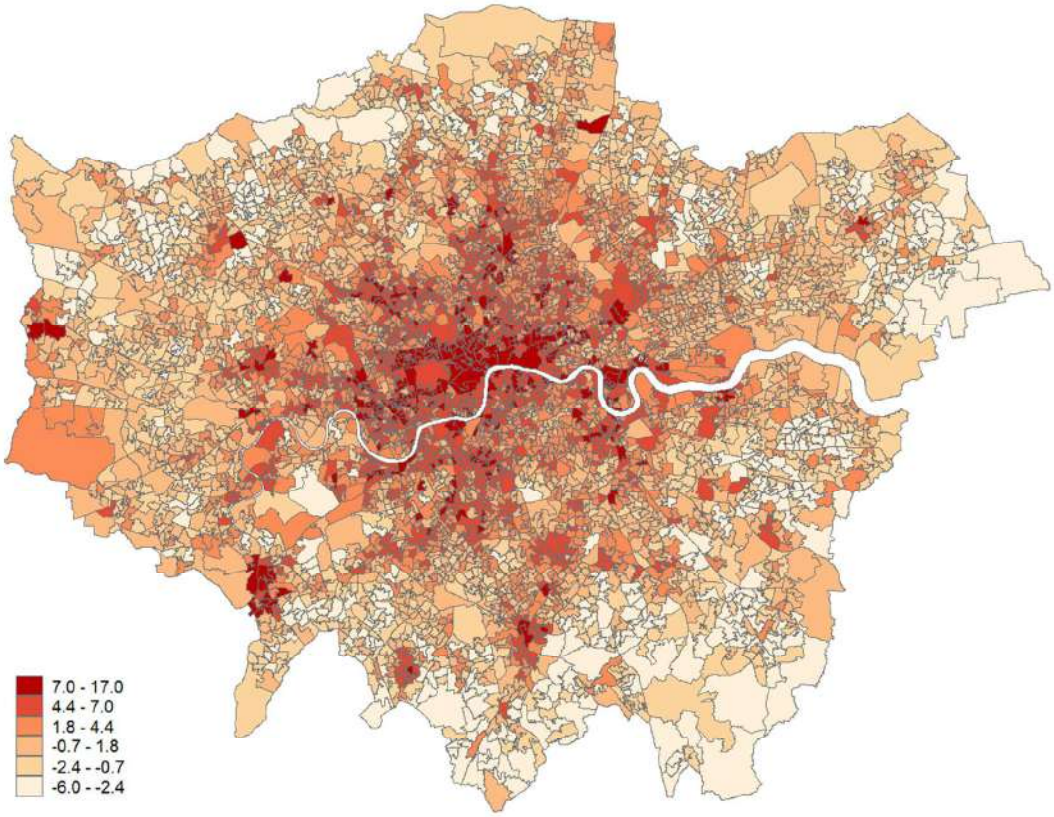
7.0 - 17.0
4.4 - 7.0
1.8 - 4.4
-0.7 - 1.8
-2.4 - -0.7
-6.0 - -2.4

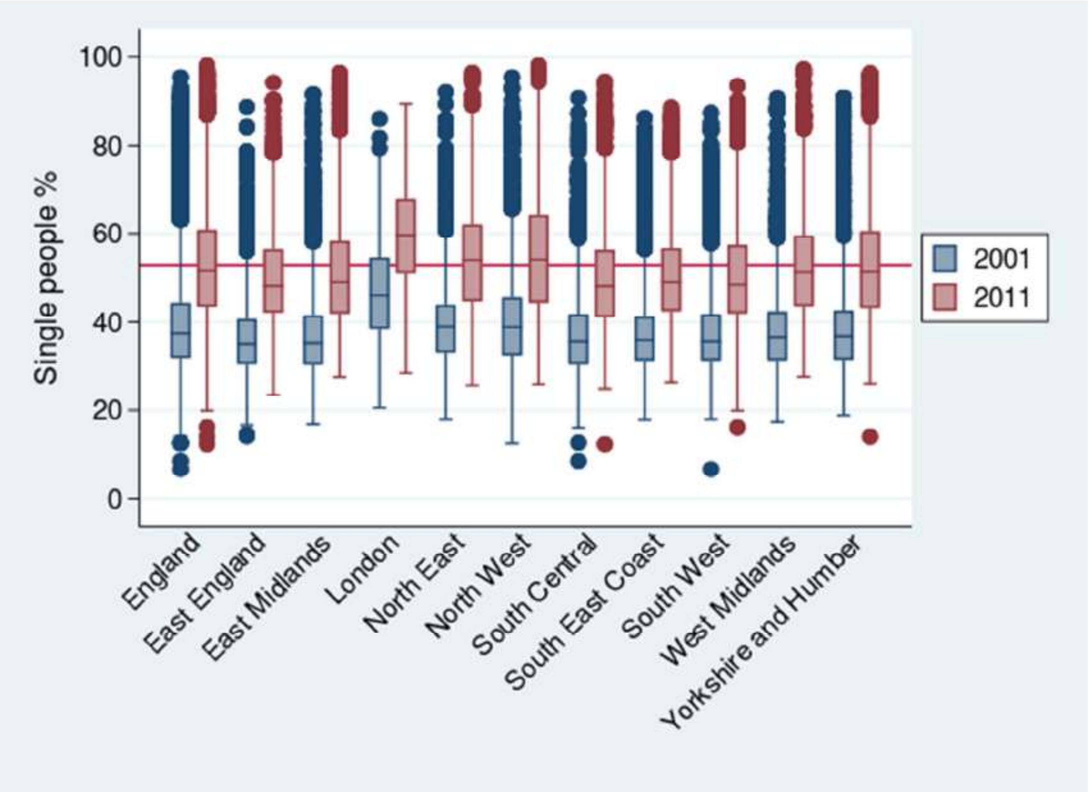
Single people %
100
80
60
40
20
0
2001
2011
England
East England
East Midlands
London
North East
North West
South Central
South East Coast
South West
West Midlands
Yorkshire and Humber

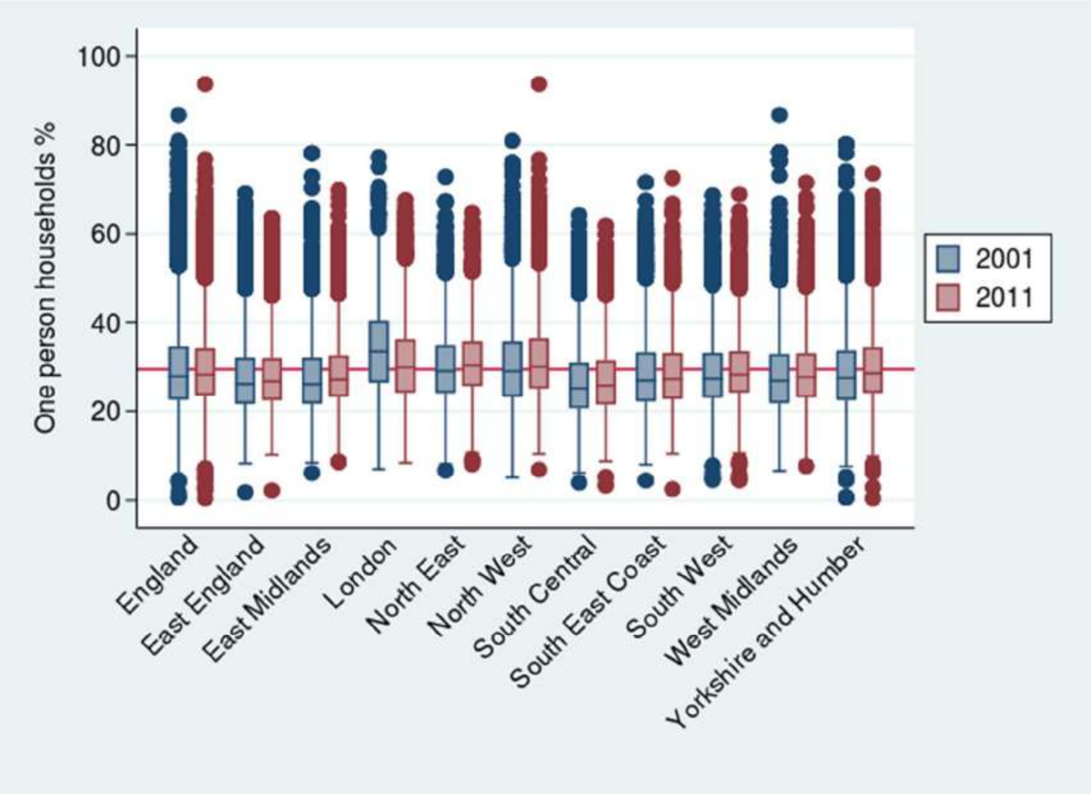
One person households %
100
80
60
40
20
0
2001
2011
England
East England
East Midlands
London
North East
North West
South Central
South East Coast
South West
West Midlands
Yorkshire and Humber

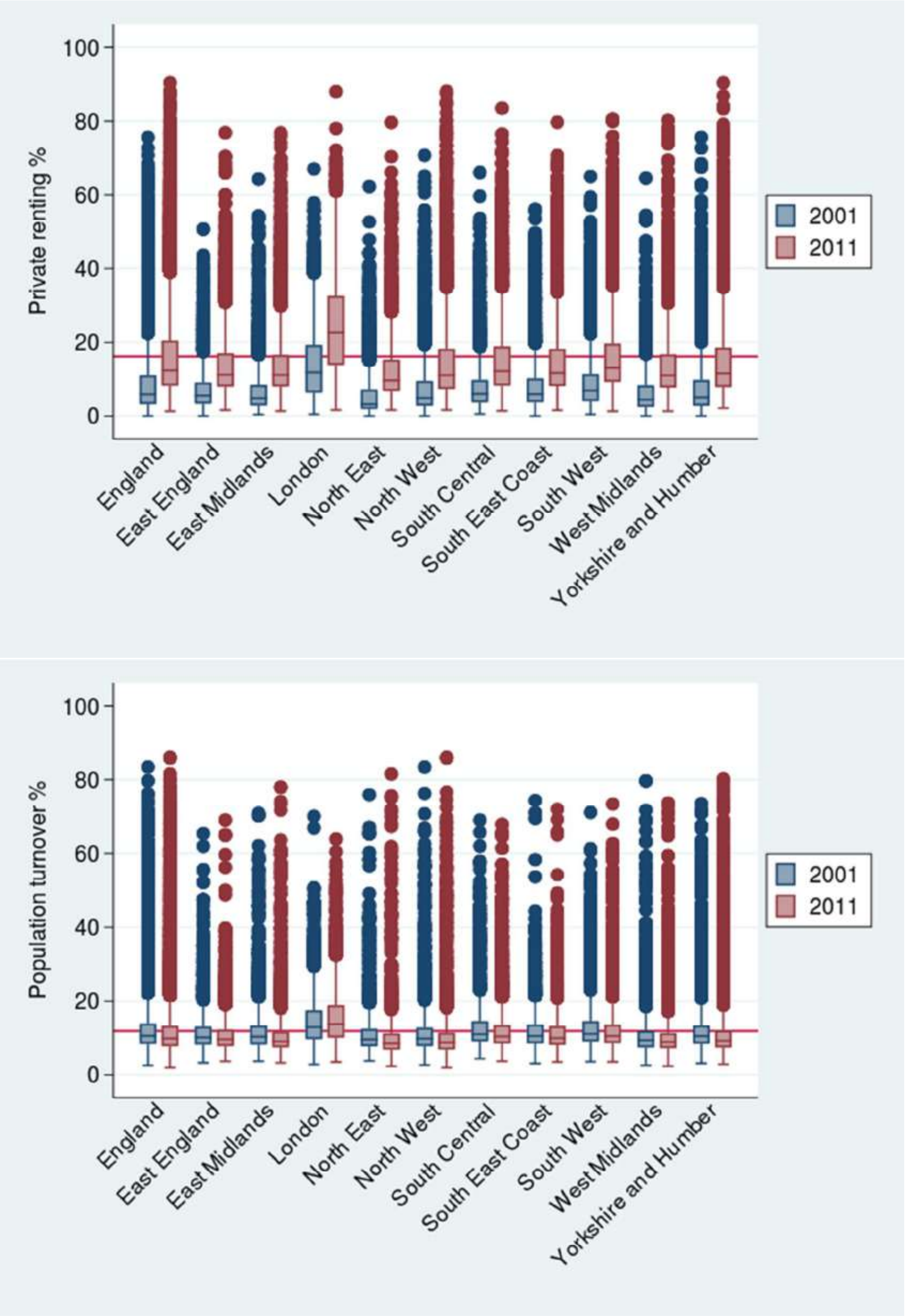
Private renting %
100
80
60
40
20
0
England
East England
East Midlands
London
North East
North West
South Central
South East Coast
South West
West Midlands
Yorkshire and Humber
2001
2011
Population turnover %
100
80
60
40
20
0
England
East England
East Midlands
London
North East
North West
South Central
South East Coast
South West
West Midlands
Yorkshire and Humber
2001
2011

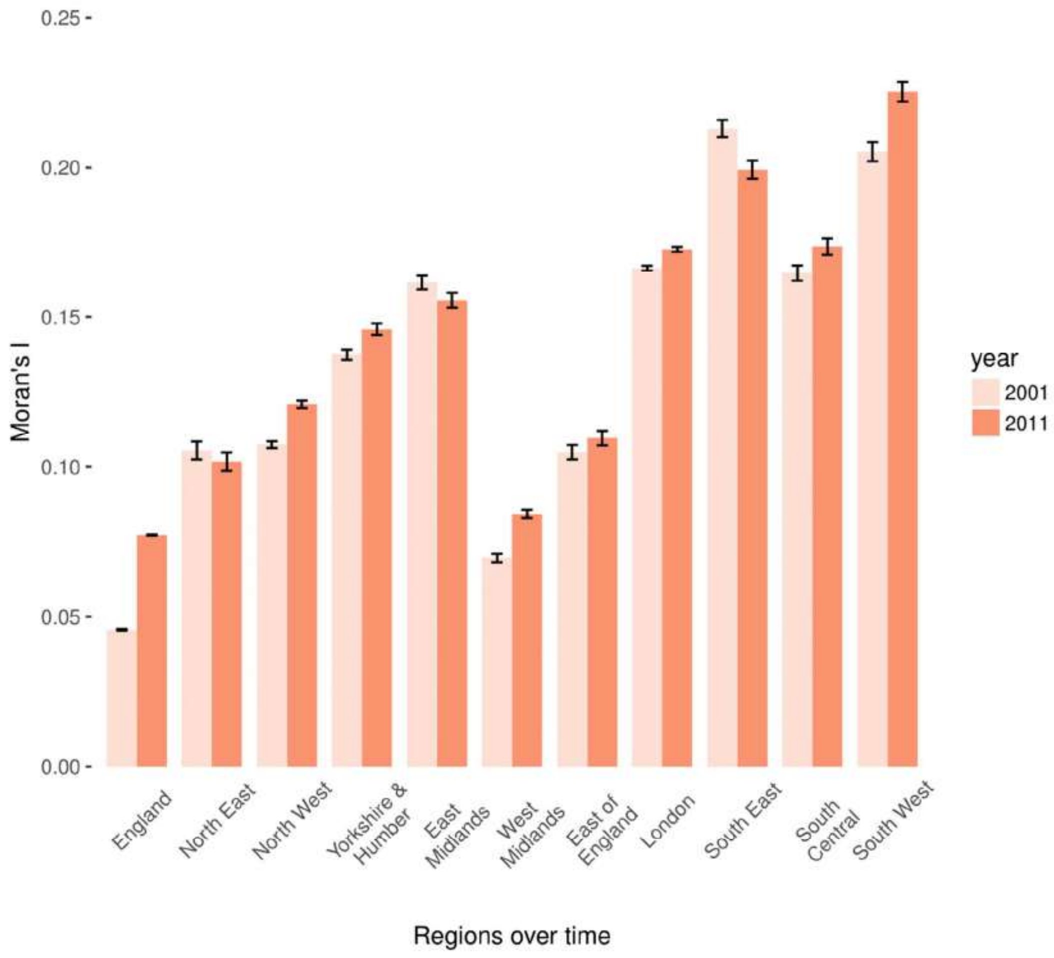
0.25
0.20
0.15
0.10
0.05
0.00
Moran's I
year
2001
2011
England
North East
North West
Yorkshire & Humber
East Midlands
West Midlands
East of England
London
South East
South Central
South West
Regions over time

Supplementary Appendix 1

Spatial distribution and temporal trends in social fragmentation in England, 2001 to 2011: a national study

For peer review only

For peer review only - http://bmjopen.bmj.com/site/about/guidelines.xhtml

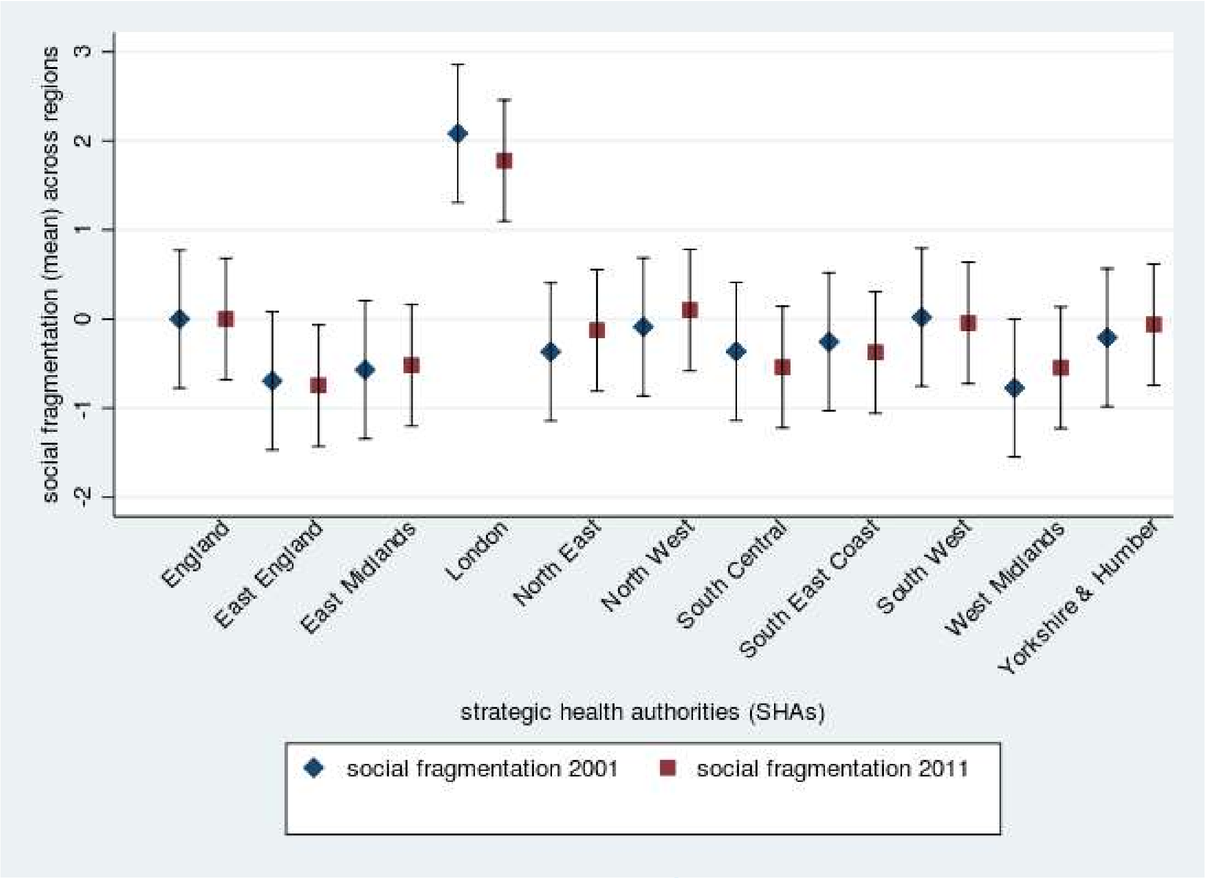

Figure A Index of Social Fragmentation (mean) for England and English regions, over time.

Figure A1: Spatial autocorrelation with Moran's I for single persons (top) and one person households (bottom), by region and over time

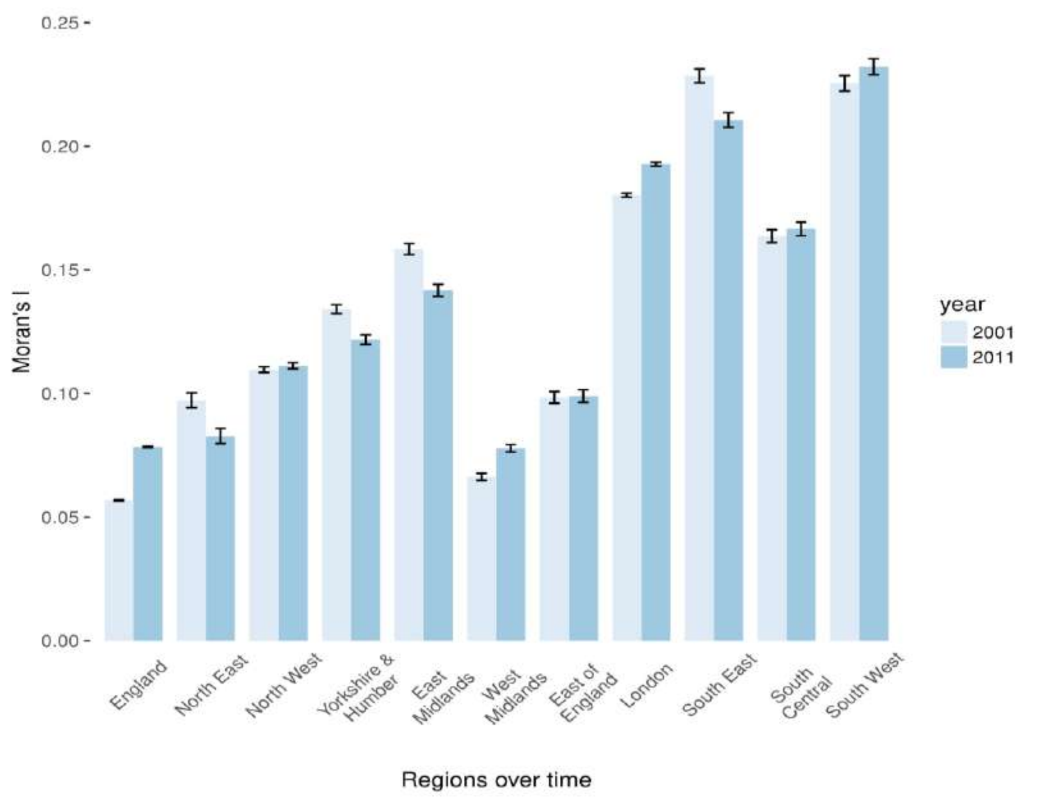

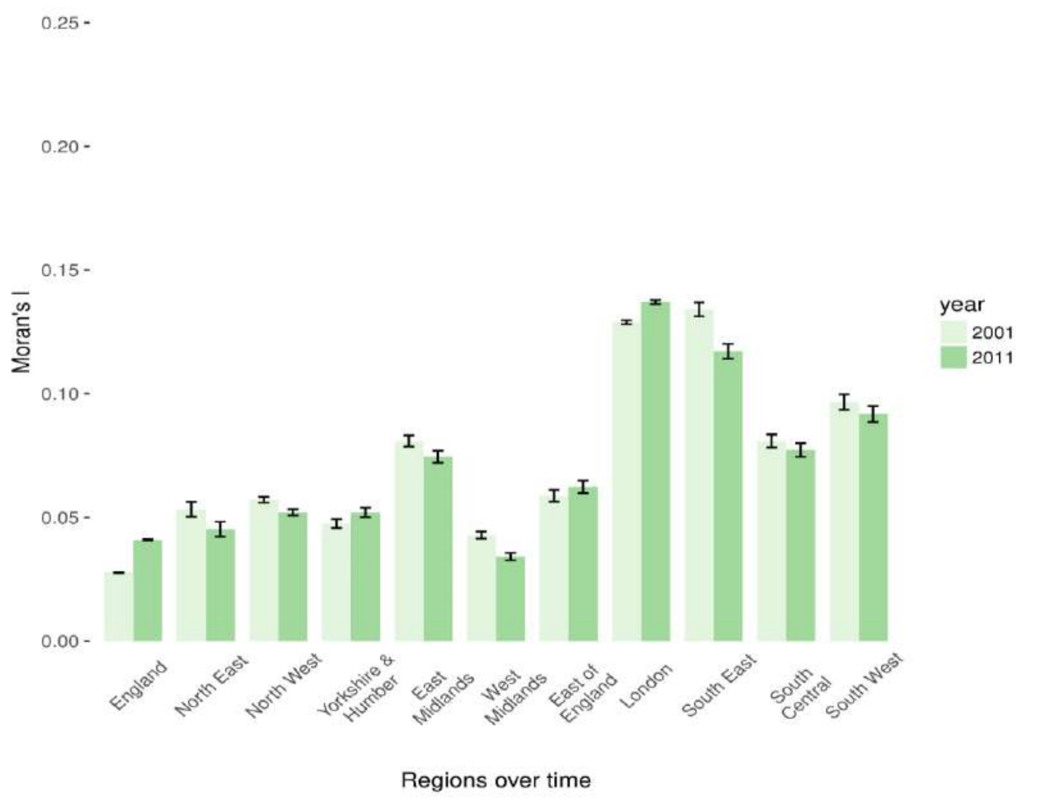

Figure A2: Spatial autocorrelation with Moran's I for private renting (top) and population turnover (bottom), by region and over time

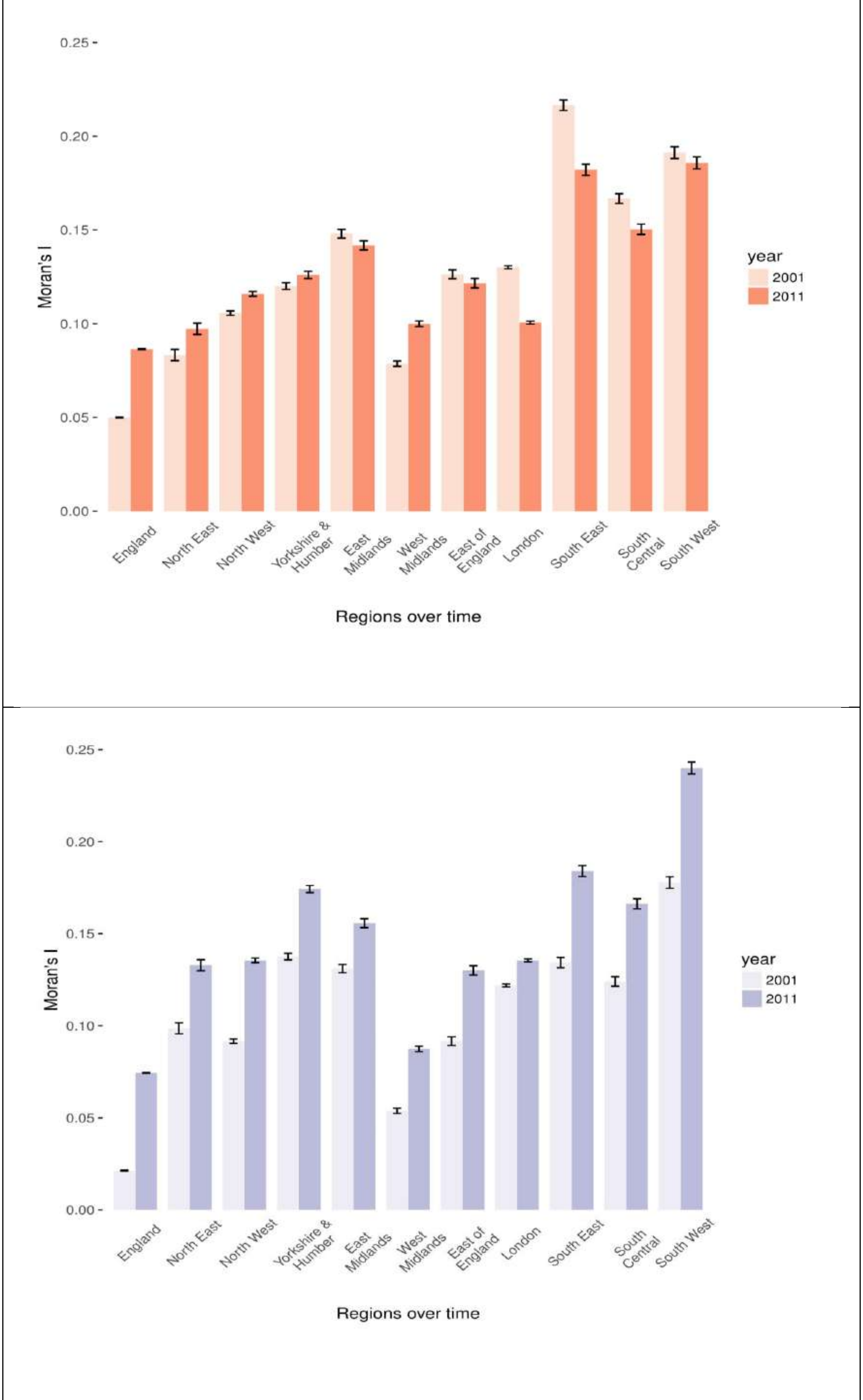

Social Fragmentation

England 2011, Social Fragmentation

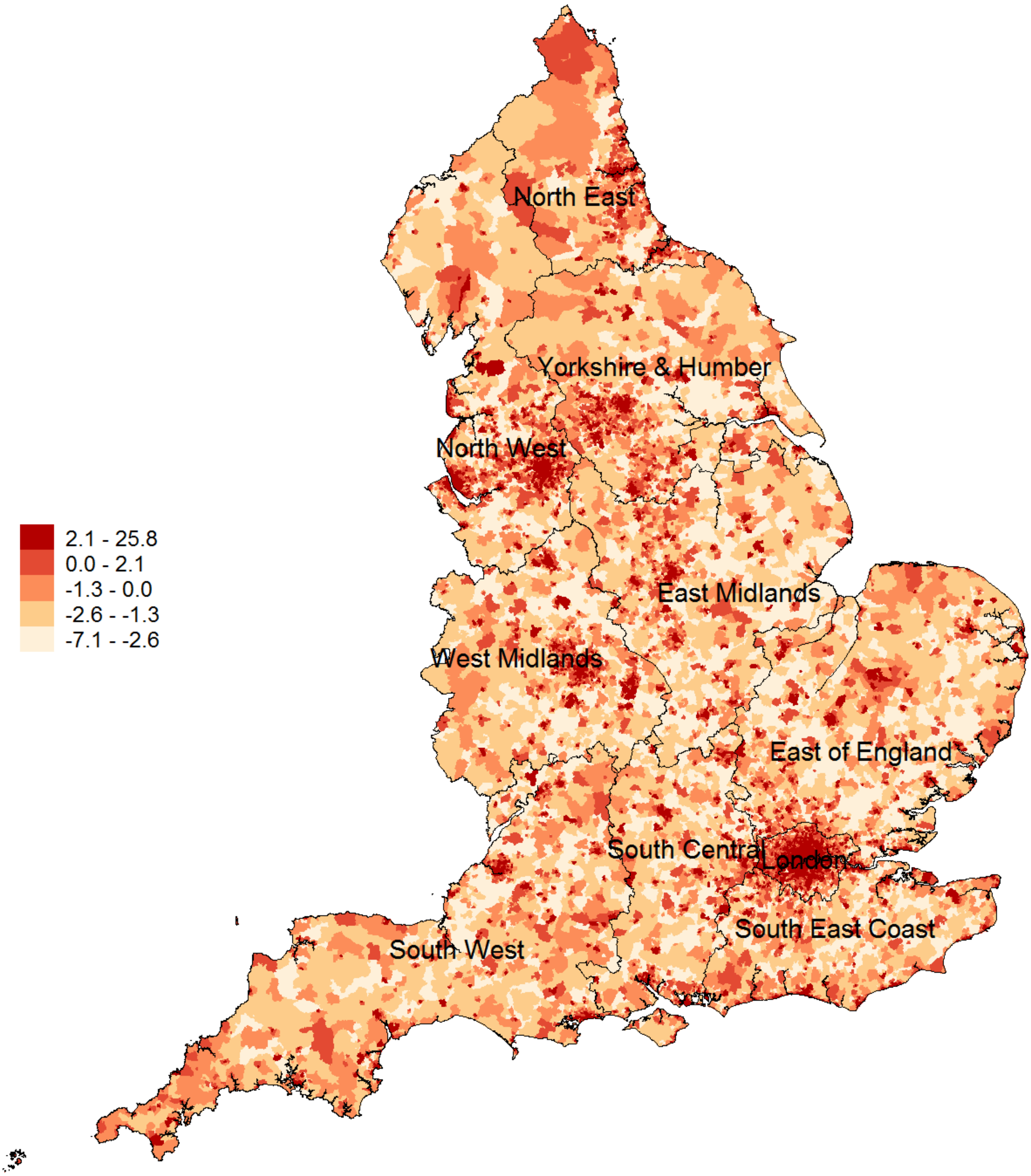

North East, Social Fragmentation

2001 | 2011

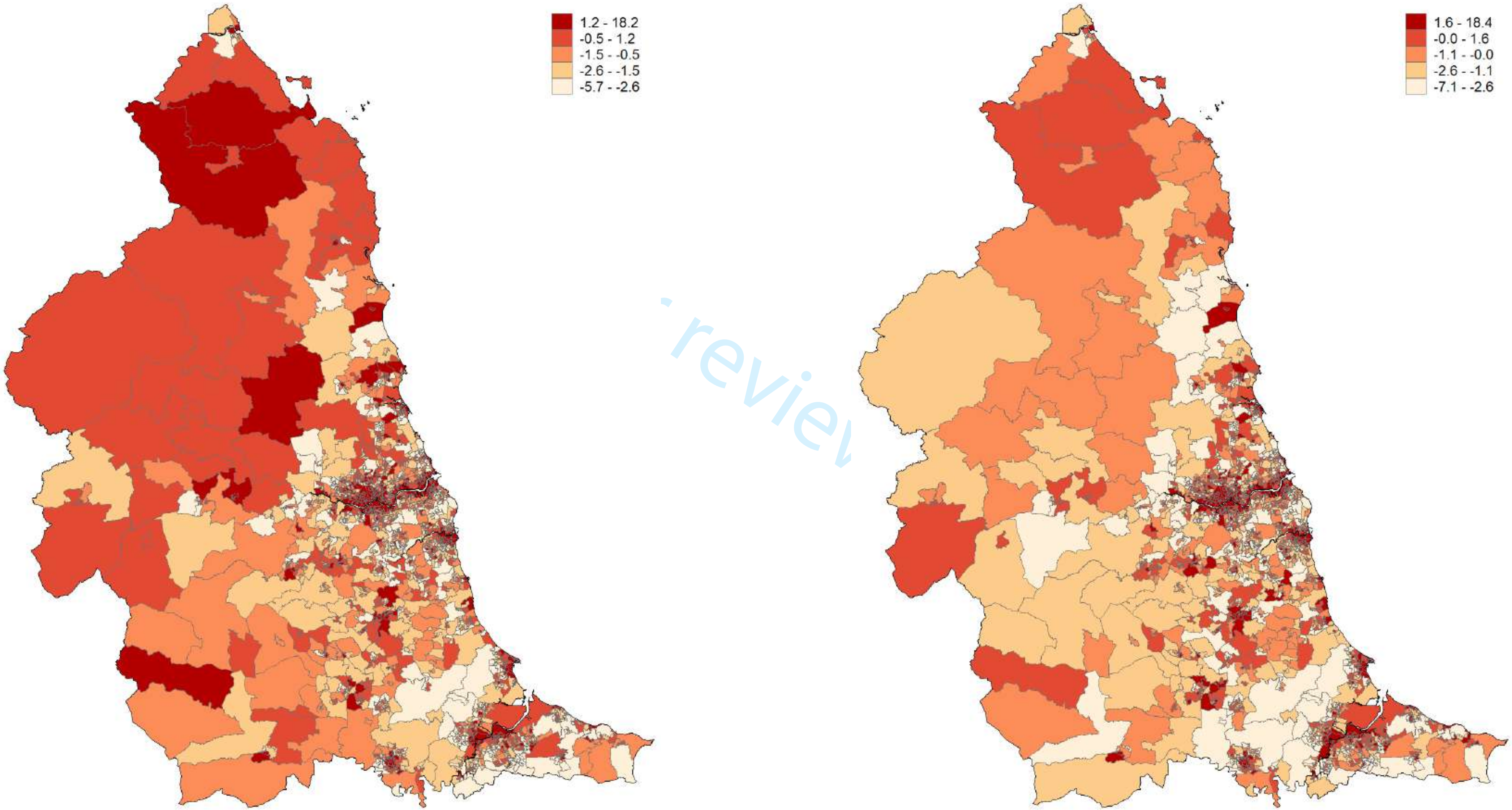

North East, Social Fragmentation

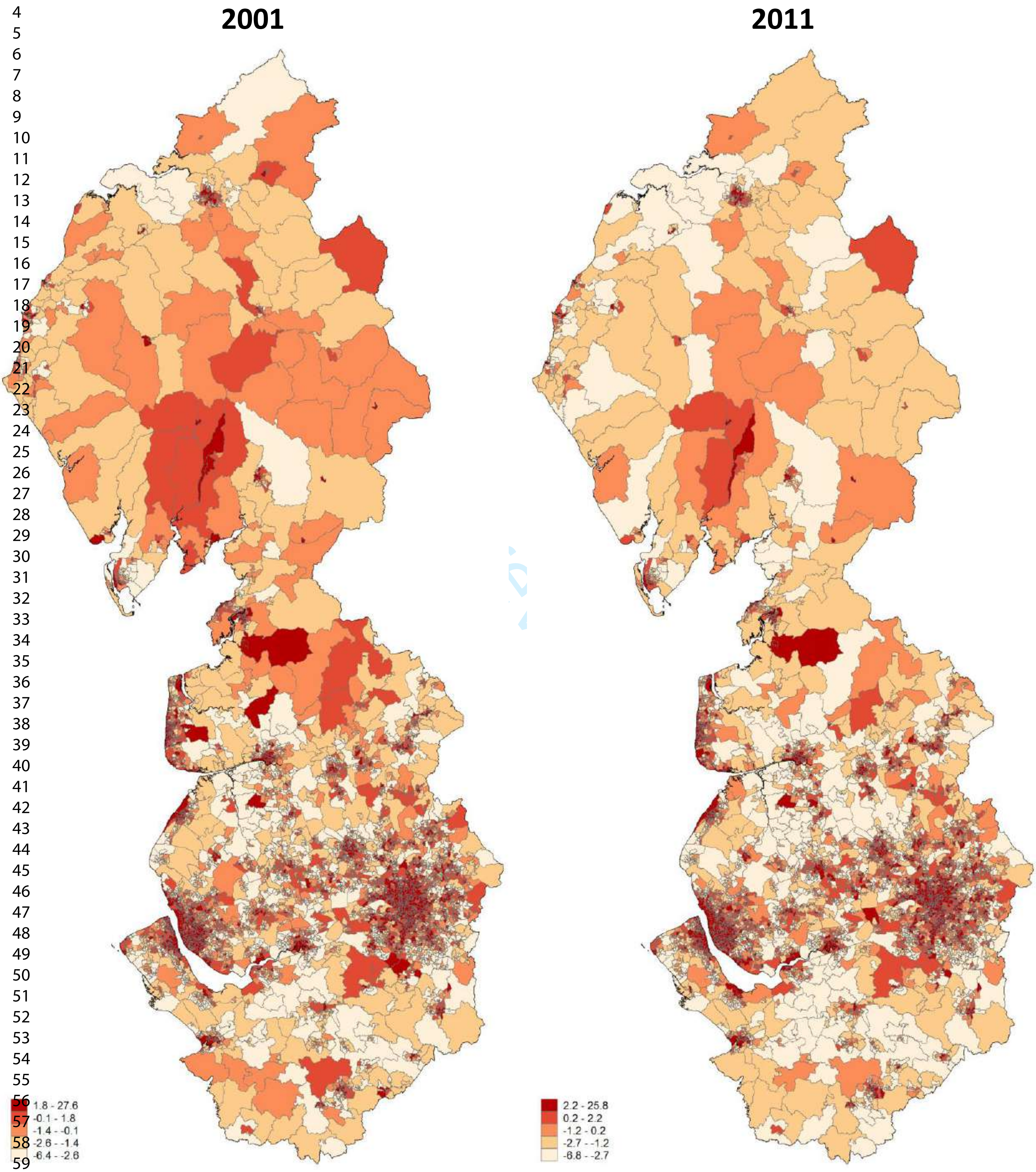

Yorkshire and the Humber, Social Fragmentation

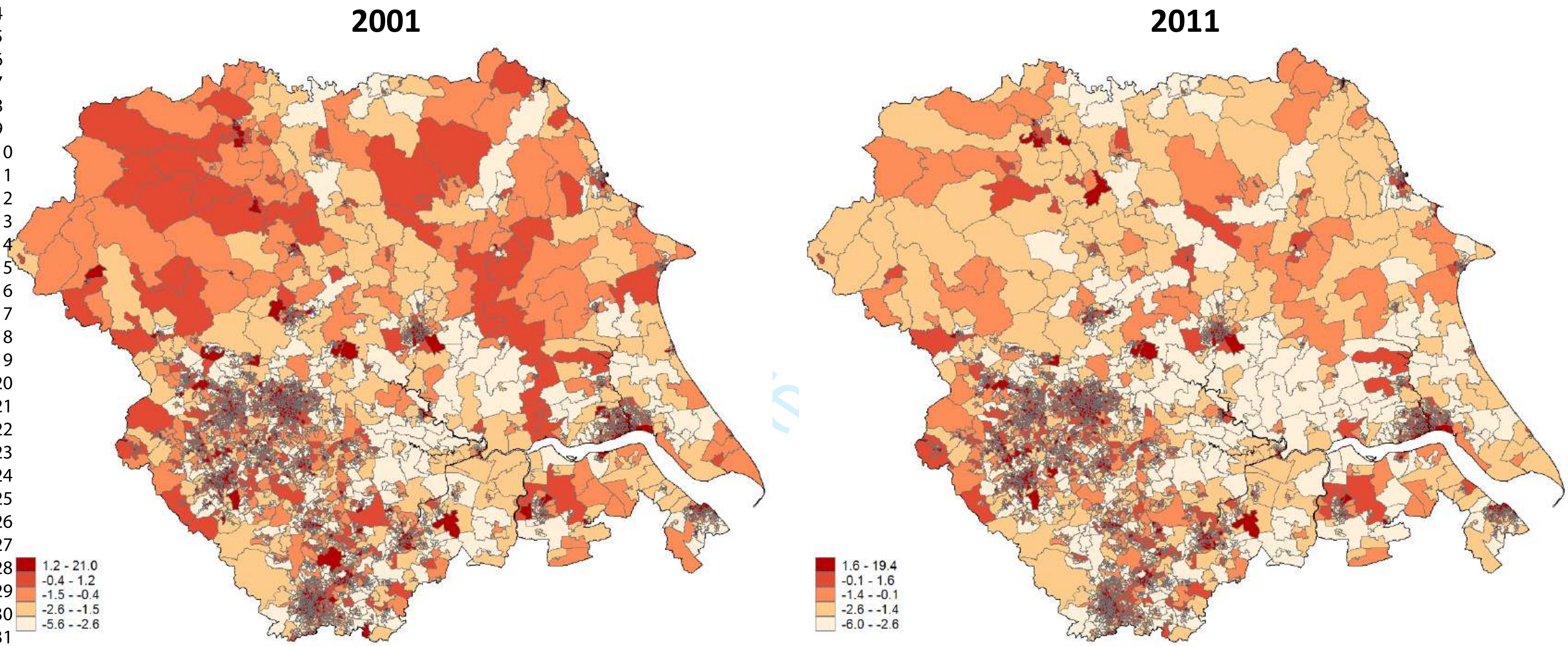

East Midlands, Social Fragmentation

2001 / 2011

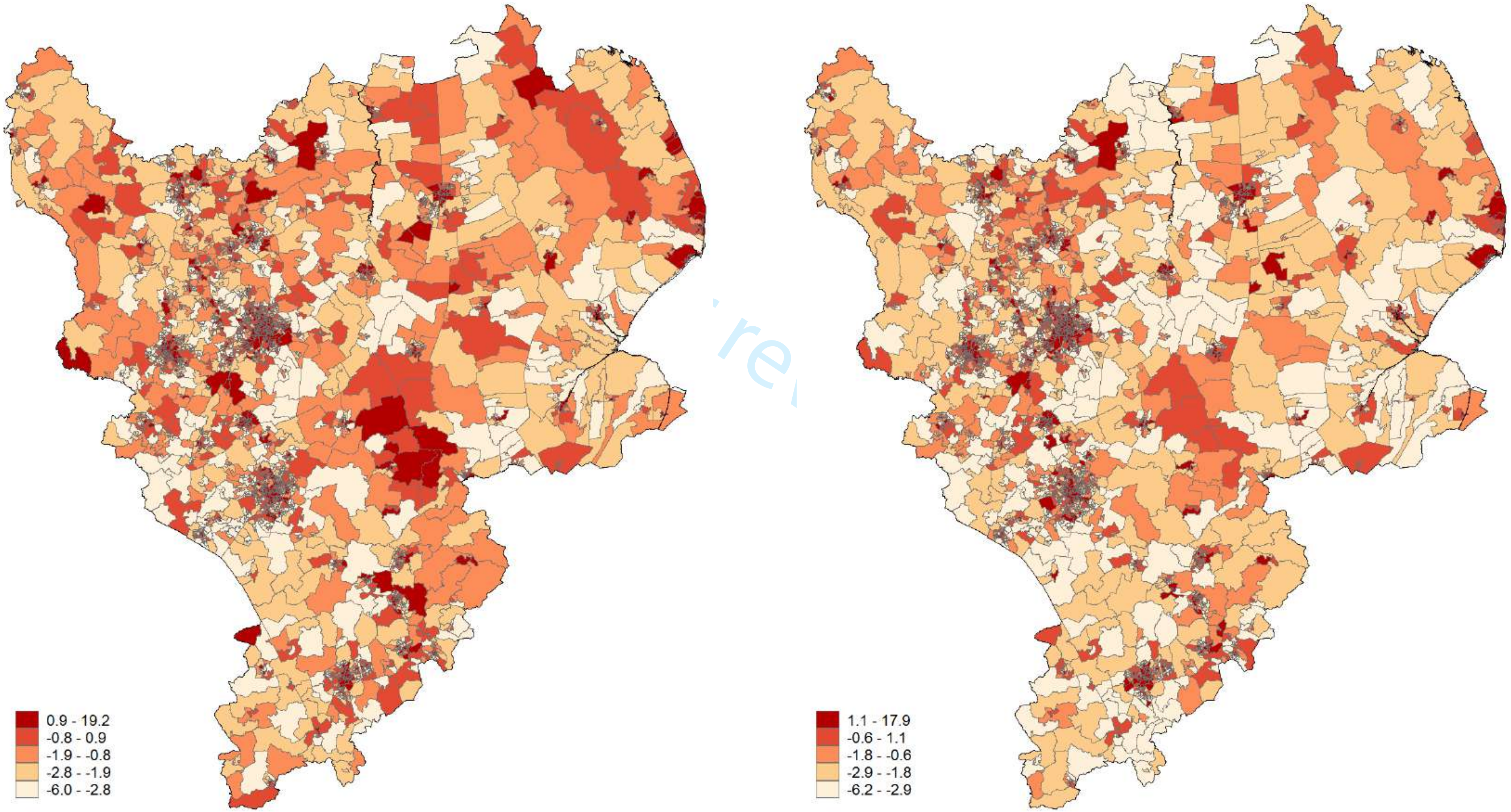

West Midlands, Social Fragmentation

2001

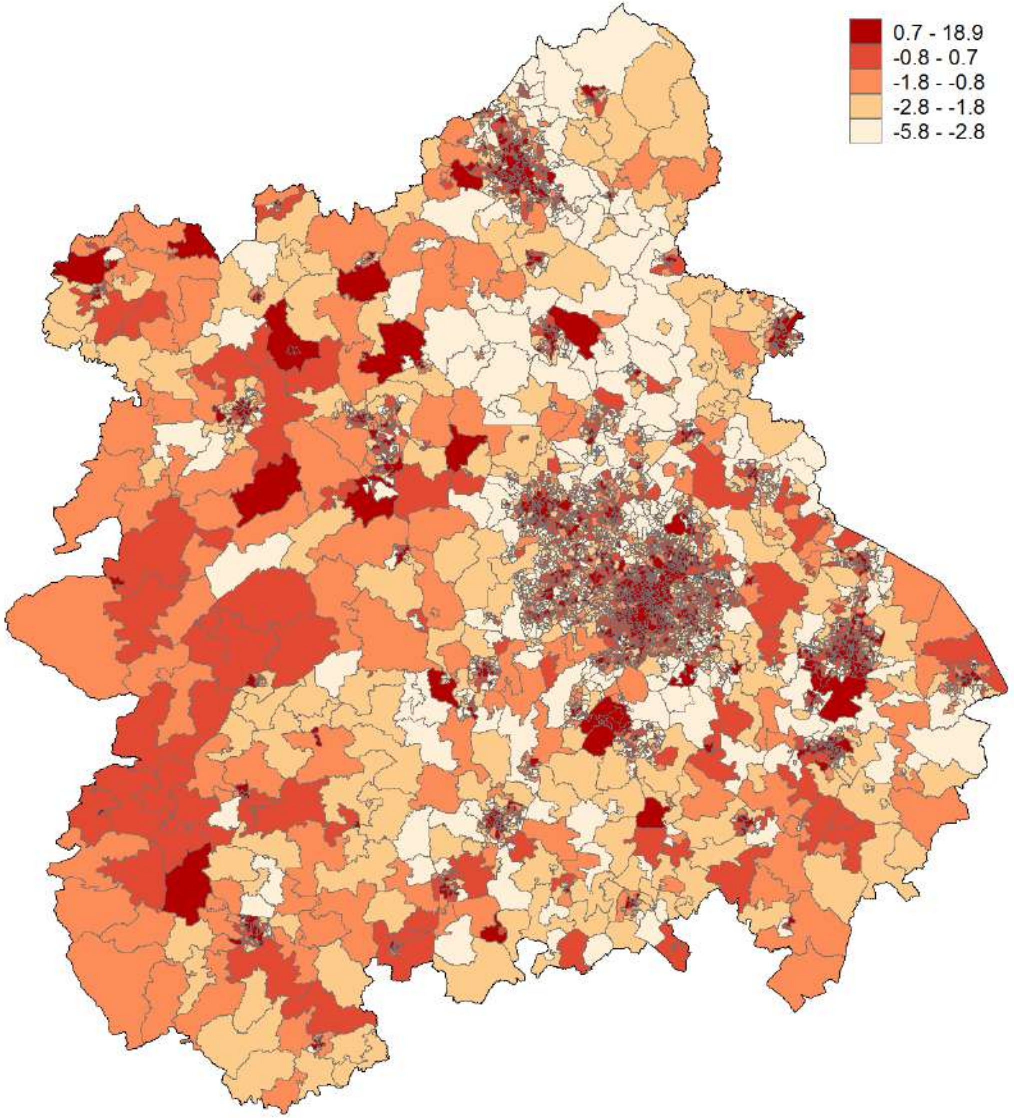

2011

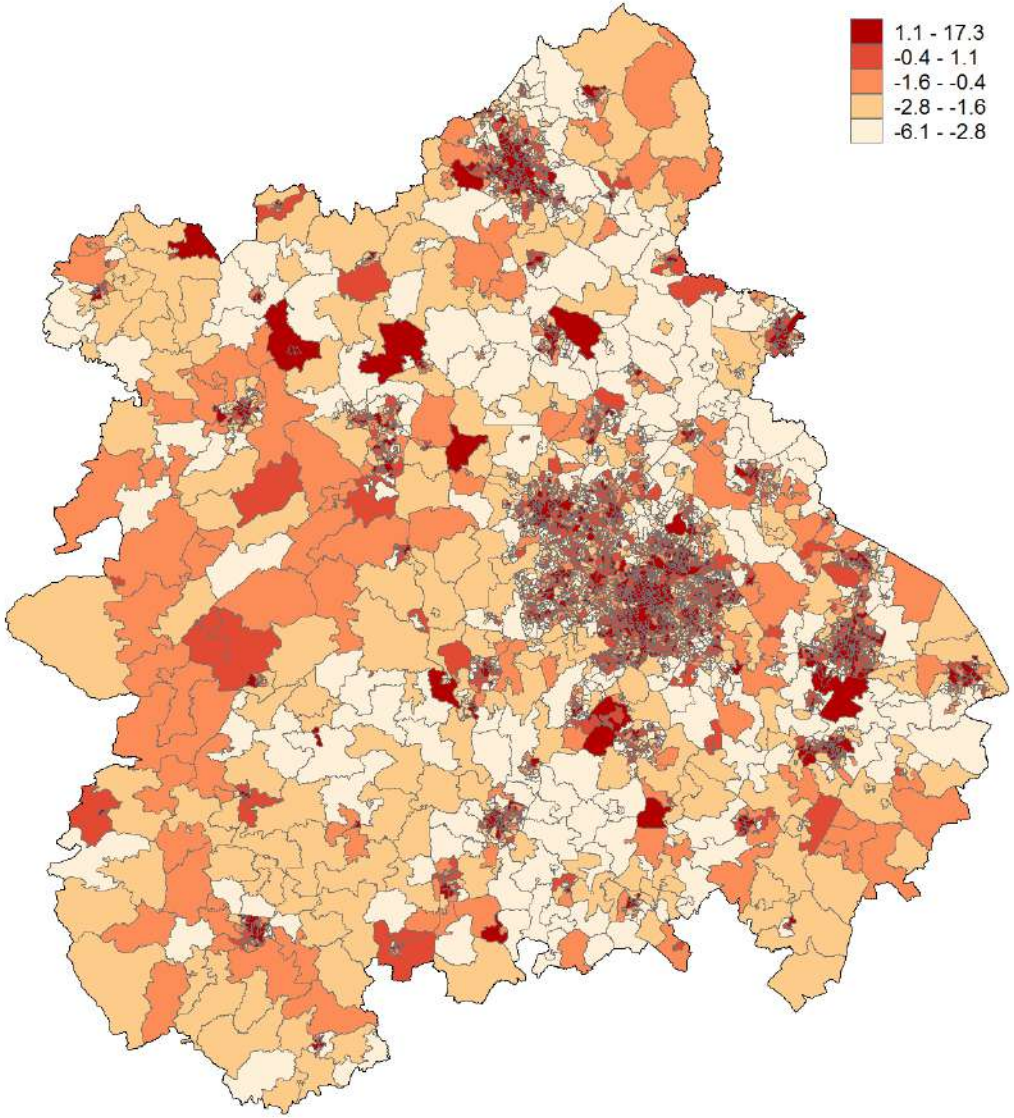

East of England, Social Fragmentation

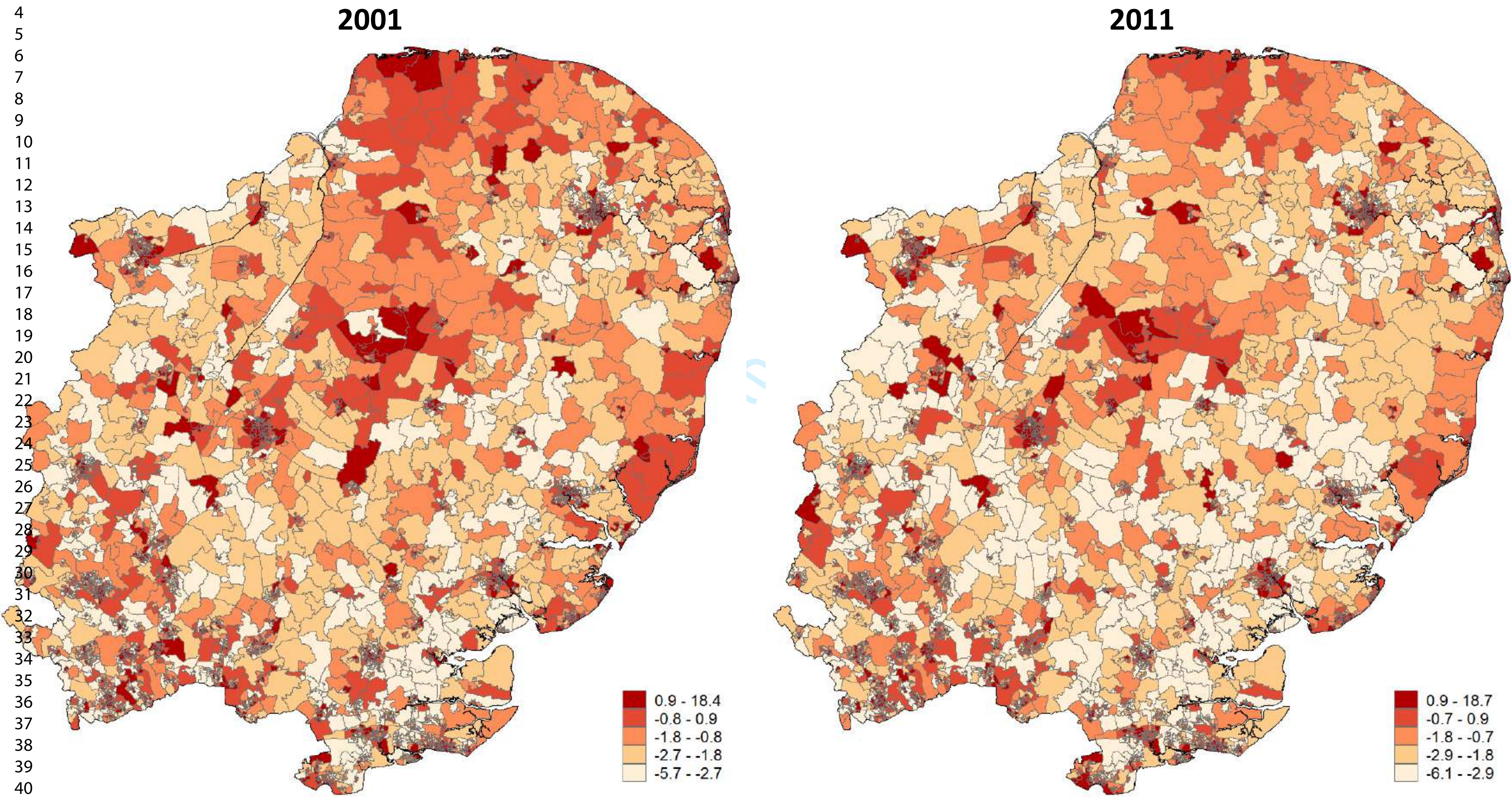

London, Social Fragmentation

2001

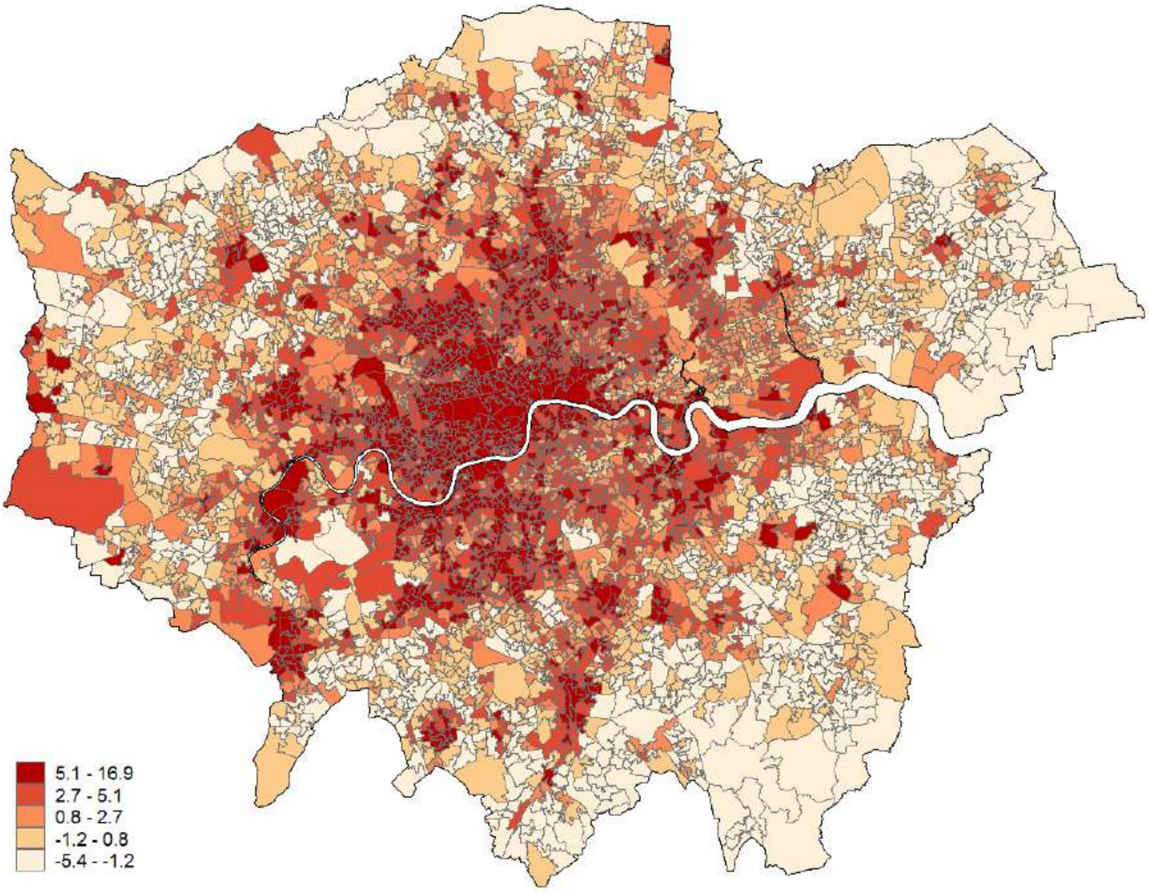

2011

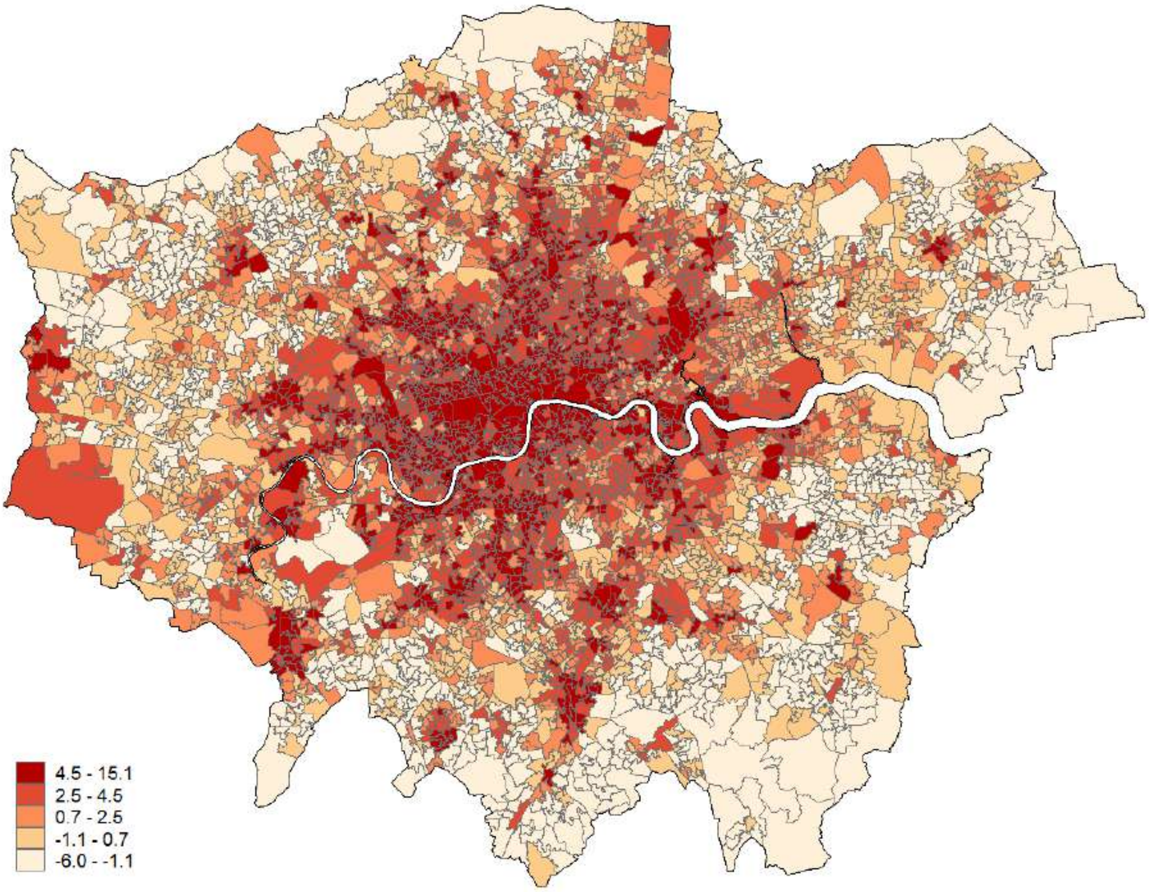

South East Coast, Social Fragmentation

2001

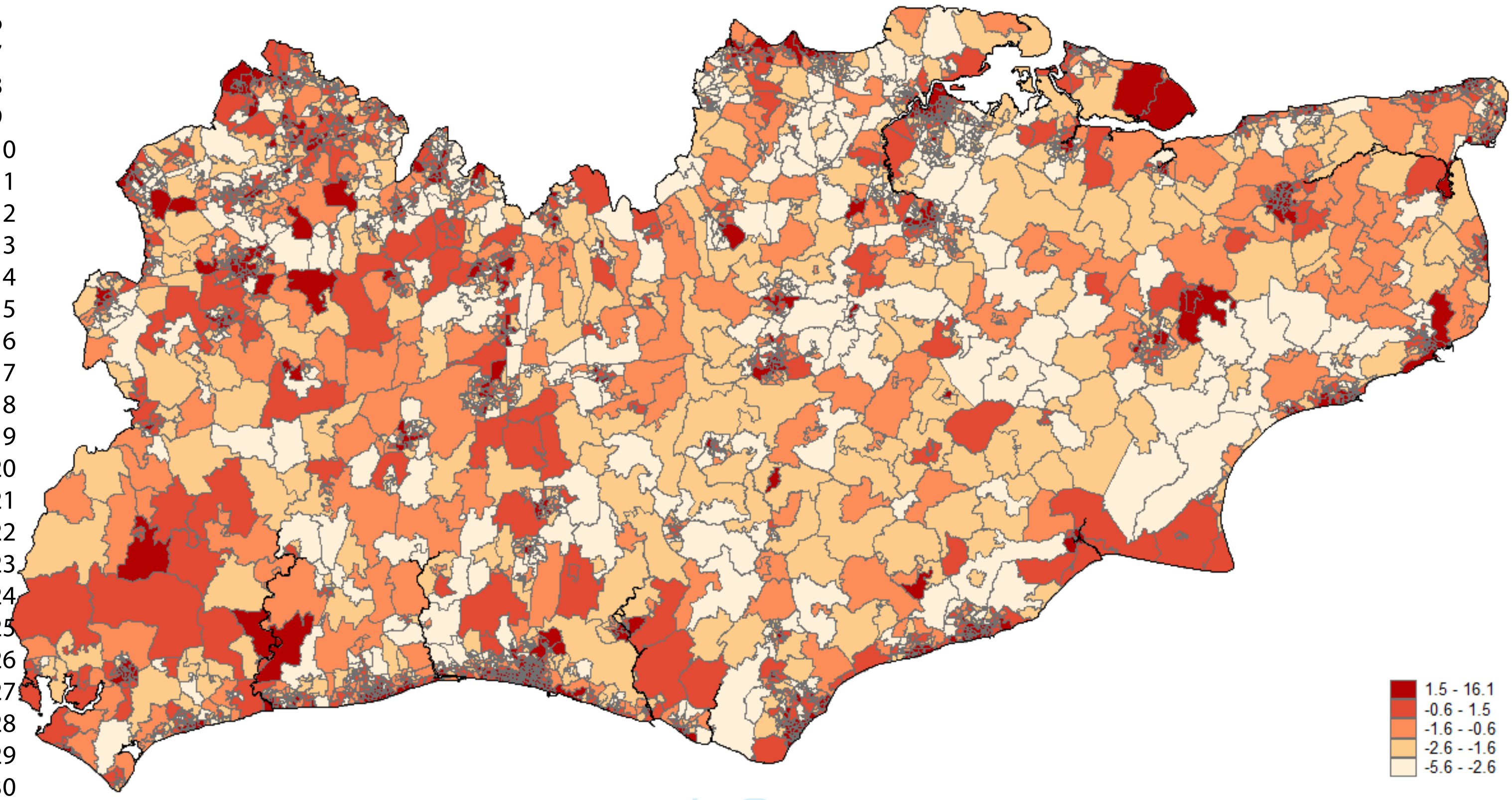

2011

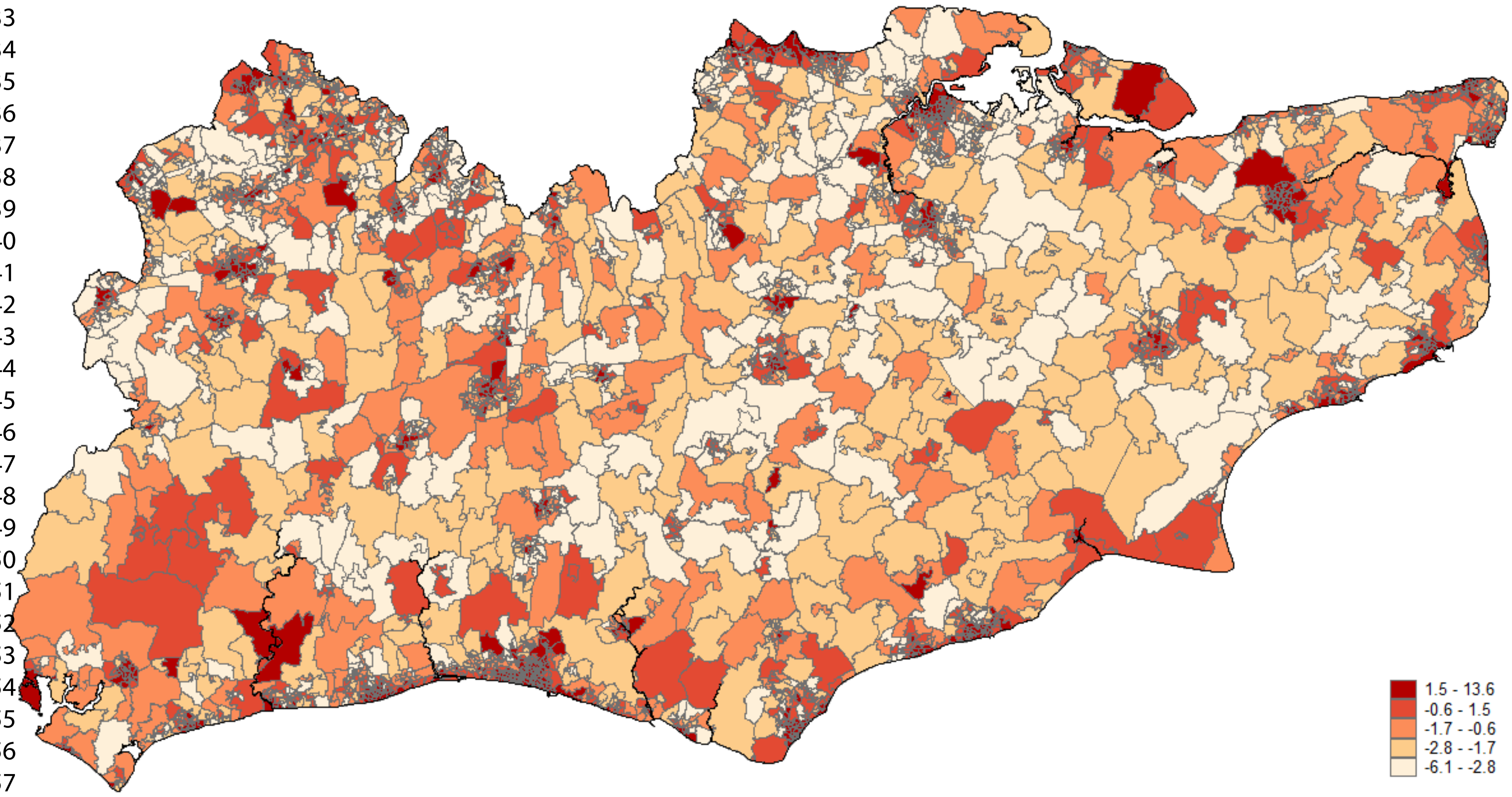

South Central, Social Fragmentation

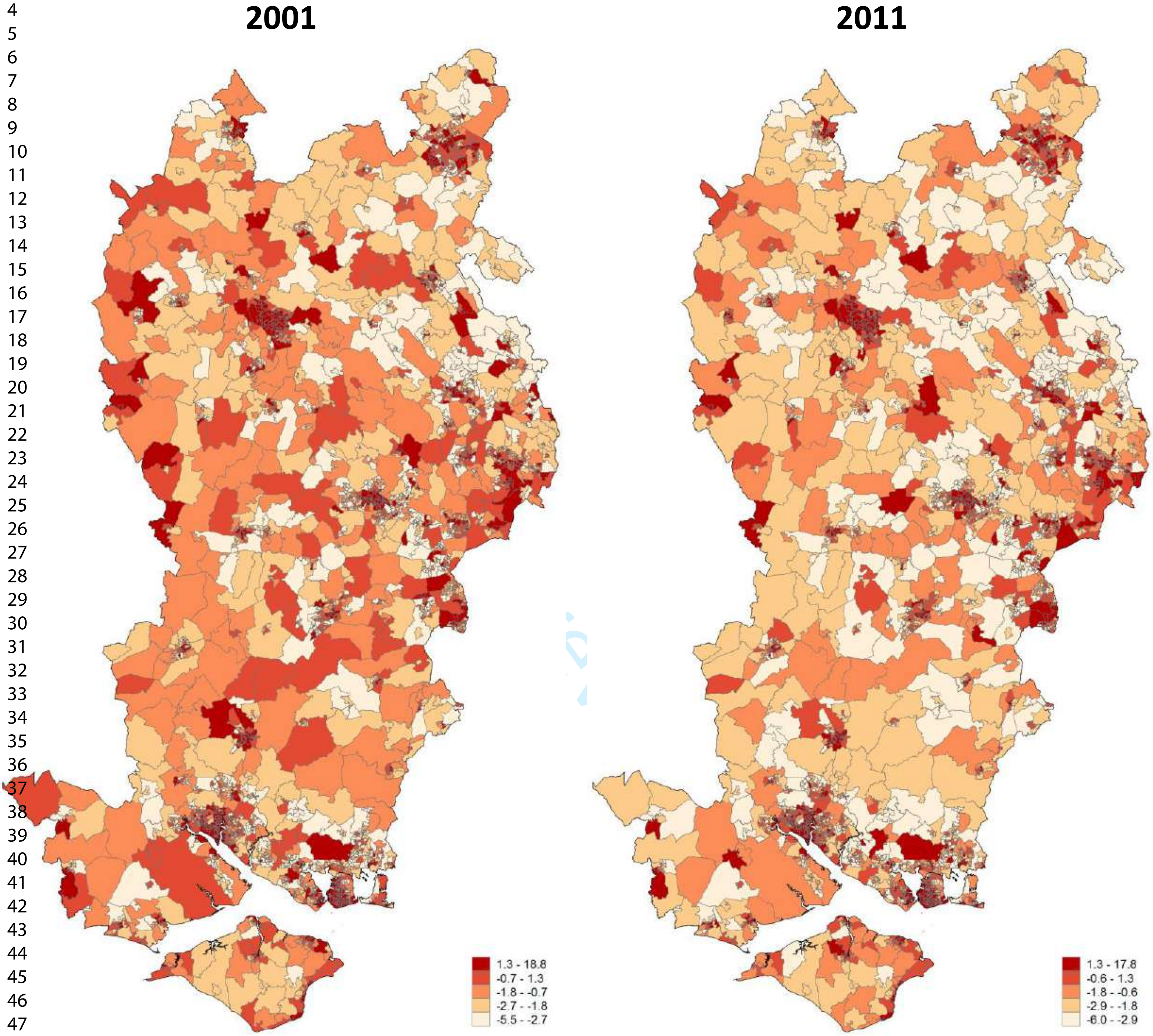

South West, Social Fragmentation

2001

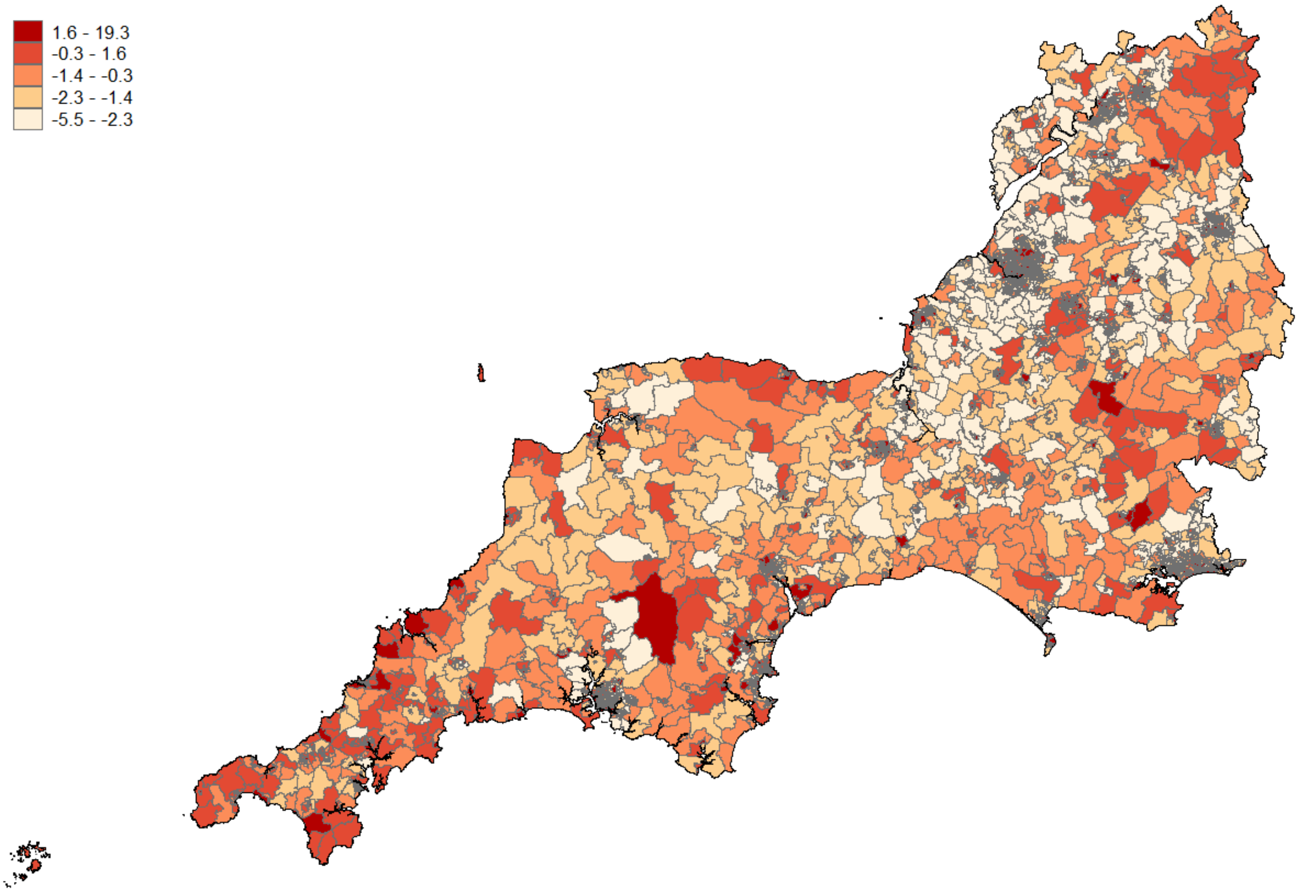

2011

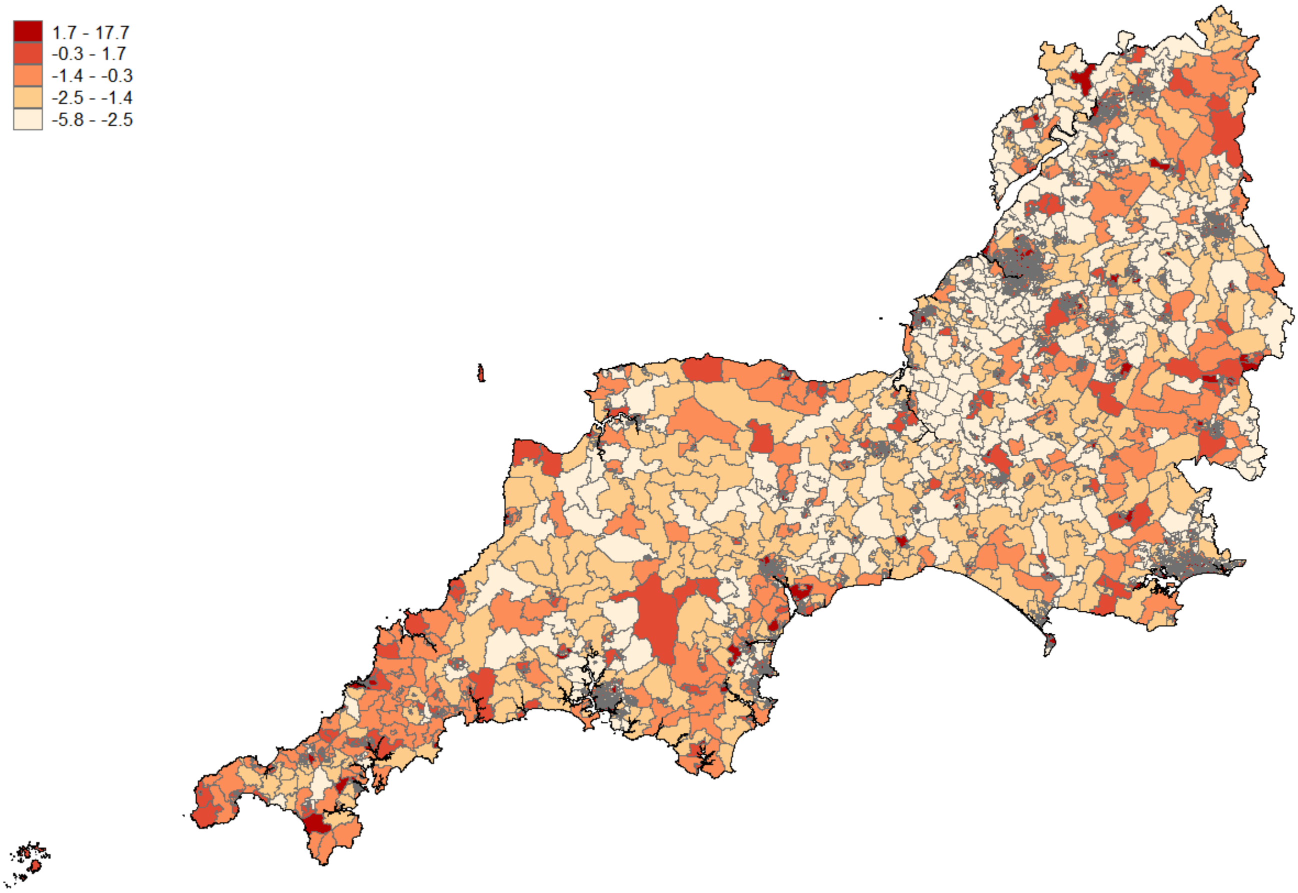

Single People

England 2011, Single People % of population

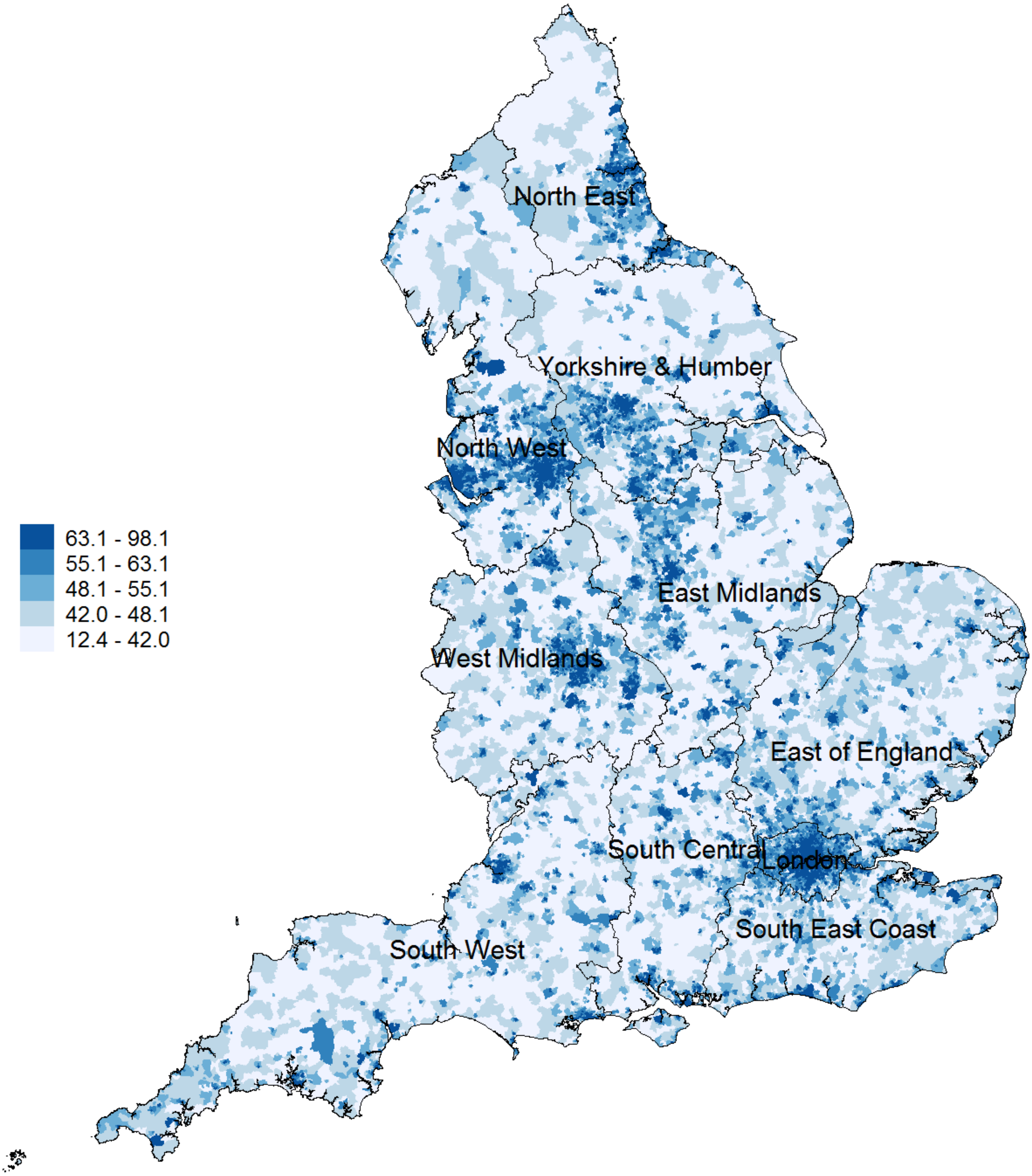

North East, Single People % of population

2001 | 2011

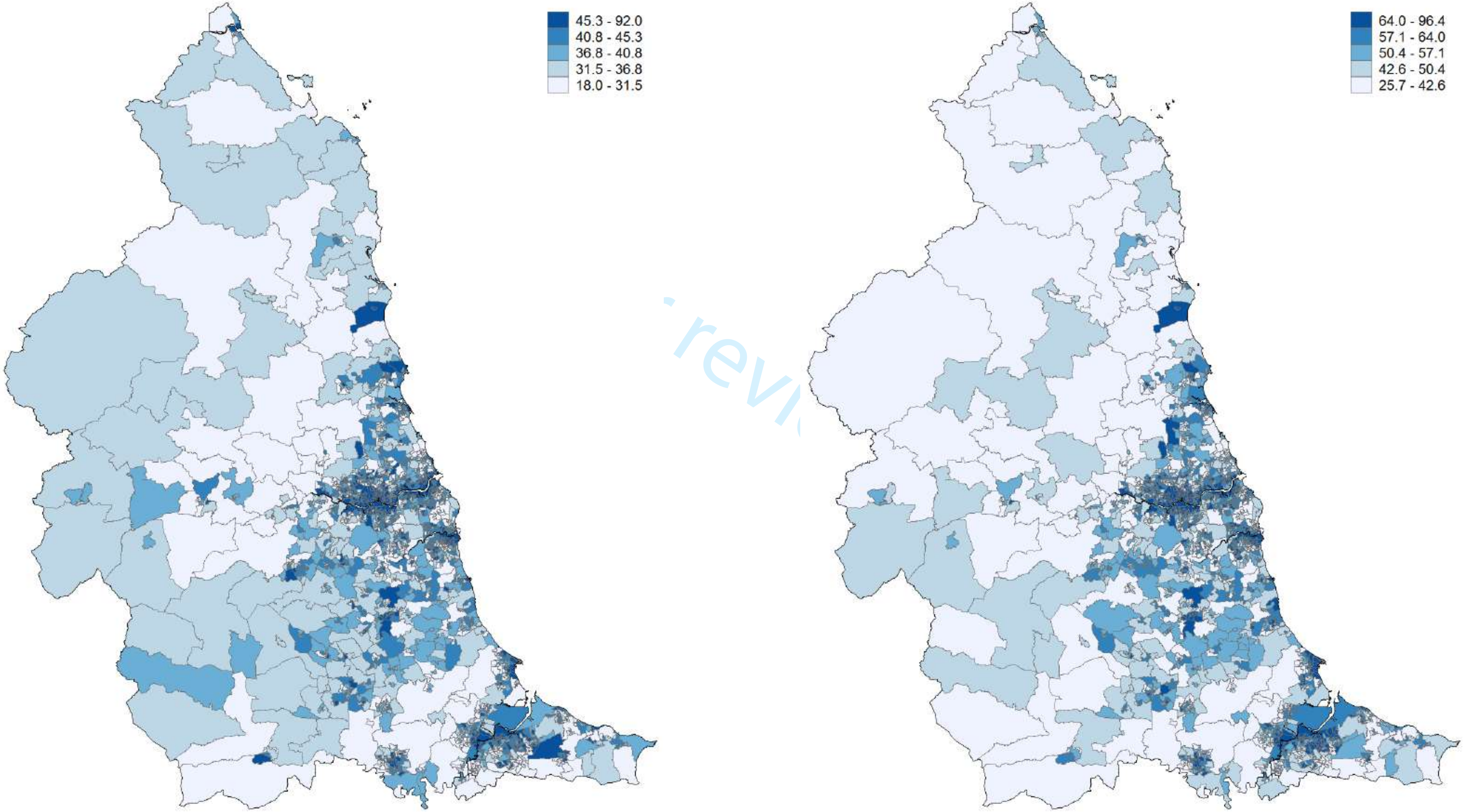

North East, Single People % of population

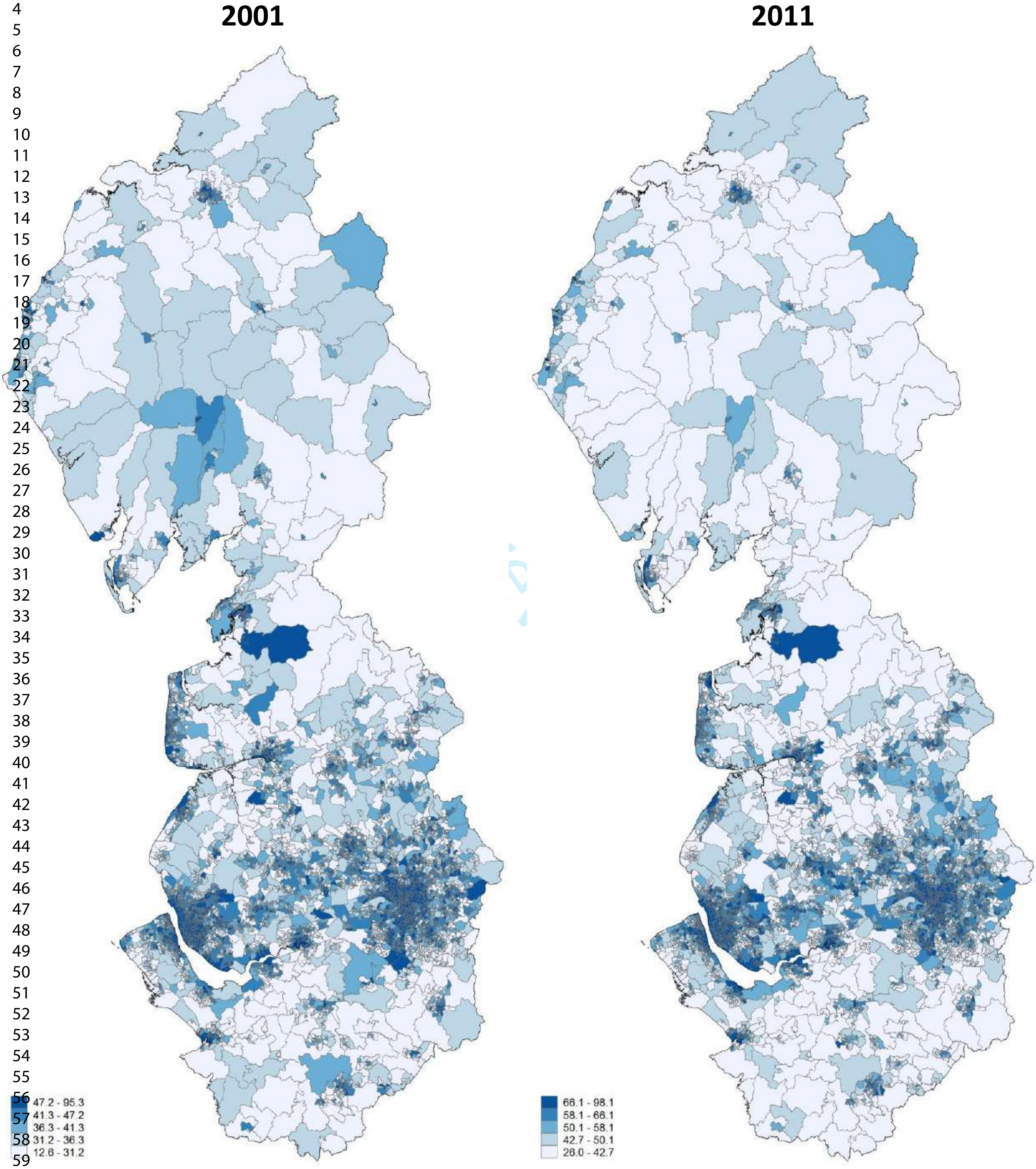

Yorkshire and the Humber, Single People % of population

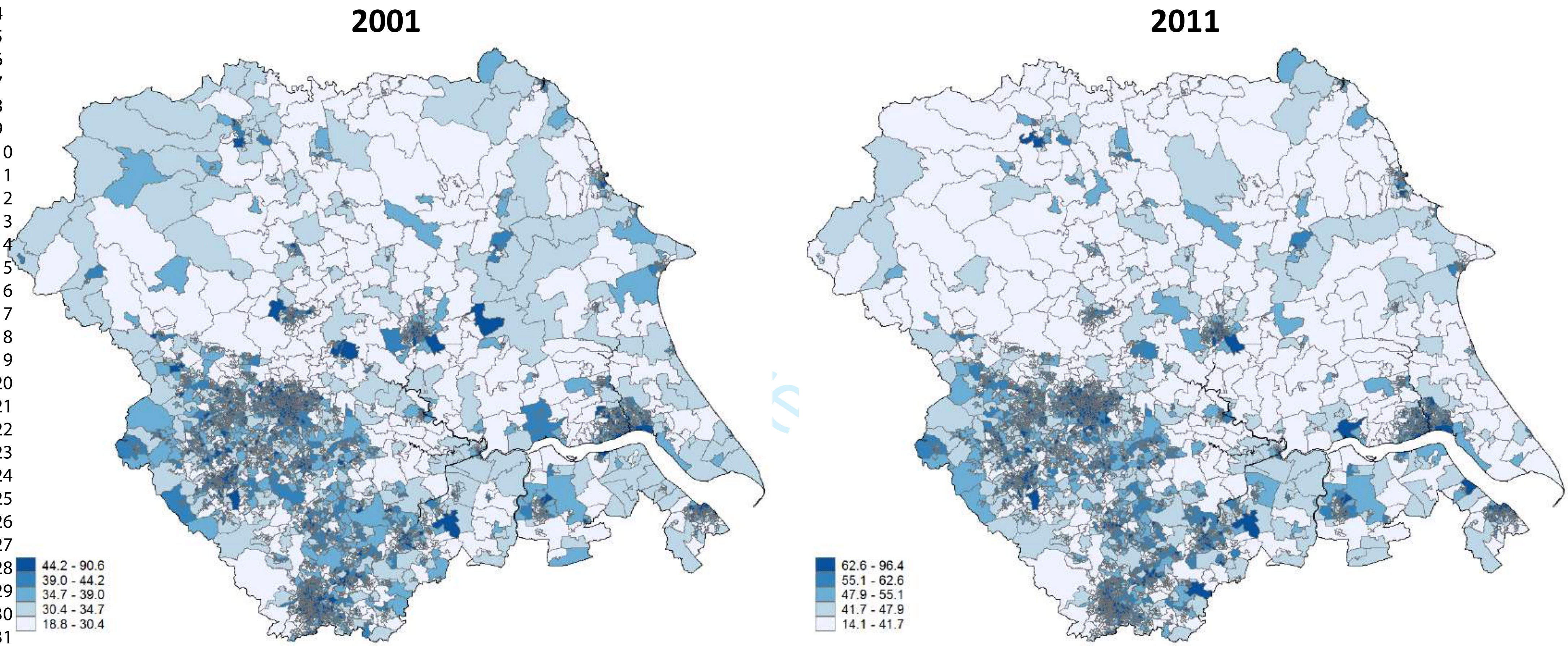

East Midlands, Single People % of population

2001 | 2011

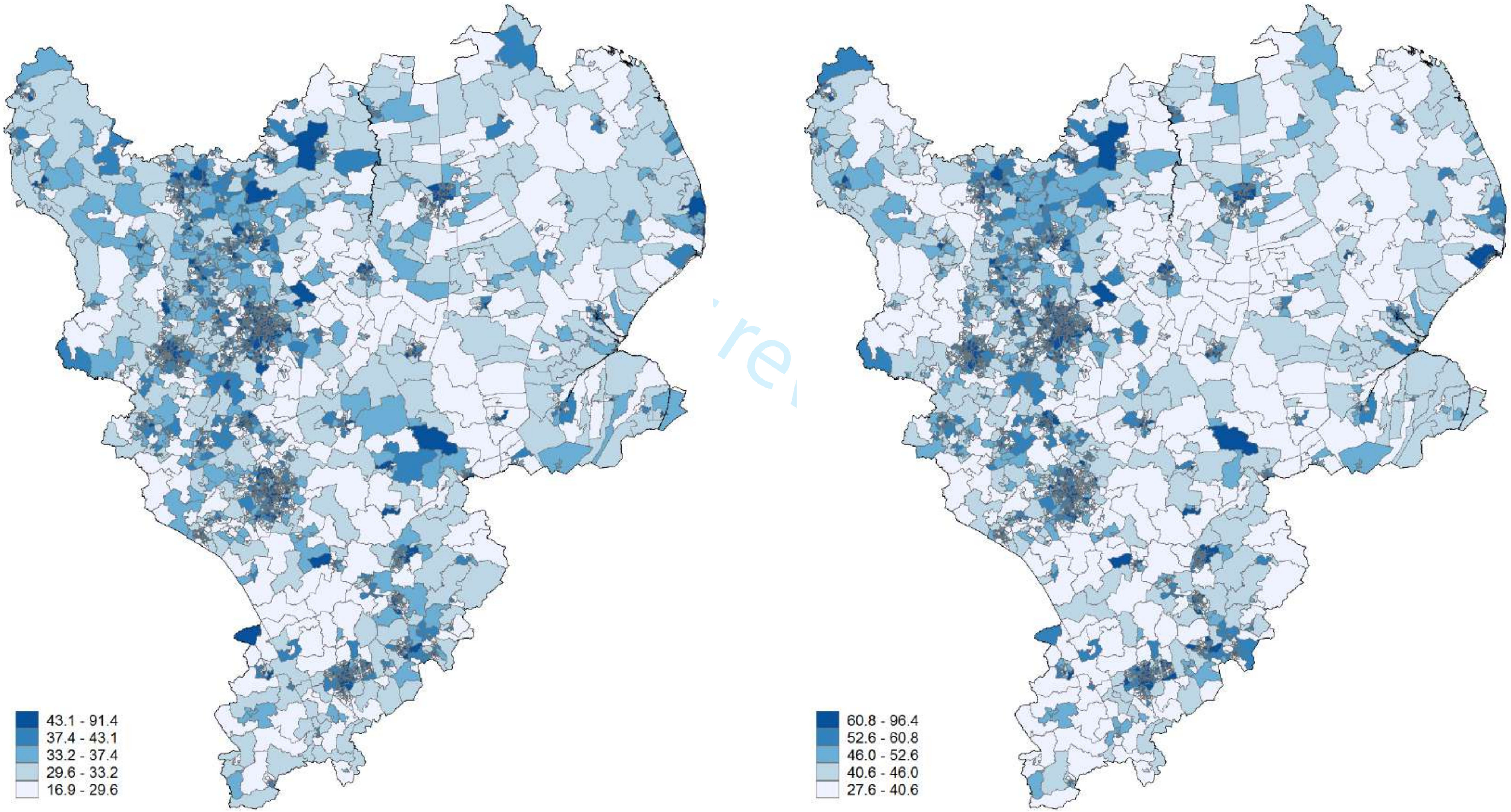

West Midlands, Single People % of population

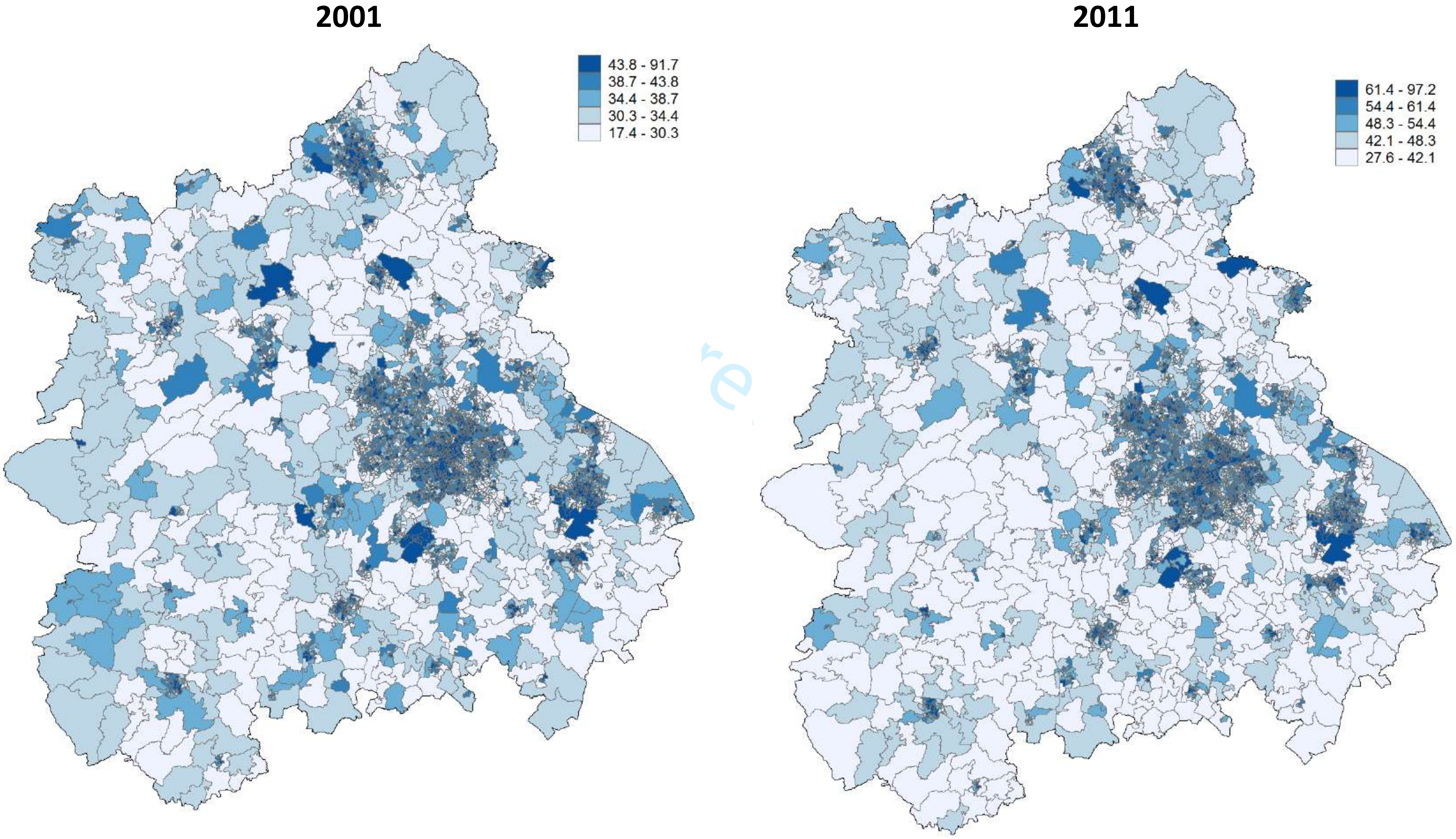

East of England, Single People % of population

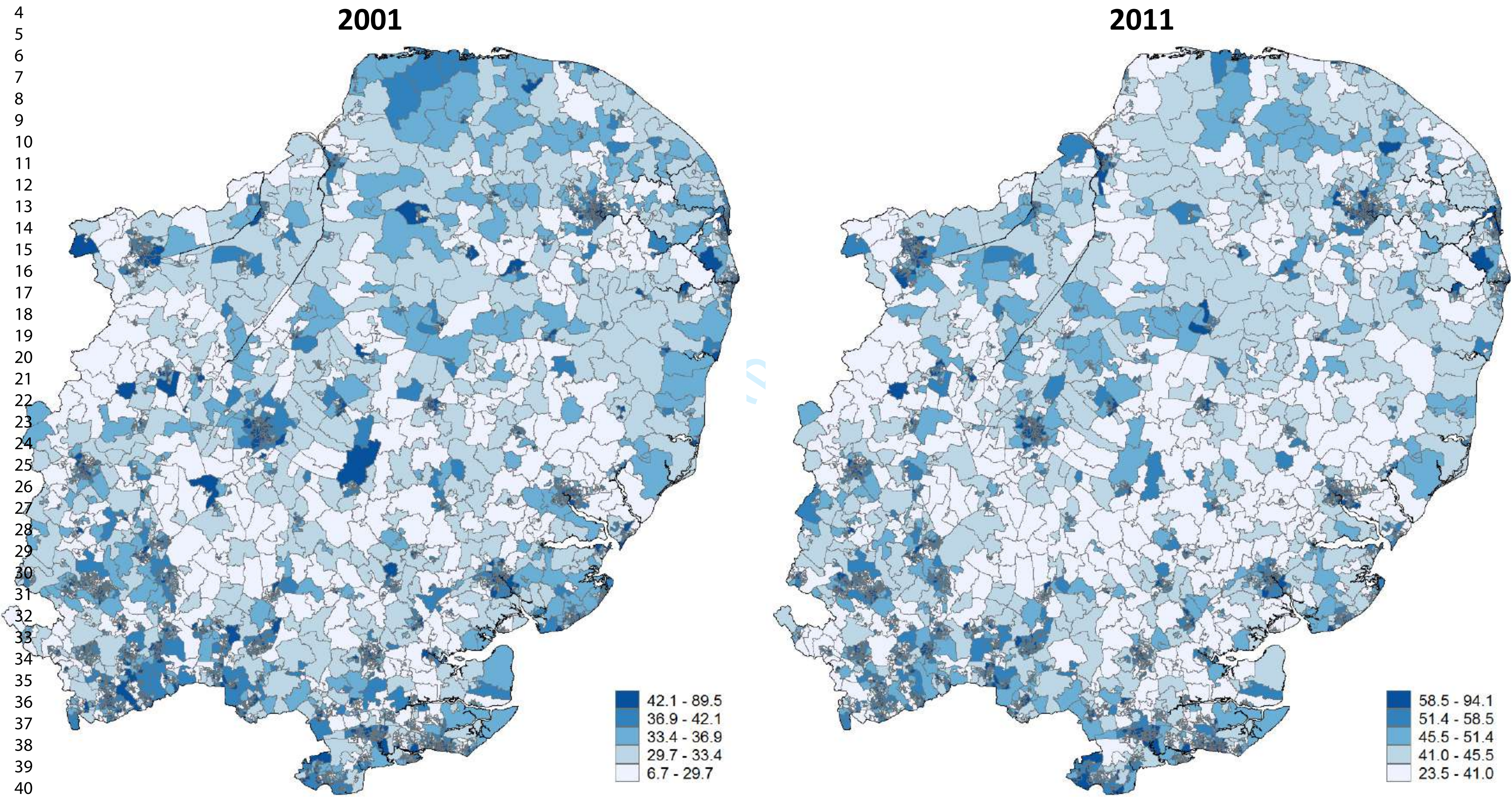

London, Single People % of population

2001

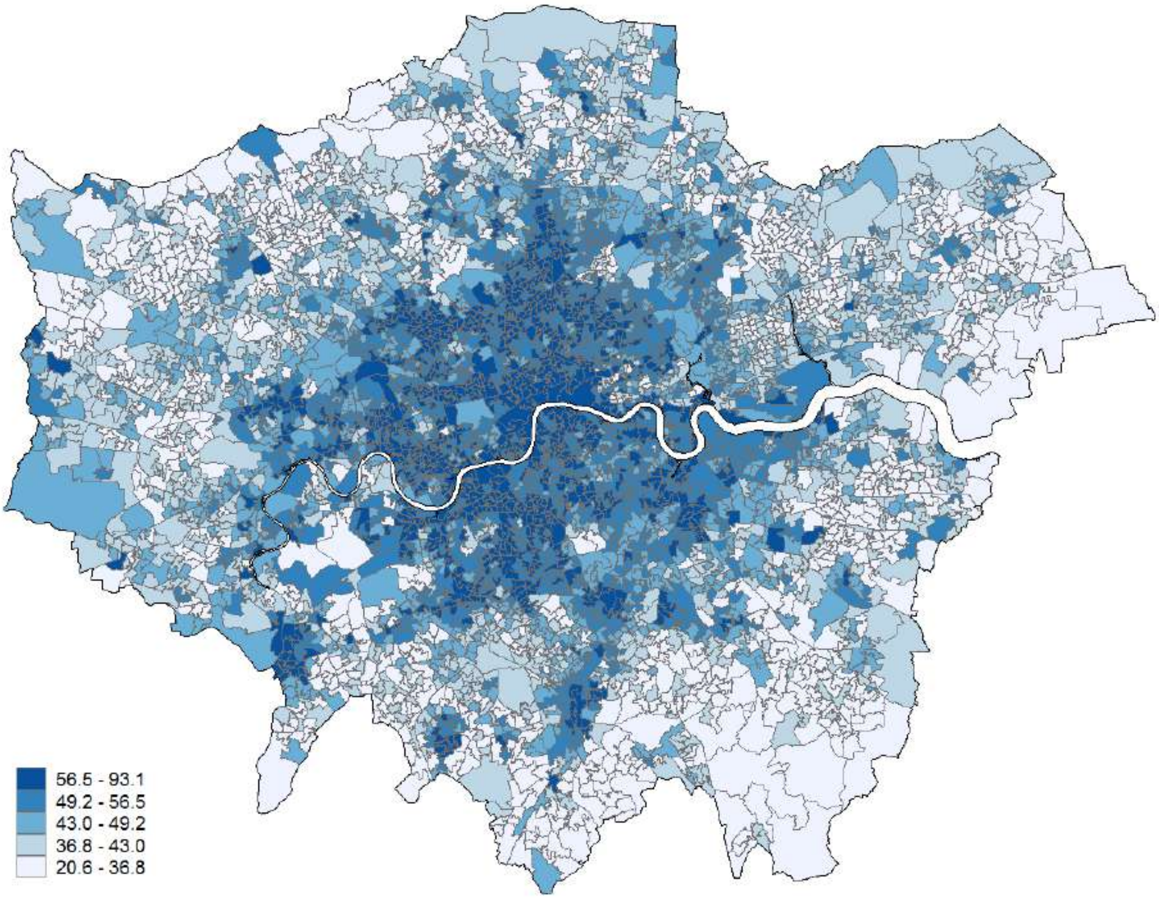

2011

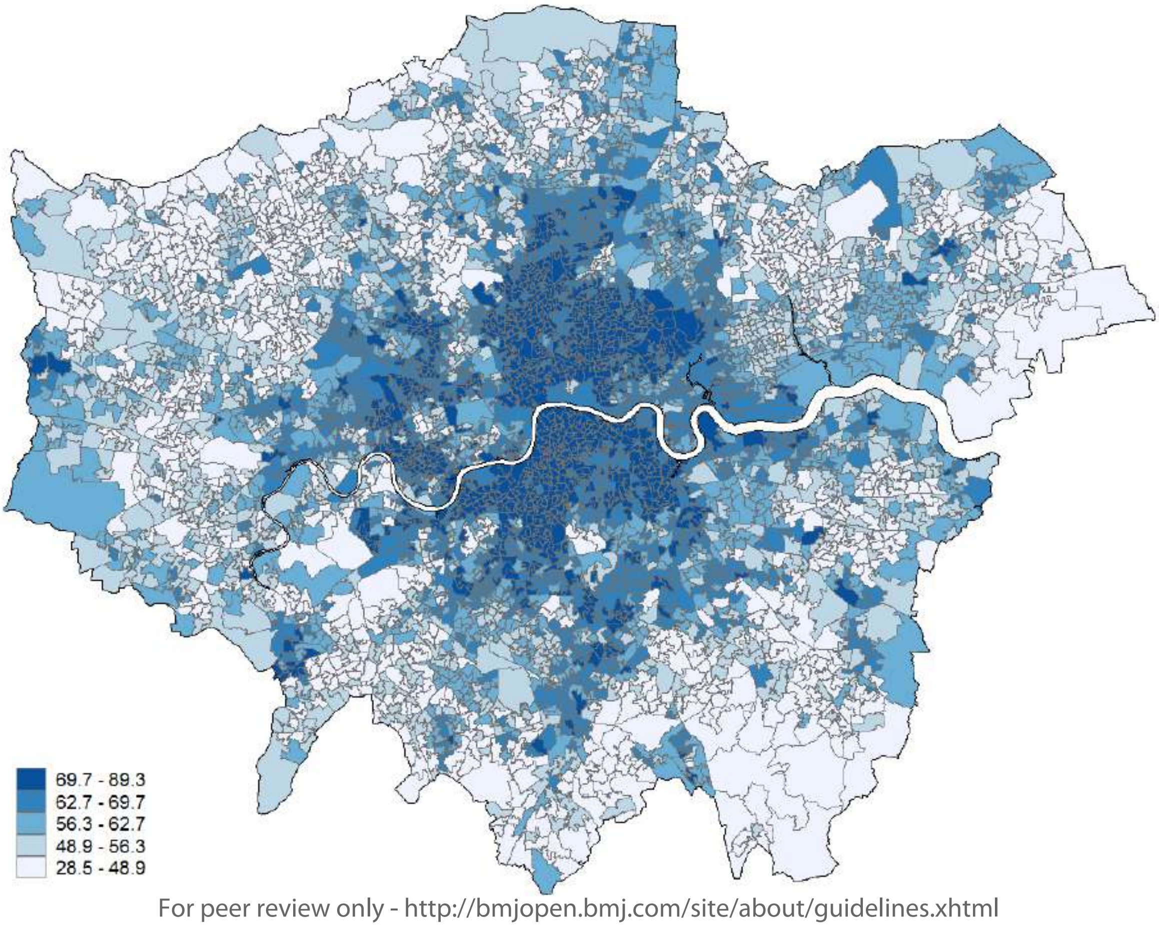

South East Coast, Single People % of population

2001

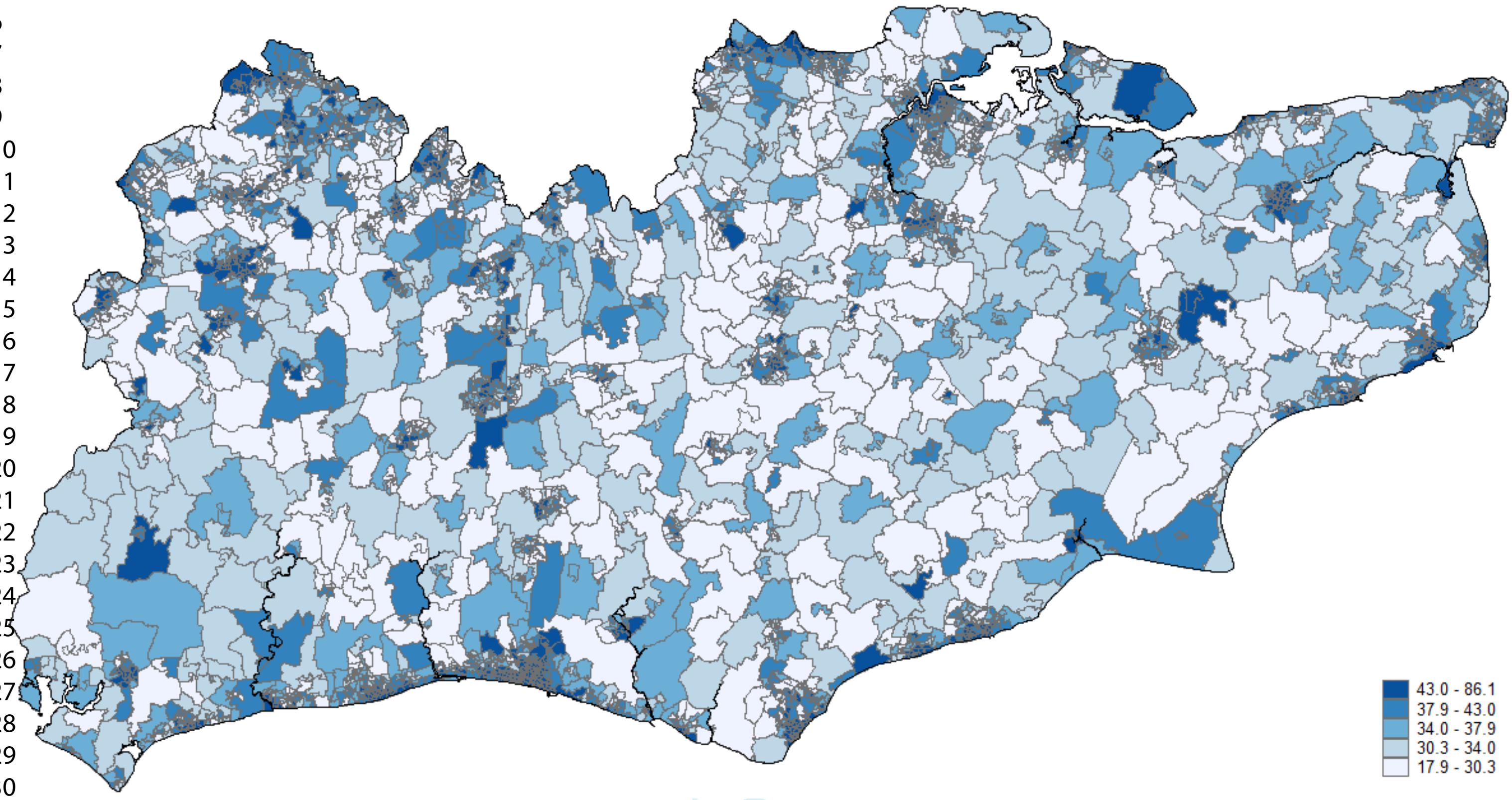

2011

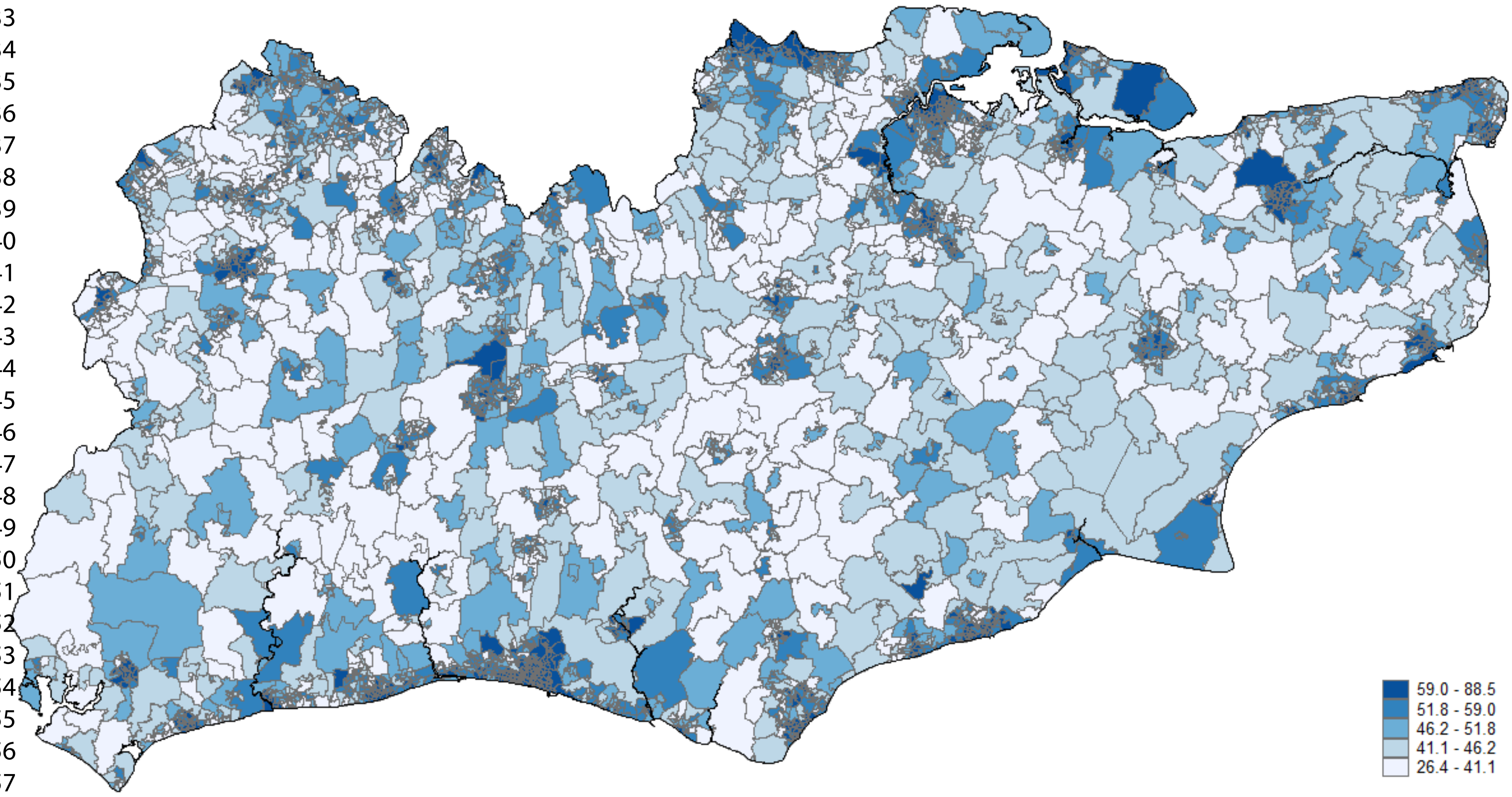

South Central, Single People % of population

2001

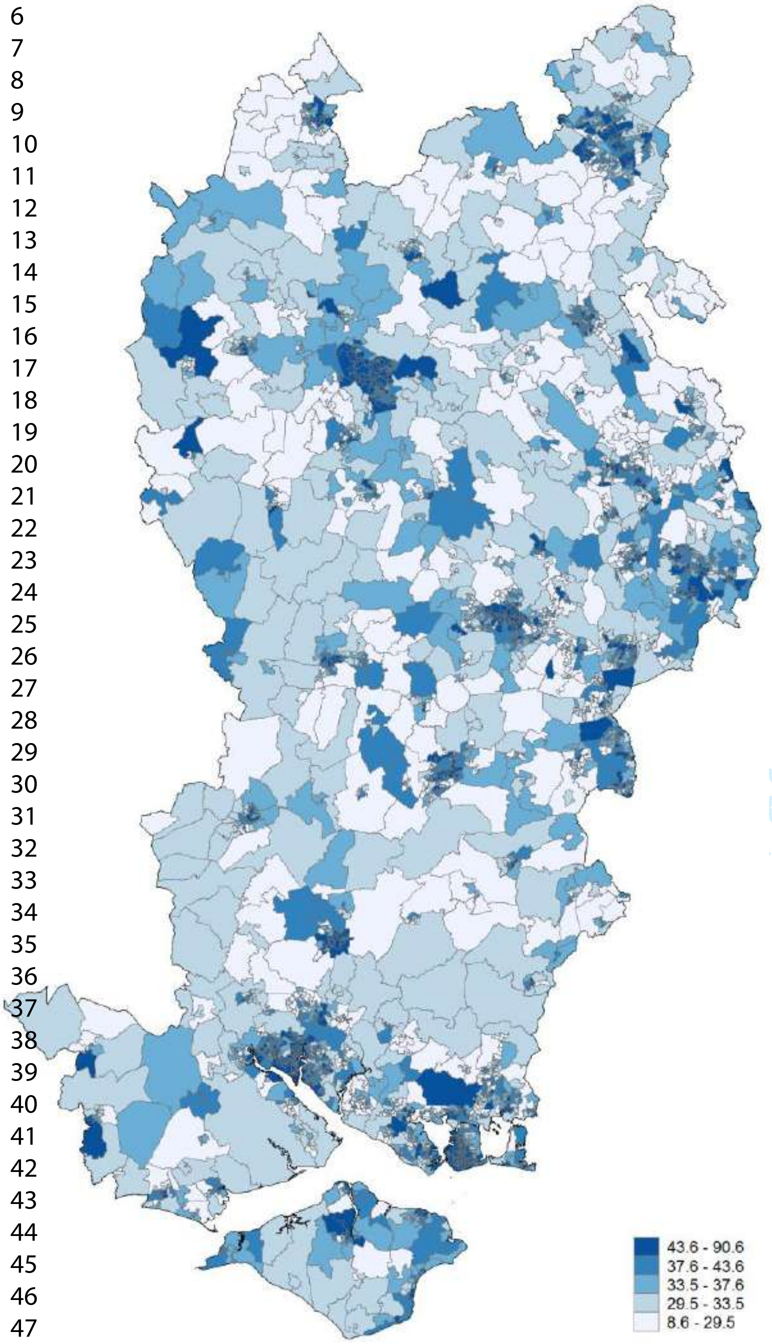

2011

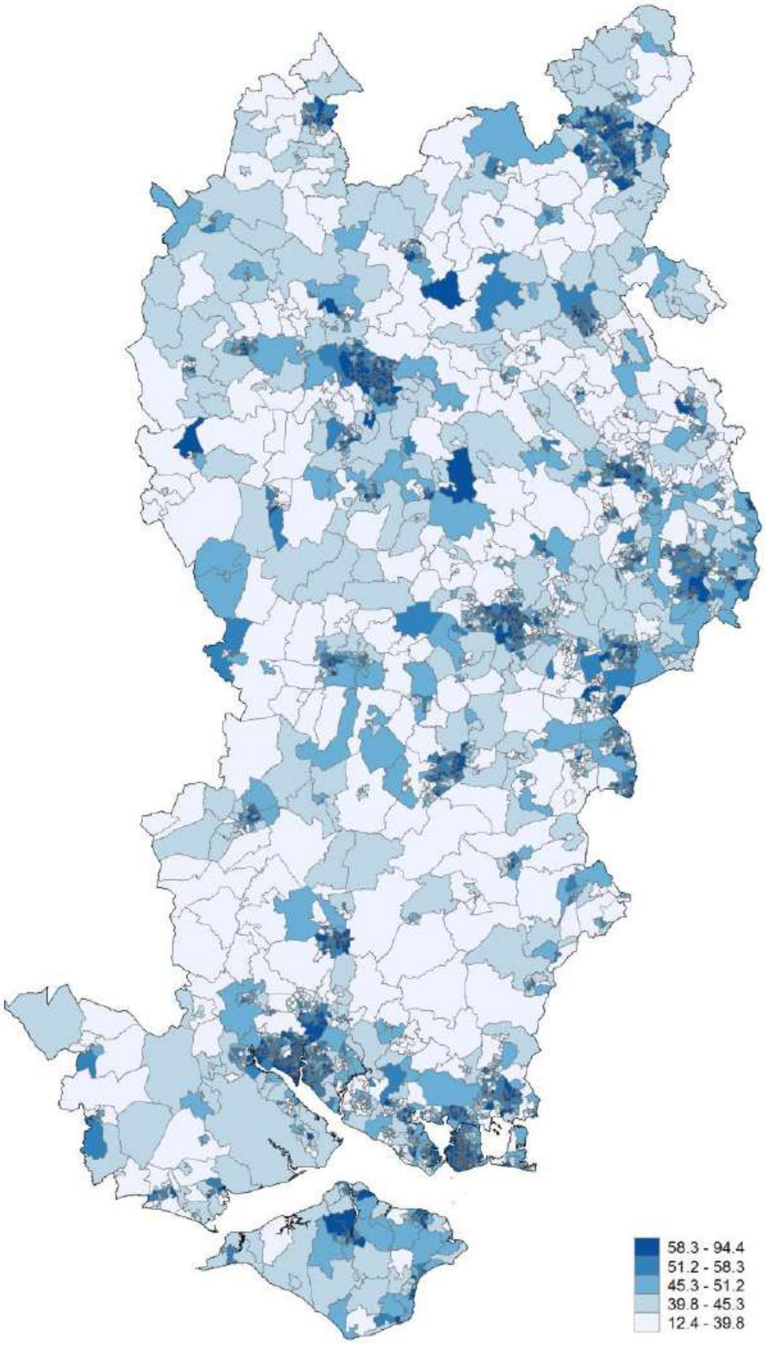

South West, Single People % of population

2001

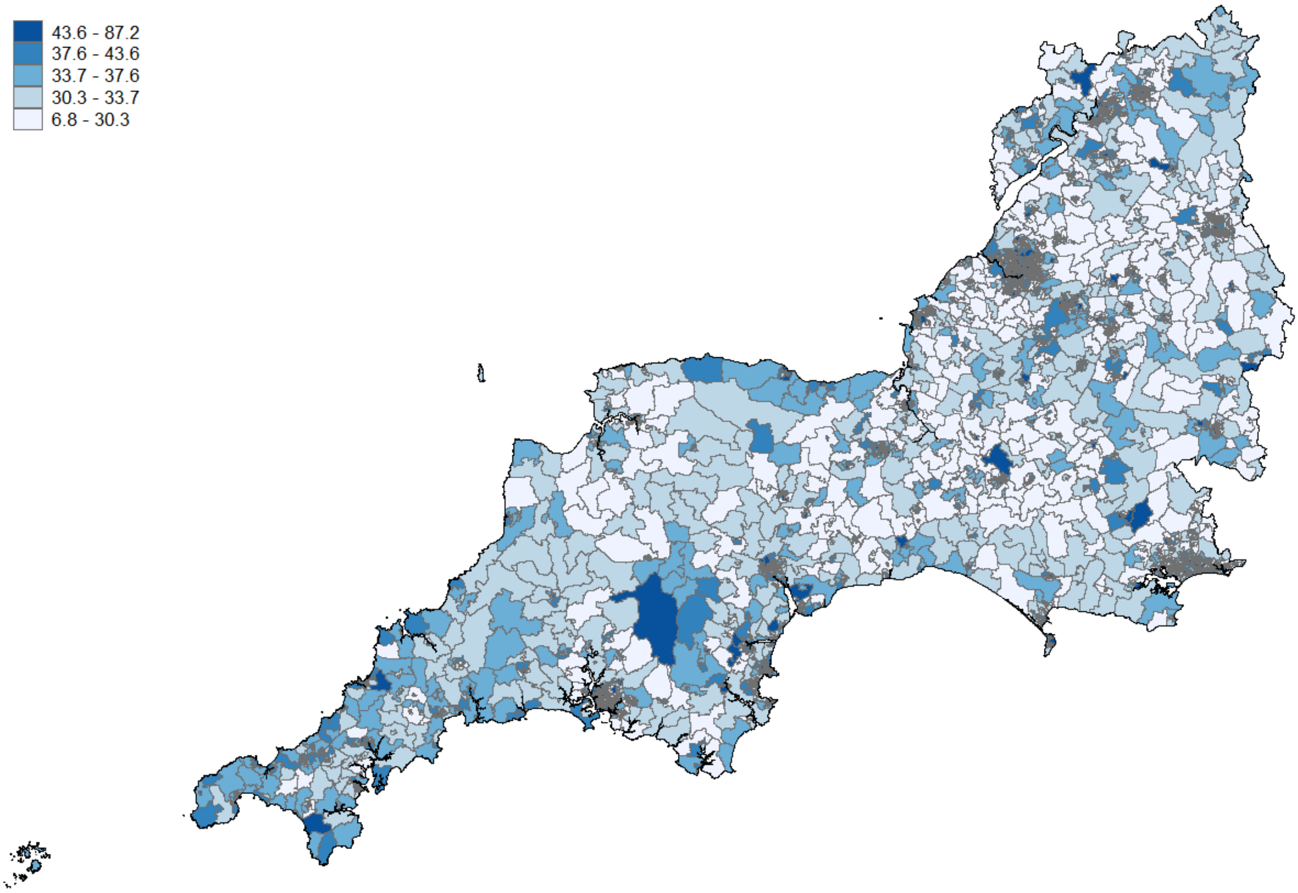

2011

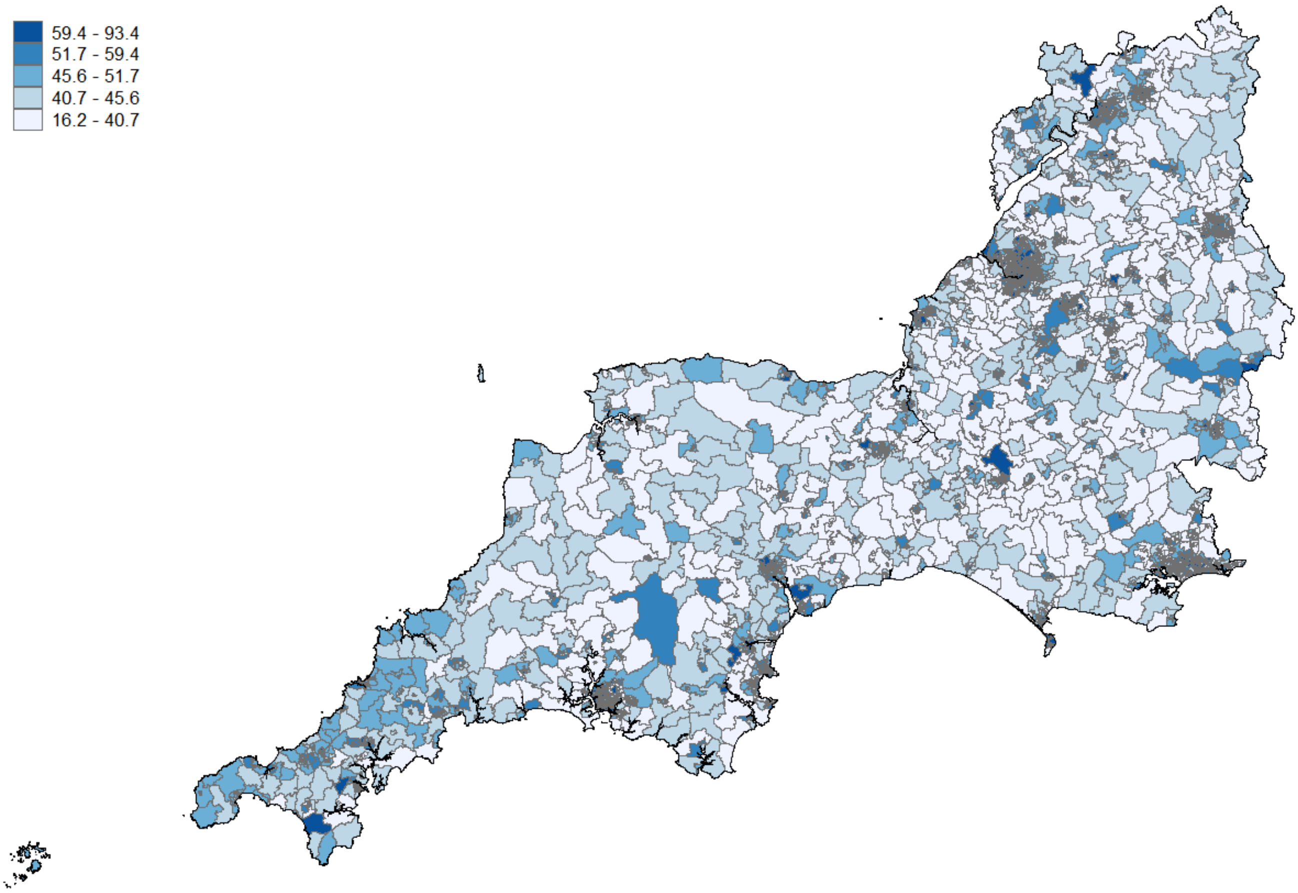

One Person Households

England 2011, One Person Households % of all households

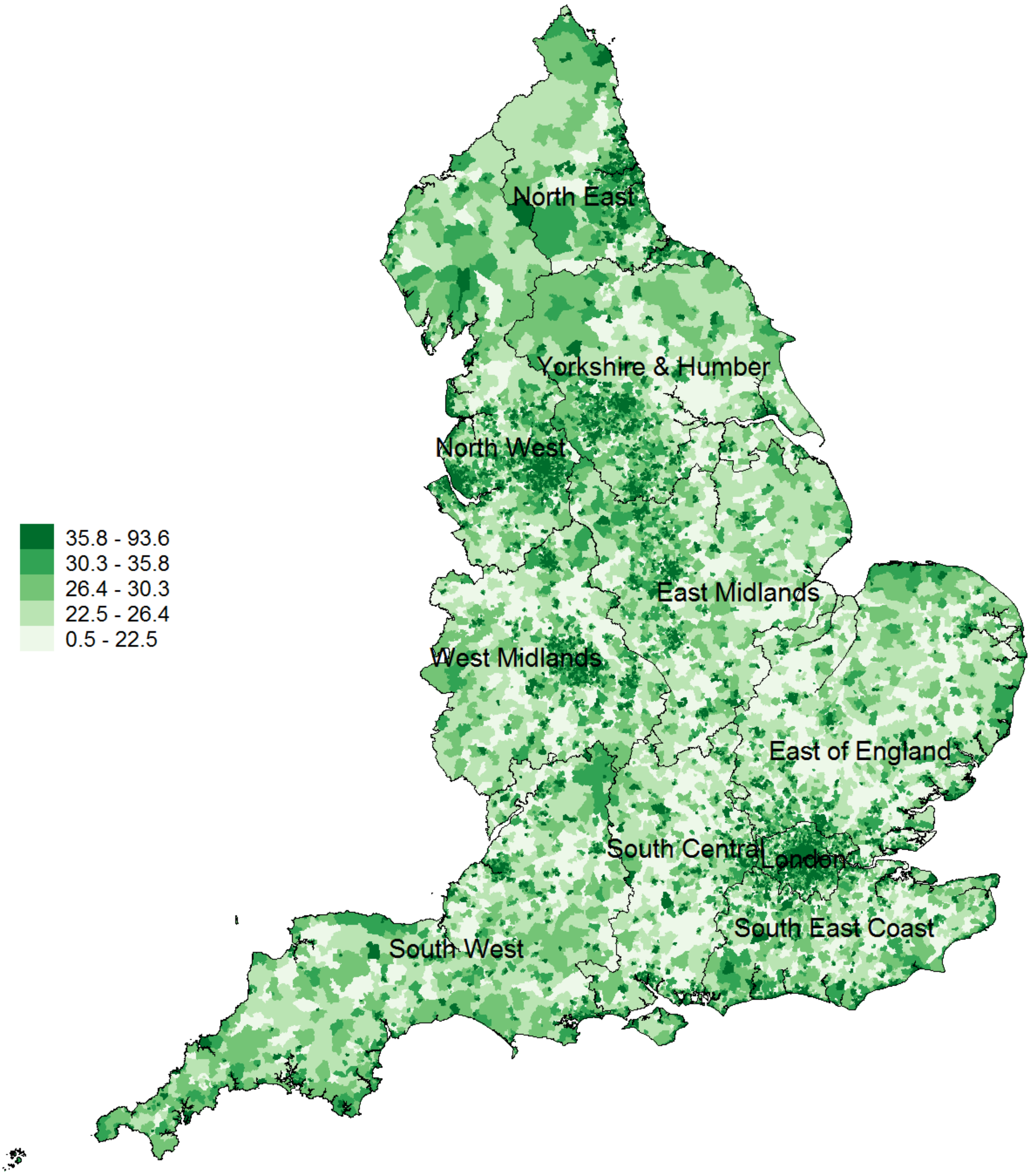

North East, One Person Households % of all households

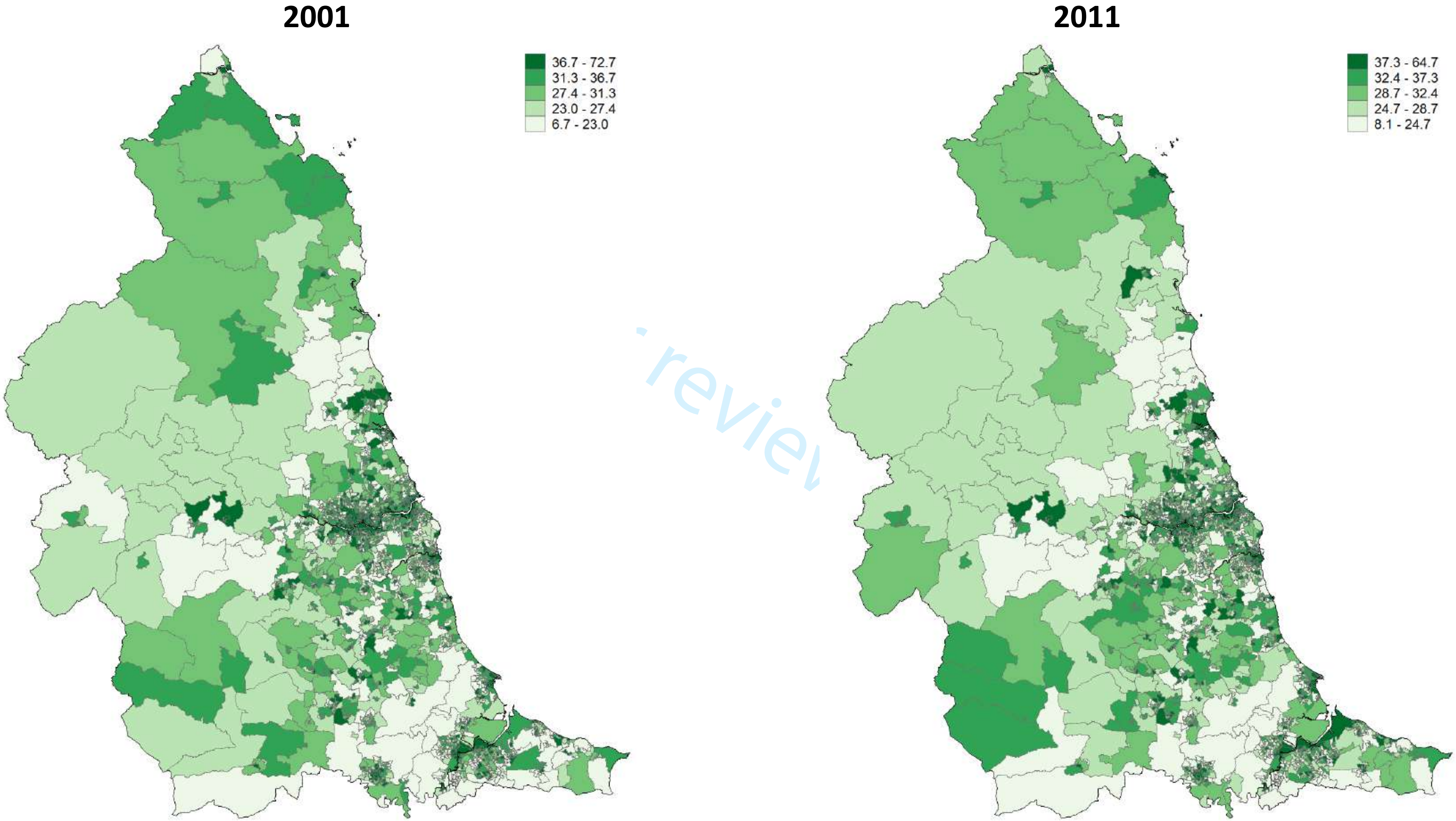

North East, One Person Households % of all households

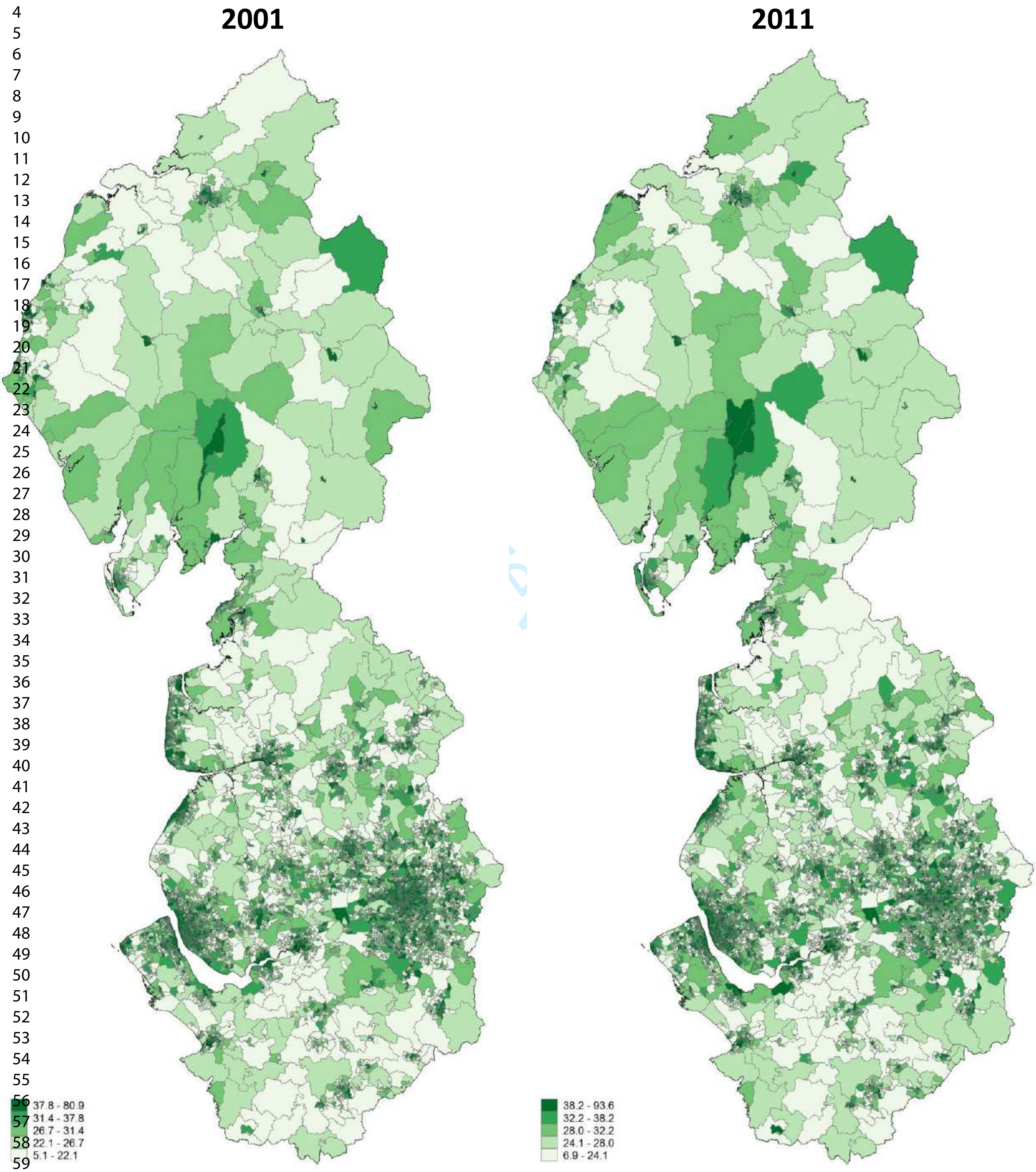

Yorkshire and the Humber, One Person Households % of all households

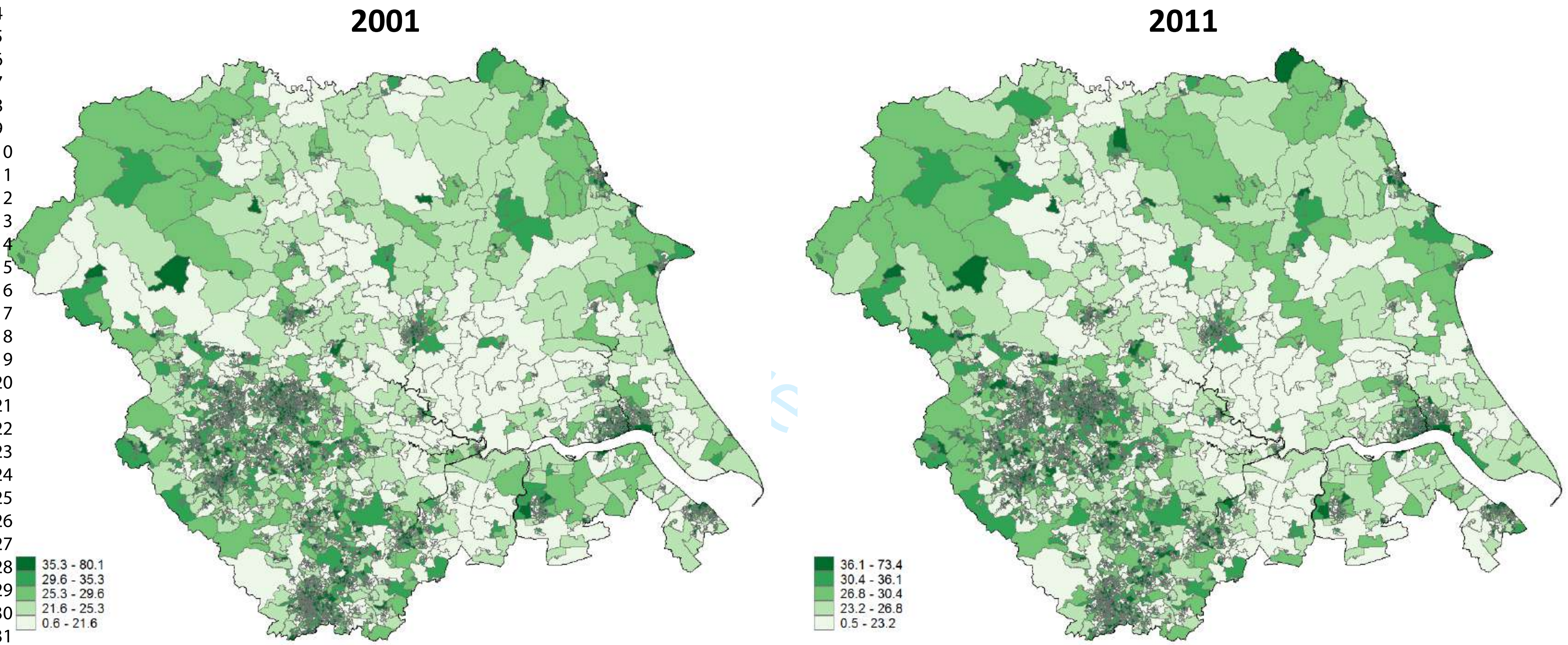

East Midlands, One Person Households % of all households

2001 **2011**

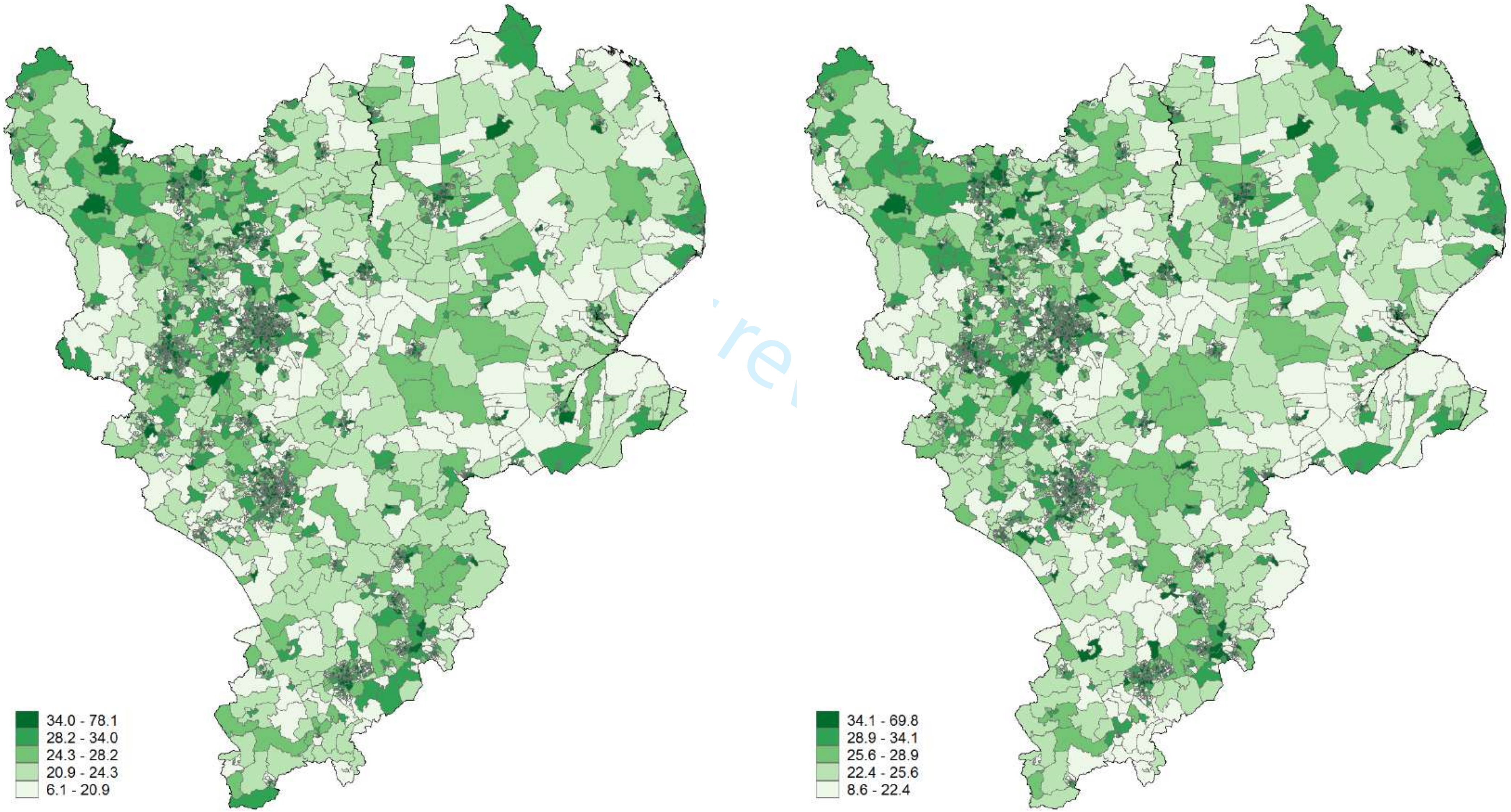

West Midlands, One Person Households % of all households

2001

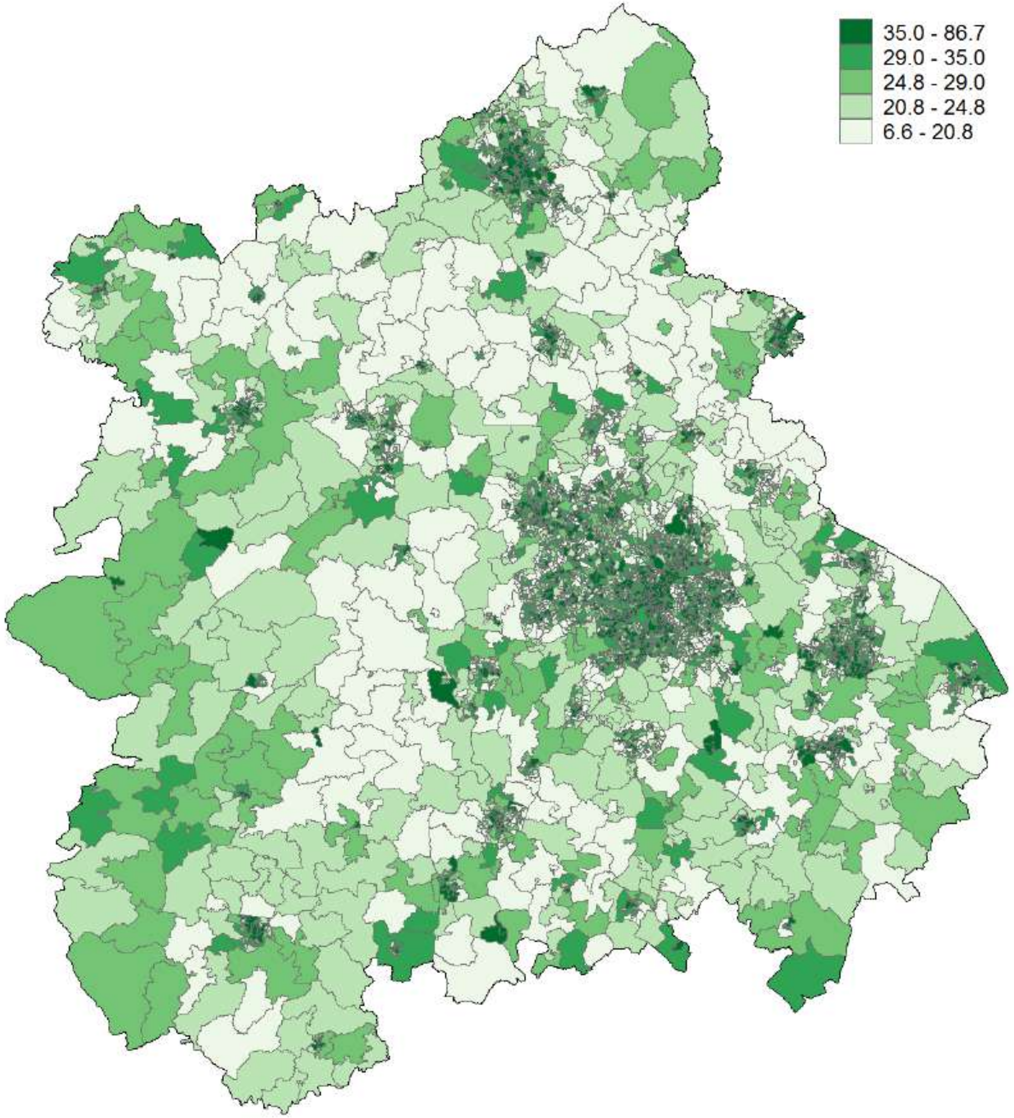

2011

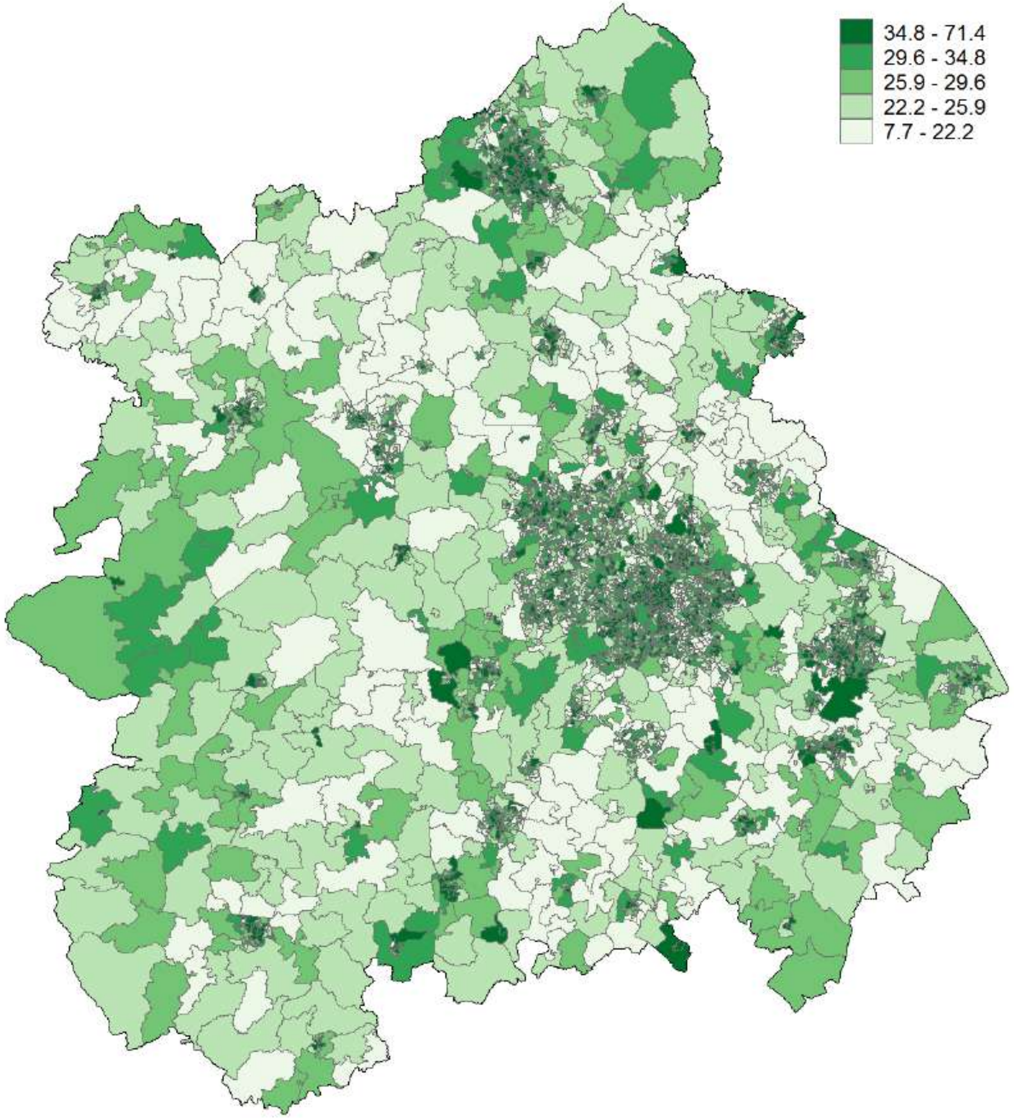

East of England, One Person Households % of all households

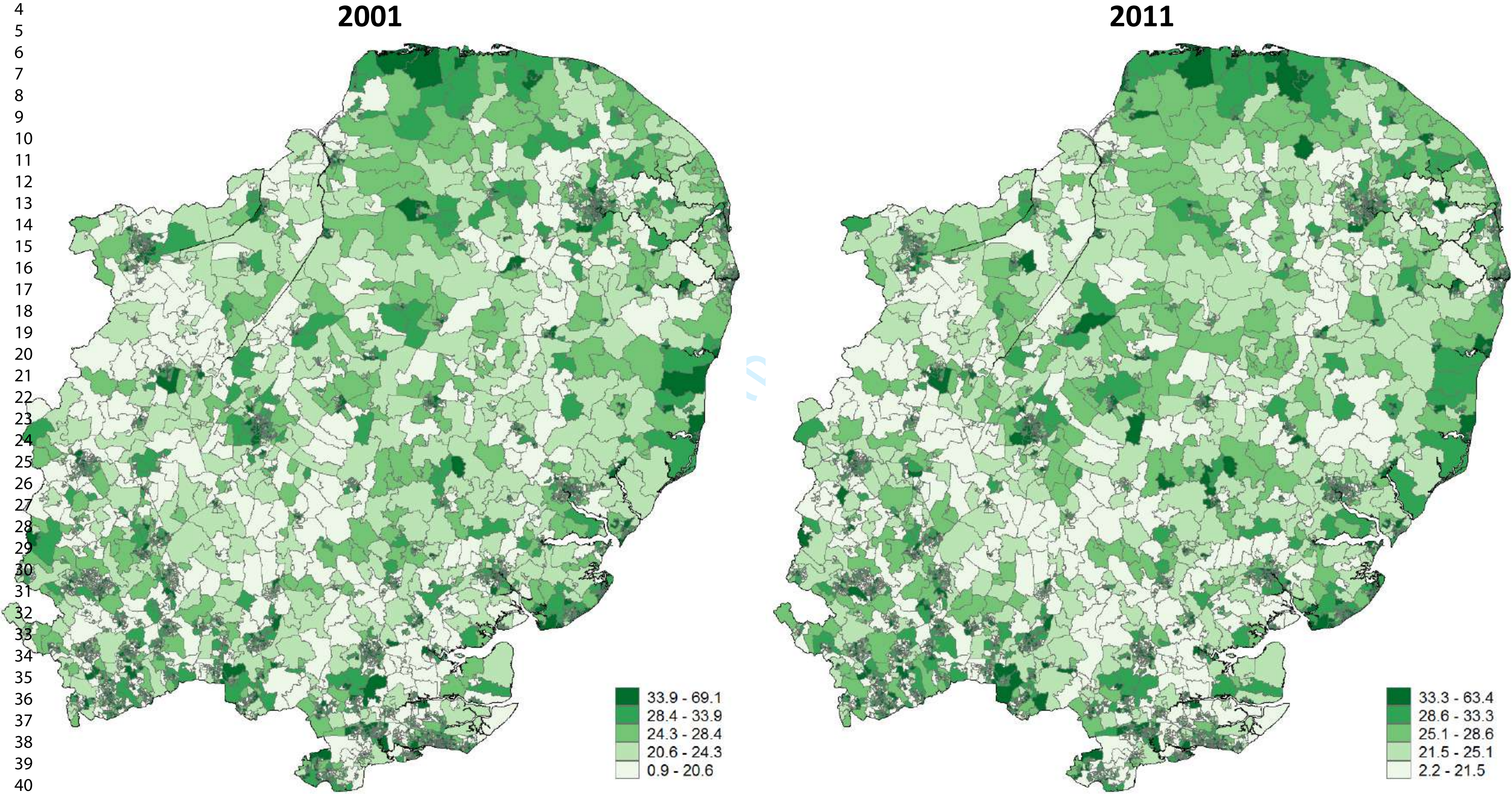

London, One Person Households % of all households

2001

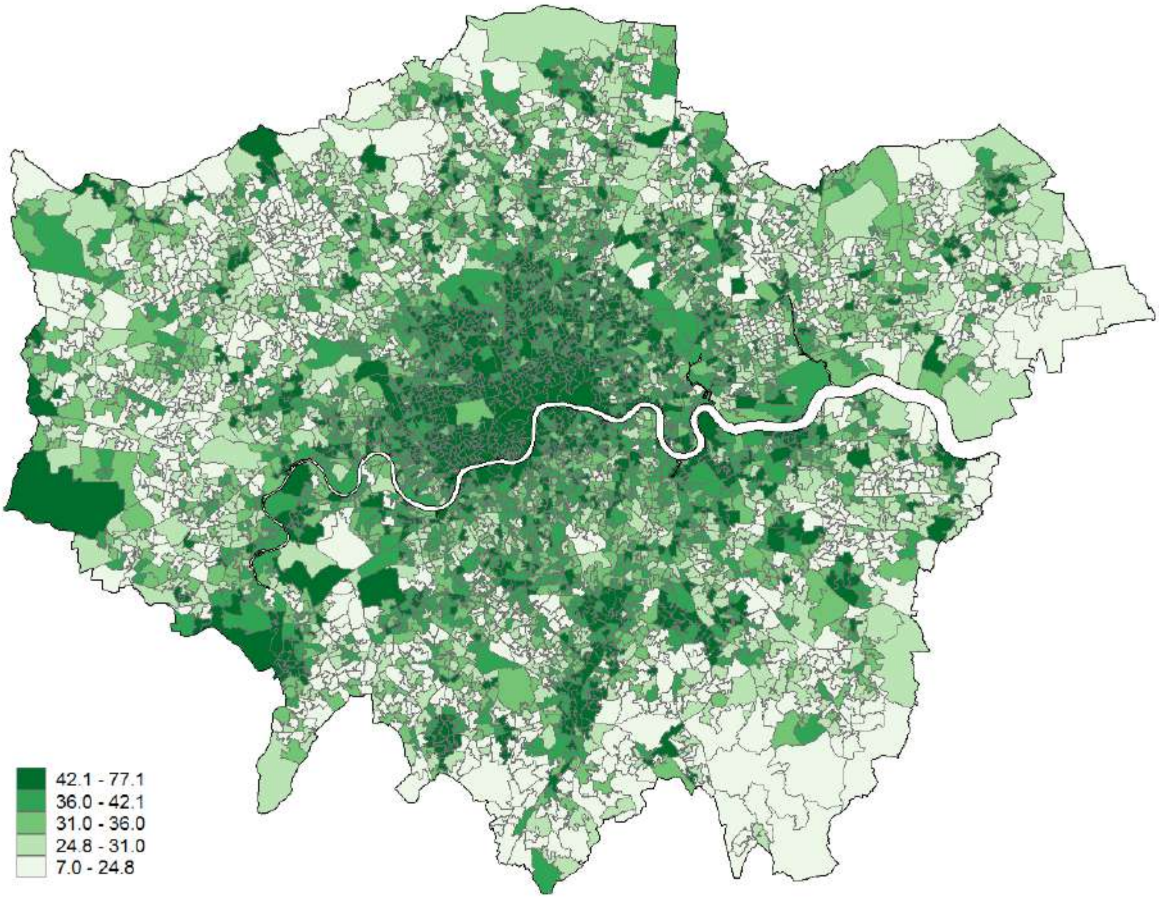

2011

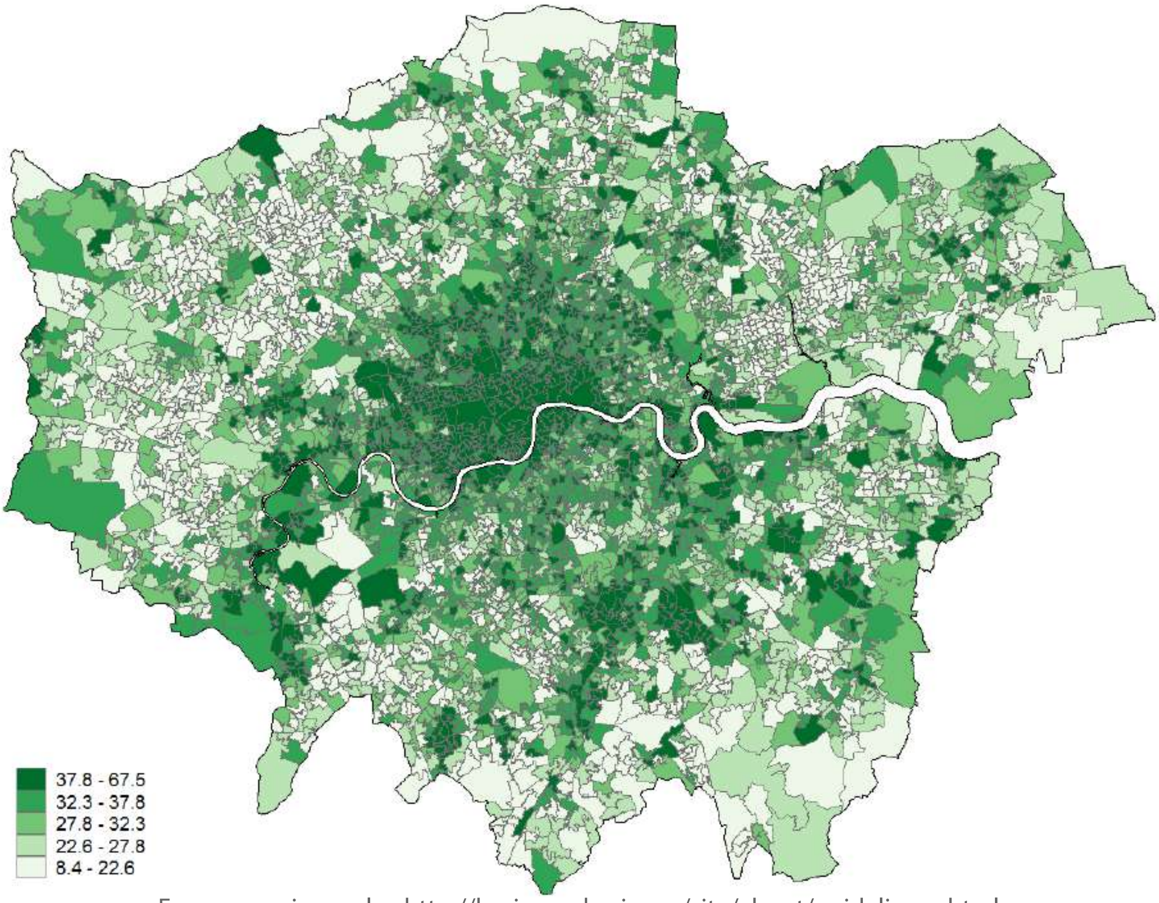

South East Coast, One Person Households % of all households

2001

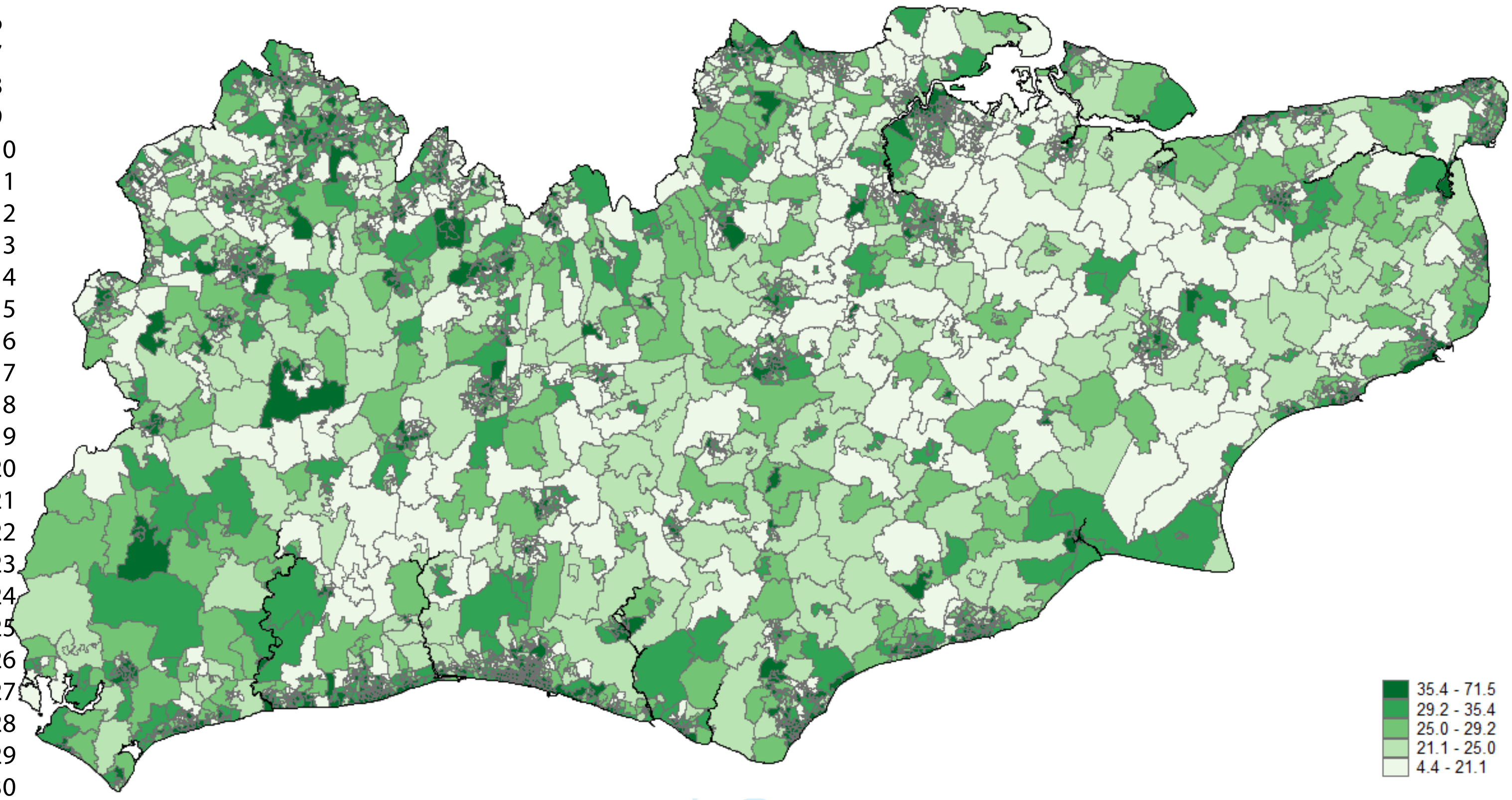

2011

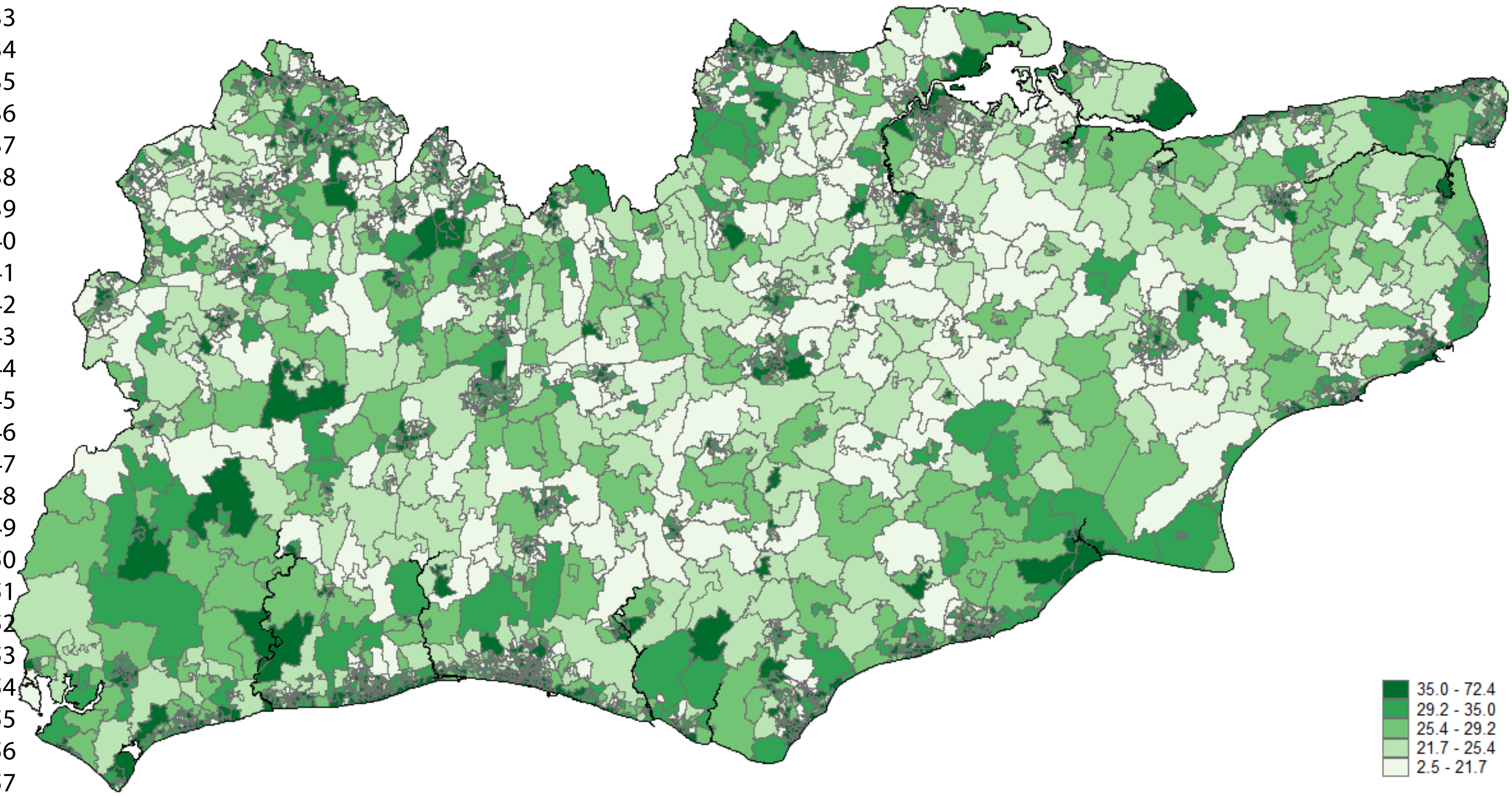

South Central, One Person Households % of all households

2001

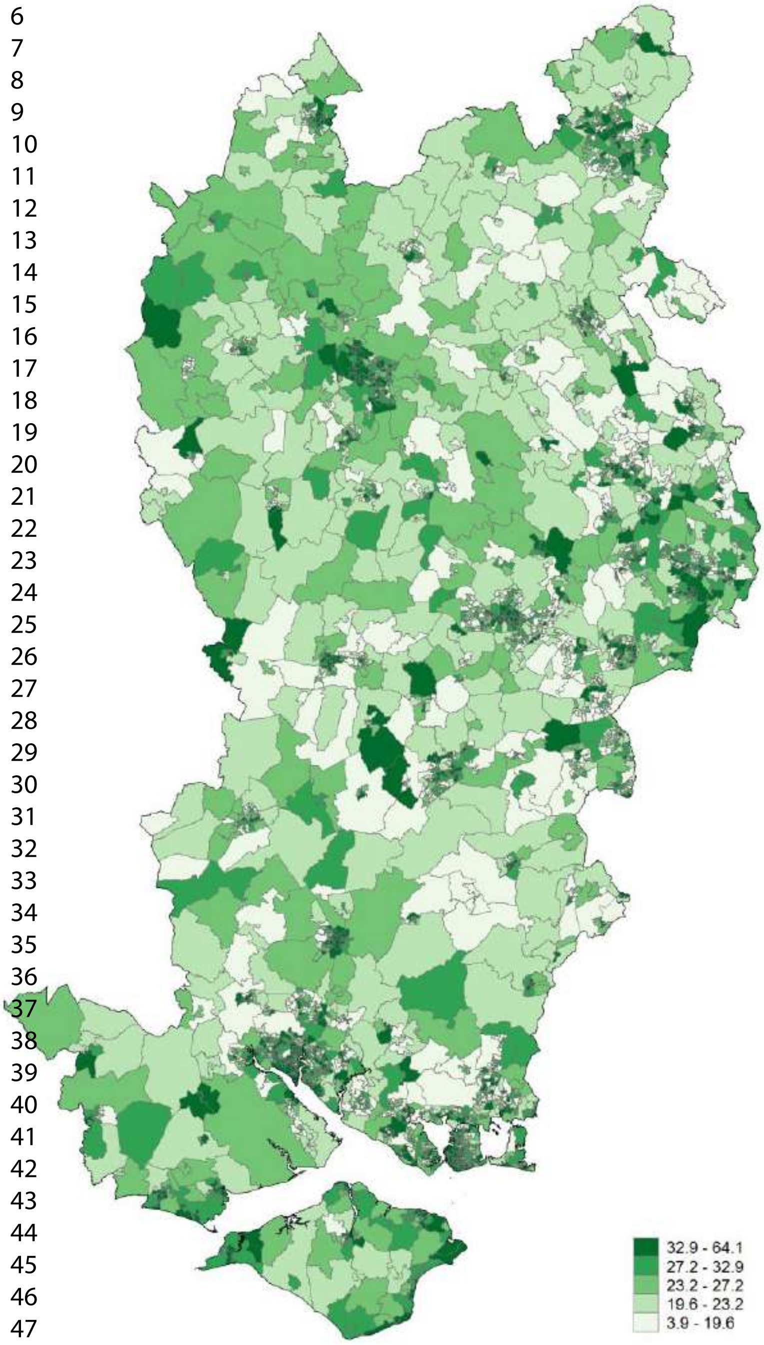

2011

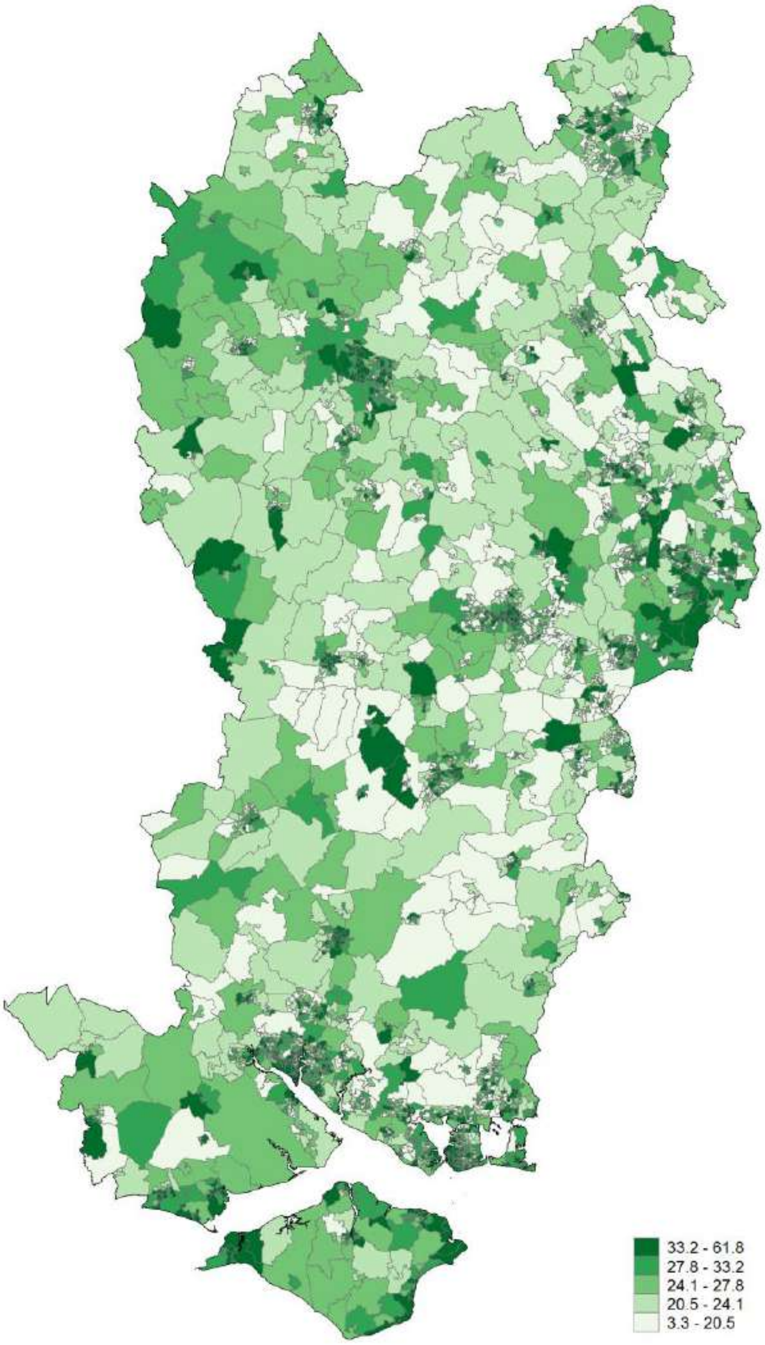

South West, One Person Households % of all households

2001

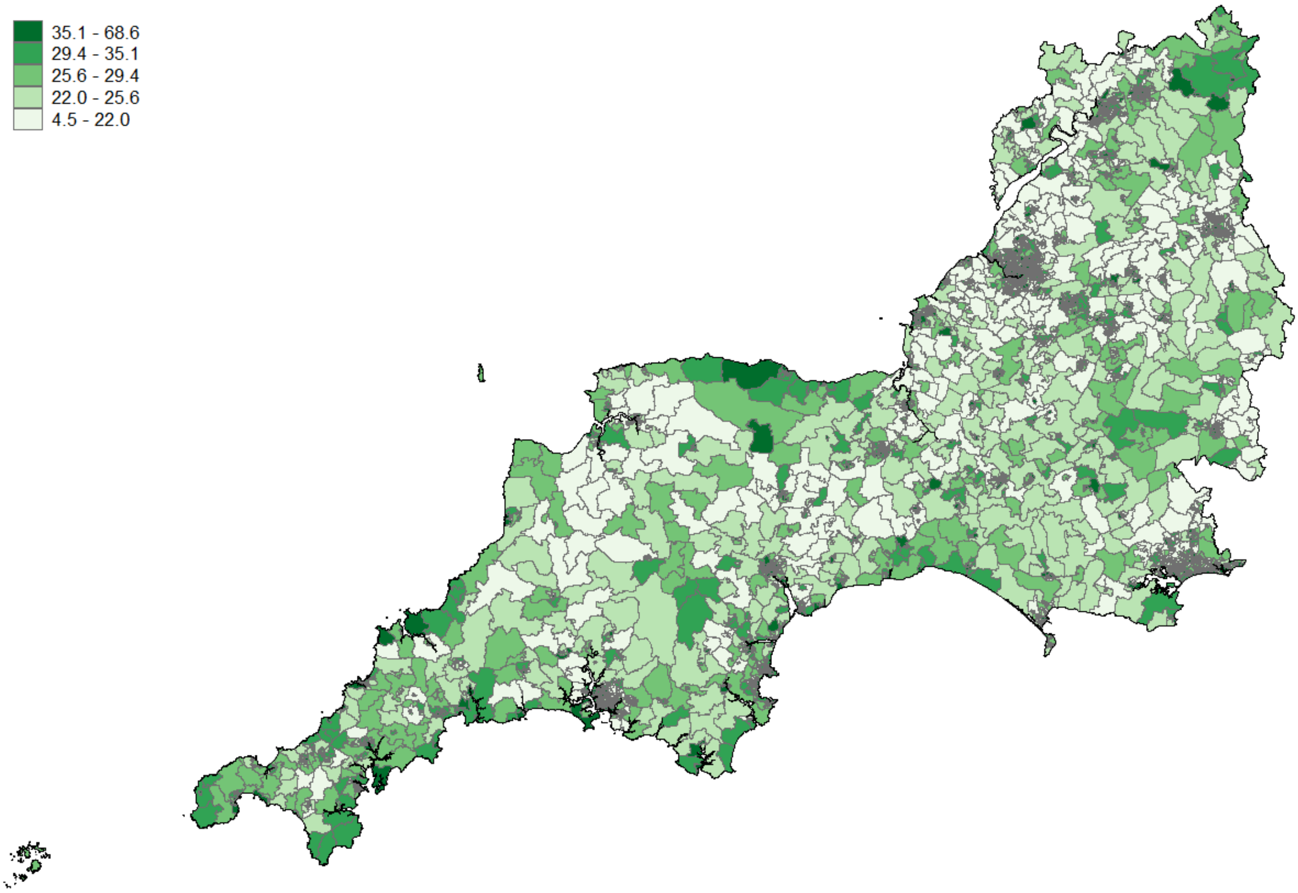

2011

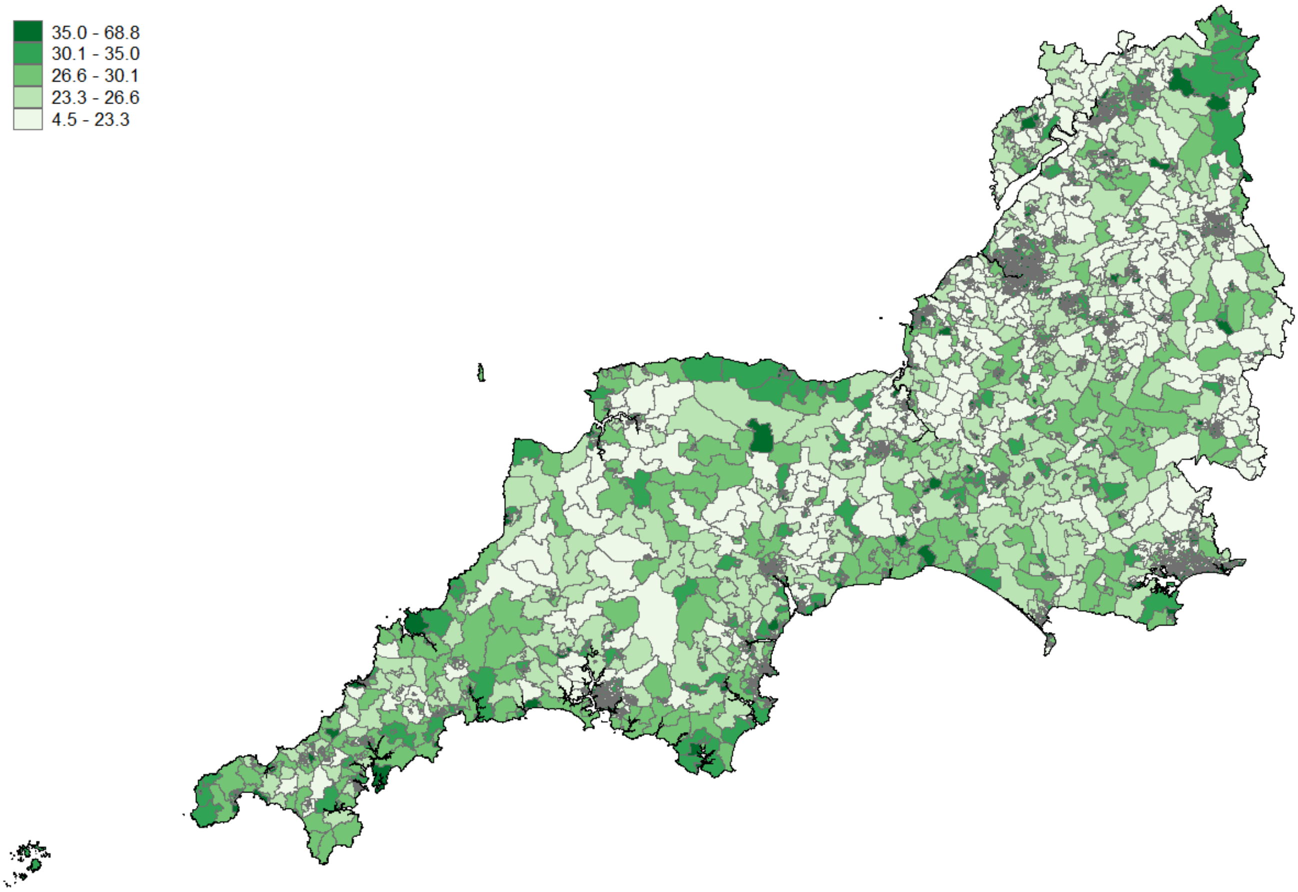

Private Renting

England 2011, Private Renting % of all households

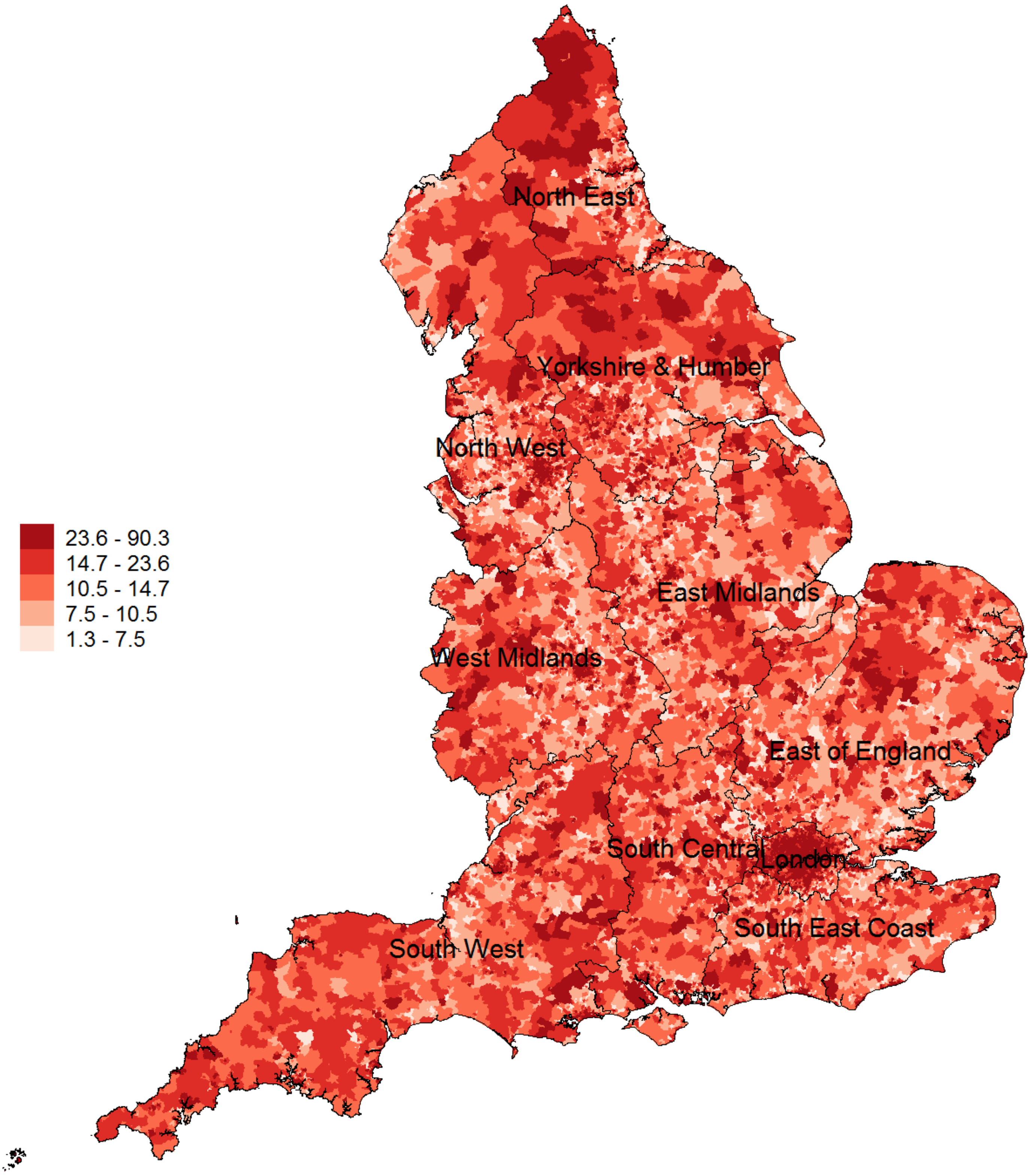

North East, Private Renting % of all households

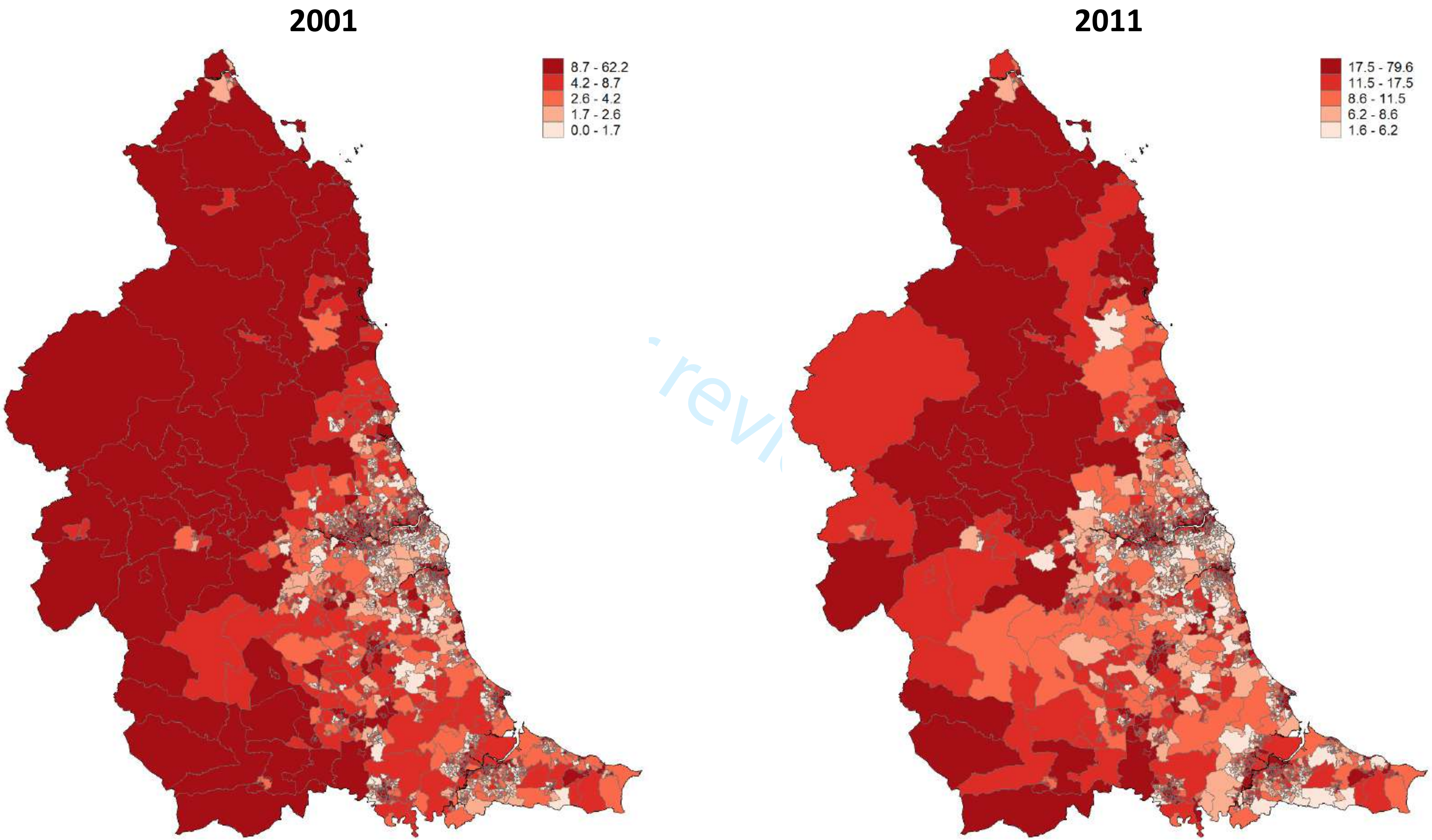

North East, Private Renting % of all households

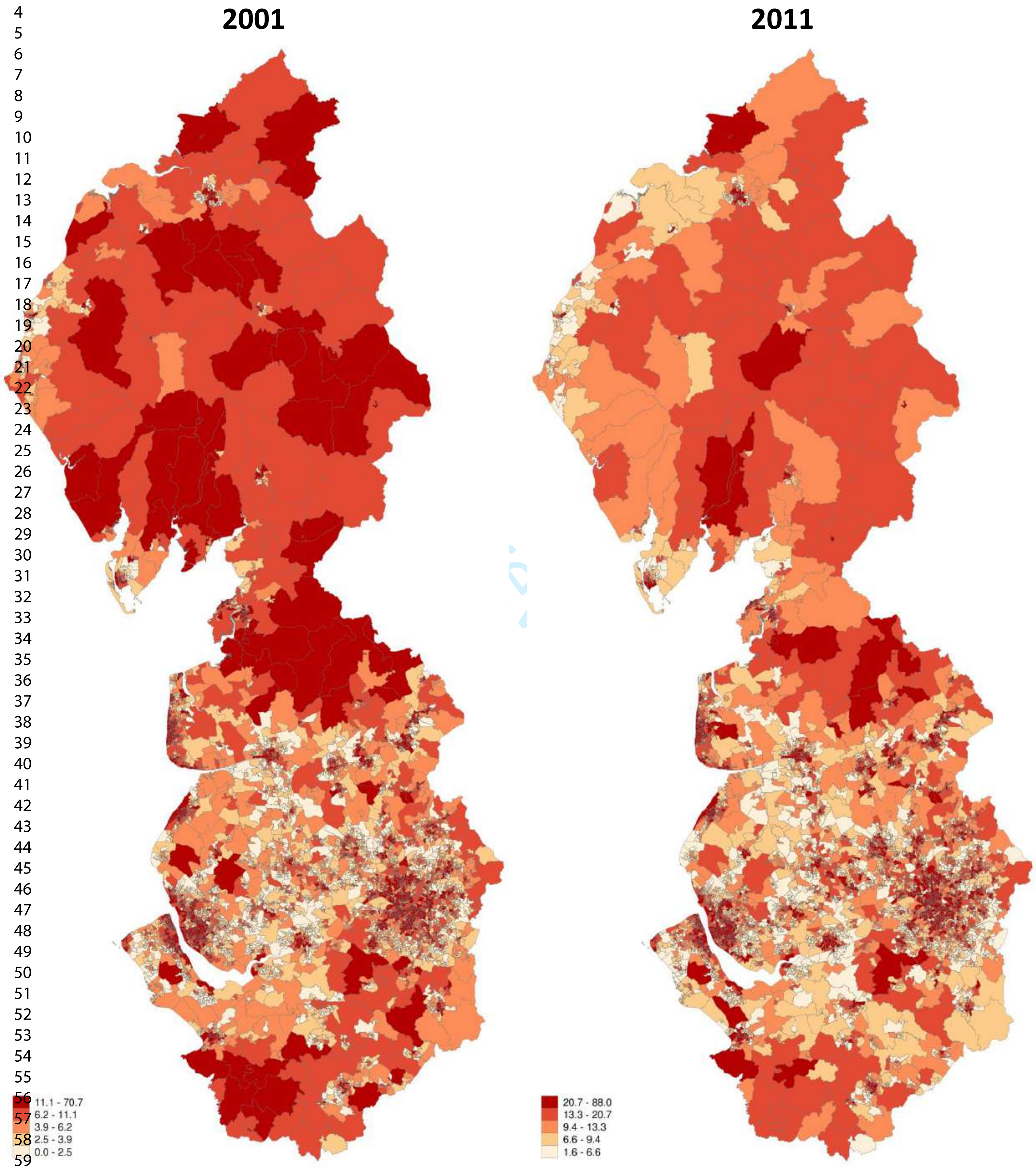

Yorkshire and the Humber, Private Renting % of all households

2001 / 2011

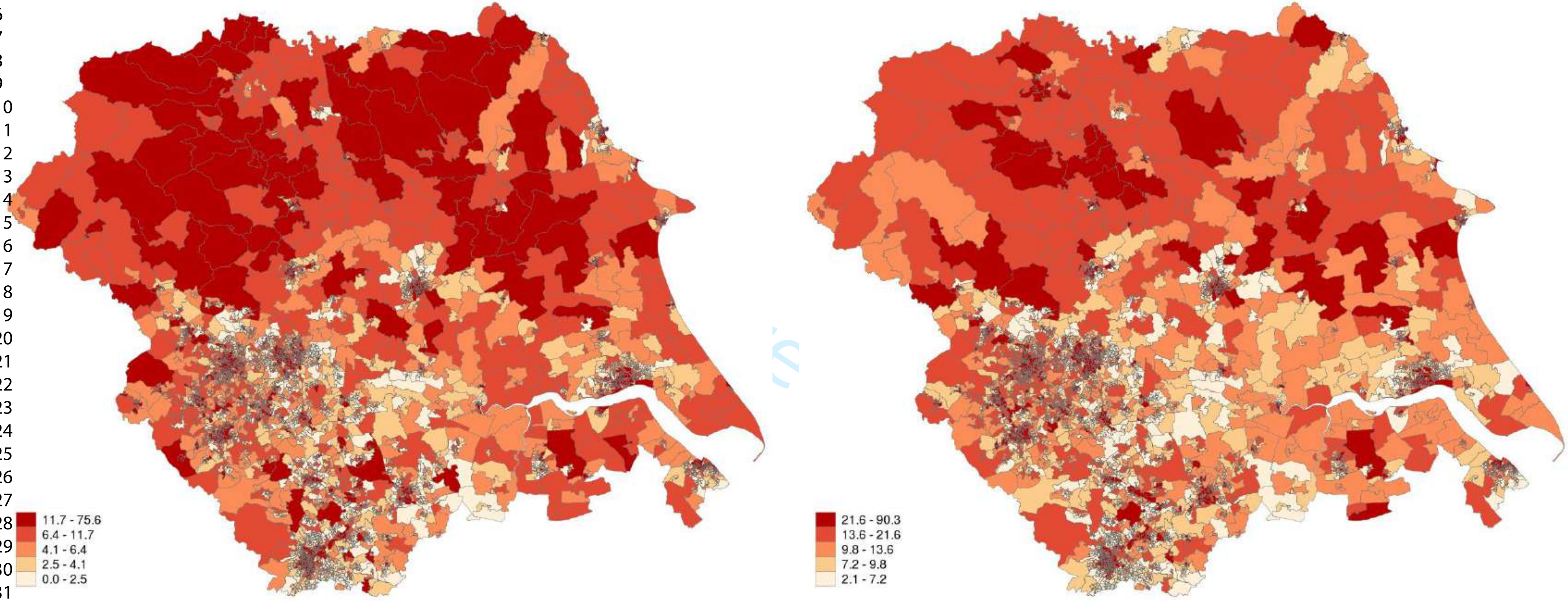

East Midlands, Private Renting % of all households

2001

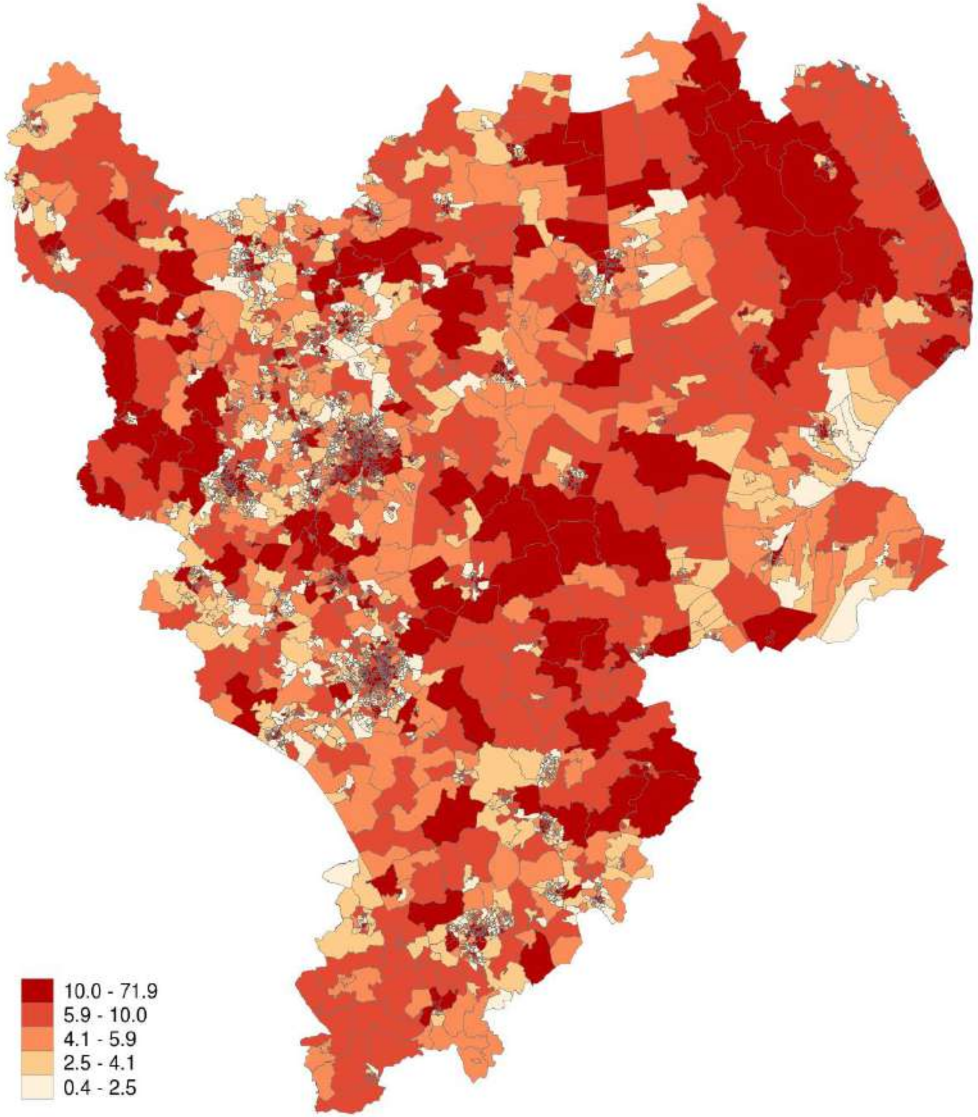

2011

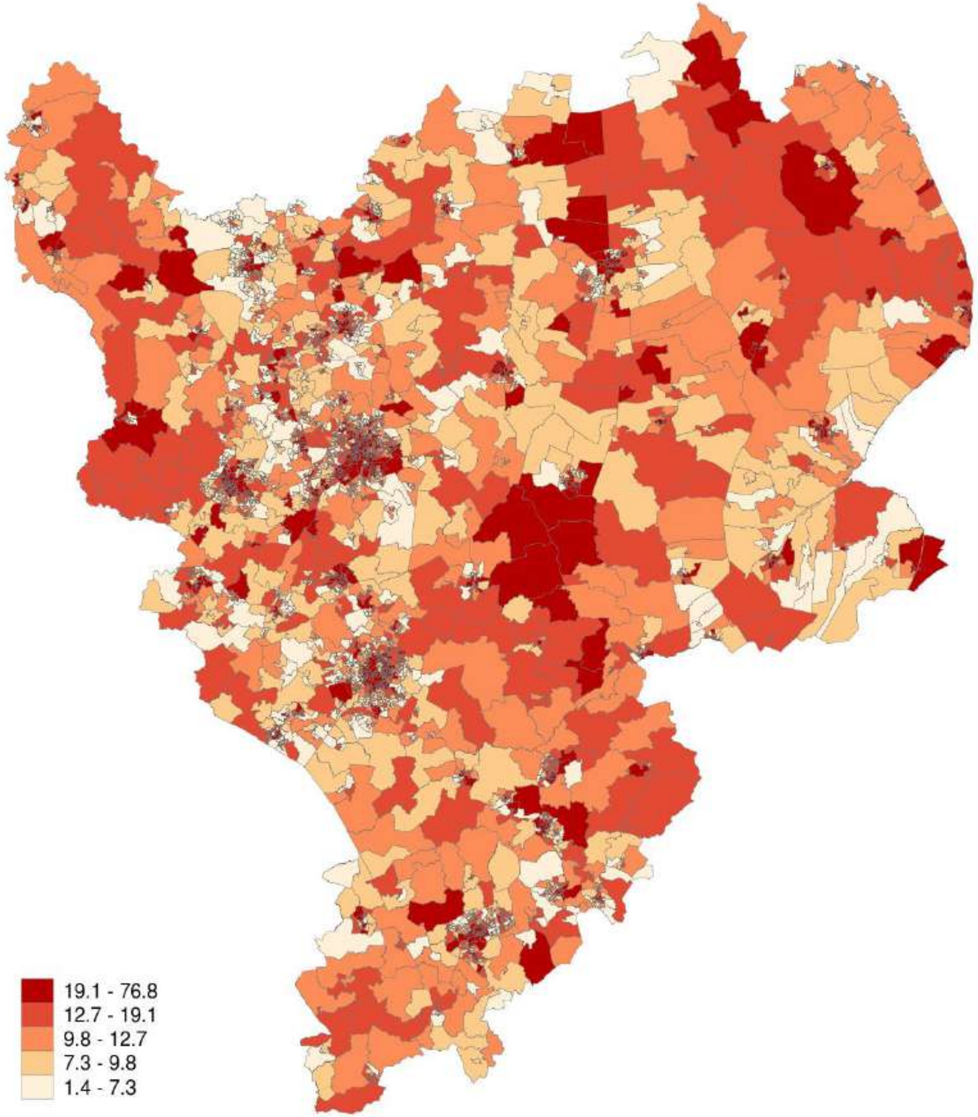

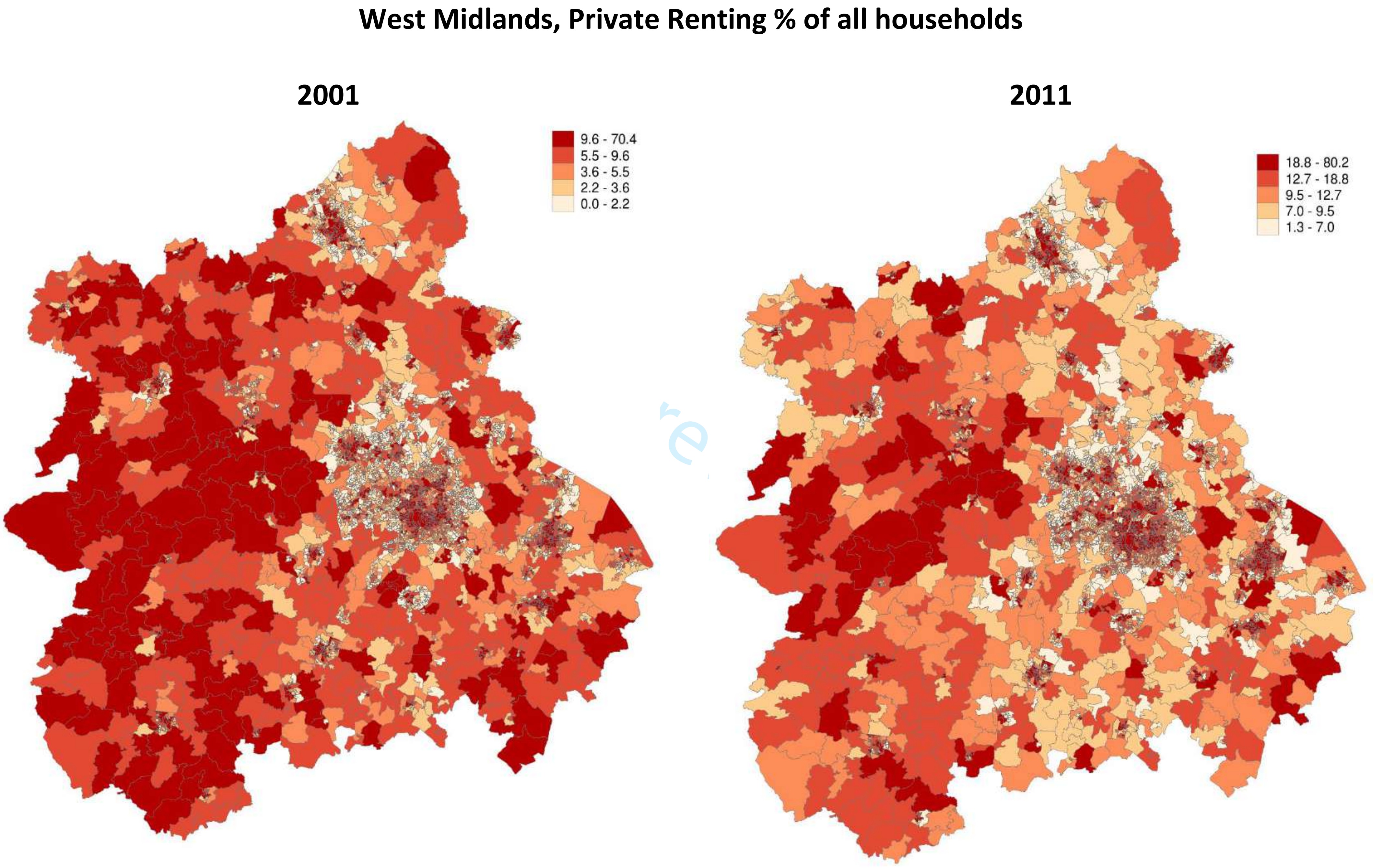
West Midlands, Private Renting % of all households
2001
9.6 - 70.4
5.5 - 9.6
3.6 - 5.5
2.2 - 3.6
0.0 - 2.2
2011
18.8 - 80.2
12.7 - 18.8
9.5 - 12.7
7.0 - 9.5
1.3 - 7.0

East of England, Private Renting % of all households

2001 **2011**

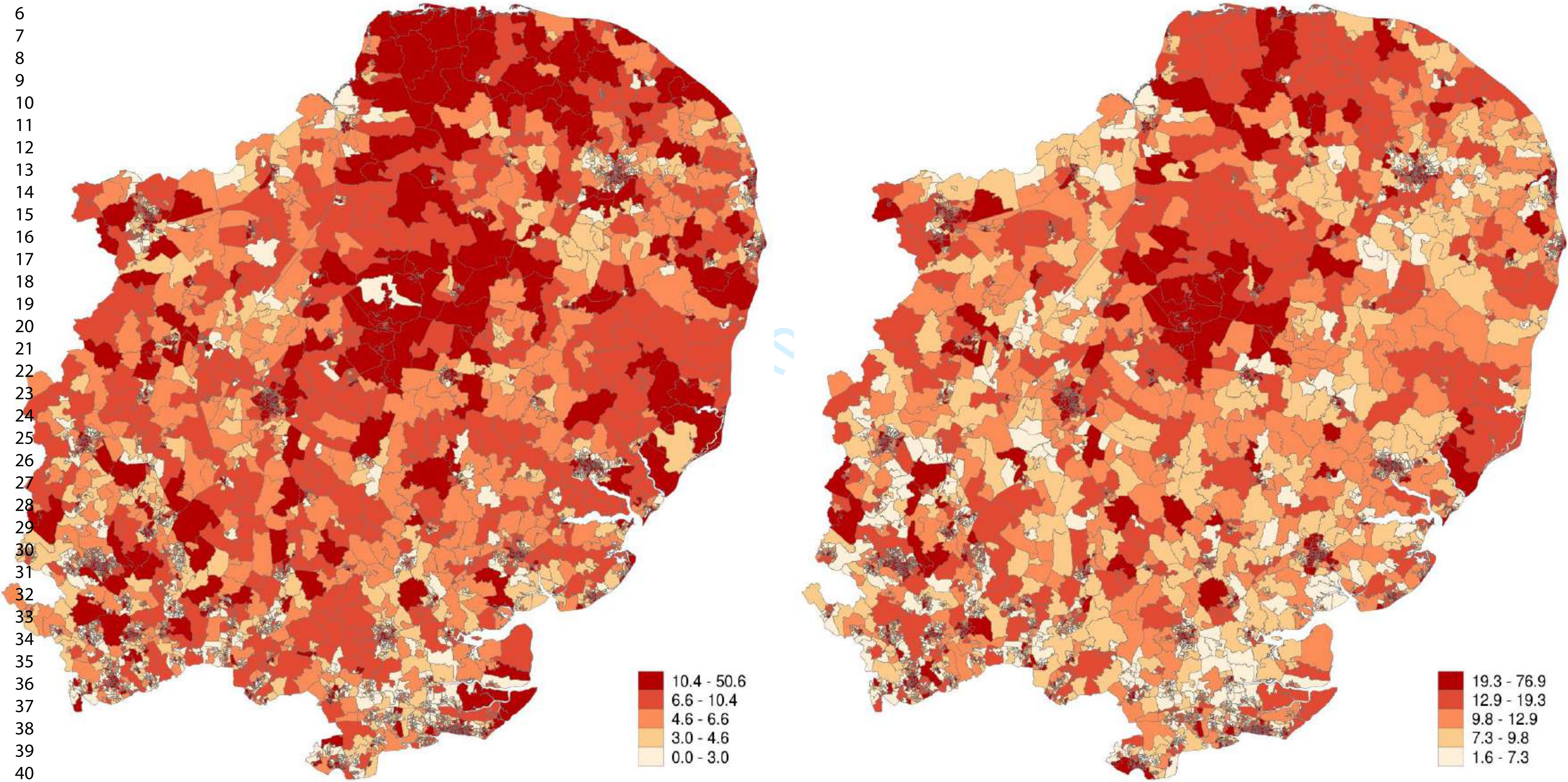

London, Private Renting % of all households

2001

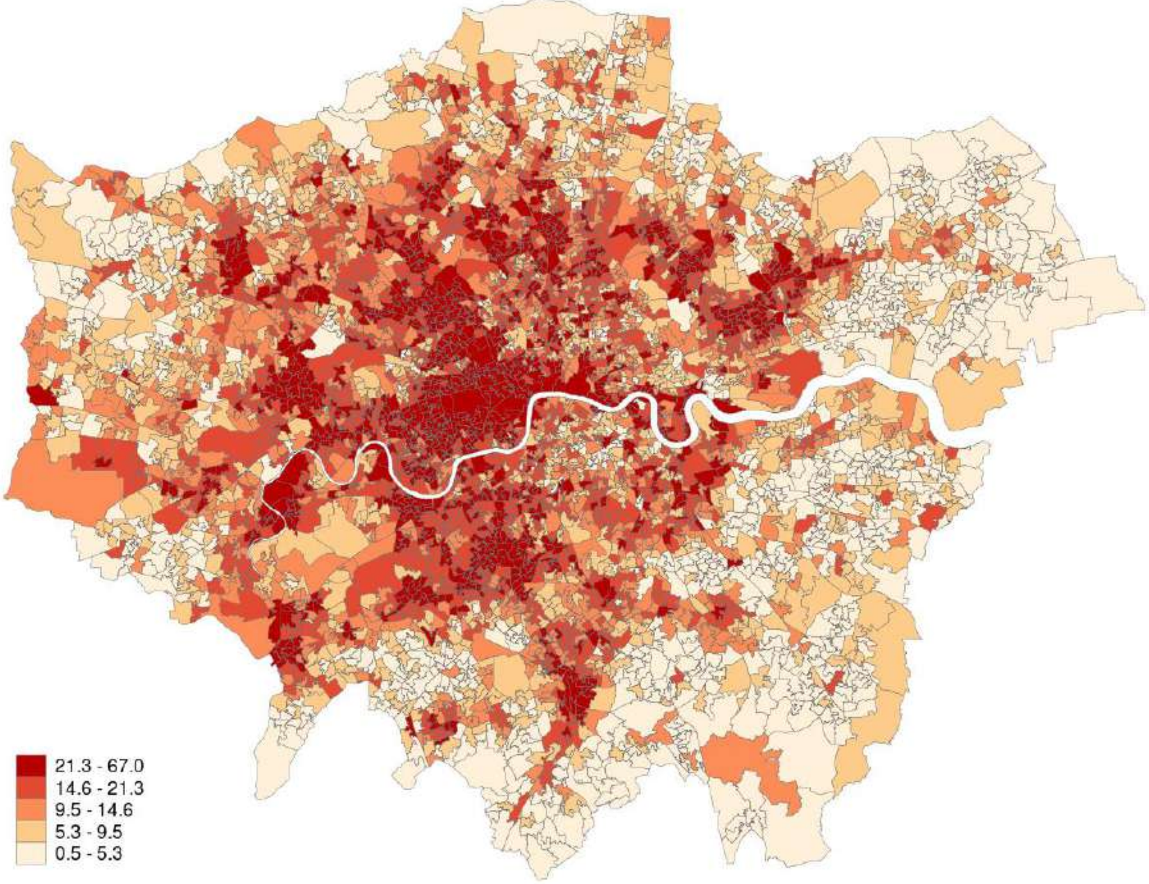

2011

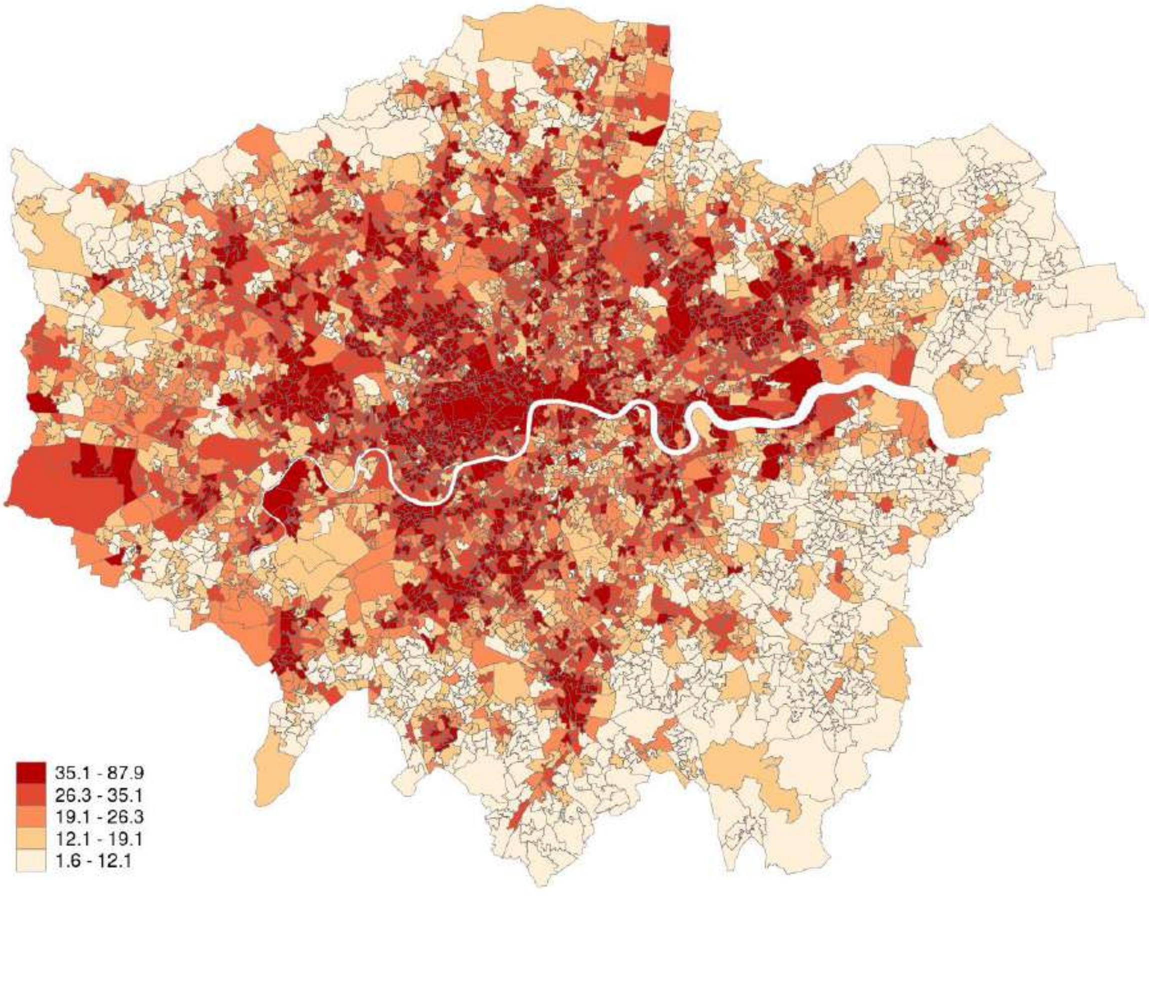

South East Coast, Private Renting % of all households

2001

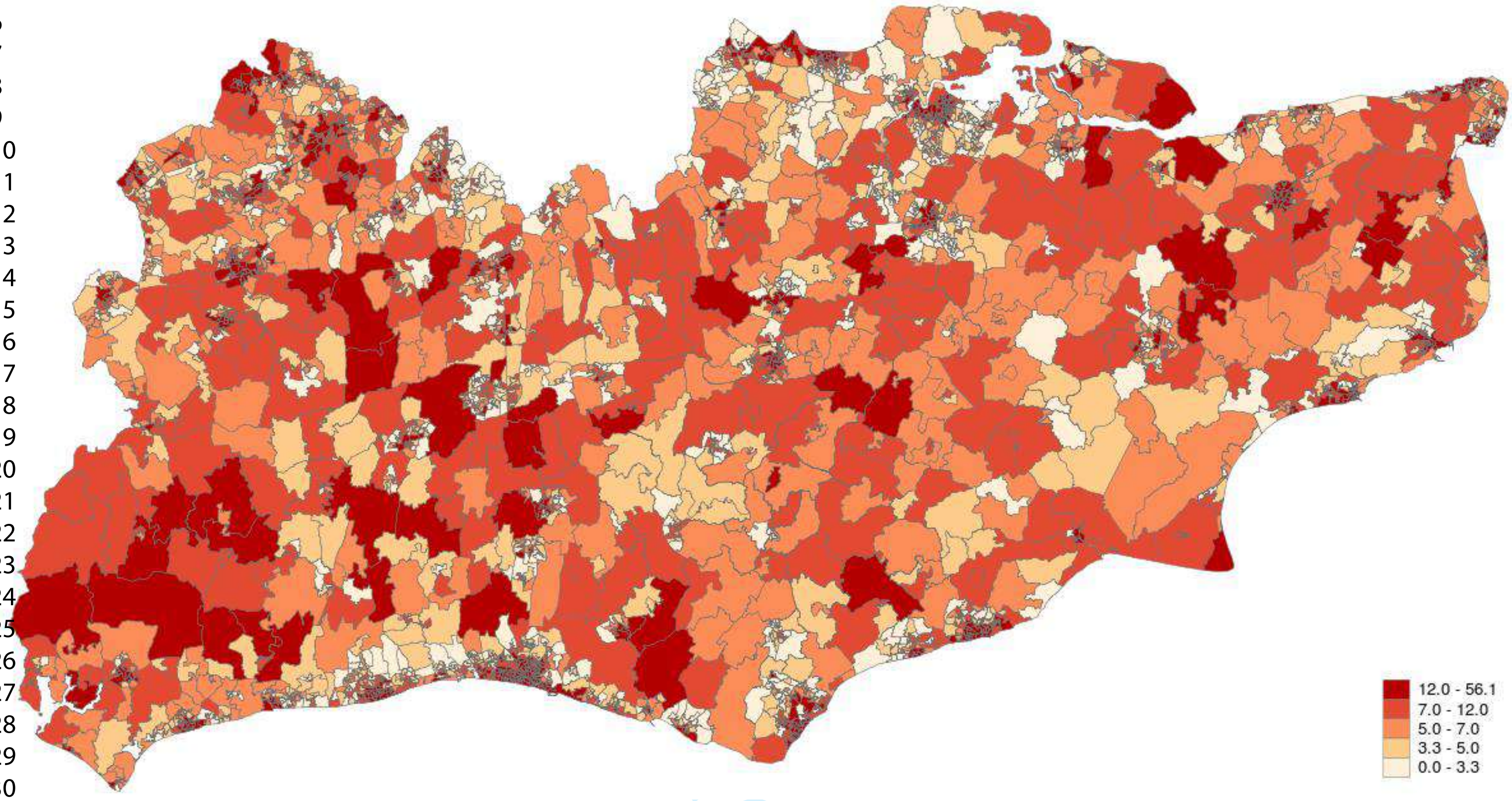

2011

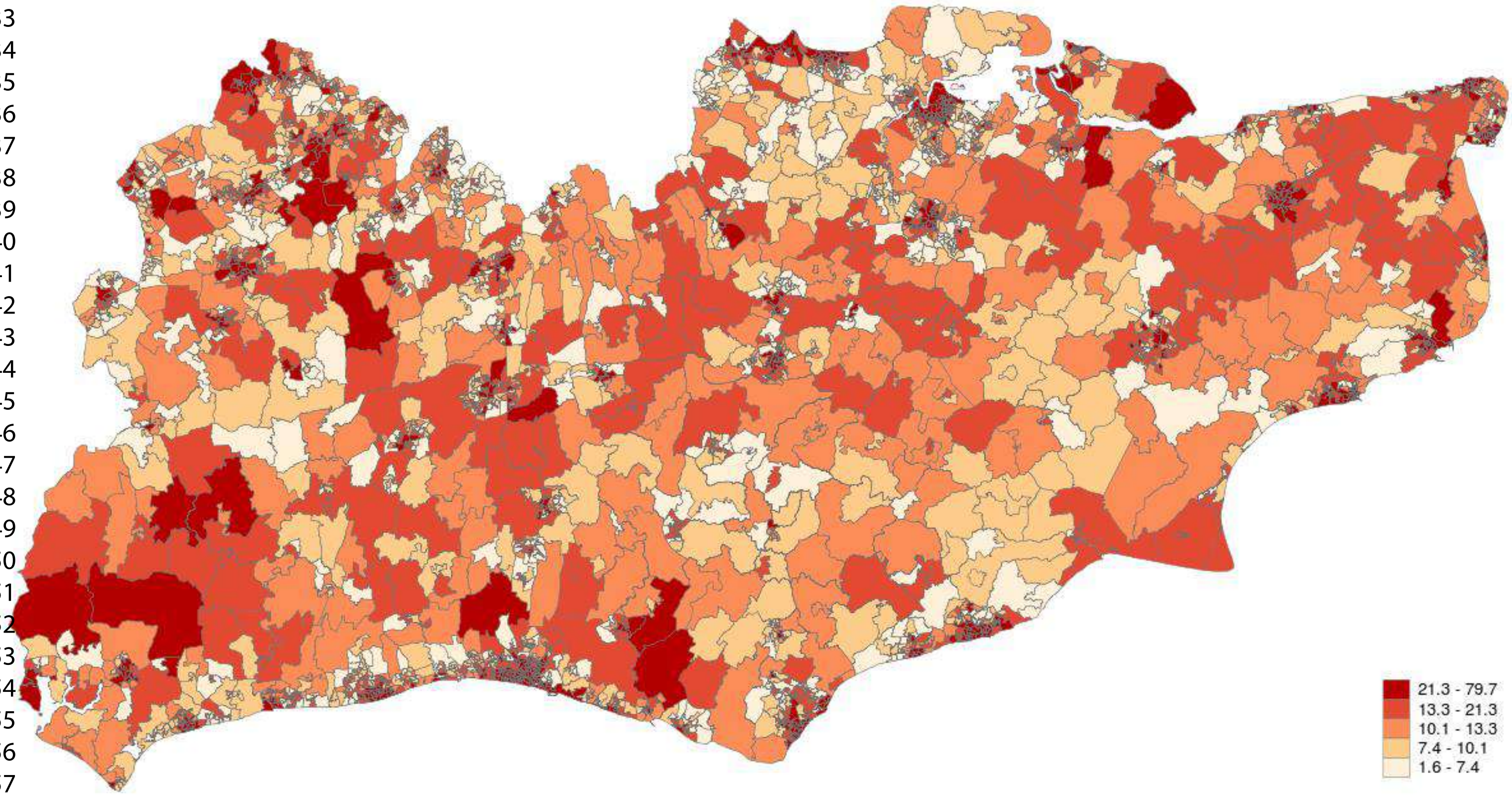

South Central, Private Renting % of all households

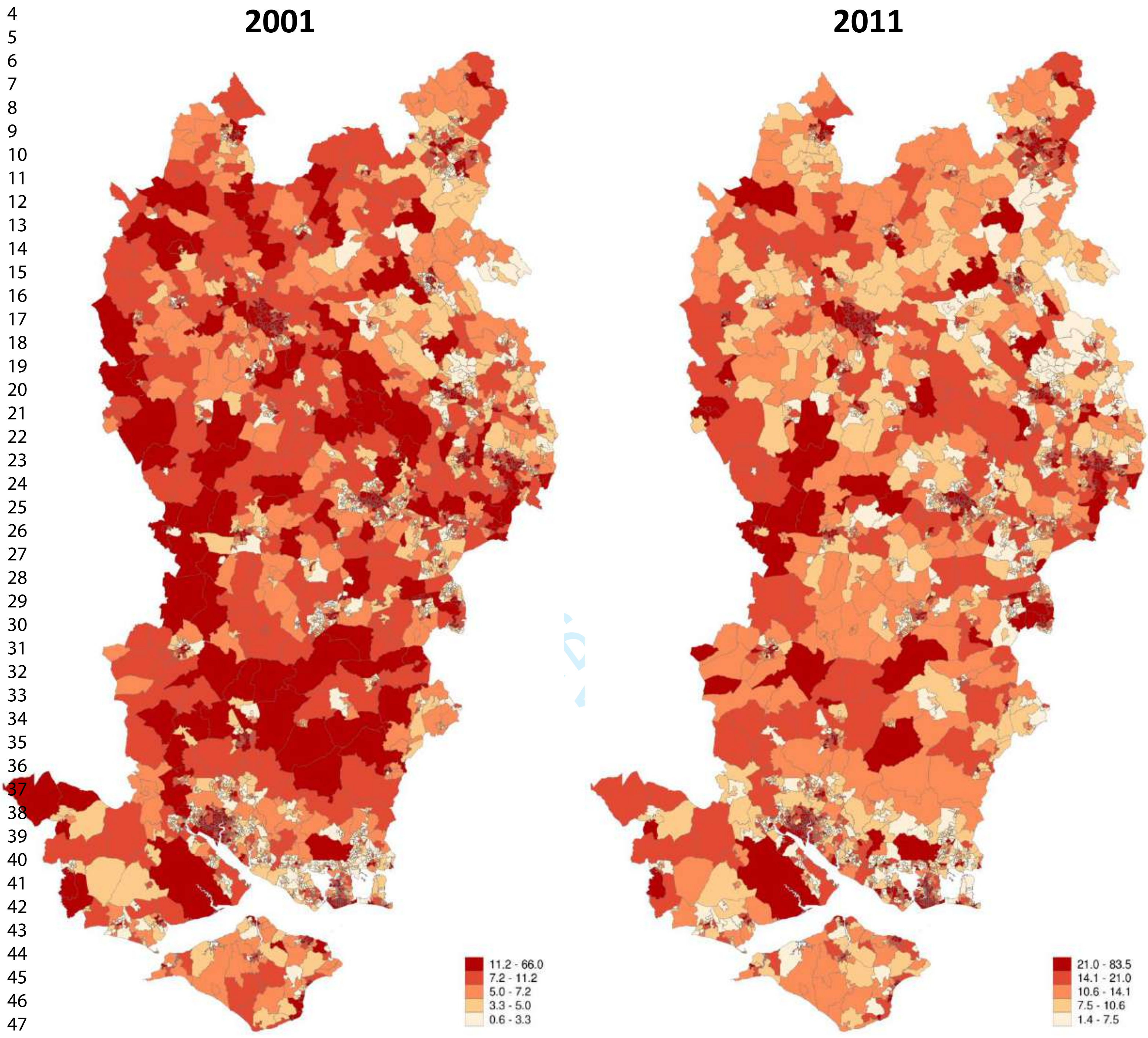

South West, Private Renting % of all households

2001

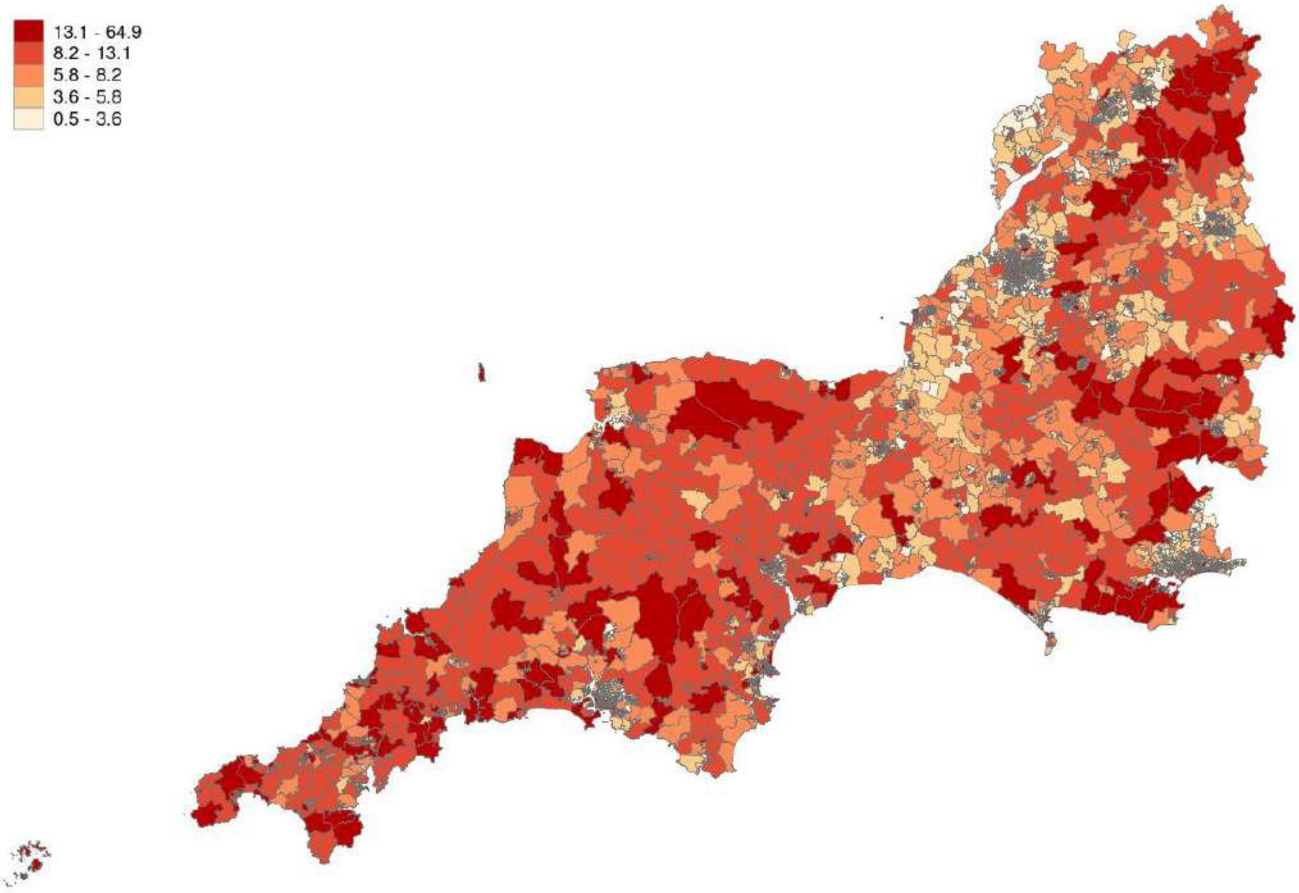

2011

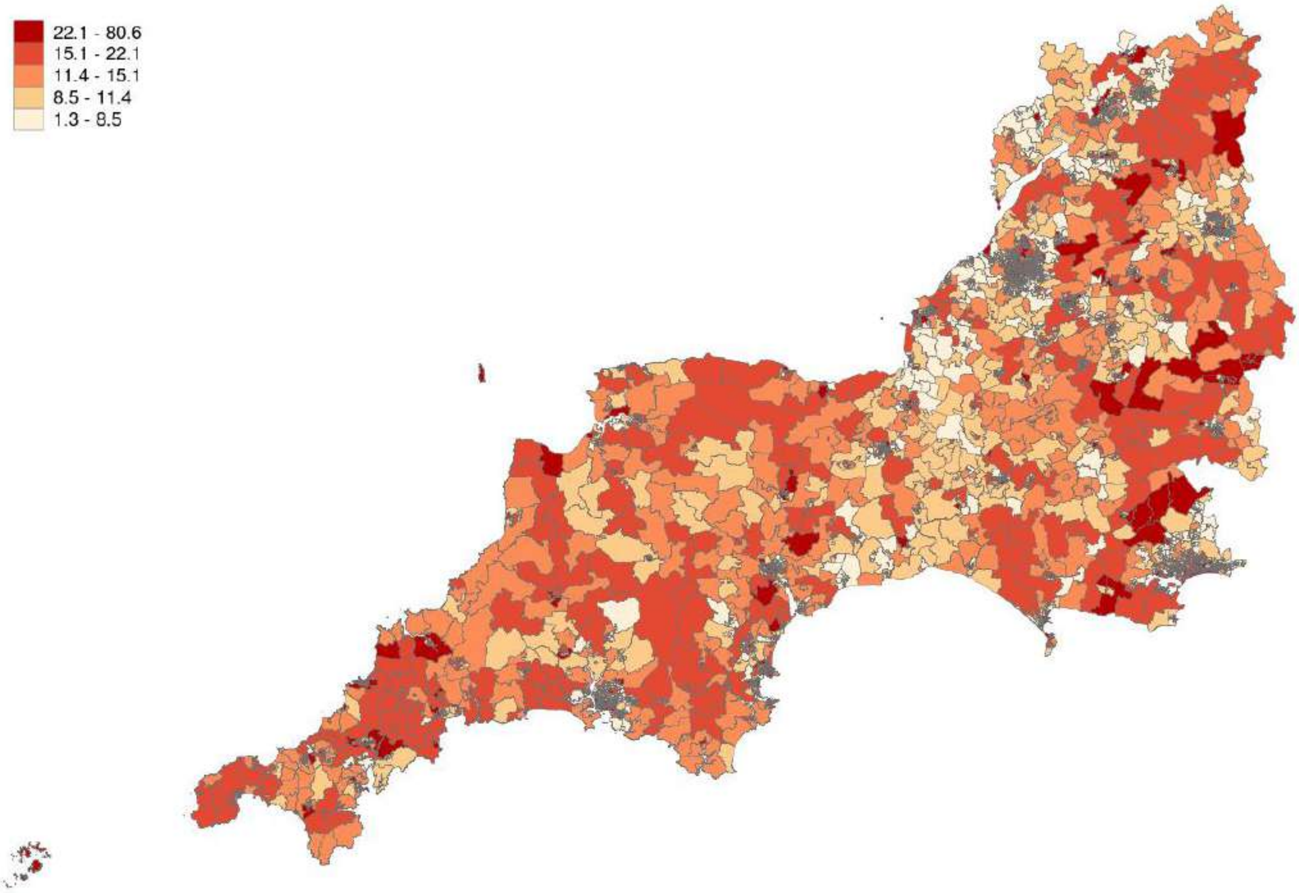

Population Turnover

England 2011, Population Turnover % of population

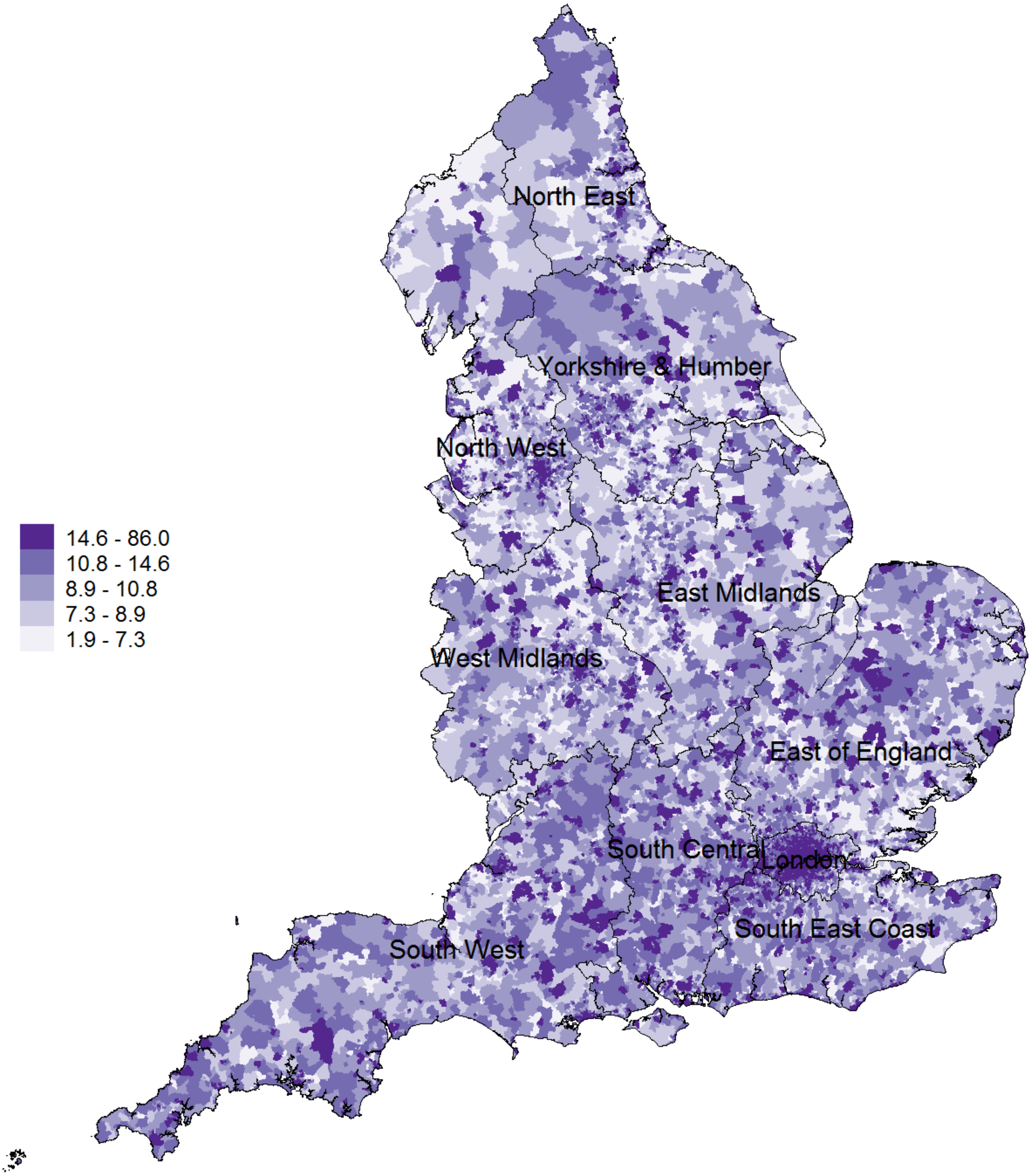

North East, Population Turnover % of population

2001 **2011**

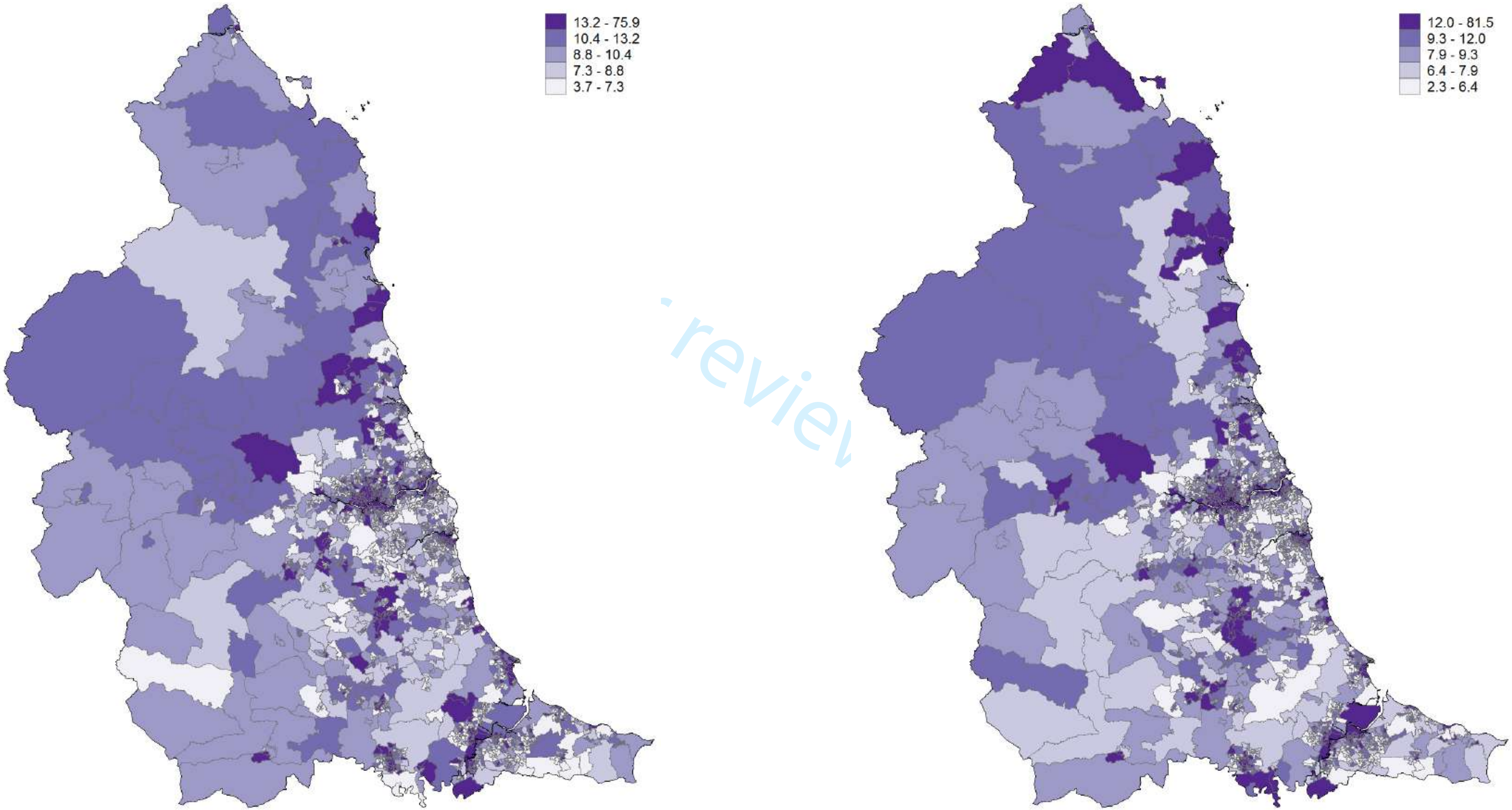

North East, Population Turnover % of population

2001

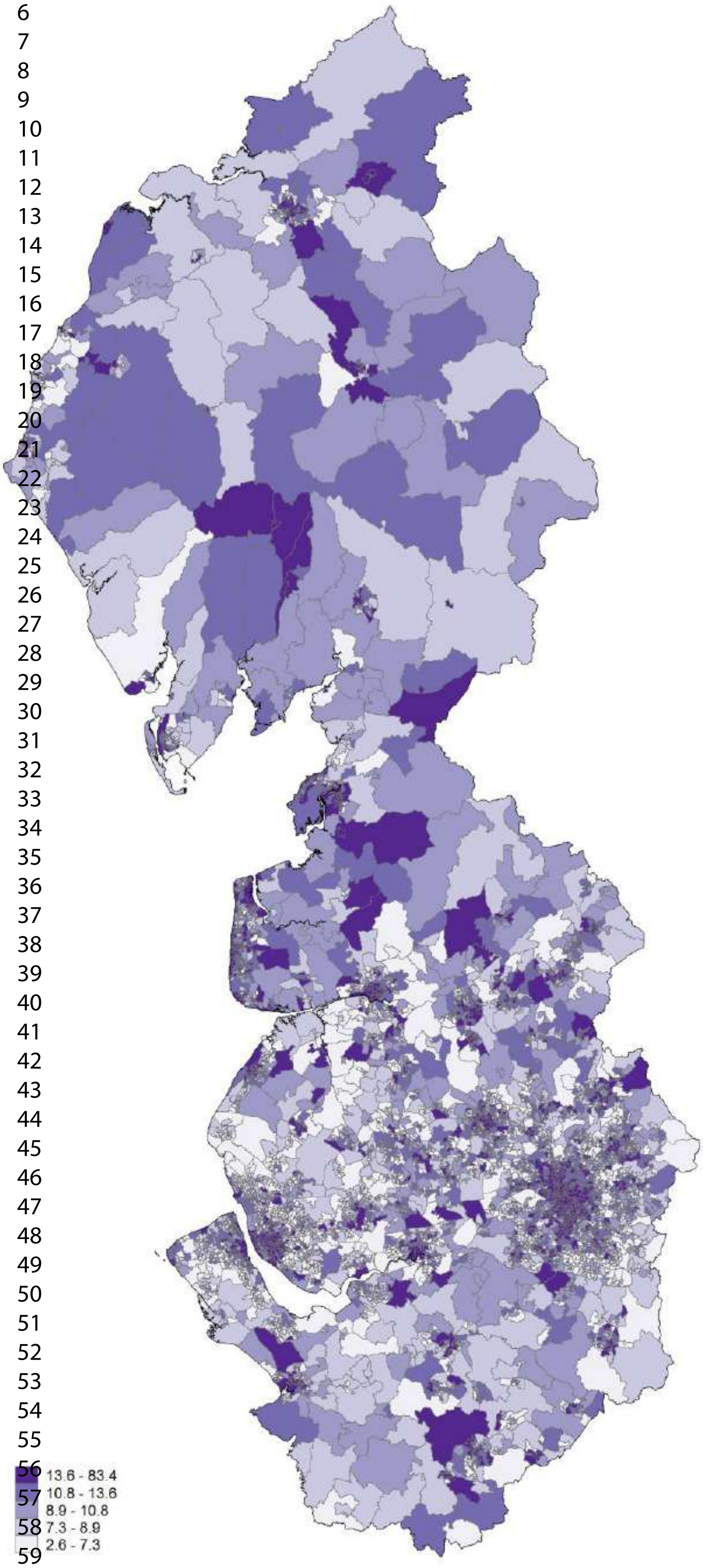

2011

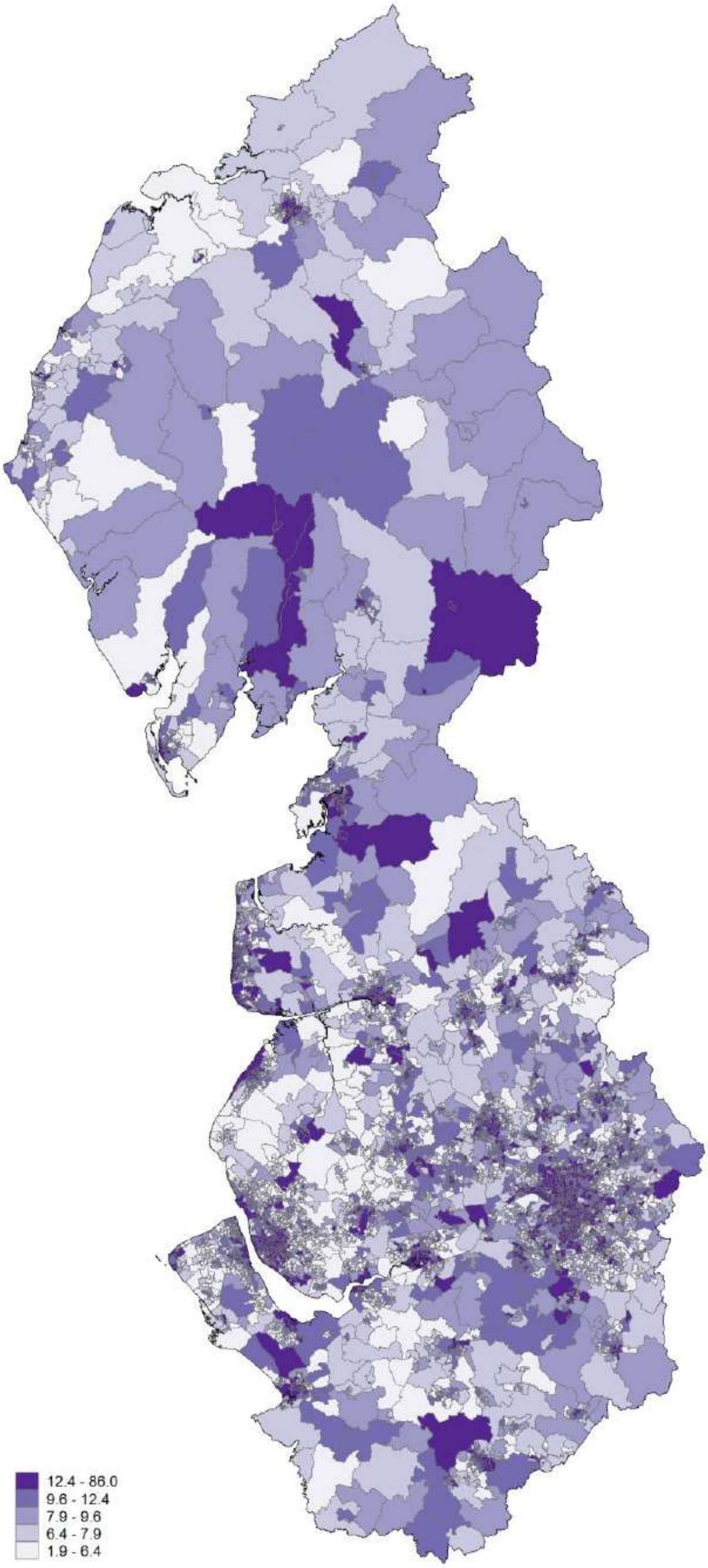

Yorkshire and the Humber, Population Turnover % of population

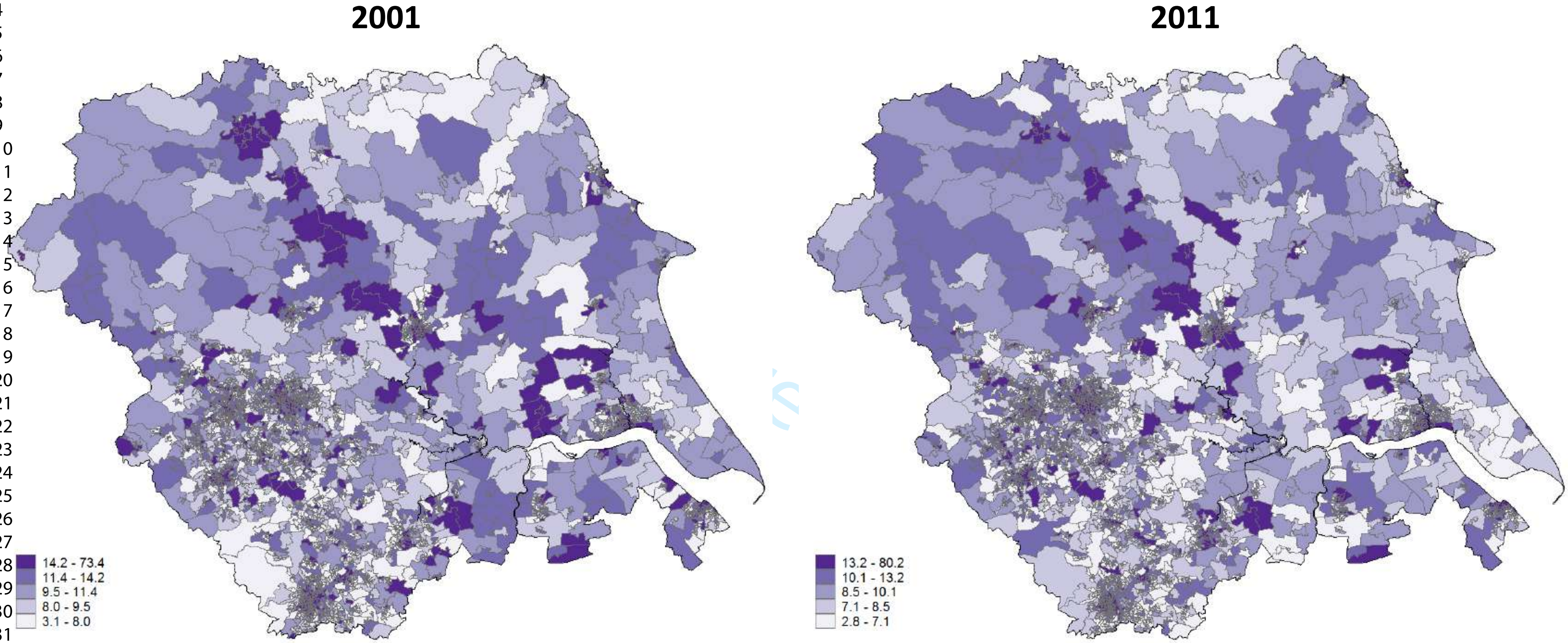

East Midlands, Population Turnover % of population

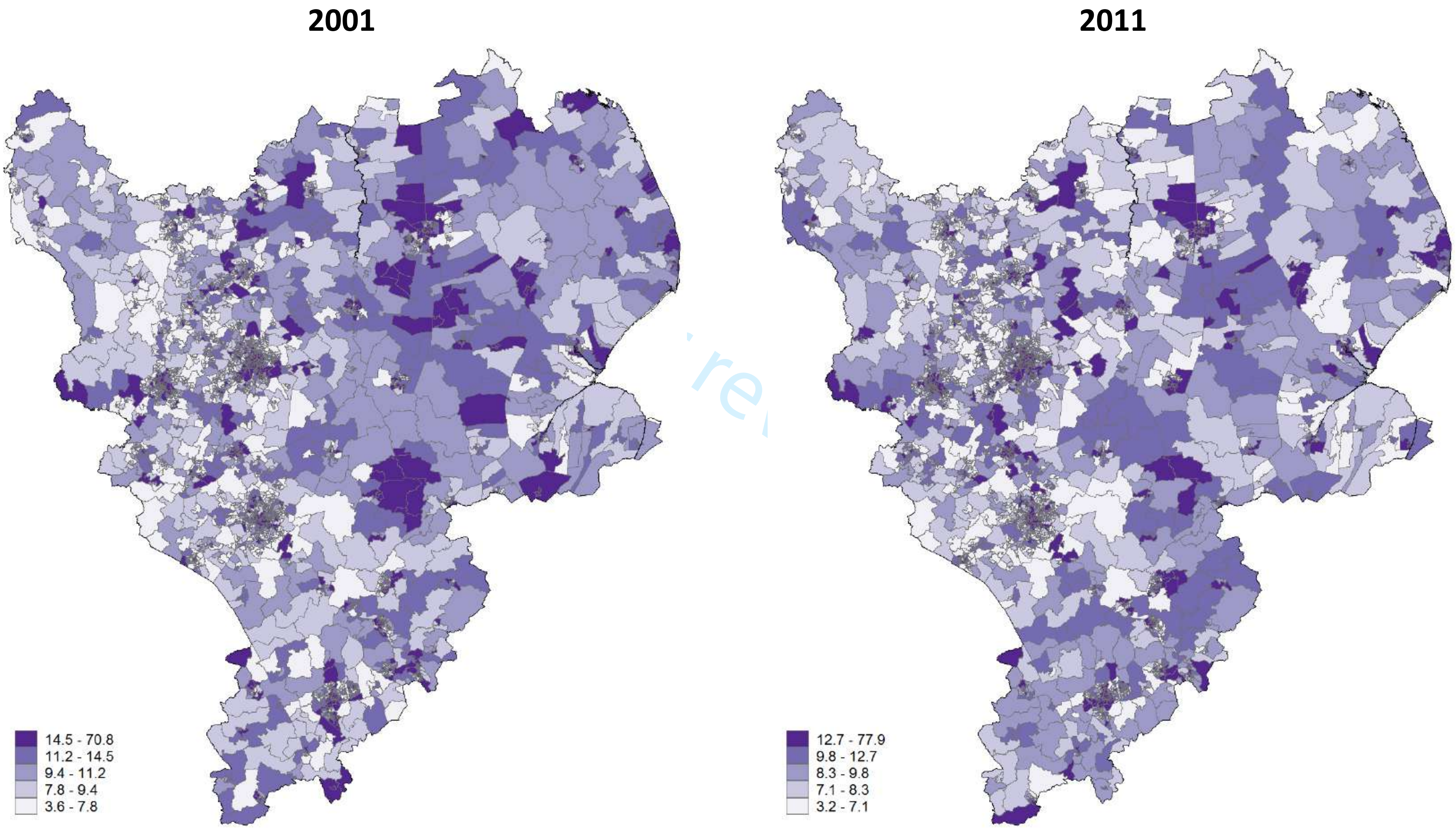

West Midlands, Population Turnover % of population

2001

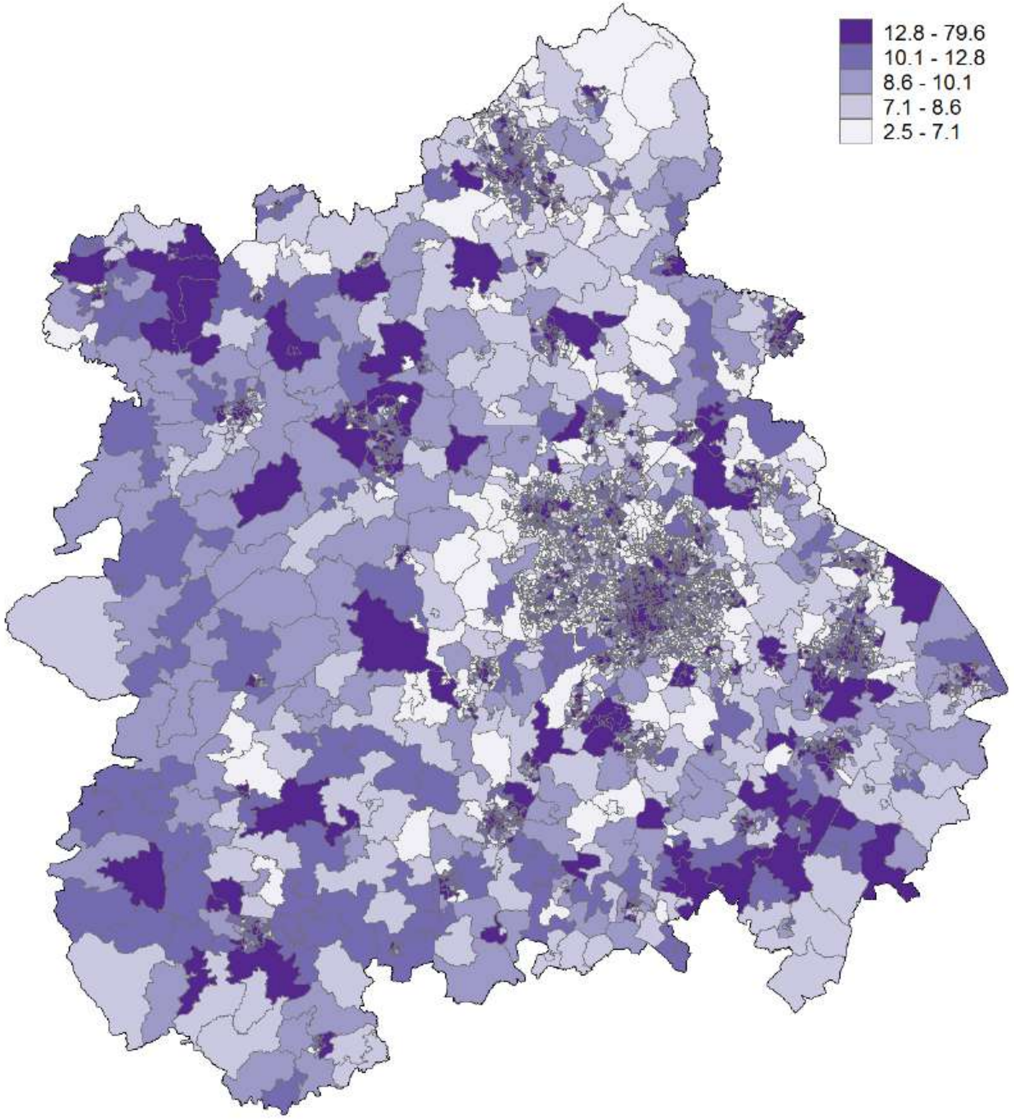

2011

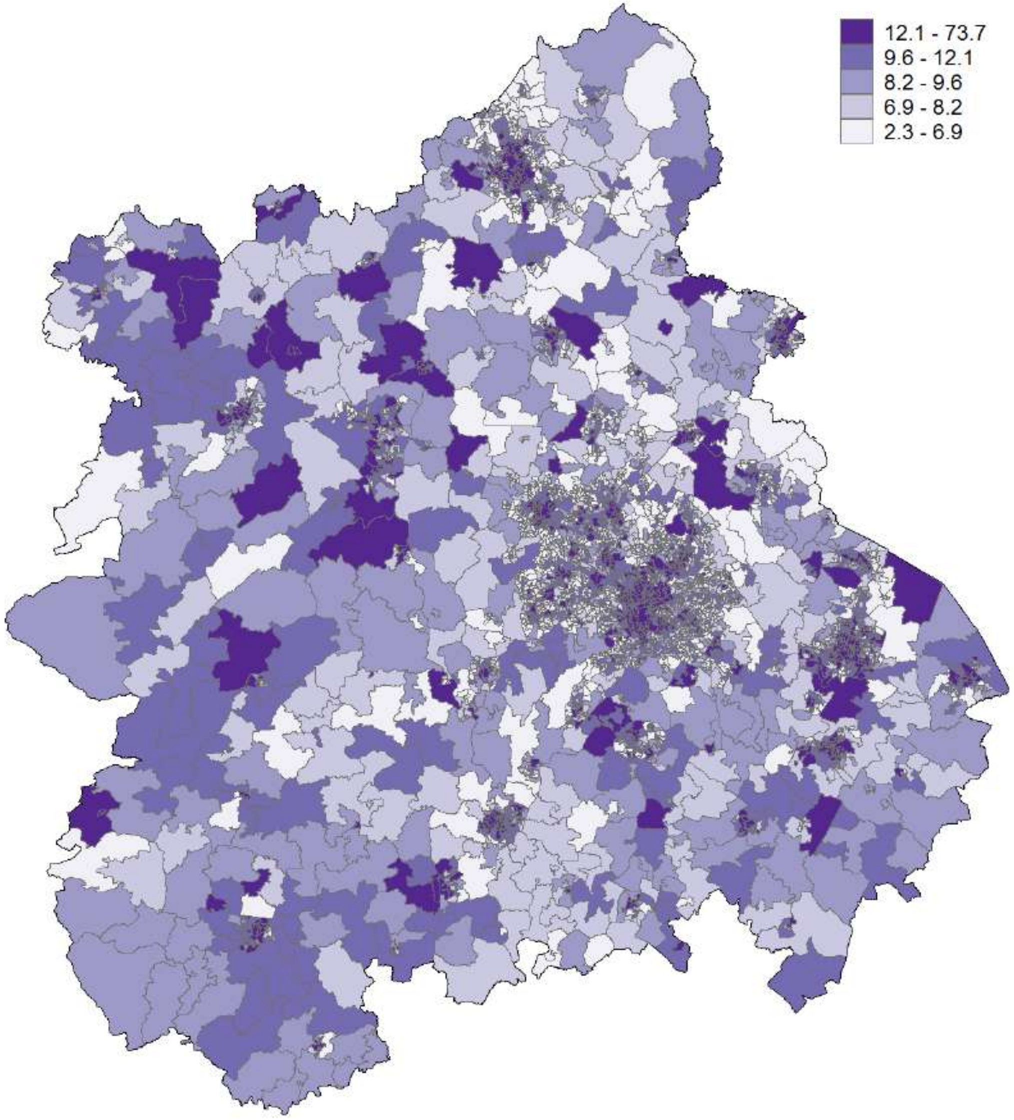

East of England, Population Turnover % of population

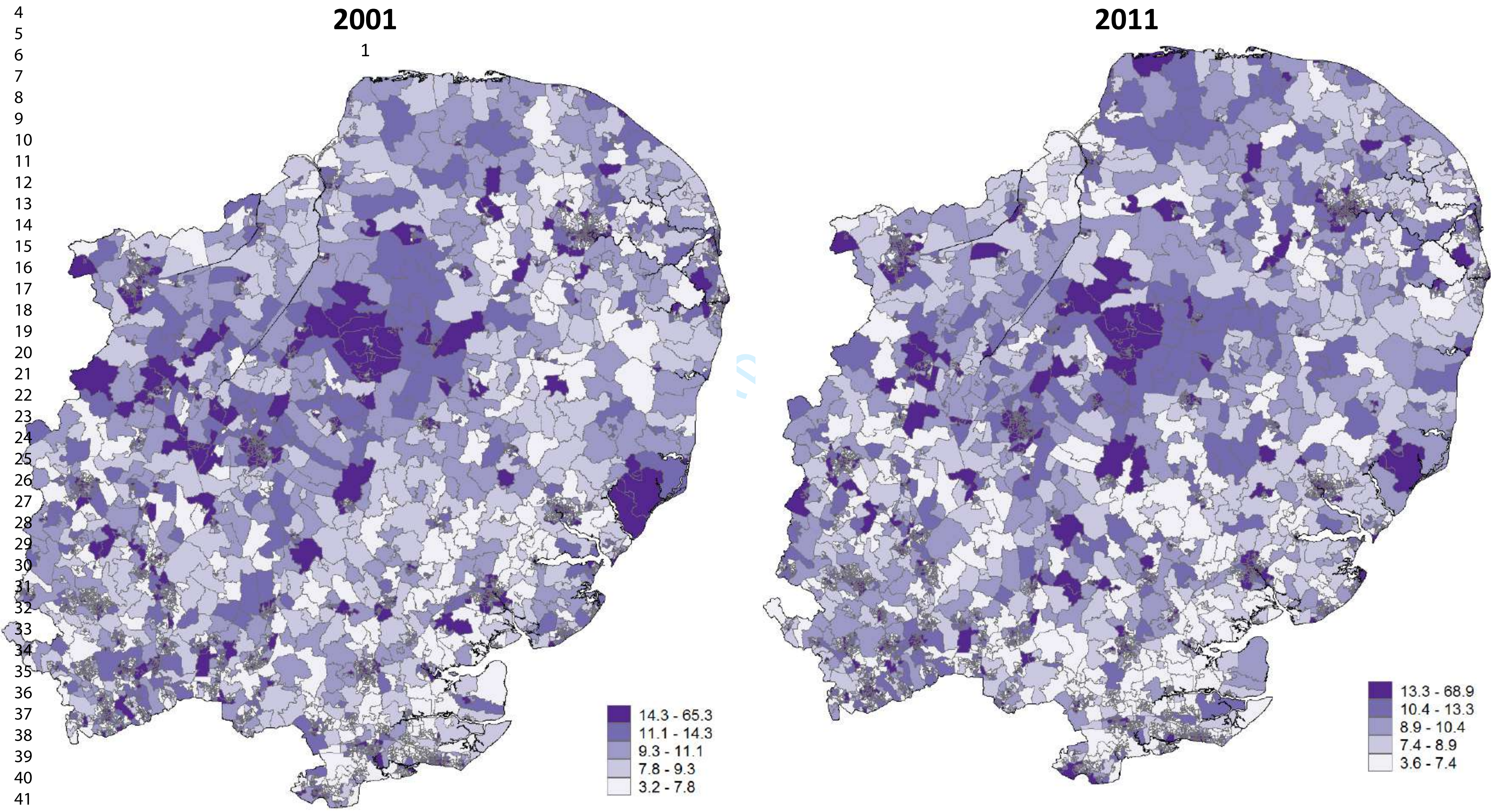

London, Population Turnover % of population

2001

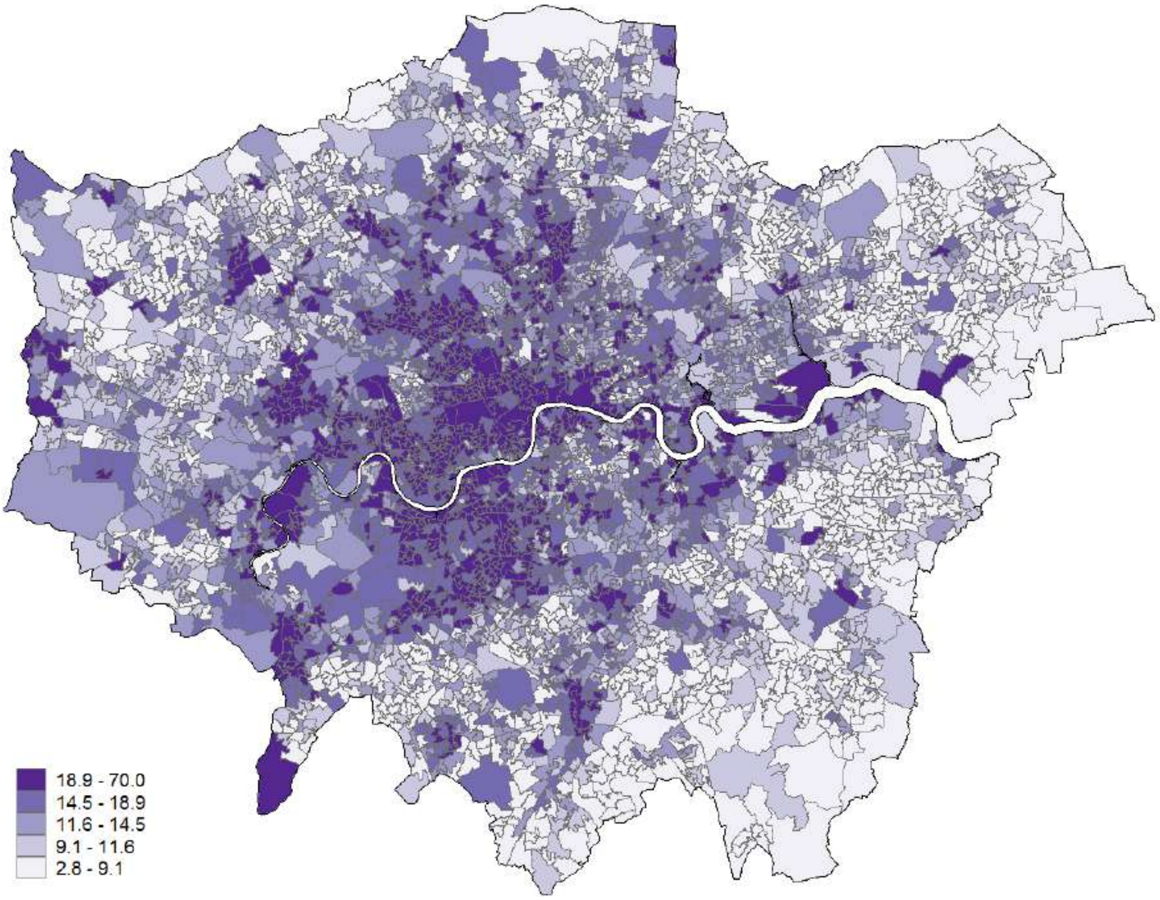

2011

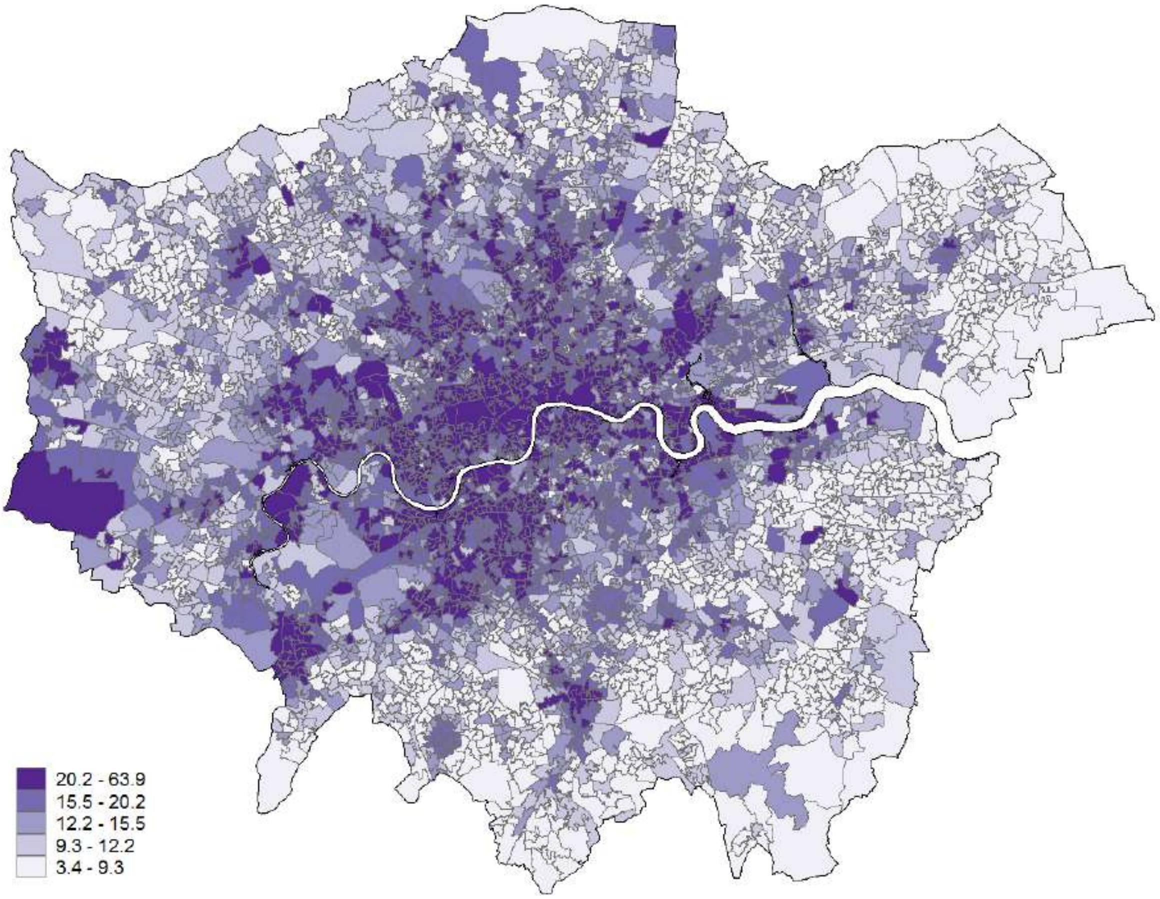

South East Coast, Population Turnover % of population

2001

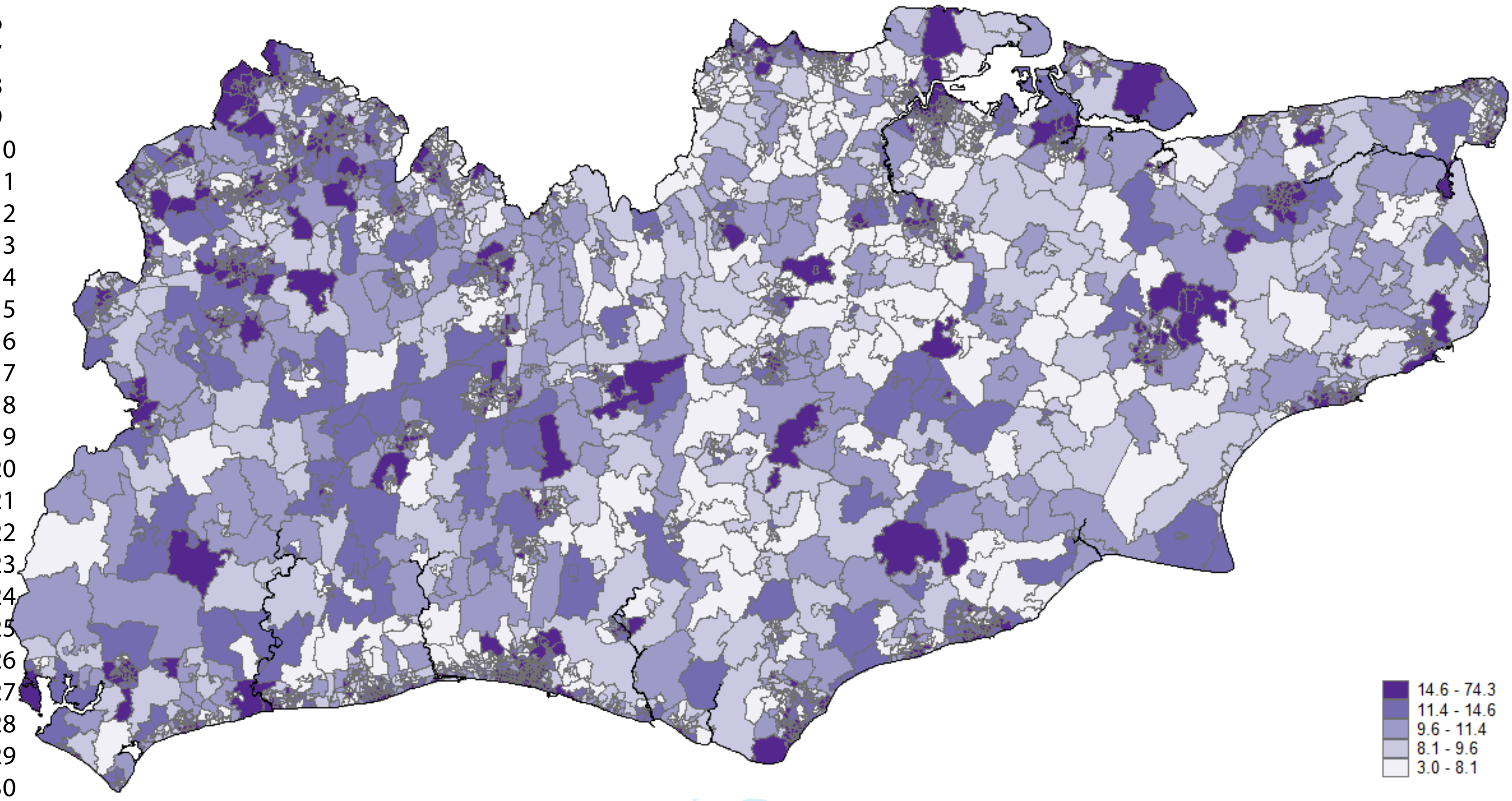

2011

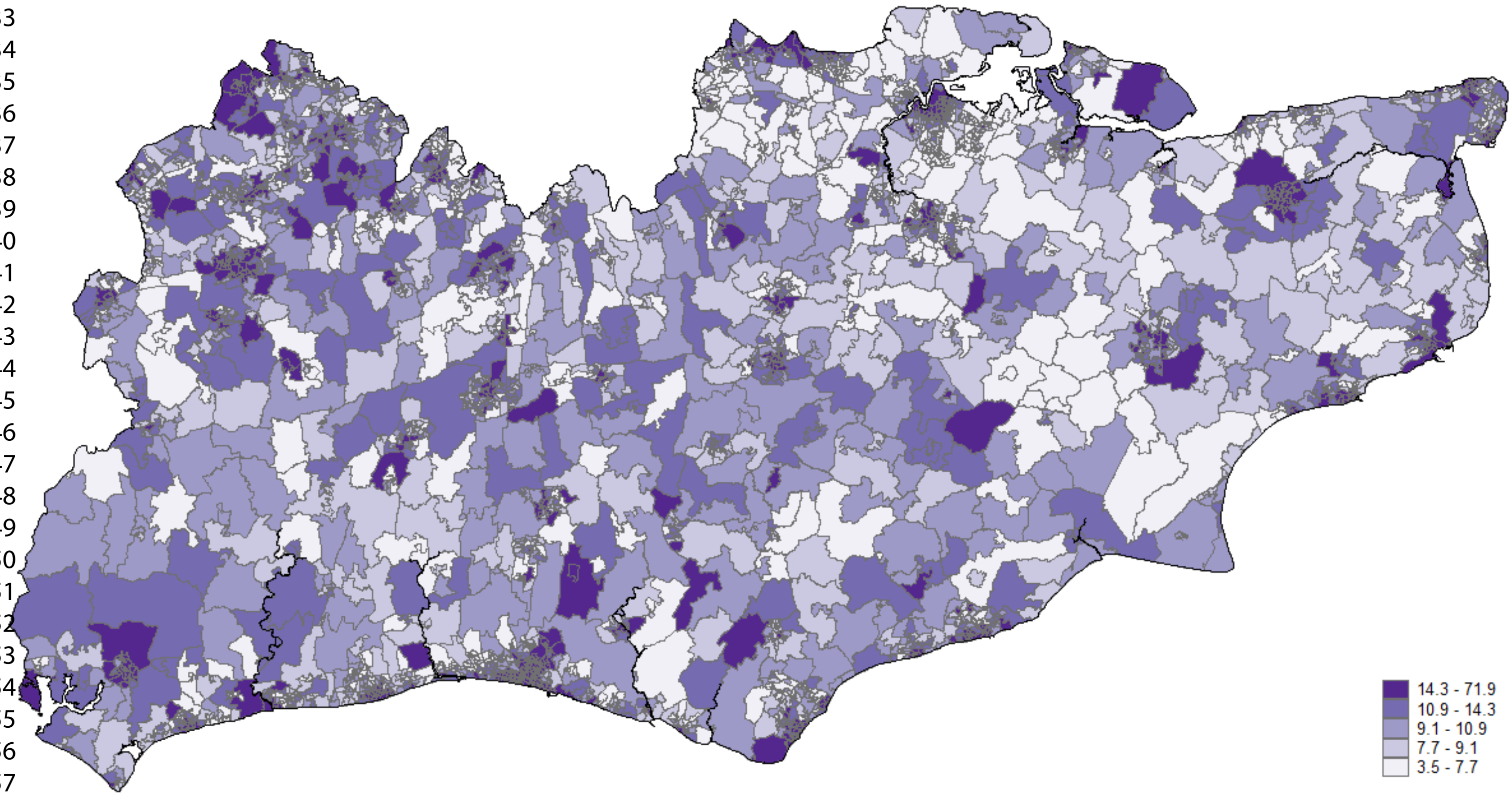

South Central, Population Turnover % of population

2001

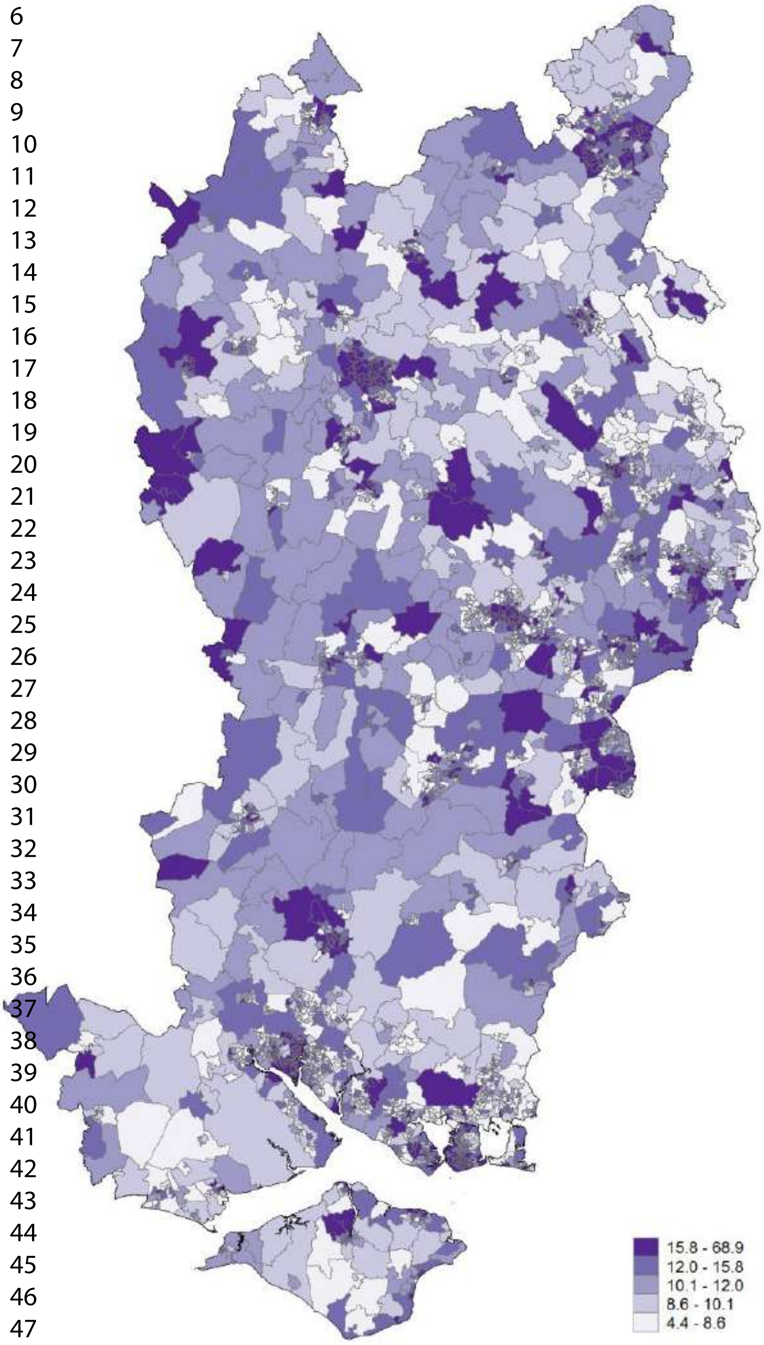

2011

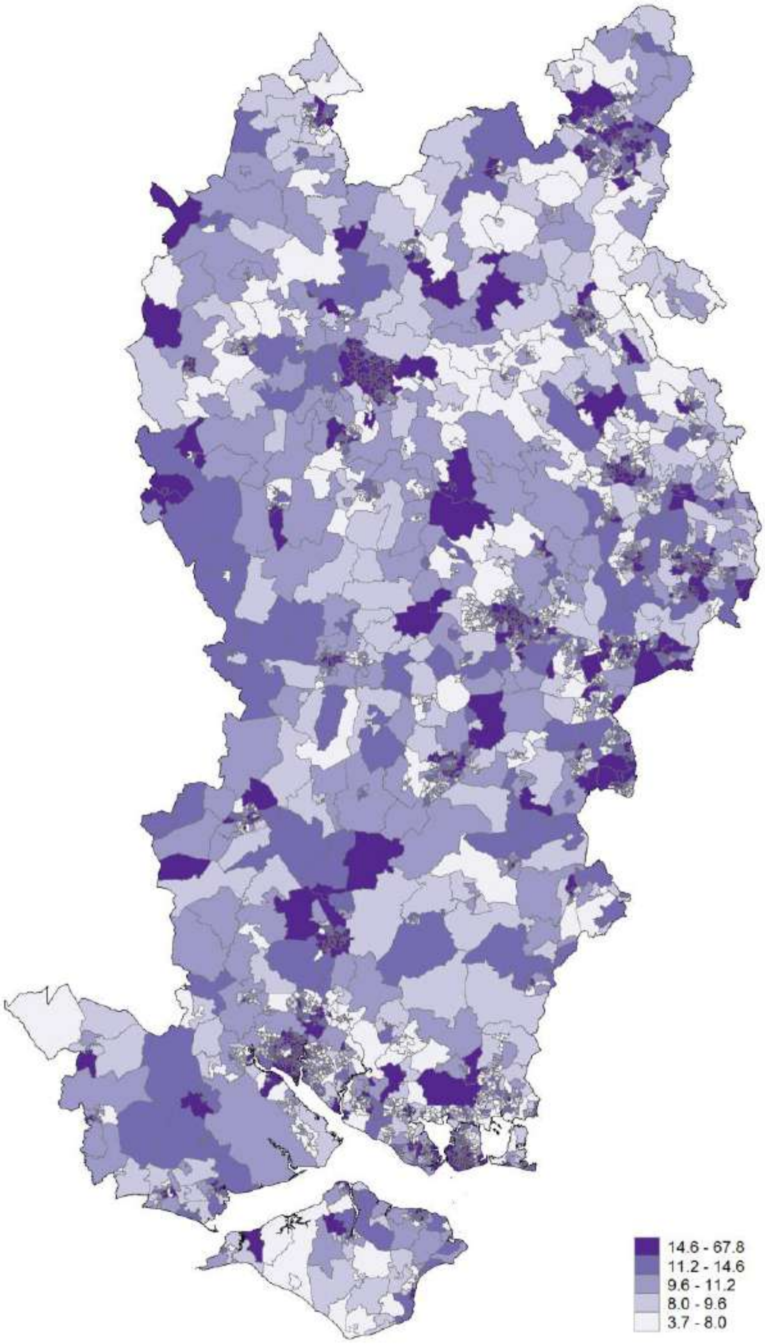

South West, Population Turnover % of population

2001

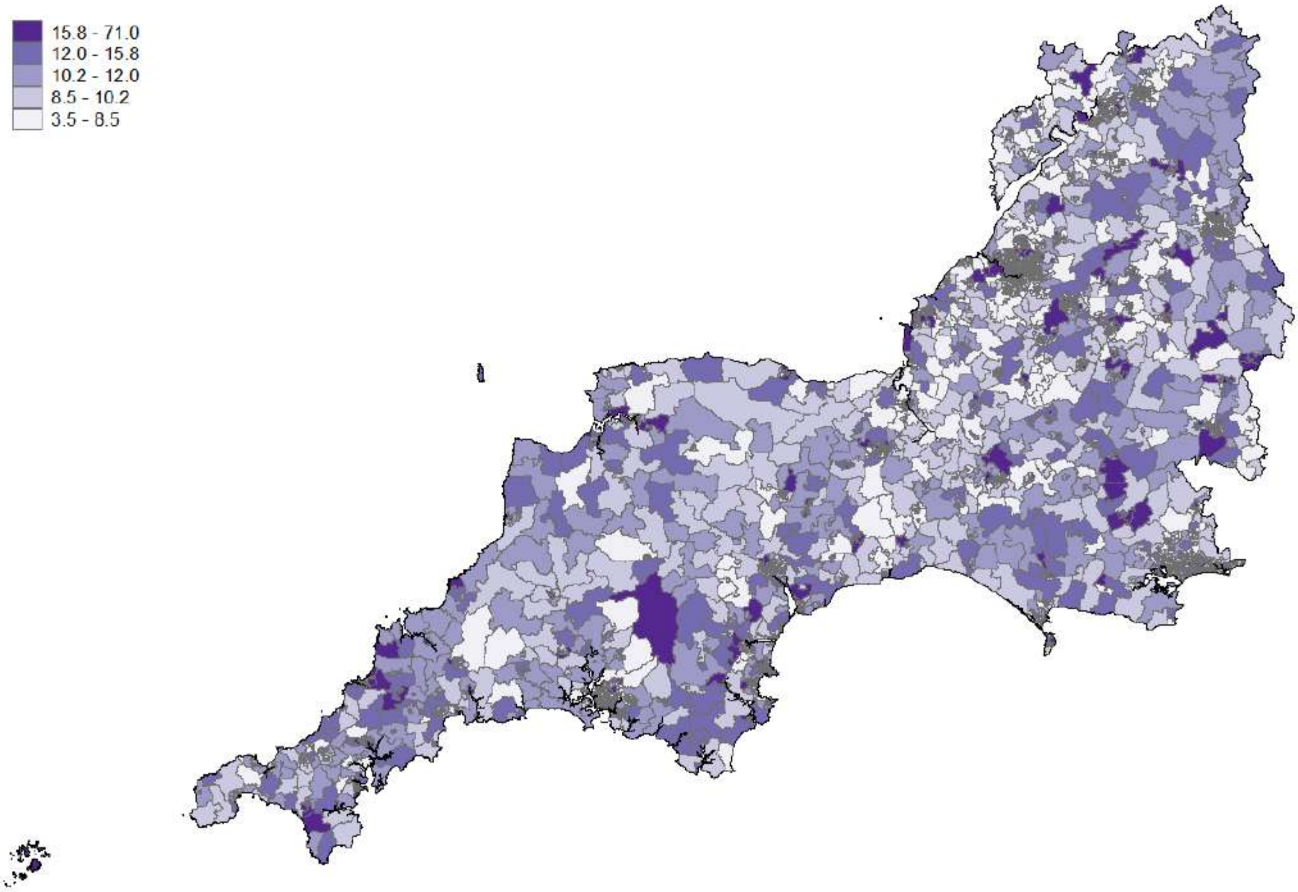

2011

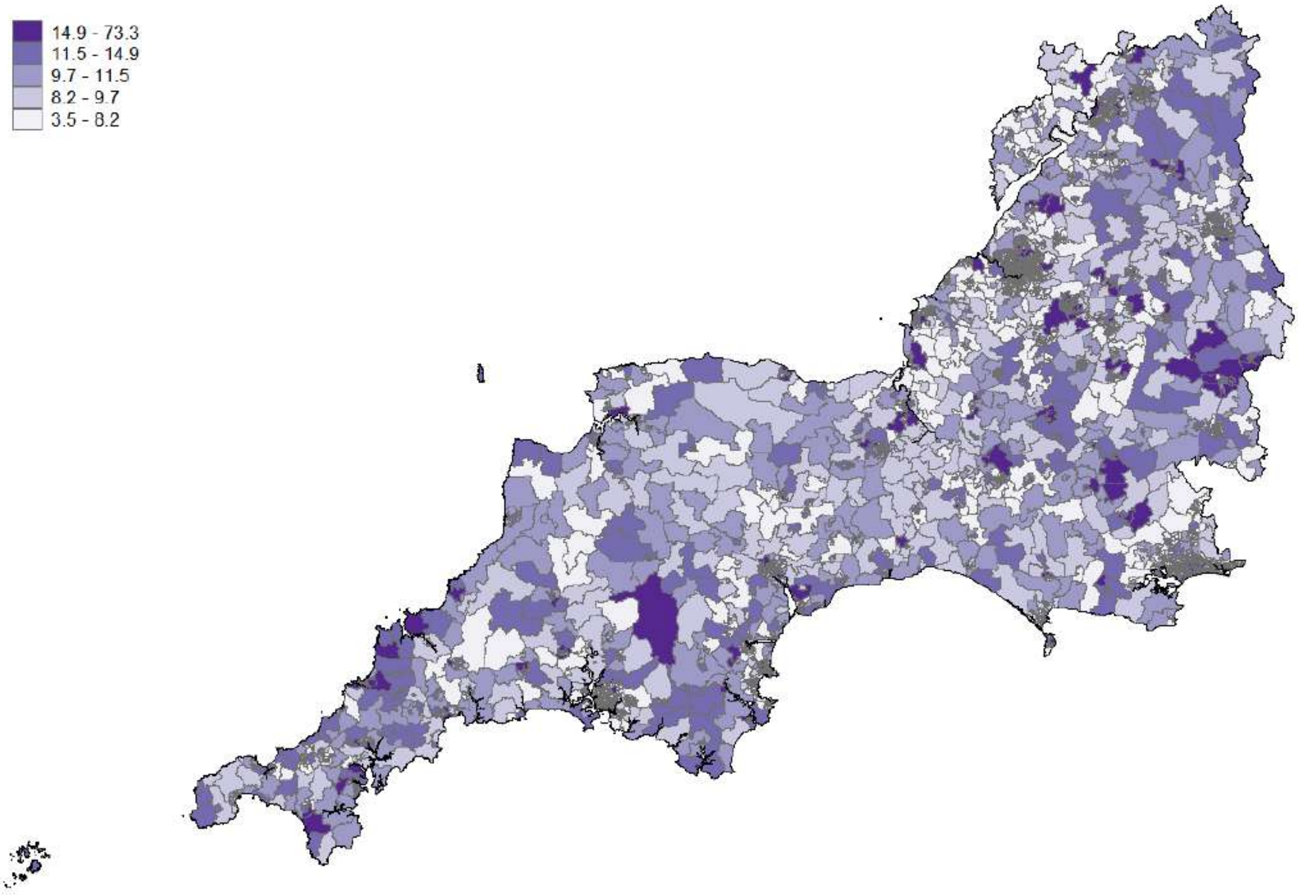

STROBE Statement—checklist of items that should be included in reports of observational studies

	Item No	Recommendation
**Title and abstract**	1	(*a*) Indicate the study's design with a commonly used term in the title or the abstract **p.1**
		(*b*) Provide in the abstract an informative and balanced summary of what was done and what was found **p.2**
**Introduction**		
Background/rationale	2	Explain the scientific background and rationale for the investigation being reported **p.3**
Objectives	3	State specific objectives, including any prespecified hypotheses **p.4**
**Methods**		
Study design	4	Present key elements of study design early in the paper
Setting	5	Describe the setting, locations, and relevant dates, including periods of recruitment, exposure, follow-up, and data collection **p.4**
Participants	6	(*a*) *Cohort study*—Give the eligibility criteria, and the sources and methods of selection of participants. Describe methods of follow-up **p.5** *Case-control study*—Give the eligibility criteria, and the sources and methods of case ascertainment and control selection. Give the rationale for the choice of cases and controls **NA** *Cross-sectional study*—Give the eligibility criteria, and the sources and methods of selection of participants **NA**
		(*b*) *Cohort study*—For matched studies, give matching criteria and number of exposed and unexposed **NA** *Case-control study*—For matched studies, give matching criteria and the number of controls per case **NA**
Variables	7	Clearly define all outcomes, exposures, predictors, potential confounders, and effect modifiers. Give diagnostic criteria, if applicable **p.4**
Data sources/ measurement	8*	For each variable of interest, give sources of data and details of methods of assessment (measurement). Describe comparability of assessment methods if there is more than one group **p.4**
Bias	9	Describe any efforts to address potential sources of bias **NA**
Study size	10	Explain how the study size was arrived at **p.5**
Quantitative variables	11	Explain how quantitative variables were handled in the analyses. If applicable, describe which groupings were chosen and why **p.5**
Statistical methods	12	(*a*) Describe all statistical methods, including those used to control for confounding **p.5**
		(*b*) Describe any methods used to examine subgroups and interactions **NA**
		(*c*) Explain how missing data were addressed **NA**
		(*d*) *Cohort study*—If applicable, explain how loss to follow-up was addressed **NA** *Case-control study*—If applicable, explain how matching of cases and controls was addressed **NA** *Cross-sectional study*—If applicable, describe analytical methods taking account of sampling strategy **NA**
		(*e*) Describe any sensitivity analyses **NA**

Continued on next page

For peer review only - http://bmjopen.bmj.com/site/about/guidelines.xhtml

**Results**		
Participants	13*	(a) Report numbers of individuals at each stage of study—eg numbers potentially eligible, examined for eligibility, confirmed eligible, included in the study, completing follow-up, and analysed **NA**
		(b) Give reasons for non-participation at each stage **NA**
		(c) Consider use of a flow diagram **NA**
Descriptive data	14*	(a) Give characteristics of study participants (eg demographic, clinical, social) and information on exposures and potential confounders p.5
		(b) Indicate number of participants with missing data for each variable of interest **NA**
		(c) *Cohort study*—Summarise follow-up time (eg, average and total amount) **p.5**
Outcome data	15*	*Cohort study*—Report numbers of outcome events or summary measures over time **p.5**
		Case-control study—Report numbers in each exposure category, or summary measures of exposure **NA**
		Cross-sectional study—Report numbers of outcome events or summary measures **NA**
Main results	16	(*a*) Give unadjusted estimates and, if applicable, confounder-adjusted estimates and their precision (eg, 95% confidence interval). Make clear which confounders were adjusted for and why they were included **NA**
		(*b*) Report category boundaries when continuous variables were categorized **NA**
		(*c*) If relevant, consider translating estimates of relative risk into absolute risk for a meaningful time period **NA**
Other analyses	17	Report other analyses done—eg analyses of subgroups and interactions, and sensitivity analyses **NA**
**Discussion**		
Key results	18	Summarise key results with reference to study objectives **p.5-p.6**
Limitations	19	Discuss limitations of the study, taking into account sources of potential bias or imprecision. Discuss both direction and magnitude of any potential bias **p.7**
Interpretation	20	Give a cautious overall interpretation of results considering objectives, limitations, multiplicity of analyses, results from similar studies, and other relevant evidence **p.8**
Generalisability	21	Discuss the generalisability (external validity) of the study results **p.9**
**Other information**		
Funding	22	Give the source of funding and the role of the funders for the present study and, if applicable, for the original study on which the present article is based **p.11**

*Give information separately for cases and controls in case-control studies and, if applicable, for exposed and unexposed groups in cohort and cross-sectional studies.

Note: An Explanation and Elaboration article discusses each checklist item and gives methodological background and published examples of transparent reporting. The STROBE checklist is best used in conjunction with this article (freely available on the Web sites of PLoS Medicine at http://www.plosmedicine.org/, Annals of Internal Medicine at http://www.annals.org/, and Epidemiology at http://www.epidem.com/). Information on the STROBE Initiative is available at www.strobe-statement.org.

For peer review only - http://bmjopen.bmj.com/site/about/guidelines.xhtml

BMJ Open

Spatial distribution and temporal trends in social fragmentation in England, 2001 to 2011: a national study

Journal:	*BMJ Open*
Manuscript ID	bmjopen-2018-025881.R1
Article Type:	Research
Date Submitted by the Author:	26-Oct-2018
Complete List of Authors:	Grigoroglou, Christos; University of Manchester, Centre for Primary Care<br>Munford, Luke; University of Manchester, Manchester Centre for Health Economics<br>Webb, Roger; University of Manchester, Centre for Suicide Prevention; Centre for Mental Health and Risk<br>Kapur, Navneet; University of Manchester, Centre for Suicide Prevention, Centre for Mental Health and Risk<br>Ashcroft, Darren; University of Manchester, Centre for Pharmacoepidemiology and Drug Safety<br>Kontopantelis, Evangelos; University of Manchester, Division of Population Health, Health Services Research and Primary Care
**Primary Subject Heading**:	Epidemiology
Secondary Subject Heading:	Mental health, Health services research
Keywords:	MENTAL HEALTH, EPIDEMIOLOGY, Organisation of health services < HEALTH SERVICES ADMINISTRATION & MANAGEMENT, SOCIAL MEDICINE

SCHOLARONE™
Manuscripts

Spatial distribution and temporal trends in social fragmentation in England, 2001 to 2011: a national study

Christos Grigoroglou[1,8], Luke Munford[2,8], Roger T. Webb[3,6,8], Nav Kapur[4,6,7,8], Darren M. Ashcroft [5,6,8], Evangelos Kontopantelis[1,8]

[1] NIHR School for Primary Care Research, Centre for Primary Care, Division of Population Health, Health Services Research and Primary Care, University of Manchester, Manchester, UK
[2] Centre for Health Economics, Division of Population Health, Health Services Research and Primary Care, University of Manchester, Manchester, UK
[3] Centre for Mental Health and Safety, Division of Psychology & Mental Health, University of Manchester, Manchester, UK
[4] Centre for Suicide Prevention, Division of Psychology and Mental Health, University of Manchester, Manchester, UK
[5] Centre for Pharmacoepidemiology and Drug Safety, School of Health Sciences, Faculty of Biology, Medicine and Health, , University of Manchester, Manchester Academic Health Sciences Centre (MAHSC), Manchester, UK
[6] NIHR Greater Manchester Patient Safety Translational Research Centre, Manchester Academic Health Sciences Centre (MAHSC), Manchester, UK
[7] Greater Manchester Mental Health Trust, Manchester, UK
[8] Manchester Academic Health Sciences Centre [MAHSC], Manchester, UK

Correspondence to:

Christos Grigoroglou
E-mail: christos.grigoroglou@postgrad.manchester.ac.uk
Telephone number: 0161-2750743
5th Floor, Williamson Building, University of Manchester,
Manchester, UK, M13 9PL

Word count: 3,370
5 Figures

For peer review only - http://bmjopen.bmj.com/site/about/guidelines.xhtml

Objective: Social fragmentation is commonly examined in epidemiological studies of mental illness as high levels of social fragmentation are often found in areas with high prevalence of mental illness. In this study we examine spatial and temporal patterns of the index of social fragmentation and its underlying indicators in England over time.

Setting: Data for social fragmentation and its underlying indicators were analysed over the decennial Censuses (2001–2011) at a small-area geographical level (mean of 1500 people). Degrees of social fragmentation and temporal changes were spatially visualised for the whole of England and its 10 administrative regions. Spatial clustering was quantified using Moran's I; changes in correlations over time were quantified using Spearman's ranking correlation.

Results: Between 2001 and 2011 we observed a strong persistence for social fragmentation nationally (Spearman's r=0.94). At the regional level, modest changes were observed over time, but marked increases were observed for two of the four social fragmentation underlying indicators, namely single people and those in private renting. Results supported our hypothesis of increasing spatial clustering over time. Moderate regional variability was observed in social fragmentation, its underlying indicators and their clustering over time.

Conclusion: Patterns of social fragmentation and its underlying indicators persisted in England which seem to be driven by the large increases in single people and those in private renting. Policies to improve social cohesion may have an impact the lives of persons who experience mental illness. The spatial aspect of social fragmentation can inform the targeting of health and social care interventions, particularly in areas with strong social fragmentation clustering.

Keywords: social fragmentation, mental health, clustering, inequalities, spatial autocorrelation

For peer review only - http://bmjopen.bmj.com/site/about/guidelines.xhtml

Strengths and Limitations of the study
➢ This study utilises data over two time points for the whole of England and is the first to describe spatial and temporal patterns of social fragmentation.
➢ Spatial aspects of social fragmentation can inform organisation of mental health services and social interventions that aim to enhance social support, particularly in areas where social fragmentation is high and is spatially clustered
➢ Over the last two decennial Censuses (2001 and 2011), the index of social fragmentation in England strongly persisted at a small-area geographical level while its clustering and its underlying indicators increased over time.
➢ Over time social fragmentation remained relatively stable, but we found marked increases in the percentage of single people and the percentage of those living in private rented accommodation.
➢ Even though census data are considered of high quality, people's perception of social fragmentation may not be fully captured in the census while demographic factors may capture social fragmentation in some areas but not in others.

For peer review only - http://bmjopen.bmj.com/site/about/guidelines.xhtml

INTRODUCTION

The impact of local area characteristics, such as material deprivation, on health is well established. [1 2] However, similar to socioeconomic position, the social networks and the amount of social support in a neighbourhood may also partially determine an individual's health. [3-6] In this context, an area-level characteristic that has been the focus of much investigation in neighbourhood health research is social fragmentation. The index of social fragmentation measures lack of social cohesion, and it is used to define areas with a breakdown or absence of social capital, and was originally created to measure the non-economic deprivation aspects of areas. [7]

Social fragmentation is distinct conceptually from deprivation, since fragmentation is a feature of household composition and demographic structure and is not intrinsically linked to socioeconomic position.[8] However, the two measures tend to be positively correlated as they are both concentrated in large towns and cities. [8 9] Thus, many urban localities are both deprived and socially fragmented, although the two phenomena do not always co-exist. Several studies suggests that fragmented communities provide less stable social institutions and social bonds, although maintaining stable institutions and bonds within the communities can contribute substantially to the creation of shared identities, persistence of social relations, promotion of healthy behaviours and good physical and mental health for the population. [10-14]

Social fragmentation has received less attention from researchers than deprivation, despite the fact that it can aid the understanding of mechanisms via which the wider social environment influences mental health. [15] Research on adults has shown that social fragmentation is more closely related to mental health than physical health outcomes, independent of material deprivation and individual-level risk factors. [7 16] In the context of mental health, social fragmentation has been linked with suicide and non-fatal self-harm, [7 17-19] mental disorders, [4 11 20] psychiatric health service use, [21] first admission for psychosis, [20-22] psychological distress, [3 11] and schizophrenia. [15 23] Nonetheless, physical illness outcomes do not appear to be as clearly linked to social fragmentation. [18 24 25]

In the UK, an index of social fragmentation was developed to measure neighbourhood-level conditions which affect lack of opportunities for social integration. [7] The index was originally based on indicators of population turnover, percentage of married people, percentage of the population in private renting and percentage of one person households using data from the UK Census. The rationale for the selection of the underlying variables used for the calculation of the index was that several studies, both at ecological and individual level, have found raised mental illness prevalence in highly urbanised areas with high population turnover, many non-

For peer review only - http://bmjopen.bmj.com/site/about/guidelines.xhtml

family households, and reduced social cohesion. [26] However, social trends towards increasing cohabitation, [10 27] a phenomenon that occurs most commonly in urban areas [24], suggest the use of proportion of single, widowed and divorced people instead of married people in the calculation of the index.

Social fragmentation in a population is likely to have profound effects on its health and wellbeing, and yet it is incompletely examined and reported in the existing literature, and a number of important research questions remain unanswered. First, how strongly clustered is social fragmentation? Second, how strongly does social fragmentation cluster and change at regional level over two time points? Third, does social fragmentation persist over time? Answering all three questions is important to inform the organisation of healthcare services and to target social and healthcare interventions, especially for mental health for which social fragmentation appears to be a salient risk factor. In this study, we temporally and spatially describe the properties of social fragmentation in England and within each of the 10 administrative English regions and make comparisons across them. Finally, we quantify the persistence of social fragmentation over the study period.

METHODS

Data sources

To derive the index of social fragmentation we accessed decennial Census data from the Nomis website [28] on: (1) One person households, % of all Census households; (2) single people (including widowed, divorced and separated people), % all usual residents aged 16 or over; (3) Population turnover (percentage of the population that moved into the area from within the UK and from outside the UK), % Census population in the year preceding the census (4) Households in private renting, % of all Census households. Our analysis was conducted at a small-area geographical level, namely the Lower Layer Super Output Area (LSOA). LSOAs are geographical areas with a mean population of 1,500 people. All data were available at the LSOA level except for the population turnover data which were available at the output area level (OA). OAs provide the finest spatial scale at which the Census is enumerated. We assigned the OA level data to the LSOA level using lookup tables provided by the Office for National Statistics (ONS). More details on the data used to derive each social fragmentation underlying indicator are provided in the Supplementary appendix.

The social fragmentation index for each LSOA was calculated by adding the unweighted z scores for each of the four characteristics into a composite score in each census year pooled. We used

For peer review only - http://bmjopen.bmj.com/site/about/guidelines.xhtml

two time points for the index of social fragmentation, i.e. 2001 and 2011 over the two decennial Censuses. Across LSOAs the scores for the index ranged from -6.42 to 27.57 for 2001 and from -7.09 to 25.79 for 2011, with the upper boundaries indicating the most socially fragmented areas and the lower boundaries the most socially cohesive areas. Since the data used to derive the index in 2001 were reported using 2001 LSOAs, we used a weighted means algorithm to assign the data for each underlying indicator to 2011 LSOAs using mid-year population estimates that were obtained from ONS. [29] A detailed description on how we attributed 2001 LSOA data to 2011 LSOAs is provided in the supplementary material. We calculated the z-scores of all social fragmentation underlying indicators for each of the two time points of interest. Finally, we obtained data on LSOA rural/urban classification at the 2011 LSOA.[30]

Analyses

Our outcome of interest was the index of social fragmentation and its four underlying indicators measured separately at the 2001 and 2011 national Censuses. We visualised temporal changes in social fragmentation and its underlying indicators with the use of spatial maps for all of England and each of the 10 English regions/strategic health authorities (SHA) (North East, North West, Yorkshire & the Humber, East Midlands, West Midlands, East of England, London, South East Coast, South Central and South West), which were the highest level of organisation available in NHS England during the study period. To assess whether levels of spatial autocorrelation for social fragmentation changed over time we compared the values of Moran's I [31] in the two time points for England and each English region to allow within-England comparisons. The measure can identify the presence or absence of spatial clusters while accounting for the multi-dimensional and multi-directional nature of spatial autocorrelation. Under a random spatial pattern a higher than expected value of Moran's I for social fragmentation would indicate that areas with high levels of social fragmentation are clustered and also that LSOAs with high social fragmentation are bordered with LSOAs with similarly high levels of social fragmentation. We also investigated local indicators for spatial autocorrelation (LISA) [32] to identify local patterns of spatial associations. We also quantified the persistency of social fragmentation over the two time points by calculating Spearman's correlation between 2001 and 2011. To visualise and compare temporal changes in the mean social fragmentation levels across the 10 English regions, we plotted population-weighted box plots for the main outcome and its underlying indicators. Finally, we used population-weighted box plots to visualise and compare temporal changes in the social fragmentation levels between the 10 English regions. Analyses were performed with Stata v.14.1 and R v.3.3.1. Due to the size of the dataset, effectively the whole of England, statistical significance is irrelevant; observed

For peer review only - http://bmjopen.bmj.com/site/about/guidelines.xhtml

associations of minimal strength would be statistically significant, and thus we focussed on effect sizes wherever possible.

RESULTS

Region-level characteristics of the index of social fragmentation at both time points are provided in Table 1. We found strong correlation for social fragmentation at the two time points (rho=0.93), using Spearman's ranking correlation to account for the relative nature of the measure. We plotted spatial maps of social fragmentation for the whole of England and for each region for the two time points analysed. Maps of social fragmentation in 2001 and 2011 and their respective changes for the whole of England and London are presented in Figure 1 and Figure 2 respectively, whilst social fragmentation and its underlying indicators maps over time for each region are presented in supplementary Appendix 2. Temporal changes in social fragmentation for England and for all English regions are presented in Figure A in the online appendix. We identified a pattern of stability with modest increases over time for regions in the North, and small decreases in social fragmentation for regions in the South. However, temporal changes of English regions for two of the four social fragmentation underlying indicators were much larger and they are presented in figures 3A and 3B.

Large changes in absolute levels of single person households and private renting were found in all regions. More specifically, in 2001 the average number of single persons of the total population was 38.4% while in 2011 the figure was 52.6% indicating a 31% increase. Similarly, in 2001 the average number of people in private renting was 8.5% of all English households while in 2011 this figure increased to 16.2% indicating a 62% increase. The largest increases in numbers of single people were found in the North East, North West and West Midlands while London had the highest levels of single people. Moreover, the largest increases for private renting were found in the North East, West Midlands and East Midlands while London had again the largest numbers of people living in private renting. For the other two social fragmentation underlying indicators, we found that the average number of one person households both nationally and regionally remained relatively stable. We also observed a modest decrease, both nationally and regionally (with the exception of London), in our measure of population turnover which reflects the levels of migration inside and outside areas. Finally, we observed great variability in levels of social fragmentation and its underlying indicators within regions at both time points. High levels of social fragmentation were concentrated in London, the North West and the South West. East England had the lowest levels of social fragmentation followed by West Midlands. There was a clear distinction between rural and urban areas, with the latter having far greater levels of social fragmentation.

For peer review only - http://bmjopen.bmj.com/site/about/guidelines.xhtml

Across the whole of England, Moran's I for social fragmentation was very low at 0.0449 in 2001 (95% CI 0.0447 to 0.0451) but by 2011 it had increased to 0.0794 (95% CI 0.0792 to 0.0797), indicating a small increase in spatial autocorrelation and clustering (Figure 4). We also observed marked regional variability in social fragmentation spatial autocorrelation levels over time (Figure 4). In both time points, the West Midlands had the lowest spatial autocorrelation in social fragmentation, followed by the East of England, while the South East and South West had the highest levels of spatial autocorrelation at both time points. In contrast to increases in spatial autocorrelation across the country between 2001 and 2011, the South East, North East and Yorkshire & the Humber had decreasing levels of spatial autocorrelation over the two time points.

Spatial clustering also appears to have increased over time for all fragmentation underlying indicators nationally, although its levels remained low (Figures A1 and A2 in supplementary Appendix). For one person households we found very low regional clustering and small regional variation. For single persons and private renting regional variation in clustering was moderate and their levels were also low with the exception of the South East and South West. Finally, for population turnover we observed the greatest increases of spatial autocorrelation both nationally and regionally but levels of regional clustering were generally low with the exception of South West whilst regional variability was moderate. We provide graphs and discuss the results from the LISA analysis for local spatial autocorrelation in the supplementary material (Figures B1 and B2).

DISCUSSION

Between 2001 and 2011, we found evidence of strong persistence of social fragmentation in England at small-area level. Regional changes over time were modest for social fragmentation, which is not surprising considering the large population denominators and the relative nature of the measure. Similarly, we observed modest increases over time for one person households and population turnover. However, changes over time for single persons and private renting were much greater, with substantial increases in both underlying indicators across regions, and these specific influences appear to have largely driven the heightened levels of social fragmentation overall. Furthermore, our findings provide evidence of increasing clustering for social fragmentation and its underlying indicators which, in conjunction with the regional variation observed between the two time points, indicates that areas in the South of England (including London) appear much more clustered than the North. Finally, we observed increased clustering for all underlying indicators of social fragmentation over the two time points.

For peer review only - http://bmjopen.bmj.com/site/about/guidelines.xhtml

Strengths and limitations of the study

The main strength of this study lies in its large dataset for the whole of England (53 million people in 2011) over two time points across 32,844 LSOAs, which we utilised to investigate spatial and temporal patterns of social fragmentation. To our knowledge, it is the first study of its kind to provide an insight into the temporal persistence of social fragmentation and its clustering.

However, some limitations exist. First, the extent to which the index captures social fragmentation may vary across the country as the demographic factors that we used may not in themselves be valid indicators of social fragmentation, or they may adequately measure social fragmentation in some areas but not in others. [22] However, the measure has been reported widely in the literature and on this basis social fragmentation has been identified as an important predictor of mental health outcomes such as suicide. Second, Census questions do not usually directly elicit the presence or quality of neighbourhood social ties or institutions, or people's relationship to their neighbourhood. Therefore, we might have failed to capture people's perception of social fragmentation through the Census data. Nevertheless, we used a measure capturing compositional aspects of neighbourhood which appear to be related to the capacity of a neighbourhood to act collectively, and is also important for the construction and maintenance of social ties and institutions. Third, whilst Census data is recognised to be of a high standard, some unobserved heterogeneity persists. [33] For example, LSOAs are defined from administrative information and this may disguise local effects as contextual characteristics within administrative neighbourhoods are not homogeneous across LSOAs. [34] An alternative approach would require the use of non-administrative data to define levels, but the Census is currently the only source from which the data on the fragmentation underlying indicators is available. Fourth, for the underlying indicator 'single people' we used data on legal marital status in 2001 and 2011 and registered civil partnership status in 2011. This has implications for the total numbers of single people, as those who cohabit will be counted as 'non-married' in the calculation of the indicator. Fifth, there were boundary changes following the 2011 Census that may have affected our findings, [35] but only 2.5% of LSOAs were affected and we developed an algorithm to make reasonable population weighted-based estimates for these localities, depending on whether they merged, split or some other change occurred. Finally, the index of social fragmentation may serve as a suitable tool to inform allocation of mental health resources in adults, although recent evidence suggests that social fragmentation may not have an effect on children's mental health.[36]

For peer review only - http://bmjopen.bmj.com/site/about/guidelines.xhtml

Interpretation of findings

A previous study showed that between 1971 and 2001, levels of social fragmentation increased steadily with large increases between 1981 and 1991 and modest increases between 1991 and 2001. Our results indicate that this trend was reversed in 2011 as we found a small decrease in the overall index of social fragmentation between 2001 and 2011. [37] Furthermore, our results suggest that urbanisation drives the high persistence of social fragmentation in England as we found that levels of social fragmentation and its underlying indicators were consistently higher in urban areas. This may be because urban areas are chosen by diverse social and cultural groups and living in these areas may lead to isolated spaces, lifestyles and problems of social integration. Furthermore, we found that the underlying indicators that drive the persistency of social fragmentation appear to be single people and households in private renting, a characteristic of urban environments, [38] as these were the underlying indicators with the largest increases (Figures 3A and 3B).

In absolute terms, private renting was considerably increased in all regions and most notably London; a finding that pertains to unaffordable housing for first-time home buyers and less availability of social housing as a result of the 2008 financial crisis.[39] Similarly, all regions had higher levels of single people in 2011, indicating that numbers of young professionals, students and divorces have increased substantially over the decade. For the other two underlying indicators we did not observe any marked changes across regions, although levels of population turnover appeared to have decreased between the decennial Censuses. The increases we observed in the proportion of single people and those in private renting warrant implications for the prevalence of mental illness especially in urban centres. The private house market is characterised by poor housing conditions as opposed to social houses or owner occupation and this can have detrimental effects on mental health. [40] Furthermore, it is suggested that married individuals may have better mental health outcomes than single individuals. [41] We believe that these changes are certainly of concern as they may highlight the need for more effective social interventions to target socially fragmented areas in England.

Spatial clustering of neighbourhood social fragmentation appears to have increased over time in England while we observed moderate regional variations in levels of clustering despite the small changes in levels of social fragmentation across regions. Increased levels of spatial clustering for social fragmentation across and within regions can have implications for the planning and organisation of health services, as this would imply needs that are not uniformly distributed across a region with the presence of hot spots of high levels of mental illness prevalence, especially in urban areas. To address such spatial health inequalities, services may need to be redesigned locally [42] while infrastructure and spending for mental health needs to be weighted towards social fragmentation hot spots rather than uniformly distributed.

For peer review only - http://bmjopen.bmj.com/site/about/guidelines.xhtml

Conclusion

In the absence of individual-level data, area-level indicators are often the only source of information to investigate how factors associated with mental illness may contribute to population health, albeit when individual-level data are available and are fitted as covariates these area-level associations may be attenuated substantially. Even though, social fragmentation is increasingly considered a determinant of mental illness as more epidemiological research emerges that investigates contextual effects on health. To improve mental health in the population, policymakers need to address both relational and structural issues, perhaps by investing in reorganisation of mental healthcare. Solving the problems of cohesion and integration needs to be seen as a collective responsibility across central and local government and all the local agencies. Policies needs to address social and educational inequalities by strengthening the economic well-being of areas by lowering local unemployment rates, encouraging populations to bring social support in the communities and encouraging education of the population, which are key strategies for healthy communities. Communities need to work towards tightening social networks and social bonds to help with mental illness prevalence and suicide prevention, for example by promoting participation in charity, voluntary or community groups and civic engagement. The spatial aspect of social fragmentation is often overlooked, but it can provide vital information for the effective organisation of health services and targeting of health or social interventions. The nature of fragmentation and the mechanisms underlying its association with mental illness are of increasing interest as the population gets older and the health care costs associated with mental illness escalate in industrialised countries.

For peer review only - http://bmjopen.bmj.com/site/about/guidelines.xhtml

Acknowledgments

We would like to thank the Office of National Statistics for the wealth of information they have collected and systematically organised, which made this study possible.

Contributors

EK, CG, DA and LM designed the study. CG extracted the data from all sources, performed the analyses and drafted the manuscript. EK, RG, DA, LM, NK critically revised the manuscript. CG is the guarantor of this work and, as such, had full access to all the data in the study and takes responsibility for the integrity of the data and the accuracy of the data analysis.

Funding

This study is funded by the National Institute for Health Research School for Primary Care Research (NIHR SPCR), through CG's PhD. This report is independent research by the National Institute for Health Research. LM acknowledges financial support from the MRC Skills Development Fellowship (MR/N015126/1). The views expressed in this publication are those of the authors and necessarily those of the NHS, the National Institute for Health Research, the Department of Health, or the MRC.

Competing interests

None declared.

Provenance and peer review

Not commissioned; externally peer reviewed

Data sharing agreement

The data used in this study are freely available and the authors are happy to share in an organised and cleaned final dataset.

Ethical approval and Patient and Public Involvement

Not applicable. The study uses freely reported data aggregates at low geography levels. No patients or members of the public were involved in the study.

Open access

This is an Open Access article distributed in accordance with the terms of the Creative Commons Attribution (CC BY 4.0) license, which permits others to distribute, remix, adapt and build upon this work, for commercial use, provided the original work is properly cited. See: http://creativecommons.org/licenses/by/4.0/ © Article author(s) (or their employer(s) unless

For peer review only

otherwise stated in the text of the article) 2018. All rights reserved. No commercial use is permitted unless otherwise expressly granted.

For peer review only

For peer review only - http://bmjopen.bmj.com/site/about/guidelines.xhtml

Table 1 Temporal changes in social fragmentation metrics across regions, 2001 - 2011

	North East	North West	Yorkshire and Humber	East Midlands	West Midlands	East England	London	South East Coast	South Central	South West	Englan
Social Fragmentation 2001											
No. of LSOAs	1,656	4,459	3,293	2,732	3,482	3,550	4,765	2,750	2,569	3,226	32,482
25th Centile	-2.32	-2.34	-2.31	-2.59	-2.59	-2.50	-0.46	-2.37	-2.51	-2.09	-2.26
Median	-0.79	-0.63	-0.87	-1.34	-1.19	-1.27	2.05	-1.09	-1.24	-0.88	-0.75
75th Centile	0.82	1.45	0.91	0.47	0.44	0.37	4.45	0.86	0.76	0.89	1.46
Weighted mean	-0.29	-0.06	-0.16	-0.55	-0.71	-0.68	2.20	-0.21	-0.33	-0.18	0.03
Standard deviation	3.26	3.50	3.50	3.24	2.94	2.75	3.28	3.24	3.16	3.28	3.32
Social Fragmentation 2011											
No. of LSOAs	1,657	4,497	3,317	2,774	3,487	3,614	4,835	2,773	2,609	3,281	32,844
25th Centile	-2.16	-2.31	-2.30	-2.64	-2.45	-2.59	-0.64	-2.50	-2.63	-2.17	-2.29
Median	-0.58	-0.51	-0.78	-1.27	-1.01	-1.30	1.58	-1.19	-1.27	-0.86	-0.70
75th Centile	0.97	1.62	1.07	0.58	0.65	0.39	3.98	0.76	0.62	1.02	1.43
Weighted mean	-0.01	0.16	0.04	-0.43	-0.45	-0.67	1.83	-0.25	-0.41	0.02	0.09
Standard deviation	3.13	3.29	3.37	3.22	2.82	2.81	3.56	3.34	3.28	3.33	3.37

For peer review only

For peer review only - http://bmjopen.bmj.com/site/about/guidelines.xhtml

Figure 1 Changes in overall social fragmentation for England, 2001 (left) – 2011 (right)

Table 1 Temporal changes in social fragmentation metrics across regions, 2001 - 2011

Figure 2 Changes in overall social fragmentation for London, 2001 (left) – 2011 (right)

Figure 3 Percentage of single people (top) and one person households (bottom) for English regions, over time (* Red line indicates mean for England in 2011)

Figure 4 Percentage of private renting (top) and population turnover (bottom) for English regions, over time (* Red line indicates mean for England in 2011)

Figure 5 Spatial Autocorrelation with Moran's I for Social Fragmentation by region, over time

For peer review only

For peer review only - http://bmjopen.bmj.com/site/about/guidelines.xhtml

References

1. Pickett KE, Pearl M. Multilevel analyses of neighbourhood socioeconomic context and health outcomes: a critical review. *J Epidemiol Ccommunity Health* 2001;55(2):111-22.
2. Stafford M, Marmot M. Neighbourhood deprivation and health: does it affect us all equally? *Int J Epidemiol* 2003;32(3):357-66.
3. Fagg J, Curtis S, Stansfeld SA, Cattell V, Tupuola AM, Arephin M. Area social fragmentation, social support for individuals and psychosocial health in young adults: evidence from a national survey in England. *Soc Sci Med* 2008;66(2):242-54.
4. Stafford M, De Silva M, Stansfeld S, Marmot M. Neighbourhood social capital and common mental disorder: testing the link in a general population sample. *Health Place* 2008;14(3):394-405.
5. Subramanian SV, Lochner KA, Kawachi I. Neighborhood differences in social capital: a compositional artifact or a contextual construct? *Health Place* 2003;9(1):33-44.
6. Veenstra G, Luginaah I, Wakefield S, Birch S, Eyles J, Elliott S. Who you know, where you live: social capital, neighbourhood and health. *Soc Sci Med* 2005;60(12):2799-818.
7. Congdon P. Suicide and parasuicide in London: A small-area study. *Urban Stud* 1996;33(1):137-58.
8. Congdon P. Commentary: Contextual effects: index construction and technique. *Int J Epidemiol* 2004;33(4):741-2.
9. Understanding patterns of deprivation. *Regional Trends* 2009;41(1):93-114.
10. Whitley E, Gunnell D, Dorling D, Smith GD. Ecological study of social fragmentation, poverty, and suicide. *BMJ* 1999;319(7216):1034-7.
11. Fagg J, Curtis S, Stansfeld S, Congdon P. Psychological distress among adolescents, and its relationship to individual, family and area characteristics in East London. *Soc Sci Med* 2006;63(3):636-48.
12. Fone D, Dunstan F, Lloyd K, Williams G, Watkins J, Palmer S. Does social cohesion modify the association between area income deprivation and mental health? A multilevel analysis. *Int J Epidemiol* 2007;36(2):338-45.
13. Evans J, Middleton N, Gunnell D. Social fragmentation, severe mental illness and suicide. *Soc Psychiatry Psychiatr Epidemiol* 2004;39(3):165-70.
14. Ivory VC, Collings SC, Blakely T, Dew K. When does neighbourhood matter? Multilevel relationships between neighbourhood social fragmentation and mental health. *Soc Sci Med* 2011;72(12):1993-2002.
15. Allardyce J, Boydell J. Review: the wider social environment and schizophrenia. *Schizophr Bull* 2006;32(4):592-8.
16. Ellen IG, Mijanovich T, Dillman K-N. Neighborhood Effects on Health: Exploring the Links and Assessing the Evidence. *J Urban Aff* 2001;23(3-4):391-408.
17. Collings S, Ivory V, Blakely T, Atkinson J. Are neighbourhood social fragmentation and suicide associated in New Zealand? A national multilevel cohort study. *J Epidemiol Community Health* 2009;63(12):1035-42.
18. Smith GD, Whitley E, Dorling D, Gunnell D. Area based measures of social and economic circumstances: cause specific mortality patterns depend on the choice of index. *J Epidemiol Community Health* 2001;55(2):149-50.
19. Corcoran P, Arensman E, Perry IJ. The area-level association between hospital-treated deliberate self-harm, deprivation and social fragmentation in Ireland. *J Epidemiol Community Health* 2007;61(12):1050-55.

For peer review only - http://bmjopen.bmj.com/site/about/guidelines.xhtml

20. Evans E, Hawton K, Rodham K. Factors associated with suicidal phenomena in adolescents: a systematic review of population-based studies. *Clin Psychol Rev* 2004;24(8):957-79.
21. Curtis S, Copeland A, Fagg J, Congdon P, Almog M, Fitzpatrick J. The ecological relationship between deprivation, social isolation and rates of hospital admission for acute psychiatric care: a comparison of London and New York City. *Health Place* 2006;12(1):19-37.
22. Allardyce J, Gilmour H, Atkinson J, Rapson T, Bishop J, McCreadie RG. Social fragmentation, deprivation and urbanicity: relation to first-admission rates for psychoses. *Br J Psychiatry* 2005;187:401-06.
23. van Os J, Driessen G, Gunther N, Delespaul P. Neighbourhood variation in incidence of schizophrenia. Evidence for person-environment interaction. *Br J Psychiatry* 2000;176:243-8.
24. Stjarne MK, Ponce de Leon A, Hallqvist J. Contextual effects of social fragmentation and material deprivation on risk of myocardial infarction--results from the Stockholm Heart Epidemiology Program (SHEEP). *Int J Epidemiol* 2004;33(4):732-41.
25. Stafford M, Gimeno D, Marmot MG. Neighbourhood characteristics and trajectories of health functioning: a multilevel prospective analysis. *Eur J Public Health* 2008;18(6):604-10.
26. Jarvis GK, Ferrence RG, Whitehead PC, Johnson FG. The ecology of self-injury: a multivariate approach. *Suicide Llife Threat Behav* 1982;12(2):90-102.
27. Office for National Statistics. Families and Households in England and Wales 2011: Statistical Bulletin. 2013.
28. Office for National Statistics.Nomis official labour market statistics. 2013.
29. Office for National Statistics. Super Output Area mid-year population estimates for England and Wales, Mid-2011. 2016.
30. Bibby P, Shepherd J. Developing a new classification of urban and rural areas for policy purposes–the methodology. *London: DEFRA* 2004.
31. Moran PAP. Notes on Continuous Stochastic Phenomena. *Biometrika* 1950;37(1-2):17-23.
32. Anselin L. Local indicators of spatial association—LISA. *Geogr Anal* 1995;27(2):93-115.
33. Fewell Z, Smith GD, Sterne JAC. The impact of residual and unmeasured confounding in epidemiologic studies: A simulation study. *Am J Epidemiol* 2007;166(6):646-55.
34. Rehkopf DH, Buka SL. The association between suicide and the socio-economic characteristics of geographical areas: a systematic review. *Psychol Med* 2006;36(2):145-57.
35. Norman P, Rees P, Boyle P. Achieving data compatibility over space and time: creating consistent geographical zones. *Pop Space Place* 2003;9(5):365-86.
36. Flouri E, Midouhas E, Joshi H, Sullivan A. Neighbourhood social fragmentation and the mental health of children in poverty. *Health Place* 2015;31:138-45.
37. Dorling D, Vickers D, Thomas B, Pritchard J, Ballas D. Changing UK. *The way we live now. Sheffield: University of Sheffield* 2008.
38. Smith CW. 2011 Census Analysis: How Have Living Arrangements and Marital Status in England and Wales Changed Since 2001. *Office for National Statistics* 2011.
39. Kemp PA. Private Renting After the Global Financial Crisis. *Housing Stud* 2015;30(4):601-20.
40. Barnes M, Cullinane, C., Scott, S. & Silvester, S. . People living in bad housing: Numbers and health impacts. *UK: Shelter.* 2013.
41. Simon RW. Revisiting the relationships among gender, marital status, and mental health. *Am J Sociol* 2002;107(4):1065-96.
42. Lorant V, Thomas I, Deliege D, Tonglet R. Deprivation and mortality: the implications of spatial autocorrelation for health resources allocation. *Soc Sci Med* 2001;53(12):1711-9.

For peer review only - http://bmjopen.bmj.com/site/about/guidelines.xhtml

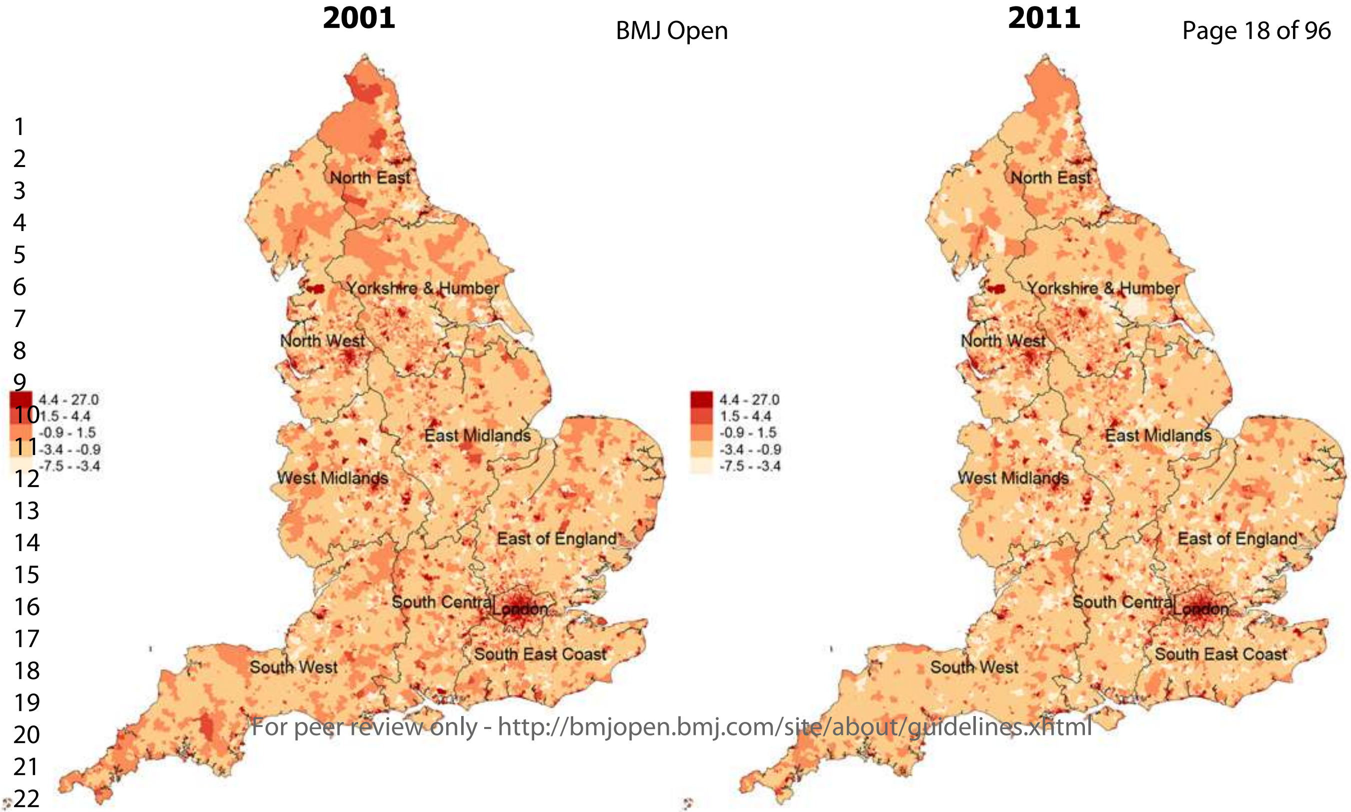

For peer review only - http://bmjopen.bmj.com/site/about/guidelines.xhtml

2001

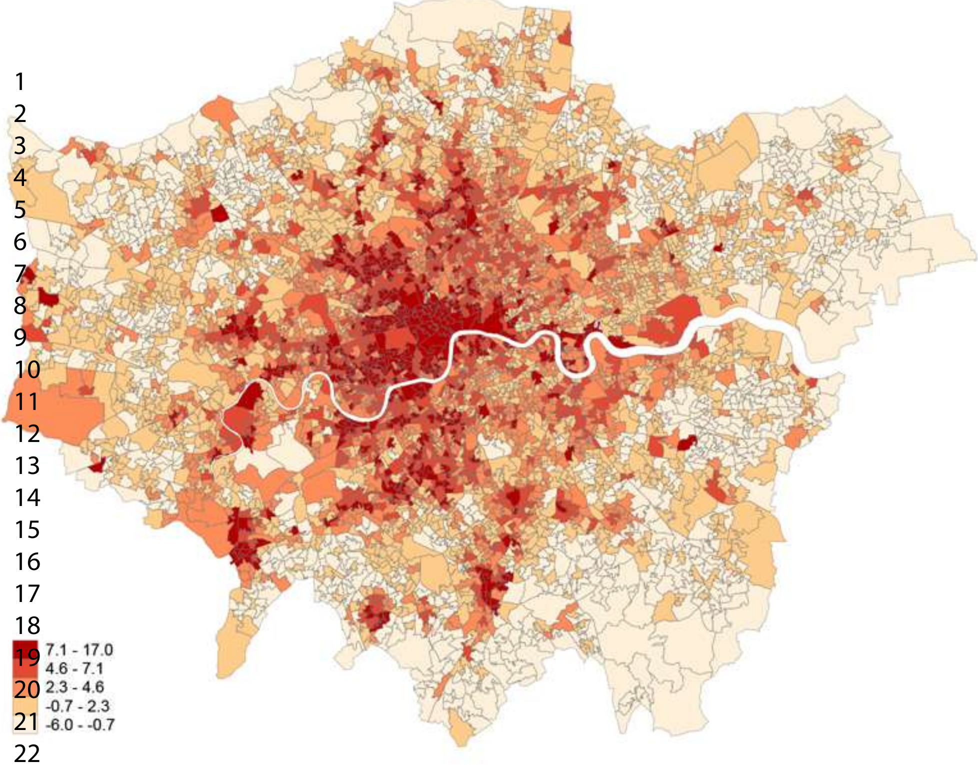

2011

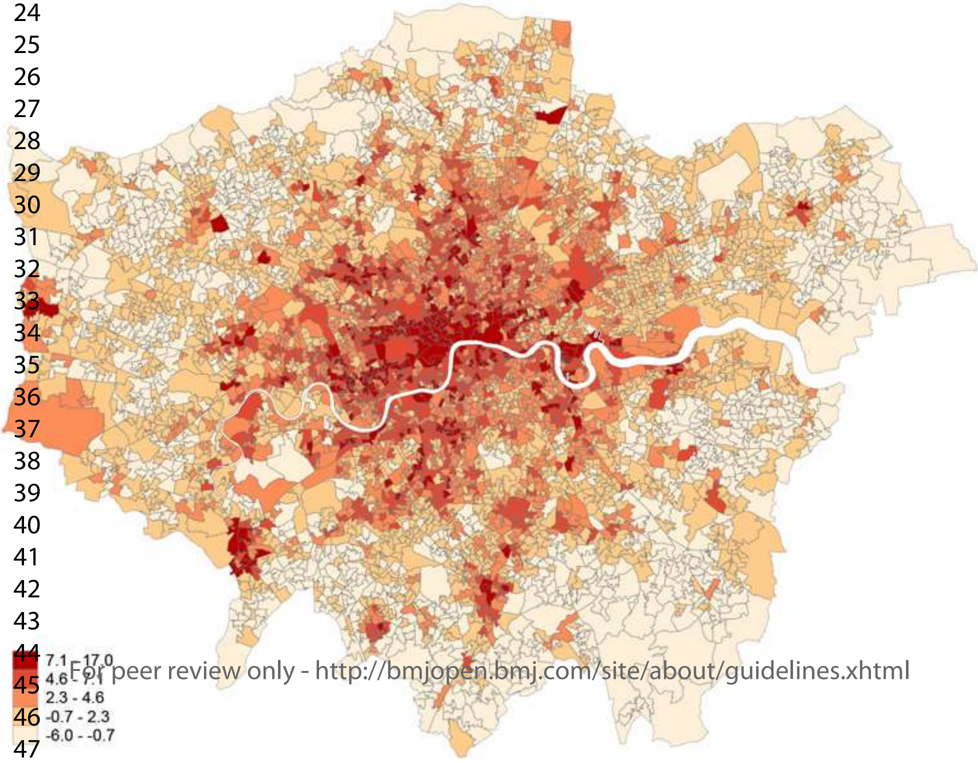

For peer review only - http://bmjopen.bmj.com/site/about/guidelines.xhtml

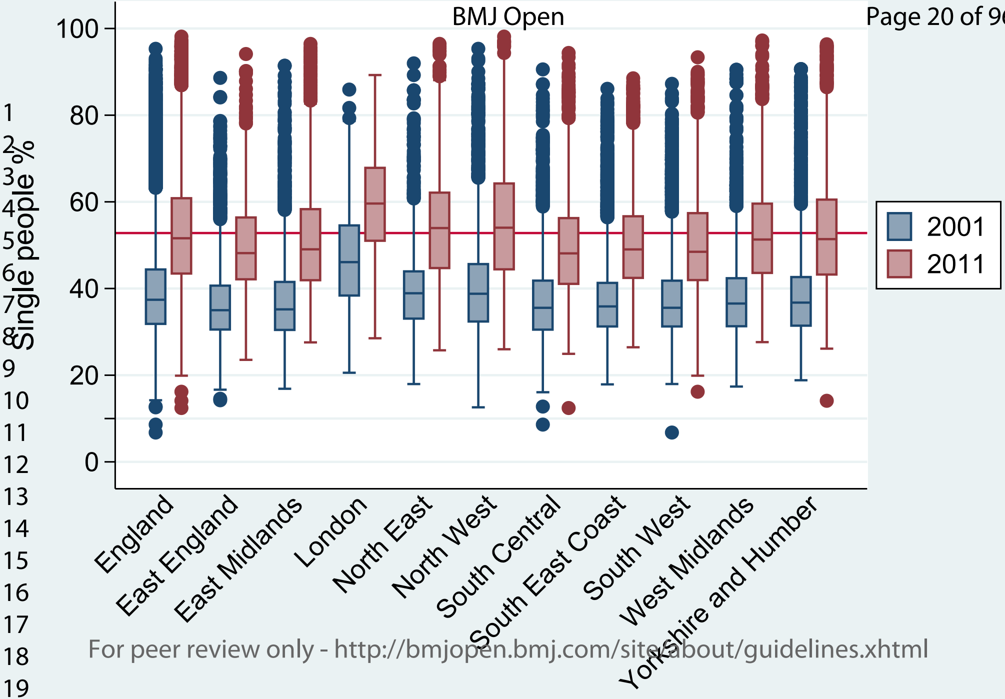

For peer review only - http://bmjopen.bmj.com/site/about/guidelines.xhtml

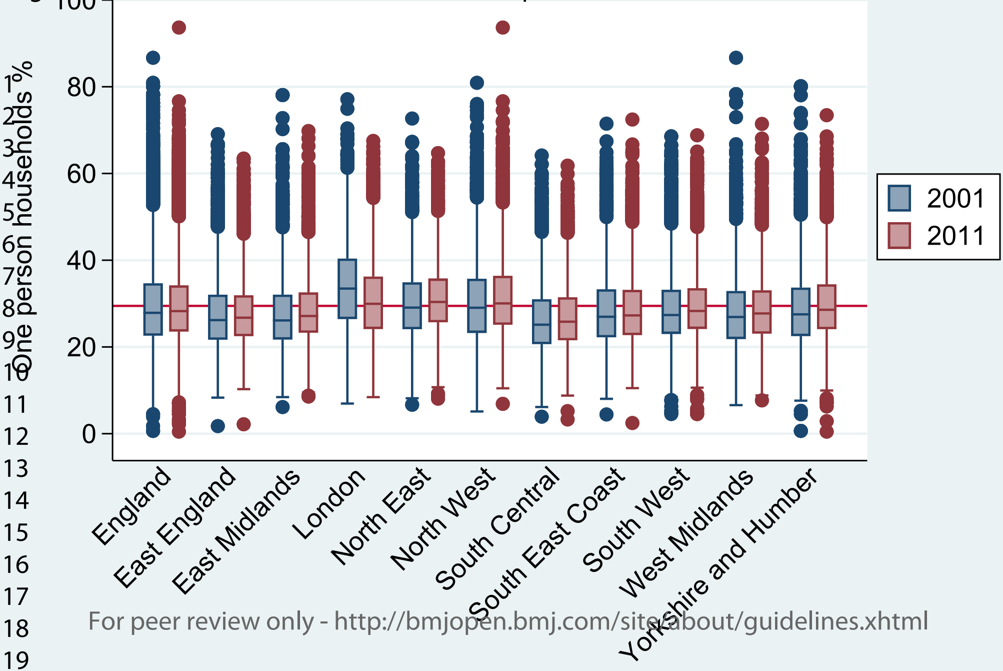
One person households %
100
80
60
40
20
0
England
East England
East Midlands
London
North East
North West
South Central
South East Coast
South West
West Midlands
Yorkshire and Humber
2001
2011

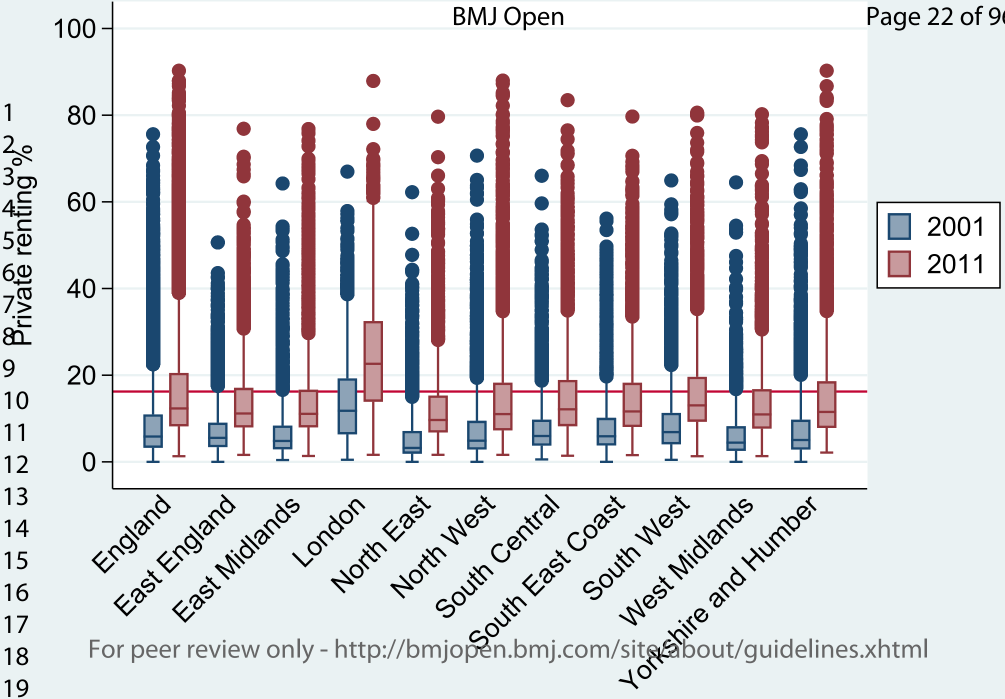

For peer review only - http://bmjopen.bmj.com/site/about/guidelines.xhtml

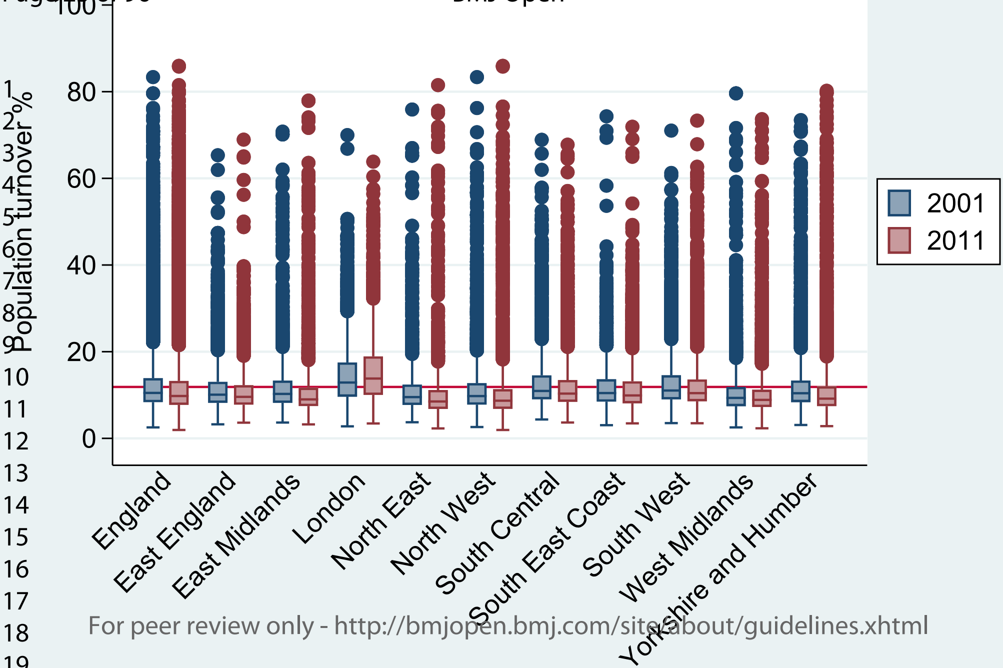

For peer review only - http://bmjopen.bmj.com/site/about/guidelines.xhtml

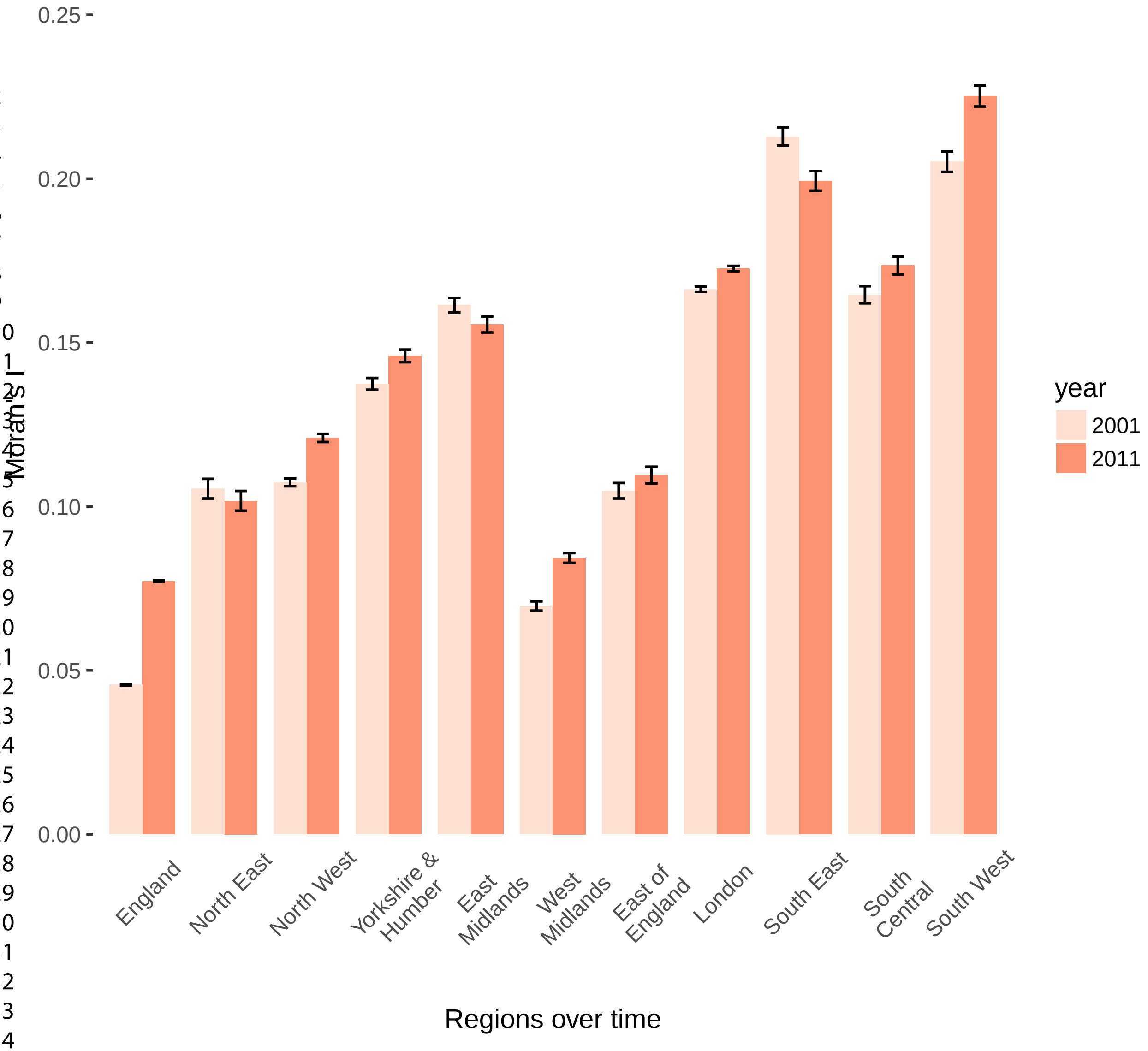
0.25
0.20
0.15
0.10
0.05
0.00
Moran's I
year
2001
2011
England
North East
North West
Yorkshire & Humber
East Midlands
West Midlands
East of England
London
South East
South Central
South West
Regions over time

Supplementary Appendix

Spatial distribution and temporal trends in social fragmentation in England, 2001 to 2011: a national study

For peer review only

For peer review only - http://bmjopen.bmj.com/site/about/guidelines.xhtml

Contents

Description of data and formulas used for the calculation of the underlying social fragmentation indicators at both time points 3

Spatial weighted regressions to attribute 2001 data to 2011 LSOAs 4

Spatial Maps 4

Figure A: Index of Social Fragmentation (mean) for England and English regions, over time. 5

Figure A1 Spatial autocorrelation with Moran's I for single persons (top) and one person households (bottom), by region and over time 6

Figure A2 Spatial autocorrelation with Moran's I for private renting (top) and population turnover (bottom), by region and over time 7

Figure B1: Local Moran scatterplot for social fragmentation at the LSOA level, 2001 8

Figure B2: Local Moran scatterplot for social fragmentation at the LSOA level, 2011 8

Results from LISA analysis: 9

References 10

For peer review only - http://bmjopen.bmj.com/site/about/guidelines.xhtml

Description of data and formulas used for the calculation of the underlying social fragmentation indicators at both time points

2001

Percentage of single people in LSOA = All singles, divorced, widowed and separated in LSOA / All usual residents aged 16 and above in LSOA (dataset available from: *https://www.nomisweb.co.uk/census/2001/ks004)*

Percentage of one person households in LSOA = Total one person households in LSOA/ All household composition in LSOA (dataset available from: *https://www.nomisweb.co.uk/census/2001/uv065*)

Percentage of private renting in LSOA = Total houses rented from a private landlord or letting agent in LSOA/ All household composition in LSOA (dataset available from: *https://www.nomisweb.co.uk/census/2001/uv063)*

Percentage of population turnover in LSOA= People who are migrants (i.e. moved into the area from within the UK & moved into the area from outside the UK / All usual residents in LSOA (dataset available from: *https://www.nomisweb.co.uk/census/2001/ks024*)

2011

Percentage of single people in LSOA = All singles, divorced, widowed and separated in LSOA / All usual residents aged 16 and above in LSOA (dataset available from: *https://www.nomisweb.co.uk/census/2011/ks103uk*)

Percentage of one person households in LSOA = Total one person households in LSOA/ All household composition in LSOA (dataset available from: *https://www.nomisweb.co.uk/census/2011/ks105ew*)

Percentage of private renting in LSOA = Total houses rented from a private landlord or letting agent in LSOA/ All household composition in LSOA (dataset available from: *https://www.nomisweb.co.uk/census/2011/ks402ew*)

Percentage of population turnover in LSOA= People who are migrants (i.e. moved into the area from within the UK & moved into the area from outside the UK / All usual residents in LSOA (dataset available from: *https://www.nomisweb.co.uk/census/2011/ukmig008*)

For peer review only

For peer review only - http://bmjopen.bmj.com/site/about/guidelines.xhtml

Spatial weighted regressions to attribute 2001 data to 2011 LSOAs

The 2003 National Statistics policy to minimise the statistical impact of frequent electoral ward boundary changes was succeeded by boundary changes across LSOAs in 2011. In that year, there was a modification of OA (Output Areas) and SOA (Super Output Areas) in England & Wales mainly due to significant population changes between the decennial censuses. As social fragmentation in 2001 was measured in 2001 LSOAs, we attributed census 2001 LSOA information to 2011 LSOAs and we used population weighted regressions to estimate 2001 data at the 2011 LSOA to enable seamless comparisons across time. More specifically, 97.4% of all LSOAs remained unchanged in 2011, and for these areas we took no action and attributed the social fragmentation values from 2001 to the 2011 LSOAs. If two or more 2001 LSOAs merged in one LSOA in 2011 (0.6% of all LSOAs), we calculated the mean value of social fragmentation weighted for 2003 LSOA population (which is the oldest population estimates dataset provided by ONS). If a 2001 LSOA split into two or more 2011 LSOAs (1.8% of all LSOAs) we assigned the relevant 2001 social fragmentation scores. Finally, there were some LSOAs for which changes did not match any of the previously described patterns (i.e. an LSOA split in two and each part merged with a different LSOA). For these very few LSOAs (0.1% of all LSOAs) we developed an algorithm to calculate population weighted mean estimates. (1) The corresponding algorithm is available from the authors.

Spatial Maps

Digital vector boundaries for the 2011 LSOAs, generalised to 20 metres and clipped to the coastline to reduce size and improve visualisation, were obtained from the ONS open geography portal (2) . The vector boundaries were inputted in the Stata shp2dta command to calculate the centroid for each LSOA in the British National Grid format (3). These were then converted from British National Grid easting and northing to longitude and latitude in degrees (4).

For peer review only - http://bmjopen.bmj.com/site/about/guidelines.xhtml

Figure A: Index of Social Fragmentation (mean) for England and English regions, over time.

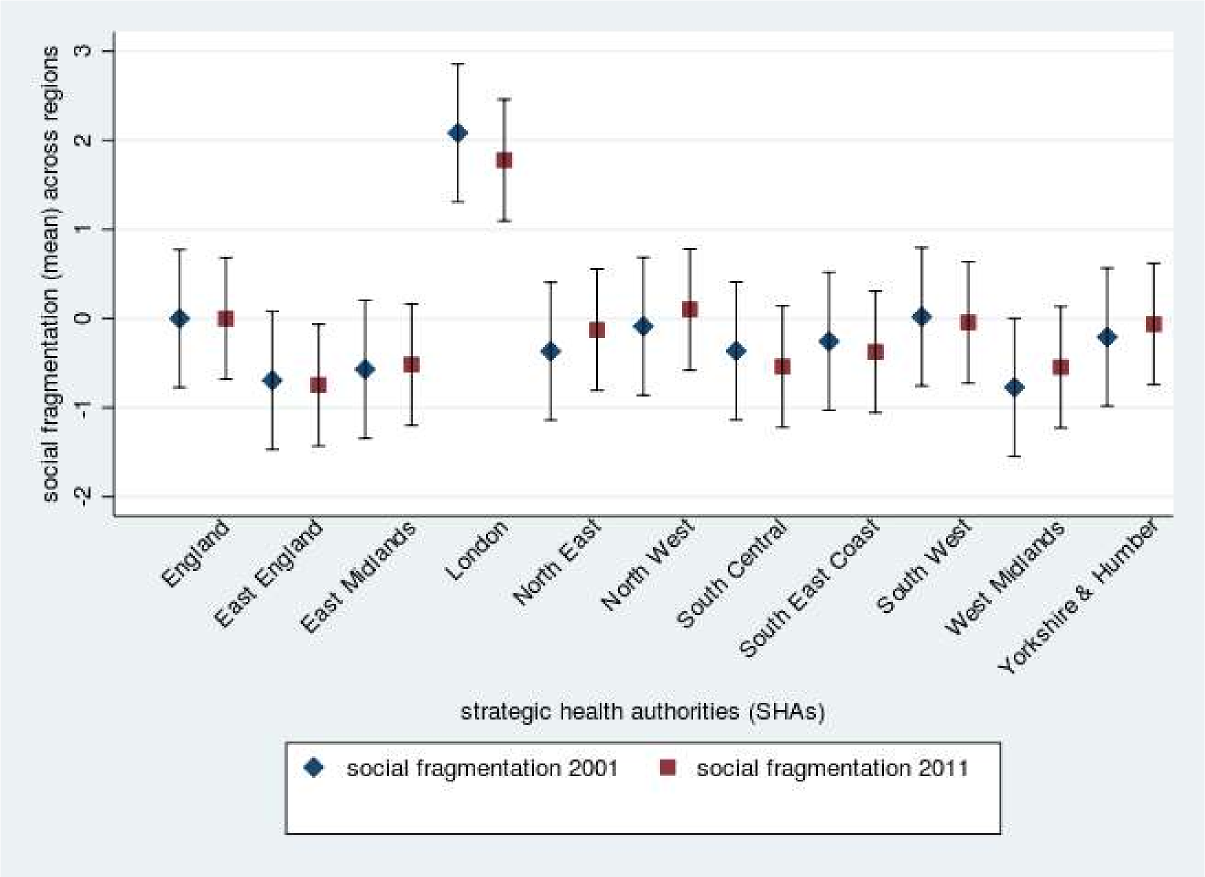

For peer review only - http://bmjopen.bmj.com/site/about/guidelines.xhtml

Figure A1: Spatial autocorrelation with Moran's I for single persons (top) and one person households (bottom), by region and over time

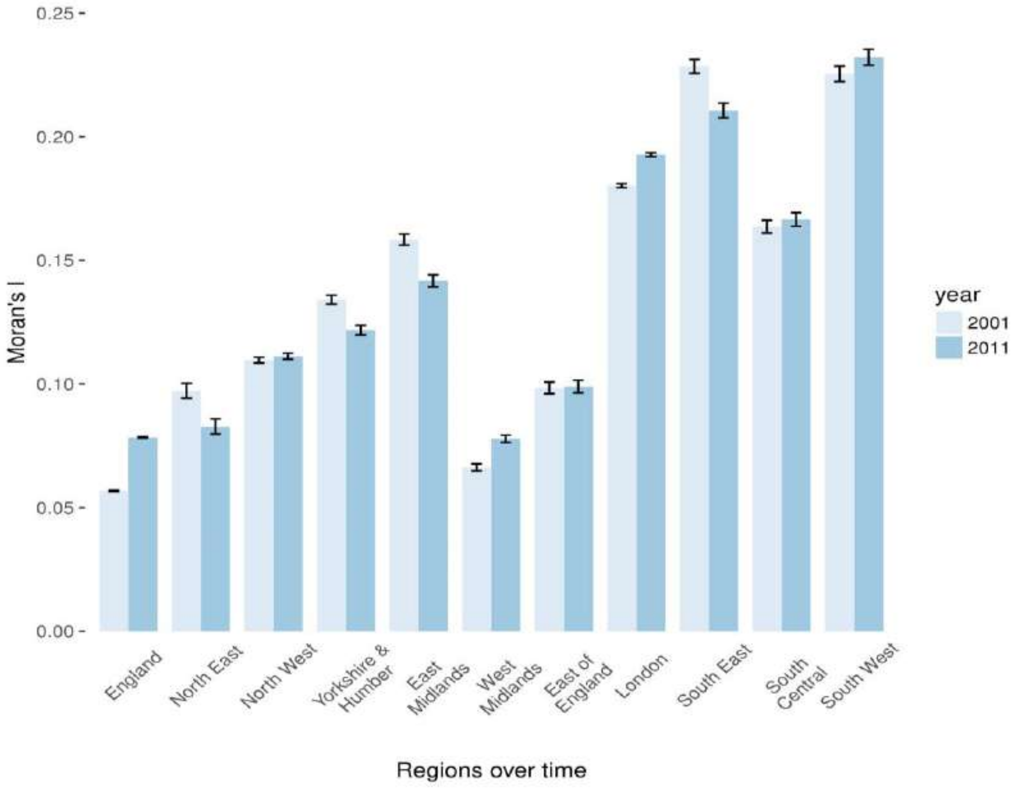

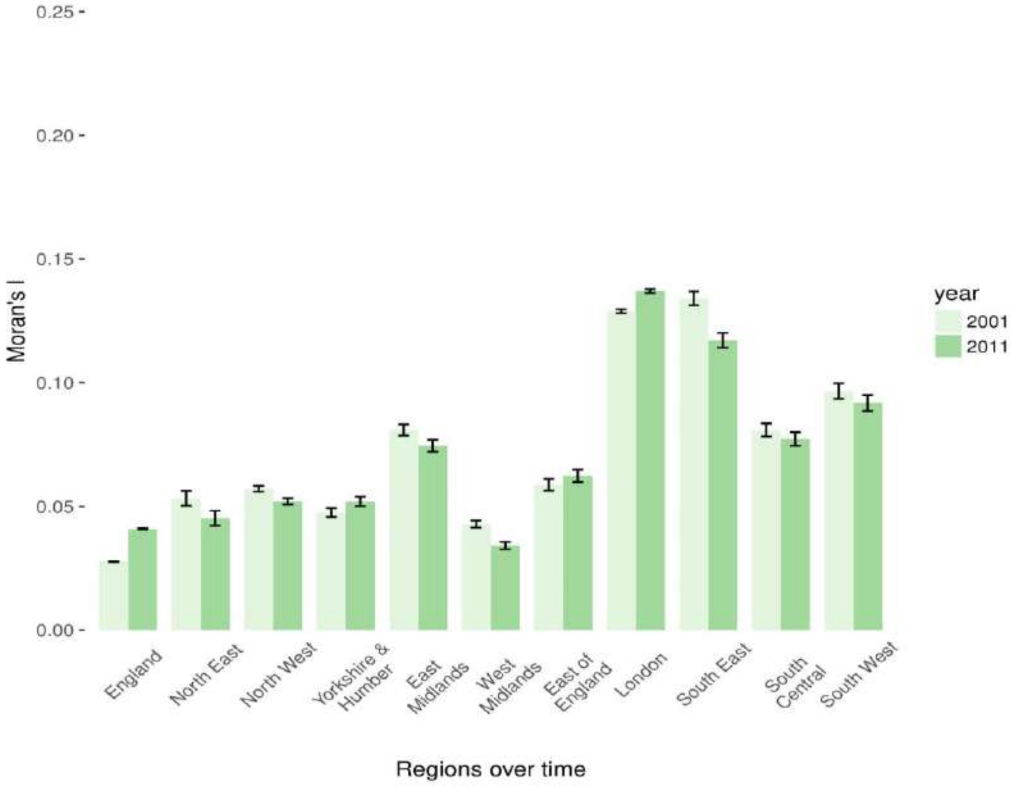

Figure A2: Spatial autocorrelation with Moran's I for private renting (top) and population turnover (bottom), by region and over time

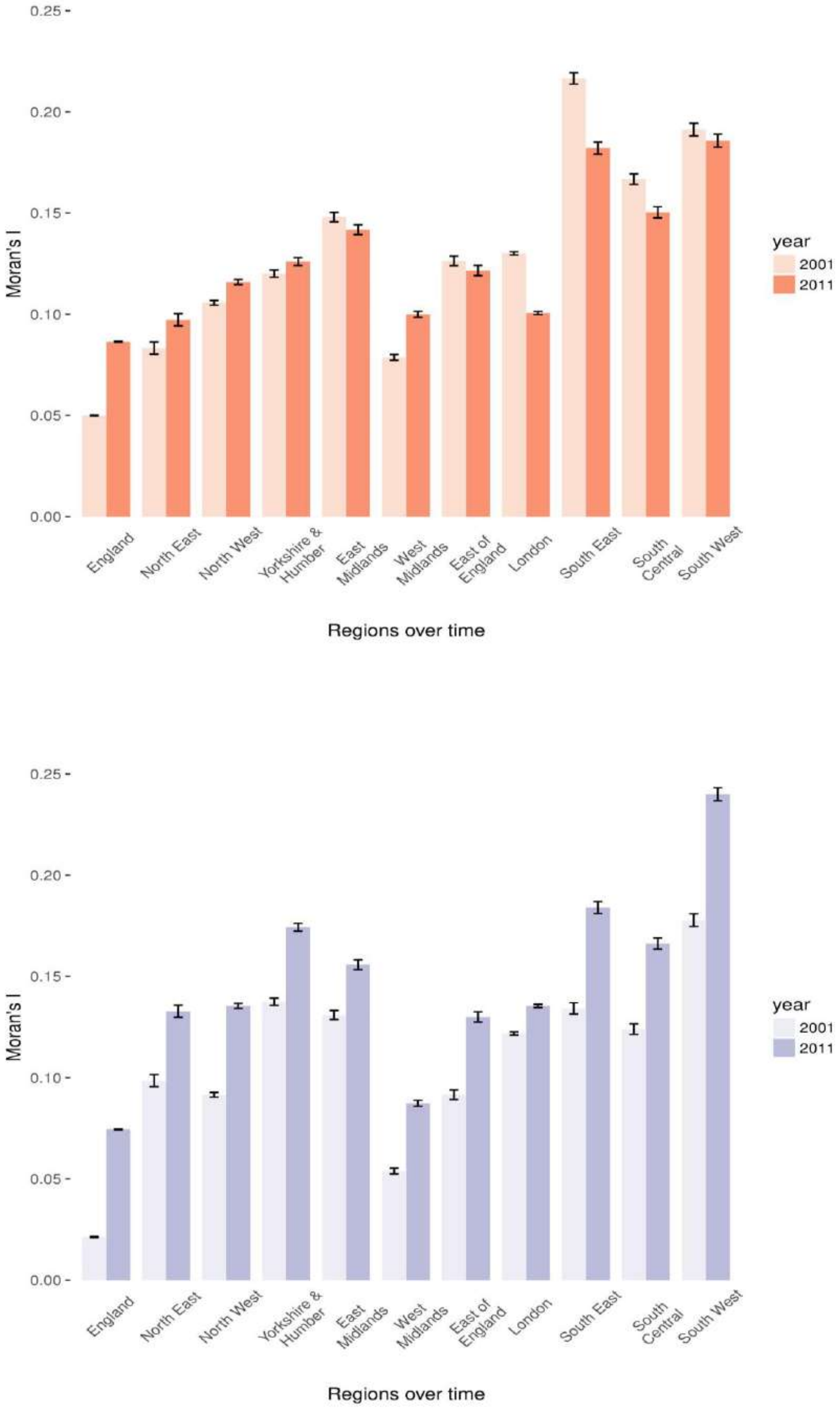

For peer review only - http://bmjopen.bmj.com/site/about/guidelines.xhtml

Figure B1: Local Moran scatterplot for social fragmentation at the LSOA level, 2001.

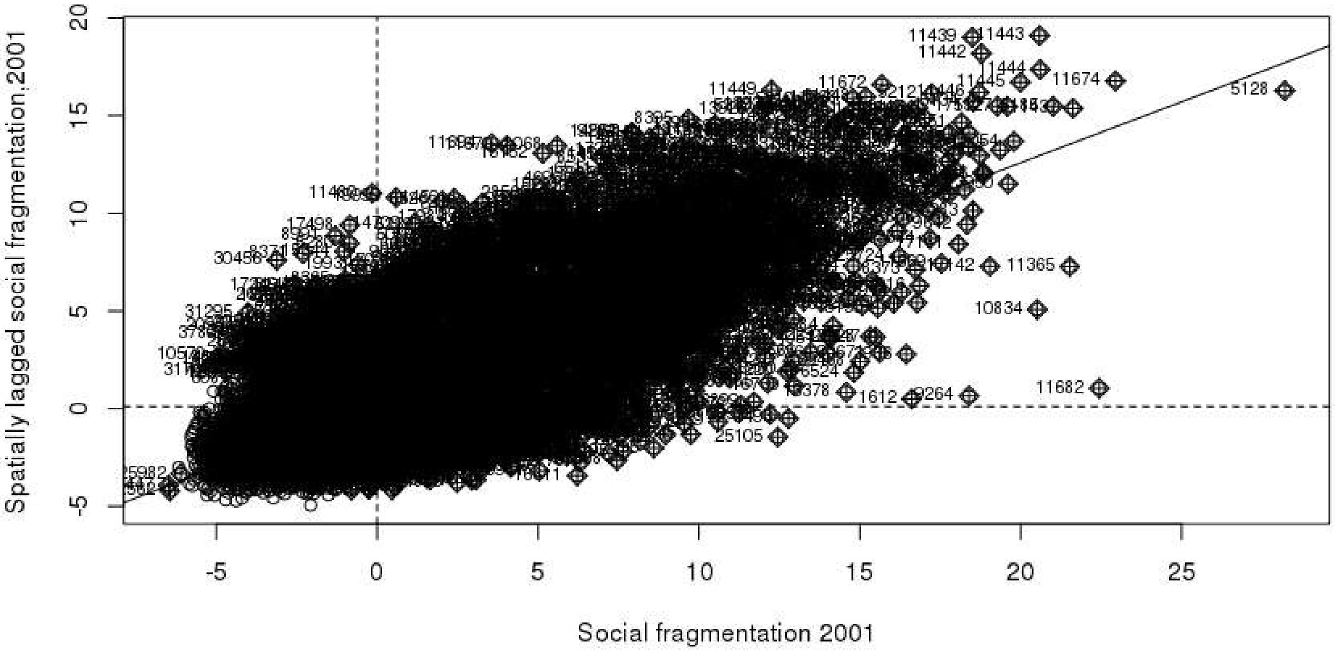

Figure B2: Local Moran scatterplot for social fragmentation at the LSOA level, 2011.

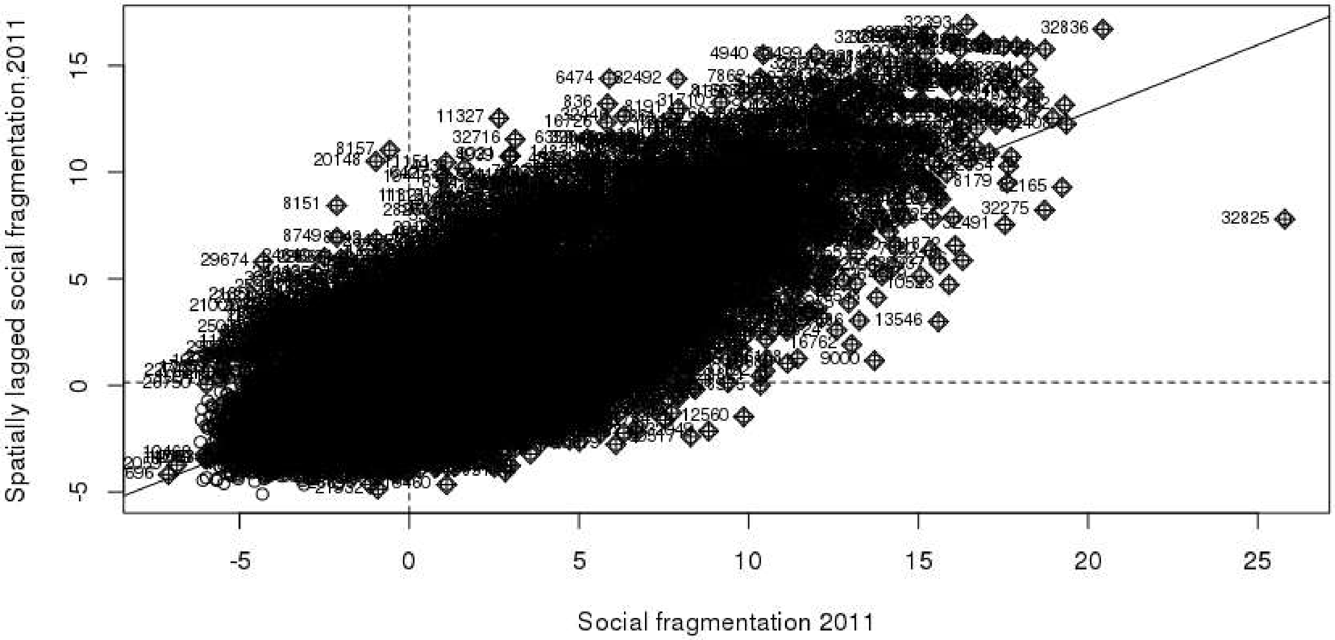

For peer review only - http://bmjopen.bmj.com/site/about/guidelines.xhtml

Results from LISA analysis: The Moran scatterplots for social fragmentation in 2001 and 2011 are provided in Figures B1 and B2 respectively. The four quadrants in each scatterplot correspond to four types of association. The lower left and upper quadrants indicate positive spatial autocorrelation, or that areas in these quadrants have similar values (lower than the mean for those areas in the lower left quadrant and higher than the mean in the upper left quadrant). The upper left and lower right quadrant indicate spatial associations of areas with dissimilar values in social fragmentation. In both graphs the slope of the regression line indicates that there is a positive spatial association at both time points with a handful of outliers in 2001 and only a few in 2011. The pattern of increasing spatial autocorrelation across the two time points that we observed in graph 4 for global spatial autocorrelation is also confirmed by comparison of Graphs B1 and B2. Spatial autocorrelation appears to have increased in 2011 when compared to 2001. At both time points, LSOAs were mainly concentrated in the upper right quadrant (Figure B1 & B2) indicating the existence of many hot spots of social fragmentation and highlighting the fact that there are important variations among LSOAs and a finer scale spatial structure that occur below the region level.

For peer review only

For peer review only - http://bmjopen.bmj.com/site/about/guidelines.xhtml

References

1. Office for National Statistics. Changes to output areas and super output areas in England and Wales, 2001 to 2011. 2012.
2. Office for National Statistics. Open Geography Portal. 2013. https://geoportal.statistics.gov.uk/geoportal/.
3. SHP2DTA:. Stata module to converts shape boundary files to Stata datasets [program]. S456718 version: Boston College Department of Economics,. 2006.
4. Ordance Survey. A guide to coordinate systems in Great Britain. ONS, 2013:43. www.ordnancesurveycouk/docs/support/guide-coordinate-systems-great-britainpdf.

For peer review only

Social Fragmentation

England 2001, Social Fragmentation

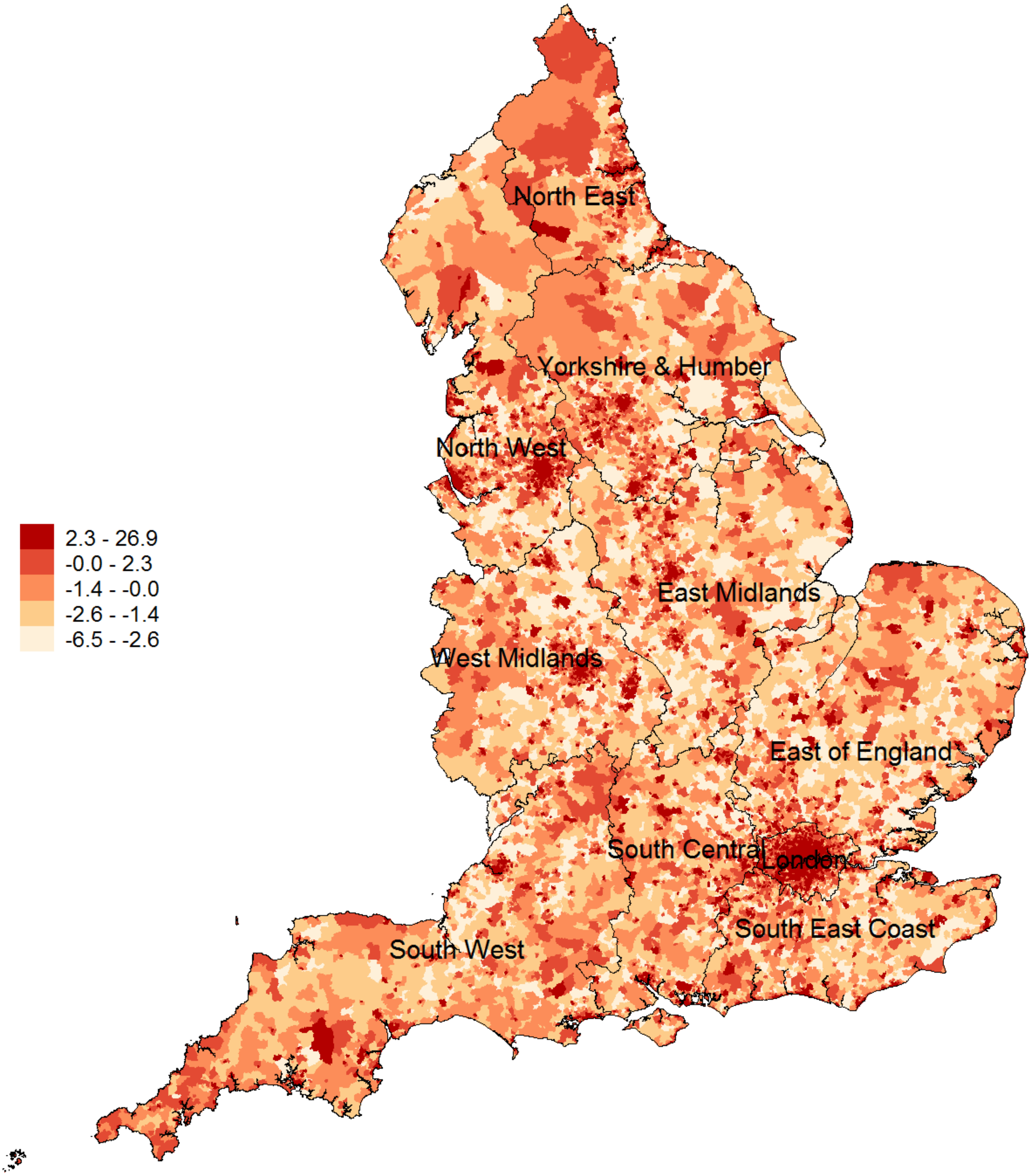

England 2011, Social Fragmentation

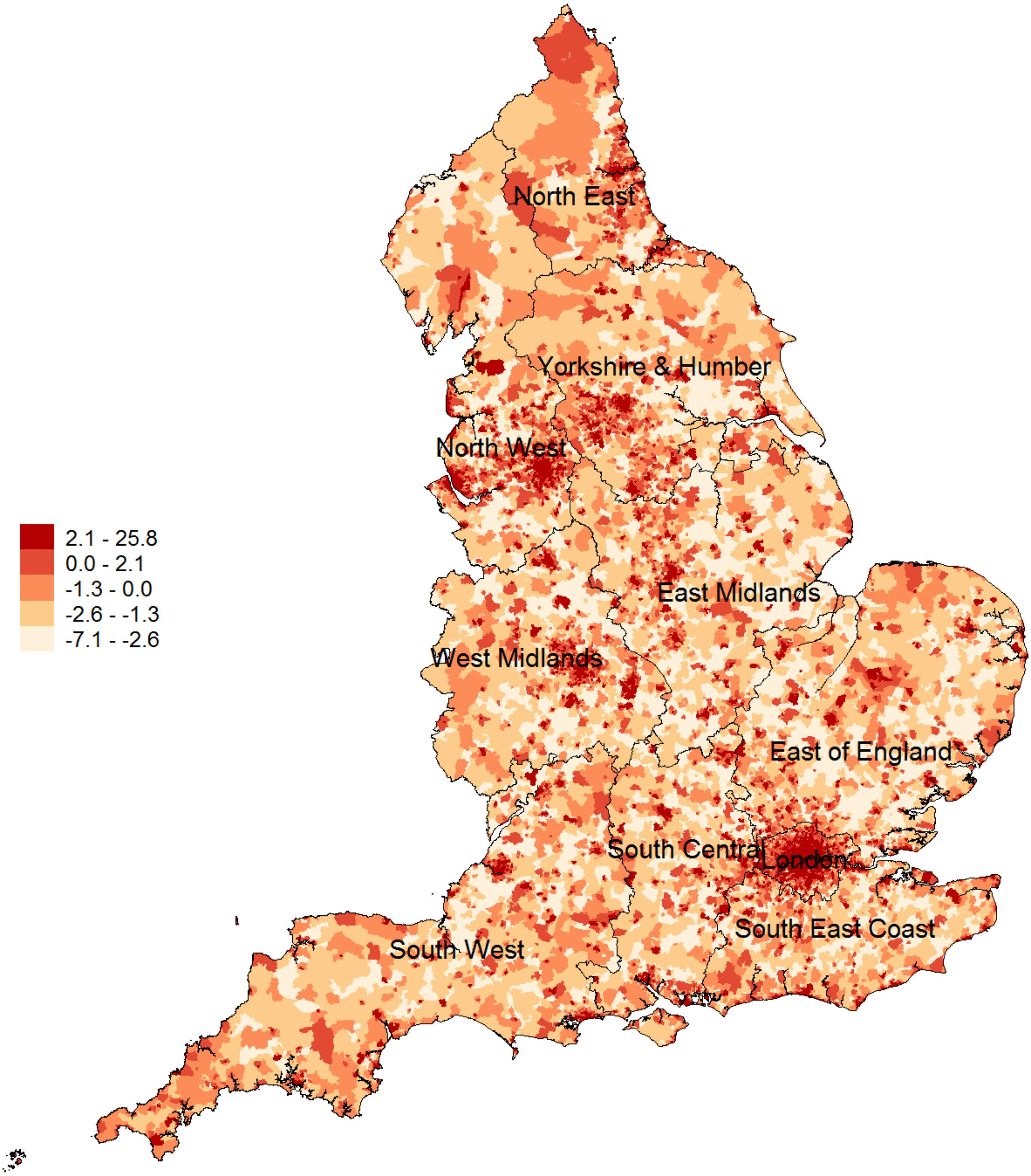

North East, Social Fragmentation

2001

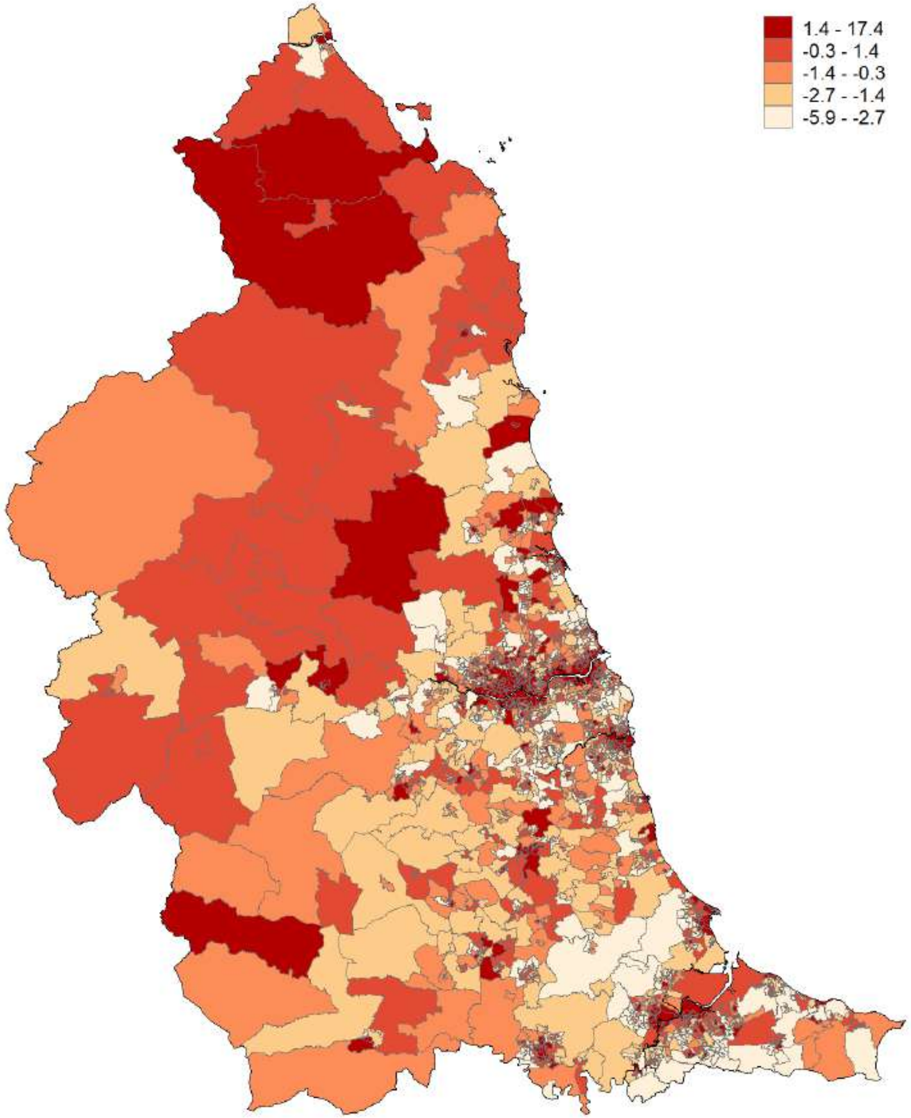

2011

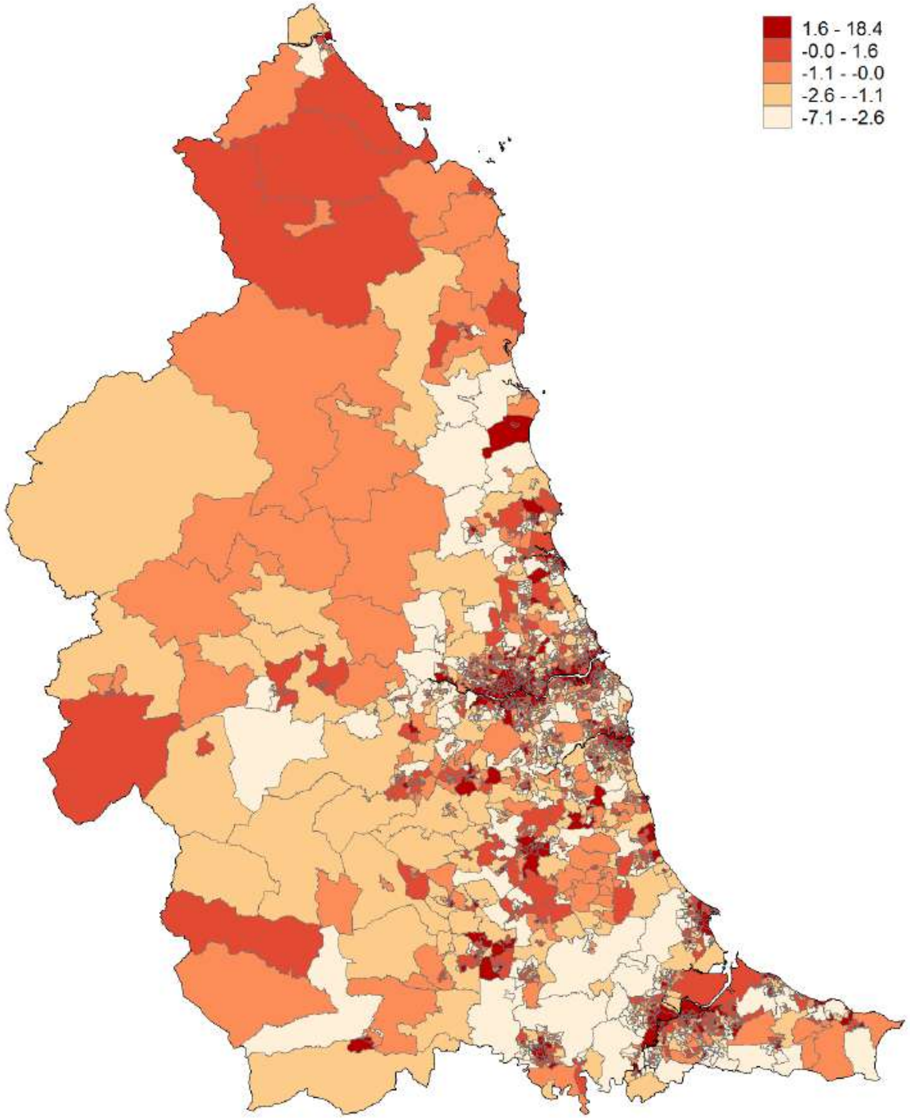

North West, Social Fragmentation

2001 | 2011

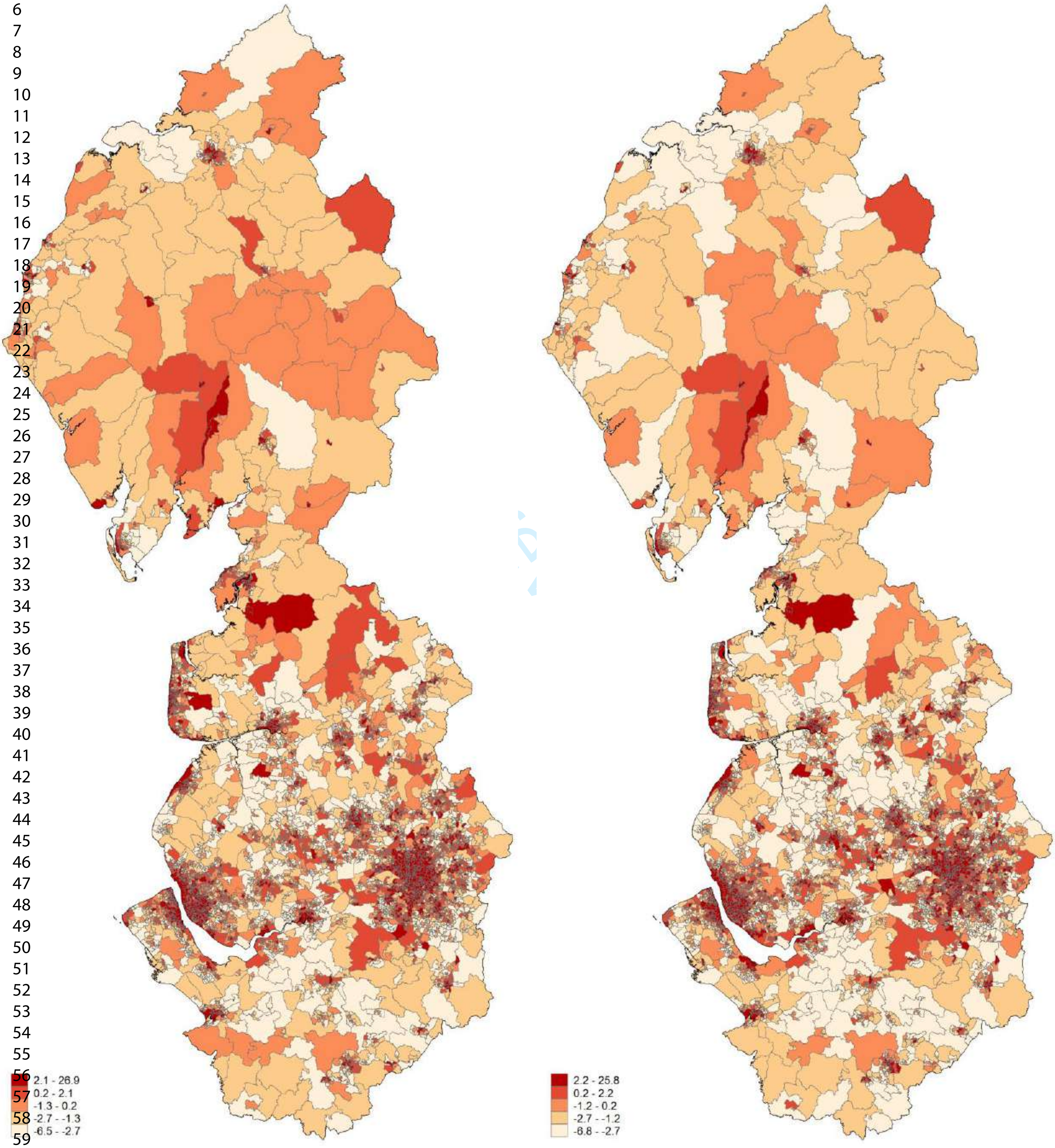

Yorkshire and the Humber, Social Fragmentation

2001 2011

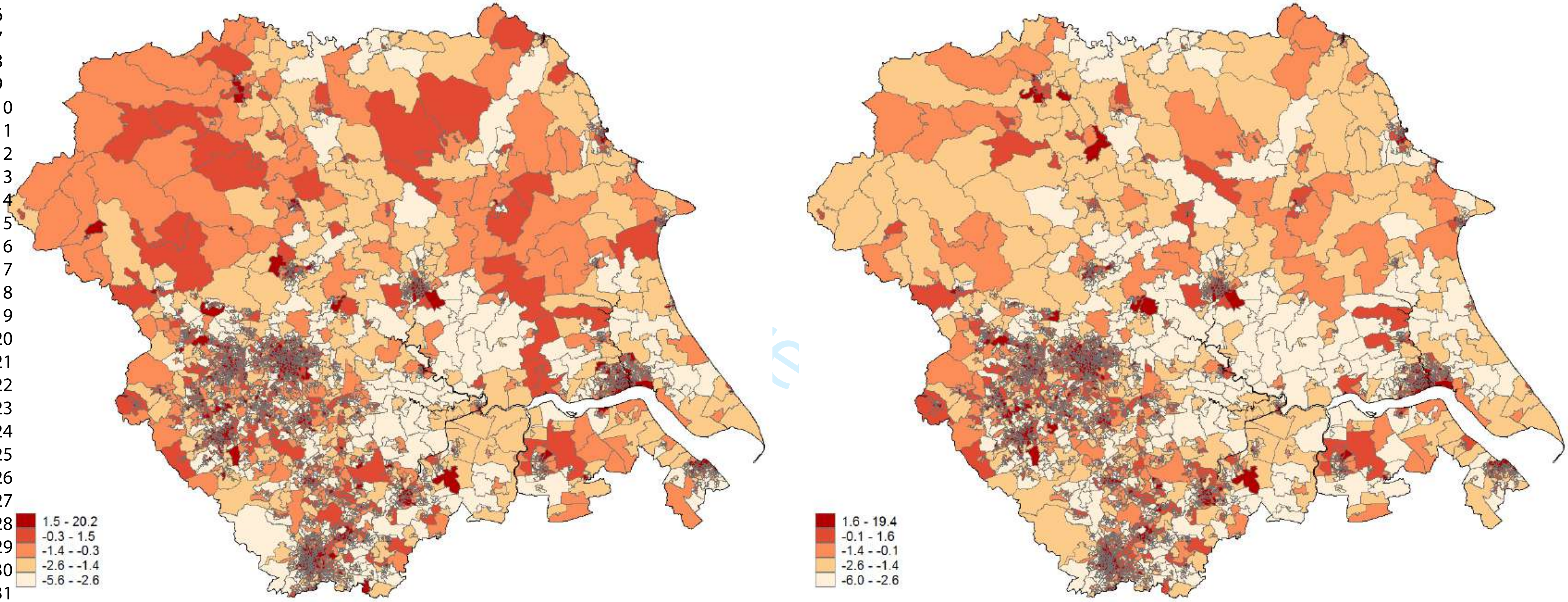

East Midlands, Social Fragmentation

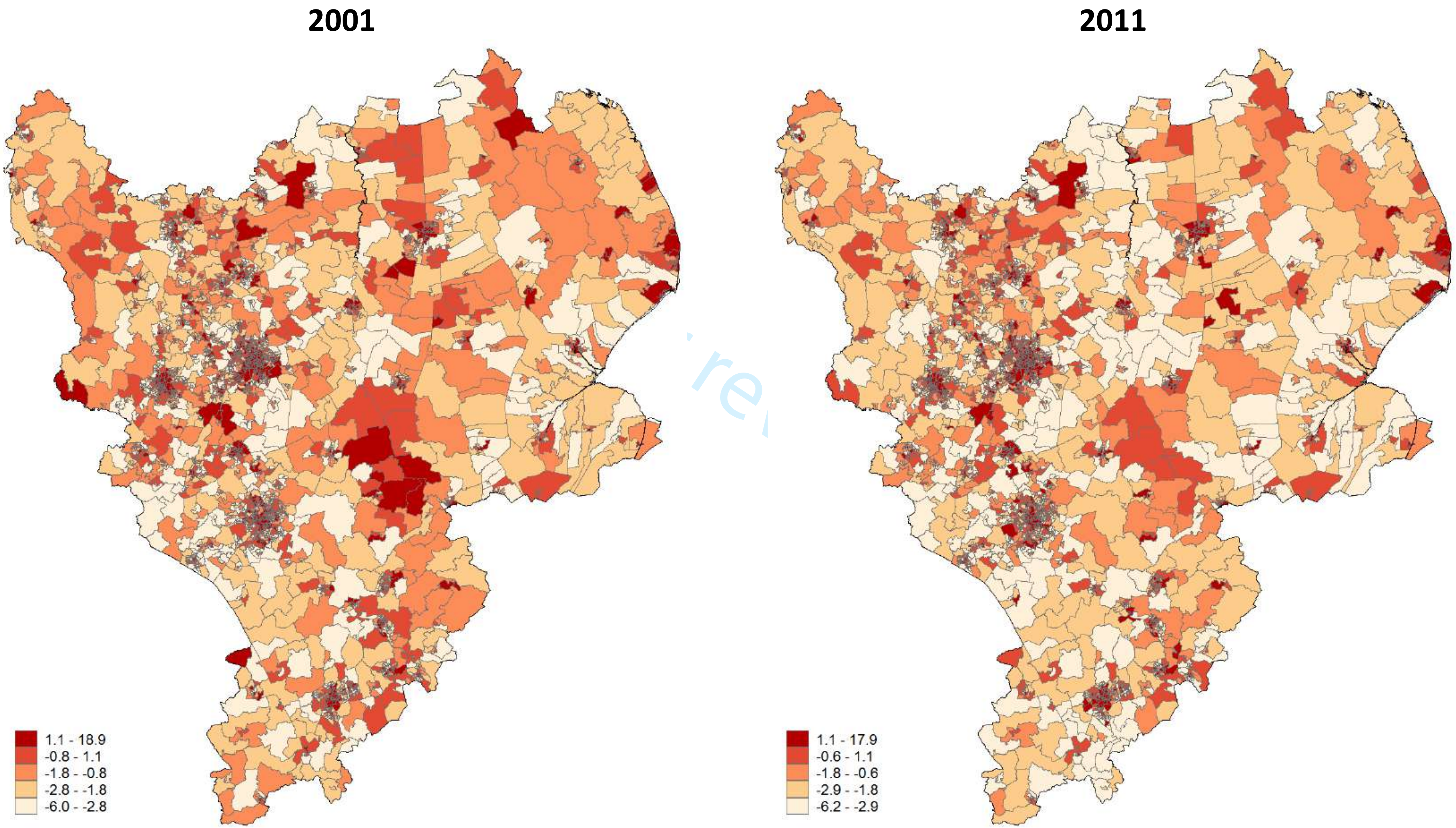

West Midlands, Social Fragmentation

2001

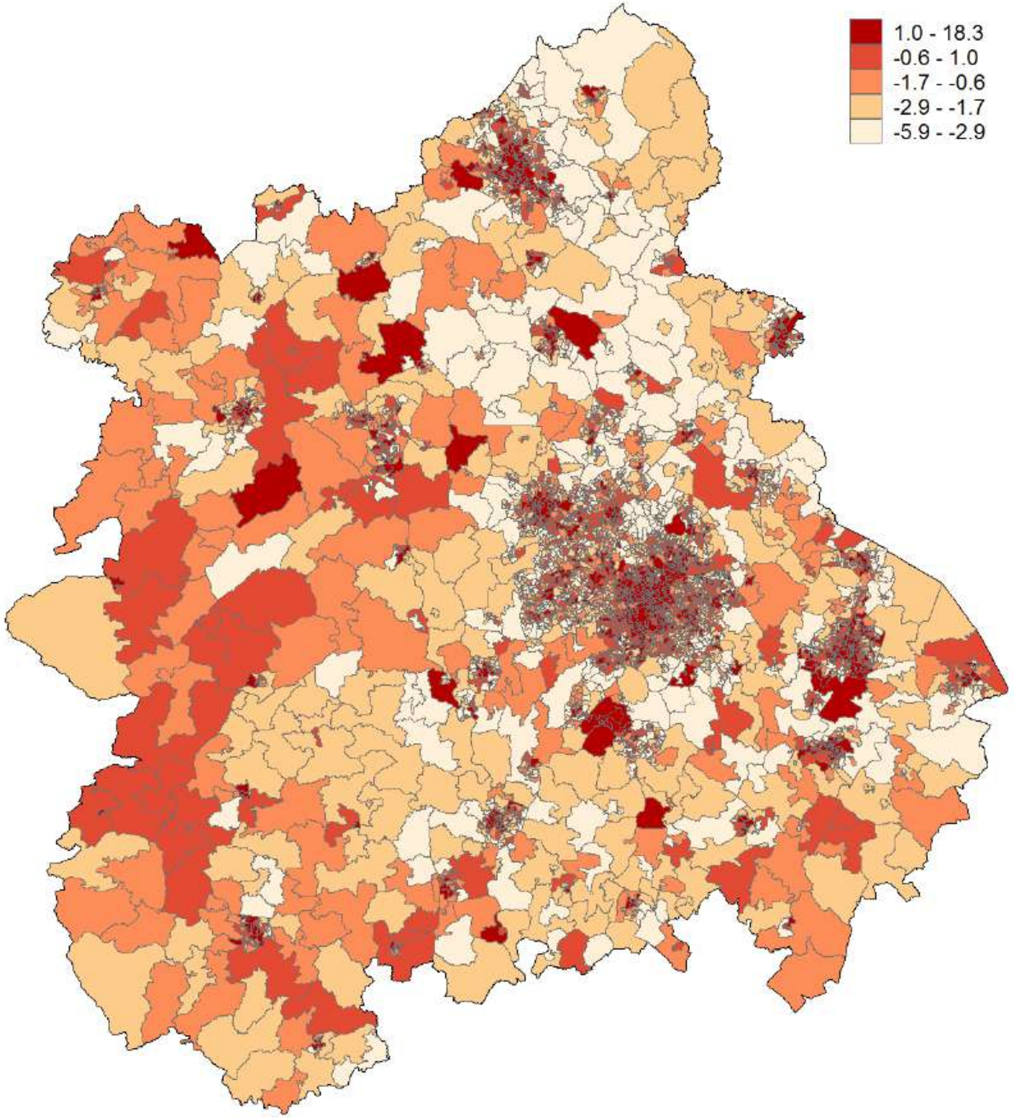

2011

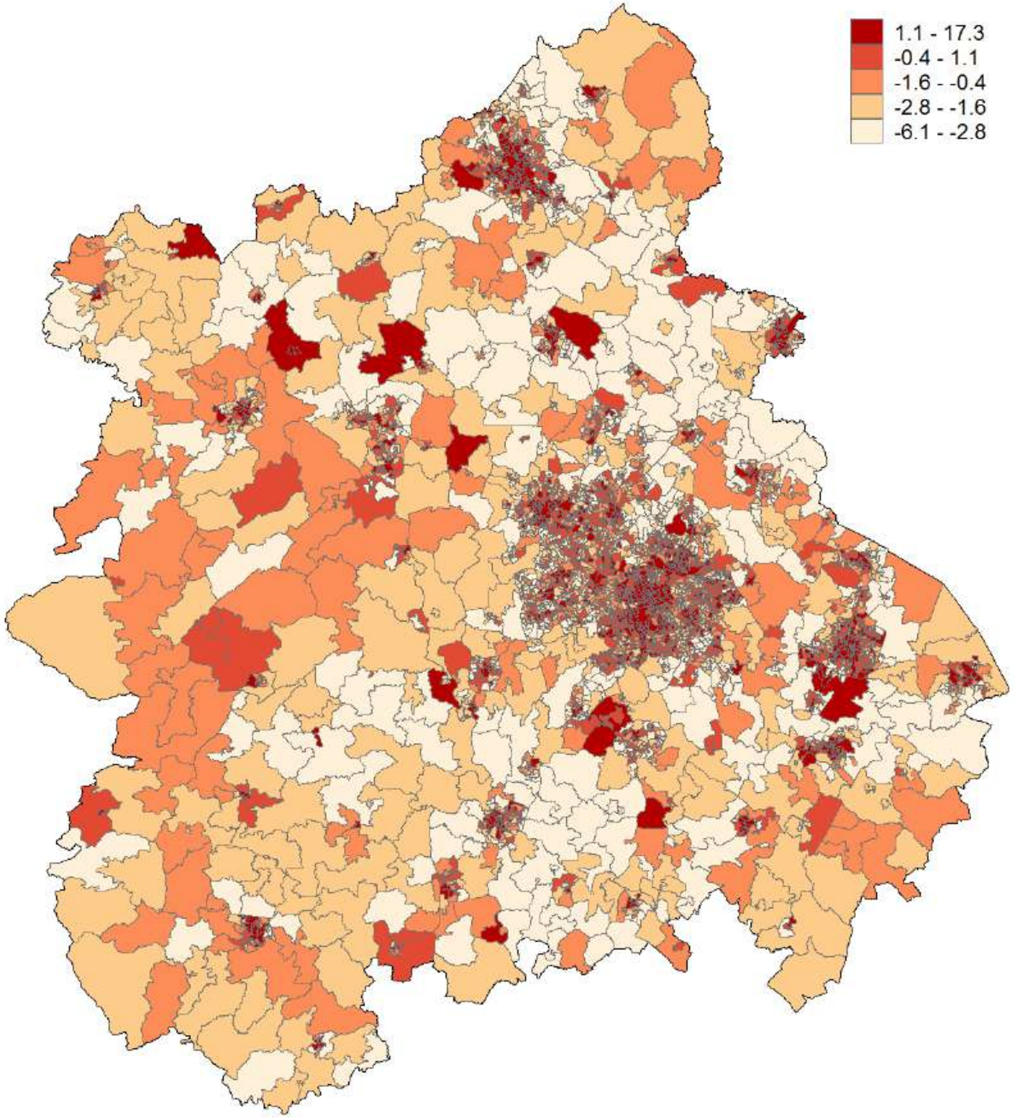

East of England, Social Fragmentation

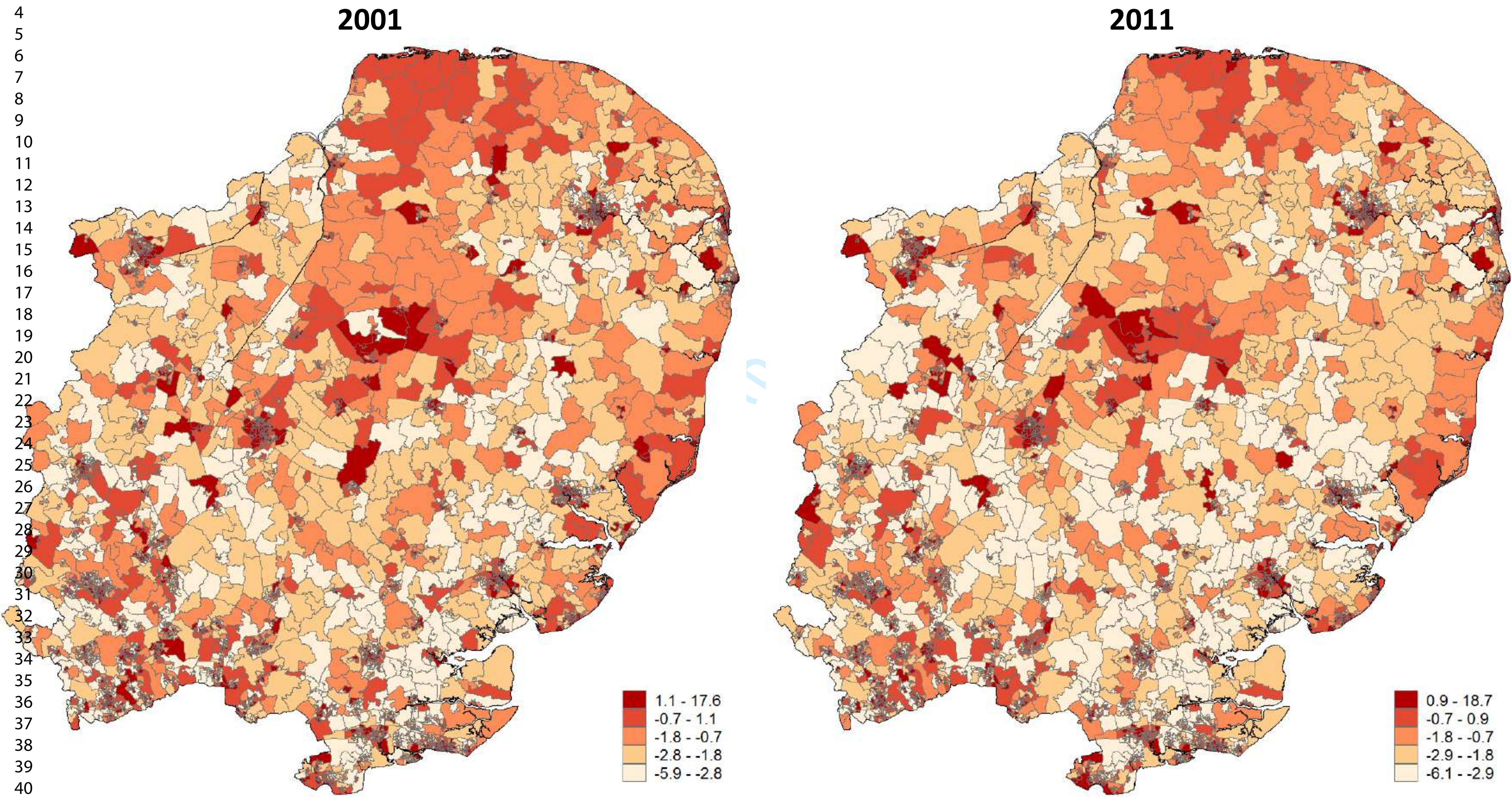

London, Social Fragmentation

2001

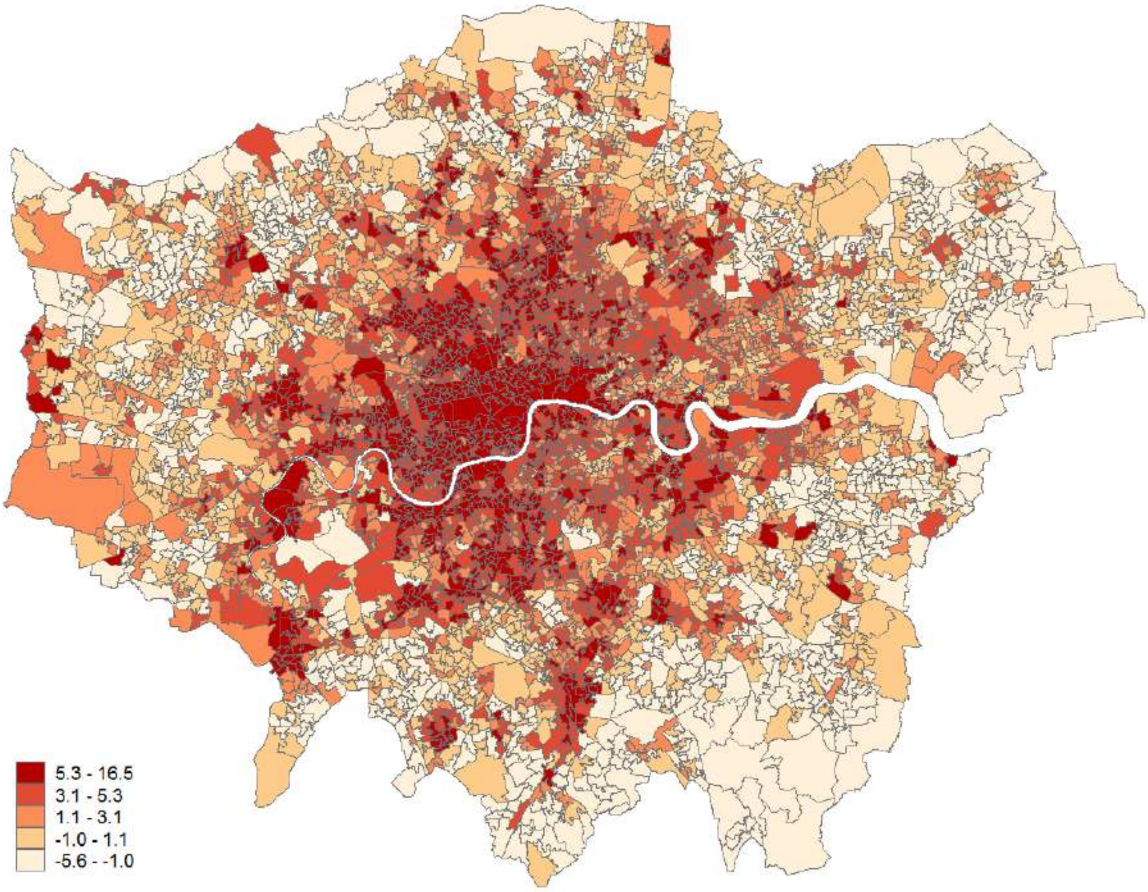

2011

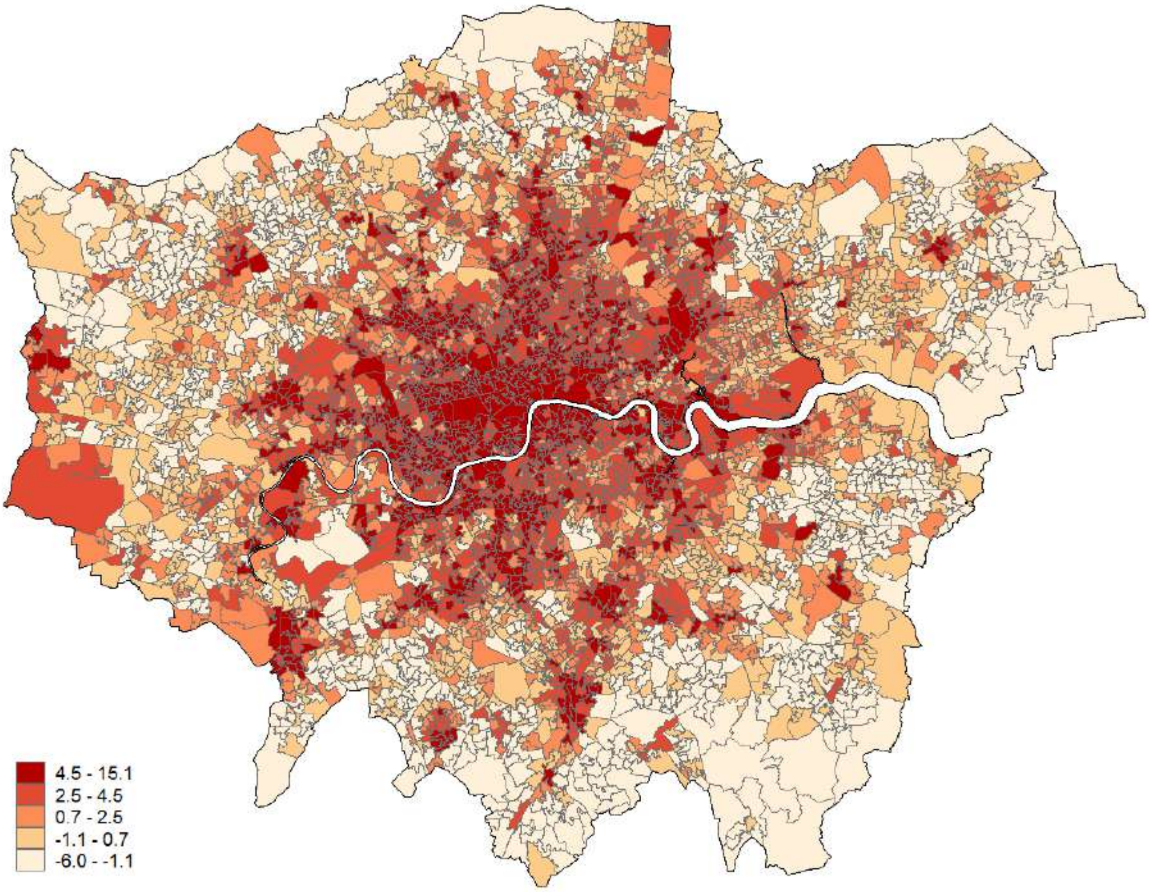

South East Coast, Social Fragmentation

2001

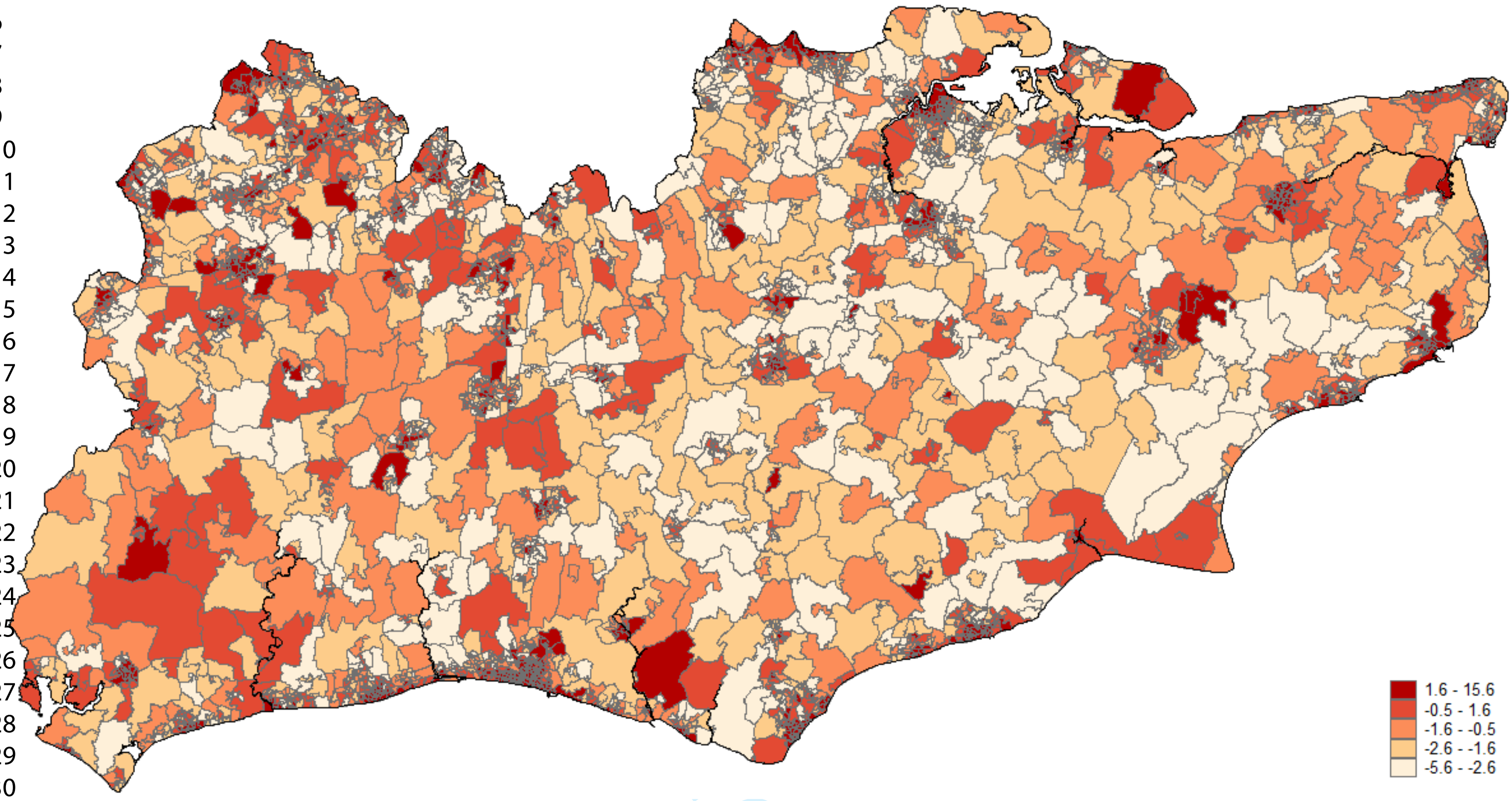

2011

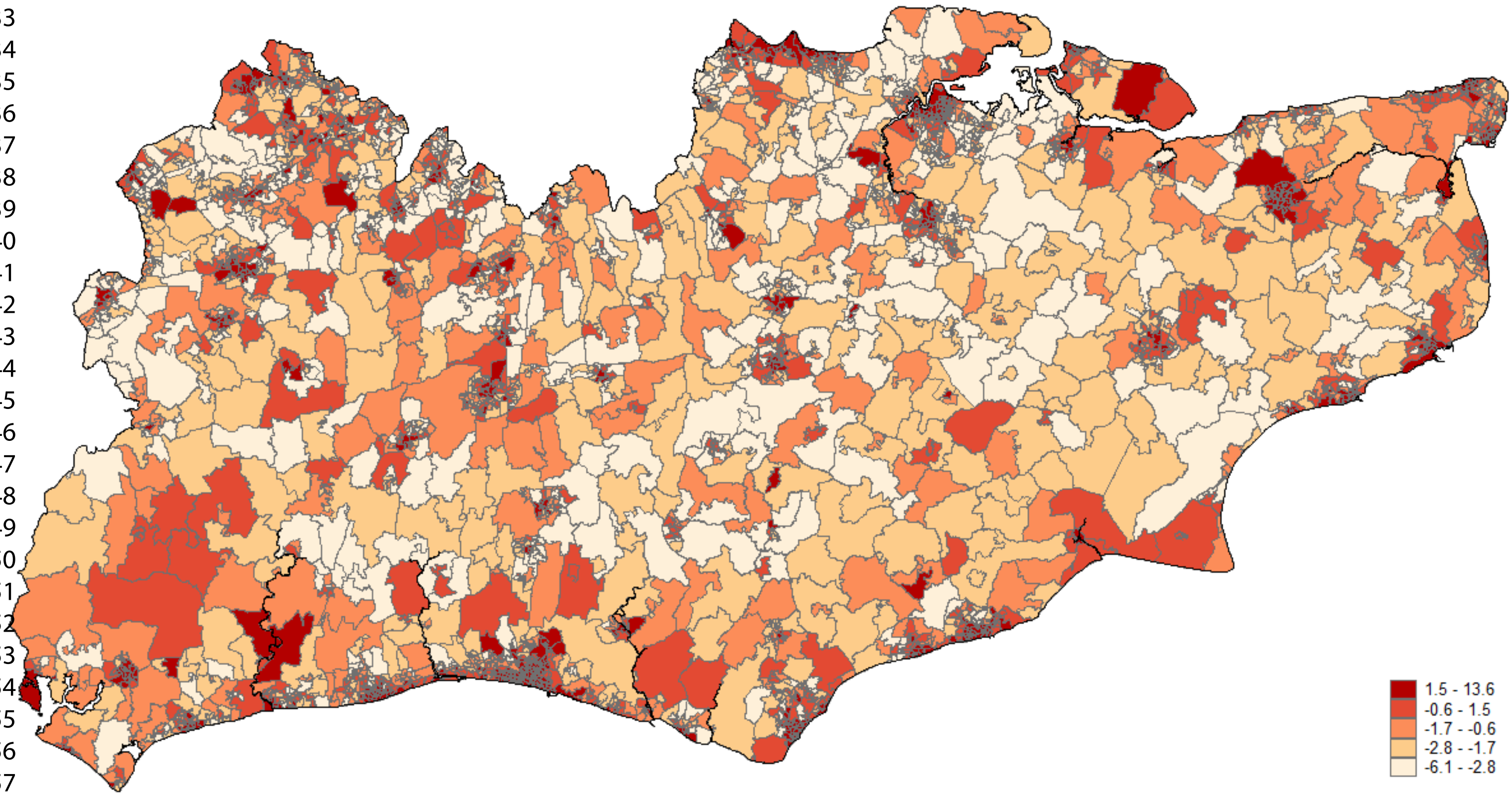

South Central, Social Fragmentation

2001 **2011**

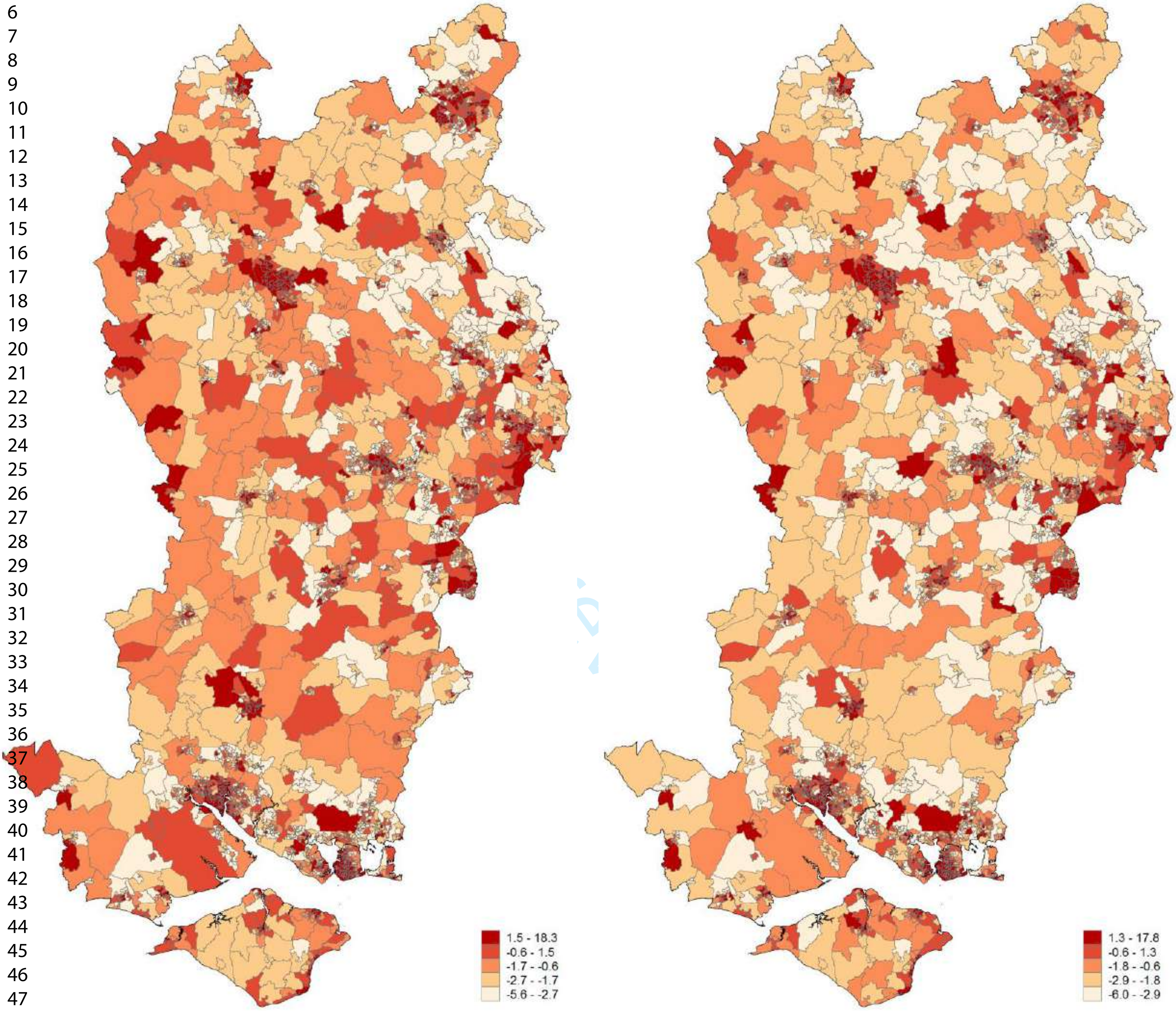

South West, Social Fragmentation

2001

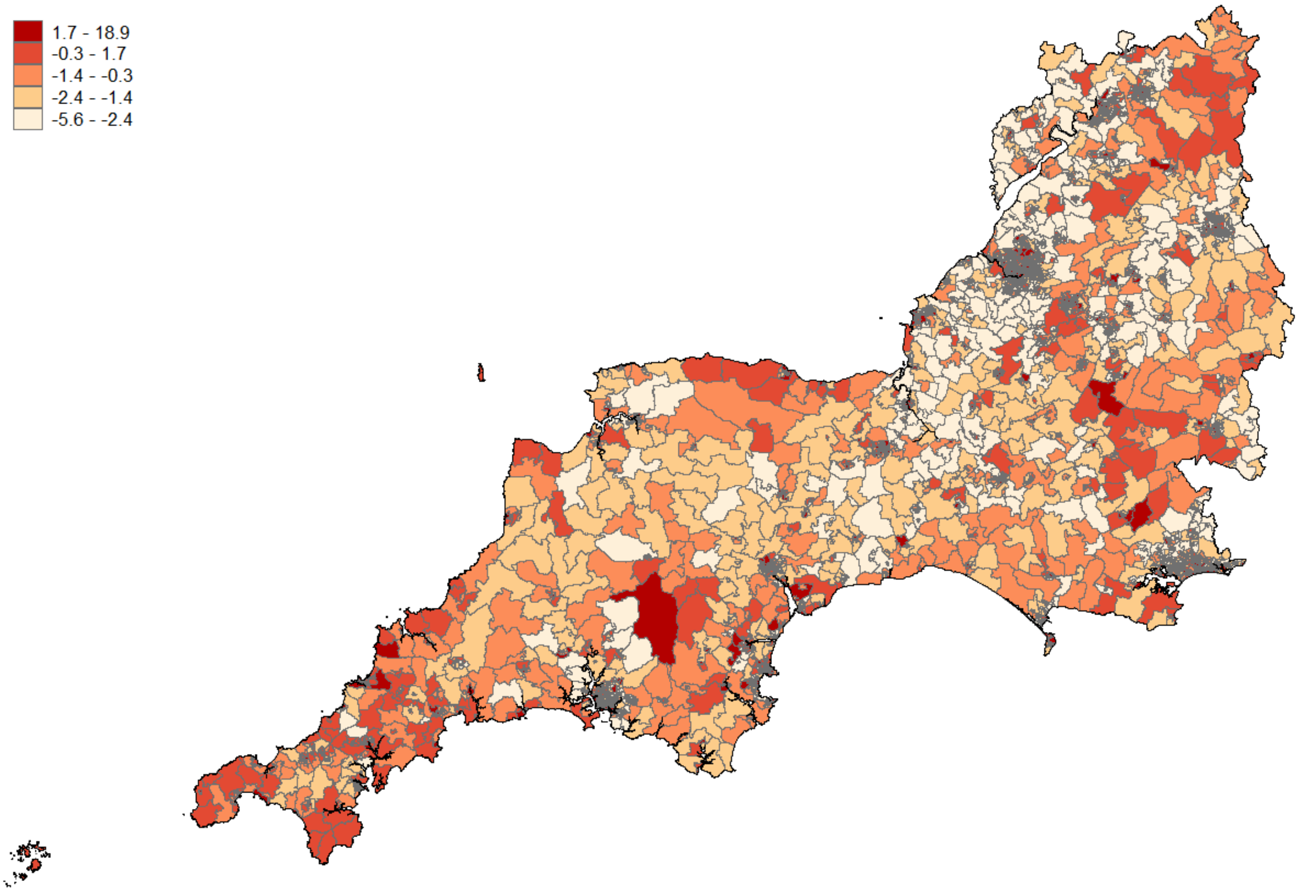

2011

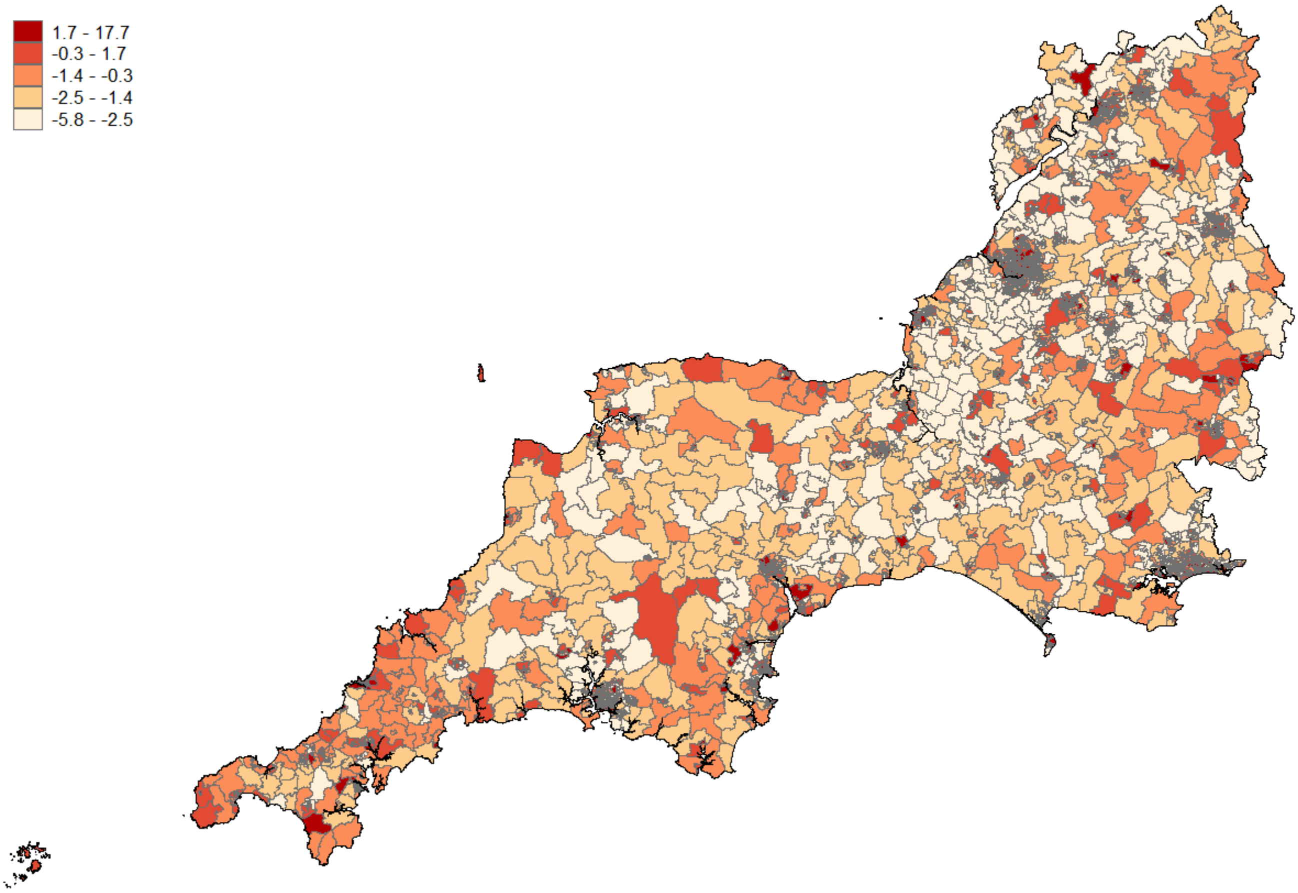

Single People

England 2001, Single People % of population

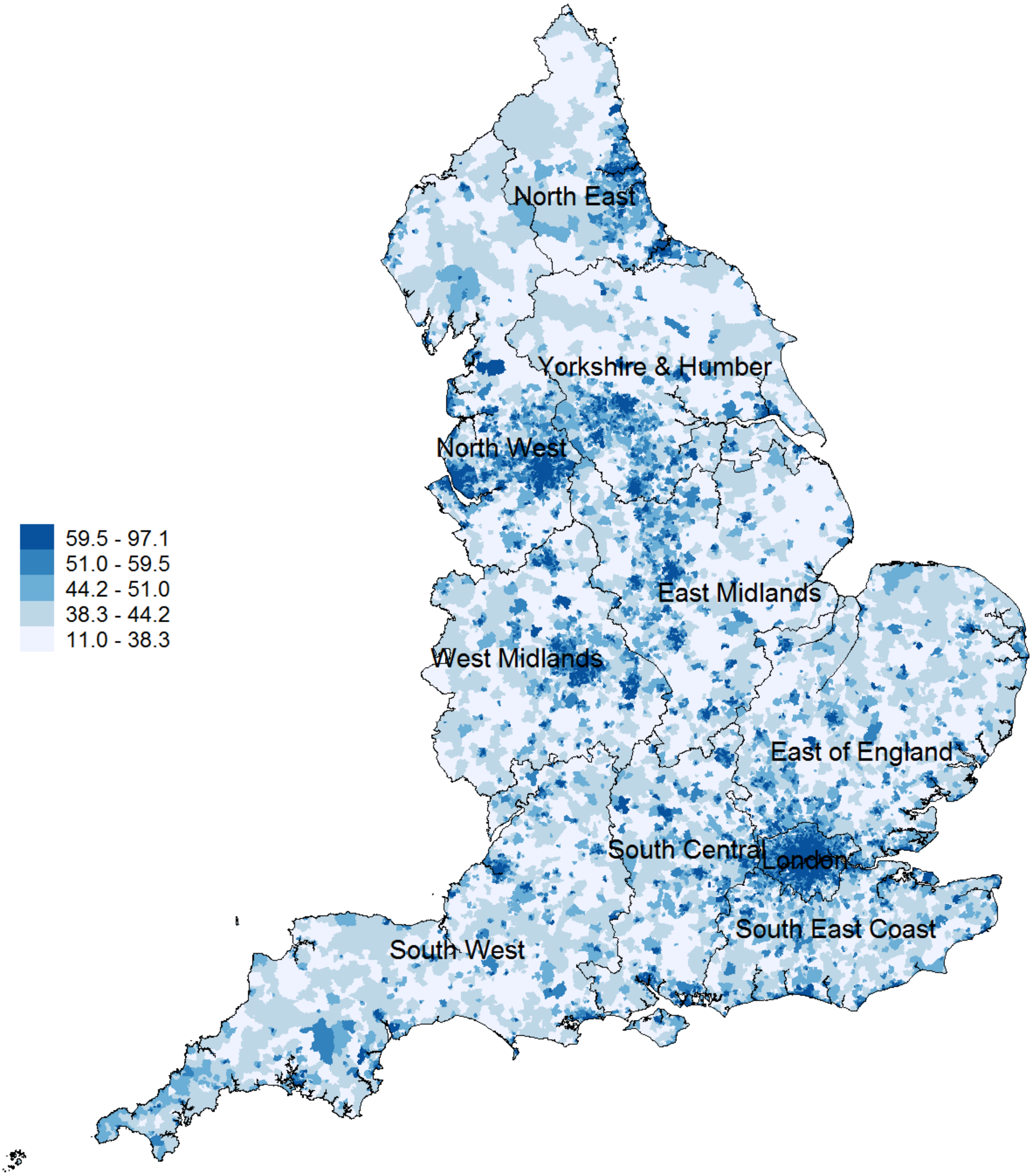

England 2011, Single People % of population

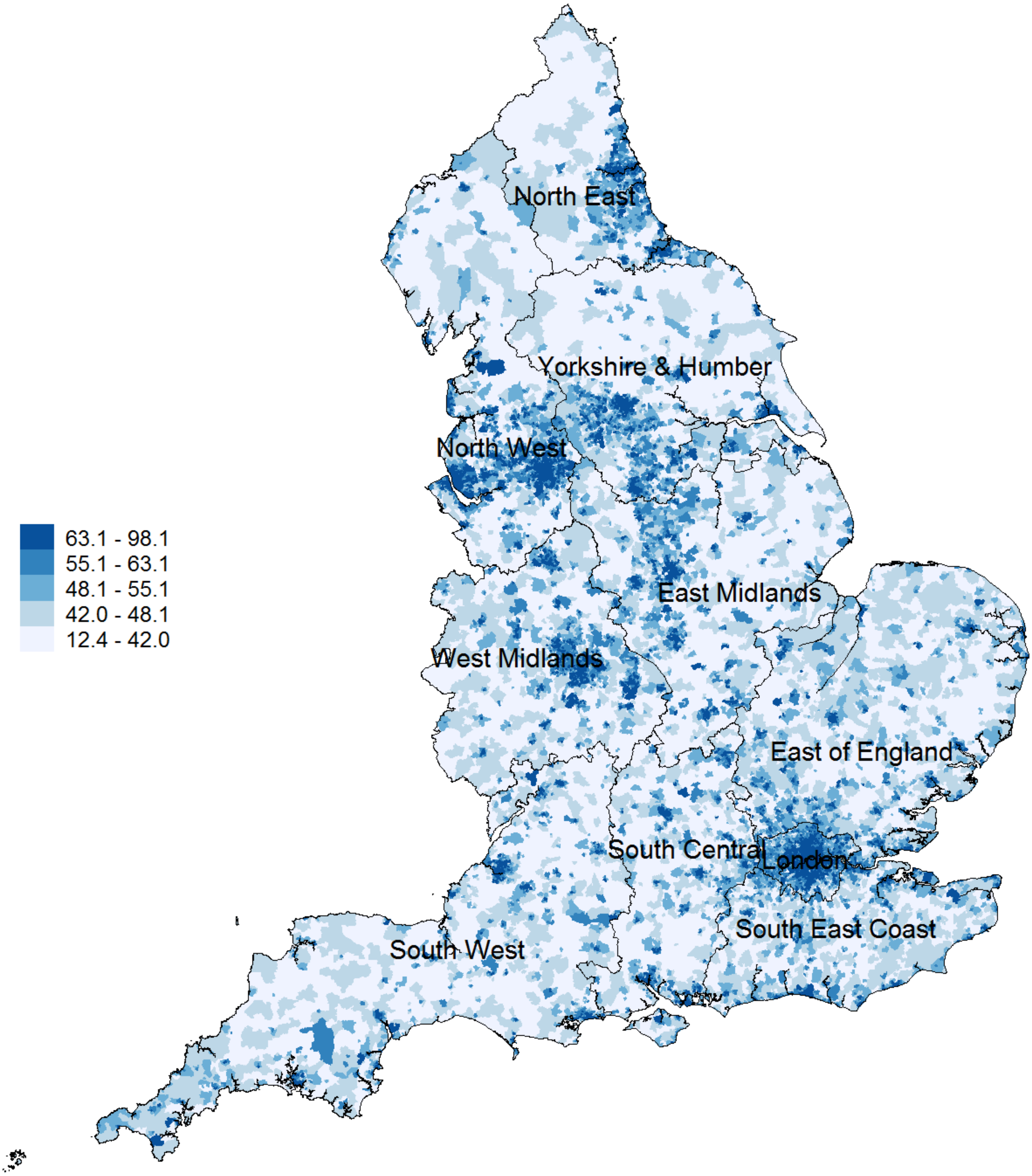

North East, Single People % of population

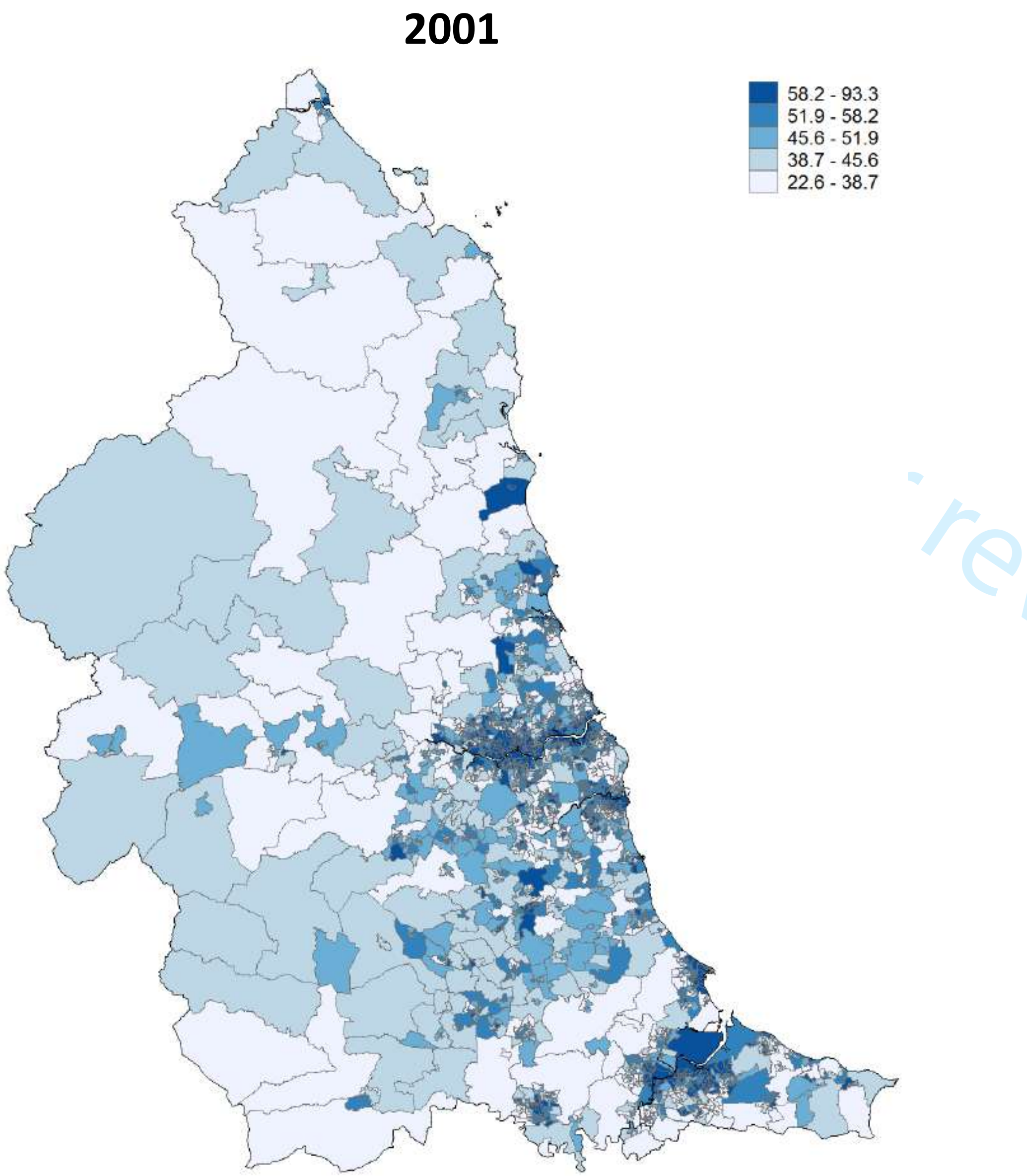

North West, Single People % of population

2001

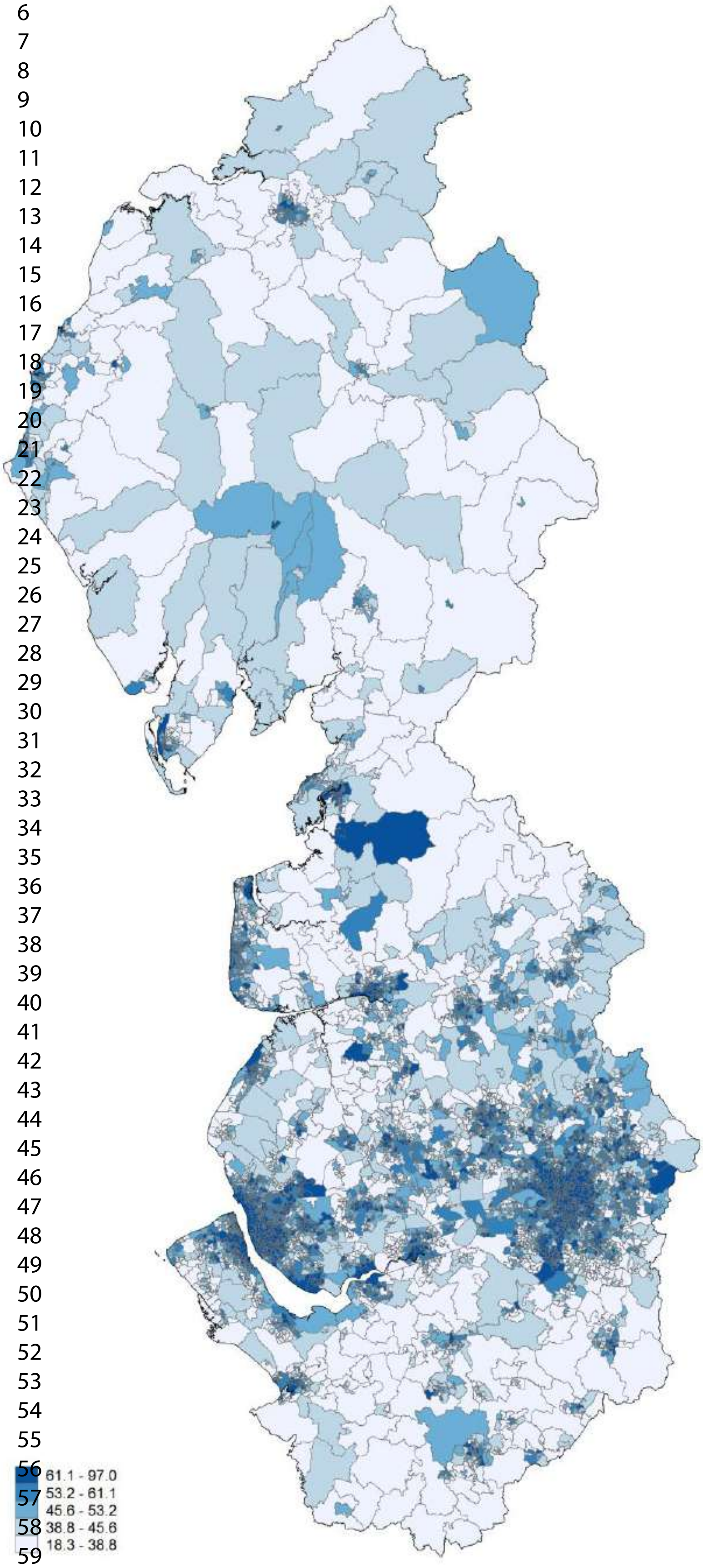

2011

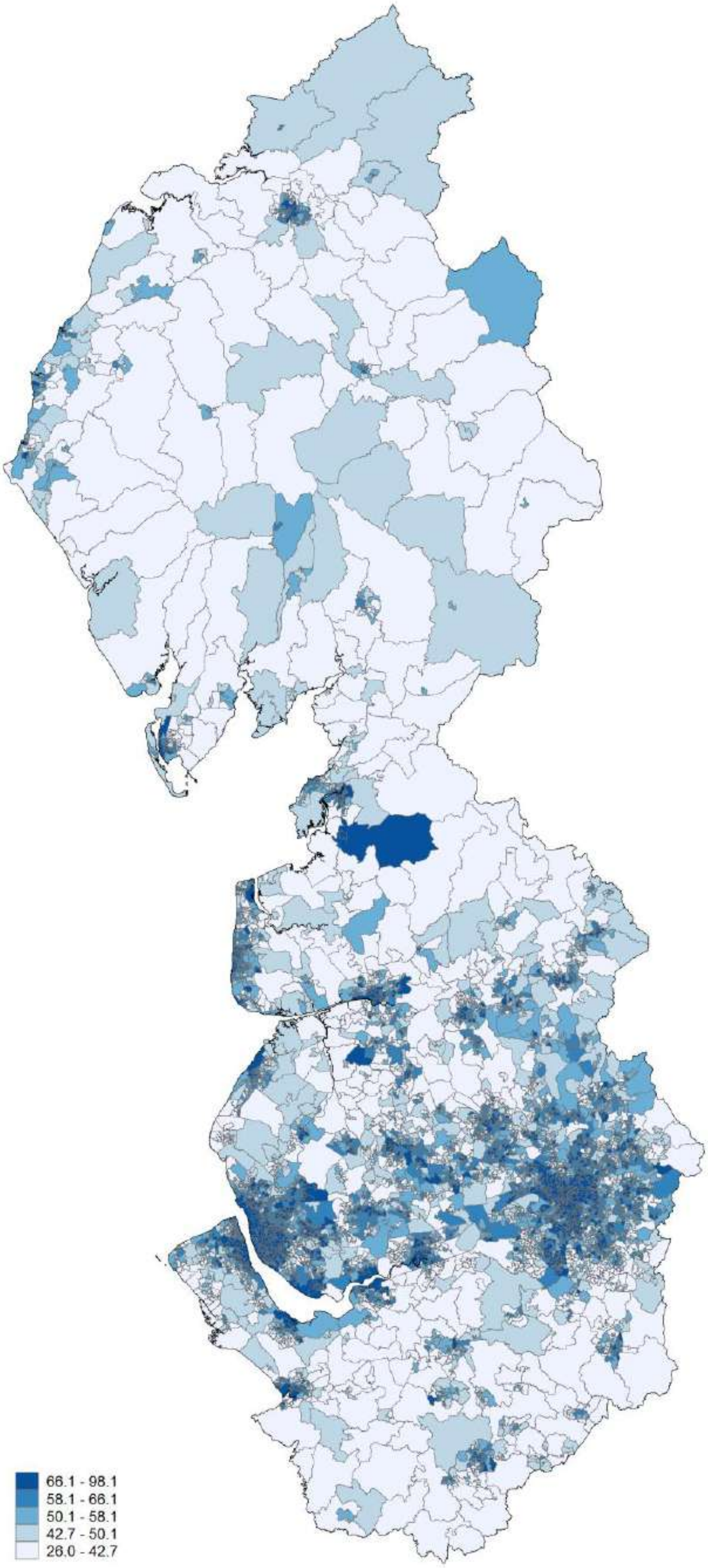

Yorkshire and the Humber, Single People % of population

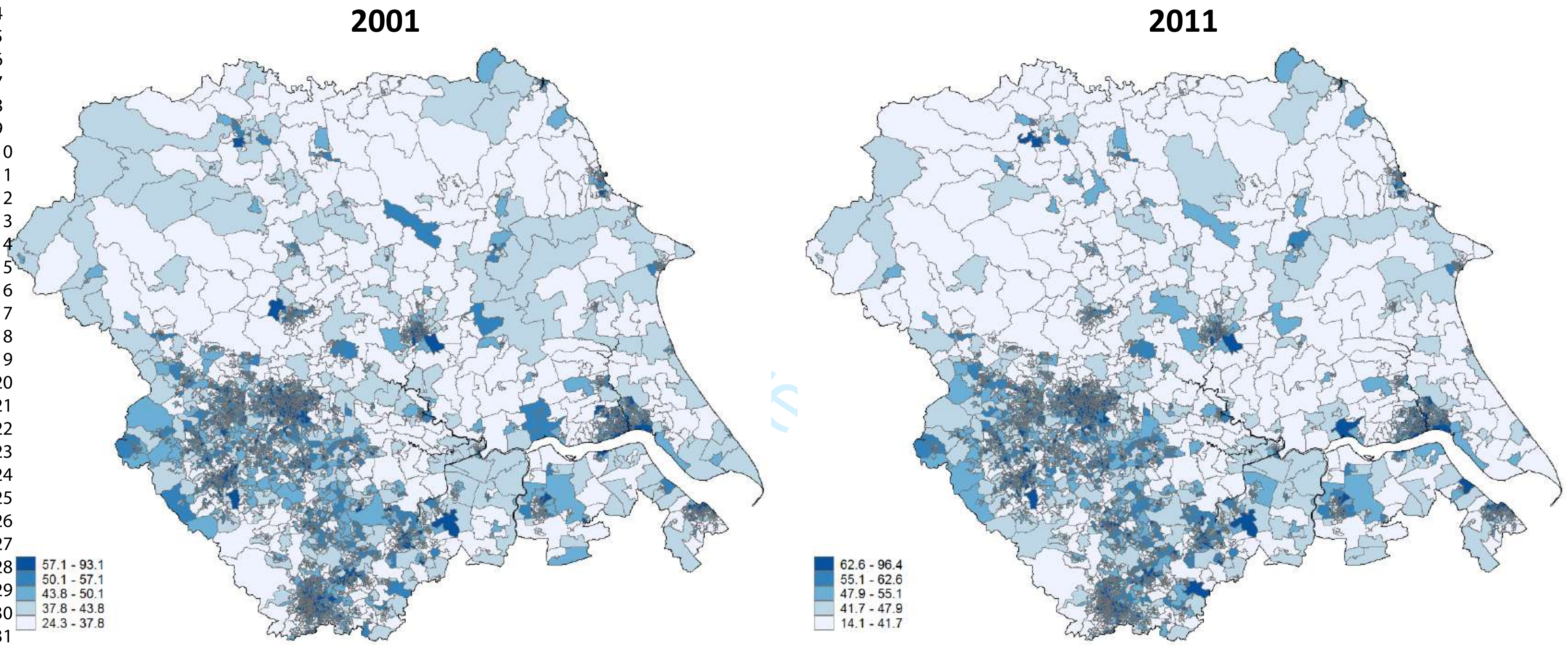

East Midlands, Single People % of population

2001

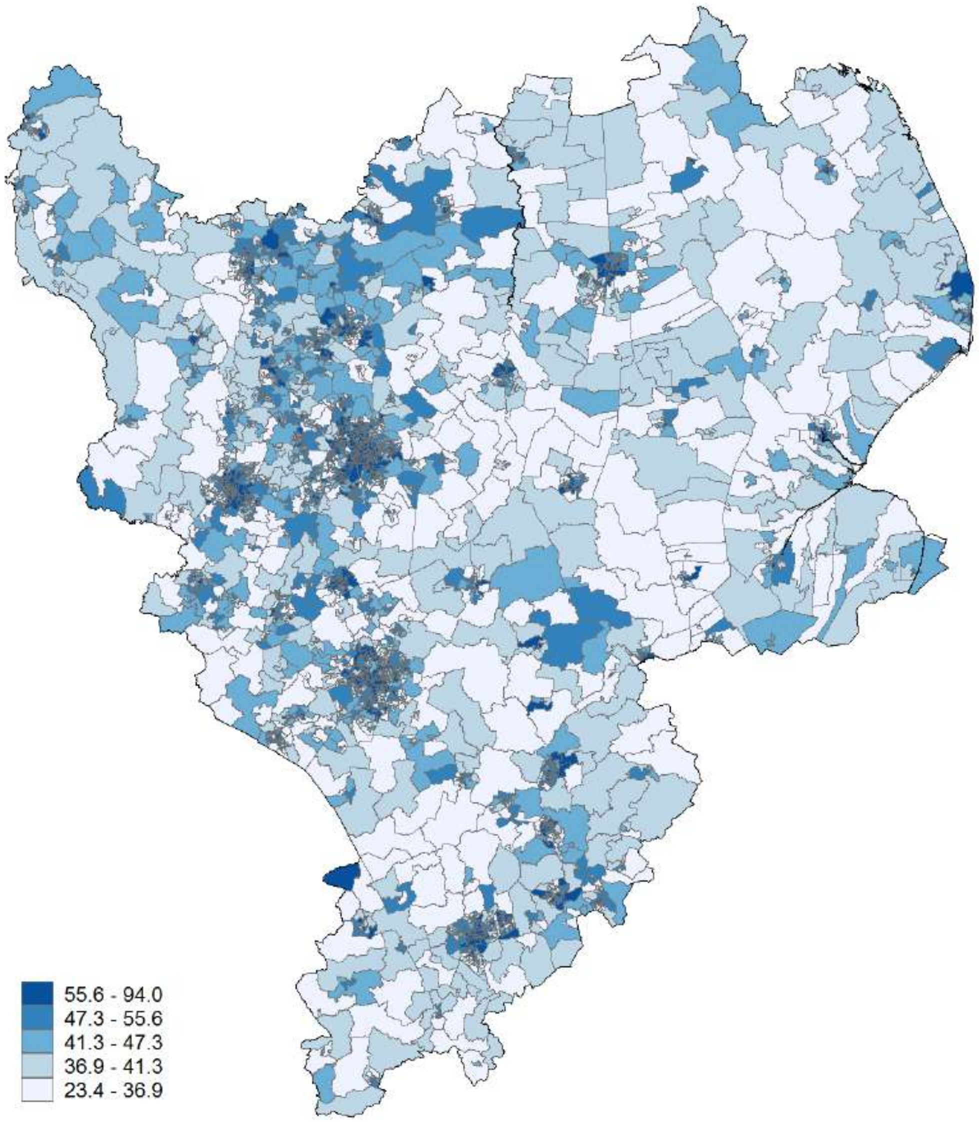

2011

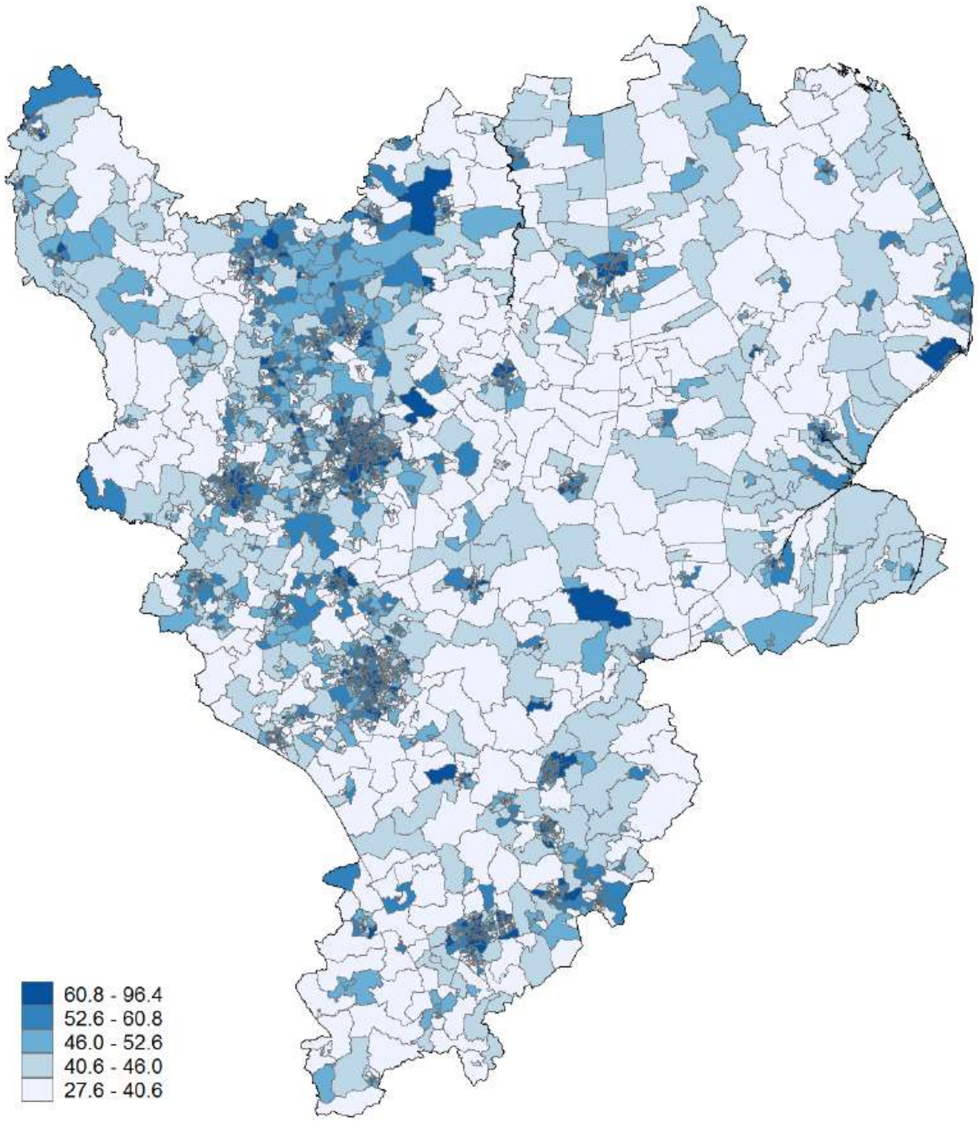

West Midlands, Single People % of population

2001

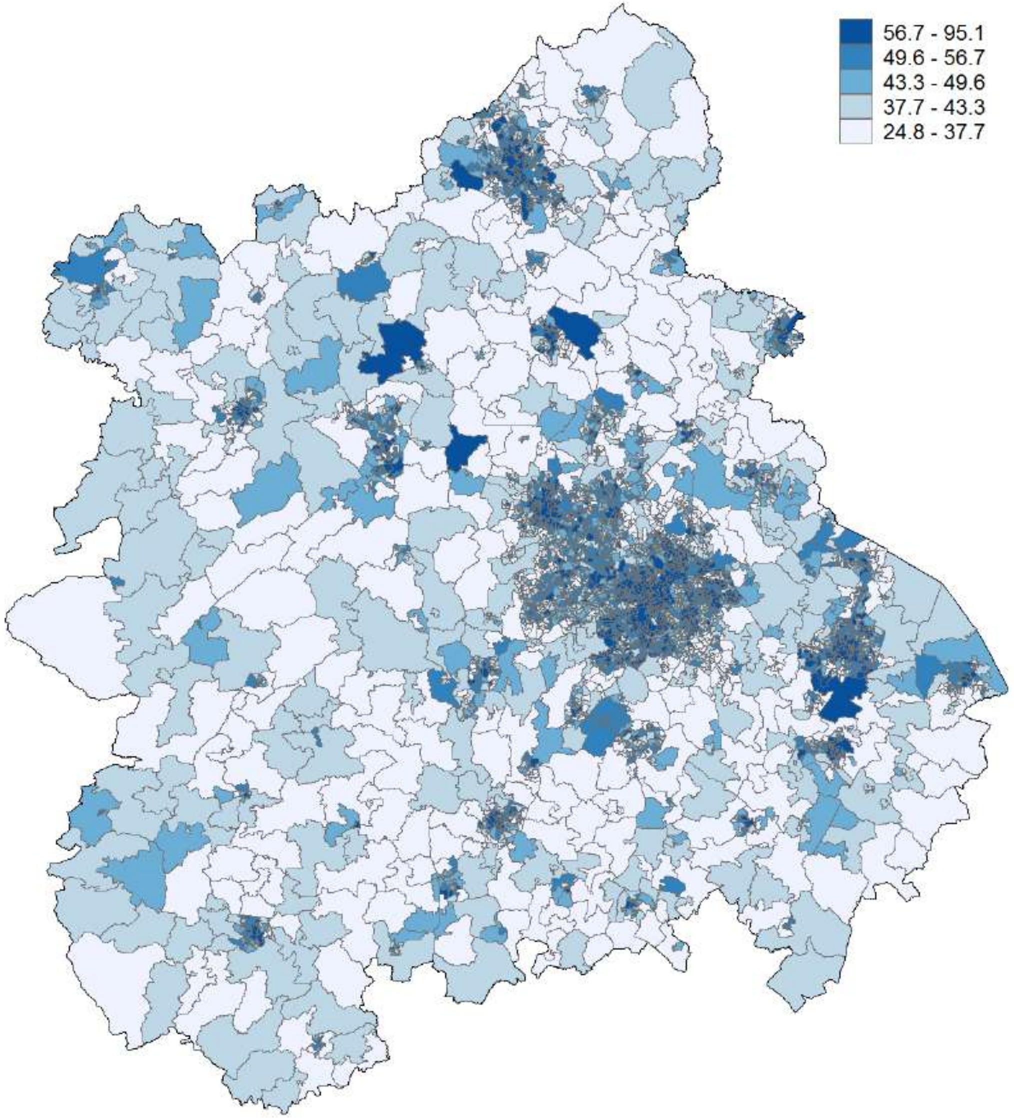

2011

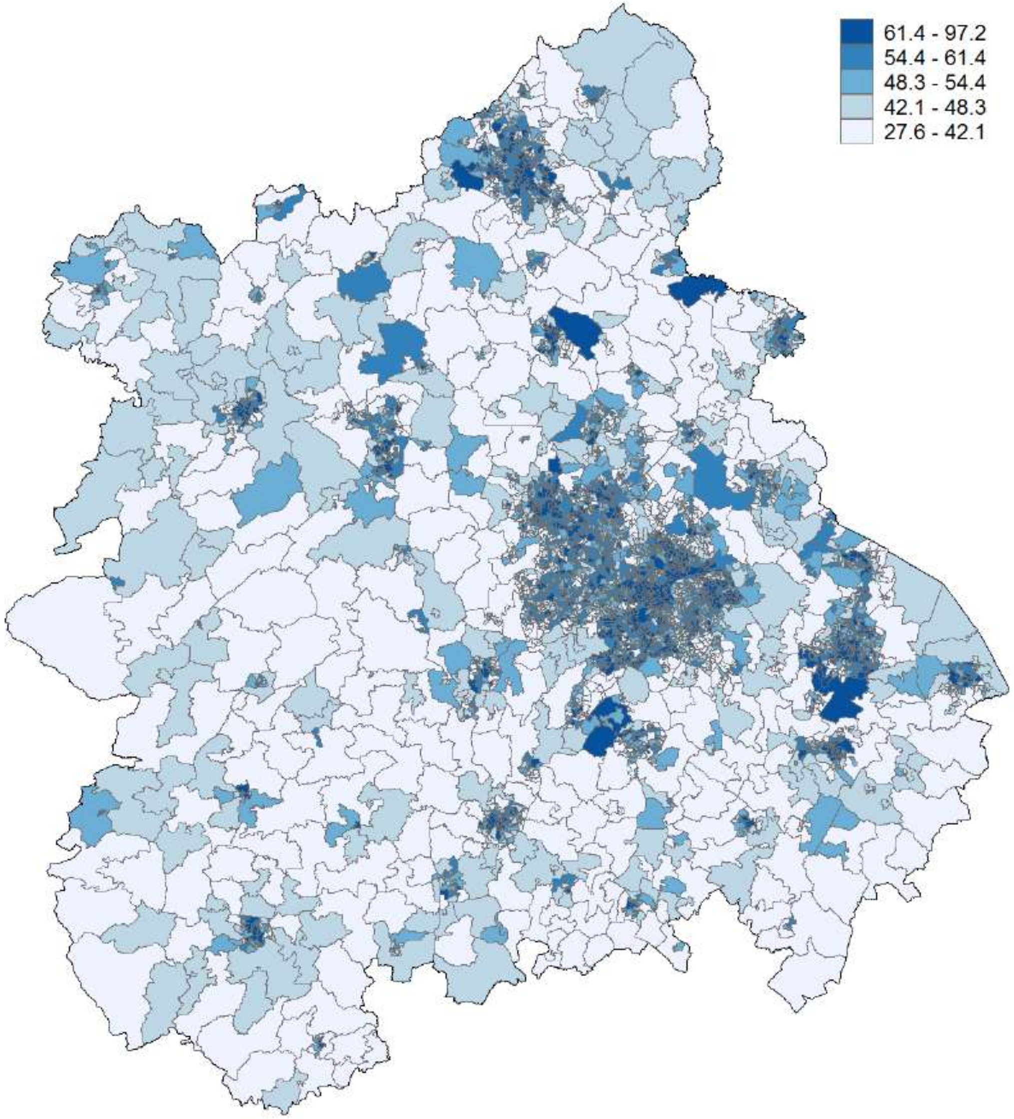

East of England, Single People % of population

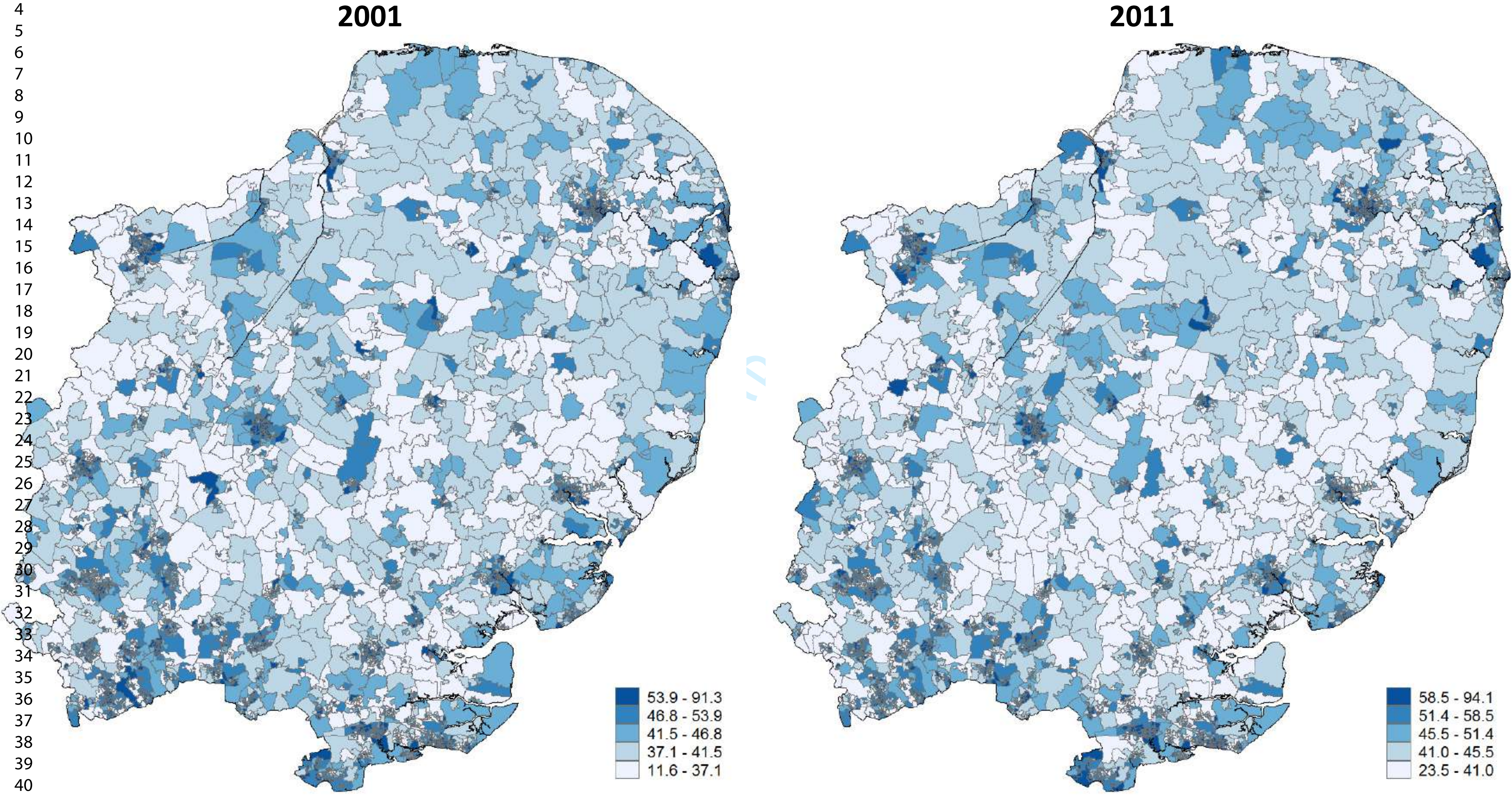

London, Single People % of population

2001

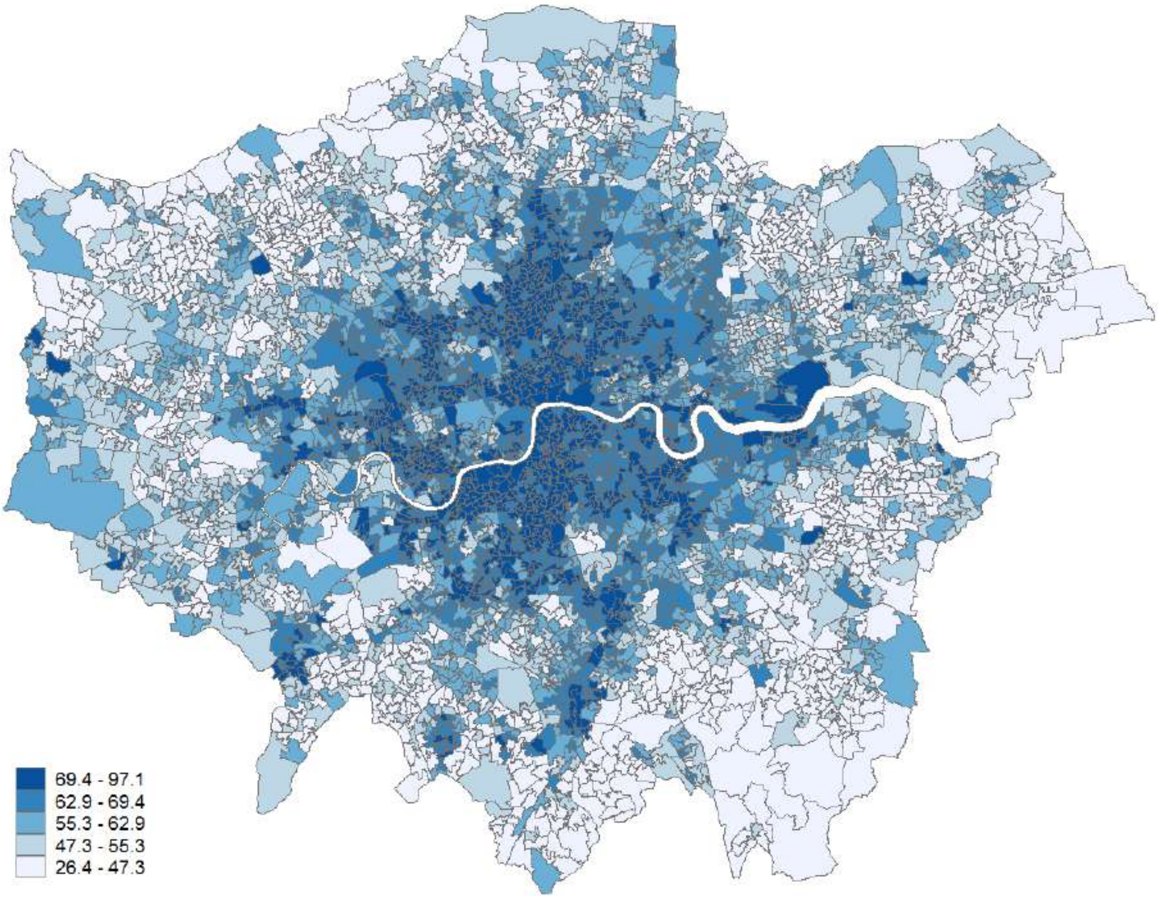

2011

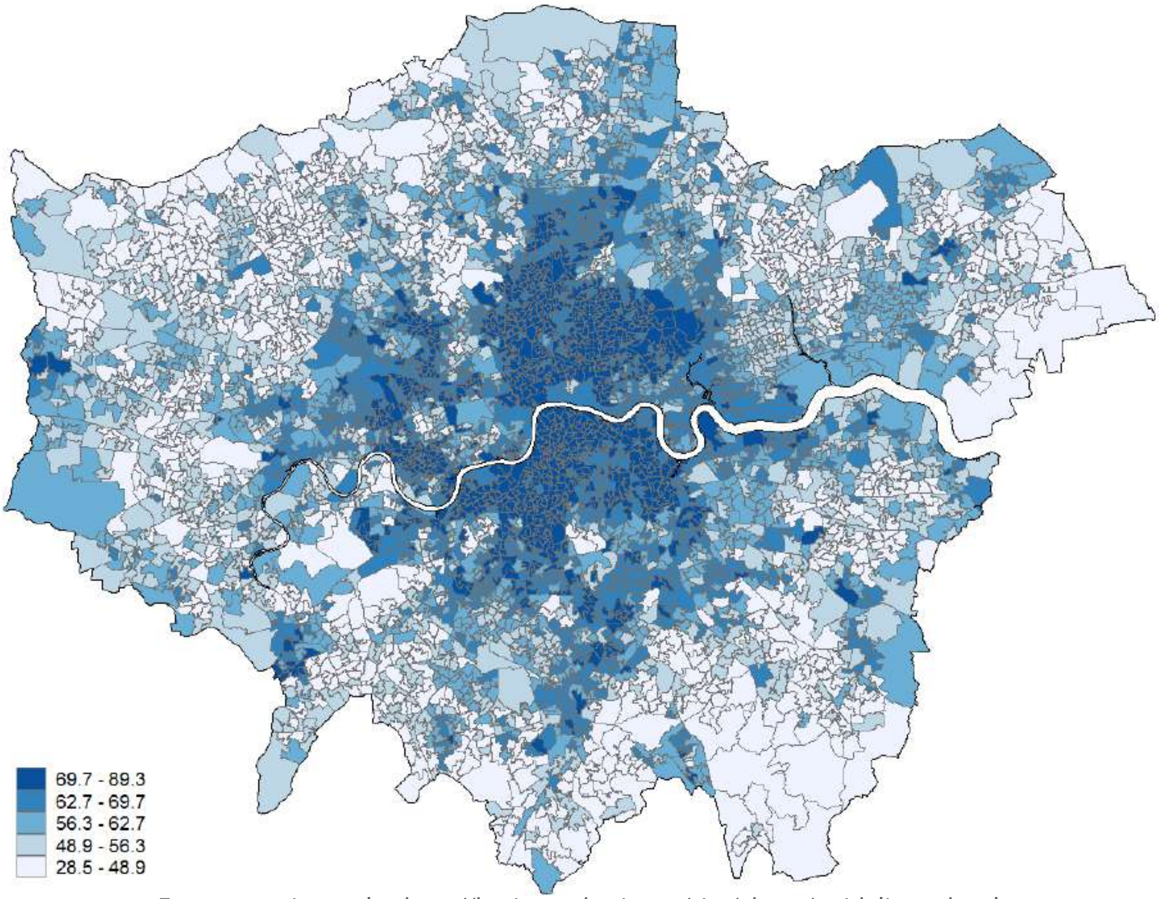

South East Coast, Single People % of population

2001

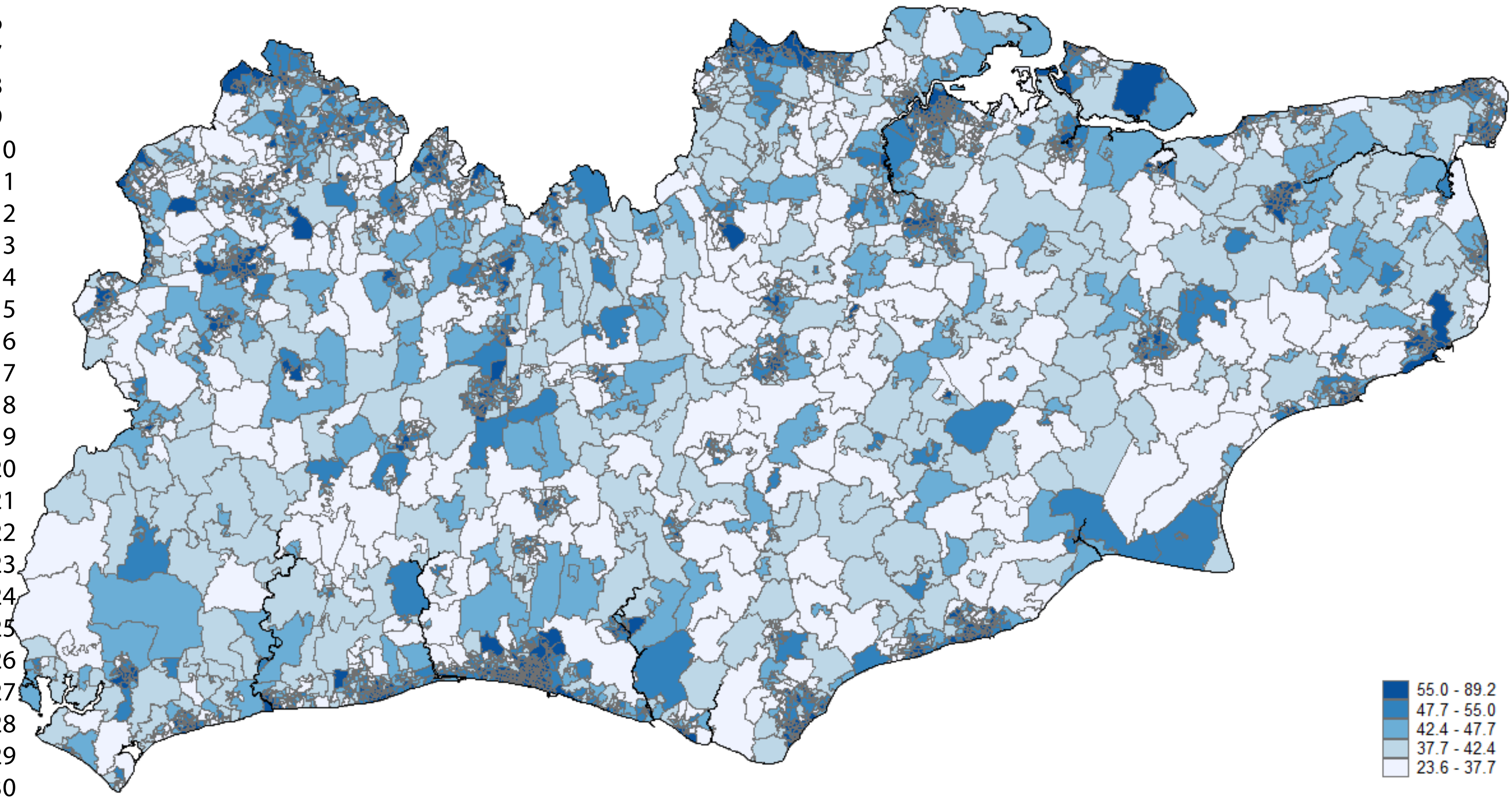

2011

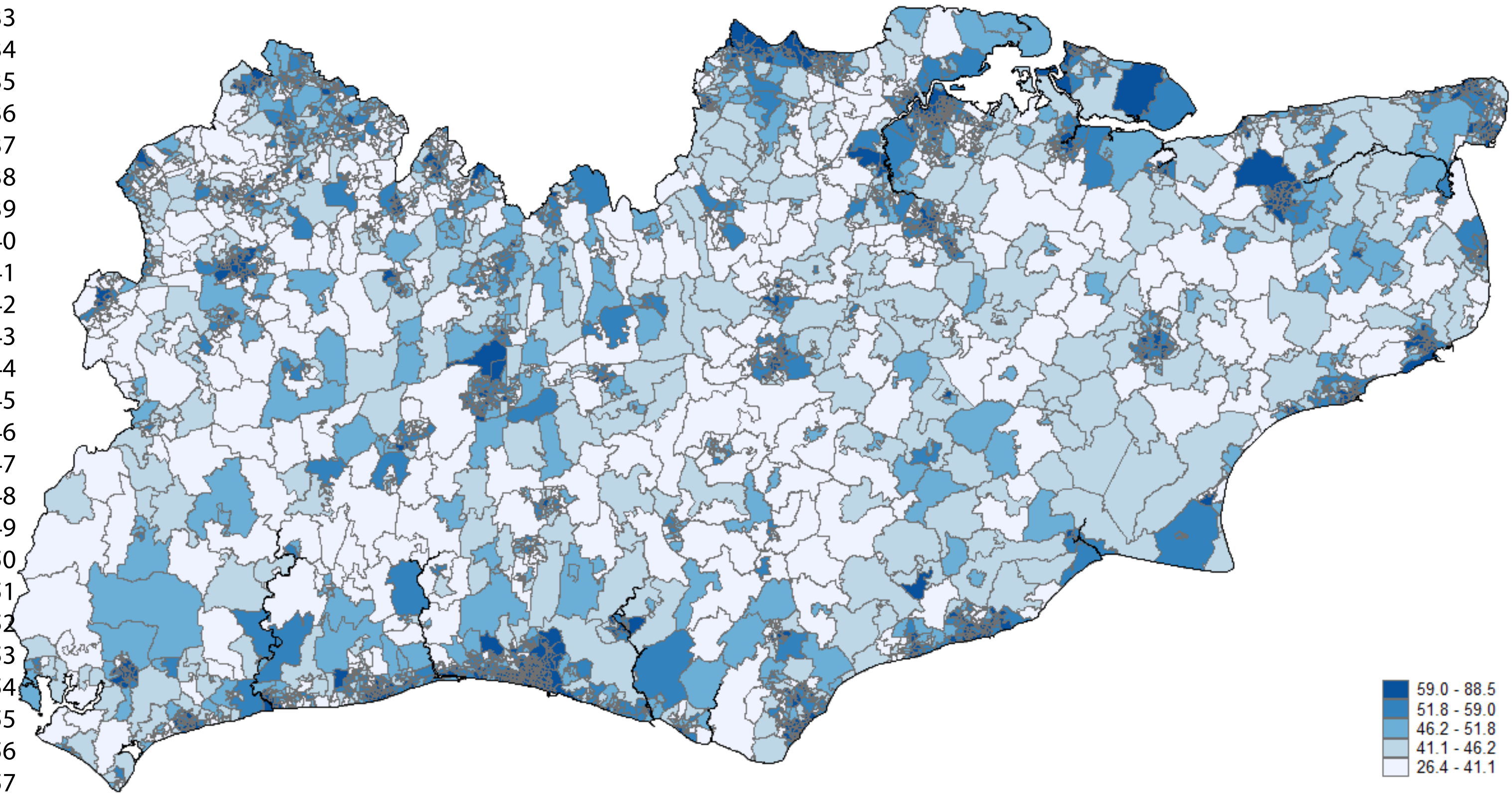

South Central, Single People % of population

2001

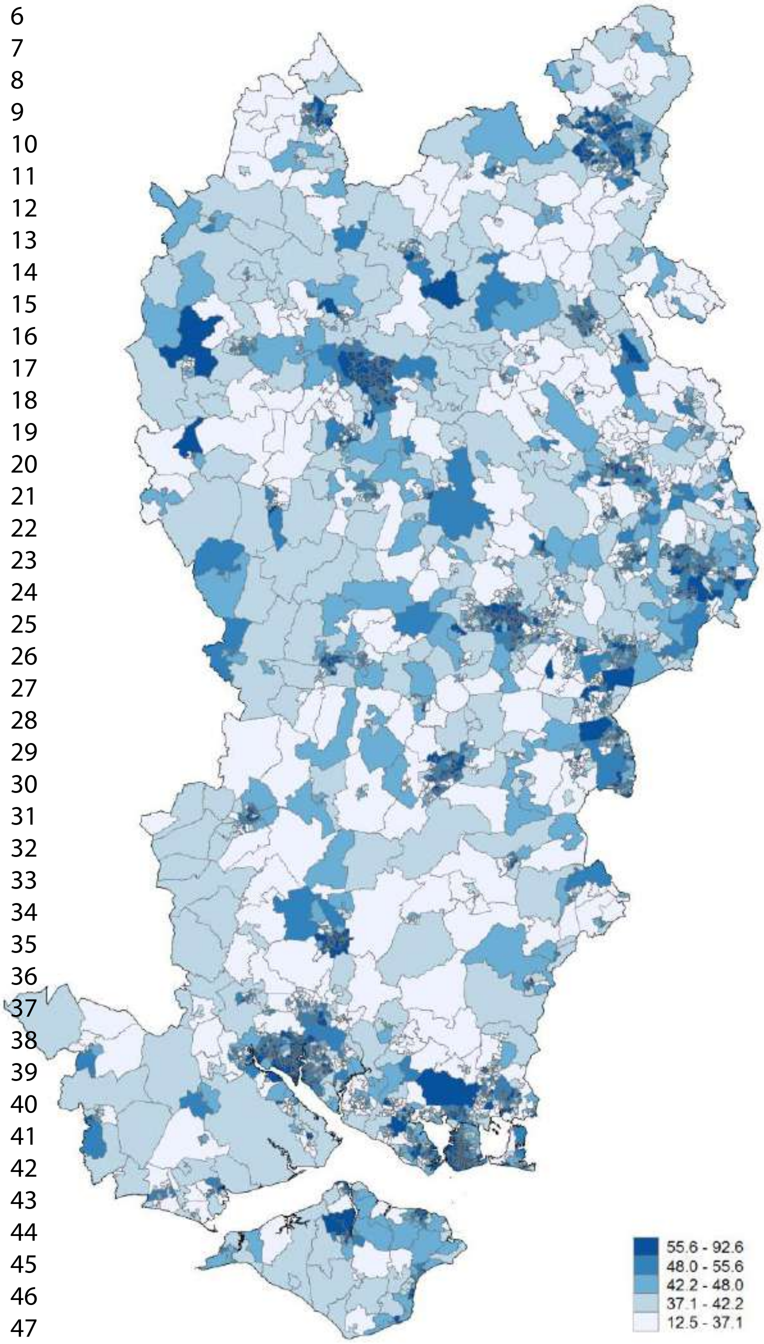

2011

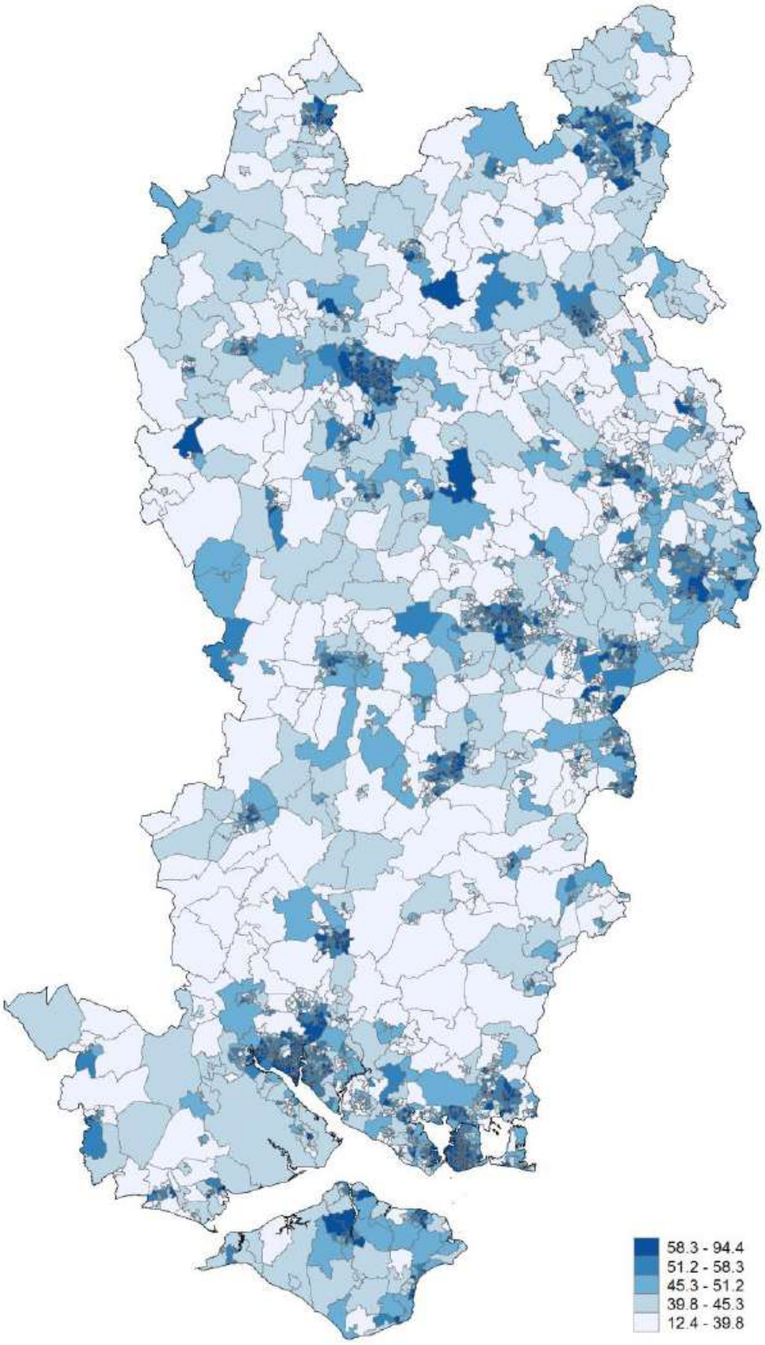

South West, Single People % of population

2001

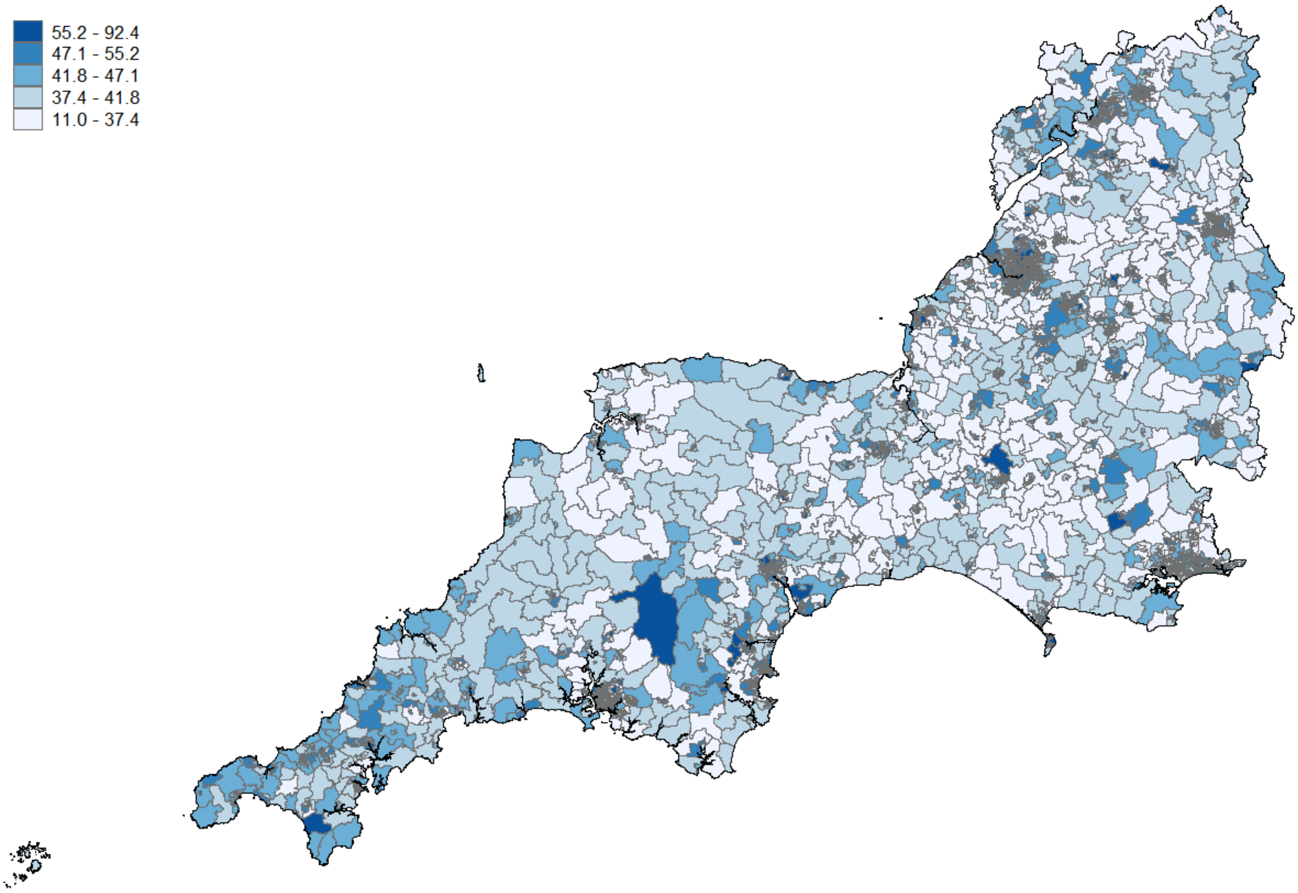

2011

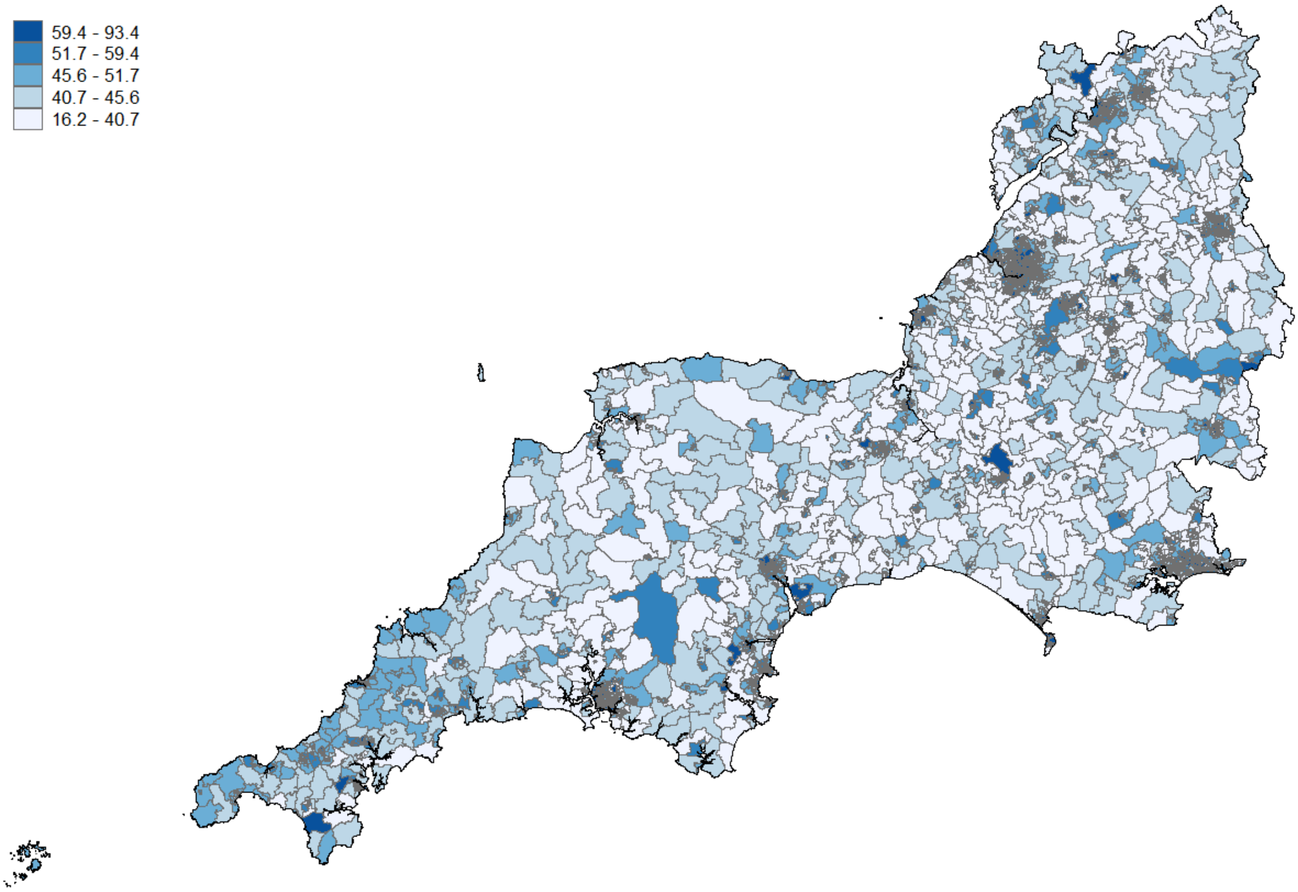

One Person Households

England 2001, One Person Households % of all households

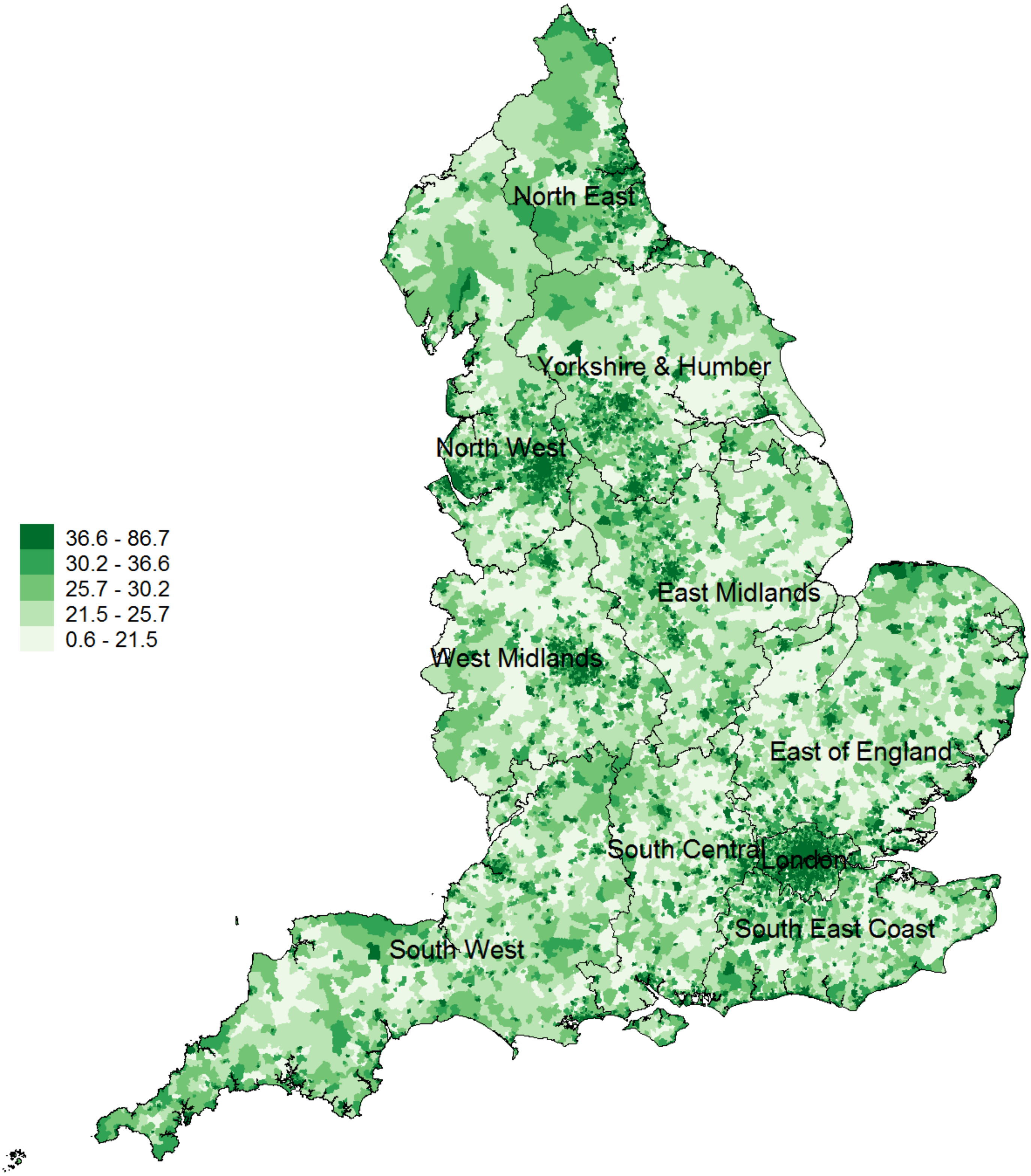

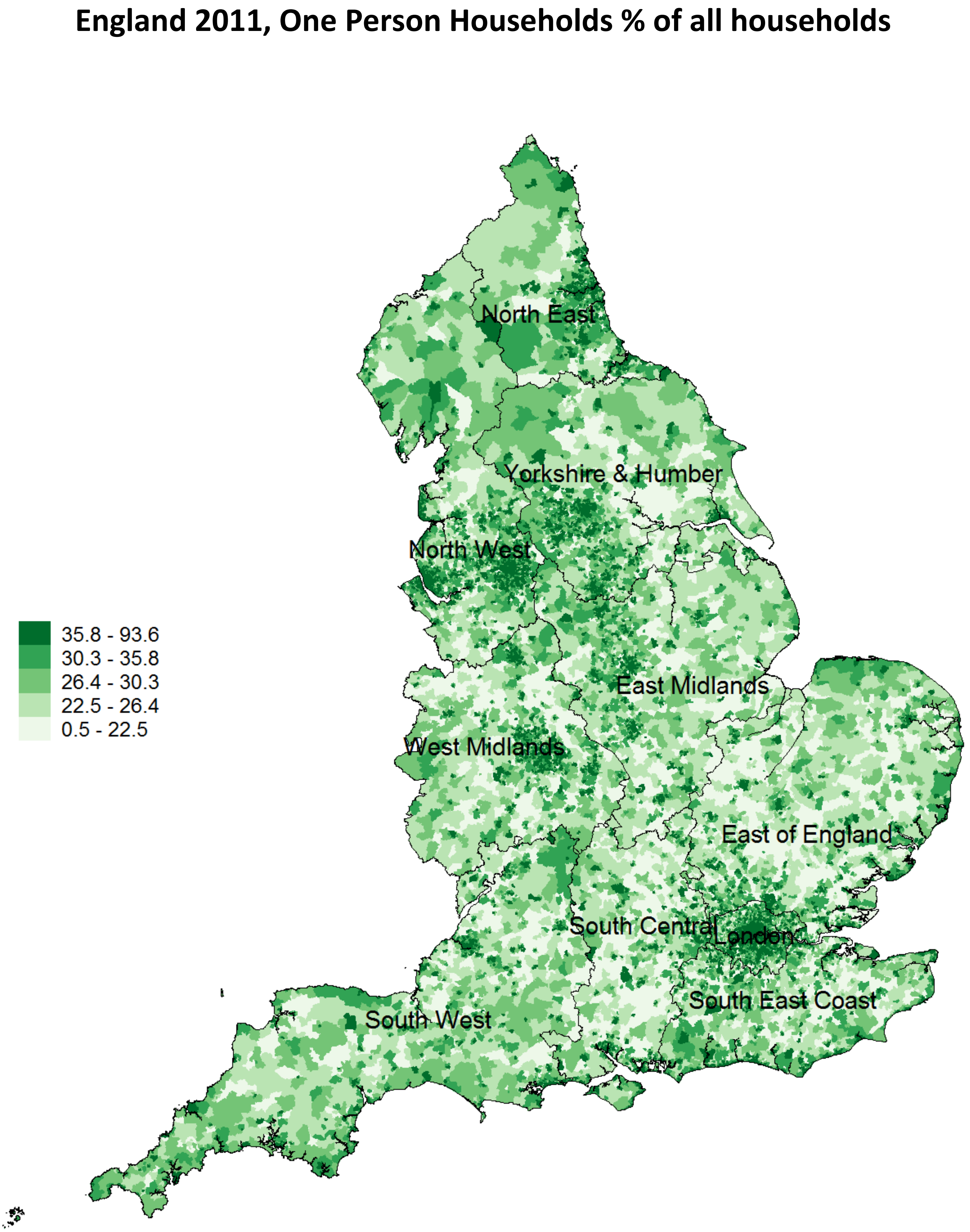
England 2011, One Person Households % of all households
35.8 - 93.6
30.3 - 35.8
26.4 - 30.3
22.5 - 26.4
0.5 - 22.5
North East
Yorkshire & Humber
North West
East Midlands
West Midlands
East of England
South Central
London
South East Coast
South West

North East, One Person Households % of all households

2001

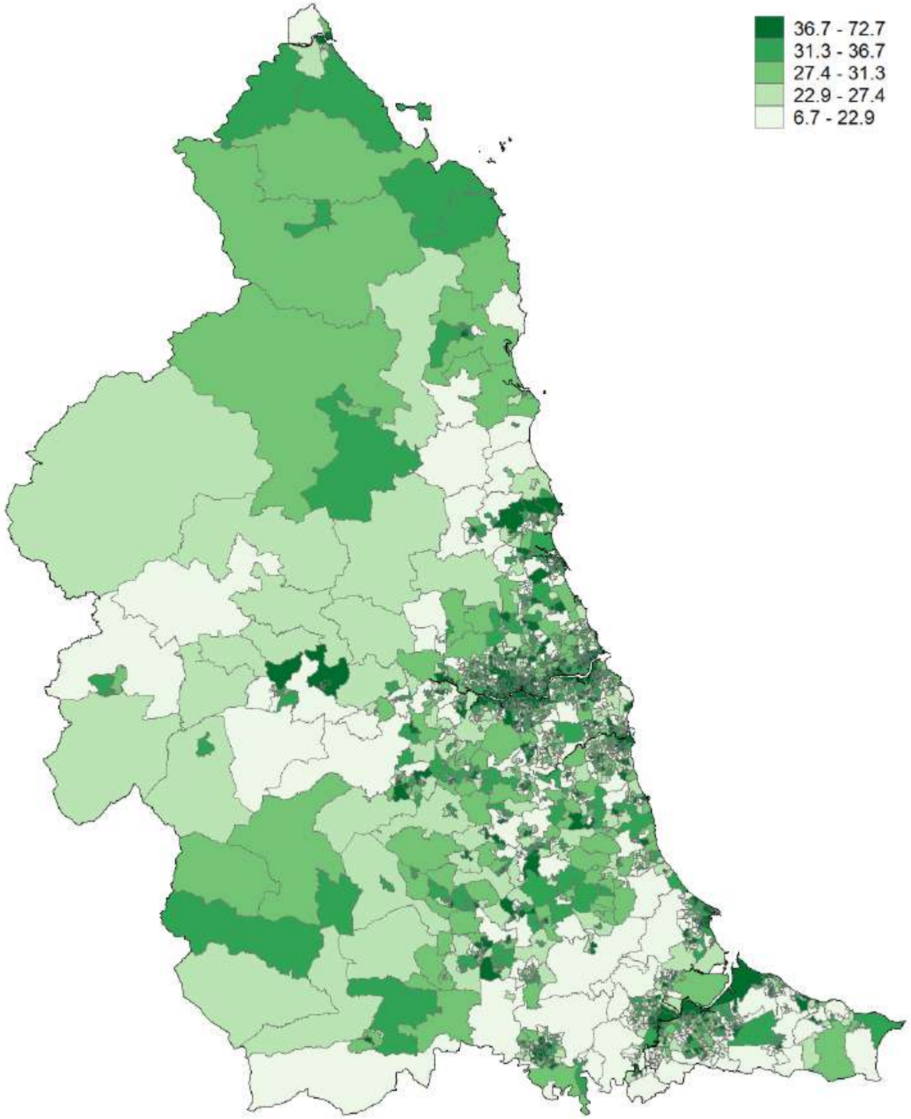

2011

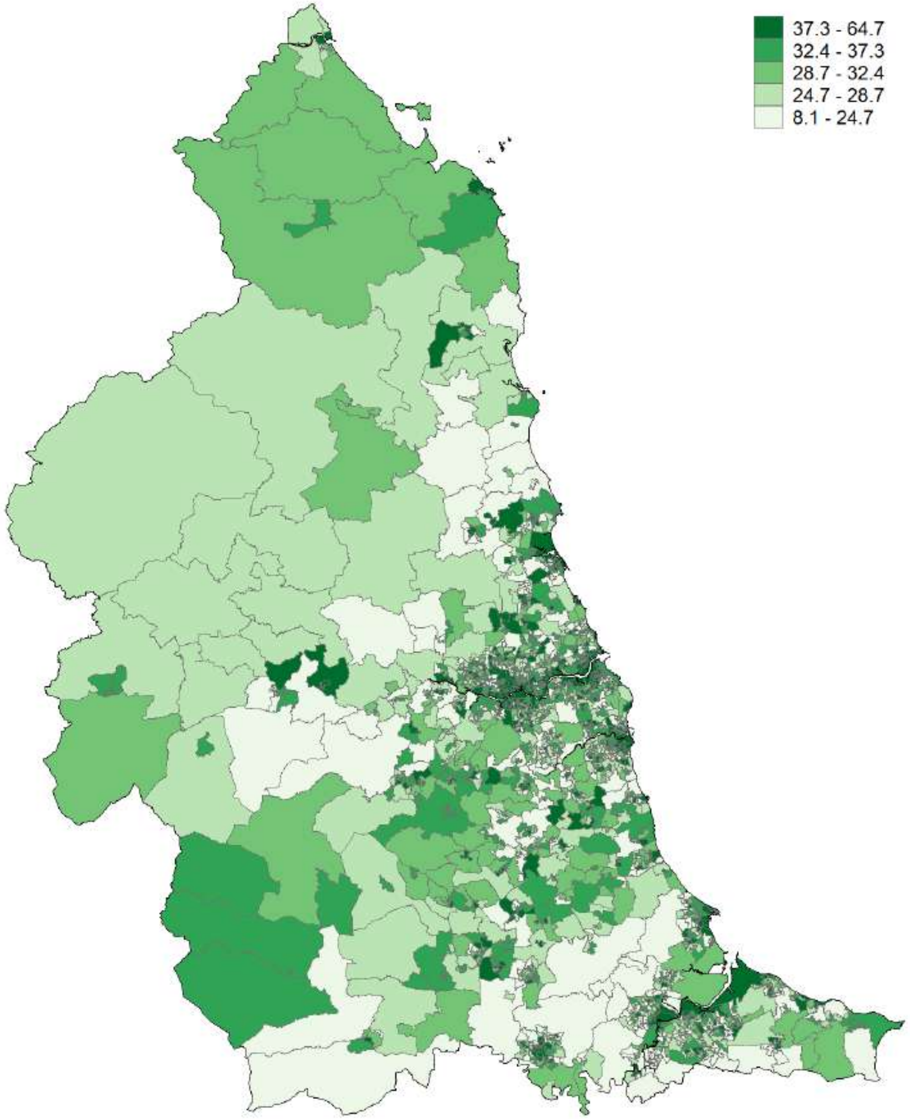

North West, One Person Households % of all households

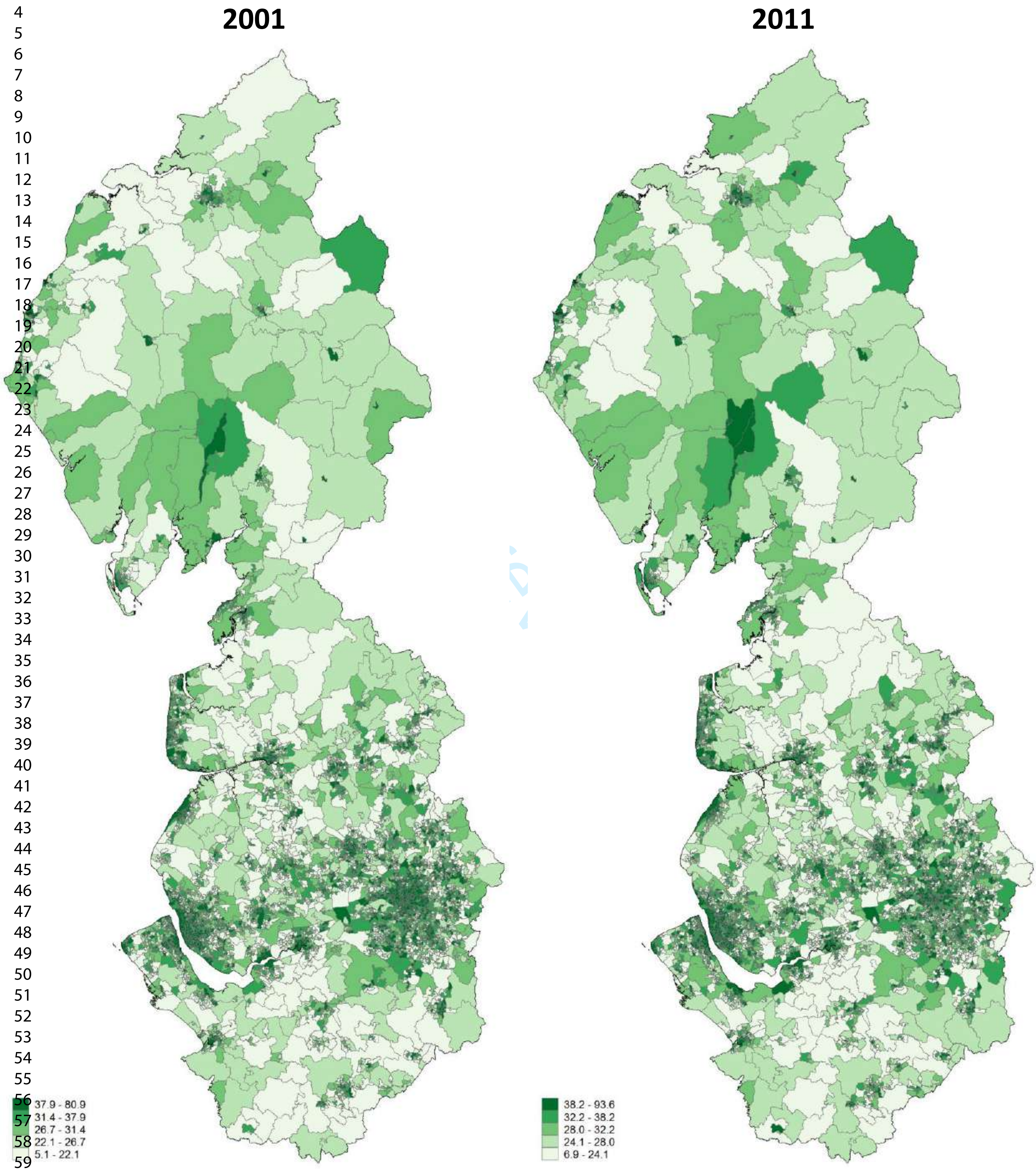

Yorkshire and the Humber, One Person Households % of all households

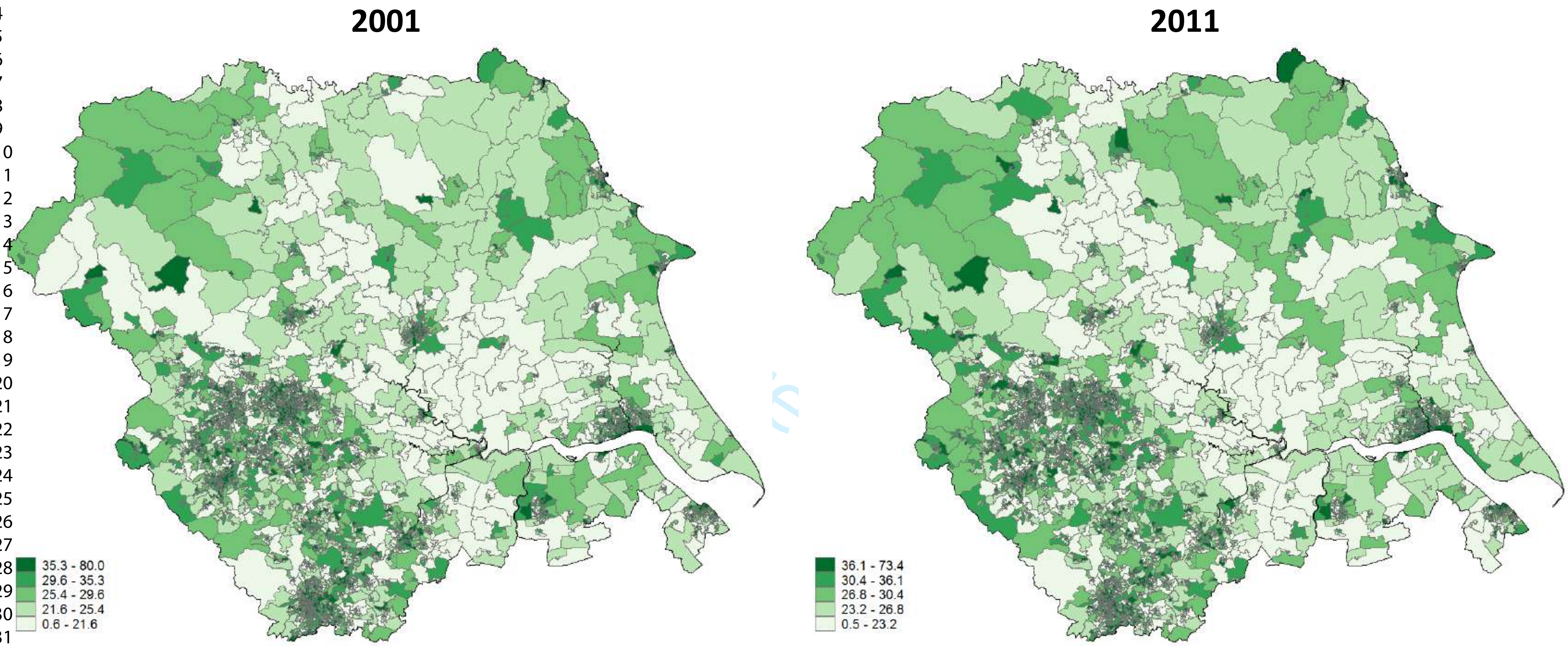

East Midlands, One Person Households % of all households

2001 2011

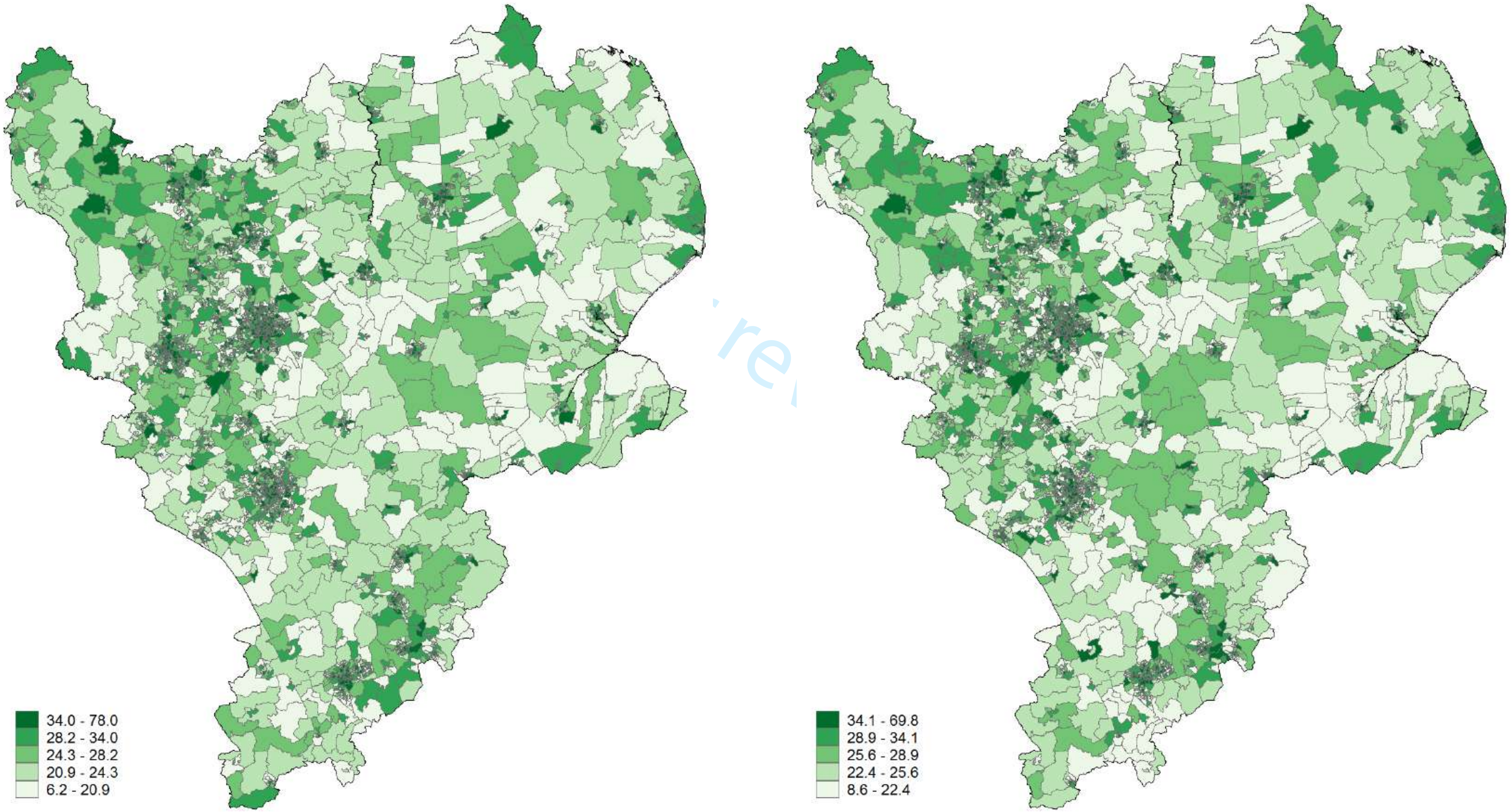

West Midlands, One Person Households % of all households

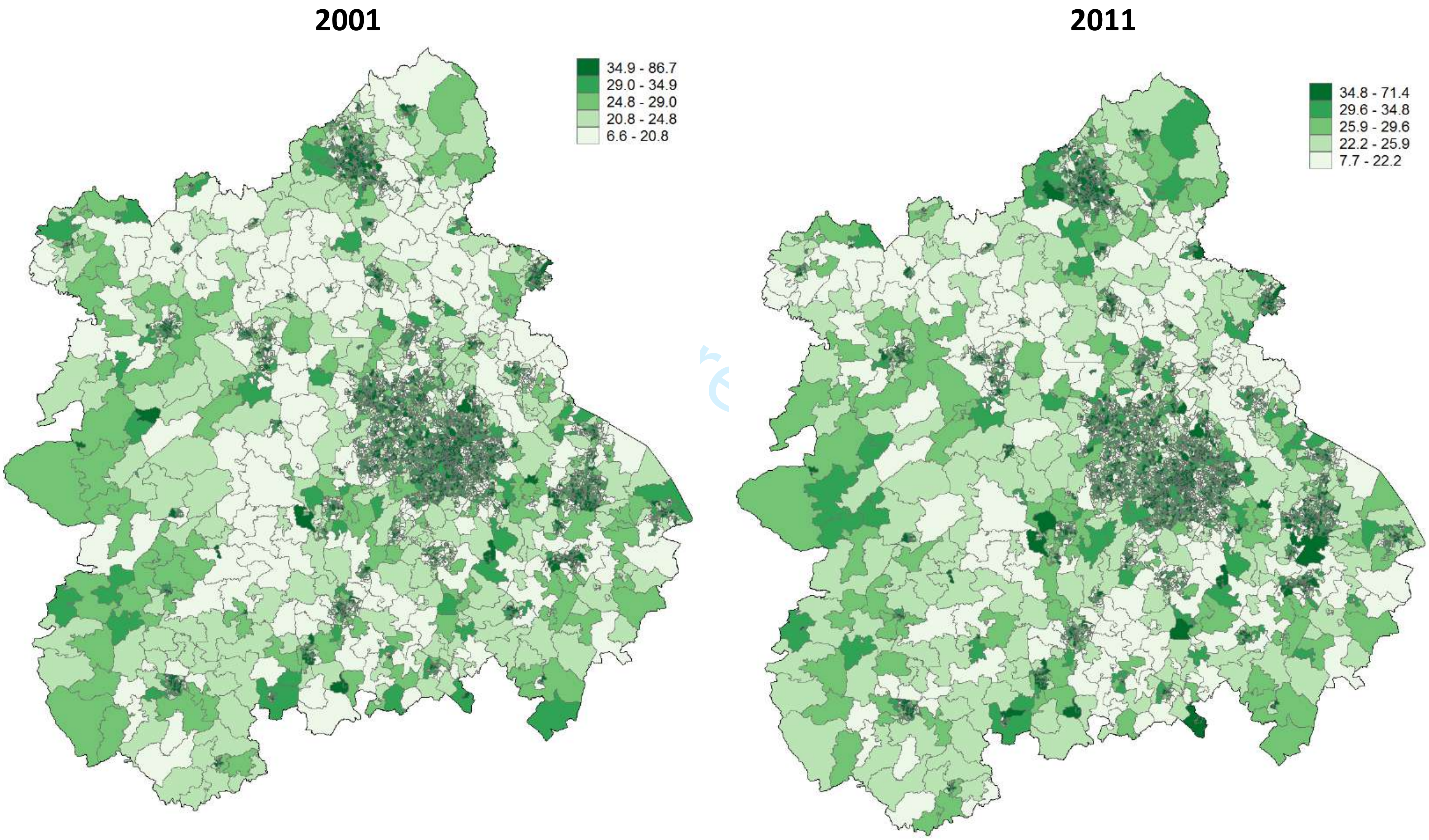

East of England, One Person Households % of all households

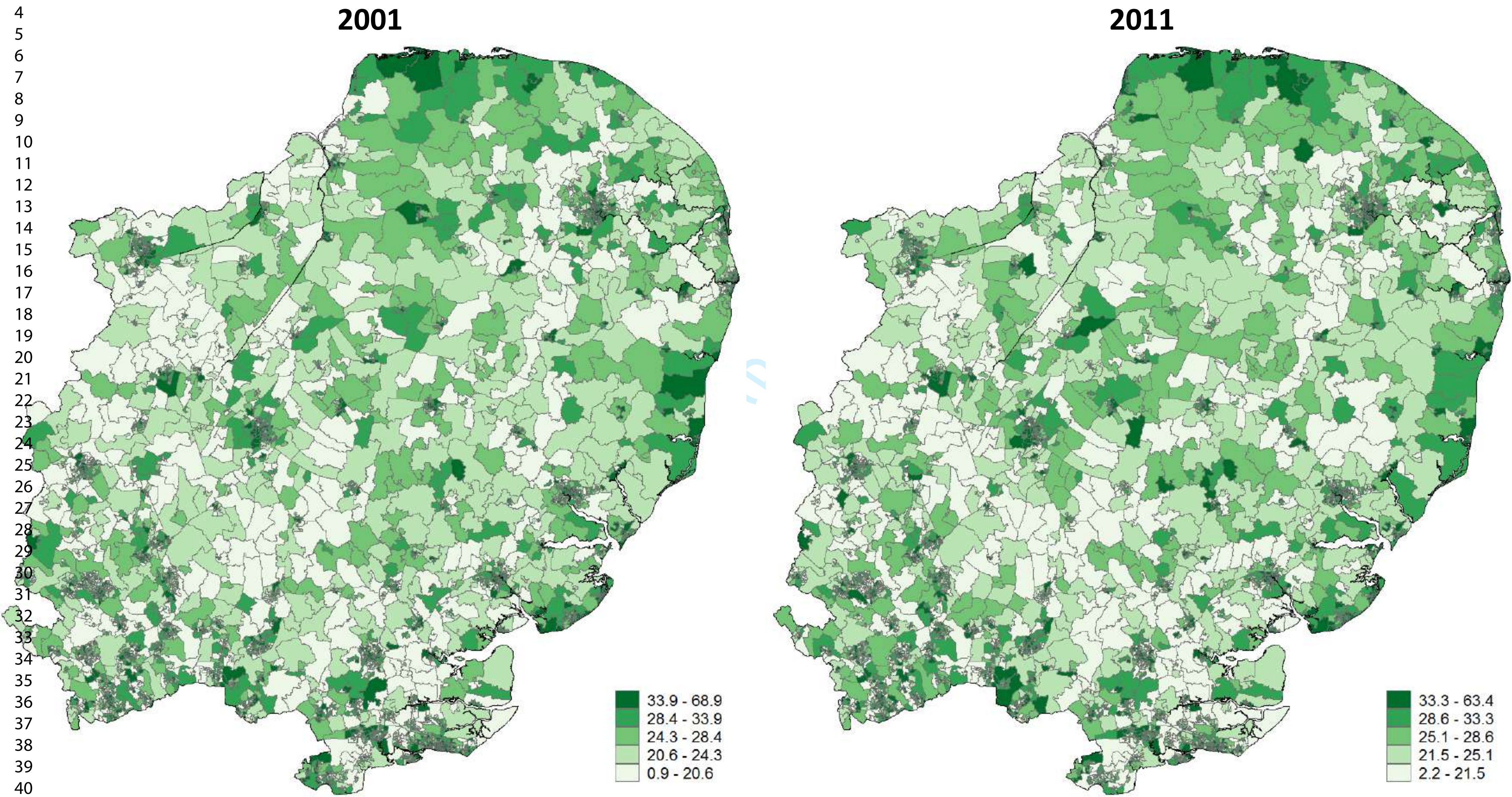

London, One Person Households % of all households

2001

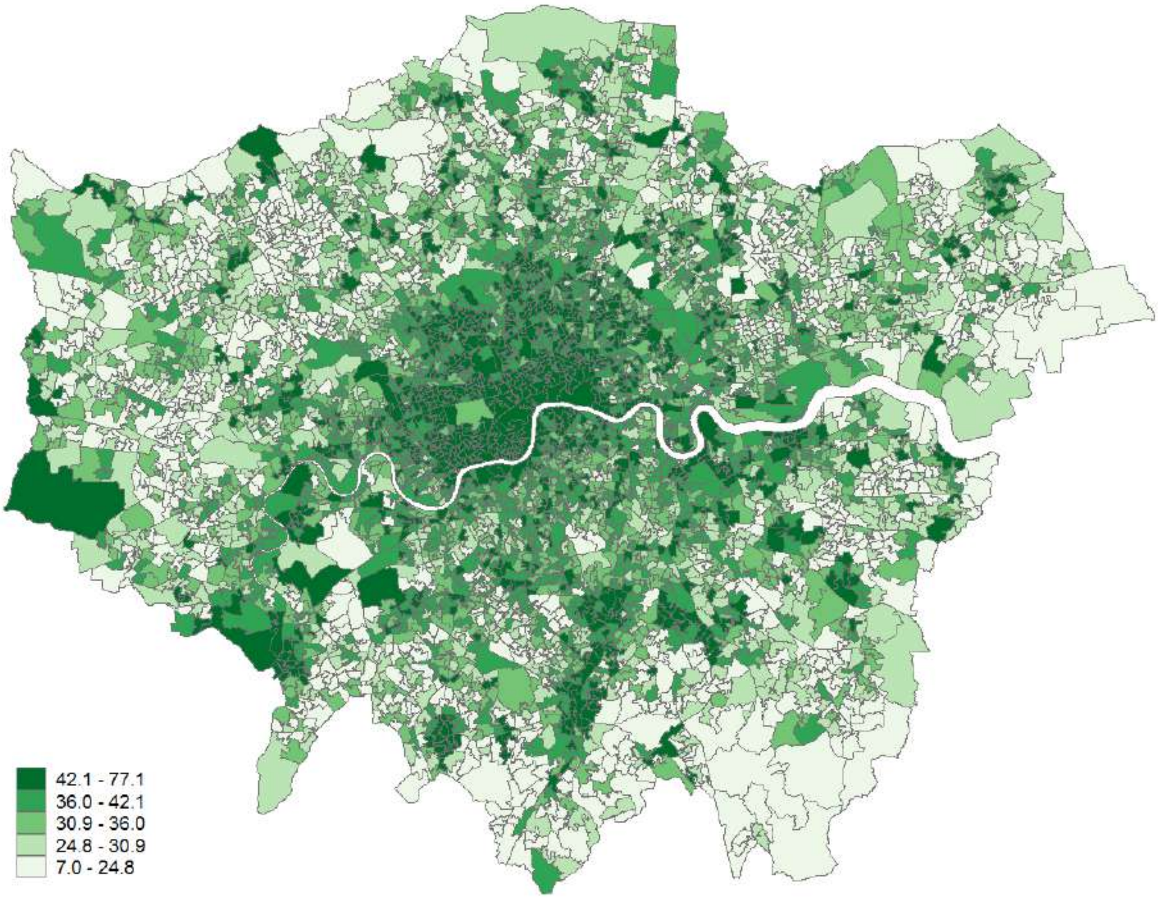

2011

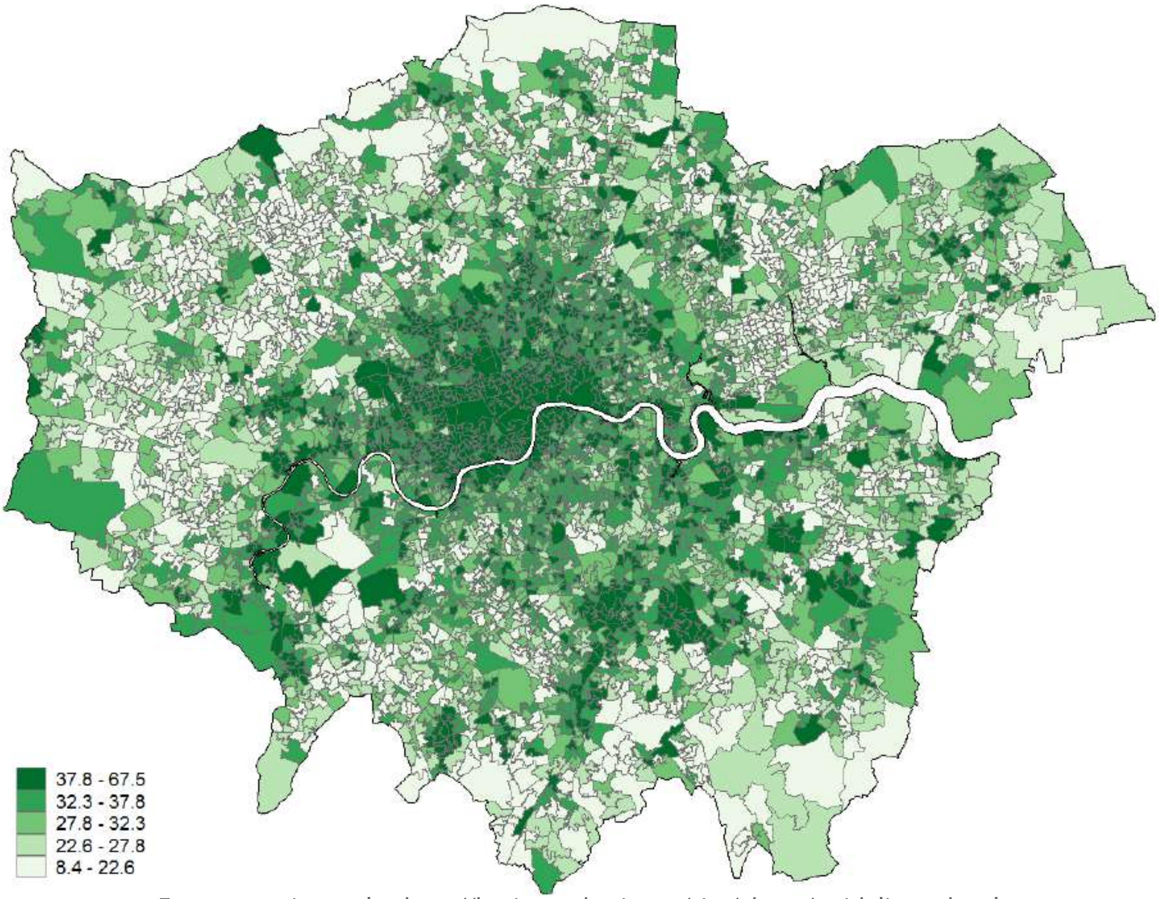

South East Coast, One Person Households % of all households

2001

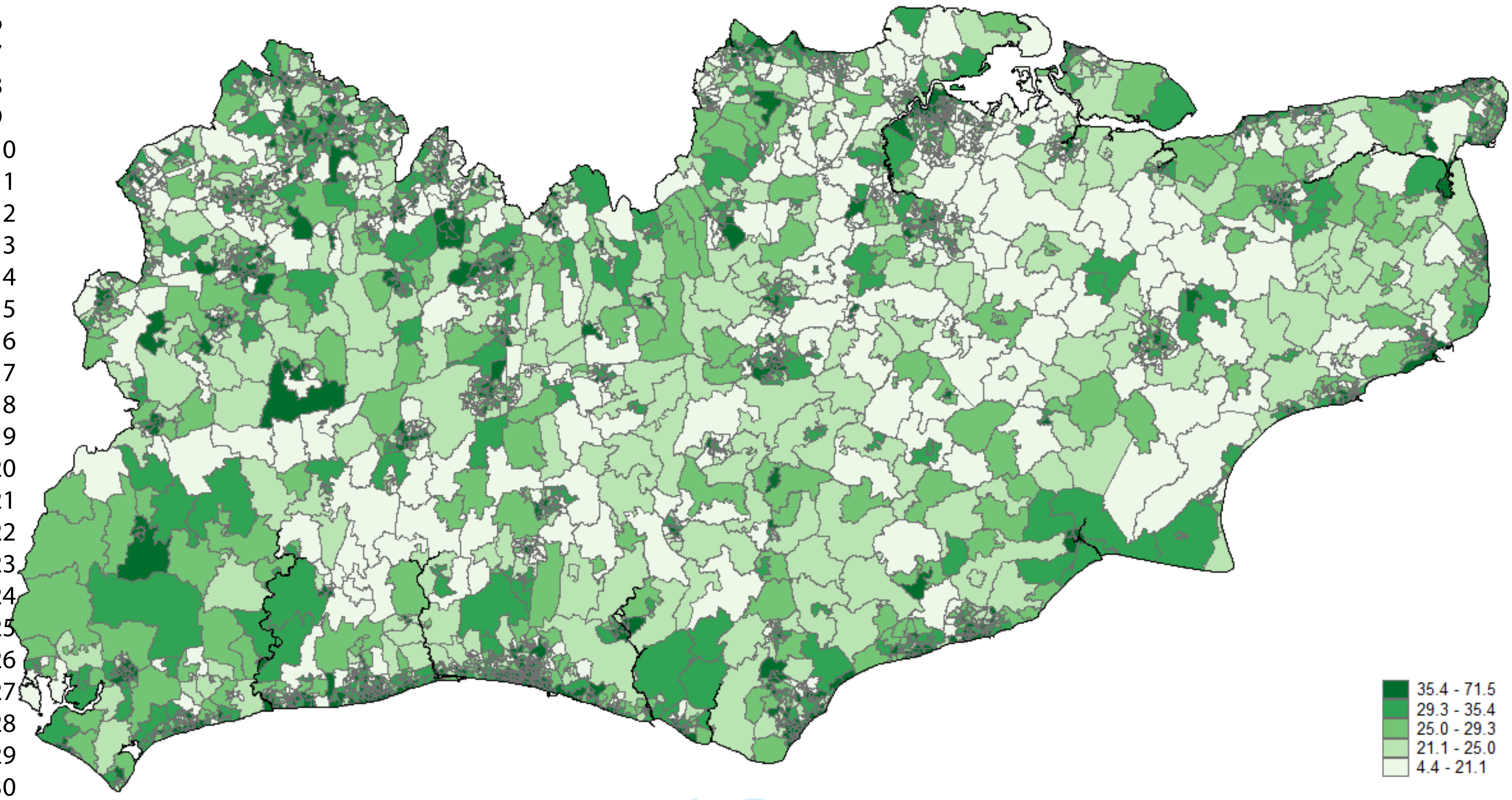

2011

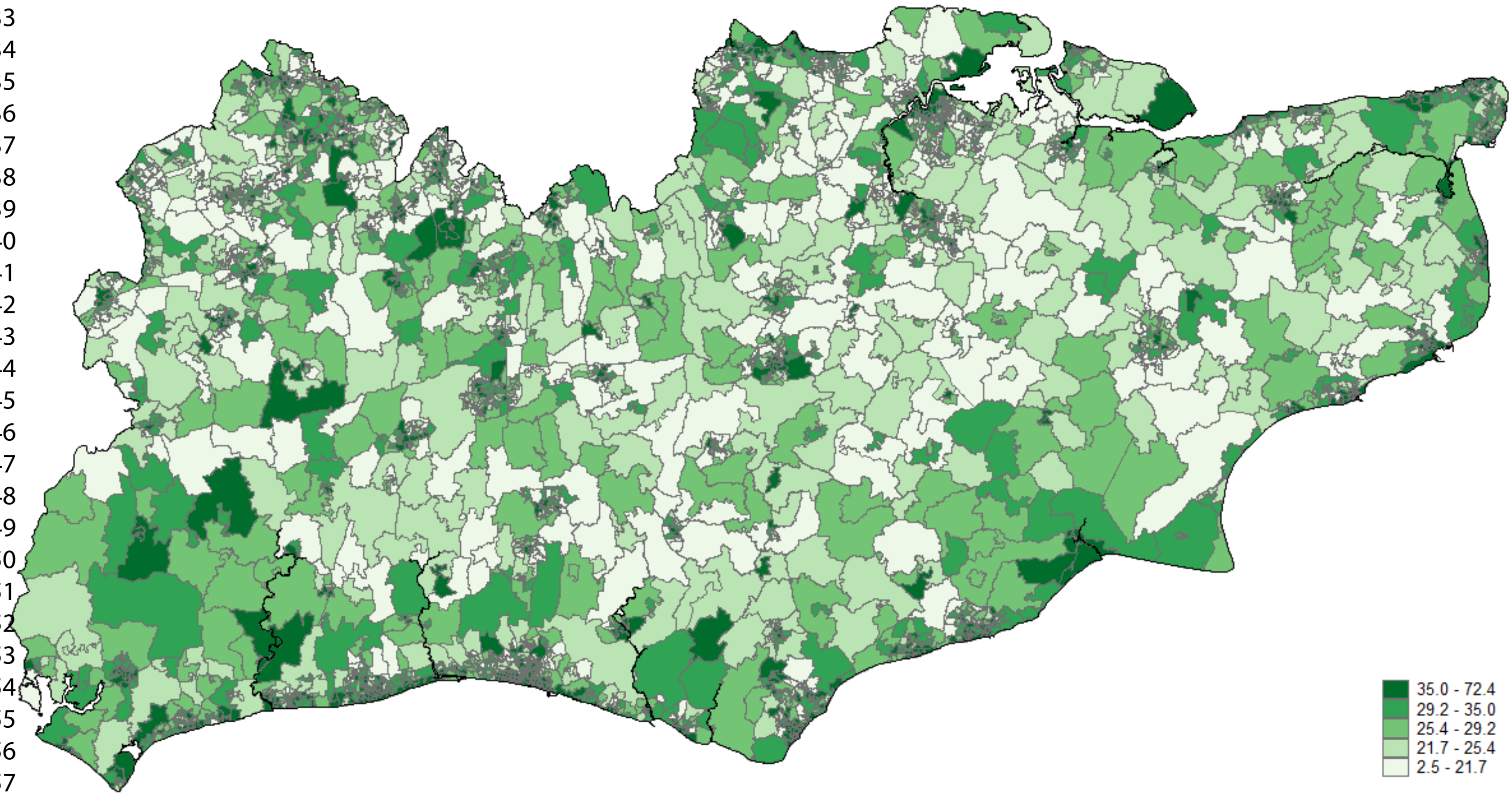

South Central, One Person Households % of all households

2001 **2011**

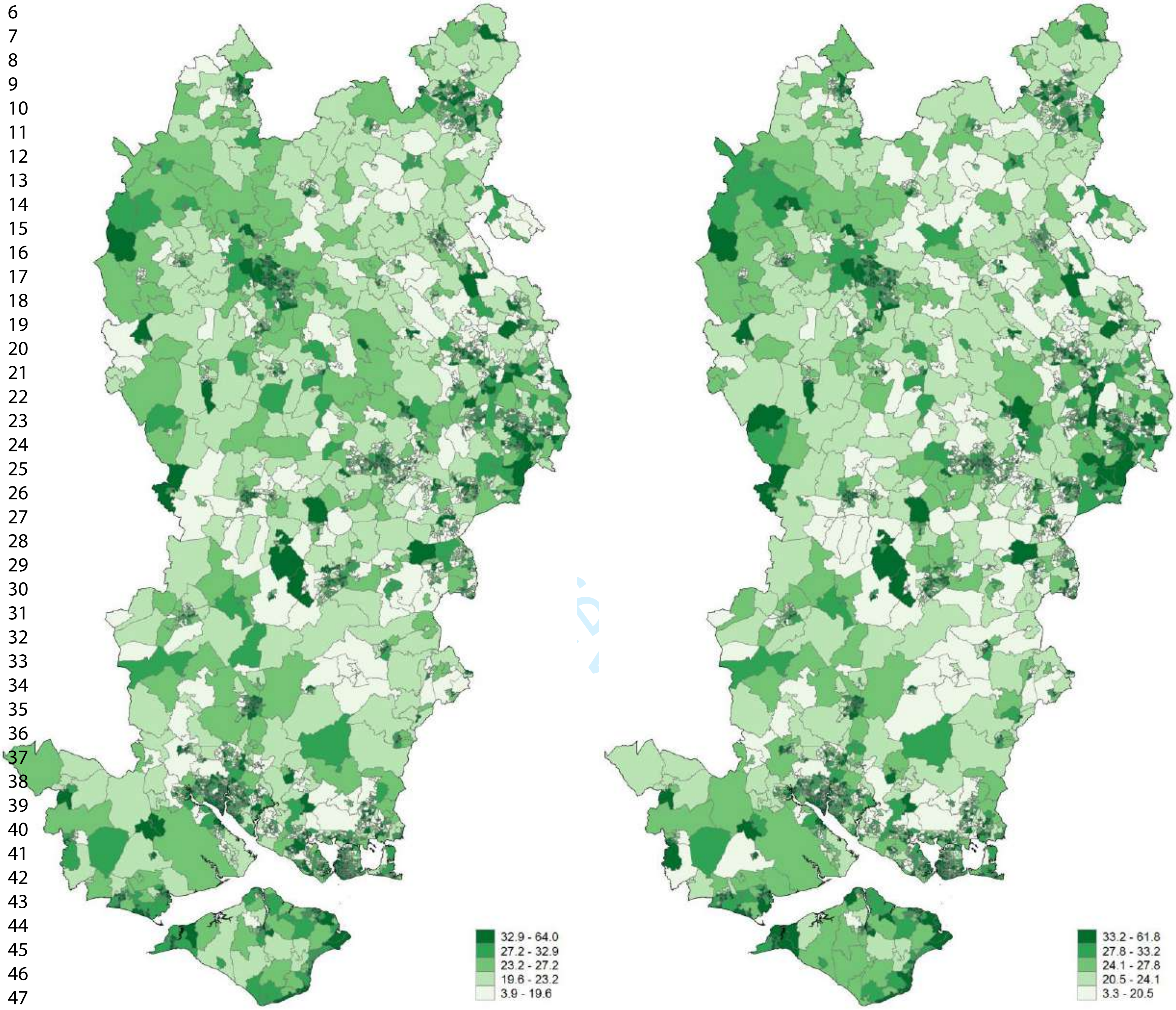

South West, One Person Households % of all households

2001

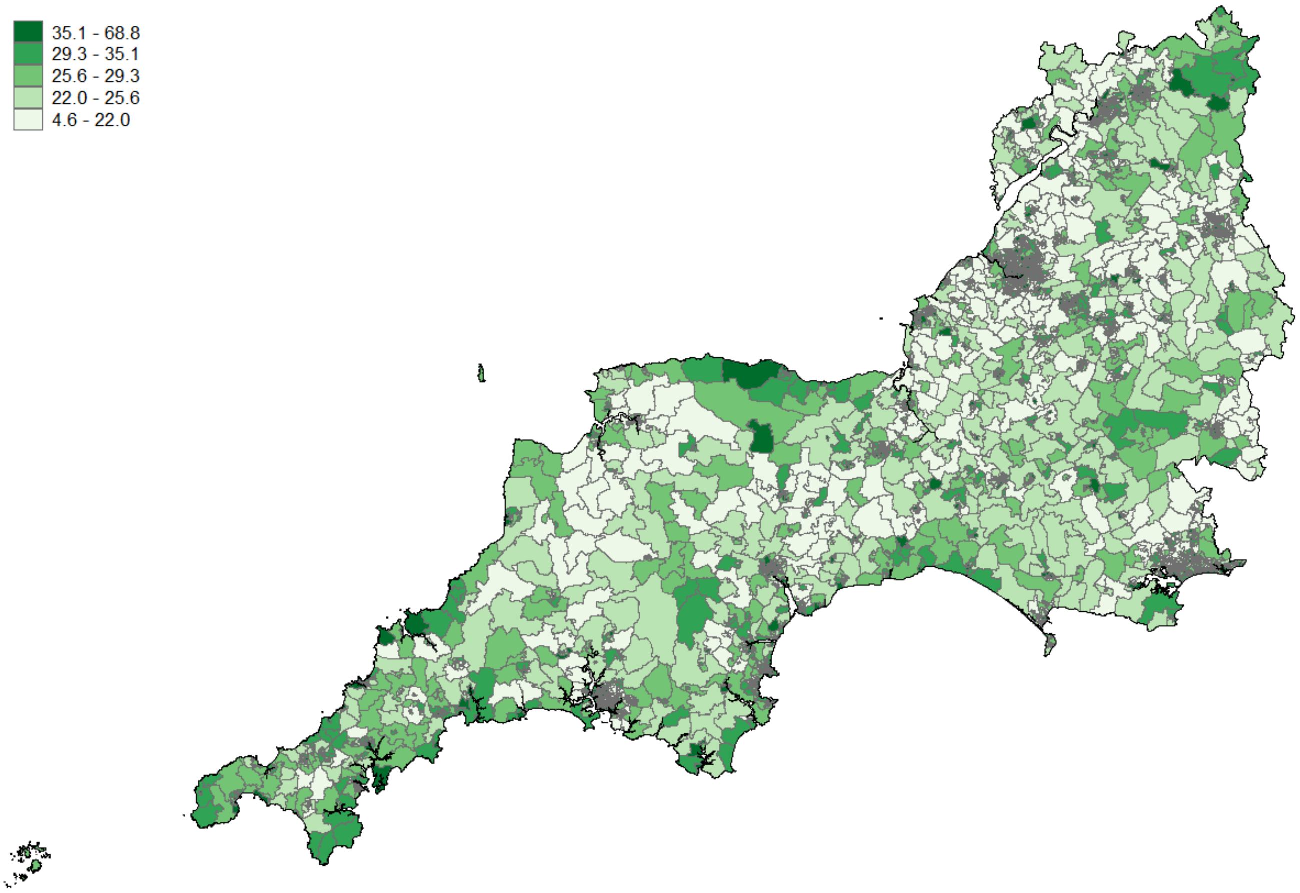

2011

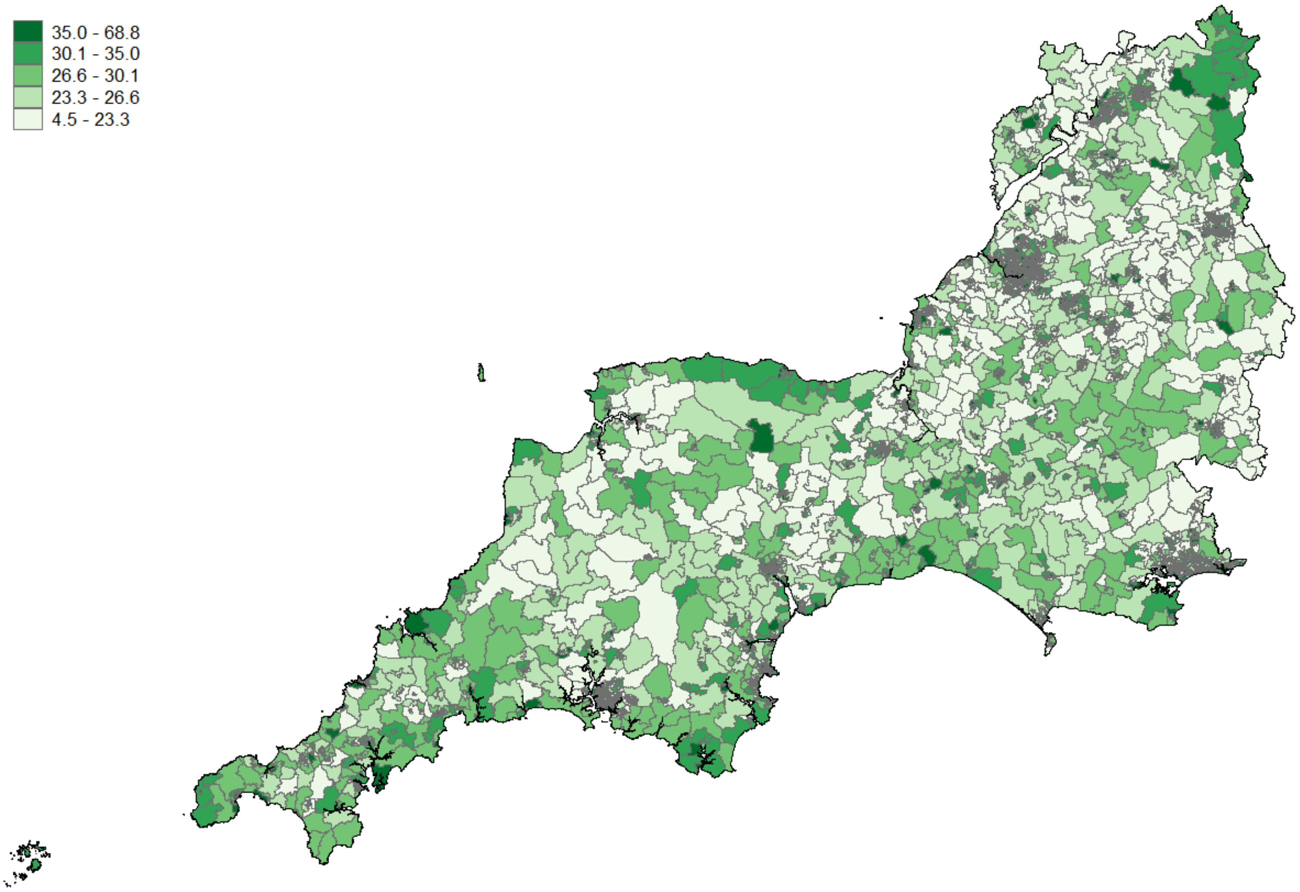

Private renting

England 2001, Private Renting % of all households

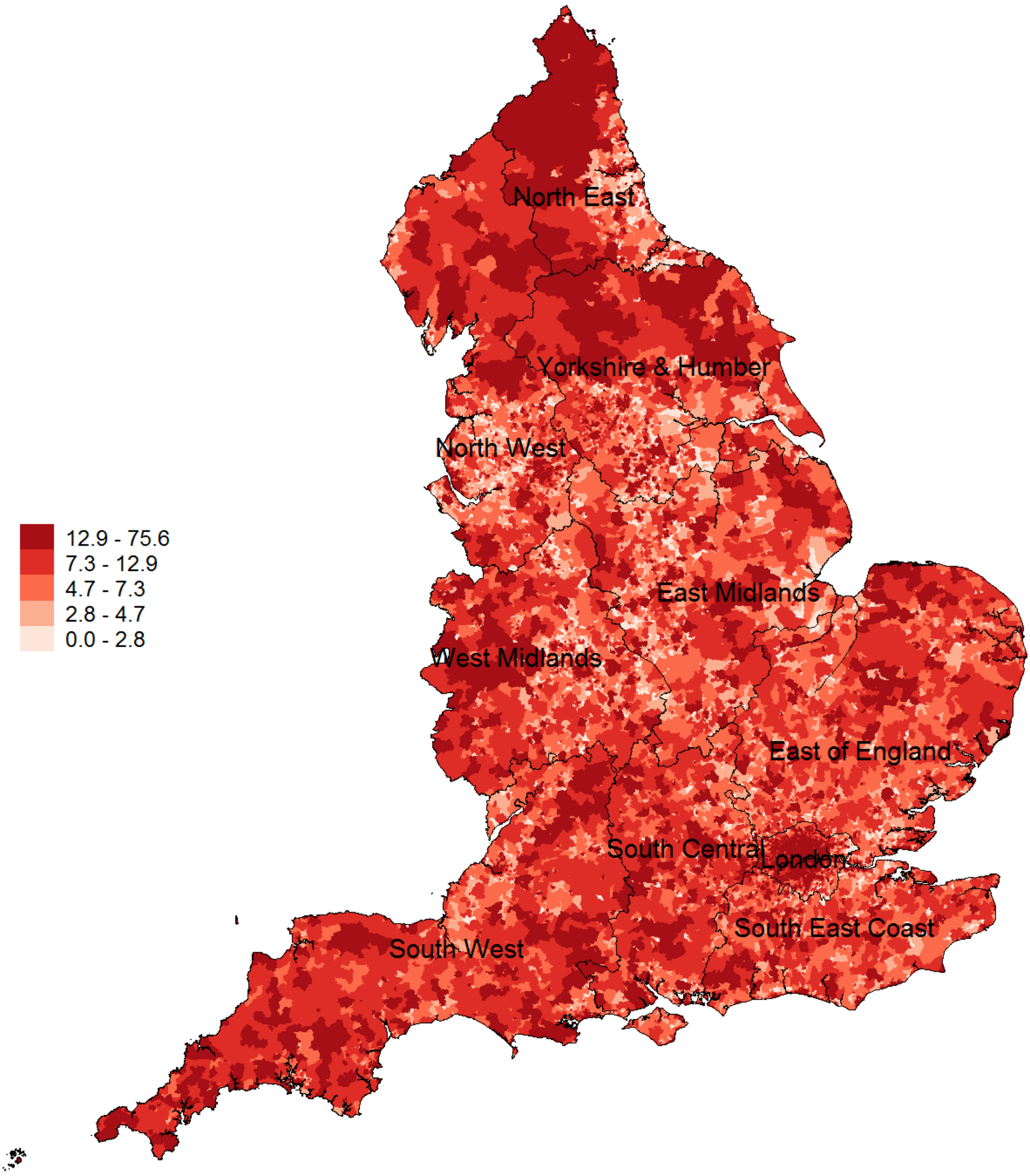

England 2011, Private Renting % of all households

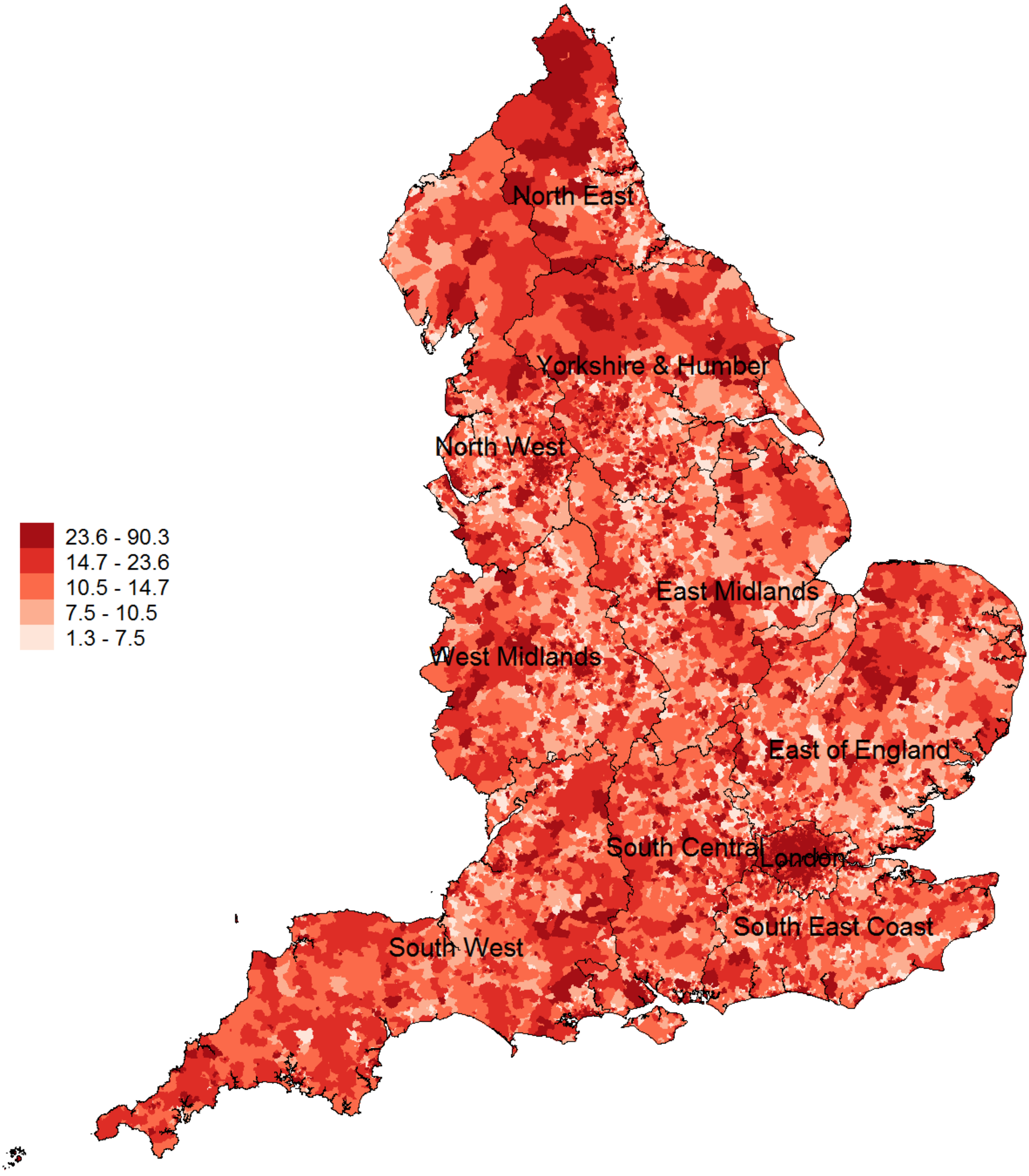

North East, Private Renting % of all households

2001

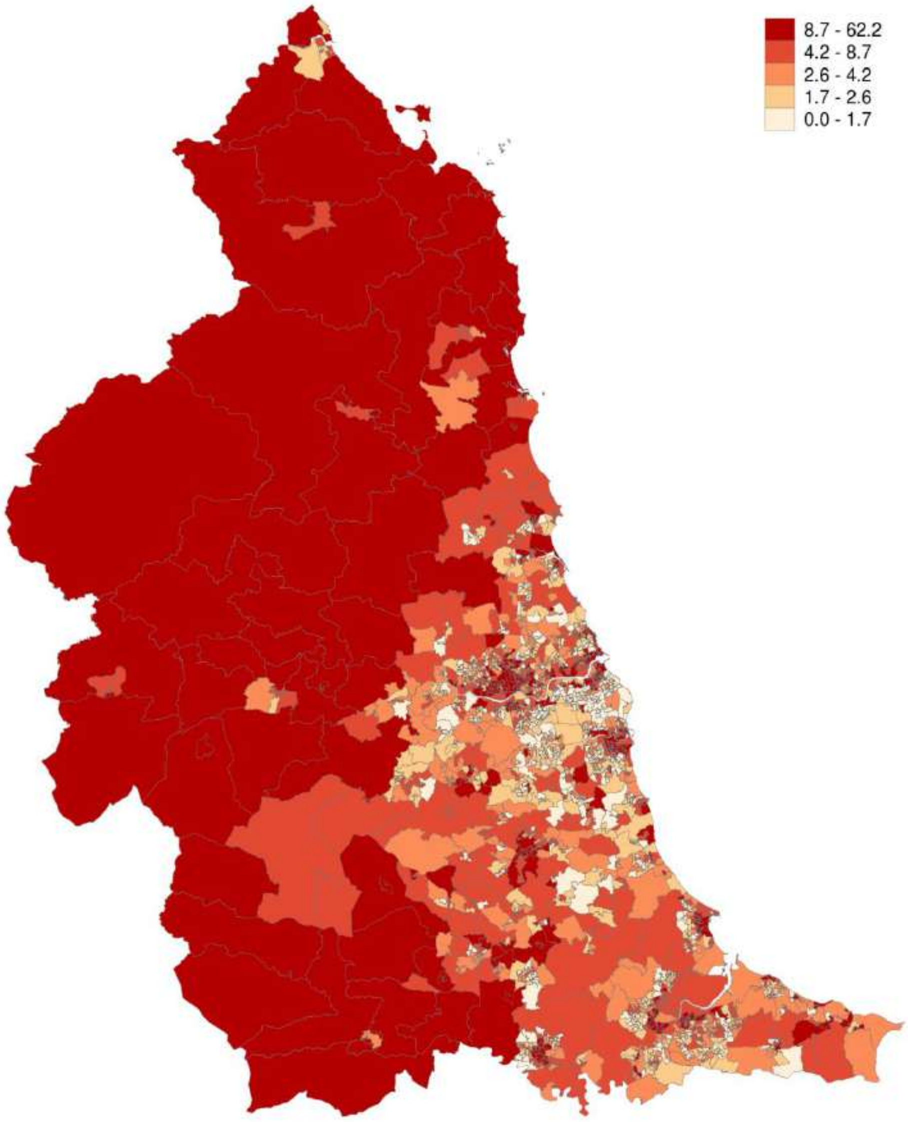

2011

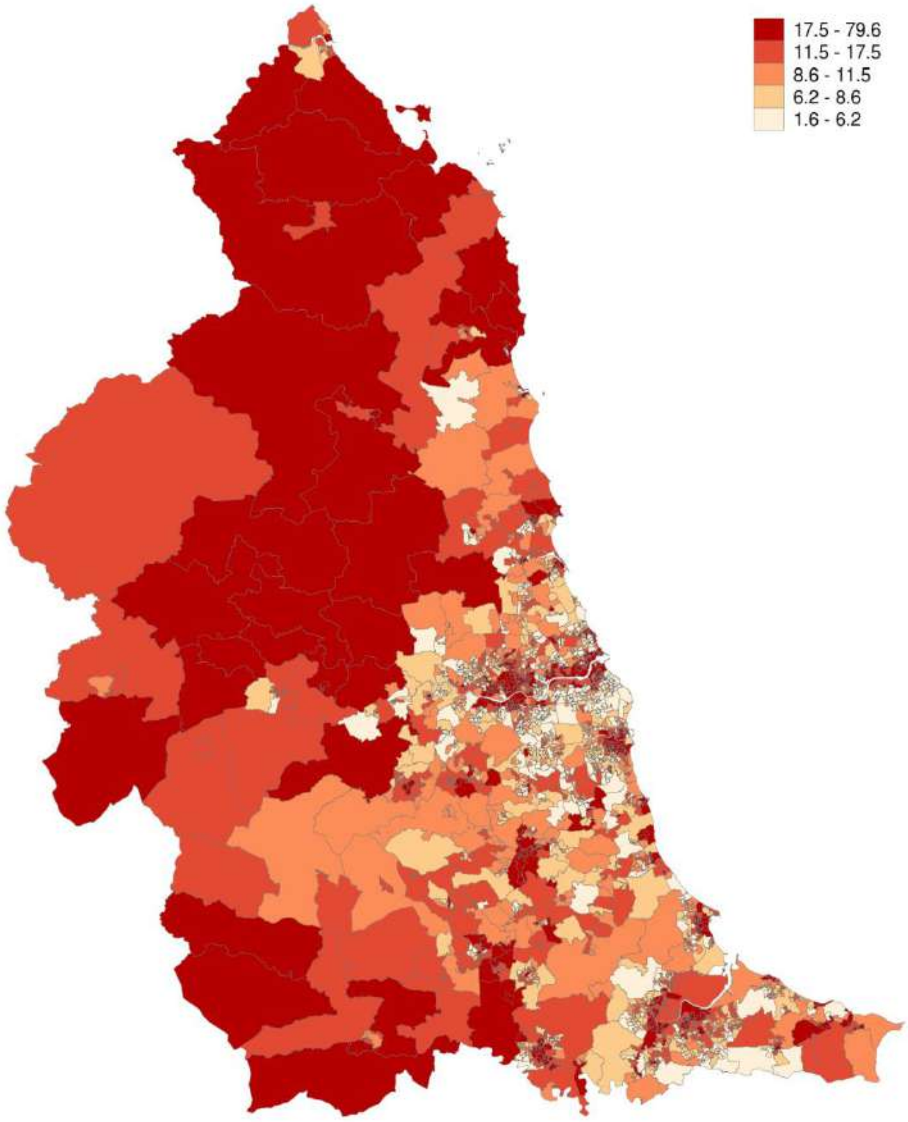

For peer review only - http://bmjopen.bmj.com/site/about/guidelines.xhtml

North West, Private Renting % of all households

2001 **2011**

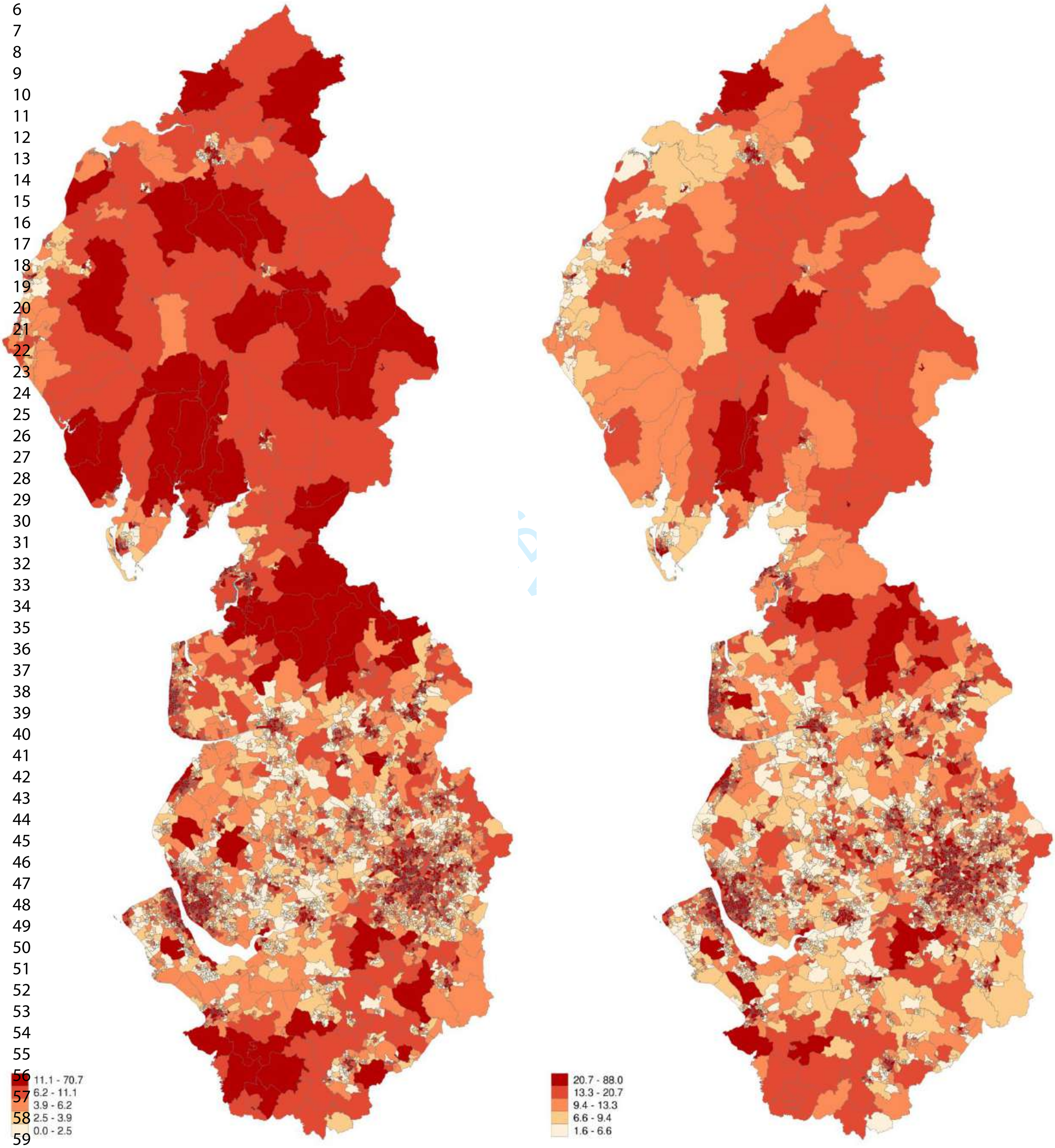

Yorkshire and the Humber, Private Renting % of all households

2001

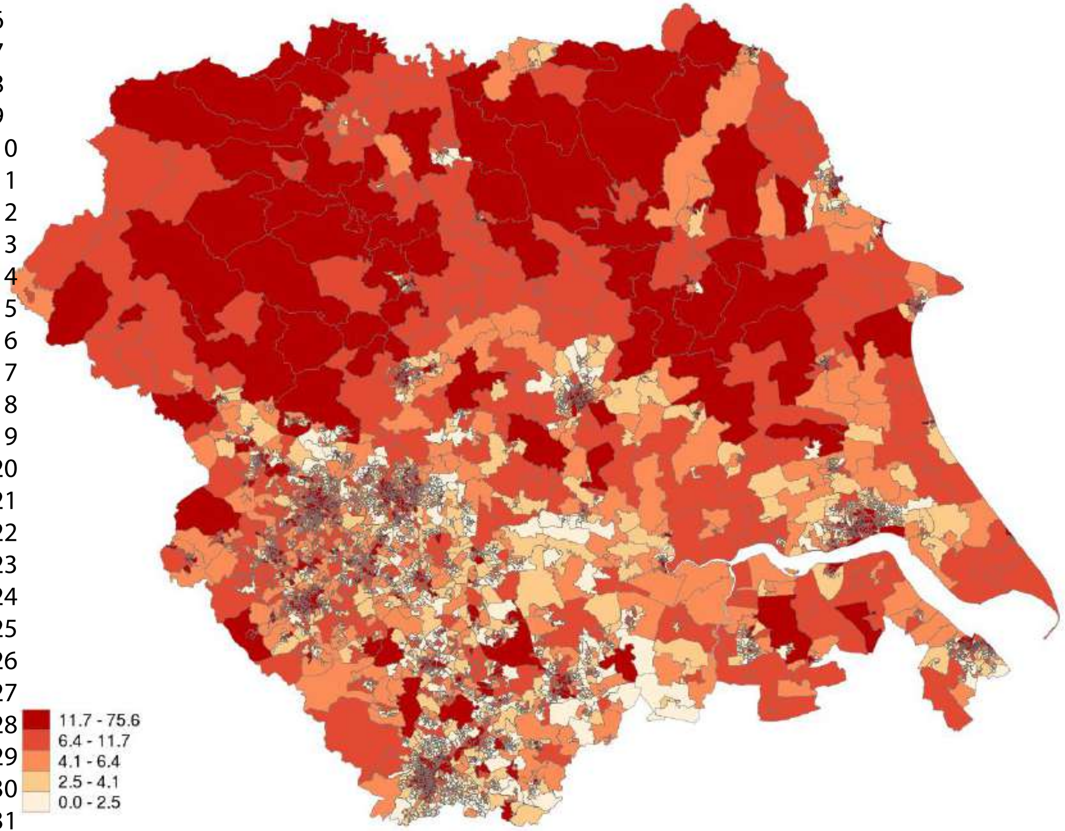

2011

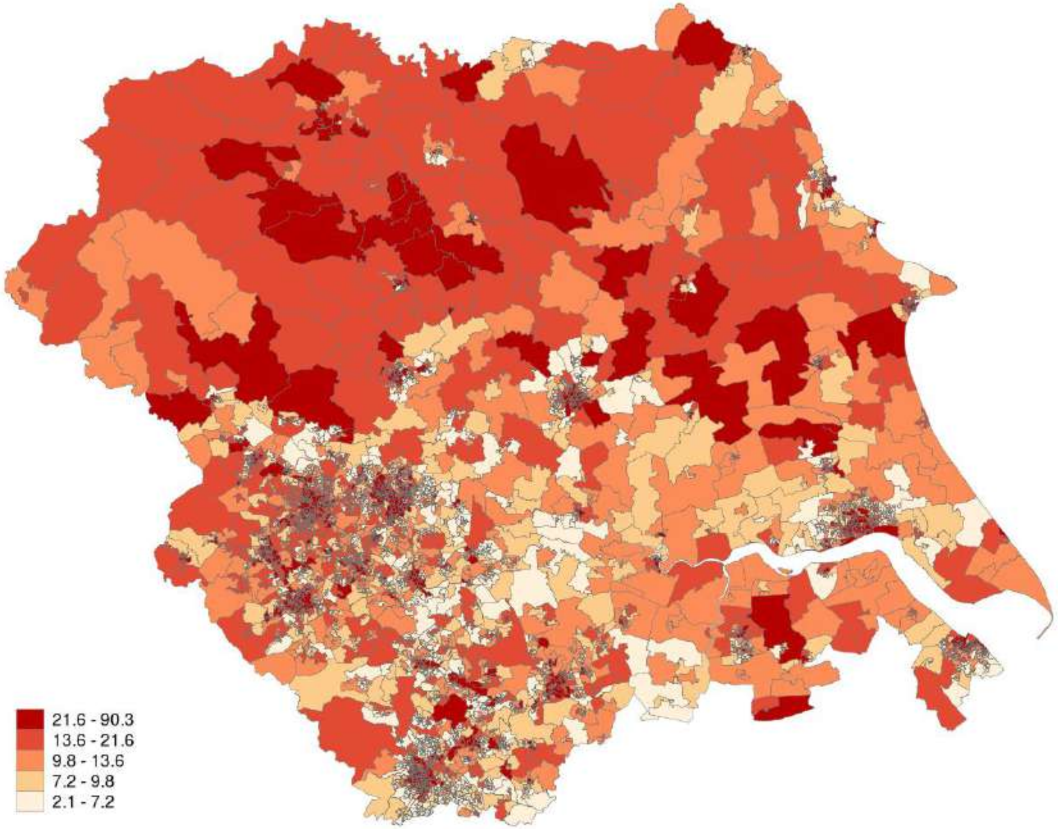

East Midlands, Private Renting % of all households

2001

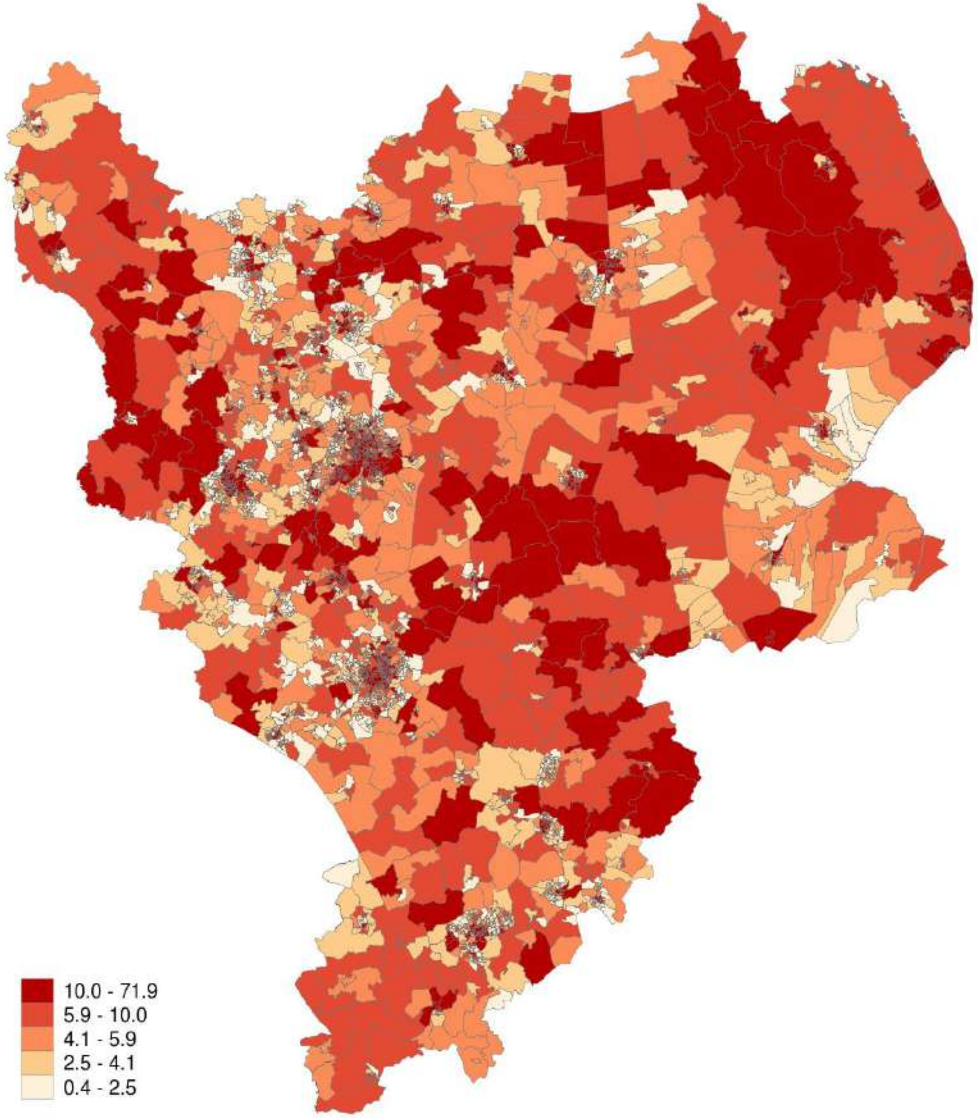

2011

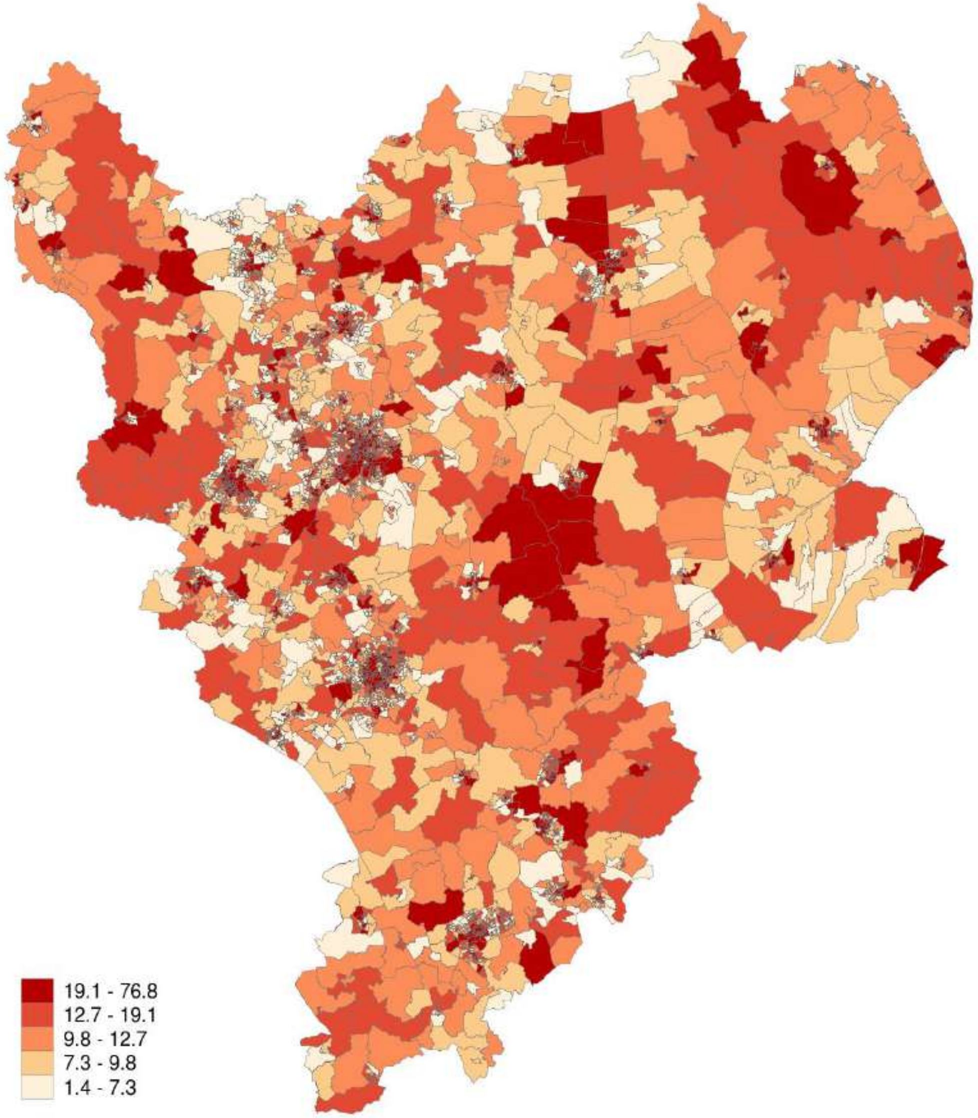

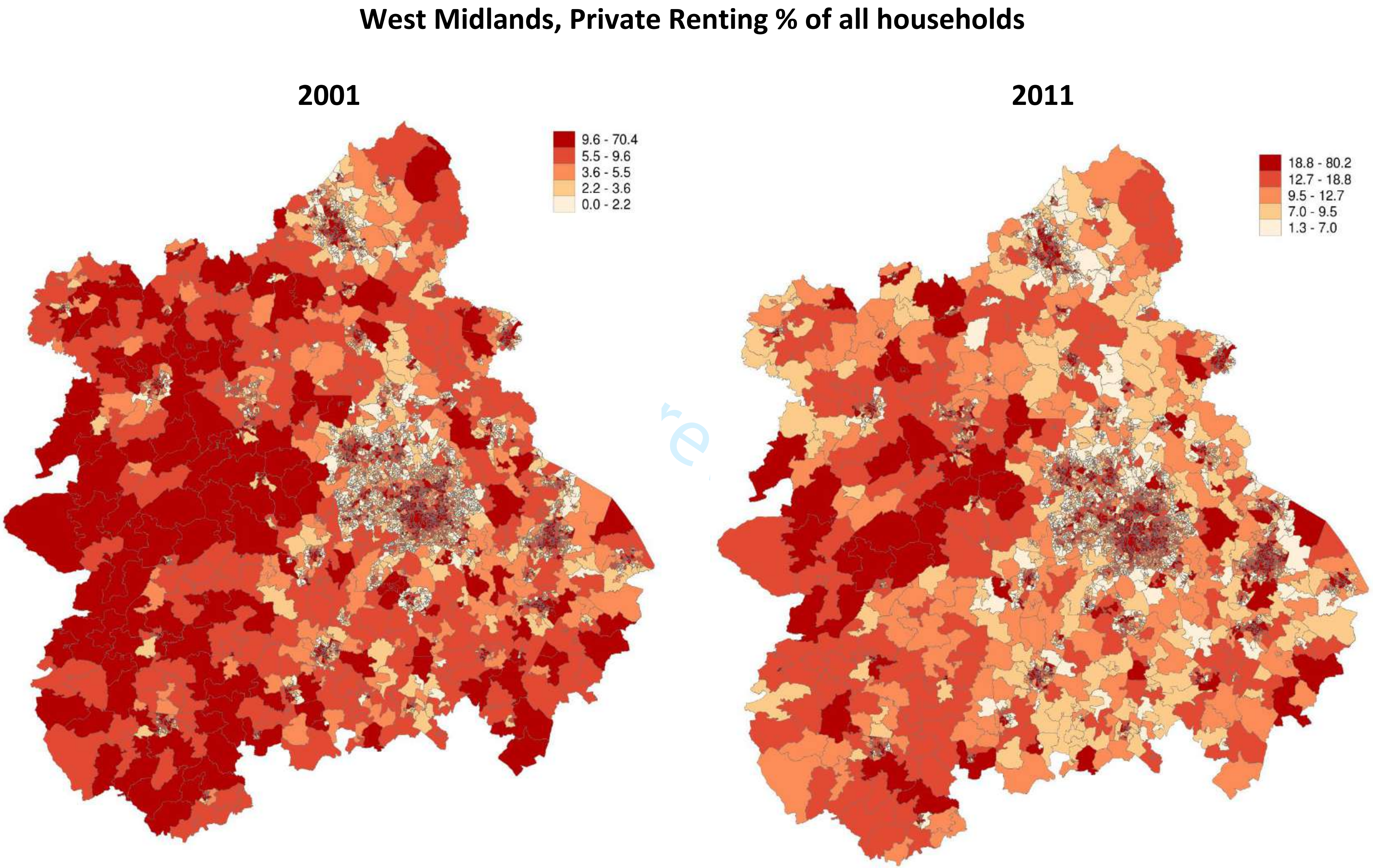
West Midlands, Private Renting % of all households
2001
9.6 - 70.4
5.5 - 9.6
3.6 - 5.5
2.2 - 3.6
0.0 - 2.2
2011
18.8 - 80.2
12.7 - 18.8
9.5 - 12.7
7.0 - 9.5
1.3 - 7.0

East of England, Private Renting % of all households

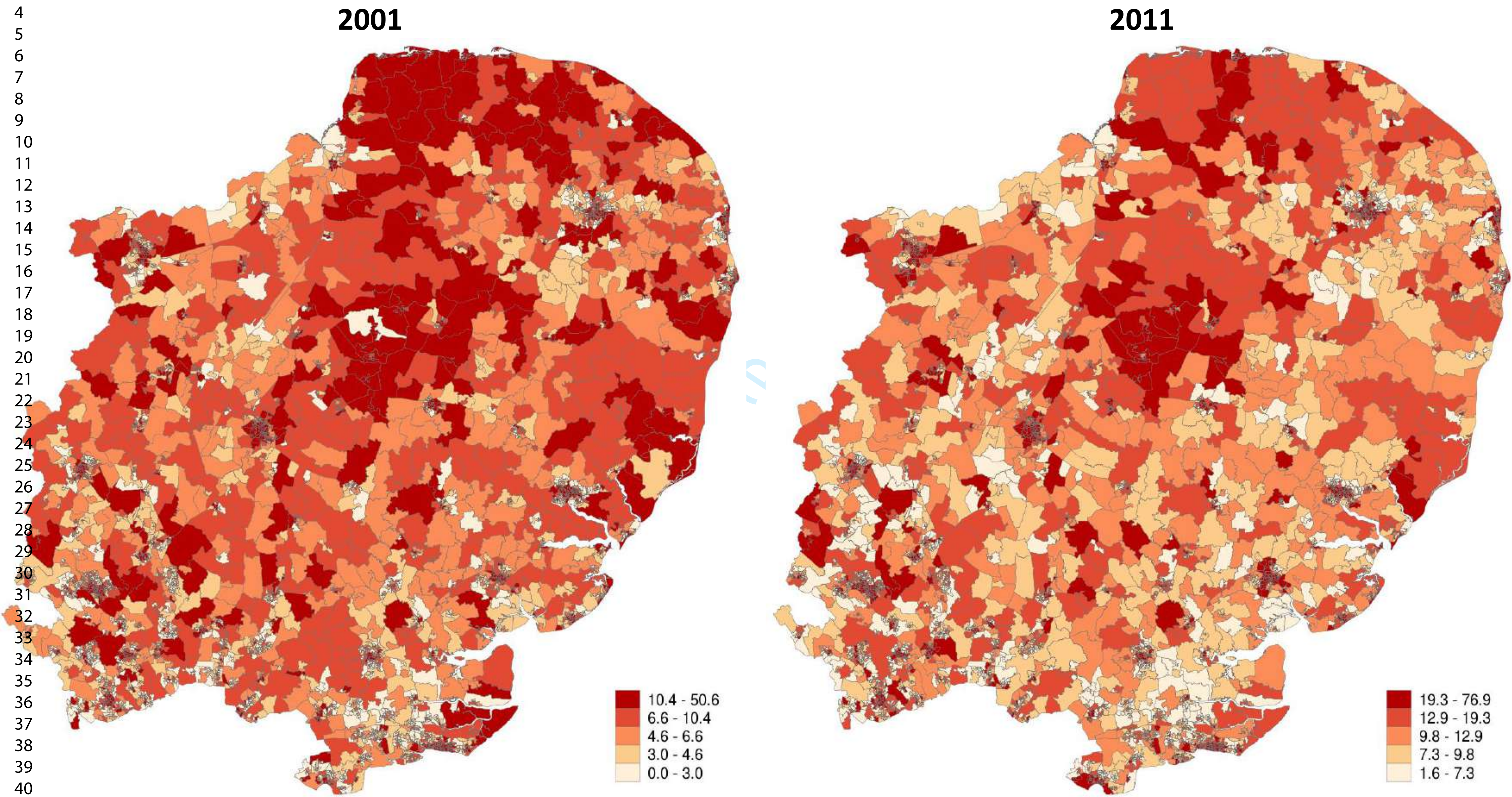

London, Private Renting % of all households

2001

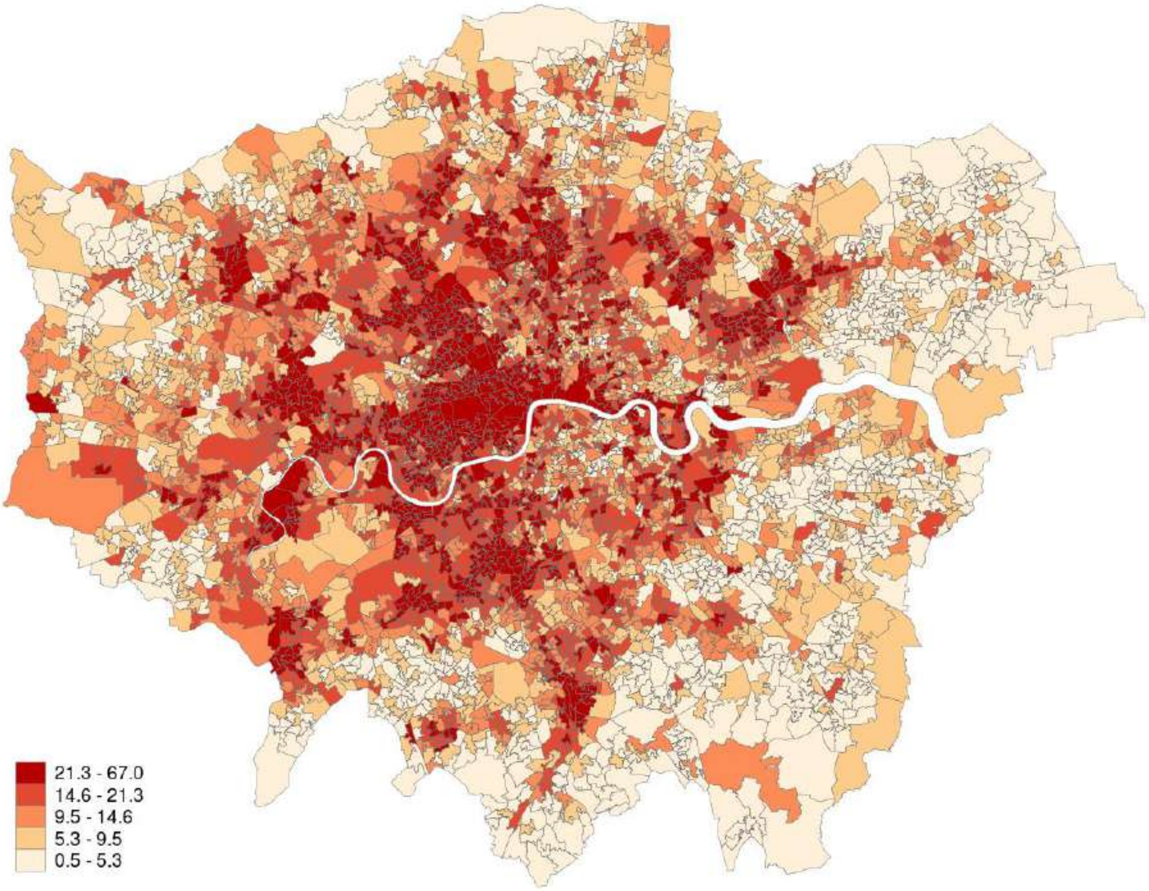

2011

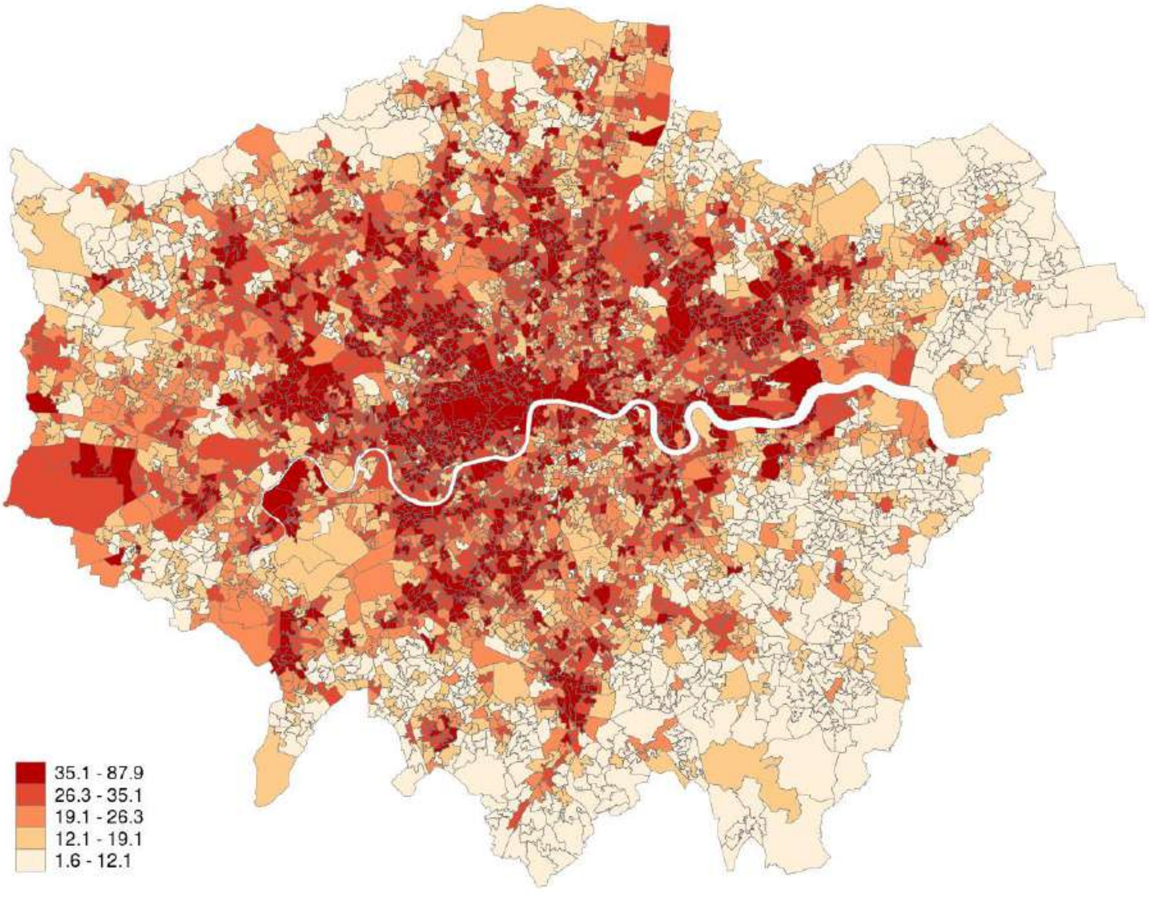

South East Coast, Private Renting % of all households

2001

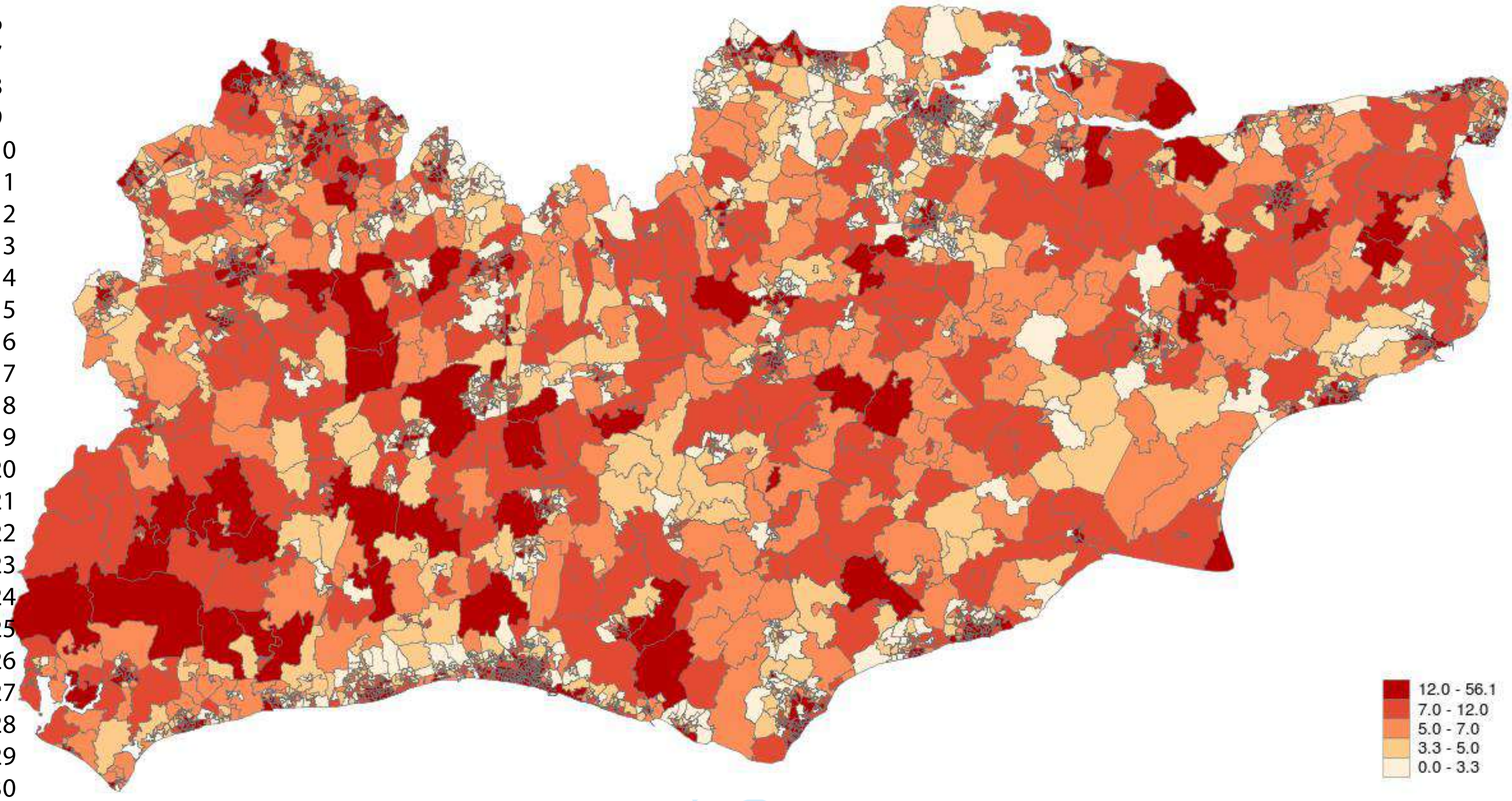

2011

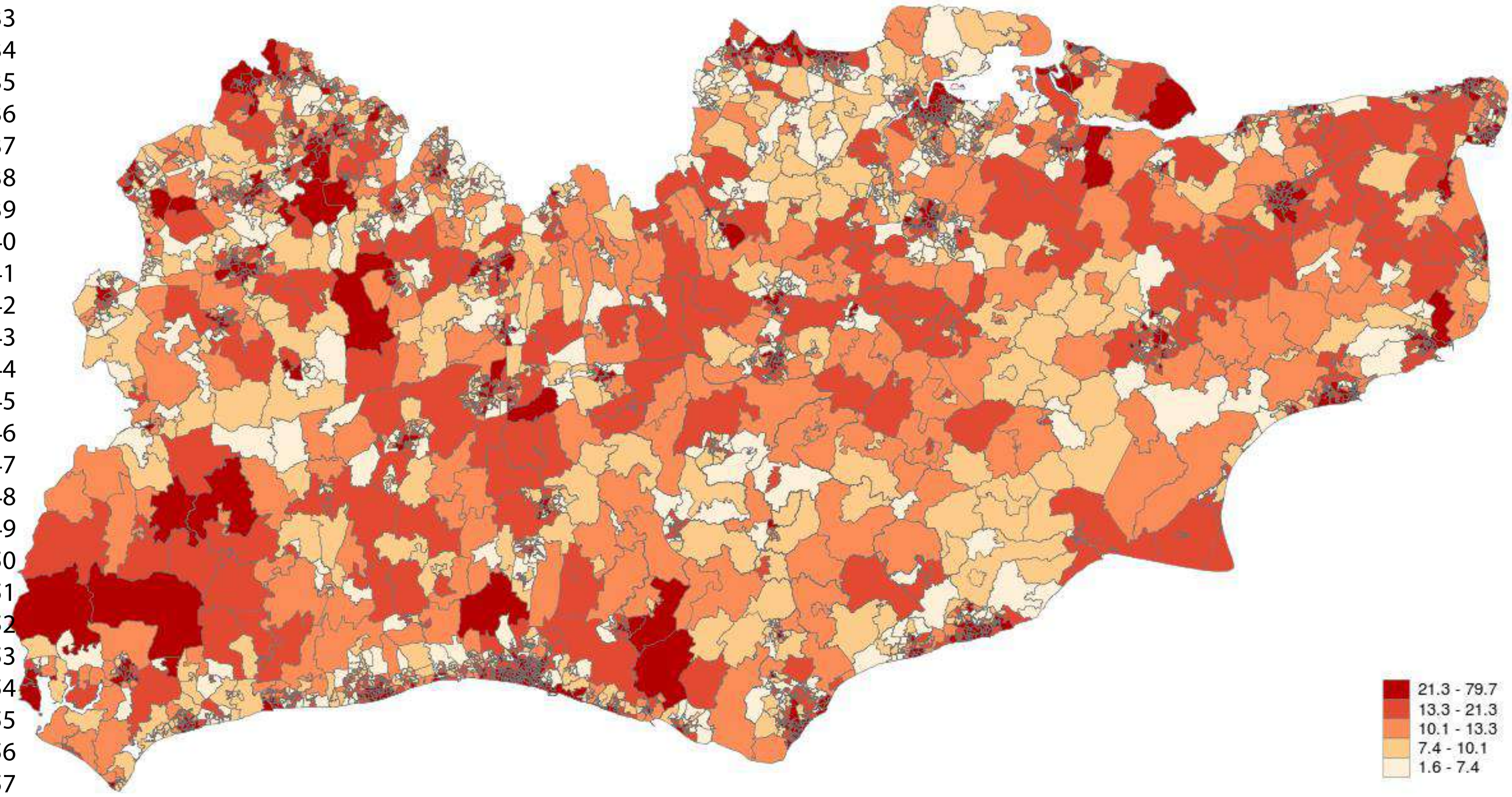

South Central, Private Renting % of all households

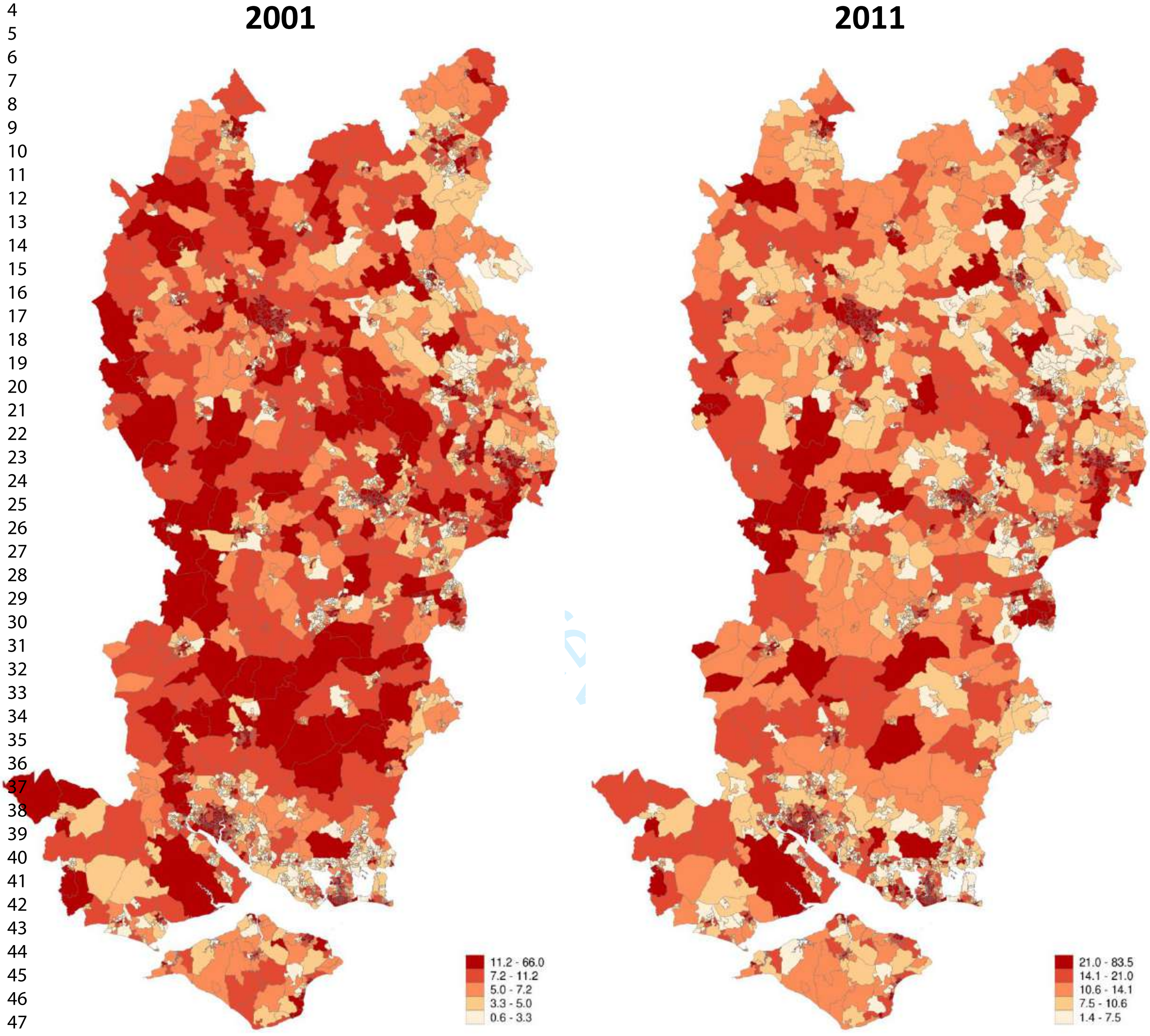

South West, Private Renting % of all households

2001

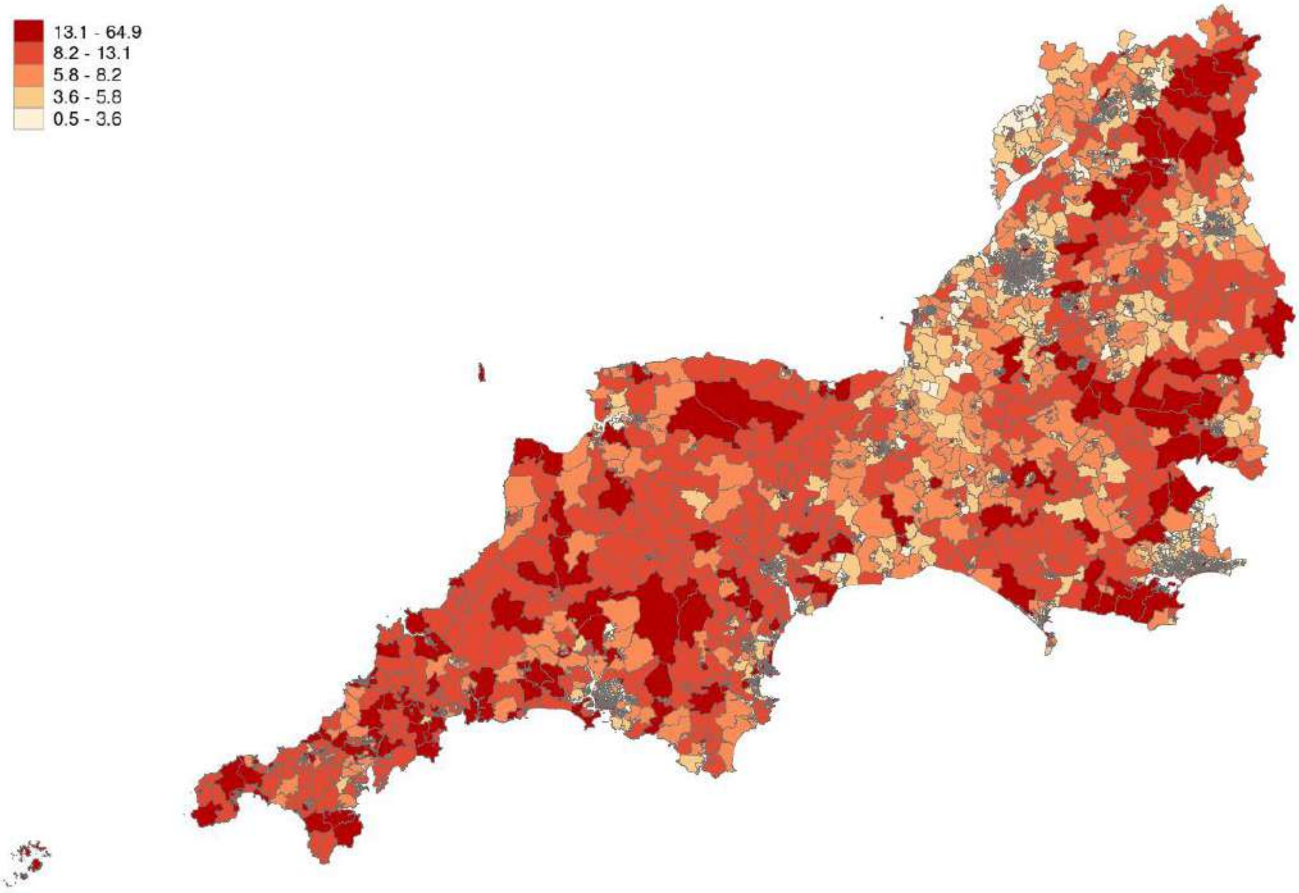

2011

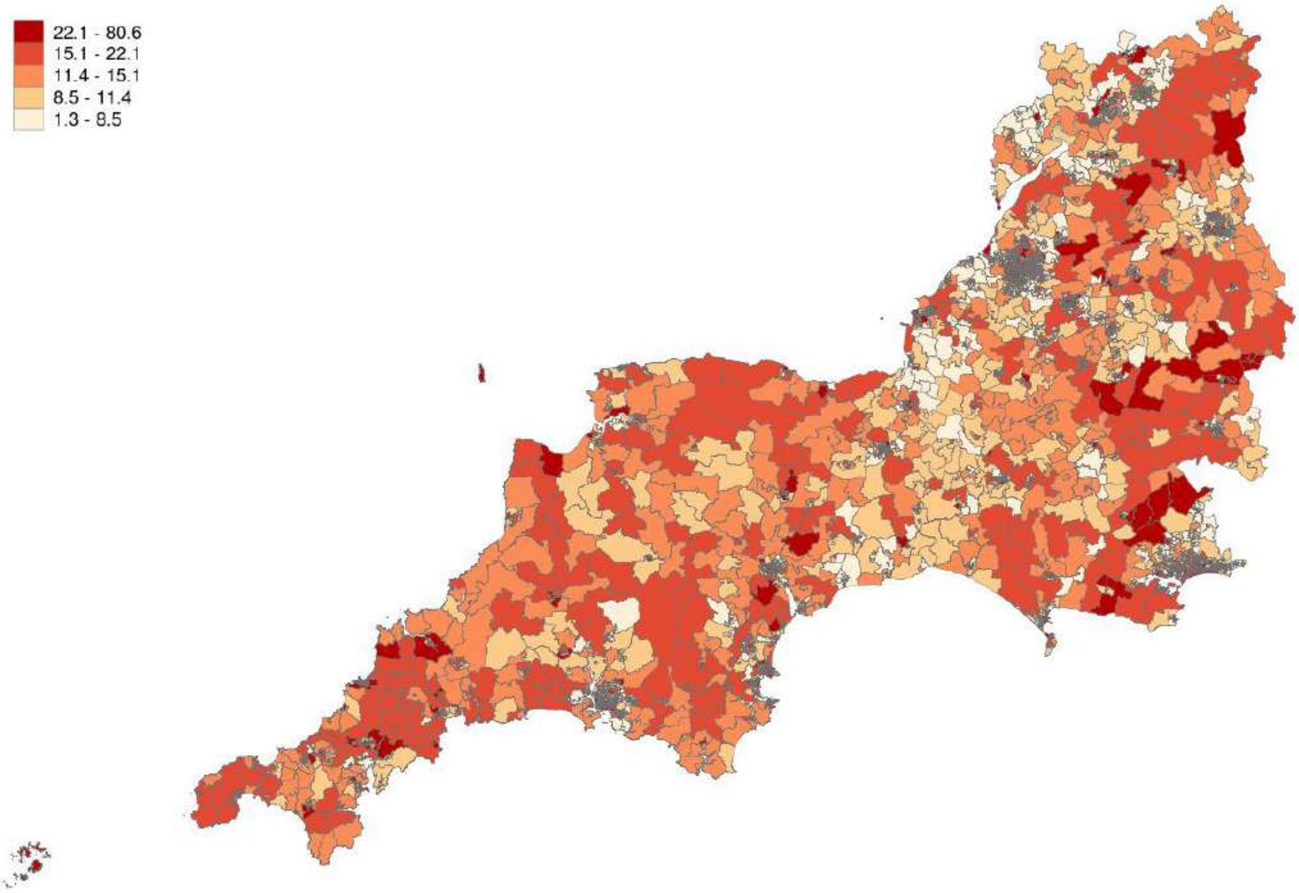

Population Turnover

England 2001, Population Turnover % of population

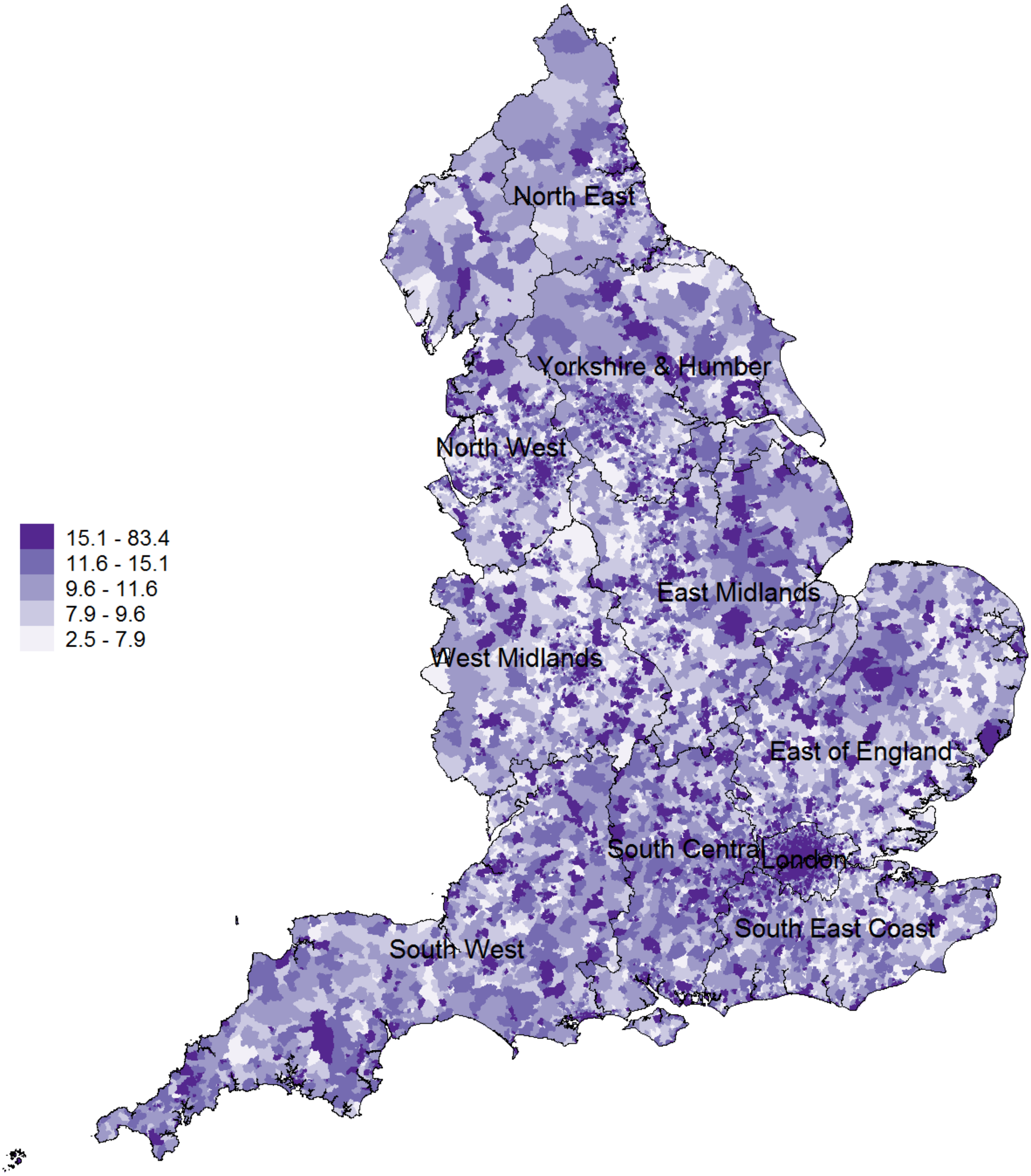

England 2011, Population Turnover % of population

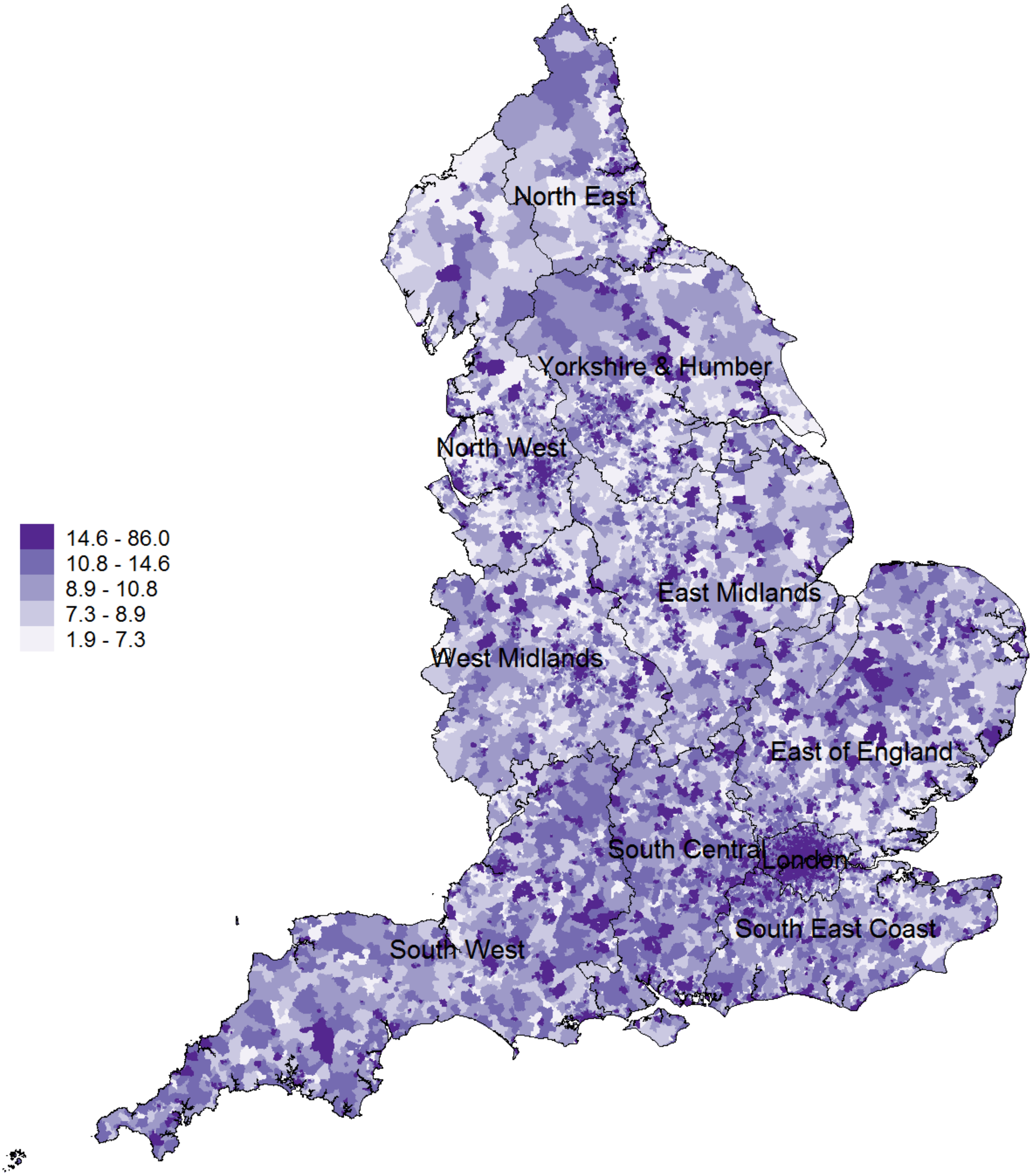

North East, Population Turnover % of population

2001

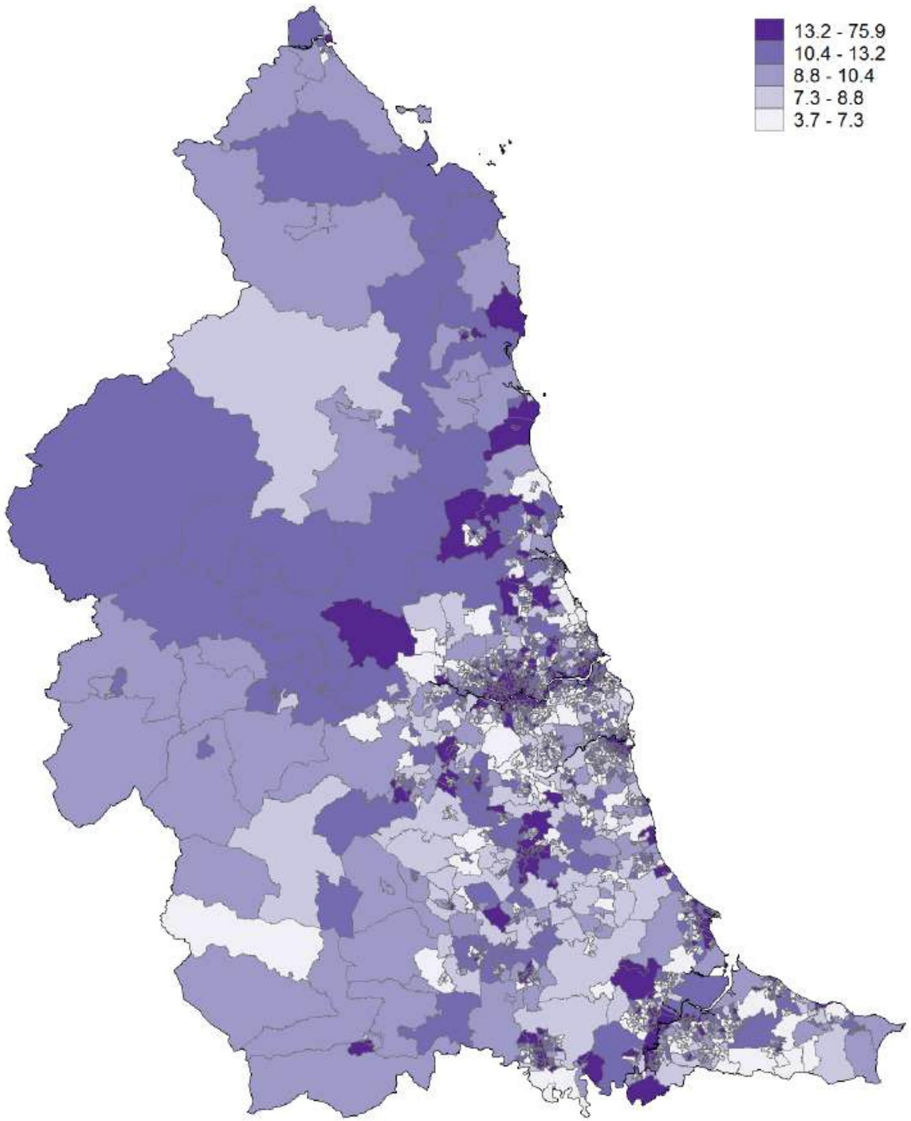

2011

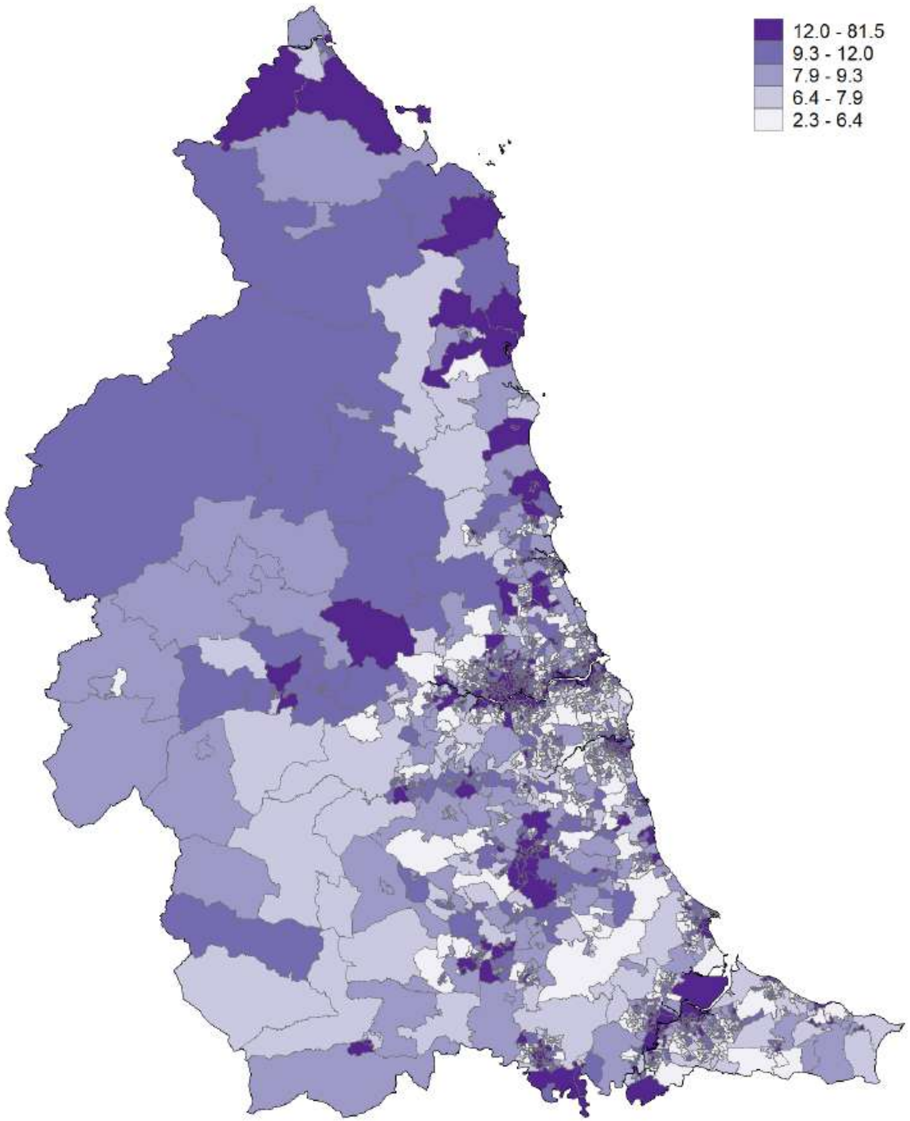

North West, Population Turnover % of population

2001 **2011**

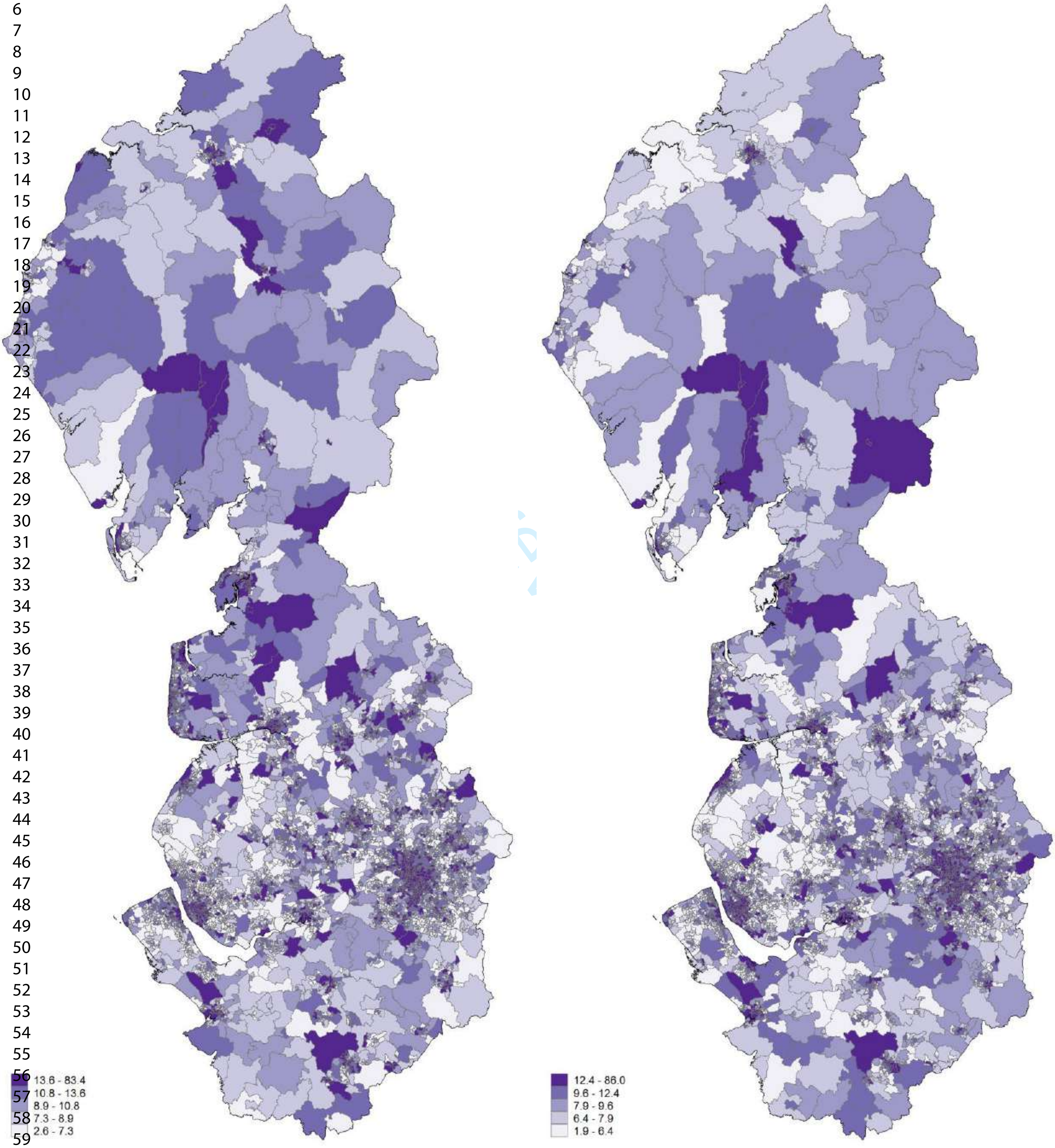

Yorkshire and the Humber, Population Turnover % of population

2001

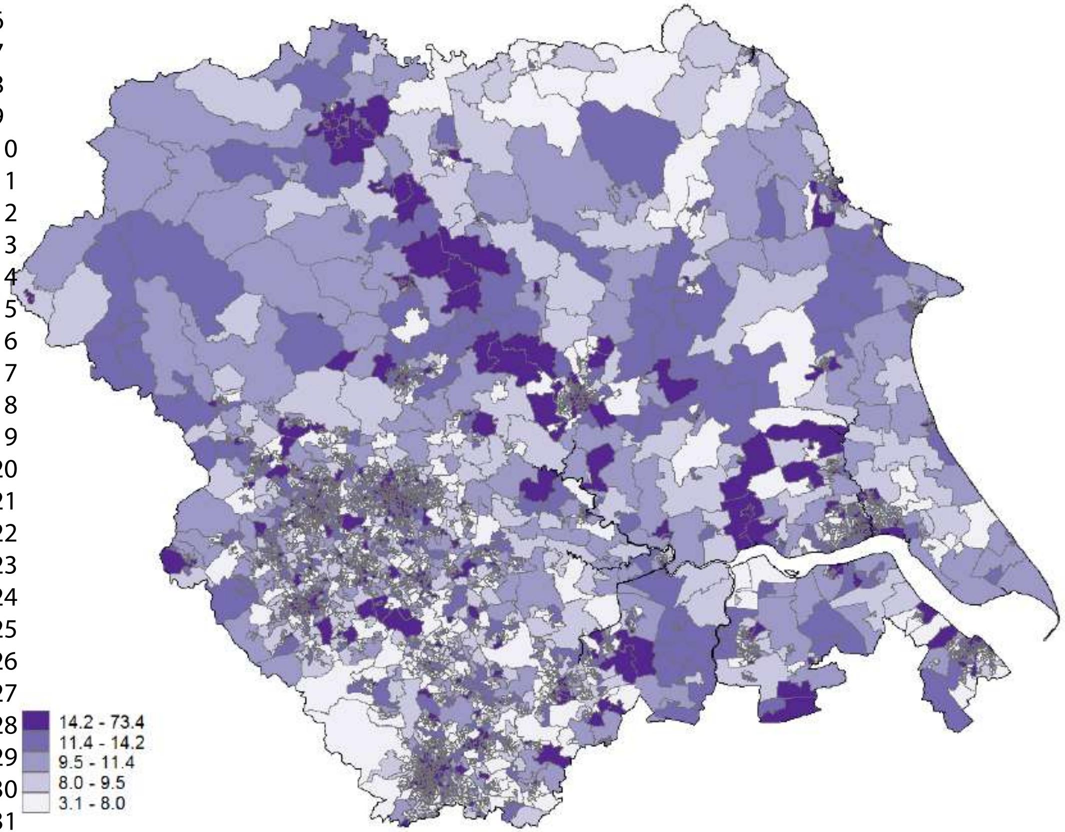

2011

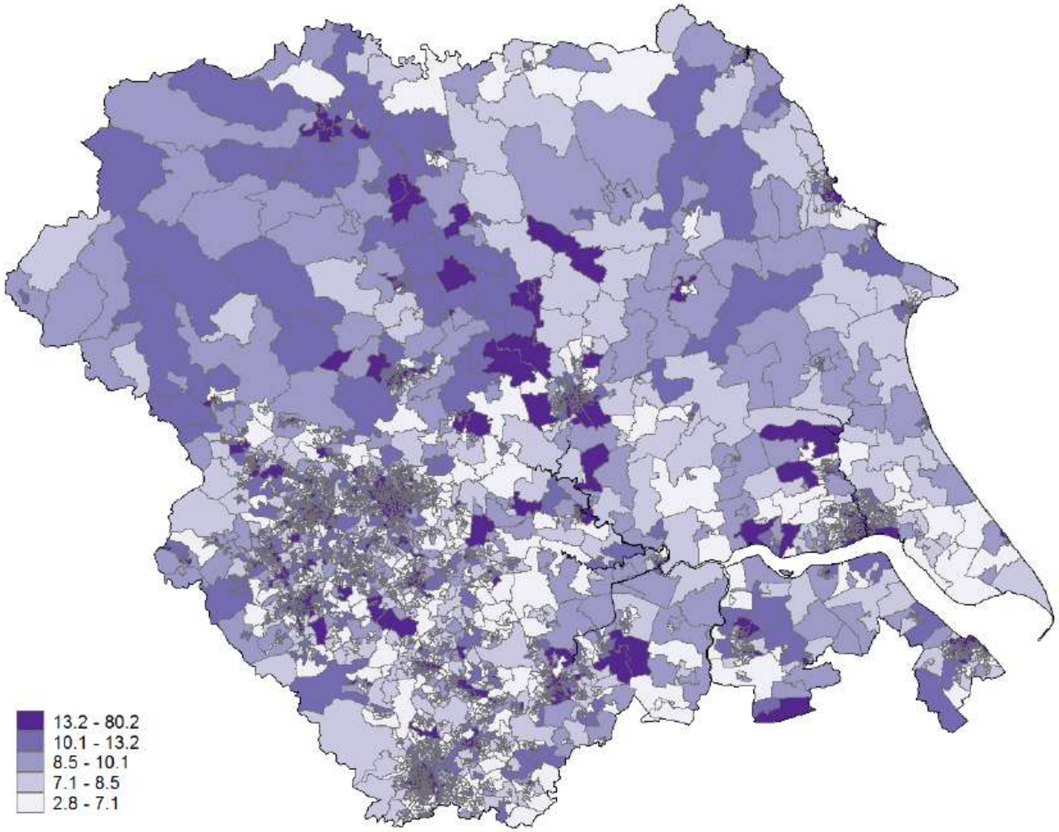

East Midlands, Population Turnover % of population

2001

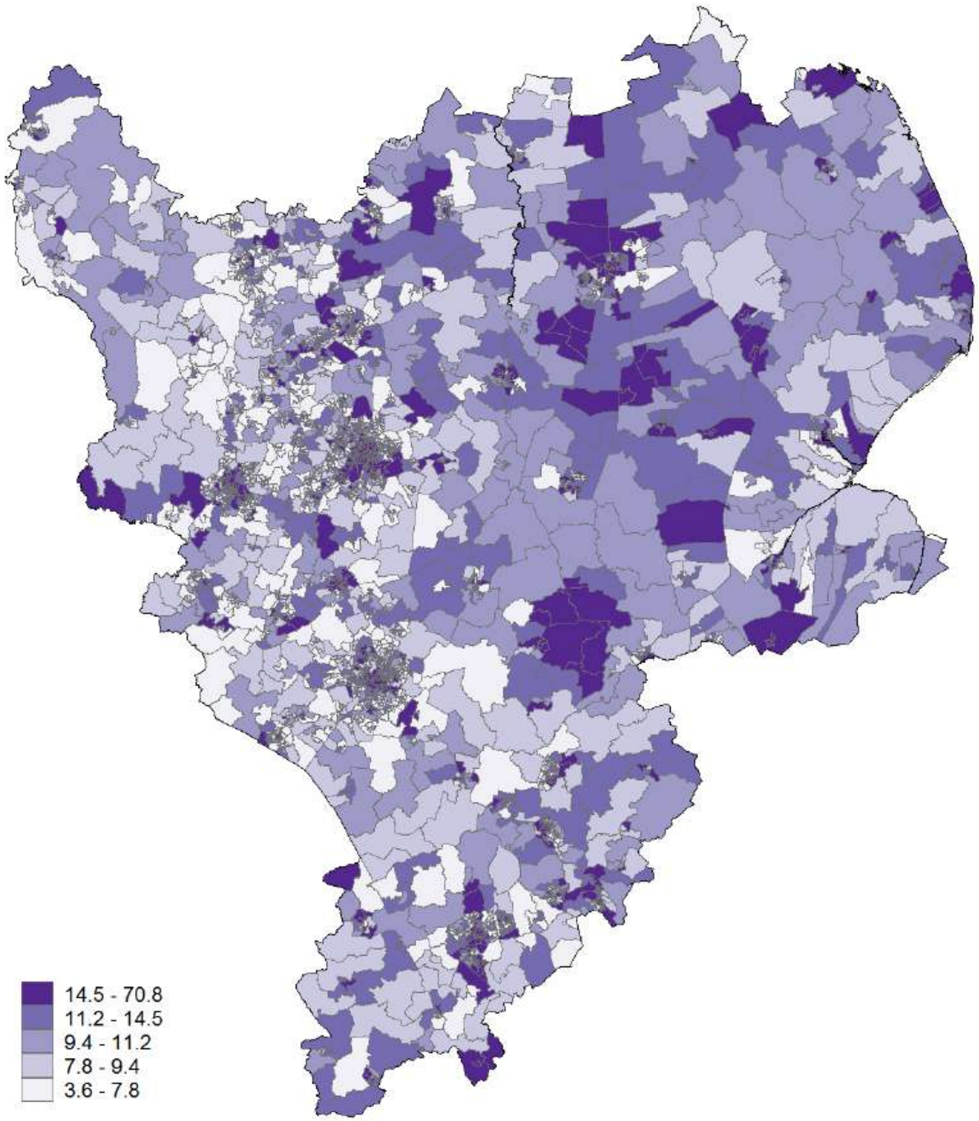

2011

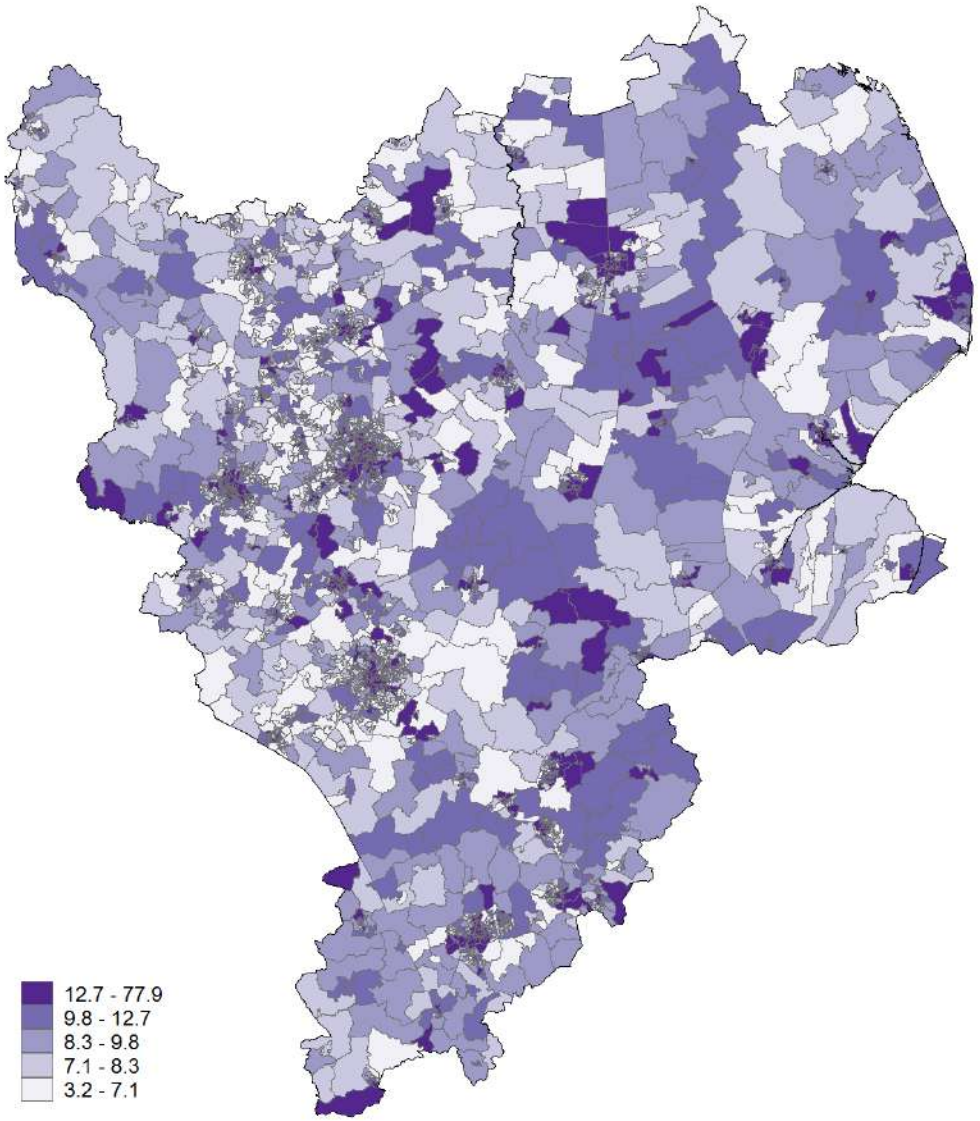

West Midlands, Population Turnover % of population

2001 | 2011

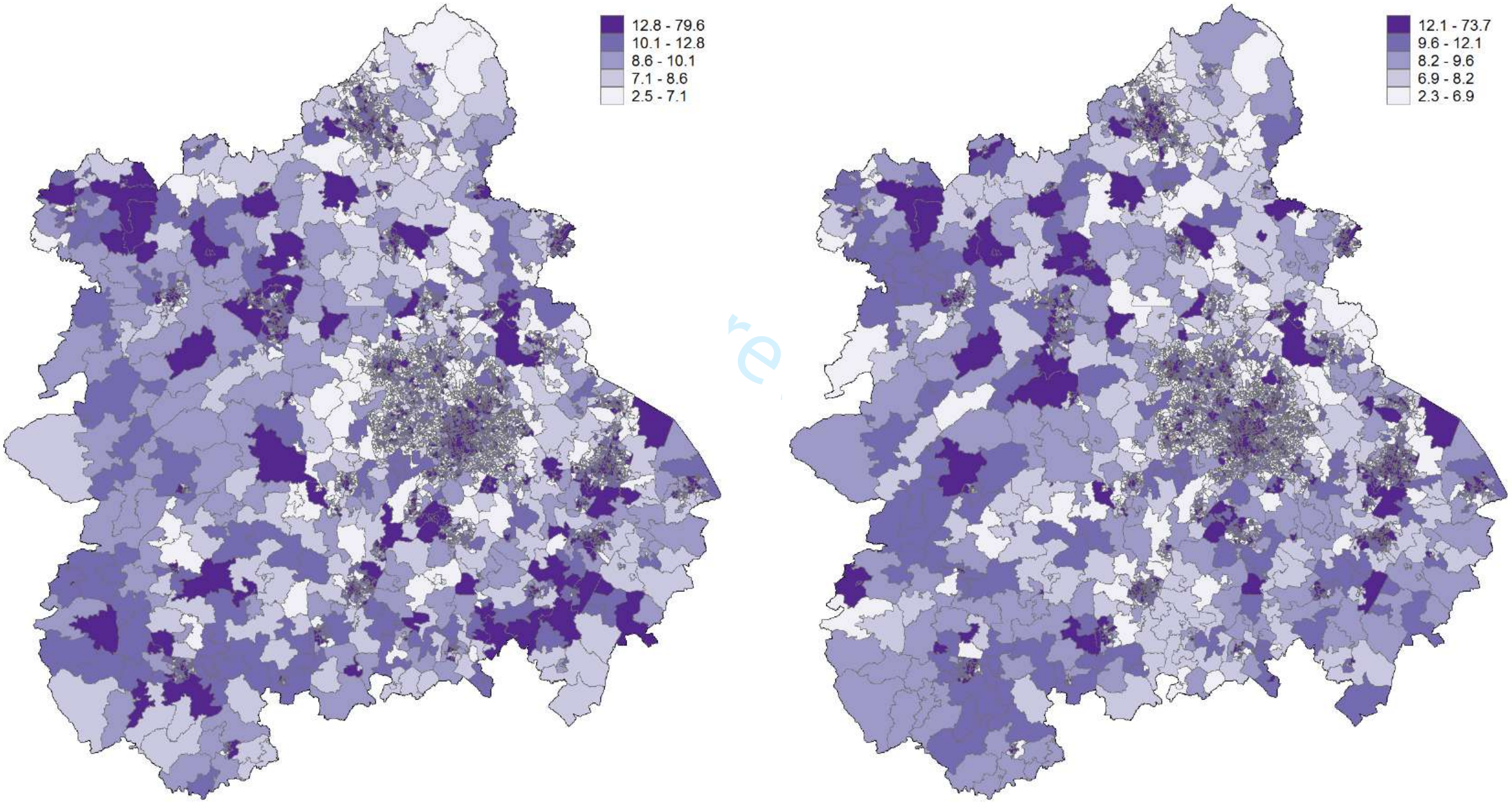

East of England, Population Turnover % of population

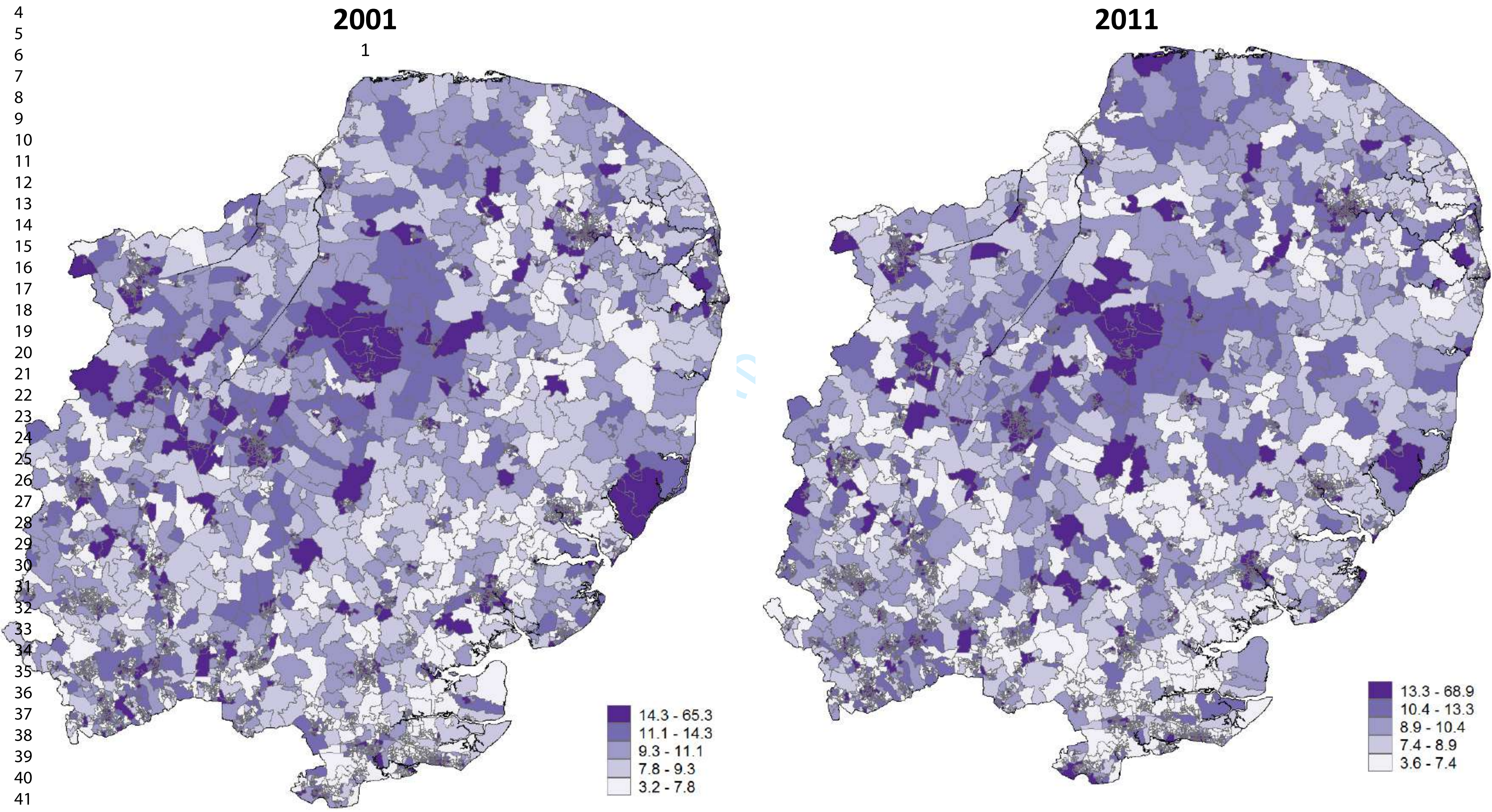

London, Population Turnover % of population

2001

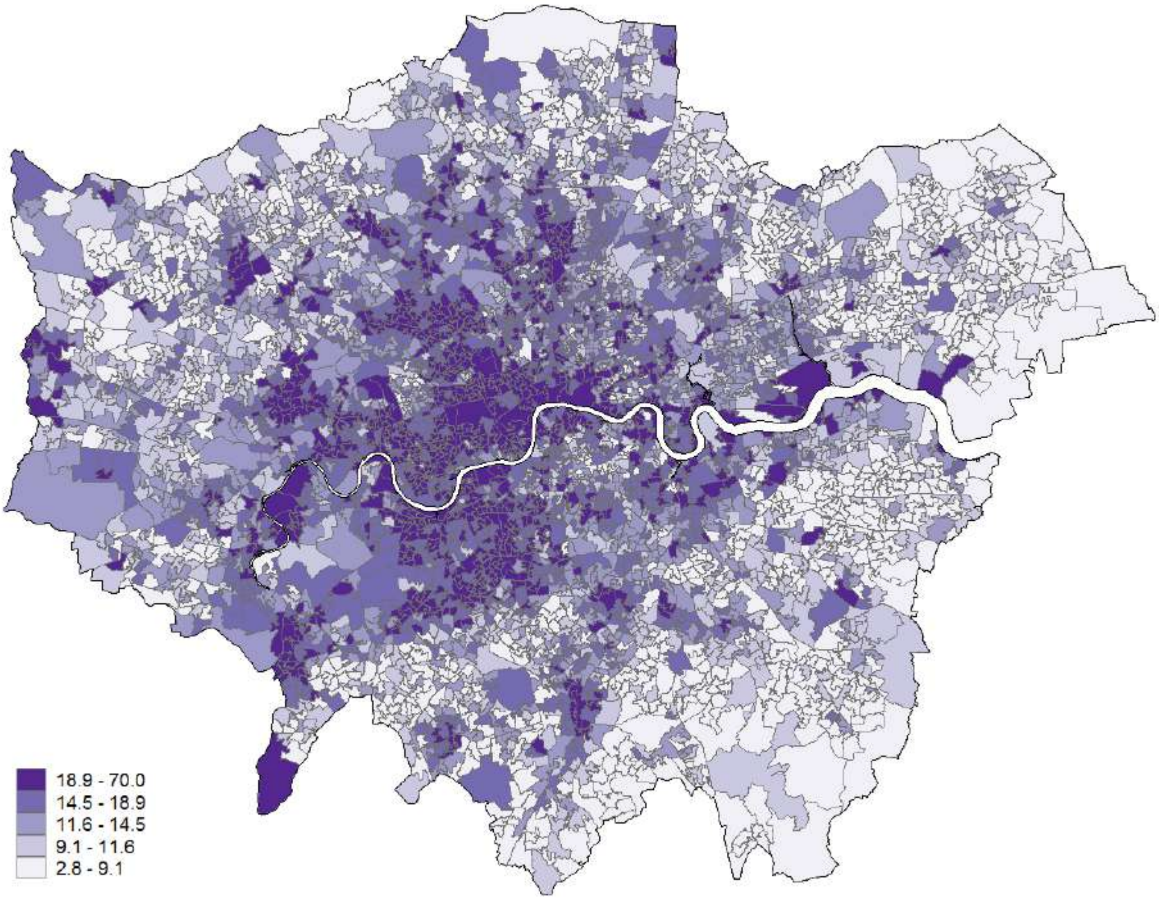

2011

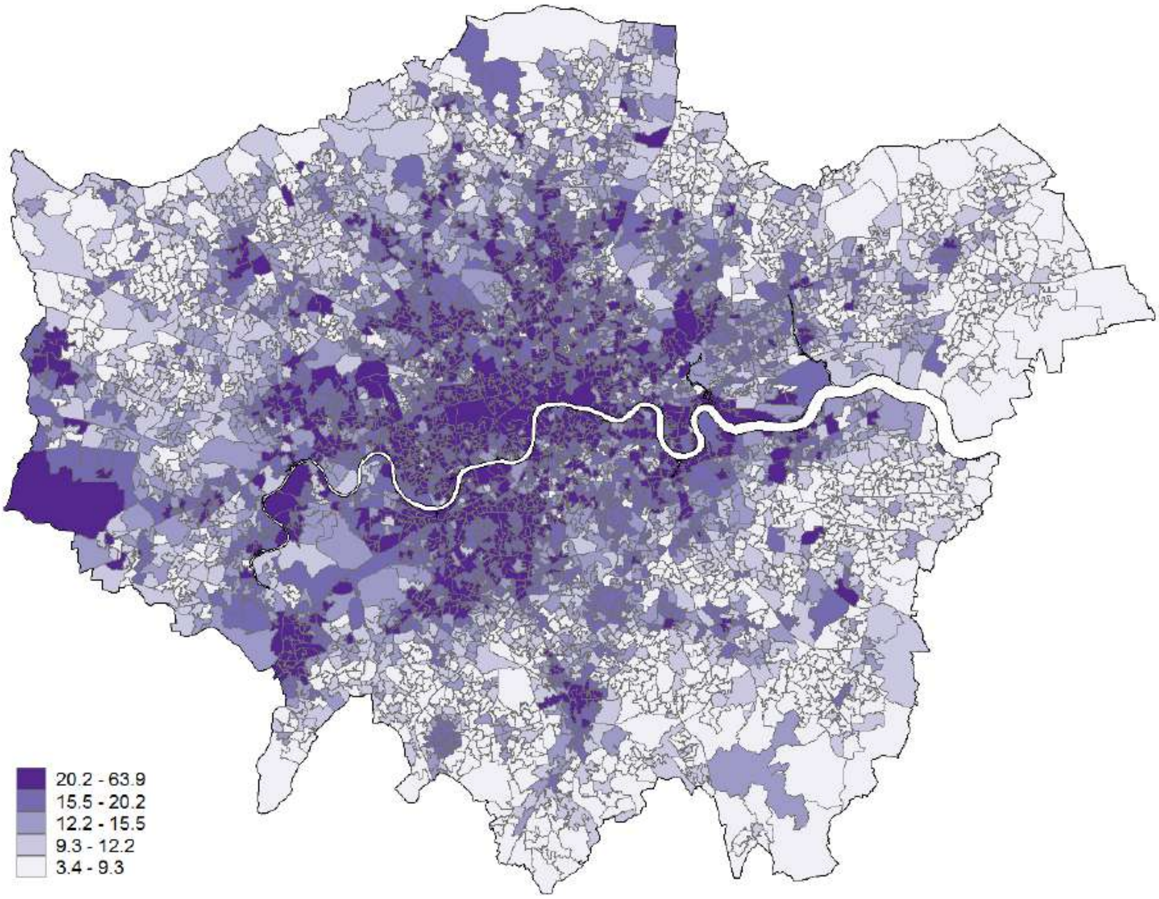

South East Coast, Population Turnover % of population

2001

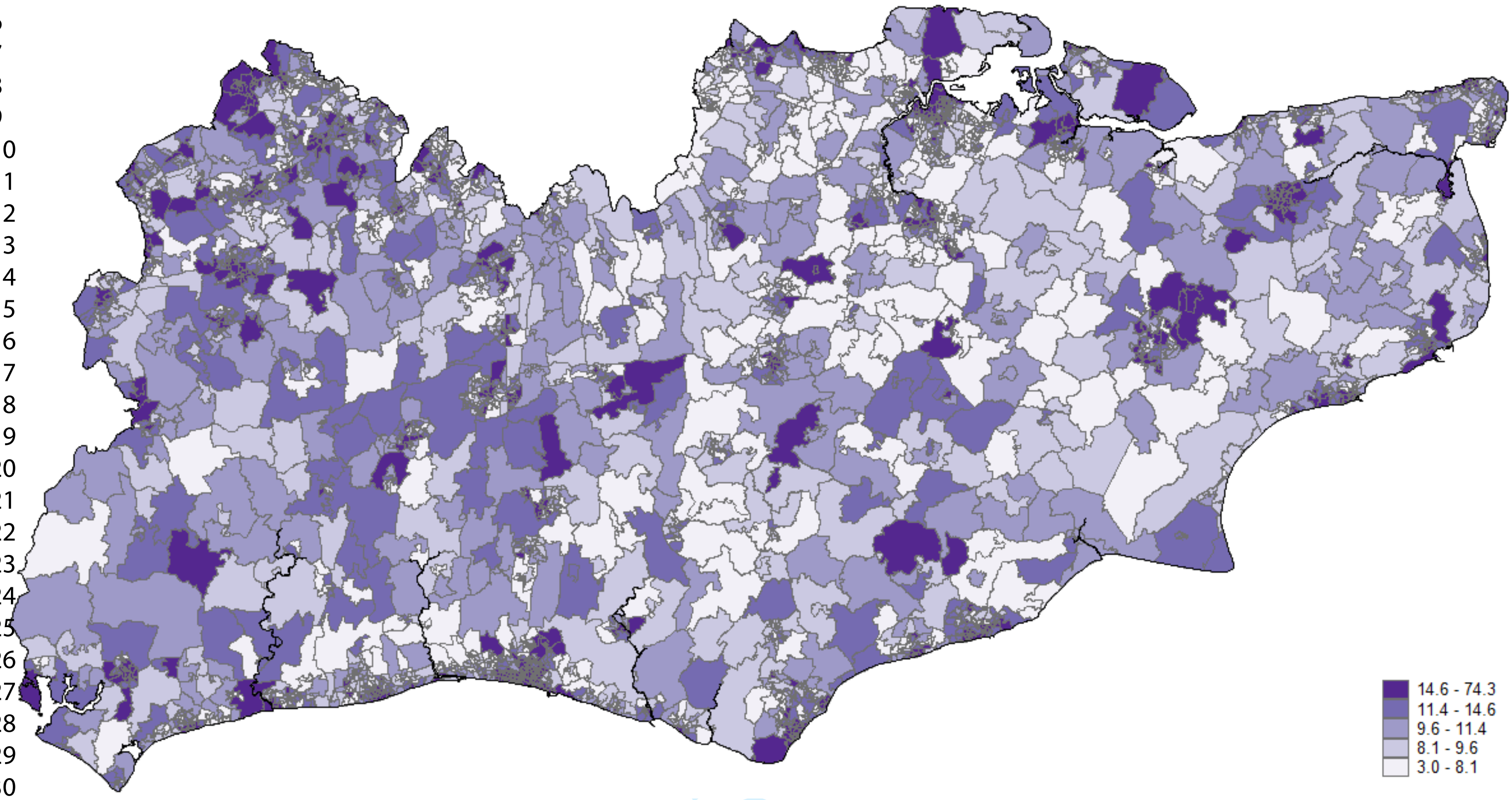

2011

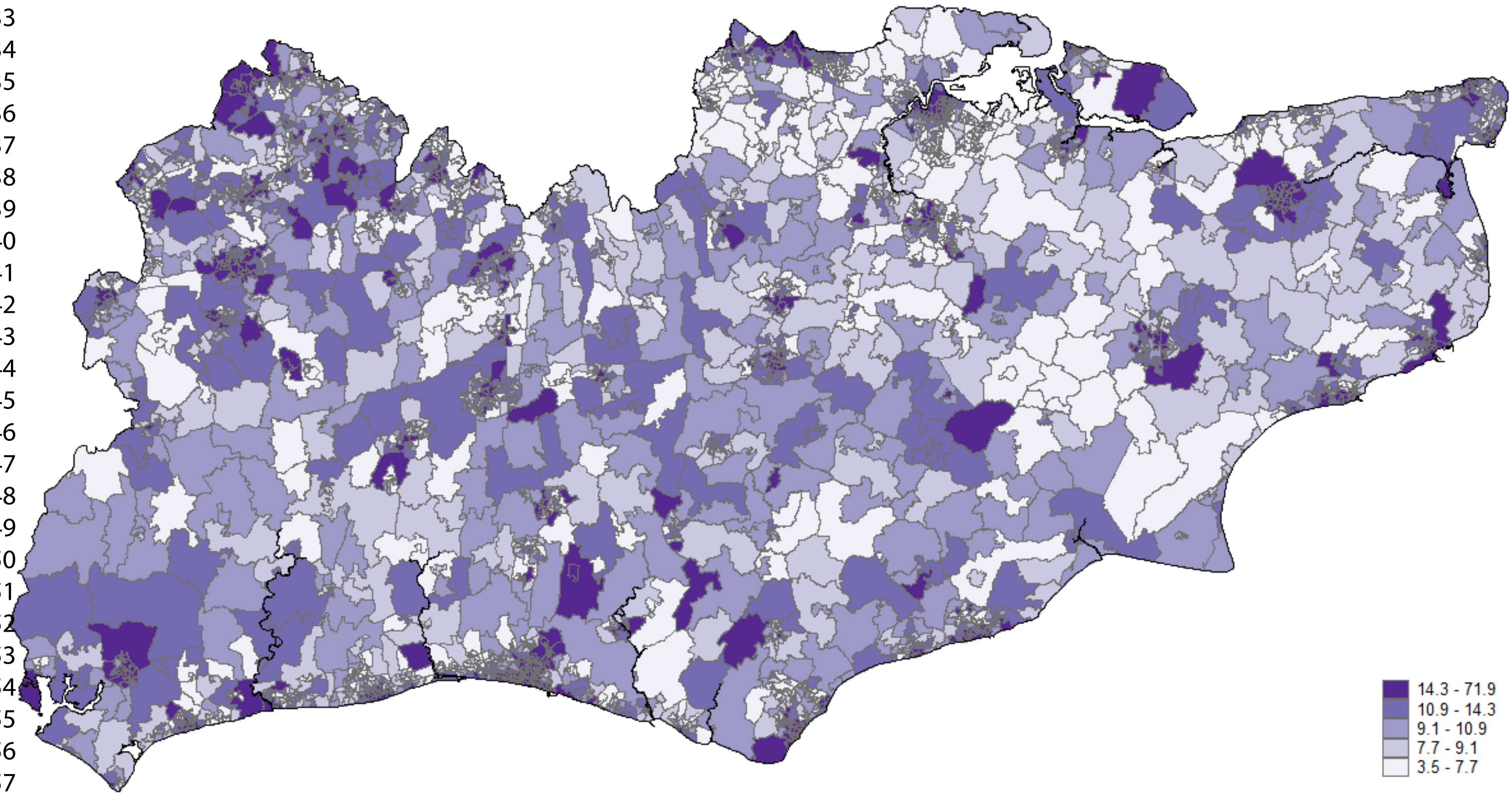

South Central, Population Turnover % of population

2001

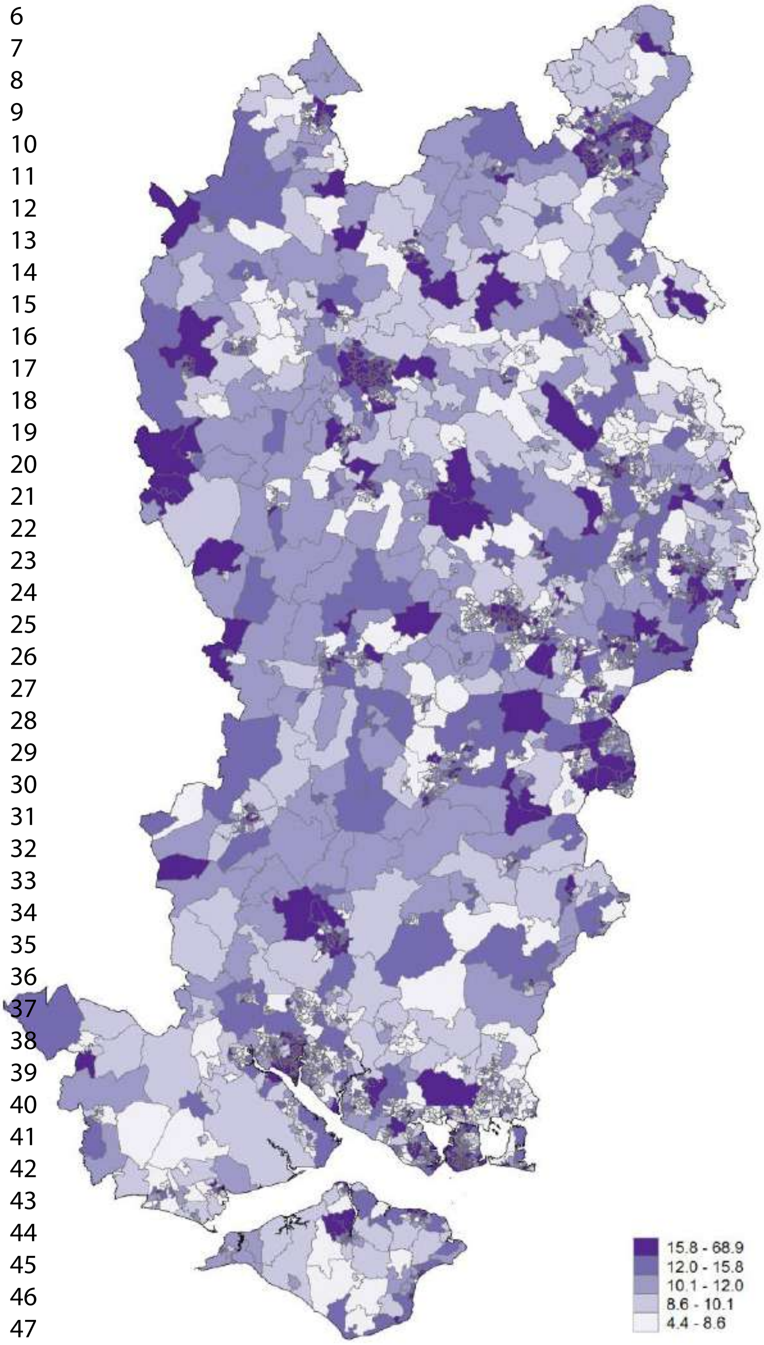

2011

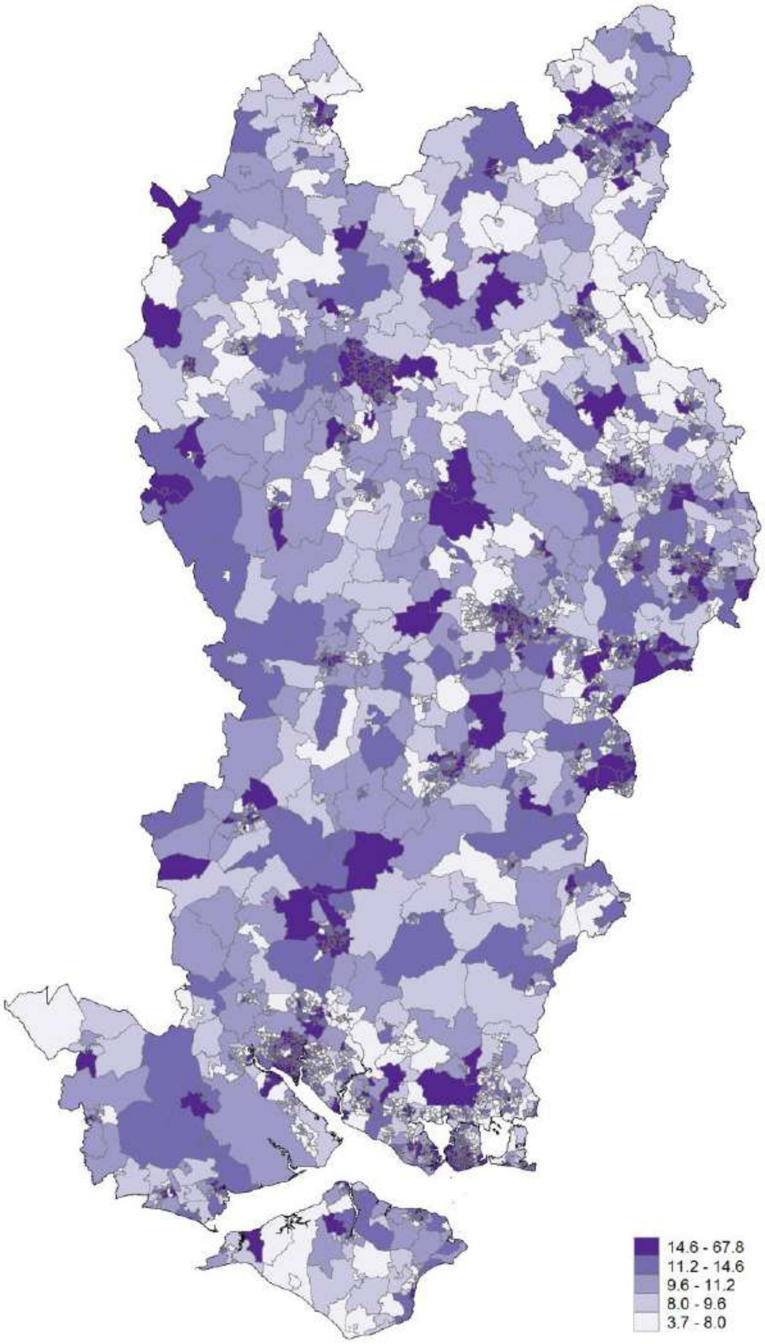

South West, Population Turnover % of population

2001

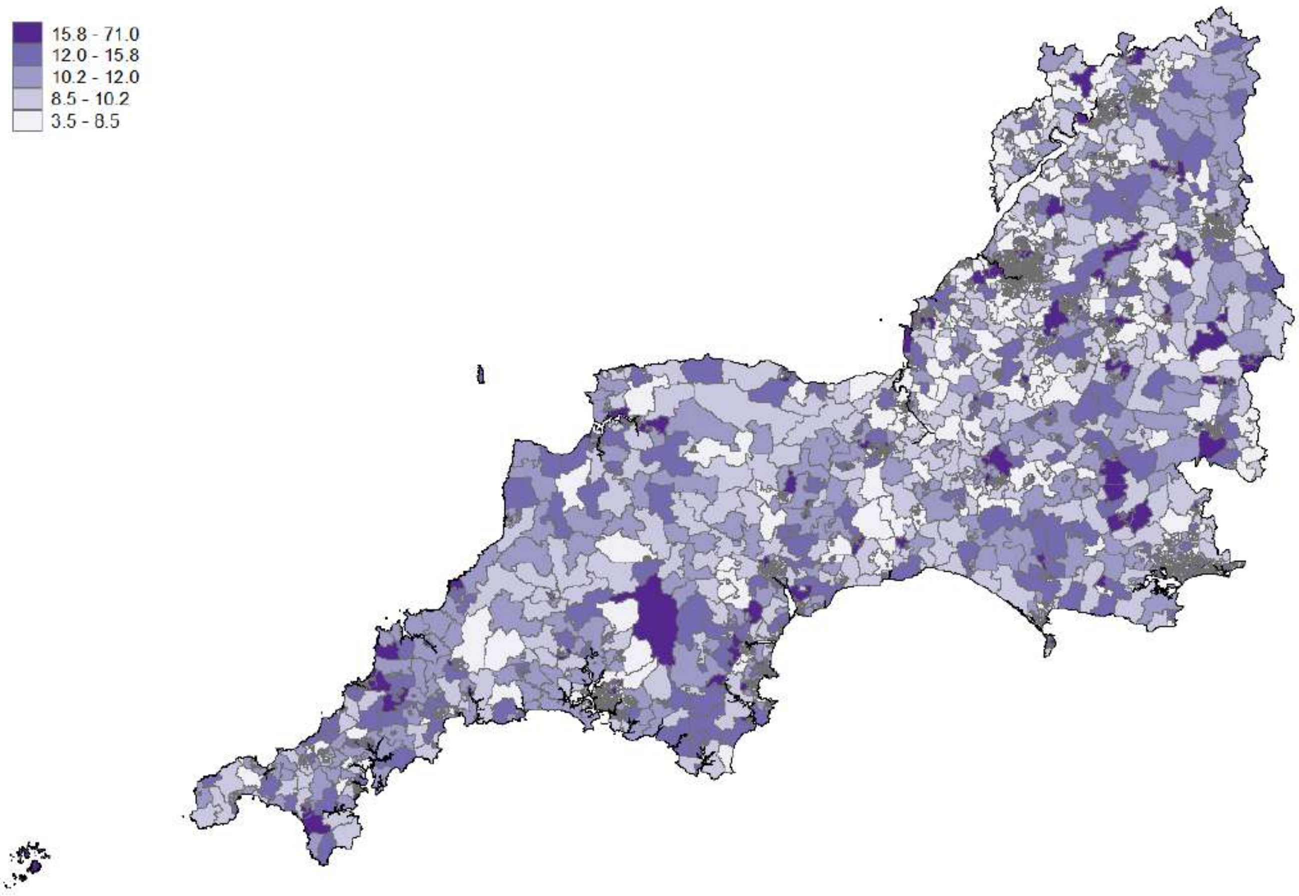

2011

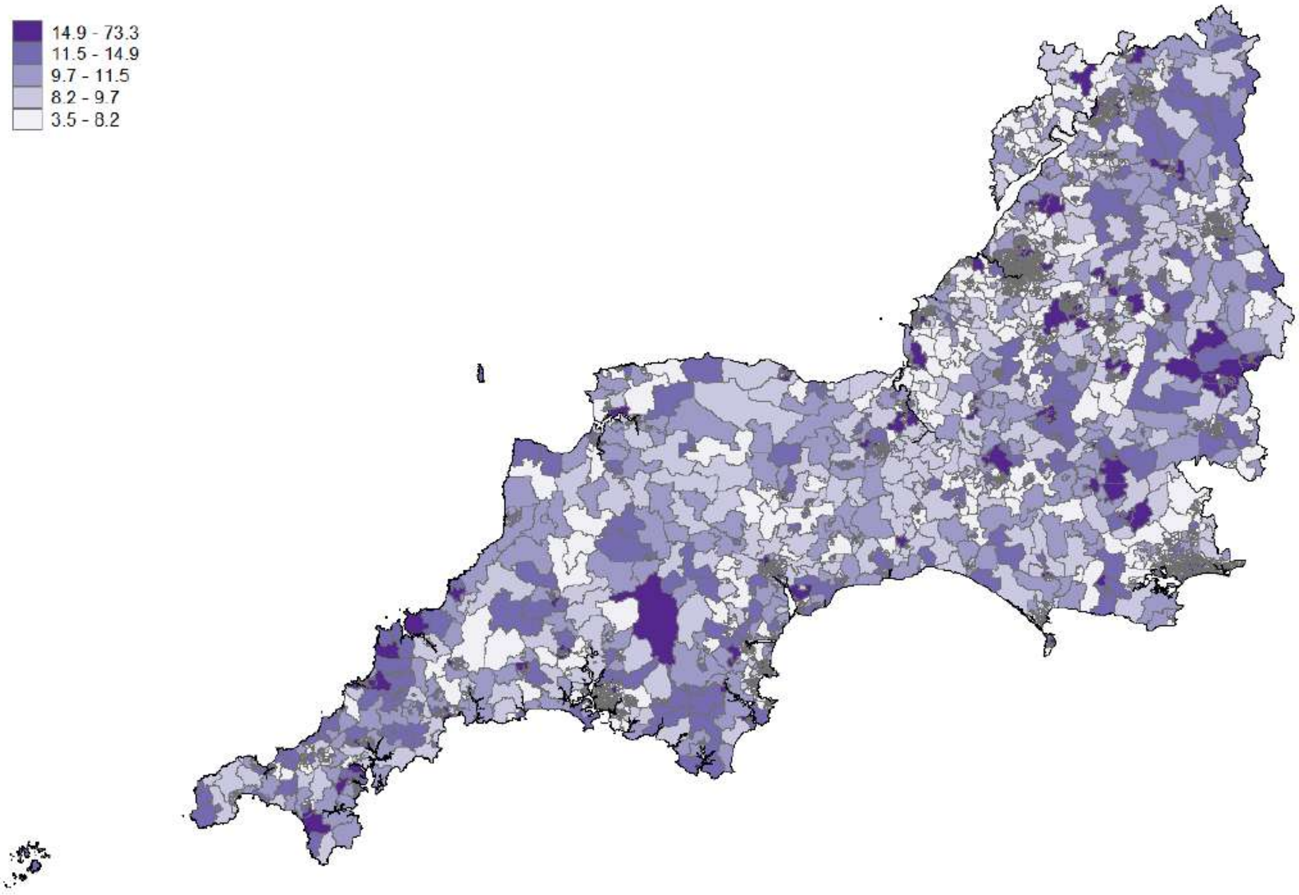

STROBE Statement—checklist of items that should be included in reports of observational studies

	Item No	Recommendation
**Title and abstract**	1	(*a*) Indicate the study's design with a commonly used term in the title or the abstract **p.1**
		(*b*) Provide in the abstract an informative and balanced summary of what was done and what was found **p.2**
**Introduction**		
Background/rationale	2	Explain the scientific background and rationale for the investigation being reported **p.3**
Objectives	3	State specific objectives, including any prespecified hypotheses **p.4**
**Methods**		
Study design	4	Present key elements of study design early in the paper
Setting	5	Describe the setting, locations, and relevant dates, including periods of recruitment, exposure, follow-up, and data collection **p.4**
Participants	6	(*a*) *Cohort study*—Give the eligibility criteria, and the sources and methods of selection of participants. Describe methods of follow-up **p.5** *Case-control study*—Give the eligibility criteria, and the sources and methods of case ascertainment and control selection. Give the rationale for the choice of cases and controls **NA** *Cross-sectional study*—Give the eligibility criteria, and the sources and methods of selection of participants **NA**
		(*b*) *Cohort study*—For matched studies, give matching criteria and number of exposed and unexposed **NA** *Case-control study*—For matched studies, give matching criteria and the number of controls per case **NA**
Variables	7	Clearly define all outcomes, exposures, predictors, potential confounders, and effect modifiers. Give diagnostic criteria, if applicable **p.4**
Data sources/ measurement	8*	For each variable of interest, give sources of data and details of methods of assessment (measurement). Describe comparability of assessment methods if there is more than one group **p.4**
Bias	9	Describe any efforts to address potential sources of bias **NA**
Study size	10	Explain how the study size was arrived at **p.5**
Quantitative variables	11	Explain how quantitative variables were handled in the analyses. If applicable, describe which groupings were chosen and why **p.5**
Statistical methods	12	(*a*) Describe all statistical methods, including those used to control for confounding **p.5**
		(*b*) Describe any methods used to examine subgroups and interactions **NA**
		(*c*) Explain how missing data were addressed **NA**
		(*d*) *Cohort study*—If applicable, explain how loss to follow-up was addressed **NA** *Case-control study*—If applicable, explain how matching of cases and controls was addressed **NA** *Cross-sectional study*—If applicable, describe analytical methods taking account of sampling strategy **NA**
		(*e*) Describe any sensitivity analyses **NA**

Continued on next page

For peer review only

**Results**		
Participants	13*	(a) Report numbers of individuals at each stage of study—eg numbers potentially eligible, examined for eligibility, confirmed eligible, included in the study, completing follow-up, and analysed **NA**
		(b) Give reasons for non-participation at each stage **NA**
		(c) Consider use of a flow diagram **NA**
Descriptive data	14*	(a) Give characteristics of study participants (eg demographic, clinical, social) and information on exposures and potential confounders p.5
		(b) Indicate number of participants with missing data for each variable of interest **NA**
		(c) *Cohort study*—Summarise follow-up time (eg, average and total amount) **p.5**
Outcome data	15*	*Cohort study*—Report numbers of outcome events or summary measures over time **p.5**
		Case-control study—Report numbers in each exposure category, or summary measures of exposure **NA**
		Cross-sectional study—Report numbers of outcome events or summary measures **NA**
Main results	16	(*a*) Give unadjusted estimates and, if applicable, confounder-adjusted estimates and their precision (eg, 95% confidence interval). Make clear which confounders were adjusted for and why they were included **NA**
		(*b*) Report category boundaries when continuous variables were categorized **NA**
		(*c*) If relevant, consider translating estimates of relative risk into absolute risk for a meaningful time period **NA**
Other analyses	17	Report other analyses done—eg analyses of subgroups and interactions, and sensitivity analyses **NA**
**Discussion**		
Key results	18	Summarise key results with reference to study objectives **p.5-p.6**
Limitations	19	Discuss limitations of the study, taking into account sources of potential bias or imprecision. Discuss both direction and magnitude of any potential bias **p.7**
Interpretation	20	Give a cautious overall interpretation of results considering objectives, limitations, multiplicity of analyses, results from similar studies, and other relevant evidence **p.8**
Generalisability	21	Discuss the generalisability (external validity) of the study results **p.9**
**Other information**		
Funding	22	Give the source of funding and the role of the funders for the present study and, if applicable, for the original study on which the present article is based **p.11**

*Give information separately for cases and controls in case-control studies and, if applicable, for exposed and unexposed groups in cohort and cross-sectional studies.

Note: An Explanation and Elaboration article discusses each checklist item and gives methodological background and published examples of transparent reporting. The STROBE checklist is best used in conjunction with this article (freely available on the Web sites of PLoS Medicine at http://www.plosmedicine.org/, Annals of Internal Medicine at http://www.annals.org/, and Epidemiology at http://www.epidem.com/). Information on the STROBE Initiative is available at www.strobe-statement.org.

For peer review only

Spatial distribution and temporal trends in social fragmentation in England, 2001 to 2011: a national study

Journal:	*BMJ Open*
Manuscript ID	bmjopen-2018-025881.R2
Article Type:	Research
Date Submitted by the Author:	09-Nov-2018
Complete List of Authors:	Grigoroglou, Christos; University of Manchester, Centre for Primary Care<br>Munford, Luke; University of Manchester, Manchester Centre for Health Economics<br>Webb, Roger; University of Manchester, Centre for Suicide Prevention; Centre for Mental Health and Risk<br>Kapur, Navneet; University of Manchester, Centre for Suicide Prevention, Centre for Mental Health and Risk<br>Ashcroft, Darren; University of Manchester, Centre for Pharmacoepidemiology and Drug Safety<br>Kontopantelis, Evangelos; University of Manchester, Division of Population Health, Health Services Research and Primary Care
**Primary Subject Heading**:	Epidemiology
Secondary Subject Heading:	Mental health, Health services research
Keywords:	MENTAL HEALTH, EPIDEMIOLOGY, Organisation of health services < HEALTH SERVICES ADMINISTRATION & MANAGEMENT, SOCIAL MEDICINE

SCHOLARONE™
Manuscripts

Spatial distribution and temporal trends in social fragmentation in England, 2001 to 2011: a national study

Christos Grigoroglou[1,8], Luke Munford[2,8], Roger T. Webb[3,6,8], Nav Kapur[4,6,7,8], Darren M. Ashcroft [5,6,8], Evangelos Kontopantelis[1,8]

[1] NIHR School for Primary Care Research, Centre for Primary Care, Division of Population Health, Health Services Research and Primary Care, University of Manchester, Manchester, UK
[2] Centre for Health Economics, Division of Population Health, Health Services Research and Primary Care, University of Manchester, Manchester, UK
[3] Centre for Mental Health and Safety, Division of Psychology & Mental Health, University of Manchester, Manchester, UK
[4] Centre for Suicide Prevention, Division of Psychology and Mental Health, University of Manchester, Manchester, UK
[5] Centre for Pharmacoepidemiology and Drug Safety, School of Health Sciences, Faculty of Biology, Medicine and Health, , University of Manchester, Manchester Academic Health Sciences Centre (MAHSC), Manchester, UK
[6] NIHR Greater Manchester Patient Safety Translational Research Centre, Manchester Academic Health Sciences Centre (MAHSC), Manchester, UK
[7] Greater Manchester Mental Health Trust, Manchester, UK
[8] Manchester Academic Health Sciences Centre [MAHSC], Manchester, UK

Correspondence to:

Christos Grigoroglou
E-mail: christos.grigoroglou@postgrad.manchester.ac.uk
Telephone number: 0161-2750743
5th Floor, Williamson Building, University of Manchester,
Manchester, UK, M13 9PL

Word count: 3,370
5 Figures

For peer review only

For peer review only - http://bmjopen.bmj.com/site/about/guidelines.xhtml

Objective: Social fragmentation is commonly examined in epidemiological studies of mental illness as high levels of social fragmentation are often found in areas with high prevalence of mental illness. In this study we examine spatial and temporal patterns of the index of social fragmentation and its underlying indicators in England over time.

Setting: Data for social fragmentation and its underlying indicators were analysed over the decennial Censuses (2001–2011) at a small-area geographical level (mean of 1500 people). Degrees of social fragmentation and temporal changes were spatially visualised for the whole of England and its 10 administrative regions. Spatial clustering was quantified using Moran's I; changes in correlations over time were quantified using Spearman's ranking correlation.

Results: Between 2001 and 2011 we observed a strong persistence for social fragmentation nationally (Spearman's r=0.94). At the regional level, modest changes were observed over time, but marked increases were observed for two of the four social fragmentation underlying indicators, namely single people and those in private renting. Results supported our hypothesis of increasing spatial clustering over time. Moderate regional variability was observed in social fragmentation, its underlying indicators and their clustering over time.

Conclusion: Patterns of social fragmentation and its underlying indicators persisted in England which seem to be driven by the large increases in single people and those in private renting. Policies to improve social cohesion may have an impact the lives of persons who experience mental illness. The spatial aspect of social fragmentation can inform the targeting of health and social care interventions, particularly in areas with strong social fragmentation clustering.

Keywords: social fragmentation, mental health, clustering, inequalities, spatial autocorrelation

For peer review only

Strengths and Limitations of the study

- This study utilises data over two time points for the whole of England and is the first to describe spatial and temporal patterns of social fragmentation.
- Spatial aspects of social fragmentation can inform organisation of mental health services and social interventions that aim to enhance social support, particularly in areas where social fragmentation is high and is spatially clustered
- Over the last two decennial Censuses (2001 and 2011), the index of social fragmentation in England strongly persisted at a small-area geographical level while its clustering and its underlying indicators increased over time.
- Over time social fragmentation remained relatively stable, but we found marked increases in the percentage of single people and the percentage of those living in private rented accommodation.
- Even though census data are considered of high quality, people's perception of social fragmentation may not be fully captured in the census while demographic factors may capture social fragmentation in some areas but not in others.

For peer review only

INTRODUCTION

The impact of local area characteristics, such as material deprivation, on health is well established. [1 2] However, similar to socioeconomic position, the social networks and the amount of social support in a neighbourhood may also partially determine an individual's health. [3-6] In this context, an area-level characteristic that has been the focus of much investigation in neighbourhood health research is social fragmentation. The index of social fragmentation measures lack of social cohesion, and it is used to define areas with a breakdown or absence of social capital, and was originally created to measure the non-economic deprivation aspects of areas. [7] Social fragmentation is usually measured using census data which offer a great potential for the design and implementation of mental health strategies when individual level data are not available.

Social fragmentation is distinct conceptually from deprivation, since fragmentation is a feature of household composition and demographic structure and is not intrinsically linked to socioeconomic position.[8] However, the two measures tend to be positively correlated as they are both concentrated in large towns and cities. [8 9] Thus, many urban localities are both deprived and socially fragmented, although the two phenomena do not always co-exist. Several studies suggests that fragmented communities provide less stable social institutions and social bonds, although maintaining stable institutions and bonds within the communities can contribute substantially to the creation of shared identities, persistence of social relations, promotion of healthy behaviours and good physical and mental health for the population. [10-14]

Social fragmentation has received less attention from researchers than deprivation, despite the fact that it can aid the understanding of mechanisms via which the wider social environment influences mental health. [15] Research on adults has shown that social fragmentation is more closely related to mental health than physical health outcomes, independent of material deprivation and individual-level risk factors. [7 16] In the context of mental health, social fragmentation has been linked with suicide and non-fatal self-harm, [7 17-19] mental disorders, [4 11 20] psychiatric health service use, [21] first admission for psychosis, [20-22] psychological distress, [3 11] and schizophrenia. [15 23] Nonetheless, physical illness outcomes do not appear to be as clearly linked to social fragmentation. [18 24 25]

In the UK, an index of social fragmentation was developed to measure neighbourhood-level conditions which affect lack of opportunities for social integration. [7] The index was originally based on indicators of population turnover, percentage of married people, percentage of the population in private renting and percentage of one person households using data from the UK Census. The rationale for the selection of the underlying variables used for the calculation of the index was that several studies, both at ecological and individual level, have found raised mental illness prevalence in highly urbanised areas with high population

For peer review only - http://bmjopen.bmj.com/site/about/guidelines.xhtml

turnover, many non-family households, and reduced social cohesion. [26] However, social trends towards increasing cohabitation [10 27] suggest the use of proportion of single, widowed and divorced people instead of married people in the calculation of the index.

Social fragmentation in a population is likely to have profound effects on its health and wellbeing, and yet it is incompletely examined and reported in the existing literature, and a number of important research questions remain unanswered. First, how strongly clustered is social fragmentation? Second, how strongly does social fragmentation cluster and change at regional level over two time points? Third, does social fragmentation persist over time? Answering all three questions is important to inform the organisation of healthcare services and to target social and healthcare interventions, especially for mental health for which social fragmentation appears to be a salient risk factor. In this study, we temporally and spatially describe the properties of social fragmentation in England and within each of the 10 administrative English regions and make comparisons across them. Finally, we quantify the persistence of social fragmentation over the study period.

METHODS

Data sources

To derive the index of social fragmentation we accessed decennial Census data from the Nomis website [28] on: (1) One person households, % of all Census households; (2) single people (including widowed, divorced and separated people), % all usual residents aged 16 or over; (3) Population turnover (percentage of the population that moved into the area from within the UK and from outside the UK), % Census population in the year preceding the census (4) Households in private renting, % of all Census households. Our analysis was conducted at a small-area geographical level, namely the Lower Layer Super Output Area (LSOA). LSOAs are geographical areas with a mean population of 1,500 people. All data were available at the LSOA level except for the population turnover data which were available at the output area level (OA). OAs provide the finest spatial scale at which the Census is enumerated. We assigned the OA level data to the LSOA level using lookup tables provided by the Office for National Statistics (ONS). More details on the data used to derive each social fragmentation underlying indicator are provided in the Supplementary appendix.

The social fragmentation index for each LSOA was calculated by adding the unweighted z scores for each of the four characteristics into a composite score in each census year pooled. We used two time points for the index of social fragmentation, i.e. 2001 and 2011 over the two decennial Censuses. Across LSOAs the scores for the index ranged from -6.42 to 27.57 for 2001 and from -7.09 to 25.79 for 2011, with the upper boundaries indicating the most socially fragmented areas and the lower boundaries the most socially cohesive

For peer review only - http://bmjopen.bmj.com/site/about/guidelines.xhtml

areas. Since the data used to derive the index in 2001 were reported using 2001 LSOAs, we used a weighted means algorithm to assign the data for each underlying indicator to 2011 LSOAs using mid-year population estimates that were obtained from ONS. [29] A detailed description on how we attributed 2001 LSOA data to 2011 LSOAs is provided in the supplementary material. We calculated the z-scores of all social fragmentation underlying indicators for each of the two time points of interest. Finally, we obtained data on LSOA rural/urban classification at the 2011 LSOA.[30]

Analyses

Our outcome of interest was the index of social fragmentation and its four underlying indicators measured separately at the 2001 and 2011 national Censuses. We visualised temporal changes in social fragmentation and its underlying indicators with the use of spatial maps for all of England and each of the 10 English regions/strategic health authorities (SHA) (North East, North West, Yorkshire & the Humber, East Midlands, West Midlands, East of England, London, South East Coast, South Central and South West), which were the highest level of organisation available in NHS England during the study period. To assess whether levels of spatial autocorrelation for social fragmentation changed over time we compared the values of Moran's I [31] in the two time points for England and each English region to allow within-England comparisons. The measure can identify the presence or absence of spatial clusters while accounting for the multi-dimensional and multi-directional nature of spatial autocorrelation. Under a random spatial pattern a higher than expected value of Moran's I for social fragmentation would indicate that areas with high levels of social fragmentation are clustered and also that LSOAs with high social fragmentation are bordered with LSOAs with similarly high levels of social fragmentation. We also investigated local indicators for spatial autocorrelation (LISA) [32] to identify local patterns of spatial associations. We also quantified the persistency of social fragmentation over the two time points by calculating Spearman's correlation between 2001 and 2011. To visualise and compare temporal changes in the mean social fragmentation levels across the 10 English regions, we plotted population-weighted box plots for the main outcome and its underlying indicators. Finally, we used population-weighted box plots to visualise and compare temporal changes in the social fragmentation levels between the 10 English regions. Analyses were performed with Stata v.14.1 and R v.3.3.1. Due to the size of the dataset, effectively the whole of England, statistical significance is irrelevant; observed associations of minimal strength would be statistically significant, and thus we focussed on effect sizes wherever possible.

RESULTS

For peer review only - http://bmjopen.bmj.com/site/about/guidelines.xhtml

Region-level characteristics of the index of social fragmentation at both time points are provided in Table 1. We found strong correlation for social fragmentation at the two time points (rho=0.93), using Spearman's ranking correlation to account for the relative nature of the measure. We plotted spatial maps of social fragmentation for the whole of England and for each region for the two time points analysed. We created maps of social fragmentation from a pooled sample in 2001 and 2011 for the whole of England and London in order to facilitate comparisons between areas over time and we present those in Figure 1 and Figure 2 respectively. Furthermore, we present maps of social fragmentation and its underlying indicators at each time point for every region in supplementary Appendix 2. Temporal changes in social fragmentation for England and for all English regions are presented in Figure A in the online appendix. We identified a pattern of stability with modest increases over time for regions in the North, and small decreases in social fragmentation for regions in the South. However, temporal changes of English regions for two of the four social fragmentation underlying indicators were much larger and they are presented in Figure 3.

Large changes in absolute levels of single person households and private renting were found in all regions. More specifically, in 2001 the average number of single persons of the total population was 38.4% while in 2011 the figure was 52.6% indicating a 31% increase. Similarly, in 2001 the average number of people in private renting was 8.5% of all English households while in 2011 this figure increased to 16.2% indicating a 62% increase. The largest increases in numbers of single people were found in the North East, North West and West Midlands while London had the highest levels of single people. Moreover, the largest increases for private renting were found in the North East, West Midlands and East Midlands while London had again the largest numbers of people living in private renting. For the other two social fragmentation underlying indicators, we found that the average number of one person households both nationally and regionally remained relatively stable. We also observed a modest decrease, both nationally and regionally (with the exception of London), in our measure of population turnover which reflects the levels of migration inside and outside areas. Finally, we observed great variability in levels of social fragmentation and its underlying indicators within regions at both time points. High levels of social fragmentation were concentrated in London, the North West and the South West. East England had the lowest levels of social fragmentation followed by West Midlands. There was a clear distinction between rural and urban areas, with the latter having far greater levels of social fragmentation.

Across the whole of England, Moran's I for social fragmentation was very low at 0.0449 in 2001 (95% CI 0.0447 to 0.0451) but by 2011 it had increased to 0.0794 (95% CI 0.0792 to 0.0797), indicating a small increase in spatial autocorrelation and clustering (Figure 4). We also observed marked regional variability in social fragmentation spatial autocorrelation levels over time (Figure 4). In both time points, the West Midlands had the lowest spatial autocorrelation in social fragmentation, followed by the East of England, while the South

For peer review only

For peer review only - http://bmjopen.bmj.com/site/about/guidelines.xhtml

East and South West had the highest levels of spatial autocorrelation at both time points. In contrast to increases in spatial autocorrelation across the country between 2001 and 2011, the South East, North East and Yorkshire & the Humber had decreasing levels of spatial autocorrelation over the two time points.

Spatial clustering also appears to have increased over time for all fragmentation underlying indicators nationally, although its levels remained low (Figures A1 and A2 in supplementary Appendix). For one person households we found very low regional clustering and small regional variation. For single persons and private renting regional variation in clustering was moderate and their levels were also low with the exception of the South East and South West. Finally, for population turnover we observed the greatest increases of spatial autocorrelation both nationally and regionally but levels of regional clustering were generally low with the exception of South West whilst regional variability was moderate. We provide graphs and discuss the results from the LISA analysis for local spatial autocorrelation in the supplementary material (Figures B1 and B2).

DISCUSSION

Between 2001 and 2011, we found evidence of strong persistence of social fragmentation in England at small-area level. Regional changes over time were modest for social fragmentation, which is not surprising considering the large population denominators and the relative nature of the measure. Similarly, we observed modest increases over time for one person households and population turnover. However, changes over time for single persons and private renting were much greater, with substantial increases in both underlying indicators across regions, and these specific influences appear to have largely driven the heightened levels of social fragmentation overall. Furthermore, our findings provide evidence of increasing clustering for social fragmentation and its underlying indicators which, in conjunction with the regional variation observed between the two time points, indicates that areas in the South of England (including London) appear much more clustered than the North. Finally, we observed increased clustering for all underlying indicators of social fragmentation over the two time points.

Strengths and limitations of the study

The main strength of this study lies in its large dataset for the whole of England (53 million people in 2011) over two time points across 32,844 LSOAs, which we utilised to investigate spatial and temporal patterns of social fragmentation. To our knowledge, it is the first study of its kind to provide an insight into the temporal persistence of social fragmentation and its clustering.

For peer review only - http://bmjopen.bmj.com/site/about/guidelines.xhtml

However, some limitations exist. First, the extent to which the index captures social fragmentation may vary across the country as the demographic factors that we used may not in themselves be valid indicators of social fragmentation, or they may adequately measure social fragmentation in some areas but not in others. [22] However, the measure has been reported widely in the literature and on this basis social fragmentation has been identified as an important predictor of mental health outcomes such as suicide. Second, Census questions do not usually directly elicit the presence or quality of neighbourhood social ties or institutions, or people's relationship to their neighbourhood. Therefore, we might have failed to capture people's perception of social fragmentation through the Census data. Nevertheless, we used a measure capturing compositional aspects of neighbourhood which appear to be related to the capacity of a neighbourhood to act collectively, and is also important for the construction and maintenance of social ties and institutions. Third, whilst Census data is recognised to be of a high standard, some unobserved heterogeneity persists. [33] For example, LSOAs are defined from administrative information and this may disguise local effects as contextual characteristics within administrative neighbourhoods are not homogeneous across LSOAs. [34] An alternative approach would require the use of non-administrative data to define levels, but the Census is currently the only source from which the data on the fragmentation underlying indicators is available. Fourth, for the underlying indicator 'single people' we used data on legal marital status in 2001 and 2011 and registered civil partnership status in 2011. However, the population base for this indicator is not restricted to those households for which legal marital status information is available and this has implications for the total numbers of single people. For example, some of those who are single, widowed or divorces could be living in institutions as well as cohabiting unions which will result in counting cohabitants as 'single, widowed or divorced' in the calculation of the indicator. Fifth, there were boundary changes following the 2011 Census that may have affected our findings, [35] but only 2.5% of LSOAs were affected and we developed an algorithm to make reasonable population weighted-based estimates for these localities, depending on whether they merged, split or some other change occurred. Finally, the index of social fragmentation may serve as a suitable tool to inform allocation of mental health resources in adults, although recent evidence suggests that social fragmentation may not have an effect on children's mental health.[36]

Interpretation of findings

A previous study showed that between 1971 and 2001, levels of social fragmentation increased steadily with large increases between 1981 and 1991 and modest increases between 1991 and 2001. Our results indicate that this trend was reversed in 2011 as we found a small decrease in the overall index of social fragmentation between 2001 and 2011. [37] Furthermore, our results suggest that urbanisation drives the high persistence of social

For peer review only - http://bmjopen.bmj.com/site/about/guidelines.xhtml

fragmentation in England as we found that levels of social fragmentation and its underlying indicators were consistently higher in urban areas. This may be because urban areas are chosen by diverse social and cultural groups and living in these areas may lead to isolated spaces, lifestyles and problems of social integration. Furthermore, we found that the underlying indicators that drive the persistency of social fragmentation appear to be single people and households in private renting, a characteristic of urban environments, [38] as these were the underlying indicators with the largest increases (Figure 3).

In absolute terms, private renting was considerably increased in all regions and most notably London; a finding that pertains to unaffordable housing for first-time home buyers and less availability of social housing as a result of the 2008 financial crisis.[39] Similarly, all regions had higher levels of single people in 2011, indicating that numbers of young professionals, students and divorces have increased substantially over the decade. For the other two underlying indicators we did not observe any marked changes across regions, although levels of population turnover appeared to have decreased between the decennial Censuses. The increases we observed in the proportion of single people and those in private renting warrant implications for the prevalence of mental illness especially in urban centres. The private house market is characterised by poor housing conditions as opposed to social houses or owner occupation and this can have detrimental effects on mental health. [40] Furthermore, it is suggested that married individuals may have better mental health outcomes than single individuals. [41] We believe that these changes are certainly of concern as they may highlight the need for more effective social interventions to target socially fragmented areas in England.

Spatial clustering of neighbourhood social fragmentation appears to have increased over time in England while we observed moderate regional variations in levels of clustering despite the small changes in levels of social fragmentation across regions. Increased levels of spatial clustering for social fragmentation across and within regions can have implications for the planning and organisation of health services, as this would imply needs that are not uniformly distributed across a region with the presence of hot spots of high levels of mental illness prevalence, especially in urban areas. To address such spatial health inequalities, services may need to be redesigned locally [42] while infrastructure and spending for mental health needs to be weighted towards social fragmentation hot spots rather than uniformly distributed.

Conclusion

In the absence of individual-level data, area-level indicators are often the only source of information to investigate how factors associated with mental illness may contribute to population health, albeit when individual-level data are available and are fitted as covariates

For peer review only

these area-level associations may be attenuated substantially. Even though, social fragmentation is increasingly considered a determinant of mental illness as more epidemiological research emerges that investigates contextual effects on health. To improve mental health in the population, policymakers need to address both relational and structural issues, perhaps by investing in reorganisation of mental healthcare. Solving the problems of cohesion and integration needs to be seen as a collective responsibility across central and local government and all the local agencies. Policies needs to address social and educational inequalities by strengthening the economic well-being of areas by lowering local unemployment rates, encouraging populations to bring social support in the communities and encouraging education of the population, which are key strategies for healthy communities. Communities need to work towards tightening social networks and social bonds to help with mental illness prevalence and suicide prevention, for example by promoting participation in charity, voluntary or community groups and civic engagement. The spatial aspect of social fragmentation is often overlooked, but it can provide vital information for the effective organisation of health services and targeting of health or social interventions. The nature of fragmentation and the mechanisms underlying its association with mental illness are of increasing interest as the population gets older and the health care costs associated with mental illness escalate in industrialised countries.

For peer review only

Acknowledgments

We would like to thank the Office of National Statistics for the wealth of information they have collected and systematically organised, which made this study possible.

Contributors

EK, CG, DA and LM designed the study. CG extracted the data from all sources, performed the analyses and drafted the manuscript. EK, RW, DA, LM, NK critically revised the manuscript. CG is the guarantor of this work and, as such, had full access to all the data in the study and takes responsibility for the integrity of the data and the accuracy of the data analysis.

Funding

This study is funded by the National Institute for Health Research School for Primary Care Research (NIHR SPCR), through CG's PhD. This report is independent research by the National Institute for Health Research. LM acknowledges financial support from the MRC Skills Development Fellowship (MR/N015126/1). The views expressed in this publication are those of the authors and necessarily those of the NHS, the National Institute for Health Research, the Department of Health, or the MRC.

Competing interests

None declared.

Provenance and peer review

Not commissioned; externally peer reviewed

Data sharing agreement

The data used in this study are freely available and the authors are happy to share in an organised and cleaned final dataset.

Ethical approval and Patient and Public Involvement

Not applicable. The study uses freely reported data aggregates at low geography levels. No patients or members of the public were involved in the study.

Open access

This is an Open Access article distributed in accordance with the terms of the Creative Commons Attribution (CC BY 4.0) license, which permits others to distribute, remix, adapt and build upon this work, for commercial use, provided the original work is properly cited. See: http://creativecommons.org/licenses/by/4.0/ © Article author(s) (or their employer(s) unless otherwise stated in the text of the article) 2018. All rights reserved. No commercial use is permitted unless otherwise expressly granted.

For peer review only

For peer review only - http://bmjopen.bmj.com/site/about/guidelines.xhtml

Table 1 Temporal changes in social fragmentation metrics across regions, 2001 - 2011

	North East	North West	Yorkshire and Humber	East Midlands	West Midlands	East England	London	South East Coast	South Central	South West	England
Social Fragmentation 2001											
No. of LSOAs	1,656	4,459	3,293	2,732	3,482	3,550	4,765	2,750	2,569	3,226	32,482
25th Centile	-2.32	-2.34	-2.31	-2.59	-2.59	-2.50	-0.46	-2.37	-2.51	-2.09	-2.26
Median	-0.79	-0.63	-0.87	-1.34	-1.19	-1.27	2.05	-1.09	-1.24	-0.88	-0.75
75th Centile	0.82	1.45	0.91	0.47	0.44	0.37	4.45	0.86	0.76	0.89	1.46
Weighted mean	-0.29	-0.06	-0.16	-0.55	-0.71	-0.68	2.20	-0.21	-0.33	-0.18	0.03
Standard deviation	3.26	3.50	3.50	3.24	2.94	2.75	3.28	3.24	3.16	3.28	3.32
Social Fragmentation 2011											
No. of LSOAs	1,657	4,497	3,317	2,774	3,487	3,614	4,835	2,773	2,609	3,281	32,844
25th Centile	-2.16	-2.31	-2.30	-2.64	-2.45	-2.59	-0.64	-2.50	-2.63	-2.17	-2.29
Median	-0.58	-0.51	-0.78	-1.27	-1.01	-1.30	1.58	-1.19	-1.27	-0.86	-0.70
75th Centile	0.97	1.62	1.07	0.58	0.65	0.39	3.98	0.76	0.62	1.02	1.43
Weighted mean	-0.01	0.16	0.04	-0.43	-0.45	-0.67	1.83	-0.25	-0.41	0.02	0.09
Standard deviation	3.13	3.29	3.37	3.22	2.82	2.81	3.56	3.34	3.28	3.33	3.37

For peer review only

For peer review only - http://bmjopen.bmj.com/site/about/guidelines.xhtml

Figure 1 Changes in overall social fragmentation for England, 2001 (left) – 2011 (right) (pooled sample)

Table 1 Temporal changes in social fragmentation metrics across regions, 2001 – 2011 (pooled sample)

Figure 2 Changes in overall social fragmentation for London, 2001 (left) – 2011 (right)

Figure 3 Percentage of single people, one person households, private renting and population turnover for English regions, over time (* Red line indicates mean for England in 2011)

Figure 4 Spatial Autocorrelation with Moran's I for Social Fragmentation by region, over time

For peer review only

For peer review only - http://bmjopen.bmj.com/site/about/guidelines.xhtml

References

1. Pickett KE, Pearl M. Multilevel analyses of neighbourhood socioeconomic context and health outcomes: a critical review. *J Epidemiol Ccommunity Health* 2001;55(2):111-22.
2. Stafford M, Marmot M. Neighbourhood deprivation and health: does it affect us all equally? *Int J Epidemiol* 2003;32(3):357-66.
3. Fagg J, Curtis S, Stansfeld SA, Cattell V, Tupuola AM, Arephin M. Area social fragmentation, social support for individuals and psychosocial health in young adults: evidence from a national survey in England. *Soc Sci Med* 2008;66(2):242-54.
4. Stafford M, De Silva M, Stansfeld S, Marmot M. Neighbourhood social capital and common mental disorder: testing the link in a general population sample. *Health Place* 2008;14(3):394-405.
5. Subramanian SV, Lochner KA, Kawachi I. Neighborhood differences in social capital: a compositional artifact or a contextual construct? *Health Place* 2003;9(1):33-44.
6. Veenstra G, Luginaah I, Wakefield S, Birch S, Eyles J, Elliott S. Who you know, where you live: social capital, neighbourhood and health. *Soc Sci Med* 2005;60(12):2799-818.
7. Congdon P. Suicide and parasuicide in London: A small-area study. *Urban Stud* 1996;33(1):137-58.
8. Congdon P. Commentary: Contextual effects: index construction and technique. *Int J Epidemiol* 2004;33(4):741-2.
9. Understanding patterns of deprivation. *Regional Trends* 2009;41(1):93-114.
10. Whitley E, Gunnell D, Dorling D, Smith GD. Ecological study of social fragmentation, poverty, and suicide. *BMJ* 1999;319(7216):1034-7.
11. Fagg J, Curtis S, Stansfeld S, Congdon P. Psychological distress among adolescents, and its relationship to individual, family and area characteristics in East London. *Soc Sci Med* 2006;63(3):636-48.
12. Fone D, Dunstan F, Lloyd K, Williams G, Watkins J, Palmer S. Does social cohesion modify the association between area income deprivation and mental health? A multilevel analysis. *Int J Epidemiol* 2007;36(2):338-45.
13. Evans J, Middleton N, Gunnell D. Social fragmentation, severe mental illness and suicide. *Soc Psychiatry Psychiatr Epidemiol* 2004;39(3):165-70.
14. Ivory VC, Collings SC, Blakely T, Dew K. When does neighbourhood matter? Multilevel relationships between neighbourhood social fragmentation and mental health. *Soc Sci Med* 2011;72(12):1993-2002.
15. Allardyce J, Boydell J. Review: the wider social environment and schizophrenia. *Schizophr Bull* 2006;32(4):592-8.
16. Ellen IG, Mijanovich T, Dillman K-N. Neighborhood Effects on Health: Exploring the Links and Assessing the Evidence. *J Urban Aff* 2001;23(3-4):391-408.
17. Collings S, Ivory V, Blakely T, Atkinson J. Are neighbourhood social fragmentation and suicide associated in New Zealand? A national multilevel cohort study. *J Epidemiol Community Health* 2009;63(12):1035-42.
18. Smith GD, Whitley E, Dorling D, Gunnell D. Area based measures of social and economic circumstances: cause specific mortality patterns depend on the choice of index. *J Epidemiol Community Health* 2001;55(2):149-50.
19. Corcoran P, Arensman E, Perry IJ. The area-level association between hospital-treated deliberate self-harm, deprivation and social fragmentation in Ireland. *J Epidemiol Community Health* 2007;61(12):1050-55.
20. Evans E, Hawton K, Rodham K. Factors associated with suicidal phenomena in adolescents: a systematic review of population-based studies. *Clin Psychol Rev* 2004;24(8):957-79.

For peer review only - http://bmjopen.bmj.com/site/about/guidelines.xhtml

21. Curtis S, Copeland A, Fagg J, Congdon P, Almog M, Fitzpatrick J. The ecological relationship between deprivation, social isolation and rates of hospital admission for acute psychiatric care: a comparison of London and New York City. *Health Place* 2006;12(1):19-37.
22. Allardyce J, Gilmour H, Atkinson J, Rapson T, Bishop J, McCreadie RG. Social fragmentation, deprivation and urbanicity: relation to first-admission rates for psychoses. *Br J Psychiatry* 2005;187:401-06.
23. van Os J, Driessen G, Gunther N, Delespaul P. Neighbourhood variation in incidence of schizophrenia. Evidence for person-environment interaction. *The British journal of psychiatry : the journal of mental science* 2000;176:243-8.
24. Stjarne MK, Ponce de Leon A, Hallqvist J. Contextual effects of social fragmentation and material deprivation on risk of myocardial infarction--results from the Stockholm Heart Epidemiology Program (SHEEP). *Int J Epidemiol* 2004;33(4):732-41.
25. Stafford M, Gimeno D, Marmot MG. Neighbourhood characteristics and trajectories of health functioning: a multilevel prospective analysis. *Eur J Public Health* 2008;18(6):604-10.
26. Jarvis GK, Ferrence RG, Whitehead PC, Johnson FG. The ecology of self-injury: a multivariate approach. *Suicide Llife Threat Behav* 1982;12(2):90-102.
27. Office for National Statistics. Families and Households in England and Wales 2011: Statistical Bulletin. 2013.
28. Office for National Statistics.Nomis official labour market statistics. 2013.
29. Office for National Statistics. Super Output Area mid-year population estimates for England and Wales, Mid-2011. 2016.
30. Bibby P, Shepherd J. Developing a new classification of urban and rural areas for policy purposes–the methodology. *London: DEFRA* 2004.
31. Moran PAP. Notes on Continuous Stochastic Phenomena. *Biometrika* 1950;37(1-2):17-23.
32. Anselin L. Local indicators of spatial association—LISA. *Geogr Anal* 1995;27(2):93-115.
33. Fewell Z, Smith GD, Sterne JAC. The impact of residual and unmeasured confounding in epidemiologic studies: A simulation study. *Am J Epidemiol* 2007;166(6):646-55.
34. Rehkopf DH, Buka SL. The association between suicide and the socio-economic characteristics of geographical areas: a systematic review. *Psychol Med* 2006;36(2):145-57.
35. Norman P, Rees P, Boyle P. Achieving data compatibility over space and time: creating consistent geographical zones. *Pop Space Place* 2003;9(5):365-86.
36. Flouri E, Midouhas E, Joshi H, Sullivan A. Neighbourhood social fragmentation and the mental health of children in poverty. *Health Place* 2015;31:138-45.
37. Dorling D, Vickers D, Thomas B, Pritchard J, Ballas D. Changing UK. *The way we live now. Sheffield: University of Sheffield* 2008.
38. Smith CW. 2011 Census Analysis: How Have Living Arrangements and Marital Status in England and Wales Changed Since 2001. *Office for National Statistics* 2011.
39. Kemp PA. Private Renting After the Global Financial Crisis. *Housing Stud* 2015;30(4):601-20.
40. Barnes M, Cullinane, C., Scott, S. & Silvester, S. . People living in bad housing: Numbers and health impacts. *UK: Shelter.* 2013.
41. Simon RW. Revisiting the relationships among gender, marital status, and mental health. *Am J Sociol* 2002;107(4):1065-96.
42. Lorant V, Thomas I, Deliege D, Tonglet R. Deprivation and mortality: the implications of spatial autocorrelation for health resources allocation. *Soc Sci Med* 2001;53(12):1711-9.

For peer review only - http://bmjopen.bmj.com/site/about/guidelines.xhtml

2001 **2011**

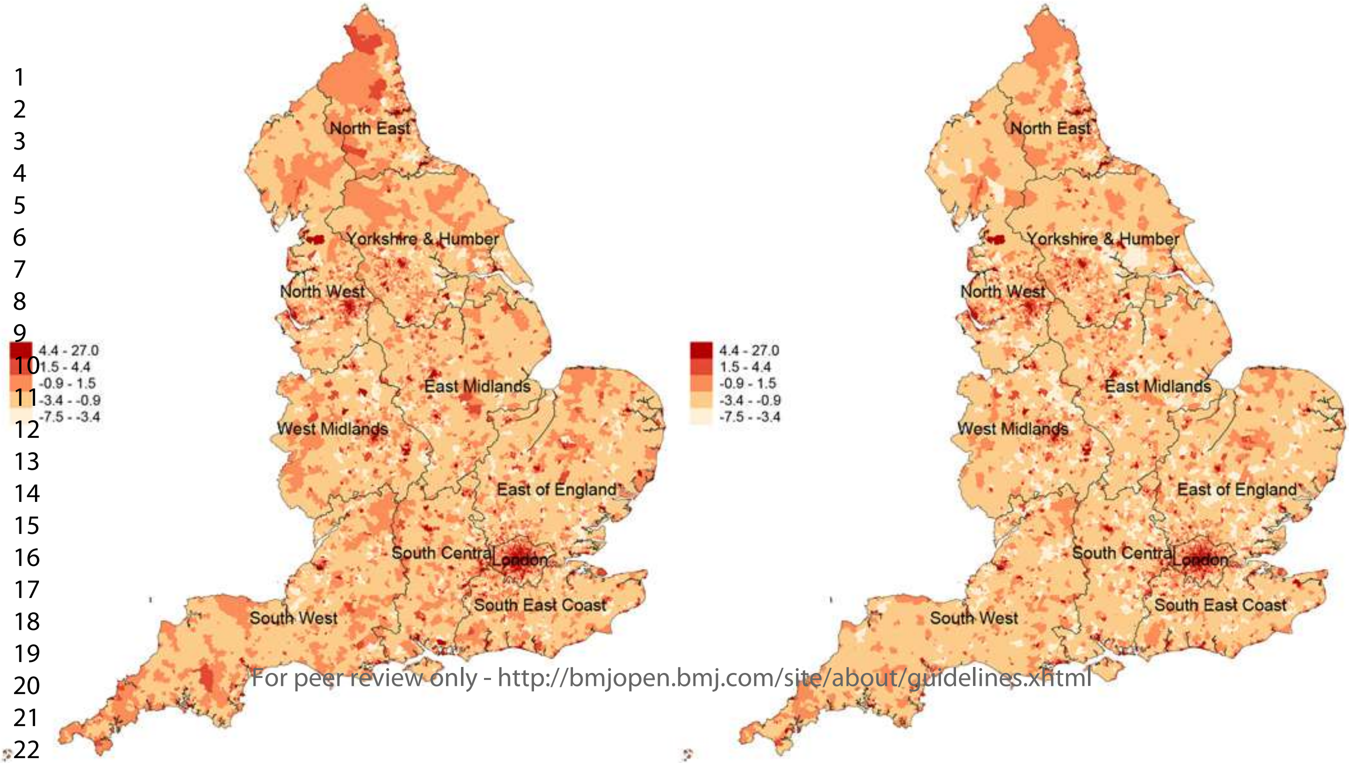
4.4 - 27.0
1.5 - 4.4
-0.9 - 1.5
-3.4 - -0.9
-7.5 - -3.4
North East
Yorkshire & Humber
North West
East Midlands
West Midlands
East of England
South Central
London
South East Coast
South West
4.4 - 27.0
1.5 - 4.4
-0.9 - 1.5
-3.4 - -0.9
-7.5 - -3.4
North East
Yorkshire & Humber
North West
East Midlands
West Midlands
East of England
South Central
London
South East Coast
South West

2001

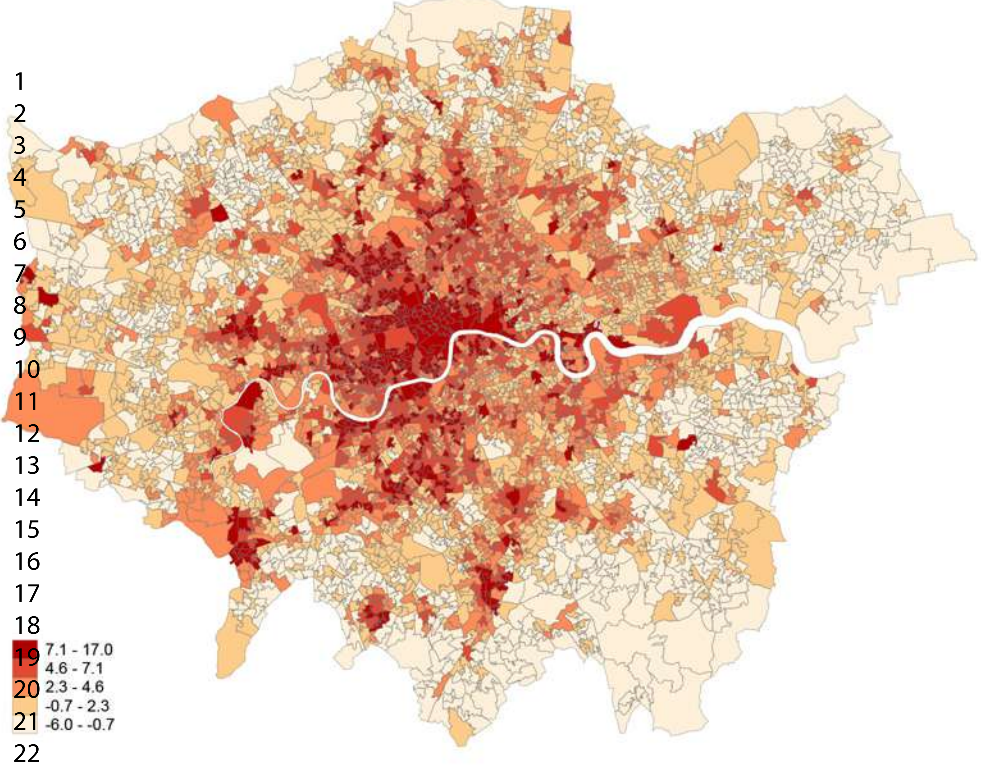

2011

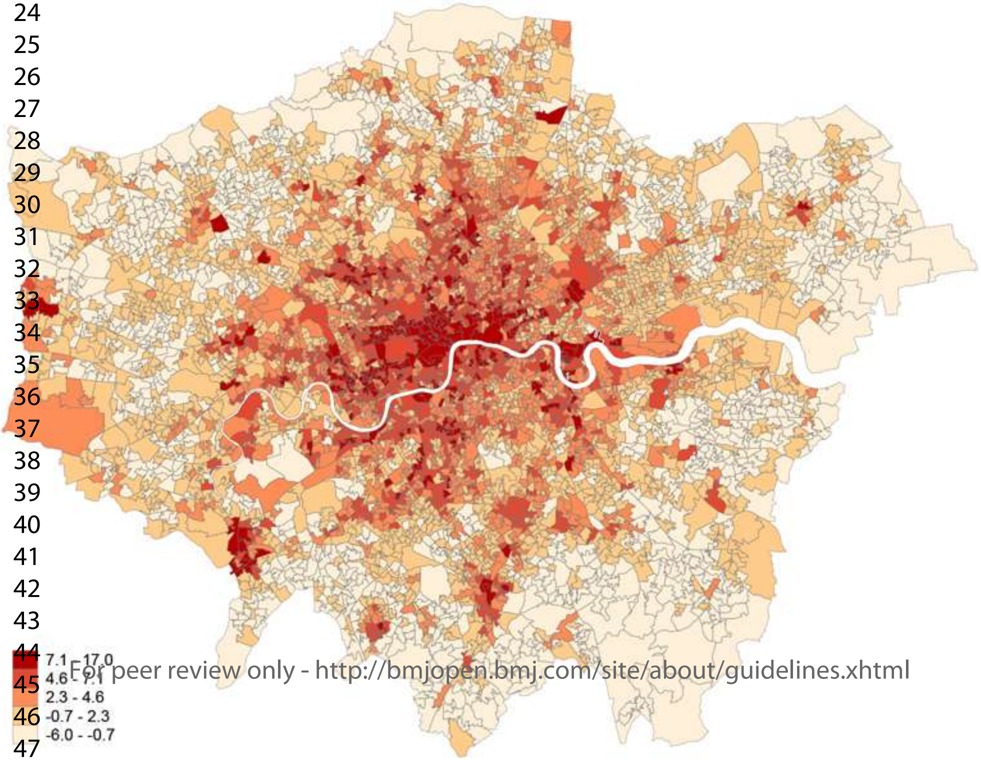

For peer review only - http://bmjopen.bmj.com/site/about/guidelines.xhtml

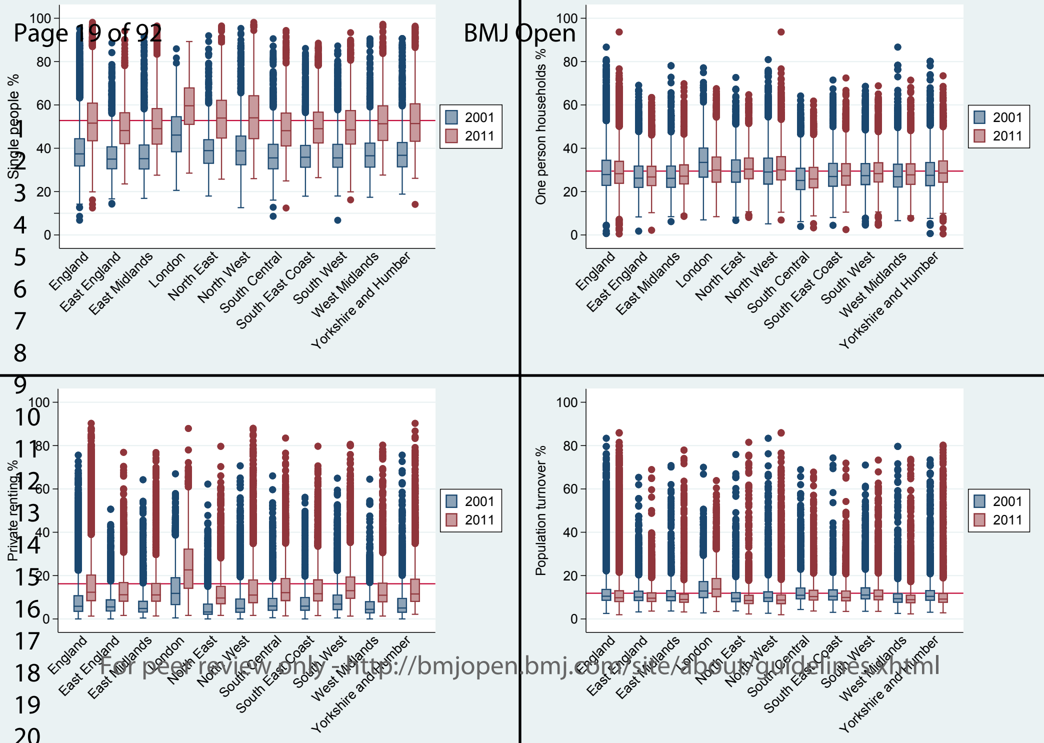

For peer review only - http://bmjopen.bmj.com/site/about/guidelines.xhtml

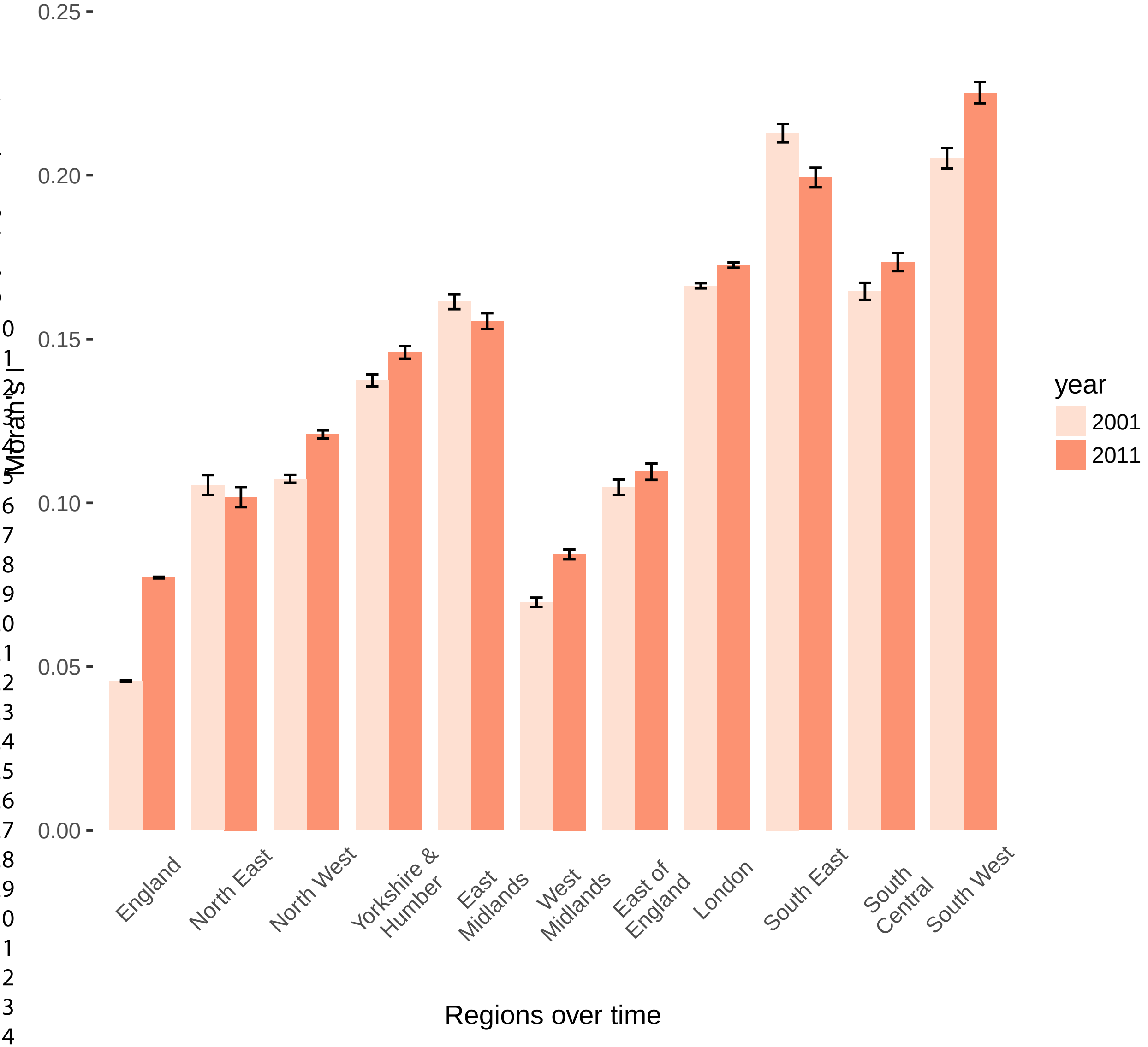
0.25
0.20
0.15
0.10
0.05
0.00
Moran's I
England
North East
North West
Yorkshire & Humber
East Midlands
West Midlands
East of England
London
South East
South Central
South West
Regions over time
year
2001
2011

Supplementary Appendix

Spatial distribution and temporal trends in social fragmentation in England, 2001 to 2011: a national study

For peer review only

For peer review only - http://bmjopen.bmj.com/site/about/guidelines.xhtml

Contents

Description of data and formulas used for the calculation of the underlying social fragmentation indicators at both time points 3

Spatial weighted regressions to attribute 2001 data to 2011 LSOAs 4

Spatial Maps........ 4

Figure A: Index of Social Fragmentation (mean) for England and English regions, over time. 5

Figure A1 Spatial autocorrelation with Moran's I for single persons (top) and one person households (bottom), by region and over time........ 6

Figure A2 Spatial autocorrelation with Moran's I for private renting (top) and population turnover (bottom), by region and over time........ 7

Figure B1: Local Moran scatterplot for social fragmentation at the LSOA level, 2001........ 8

Figure B2: Local Moran scatterplot for social fragmentation at the LSOA level, 2011........ 8

Results from LISA analysis:........ 9

References 10

For peer review only

Description of data and formulas used for the calculation of the underlying social fragmentation indicators at both time points

2001

Percentage of single people in LSOA = All singles, divorced, widowed and separated in LSOA / All usual residents aged 16 and above in LSOA (dataset available from: https://www.nomisweb.co.uk/census/2001/ks004)

Percentage of one person households in LSOA = Total one person households in LSOA/ All household composition in LSOA (dataset available from: https://www.nomisweb.co.uk/census/2001/uv065)

Percentage of private renting in LSOA = Total houses rented from a private landlord or letting agent in LSOA/ All household composition in LSOA (dataset available from: https://www.nomisweb.co.uk/census/2001/uv063)

Percentage of population turnover in LSOA= People who are migrants (i.e. moved into the area from within the UK & moved into the area from outside the UK within the year before the census / All usual residents in LSOA (dataset available from: https://www.nomisweb.co.uk/census/2001/ks024)

2011

Percentage of single people in LSOA = All singles, divorced, widowed and separated in LSOA / All usual residents aged 16 and above in LSOA (dataset available from: https://www.nomisweb.co.uk/census/2011/ks103uk)

Percentage of one person households in LSOA = Total one person households in LSOA/ All household composition in LSOA (dataset available from: https://www.nomisweb.co.uk/census/2011/ks105ew)

Percentage of private renting in LSOA = Total houses rented from a private landlord or letting agent in LSOA/ All household composition in LSOA (dataset available from: https://www.nomisweb.co.uk/census/2011/ks402ew)

Percentage of population turnover in LSOA= People who are migrants (i.e. moved into the area from within the UK & moved into the area from outside the UK within the year before the census / All usual residents in LSOA (dataset available from: https://www.nomisweb.co.uk/census/2011/ukmig008)

For peer review only - http://bmjopen.bmj.com/site/about/guidelines.xhtml

For peer review only

Spatial weighted regressions to attribute 2001 data to 2011 LSOAs

The 2003 National Statistics policy to minimise the statistical impact of frequent electoral ward boundary changes was succeeded by boundary changes across LSOAs in 2011. In that year, there was a modification of OA (Output Areas) and SOA (Super Output Areas) in England & Wales mainly due to significant population changes between the decennial censuses. As social fragmentation in 2001 was measured in 2001 LSOAs, we attributed census 2001 LSOA information to 2011 LSOAs and we used population weighted regressions to estimate 2001 data at the 2011 LSOA to enable seamless comparisons across time. More specifically, 97.4% of all LSOAs remained unchanged in 2011, and for these areas we took no action and attributed the social fragmentation values from 2001 to the 2011 LSOAs. If two or more 2001 LSOAs merged in one LSOA in 2011 (0.6% of all LSOAs), we calculated the mean value of social fragmentation weighted for 2003 LSOA population (which is the oldest population estimates dataset provided by ONS). If a 2001 LSOA split into two or more 2011 LSOAs (1.8% of all LSOAs) we assigned the relevant 2001 social fragmentation scores. Finally, there were some LSOAs for which changes did not match any of the previously described patterns (i.e. an LSOA split in two and each part merged with a different LSOA). For these very few LSOAs (0.1% of all LSOAs) we developed an algorithm to calculate population weighted mean estimates. (1) The corresponding algorithm is available from the authors.

Spatial Maps

Digital vector boundaries for the 2011 LSOAs, generalised to 20 metres and clipped to the coastline to reduce size and improve visualisation, were obtained from the ONS open geography portal (2) . The vector boundaries were inputted in the Stata shp2dta command to calculate the centroid for each LSOA in the British National Grid format (3). These were then converted from British National Grid easting and northing to longitude and latitude in degrees (4).

For peer review only - http://bmjopen.bmj.com/site/about/guidelines.xhtml

Figure A: Index of Social Fragmentation (mean) for England and English regions, over time.

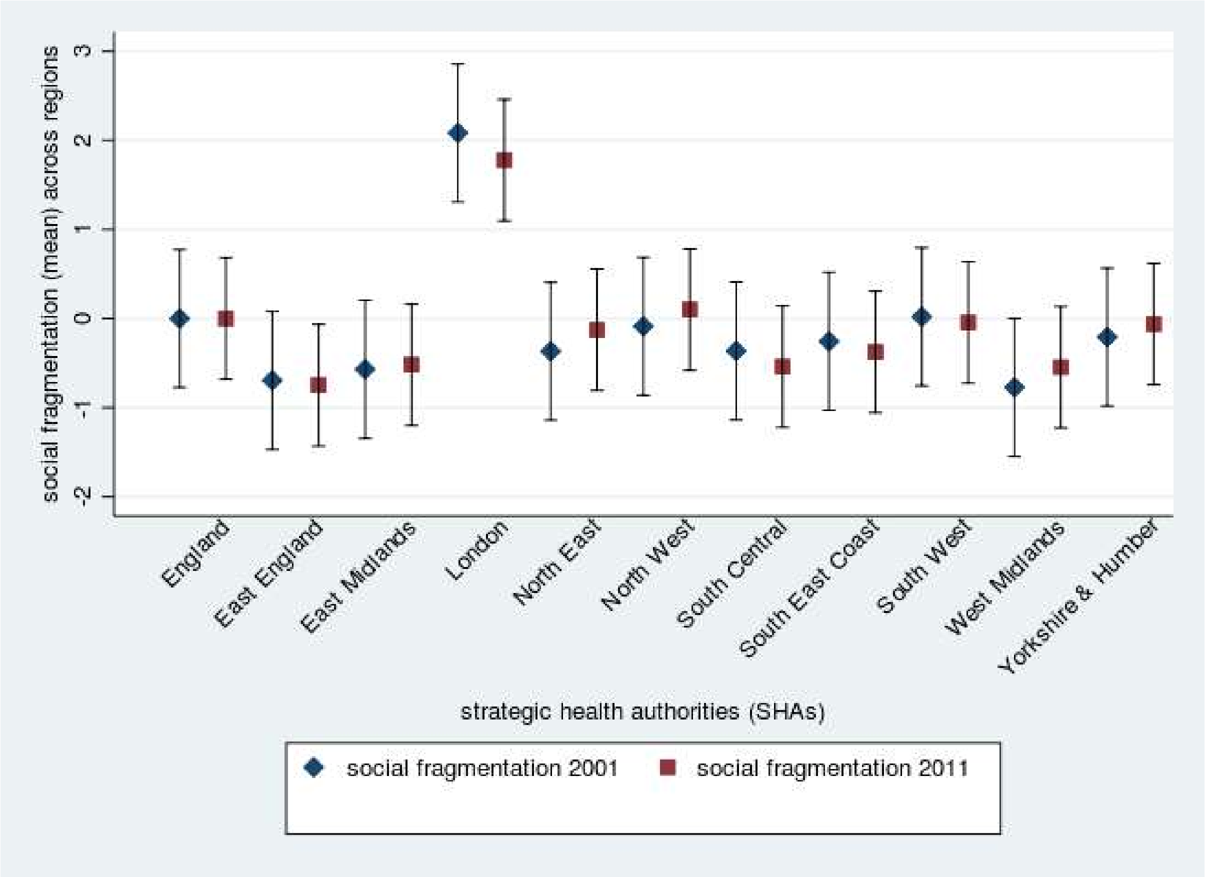

For peer review only - http://bmjopen.bmj.com/site/about/guidelines.xhtml

Figure A1: Spatial autocorrelation with Moran's I for single persons (top) and one person households (bottom), by region and over time

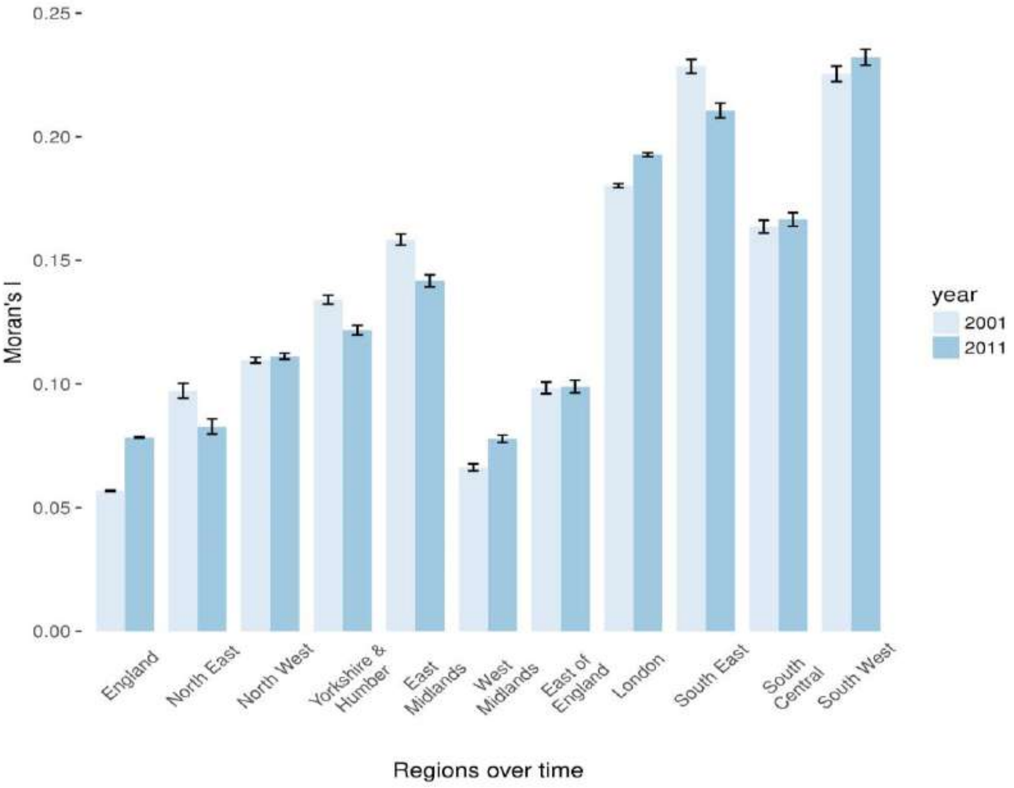

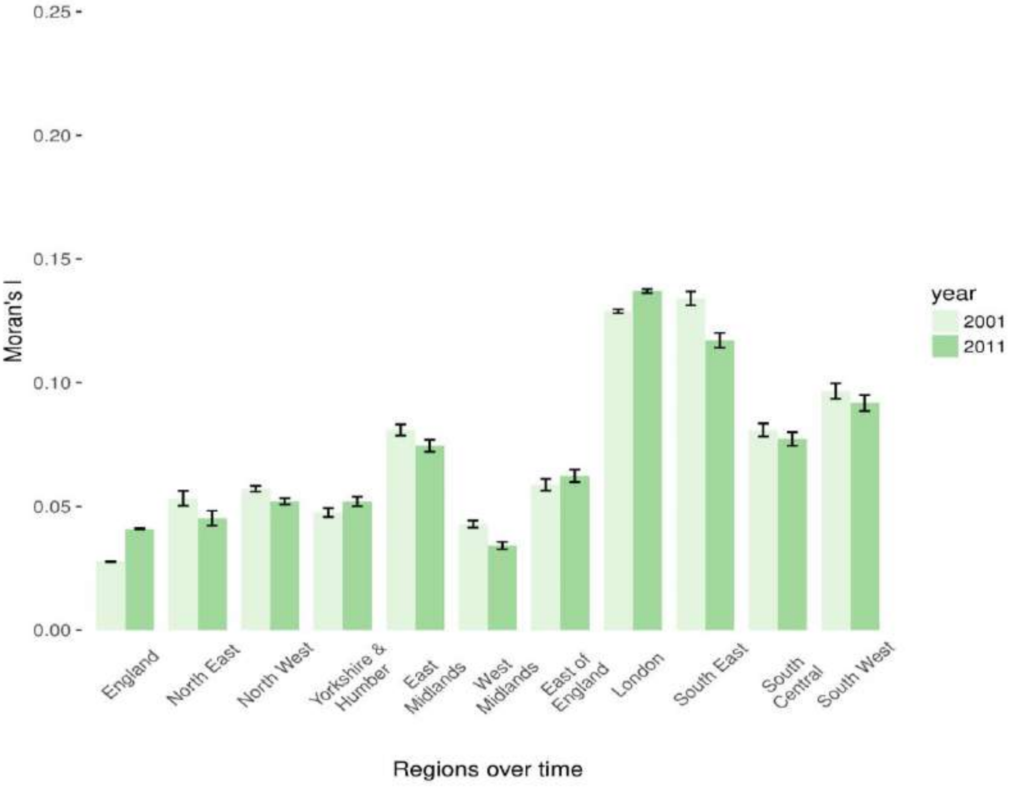

For peer review only - http://bmjopen.bmj.com/site/about/guidelines.xhtml

Figure A2: Spatial autocorrelation with Moran's I for private renting (top) and population turnover (bottom), by region and over time

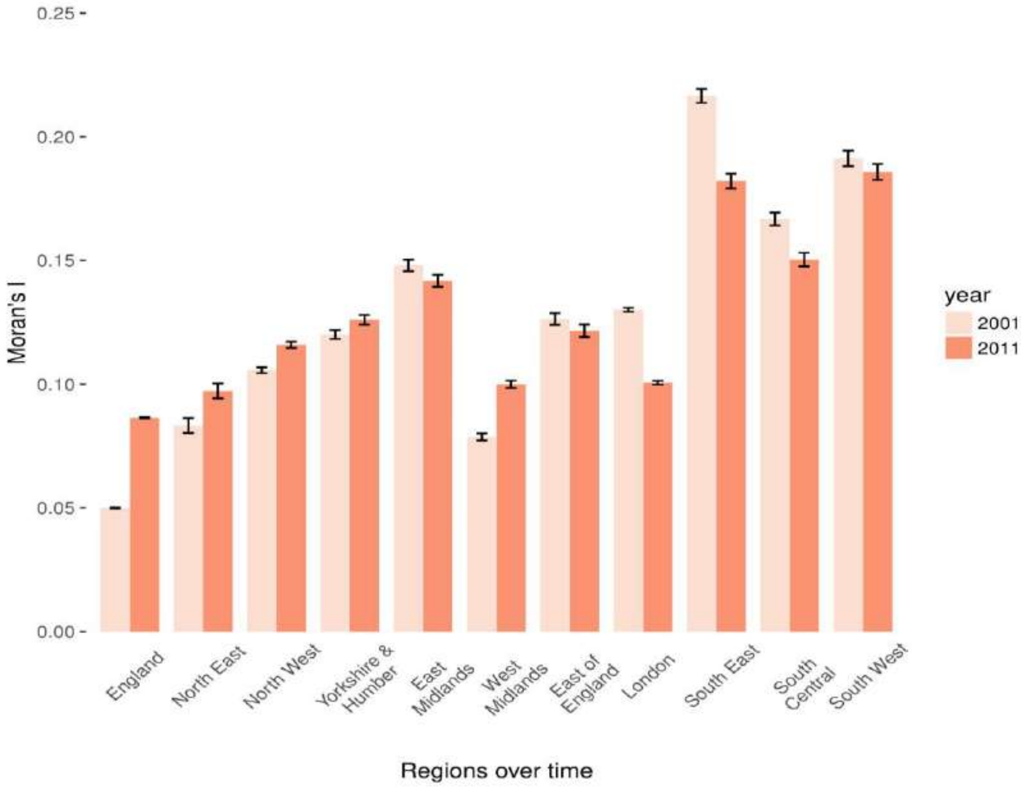

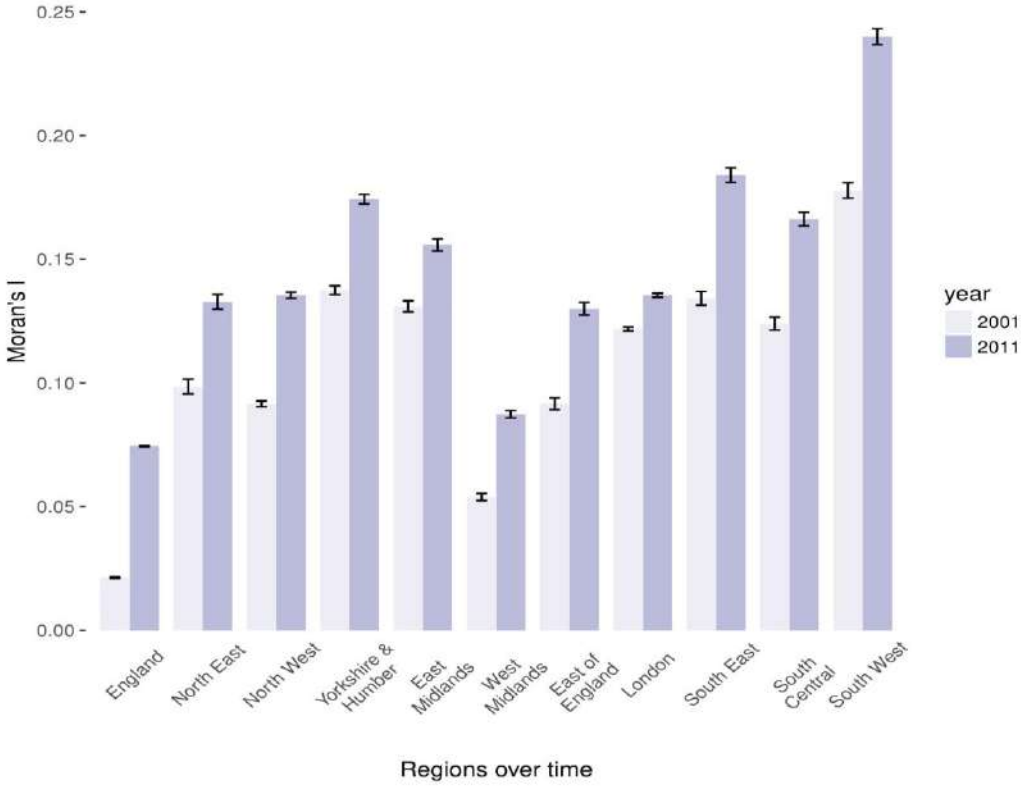

Figure B1: Local Moran scatterplot for social fragmentation at the LSOA level, 2001.

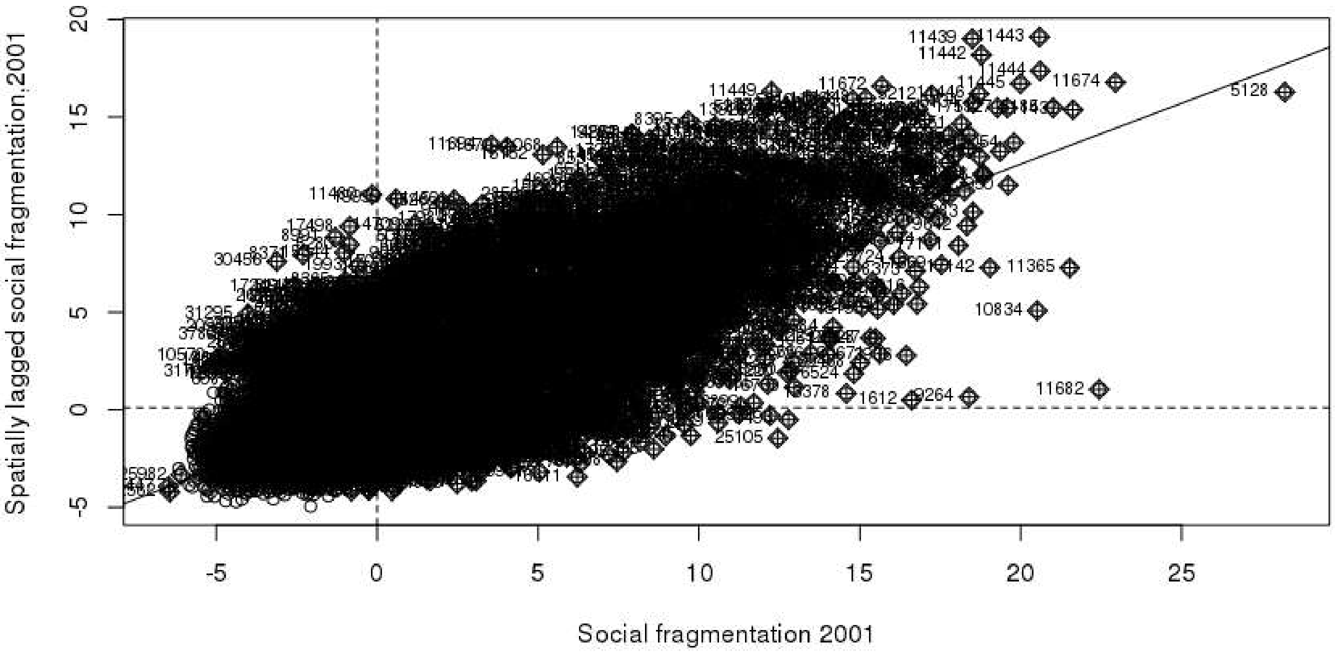

Figure B2: Local Moran scatterplot for social fragmentation at the LSOA level, 2011.

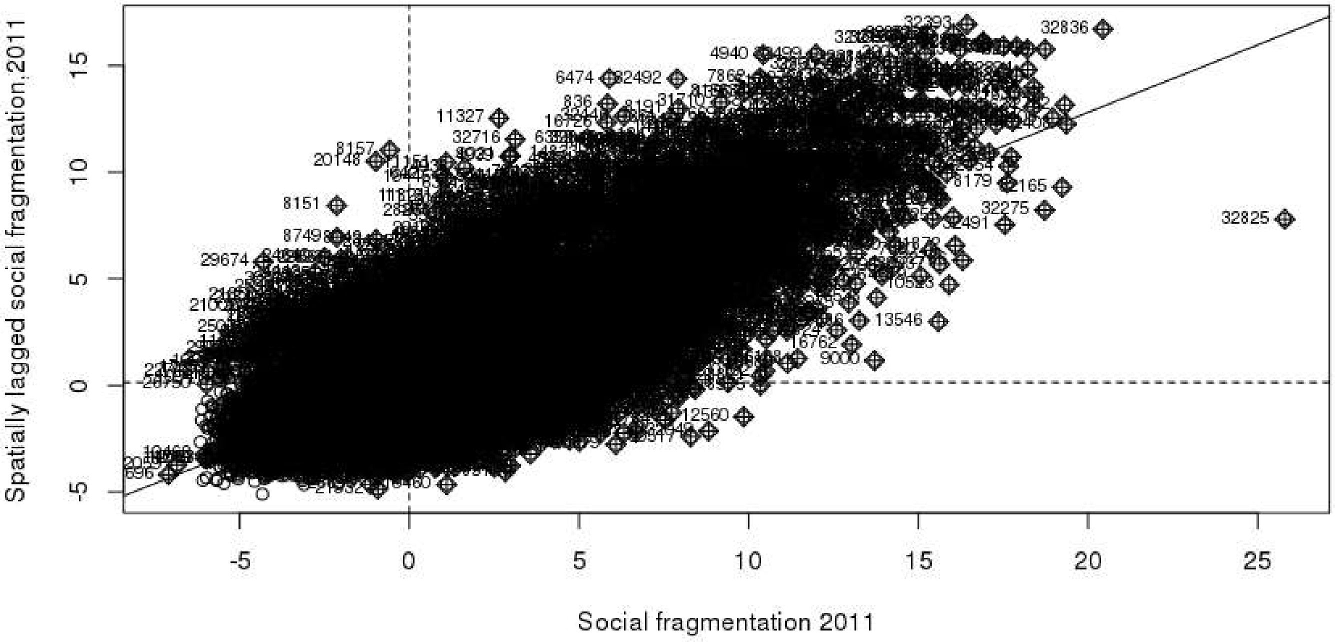

For peer review only - http://bmjopen.bmj.com/site/about/guidelines.xhtml

Results from Local Indicator of Spatial Association (LISA) analysis: The Moran scatterplots for social fragmentation in 2001 and 2011 are provided in Figures B1 and B2 respectively. The four quadrants in each scatterplot correspond to four types of association. The lower left and upper quadrants indicate positive spatial autocorrelation, or that areas in these quadrants have similar values (lower than the mean for those areas in the lower left quadrant and higher than the mean in the upper left quadrant). The upper left and lower right quadrant indicate spatial associations of areas with dissimilar values in social fragmentation. In both graphs the slope of the regression line indicates that there is a positive spatial association at both time points with a handful of outliers in 2001 and only a few in 2011. The pattern of increasing spatial autocorrelation across the two time points that we observed in graph 4 for global spatial autocorrelation is also confirmed by comparison of Graphs B1 and B2. Spatial autocorrelation appears to have increased in 2011 when compared to 2001. At both time points, LSOAs were mainly concentrated in the upper right quadrant (Figure B1 & B2) indicating the existence of many hot spots of social fragmentation and highlighting the fact that there are important variations among LSOAs and a finer scale spatial structure that occur below the region level.

For peer review only

References

1. Office for National Statistics. Changes to output areas and super output areas in England and Wales, 2001 to 2011. 2012.
2. Office for National Statistics. Open Geography Portal. 2013. https://geoportal.statistics.gov.uk/geoportal/.
3. SHP2DTA:. Stata module to converts shape boundary files to Stata datasets [program]. S456718 version: Boston College Department of Economics,. 2006.
4. Ordance Survey. A guide to coordinate systems in Great Britain. ONS, 2013:43. www.ordnancesurveycouk/docs/support/guide-coordinate-systems-great-britainpdf.

For peer review only

Social Fragmentation

England 2001, Social Fragmentation

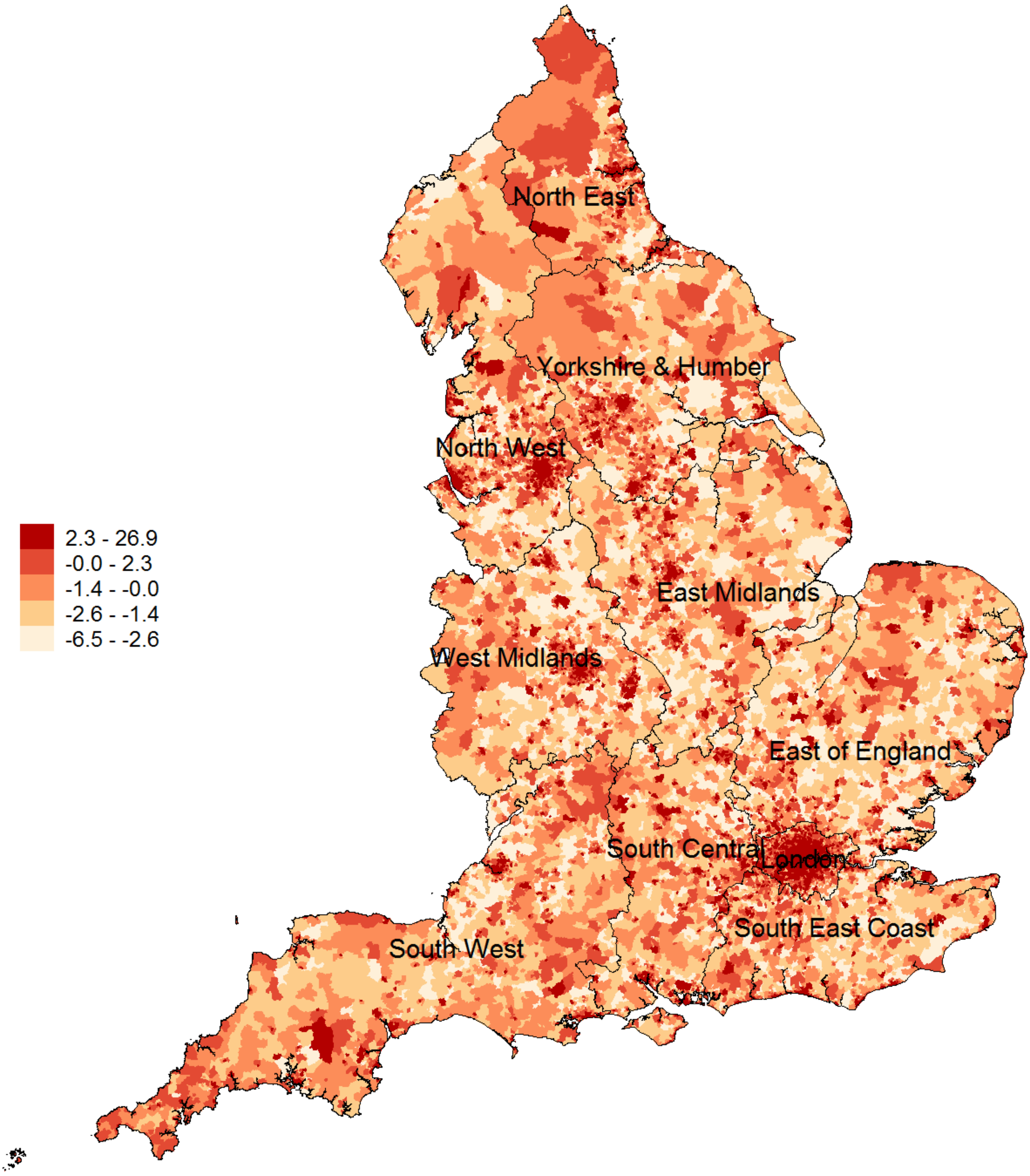

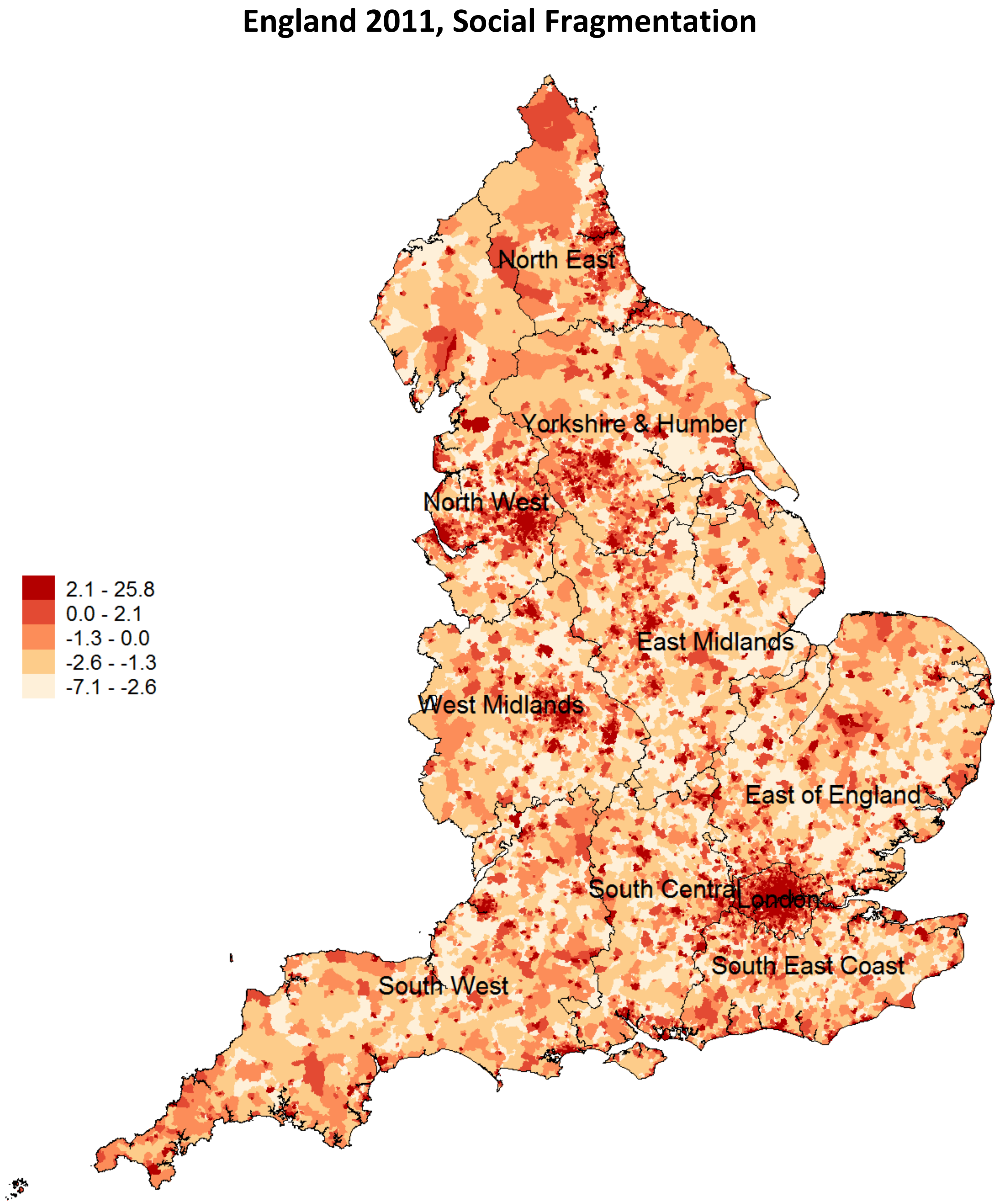
England 2011, Social Fragmentation
2.1 - 25.8
0.0 - 2.1
-1.3 - 0.0
-2.6 - -1.3
-7.1 - -2.6
North East
Yorkshire & Humber
North West
East Midlands
West Midlands
East of England
South Central
London
South East Coast
South West

North East, Social Fragmentation

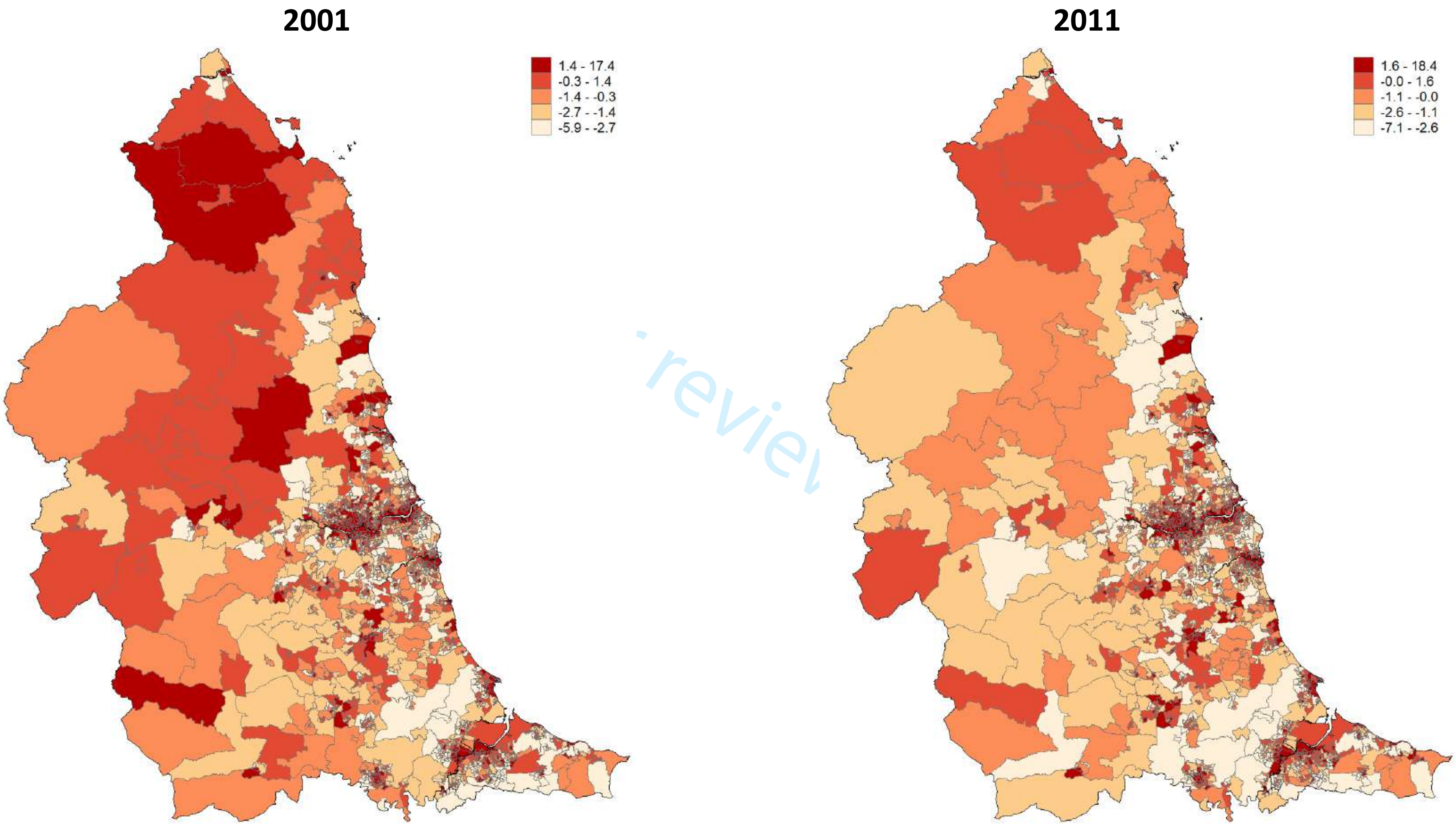

North West, Social Fragmentation

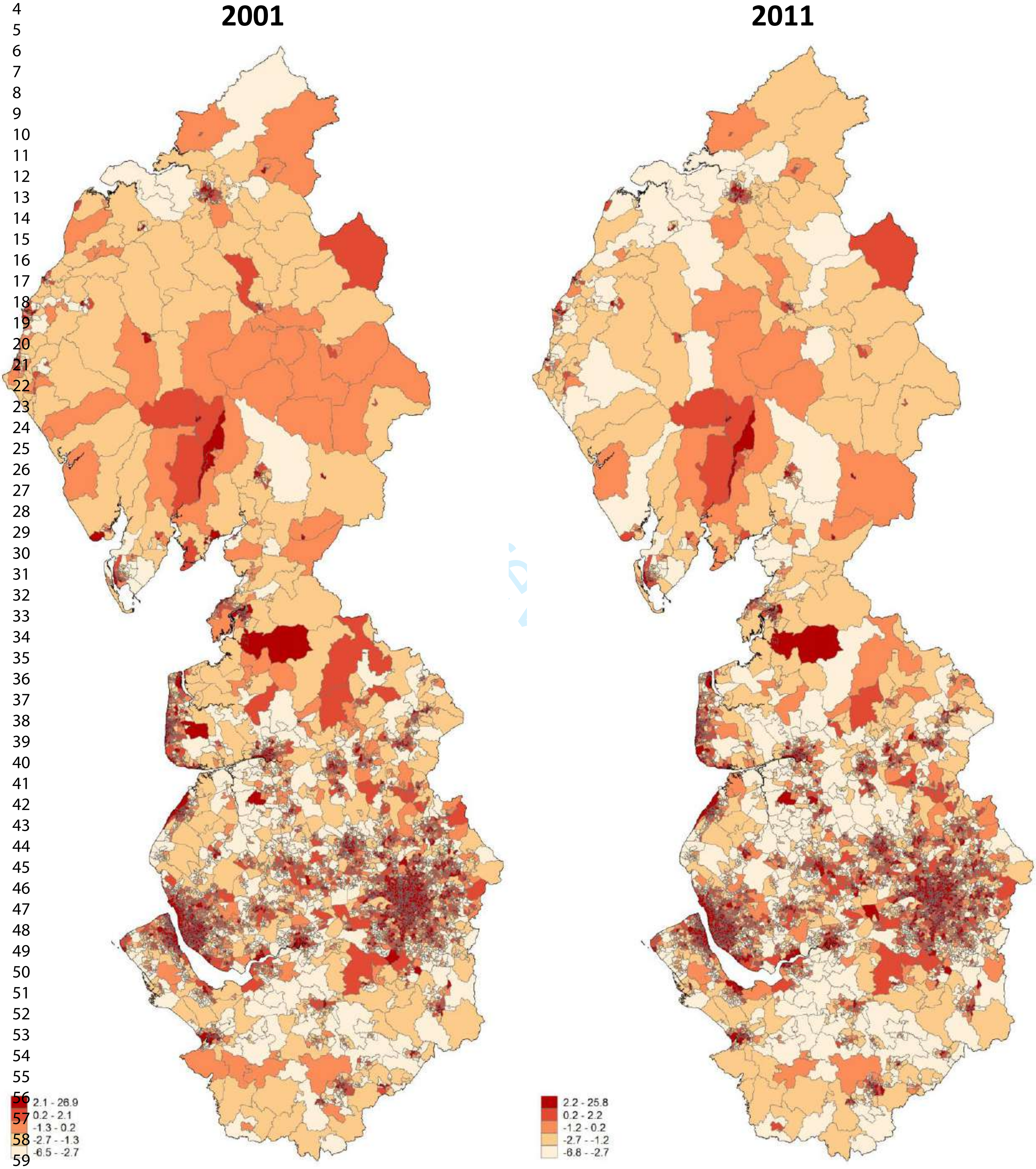

Yorkshire and the Humber, Social Fragmentation

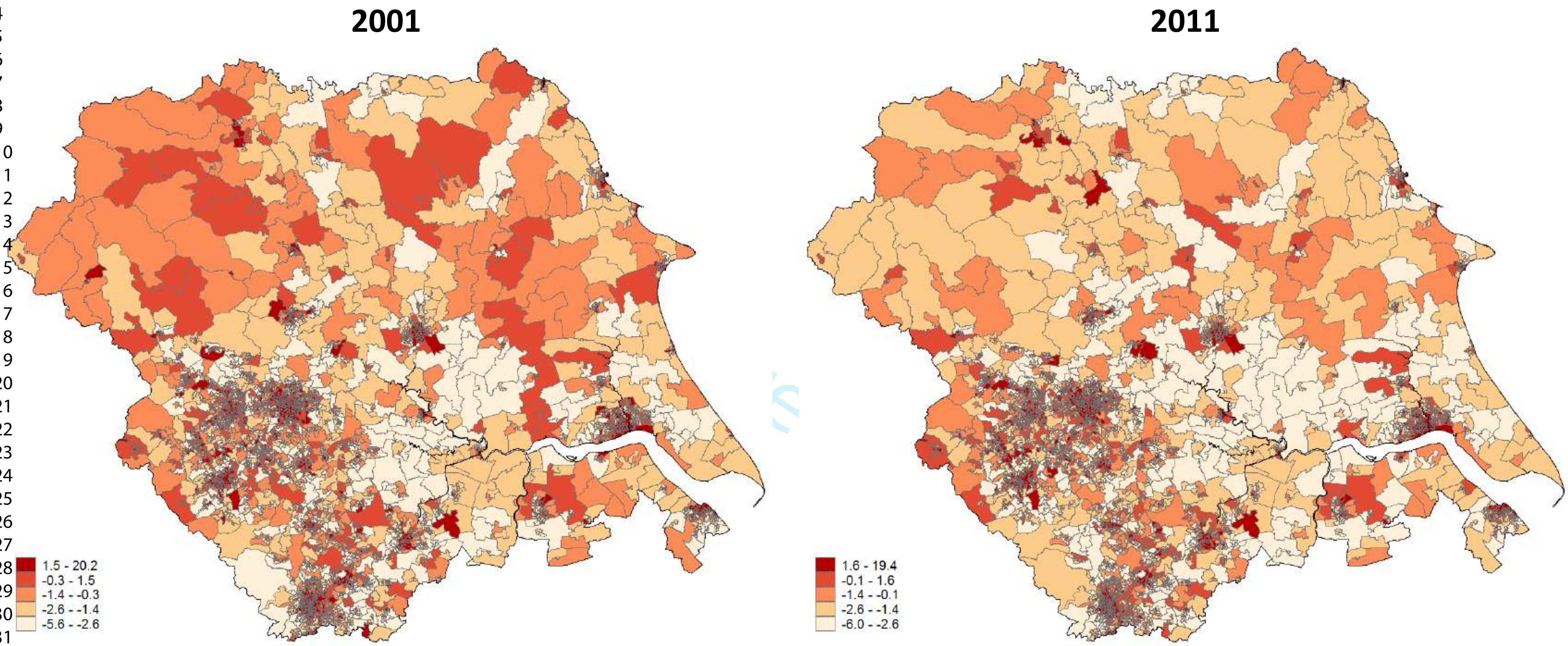

East Midlands, Social Fragmentation

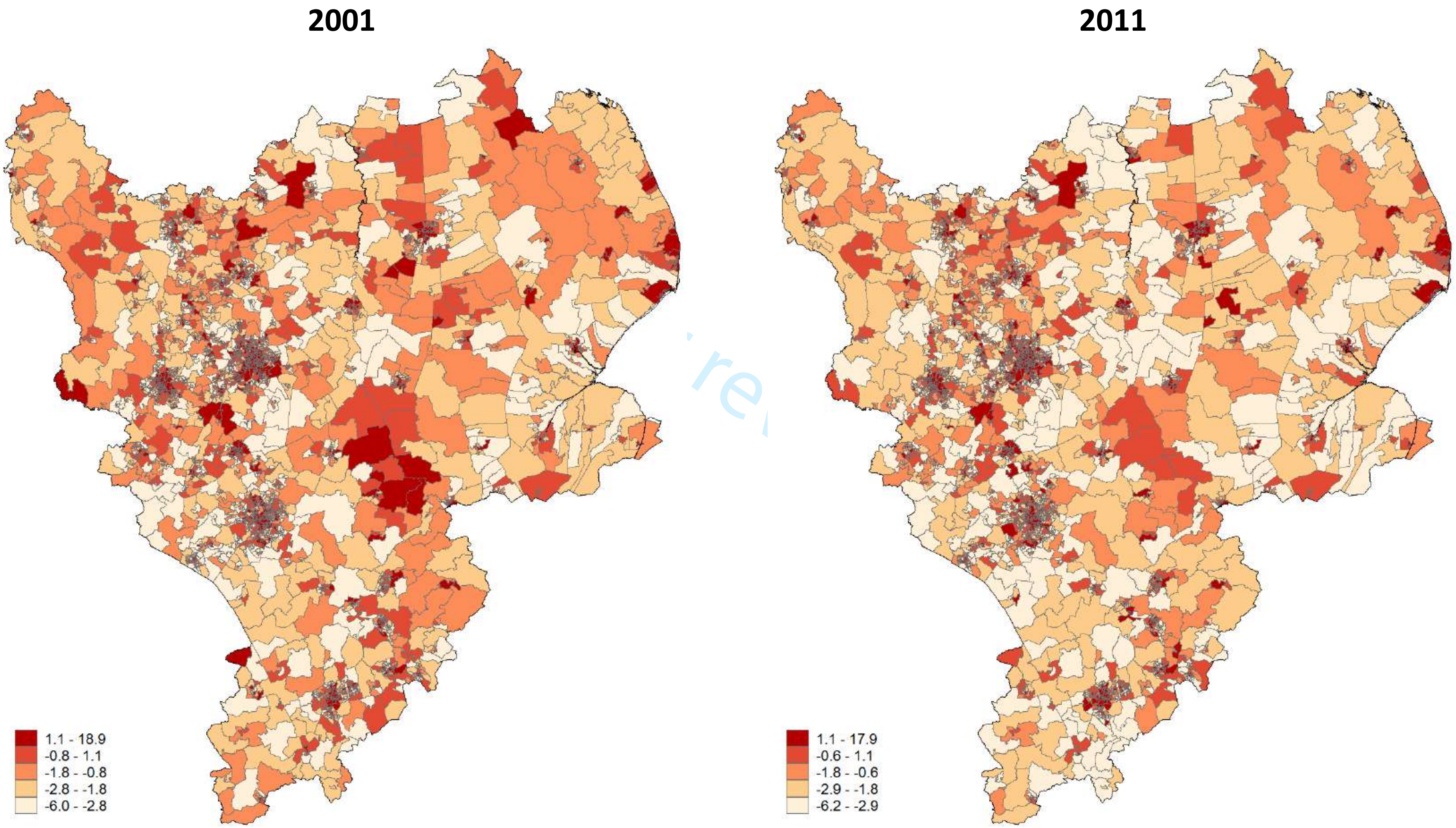

West Midlands, Social Fragmentation

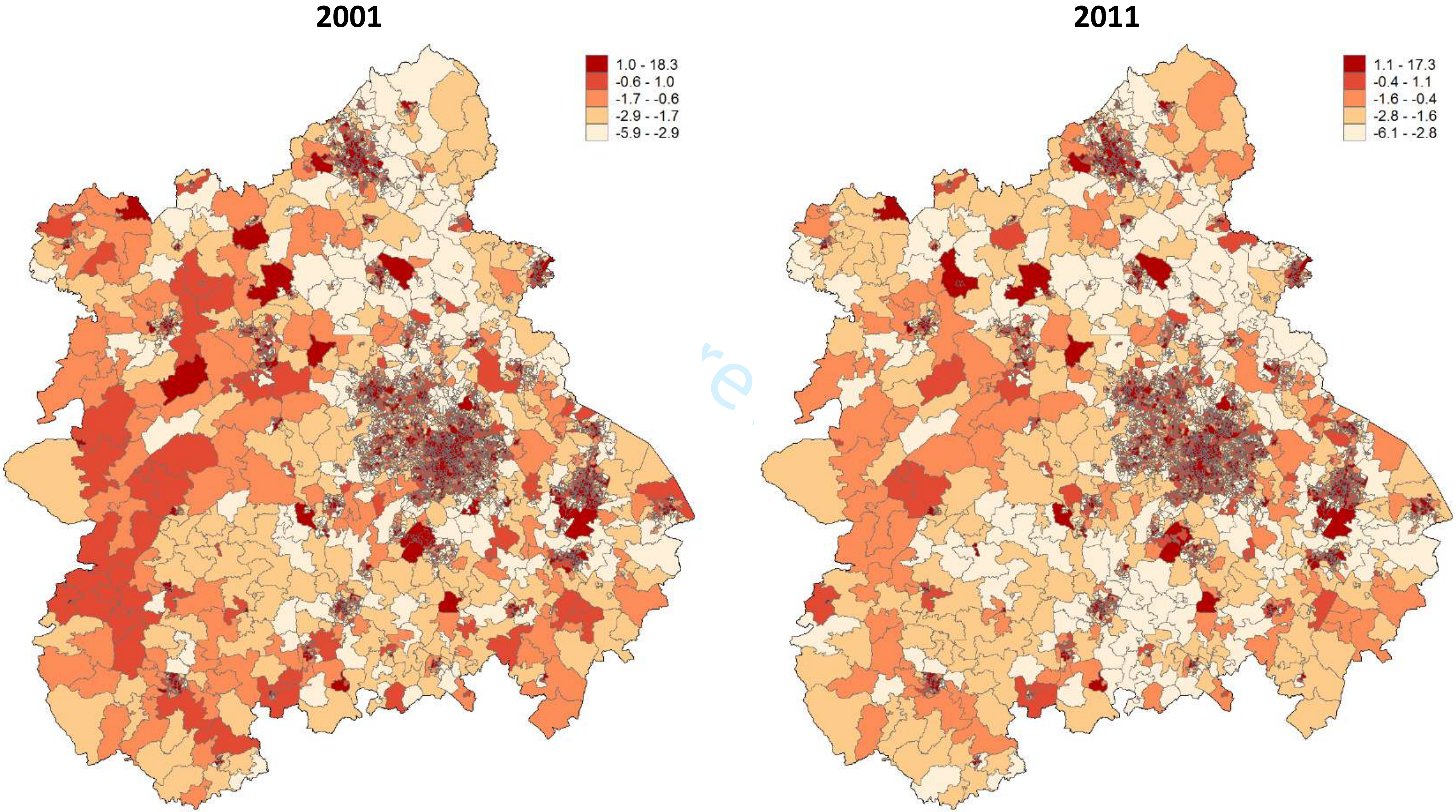

East of England, Social Fragmentation

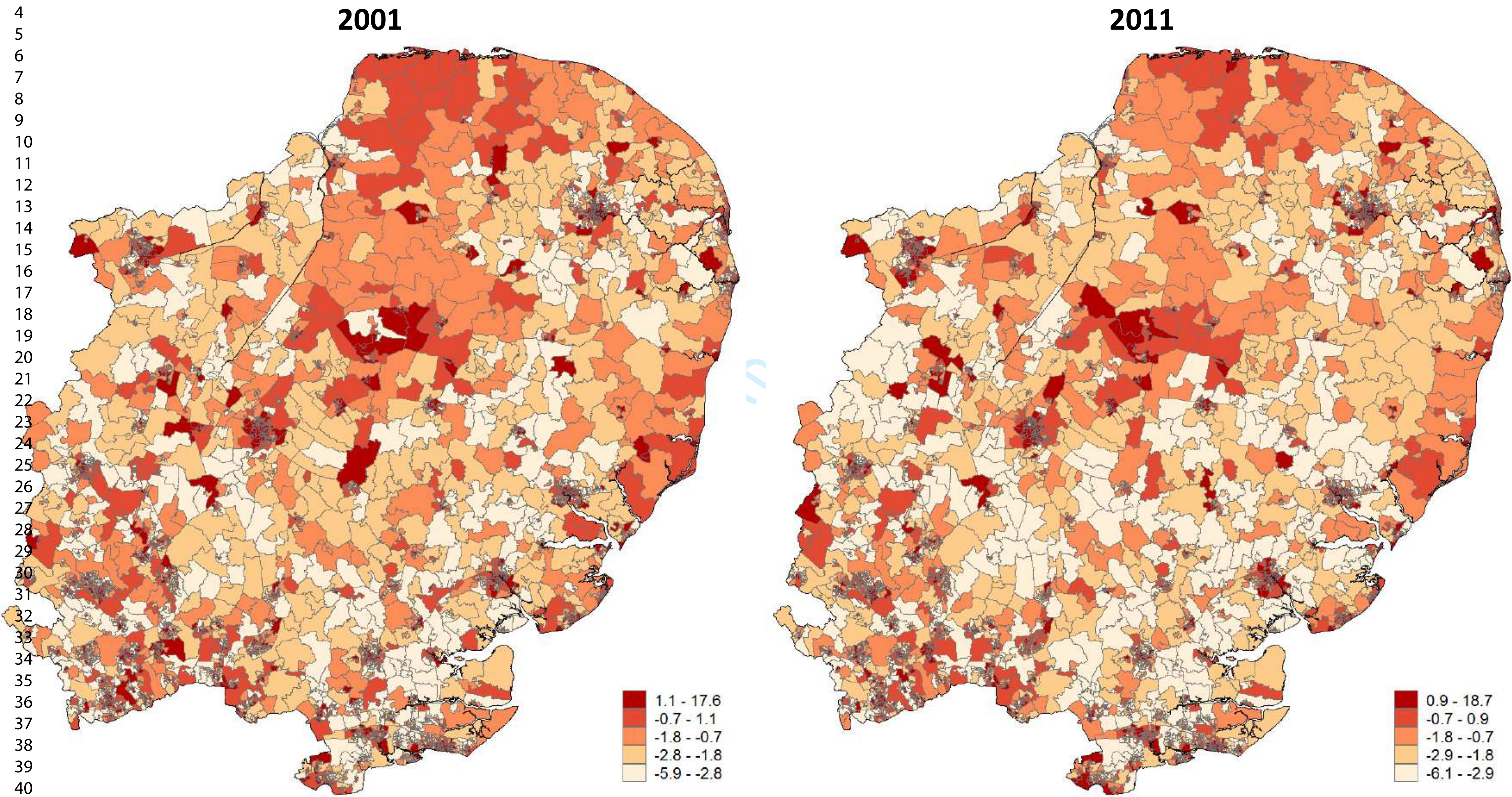

London, Social Fragmentation

2001

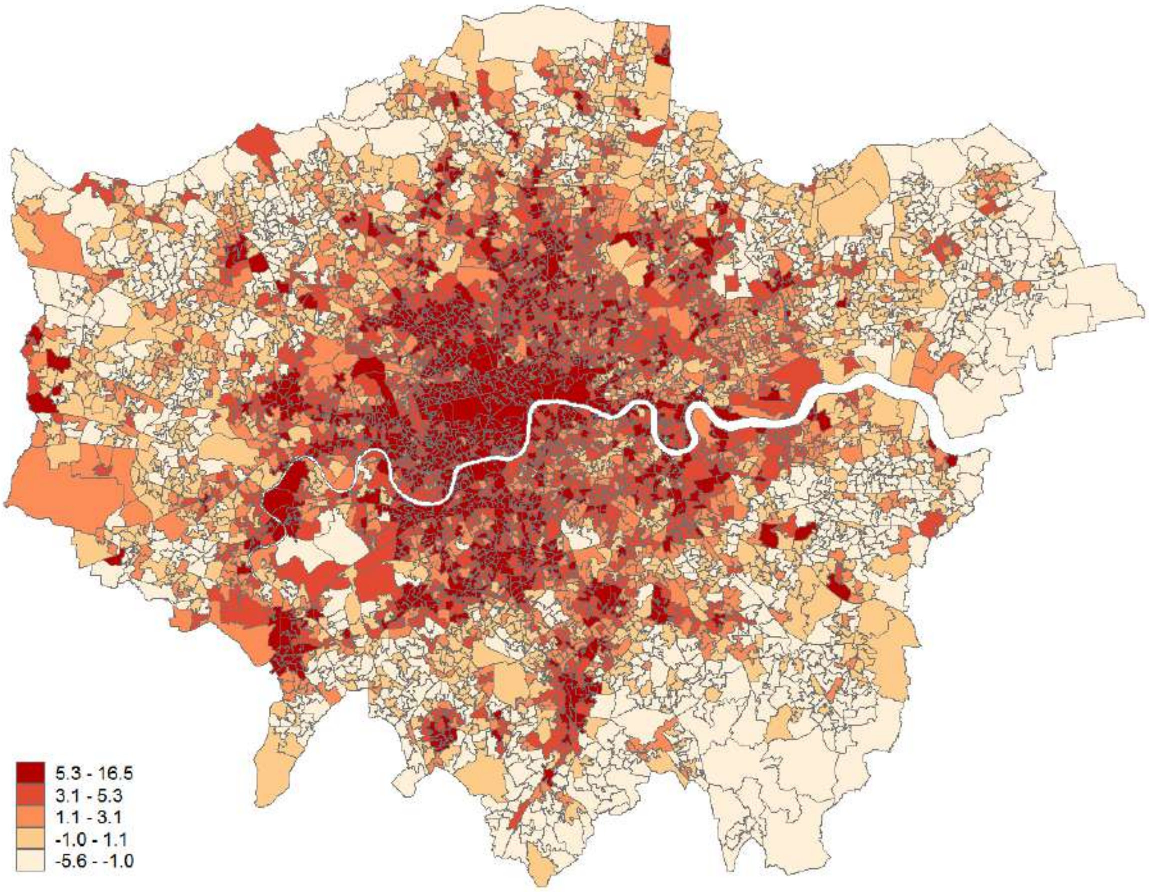

2011

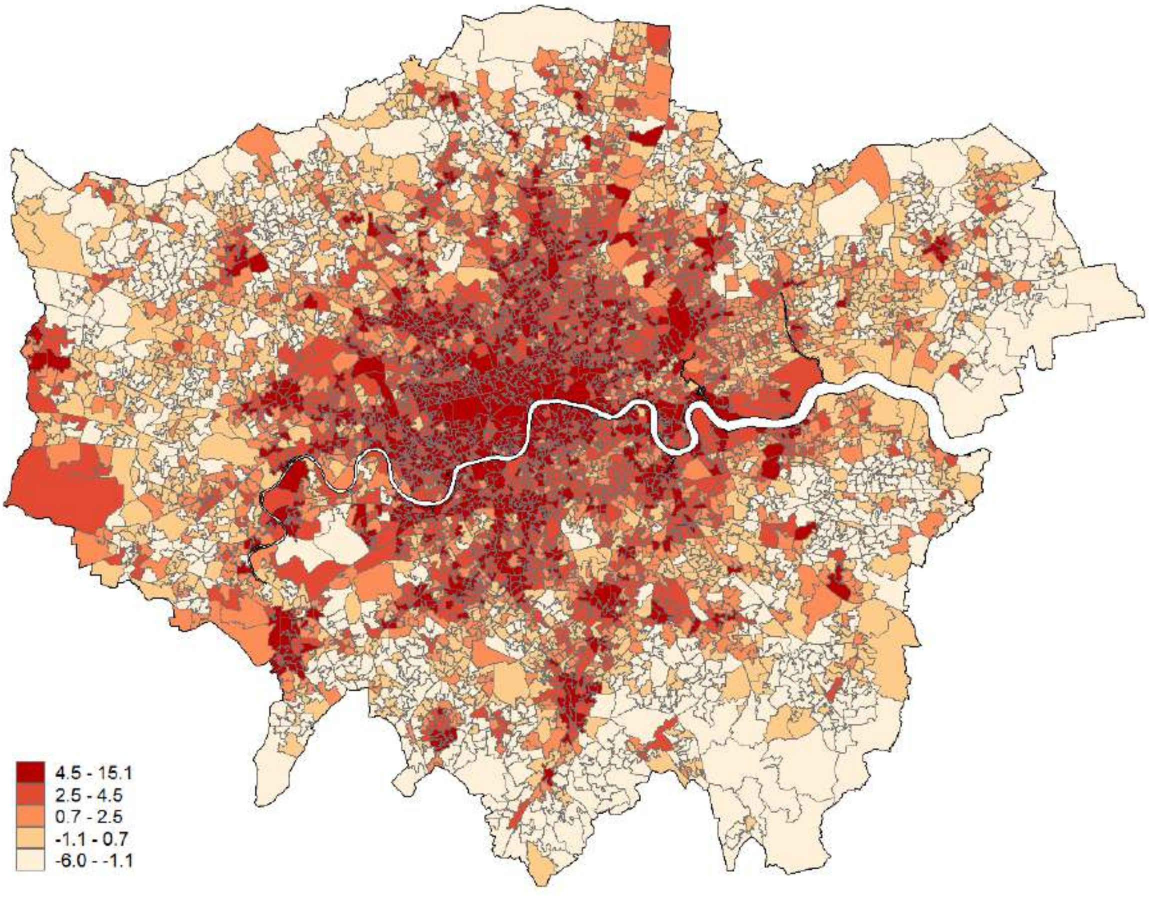

South East Coast, Social Fragmentation

2001

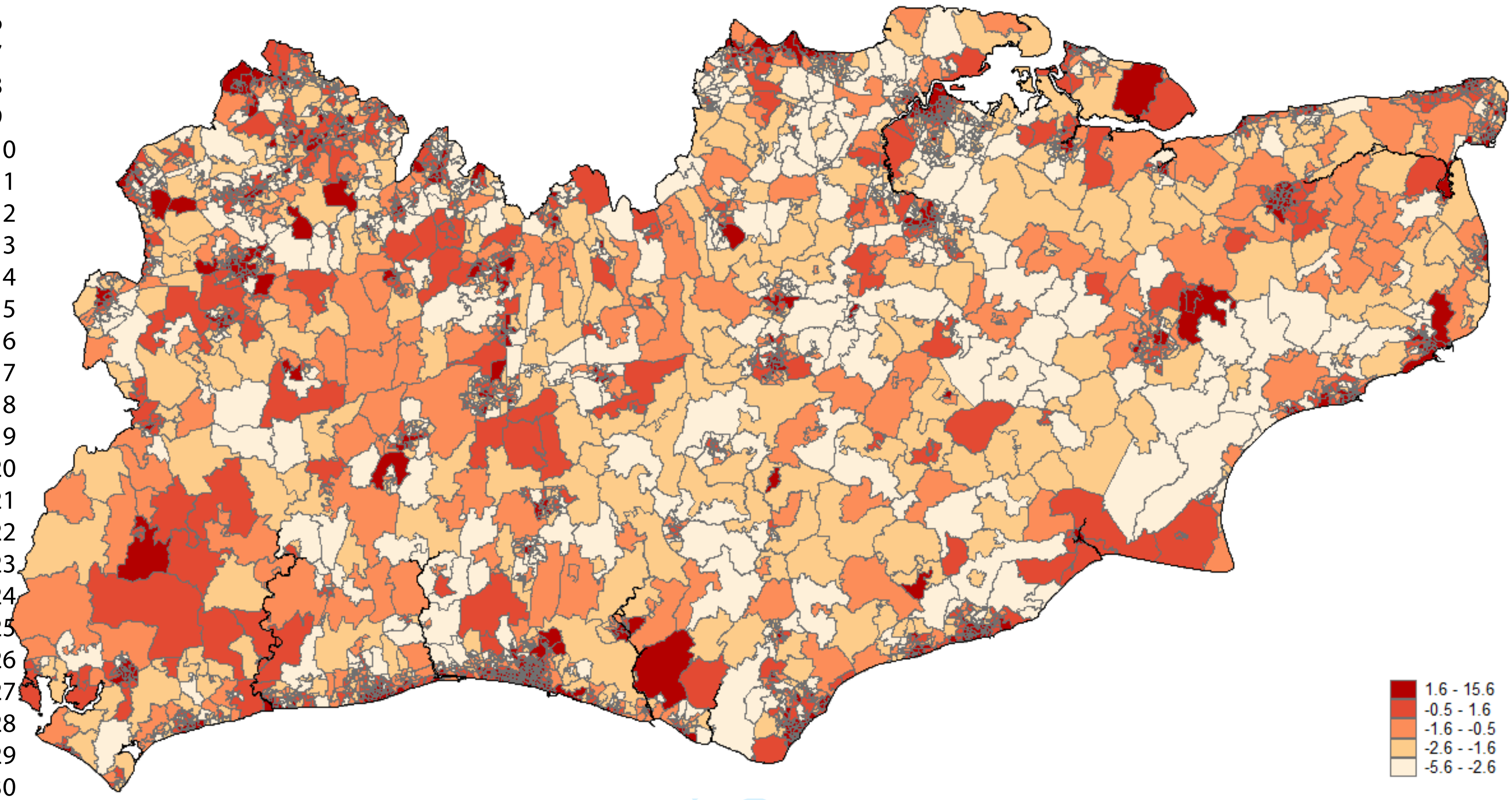

2011

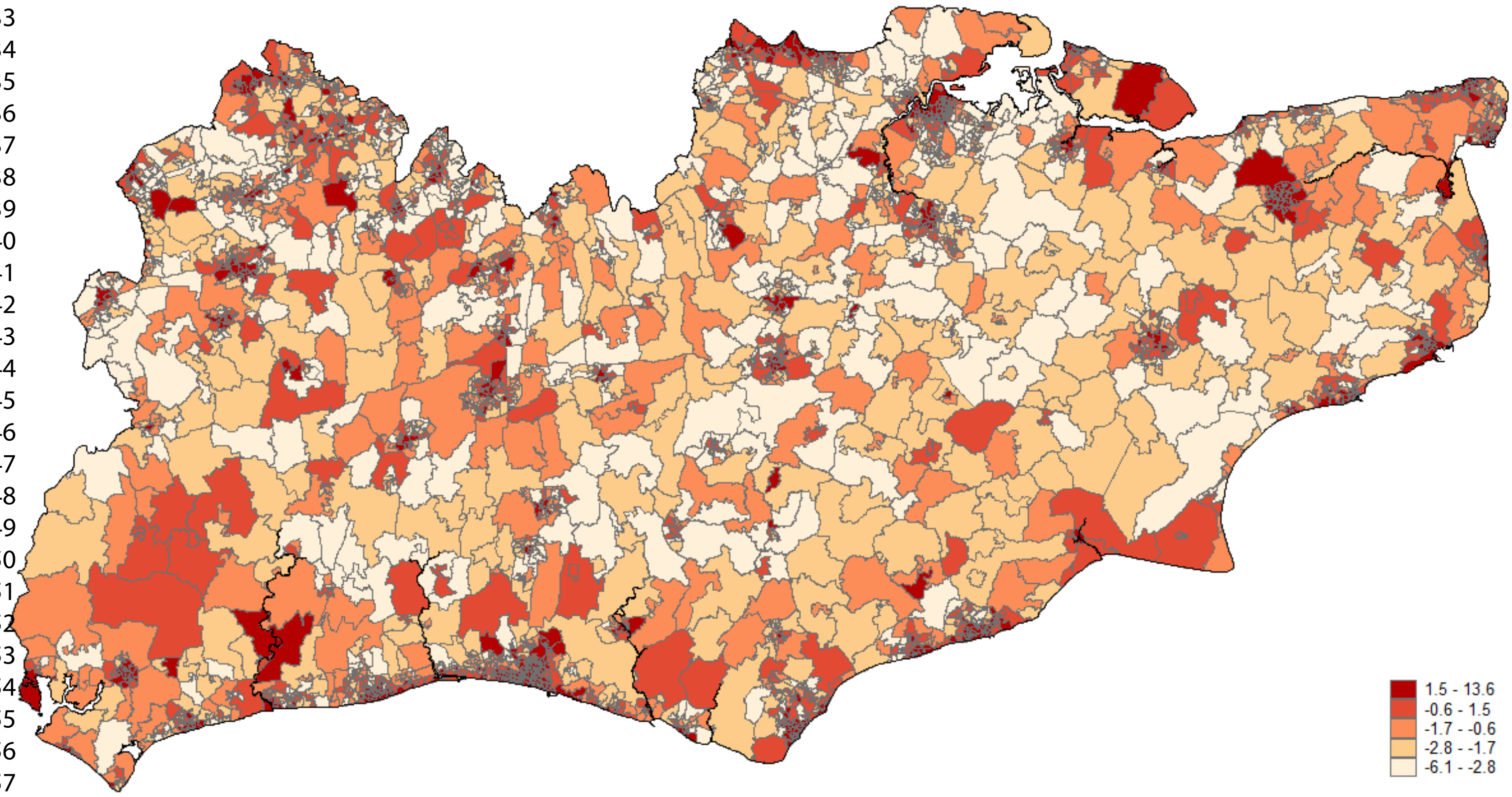

South Central, Social Fragmentation

2001 **2011**

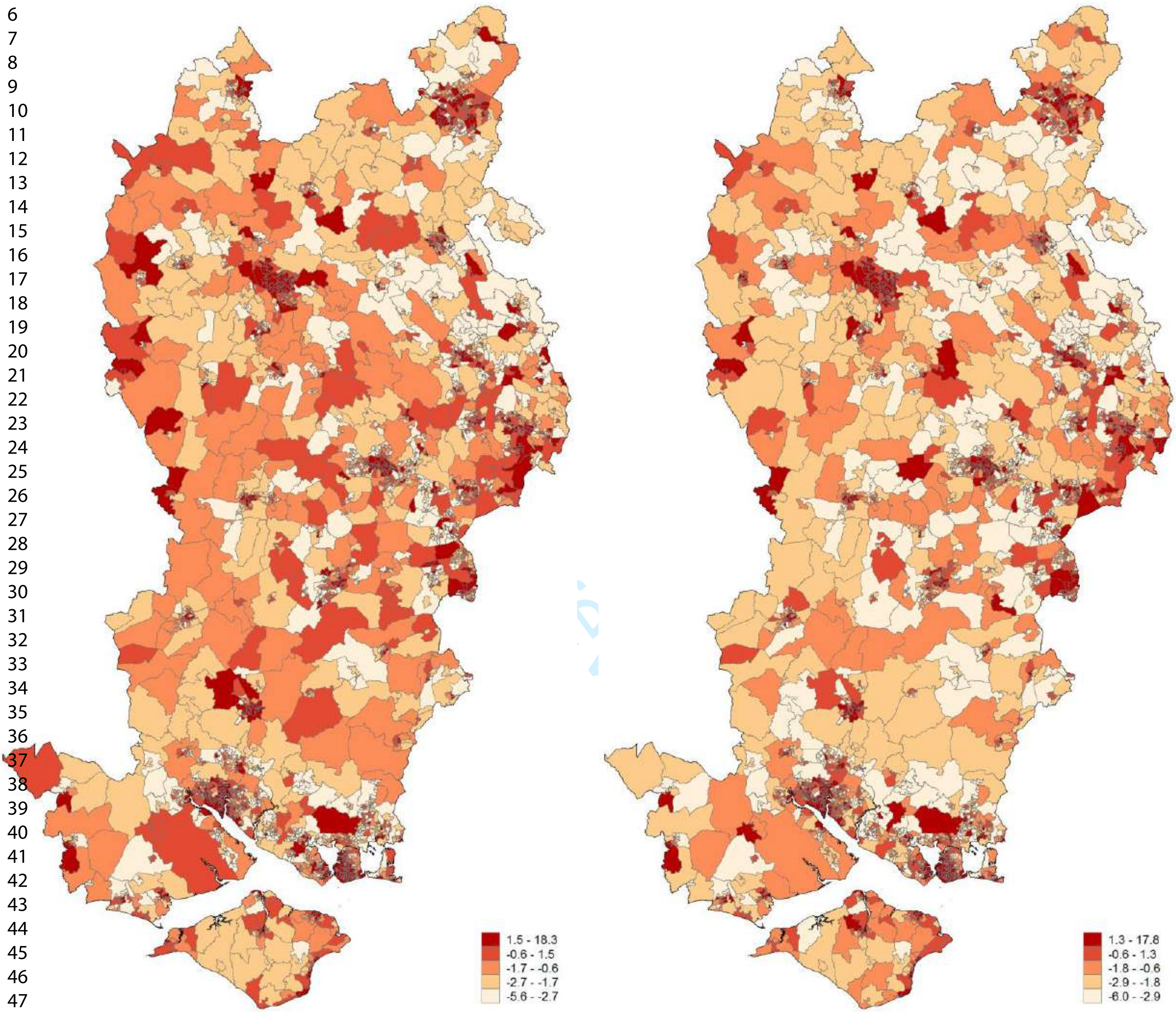

South West, Social Fragmentation

2001

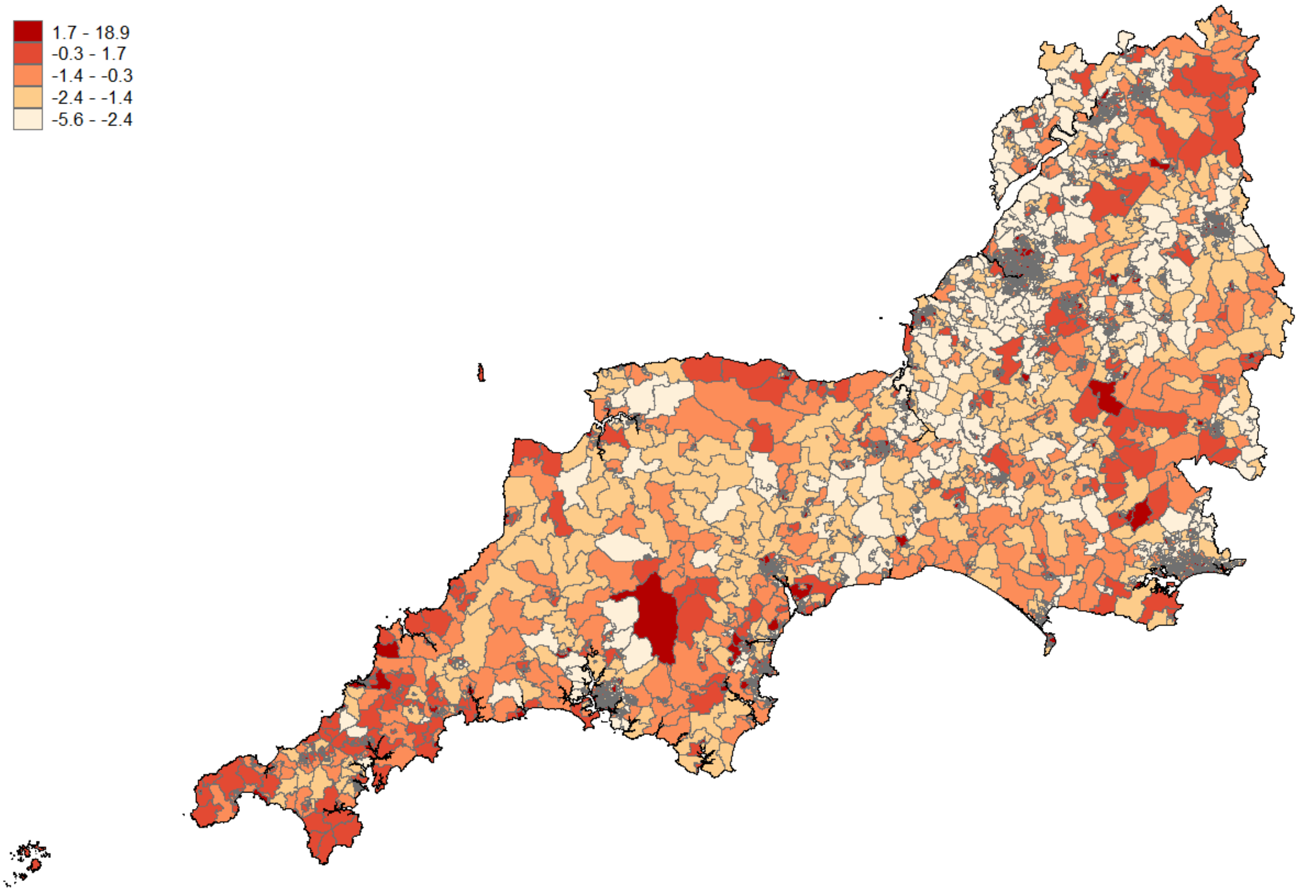

2011

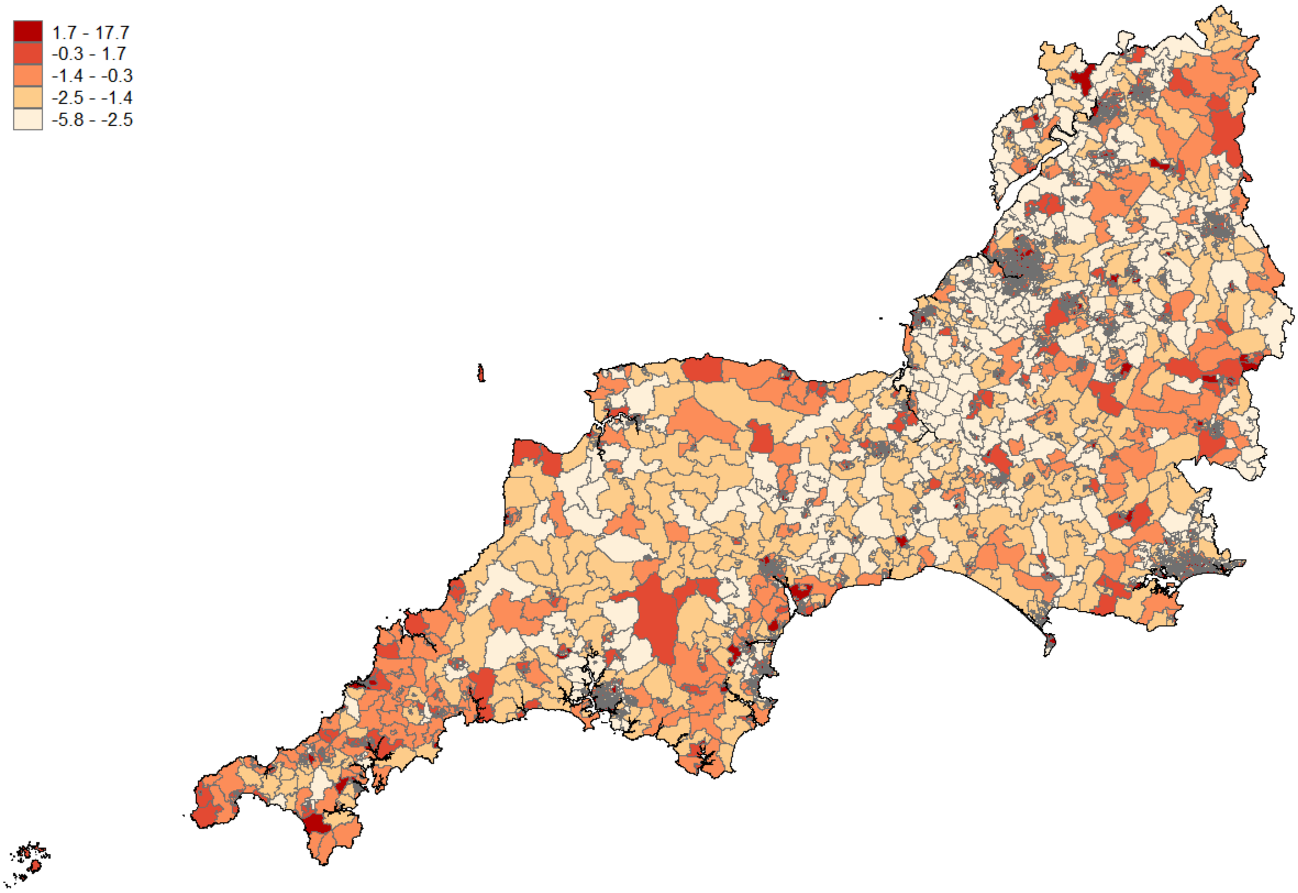

Single People

England 2001, Single People % of population

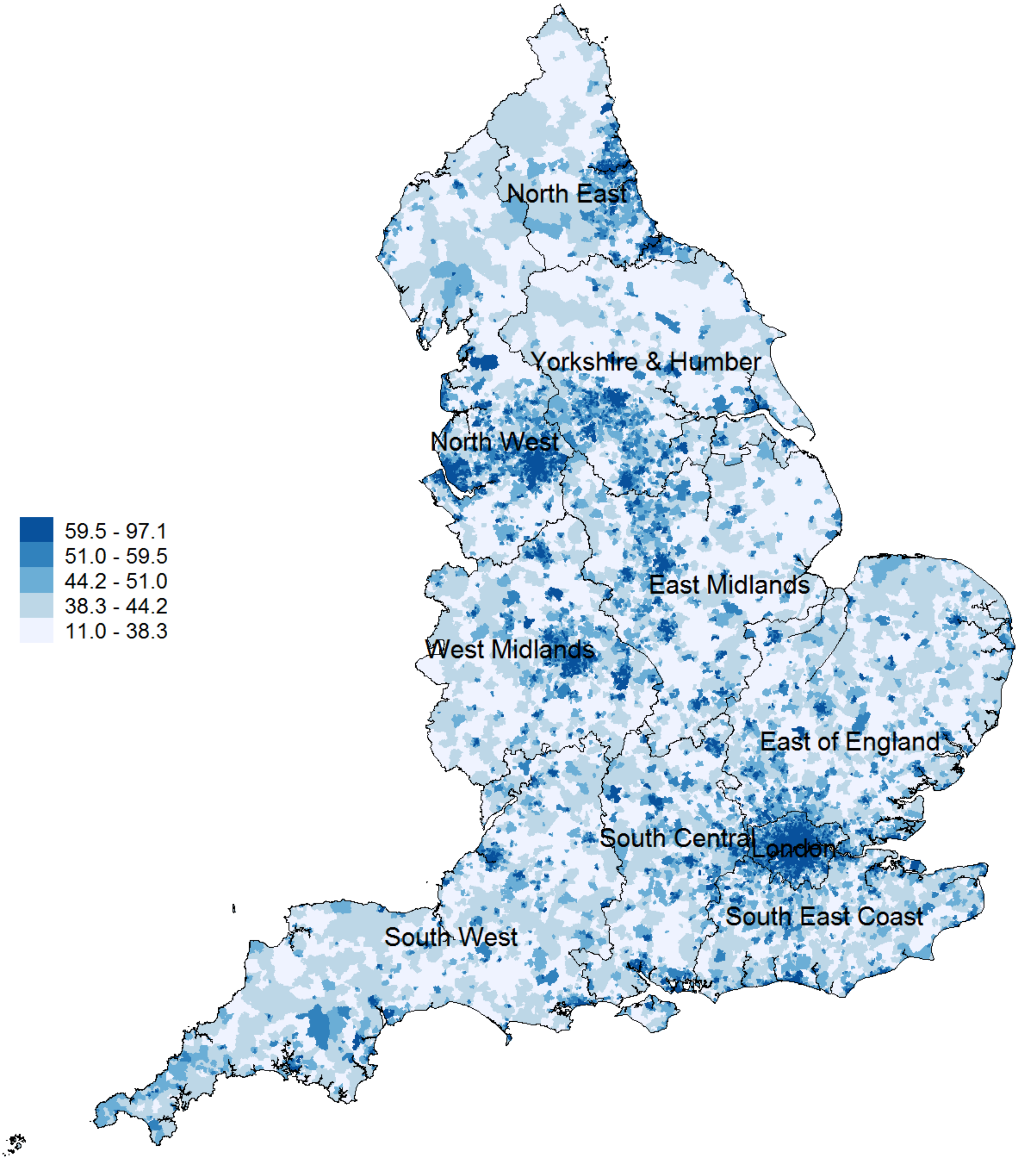

England 2011, Single People % of population

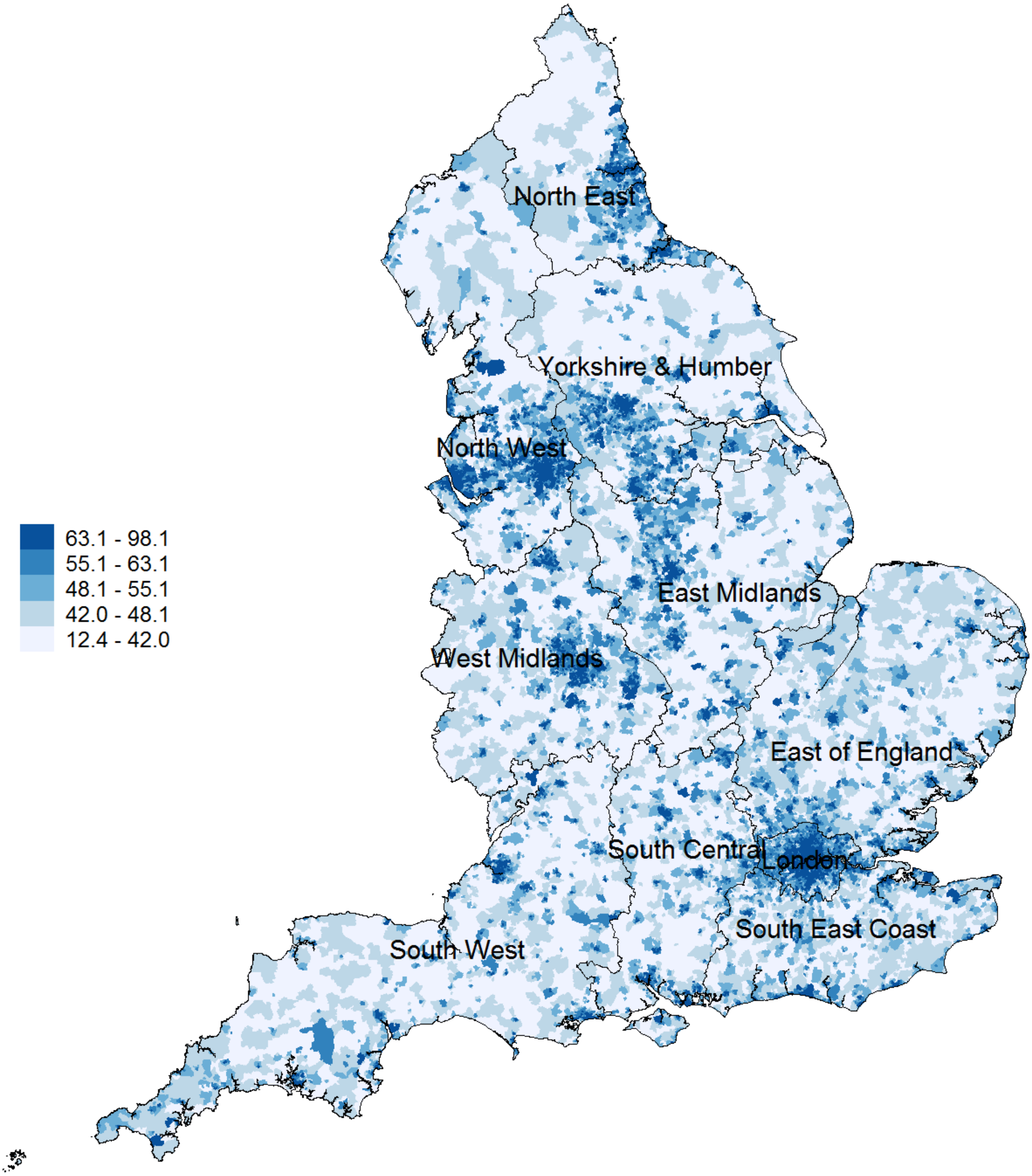

North East, Single People % of population

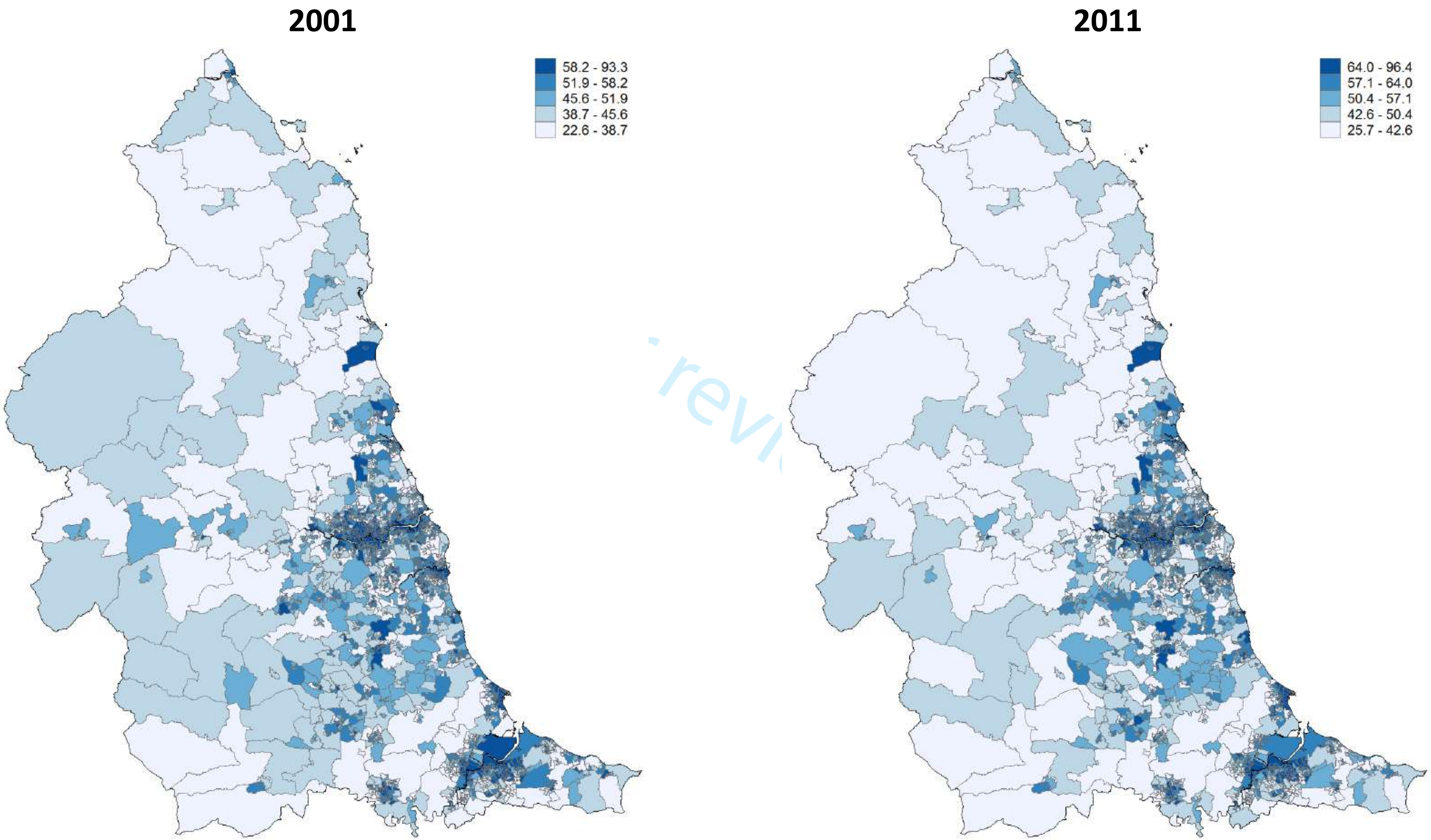

North West, Single People % of population

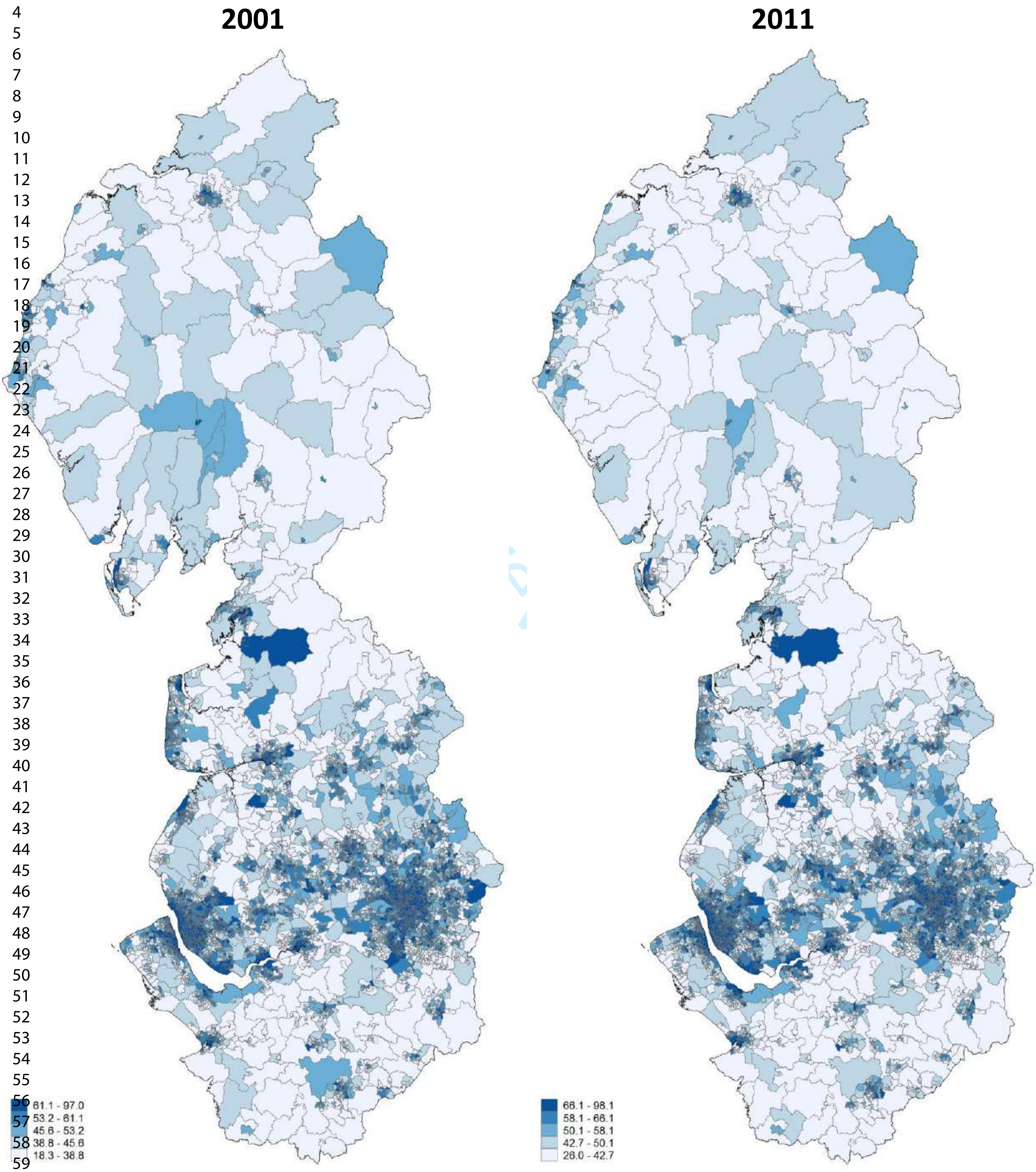

Yorkshire and the Humber, Single People % of population

2001

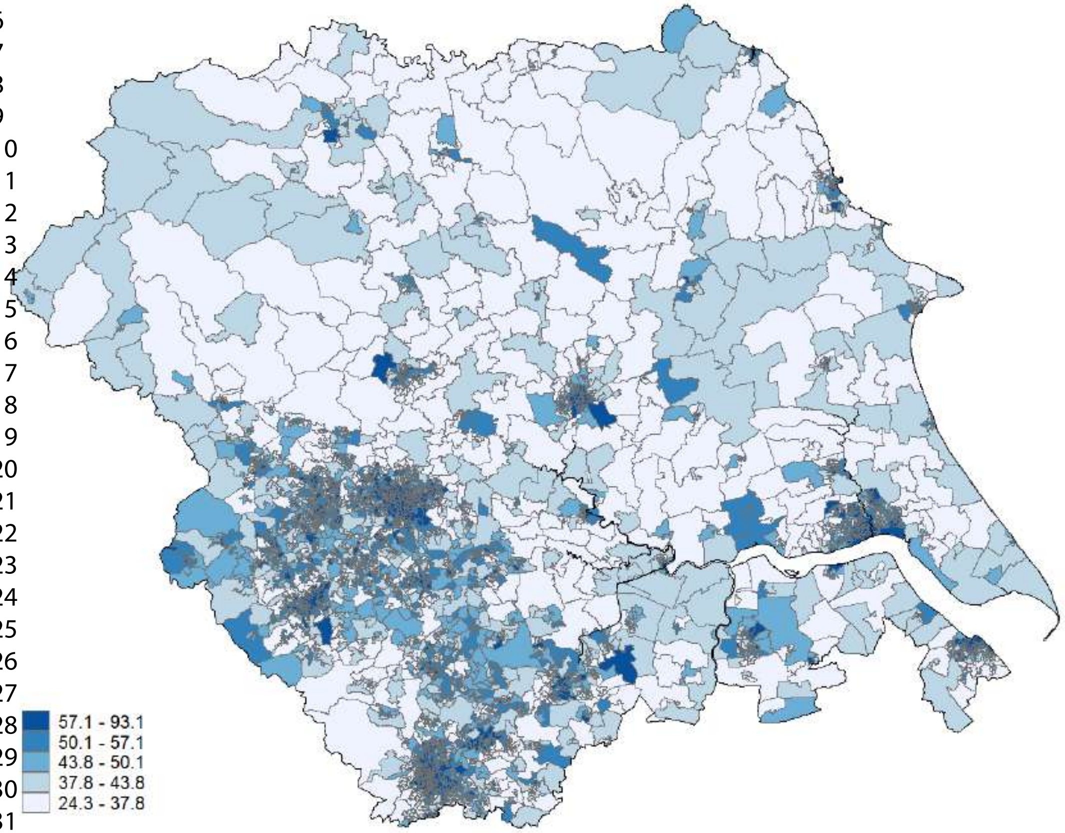

2011

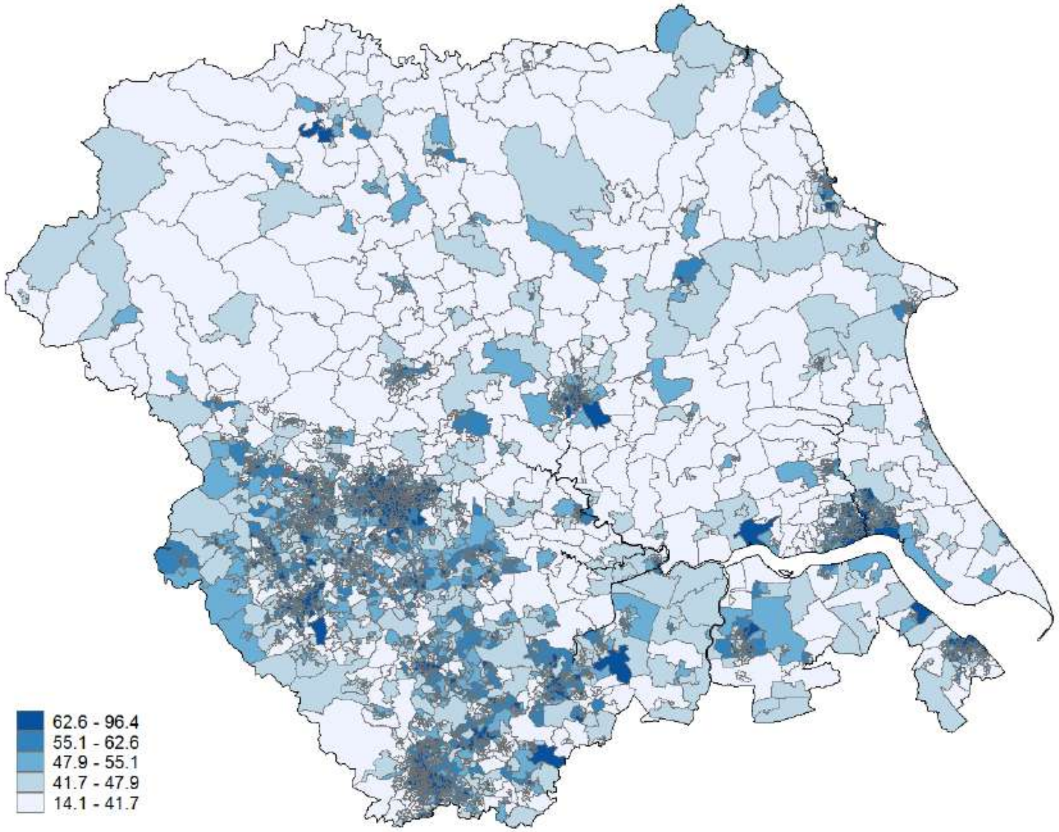

East Midlands, Single People % of population

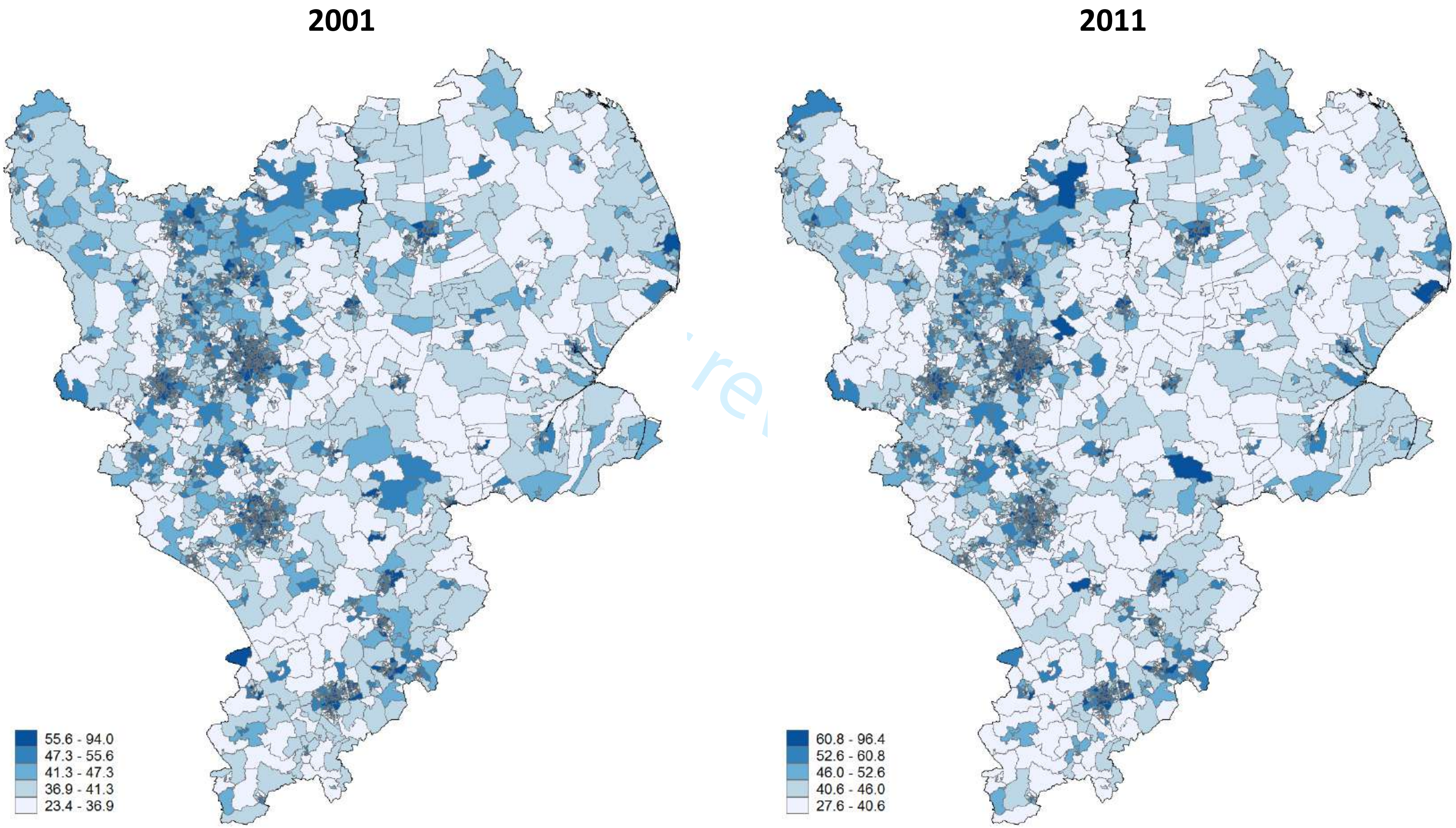

West Midlands, Single People % of population

2001

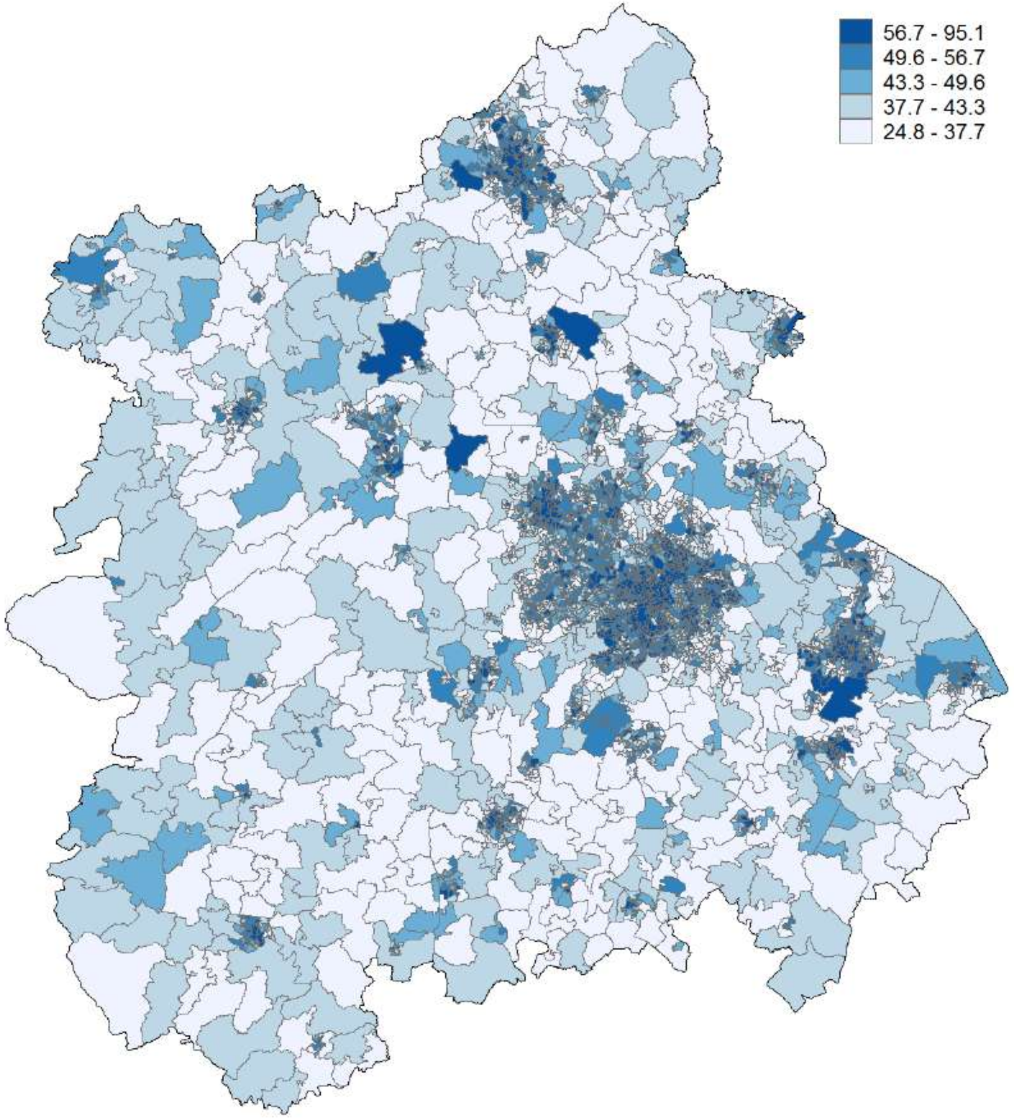

2011

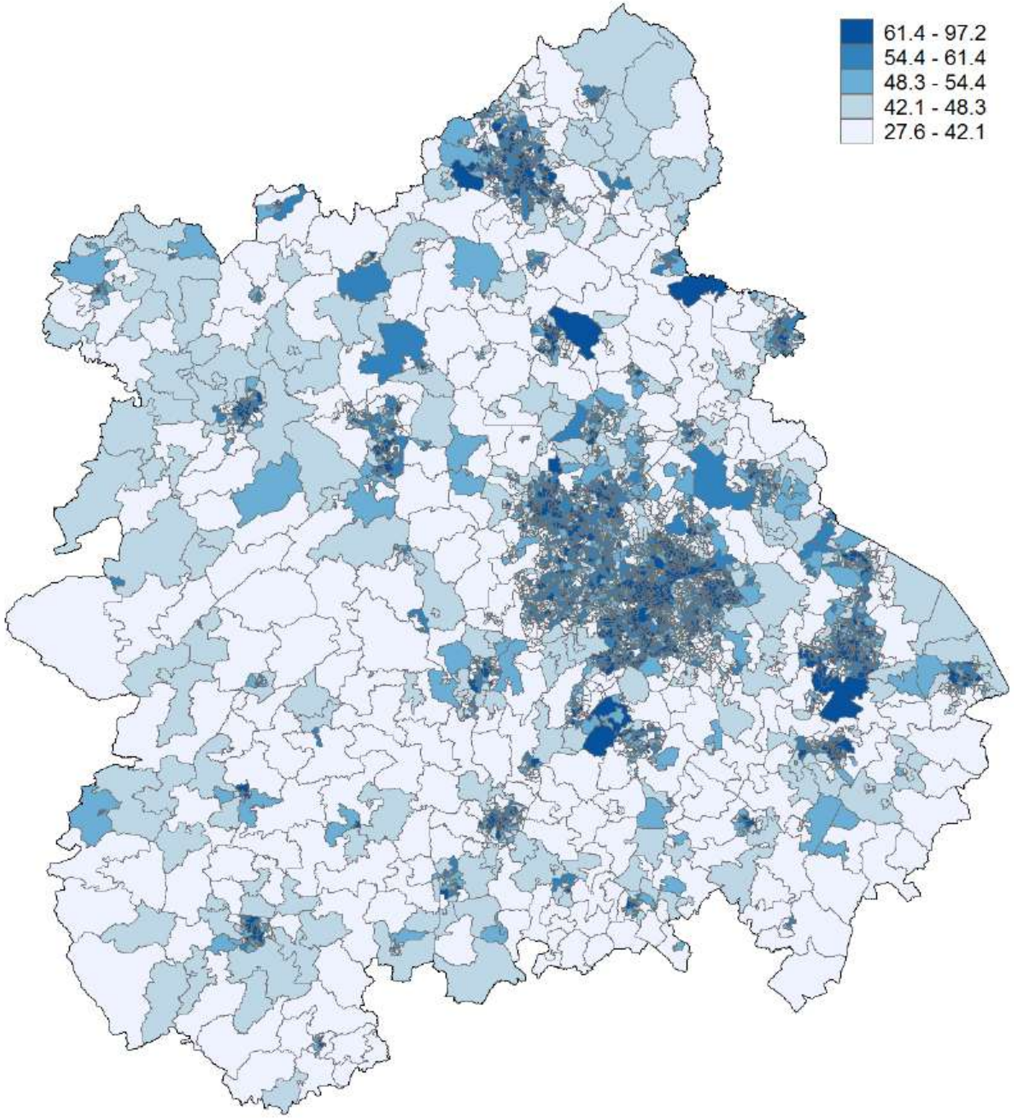

East of England, Single People % of population

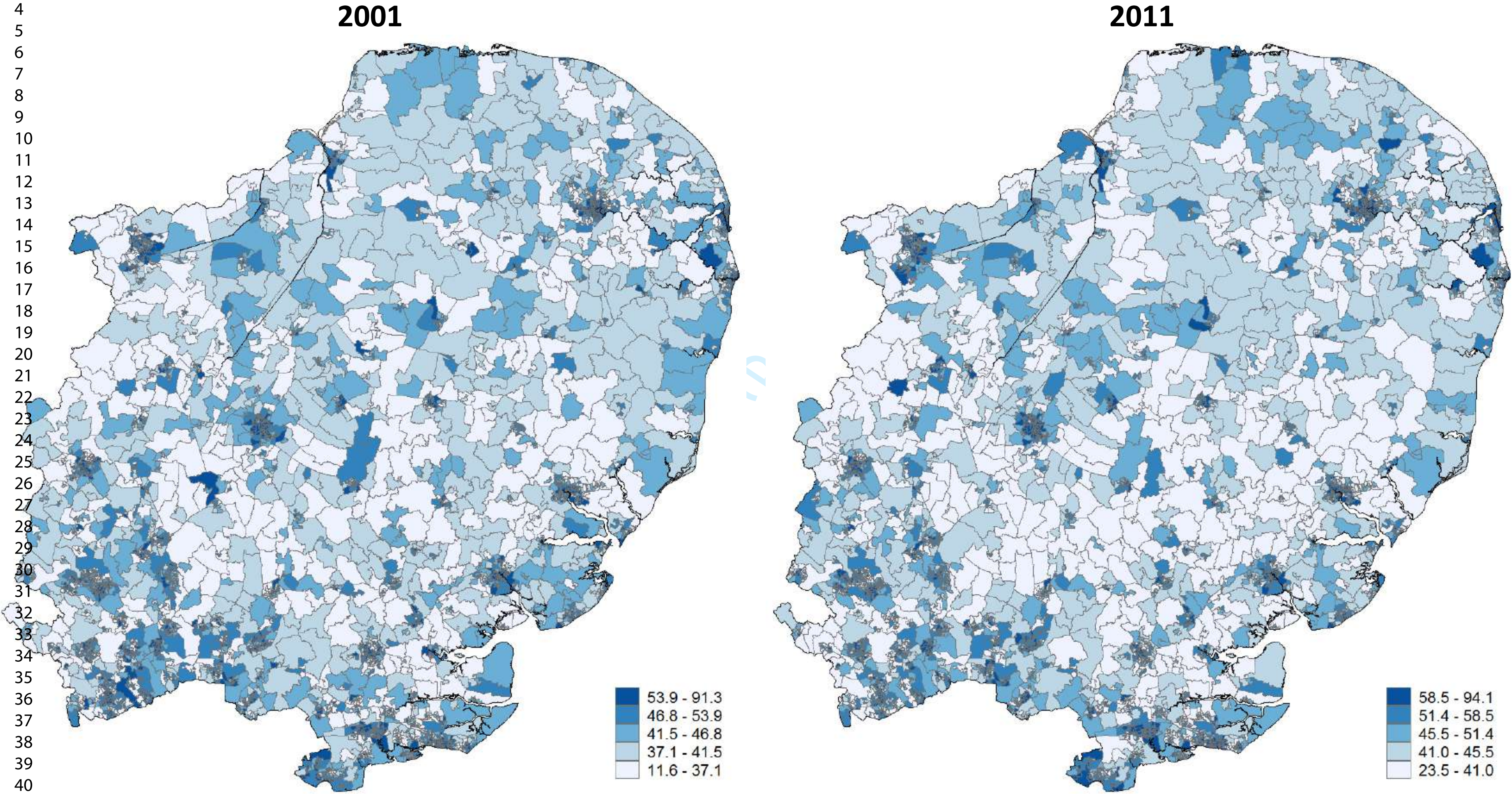

London, Single People % of population

2001

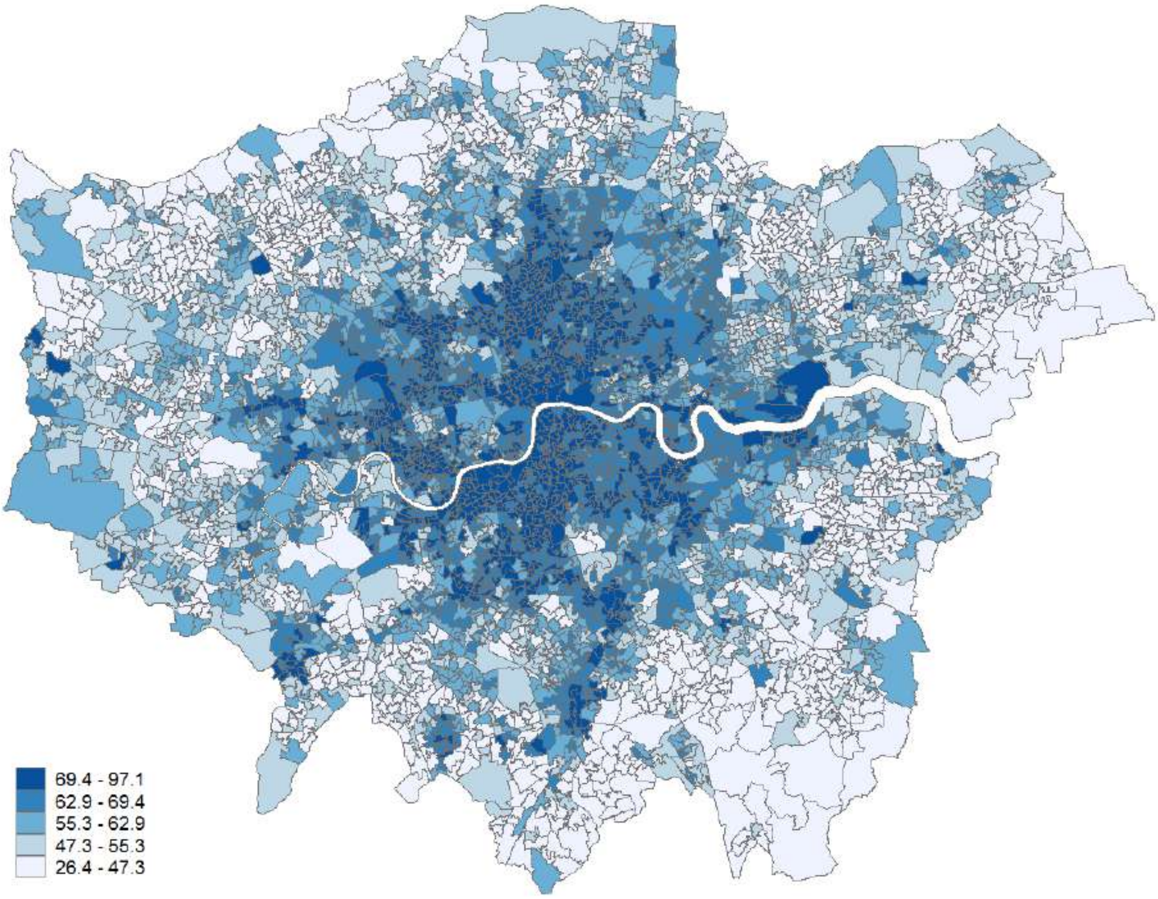

2011

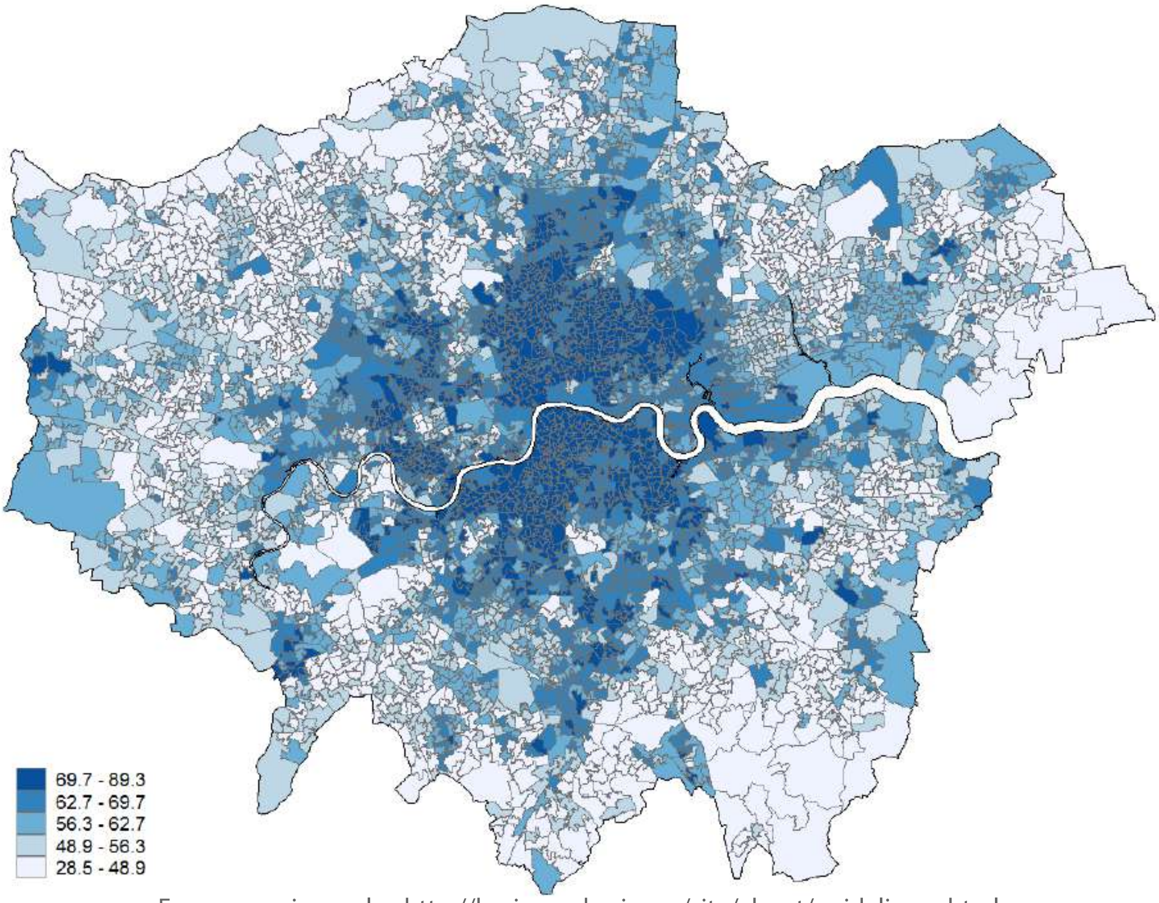

South East Coast, Single People % of population

2001

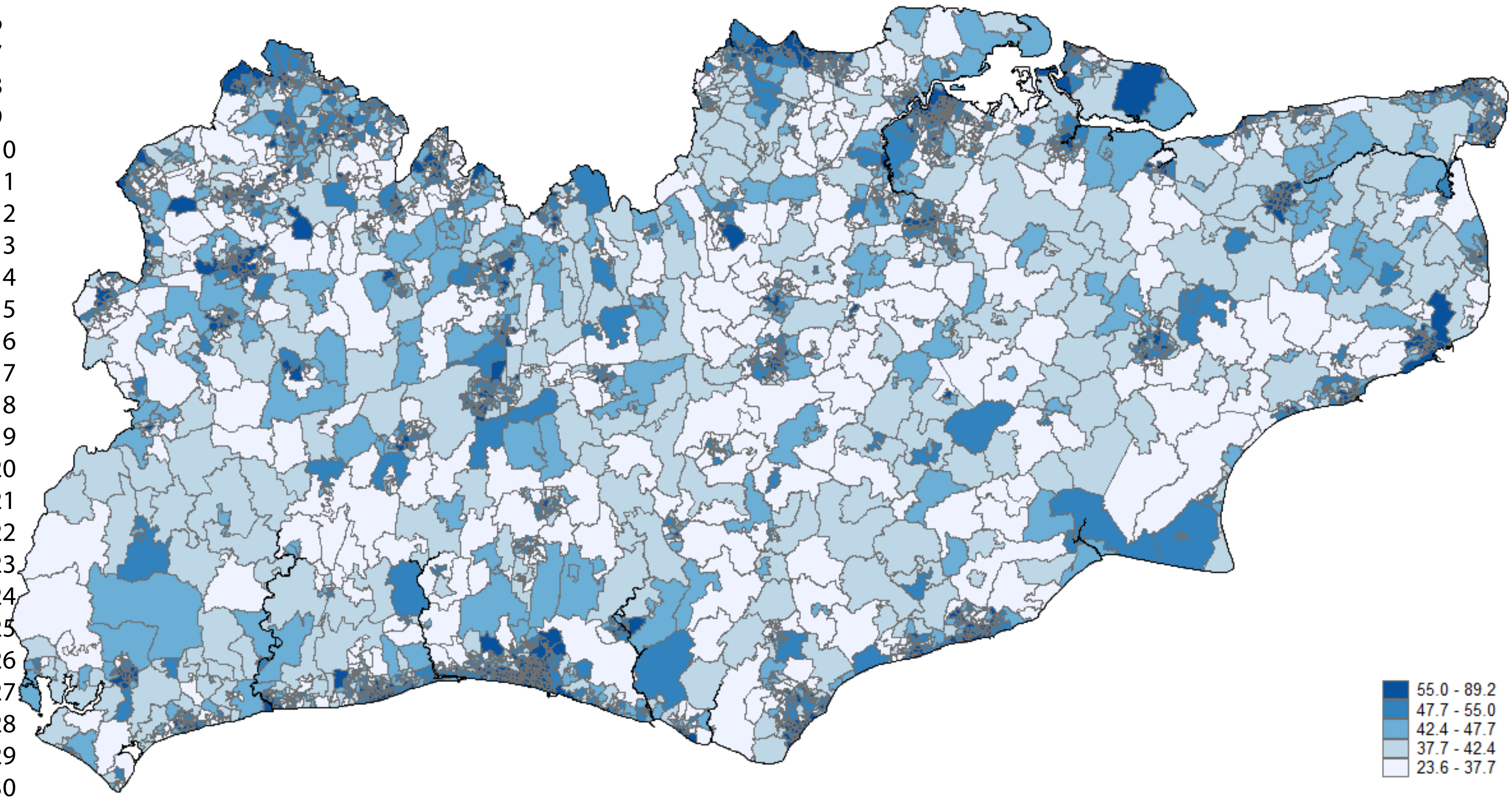

2011

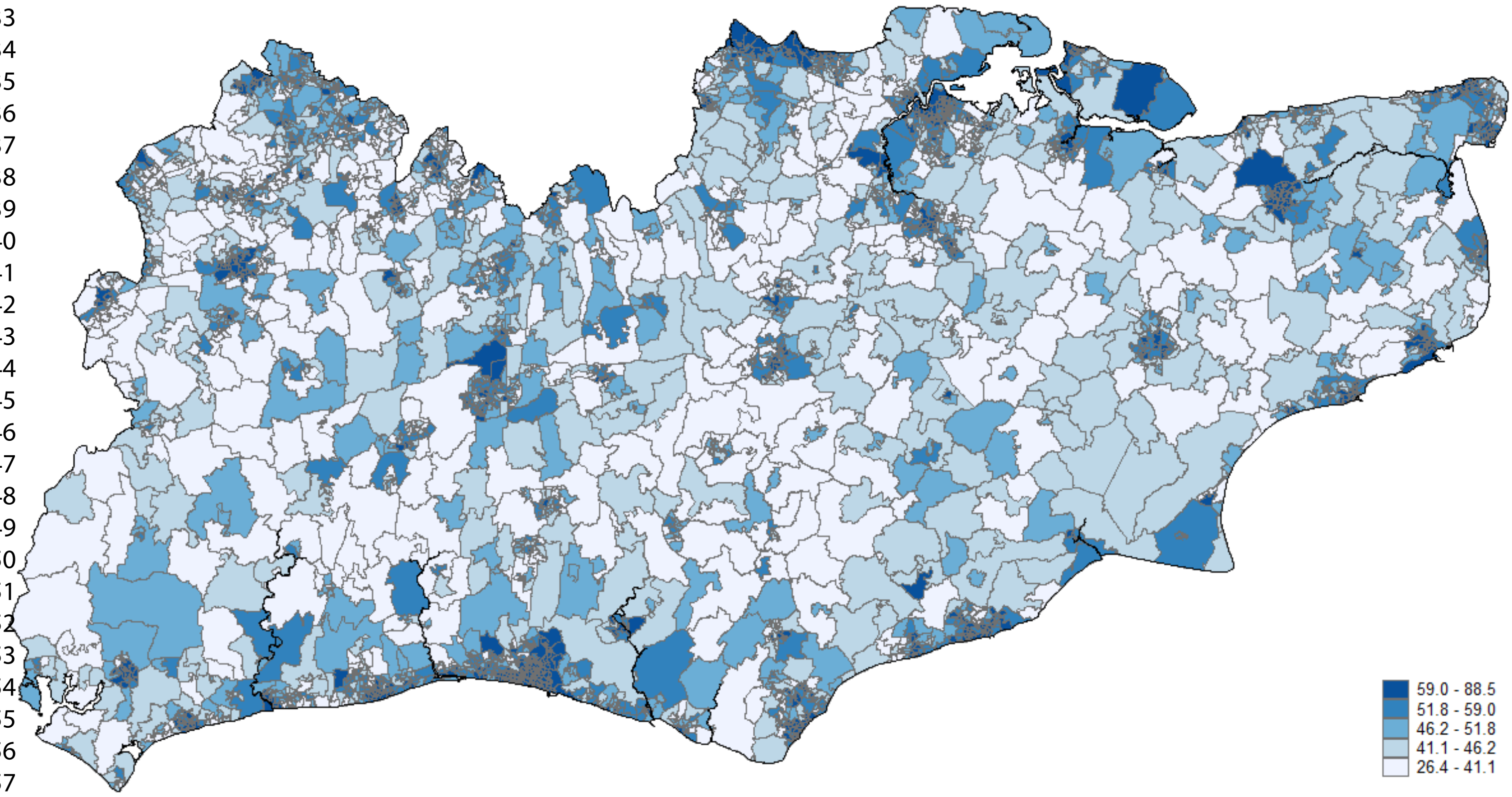

South Central, Single People % of population

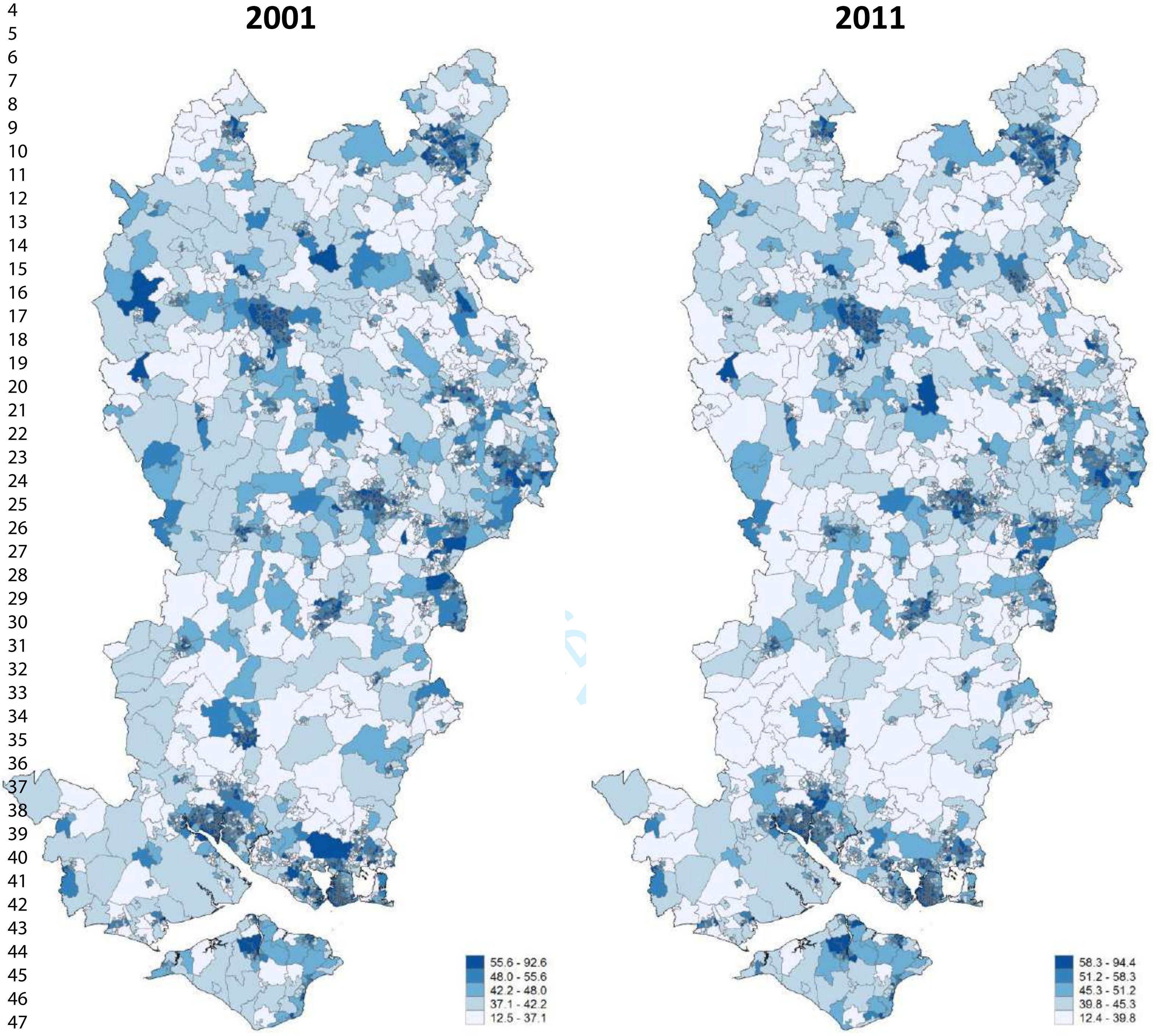

South West, Single People % of population

2001

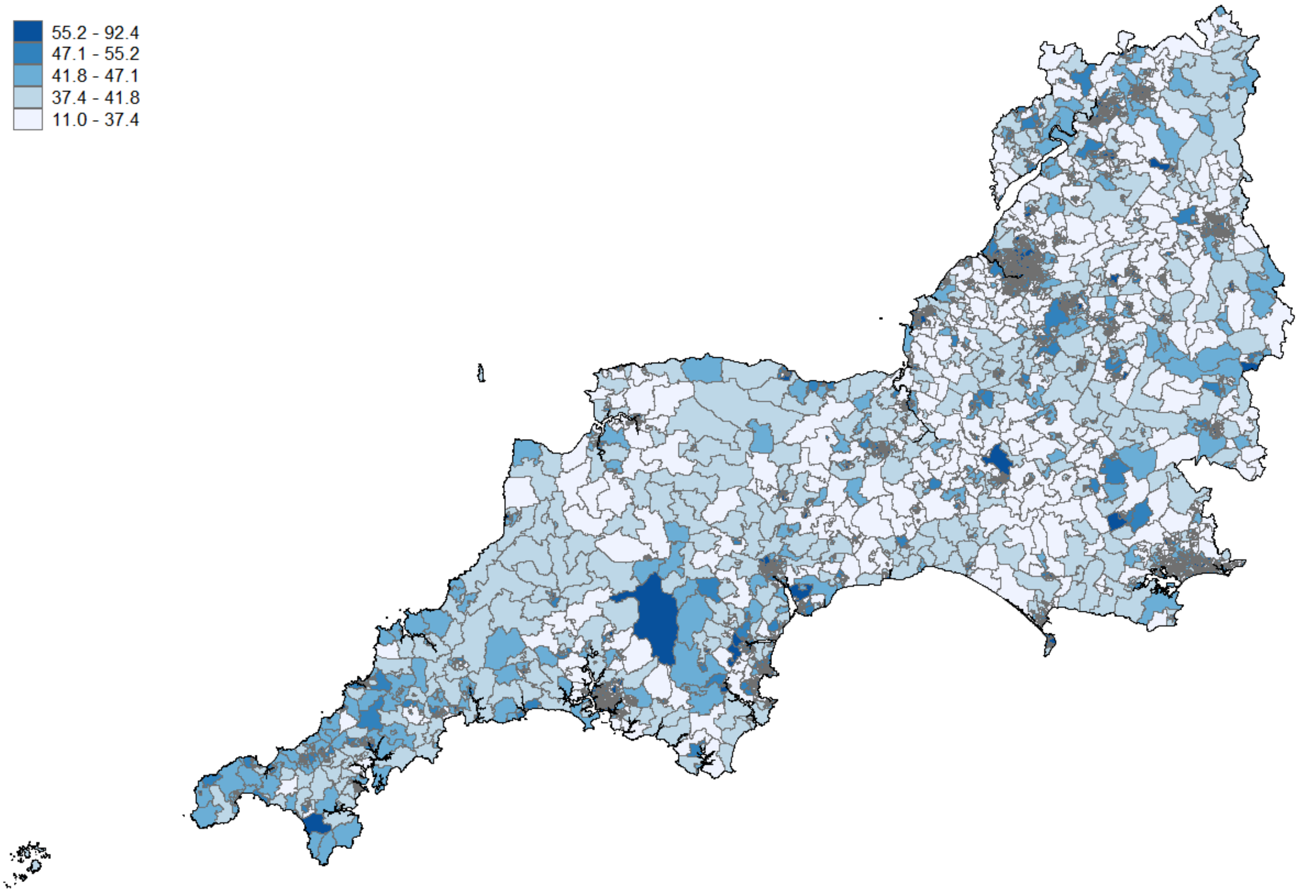

2011

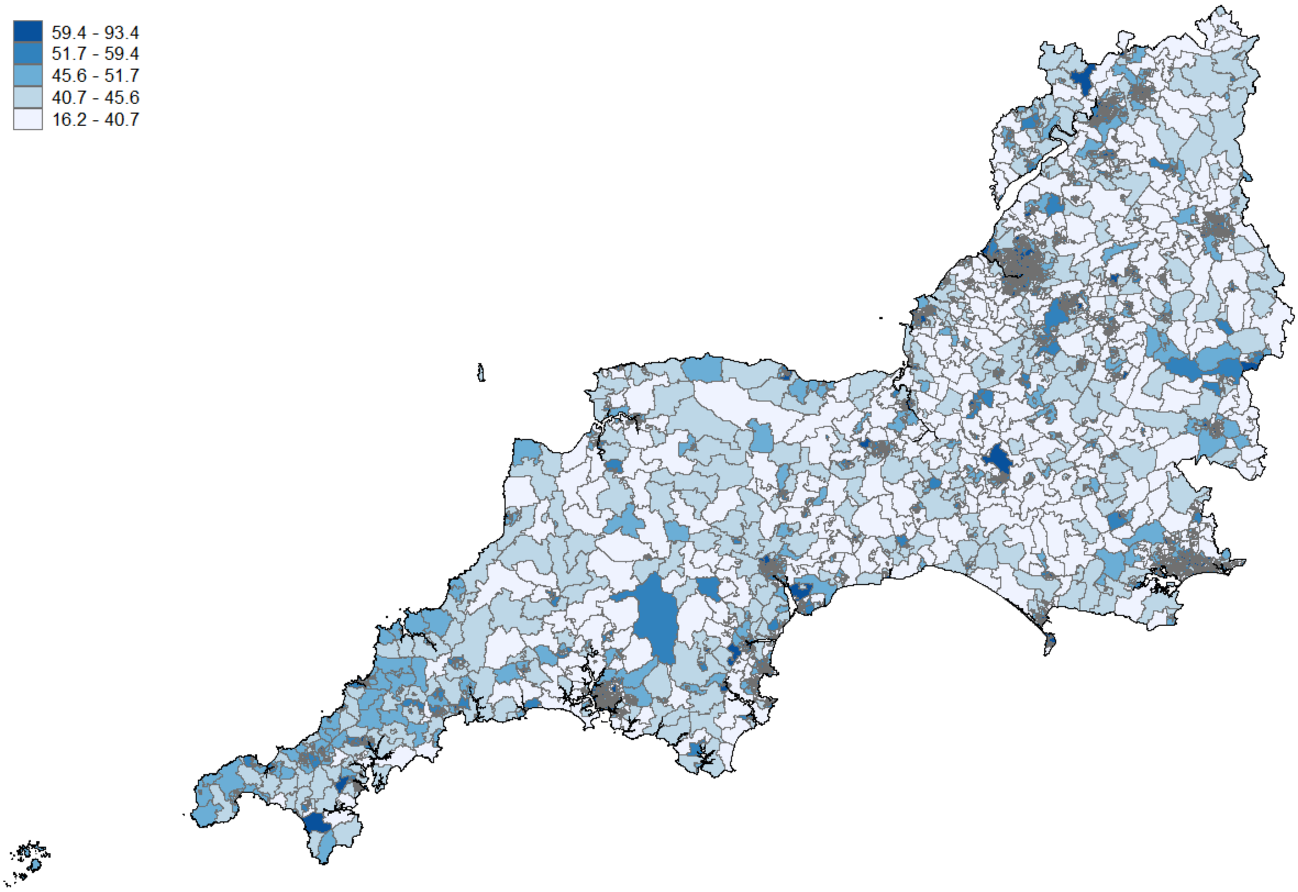

One Person Households

England 2001, One Person Households % of all households

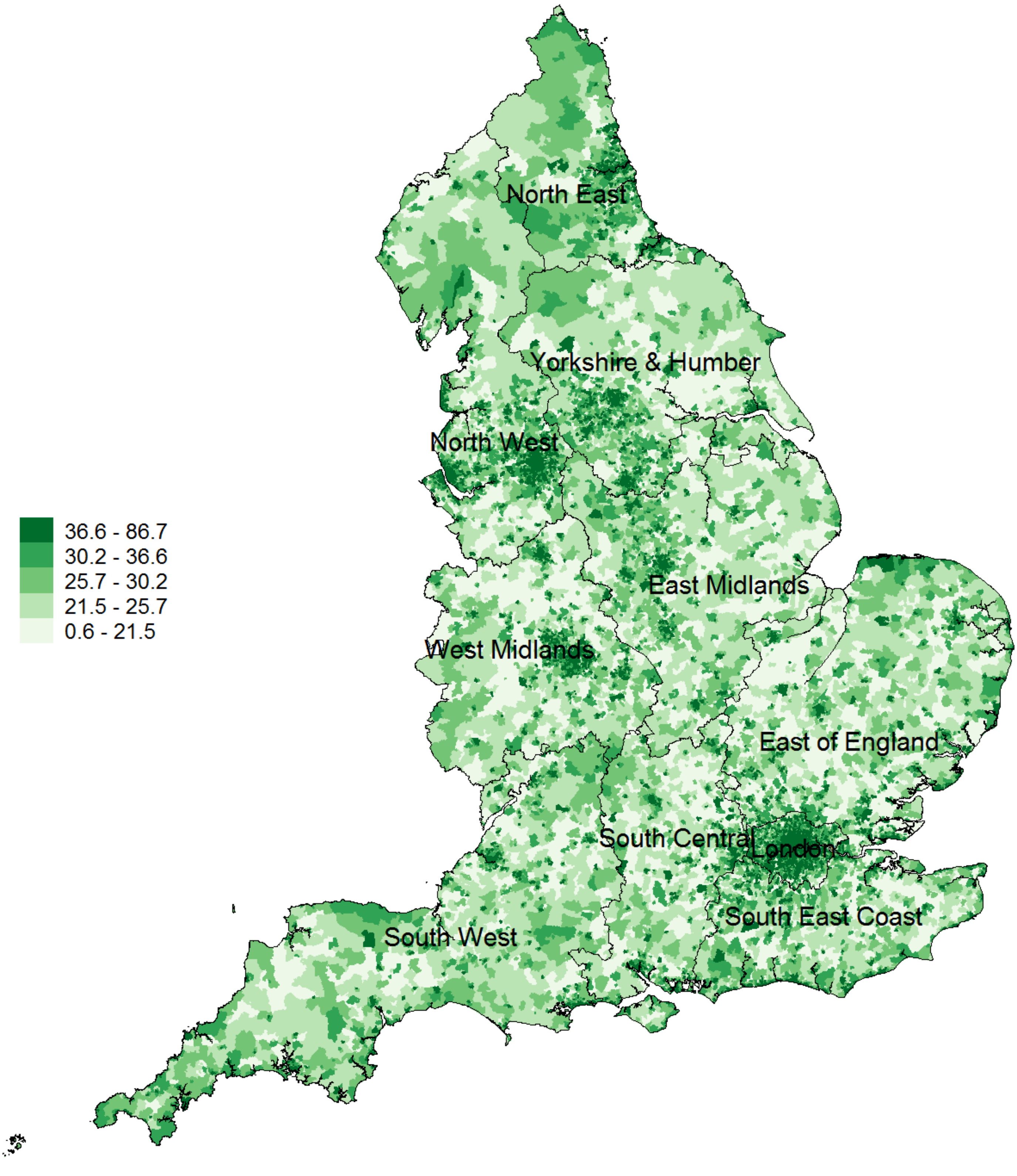

England 2011, One Person Households % of all households

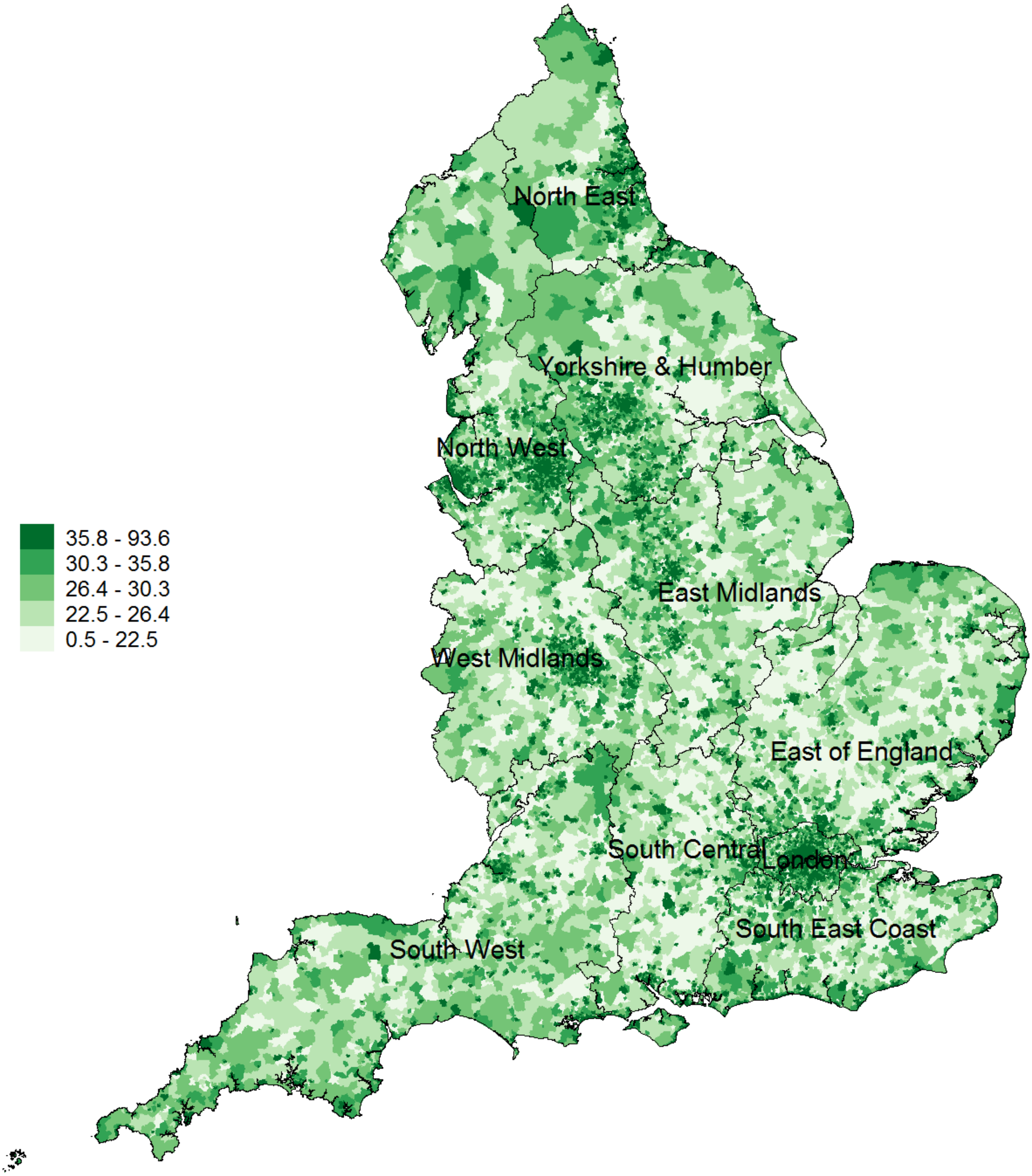

North East, One Person Households % of all households

2001

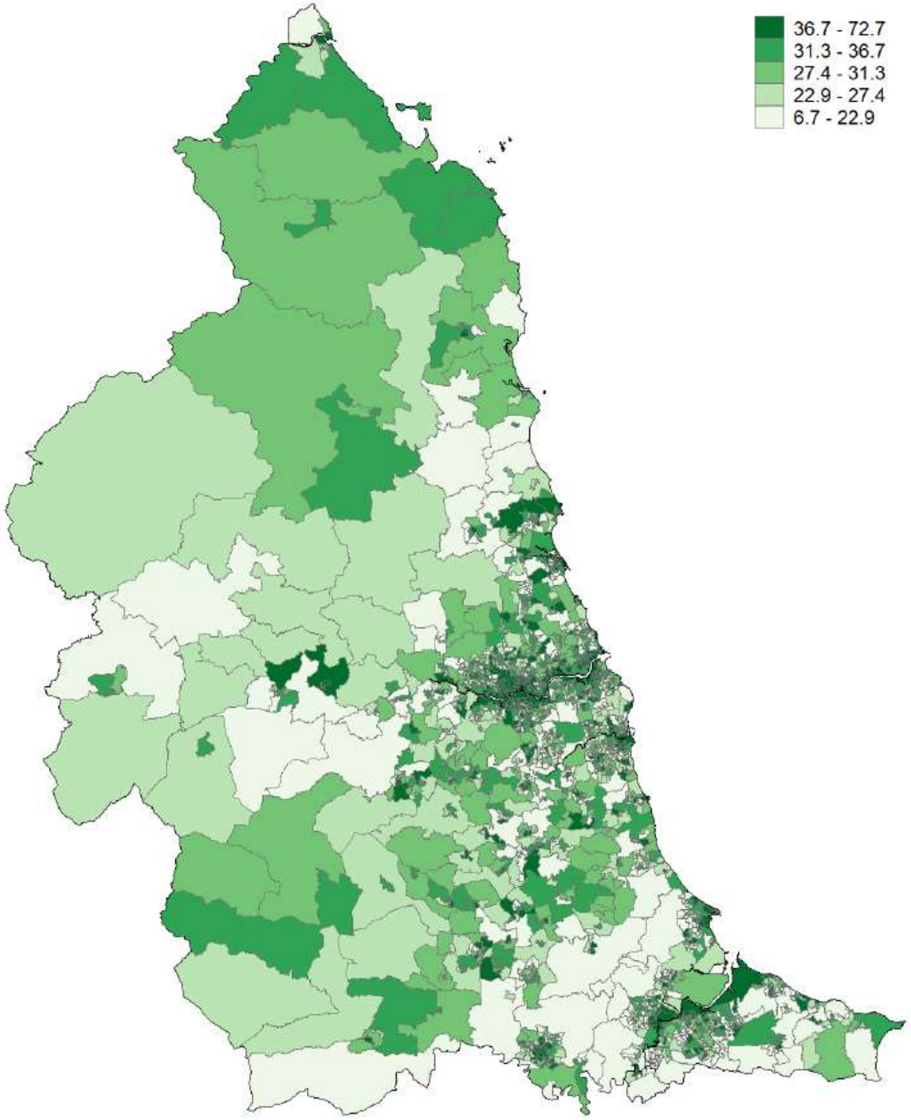

2011

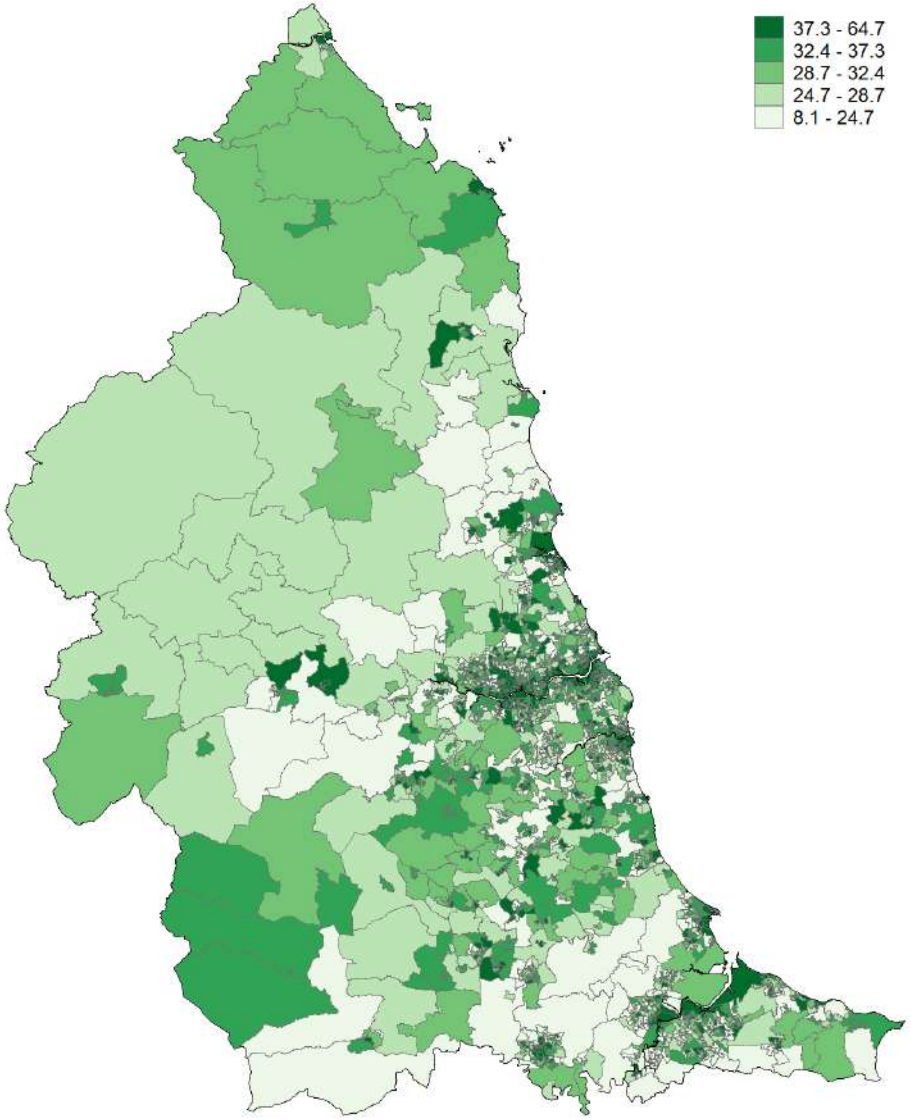

North West, One Person Households % of all households

2001

2011

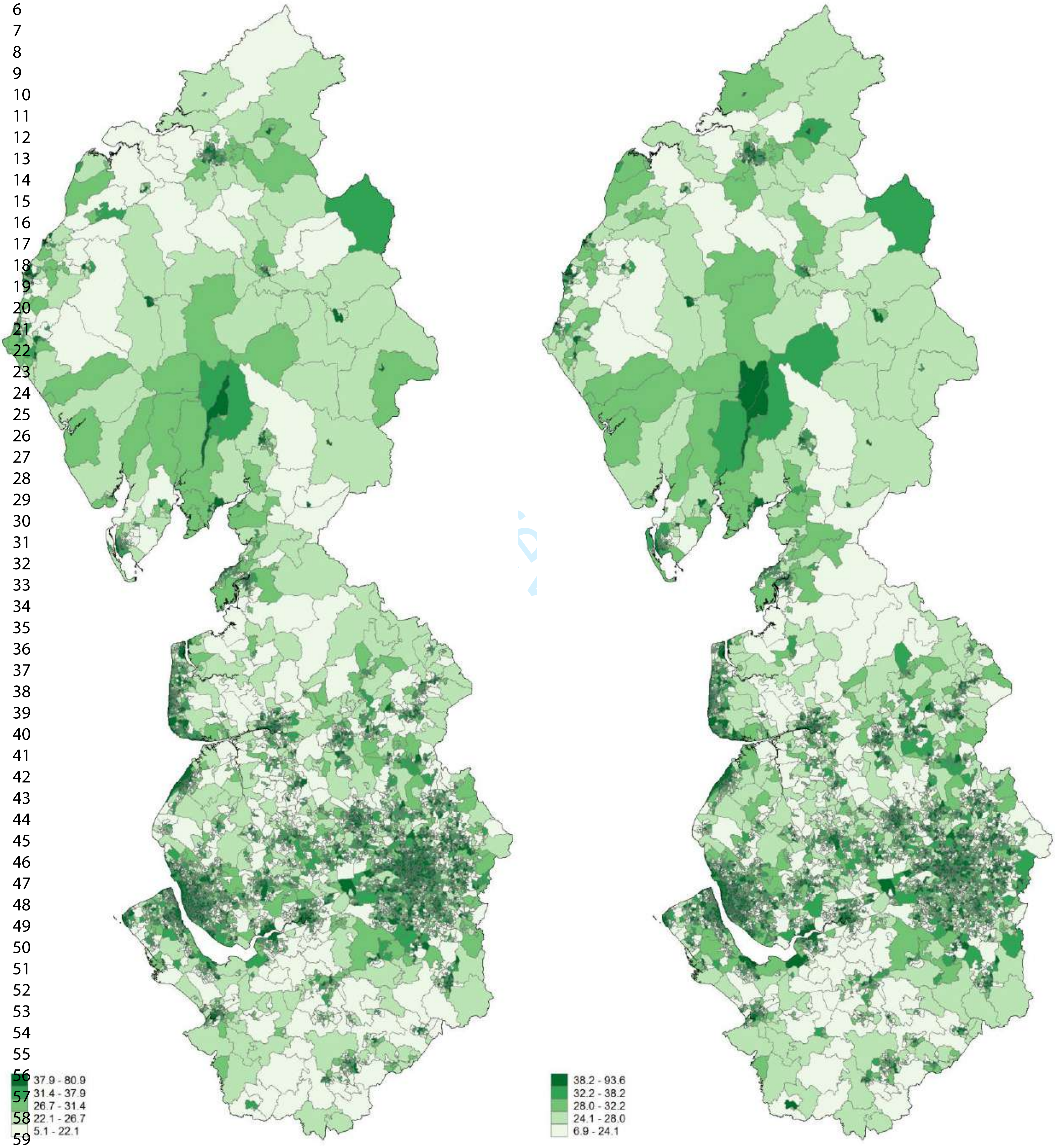

Yorkshire and the Humber, One Person Households % of all households

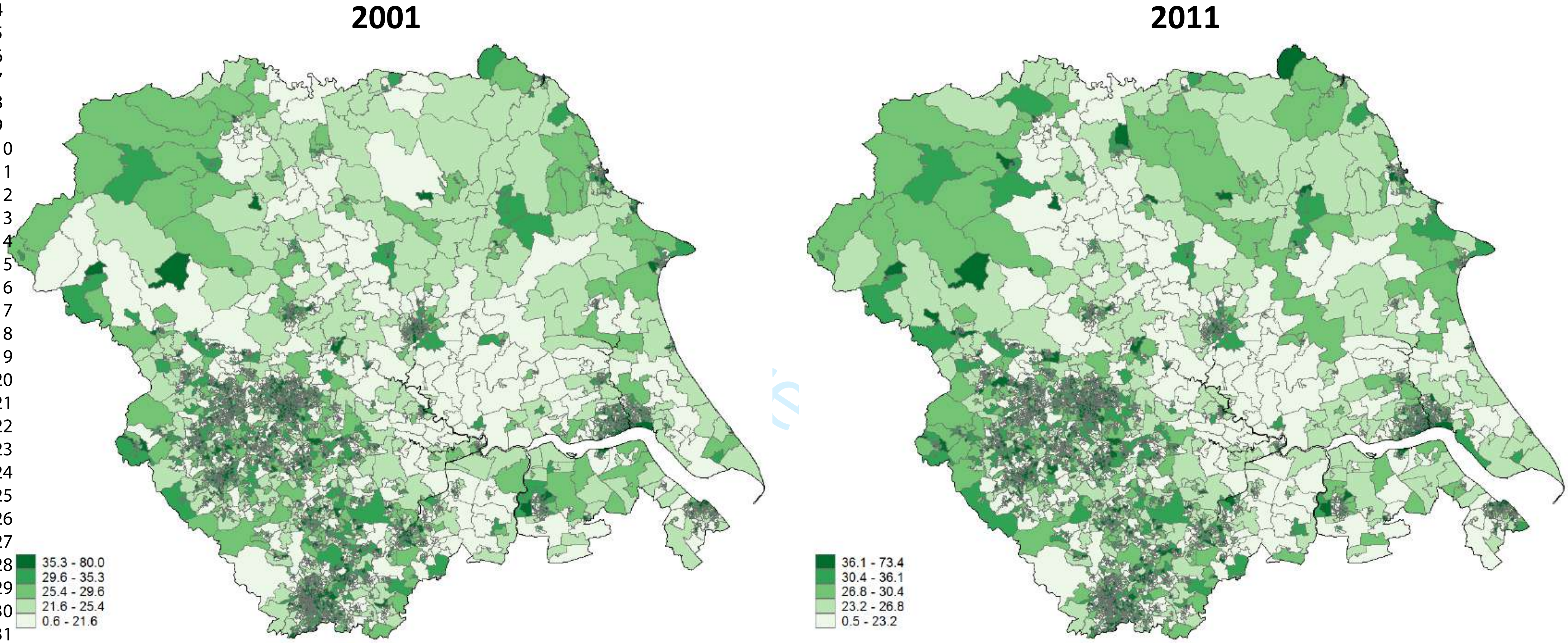

East Midlands, One Person Households % of all households

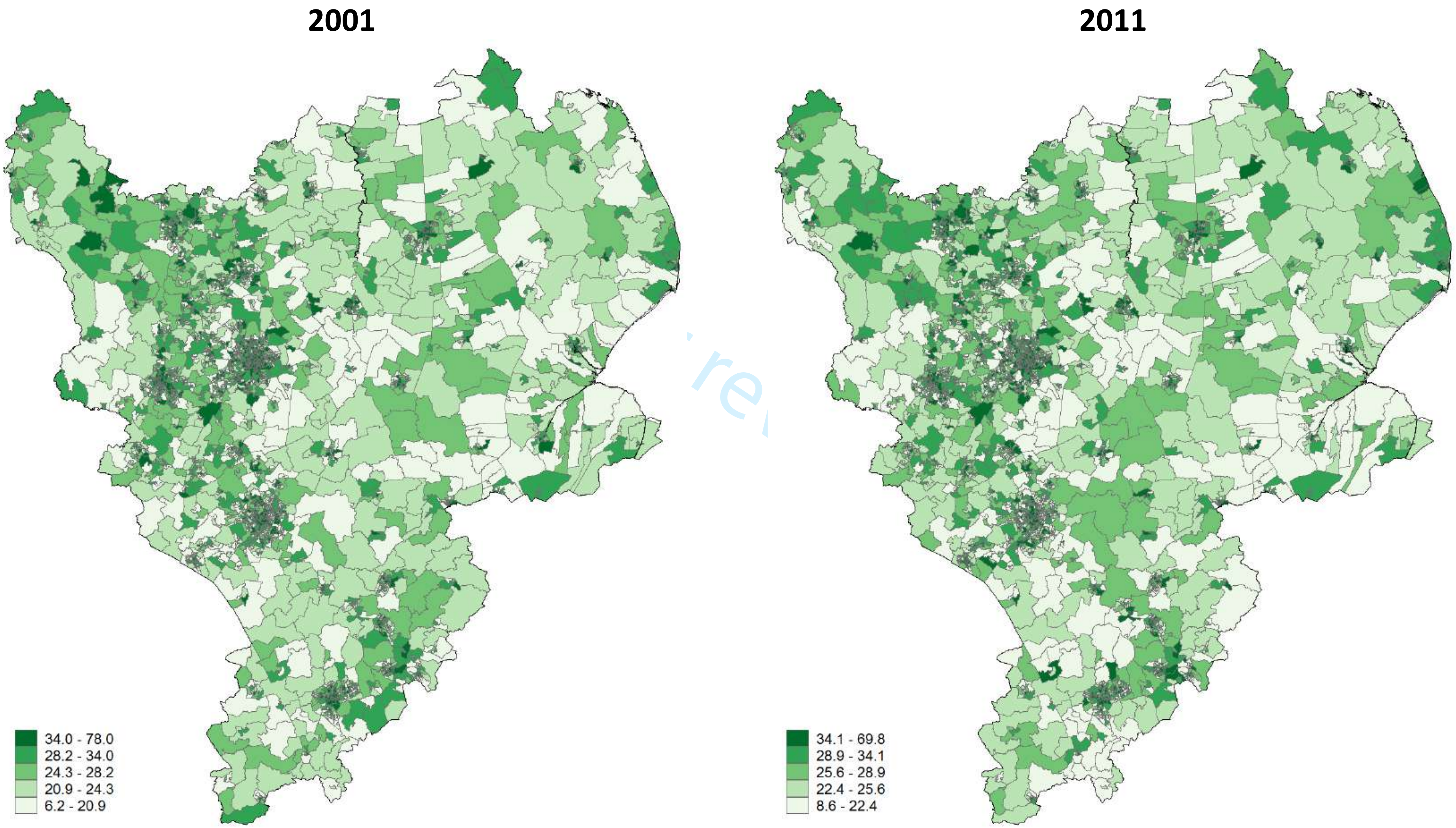

West Midlands, One Person Households % of all households

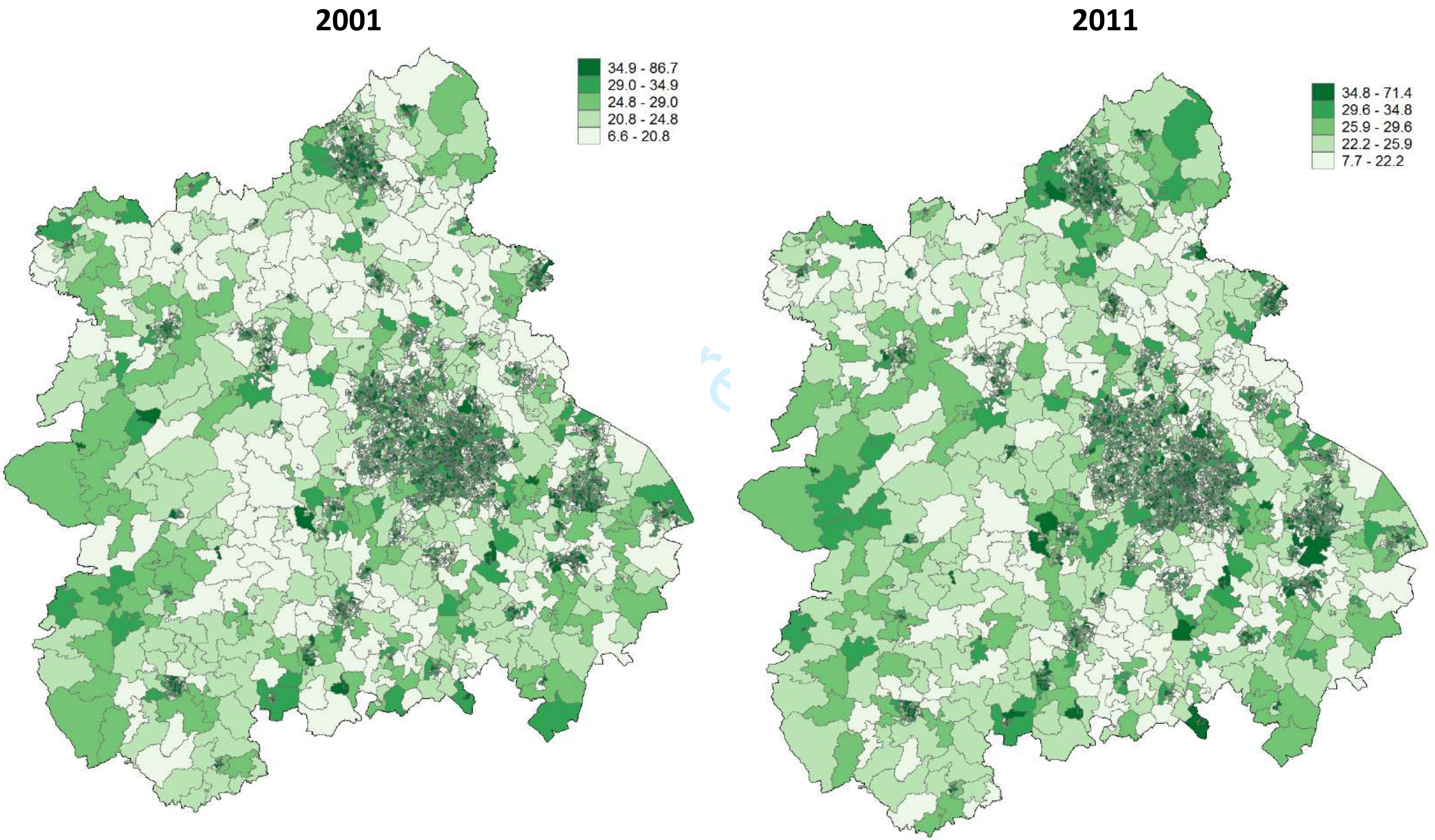

East of England, One Person Households % of all households

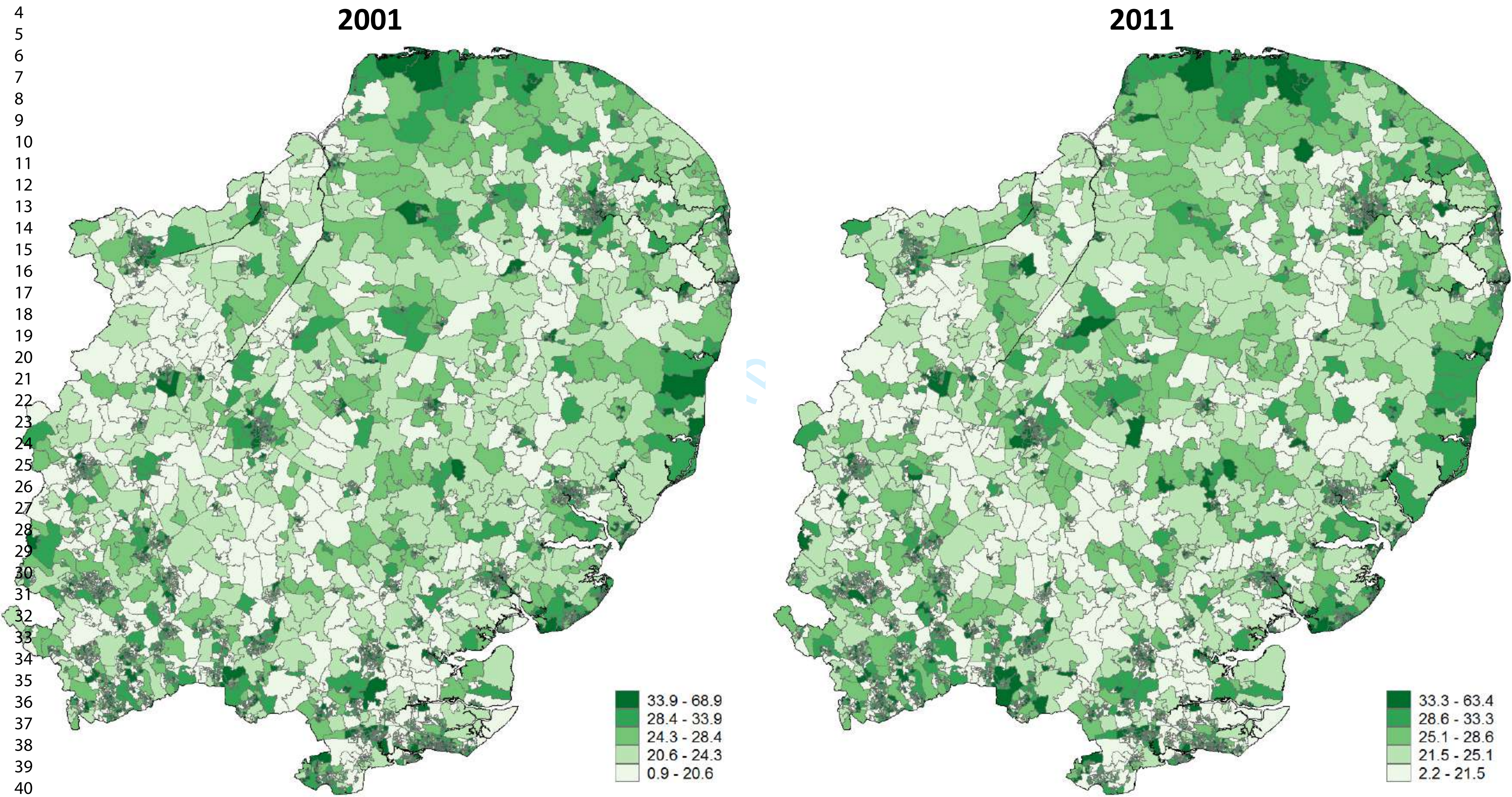

London, One Person Households % of all households

2001

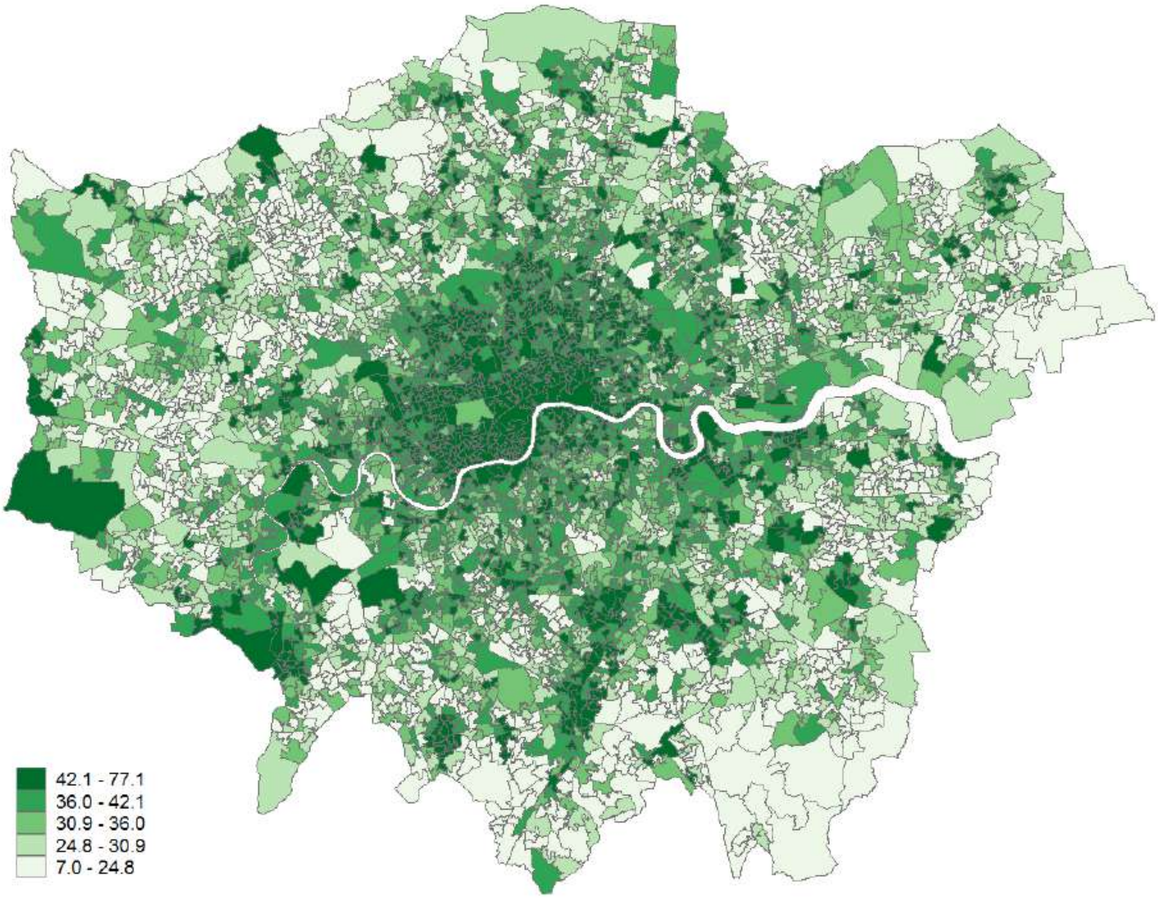

2011

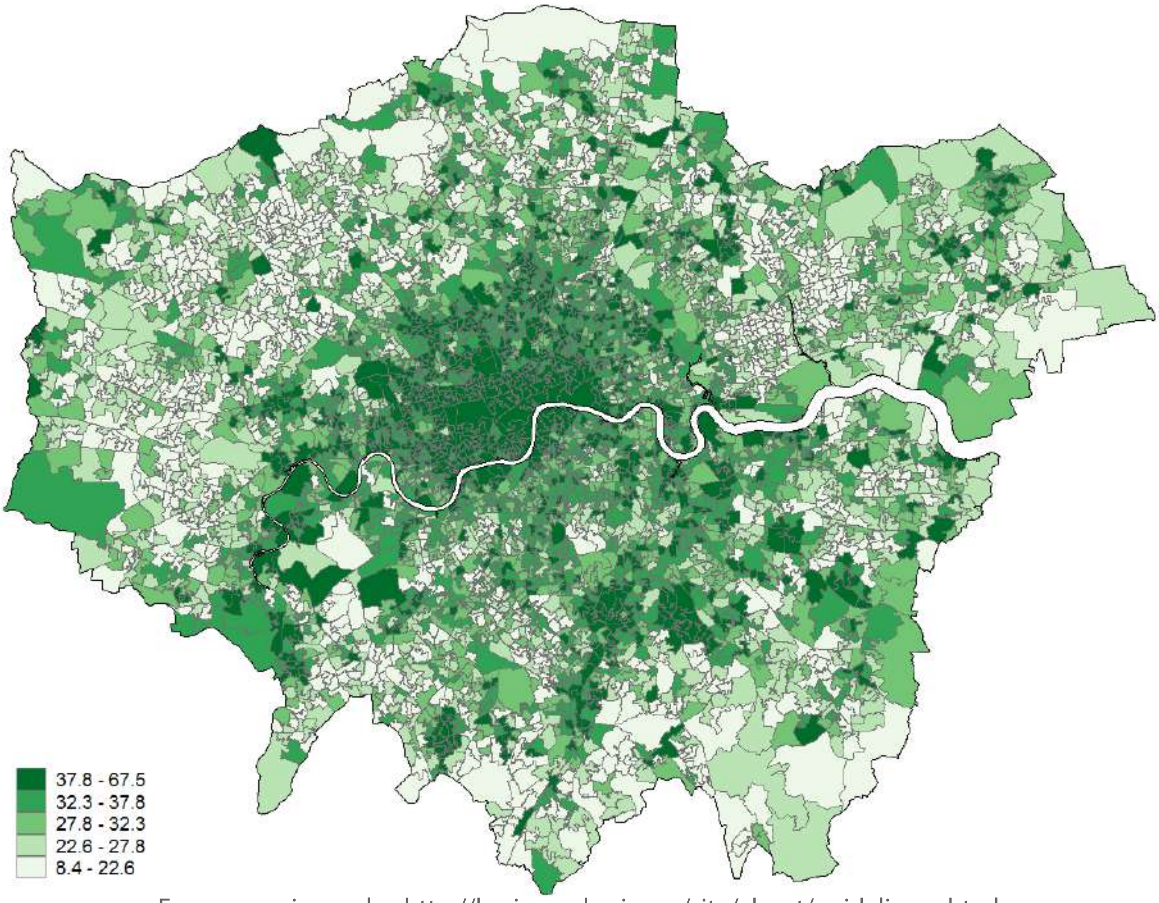

South East Coast, One Person Households % of all households

2001

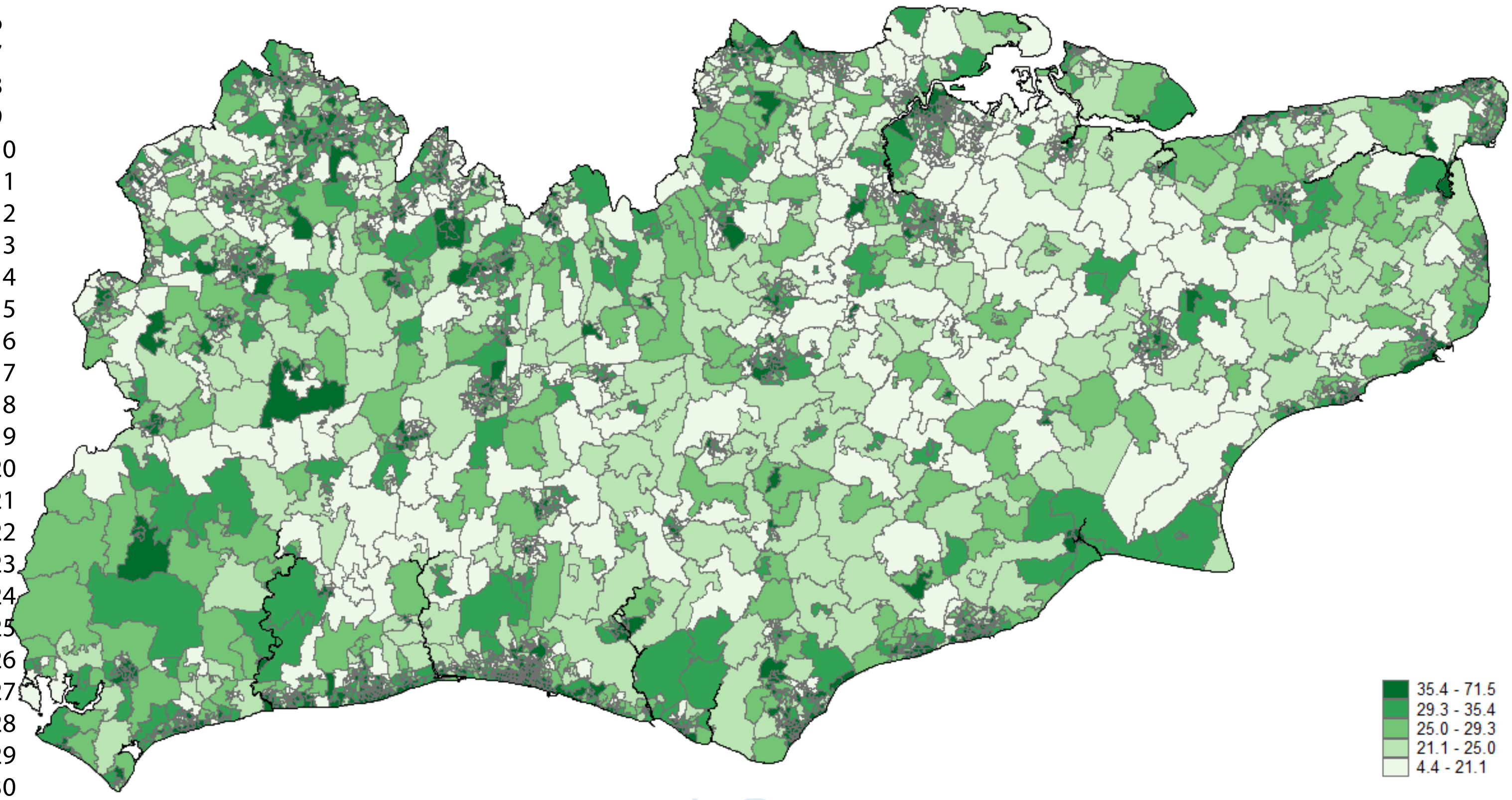

2011

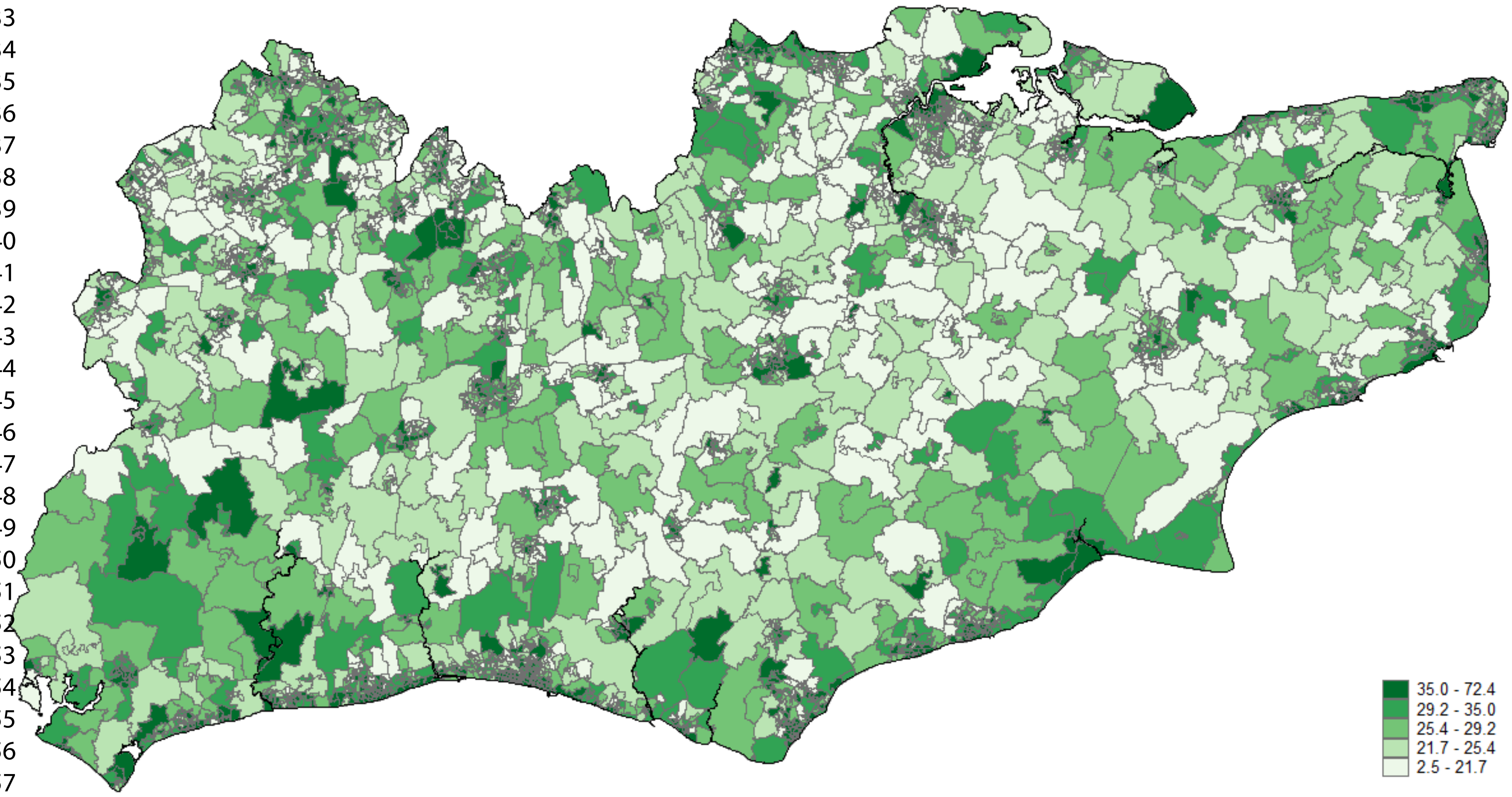

South Central, One Person Households % of all households

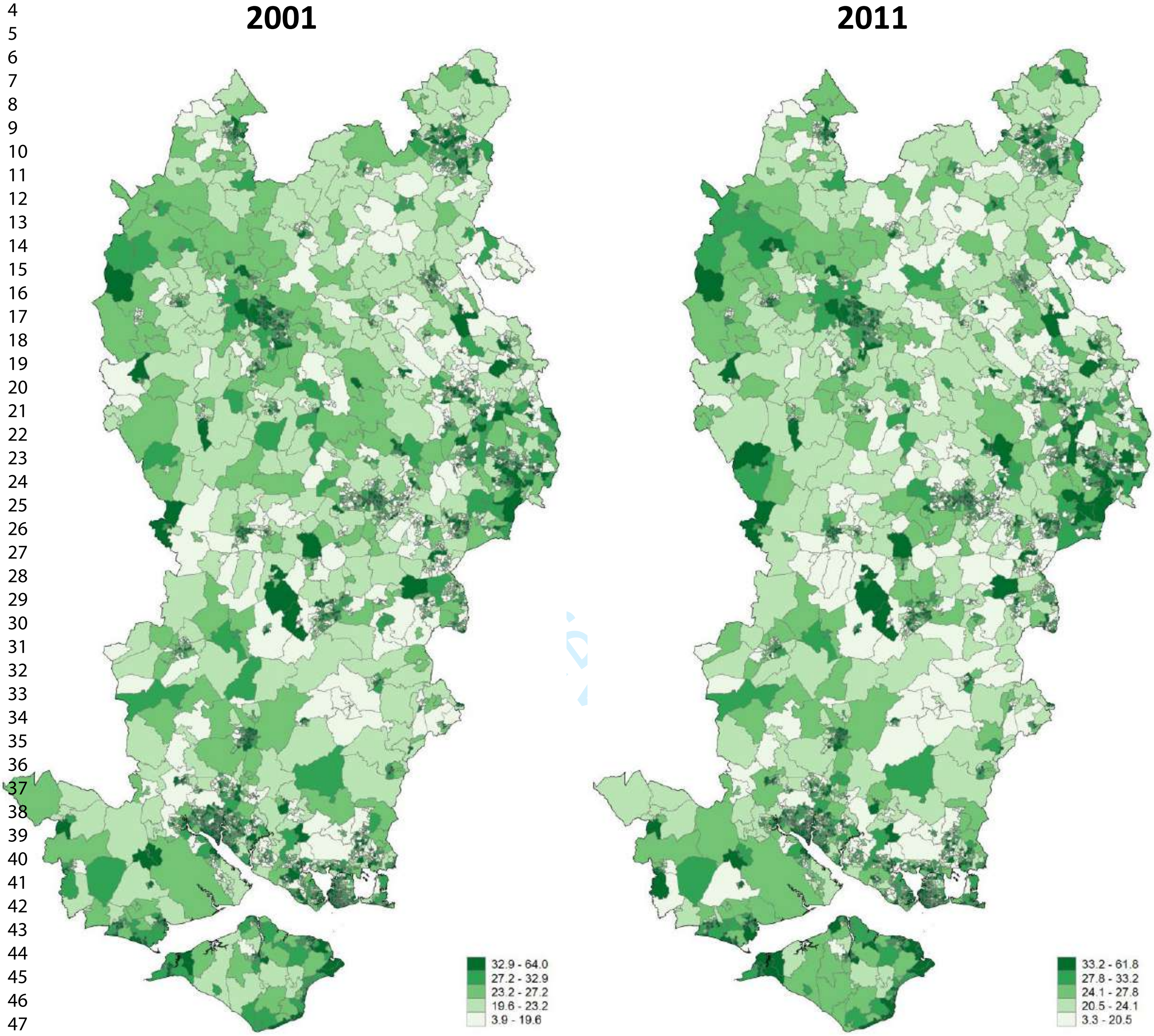

South West, One Person Households % of all households

2001

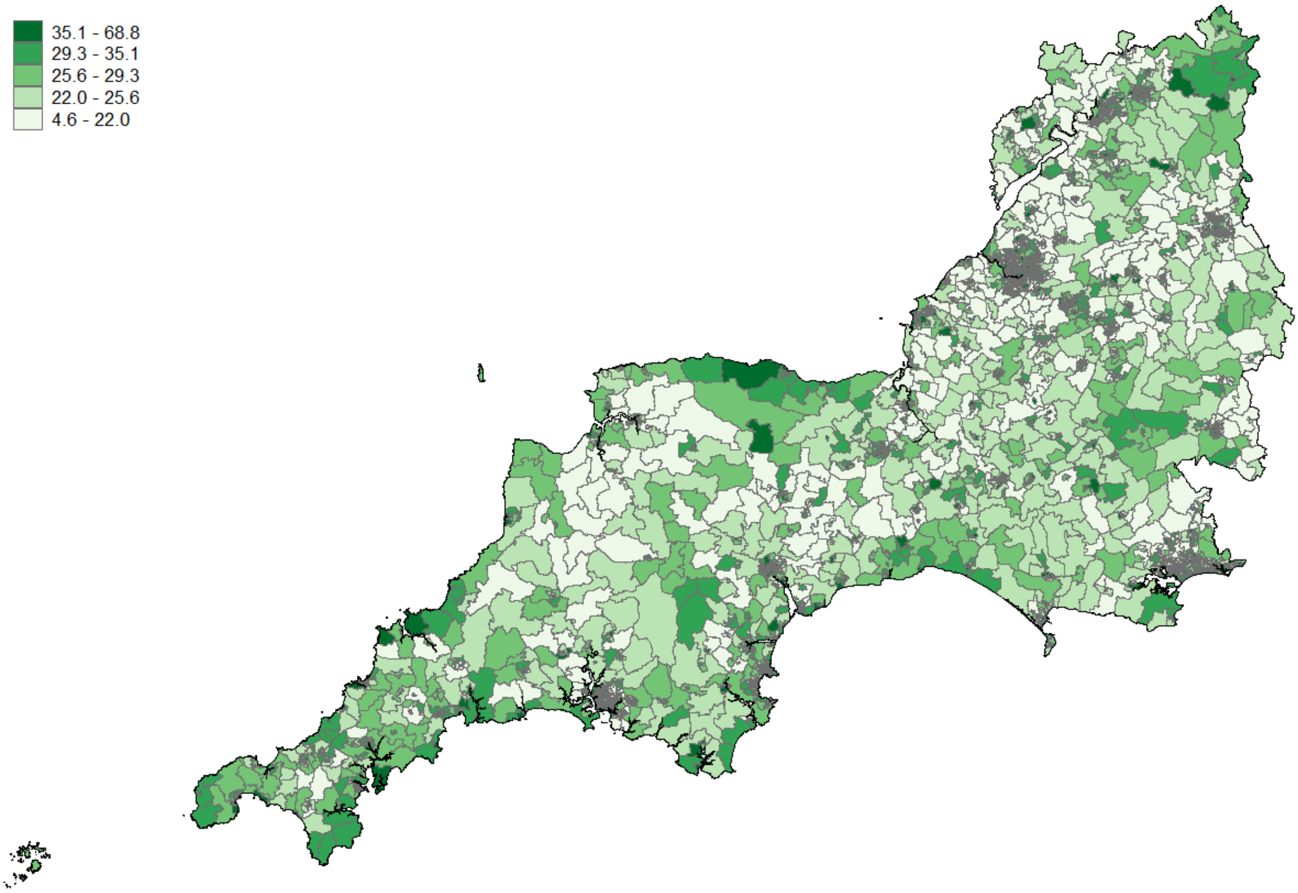

2011

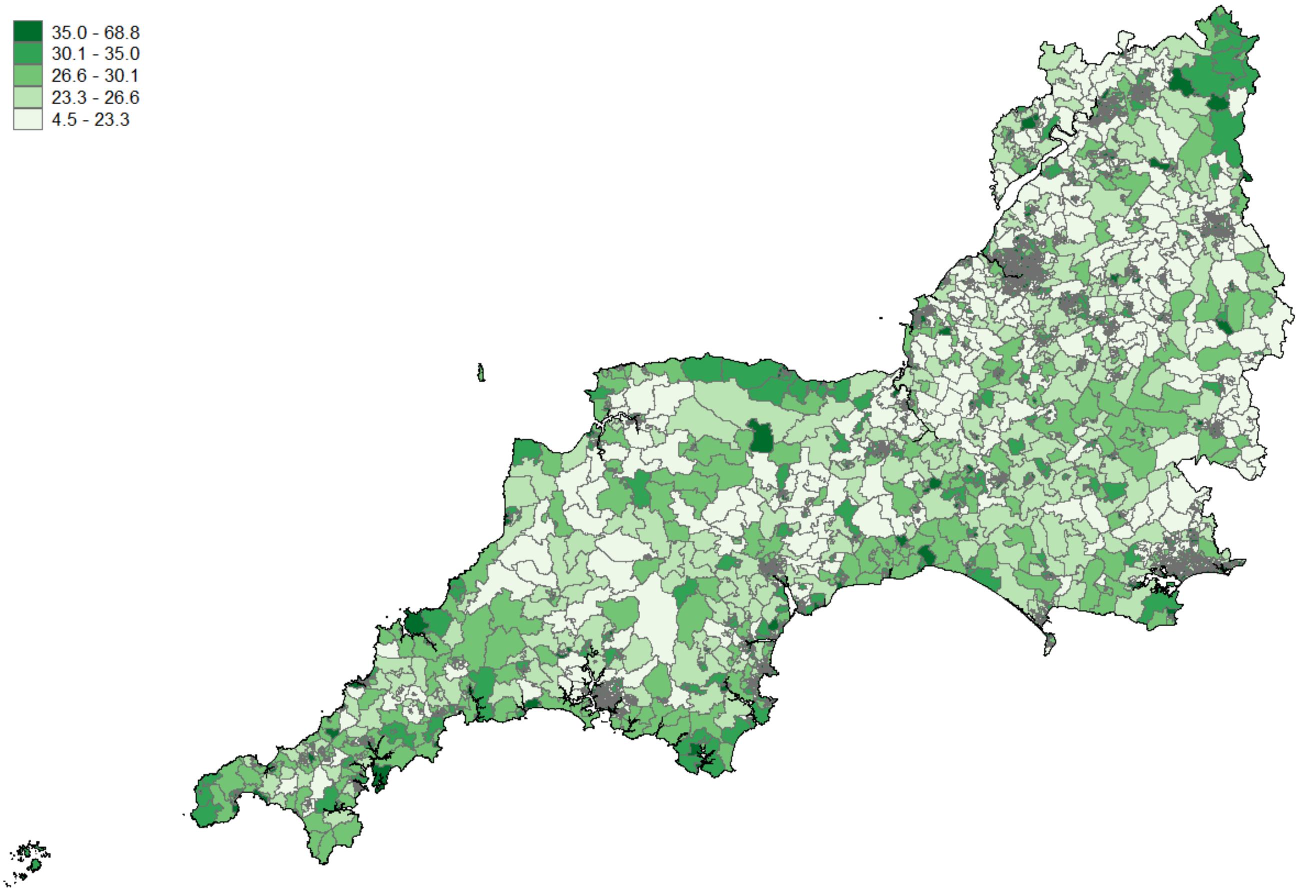

Private renting

England 2001, Private Renting % of all households

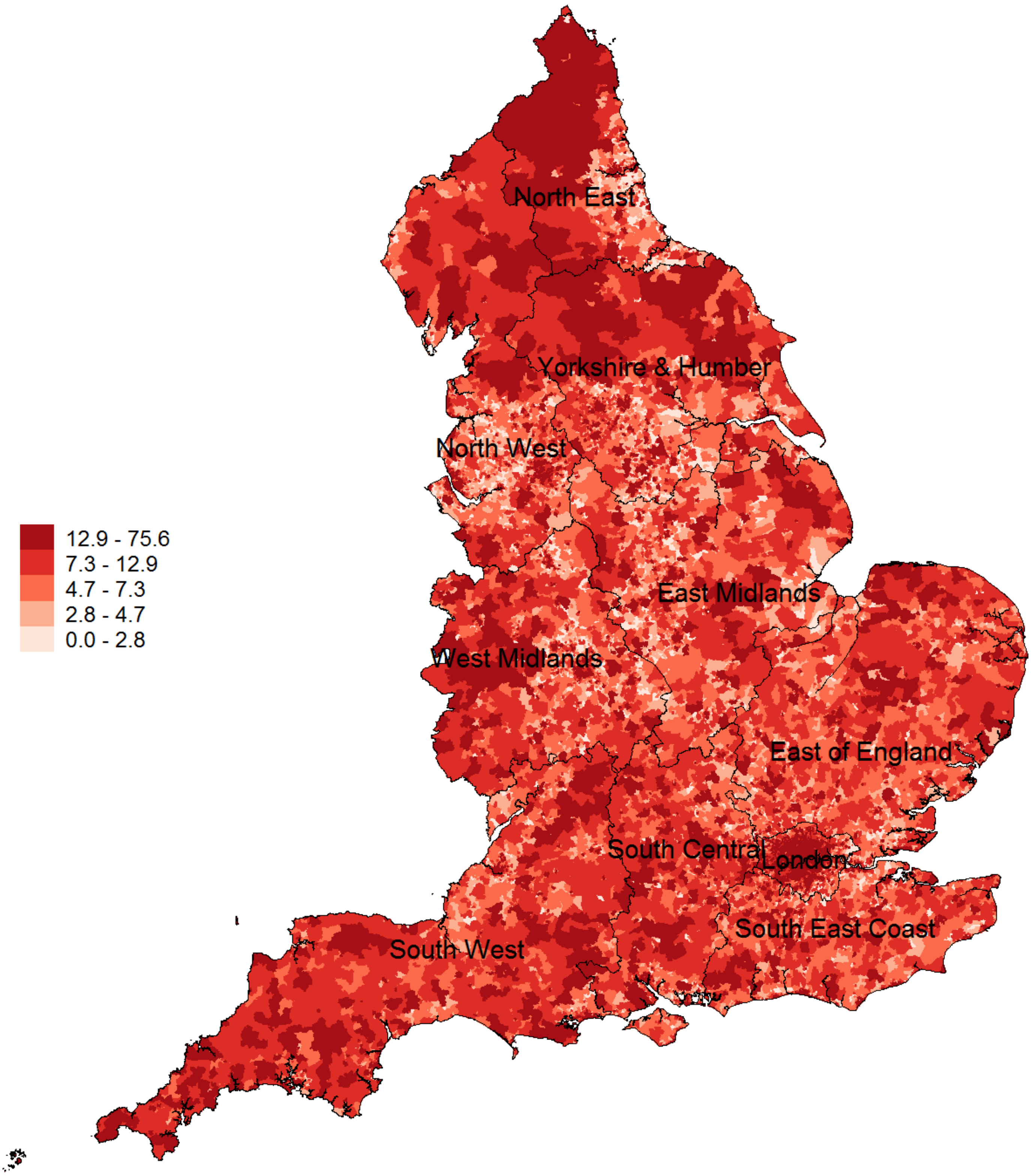

England 2011, Private Renting % of all households

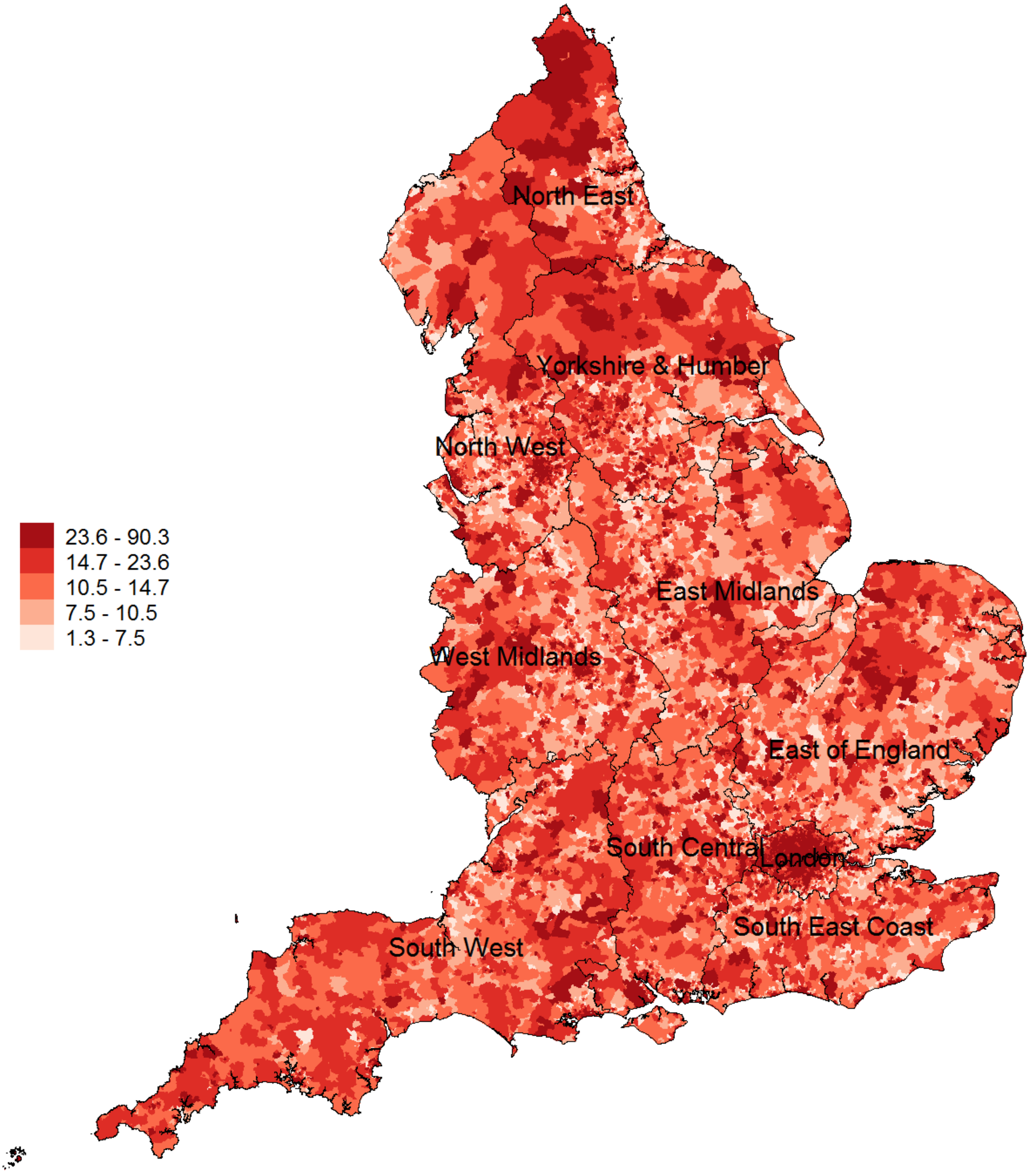

North East, Private Renting % of all households

2001

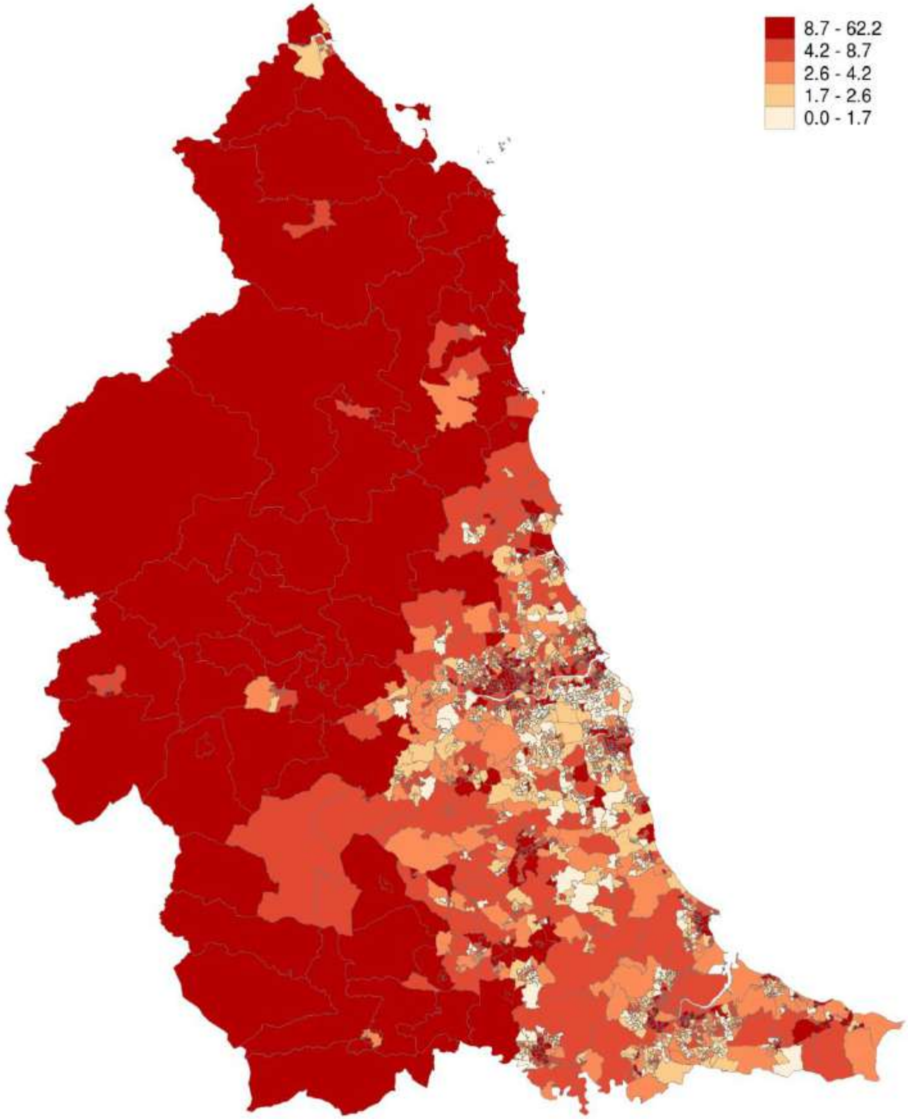

2011

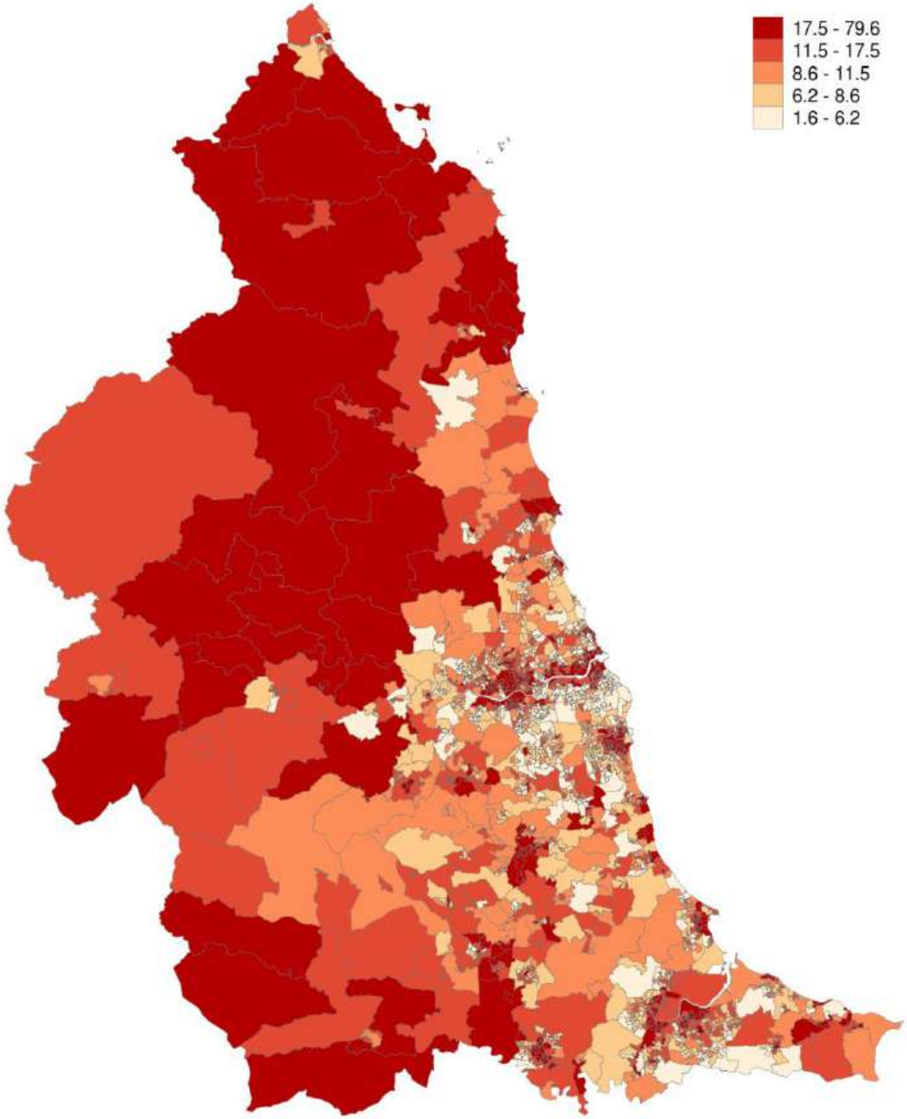

For peer review only - http://bmjopen.bmj.com/site/about/guidelines.xhtml

North West, Private Renting % of all households

2001 | 2011

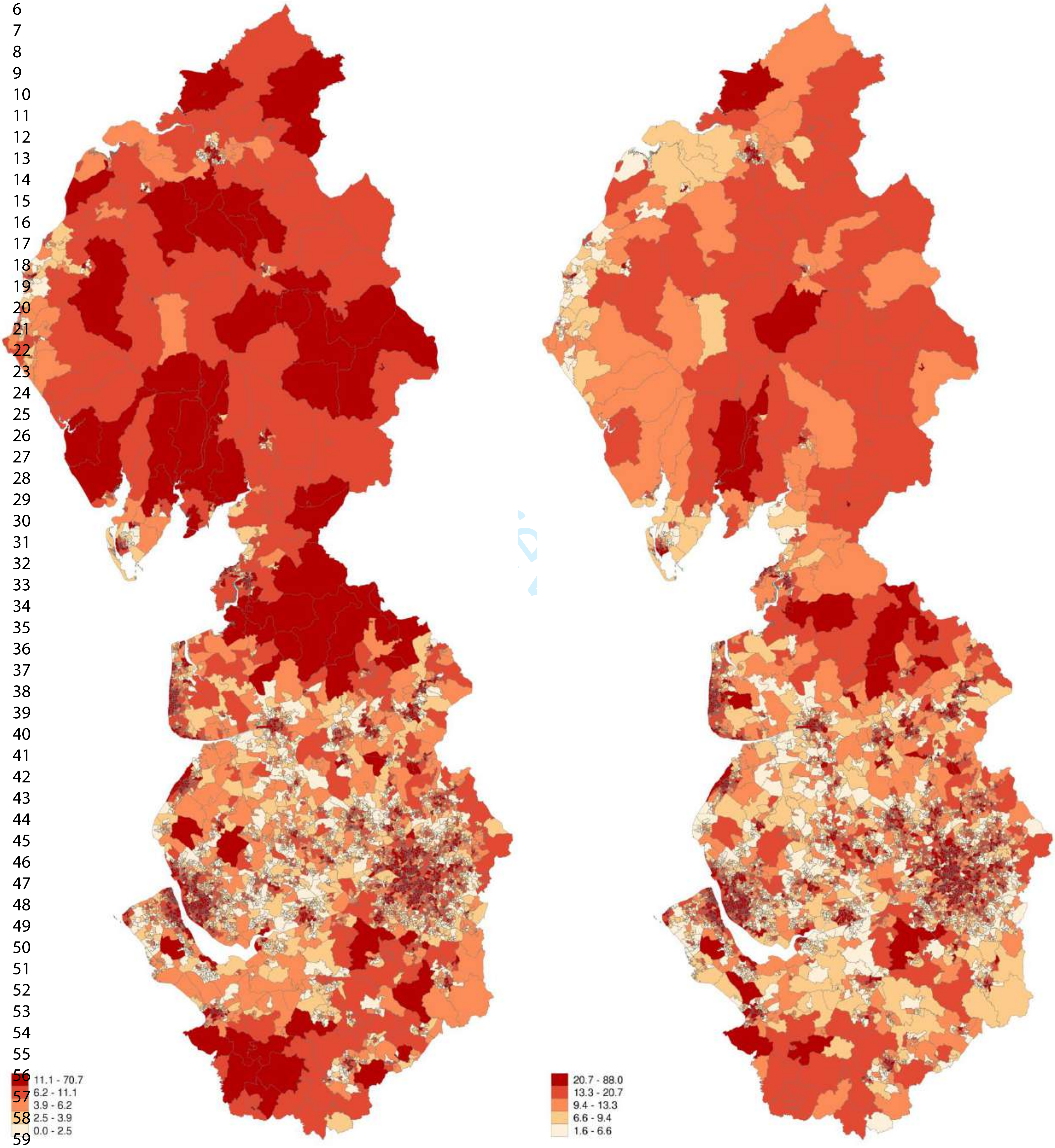

Yorkshire and the Humber, Private Renting % of all households

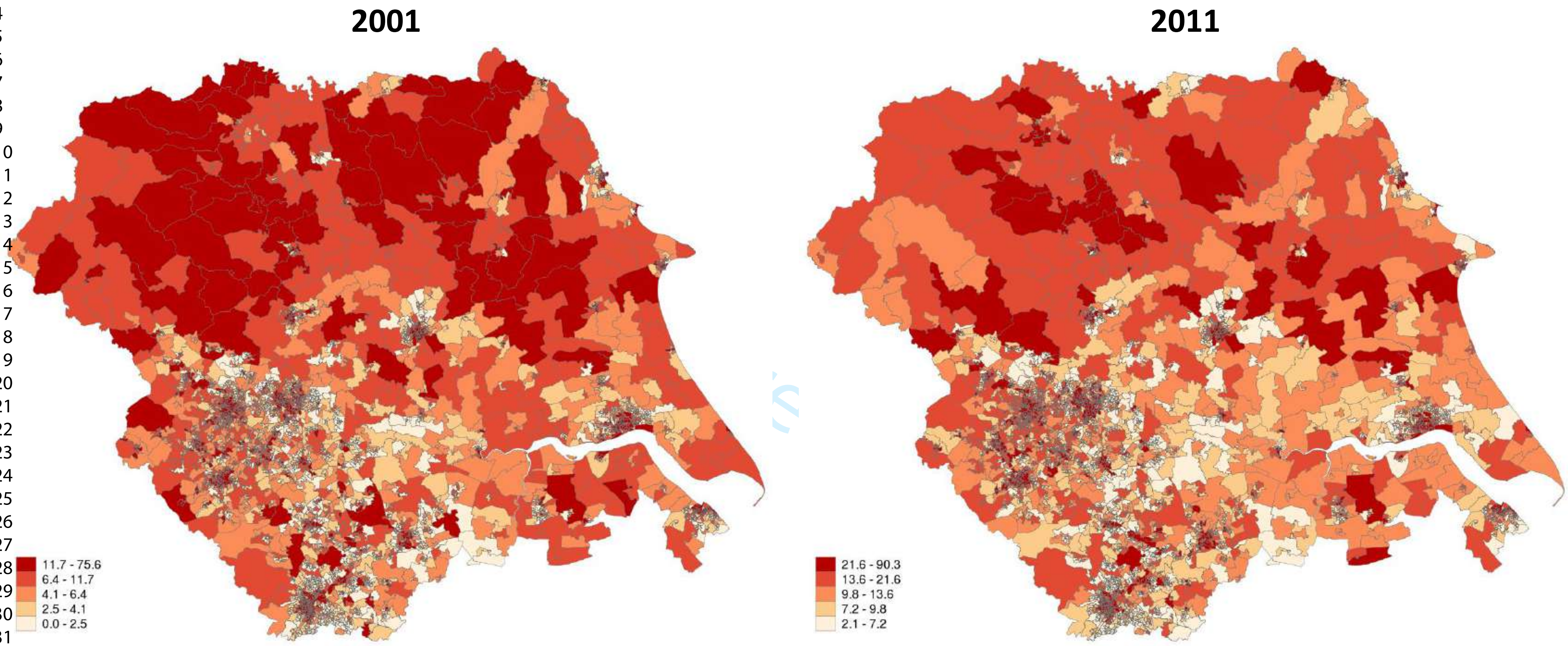

East Midlands, Private Renting % of all households

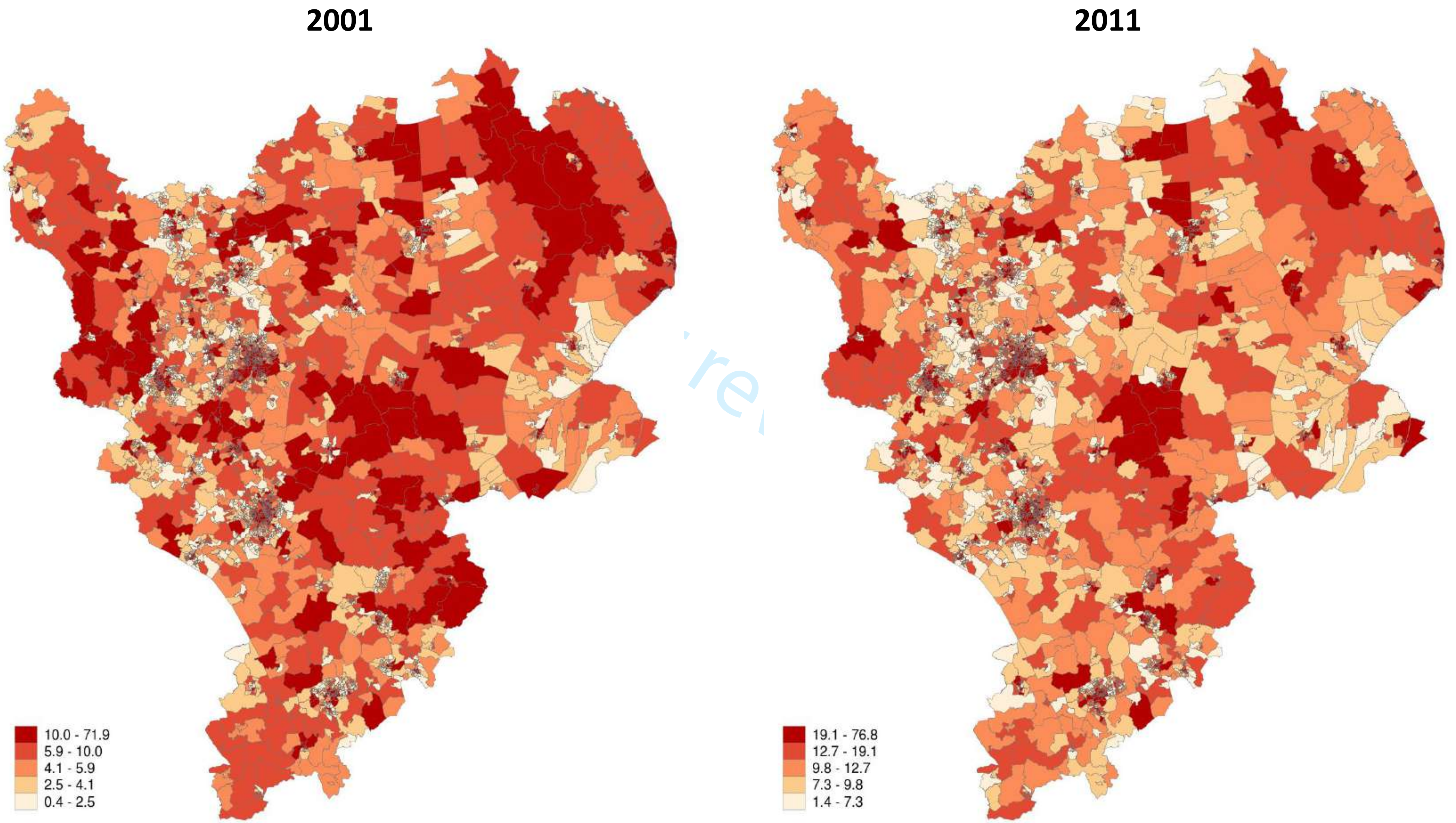

West Midlands, Private Renting % of all households

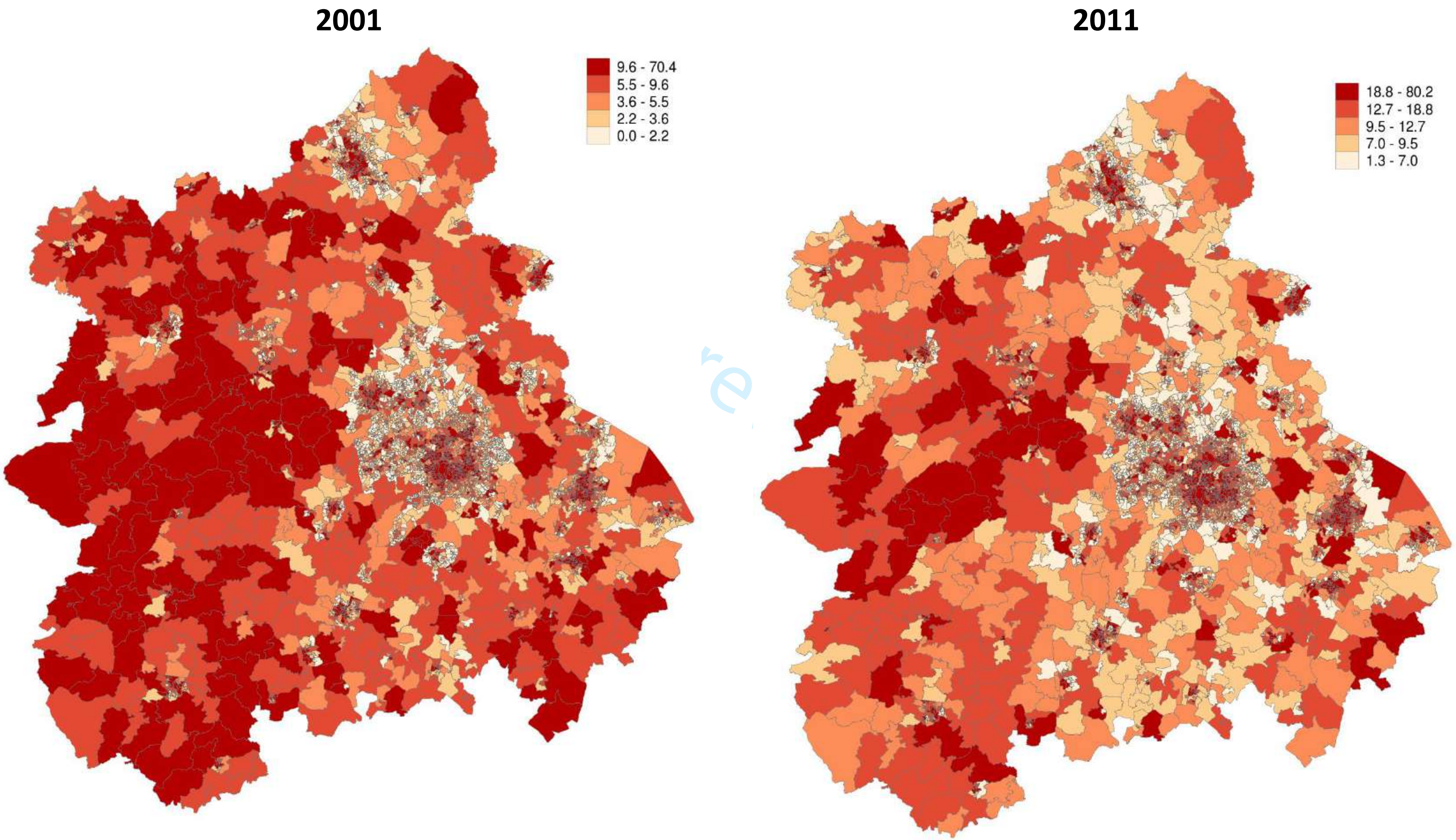

East of England, Private Renting % of all households

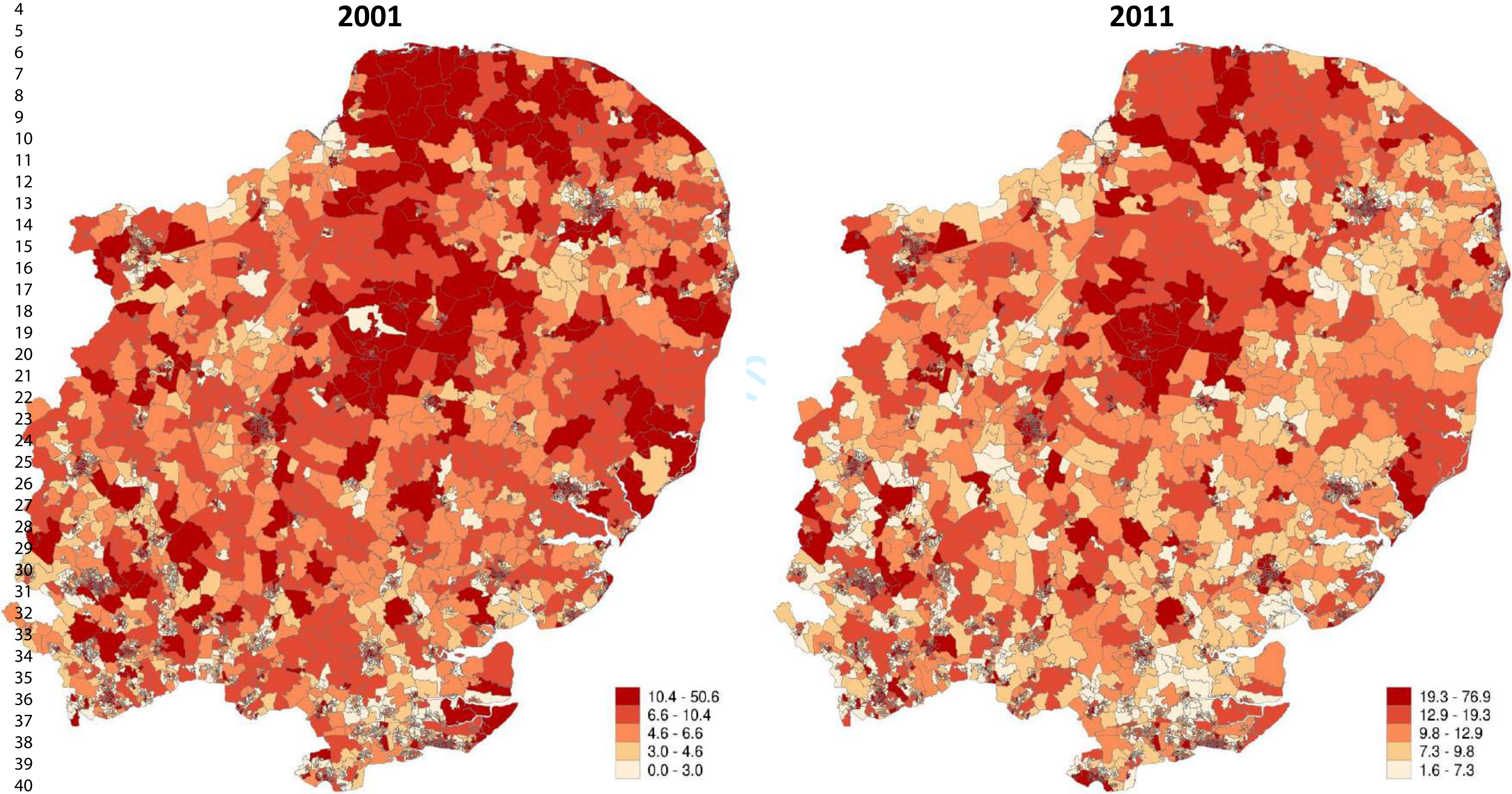

London, Private Renting % of all households

2001

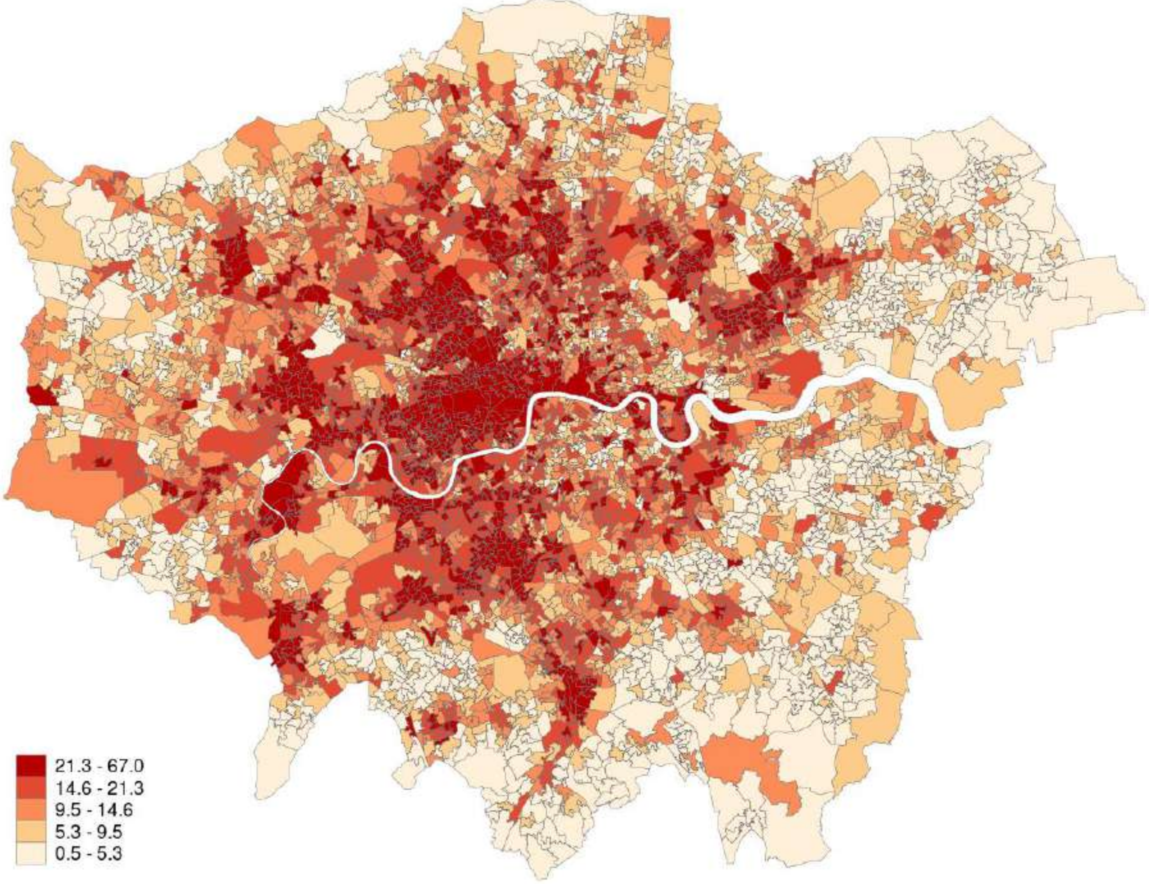

2011

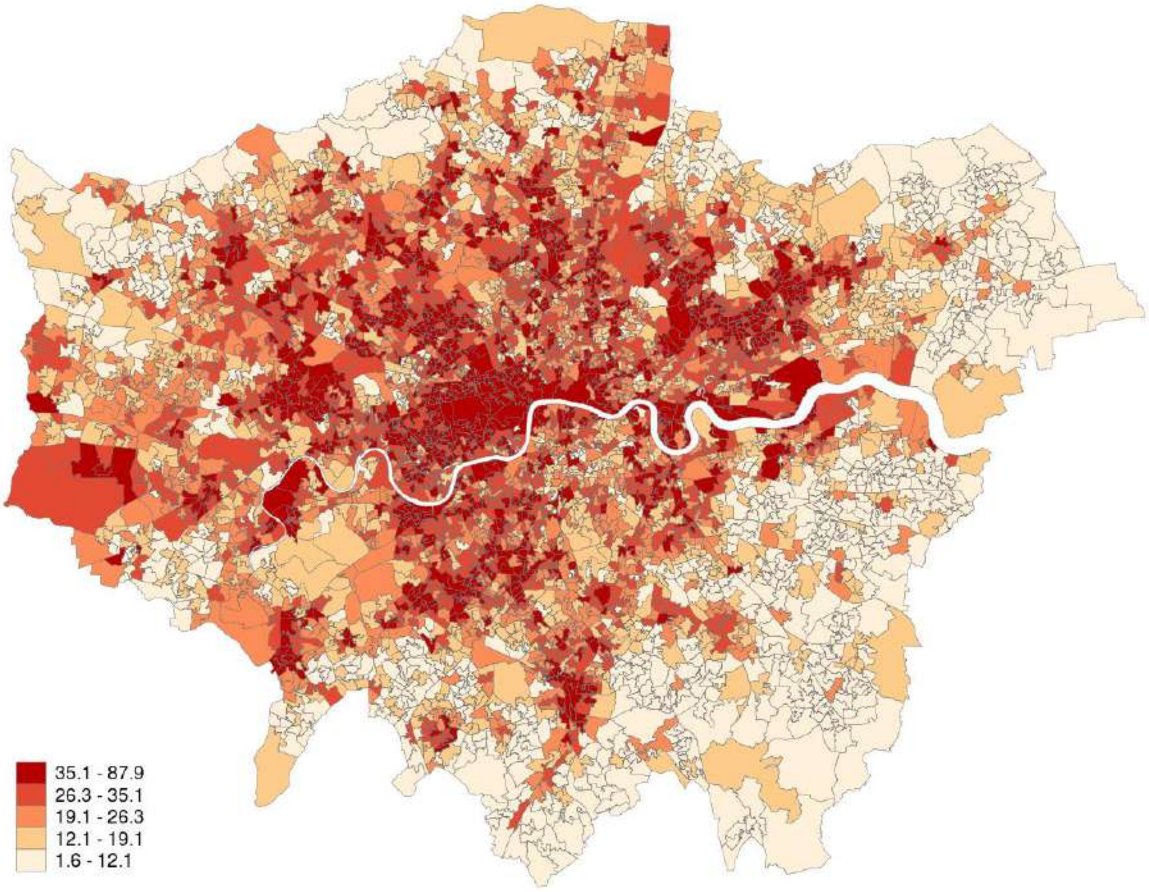

South East Coast, Private Renting % of all households

2001

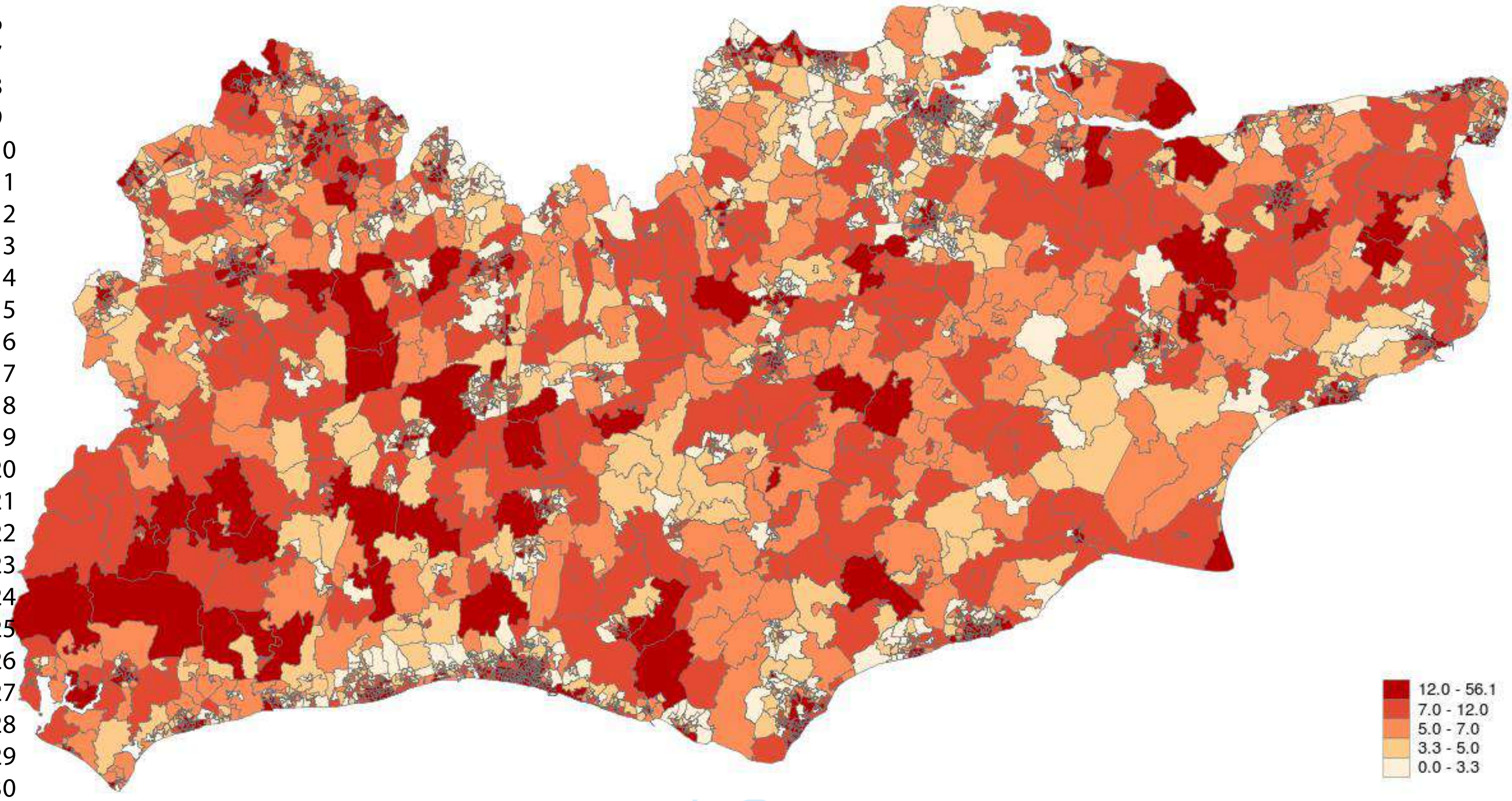

2011

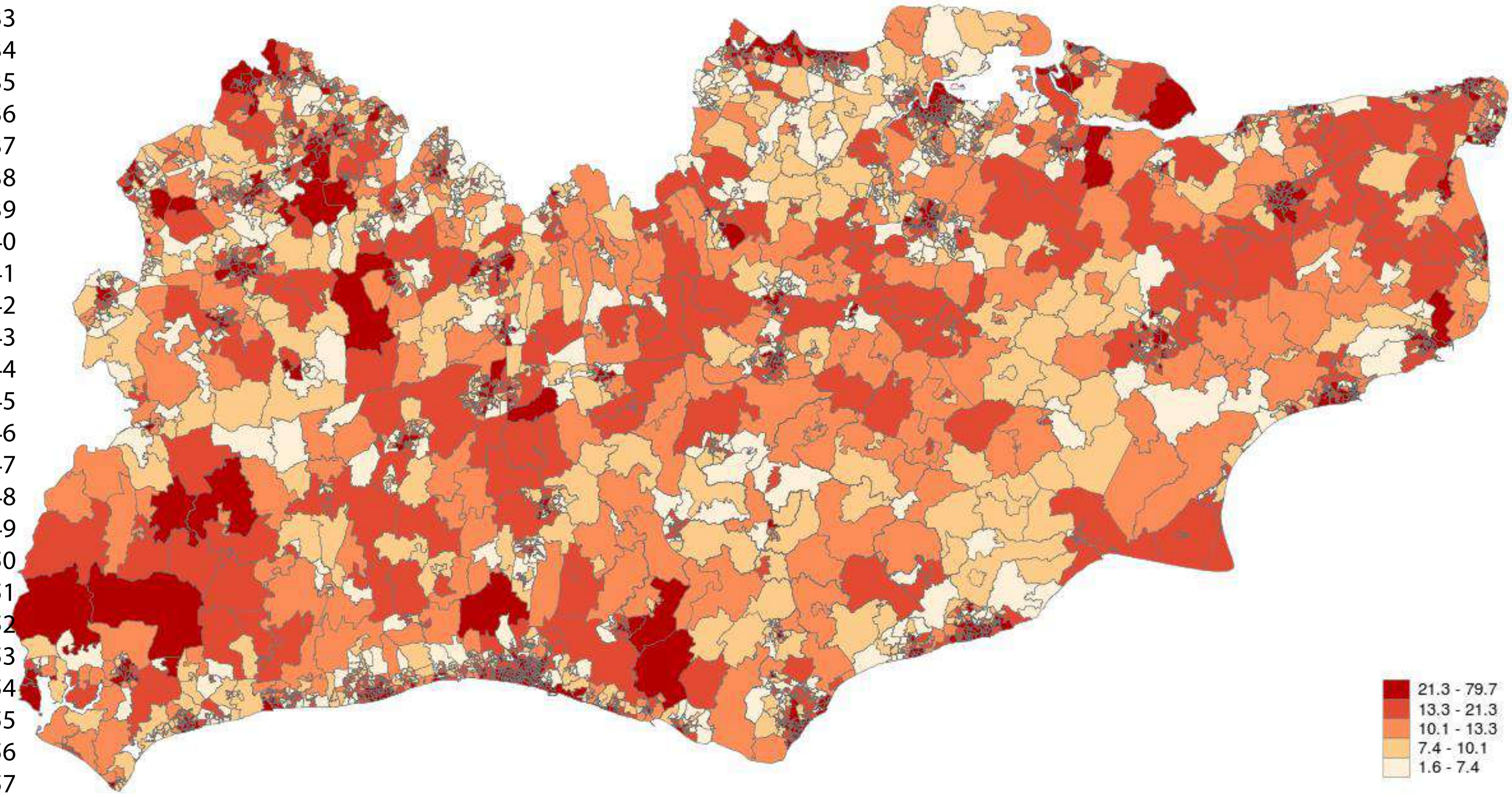

South Central, Private Renting % of all households

2001

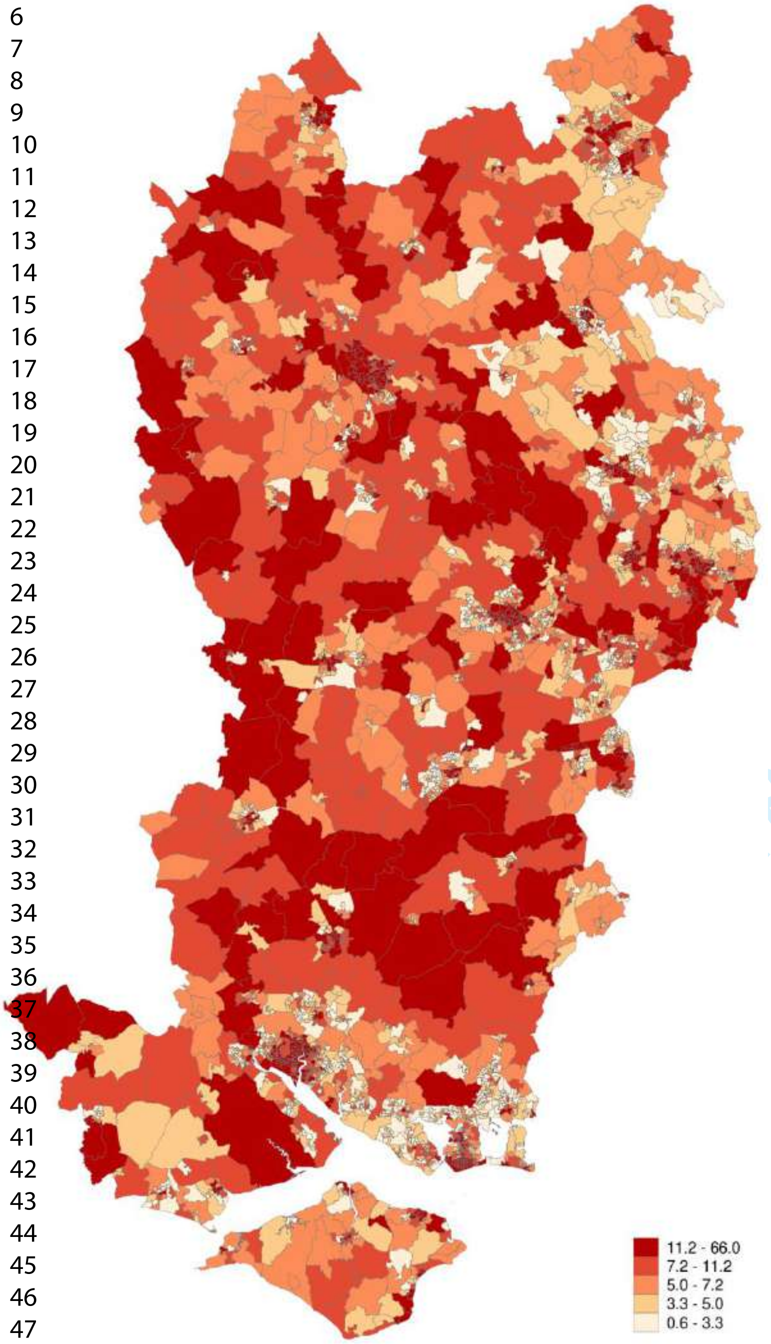

2011

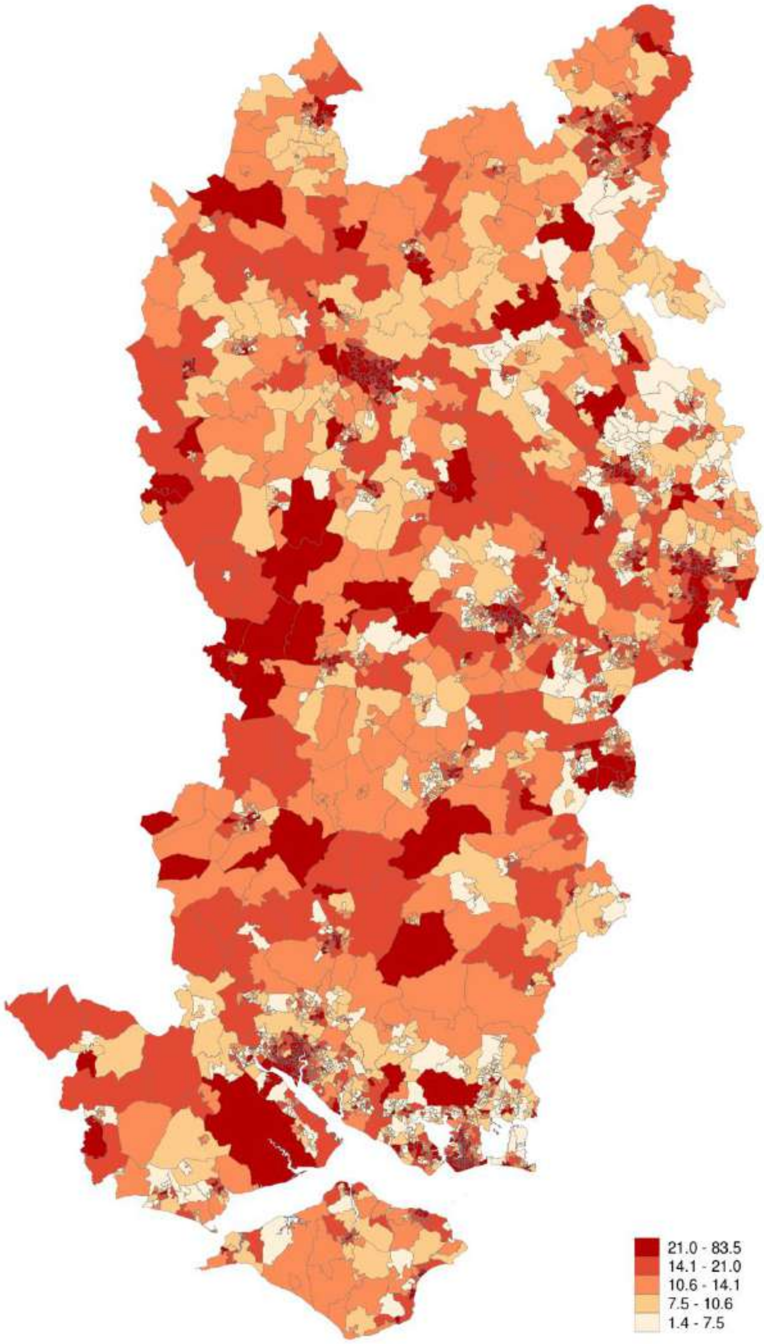

South West, Private Renting % of all households

2001

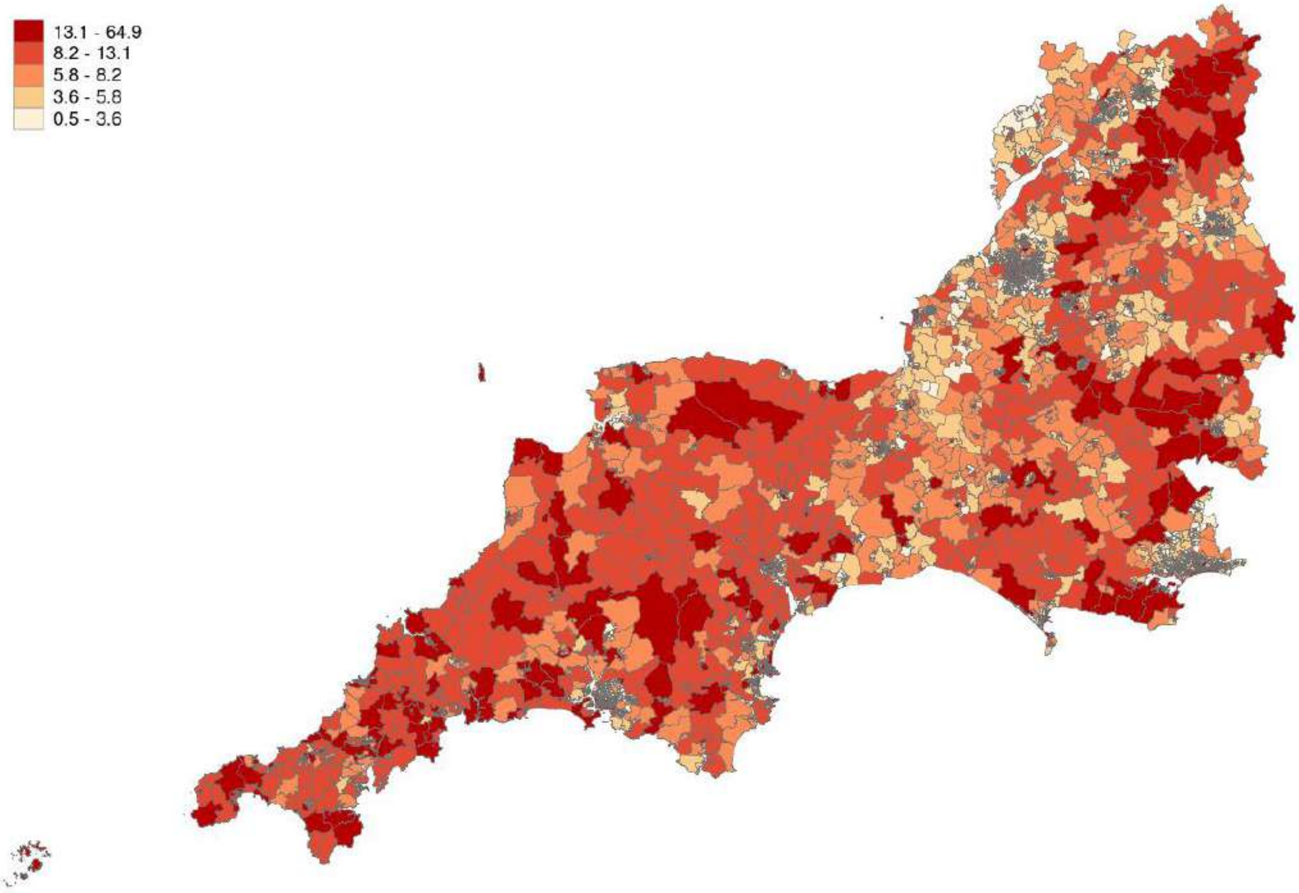

2011

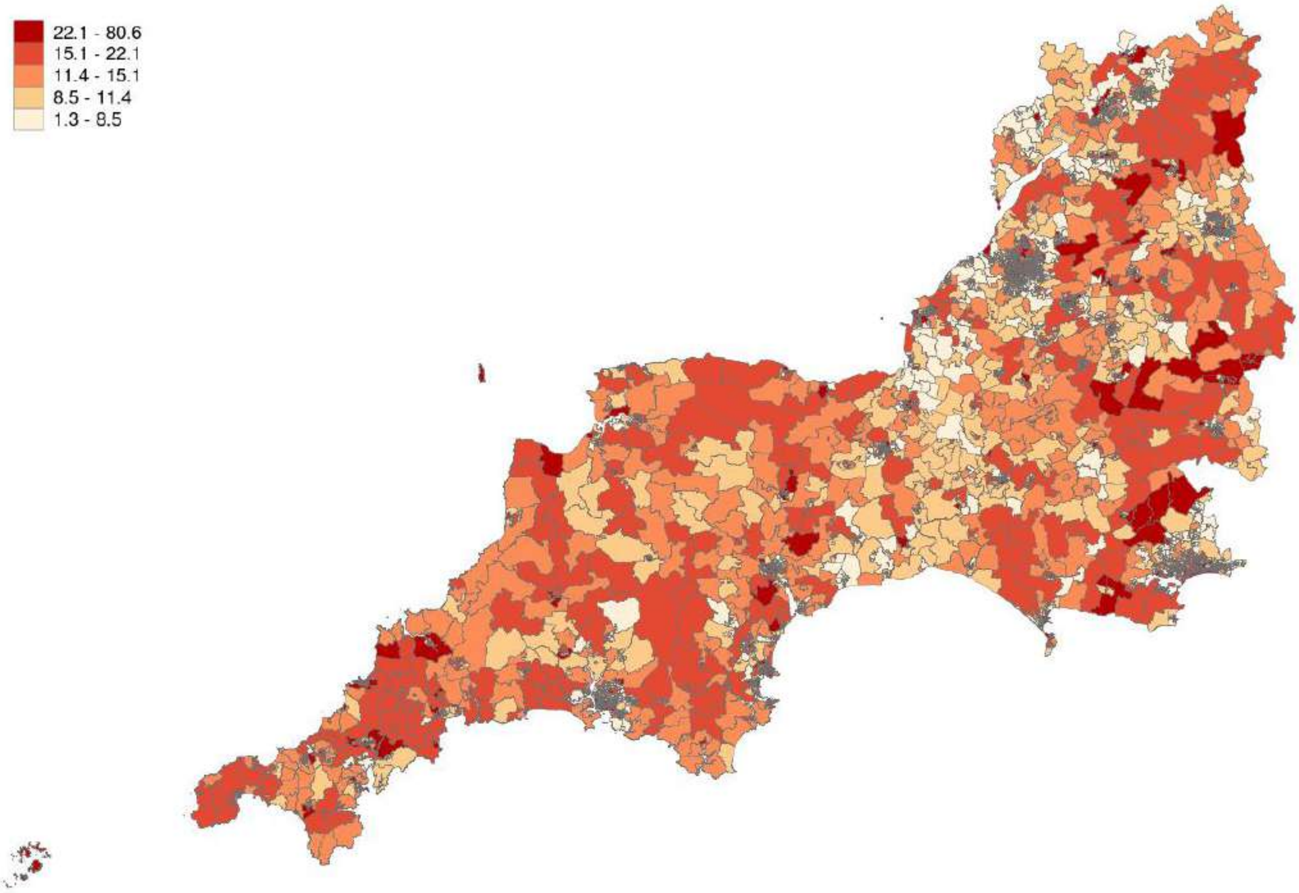

Population Turnover

England 2001, Population Turnover % of population

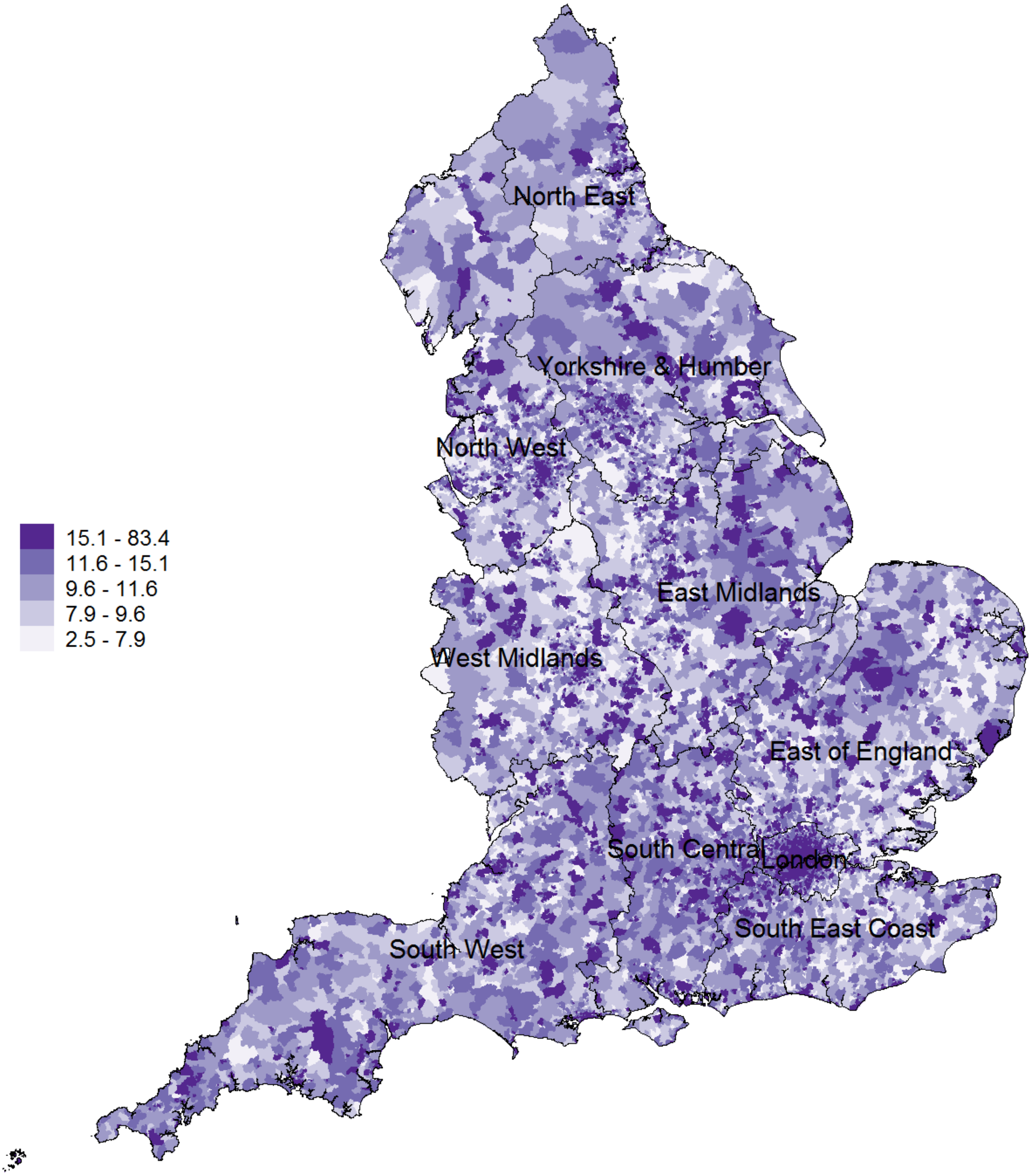

England 2011, Population Turnover % of population

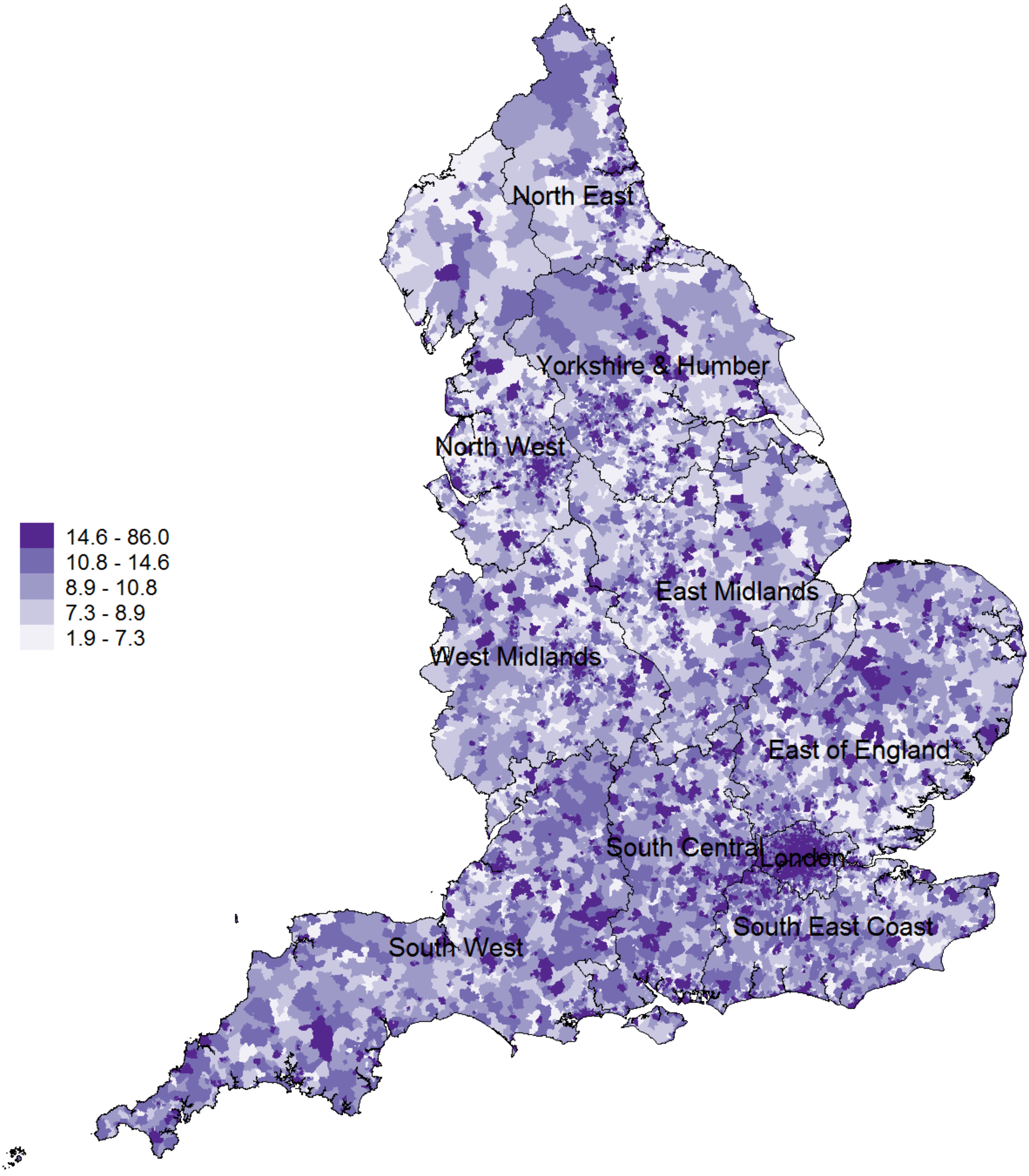

North East, Population Turnover % of population

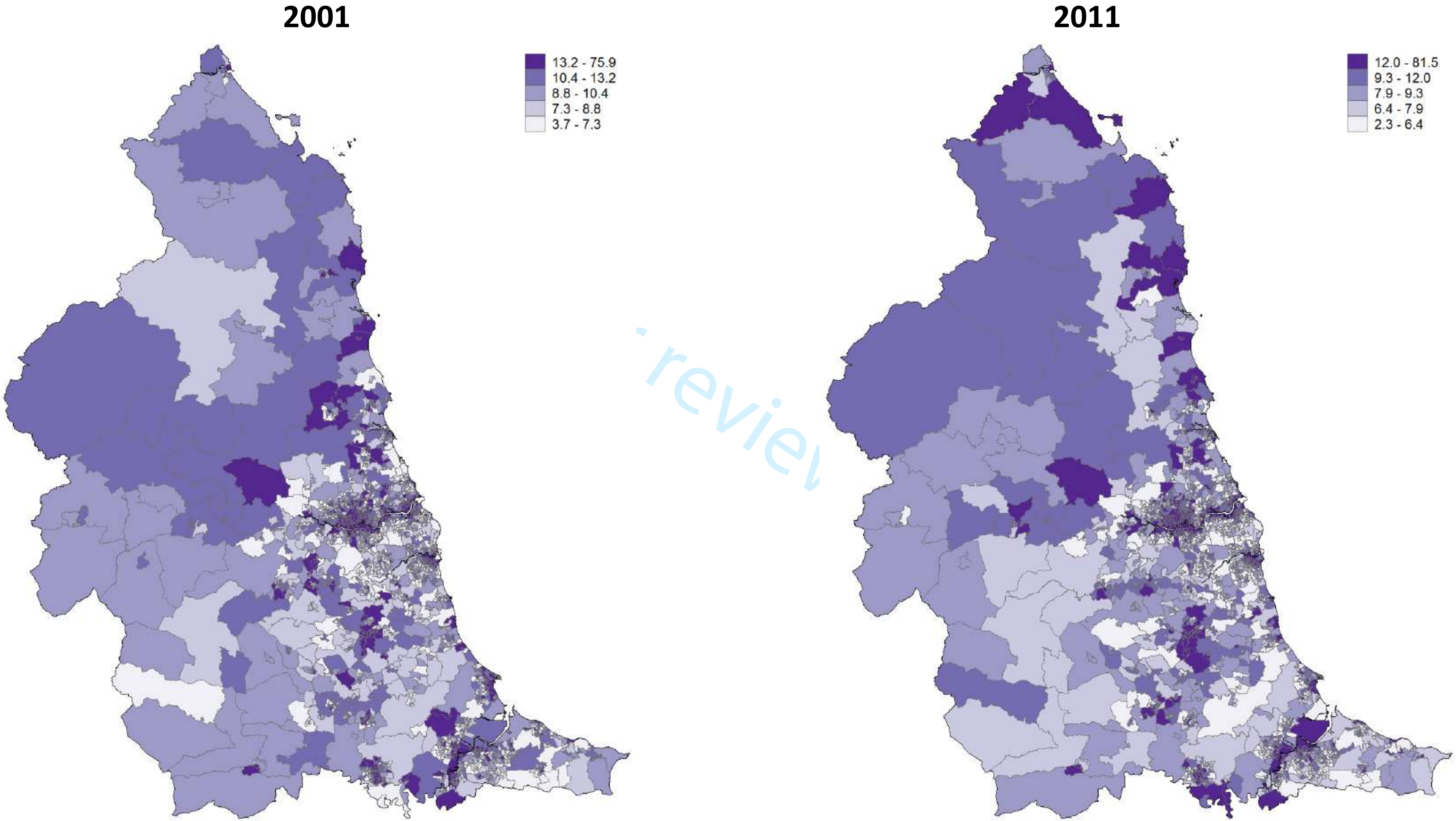

North West, Population Turnover % of population

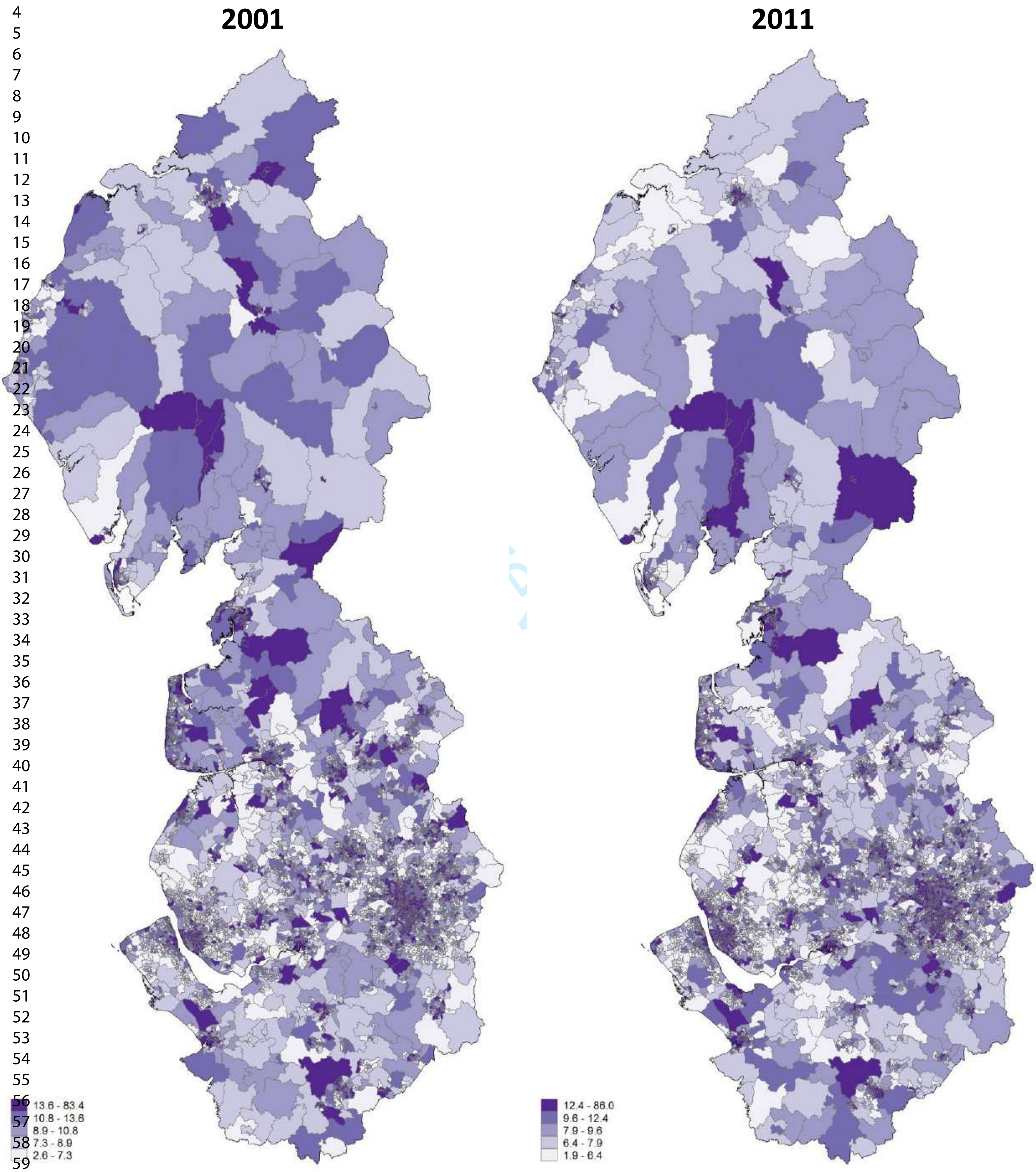

Yorkshire and the Humber, Population Turnover % of population

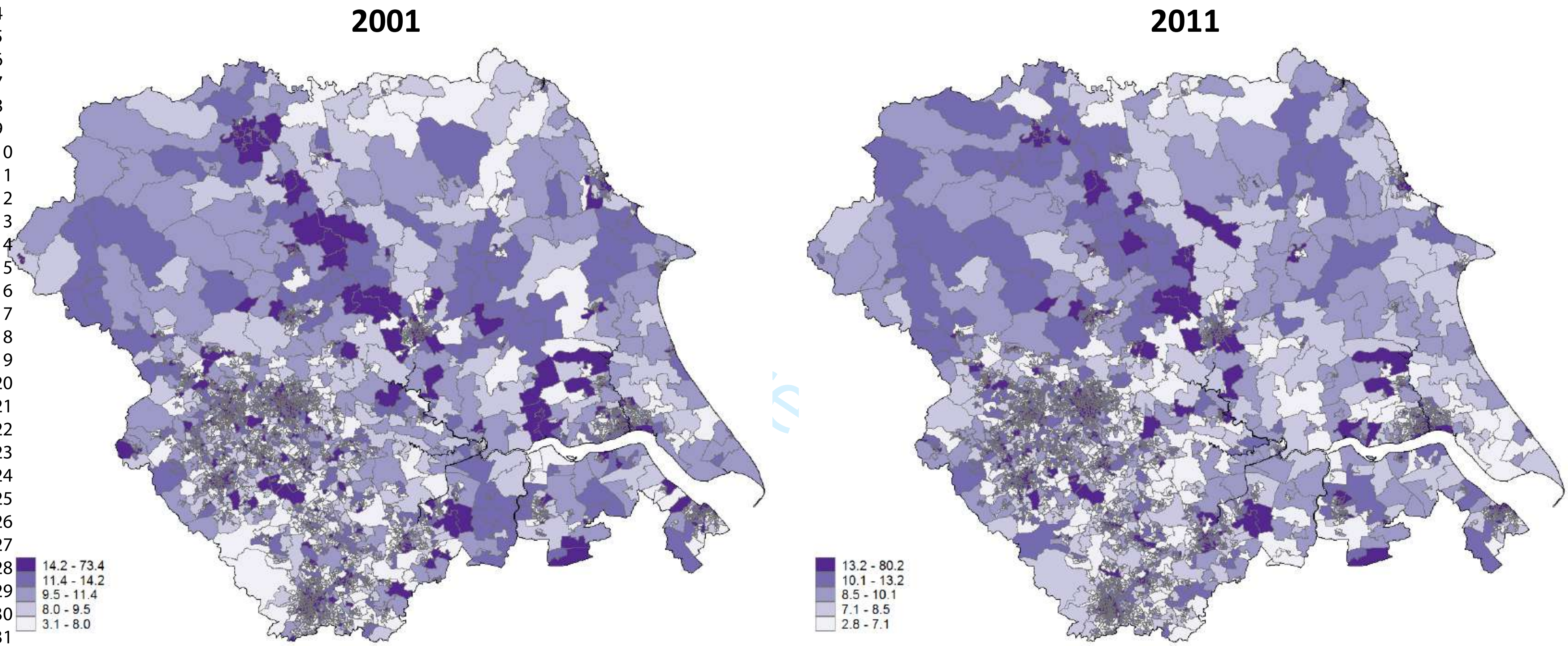

East Midlands, Population Turnover % of population

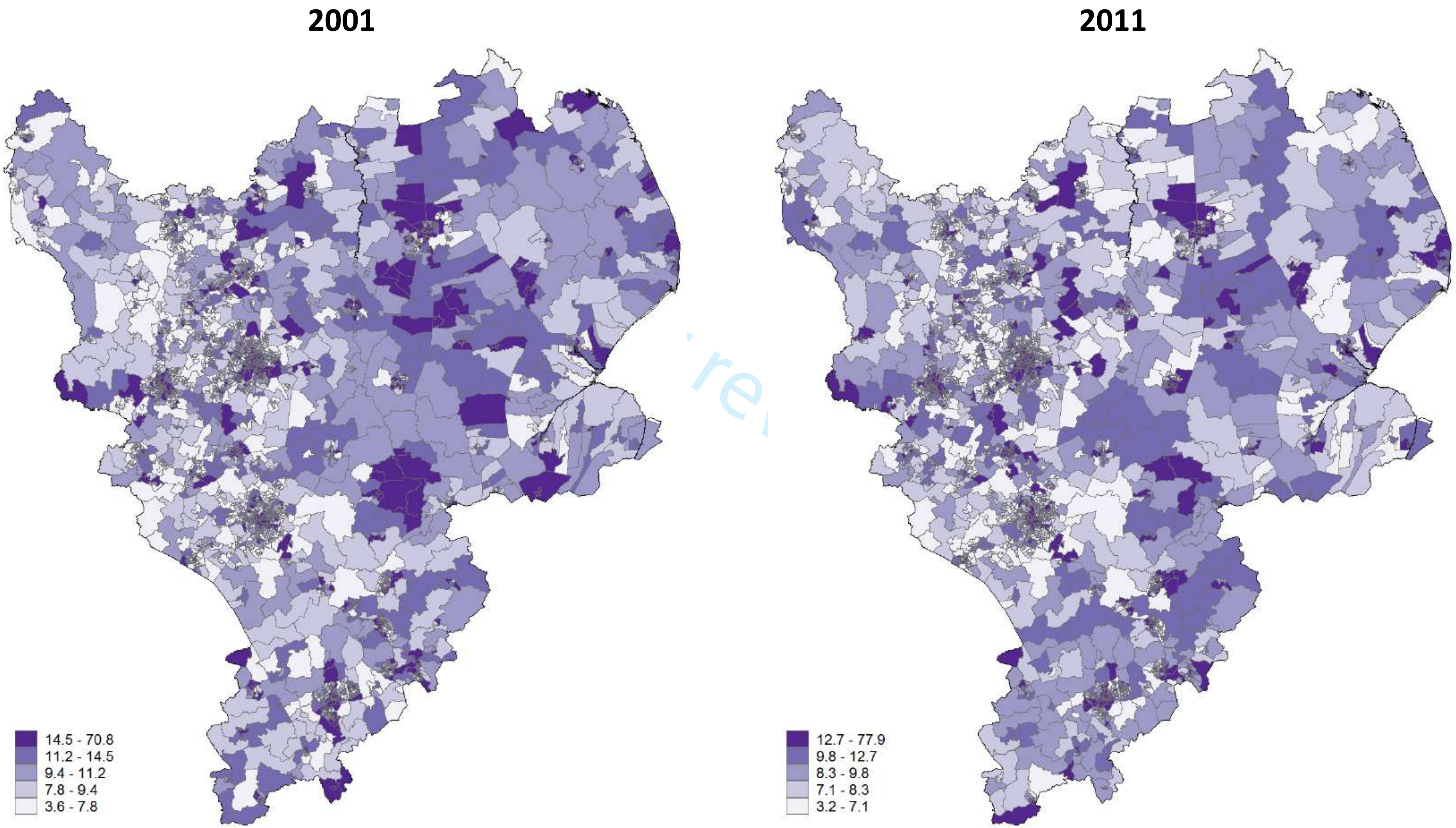

West Midlands, Population Turnover % of population

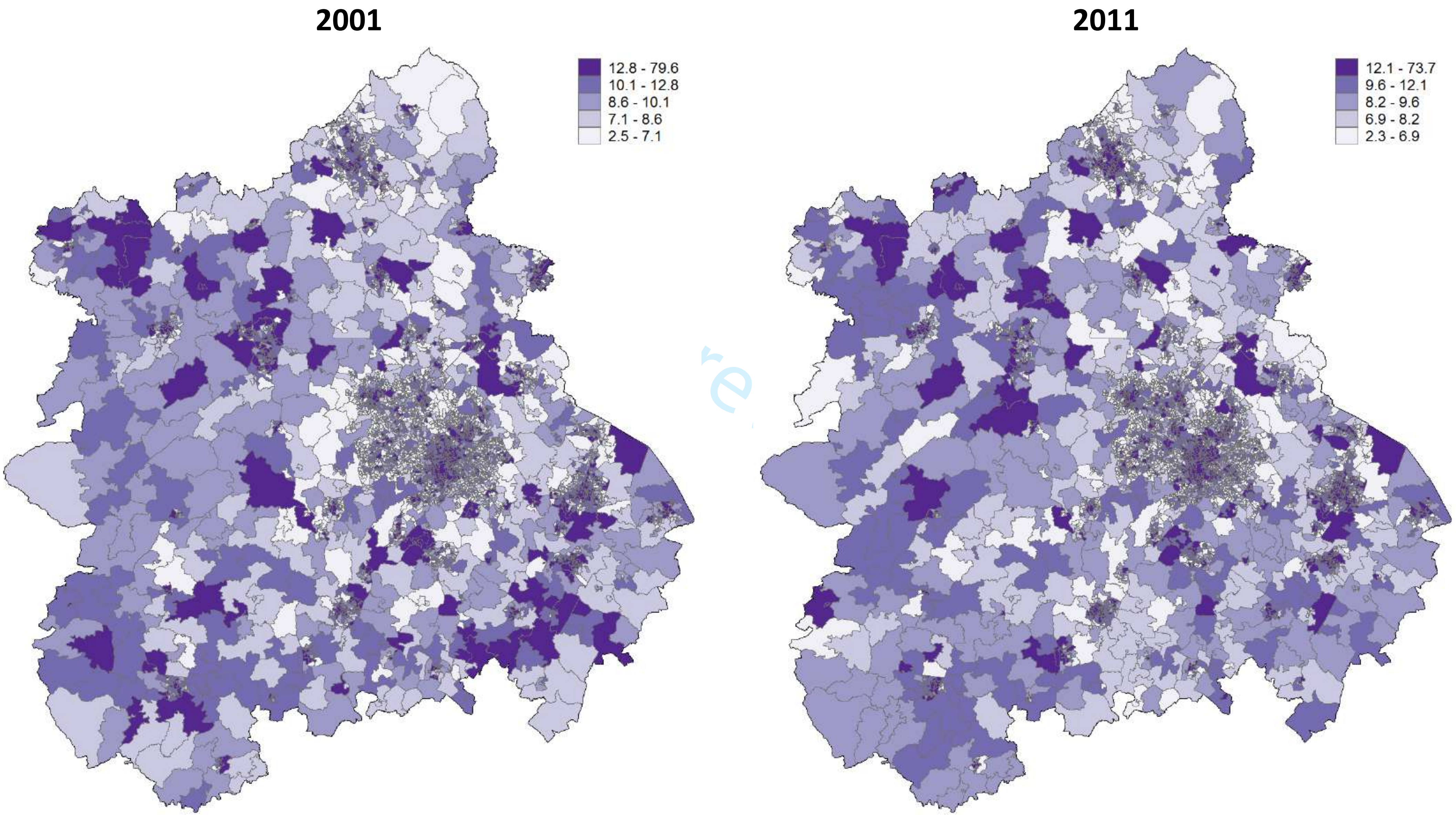

East of England, Population Turnover % of population

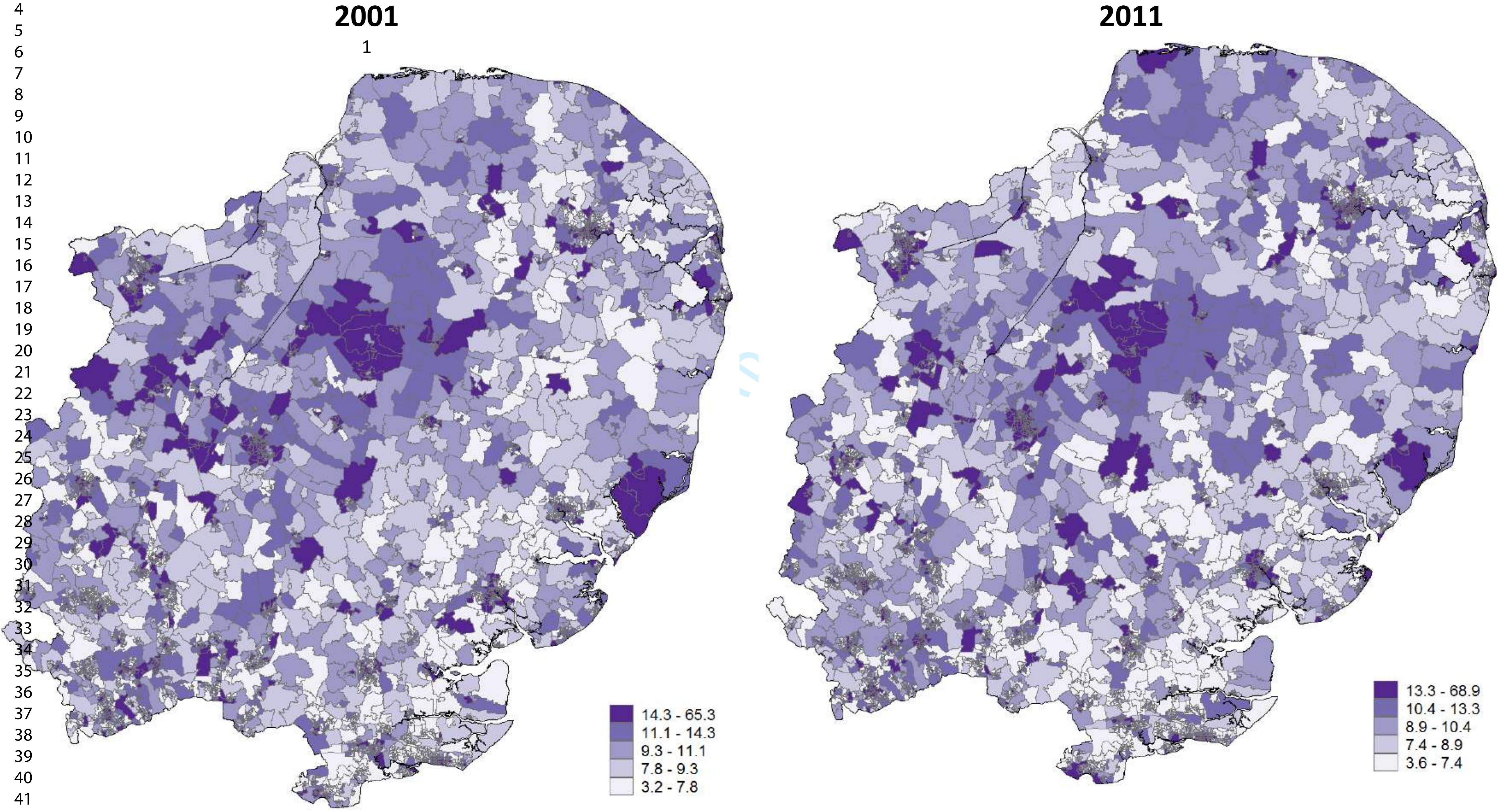

London, Population Turnover % of population

2001

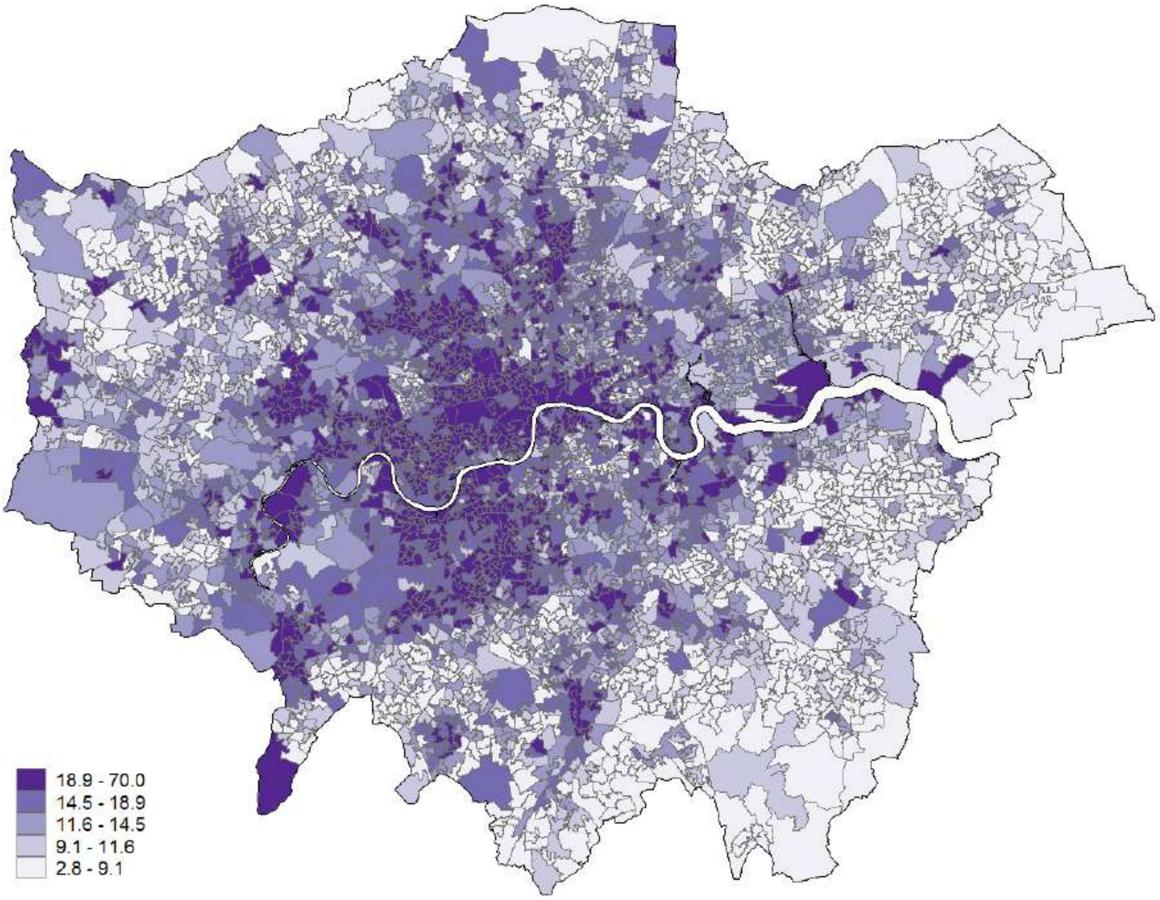

2011

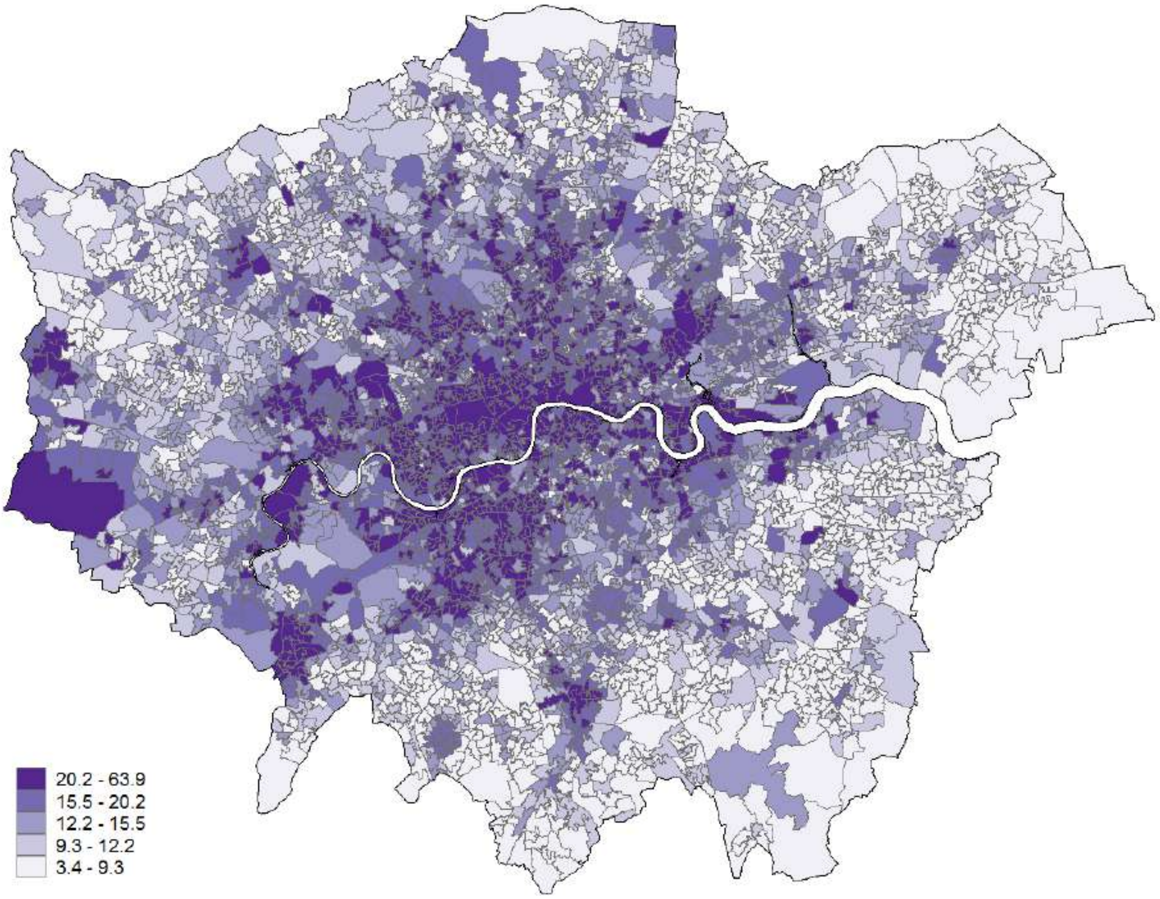

South East Coast, Population Turnover % of population

2001

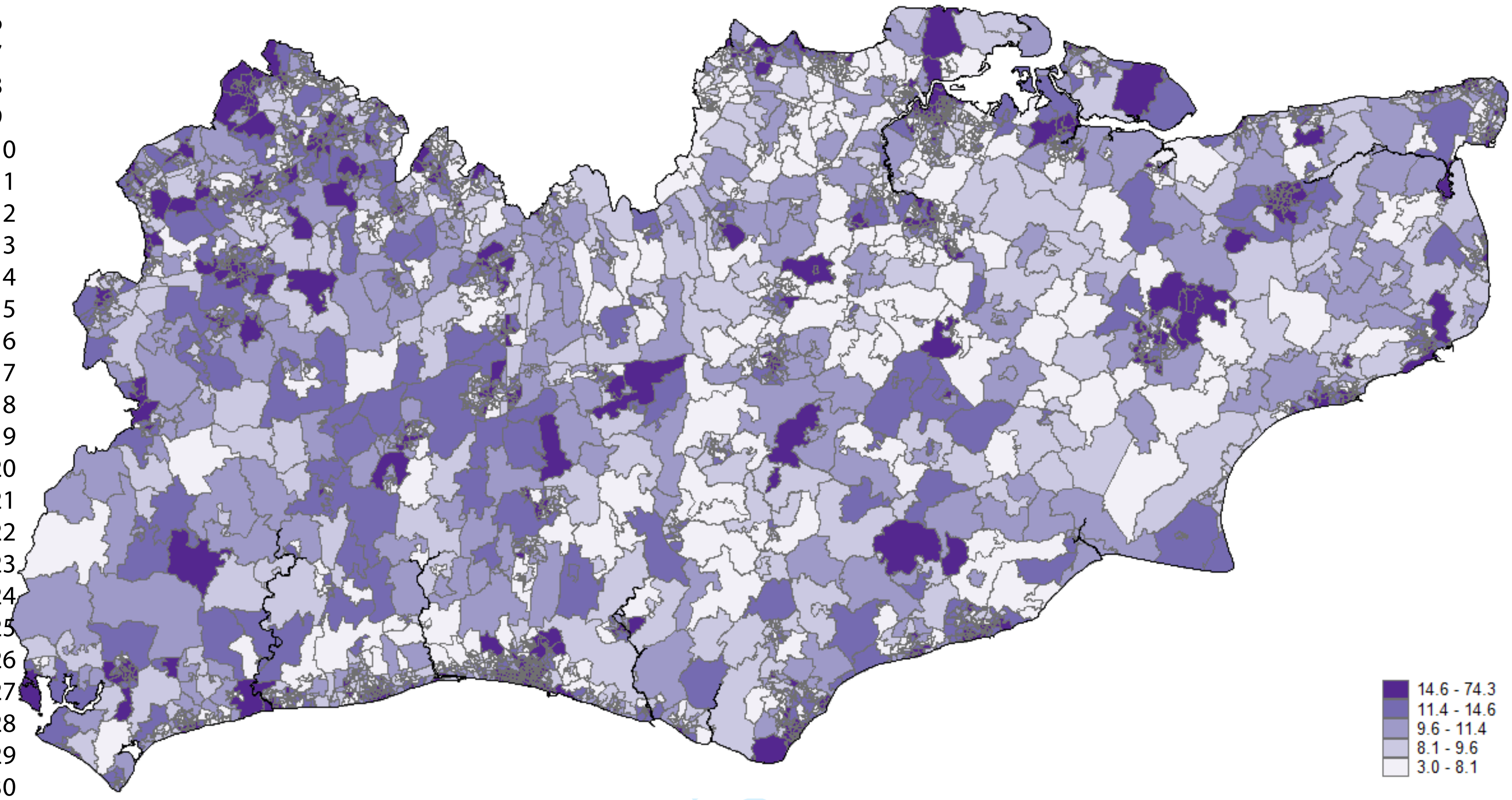

2011

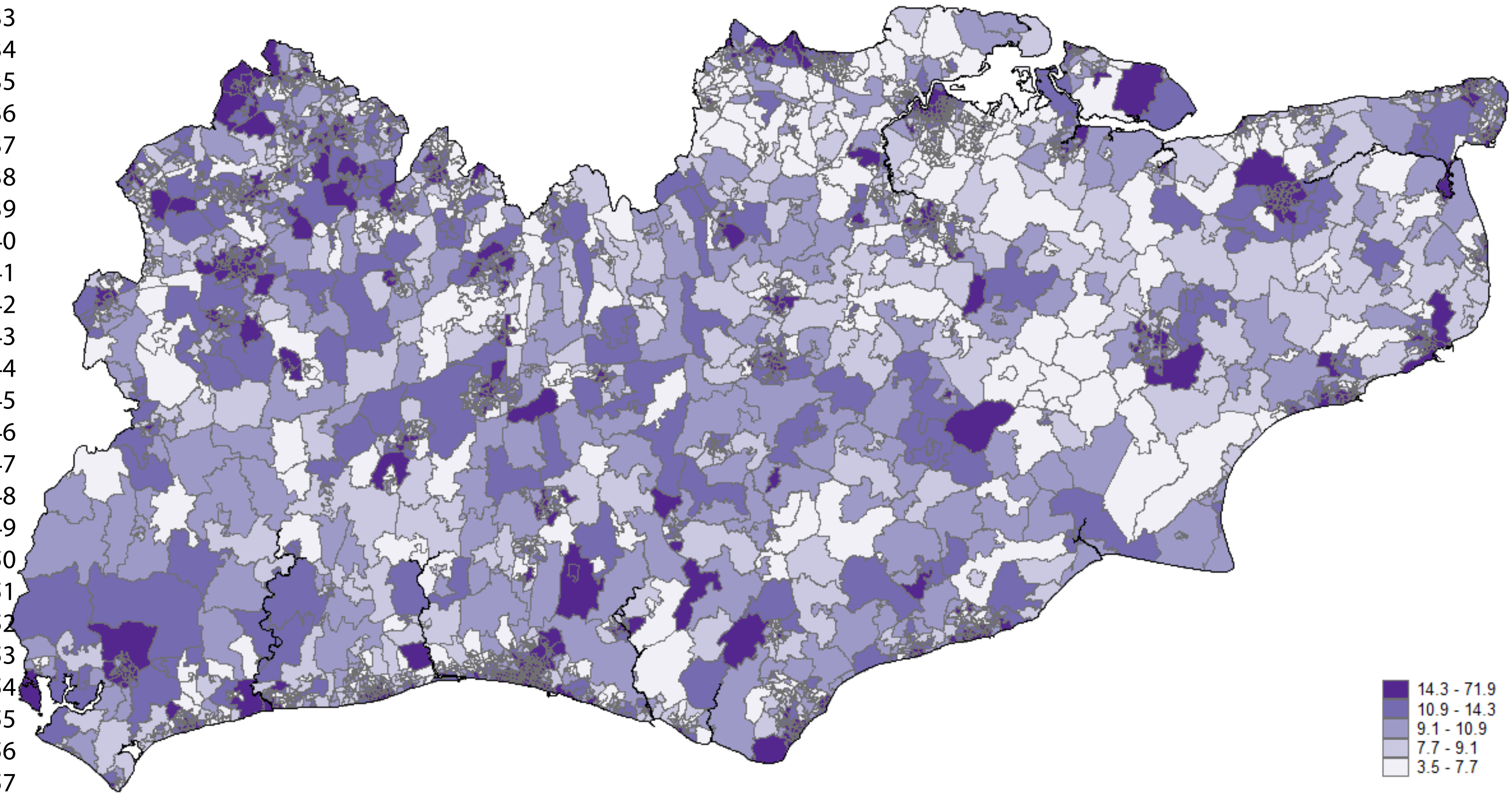

South Central, Population Turnover % of population

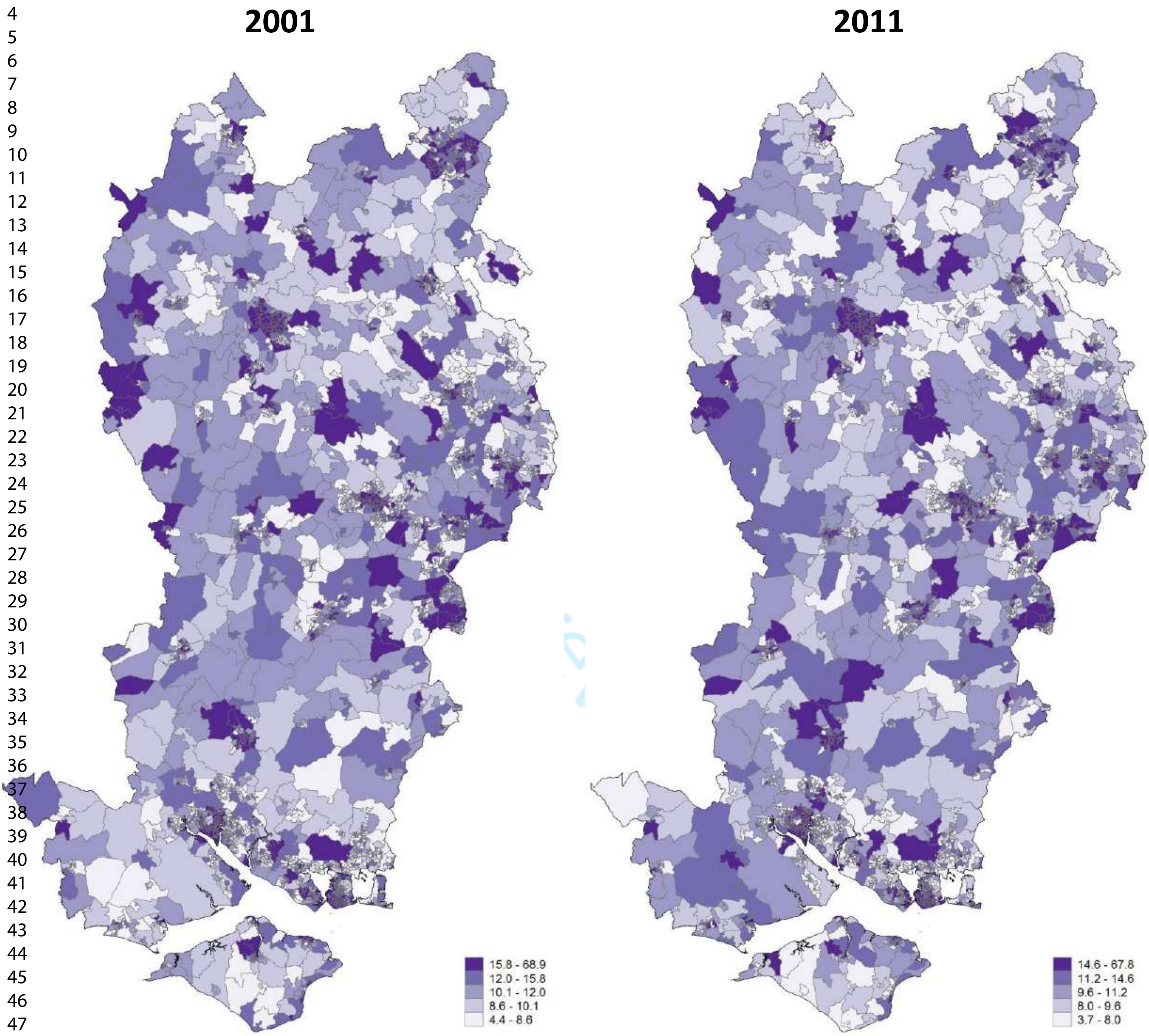

South West, Population Turnover % of population

2001

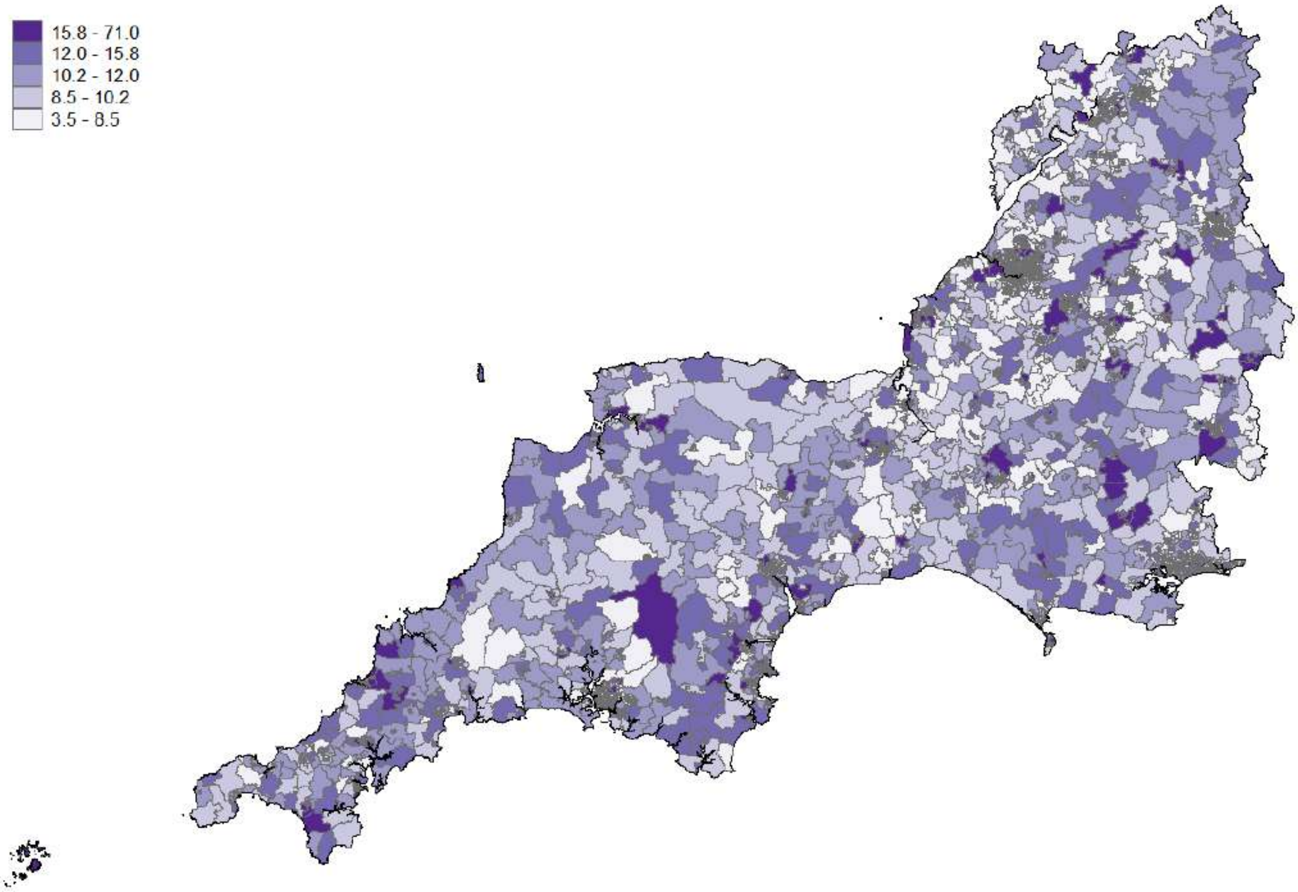

2011

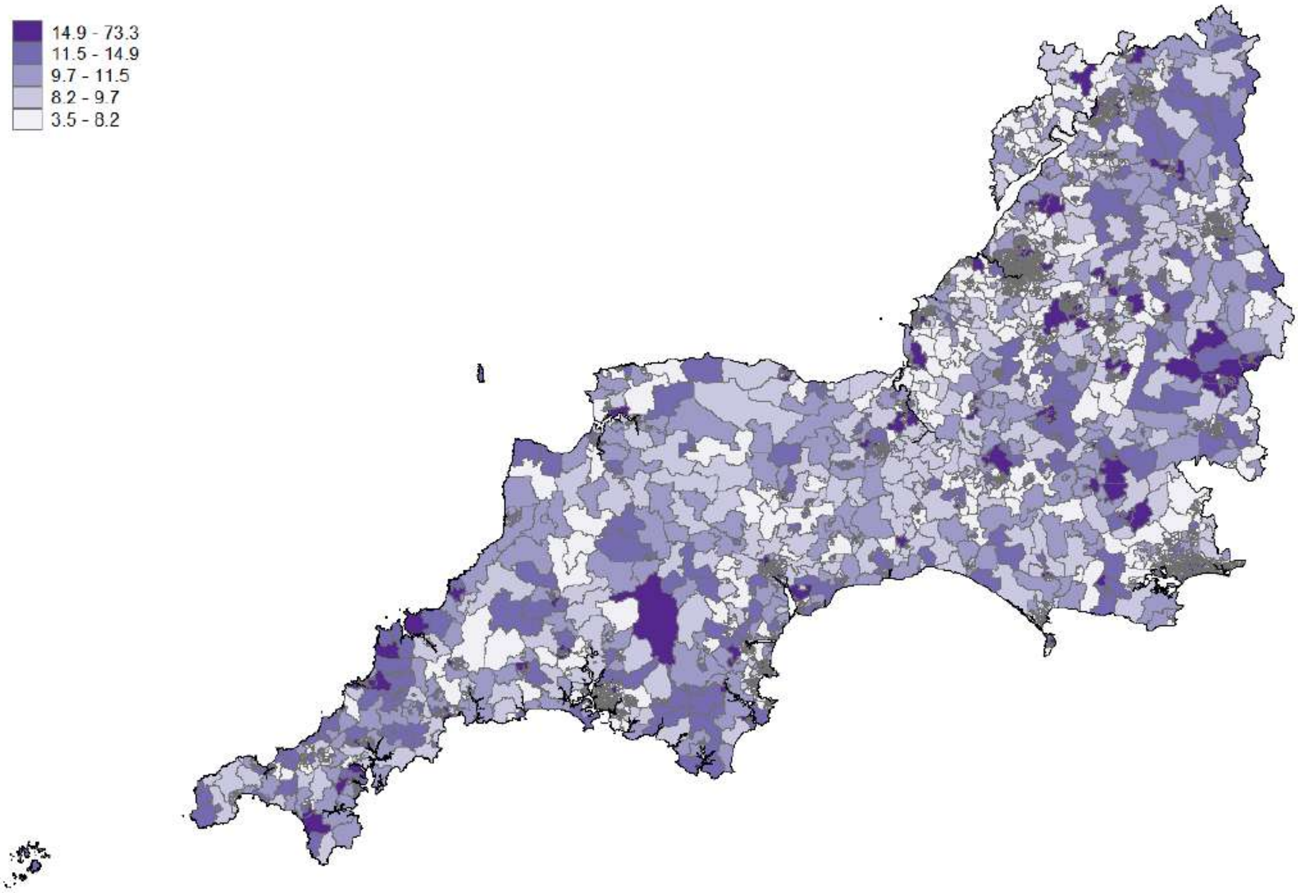

STROBE Statement—checklist of items that should be included in reports of observational studies

	Item No	Recommendation
**Title and abstract**	1	(*a*) Indicate the study's design with a commonly used term in the title or the abstract **p.1**
		(*b*) Provide in the abstract an informative and balanced summary of what was done and what was found **p.2**
**Introduction**		
Background/rationale	2	Explain the scientific background and rationale for the investigation being reported **p.3**
Objectives	3	State specific objectives, including any prespecified hypotheses **p.4**
**Methods**		
Study design	4	Present key elements of study design early in the paper
Setting	5	Describe the setting, locations, and relevant dates, including periods of recruitment, exposure, follow-up, and data collection **p.4**
Participants	6	(*a*) *Cohort study*—Give the eligibility criteria, and the sources and methods of selection of participants. Describe methods of follow-up **p.5** *Case-control study*—Give the eligibility criteria, and the sources and methods of case ascertainment and control selection. Give the rationale for the choice of cases and controls **NA** *Cross-sectional study*—Give the eligibility criteria, and the sources and methods of selection of participants **NA**
		(*b*) *Cohort study*—For matched studies, give matching criteria and number of exposed and unexposed **NA** *Case-control study*—For matched studies, give matching criteria and the number of controls per case **NA**
Variables	7	Clearly define all outcomes, exposures, predictors, potential confounders, and effect modifiers. Give diagnostic criteria, if applicable **p.4**
Data sources/ measurement	8*	For each variable of interest, give sources of data and details of methods of assessment (measurement). Describe comparability of assessment methods if there is more than one group **p.4**
Bias	9	Describe any efforts to address potential sources of bias **NA**
Study size	10	Explain how the study size was arrived at **p.5**
Quantitative variables	11	Explain how quantitative variables were handled in the analyses. If applicable, describe which groupings were chosen and why **p.5**
Statistical methods	12	(*a*) Describe all statistical methods, including those used to control for confounding **p.5**
		(*b*) Describe any methods used to examine subgroups and interactions **NA**
		(*c*) Explain how missing data were addressed **NA**
		(*d*) *Cohort study*—If applicable, explain how loss to follow-up was addressed **NA** *Case-control study*—If applicable, explain how matching of cases and controls was addressed **NA** *Cross-sectional study*—If applicable, describe analytical methods taking account of sampling strategy **NA**
		(*e*) Describe any sensitivity analyses **NA**

Continued on next page

For peer review only

For peer review only - http://bmjopen.bmj.com/site/about/guidelines.xhtml

**Results**		
Participants	13*	(a) Report numbers of individuals at each stage of study—eg numbers potentially eligible, examined for eligibility, confirmed eligible, included in the study, completing follow-up, and analysed **NA**
		(b) Give reasons for non-participation at each stage **NA**
		(c) Consider use of a flow diagram **NA**
Descriptive data	14*	(a) Give characteristics of study participants (eg demographic, clinical, social) and information on exposures and potential confounders p.5
		(b) Indicate number of participants with missing data for each variable of interest **NA**
		(c) *Cohort study*—Summarise follow-up time (eg, average and total amount) **p.5**
Outcome data	15*	*Cohort study*—Report numbers of outcome events or summary measures over time **p.5**
		Case-control study—Report numbers in each exposure category, or summary measures of exposure **NA**
		Cross-sectional study—Report numbers of outcome events or summary measures **NA**
Main results	16	(*a*) Give unadjusted estimates and, if applicable, confounder-adjusted estimates and their precision (eg, 95% confidence interval). Make clear which confounders were adjusted for and why they were included **NA**
		(*b*) Report category boundaries when continuous variables were categorized **NA**
		(*c*) If relevant, consider translating estimates of relative risk into absolute risk for a meaningful time period **NA**
Other analyses	17	Report other analyses done—eg analyses of subgroups and interactions, and sensitivity analyses **NA**
**Discussion**		
Key results	18	Summarise key results with reference to study objectives **p.5-p.6**
Limitations	19	Discuss limitations of the study, taking into account sources of potential bias or imprecision. Discuss both direction and magnitude of any potential bias **p.7**
Interpretation	20	Give a cautious overall interpretation of results considering objectives, limitations, multiplicity of analyses, results from similar studies, and other relevant evidence **p.8**
Generalisability	21	Discuss the generalisability (external validity) of the study results **p.9**
**Other information**		
Funding	22	Give the source of funding and the role of the funders for the present study and, if applicable, for the original study on which the present article is based **p.11**

*Give information separately for cases and controls in case-control studies and, if applicable, for exposed and unexposed groups in cohort and cross-sectional studies.

Note: An Explanation and Elaboration article discusses each checklist item and gives methodological background and published examples of transparent reporting. The STROBE checklist is best used in conjunction with this article (freely available on the Web sites of PLoS Medicine at http://www.plosmedicine.org/, Annals of Internal Medicine at http://www.annals.org/, and Epidemiology at http://www.epidem.com/). Information on the STROBE Initiative is available at www.strobe-statement.org.

For peer review only

For peer review only - http://bmjopen.bmj.com/site/about/guidelines.xhtml

BMJ Open

Spatial distribution and temporal trends in social fragmentation in England, 2001 to 2011: a national study

Journal:	*BMJ Open*
Manuscript ID	bmjopen-2018-025881.R3
Article Type:	Research
Date Submitted by the Author:	15-Nov-2018
Complete List of Authors:	Grigoroglou, Christos; University of Manchester, Centre for Primary Care<br>Munford, Luke; University of Manchester, Manchester Centre for Health Economics<br>Webb, Roger; University of Manchester, Centre for Suicide Prevention; Centre for Mental Health and Risk<br>Kapur, Navneet; University of Manchester, Centre for Suicide Prevention, Centre for Mental Health and Risk<br>Ashcroft, Darren; University of Manchester, Centre for Pharmacoepidemiology and Drug Safety<br>Kontopantelis, Evangelos; University of Manchester, Division of Population Health, Health Services Research and Primary Care
**Primary Subject Heading**:	Epidemiology
Secondary Subject Heading:	Mental health, Health services research
Keywords:	MENTAL HEALTH, EPIDEMIOLOGY, Organisation of health services < HEALTH SERVICES ADMINISTRATION & MANAGEMENT, SOCIAL MEDICINE

SCHOLARONE™
Manuscripts

Spatial distribution and temporal trends in social fragmentation in England, 2001 to 2011: a national study

Christos Grigoroglou[1,8], Luke Munford[2,8], Roger T. Webb[3,6,8], Nav Kapur[4,6,7,8], Darren M. Ashcroft [5,6,8], Evangelos Kontopantelis[1,8]

[1] NIHR School for Primary Care Research, Centre for Primary Care, Division of Population Health, Health Services Research and Primary Care, University of Manchester, Manchester, UK
[2] Centre for Health Economics, Division of Population Health, Health Services Research and Primary Care, University of Manchester, Manchester, UK
[3] Centre for Mental Health and Safety, Division of Psychology & Mental Health, University of Manchester, Manchester, UK
[4] Centre for Suicide Prevention, Division of Psychology and Mental Health, University of Manchester, Manchester, UK
[5] Centre for Pharmacoepidemiology and Drug Safety, School of Health Sciences, Faculty of Biology, Medicine and Health, , University of Manchester, Manchester Academic Health Sciences Centre (MAHSC), Manchester, UK
[6] NIHR Greater Manchester Patient Safety Translational Research Centre, Manchester Academic Health Sciences Centre (MAHSC), Manchester, UK
[7] Greater Manchester Mental Health Trust, Manchester, UK
[8] Manchester Academic Health Sciences Centre [MAHSC], Manchester, UK

Correspondence to:

Christos Grigoroglou
E-mail: christos.grigoroglou@postgrad.manchester.ac.uk
Telephone number: 0161-2750743
5th Floor, Williamson Building, University of Manchester,
Manchester, UK, M13 9PL

Word count: 3,414
5 Figures

For peer review only

Objective: Social fragmentation is commonly examined in epidemiological studies of mental illness as high levels of social fragmentation are often found in areas with high prevalence of mental illness. In this study we examine spatial and temporal patterns of the index of social fragmentation and its underlying indicators in England over time.

Setting: Data for social fragmentation and its underlying indicators were analysed over the decennial Censuses (2001–2011) at a small-area geographical level (mean of 1500 people). Degrees of social fragmentation and temporal changes were spatially visualised for the whole of England and its 10 administrative regions. Spatial clustering was quantified using Moran's I; changes in correlations over time were quantified using Spearman's ranking correlation.

Results: Between 2001 and 2011 we observed a strong persistence for social fragmentation nationally (Spearman's r=0.94). At the regional level, modest changes were observed over time, but marked increases were observed for two of the four social fragmentation underlying indicators, namely single people and those in private renting. Results supported our hypothesis of increasing spatial clustering over time. Moderate regional variability was observed in social fragmentation, its underlying indicators and their clustering over time.

Conclusion: Patterns of social fragmentation and its underlying indicators persisted in England which seem to be driven by the large increases in single people and those in private renting. Policies to improve social cohesion may have an impact the lives of persons who experience mental illness. The spatial aspect of social fragmentation can inform the targeting of health and social care interventions, particularly in areas with strong social fragmentation clustering.

Keywords: social fragmentation, mental health, clustering, inequalities, spatial autocorrelation

For peer review only - http://bmjopen.bmj.com/site/about/guidelines.xhtml

Strengths and Limitations of the study
➢ This study utilises data over two time points for the whole of England and is the first to describe spatial and temporal patterns of social fragmentation.
➢ Spatial aspects of social fragmentation can inform organisation of mental health services and social interventions that aim to enhance social support, particularly in areas where social fragmentation is high and is spatially clustered
➢ Over the last two decennial Censuses (2001 and 2011), the index of social fragmentation in England strongly persisted at a small-area geographical level while its clustering and its underlying indicators increased over time.
➢ Over time social fragmentation remained relatively stable, but we found marked increases in the percentage of single people and the percentage of those living in private rented accommodation.
➢ Even though census data are considered of high quality, people's perception of social fragmentation may not be fully captured in the census while demographic factors may capture social fragmentation in some areas but not in others.

For peer review only - http://bmjopen.bmj.com/site/about/guidelines.xhtml

INTRODUCTION

The impact of local area characteristics, such as material deprivation, on health is well established. [1 2] However, similar to socioeconomic position, the social networks and the amount of social support in a neighbourhood may also partially determine an individual's health. [3-6] In this context, an area-level characteristic that has been the focus of much investigation in neighbourhood health research is social fragmentation. The index of social fragmentation measures lack of social cohesion, and it is used to define areas with a breakdown or absence of social capital, and was originally created to measure the non-economic deprivation aspects of areas. [7] Social fragmentation is usually measured using census data which offer a great potential for the design and implementation of mental health strategies when individual level data are not available.

Social fragmentation is distinct conceptually from deprivation, since fragmentation is a feature of household composition and demographic structure and is not intrinsically linked to socioeconomic position.[8] However, the two measures tend to be positively correlated as they are both concentrated in large towns and cities. [8 9] Thus, many urban localities are both deprived and socially fragmented, although the two phenomena do not always co-exist. Several studies suggests that fragmented communities provide less stable social institutions and social bonds, although maintaining stable institutions and bonds within the communities can contribute substantially to the creation of shared identities, persistence of social relations, promotion of healthy behaviours and good physical and mental health for the population. [10-14]

Social fragmentation has received less attention from researchers than deprivation, despite the fact that it can aid the understanding of mechanisms via which the wider social environment influences mental health. [15] Research on adults has shown that social fragmentation is more closely related to mental health than physical health outcomes, independent of material deprivation and individual-level risk factors. [7 16] In the context of mental health, social fragmentation has been linked with suicide and non-fatal self-harm, [7 17-19] mental disorders, [4 11 20] psychiatric health service use, [21] first admission for psychosis, [20-22] psychological distress, [3 11] and schizophrenia. [15 23] Nonetheless, physical illness outcomes do not appear to be as clearly linked to social fragmentation. [18 24 25]

In the UK, an index of social fragmentation was developed to measure neighbourhood-level conditions which affect lack of opportunities for social integration. [7] The index was originally based on indicators of population turnover, percentage of married people, percentage of the population in private renting and percentage of one person households using data from the UK Census. The rationale for the selection of the underlying variables used for the calculation of the index was that several studies, both at ecological and individual level,

For peer review only - http://bmjopen.bmj.com/site/about/guidelines.xhtml

have found raised mental illness prevalence in highly urbanised areas with high population turnover, many non-family households, and reduced social cohesion. [26]

Social fragmentation in a population is likely to have profound effects on its health and wellbeing, and yet it is incompletely examined and reported in the existing literature, and a number of important research questions remain unanswered. First, how strongly clustered is social fragmentation? Second, how strongly does social fragmentation cluster and change at regional level over two time points? Third, does social fragmentation persist over time? Answering all three questions is important to inform the organisation of healthcare services and to target social and healthcare interventions, especially for mental health for which social fragmentation appears to be a salient risk factor. In this study, we temporally and spatially describe the properties of social fragmentation in England and within each of the 10 administrative English regions and make comparisons across them. Finally, we quantify the persistence of social fragmentation over the study period.

METHODS

Data sources

To derive the index of social fragmentation we accessed decennial Census data from the Nomis website [27] on: (1) One person households, % of all Census households; (2) single people (including widowed, divorced and separated people), % all usual residents aged 16 or over; (3) Population turnover (percentage of the population that moved into the area from within the UK and from outside the UK), % Census population in the year preceding the census (4) Households in private renting, % of all Census households. Our analysis was conducted at a small-area geographical level, namely the Lower Layer Super Output Area (LSOA). LSOAs are geographical areas with a mean population of 1,500 people. All data were available at the LSOA level except for the population turnover data which were available at the output area level (OA). OAs provide the finest spatial scale at which the Census is enumerated. We assigned the OA level data to the LSOA level using lookup tables provided by the Office for National Statistics (ONS). More details on the data used to derive each social fragmentation underlying indicator are provided in the Supplementary appendix 1.

The social fragmentation index for each LSOA was calculated by adding the unweighted z scores for each of the four characteristics into a composite score in each census year pooled. We used two time points for the index of social fragmentation, i.e. 2001 and 2011 over the two decennial Censuses. Across LSOAs the scores for the index ranged from -6.42 to 27.57 for 2001 and from -7.09 to 25.79 for 2011, with the upper boundaries indicating the most socially fragmented areas and the lower boundaries the most socially cohesive areas. Since the data used to derive the index in 2001 were reported using 2001 LSOAs, we

For peer review only - http://bmjopen.bmj.com/site/about/guidelines.xhtml

used a weighted means algorithm to assign the data for each underlying indicator to 2011 LSOAs using mid-year population estimates that were obtained from ONS. [28] A detailed description on how we attributed 2001 LSOA data to 2011 LSOAs is provided in the supplementary appendix 1. We calculated the z-scores of all social fragmentation underlying indicators for each of the two time points of interest. Finally, we obtained data on LSOA rural/urban classification at the 2011 LSOA.[29]

Analyses

Our outcome of interest was the index of social fragmentation and its four underlying indicators measured separately at the 2001 and 2011 national Censuses. We visualised temporal changes in social fragmentation and its underlying indicators with the use of spatial maps for all of England and each of the 10 English regions/strategic health authorities (SHA) (North East, North West, Yorkshire & the Humber, East Midlands, West Midlands, East of England, London, South East Coast, South Central and South West), which were the highest level of organisation available in NHS England during the study period. To assess whether levels of spatial autocorrelation for social fragmentation changed over time we compared the values of Moran's I [30] in the two time points for England and each English region to allow within-England comparisons. The measure can identify the presence or absence of spatial clusters while accounting for the multi-dimensional and multi-directional nature of spatial autocorrelation. Under a random spatial pattern a higher than expected value of Moran's I for social fragmentation would indicate that areas with high levels of social fragmentation are clustered and also that LSOAs with high social fragmentation are bordered with LSOAs with similarly high levels of social fragmentation. We also investigated local indicators for spatial autocorrelation (LISA) [31] to identify local patterns of spatial associations. We also quantified the persistency of social fragmentation over the two time points by calculating Spearman's correlation between 2001 and 2011. To visualise and compare temporal changes in the mean social fragmentation levels across the 10 English regions, we plotted population-weighted box plots for the main outcome and its underlying indicators. Finally, we used population-weighted box plots to visualise and compare temporal changes in the social fragmentation levels between the 10 English regions. Analyses were performed with Stata v.14.1 and R v.3.3.1. Due to the size of the dataset, effectively the whole of England, statistical significance is irrelevant; observed associations of minimal strength would be statistically significant, and thus we focussed on effect sizes wherever possible.

RESULTS

Region-level characteristics of the index of social fragmentation at both time points are provided in Table 1. We found strong correlation for social fragmentation at the two time

For peer review only - http://bmjopen.bmj.com/site/about/guidelines.xhtml

points (rho=0.93), using Spearman's ranking correlation to account for the relative nature of the measure. We plotted spatial maps of social fragmentation for the whole of England and for each region for the two time points analysed. We created maps of social fragmentation from a pooled sample in 2001 and 2011 for the whole of England and London in order to facilitate comparisons between areas over time and we present those in Figure 1 and Figure 2 respectively. Furthermore, we present maps of social fragmentation and its underlying indicators at each time point for every region in supplementary Appendix 2. Temporal changes in social fragmentation for England and for all English regions are presented in Figure A in the supplementary appendix 1. We identified a pattern of stability with modest increases over time for regions in the North, and small decreases in social fragmentation for regions in the South. However, temporal changes of English regions for two of the four social fragmentation underlying indicators were much larger and they are presented in Figure 3.

Large changes in absolute levels of single person households and private renting were found in all regions. More specifically, in 2001 the average number of single persons of the total population was 38.4% while in 2011 the figure was 52.6% indicating a 31% increase. Similarly, in 2001 the average number of people in private renting was 8.5% of all English households while in 2011 this figure increased to 16.2% indicating a 62% increase. The largest increases in numbers of single people were found in the North East, North West and West Midlands while London had the highest levels of single people. Moreover, the largest increases for private renting were found in the North East, West Midlands and East Midlands while London had again the largest numbers of people living in private renting. For the other two social fragmentation underlying indicators, we found that the average number of one person households both nationally and regionally remained relatively stable. We also observed a modest decrease, both nationally and regionally (with the exception of London), in our measure of population turnover which reflects the levels of migration inside and outside areas. Finally, we observed great variability in levels of social fragmentation and its underlying indicators within regions at both time points. High levels of social fragmentation were concentrated in London, the North West and the South West. East England had the lowest levels of social fragmentation followed by West Midlands. There was a clear distinction between rural and urban areas, with the latter having far greater levels of social fragmentation.

Across the whole of England, Moran's I for social fragmentation was very low at 0.0449 in 2001 (95% CI 0.0447 to 0.0451) but by 2011 it had increased to 0.0794 (95% CI 0.0792 to 0.0797), indicating a small increase in spatial autocorrelation and clustering (Figure 4). We also observed marked regional variability in social fragmentation spatial autocorrelation levels over time (Figure 4). In both time points, the West Midlands had the lowest spatial autocorrelation in social fragmentation, followed by the East of England, while the South East and South West had the highest levels of spatial autocorrelation at both time points. In

For peer review only - http://bmjopen.bmj.com/site/about/guidelines.xhtml

contrast to increases in spatial autocorrelation across the country between 2001 and 2011, the South East, North East and Yorkshire & the Humber had decreasing levels of spatial autocorrelation over the two time points.

Spatial clustering also appears to have increased over time for all fragmentation underlying indicators nationally, although its levels remained low (Figures A1 and A2 in supplementary Appendix 1). For one person households we found very low regional clustering and small regional variation. For single persons and private renting regional variation in clustering was moderate and their levels were also low with the exception of the South East and South West. Finally, for population turnover we observed the greatest increases of spatial autocorrelation both nationally and regionally but levels of regional clustering were generally low with the exception of South West whilst regional variability was moderate. We provide graphs and discuss the results from the LISA analysis for local spatial autocorrelation in the supplementary appendix 1 (Figures B1 and B2).

DISCUSSION

Between 2001 and 2011, we found evidence of strong persistence of social fragmentation in England at small-area level. Regional changes over time were modest for social fragmentation, which is not surprising considering the large population denominators and the relative nature of the measure. Similarly, we observed modest increases over time for one person households and population turnover. However, changes over time for single persons and private renting were much greater, with substantial increases in both underlying indicators across regions, and these specific influences appear to have largely driven the heightened levels of social fragmentation overall. Furthermore, our findings provide evidence of increasing clustering for social fragmentation and its underlying indicators which, in conjunction with the regional variation observed between the two time points, indicates that areas in the South of England (including London) appear much more clustered than the North. Finally, we observed increased clustering for all underlying indicators of social fragmentation over the two time points.

Strengths and limitations of the study

The main strength of this study lies in its large dataset for the whole of England (53 million people in 2011) over two time points across 32,844 LSOAs, which we utilised to investigate spatial and temporal patterns of social fragmentation. To our knowledge, it is the first study of its kind to provide an insight into the temporal persistence of social fragmentation and its clustering.

For peer review only - http://bmjopen.bmj.com/site/about/guidelines.xhtml

For peer review only

However, some limitations exist. First, the extent to which the index captures social fragmentation may vary across the country as the demographic factors that we used may not in themselves be valid indicators of social fragmentation, or they may adequately measure social fragmentation in some areas but not in others. [22] However, the measure has been reported widely in the literature and on this basis social fragmentation has been identified as an important predictor of mental health outcomes such as suicide. Second, Census questions do not usually directly elicit the presence or quality of neighbourhood social ties or institutions, or people's relationship to their neighbourhood. Therefore, we might have failed to capture people's perception of social fragmentation through the Census data. Nevertheless, we used a measure capturing compositional aspects of neighbourhood which appear to be related to the capacity of a neighbourhood to act collectively, and is also important for the construction and maintenance of social ties and institutions. Third, whilst Census data is recognised to be of a high standard, some unobserved heterogeneity persists. [32] For example, LSOAs are defined from administrative information and this may disguise local effects as contextual characteristics within administrative neighbourhoods are not homogeneous across LSOAs. [33] An alternative approach would require the use of non-administrative data to define levels, but the Census is currently the only source from which the data on the fragmentation underlying indicators is available. Fourth, for the underlying indicator 'single people' we used data on legal marital status in 2001 and 2011 and registered civil partnership status in 2011. However, the population base for this indicator is not restricted to the household population and this has implications for the total numbers of single people. For example, some of those who are single, widowed or divorces could be living in institutions as well as cohabiting unions which will result in counting cohabitants as 'single, widowed or divorced' in the calculation of the indicator. Fifth, there were boundary changes following the 2011 Census that may have affected our findings, [34] but only 2.5% of LSOAs were affected and we developed an algorithm to make reasonable population weighted-based estimates for these localities, depending on whether they merged, split or some other change occurred. Finally, the index of social fragmentation may serve as a suitable tool to inform allocation of mental health resources in adults, although recent evidence suggests that social fragmentation may not have an effect on children's mental health.[35]

Interpretation of findings

A previous study showed that between 1971 and 2001, levels of social fragmentation increased steadily with large increases between 1981 and 1991 and modest increases between 1991 and 2001. Our results indicate that this trend was reversed in 2011 as we found a small decrease in the overall index of social fragmentation between 2001 and 2011. [36] Furthermore, our results suggest that urbanisation drives the high persistence of social fragmentation in England as we found that levels of social fragmentation and its underlying

For peer review only - http://bmjopen.bmj.com/site/about/guidelines.xhtml

indicators were consistently higher in urban areas. This may be because urban areas are chosen by diverse social and cultural groups and living in these areas may lead to isolated spaces, lifestyles and problems of social integration. Furthermore, we found that the underlying indicators that drive the persistency of social fragmentation appear to be single people and households in private renting, a characteristic of urban environments, [37] as these were the underlying indicators with the largest increases (Figure 3).

In absolute terms, private renting was considerably increased in all regions and most notably London; a finding that pertains to unaffordable housing for first-time home buyers and less availability of social housing as a result of the 2008 financial crisis.[38] Similarly, all regions had higher levels of single people in 2011, indicating that numbers of young professionals, students and divorces have increased substantially over the decade. For the other two underlying indicators we did not observe any marked changes across regions, although levels of population turnover appeared to have decreased between the decennial Censuses. The increases we observed in the proportion of single people and those in private renting warrant implications for the prevalence of mental illness especially in urban centres. The private house market is characterised by poor housing conditions as opposed to social houses or owner occupation and this can have detrimental effects on mental health. [39] Furthermore, it is suggested that married individuals may have better mental health outcomes than single individuals. [40] We believe that these changes are certainly of concern as they may highlight the need for more effective social interventions to target socially fragmented areas in England.

Spatial clustering of neighbourhood social fragmentation appears to have increased over time in England while we observed moderate regional variations in levels of clustering despite the small changes in levels of social fragmentation across regions. Increased levels of spatial clustering for social fragmentation across and within regions can have implications for the planning and organisation of health services, as this would imply needs that are not uniformly distributed across a region with the presence of hot spots of high levels of mental illness prevalence, especially in urban areas. To address such spatial health inequalities, services may need to be redesigned locally [41] while infrastructure and spending for mental health needs to be weighted towards social fragmentation hot spots rather than uniformly distributed.

Conclusion

In the absence of individual-level data, area-level indicators are often the only source of information to investigate how factors associated with mental illness may contribute to population health, albeit when individual-level data are available and are fitted as covariates these area-level associations may be attenuated substantially. Even though, social

For peer review only - http://bmjopen.bmj.com/site/about/guidelines.xhtml

fragmentation is increasingly considered a determinant of mental illness as more epidemiological research emerges that investigates contextual effects on health. To improve mental health in the population, policymakers need to address both relational and structural issues, perhaps by investing in reorganisation of mental healthcare. Solving the problems of cohesion and integration needs to be seen as a collective responsibility across central and local government and all the local agencies. Policies needs to address social and educational inequalities by strengthening the economic well-being of areas by lowering local unemployment rates, encouraging populations to bring social support in the communities and encouraging education of the population, which are key strategies for healthy communities. Communities need to work towards tightening social networks and social bonds to help with mental illness prevalence and suicide prevention, for example by promoting participation in charity, voluntary or community groups and civic engagement. The spatial aspect of social fragmentation is often overlooked, but it can provide vital information for the effective organisation of health services and targeting of health or social interventions. The nature of fragmentation and the mechanisms underlying its association with mental illness are of increasing interest as the population gets older and the health care costs associated with mental illness escalate in industrialised countries.

For peer review only

Acknowledgments

We would like to thank the Office of National Statistics for the wealth of information they have collected and systematically organised, which made this study possible.

Contributors

EK, CG, DA and LM designed the study. CG extracted the data from all sources, performed the analyses and drafted the manuscript. EK, RW, DA, LM, NK critically revised the manuscript. CG is the guarantor of this work and, as such, had full access to all the data in the study and takes responsibility for the integrity of the data and the accuracy of the data analysis.

Funding

This study is funded by the National Institute for Health Research School for Primary Care Research (NIHR SPCR), through CG's PhD. This report is independent research by the National Institute for Health Research. LM acknowledges financial support from the MRC Skills Development Fellowship (MR/N015126/1). The views expressed in this publication are those of the authors and necessarily those of the NHS, the National Institute for Health Research, the Department of Health, or the MRC.

Competing interests

None declared.

Provenance and peer review

Not commissioned; externally peer reviewed

Data sharing agreement

The data used in this study are freely available and the authors are happy to share in an organised and cleaned final dataset.

Ethical approval and Patient and Public Involvement

Not applicable. The study uses freely reported data aggregates at low geography levels. No patients or members of the public were involved in the study.

Open access

This is an Open Access article distributed in accordance with the terms of the Creative Commons Attribution (CC BY 4.0) license, which permits others to distribute, remix, adapt and build upon this work, for commercial use, provided the original work is properly cited. See: http://creativecommons.org/licenses/by/4.0/ © Article author(s) (or their employer(s) unless otherwise stated in the text of the article) 2018. All rights reserved. No commercial use is permitted unless otherwise expressly granted.

For peer review only - http://bmjopen.bmj.com/site/about/guidelines.xhtml

Table 1 Temporal changes in social fragmentation metrics across regions, 2001 - 2011

	North East	North West	Yorkshire and Humber	East Midlands	West Midlands	East England	London	South East Coast	South Central	South West	England
Social Fragmentation 2001											
No. of LSOAs	1,656	4,459	3,293	2,732	3,482	3,550	4,765	2,750	2,569	3,226	32,482
25th Centile	-2.32	-2.34	-2.31	-2.59	-2.59	-2.50	-0.46	-2.37	-2.51	-2.09	-2.26
Median	-0.79	-0.63	-0.87	-1.34	-1.19	-1.27	2.05	-1.09	-1.24	-0.88	-0.75
75th Centile	0.82	1.45	0.91	0.47	0.44	0.37	4.45	0.86	0.76	0.89	1.46
Weighted mean	-0.29	-0.06	-0.16	-0.55	-0.71	-0.68	2.20	-0.21	-0.33	-0.18	0.03
Standard deviation	3.26	3.50	3.50	3.24	2.94	2.75	3.28	3.24	3.16	3.28	3.32
Social Fragmentation 2011											
No. of LSOAs	1,657	4,497	3,317	2,774	3,487	3,614	4,835	2,773	2,609	3,281	32,844
25th Centile	-2.16	-2.31	-2.30	-2.64	-2.45	-2.59	-0.64	-2.50	-2.63	-2.17	-2.29
Median	-0.58	-0.51	-0.78	-1.27	-1.01	-1.30	1.58	-1.19	-1.27	-0.86	-0.70
75th Centile	0.97	1.62	1.07	0.58	0.65	0.39	3.98	0.76	0.62	1.02	1.43
Weighted mean	-0.01	0.16	0.04	-0.43	-0.45	-0.67	1.83	-0.25	-0.41	0.02	0.09
Standard deviation	3.13	3.29	3.37	3.22	2.82	2.81	3.56	3.34	3.28	3.33	3.37

For peer review only

Figure 1 Changes in overall social fragmentation for England, 2001 (left) – 2011 (right) (pooled sample)

Table 1 Temporal changes in social fragmentation metrics across regions, 2001 – 2011 (pooled sample)

Figure 2 Changes in overall social fragmentation for London, 2001 (left) – 2011 (right)

Figure 3 Percentage of single people,one person households, private renting and population turnover for English regions, over time (* Red line indicates mean for England in 2011)

Figure 4 Spatial Autocorrelation with Moran's I for Social Fragmentation by region, over time

For peer review only

For peer review only - http://bmjopen.bmj.com/site/about/guidelines.xhtml

References

1. Pickett KE, Pearl M. Multilevel analyses of neighbourhood socioeconomic context and health outcomes: a critical review. *J Epidemiol Ccommunity Health* 2001;55(2):111-22.
2. Stafford M, Marmot M. Neighbourhood deprivation and health: does it affect us all equally? *Int J Epidemiol* 2003;32(3):357-66.
3. Fagg J, Curtis S, Stansfeld SA, Cattell V, Tupuola AM, Arephin M. Area social fragmentation, social support for individuals and psychosocial health in young adults: evidence from a national survey in England. *Soc Sci Med* 2008;66(2):242-54.
4. Stafford M, De Silva M, Stansfeld S, Marmot M. Neighbourhood social capital and common mental disorder: testing the link in a general population sample. *Health Place* 2008;14(3):394-405.
5. Subramanian SV, Lochner KA, Kawachi I. Neighborhood differences in social capital: a compositional artifact or a contextual construct? *Health Place* 2003;9(1):33-44.
6. Veenstra G, Luginaah I, Wakefield S, Birch S, Eyles J, Elliott S. Who you know, where you live: social capital, neighbourhood and health. *Soc Sci Med* 2005;60(12):2799-818.
7. Congdon P. Suicide and parasuicide in London: A small-area study. *Urban Stud* 1996;33(1):137-58.
8. Congdon P. Commentary: Contextual effects: index construction and technique. *Int J Epidemiol* 2004;33(4):741-2.
9. Understanding patterns of deprivation. *Regional Trends* 2009;41(1):93-114.
10. Whitley E, Gunnell D, Dorling D, Smith GD. Ecological study of social fragmentation, poverty, and suicide. *BMJ* 1999;319(7216):1034-7.
11. Fagg J, Curtis S, Stansfeld S, Congdon P. Psychological distress among adolescents, and its relationship to individual, family and area characteristics in East London. *Soc Sci Med* 2006;63(3):636-48.
12. Fone D, Dunstan F, Lloyd K, Williams G, Watkins J, Palmer S. Does social cohesion modify the association between area income deprivation and mental health? A multilevel analysis. *Int J Epidemiol* 2007;36(2):338-45.
13. Evans J, Middleton N, Gunnell D. Social fragmentation, severe mental illness and suicide. *Soc Psychiatry Psychiatr Epidemiol* 2004;39(3):165-70.
14. Ivory VC, Collings SC, Blakely T, Dew K. When does neighbourhood matter? Multilevel relationships between neighbourhood social fragmentation and mental health. *Soc Sci Med* 2011;72(12):1993-2002.
15. Allardyce J, Boydell J. Review: the wider social environment and schizophrenia. *Schizophr Bull* 2006;32(4):592-8.
16. Ellen IG, Mijanovich T, Dillman K-N. Neighborhood Effects on Health: Exploring the Links and Assessing the Evidence. *J Urban Aff* 2001;23(3-4):391-408.
17. Collings S, Ivory V, Blakely T, Atkinson J. Are neighbourhood social fragmentation and suicide associated in New Zealand? A national multilevel cohort study. *J Epidemiol Community Health* 2009;63(12):1035-42.
18. Smith GD, Whitley E, Dorling D, Gunnell D. Area based measures of social and economic circumstances: cause specific mortality patterns depend on the choice of index. *J Epidemiol Community Health* 2001;55(2):149-50.
19. Corcoran P, Arensman E, Perry IJ. The area-level association between hospital-treated deliberate self-harm, deprivation and social fragmentation in Ireland. *J Epidemiol Community Health* 2007;61(12):1050-55.
20. Evans E, Hawton K, Rodham K. Factors associated with suicidal phenomena in adolescents: a systematic review of population-based studies. *Clin Psychol Rev* 2004;24(8):957-79.

For peer review only

For peer review only - http://bmjopen.bmj.com/site/about/guidelines.xhtml

21. Curtis S, Copeland A, Fagg J, Congdon P, Almog M, Fitzpatrick J. The ecological relationship between deprivation, social isolation and rates of hospital admission for acute psychiatric care: a comparison of London and New York City. *Health Place* 2006;12(1):19-37.
22. Allardyce J, Gilmour H, Atkinson J, Rapson T, Bishop J, McCreadie RG. Social fragmentation, deprivation and urbanicity: relation to first-admission rates for psychoses. *Br J Psychiatry* 2005;187:401-06.
23. van Os J, Driessen G, Gunther N, Delespaul P. Neighbourhood variation in incidence of schizophrenia. Evidence for person-environment interaction. *Br J Psychiatry* 2000;176:243-8.
24. Stjarne MK, Ponce de Leon A, Hallqvist J. Contextual effects of social fragmentation and material deprivation on risk of myocardial infarction--results from the Stockholm Heart Epidemiology Program (SHEEP). *International journal of epidemiology* 2004;33(4):732-41.
25. Stafford M, Gimeno D, Marmot MG. Neighbourhood characteristics and trajectories of health functioning: a multilevel prospective analysis. *Eur J Public Health* 2008;18(6):604-10.
26. Jarvis GK, Ferrence RG, Whitehead PC, Johnson FG. The ecology of self-injury: a multivariate approach. *Suicide Llife Threat Behav* 1982;12(2):90-102.
27. Office for National Statistics.Nomis official labour market statistics. 2013.
28. Office for National Statistics. Super Output Area mid-year population estimates for England and Wales, Mid-2011. 2016.
29. Bibby P, Shepherd J. Developing a new classification of urban and rural areas for policy purposes–the methodology. *London: DEFRA* 2004.
30. Moran PAP. Notes on Continuous Stochastic Phenomena. *Biometrika* 1950;37(1-2):17-23.
31. Anselin L. Local indicators of spatial association—LISA. *Geogr Anal* 1995;27(2):93-115.
32. Fewell Z, Smith GD, Sterne JAC. The impact of residual and unmeasured confounding in epidemiologic studies: A simulation study. *Am J Epidemiol* 2007;166(6):646-55.
33. Rehkopf DH, Buka SL. The association between suicide and the socio-economic characteristics of geographical areas: a systematic review. *Psychol Med* 2006;36(2):145-57.
34. Norman P, Rees P, Boyle P. Achieving data compatibility over space and time: creating consistent geographical zones. *Pop Space Place* 2003;9(5):365-86.
35. Flouri E, Midouhas E, Joshi H, Sullivan A. Neighbourhood social fragmentation and the mental health of children in poverty. *Health Place* 2015;31:138-45.
36. Dorling D, Vickers D, Thomas B, Pritchard J, Ballas D. Changing UK. *The way we live now. Sheffield: University of Sheffield* 2008.
37. Smith CW. 2011 Census Analysis: How Have Living Arrangements and Marital Status in England and Wales Changed Since 2001. *Office for National Statistics* 2011.
38. Kemp PA. Private Renting After the Global Financial Crisis. *Housing Stud* 2015;30(4):601-20.
39. Barnes M, Cullinane, C., Scott, S. & Silvester, S. . People living in bad housing: Numbers and health impacts. *UK: Shelter.* 2013.
40. Simon RW. Revisiting the relationships among gender, marital status, and mental health. *Am J Sociol* 2002;107(4):1065-96.
41. Lorant V, Thomas I, Deliege D, Tonglet R. Deprivation and mortality: the implications of spatial autocorrelation for health resources allocation. *Soc Sci Med* 2001;53(12):1711-9.

For peer review only

For peer review only - http://bmjopen.bmj.com/site/about/guidelines.xhtml

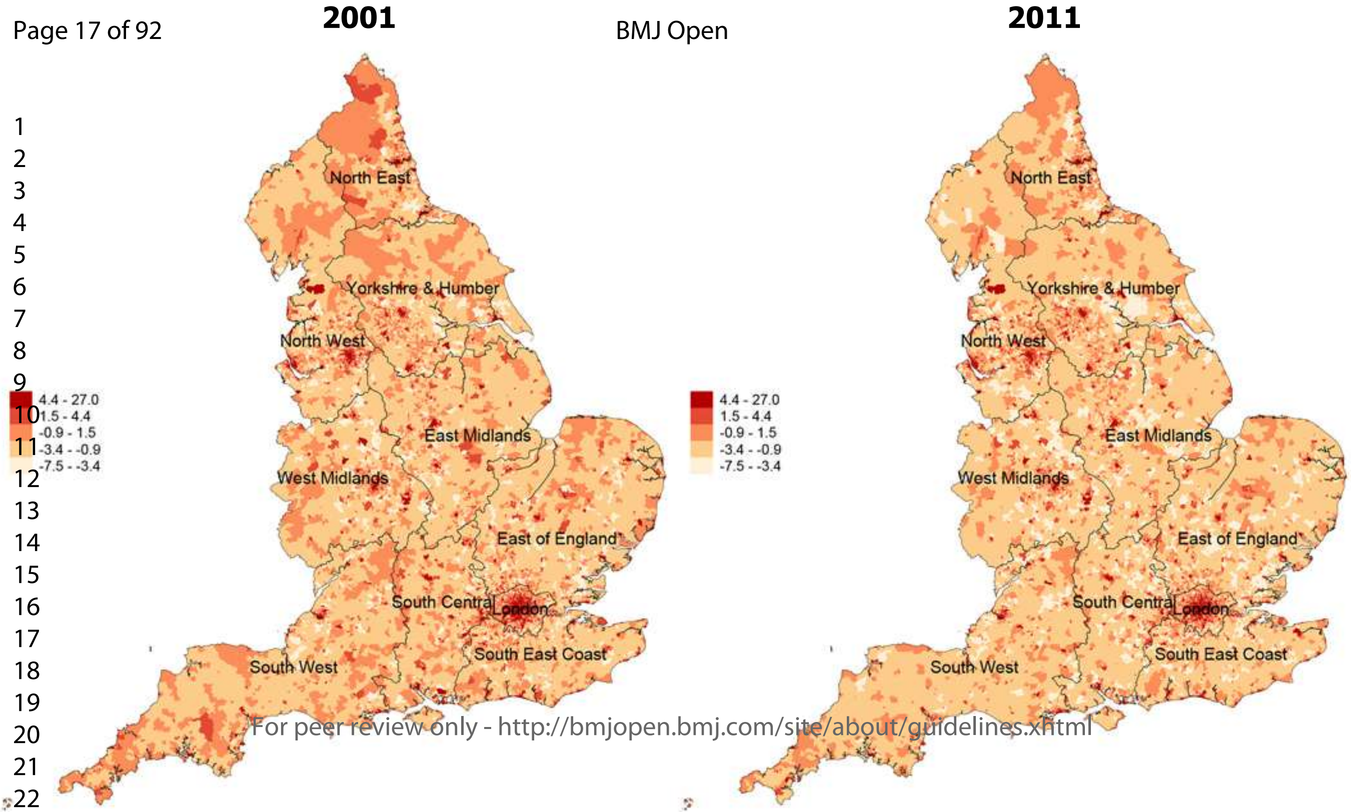

For peer review only - http://bmjopen.bmj.com/site/about/guidelines.xhtml

2001

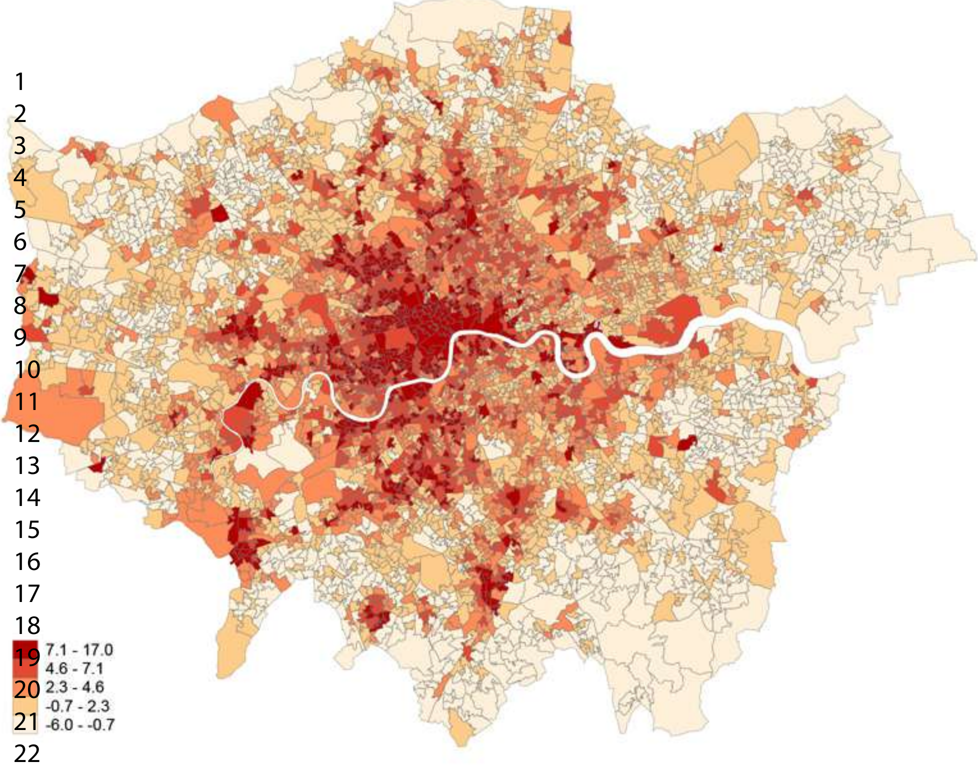

2011

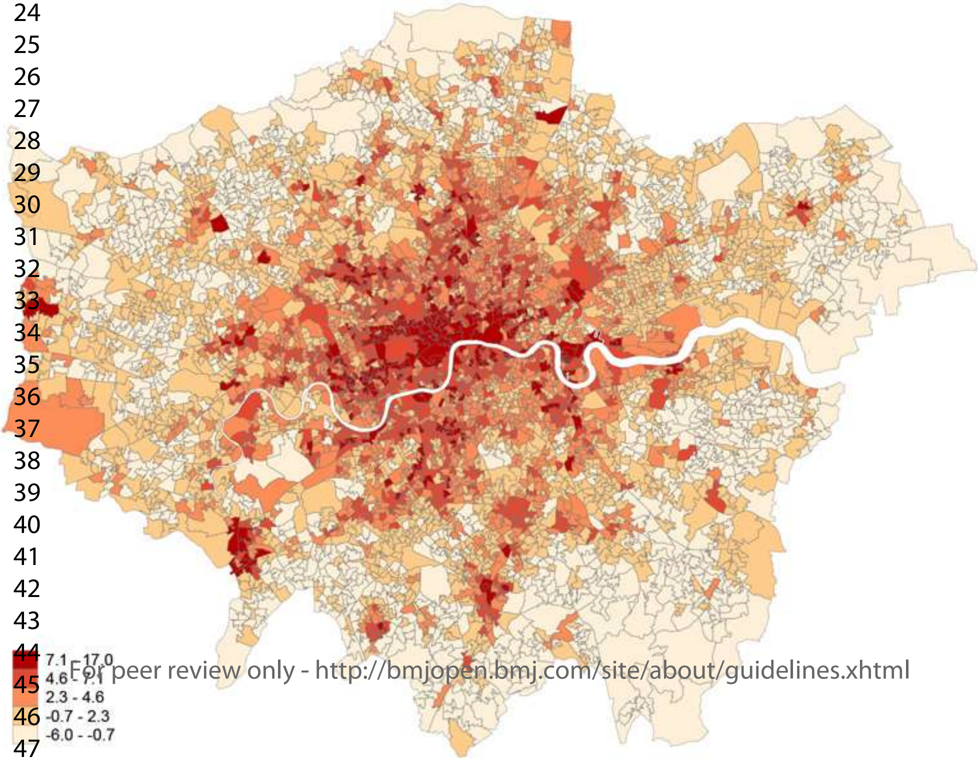

For peer review only - http://bmjopen.bmj.com/site/about/guidelines.xhtml

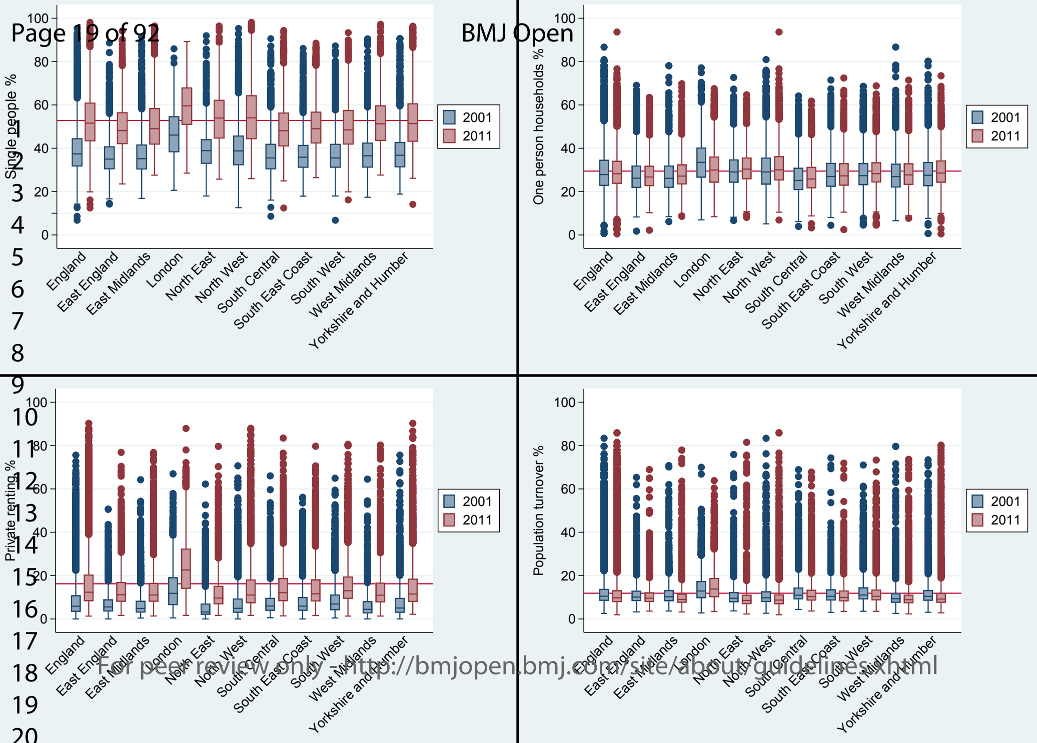

For peer review only - http://bmjopen.bmj.com/site/about/guidelines.xhtml

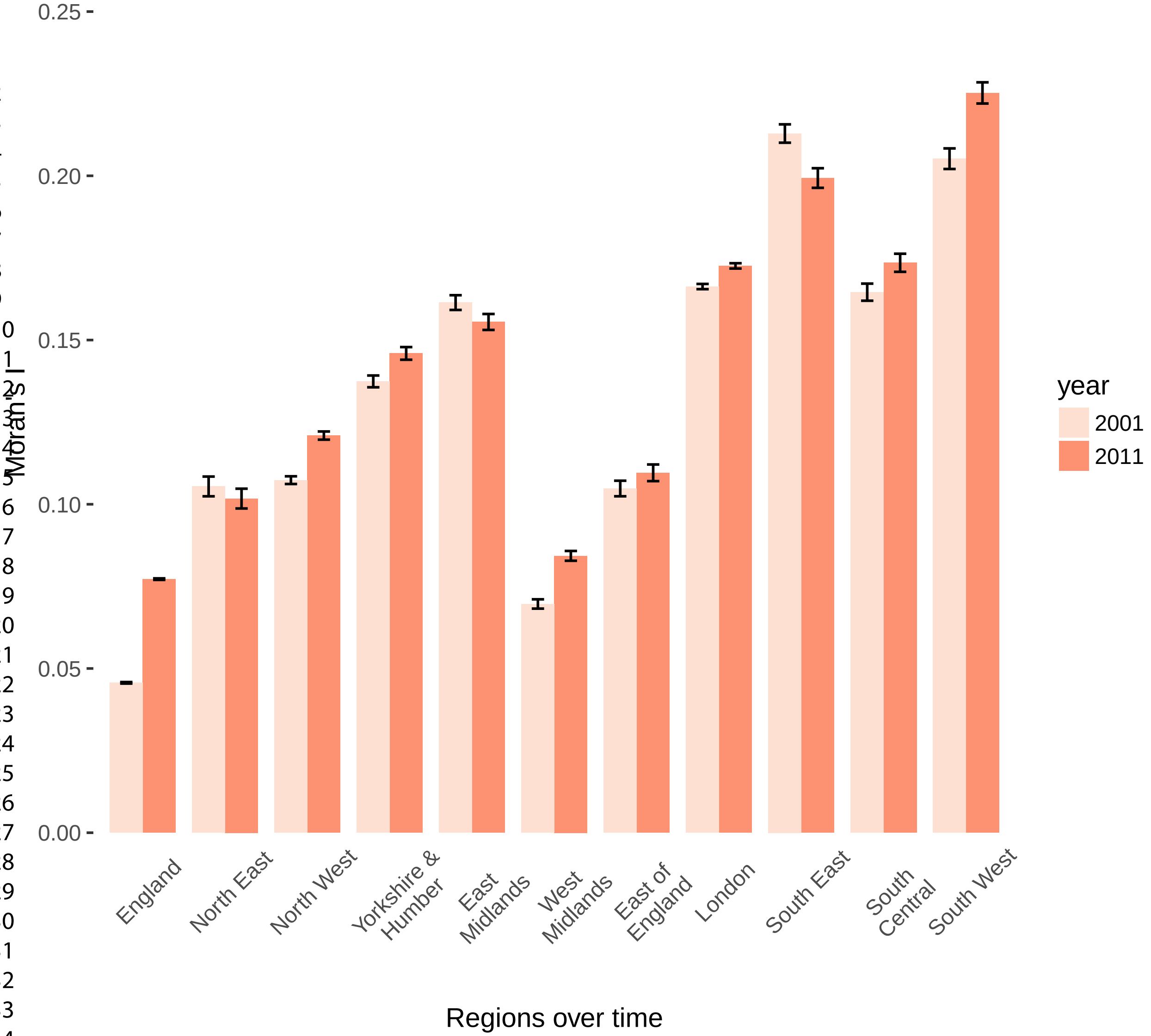
0.25
0.20
0.15
0.10
0.05
0.00
Moran's I
year
2001
2011
England
North East
North West
Yorkshire & Humber
East Midlands
West Midlands
East of England
London
South East
South Central
South West
Regions over time

Supplementary Appendix

Spatial distribution and temporal trends in social fragmentation in England, 2001 to 2011: a national study

For peer review only

For peer review only - http://bmjopen.bmj.com/site/about/guidelines.xhtml

Contents

Description of data and formulas used for the calculation of the underlying social fragmentation indicators at both time points 3

Spatial weighted regressions to attribute 2001 data to 2011 LSOAs 4

Spatial Maps 4

Figure A: Index of Social Fragmentation (mean) for England and English regions, over time. 5

Figure A1 Spatial autocorrelation with Moran's I for single persons (top) and one person households (bottom), by region and over time 6

Figure A2 Spatial autocorrelation with Moran's I for private renting (top) and population turnover (bottom), by region and over time 7

Figure B1: Local Moran scatterplot for social fragmentation at the LSOA level, 2001 8

Figure B2: Local Moran scatterplot for social fragmentation at the LSOA level, 2011 8

Results from LISA analysis: 9

References 10

For peer review only

Description of data and formulas used for the calculation of the underlying social fragmentation indicators at both time points

2001

Percentage of single people in LSOA = All singles, divorced, widowed and separated in LSOA / All usual residents aged 16 and above in LSOA (dataset available from: https://www.nomisweb.co.uk/census/2001/ks004)

Percentage of one person households in LSOA = Total one person households in LSOA/ All household composition in LSOA (dataset available from: https://www.nomisweb.co.uk/census/2001/uv065)

Percentage of private renting in LSOA = Total houses rented from a private landlord or letting agent in LSOA/ All household composition in LSOA (dataset available from: https://www.nomisweb.co.uk/census/2001/uv063)

Percentage of population turnover in LSOA= People who are migrants (i.e. moved into the area from within the UK & moved into the area from outside the UK within the year before the census / All usual residents in LSOA (dataset available from: https://www.nomisweb.co.uk/census/2001/ks024)

2011

Percentage of single people in LSOA = All singles, divorced, widowed and separated in LSOA / All usual residents aged 16 and above in LSOA (dataset available from: https://www.nomisweb.co.uk/census/2011/ks103uk)

Percentage of one person households in LSOA = Total one person households in LSOA/ All household composition in LSOA (dataset available from: https://www.nomisweb.co.uk/census/2011/ks105ew)

Percentage of private renting in LSOA = Total houses rented from a private landlord or letting agent in LSOA/ All household composition in LSOA (dataset available from: https://www.nomisweb.co.uk/census/2011/ks402ew)

Percentage of population turnover in LSOA= People who are migrants (i.e. moved into the area from within the UK & moved into the area from outside the UK within the year before the census / All usual residents in LSOA (dataset available from: https://www.nomisweb.co.uk/census/2011/ukmig008)

For peer review only

Spatial weighted regressions to attribute 2001 data to 2011 LSOAs

The 2003 National Statistics policy to minimise the statistical impact of frequent electoral ward boundary changes was succeeded by boundary changes across LSOAs in 2011. In that year, there was a modification of OA (Output Areas) and SOA (Super Output Areas) in England & Wales mainly due to significant population changes between the decennial censuses. As social fragmentation in 2001 was measured in 2001 LSOAs, we attributed census 2001 LSOA information to 2011 LSOAs and we used population weighted regressions to estimate 2001 data at the 2011 LSOA to enable seamless comparisons across time. More specifically, 97.4% of all LSOAs remained unchanged in 2011, and for these areas we took no action and attributed the social fragmentation values from 2001 to the 2011 LSOAs. If two or more 2001 LSOAs merged in one LSOA in 2011 (0.6% of all LSOAs), we calculated the mean value of social fragmentation weighted for 2003 LSOA population (which is the oldest population estimates dataset provided by ONS). If a 2001 LSOA split into two or more 2011 LSOAs (1.8% of all LSOAs) we assigned the relevant 2001 social fragmentation scores. Finally, there were some LSOAs for which changes did not match any of the previously described patterns (i.e. an LSOA split in two and each part merged with a different LSOA). For these very few LSOAs (0.1% of all LSOAs) we developed an algorithm to calculate population weighted mean estimates. (1) The corresponding algorithm is available from the authors.

Spatial Maps

Digital vector boundaries for the 2011 LSOAs, generalised to 20 metres and clipped to the coastline to reduce size and improve visualisation, were obtained from the ONS open geography portal (2) . The vector boundaries were inputted in the Stata shp2dta command to calculate the centroid for each LSOA in the British National Grid format (3). These were then converted from British National Grid easting and northing to longitude and latitude in degrees (4).

For peer review only

Figure A: Index of Social Fragmentation (mean) for England and English regions, over time.

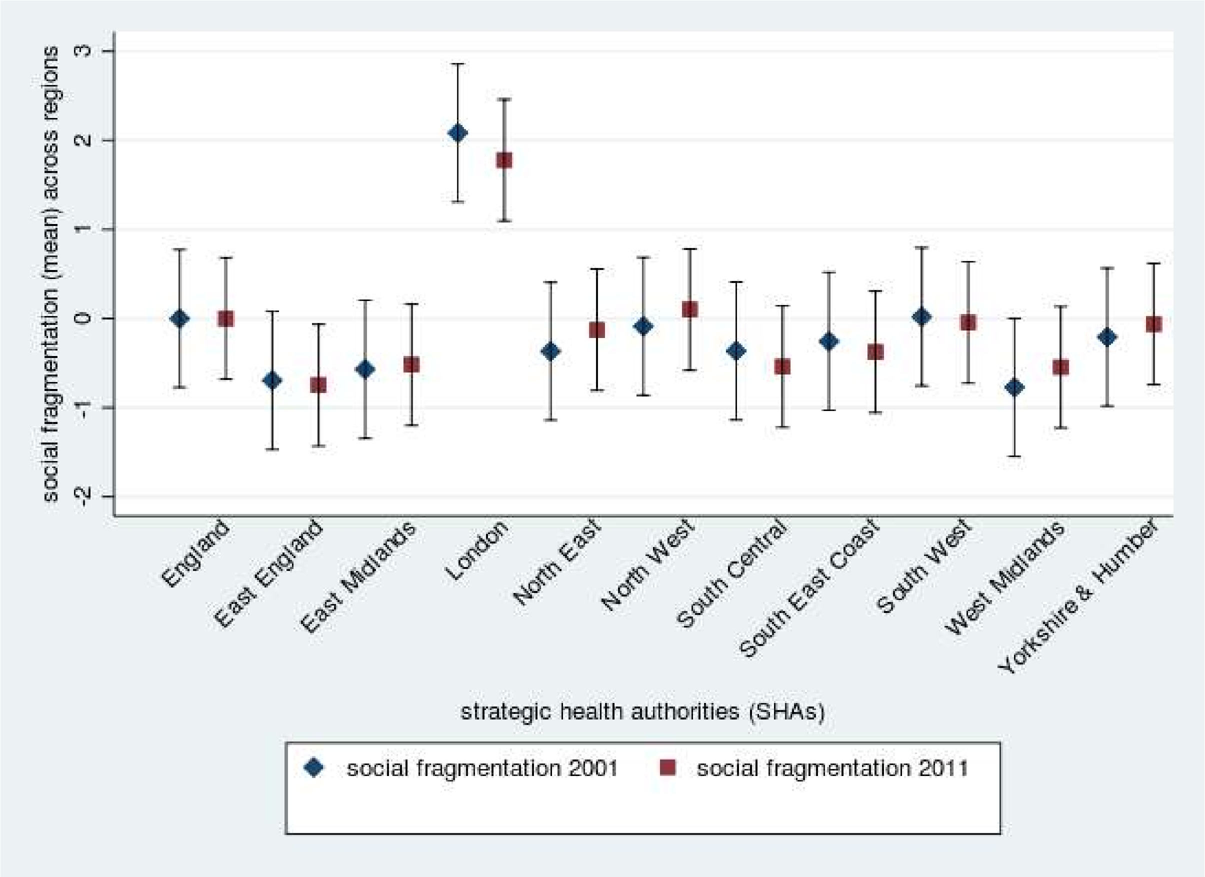

eview only

Figure A1: Spatial autocorrelation with Moran's I for single persons (top) and one person households (bottom), by region and over time

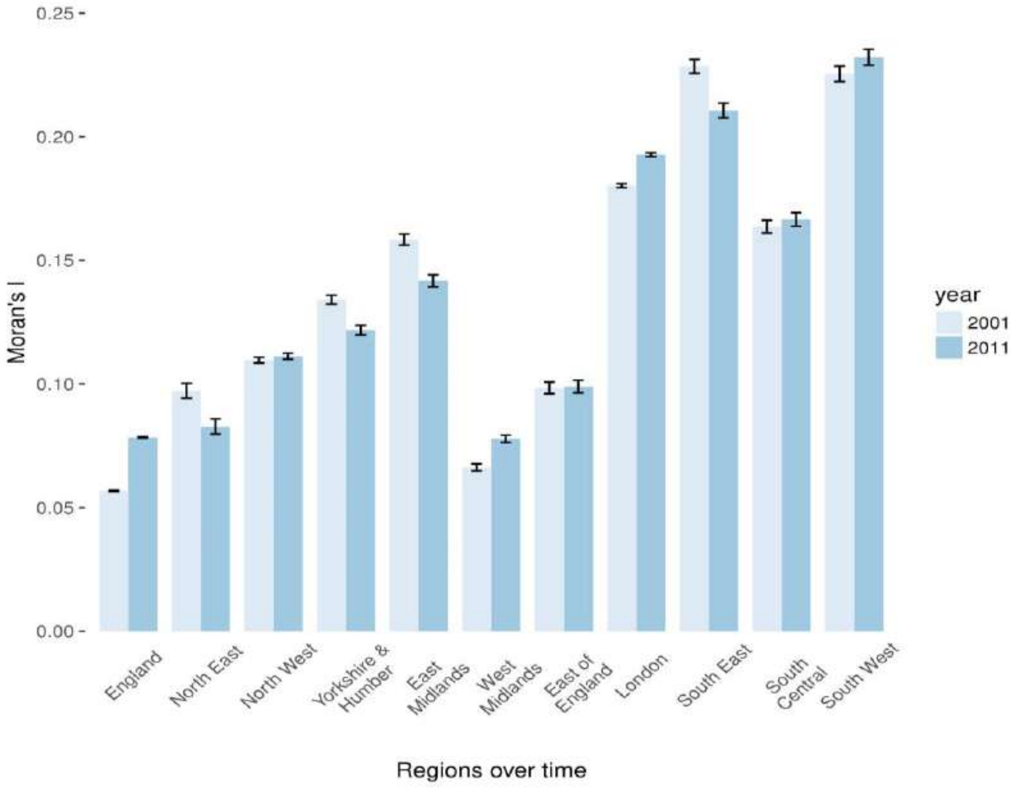

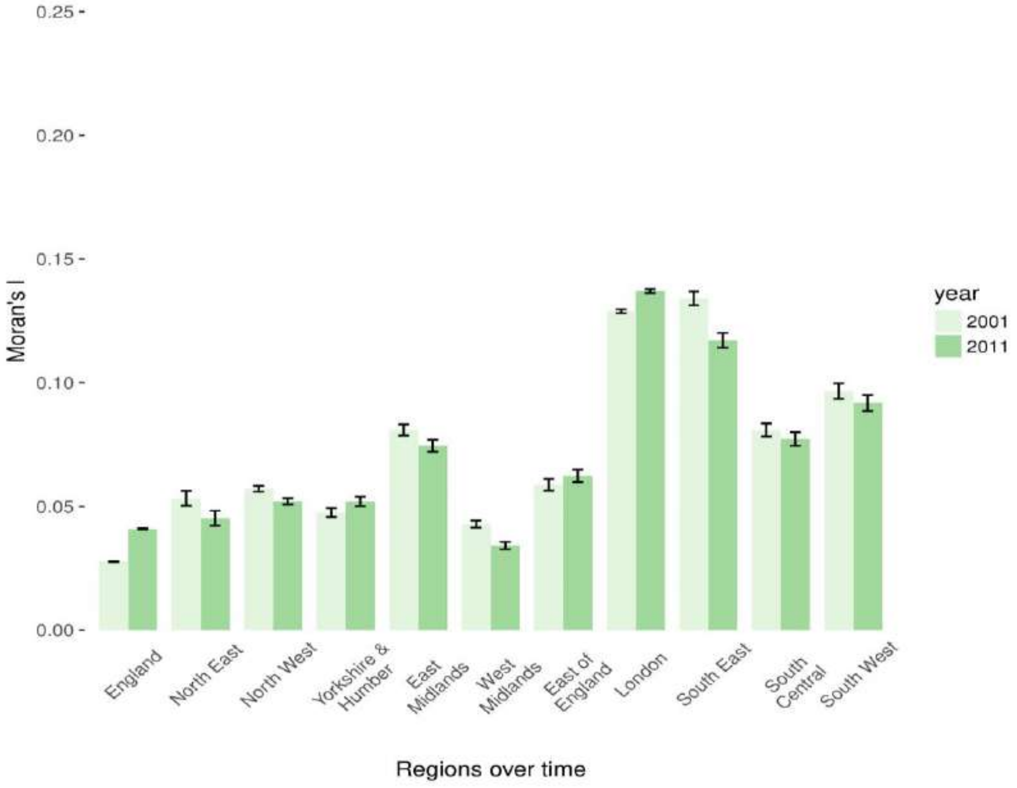

Figure A2: Spatial autocorrelation with Moran's I for private renting (top) and population turnover (bottom), by region and over time

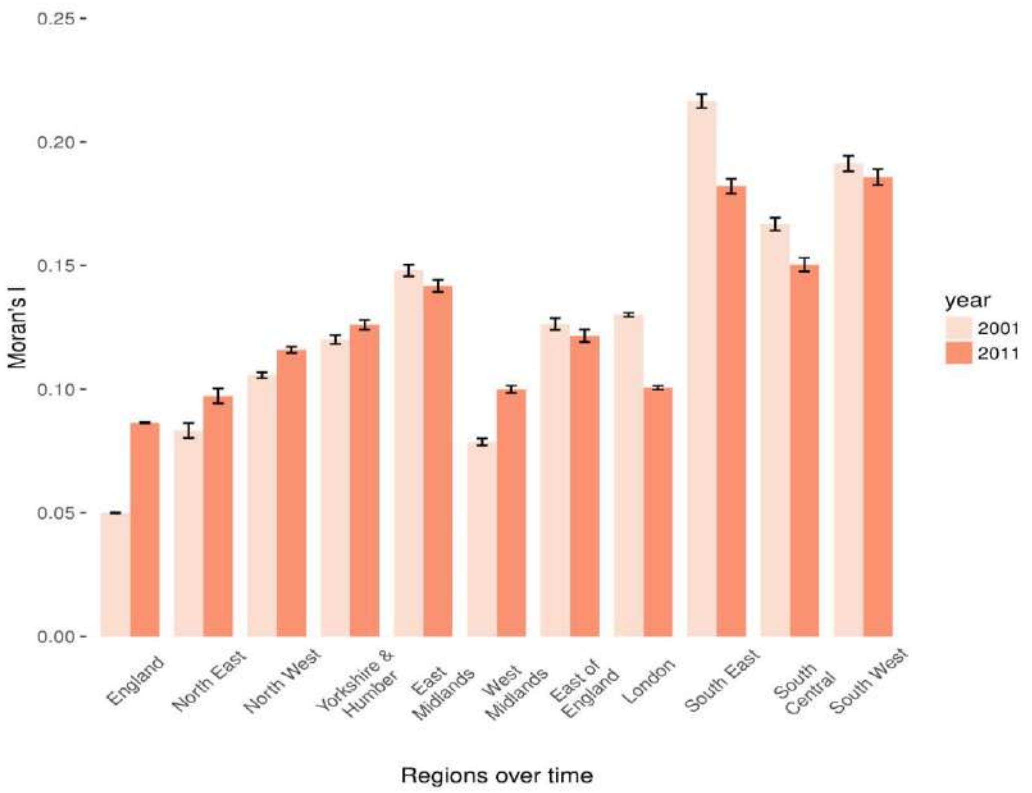

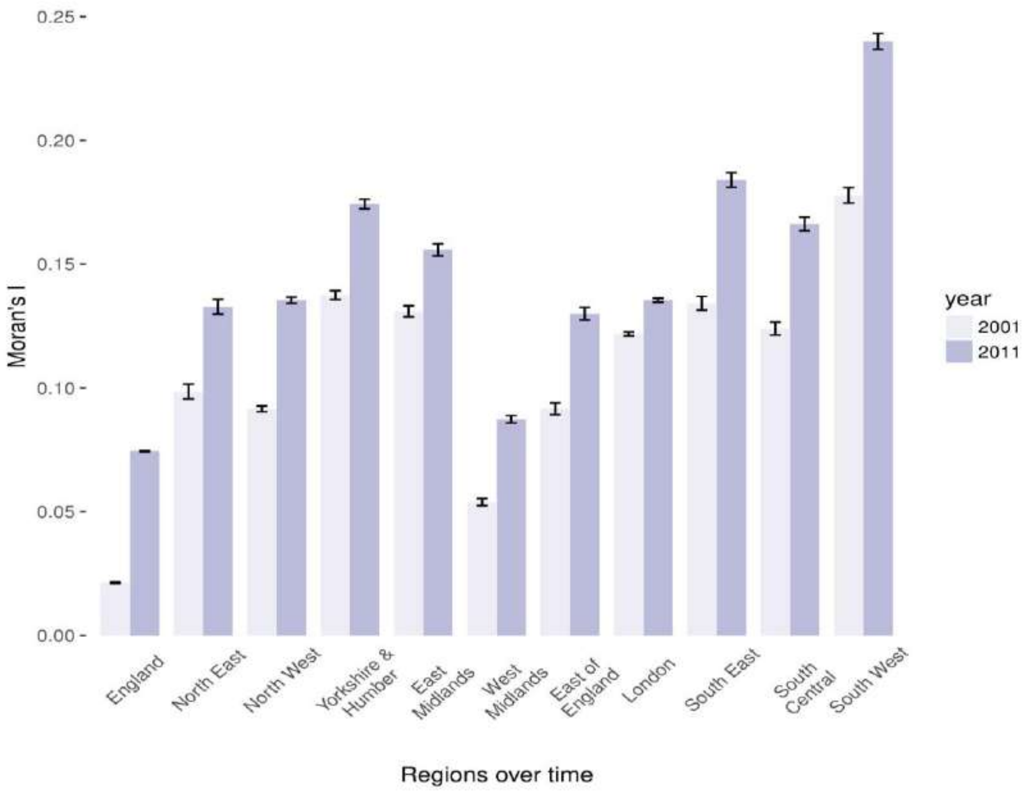

Figure B1: Local Moran scatterplot for social fragmentation at the LSOA level, 2001.

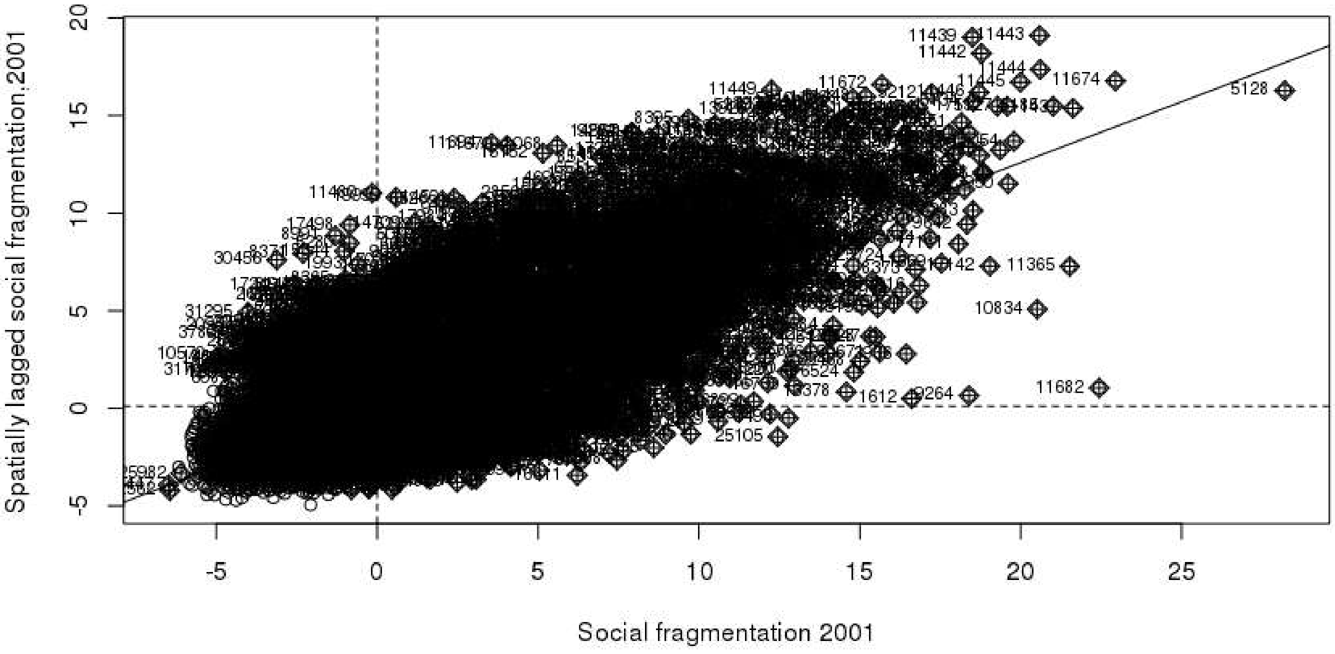

Figure B2: Local Moran scatterplot for social fragmentation at the LSOA level, 2011.

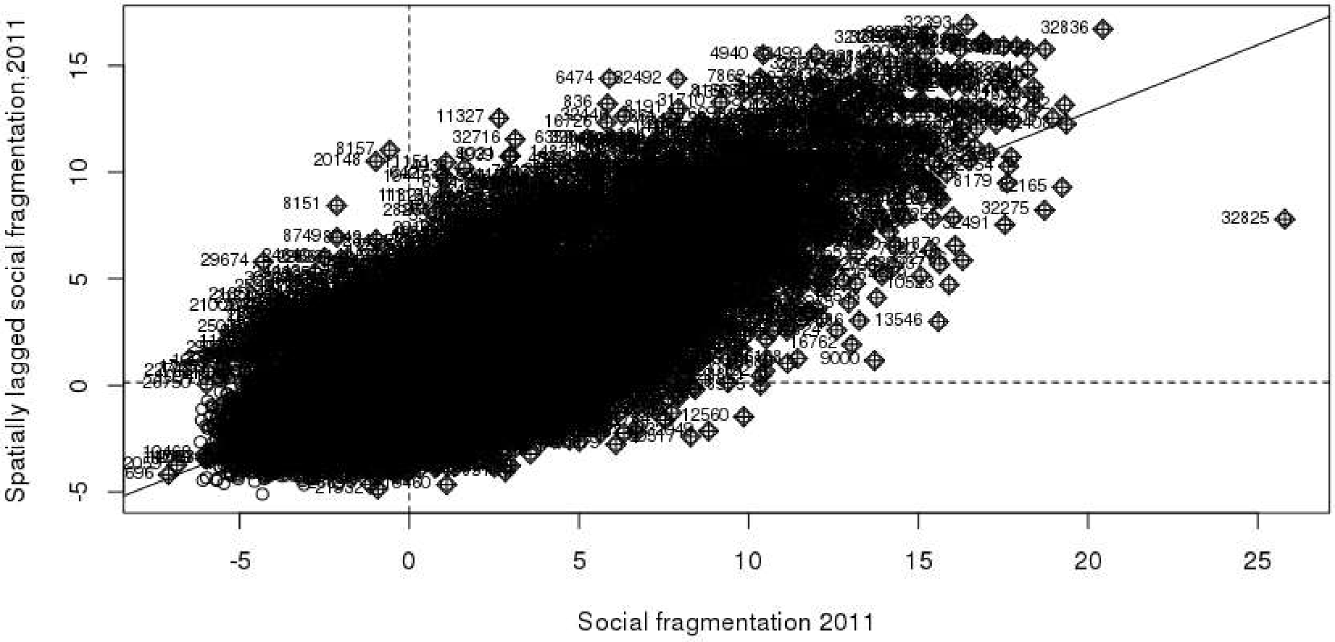

Results from Local Indicator of Spatial Association (LISA) analysis: The Moran scatterplots for social fragmentation in 2001 and 2011 are provided in Figures B1 and B2 respectively. The four quadrants in each scatterplot correspond to four types of association. The lower left and upper quadrants indicate positive spatial autocorrelation, or that areas in these quadrants have similar values (lower than the mean for those areas in the lower left quadrant and higher than the mean in the upper left quadrant). The upper left and lower right quadrant indicate spatial associations of areas with dissimilar values in social fragmentation. In both graphs the slope of the regression line indicates that there is a positive spatial association at both time points with a handful of outliers in 2001 and only a few in 2011. The pattern of increasing spatial autocorrelation across the two time points that we observed in graph 4 for global spatial autocorrelation is also confirmed by comparison of Graphs B1 and B2. Spatial autocorrelation appears to have increased in 2011 when compared to 2001. At both time points, LSOAs were mainly concentrated in the upper right quadrant (Figure B1 & B2) indicating the existence of many hot spots of social fragmentation and highlighting the fact that there are important variations among LSOAs and a finer scale spatial structure that occur below the region level.

For peer review only

For peer review only - http://bmjopen.bmj.com/site/about/guidelines.xhtml

References

1. Office for National Statistics. Changes to output areas and super output areas in England and Wales, 2001 to 2011. 2012.
2. Office for National Statistics. Open Geography Portal. 2013. https://geoportal.statistics.gov.uk/geoportal/.
3. SHP2DTA:. Stata module to converts shape boundary files to Stata datasets [program]. S456718 version: Boston College Department of Economics,. 2006.
4. Ordance Survey. A guide to coordinate systems in Great Britain. ONS, 2013:43. www.ordnancesurveycouk/docs/support/guide-coordinate-systems-great-britainpdf.

For peer review only

For peer review only - http://bmjopen.bmj.com/site/about/guidelines.xhtml

Social Fragmentation

England 2001, Social Fragmentation

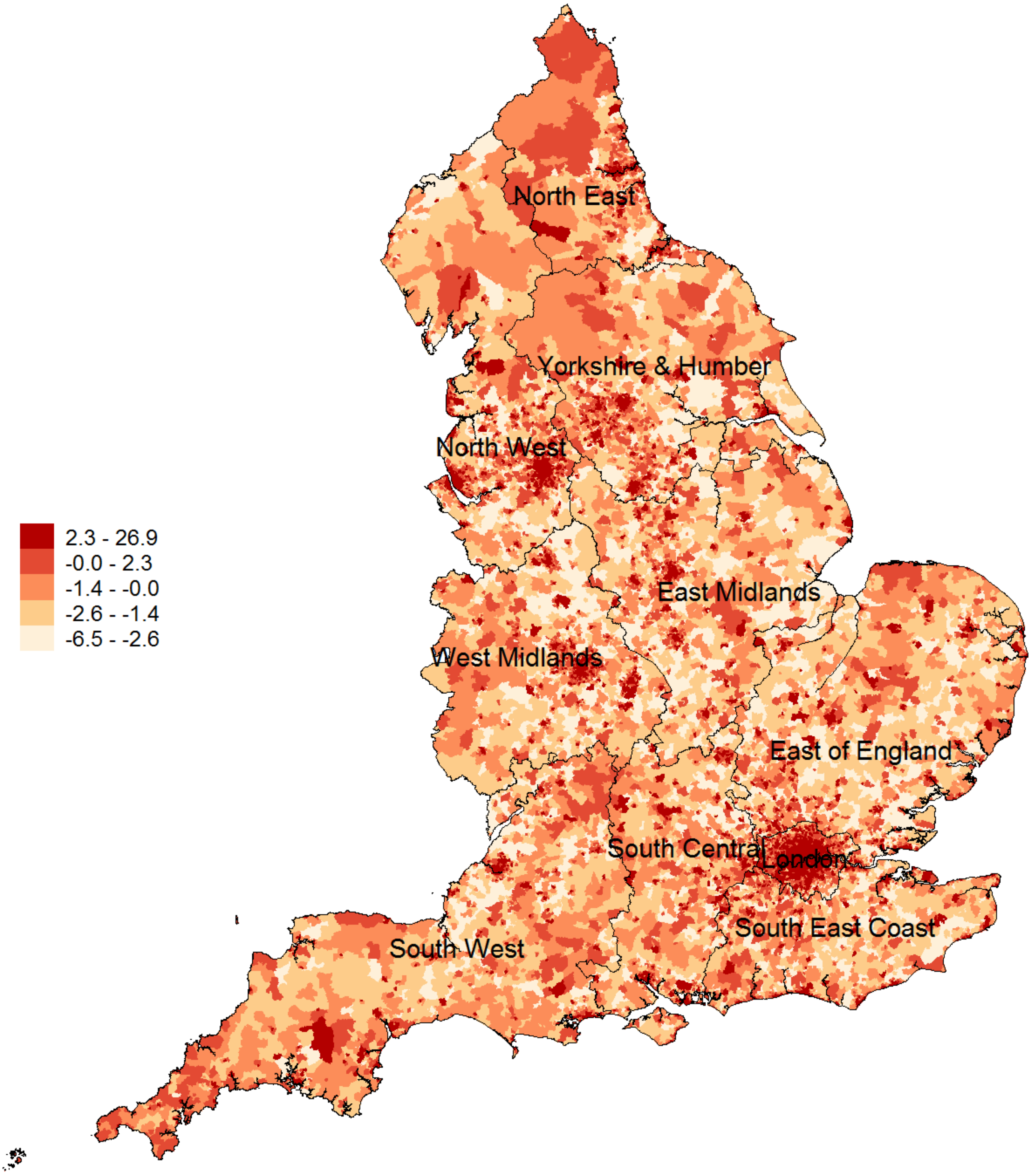

England 2011, Social Fragmentation

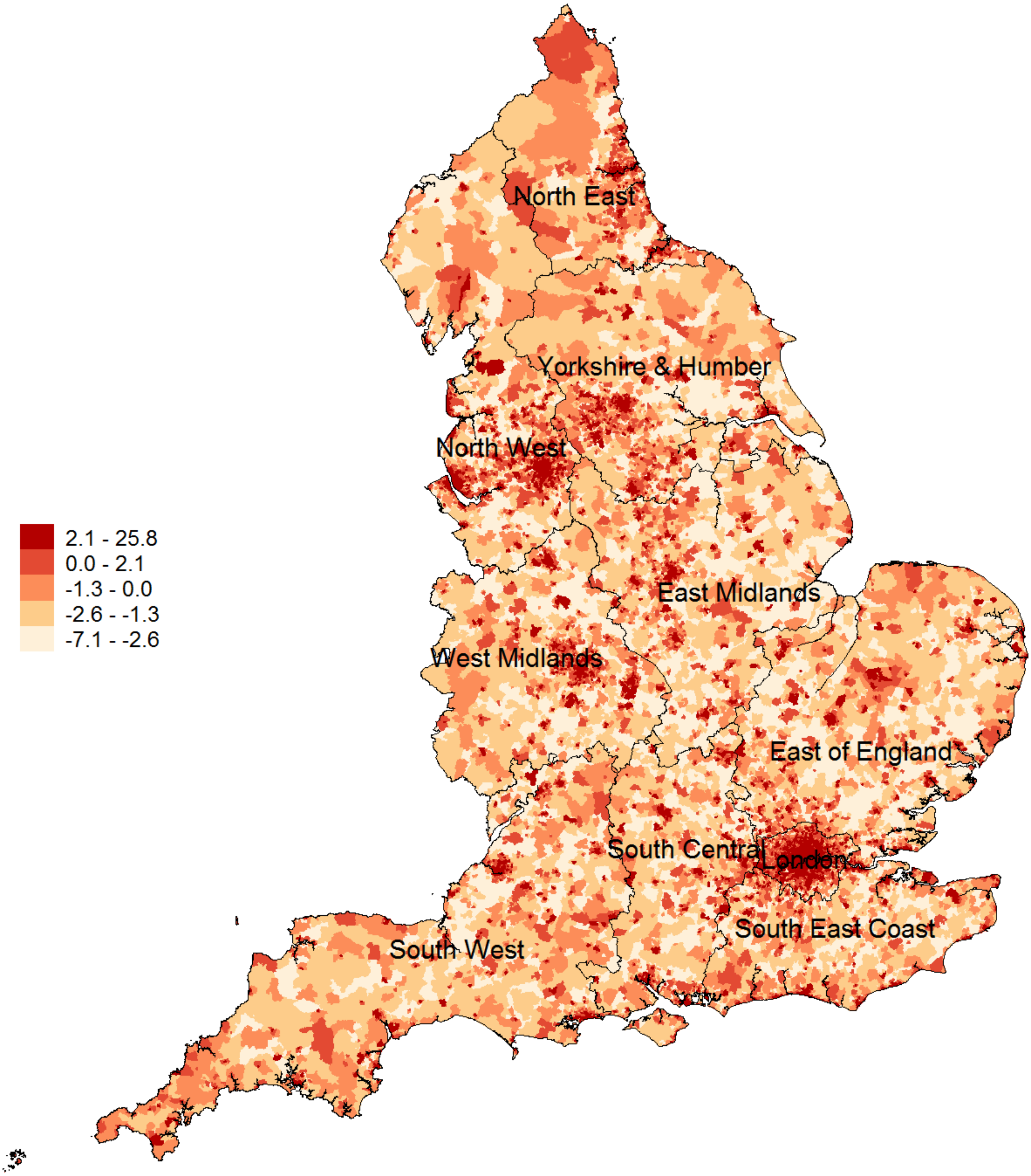

North East, Social Fragmentation

2001 2011

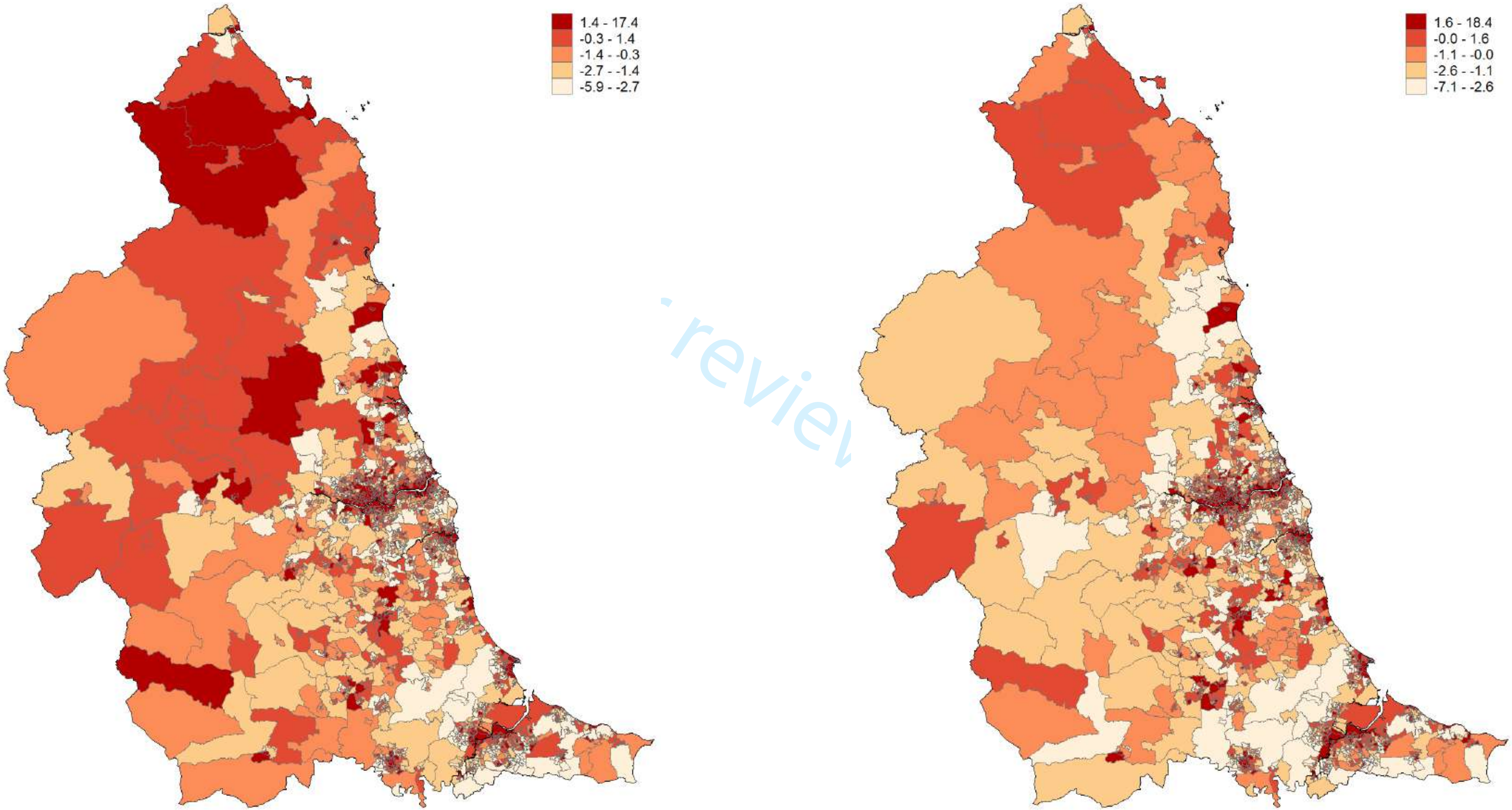

For peer review only - http://bmjopen.bmj.com/site/about/guidelines.xhtml

North West, Social Fragmentation

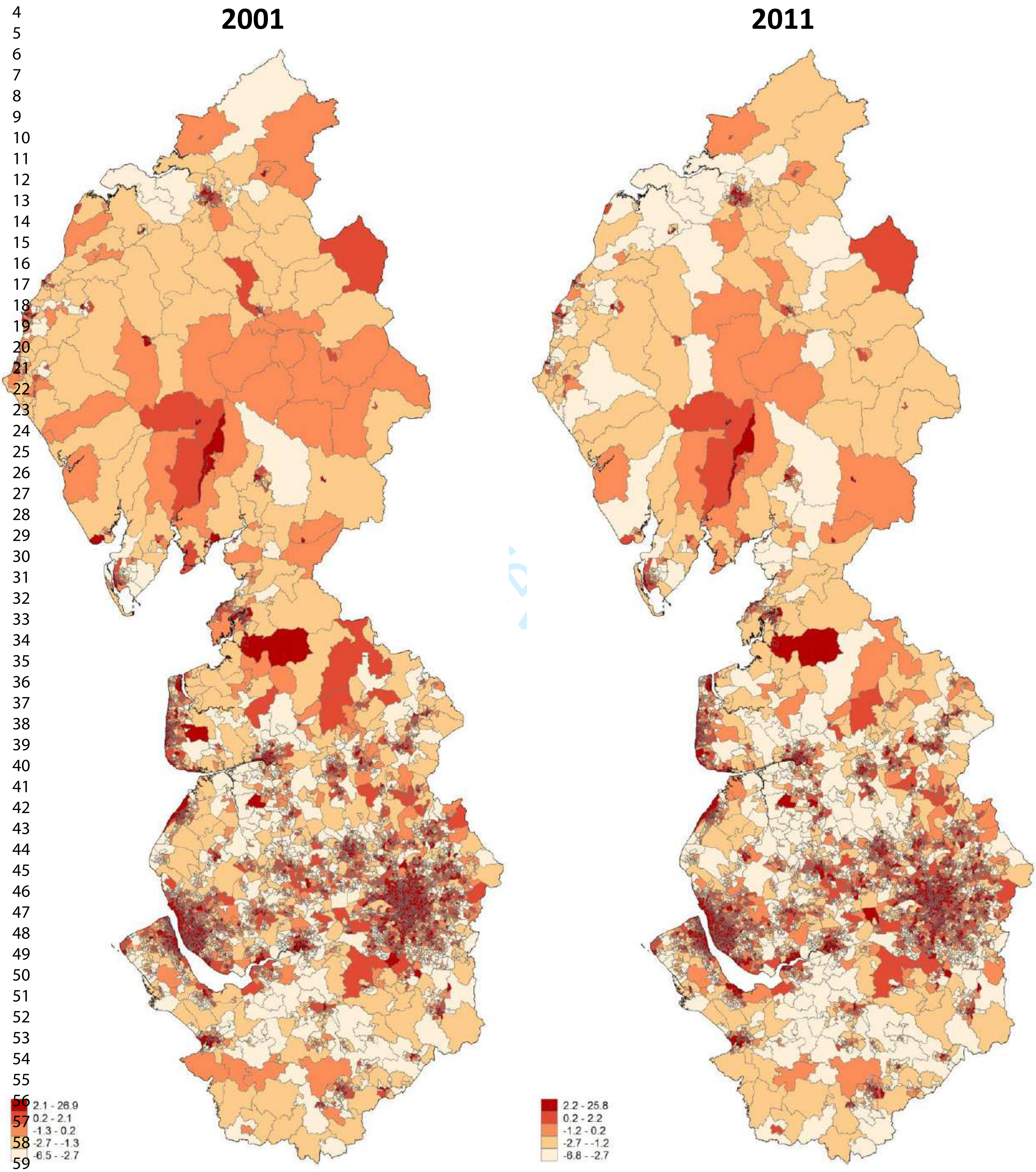

Yorkshire and the Humber, Social Fragmentation

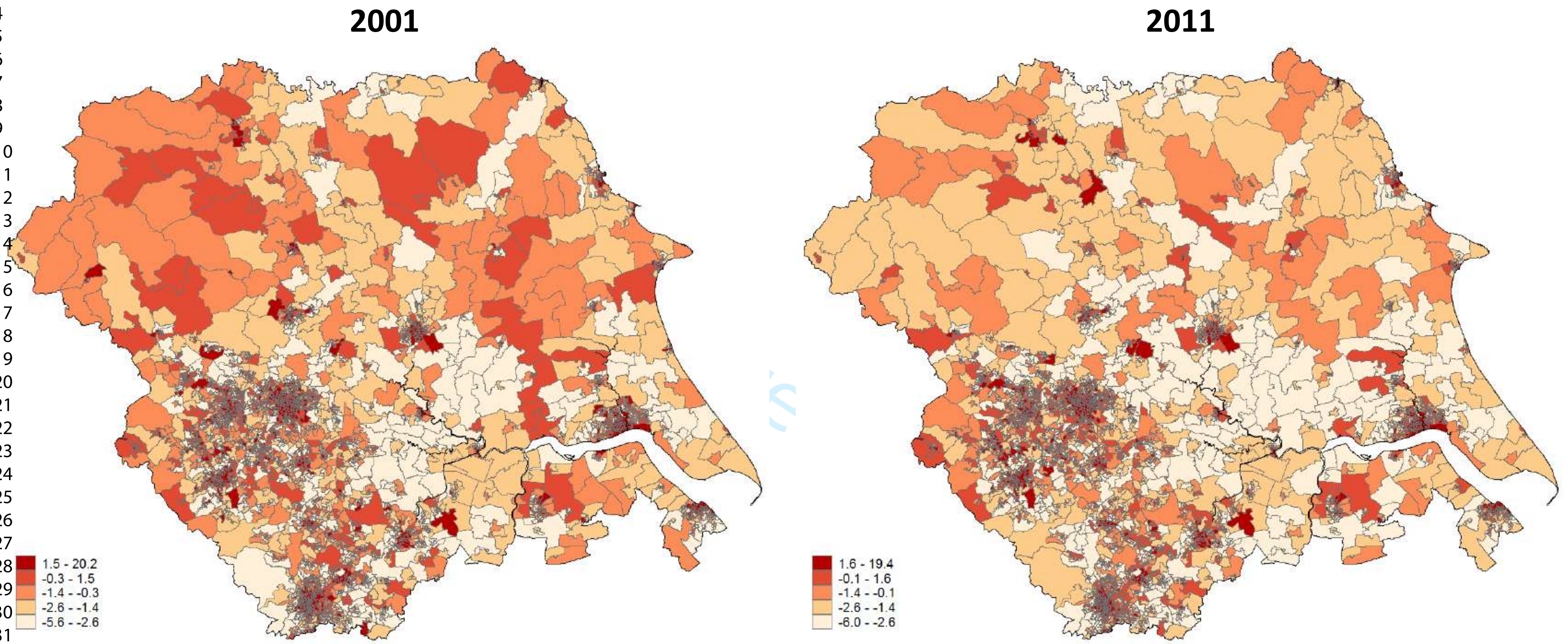

East Midlands, Social Fragmentation

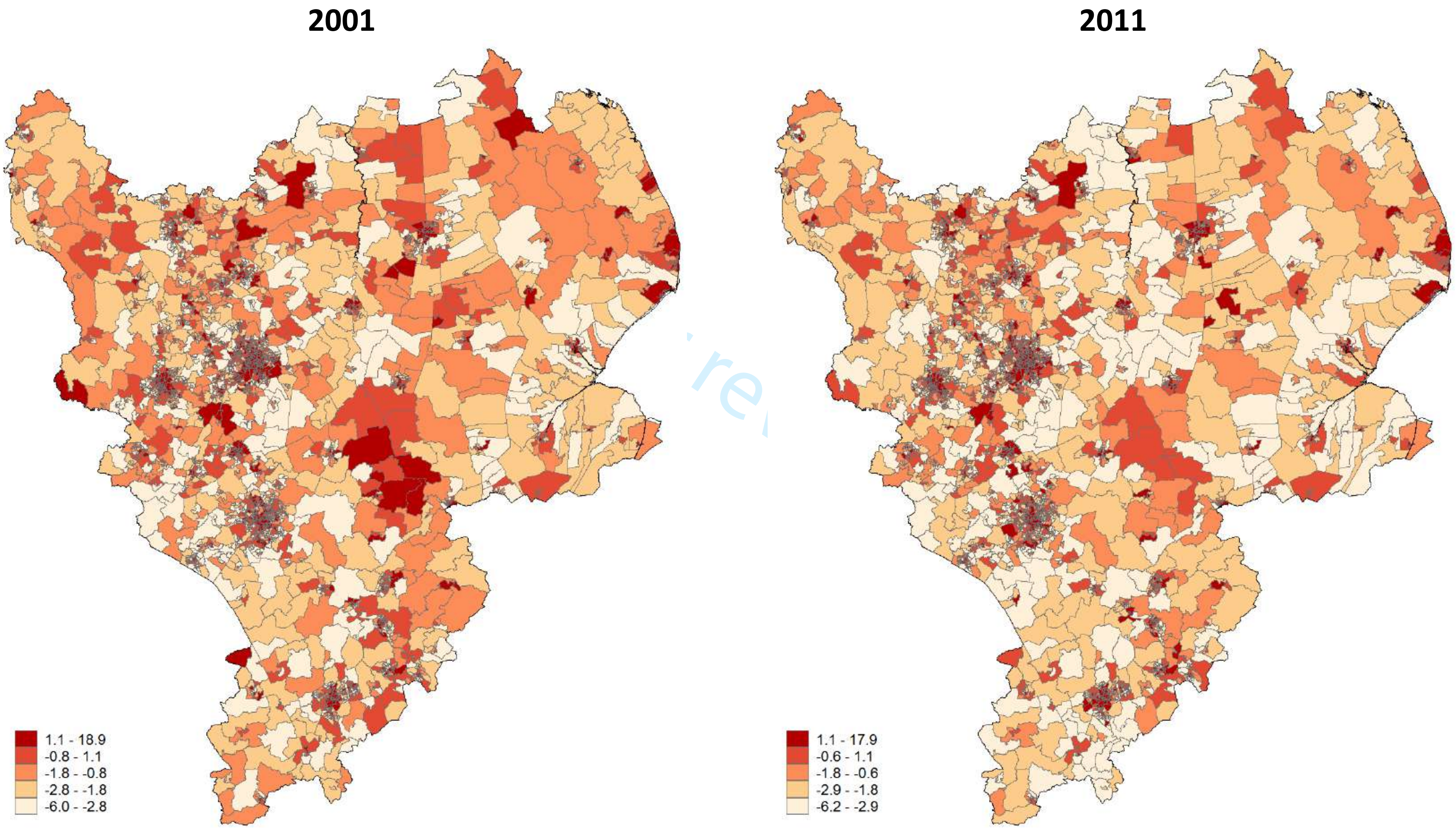

West Midlands, Social Fragmentation

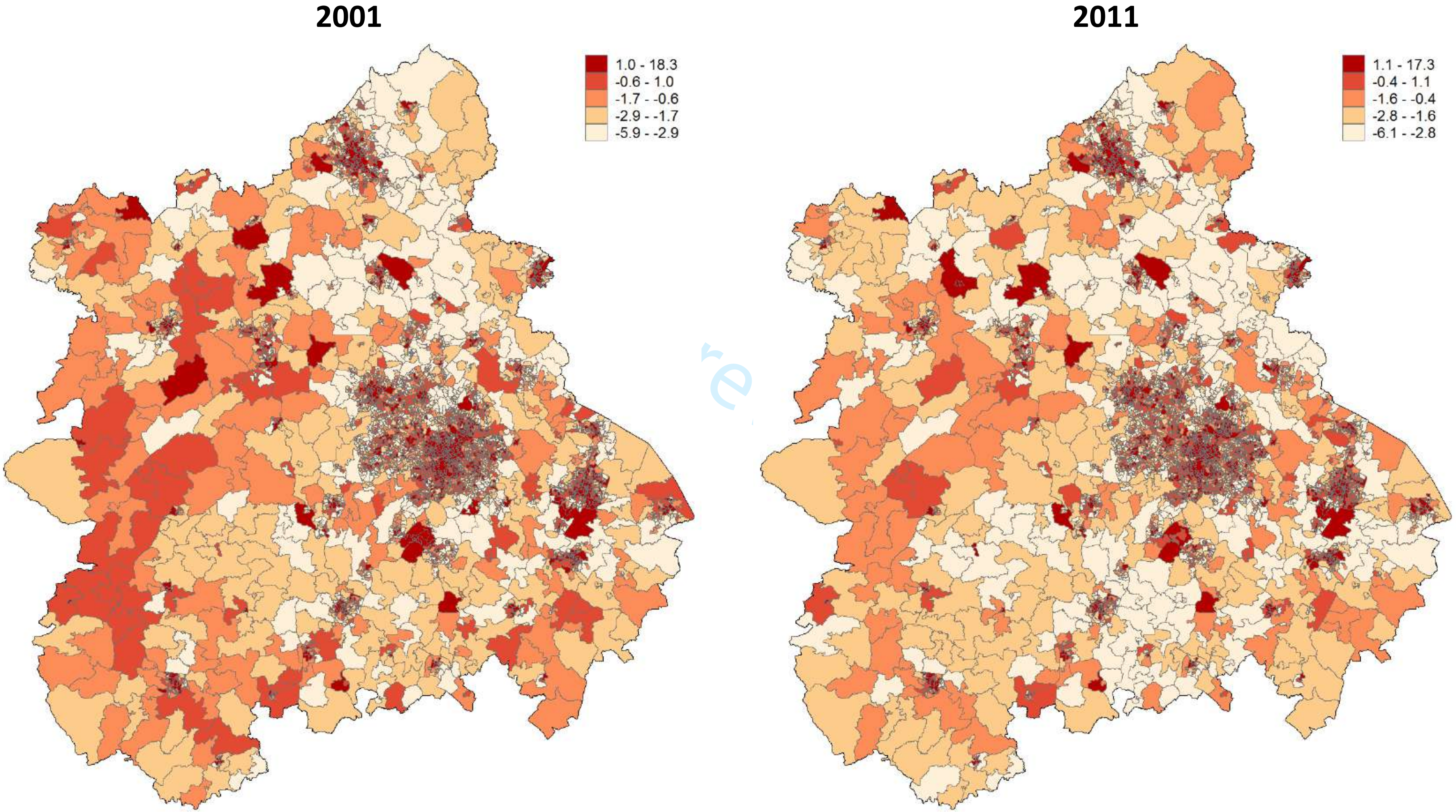

East of England, Social Fragmentation

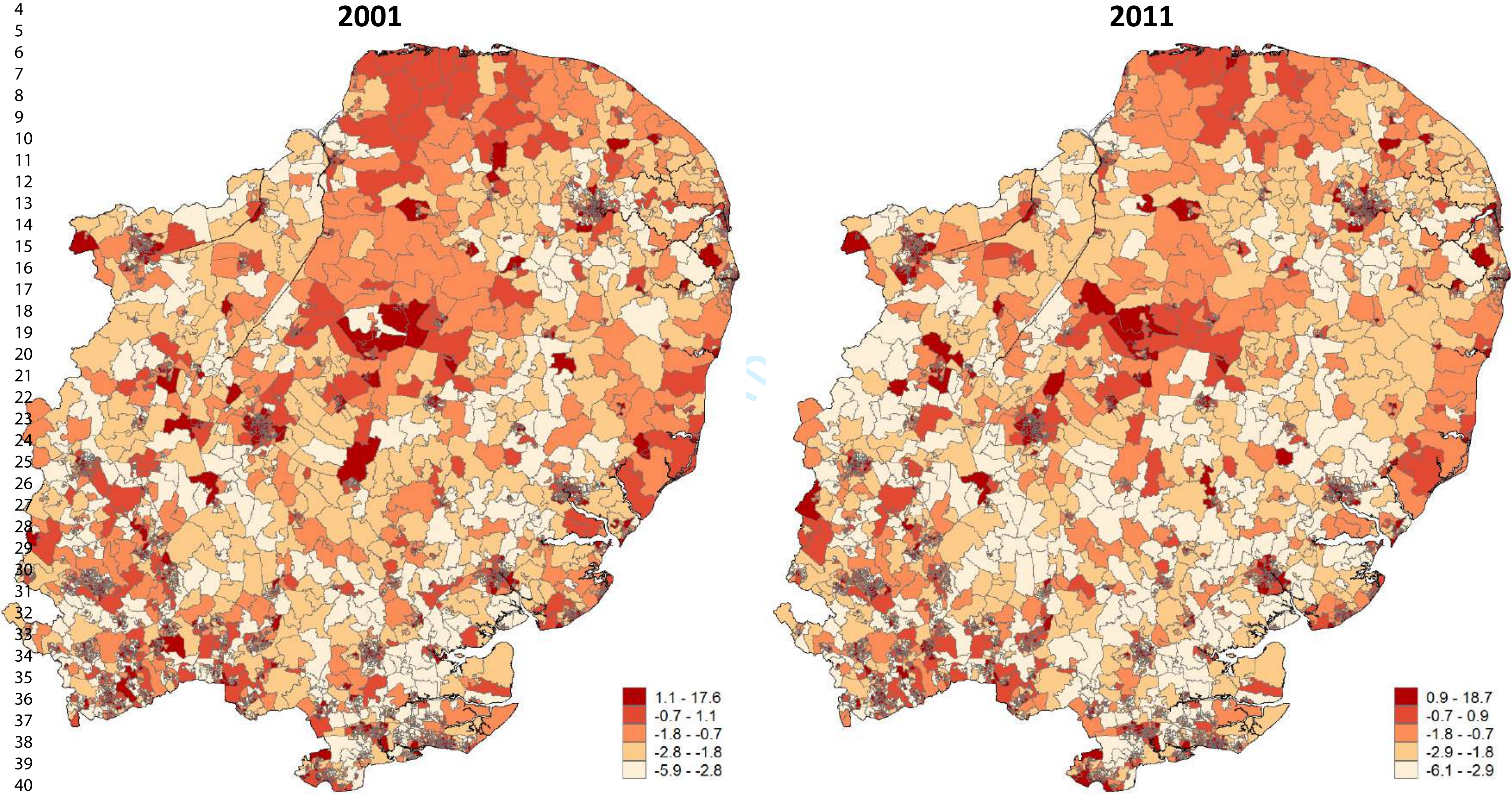

London, Social Fragmentation

2001

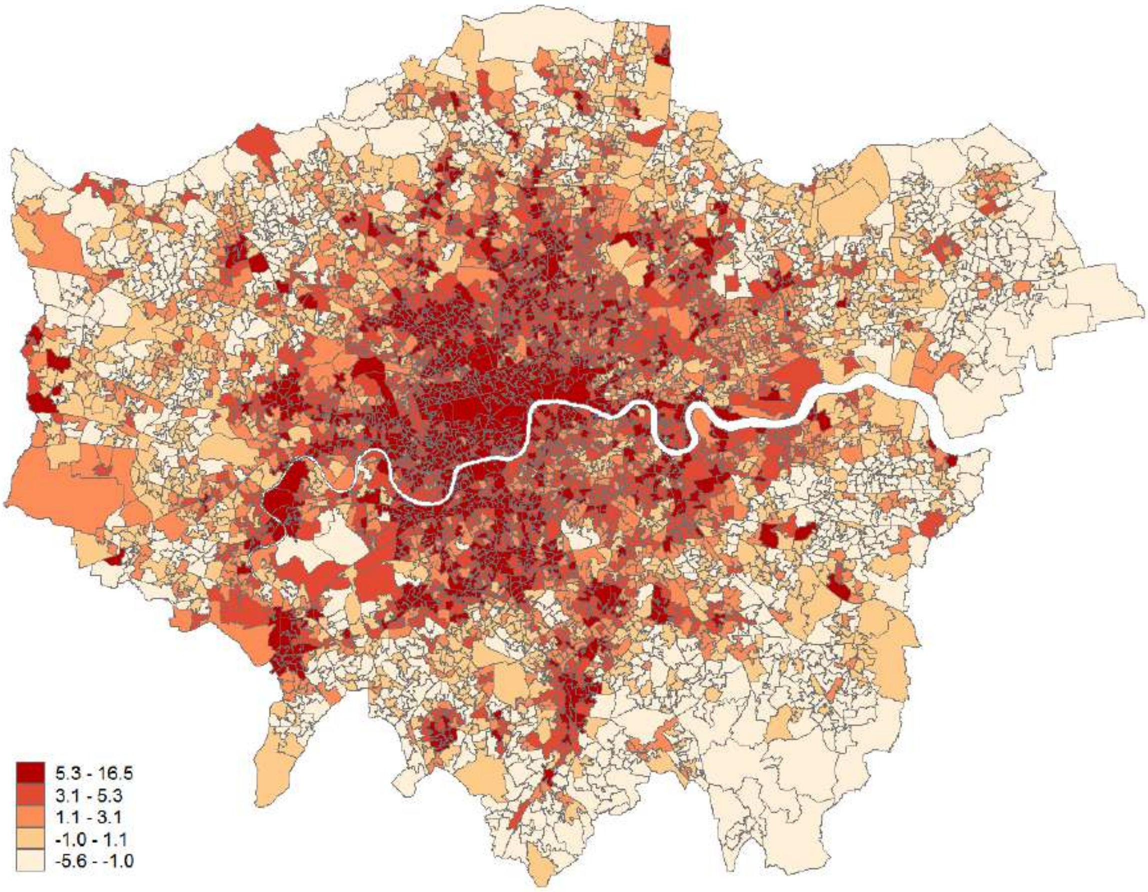

2011

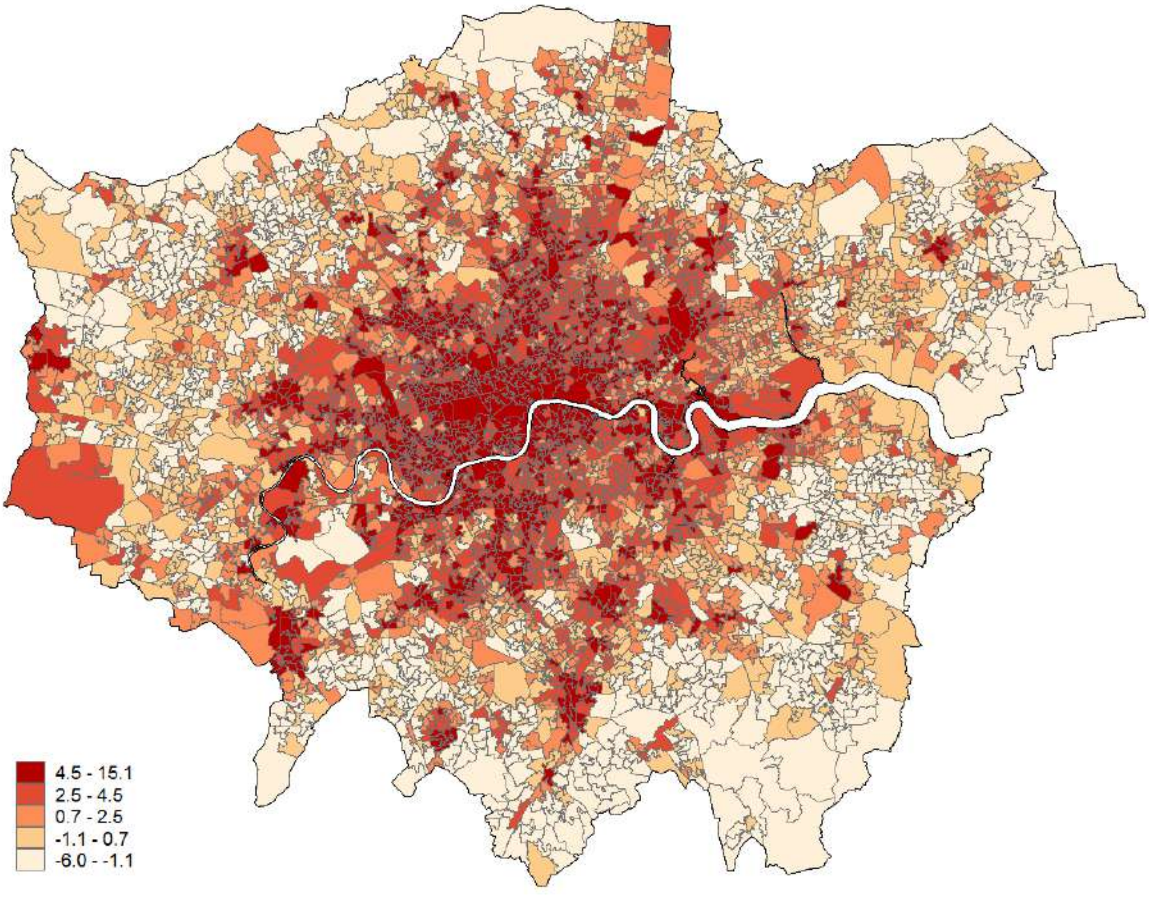

South East Coast, Social Fragmentation

2001

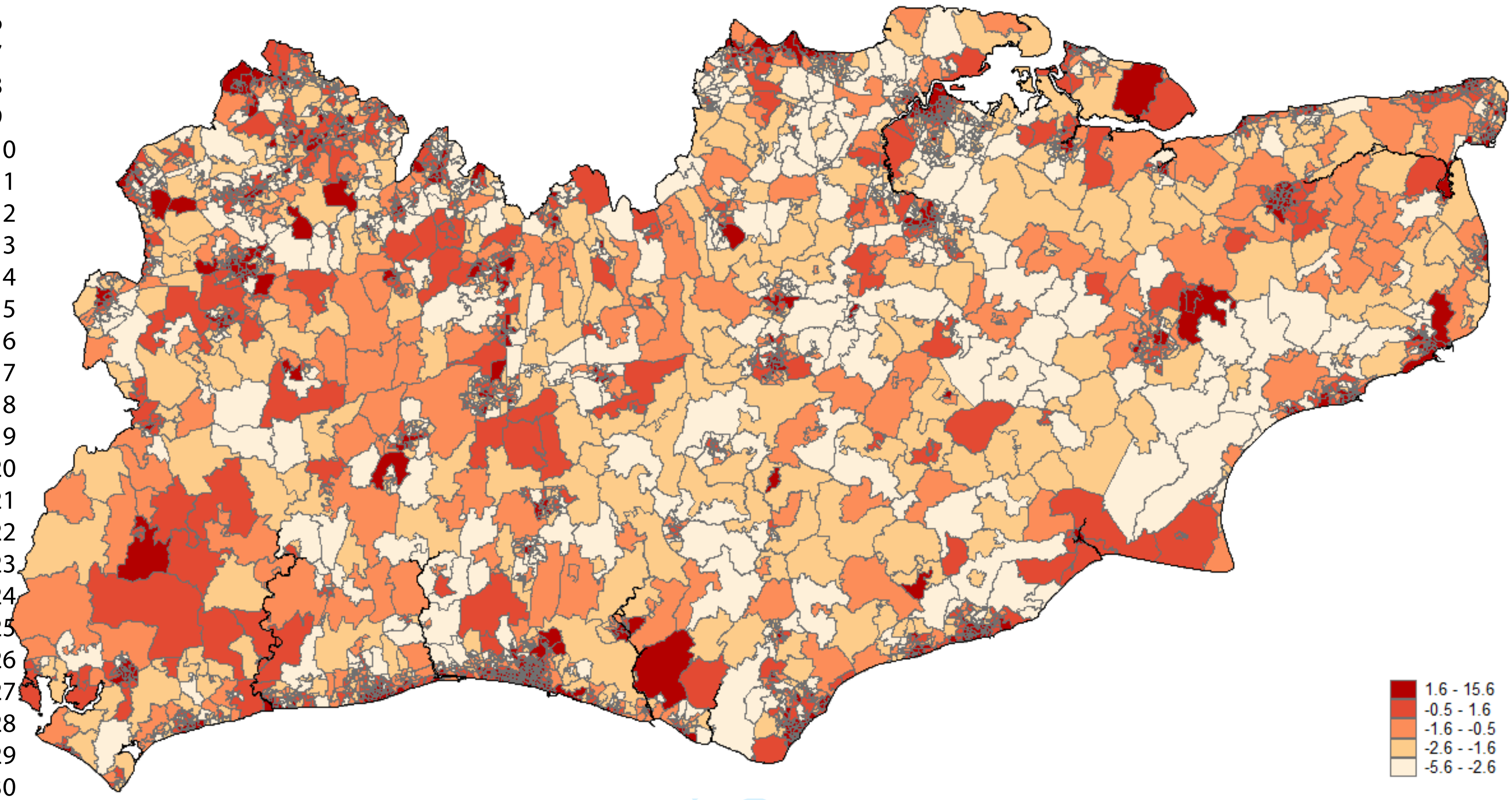

2011

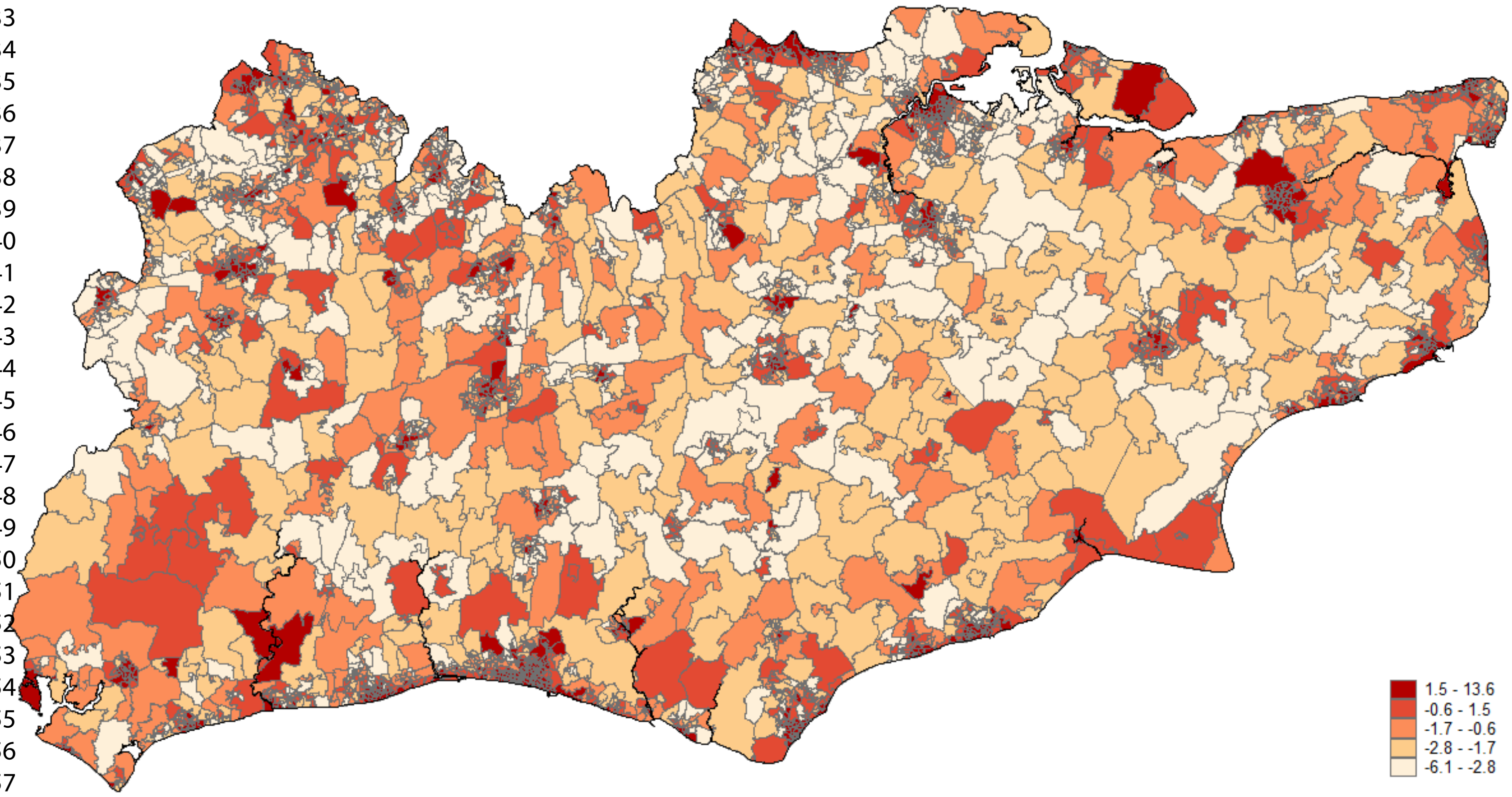

South Central, Social Fragmentation

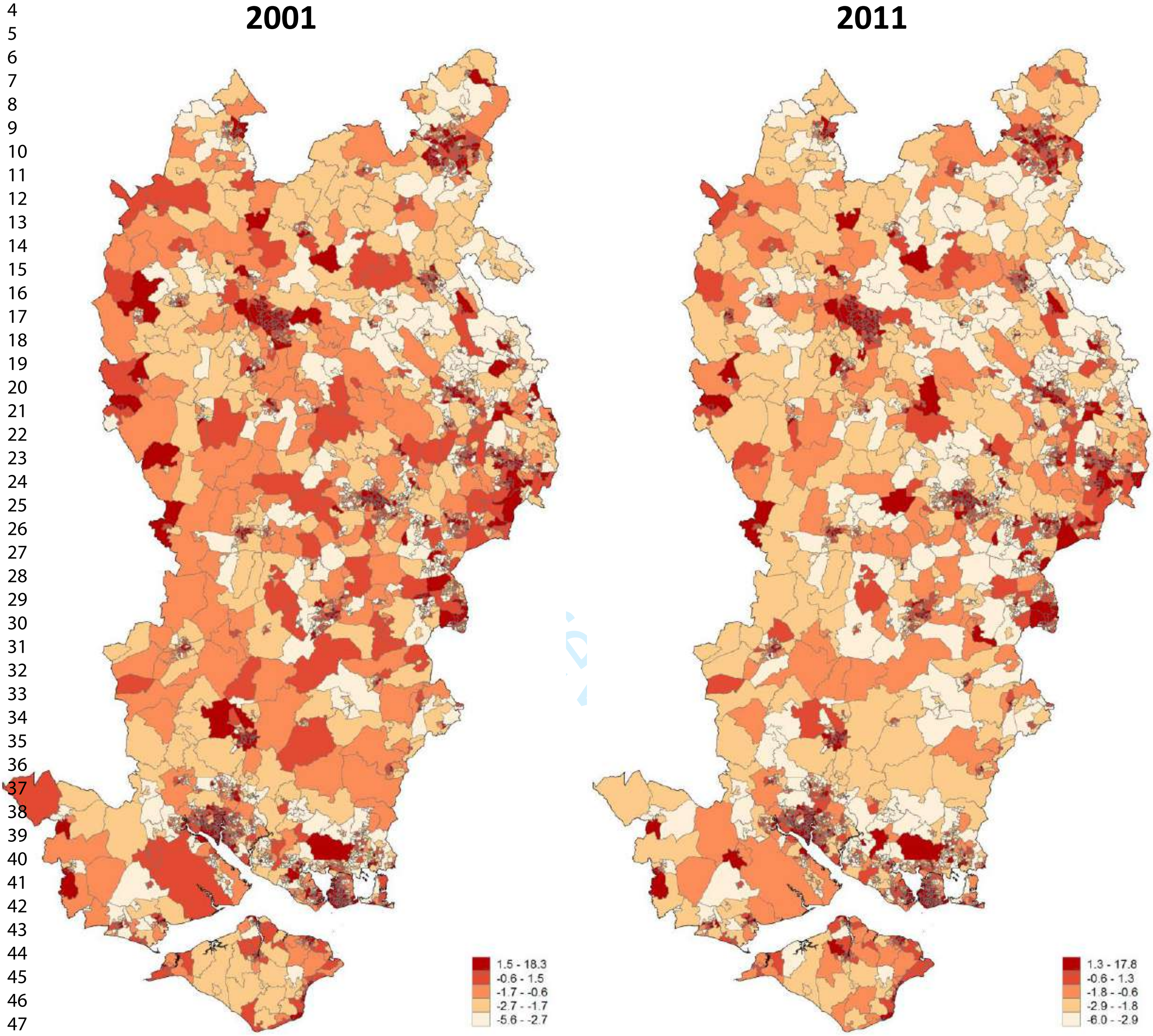

South West, Social Fragmentation

2001

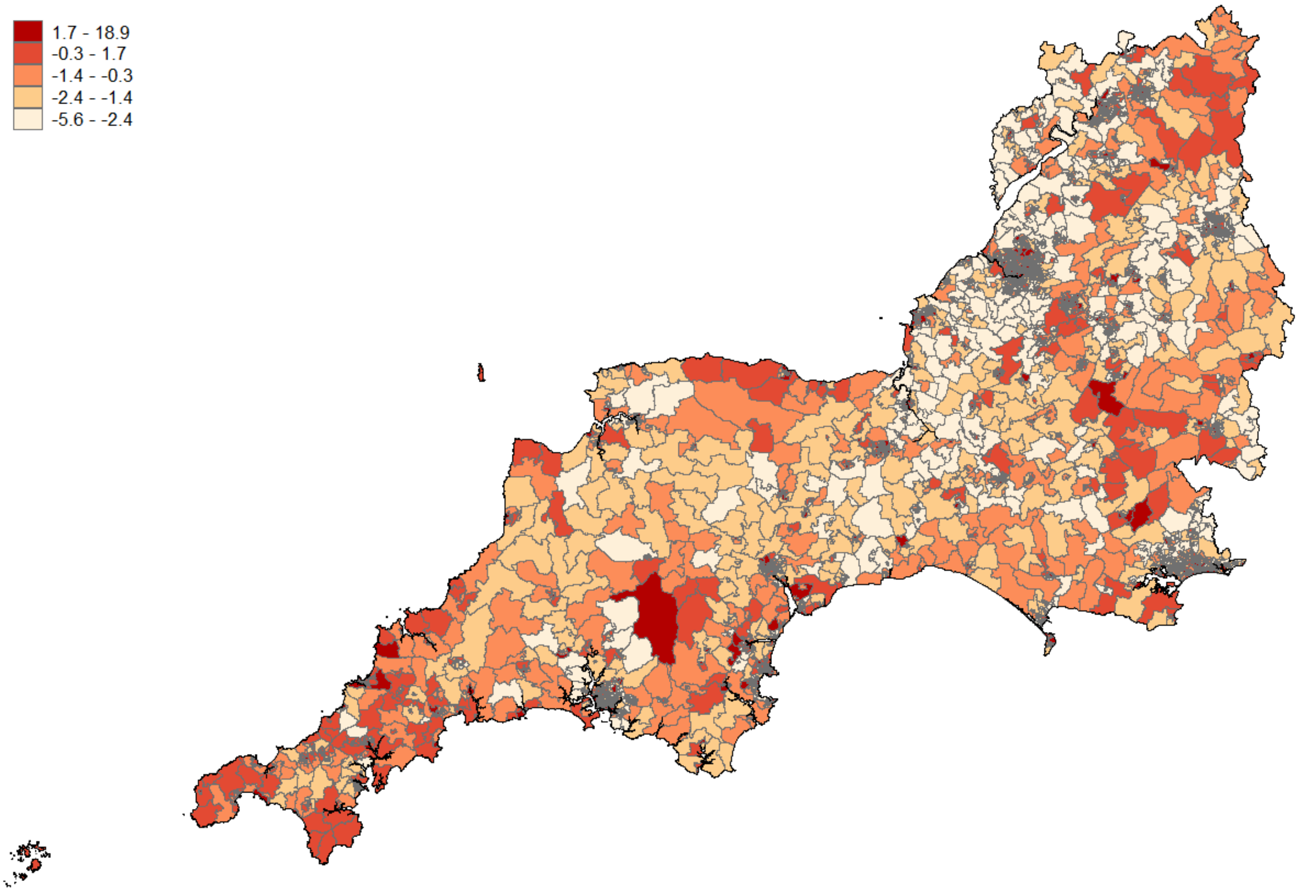

2011

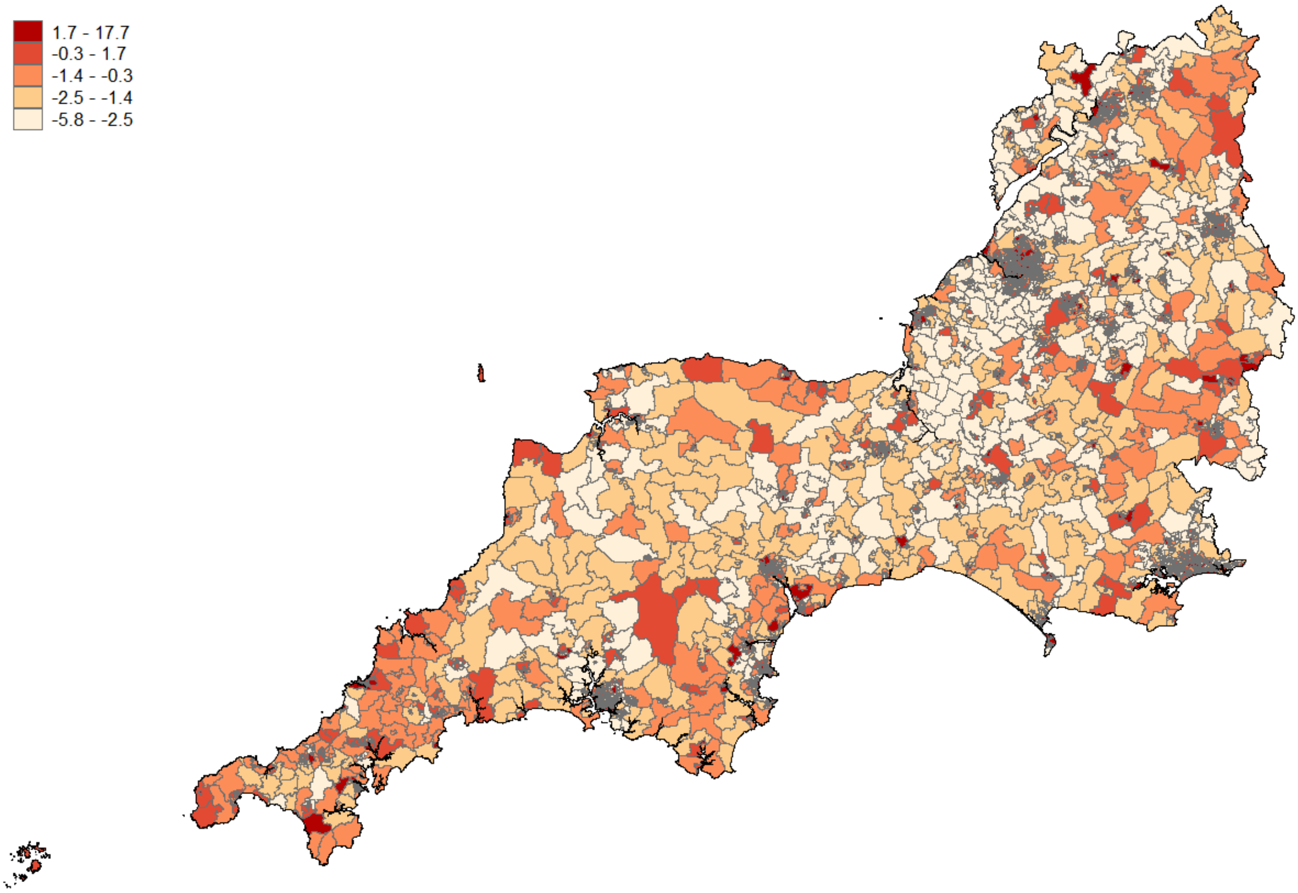

Single People

England 2001, Single People % of population

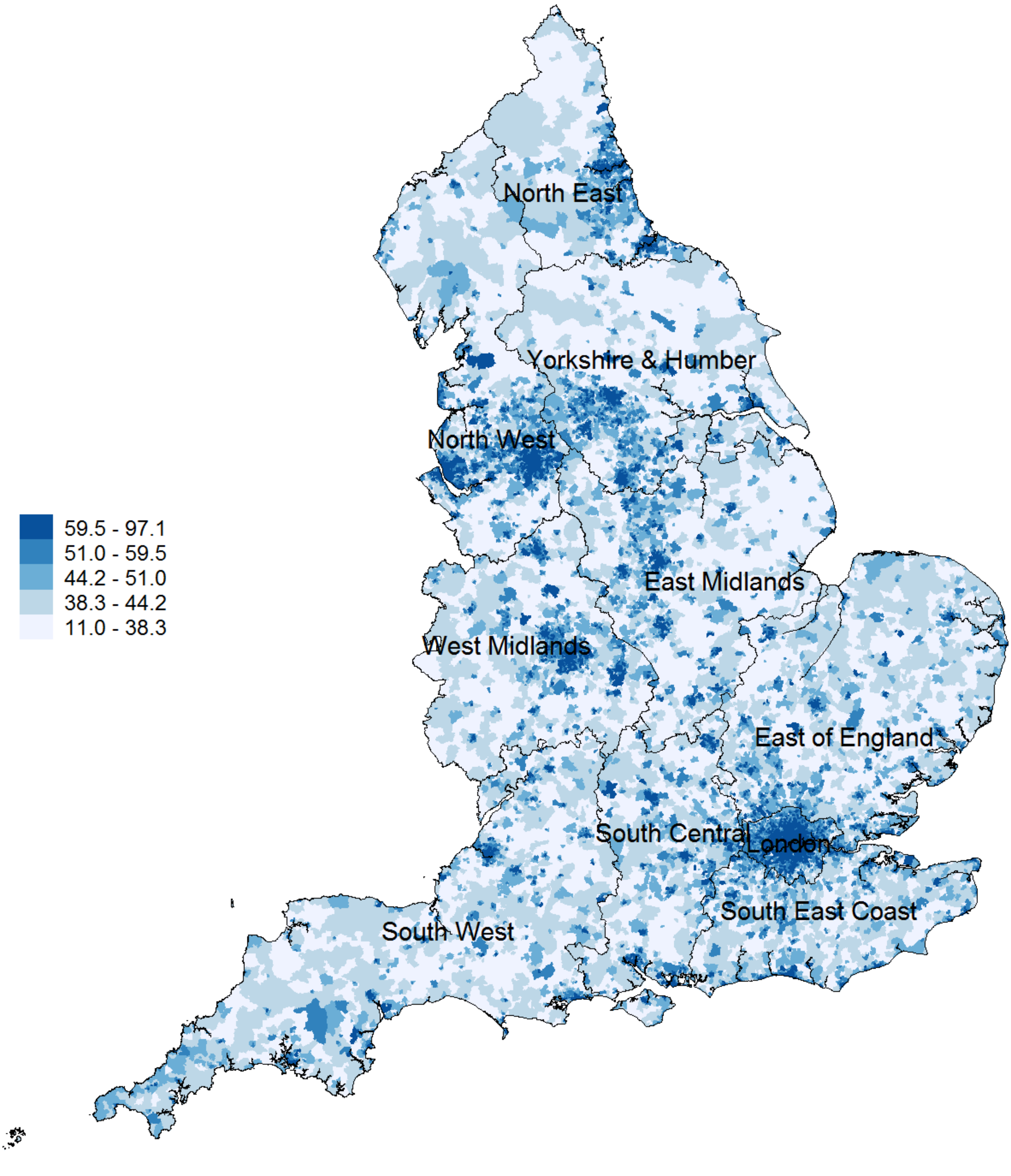

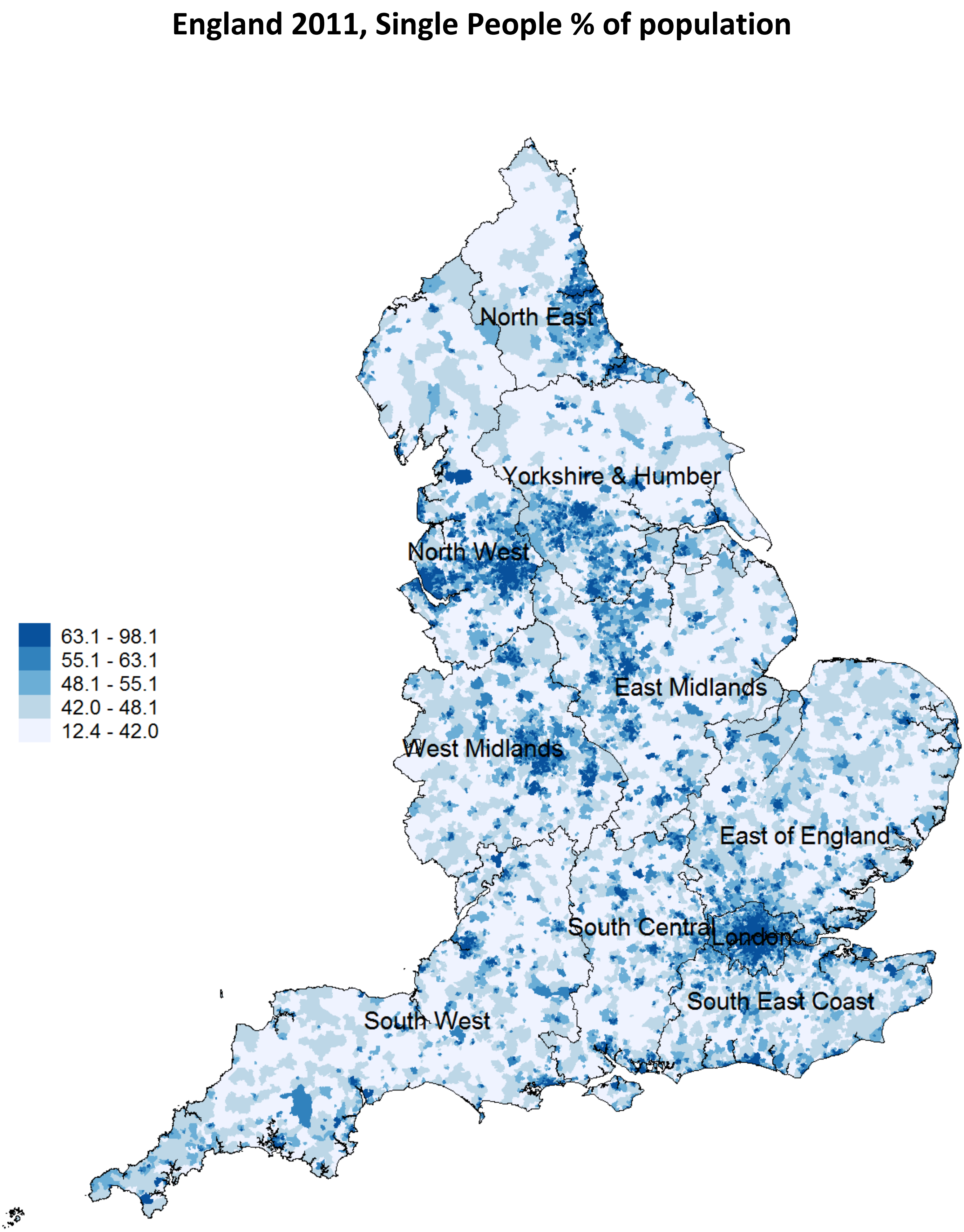
England 2011, Single People % of population
63.1 - 98.1
55.1 - 63.1
48.1 - 55.1
42.0 - 48.1
12.4 - 42.0
North East
Yorkshire & Humber
North West
East Midlands
West Midlands
East of England
South Central
London
South East Coast
South West

North East, Single People % of population

2001

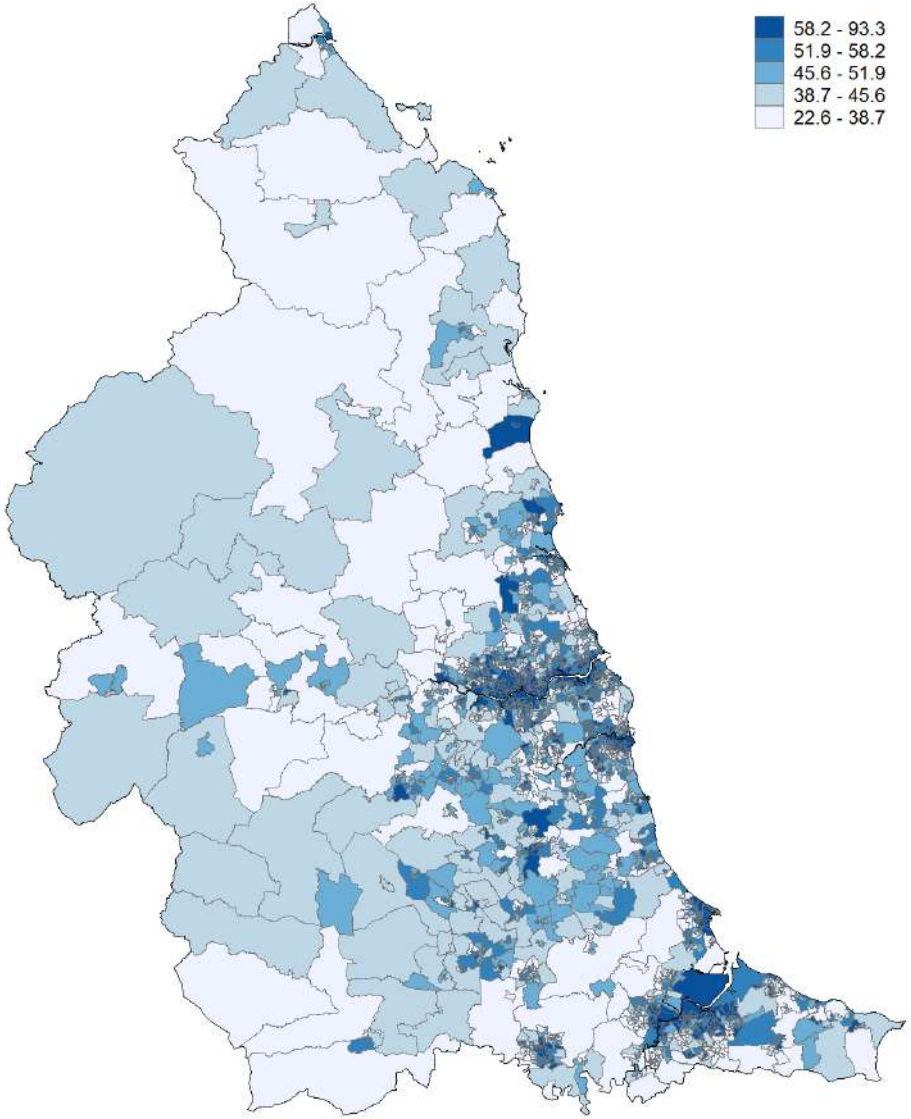

2011

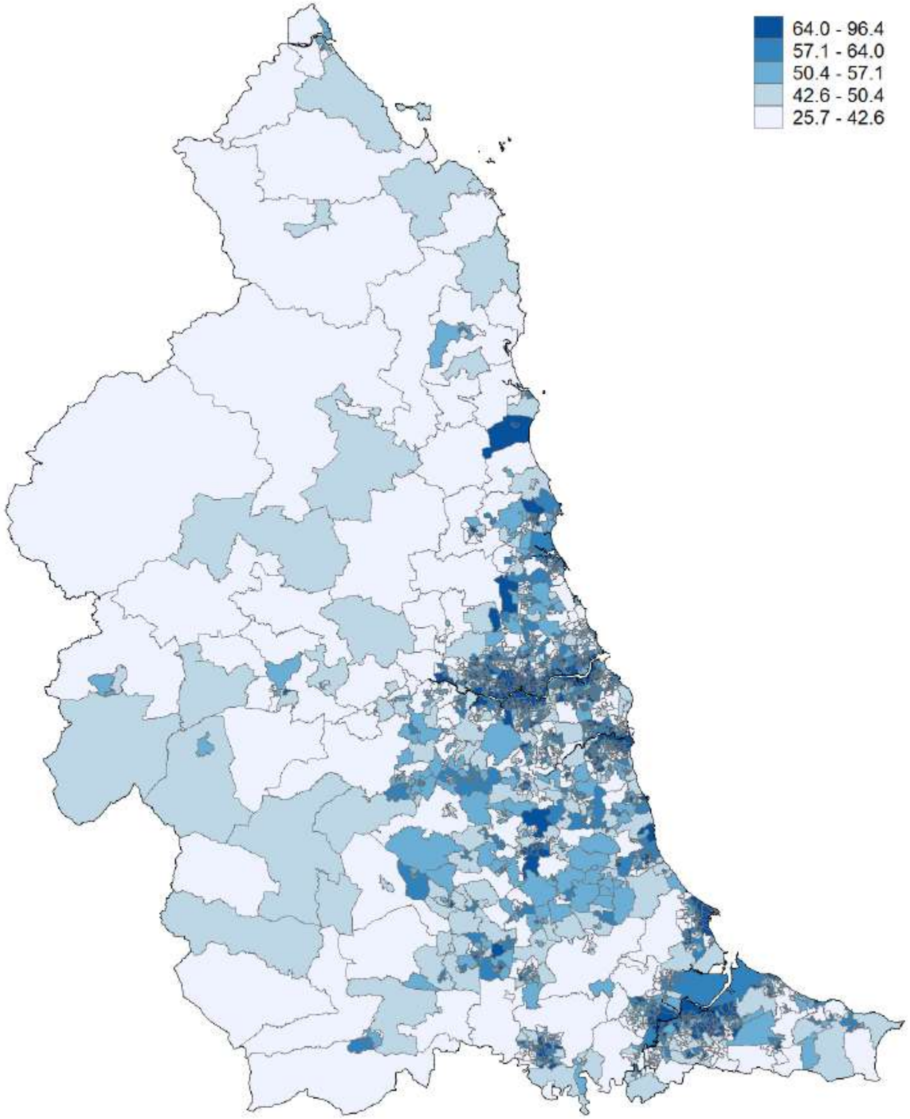

North West, Single People % of population

2001

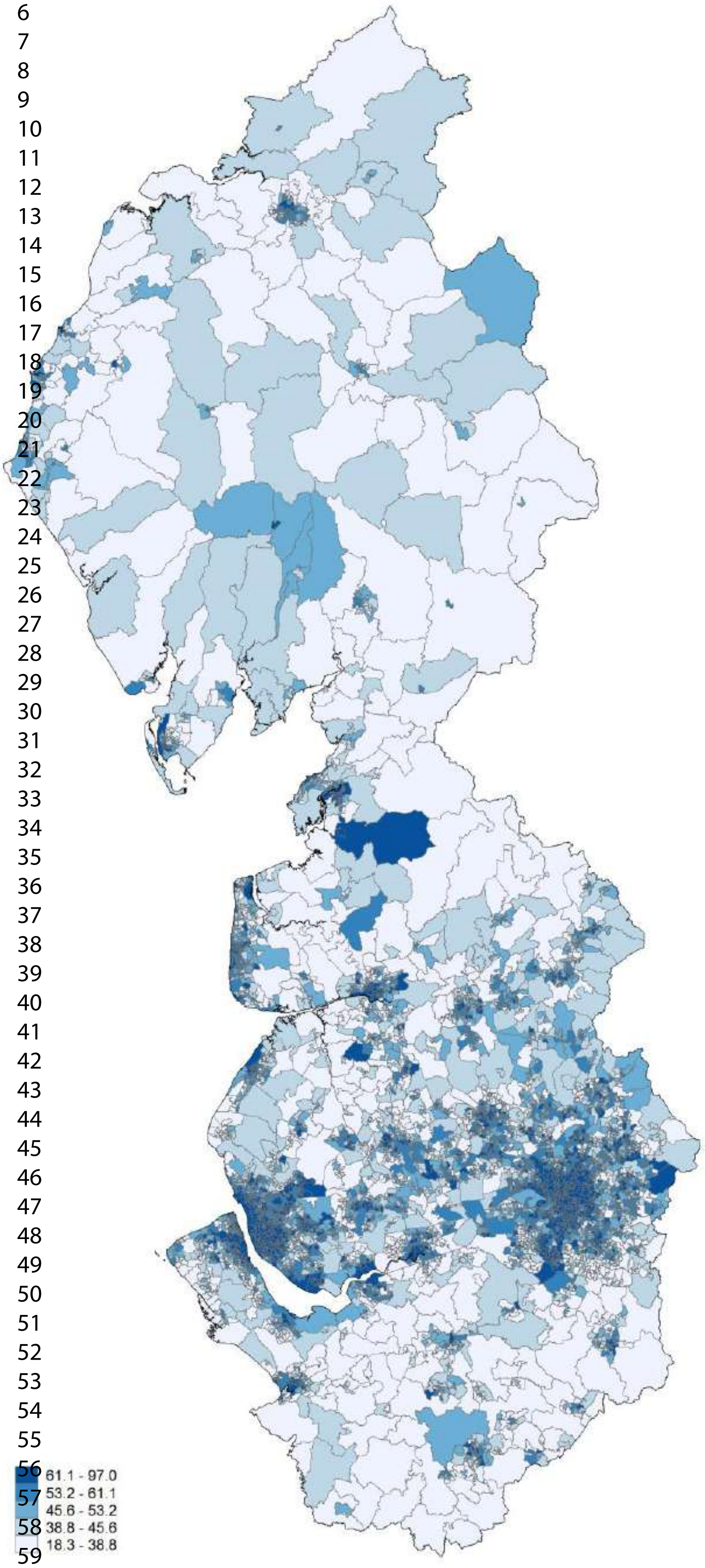

2011

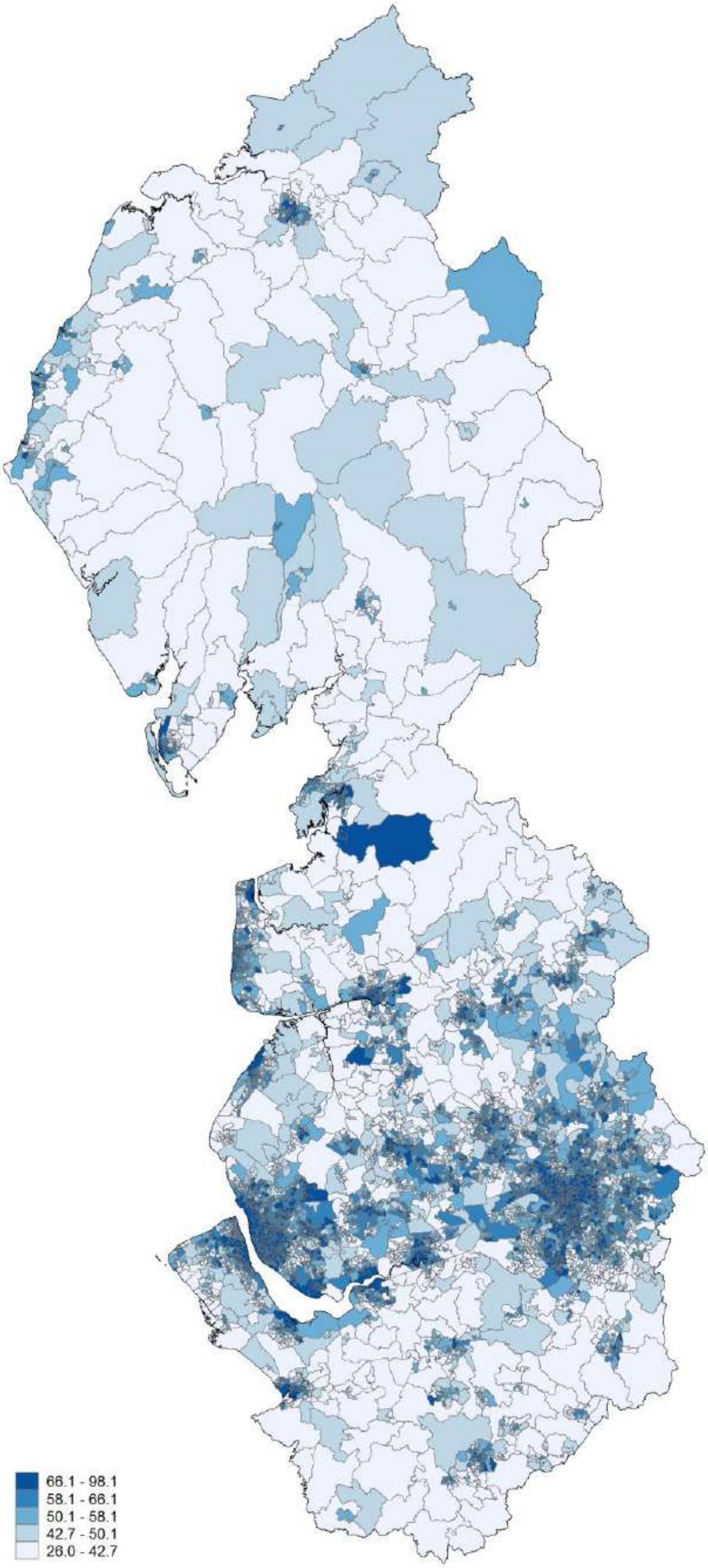

Yorkshire and the Humber, Single People % of population

2001

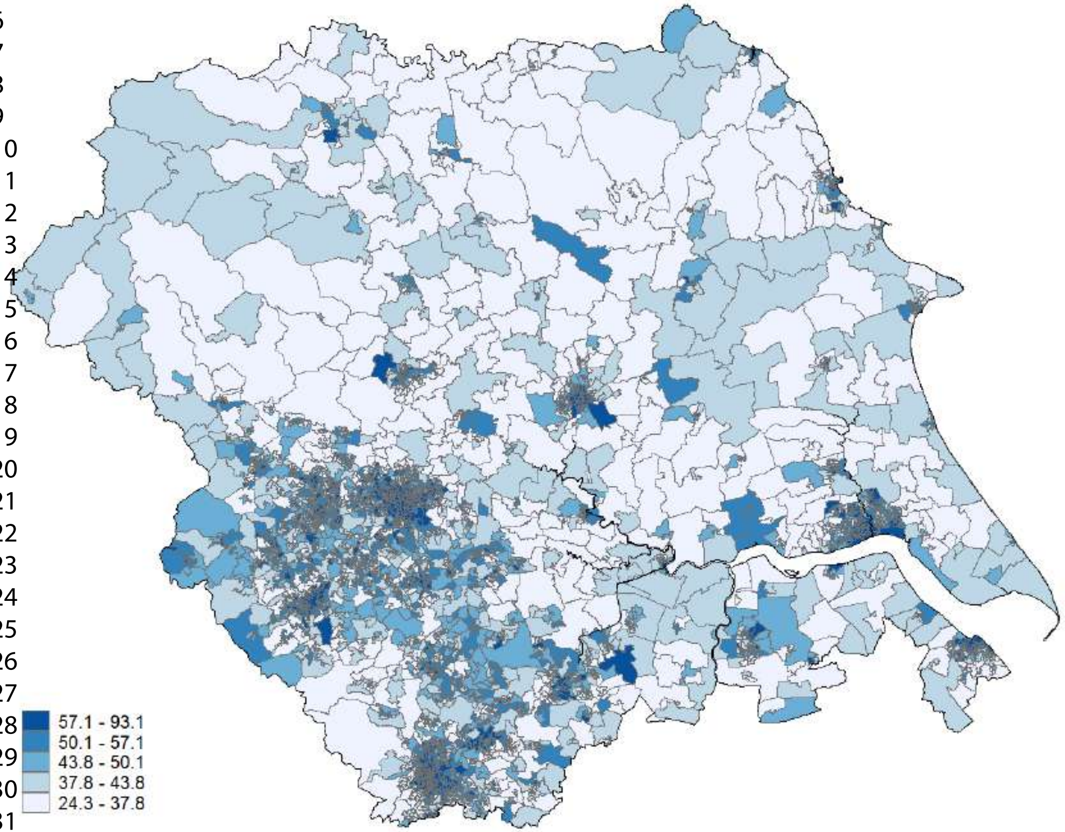

2011

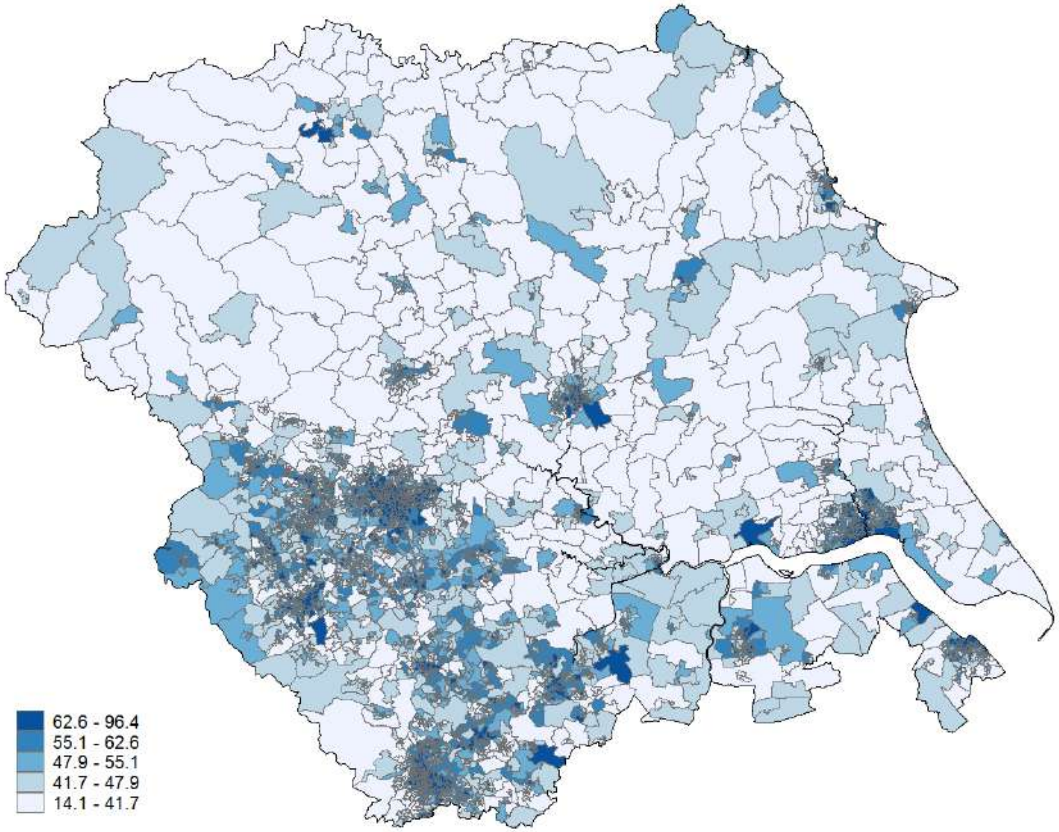

East Midlands, Single People % of population

2001 **2011**

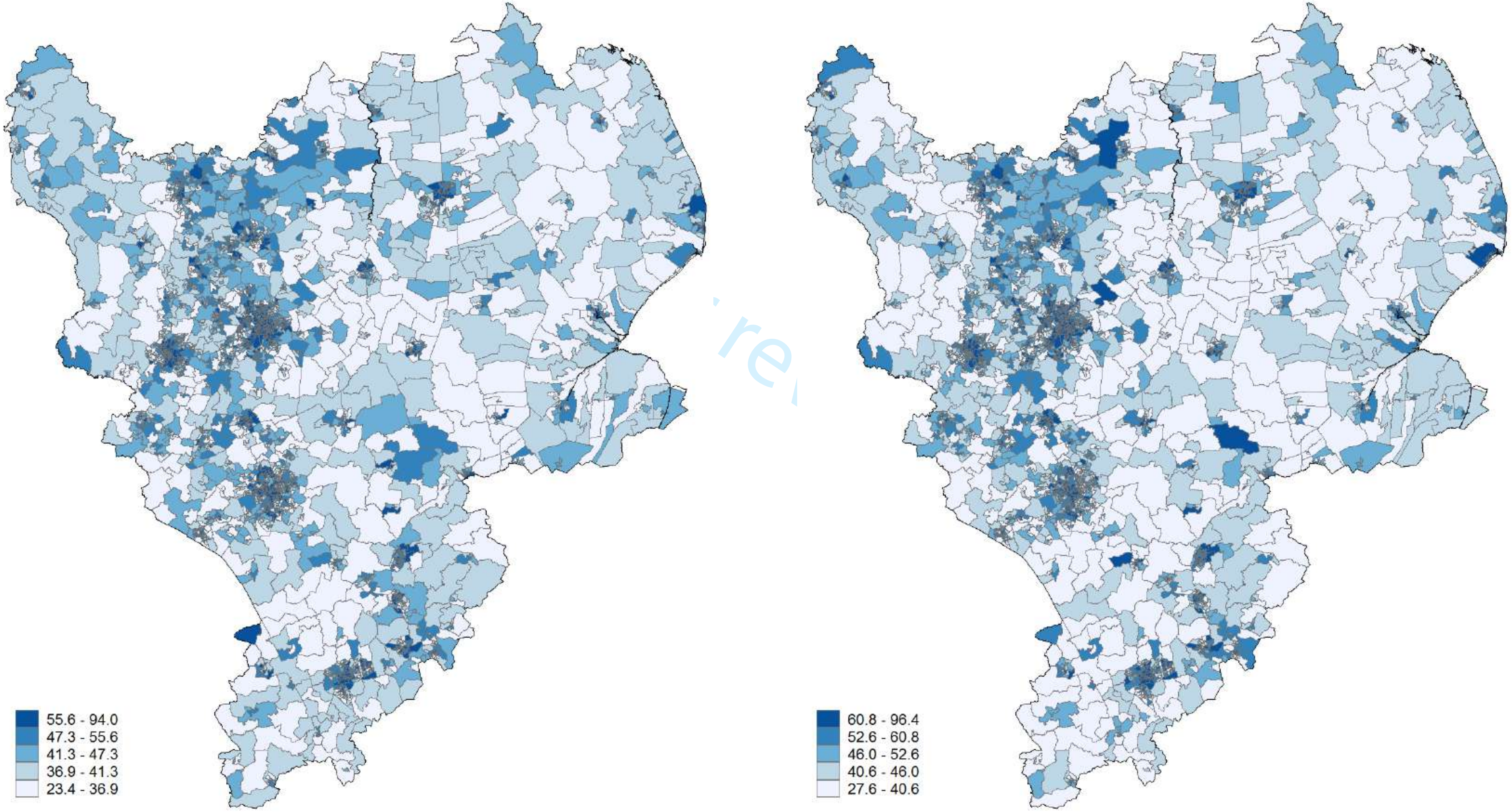

West Midlands, Single People % of population

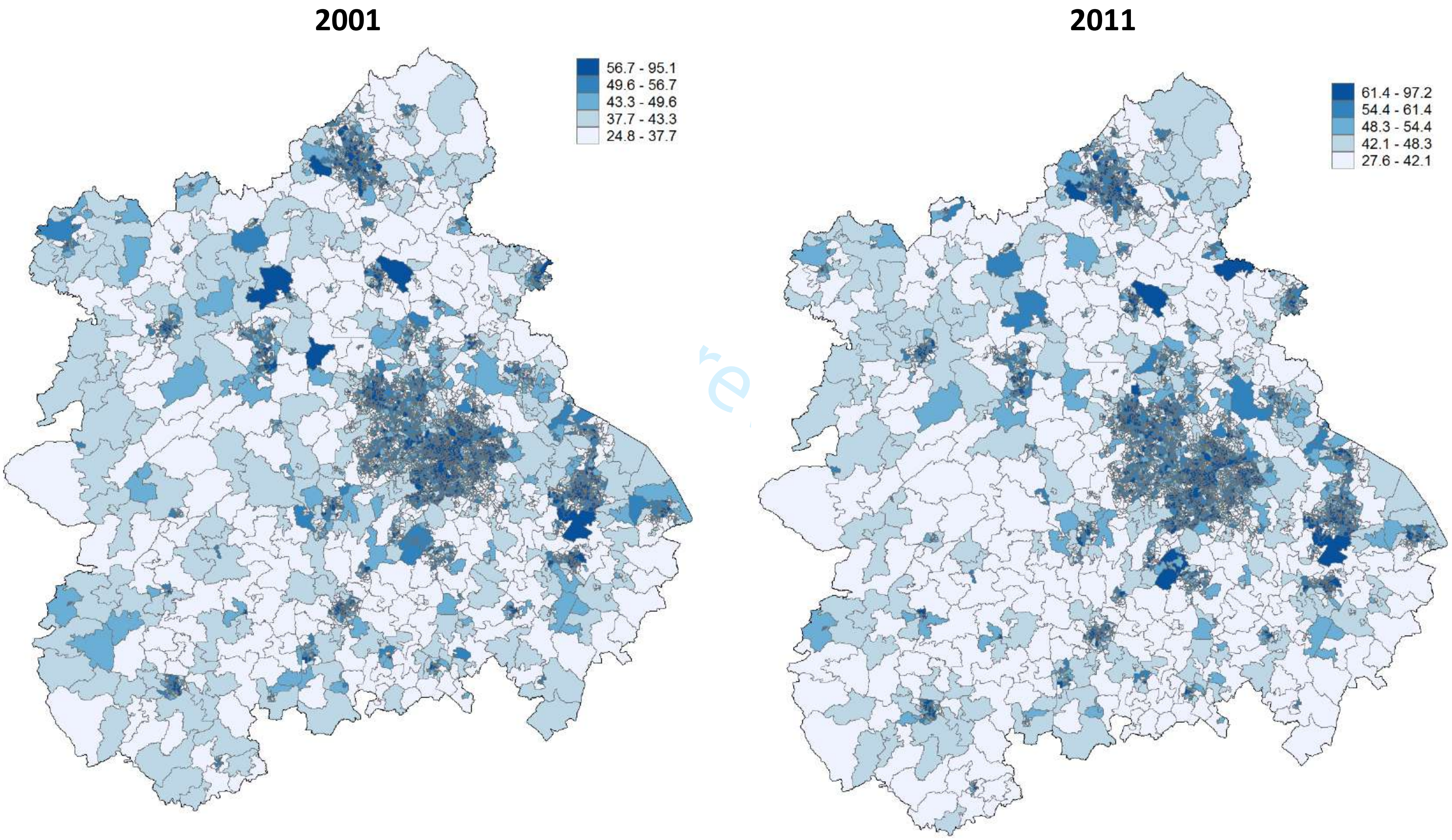

East of England, Single People % of population

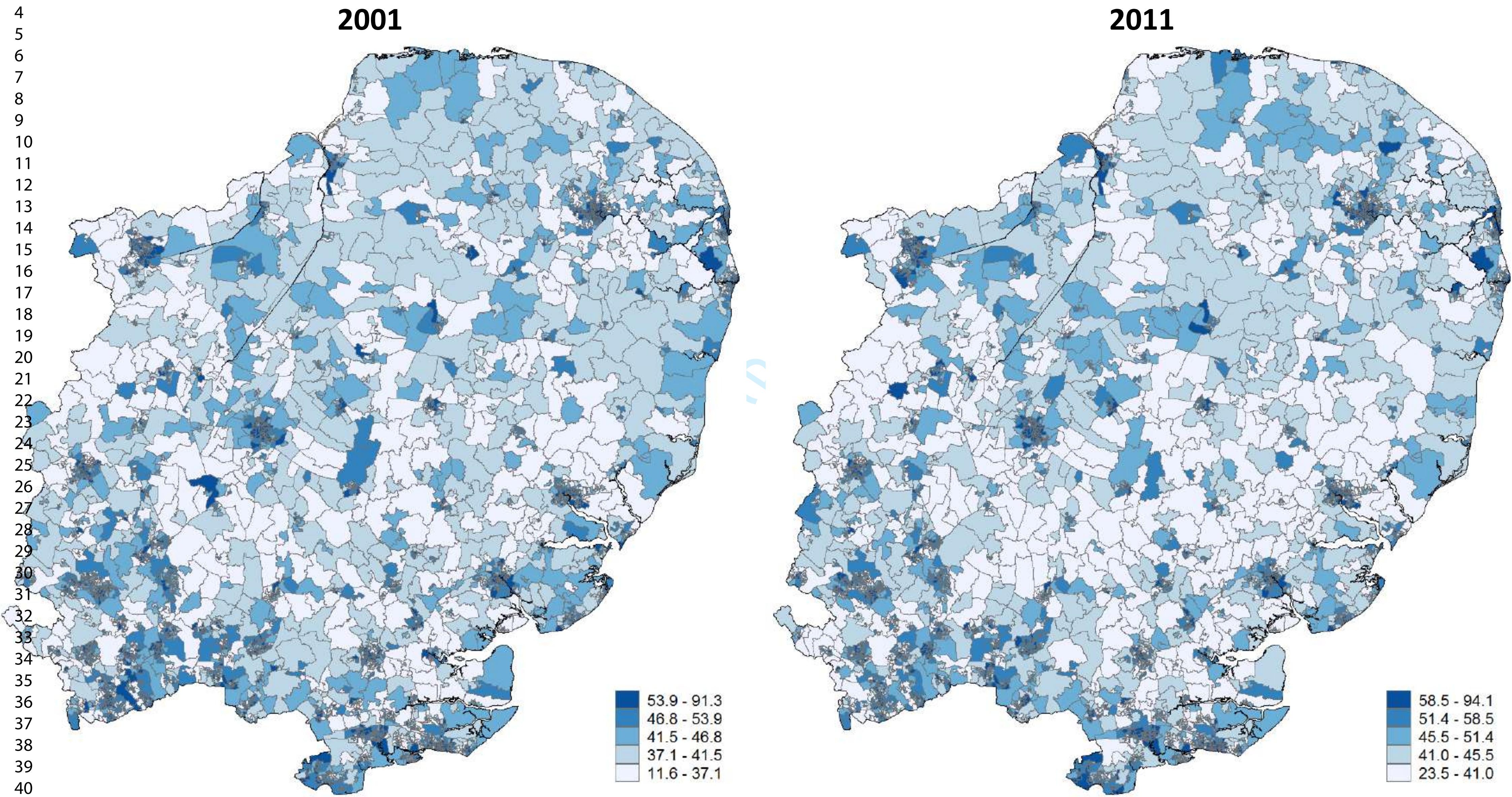

London, Single People % of population

2001

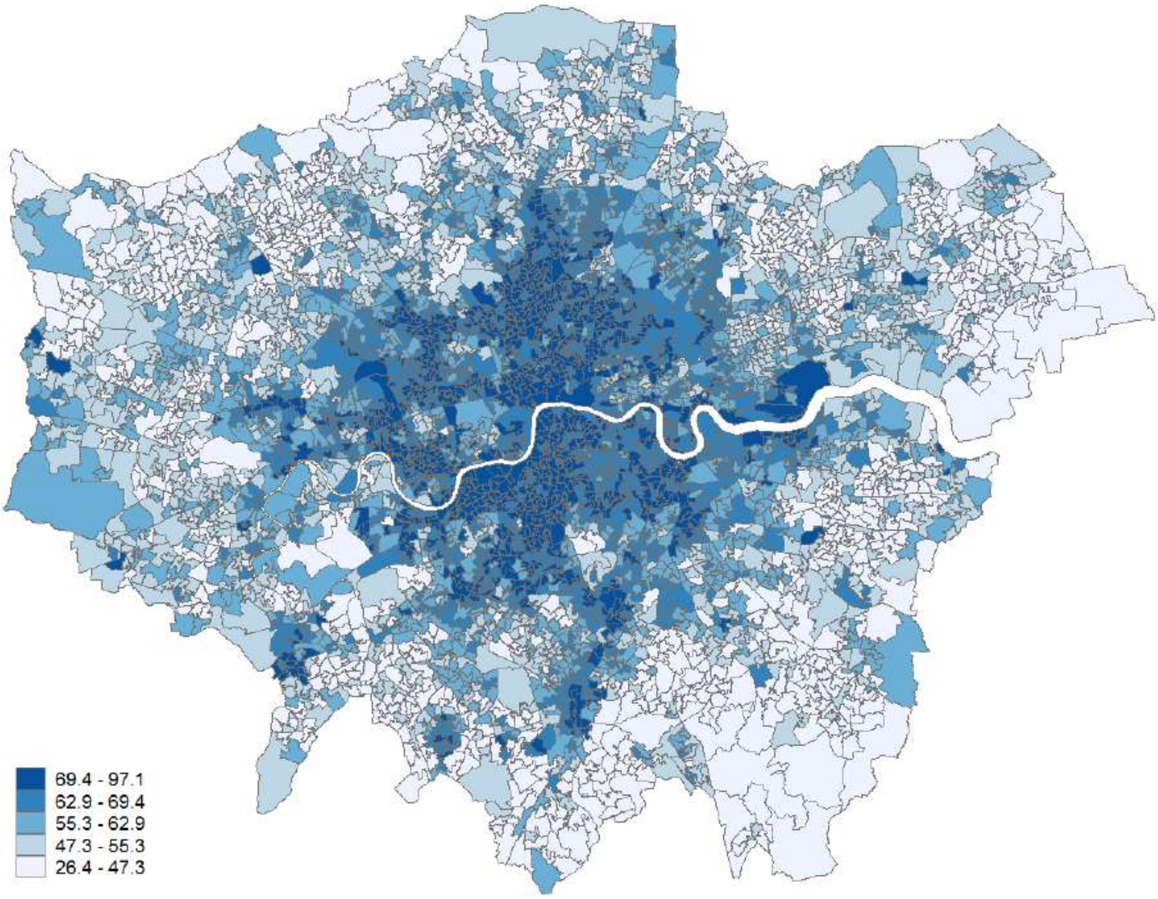

2011

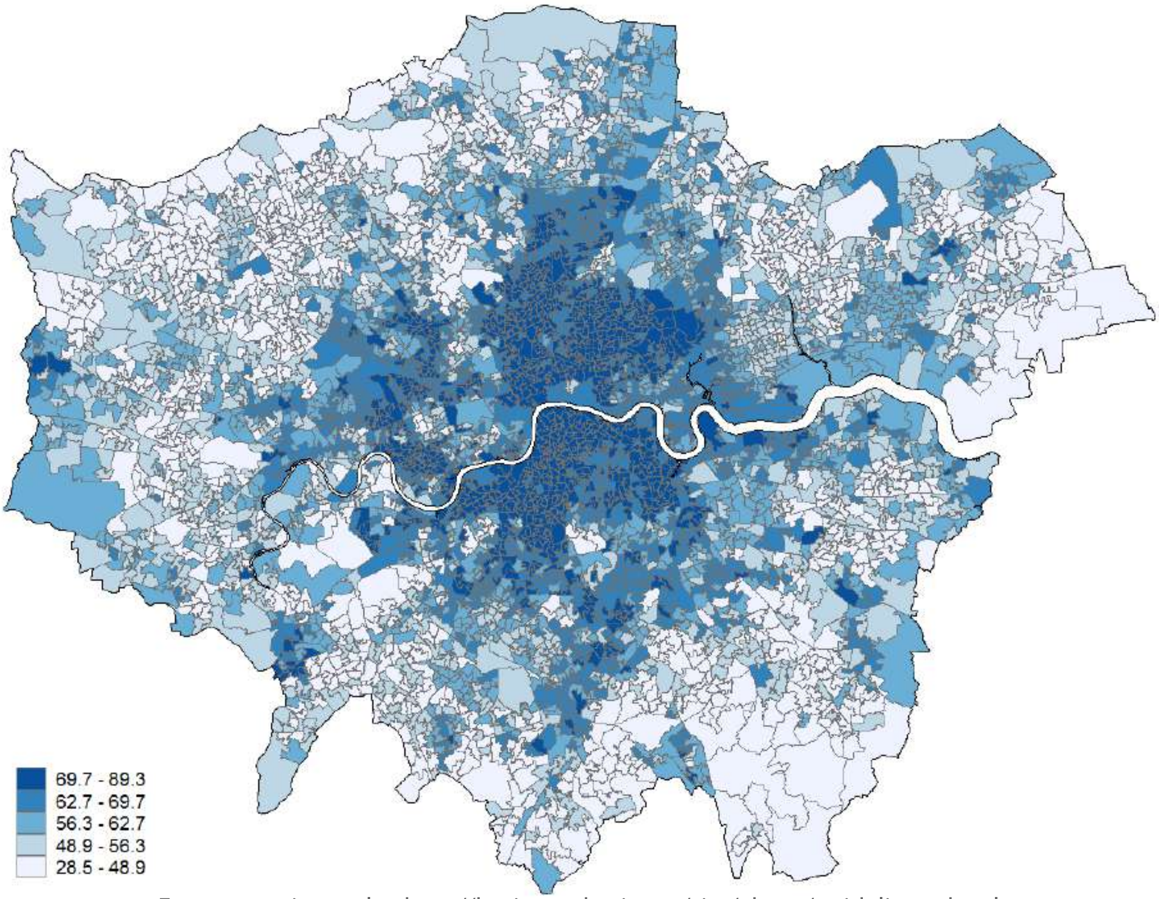

South East Coast, Single People % of population

2001

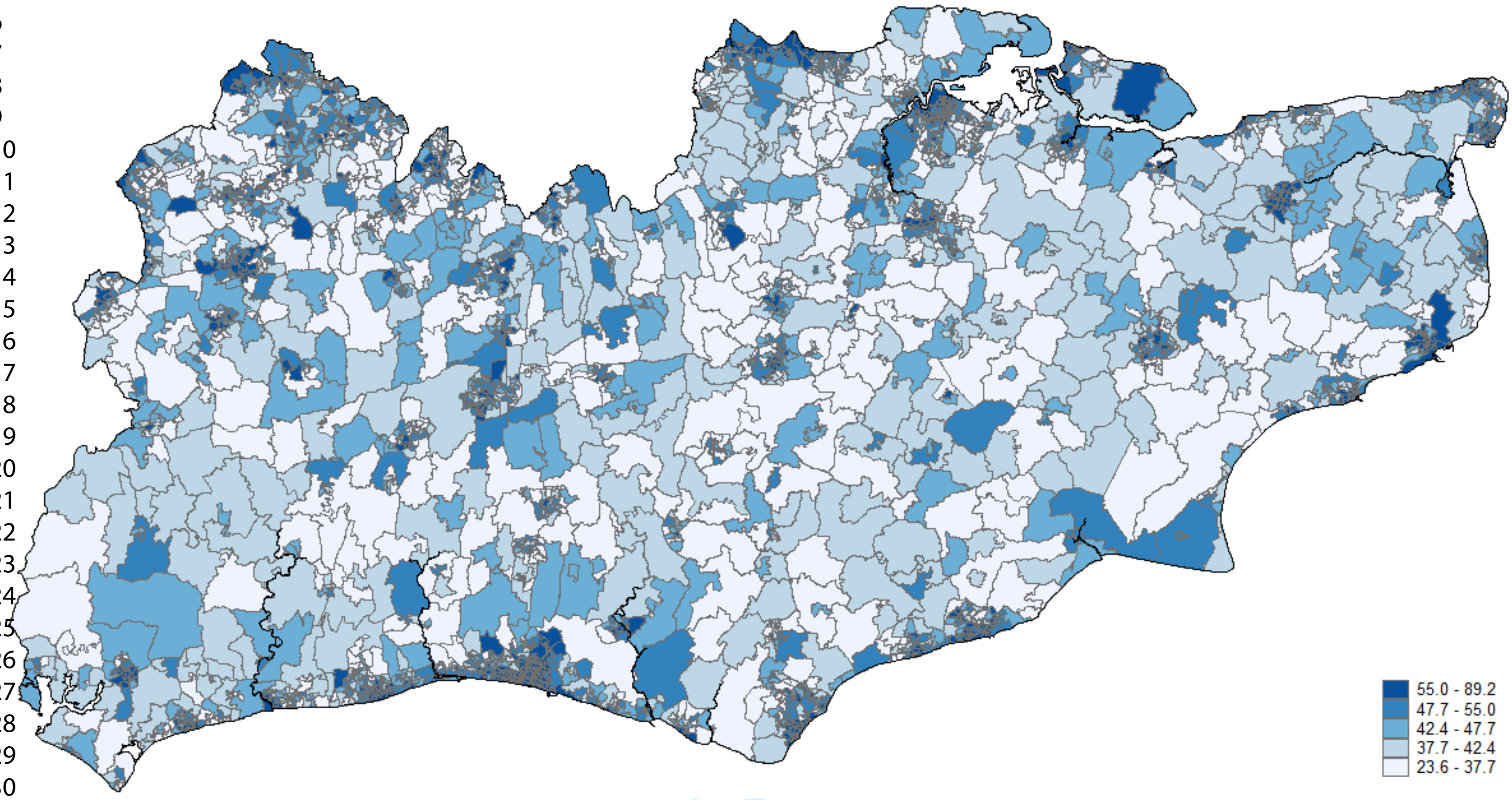

2011

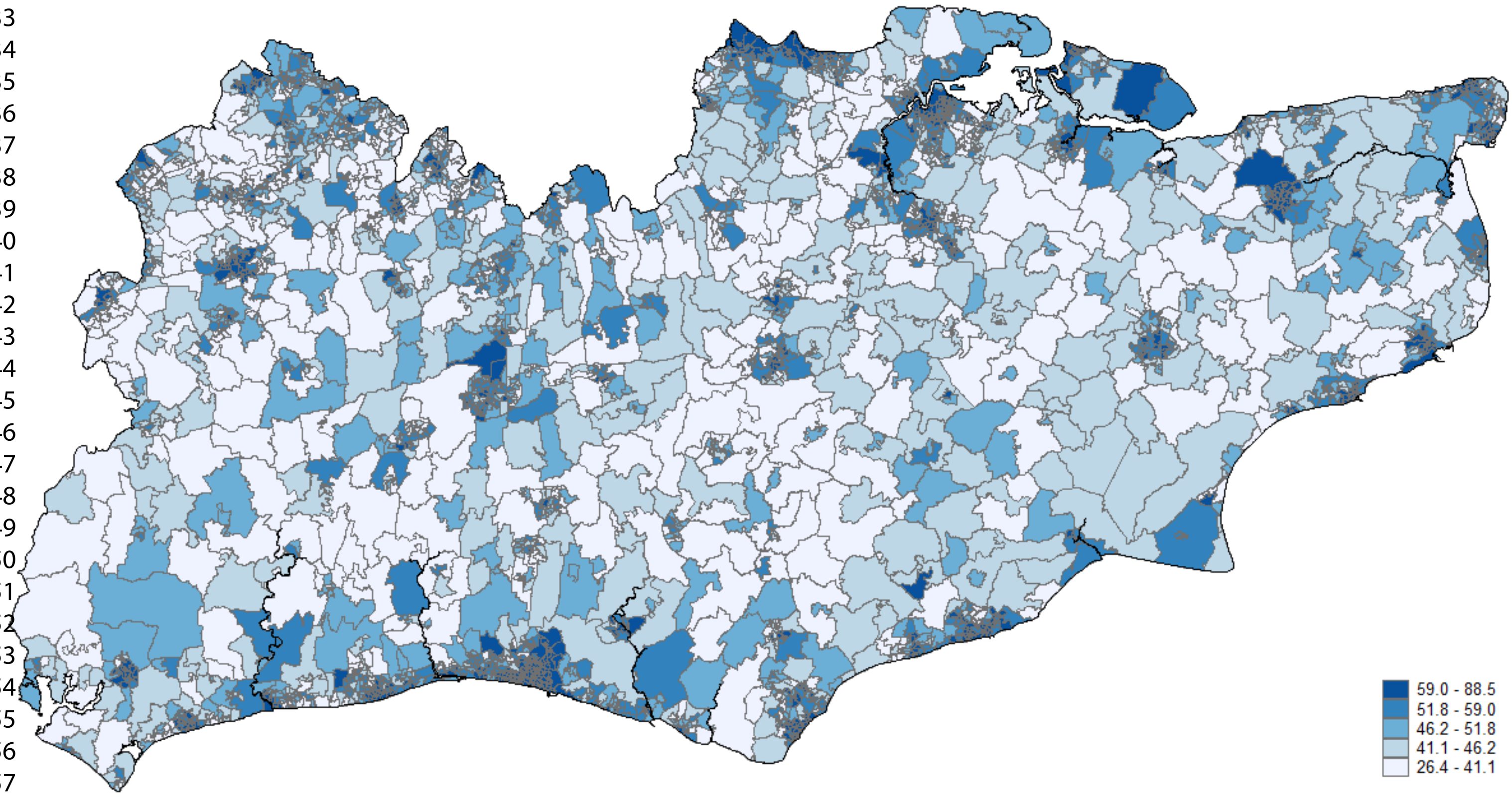

South Central, Single People % of population

2001

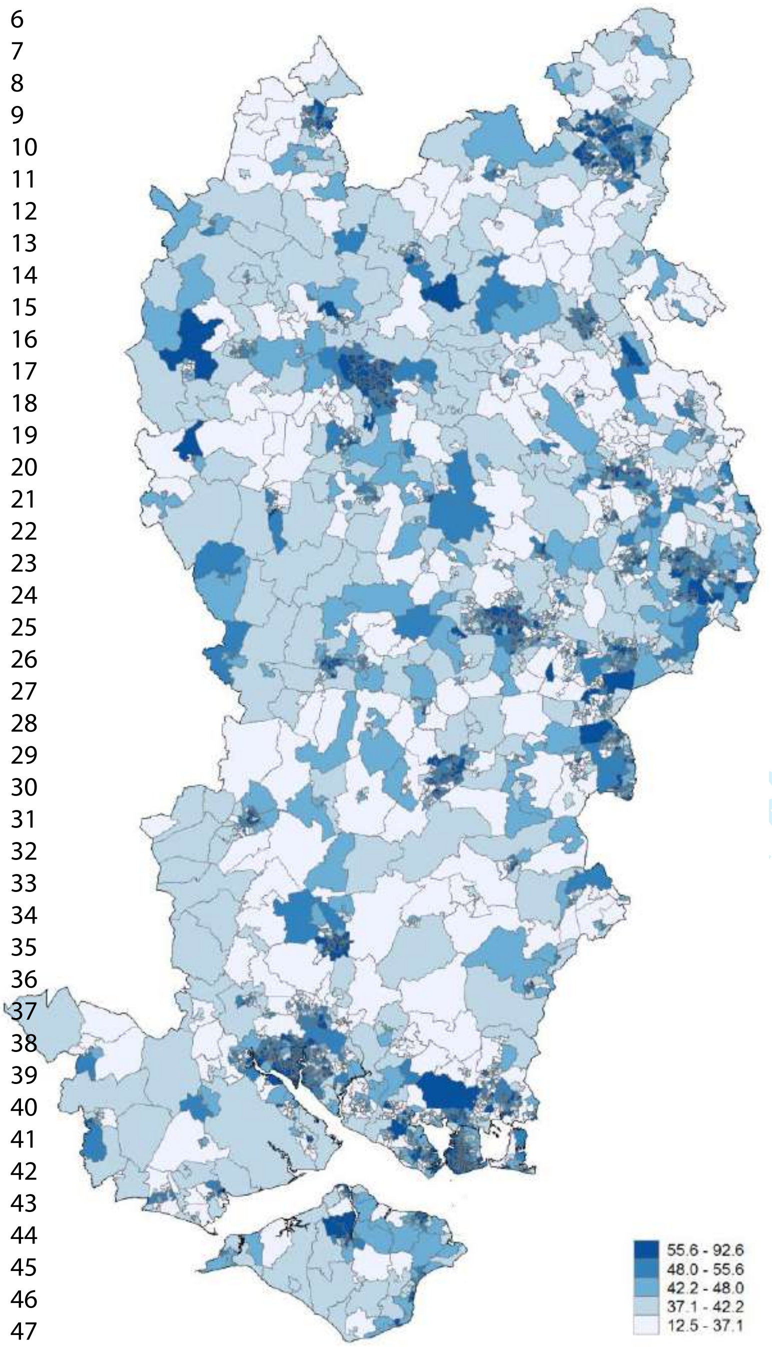

2011

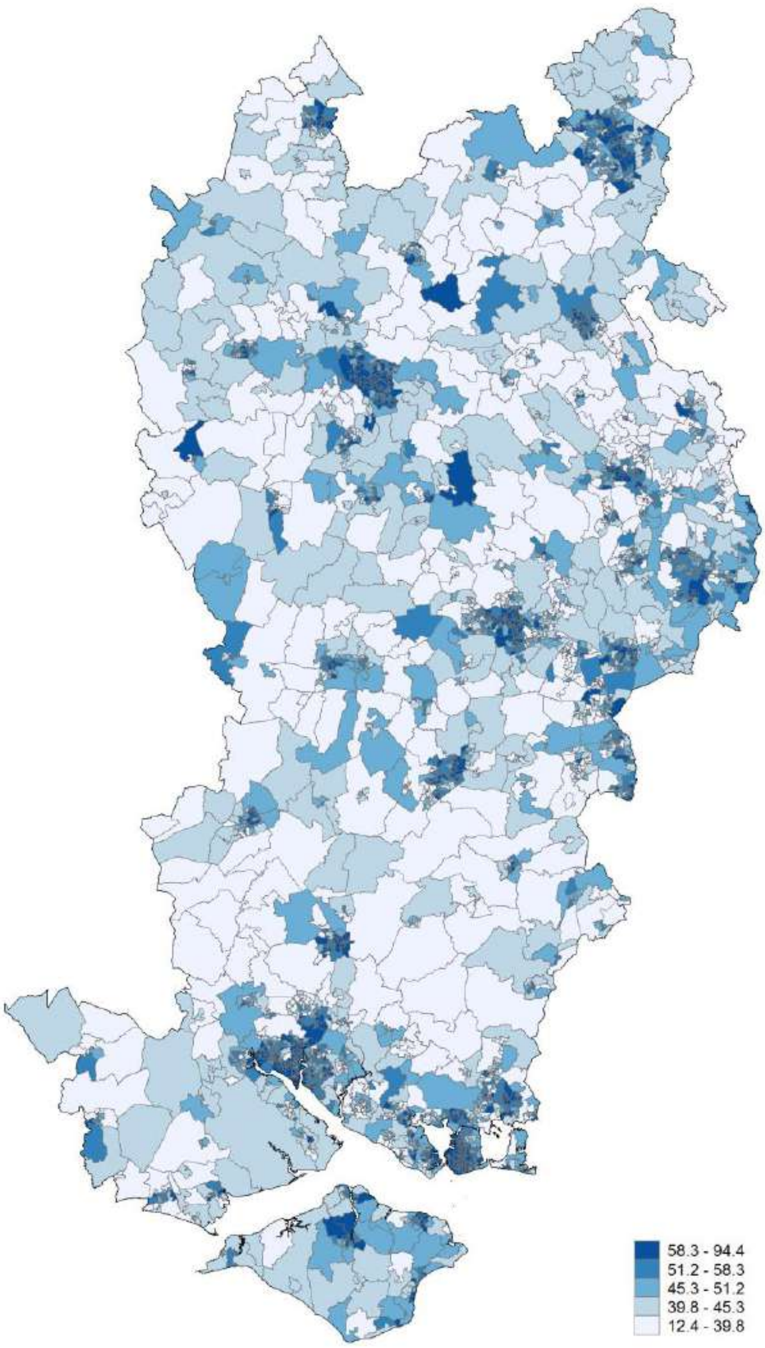

South West, Single People % of population

2001

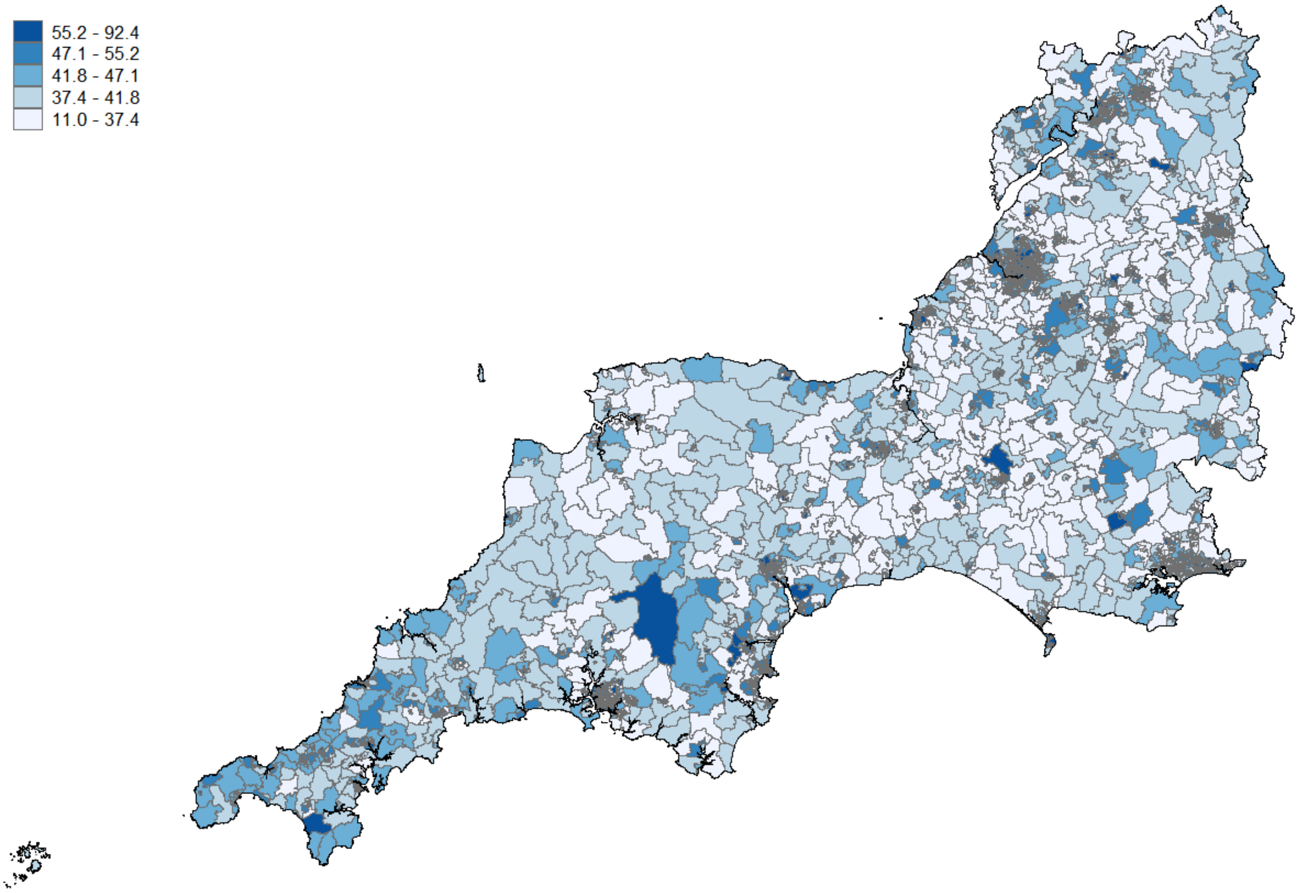

2011

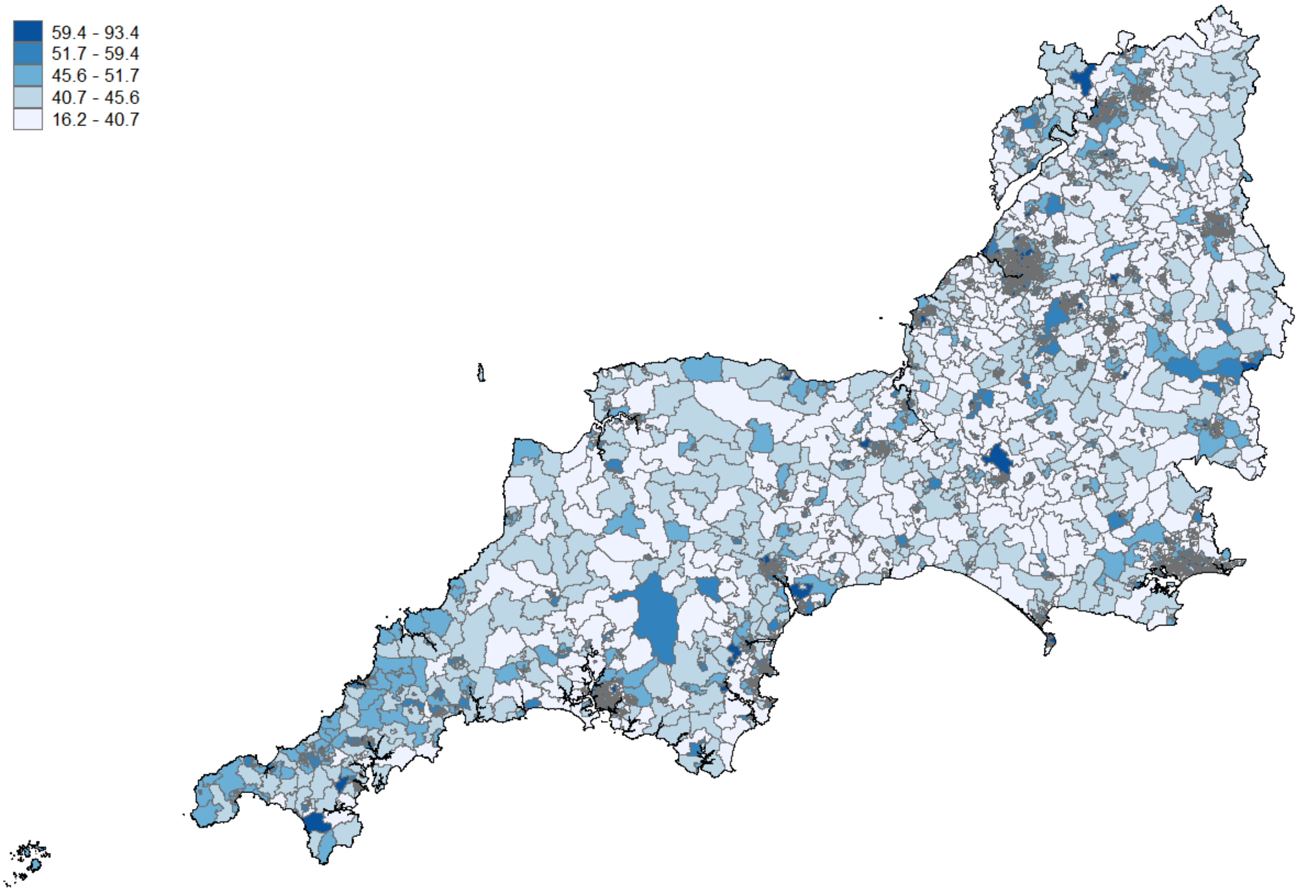

One Person Households

England 2001, One Person Households % of all households

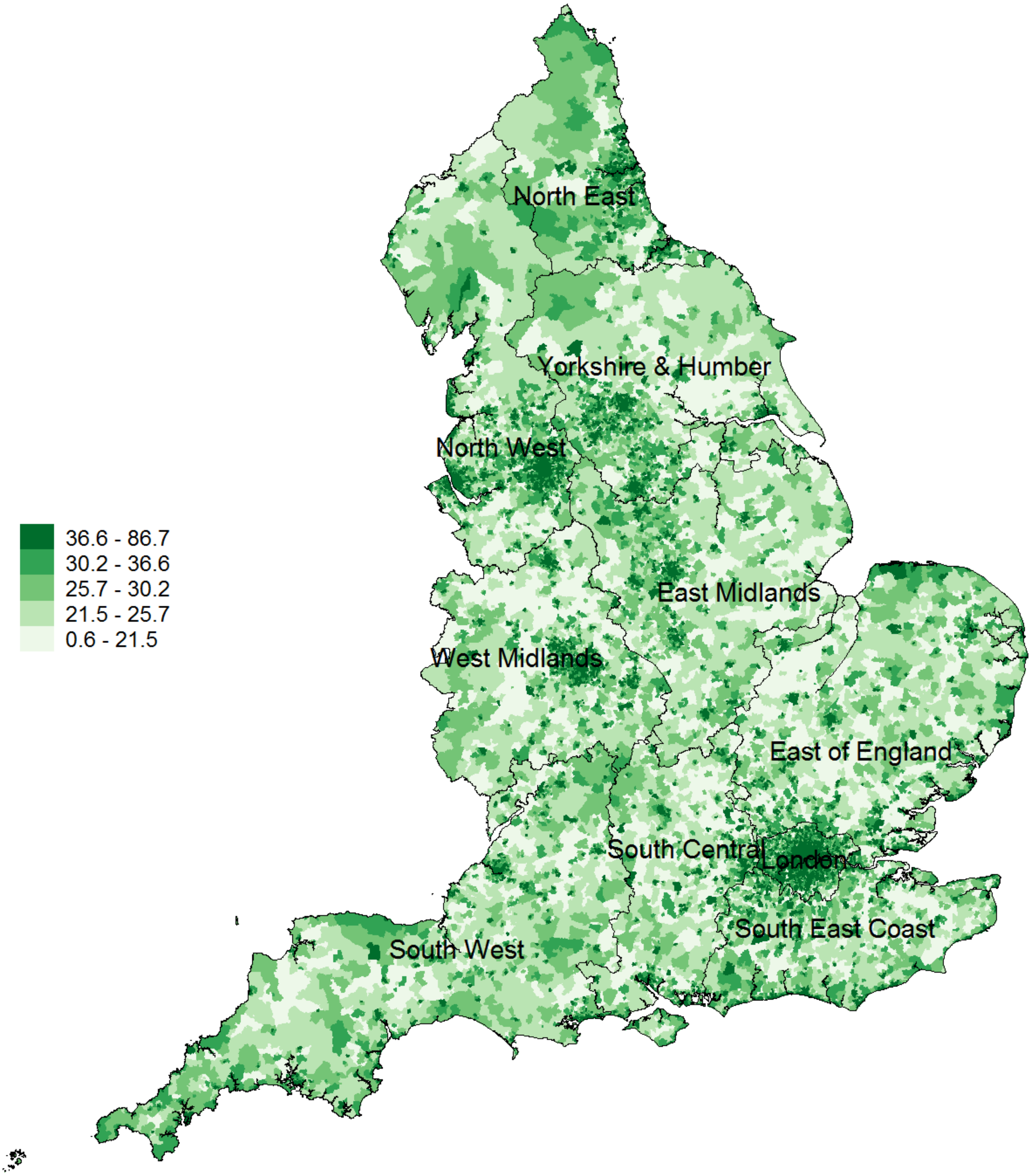

England 2011, One Person Households % of all households

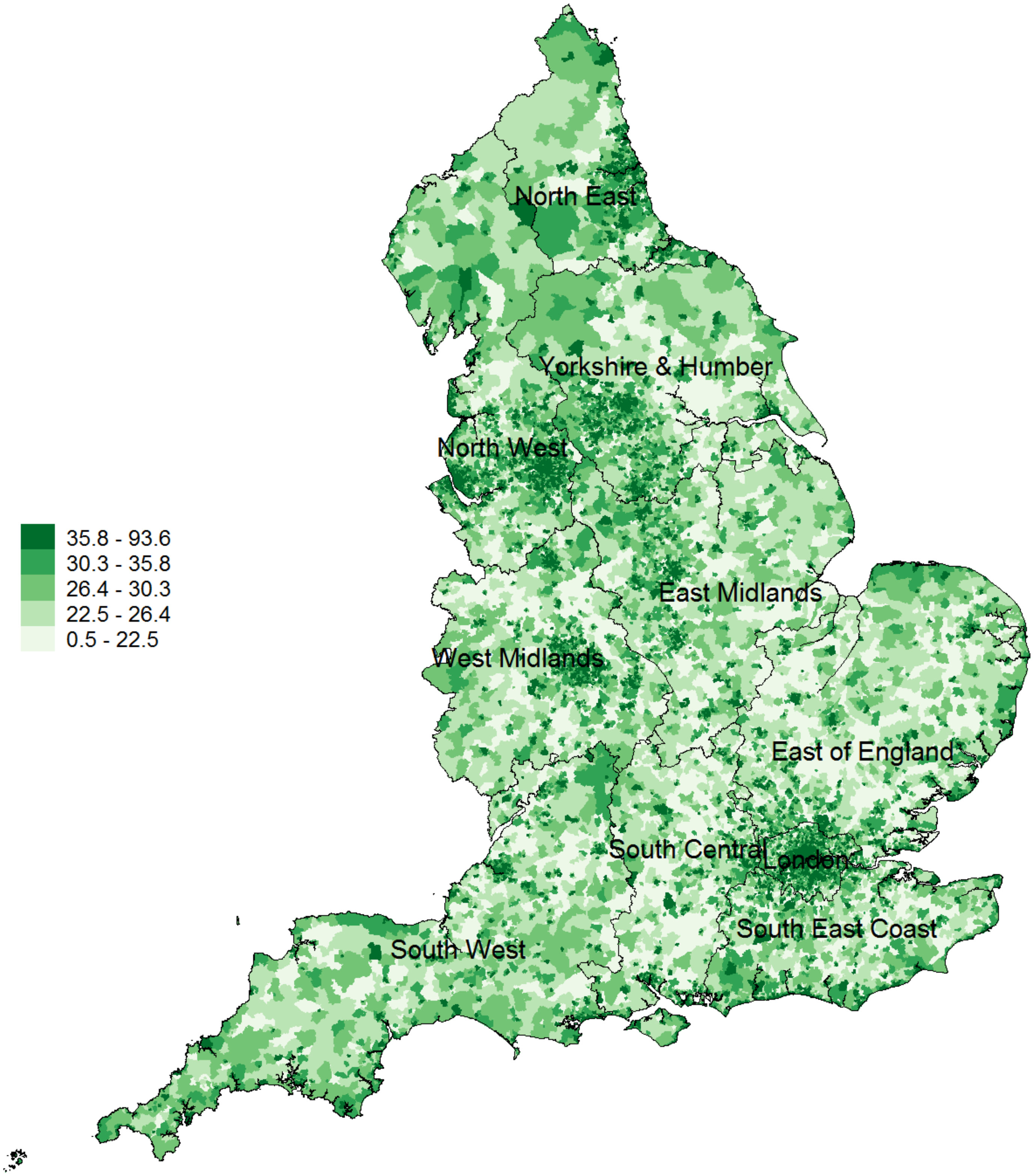

North East, One Person Households % of all households

2001

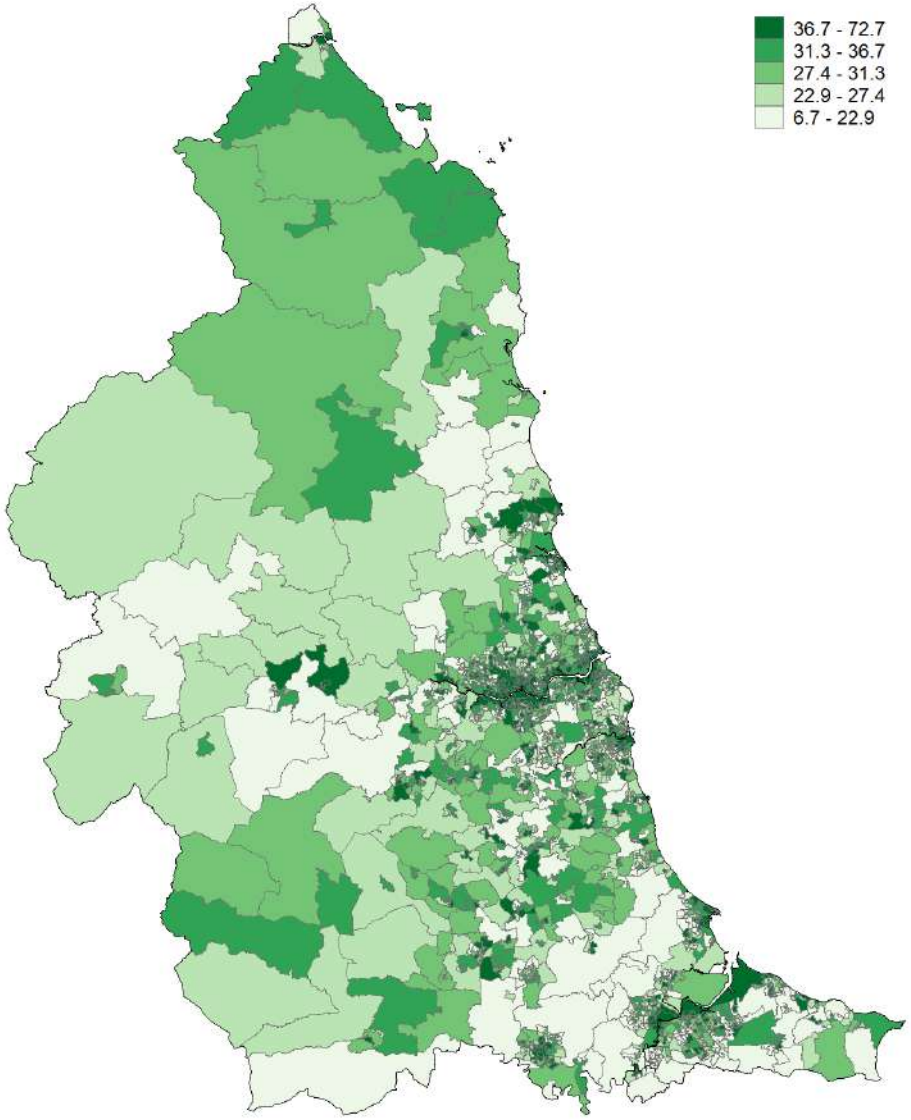

2011

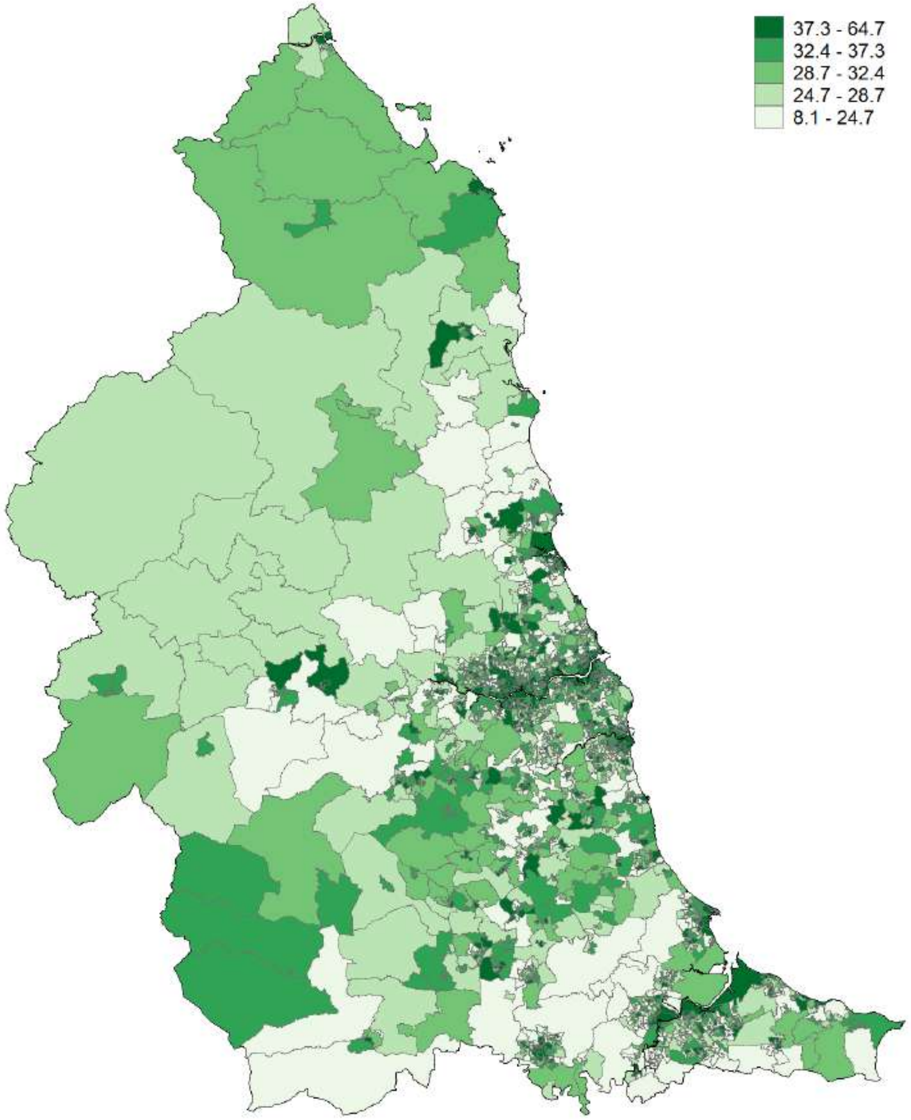

North West, One Person Households % of all households

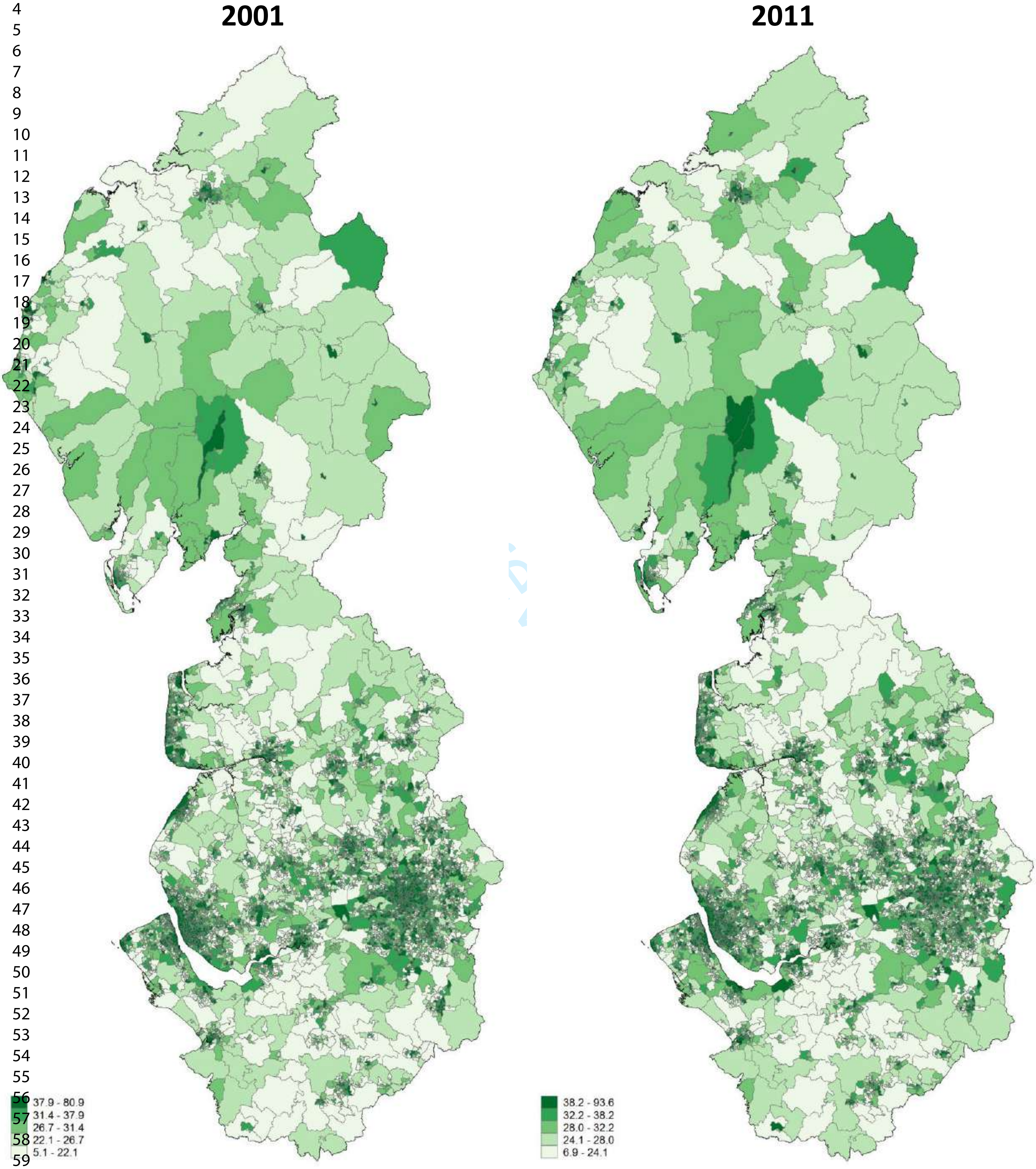

Yorkshire and the Humber, One Person Households % of all households

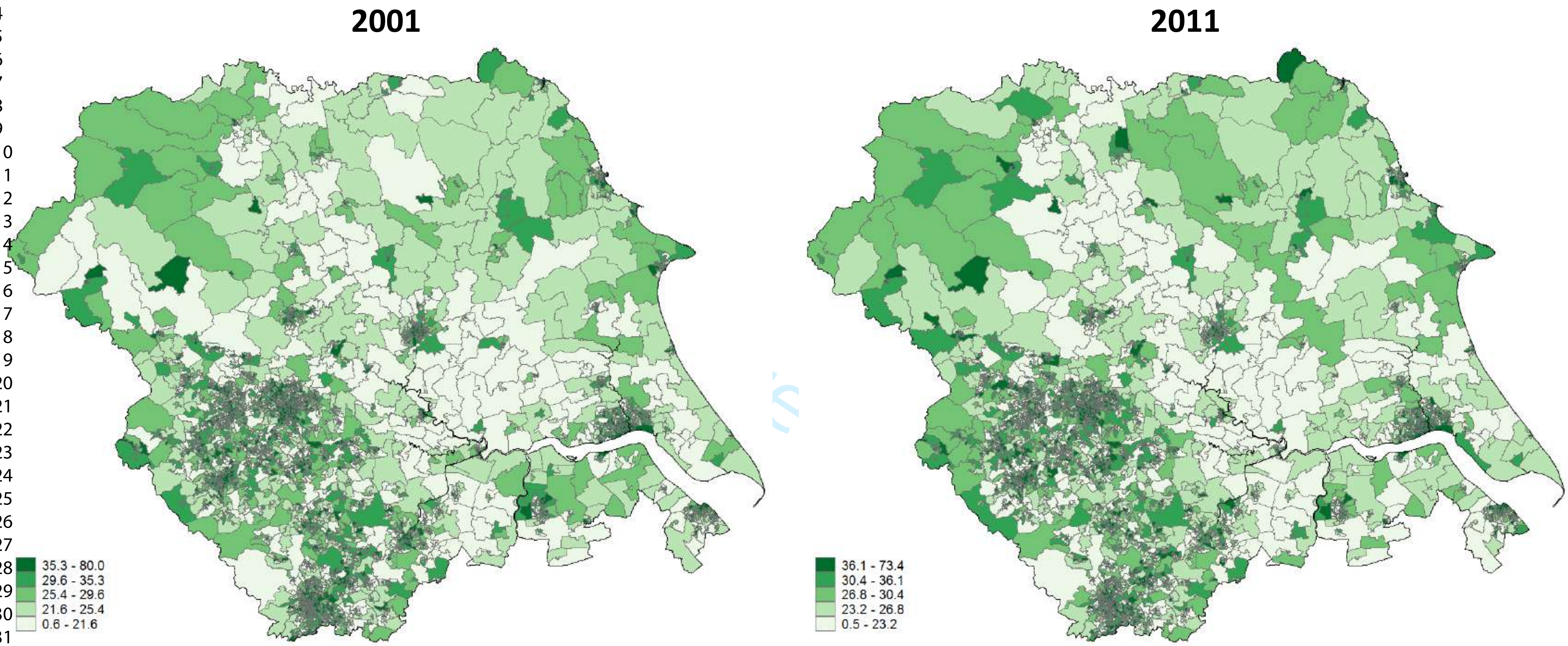

East Midlands, One Person Households % of all households

2001

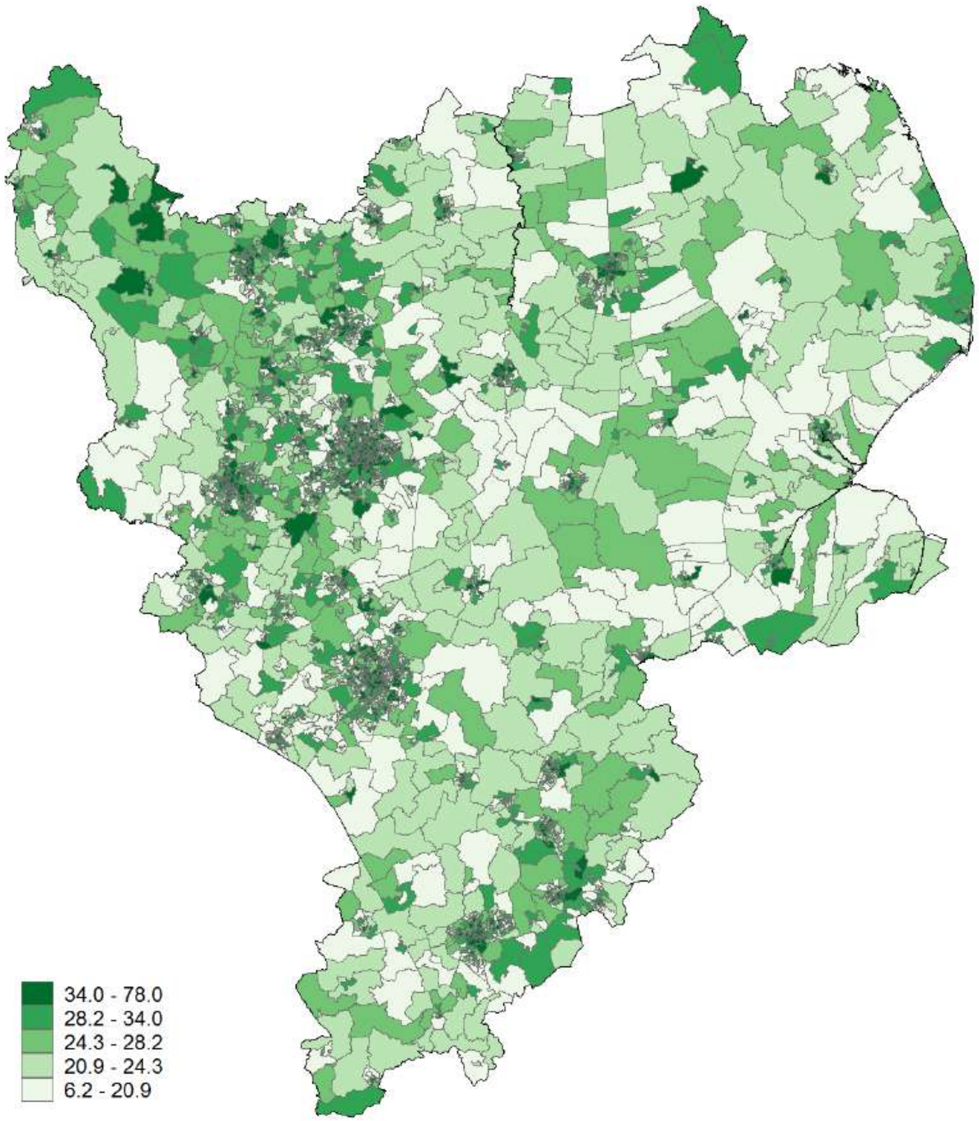

2011

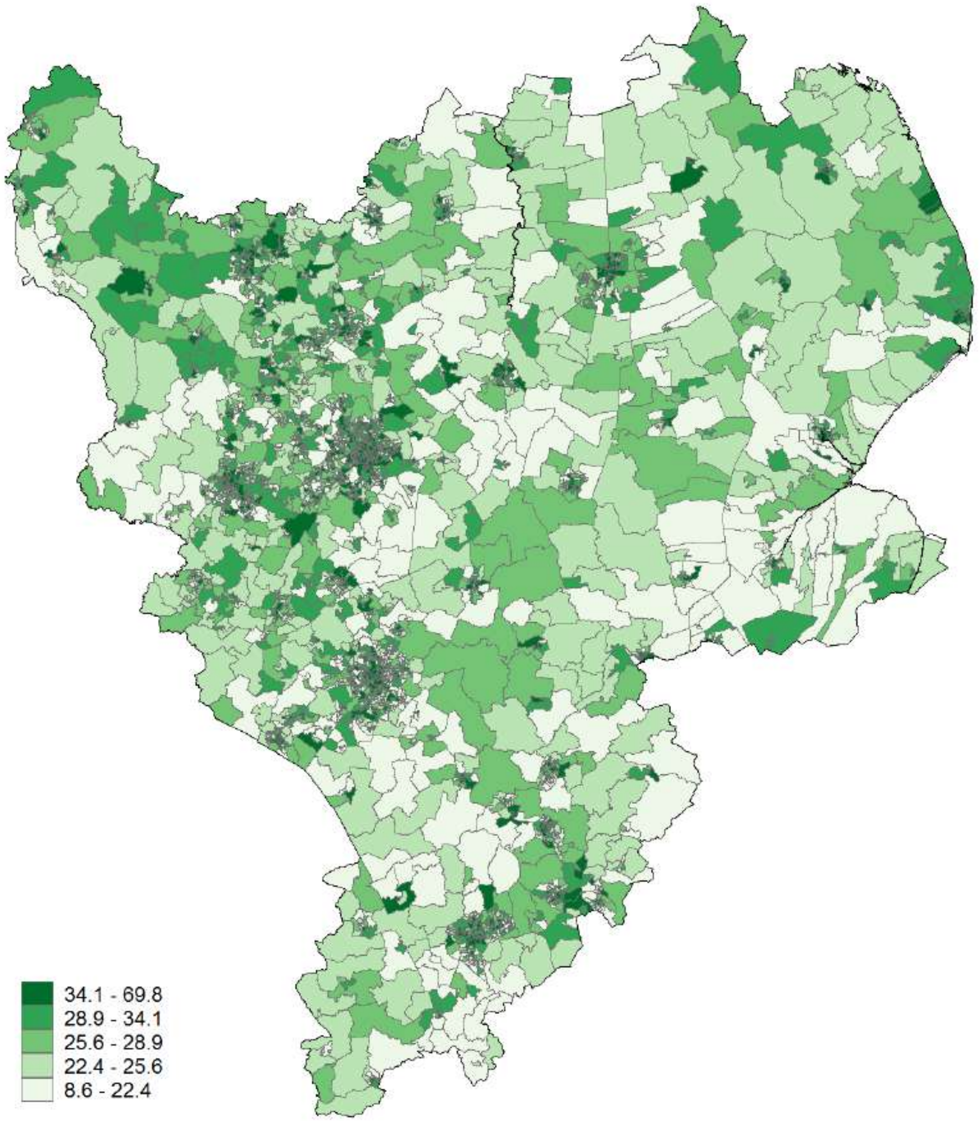

West Midlands, One Person Households % of all households

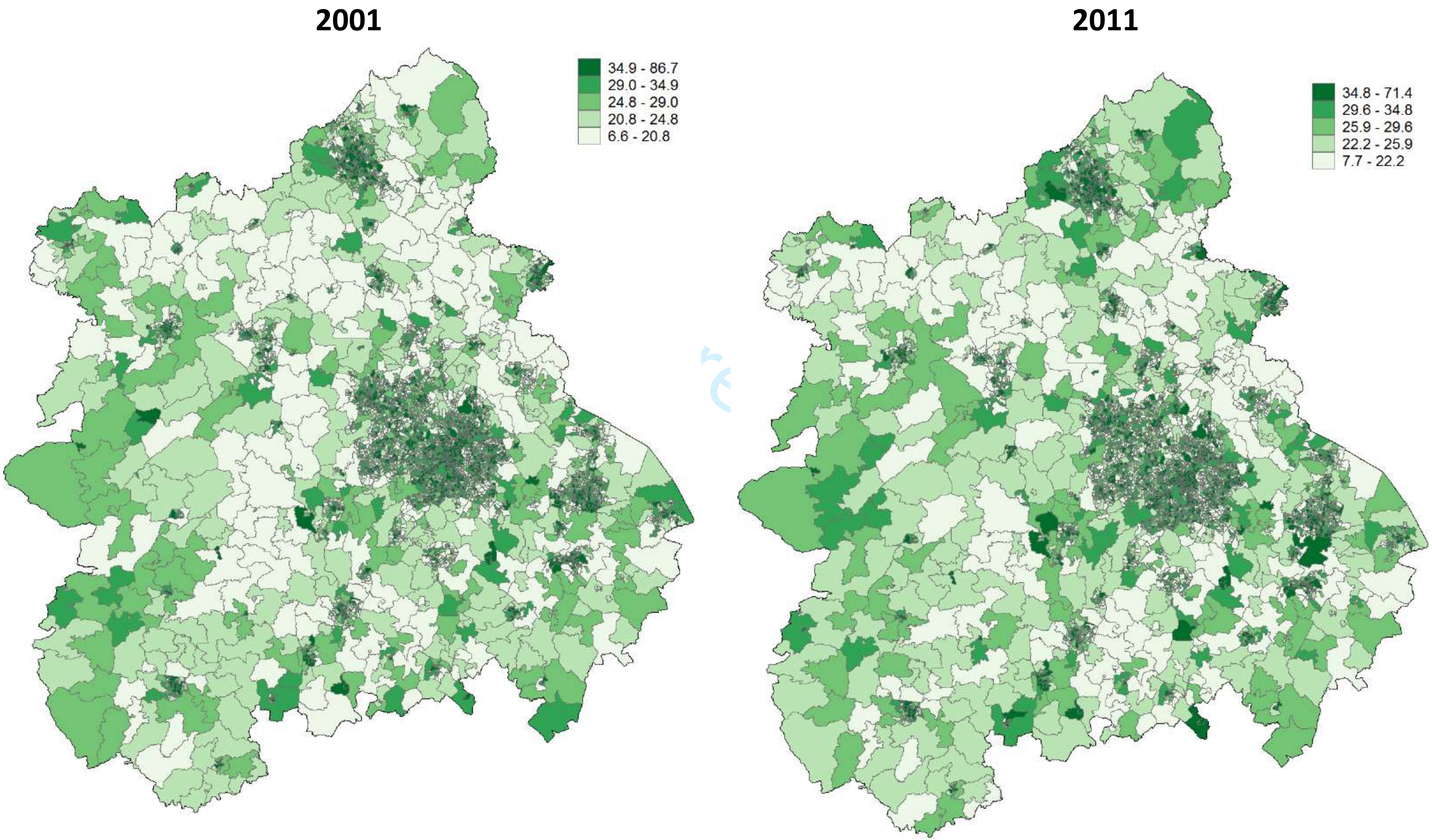

East of England, One Person Households % of all households

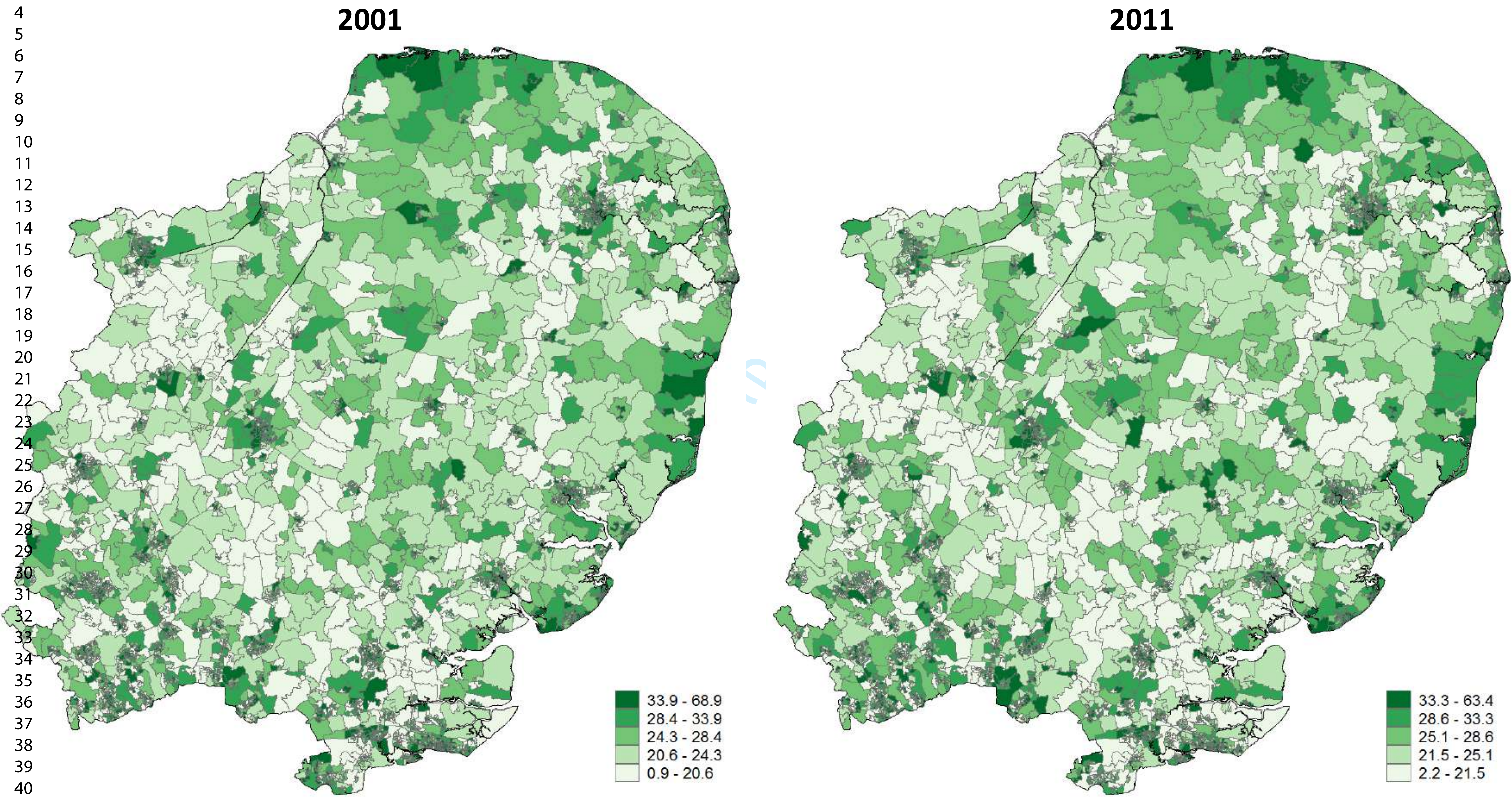

London, One Person Households % of all households

2001

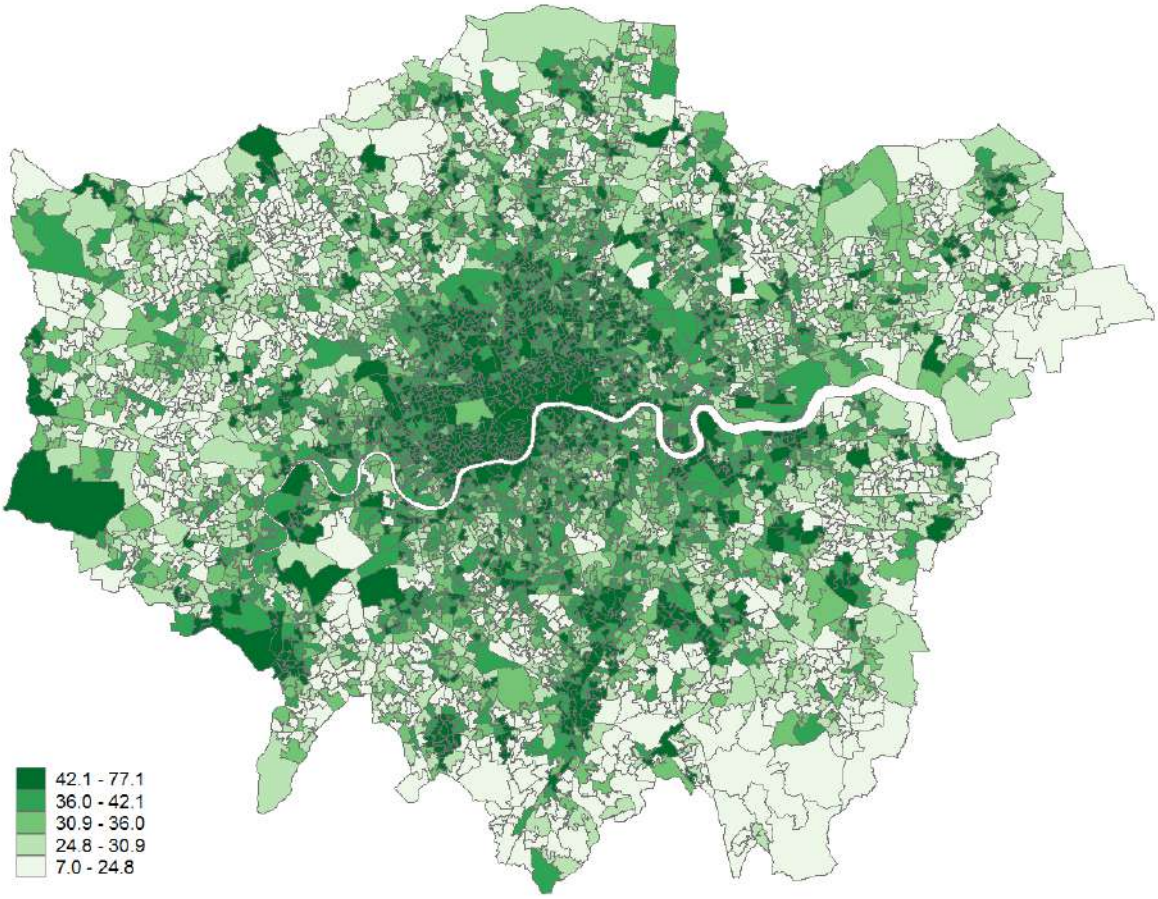

2011

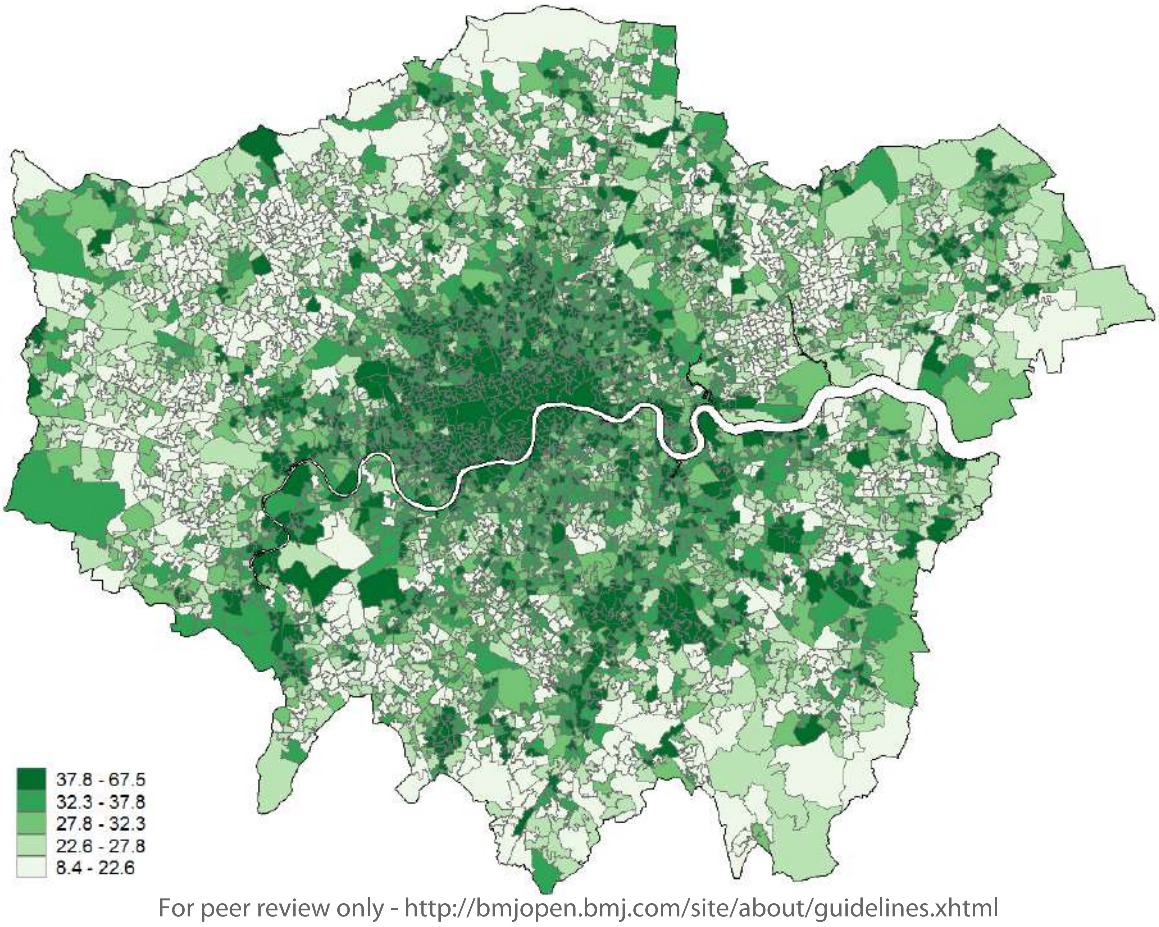

South East Coast, One Person Households % of all households

2001

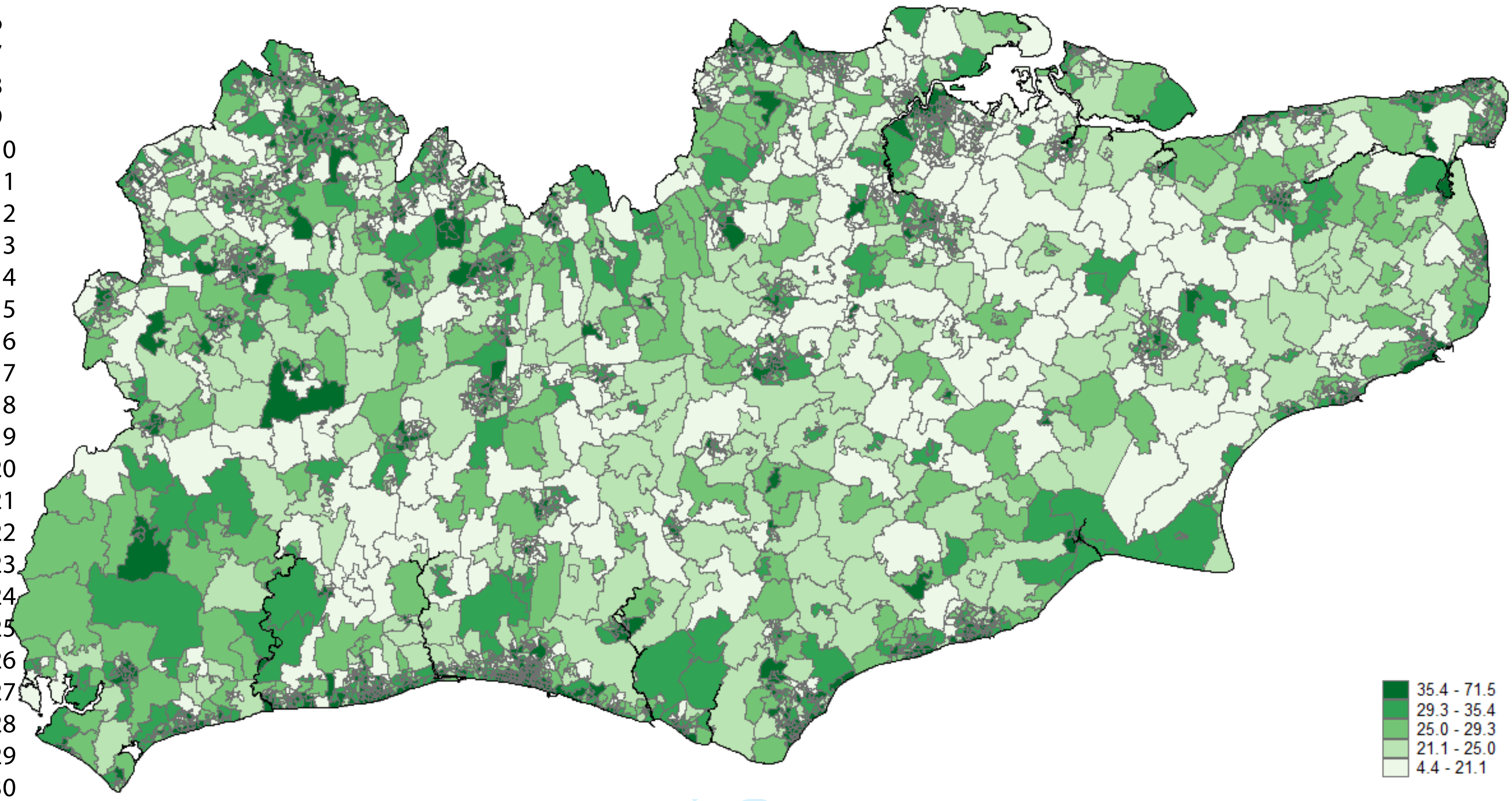

2011

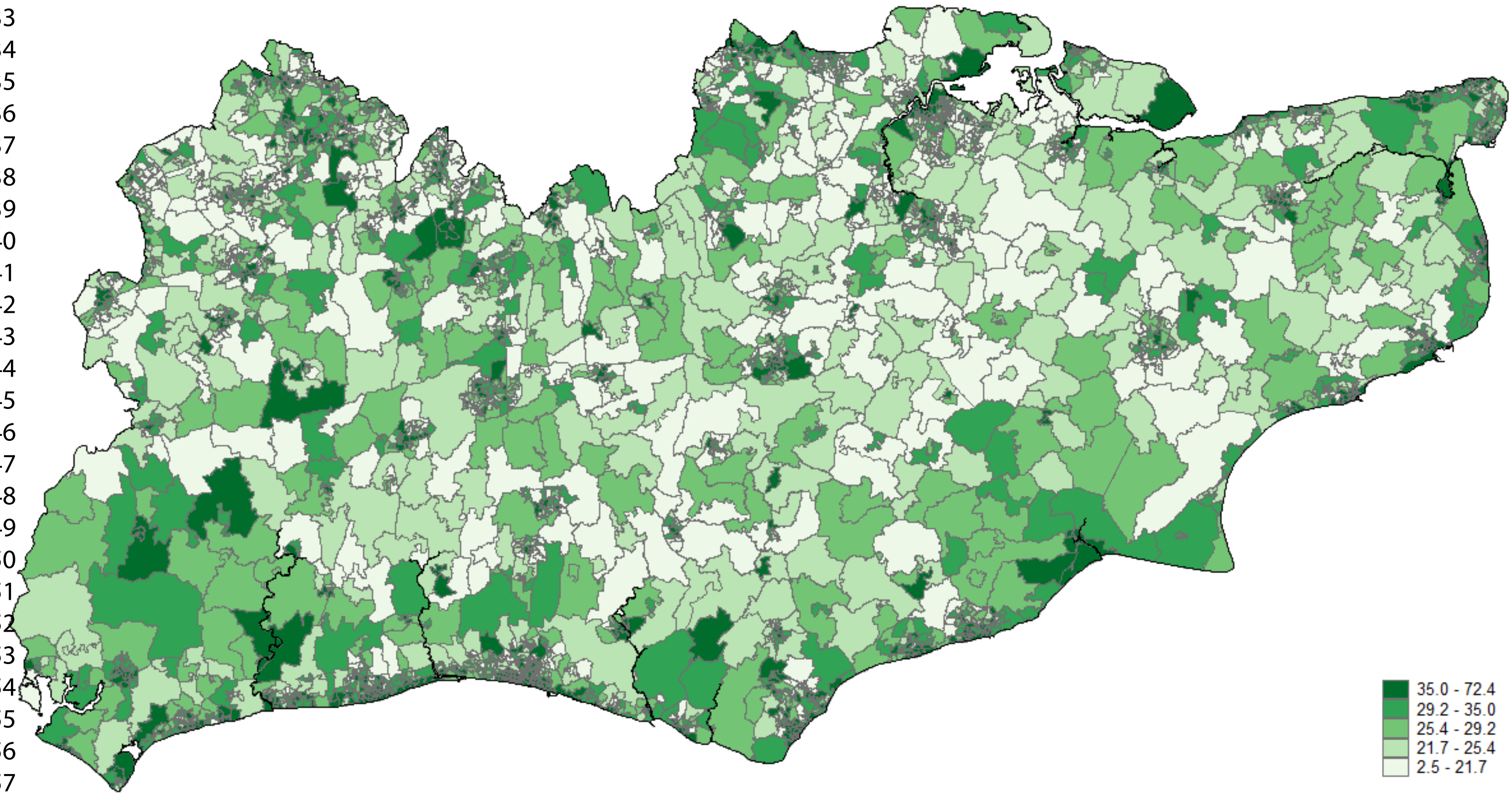

South Central, One Person Households % of all households

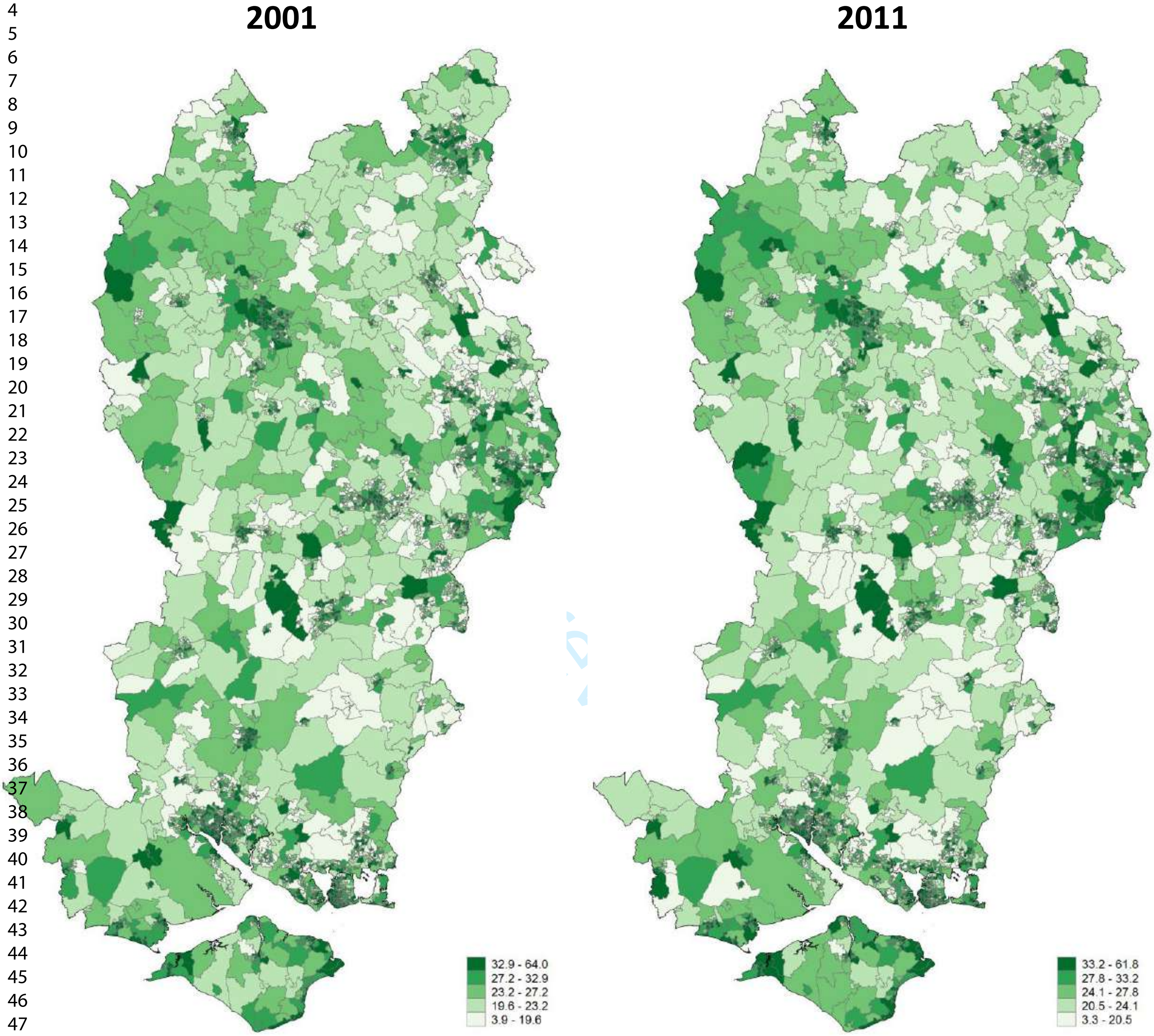

South West, One Person Households % of all households

2001

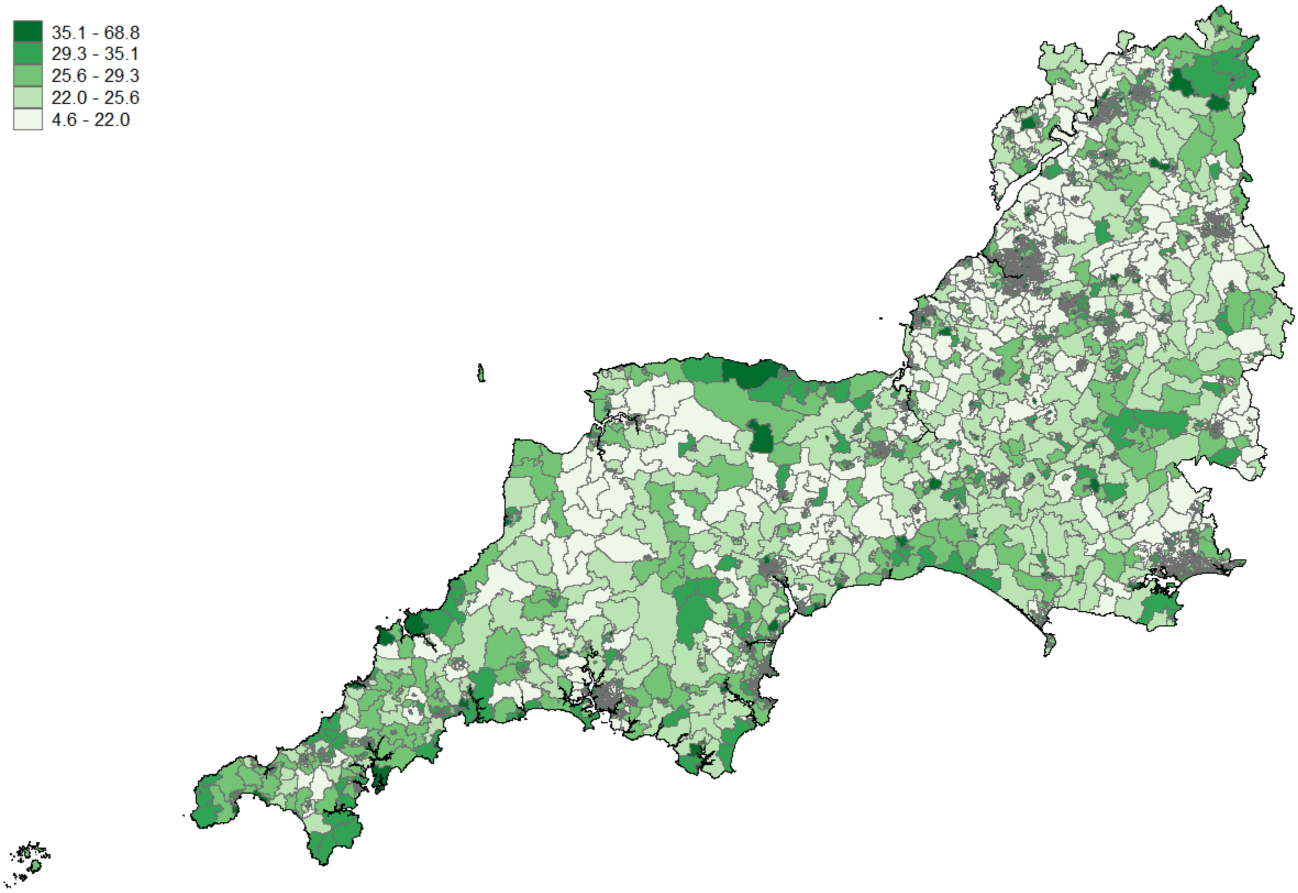

2011

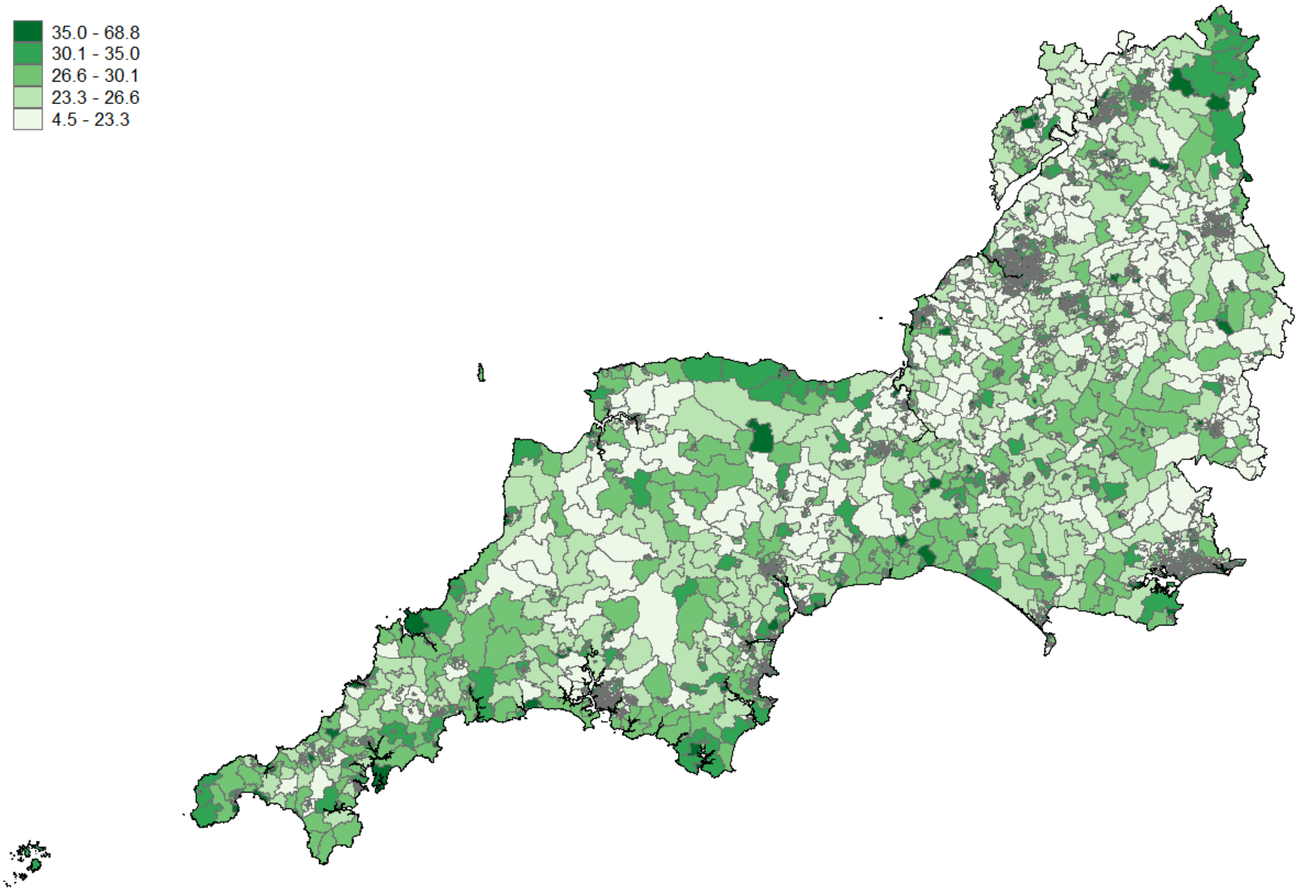

Private renting

England 2001, Private Renting % of all households

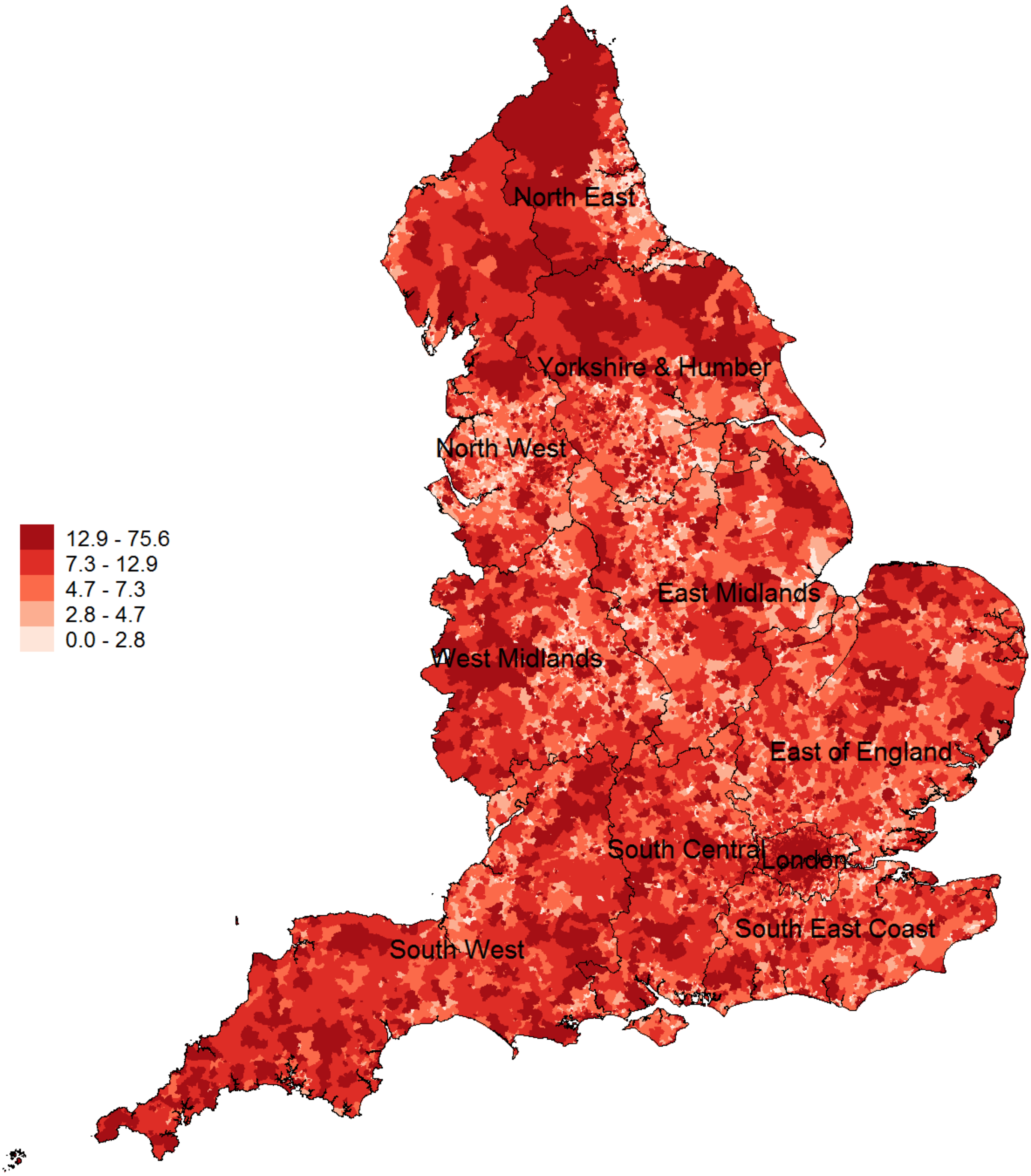

England 2011, Private Renting % of all households

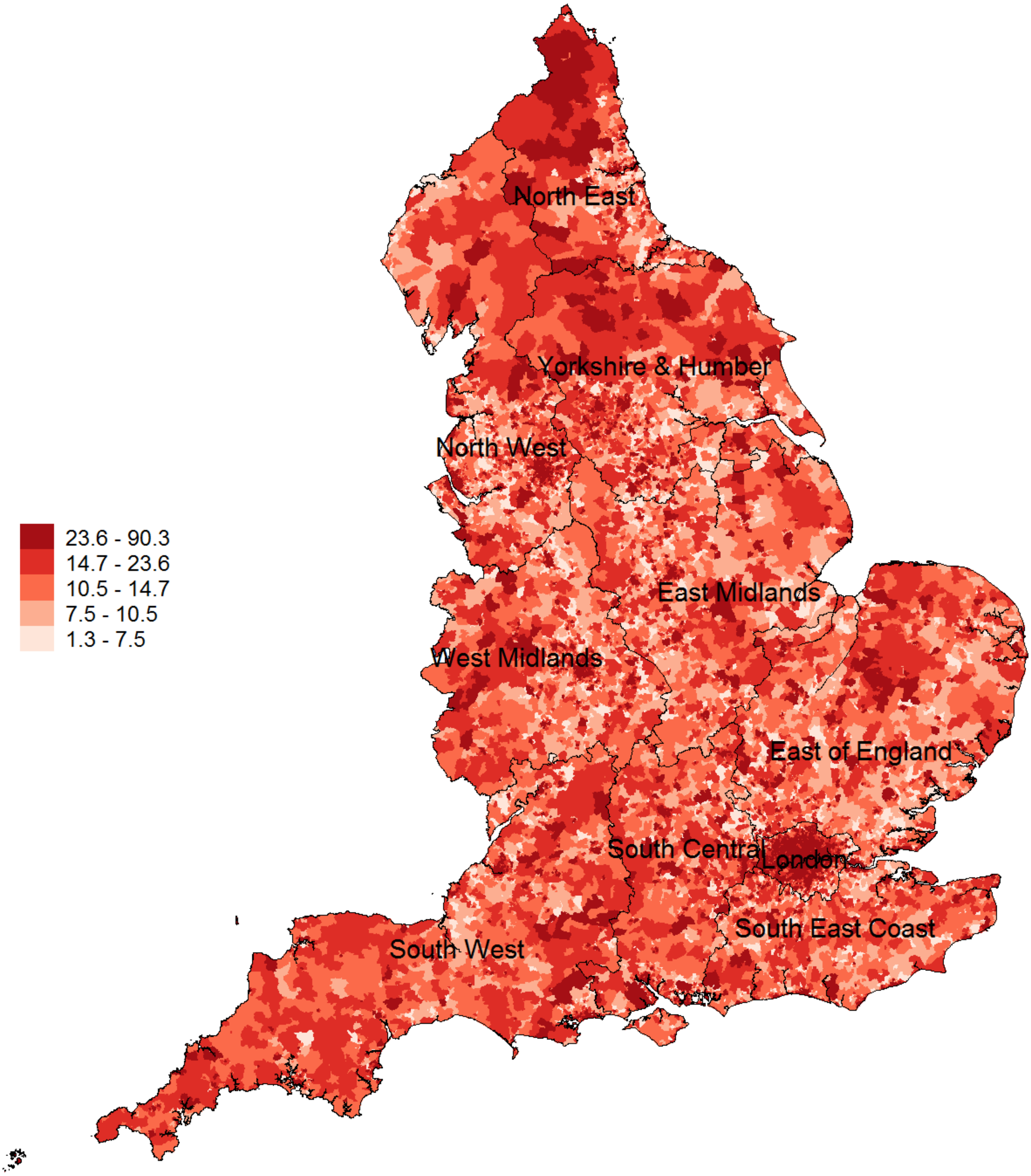

North East, Private Renting % of all households

2001

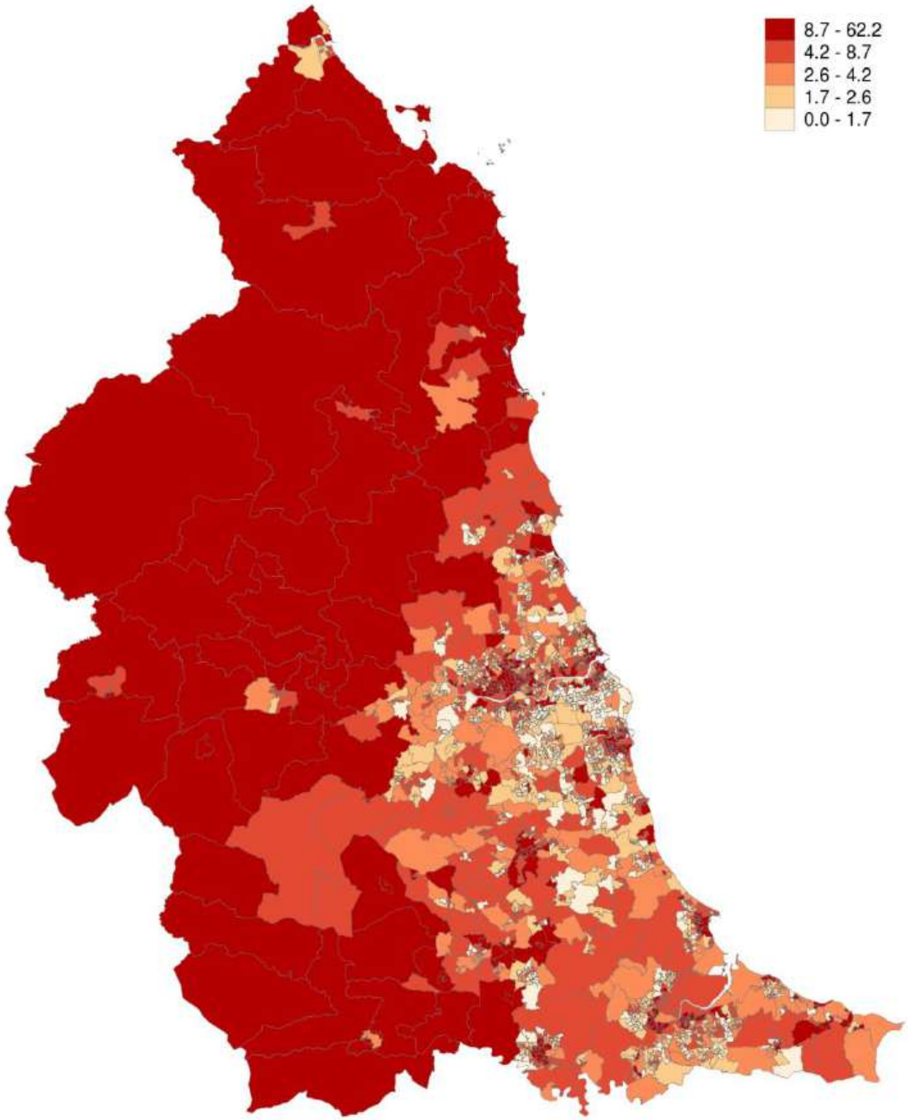

2011

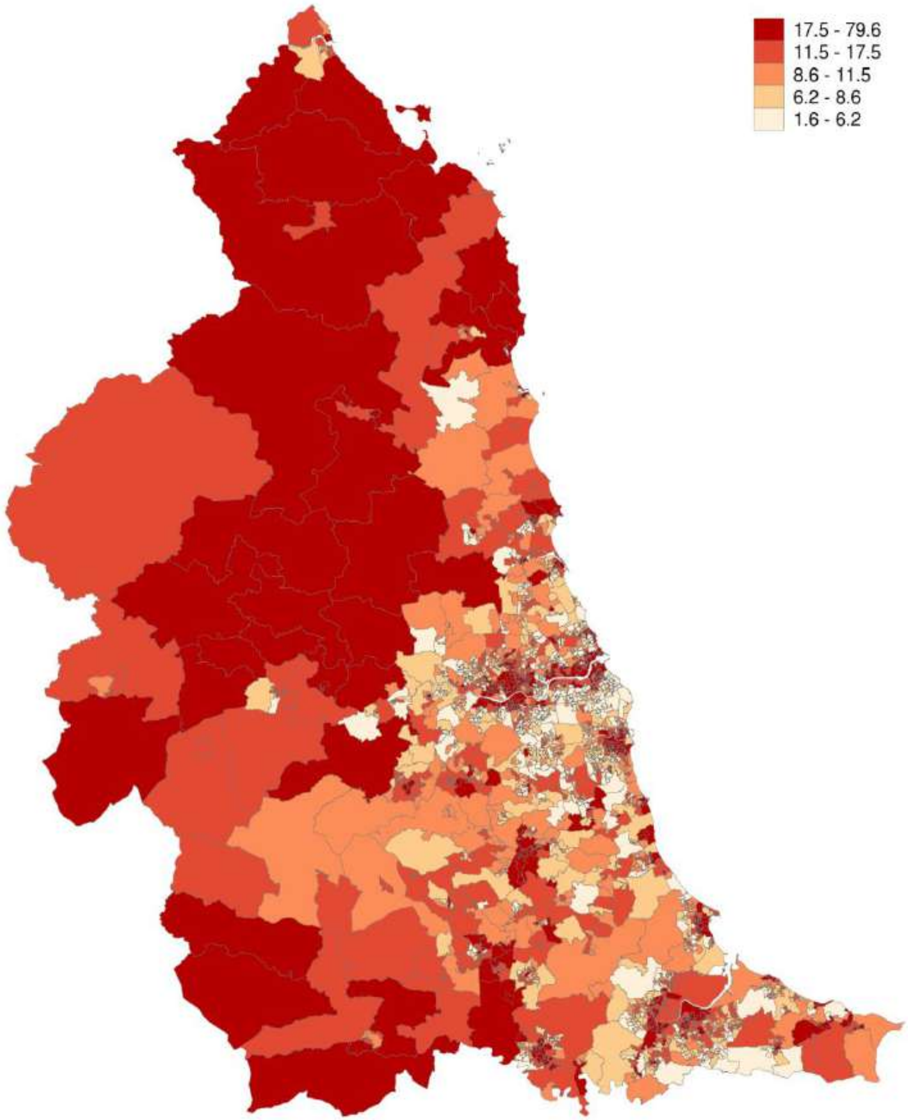

North West, Private Renting % of all households

2001

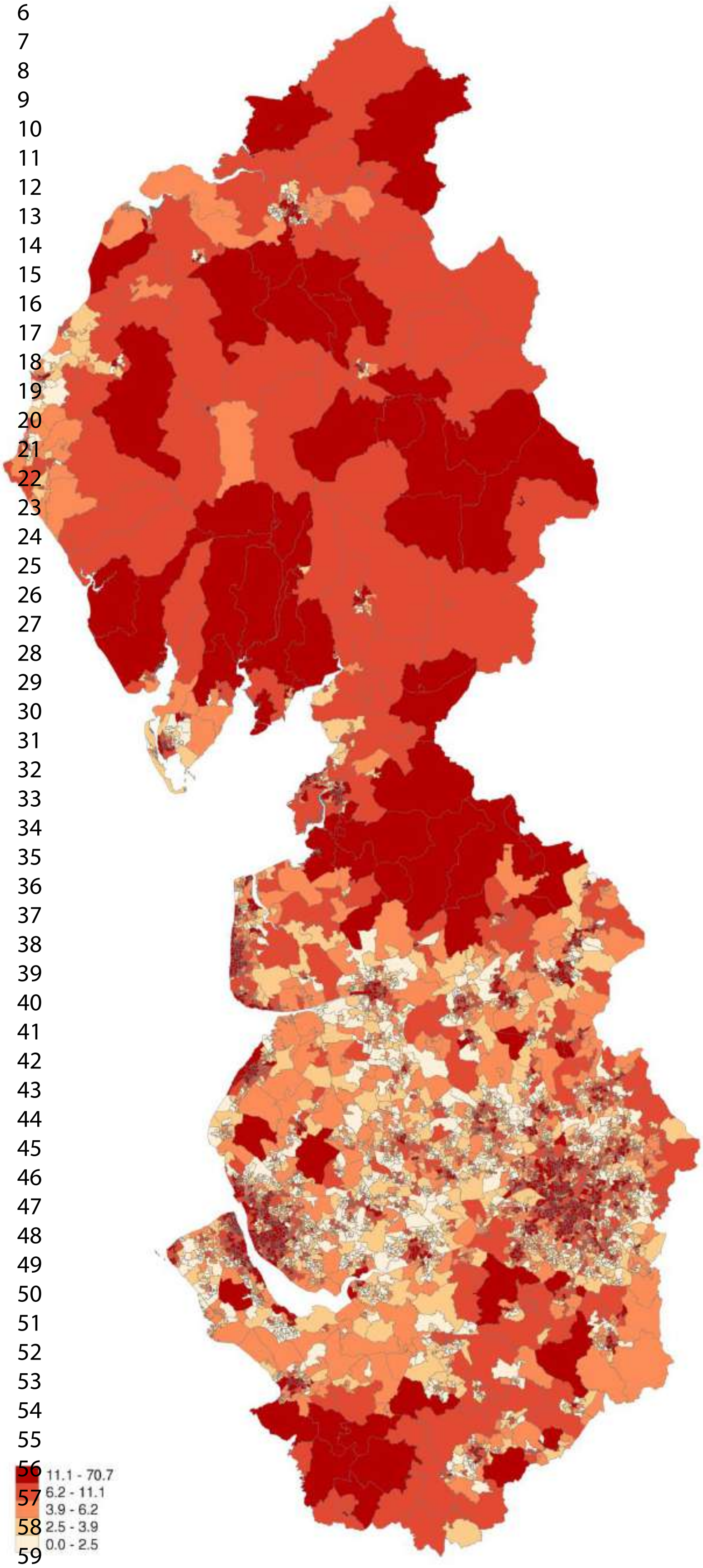

2011

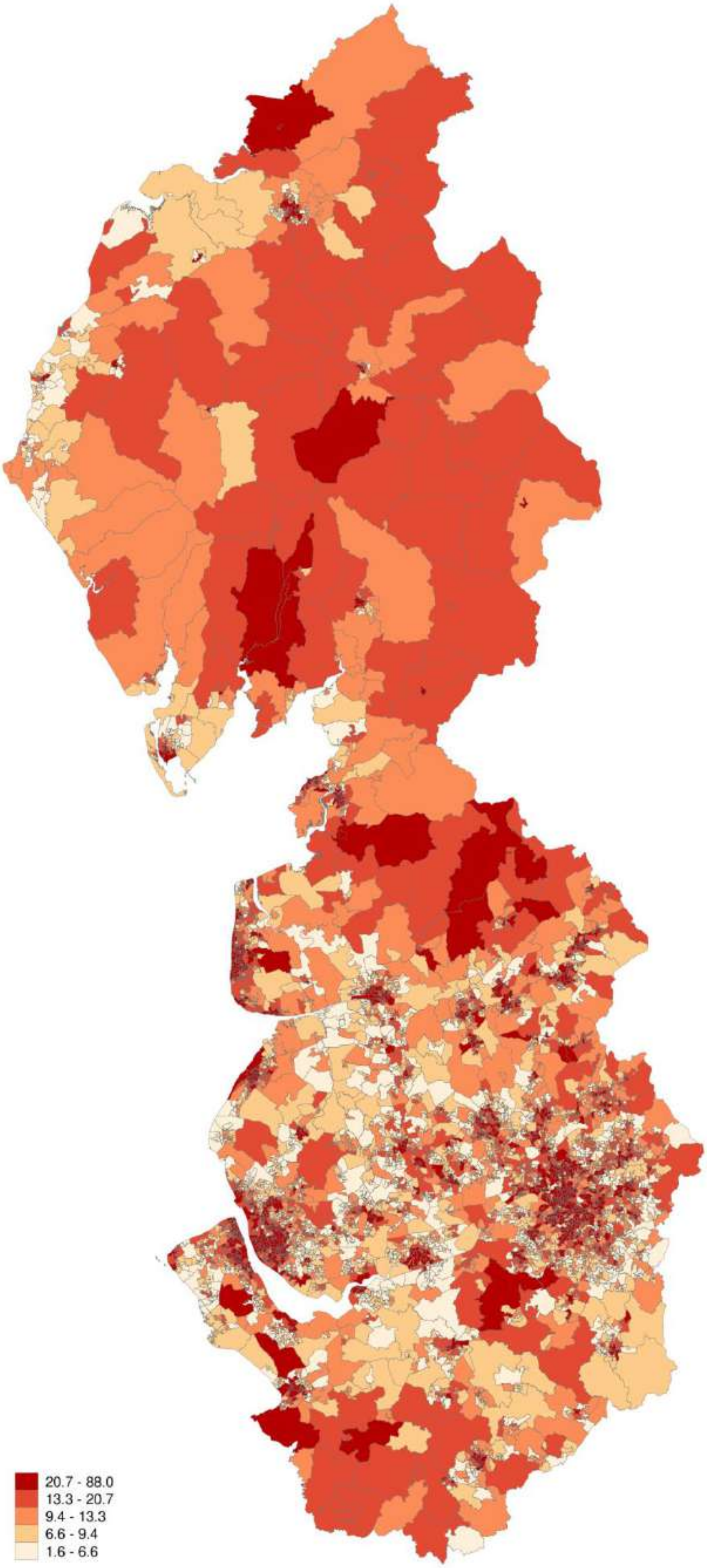

Yorkshire and the Humber, Private Renting % of all households

2001

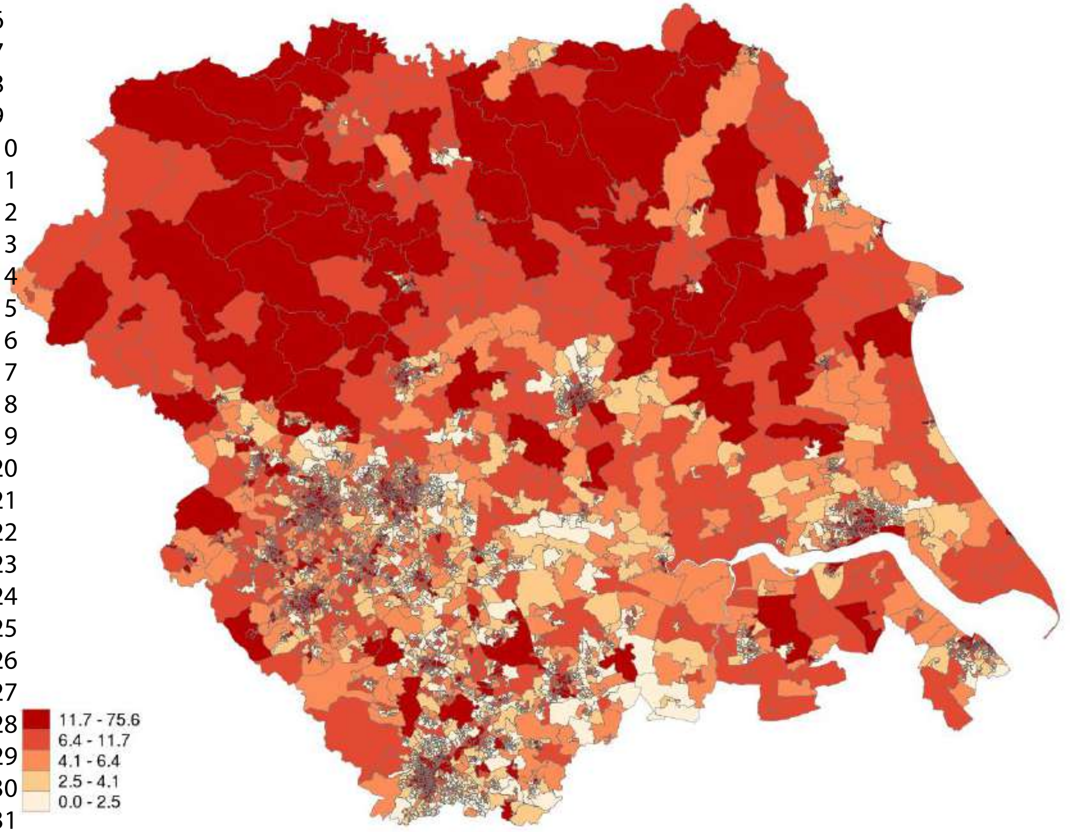

2011

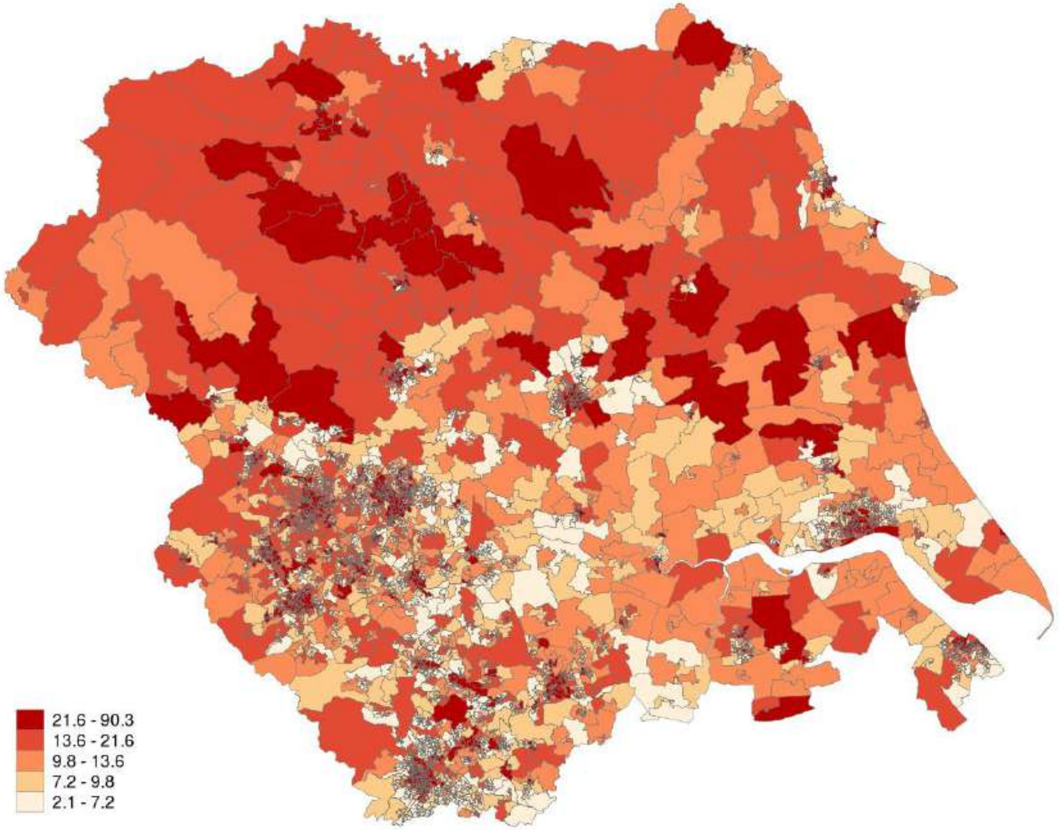

East Midlands, Private Renting % of all households

2001 2011

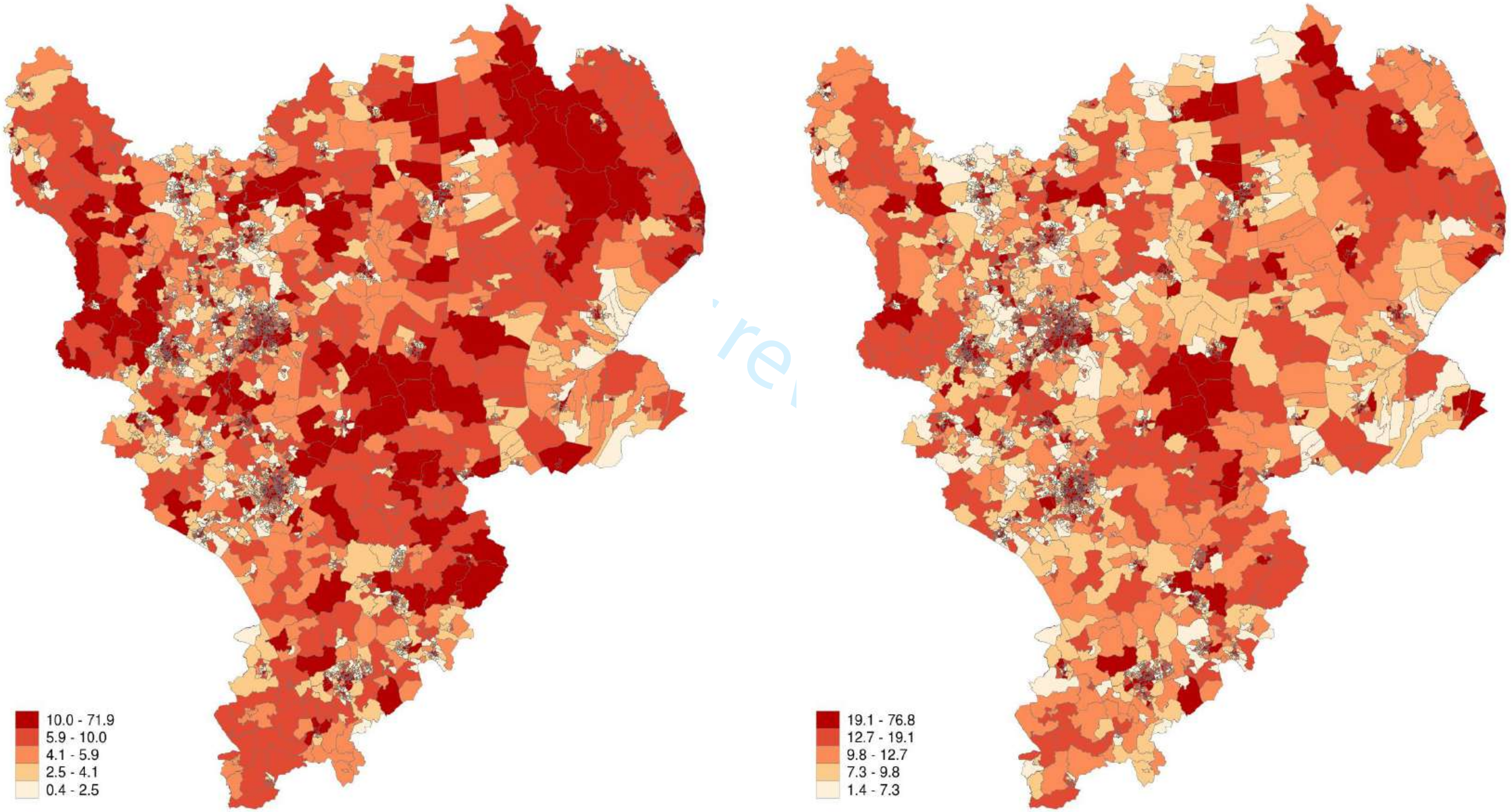

West Midlands, Private Renting % of all households

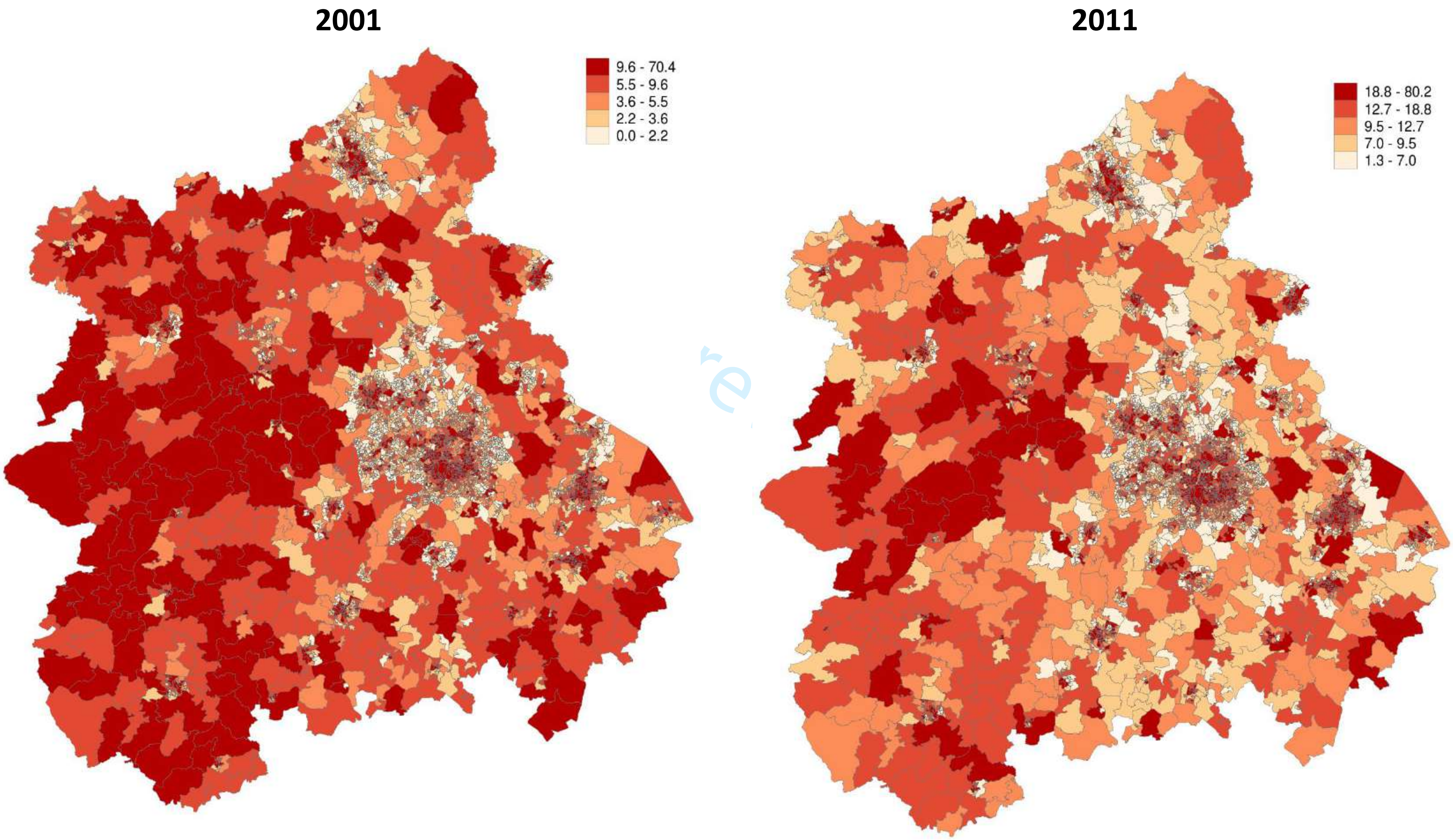

East of England, Private Renting % of all households

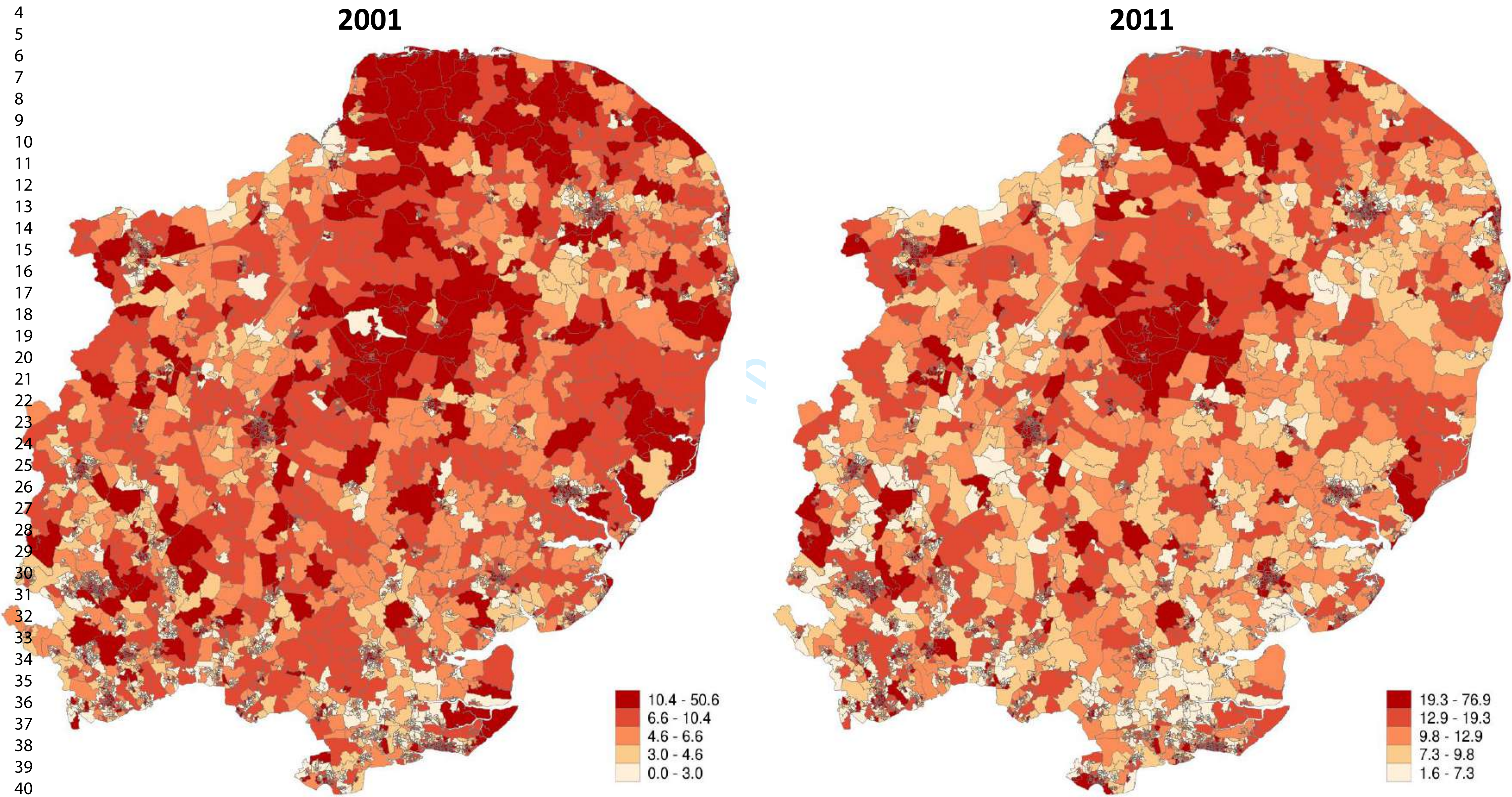

London, Private Renting % of all households

2001

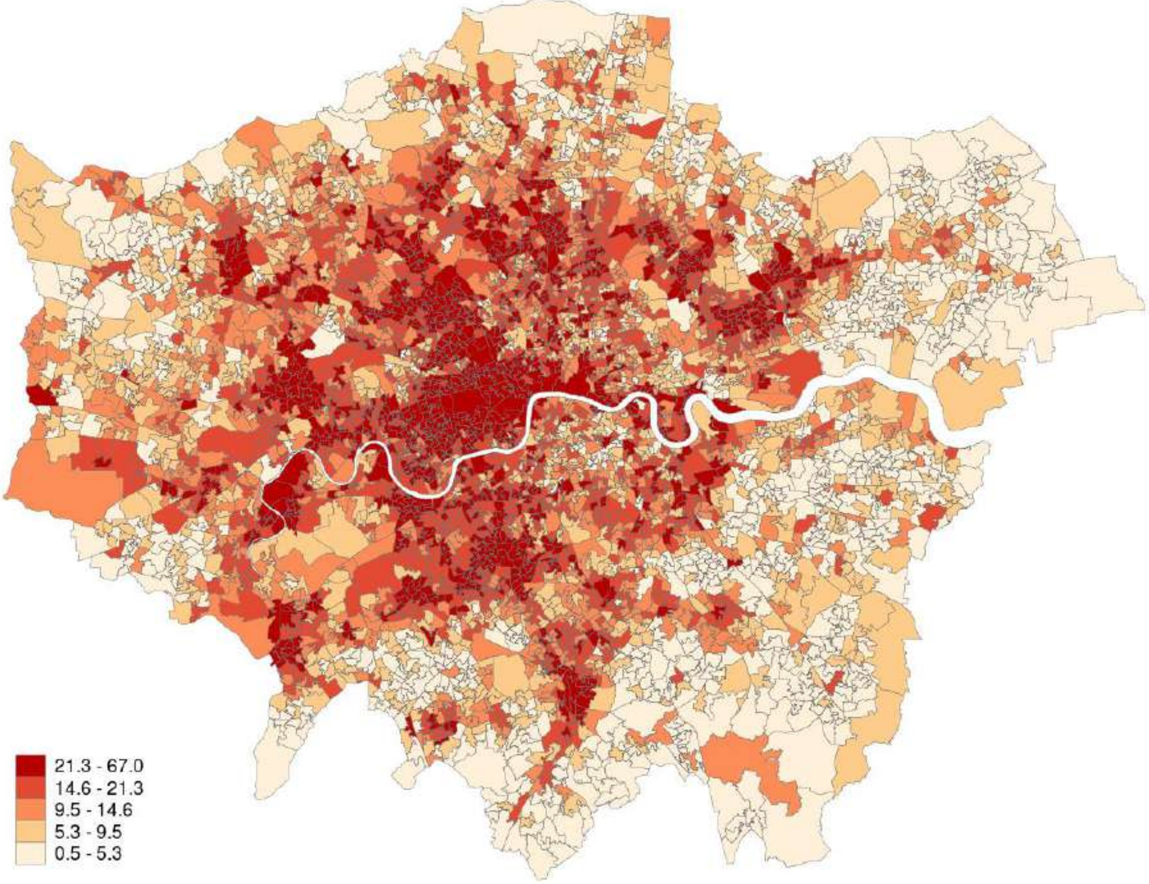

2011

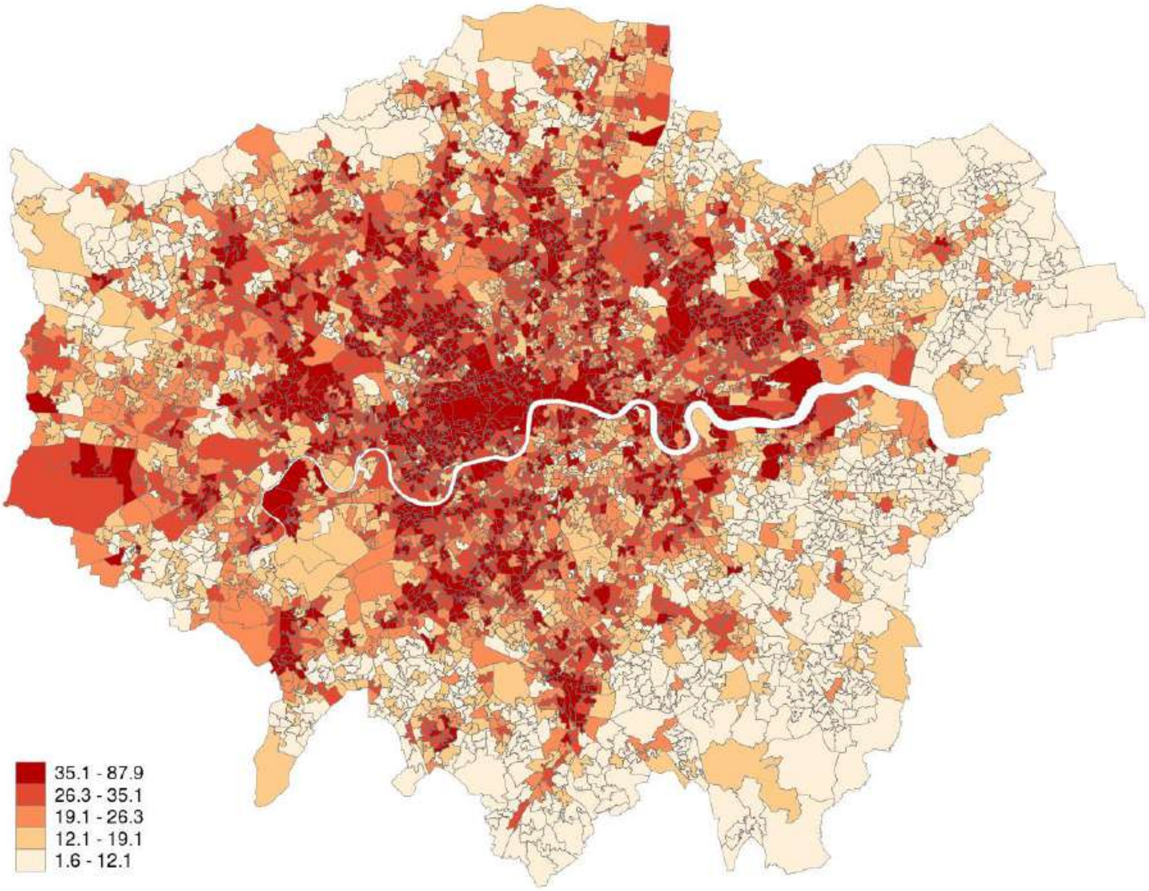

South East Coast, Private Renting % of all households

2001

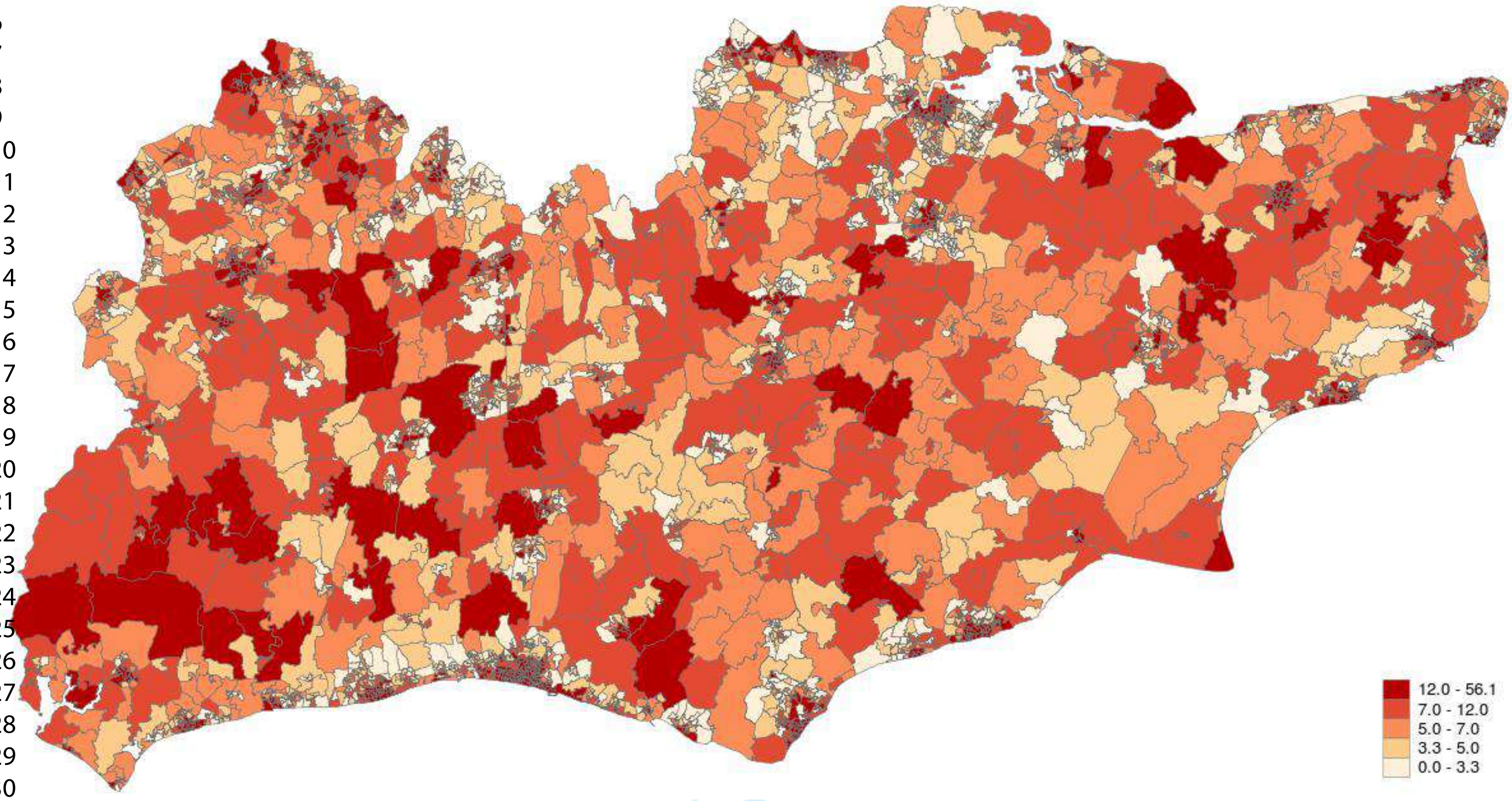

2011

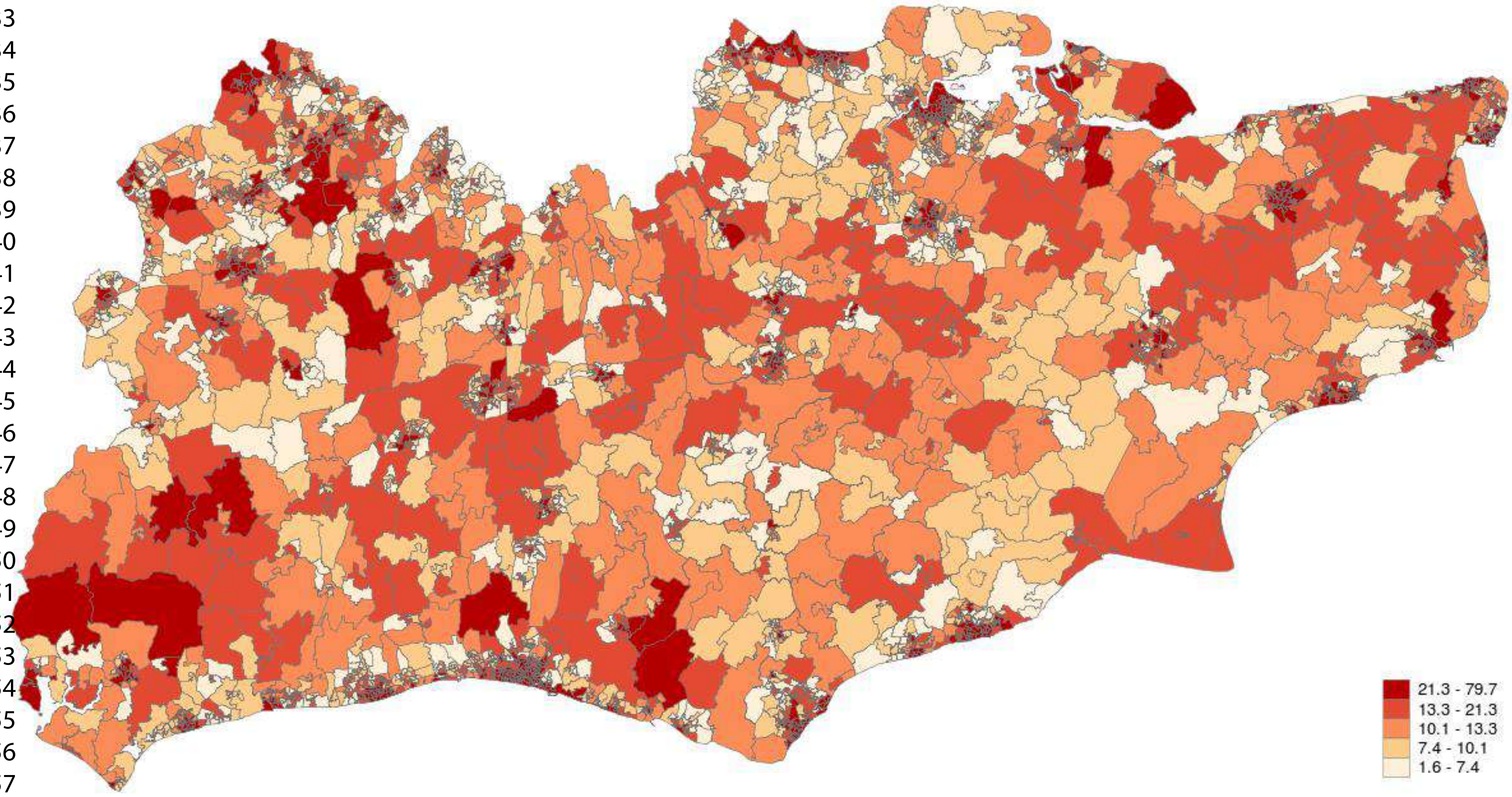

South Central, Private Renting % of all households

2001

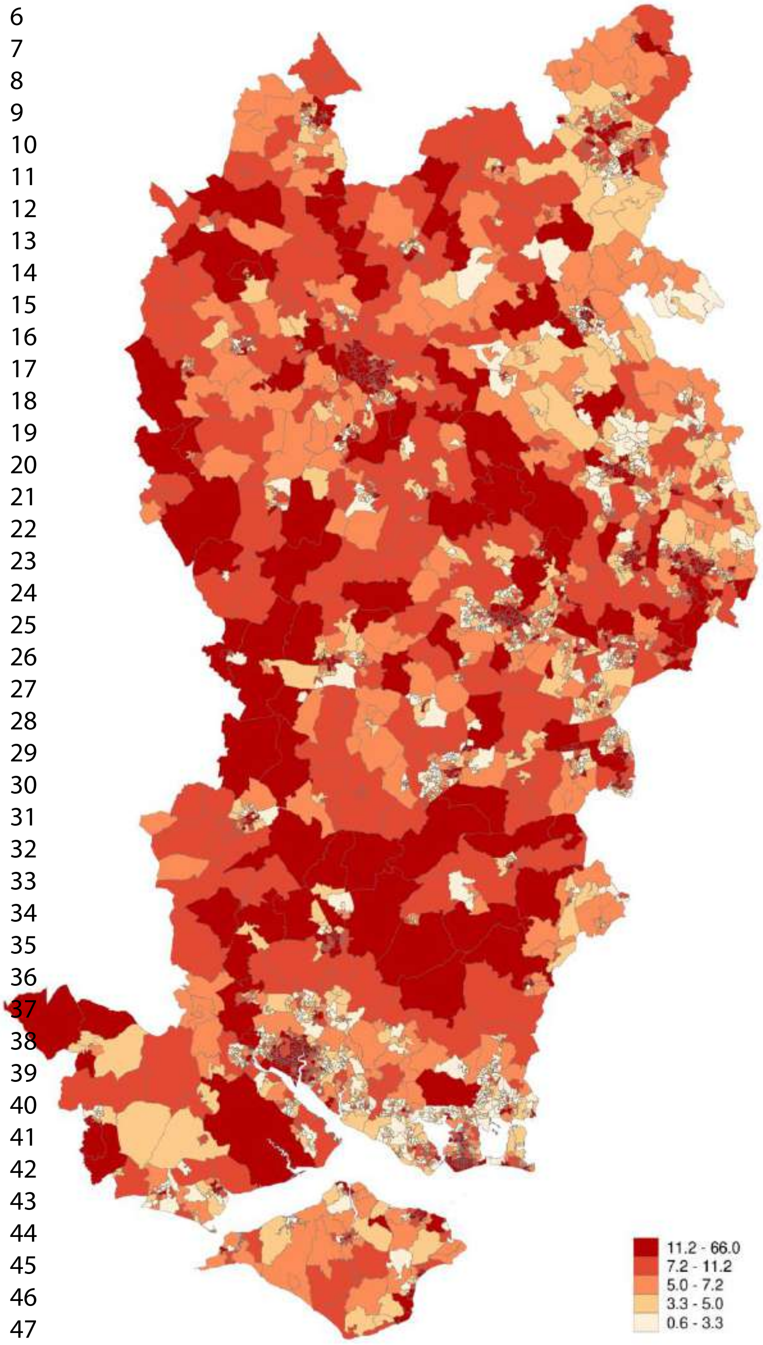

2011

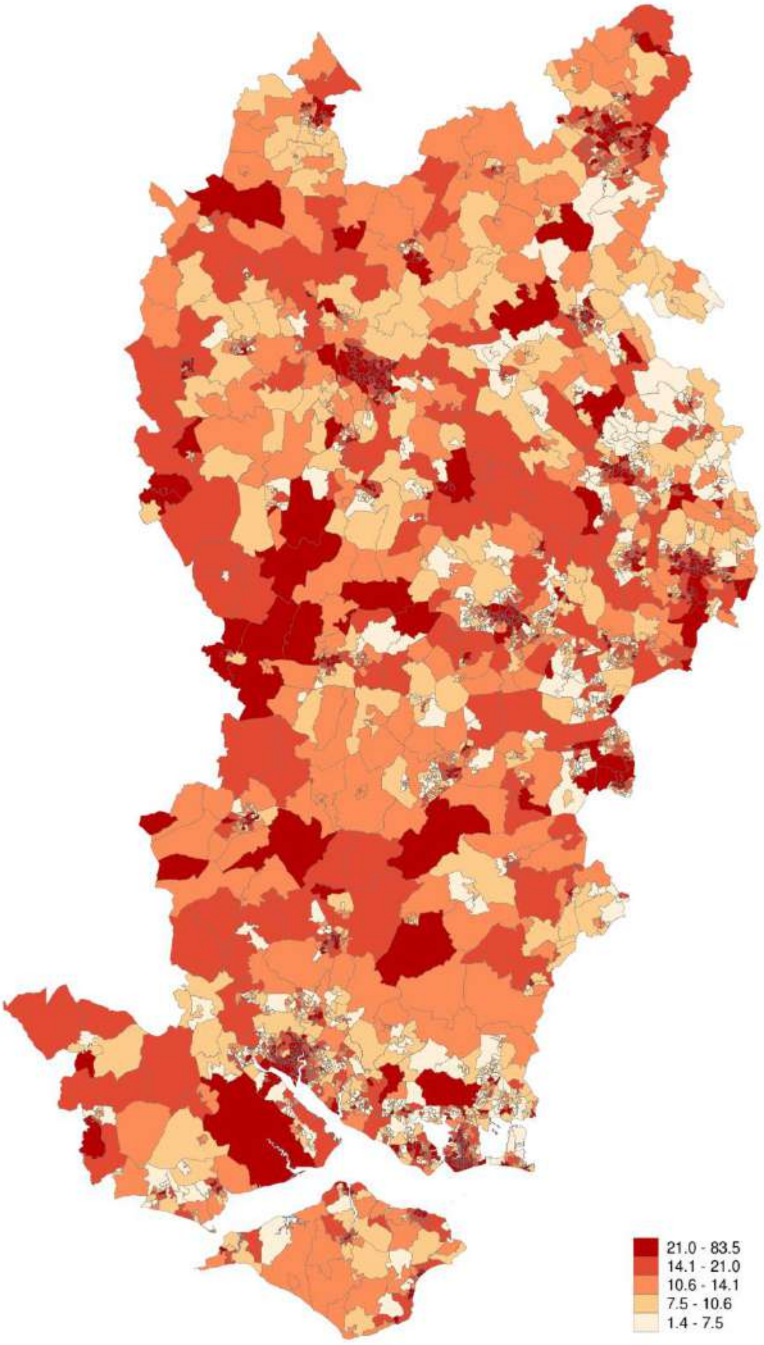

South West, Private Renting % of all households

2001

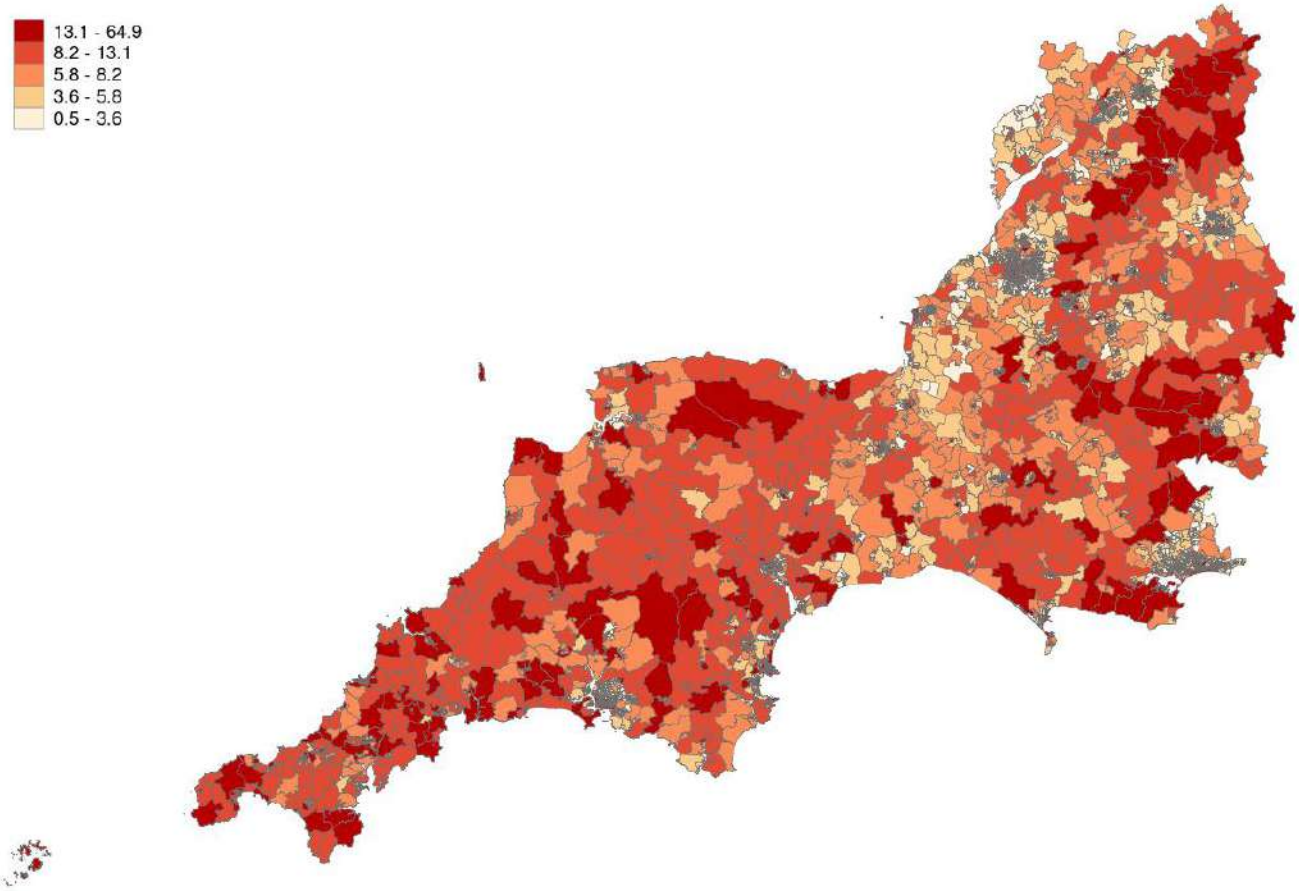

2011

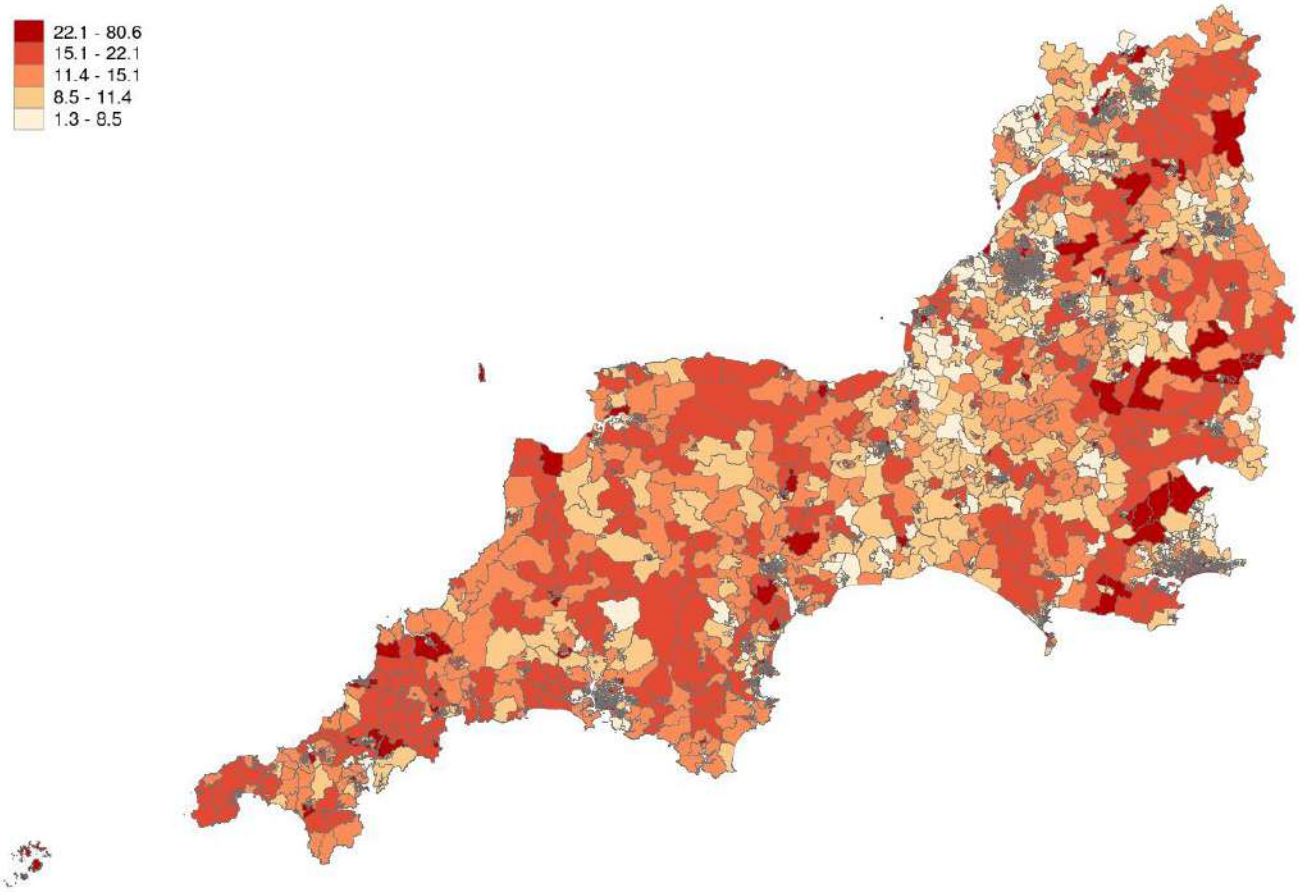

Population Turnover

England 2001, Population Turnover % of population

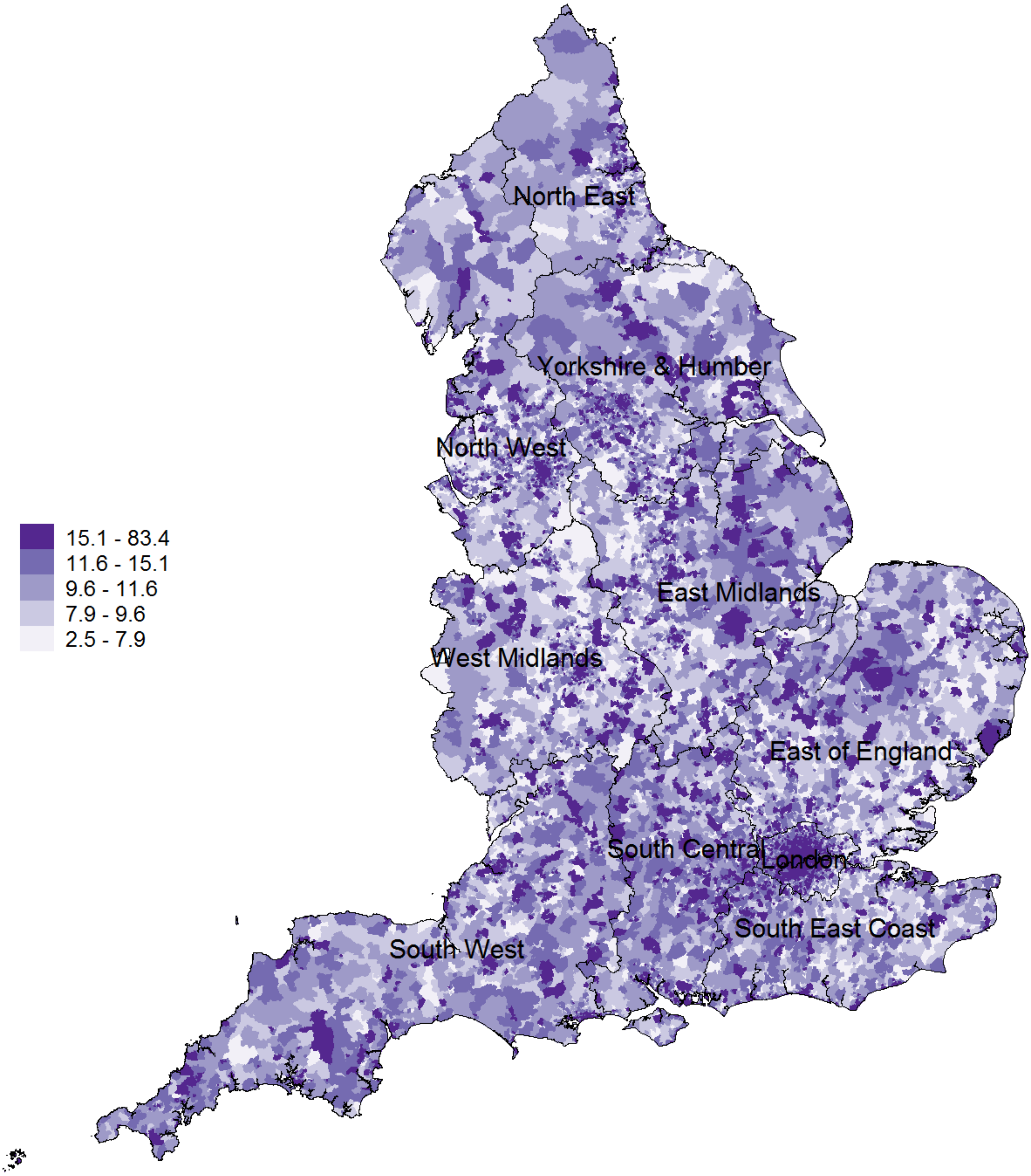

England 2011, Population Turnover % of population

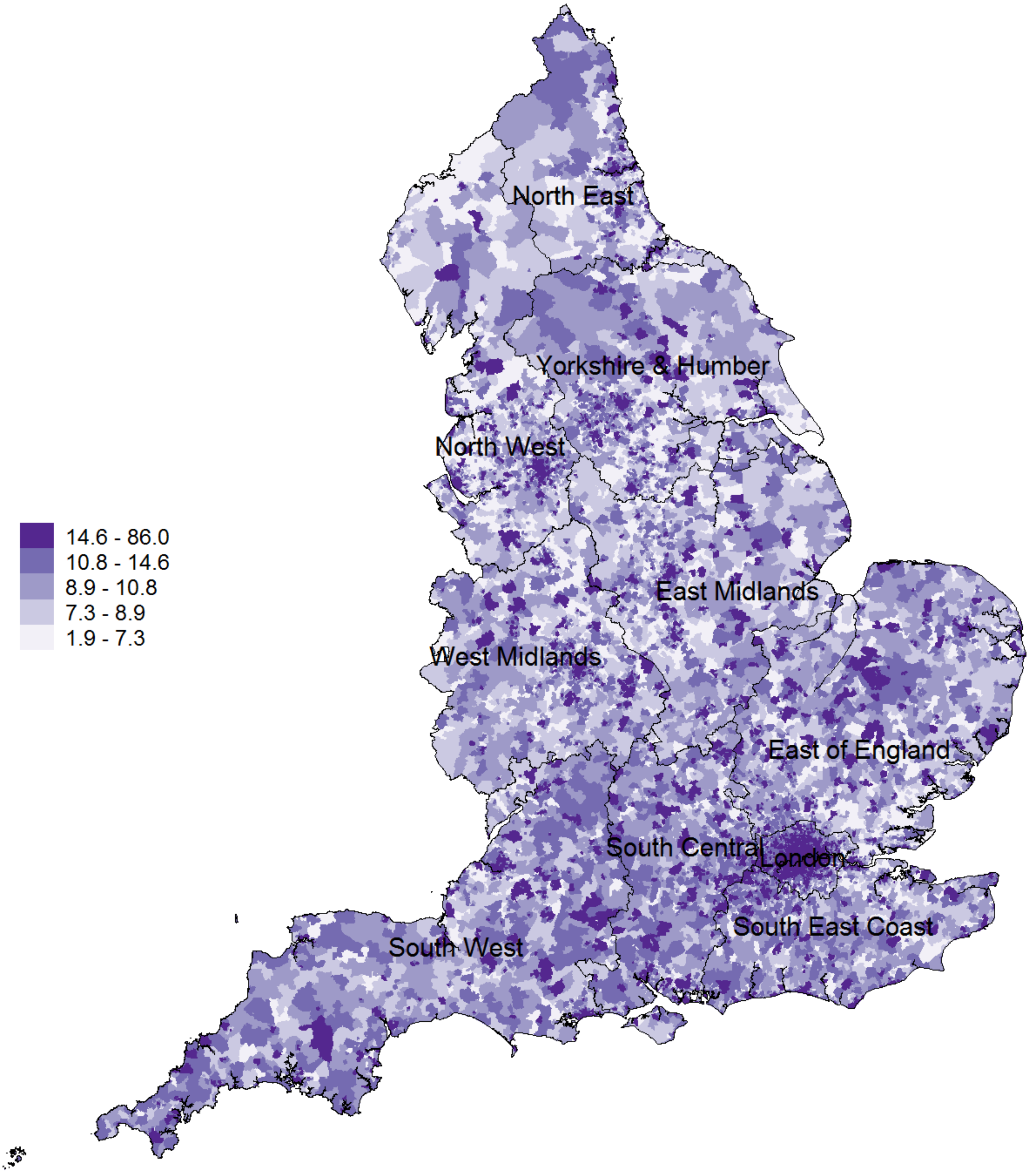

North East, Population Turnover % of population

2001

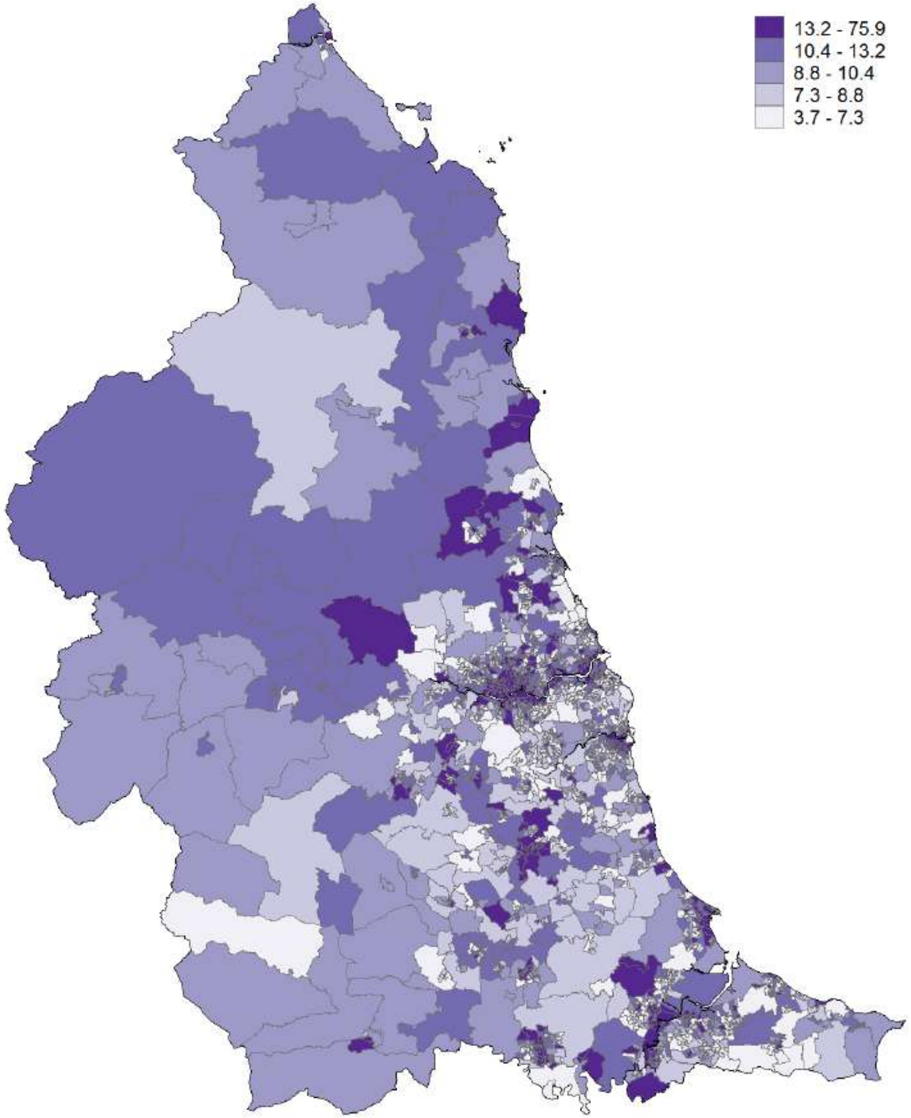

2011

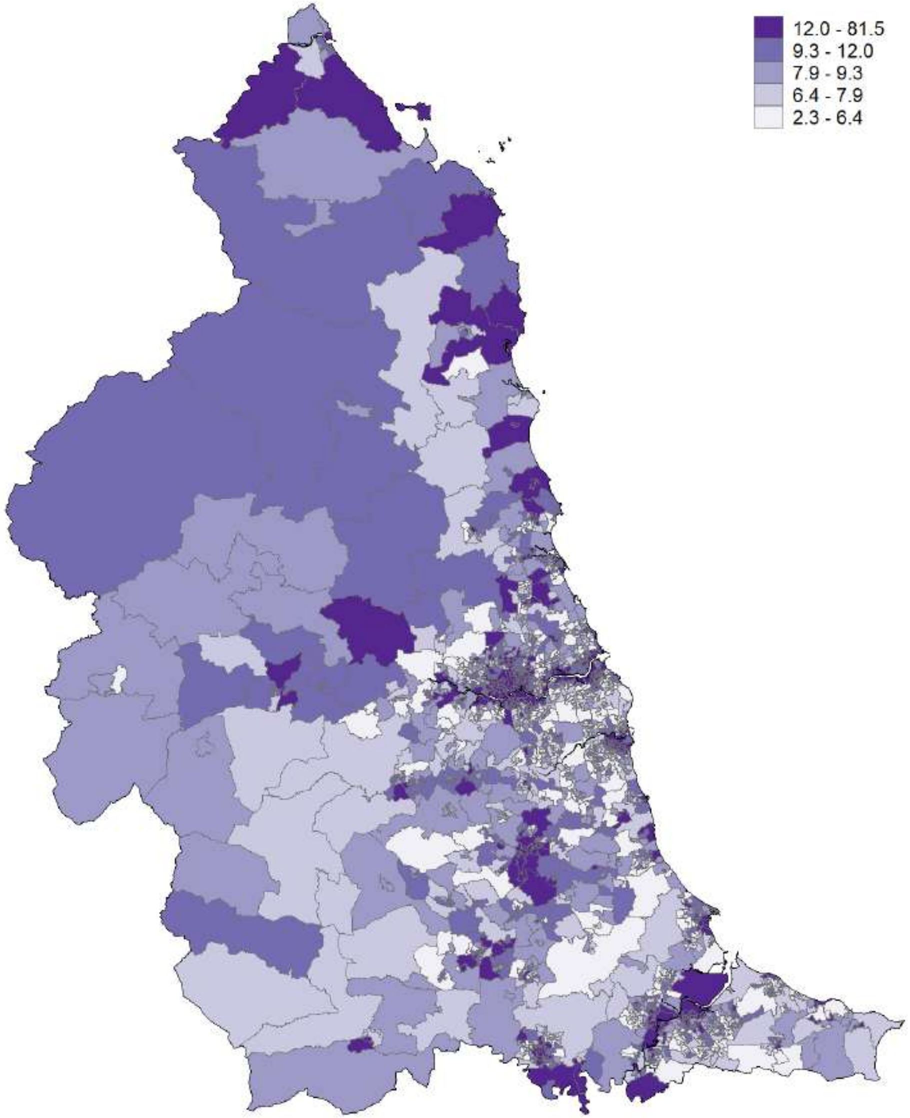

North West, Population Turnover % of population

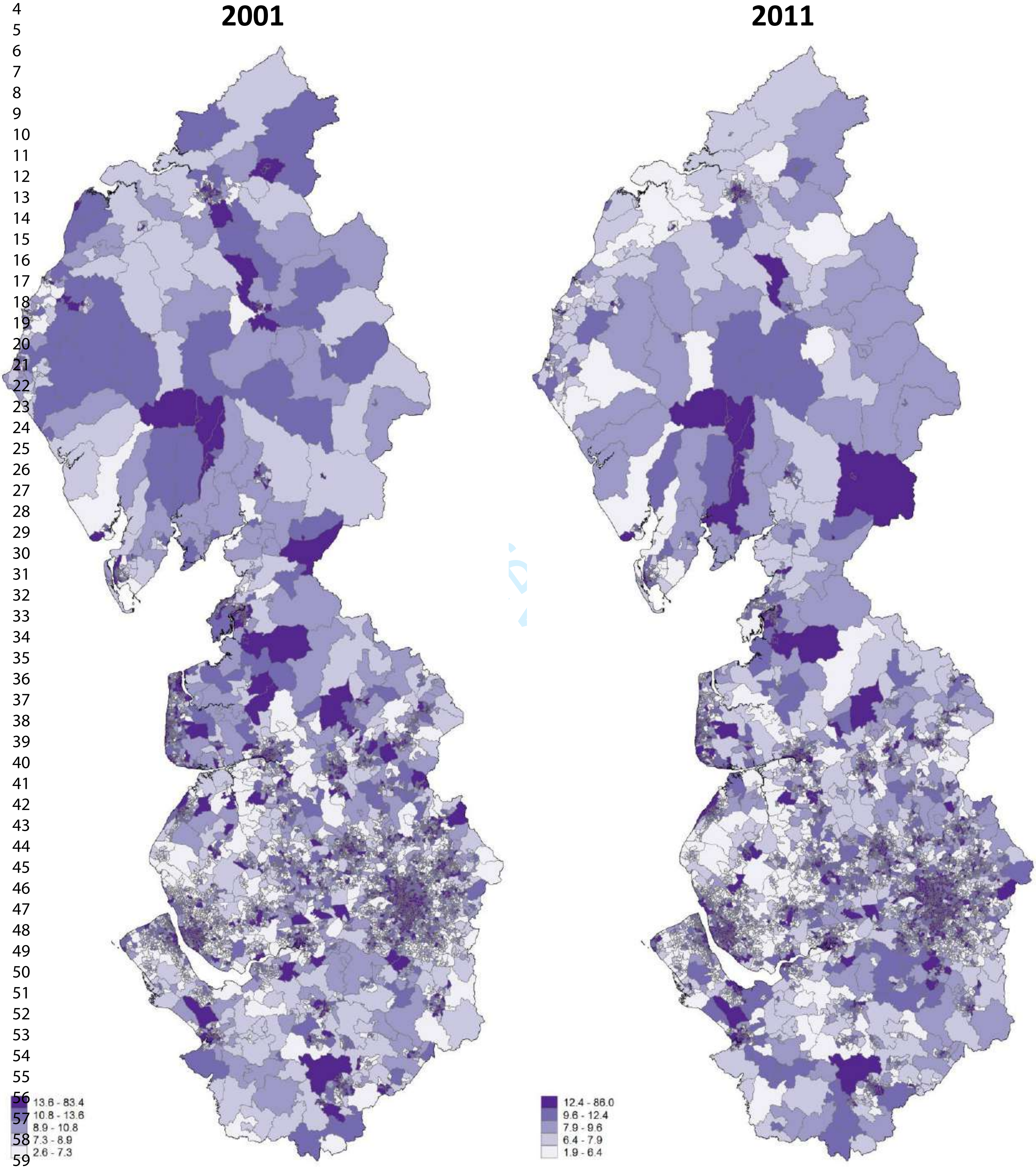

Yorkshire and the Humber, Population Turnover % of population

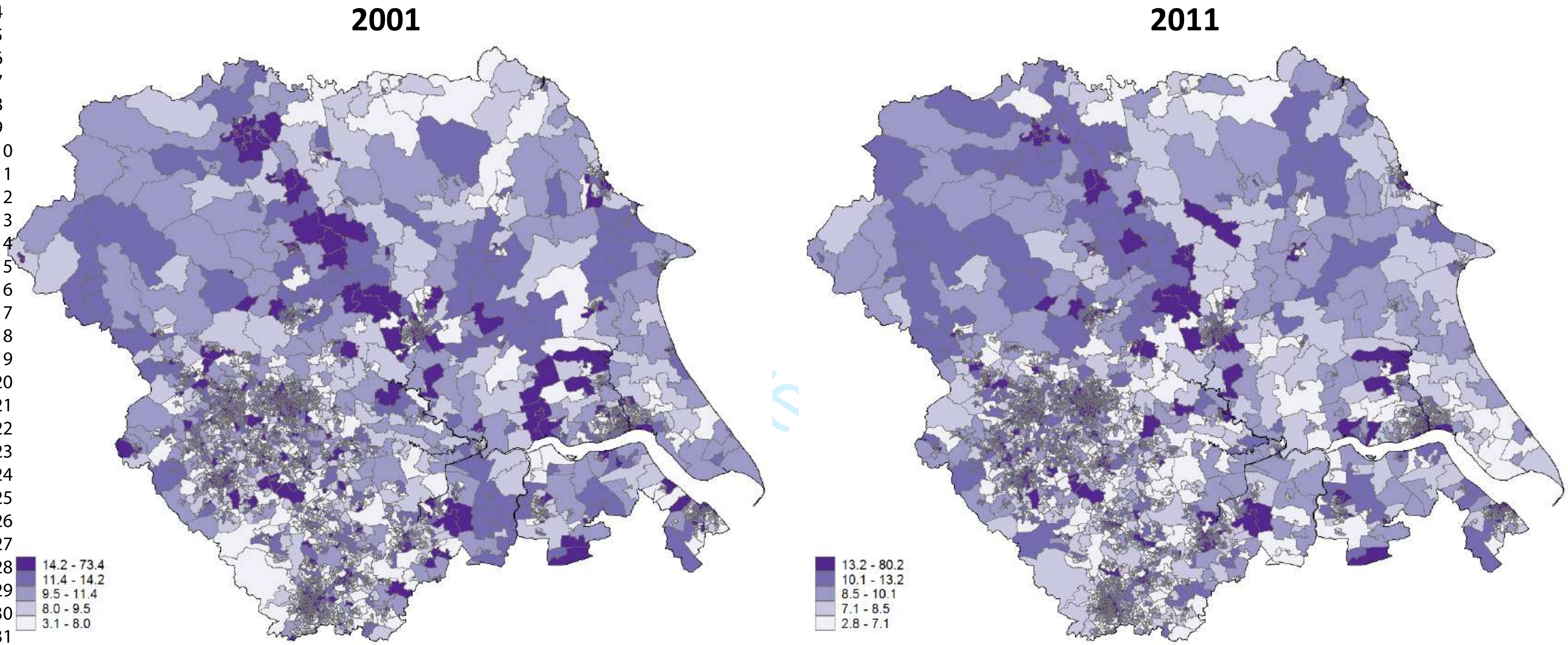

East Midlands, Population Turnover % of population

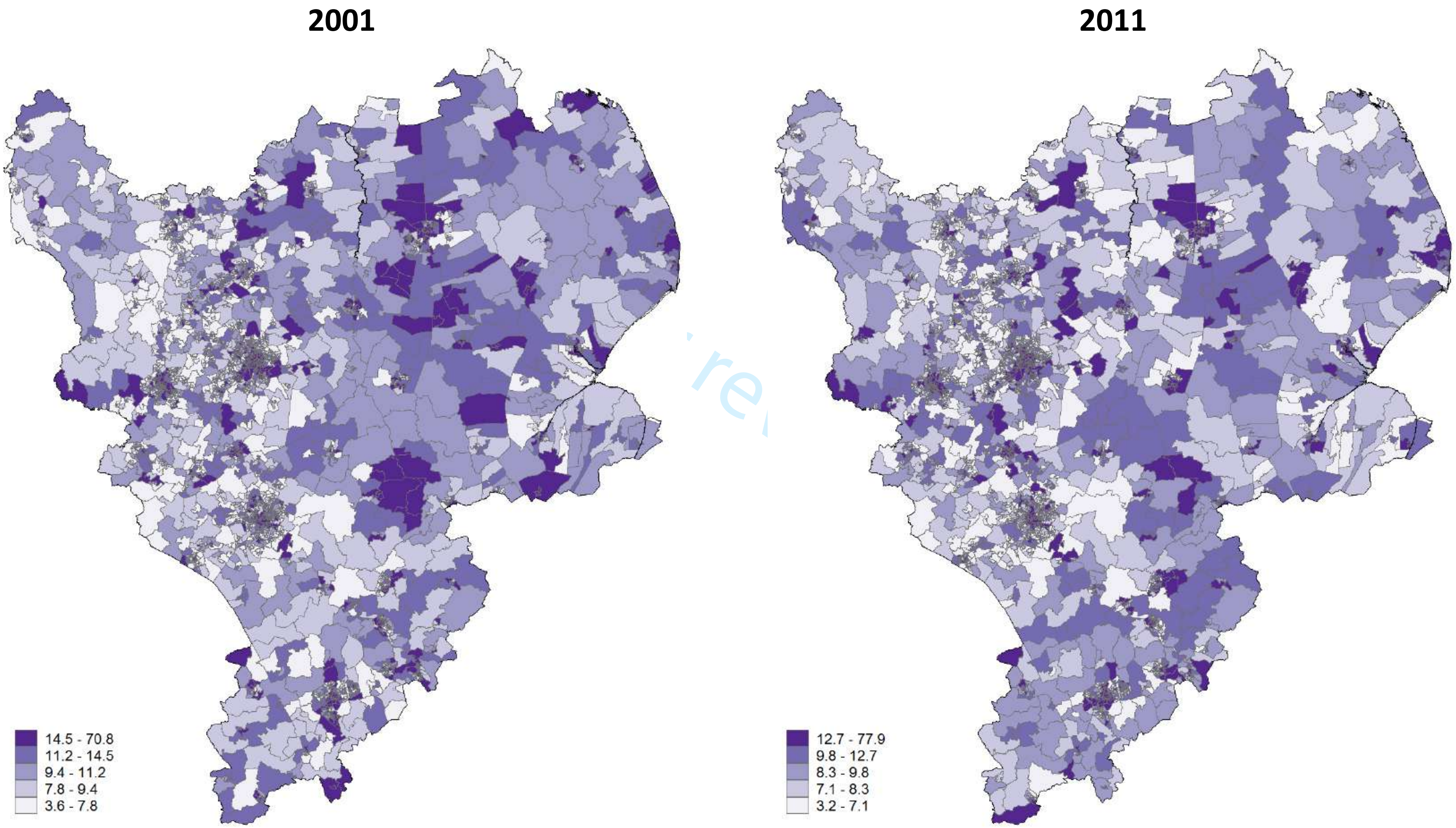

West Midlands, Population Turnover % of population

2001

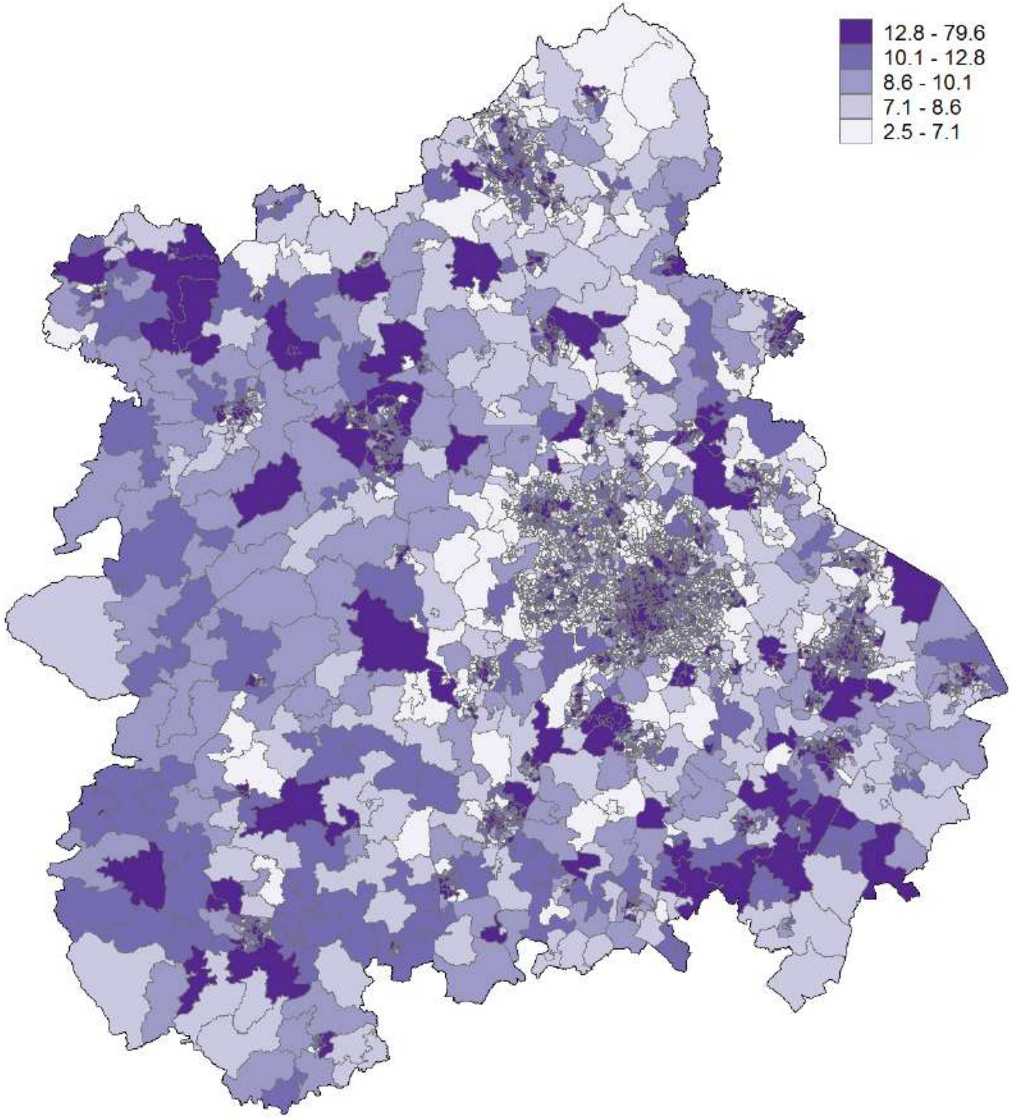

2011

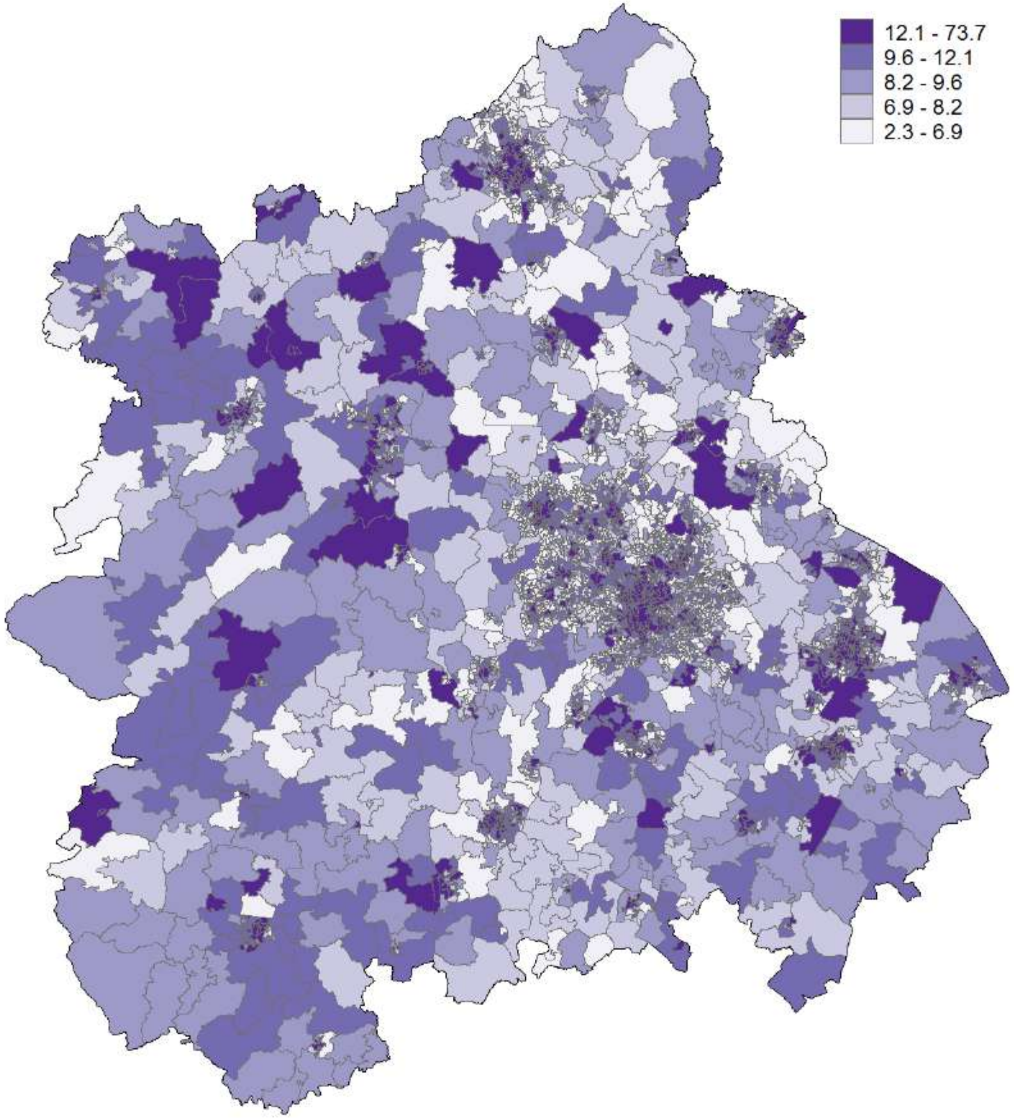

East of England, Population Turnover % of population

2001

1

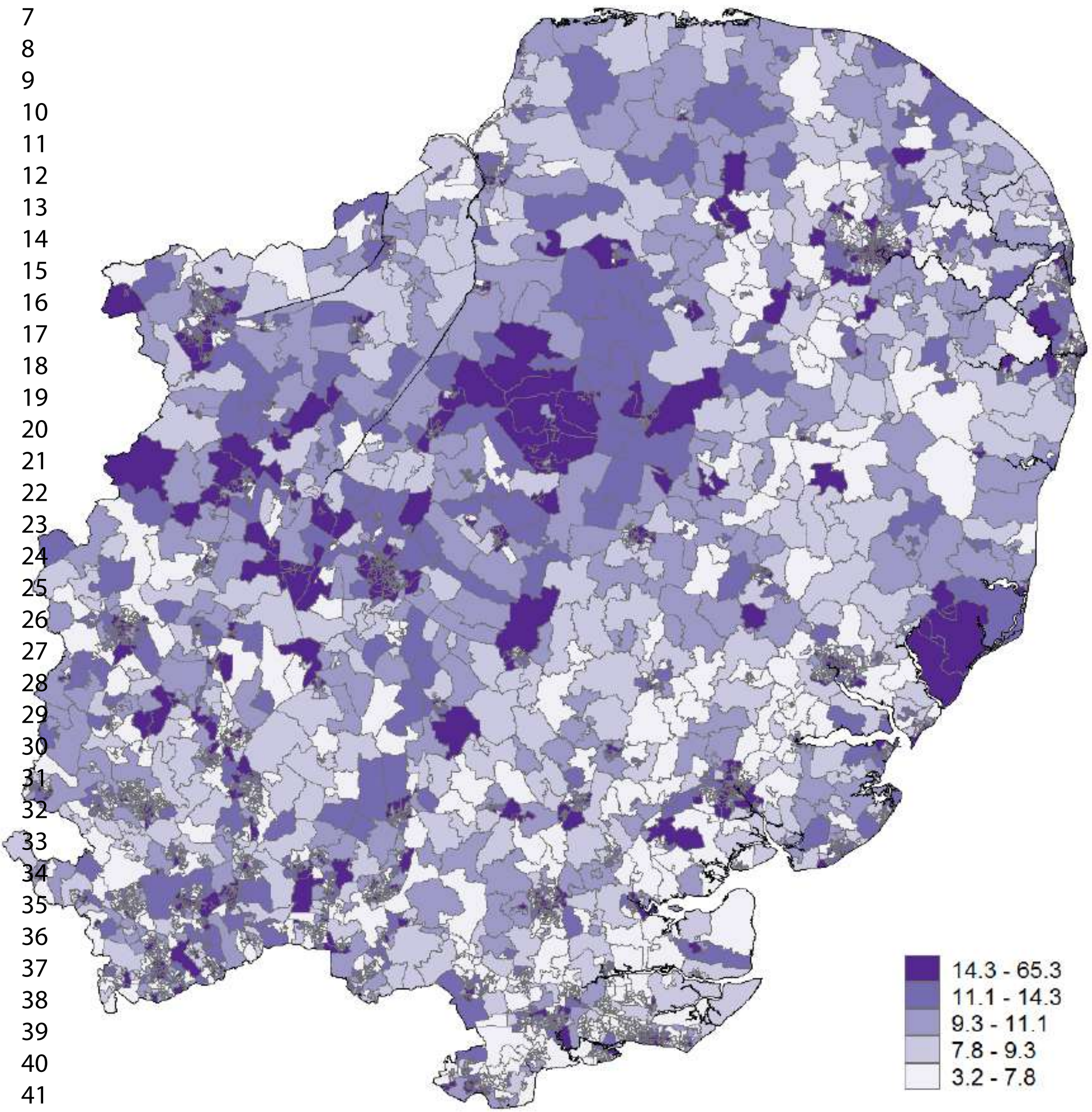

2011

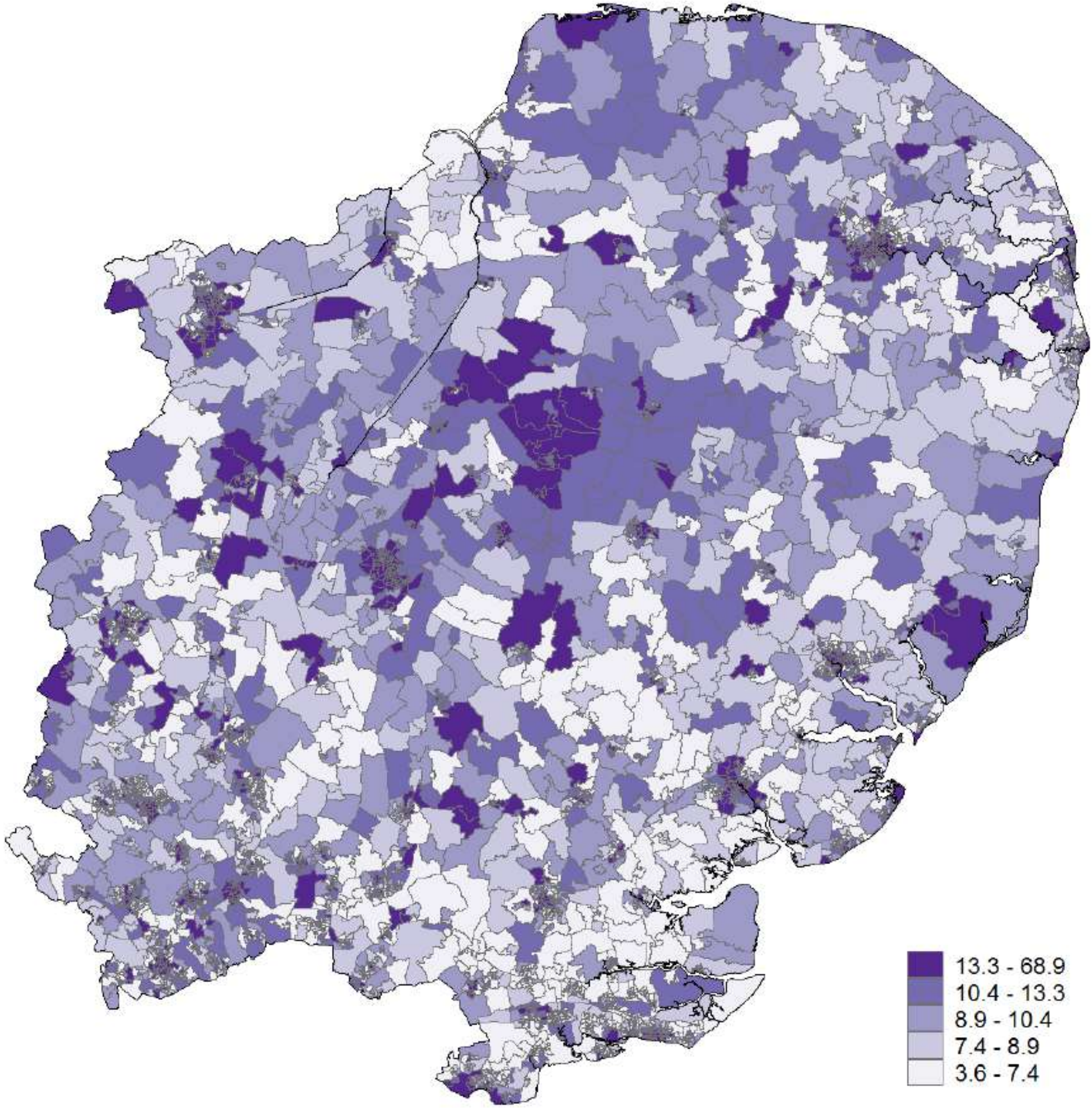

London, Population Turnover % of population

2001

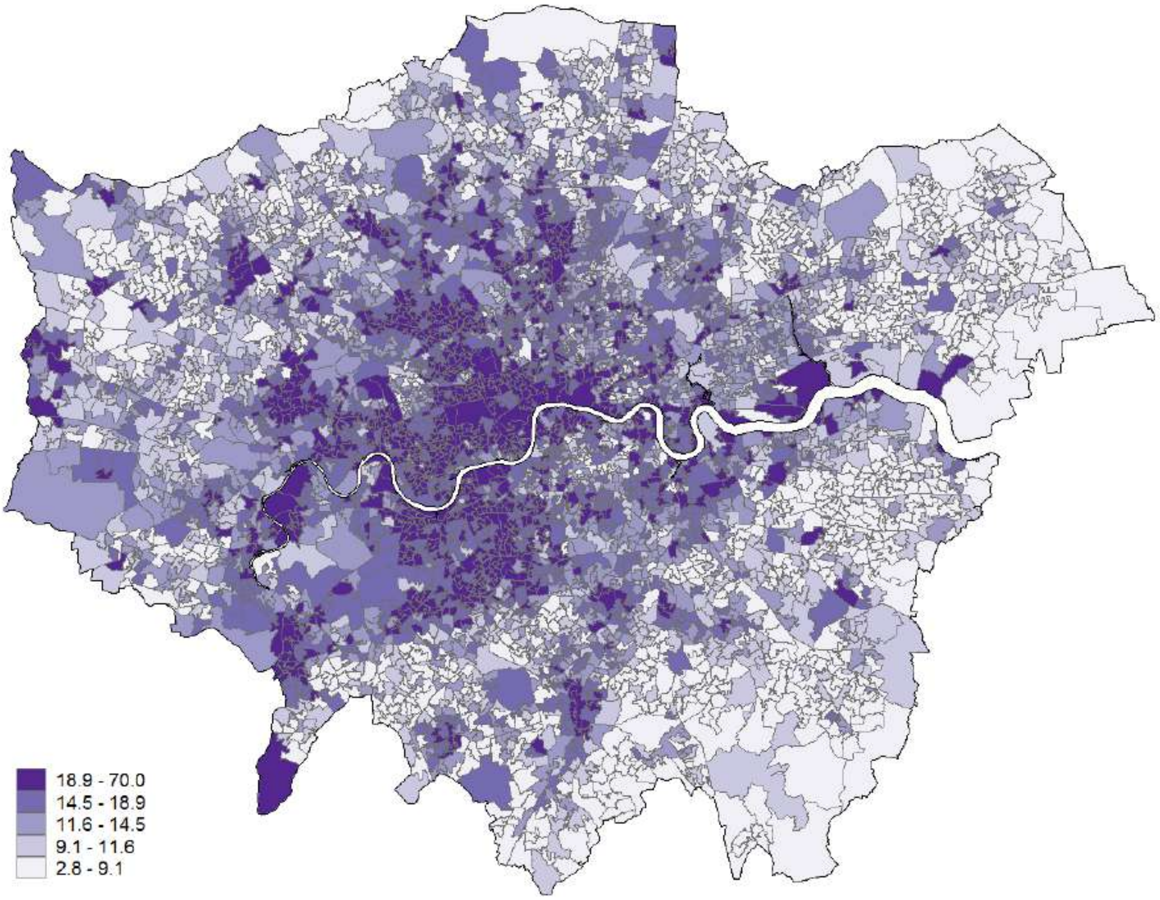

2011

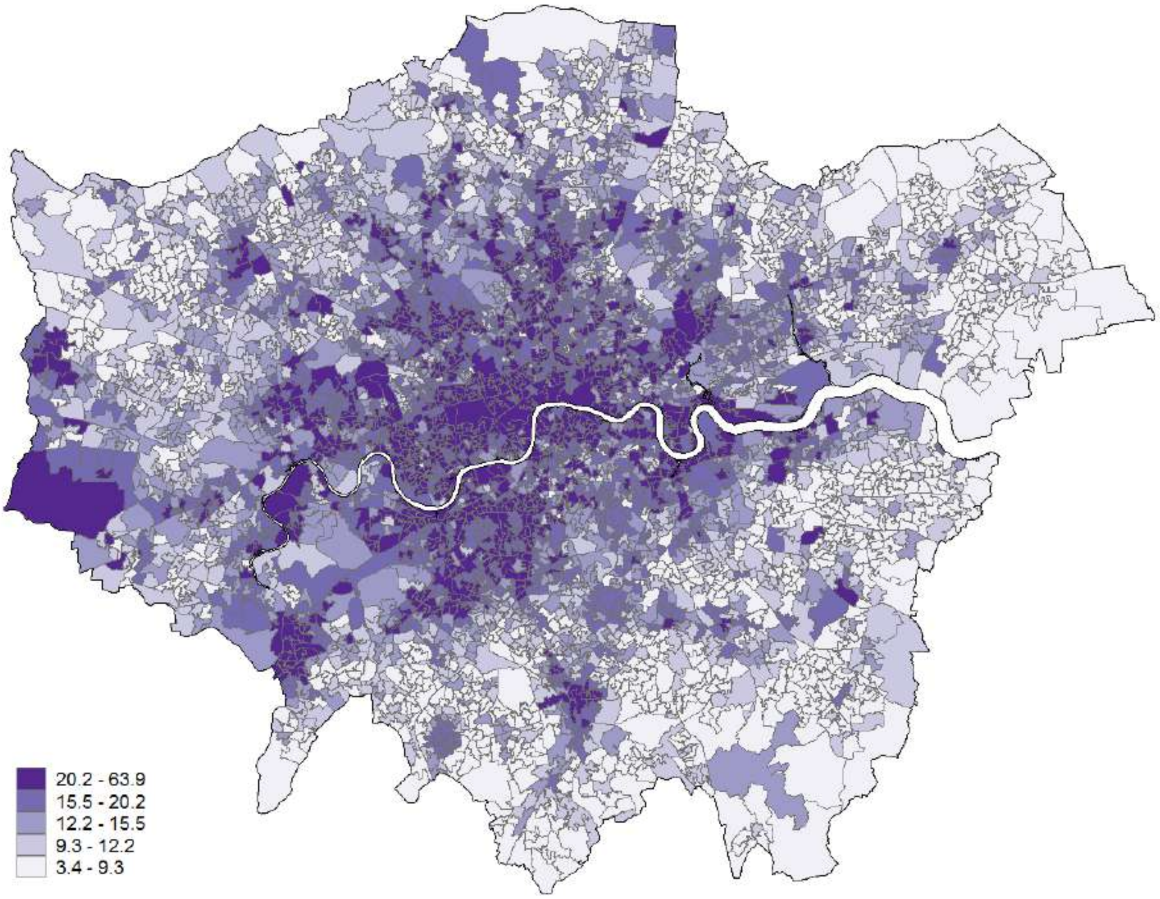

South East Coast, Population Turnover % of population

2001

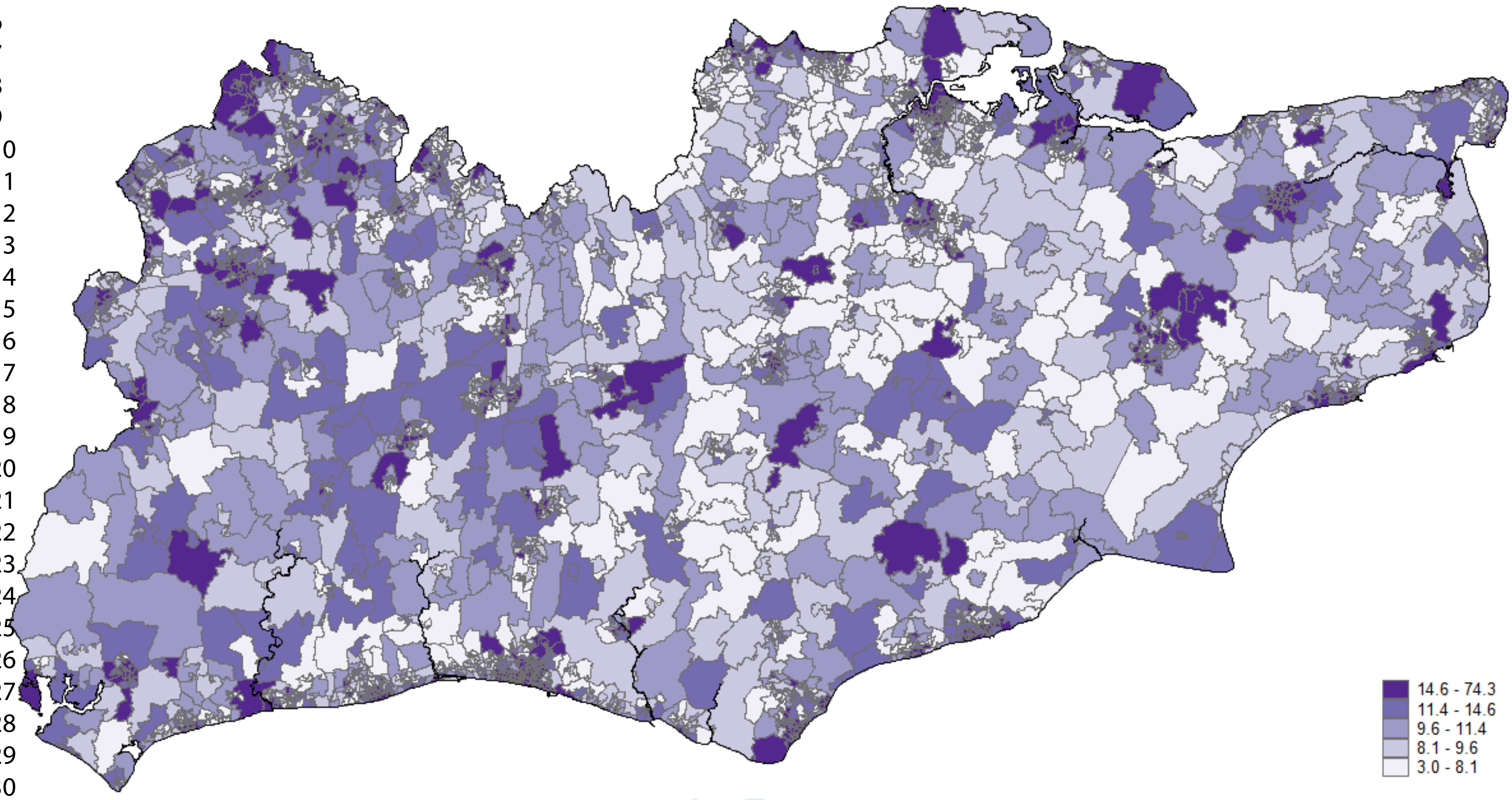

2011

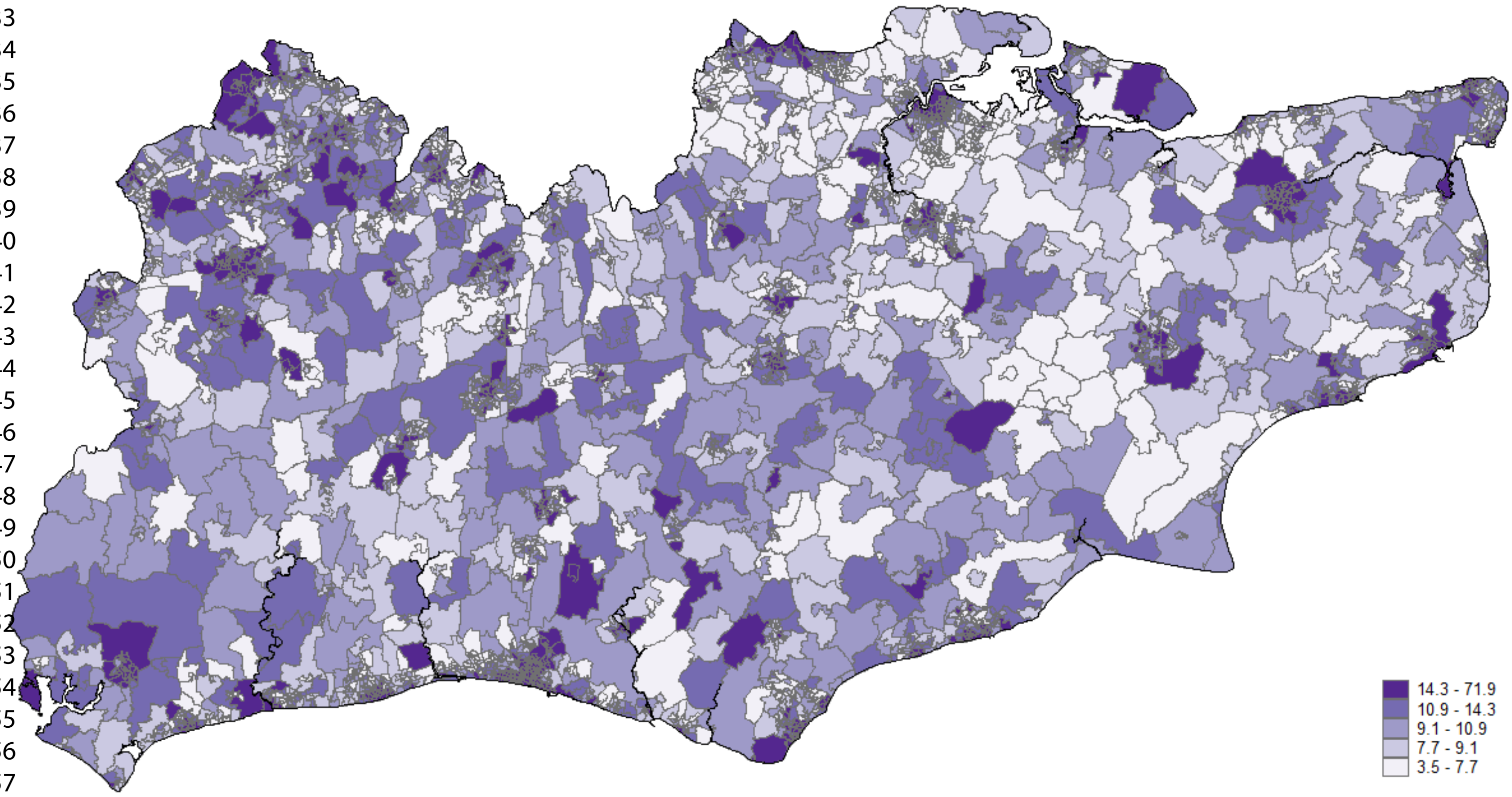

South Central, Population Turnover % of population

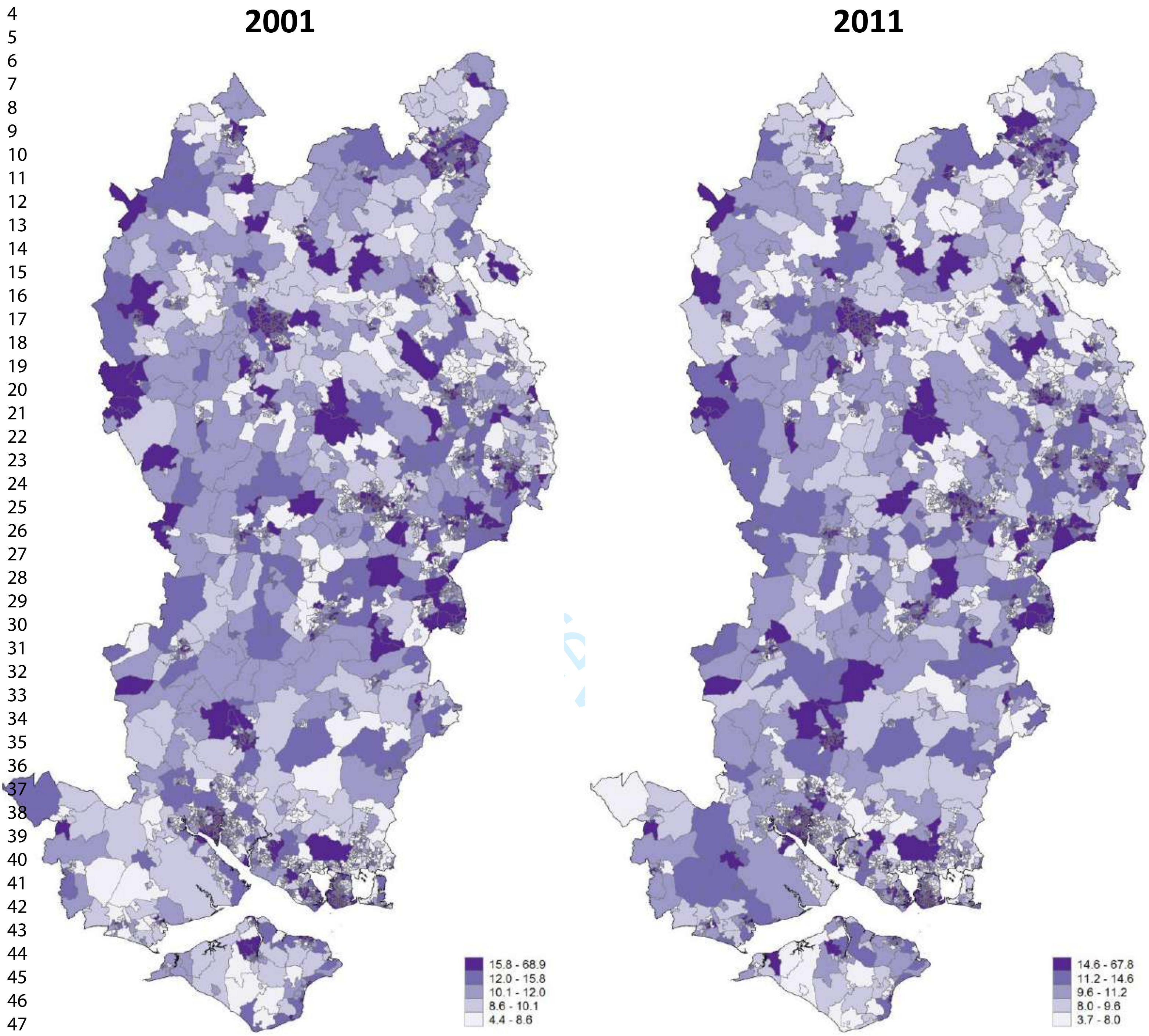

South West, Population Turnover % of population

2001

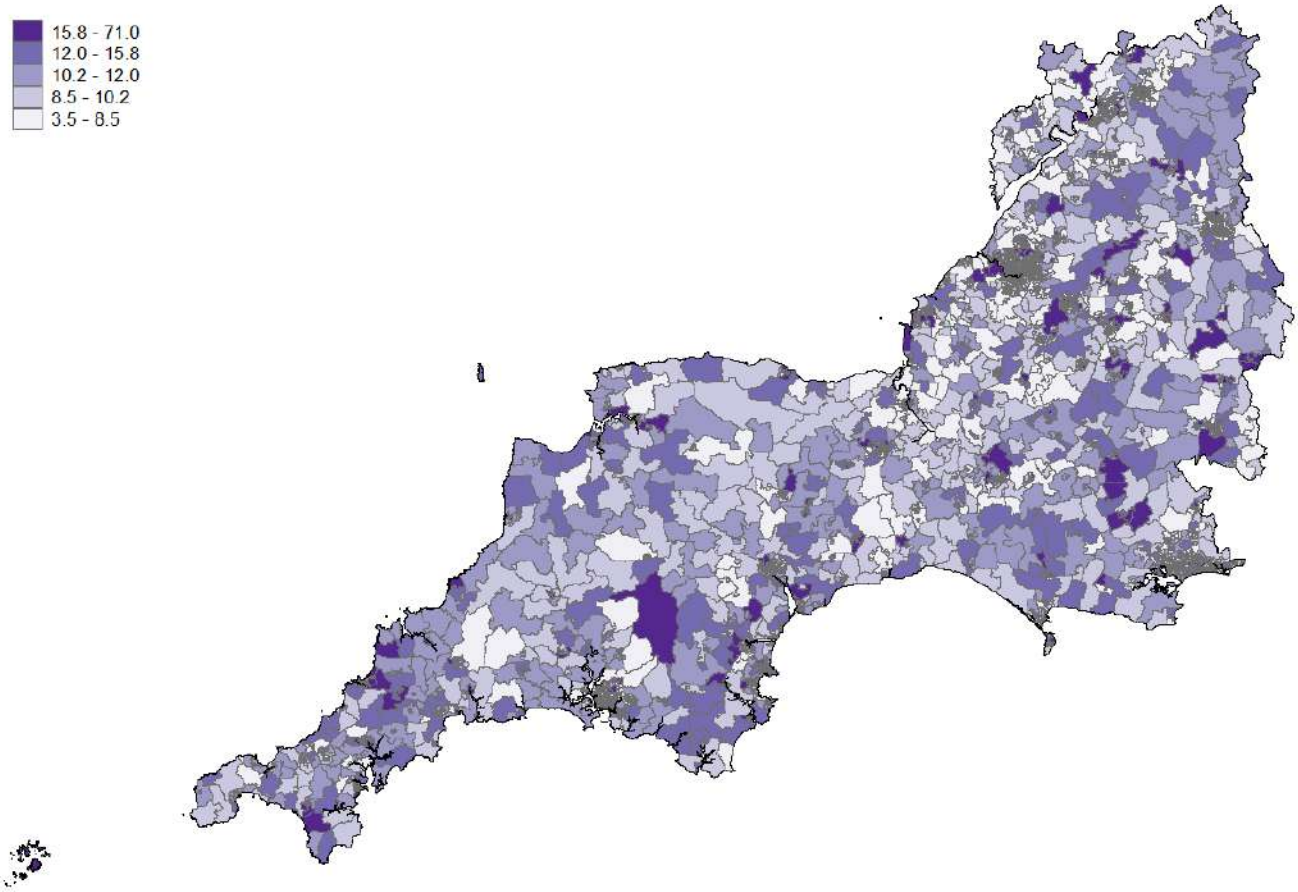

2011

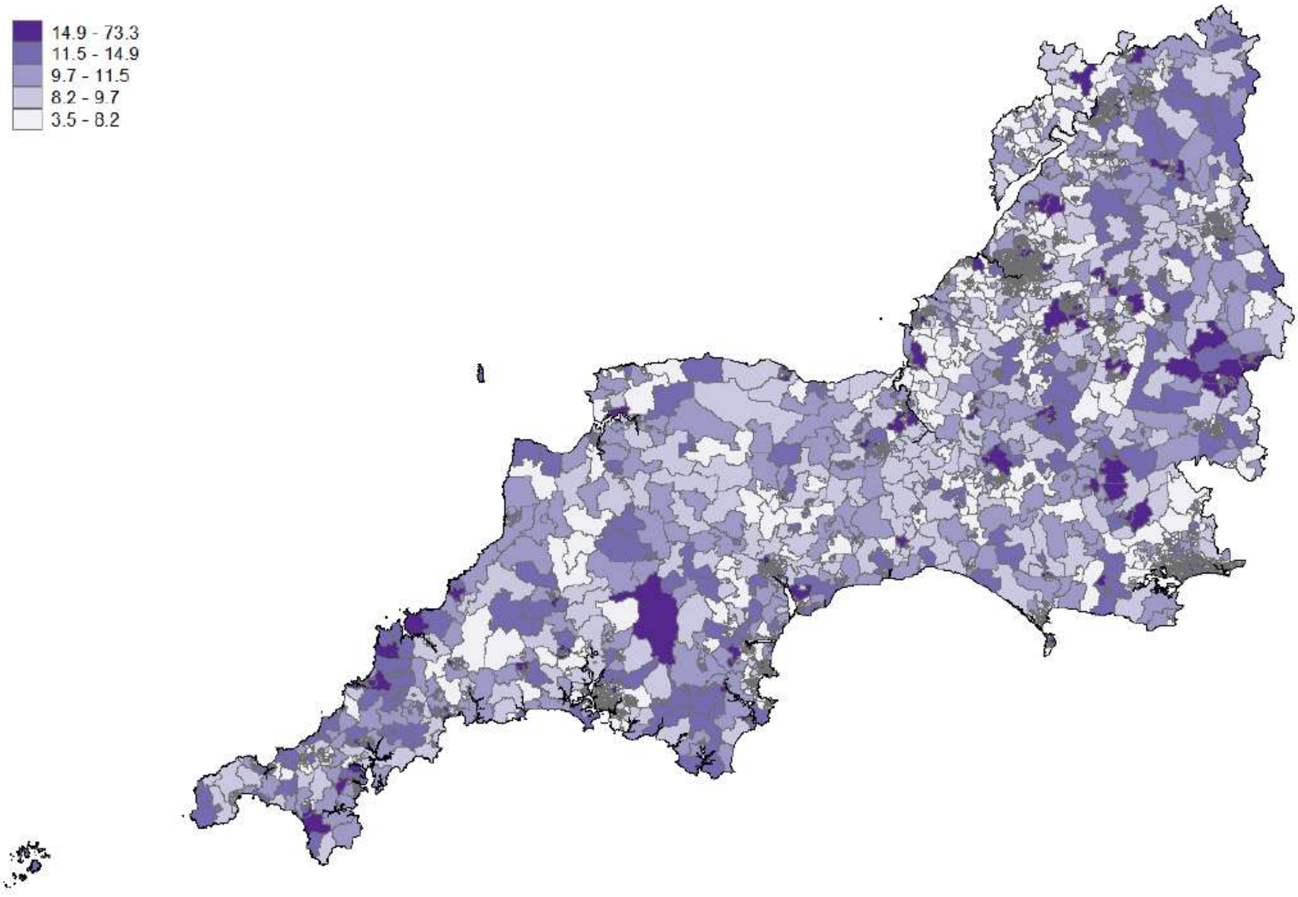

STROBE Statement—checklist of items that should be included in reports of observational studies

	Item No	Recommendation
**Title and abstract**	1	(*a*) Indicate the study's design with a commonly used term in the title or the abstract **p.1**
		(*b*) Provide in the abstract an informative and balanced summary of what was done and what was found **p.2**
**Introduction**		
Background/rationale	2	Explain the scientific background and rationale for the investigation being reported **p.3**
Objectives	3	State specific objectives, including any prespecified hypotheses **p.4**
**Methods**		
Study design	4	Present key elements of study design early in the paper
Setting	5	Describe the setting, locations, and relevant dates, including periods of recruitment, exposure, follow-up, and data collection **p.4**
Participants	6	(*a*) *Cohort study*—Give the eligibility criteria, and the sources and methods of selection of participants. Describe methods of follow-up **p.5**<br>*Case-control study*—Give the eligibility criteria, and the sources and methods of case ascertainment and control selection. Give the rationale for the choice of cases and controls **NA**<br>*Cross-sectional study*—Give the eligibility criteria, and the sources and methods of selection of participants **NA**
		(*b*) *Cohort study*—For matched studies, give matching criteria and number of exposed and unexposed **NA**<br>*Case-control study*—For matched studies, give matching criteria and the number of controls per case **NA**
Variables	7	Clearly define all outcomes, exposures, predictors, potential confounders, and effect modifiers. Give diagnostic criteria, if applicable **p.4**
Data sources/ measurement	8*	For each variable of interest, give sources of data and details of methods of assessment (measurement). Describe comparability of assessment methods if there is more than one group **p.4**
Bias	9	Describe any efforts to address potential sources of bias **NA**
Study size	10	Explain how the study size was arrived at **p.5**
Quantitative variables	11	Explain how quantitative variables were handled in the analyses. If applicable, describe which groupings were chosen and why **p.5**
Statistical methods	12	(*a*) Describe all statistical methods, including those used to control for confounding **p.5**
		(*b*) Describe any methods used to examine subgroups and interactions **NA**
		(*c*) Explain how missing data were addressed **NA**
		(*d*) *Cohort study*—If applicable, explain how loss to follow-up was addressed **NA**<br>*Case-control study*—If applicable, explain how matching of cases and controls was addressed **NA**<br>*Cross-sectional study*—If applicable, describe analytical methods taking account of sampling strategy **NA**
		(*e*) Describe any sensitivity analyses **NA**

Continued on next page

For peer review only - http://bmjopen.bmj.com/site/about/guidelines.xhtml

Results		
Participants	13*	(a) Report numbers of individuals at each stage of study—eg numbers potentially eligible, examined for eligibility, confirmed eligible, included in the study, completing follow-up, and analysed **NA**
		(b) Give reasons for non-participation at each stage **NA**
		(c) Consider use of a flow diagram **NA**
Descriptive data	14*	(a) Give characteristics of study participants (eg demographic, clinical, social) and information on exposures and potential confounders p.5
		(b) Indicate number of participants with missing data for each variable of interest **NA**
		(c) *Cohort study*—Summarise follow-up time (eg, average and total amount) **p.5**
Outcome data	15*	*Cohort study*—Report numbers of outcome events or summary measures over time **p.5**
		Case-control study—Report numbers in each exposure category, or summary measures of exposure **NA**
		Cross-sectional study—Report numbers of outcome events or summary measures **NA**
Main results	16	(*a*) Give unadjusted estimates and, if applicable, confounder-adjusted estimates and their precision (eg, 95% confidence interval). Make clear which confounders were adjusted for and why they were included **NA**
		(*b*) Report category boundaries when continuous variables were categorized **NA**
		(*c*) If relevant, consider translating estimates of relative risk into absolute risk for a meaningful time period **NA**
Other analyses	17	Report other analyses done—eg analyses of subgroups and interactions, and sensitivity analyses **NA**
**Discussion**		
Key results	18	Summarise key results with reference to study objectives **p.5-p.6**
Limitations	19	Discuss limitations of the study, taking into account sources of potential bias or imprecision. Discuss both direction and magnitude of any potential bias **p.7**
Interpretation	20	Give a cautious overall interpretation of results considering objectives, limitations, multiplicity of analyses, results from similar studies, and other relevant evidence **p.8**
Generalisability	21	Discuss the generalisability (external validity) of the study results **p.9**
**Other information**		
Funding	22	Give the source of funding and the role of the funders for the present study and, if applicable, for the original study on which the present article is based **p.11**

*Give information separately for cases and controls in case-control studies and, if applicable, for exposed and unexposed groups in cohort and cross-sectional studies.

Note: An Explanation and Elaboration article discusses each checklist item and gives methodological background and published examples of transparent reporting. The STROBE checklist is best used in conjunction with this article (freely available on the Web sites of PLoS Medicine at http://www.plosmedicine.org/, Annals of Internal Medicine at http://www.annals.org/, and Epidemiology at http://www.epidem.com/). Information on the STROBE Initiative is available at www.strobe-statement.org.

For peer review only
